# Prognostic value of lncRNAs related to fatty acid metabolism in lung adenocarcinoma and their correlation with tumor microenvironment based on bioinformatics analysis

**DOI:** 10.3389/fonc.2022.1022097

**Published:** 2022-10-10

**Authors:** Ya-Qiang Pan, Ying Xiao, Tao Long, Chao Liu, Wen-Hui Gao, Yang-Yong Sun, Chang Liu, Yi-Jun Shi, Shuang Li, Ai-Zhong Shao

**Affiliations:** ^1^ Department of Cardiothoracic Surgery, People's Hospital Affiliated to Jiangsu University, Zhenjiang, China; ^2^ Department of Oncology, Hospital Affiliated to Hebei University of Engineering, Handan, China; ^3^ School of Medicine, Jiangsu University, Zhenjiang, China

**Keywords:** long non-coding RNA (lncRNA), fatty acid metabolism, lung adenocarcinoma (LUAD), bioinformatics, prognosis, competing endogenous RNA (ceRNA)

## Abstract

**Background:**

As a key regulator of metabolic pathways, long non-coding RNA (lncRNA) has received much attention for its relationship with reprogrammed fatty acid metabolism (FAM). This study aimed to investigate the role of the FAM-related lncRNAs in the prognostic management of patients with lung adenocarcinoma (LUAD) using bioinformatics analysis techniques.

**Methods:**

We obtained LUAD-related transcriptomic data and clinical information from The Cancer Genome Atlas (TCGA) database. The lncRNA risk models associated with FMA were constructed by single-sample gene set enrichment analysis (ssGSEA), weighted gene co-expression network (WGCNA), differential expression analysis, overlap analysis, and Cox regression analysis. Kaplan-Meier (K-M) and receiver operating characteristic (ROC) curves were utilized to assess the predictive validity of the risk model. Gene set variation analysis (GSVA) revealed molecular mechanisms associated with the risk model. ssGSEA and microenvironment cell populations-counter (MCP-counter) demonstrated the immune landscape of LUAD patients. The relationships between lncRNAs, miRNAs, and mRNAs were predicted by using LncBase v.2 and miRTarBase. The lncRNA-miRNA-mRNA regulatory network was visualized with Cytoscape v3.4.0. Gene Ontology (GO) and Kyoto Encyclopedia of Genes and Genomes (KEGG) pathway enrichment analysis was performed using DAVID v6.8. Quantitative real-time fluorescence PCR (qRT-PCR) was performed to verify the expression levels of the prognostic lncRNAs.

**Results:**

We identified 249 differentially expressed FMA-related lncRNAs in TCGA-LUAD, six of which were used to construct a risk model with appreciable predictive power. GSVA results suggested that the risk model may be involved in regulating fatty acid synthesis/metabolism, gene repair, and immune/inflammatory responses in the LUAD process. Immune landscape analysis demonstrated a lower abundance of immune cells in the high-risk group of patients associated with poor prognosis. Moreover, we predicted 279 competing endogenous RNA (ceRNA) mechanisms for 6 prognostic lncRNAs with 39 miRNAs and 201 mRNAs. Functional enrichment analysis indicated that the ceRNA network may be involved in the process of LUAD by participating in genomic transcription, influencing the cell cycle, and regulating tissue and organogenesis. *In vitro* experiments showed that prognostic lncRNA CTA-384D8.35, lncRNA RP5-1059L7.1, and lncRNA Z83851.4 were significantly upregulated in LUAD primary tumor tissues, while lncRNA RP11-401P9.4, lncRNA CTA-384D8.35, and lncRNA RP11-259K15.2 were expressed at higher levels in paraneoplastic tissues.

**Conclusion:**

In summary, the prognostic factors identified in this study can be used as potential biomarkers for clinical applications. ceRNA network construction provides a new vision for the study of LUAD pathogenesis.

## Introduction

According to GLOBOCAN 2020 cancer incidence estimates, lung cancer is the leading cause of cancer-related death and has the highest incidence of mortality globally (1). Lung cancer is subdivided into small cell lung cancer (SCLC) and non-small cell lung cancer (NSCLC), with lung adenocarcinoma (LUAD) and lung squamous cell carcinoma (LUSC) constituting non-small cell lung cancer. LUAD is the most common form of lung cancer, including 85% of non-small cell lung cancers and 40% of all forms (2). Patients with LUAD are typically diagnosed with advanced illness or metastases despite considerable advances in clinical diagnosis and multimodal therapy, including surgery, chemotherapy, and targeted medicines (3), and 5-year lung cancer survival rates range from 10 to 20% in the majority of nations (4). The lack of understanding of the molecular mechanisms leads to limitationsin treatment outcomes. Therefore, further exploration of new prognosticbiomarkers for patients with LUAD is necessary.

Long non-coding RNAs (lncRNAs) are defined as non-protein-coding RNA transcripts that include more than 200 nucleotides, which play an essential role in the regulation of genes as well as a wide variety biological processes (5, 6). The aberrant regulation of lncRNAs is common in cancer, and it has been shown to have a role in the development and progression of the disease (7). lncRNAs contribute to the regulation of tumor development by promoting carcinogenesis, invasion, and drug resistance (8). lncRNAs have the potential to engage in interactions with mRNA, microRNA (miRNA), DNA, and a wide variety of proteins, which might have significant implications for a variety of pathophysiological processes, such as epigenetic regulation, glycolysis, DNA repair, and cellular stem cells (9–11). As a result of the advent of high-throughput sequencing technology, an increasing number of lncRNAs, such as H19, MALAT1, HOTAIR, and JPX, are being recognized as prognostic biomarkers for LUAD (12–15). Consequently, understanding the role of lncRNAs in LUAD might result in the development of new prognostic biomarkers and the finding of potential therapy targets.

Since immune, methylation, ferroptosis, cell scorching and necroptosis-related pathways have been extensively reported in the field of LUAD, but studies on fatty acid metabolism-related pathways are still scarce, its important impact on the prognosis of LUAD was further investigated. Fatty acids play an essential role in the body’s metabolic processes, as well as cell growth and signaling (16). There is a connection between the fatty acid signaling system and the development and progression of tumors. Synthesized lipids are used by cancer cells for the purposes of proliferation, survival, invasion, and angiogenesis (17). A research indicates that lncRNAs may influence the development of cancer *via* altering fatty acid metabolism (FAM) (18). There have been many studies that have shown a connection between FAM-related lncRNAs and the proliferation and differentiation of LUAD cells. There was a significant correlation between high FAM83A-AS1 expression in LUAD and poor overall and progression-free survival (OS and PFS) (19). Proliferation and differentiation of LUAD are promoted by FAM83A-AS1 through the HIF-1/glycolytic axis (20). In addition, the competing endogenous RNA hypothesis (ceRNA), which was proposed by Salmena et al., is predicated on a massive regulatory network system that depicts a sophisticated interplay between coding and non-coding RNAs (21). According to this theory, the expression of mRNAs that are involved in FAM may be influenced by lncRNAs in LUAD by sponging on the function of miRNAs in the region. The ceRNA networks have been generated in a variety of cancers by a number of different researches (22), but their regulatory role in LUAD is unknown. Therefore, gaining an understanding of the role that FAM-related lncRNAs play in LUAD might help in the development of new prognostic biomarkers as well as potential therapy targets.

In the beginning of our analysis, we collected information from the MsigDB, TCGA, and GEO databases. Using several bioinformatics techniques, we establish lncRNA signatures related with FAM. Then, we identified six survival-related FAM-related lncRNAs and developed ceRNA networks associated with FAM. To explore the link between FAM pathways and LUAD, Gene set variation analysis (GSVA) uncovered molecular processes related with the risk model. We evaluated prognostic lncRNAs using TCGA internal and quantitative real-time polymerase chain reaction (qRT-PCR) as well as external cohorts.

## Materials and methods

### Data source

The TCGA database was queried for transcriptome data, survival details, and clinical information on LUAD. There are 510 LUAD samples and 58 normal control samples in the RNA-seq expression matrix (mRNA, miRNA, and lncRNA). For the screening of prognostic genes and assessment of the prognostic model, 497 LUAD patients with complete survival data were employed. In addition, we retrieved the GSE31210 dataset (https://www.ncbi.nlm.nih.gov/geo/query/acc.cgi?acc=GSE31210) from the GEO database as an independent validation set. This dataset consists of lncRNA expression profiles and complete survival details from 226 LUAD patients (23, 24).

### FMA score based on ssGSEA

Based on the expression profile of FMA-related genes in the TCGA dataset, the ssGSEA method was used to calculate the FAM score for each of the 568 samples. In order to acquire FMA-related genes, we first downloaded four datasets from MsigDB: the hallmark gene sets, the Gene Ontology gene sets, the KEGG Canonical pathways, and the Reactome pathway. After that, we retrieved the FMA, and the genes that we obtained were regarded to as FMA-related genes. In the meanwhile, we also got genes associated to metabolism from a list of 2752 human metabolic enzymes and transporter proteins genes reported in earlier research. After that, we collected FMA-related genes using the phrase “fatty acid.” Combining the FMA-related genes that were previously collected and deleting duplicate genes(retaining unique genes) led to the discovery of a total of 525 FMA-related genes that were used in this investigation. Following this, we matched the expression profiles of the 525 FMA-related genes in the TCGA database. Using the ssGSEA algorithm, we obtained the FMA scores of 510 LUAD and 58 normal samples. We then divided the samples into groups with high and low FMA scores based on the median values of the scores. (**Supplementary Table 1**).

### WGCNA

We created co-expression networks in the R package WGCNA (1.69 version) utilizing all lncRNAs in 510 LUAD and 58 normal samples from the TCGA database in order to study the link between gene expression data and clinical characteristics (high- and low- FMA scores) (25, 26). Using flashClust, outliers were evaluated and a sample tree was generated for 568 samples. No niche samples were detected in the current investigation. (**Supplementary Figure 1A**). To ensure that the gene distribution corresponded to the scale-free network, the β values were used to create the neighbor matrix (27). Using an approach of dynamic cutting, the tree was divided into several modules. The appropriate MEDissThres settings were set to merge comparable modules (**Supplementary Figure 1B**). The Pearson correlation coefficient was used to determine the association between co-expression modules and the high and low FMA score groups, and a heatmap was then generated. The module having the strongest association with the two score groups was chosen for further investigation as the hub module. All lncRNAs in the discovered hub module were regarded as FMA-related lncRNAs.

### Differential expression analysis

The R package limma was used to identify DE-lncRNAs between 510 LUAD and 58 normal samples. DE-lncRNAs between LUAD and normal samples were identified as lncRNAs with |log_2_ fold change (FC)| > 0.5 and adjusted (adj.) *P*< 0.05. (LUAD vs. normal). The adj. *P* was determined using the Benjamin & Hochberg technique with multiple testing adjustments. A total of 5265 DE-mRNAs (**Supplementary Table 2**) and 333 DE-miRNAs (**Supplementary Table 3**) associated with LUAD in the TCGA database were obtained using the same methods and thresholds.

### Overlap analysis

To identify lncRNAs associated with DE-FMA, an overlap analysis was conducted. Essentially, the list of FMA-related lncRNAs obtained by WGCNA and the list of DE-lncRNAs acquired were submitted to the Jvenn online tool (http://jvenn.toulouse.inra.fr/app/example.html) to find the common elements. Adobe Illustrator 2020 was used to create the Venn diagram.

### The construction, evaluation, and validation of risk model

We chose a cohort of 497 LUAD patients from the TCGA database containing complete clinical information (including survival time) as the training set for identifying prognostic genes and constructing and evaluating prognostic models. The GSE31210 dataset (n = 226) was used to validate the prognostic model externally and independently. For the discovered DE-FMA-related lncRNAs, we conducted a univariate Cox regression analysis on the training set. Variables that satisfied the requirements based on univariate P values with a significance level of less than 0.05 were subjected to multivariate Cox analysis. The significance criterion for identifying the best prognostic gene was set at *P*< 0.05. Based on the expression and regression coefficients (coef, output of multivariate Cox analysis) of the prognostic genes, risk scores were generated for each sample in the training set and the independent external validation set.The formula for the risk score as shown below:


$$riskscore=(coef_{gene_{1}}\times expressionvalueofgene_{1})+(coef_{gene_{2}}\times expressionvalueofgene_{2})+\cdots (coef_{gene_{n}}\times expressionvalueofgene_{n})$$

Each dataset’s LUAD samples were separated into high- and low-risk groups based on the corresponding median risk score value. The high-risk group consisted of samples with risk scores above the median value, whereas the low-risk group consisted of samples with risk scores below the median value. The MEDIAN function was used to obtain the median value of the risk scores. After that, variations in OS of LUAD patients between high- and low-risk groups in the training set and independent external validation set were evaluated using Kaplan-Meier (K-M) analysis and log-rank test, and *P*< 0.05 was regarded statistically significant. Then, time-dependent (1, 2, and 3 years) ROC curves were produced using the risk scores, and the prognostic prediction performance of the prognostic model was assessed by calculating the area under the curve (AUC) in the training set and the independent external validation set.

### Stratified survival analysis

On the basis of clinical characteristics, we divided TCGA-LUAD patients into various clinical subgroups, including age subgroups (≤ 65 and > 65), gender subgroups (male and female), tissue origin subgroups (upper and lower pages), stage subgroups (stage i-ii and stage iii-iv), pathological T stage (T1 and T2-4), and pathological N stage (T1 and T2-4) (N1 and N1-3). The Wilcoxon rank-sum test was used to identify variances in risk score levels among subgroups. The K-M curve was used to evaluate the capacity of the risk score system to discriminate clinical outcomes among various clinical subgroups of patients. *P*< 0.05 was deemed statistically significant.

### GSVA

We segregated LUAD samples from the TCGA dataset into high- and low-risk categories. Using the c2.cp.kegg.v7.4.symbols.gmt as the reference gene set and setting the adj. P-value to< 0.05 and the |t value| > 4, we ran GSVA comparing high- and low-risk patients using the GSVA package 1.38 in R (28).

### ESTIMATE, ssGSEA, and MCP-counter of the LUAD samples

Estimation of STromal and Immune cells in MAlignant Tumor tissues using Expression data (ESTIMATE) is a technique for assessing tumor purity and the existence of stromal/immune cells in tumor samples (29). Using ESTIMATE, we assessed the immune score (levels of immune cell infiltration), stromal score (stromal content), and ESTIMATE score (which suggests tumor purity) for each LUAD sample. The ratio of the appropriate component in the tumor immune microenvironment is proportional to the value of the related score.

We calculated the enrichment levels of 28 immune-associated datasets in each LUAD sample in the form of ssGSEA (30) scores in the GSVA R package. Marker genes expressions-based microenvironment cell populations-counter (MCP-counter) (31), was also used to evaluate the number of immune-infiltrating cells in each sample; this method yielded an abundance score for eight immune populations (CD8+ T cells, myeloid dendritic cells, T cells, natural killer cells, monocytic lineage, B lineages, cytotoxic lymphocytes, and neutrophils) and two stromal populations (fibroblasts and endothelial cells). The analysis of gene expression in cell markers served as the foundation for the subpopulation classification of these cells. T-test was used to find statistically significant differences in immune cell counts.

### ceRNA network

On the basis of the targeting interactions, a lncRNA-miRNA-mRNA ceRNA network was created. This ceRNA network established a complex post-transcriptional regulatory network in LUAD as a result of revealing the competing binding of miRNAs by lncRNAs and mRNAs. The LncBase V2.0 (32) database (www.microrna.gr/LncBase) was used to predict the target miRNAs of prognostic lncRNAs, and the threshold was set to score > 0.6 to obtain lncRNA-miRNA relationship pairs. Subsequently, DE-miRNA-targeted mRNAs were obtained through the miRTarBase (http://mirtarbase.mbc.nctu.edu.tw/) database, which provides experimentally validated miRNA-mRNA relationship pairs (33). Based on the ceRNA hypothesis, we selected DE-mRNAs with the same expression trend as the prognostic lncRNA and DE-miRNAs with the opposite expression trend to the prognostic lncRNA to form lncRNA-miRNA and miRNA-mRNA relationship pairs. In addition, we obtained lncRNA-mRNA co-expression relationship pairs with correlation coefficients (r) > 0.4 and p< 0.05 by Pearson correlation analysis performed on prognostic lncRNAs and all DE-mRNAs. Ultimately, ceRNA networks were constructed using lncRNA-miRNA-mRNA relationship pairs with overlapping relationships. The ceRNA network was visualized using Cytoscape V3.4.07.

### DAVID database

We used the online tool known as the Database for Annotation, Visualization, and Integrated Discovery (DAVID, http://david.ncifcrf.gov/, version 6.8) in order to carry out functional annotation and pathway enrichment analysis on the mRNAs that make up the ceRNA network (34). This allowed us to better understand the biological roles that these mRNAs play. The threshold value was determined to be *P*< 0.05 and count ≥ 2. The R program was used to visualize the findings of GO enrichment and KEGG pathway analysis as bubble charts. GO enrichment and KEGG pathway results were visualized as bubble charts by R software.

### Patient preparation and collection

We collected frozen and surgically resected tumor tissues from 20 patients with pathologically diagnosed LUAD at the Affiliated People’s Hospital of Jiangsu University. After removal, the surgical specimens were immediately frozen in liquid nitrogen and stored at −80°C. The studies involving human participants were reviewed and approved by Ethics Committee of the Affiliated People’s Hospital of Jiangsu University (approval NO.K-20200097-Y). The patients provided their written informed consent to participate in this study.

### RNA isolation and quantitative real-time polymerase chain reaction (qRT-PCR)

Total RNA was extracted from all 20 tissues using TRIzol Reagent (Life Technologies-Invitrogen, Carlsbad, CA, USA) according to the manufacturer’s recommendations. A NanoDrop 2000FC-3100 nucleic acid protein quantifier was then used to measure the RNA solution’s concentration and purity (Thermo Fisher Scientific, Waltham, MA, USALife Real). Prior to quantitative real-time PCR, the isolated RNA was reverse-transcribed to cDNA using the SureScript-First-strand-cDNA-synthesis-kit (Genecopoeia, Guangzhou, China). The qRT-PCR experiment included 3 µl of reverse transcription product, 5 µl of 5×BlazeTaq qPCR Mix (Genecopoeia, Guangzhou, China), and 1µl of forward and reverse primer, respectively. Initial denaturation at 95°C for 1 minute was followed by 40 cycles of incubation at 95°C for 20 seconds, 55°C for 20 seconds, and 72°C for 30 seconds. **Table 1** displays the sequence information for all primers that were manufactured by Servicebio (Wuhan, China). The GAPDH gene served as an internal reference, and the 2-ΔΔCt method technique was used to measure the relative expression of six prognostic genes (35). The experiment was done three times on separate dates. Six prognostic lncRNAs were compared between para-cancer and LUAD samples using paired t-tests and GraphPad Prism V6 (GraphPad Software, La Jolla, CA, USA). The degree of statistical significance was determined and reported as follows: * for *P*< 0.05; ** for *P*< 0.01.

**Table 1:** Sequence information of 6 lncRNAs.

The specific primer sequences of prognostic lncRNAs	
Gene name	Primer sequence (5′ to 3′)
RP11-401P9.4	Forward: 5′-TGTTACTTGGGGTTCCTGTTGC-3′
	Reverse: 5′-TGGGATGGGTTATGATGCTTTC-3′
RP11-4B16.3	Forward: 5′-TGCTGTGGGCAAAAGAA-3′
	Reverse: 5′-GCCTCAGGGCAATGTAA-3′
CTA-384D8.35	Forward: 5′-GTTGCTAGTCCTCCGCTTCG-3′
	Reverse: 5′-CTTTCAGTCAGGTGTTCCCC-3′
RP5-1059L7.1	Forward: 5′-CCTGGGGACAAAGTAAGCTAGT-3′
	Reverse: 5′-GATGATTCTGTGTTCCACGGAT-3′
Z83851.4	Forward: 5′-GCAGCAGGAGCCGTGAATT-3′
	Reverse: 5′-ATGGGTGGGCAGGGAAAAG-3′
RP11-259K15.2	Forward: 5′-AGGGTAACTGAGGGAGGTAAG-3′
	Reverse: 5′-TAAGGTGTAATTGGGAAGAGG-3′
GAPDH	Forward: 5′-CCCATCACCATCTTCCAGG-3′
	Reverse: 5′-CATCACGCCACAGTTTCCC-3′

### Statistical analysis

All analyses and data plotting were performed using R software (https://www.r-project.org/, version 4.0.1, R Project for Statistical Computing). All box plots were generated by the R package ggplot2 (version 3.3.2). The survival analysis and Cox regression analysis were performed in the R package Survival (version 3.2-3). The R package pROC (version 1.12.1) was used to draw the ROC curves. Significance thresholds were labeled where appropriate.

## Results

### Identification of the FMA-related lncRNAs in TCGA-LUAD

**Figure 1:**
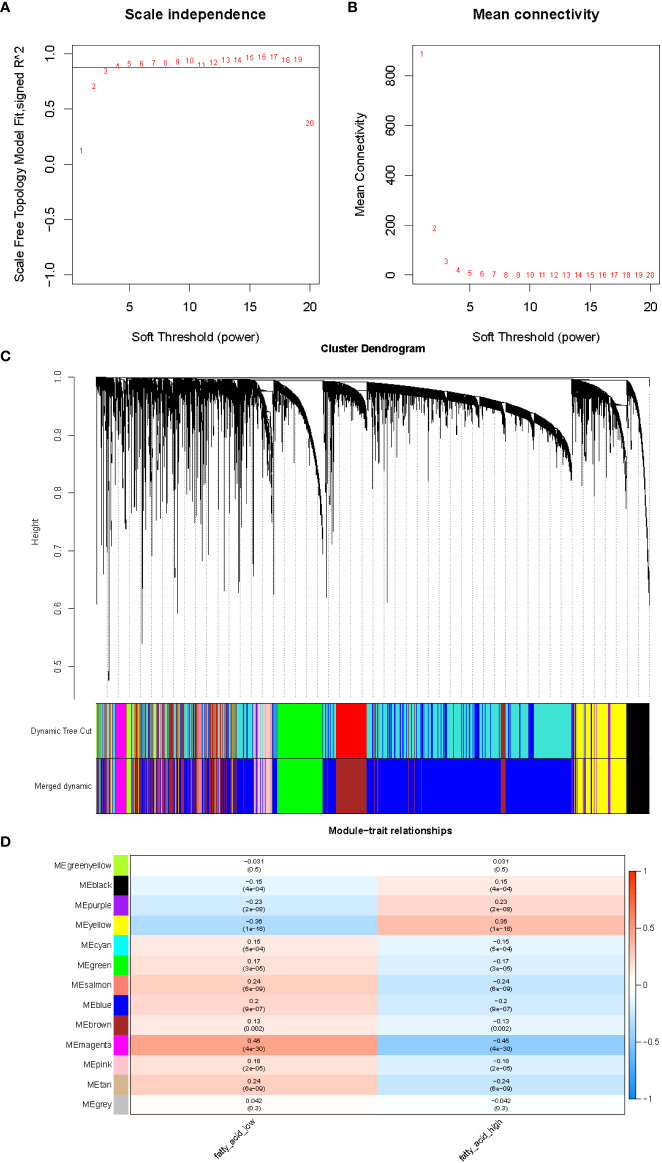
The evaluation of the weighted gene co-expression network. **(A)** Analysis of network topology for various soft-thresholding powers. The left panel shows the scale-free fit index (y-axis) as a function of the soft-thresholding power (x-axis). **(B)** The right panel displays the mean connectivity (degree, y-axis) as a function of the soft-thresholding power (x-axis). **(C)** Clustering dendrogram of genes, with dissimilarity based on topological overlap, together with assigned merged module colors and the original module colors. **(D)** Module-trait associations. Each row corresponds to a module eigengene, column to a trait. Each cell contains the corresponding correlation and p-value. The table is color-coded by correlation according to the color legend.

After integration of the obtained expression profiles of 525 FMA genes in TCGA-LUAD (497 LUAD and 58 normal samples), the FMA score was calculated for each sample by the ssGSEA algorithm. All samples were divided into high and low FMA score groups based on the median value of this score (median value = 1.586049) (**Supplementary Table 1**). Following that, the lncRNA profiles of 497 LUAD tissue samples and 58 normal tissue samples from the TCGA were included into the WGCNA. In order to develop a scale-free co-expression network in accordance with the analysis, the β-value was established at 4, which scale-free R^2^ ≥ 0.85. (**Figure 1A**). After that, the hierarchical clustering technique and the dynamic cutting algorithm were used to produce a total of 12 modules (gray modules were excluded because they were not assigned into any cluster) (**Figure 1B**). We estimated the correlation of FMA scores with WGCNA modules. Among the 12 modules, the yellow module (cor = ± 0.36, *P* = 1e-18; 781 lncRNAs) and magenta module (cor = ± 0.45, *P* = 4e-30; 147 lncRNAs) were highly correlated between groups with high and low FMA scores (**Figure 1C**). From these two modules, 928 lncRNAs (**Supplementary Table 4**) defined as FMA-related lncRNAs were selected for further analysis.

### Identification of the abnormally expressed FMA-related lncRNAs in LUAD

A normalized lncRNA expression matrix of 568 samples (510 LUAD and 58 normal) obtained from the TCGA database was selected as the basis for differential expression analysis. Using the R package limma, a total of 538 DE-lncRNAs satisfying |log_2_ FC| > 0.5 and adj. *P*< 0.05 was identified based on LUAD vs. normal, of which 298 were up-regulated and 240 were down-regulated (**Supplementary Table 5**).

**Figure 2:**
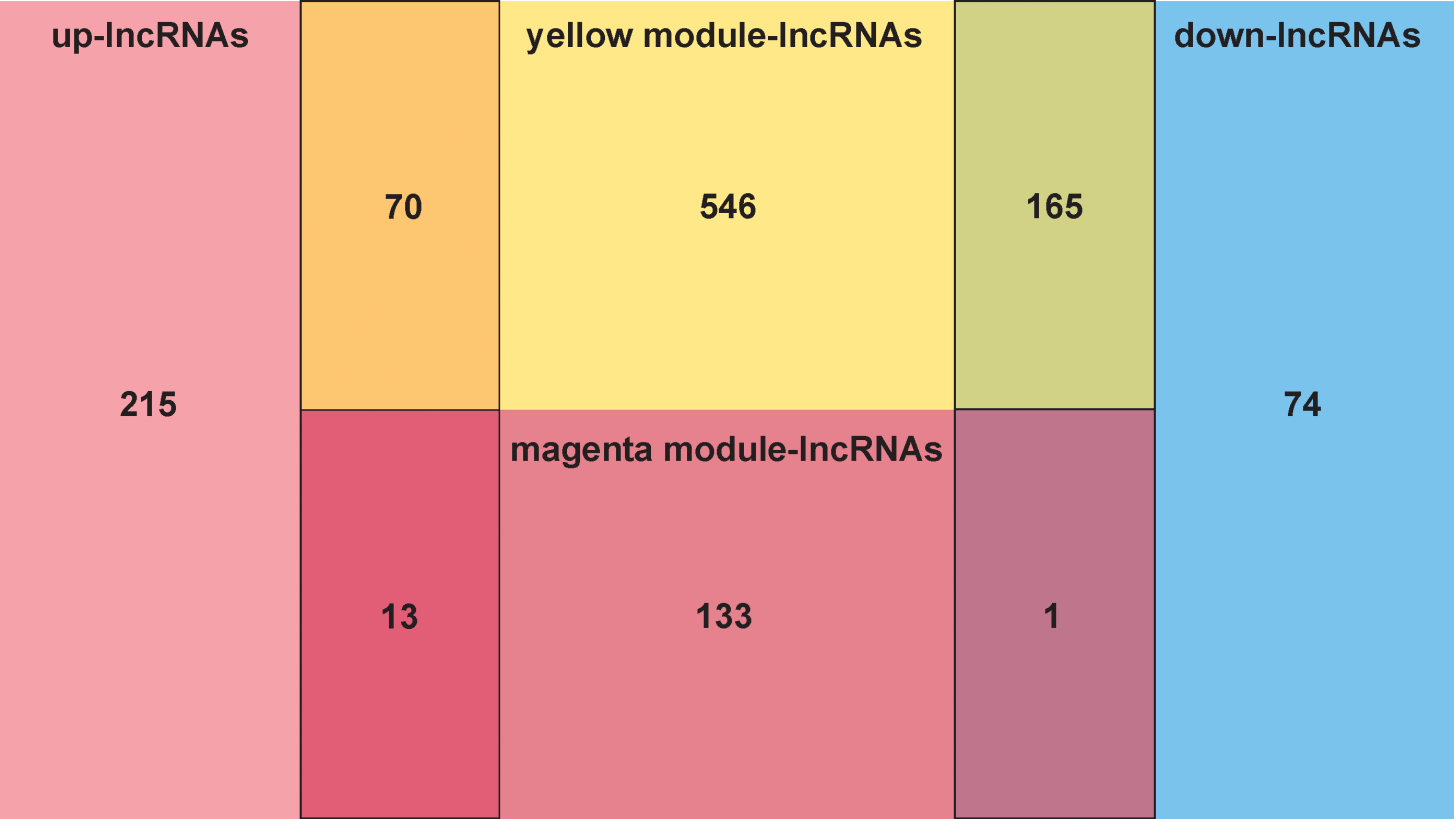
The overlap screening of lncRNAs associated with fatty acid metabolism and DE-lncRNAs showed a total of 249 common lncRNAs.

**Figure 3:**
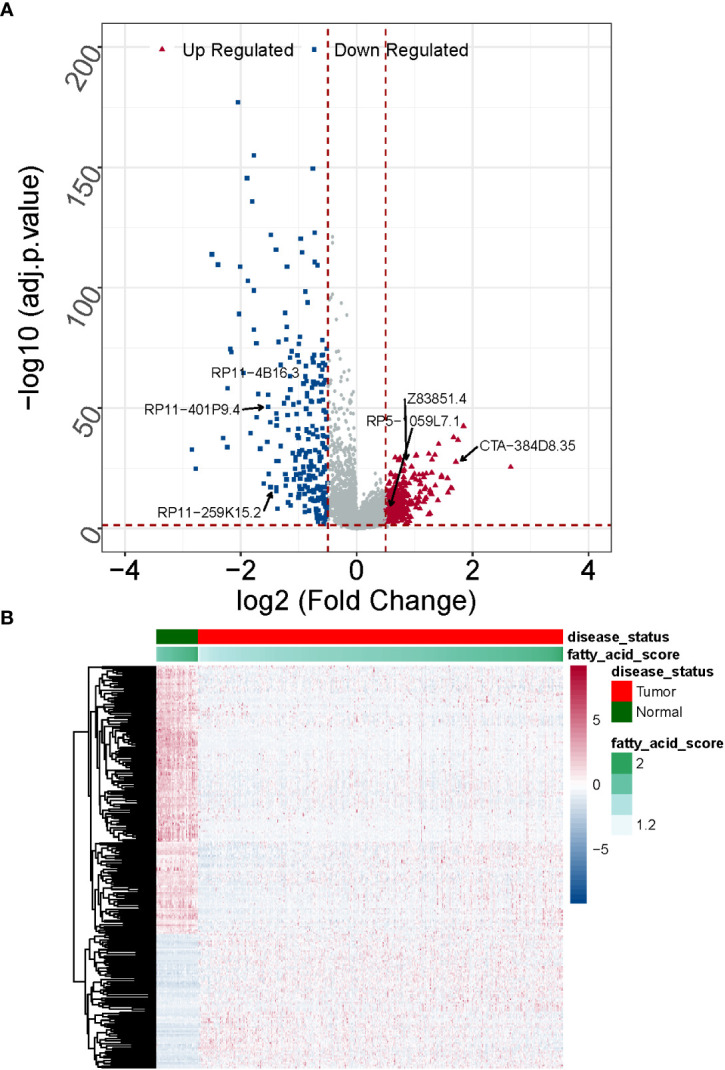
Expression patterns of DE-fatty acid metabolism-related lncRNAs in normal and cancerous tissues are compared. **(A)** A clustering analysis based on DE-fatty acid metabolism-related lncRNAs was shown in Heatmap. **(B)** The Volcano plot displays substantially differently expressed lncRNAs associated with fatty acid metabolism. .

The overlap analysis of FMA-related lncRNAs and DE-lncRNAs identified a total of 249 common lncRNAs (**Figure 2**; **Supplementary Table 6**), of which 83 were up-regulated and 166 were down-regulated (**Figure 3**), defining these lncRNAs as DE-FMA-related lncRNAs in LUAD.

### Construction of a prognostic signature on the FMA-related lncRNA

**Figure 4:**
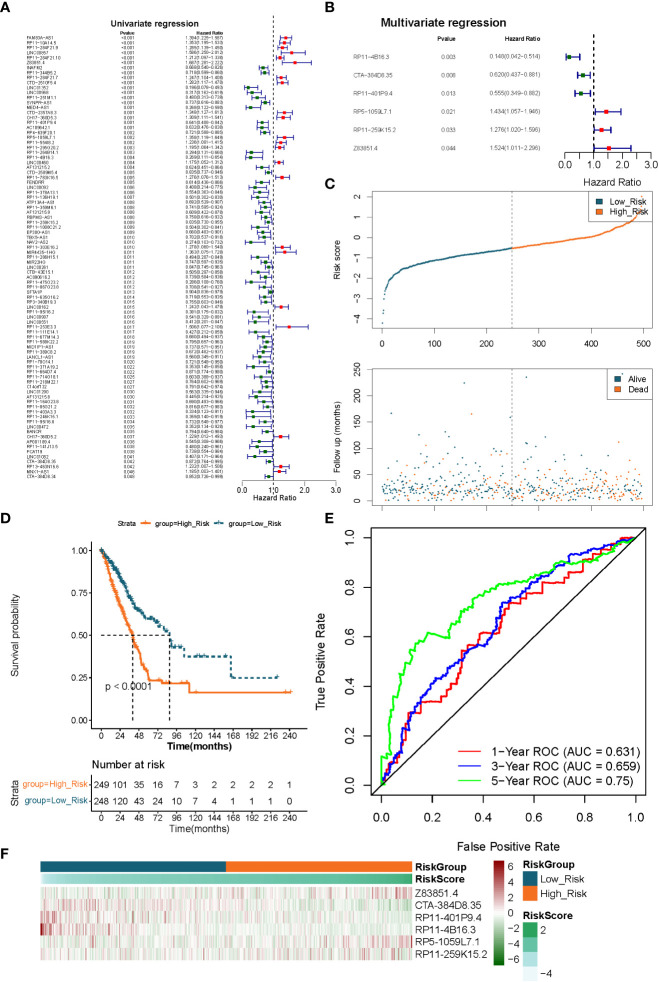
Extraction of the prognostic signature of fatty acid metabolism-related lncRNAs in LUAD. **(A)** The prognostic lncRNAs identified using a univariate Cox regression model. **(B)** The prognostic lncRNAs identified using a multivariable Cox regression model. **(C)** Distributions of risk score, survival status, and hallmark gene expression profiles. **(D)** Kaplan–Meier plot of overall survival for patients in low-risk and high-risk categories according to a prognostic classifier based on fatty acid metabolism in the TCGA cohort. **(E)** The accuracy of the risk score’s ability to predict patient outcomes at 1, 3, and 5 years was evaluated using ROC curves. **(F)** Clustering analysis based on predictive fatty acid metabolism-related lncRNAs was shown in Heatmap for the TCGA cohort.

We identified the optimal prognostic lncRNAs and constructed a prognostic signature by Cox regression analysis in the TCGA-LUAD (n = 497) dataset. Eighty-nine lncRNAs significantly associated with survival in LUAD patients were identified from 249 DE-FMA-related lncRNAs by univariate Cox regression analysis with a significance threshold of *P*< 0.05 (**Figure 4A**; **Supplementary Table 7**). Subsequently, we employed multivariate Cox regression analysis to recognize the optimal variables from the above 89 lncRNAs. According to *P*< 0.05, lncRNA RP11-4B16.3 (*P* = 0.003), lncRNA CTA-384D8.35 (*P* = 0.008), lncRNA RP11-401P9.4 (*P* = 0.013), lncRNA RP5-1059L7.1 (*P* = 0.021), lncRNA RP11 -259K15.2 (*P* = 0.033), and lncRNA Z83851.4 (*P* = 0.043984) were filtered out as the best prognostic variables (**Figure 4B**). Where HR > 1 for lncRNA Z83851.4 (HR = 1.524, 95% CI: 1.011-2.296), lncRNA RP5-1059L7.1 (HR = 1.434, 95% CI: 1.057-1.946), and lncRNA RP11-259K15.2 (HR = 1.276, 95% CI: 1.020-1.596) may be pro-LUAD progression genes; while with HR< 1, the lncRNAs RP11-4B16.3 (HR = 0.148, 95% CI: 0.042-0.514), lncRNA RP11-401P9.4 (HR = 0.555, 95% CI: 0.349-0.882), and lncRNA CTA-384D8.35 (HR = 0.620, 95% CI: 0.437-0.881) were the potential suppressor oncogenes (**Supplementary Table 8**). Therefore, we constructed a prognostic signature related to lipid metabolism based on the above six lncRNAs.

### Evaluation and validation of a risk model based on 6 lncRNAs

We utilized a risk scoring system to evaluate and validate the efficacy of the constructed 6 lncRNAs-based signature for prognostic prediction of LUAD patients in the TCGA-LUAD (n = 497) and GSE31210 (n = 226) datasets, respectively. Risk scores were calculated for each sample in the TCGA-LUAD dataset according to the previously described formula, and the samples were divided into high- (n = 249) low- (n = 248) risk groups based on the median value of the risk scores. Risk curves and scatter plots of patient survival distributions showed that patient death clustered with increasing risk scores (**Figure 4C**). K-M survival curves demonstrated that the risk score could significantly differentiate the prognosis of LUAD patients, with high-risk scores being inextricably linked to poor prognosis in LUAD patients (*P*< 0.0001; **Figure 4D**). ROC curves were applied to assess the accuracy of the risk score’s ability to predict patient prognosis. The results were presented in **Figure 4E**, and the AUC of the risk score in predicting patients’ OS at 1, 3, and 5 years was 0.631, 0.659, and 0.750, respectively, demonstrating an acceptable prognostic predictive validity. Moreover, the prognostic lncRNA expression heatmap revealed that lncRNA Z83851.4, lncRNA RP5-1059L7.1, and lncRNA RP11-259K15.2 were highly expressed in the high-risk group; while lncRNA CTA-384D8.35, lncRNA RP11-401P9.4, and lncRNA RP11-4B16.3, on the other hand, were overexpressed in the low-risk group (**Figure 4F**).

**Figure 5:**
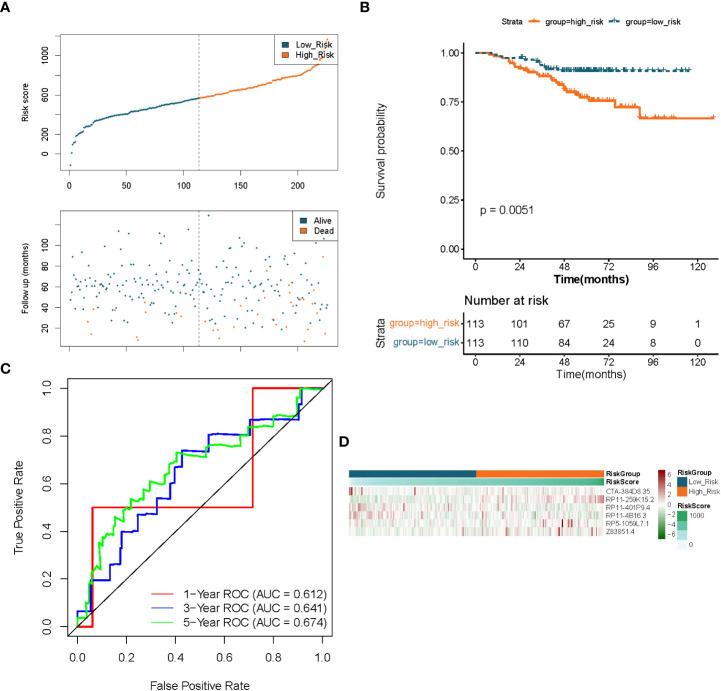
Validation of the prognostic signature of lncRNAs associated to fatty acid metabolism in LUAD. **(A)** Distributions of risk score, survival status, and expression profiles for hallmark genes. **(B)** Kaplan–Meier plot of overall survival for patients in low-risk and high-risk categories based on the fatty acid metabolism prognostic classifier in the GEO cohort. **(C)** ROC curves were used to evaluate the risk score’s capacity to accurately predict patient outcomes at 1, 3, and 5 years in the GEO cohort. **(D)** Heatmap in the GEO cohort displays clustering analysis based on prognostic fatty acid metabolism-related lncRNAs.

To demonstrate the applicability of the six lncRNAs-based prognostic signature, we performed the same analysis as described above in the GSE31210-LUAD dataset (independent validation set). Using the expression profiles of the 6 lncRNAs in the GSE31210 dataset, risk scores for each LUAD sample in the independent validation set were obtained by the aforementioned formula and divided into high- (n = 113) and low- (n = 113) risk groups based on median values. The prognostic predictive performance of the risk score in the independent validation set was comparable to their performance in the TCGA dataset. **Figures 5A, B** indicated that patients in the low-risk group had a longer survival time and a greater likelihood of survival compared to the high-risk group. Meanwhile, the risk profile also showed a tolerable predictive accuracy in the independent validation set with AUC values of 0.612, 0.641, and 0.674 at 1, 3, and 5 years, respectively (**Figure 5C**). Furthermore, the expression patterns of prognostic lncRNAs in different risk groups were consistent with their expression in the TCGA dataset (**Figure 5D**).

### Relationship between the risk score and clinical characteristics

We divided TCGA-LUAD patients into different clinical subgroups based on clinical characteristics, including age subgroups (≤ 65 and > 65), gender subgroups (male and female), tissue origin subgroups (upper and lower pages), stage subgroups (stage i-ii and stage iii-iv), pathological T stage (T1 and T2-4), and pathological N stage (N1 and N1-3). The risk score was found to be remarkably correlated with pathological T and N stages by Wilcoxon rank-sum test. Detailly, in the pathological T-stage subgroup, risk score levels were considerably higher in the T2-4 group compared with the T1 group (*P*< 0.01; **Supplementary Figure 2A**); in the pathological N-stage subgroup, risk score levels were proportional to lymph node involvement, which was significantly upregulated in the N1-3 group (*P*< 0.01; **Supplementary Figure 2B**). However, the distribution of risk scores among other clinical characteristics subgroups was relatively uneventful (**Supplementary Figures 2C–F**). Subsequently, we performed a stratified survival analysis based on the above clinical characteristic’s subgroup information. The results indicated that the risk score distinguished significantly between the prognosis of the different clinical subgroups (except for the T1 group), with a low-risk score implying a relatively better prognosis (**Supplementary Figure 3**).

### Functional enrichment analysis based on the risk score

**Figure 6:**
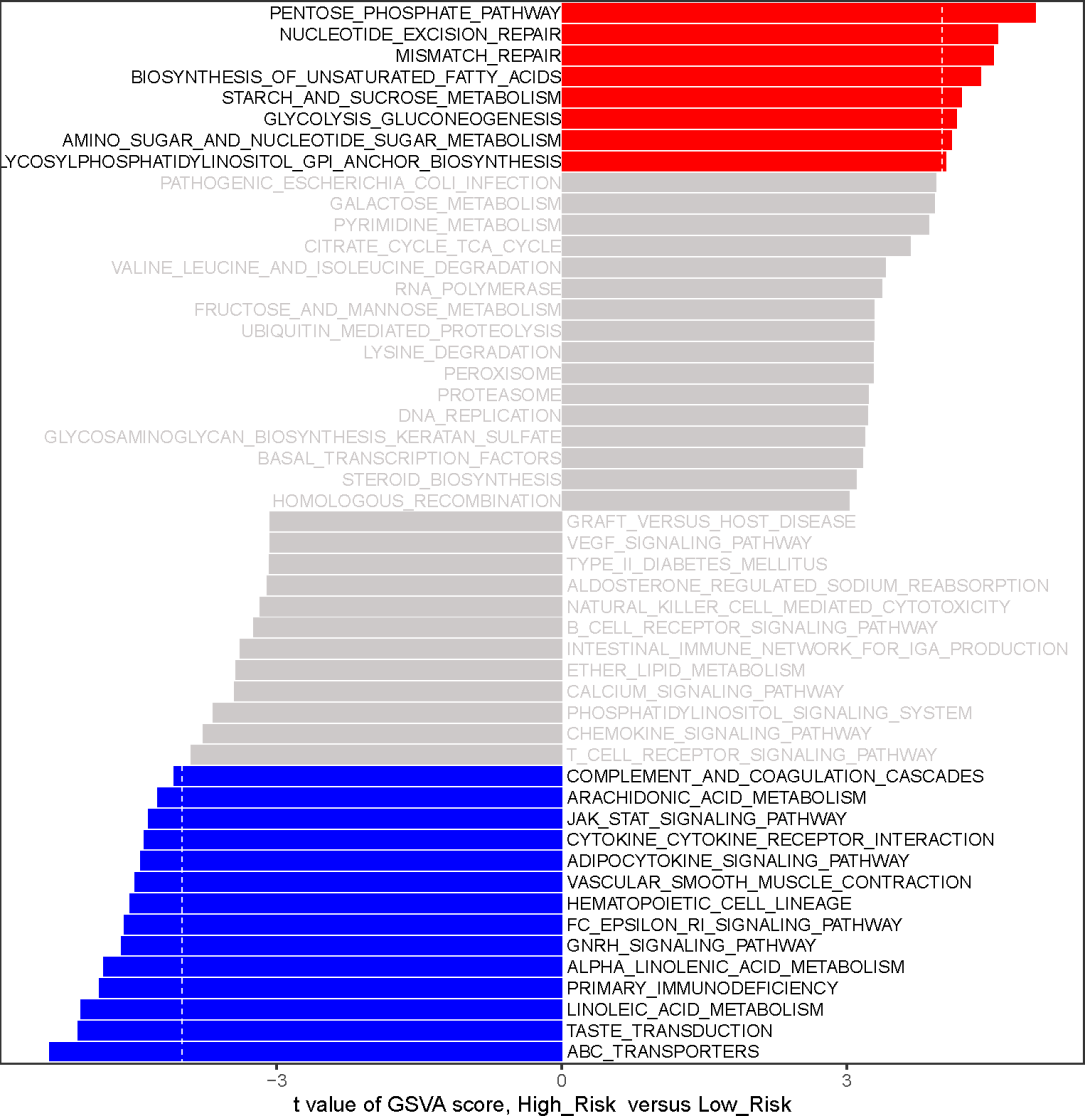
The analysis of gene-set variation (GSVA) indicates functional differences between high- and low-risk subgroups. Variations in pathway activity evaluated by GSVA between patients at high and low risk. The T values are shown using a linear model. We establish |t| > 4 and P value< 0.05 as the cutoff value. The red column represents active pathways in individuals at high risk, whereas the blue column represents activated pathways in patients at low risk.

To initially reveal the potential mechanisms by which risk score affects prognosis in LUAD patients, we performed a GSVA by R package GSVA. 22 KEGG pathways were identified to be substantially differentially enriched between the high-risk group and the low-risk group based on the comparison of the high-risk group to the low-risk group at |t| > 4 and adj. *P*< 0.05.(**Figure 6**; **Supplementary Table 9**). Pathways ‘PENTOSE PHOSPHATE PATHWAY’, ‘STARCH AND SUCROSE METABOLISM’, ‘GLYCOLYSIS GLUCONEOGENESIS’, ‘AMINO SUGAR AND NUCLEOTIDE SUGAR METABOLISM’, and ‘GLYCOSYLPHOSPHATIDYLINOSITOL GPI ANCHOR BIOSYNTHESIS’ with t > 4 were markedly activated in the high-risk group compared to the low-risk group; notably, high-risk patients might be implicated in the upregulation of ‘NUCLEOTIDE EXCISION REPAIR’, ‘MISMATCH REPAIR’, and ‘BIOSYNTHESIS OF UNSATURATED FATTY ACIDS’. Comparatively, the low-risk group was preferentially associated with ‘ABC TRANSPORTERS’, ‘TASTE TRANSDUCTION’, and ‘LINOLEIC ACID METABOLISM’. Moreover, PRIMARY IMMUNODEFICIENCY, CYTOKINE RECEPTOR INTERACTION, ARACHIDONIC ACID METABOLISM, and ADIPOCYTOKINE SIGNALING PATHWAY were upregulated in the low-risk group. The above evidence suggested that the risk score was possibly involved in regulating fatty acid synthesis/metabolism, gene repair, and immune/inflammatory responses in the LUAD process.

### Analysis of the immune landscape of LUAD patients based on the risk score

**Figure 7:**
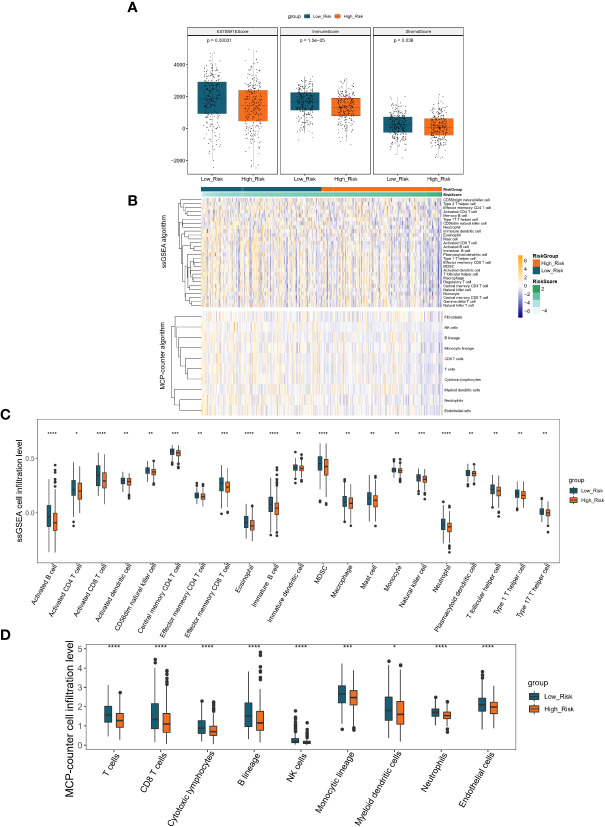
Immune scores and stromal scores correlate with subtypes of LUAD. **(A)** The P values for the distribution of immune scores in high and low risk groups, stromal scores in histological types, and ESTIMATE scores in histological types are all less than 0.05. **(B)** Heatmap displaying the percentage of 28 immune cells and 10 immune cells in the TCGA-LUAD sample utilizing ssGSEA and MCP-counter, respectively. **(C)** The violin plot of various immune cell infiltration levels between high-risk and low-risk patients as determined by ssGSEA. **(D)** The violin plot of varied immune cell infiltration levels between high-risk and low-risk patients as assessed by MCP-counter. *P < 0.05; **P < 0.01;***P < 0.001;****P < 0.0001.

Inspired by the above results, we proposed to assess the differences in the immune microenvironment between risk groups using the ESTIMATE, ssGSEA, and MCP-counter algorithms. According to the ESTIMATE methodology, the immunological score, the stromal score, and the ESTIMATE score were all considerably lower in the high-risk group in comparison to the low-risk group (all *P*< 0.05; **Figure 7A**). Subsequently, we evaluated the proportion of 28 immune cells and 10 immune cells in the TCGA-LUAD sample (n = 497) using the ssGSEA and MCP-counter algorithms, respectively (**Figure 7B**). According to the outcome of the t-test, the abundance of immune cell infiltration was substantially different between the groups of patients who were at high risk and those who were at low risk. In the ssGSEA analysis, 21 (Neutrophil, Activated B cell, Eosinophil, Activated CD8 T cell, Immature, B cell, MDSC, Effector memeory CD8 T cell, Natural killer cell, Central memory CD4, T cell, CD56dim natural killer cell, Activated dendritic cell, Monocyte, T follicular, helper cell, Type 1 T helper cell, Effector memeory CD4 T cell, Type 17 T helper cell, Macrophage, Immature dendritic cell, Mast cell, Plasmacytoid dendritic cell, and Activated CD4 T cell) of the 28 immune cells were significantly different between the high- and low-risk groups, and surprisingly, all of these differential immune cells were more infiltrative in the low-risk group (all *P*< 0.05; **Figure 7C**). Similarly, in the MCP-counter analysis, nine immune cells (T cells, NK cells, Neutrophils, Cytotoxic, lymphocytes, B lineage, CD8 T cells, Endothelial cells, Monocytic lineage, and Myeloid dendritic cells) with significantly different levels of infiltration were also significantly higher in the low-risk group (all *P*< 0.05; **Figure 7D**). These results implied that patients in the high-risk group associated with poor prognosis may be characterized by immune deficiency.

### Analysis of the ceRNA mechanism of prognostic lncRNAs

To further investigate the regulatory mechanisms of prognostic lncRNAs, we combined differential expression analysis, LncBase V2.0 database, miRTarBase database, and lncRNA-mRNA co-expression analysis to construct a prognostic lncRNA-miRNA-mRNA ceRNA regulatory network.

**Figure 8:**
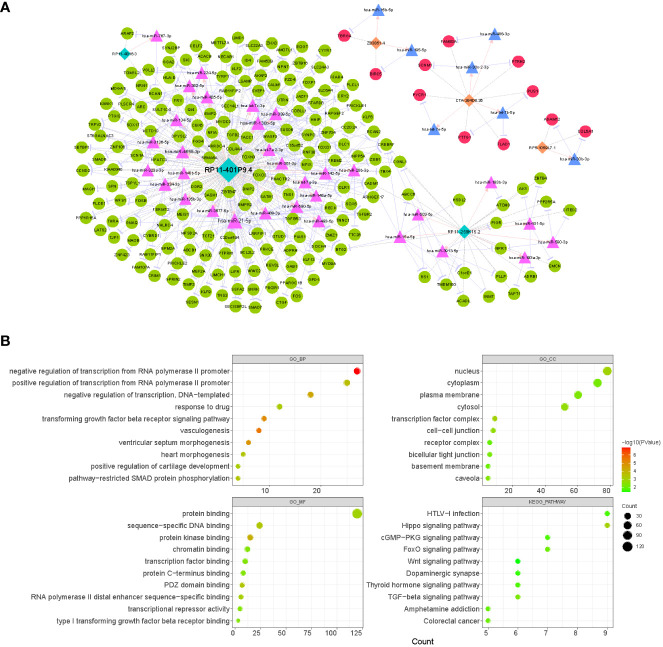
Analysis of the CeRNA network and function enrichment. **(A)** Interactions of LncRNA–miRNA–mRNA in LUAD. Orange rectangles represent up-regulated lncRNAs, light blue rectangles represent down-regulated lncRNAs, red circles indicate up-regulated mRNAs, green circles indicate down-regulated mRNAs, pink triangles represent up-regulated miRNAs, and light blue triangles represent down-regulated miRNAs. **(B)** The bubble plots displaying GO and KEGG enrichment data for mRNAs in ceRNA. The size of the dot denoted the gene count, and the color of the dot indicated the P value.

Based on the TCGA database, 5262 mRNAs (**Supplementary Table 2**) and 333 miRNAs (**Supplementary Table 3**) that were aberrantly expressed in LUAD were obtained by R package limma with the difference threshold set to |log_2_ FC| > 0.5 and adj. *P*< 0.05 (LUAD vs. normal). Subsequently, a total of 49 lncRNA-miRNA relationship pairs (6 lncRNAs and 49 miRNAs; **Supplementary Table 10**) were obtained by screening DE-miRNAs that interacted with prognostic lncRNAs and had opposite expression trends using the LncBase V2.0 database with a score > 0.6 as the criterion. Meanwhile, through the miRTarBase database, we obtained 9021 miRNA-mRNA relationship pairs for 322 DE-miRNAs and 2624 DE-mRNAs (miRNAs were expressed in opposite trends to mRNAs; **Supplementary Table 11**). Subsequently, co-expression analysis of prognostic lncRNAs with DE-mRNAs was performed using Pearson correlation analysis, and 2173 lncRNA-mRNA co-expression relationships were obtained based on r > 4 and p< 0.05, which contained 1380 DE-mRNAs and 6 prognostic lncRNAs (**Supplementary Table 12**). Finally, we obtained a total of 279 lncRNA-miRNA-mRNA ceRNA mechanisms (**Supplementary Table 13**) by combining the above obtained lncRNA-miRNA relationship pairs, miRNA-mRNA relationship pairs, and lncRNA-mRNA co-expression relationship pairs, which contained 6 prognostic lncRNAs, 39 miRNAs, and 201 mRNAs (**Figure 8A**). Concretely, the lncRNA CTA-384D8.35 (up-regulated) was able to regulate the expression of 7 mRNAs (up-regulated) by competitive binding with 4 miRNAs (down-regulated). lncRNA RP11-259K15.2 (down-regulated) controlled the expression of 22 mRNAs (down-regulated) by competitive binding with 7 miRNAs (up-regulated). The lncRNA RP11-401P9.4 (down-regulated) with 24 miRNAs (up-regulated) and 171 mRNAs (down-regulated) comprised 239 ceRNA regulatory mechanisms. The lncRNA RP11-4B16.3 (down-regulated) could regulate the expression of ARAP2 and RCAN1 (down-regulated) through the sponge hsa-miR-767-3p (up-regulated). The lncRNA RP5-1059L7.1 (up-regulated) could combine with the sponge hsa-miR-30b-3p (down-regulated) to regulate the expression of ADAM12 and COL5A1 (up-regulated). lncRNA Z83851.4 (up-regulated) was found to incorporate with 2 sponge miRNAs (hsa-miR-15b-5p and hsa-miR-195-5p; down-regulated) to regulate the expression of TBRG4 and BIRC5, respectively (up-regulated). Overall, the three prognostic lncRNAs upregulated in LUAD, lncRNA CTA-384D8.35, lncRNA Z83851.4, and lncRNA RP5-1059L7.1 had relatively independent ceRNA regulatory relationships. Whereas, lncRNA RP11-401P9.4, lncRNA RP11-259K15.2, and lncRNA RP11-4B16.3 had crosstalk among their ceRNA mechanisms. Detailly, RCAN1 could be competitively regulated by both lncRNA RP11-4B16.3-hsa-miR-767-3p and lncRNA RP11-401P9.4-hsa-miR-130b-5p/hsa-miR-134-5p/hsa-miR-339-5p/hsa-miR-4668-3 binding mode; lncRNA RP11-259K15.2-hsa-miR-15a-5p and lncRNA RP11-401P9.4-hsa-miR-21-5p/hsa-miR-339-5p/hsa-miR-590-5p both regulated the expression of BTG2; CADM1 was regulated by lncRNA RP11-259K15.2-hsa-miR-15a-5p and lncRNA RP11-401P9.4-hsa-let-7a-2-3p/hsa-let-7g-3p/hsa-miR-21-5p/hsa-miR-361-3p; lncRNA RP11-259K15.2 and lncRNA RP11-401P9.4 competitive binding to hsa-miR-3913-5p and hsa-miR-130b-5p, respectively, could act on CGNL1 simultaneously; CREBRF could be regulated by lncRNA RP11-259K15.2-hsa-miR-15a-5p/hsa-miR-503-5p, also regulated by lncRNA RP11-401P9.4-hsa-let-7c-3p/hsa-miR-142-5p/hsa-miR-20b-3p.

Moreover, to reveal the potential functions of the ceRNA network, we extracted 201 mRNAs from the network and performed functional enrichment analysis (**Figure 8B**; **Supplementary Table 14**). GO analysis revealed that these genes were enriched for a total of 67 biological processes (BP), 13 cellular components (CC), and 30 molecular functions (MF) terms. In the BP category, we found that these genes were significantly enriched in biological processes related to RNA (‘negative regulation of transcription from RNA polymerase II promoter’, ‘positive regulation of transcription from RNA polymerase II promoter’, ‘regulation of transcription from RNA polymerase II promoter’, *etc.*)/DNA (‘negative regulation of transcription, DNA-templated’, ‘positive regulation of transcription, DNA-templated’, ‘transcription, DNA-templated’, *etc.*) transcription, angiogenesis (‘vasculogenesis’, ‘blood vessel development’, ‘angiogenesis’, *etc.*), cell proliferation (‘BMP signaling pathway’, ‘negative regulation of cell proliferation’, ‘negative regulation of canonical Wnt signaling pathway’, ‘positive regulation of cell proliferation’, *etc.*), and organ/tissue formation (‘heart morphogenesis’, ‘heart development’, ‘lung alveolus development’, ‘tricuspid valve morphogenesis’, *etc.*); the enrichment results in the CC and MF categories suggested that these genes may serve molecular functions for protein (‘protein kinase binding’, ‘protein binding’, ‘protein C-terminus binding’, *etc.*), RNA/DNA (‘sequence-specific DNA binding’, ‘sequence-specific DNA binding’, ‘RNA polymerase II distal enhancer sequence-specific binding’, *etc.*), and transcription factor (‘transcription factor binding’) binding in a variety of membrane (‘plasma membrane’, ‘basement membrane’, ‘apical plasma membrane’, *etc.*), plasma (‘cytosol’ and ‘cytoplasm’), nuclear (‘nucleus’ and ‘nuclear body’), and complexes (‘transcription factor complex’ and ‘receptor complex’). KEGG analysis revealed that these genes were involved in a total of 14 pathways, of which several pathways that were shown to be associated with multiple cell physiological events (e.g., cell proliferation and apoptosis) were significantly enriched, such as ‘Hippo signaling pathway’, ‘TGF-beta signaling pathway’, ‘FoxO signaling pathway’, and ‘Wnt signaling pathway’. In addition, they were involved in ‘Pathways in cancer’ and ‘MicroRNAs in cancer’ pathways. This evidence indicated that prognostic lncRNAs may be involved in the process of LUAD by regulating genomic transcription, influencing the cell cycle, and modulating tissue and organogenesis.

### Expression validation of 6 prognostic lncRNAs

**Figure 9:**
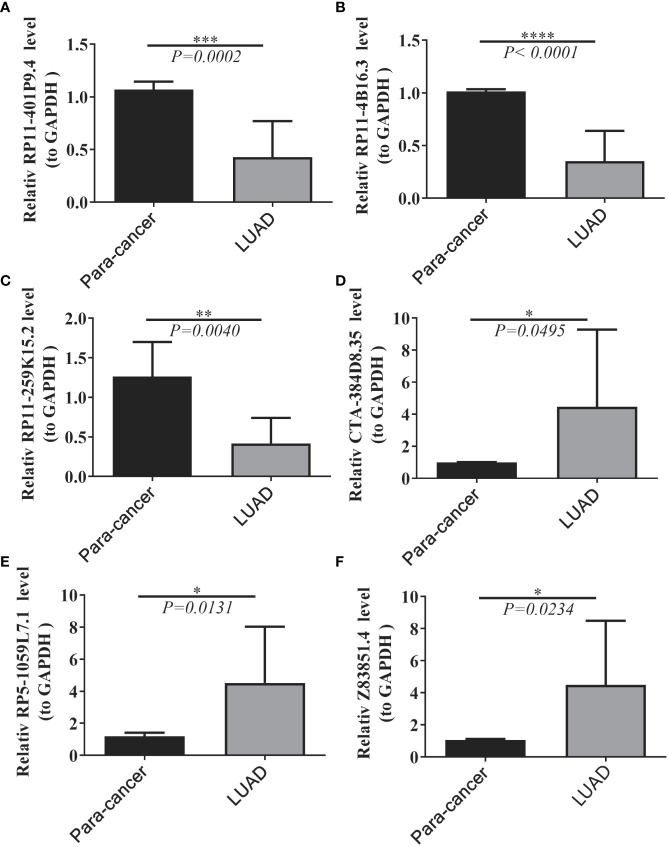
Expression levels of six lncRNAs between LUAD and normal tissues. **(A–F)** Results of quantitative real-time PCR for the six lncRNAs. The expression of hub genes was standardized relative to the expression of GAPDH. The significance of differences was determined using the Student’s t-test; *P<0.05, **P<0.01, ***P<0.001.

We collected 20 samples and para-cancer samples from 10 pairs of LUADs and elucidated the expression changes of selected prognostic lncRNAs in LUADs by qRT-PCR. Six lncRNAs were significantly differentially expressed between LUAD and paraneoplastic samples. The expression levels of lncRNA RP11-401P9.4, RP11-4B16.3, and lncRNA RP11-259K15.2 were remarkably reduced in LUAD samples compared with paraneoplastic samples (**Figures 9A–C**); whereas lncRNA CTA-384D8.35, lncRNA RP5-1059L7.1, and lncRNA Z83851.4 were notably up-regulated in LUAD samples (**Figures 9D–F**), which was consistent with the results in the TCGA-LUAD dataset.

## Discussion

Lung cancer has the highest mortality rate of all malignancies, accounting for more than a quarter of all cancer deaths (36). Despite recent advances in lung cancer treatment, the patient survival rate remains bleak (37). LUAD is among the most common kinds of lung cancer (38). Therefore, it is vital to discover other prognostic characteristics for LUAD. To improve prognosis assessment and individualized treatment, it is essential to research potential biological pathways and find reliable prognostic biomarkers. Researchers are beginning to comprehend the significance of FAM in relation to tumorigenesis, development, drug resistance, and prognosis in LUAD as a consequence of rigorous studies on metabolic reprogramming (39, 40). We have a limited understanding of the connection between lncRNA and FAM in LUAD. To determine the prognostic pattern of LUAD, a network of FAM-related ceRNAs and six FAM-related lncRNAs were created. The prediction capability was enhanced by combining prognostic features with clinical characteristics to develop risk molde with great repetition and reliability. A prognostic signature based on lncRNAs related with FAM may be used to stratify the prognosis of LUAD patients, according to the current study. These prognostic markers will aid in the elucidation of the molecular mechanisms behind LUAD and offer innovative FAM-targeted treatment options.

Several studies have been conducted to develop different types of prognostic models for patients with LUAD, addressing ferroptosis (41–43), methylation (44–46), immune-related genes (47–49), and the tumor microenvironment (TME) (43, 50, 51) and so on, and to elucidate the underlying mechanisms of LUAD. The present study is based on the relationship between FAM and LUAD (52), and research in this area is still limited. Moreover, as essential regulators of several physiological and pathological processes, lncRNAs have a crucial role in regulating FAM in diverse malignancies through ceRNA-related pathways (18, 53, 54). Therefore, in this study, the prognostic performance of FAM-associated lncRNAs in LUAD was extensively investigated. Based on univariate and multi-causal Cox regression analyses, a lncRNA signature consisting of six lncRNAs (i.e. lncRNA RP11-4B16.3, lncRNA CTA-384D8.35, lncRNA RP11-401P9.4, lncRNA RP5-1059L7.1, lncRNA RP11-259K15.2, and lncRNA Z83851.4) as important independent prognostic factors. Tang et al. (55) discovered that CTA-384D8.35 is a crucial survival gene in individuals with pancreatic ductal adenocarcinoma. CTA-384D8.35 was considerably enhanced in the expression profile of peripheral blood mononuclear cells in individuals with primary Sjogren’s disease, according to Peng et al. (56). Based on the mechanism by which Rho-GTPase lead to activation protein 30 (ARHGAP30) may enhance the intrinsic hydrolysis of GTP and negatively regulate Rho-GTPase, ARHGAP30 can promote the hydrolysis of GTP. Hu et al. (57) investigated the relationship between ARHGAP30 expression and LUAD and identified survival curves of LUAD patients with a more favorable prognosis for Z83851.4 with low ARHGAP30 expression. The mechanism of the other LUAD lncRNAs is yet unknown.

A scoring model was established in the TCGA training cohort for the expression levels of six lncRNAs related with metabolism in normal and malignant tissues. An independent prognostic score method was used to predict OS in individuals with LUAD. With similar findings, we validated the model using the TCGA test cohort and the whole GEO cohort. We performed clinical correlation studies to better understand the significance of high- and low-risk models in LUAD and discovered that survival was substantially different between high- and low-risk groups, with the high-risk group having a dismal prognosis. To further comprehend the processes behind differential prognosis between high- and low-risk groupings, we used GSVA to identify variations in immunological state between these subgroups. Energy metabolism pathways, such as the pentose phosphate pathway, are abundant in the high-risk group. Liu et al. (58) discovered that LUAD patients with a poor prognosis are predominantly enriched in the pentose phosphate pathway, which is consistent with the findings of the current investigation. Moreover, unlike protein-coding genes, the majority of pentose phosphate-related lncRNAs were negatively correlated with pentose phosphate activity and were linked with a poor prognosis (59, 60).

Taking into account the heterogeneity of the TME in LUAD, we found variations between high- and low-risk groups using TME patterns derived by Estimate, ssGSEA, and MCP-counter methods. The study revealed that the low-risk group had a greater number of immune cell infiltrations than the high-risk group. In addition, the low-risk group had a better clinical prognosis than the high-risk group. We assumed that the low-risk group was the immune-response-activatied subtype and the high-risk group was the immune-response-suppressed subtype. TME is recognized to have a crucial role in lipid metabolism between cancer cells and the full populations of immune and stromal cells (61). The TME contains both immunosuppressive and activating cells, and tumor infiltrates are very varied based on the kind of cancer or patient model. T-cell infiltration is a strong prognostic indicator and has been used in the treatment of several malignancies (62). Studies have shown the beneficial effect of T lymphocytes on tumor development (63), and their absence from the TME results in immunological privilege (64). Moreover, the significant infiltration of CD8 T cells and active CD8 T cells indicated a favorable prognosis and suggested that activating these cells in the TME may have therapeutic benefits. Immunosuppressive cells, such as tumor-associated macrophages (TAMs) and myeloid-derived suppressor cells (MDSCs), have a substantial impact on the longevity of LUAD patients (65, 66).

Moreover, as essential regulators of several physiological and pathological processes, lncRNAs play crucial roles in regulating FMA in different malignancies through ceRNA-related pathways (18, 53, 67). We conducted a functional enrichment analysis on the all mRNAs of the ceRNA network and discovered that they were mostly enriched in the Hippo signaling pathway and the FoxO signaling circuit. Several studies have shown that the modulation of the Hippo signaling pathway accelerates the advancement of LUAD (68–71). This result shows that there may be a regulatory network of ceRNAs involving FAM-related lncRNAs that may potentially be involved in the regulation of the Hippo signaling pathway, which plays an important role in the progression of LUAD

In conclusion, we created and validated a risk score model for prognosis and risk stratification based on FAM-related lncRNAs in TCGA and GEO datasets. High predictive accuracy was found for 1-, 3-, and 5-year OS. To comprehend the pathogenic processes of LUAD, it is possible to target certain genes. In addition, GSEA, tumor immune infiltration, and other analyses suggested that FAM may have a role in carcinogenesis, progression, and tumor microenvironment. These findings indicate a potential treatment target for LUAD. However, it is important to acknowledge the study’s shortcomings. First, the predictive model used in this study was created utilizing data from a single source (TCGA). External, independent data sets and long-term follow-up are required to validate the predictive utility of our innovative LUAD model. As this was a retrospective research, data gaps and selection biases were unavoidable. Third, future research should expand on explicit processes.

## Conclusion

We created a 6 FAM-related lncRNA prognostic model to predict the OS of LUAD patients and were the first to build a FAM-related ceRNA network for LUAD, which may illuminate the molecular regulatory mechanism of FAM in LUAD. In addition, this study adds to our knowledge of the regulation of FAM-related lncRNAs in the course of LUAD and identifies novel potential biomarkers for diagnosis, prognosis, and treatment.

## Data availability statement

The datasets presented in this study can be found in online repositories. The names of the repository/repositories and accession number(s) can be found in the article/**Supplementary Material**.

## Ethics statement

The studies involving human participants were reviewed and approved by Ethics Committee of the Affiliated People’s Hospital of Jiangsu University (approval NO.K-20200097-Y). The patients provided their written informed consent to participate in this study.

## Author contributions

Y-QP conceived the manuscript. YX and TL wrote the manuscript. ChaoL, W-HG, and Y-YS conducted the statistical analysis. ChangL, Y-JS, and SL explain the results. All authors contributed to the article and approved the submitted version.

## Funding

This study was funded by the Doctoral Research Start-up Project Fund of the People's Hospital Affiliated to Jiangsu University, China (KFB2020004).

## Acknowledgments

We would like to thank to People's Hospital Affiliated to Jiangsu University for for providing samples.

## Conflict of interest

The authors declare that the research was conducted in the absence of any commercial or financial relationships that could be construed as a potential conflict of interest.

## Publisher’s note

All claims expressed in this article are solely those of the authors and do not necessarily represent those of their affiliated organizations, or those of the publisher, the editors and the reviewers. Any product that may be evaluated in this article, or claim that may be made by its manufacturer, is not guaranteed or endorsed by the publisher.

## Supplementary material

Supplementary Table 1 All samples were divided into high and low fatty acid metabolism score groups based on the median value of this score.

Supplementary Table 2 Differentially expressed mRNAs between LUAD and normal samples.

Supplementary Table 3 Differentially expressed miRNAs between LUAD and normal samples.

Supplementary Table 4 928 lncRNAs defined as fatty acid metabolism-related lncRNAs in the yellow and magenta modules.

Supplementary Table 5 Identification of differential expression of lncRNAs between LUAD and normal samples.

Supplementary Table 6 The overlap analysis of fatty acid metabolism-related lncRNAs and DE-lncRNAs.

Supplementary Table 7 Eighty-nine lncRNAs significantly associated with survival in LUAD patients were identified from 249 DE-fatty acid metabolism-related lncRNAs by univariate Cox regression analysis.

Supplementary Table 8 Multivariate Cox regression analysis to recognize the optimal lncRNAs.

Supplementary Table 9 Results of 22 KEGG pathways based on the high-risk group vs. the low-risk group.

Supplementary Table 10 Results of lncRNA-miRNA relationship pairs.

Supplementary Table 11 Results of miRNA-mRNA relationship pairs for 322 DE-miRNAs and 2624 DE-mRNAs.

Supplementary Table 12 Results of co-expression analysis of prognostic lncRNAs with DE-mRNAs was performed using Pearson correlation analysis.

Supplementary Table 13 Results of lncRNA-miRNA-mRNA ceRNA.

Supplementary Table 14 Results of mRNAs from the network and performed functional enrichment analysis in ceRNA network.

Supplementary Figure 1 The identification of outlier samples, the selection and validation of the best soft threshold power for gene coexpression network construction. **(A)** Clustering dendrogram of LUAD data used to identify outliers. **(B)** Clustering of genes having similar features.

Supplementary Figure 2 Variations in risk scores between clinical characteristics. **(A)** Variations in risk scores based on the T stage. **(B)** Variations in risk scores during the N stage. **(C)** Variations in risk score based on age. **(D)** Differences in risk score based on gender. **(E)** Variations in risk score based on the tissue of origin. **(F)** Variations in risk score based on tumor stage.

Supplementary Figure 3 Differences of survival between high- and low-risk groups based on clinical features. **(A)** Differences of survival between high-risk and low-risk groups for individuals under 65 years old. **(B)** Differences of survival between high-risk and low-risk groups for individuals over 65 years of age. **(C)** Differences of survival between high- and low-risk groups based on female. **(D)** Differences of survival between high- and low-risk groups based on male. **(E)** Differences of survival between high- and low-risk groups based on lower lobe. **(F)** Differences of survival between high- and low-risk groups based on upper lobe. **(G)** Differences of survival between high- and low-risk groups based on stage III-IV. **(H)** Differences of survival between high- and low-risk groups based on stage iii. **(I)** Differences of survival between high- and low-risk groups based on T1 stage. **(J)** Differences of survival between high- and low-risk groups based on T2-4 stage. **(K)** Differences of survival between high- and low-risk groups based on N13 stage. **(L)** Differences of survival between high- and low-risk groups based on N0 stage.

## Abbreviations

lncRNA, long non-coding RNA; LUAD, lung adenocarcinoma; TCGA, The Cancer Genome Atlas; ssGSEA, single-sample gene set enrichment analysis; WGCNA, weighted gene co-expression network; K-M, Kaplan-Meier; ROC, receiver operating characteristic; GSVA, Gene set variation analysis; MCP-counter, microenvironment cell populations-counter; ceRNA, competing endogenous RNA; GO, Gene Ontology; KEGG, Kyoto Encyclopedia of Genes and Genomes; SCLC, small cell lung cancer; NSCLC, non-small cell lung cancer; LUSC, lung squamous cell carcinoma; FAM, fatty acid metabolism; qRT-PCR, quantitative real-time polymerase chain reaction; OS, overall survival; BP, biological processes; CC, cellular components; MF, molecular functions.
