# Supplementary material for: Prognostic value of lncRNAs related to fatty acid metabolism in lung adenocarcinoma and their correlation with tumor microenvironment based on bioinformatics analysis
Source: Front Oncol. 2022 Oct 10;12:1022097. doi: 10.3389/fonc.2022.1022097 (PMC9590110; doi:10.3389/fonc.2022.1022097)
Supplement: Supplementary Table 1 — All samples were divided into high and low fatty acid metabolism score groups based on the median value of this score. [file DataSheet_1.zip › raw data and R code for checking/raw data/1.docx]

| samples | fatty_acid_score | disease_status | fatty_acid_group | median |
| --- | --- | --- | --- | --- |
| TCGA-50-6591-01A | 1.02630199 | Tumor | fatty_acid_score_low | 1.586049 |
| TCGA-91-6848-01A | 1.09909758 | Tumor | fatty_acid_score_low | 1.586049 |
| TCGA-78-7155-01A | 1.144773724 | Tumor | fatty_acid_score_low | 1.586049 |
| TCGA-86-8358-01A | 1.184969134 | Tumor | fatty_acid_score_low | 1.586049 |
| TCGA-91-6847-01A | 1.214254159 | Tumor | fatty_acid_score_low | 1.586049 |
| TCGA-83-5908-01A | 1.229517927 | Tumor | fatty_acid_score_low | 1.586049 |
| TCGA-91-A4BC-01A | 1.230597668 | Tumor | fatty_acid_score_low | 1.586049 |
| TCGA-55-7726-01A | 1.232782389 | Tumor | fatty_acid_score_low | 1.586049 |
| TCGA-55-A493-01A | 1.240002097 | Tumor | fatty_acid_score_low | 1.586049 |
| TCGA-38-4630-01A | 1.240698254 | Tumor | fatty_acid_score_low | 1.586049 |
| TCGA-50-6595-01A | 1.241200799 | Tumor | fatty_acid_score_low | 1.586049 |
| TCGA-44-2668-01A | 1.243646456 | Tumor | fatty_acid_score_low | 1.586049 |
| TCGA-44-5644-01A | 1.254893053 | Tumor | fatty_acid_score_low | 1.586049 |
| TCGA-50-6590-01A | 1.270498722 | Tumor | fatty_acid_score_low | 1.586049 |
| TCGA-55-6968-01A | 1.27351992 | Tumor | fatty_acid_score_low | 1.586049 |
| TCGA-64-5775-01A | 1.281270668 | Tumor | fatty_acid_score_low | 1.586049 |
| TCGA-MP-A4TI-01A | 1.282027998 | Tumor | fatty_acid_score_low | 1.586049 |
| TCGA-MP-A4TF-01A | 1.28282926 | Tumor | fatty_acid_score_low | 1.586049 |
| TCGA-91-6836-01A | 1.286996362 | Tumor | fatty_acid_score_low | 1.586049 |
| TCGA-MN-A4N4-01A | 1.289752079 | Tumor | fatty_acid_score_low | 1.586049 |
| TCGA-55-6978-01A | 1.306393178 | Tumor | fatty_acid_score_low | 1.586049 |
| TCGA-44-7662-01A | 1.308341272 | Tumor | fatty_acid_score_low | 1.586049 |
| TCGA-64-5779-01A | 1.310389867 | Tumor | fatty_acid_score_low | 1.586049 |
| TCGA-50-5946-01A | 1.314056575 | Tumor | fatty_acid_score_low | 1.586049 |
| TCGA-95-7944-01A | 1.315815726 | Tumor | fatty_acid_score_low | 1.586049 |
| TCGA-44-3918-01A | 1.317400476 | Tumor | fatty_acid_score_low | 1.586049 |
| TCGA-93-A4JQ-01A | 1.317540392 | Tumor | fatty_acid_score_low | 1.586049 |
| TCGA-44-7667-01A | 1.32278428 | Tumor | fatty_acid_score_low | 1.586049 |
| TCGA-44-6774-01A | 1.334993019 | Tumor | fatty_acid_score_low | 1.586049 |
| TCGA-91-6840-01A | 1.340771572 | Tumor | fatty_acid_score_low | 1.586049 |
| TCGA-86-8054-01A | 1.351678151 | Tumor | fatty_acid_score_low | 1.586049 |
| TCGA-49-6767-01A | 1.352188777 | Tumor | fatty_acid_score_low | 1.586049 |
| TCGA-MN-A4N5-01A | 1.353136061 | Tumor | fatty_acid_score_low | 1.586049 |
| TCGA-86-6851-01A | 1.353550361 | Tumor | fatty_acid_score_low | 1.586049 |
| TCGA-49-6761-01A | 1.353767498 | Tumor | fatty_acid_score_low | 1.586049 |
| TCGA-93-A4JN-01A | 1.356472683 | Tumor | fatty_acid_score_low | 1.586049 |
| TCGA-50-5931-01A | 1.357063531 | Tumor | fatty_acid_score_low | 1.586049 |
| TCGA-55-A48Z-01A | 1.36072046 | Tumor | fatty_acid_score_low | 1.586049 |
| TCGA-69-7980-01A | 1.362412699 | Tumor | fatty_acid_score_low | 1.586049 |
| TCGA-78-7542-01A | 1.362570045 | Tumor | fatty_acid_score_low | 1.586049 |
| TCGA-44-7661-01A | 1.363667027 | Tumor | fatty_acid_score_low | 1.586049 |
| TCGA-50-5933-01A | 1.366764874 | Tumor | fatty_acid_score_low | 1.586049 |
| TCGA-44-2665-01A | 1.368303869 | Tumor | fatty_acid_score_low | 1.586049 |
| TCGA-05-4427-01A | 1.368307361 | Tumor | fatty_acid_score_low | 1.586049 |
| TCGA-86-8673-01A | 1.371353335 | Tumor | fatty_acid_score_low | 1.586049 |
| TCGA-05-4398-01A | 1.371711354 | Tumor | fatty_acid_score_low | 1.586049 |
| TCGA-49-AAR3-01A | 1.371898294 | Tumor | fatty_acid_score_low | 1.586049 |
| TCGA-91-6831-01A | 1.372433879 | Tumor | fatty_acid_score_low | 1.586049 |
| TCGA-50-5044-01A | 1.377953381 | Tumor | fatty_acid_score_low | 1.586049 |
| TCGA-44-8119-01A | 1.37987373 | Tumor | fatty_acid_score_low | 1.586049 |
| TCGA-75-6214-01A | 1.380174061 | Tumor | fatty_acid_score_low | 1.586049 |
| TCGA-78-7146-01A | 1.384455044 | Tumor | fatty_acid_score_low | 1.586049 |
| TCGA-95-7567-01A | 1.385403927 | Tumor | fatty_acid_score_low | 1.586049 |
| TCGA-MP-A4T4-01A | 1.386756119 | Tumor | fatty_acid_score_low | 1.586049 |
| TCGA-L9-A8F4-01A | 1.387422121 | Tumor | fatty_acid_score_low | 1.586049 |
| TCGA-78-7536-01A | 1.388350874 | Tumor | fatty_acid_score_low | 1.586049 |
| TCGA-86-7701-01A | 1.388366799 | Tumor | fatty_acid_score_low | 1.586049 |
| TCGA-91-8499-01A | 1.388774285 | Tumor | fatty_acid_score_low | 1.586049 |
| TCGA-55-7994-01A | 1.38991785 | Tumor | fatty_acid_score_low | 1.586049 |
| TCGA-55-A490-01A | 1.393404672 | Tumor | fatty_acid_score_low | 1.586049 |
| TCGA-95-A4VN-01A | 1.39908247 | Tumor | fatty_acid_score_low | 1.586049 |
| TCGA-55-8204-01A | 1.400173006 | Tumor | fatty_acid_score_low | 1.586049 |
| TCGA-53-7624-01A | 1.400620438 | Tumor | fatty_acid_score_low | 1.586049 |
| TCGA-49-AAR4-01A | 1.401985285 | Tumor | fatty_acid_score_low | 1.586049 |
| TCGA-69-7978-01A | 1.403726247 | Tumor | fatty_acid_score_low | 1.586049 |
| TCGA-44-A47B-01A | 1.404843924 | Tumor | fatty_acid_score_low | 1.586049 |
| TCGA-64-5774-01A | 1.405999087 | Tumor | fatty_acid_score_low | 1.586049 |
| TCGA-55-8511-01A | 1.409112636 | Tumor | fatty_acid_score_low | 1.586049 |
| TCGA-55-8205-01A | 1.410520317 | Tumor | fatty_acid_score_low | 1.586049 |
| TCGA-55-8506-01A | 1.411188226 | Tumor | fatty_acid_score_low | 1.586049 |
| TCGA-64-1679-01A | 1.413084286 | Tumor | fatty_acid_score_low | 1.586049 |
| TCGA-MP-A4TC-01A | 1.413950048 | Tumor | fatty_acid_score_low | 1.586049 |
| TCGA-78-7159-01A | 1.414648962 | Tumor | fatty_acid_score_low | 1.586049 |
| TCGA-97-7554-01A | 1.415805993 | Tumor | fatty_acid_score_low | 1.586049 |
| TCGA-MP-A4SV-01A | 1.417235181 | Tumor | fatty_acid_score_low | 1.586049 |
| TCGA-50-7109-01A | 1.418491963 | Tumor | fatty_acid_score_low | 1.586049 |
| TCGA-75-5125-01A | 1.418569983 | Tumor | fatty_acid_score_low | 1.586049 |
| TCGA-62-8402-01A | 1.419121254 | Tumor | fatty_acid_score_low | 1.586049 |
| TCGA-49-AAR9-01A | 1.419645793 | Tumor | fatty_acid_score_low | 1.586049 |
| TCGA-86-7954-01A | 1.419885152 | Tumor | fatty_acid_score_low | 1.586049 |
| TCGA-55-6642-01A | 1.420184883 | Tumor | fatty_acid_score_low | 1.586049 |
| TCGA-L4-A4E5-01A | 1.420192558 | Tumor | fatty_acid_score_low | 1.586049 |
| TCGA-55-7910-01A | 1.420693668 | Tumor | fatty_acid_score_low | 1.586049 |
| TCGA-95-7562-01A | 1.421217081 | Tumor | fatty_acid_score_low | 1.586049 |
| TCGA-95-8494-01A | 1.421672144 | Tumor | fatty_acid_score_low | 1.586049 |
| TCGA-91-6835-01A | 1.425612824 | Tumor | fatty_acid_score_low | 1.586049 |
| TCGA-55-8614-01A | 1.425880386 | Tumor | fatty_acid_score_low | 1.586049 |
| TCGA-78-7535-01A | 1.427566907 | Tumor | fatty_acid_score_low | 1.586049 |
| TCGA-69-A59K-01A | 1.429883901 | Tumor | fatty_acid_score_low | 1.586049 |
| TCGA-55-7574-01A | 1.430452189 | Tumor | fatty_acid_score_low | 1.586049 |
| TCGA-MN-A4N1-01A | 1.432861674 | Tumor | fatty_acid_score_low | 1.586049 |
| TCGA-50-8459-01A | 1.432903672 | Tumor | fatty_acid_score_low | 1.586049 |
| TCGA-49-4487-01A | 1.433932932 | Tumor | fatty_acid_score_low | 1.586049 |
| TCGA-93-A4JO-01A | 1.434420635 | Tumor | fatty_acid_score_low | 1.586049 |
| TCGA-86-8279-01A | 1.437511467 | Tumor | fatty_acid_score_low | 1.586049 |
| TCGA-91-6829-01A | 1.438290001 | Tumor | fatty_acid_score_low | 1.586049 |
| TCGA-86-8278-01A | 1.441335522 | Tumor | fatty_acid_score_low | 1.586049 |
| TCGA-97-A4M0-01A | 1.444394015 | Tumor | fatty_acid_score_low | 1.586049 |
| TCGA-75-6207-01A | 1.444603704 | Tumor | fatty_acid_score_low | 1.586049 |
| TCGA-44-3396-01A | 1.447279647 | Tumor | fatty_acid_score_low | 1.586049 |
| TCGA-44-A479-01A | 1.447733066 | Tumor | fatty_acid_score_low | 1.586049 |
| TCGA-55-6975-01A | 1.449175667 | Tumor | fatty_acid_score_low | 1.586049 |
| TCGA-MP-A4TJ-01A | 1.450831 | Tumor | fatty_acid_score_low | 1.586049 |
| TCGA-35-4123-01A | 1.451334993 | Tumor | fatty_acid_score_low | 1.586049 |
| TCGA-86-8075-01A | 1.453081748 | Tumor | fatty_acid_score_low | 1.586049 |
| TCGA-38-6178-01A | 1.453102859 | Tumor | fatty_acid_score_low | 1.586049 |
| TCGA-55-6971-01A | 1.454208114 | Tumor | fatty_acid_score_low | 1.586049 |
| TCGA-MP-A4TK-01A | 1.45426559 | Tumor | fatty_acid_score_low | 1.586049 |
| TCGA-86-8359-01A | 1.457339236 | Tumor | fatty_acid_score_low | 1.586049 |
| TCGA-55-8299-01A | 1.457736526 | Tumor | fatty_acid_score_low | 1.586049 |
| TCGA-49-AARO-01A | 1.458077934 | Tumor | fatty_acid_score_low | 1.586049 |
| TCGA-55-8620-01A | 1.459236387 | Tumor | fatty_acid_score_low | 1.586049 |
| TCGA-75-6205-01A | 1.460133745 | Tumor | fatty_acid_score_low | 1.586049 |
| TCGA-99-8032-01A | 1.461002977 | Tumor | fatty_acid_score_low | 1.586049 |
| TCGA-53-7813-01A | 1.462771312 | Tumor | fatty_acid_score_low | 1.586049 |
| TCGA-55-8301-01A | 1.463350692 | Tumor | fatty_acid_score_low | 1.586049 |
| TCGA-86-8074-01A | 1.463452537 | Tumor | fatty_acid_score_low | 1.586049 |
| TCGA-73-A9RS-01A | 1.463757764 | Tumor | fatty_acid_score_low | 1.586049 |
| TCGA-44-7669-01A | 1.465760215 | Tumor | fatty_acid_score_low | 1.586049 |
| TCGA-05-4426-01A | 1.467103642 | Tumor | fatty_acid_score_low | 1.586049 |
| TCGA-MP-A4T6-01A | 1.468187603 | Tumor | fatty_acid_score_low | 1.586049 |
| TCGA-MP-A4T8-01A | 1.468410812 | Tumor | fatty_acid_score_low | 1.586049 |
| TCGA-44-6779-01A | 1.46856756 | Tumor | fatty_acid_score_low | 1.586049 |
| TCGA-55-A494-01A | 1.468851358 | Tumor | fatty_acid_score_low | 1.586049 |
| TCGA-86-8055-01A | 1.469357031 | Tumor | fatty_acid_score_low | 1.586049 |
| TCGA-55-8090-01A | 1.469394334 | Tumor | fatty_acid_score_low | 1.586049 |
| TCGA-55-6985-01A | 1.470283646 | Tumor | fatty_acid_score_low | 1.586049 |
| TCGA-55-6979-01A | 1.473616811 | Tumor | fatty_acid_score_low | 1.586049 |
| TCGA-44-6778-01A | 1.473716874 | Tumor | fatty_acid_score_low | 1.586049 |
| TCGA-55-7911-01A | 1.473906025 | Tumor | fatty_acid_score_low | 1.586049 |
| TCGA-44-6775-01A | 1.474239926 | Tumor | fatty_acid_score_low | 1.586049 |
| TCGA-05-4405-01A | 1.475885519 | Tumor | fatty_acid_score_low | 1.586049 |
| TCGA-55-6712-01A | 1.475945036 | Tumor | fatty_acid_score_low | 1.586049 |
| TCGA-44-3917-01A | 1.476453678 | Tumor | fatty_acid_score_low | 1.586049 |
| TCGA-55-8089-01A | 1.476724981 | Tumor | fatty_acid_score_low | 1.586049 |
| TCGA-44-A4SU-01A | 1.476885141 | Tumor | fatty_acid_score_low | 1.586049 |
| TCGA-55-7816-01A | 1.477472807 | Tumor | fatty_acid_score_low | 1.586049 |
| TCGA-50-5941-01A | 1.478405713 | Tumor | fatty_acid_score_low | 1.586049 |
| TCGA-05-4250-01A | 1.480442711 | Tumor | fatty_acid_score_low | 1.586049 |
| TCGA-71-6725-01A | 1.481864082 | Tumor | fatty_acid_score_low | 1.586049 |
| TCGA-95-7948-01A | 1.483854251 | Tumor | fatty_acid_score_low | 1.586049 |
| TCGA-44-8117-01A | 1.484026793 | Tumor | fatty_acid_score_low | 1.586049 |
| TCGA-44-6147-01A | 1.485400067 | Tumor | fatty_acid_score_low | 1.586049 |
| TCGA-69-7765-01A | 1.48547059 | Tumor | fatty_acid_score_low | 1.586049 |
| TCGA-NJ-A4YP-01A | 1.485675334 | Tumor | fatty_acid_score_low | 1.586049 |
| TCGA-44-7672-01A | 1.486969522 | Tumor | fatty_acid_score_low | 1.586049 |
| TCGA-97-A4LX-01A | 1.487057301 | Tumor | fatty_acid_score_low | 1.586049 |
| TCGA-44-2662-01A | 1.489011319 | Tumor | fatty_acid_score_low | 1.586049 |
| TCGA-69-7974-01A | 1.492120346 | Tumor | fatty_acid_score_low | 1.586049 |
| TCGA-62-8399-01A | 1.492560049 | Tumor | fatty_acid_score_low | 1.586049 |
| TCGA-86-8585-01A | 1.492940174 | Tumor | fatty_acid_score_low | 1.586049 |
| TCGA-97-7937-01A | 1.493810958 | Tumor | fatty_acid_score_low | 1.586049 |
| TCGA-50-5055-01A | 1.49460959 | Tumor | fatty_acid_score_low | 1.586049 |
| TCGA-55-1594-01A | 1.495367662 | Tumor | fatty_acid_score_low | 1.586049 |
| TCGA-05-4249-01A | 1.495911227 | Tumor | fatty_acid_score_low | 1.586049 |
| TCGA-86-7713-01A | 1.496071131 | Tumor | fatty_acid_score_low | 1.586049 |
| TCGA-86-A4JF-01A | 1.496492987 | Tumor | fatty_acid_score_low | 1.586049 |
| TCGA-50-5049-01A | 1.496956704 | Tumor | fatty_acid_score_low | 1.586049 |
| TCGA-49-AARE-01A | 1.497369312 | Tumor | fatty_acid_score_low | 1.586049 |
| TCGA-44-2659-01A | 1.499044553 | Tumor | fatty_acid_score_low | 1.586049 |
| TCGA-78-8655-01A | 1.500856615 | Tumor | fatty_acid_score_low | 1.586049 |
| TCGA-NJ-A55A-01A | 1.502118028 | Tumor | fatty_acid_score_low | 1.586049 |
| TCGA-69-7760-01A | 1.502725716 | Tumor | fatty_acid_score_low | 1.586049 |
| TCGA-86-8671-01A | 1.504194666 | Tumor | fatty_acid_score_low | 1.586049 |
| TCGA-55-8508-01A | 1.504785598 | Tumor | fatty_acid_score_low | 1.586049 |
| TCGA-55-6987-01A | 1.505376293 | Tumor | fatty_acid_score_low | 1.586049 |
| TCGA-55-A48X-01A | 1.506147891 | Tumor | fatty_acid_score_low | 1.586049 |
| TCGA-73-4668-01A | 1.507106704 | Tumor | fatty_acid_score_low | 1.586049 |
| TCGA-55-7576-01A | 1.508314801 | Tumor | fatty_acid_score_low | 1.586049 |
| TCGA-44-3919-01A | 1.509724721 | Tumor | fatty_acid_score_low | 1.586049 |
| TCGA-64-5781-01A | 1.51020475 | Tumor | fatty_acid_score_low | 1.586049 |
| TCGA-95-7947-01A | 1.510277021 | Tumor | fatty_acid_score_low | 1.586049 |
| TCGA-78-7143-01A | 1.511176847 | Tumor | fatty_acid_score_low | 1.586049 |
| TCGA-97-8175-01A | 1.512115383 | Tumor | fatty_acid_score_low | 1.586049 |
| TCGA-49-4490-01A | 1.512445882 | Tumor | fatty_acid_score_low | 1.586049 |
| TCGA-44-5645-01A | 1.513176264 | Tumor | fatty_acid_score_low | 1.586049 |
| TCGA-L9-A444-01A | 1.513652904 | Tumor | fatty_acid_score_low | 1.586049 |
| TCGA-86-7953-01A | 1.514077619 | Tumor | fatty_acid_score_low | 1.586049 |
| TCGA-75-5122-01A | 1.514385946 | Tumor | fatty_acid_score_low | 1.586049 |
| TCGA-NJ-A4YQ-01A | 1.514683787 | Tumor | fatty_acid_score_low | 1.586049 |
| TCGA-55-7227-01A | 1.515984928 | Tumor | fatty_acid_score_low | 1.586049 |
| TCGA-86-A4P7-01A | 1.516207634 | Tumor | fatty_acid_score_low | 1.586049 |
| TCGA-95-A4VP-01A | 1.5171374 | Tumor | fatty_acid_score_low | 1.586049 |
| TCGA-05-4382-01A | 1.51718554 | Tumor | fatty_acid_score_low | 1.586049 |
| TCGA-62-A46O-01A | 1.517719805 | Tumor | fatty_acid_score_low | 1.586049 |
| TCGA-53-7626-01A | 1.517806115 | Tumor | fatty_acid_score_low | 1.586049 |
| TCGA-05-4415-01A | 1.518115513 | Tumor | fatty_acid_score_low | 1.586049 |
| TCGA-97-7546-01A | 1.518289699 | Tumor | fatty_acid_score_low | 1.586049 |
| TCGA-MP-A4TH-01A | 1.518707256 | Tumor | fatty_acid_score_low | 1.586049 |
| TCGA-64-5815-01A | 1.518789054 | Tumor | fatty_acid_score_low | 1.586049 |
| TCGA-44-7670-01A | 1.520942871 | Tumor | fatty_acid_score_low | 1.586049 |
| TCGA-MP-A4SY-01A | 1.521433626 | Tumor | fatty_acid_score_low | 1.586049 |
| TCGA-38-7271-01A | 1.521548729 | Tumor | fatty_acid_score_low | 1.586049 |
| TCGA-L9-A7SV-01A | 1.522250998 | Tumor | fatty_acid_score_low | 1.586049 |
| TCGA-86-7955-01A | 1.52260331 | Tumor | fatty_acid_score_low | 1.586049 |
| TCGA-55-8096-01A | 1.52345981 | Tumor | fatty_acid_score_low | 1.586049 |
| TCGA-50-8457-01A | 1.523842809 | Tumor | fatty_acid_score_low | 1.586049 |
| TCGA-86-8672-01A | 1.524729401 | Tumor | fatty_acid_score_low | 1.586049 |
| TCGA-55-8203-01A | 1.524890156 | Tumor | fatty_acid_score_low | 1.586049 |
| TCGA-J2-A4AG-01A | 1.525653727 | Tumor | fatty_acid_score_low | 1.586049 |
| TCGA-05-4417-01A | 1.525881587 | Tumor | fatty_acid_score_low | 1.586049 |
| TCGA-55-7903-01A | 1.52628445 | Tumor | fatty_acid_score_low | 1.586049 |
| TCGA-69-7979-01A | 1.527626088 | Tumor | fatty_acid_score_low | 1.586049 |
| TCGA-73-4666-01A | 1.528164586 | Tumor | fatty_acid_score_low | 1.586049 |
| TCGA-J2-8192-01A | 1.530016668 | Tumor | fatty_acid_score_low | 1.586049 |
| TCGA-NJ-A4YF-01A | 1.531181242 | Tumor | fatty_acid_score_low | 1.586049 |
| TCGA-99-8025-01A | 1.531572417 | Tumor | fatty_acid_score_low | 1.586049 |
| TCGA-97-8547-01A | 1.532039985 | Tumor | fatty_acid_score_low | 1.586049 |
| TCGA-49-6745-01A | 1.533531588 | Tumor | fatty_acid_score_low | 1.586049 |
| TCGA-38-4629-01A | 1.534268851 | Tumor | fatty_acid_score_low | 1.586049 |
| TCGA-71-8520-01A | 1.53485471 | Tumor | fatty_acid_score_low | 1.586049 |
| TCGA-64-1677-01A | 1.535658655 | Tumor | fatty_acid_score_low | 1.586049 |
| TCGA-55-7570-01A | 1.535664178 | Tumor | fatty_acid_score_low | 1.586049 |
| TCGA-97-8172-01A | 1.535899798 | Tumor | fatty_acid_score_low | 1.586049 |
| TCGA-55-A57B-01A | 1.536133004 | Tumor | fatty_acid_score_low | 1.586049 |
| TCGA-86-8668-01A | 1.537877424 | Tumor | fatty_acid_score_low | 1.586049 |
| TCGA-78-8648-01A | 1.538291285 | Tumor | fatty_acid_score_low | 1.586049 |
| TCGA-49-4512-01A | 1.5390901 | Tumor | fatty_acid_score_low | 1.586049 |
| TCGA-44-4112-01A | 1.539346574 | Tumor | fatty_acid_score_low | 1.586049 |
| TCGA-44-5643-01A | 1.53965931 | Tumor | fatty_acid_score_low | 1.586049 |
| TCGA-55-8302-01A | 1.539703936 | Tumor | fatty_acid_score_low | 1.586049 |
| TCGA-75-7025-01A | 1.539944031 | Tumor | fatty_acid_score_low | 1.586049 |
| TCGA-44-7660-01A | 1.54029462 | Tumor | fatty_acid_score_low | 1.586049 |
| TCGA-55-7281-01A | 1.540420253 | Tumor | fatty_acid_score_low | 1.586049 |
| TCGA-S2-AA1A-01A | 1.541049666 | Tumor | fatty_acid_score_low | 1.586049 |
| TCGA-55-8097-01A | 1.542242327 | Tumor | fatty_acid_score_low | 1.586049 |
| TCGA-49-AARN-01A | 1.546096473 | Tumor | fatty_acid_score_low | 1.586049 |
| TCGA-55-6986-01A | 1.546218034 | Tumor | fatty_acid_score_low | 1.586049 |
| TCGA-99-8033-01A | 1.546265995 | Tumor | fatty_acid_score_low | 1.586049 |
| TCGA-55-6984-01A | 1.546630329 | Tumor | fatty_acid_score_low | 1.586049 |
| TCGA-44-8120-01A | 1.552331393 | Tumor | fatty_acid_score_low | 1.586049 |
| TCGA-49-AARQ-01A | 1.552978479 | Tumor | fatty_acid_score_low | 1.586049 |
| TCGA-44-A47A-01A | 1.553086094 | Tumor | fatty_acid_score_low | 1.586049 |
| TCGA-69-7973-01A | 1.555443329 | Tumor | fatty_acid_score_low | 1.586049 |
| TCGA-50-6594-01A | 1.556424514 | Tumor | fatty_acid_score_low | 1.586049 |
| TCGA-44-6777-01A | 1.556653495 | Tumor | fatty_acid_score_low | 1.586049 |
| TCGA-55-8615-01A | 1.557581142 | Tumor | fatty_acid_score_low | 1.586049 |
| TCGA-05-4420-01A | 1.557684378 | Tumor | fatty_acid_score_low | 1.586049 |
| TCGA-MP-A4T9-01A | 1.557846694 | Tumor | fatty_acid_score_low | 1.586049 |
| TCGA-99-8028-01A | 1.557901521 | Tumor | fatty_acid_score_low | 1.586049 |
| TCGA-55-A4DF-01A | 1.558085735 | Tumor | fatty_acid_score_low | 1.586049 |
| TCGA-69-7761-01A | 1.558172556 | Tumor | fatty_acid_score_low | 1.586049 |
| TCGA-55-6980-01A | 1.558288683 | Tumor | fatty_acid_score_low | 1.586049 |
| TCGA-62-A472-01A | 1.558973008 | Tumor | fatty_acid_score_low | 1.586049 |
| TCGA-86-7711-01A | 1.560174572 | Tumor | fatty_acid_score_low | 1.586049 |
| TCGA-55-8507-01A | 1.560881432 | Tumor | fatty_acid_score_low | 1.586049 |
| TCGA-69-7764-01A | 1.561259664 | Tumor | fatty_acid_score_low | 1.586049 |
| TCGA-L9-A743-01A | 1.561972025 | Tumor | fatty_acid_score_low | 1.586049 |
| TCGA-49-6743-01A | 1.562165187 | Tumor | fatty_acid_score_low | 1.586049 |
| TCGA-44-6147-11A | 1.562542338 | Normal | fatty_acid_score_low | 1.586049 |
| TCGA-05-4390-01A | 1.562887775 | Tumor | fatty_acid_score_low | 1.586049 |
| TCGA-97-A4M7-01A | 1.563648436 | Tumor | fatty_acid_score_low | 1.586049 |
| TCGA-L9-A5IP-01A | 1.564324251 | Tumor | fatty_acid_score_low | 1.586049 |
| TCGA-73-4662-01A | 1.564495591 | Tumor | fatty_acid_score_low | 1.586049 |
| TCGA-73-4658-01A | 1.564750344 | Tumor | fatty_acid_score_low | 1.586049 |
| TCGA-49-4507-01A | 1.565037693 | Tumor | fatty_acid_score_low | 1.586049 |
| TCGA-55-7573-01A | 1.565390141 | Tumor | fatty_acid_score_low | 1.586049 |
| TCGA-05-4430-01A | 1.565430293 | Tumor | fatty_acid_score_low | 1.586049 |
| TCGA-NJ-A4YI-01A | 1.56554993 | Tumor | fatty_acid_score_low | 1.586049 |
| TCGA-49-AAQV-01A | 1.567145093 | Tumor | fatty_acid_score_low | 1.586049 |
| TCGA-78-7161-01A | 1.568693757 | Tumor | fatty_acid_score_low | 1.586049 |
| TCGA-55-A491-01A | 1.570732203 | Tumor | fatty_acid_score_low | 1.586049 |
| TCGA-62-A46R-01A | 1.571185424 | Tumor | fatty_acid_score_low | 1.586049 |
| TCGA-L9-A443-01A | 1.571209949 | Tumor | fatty_acid_score_low | 1.586049 |
| TCGA-05-4432-01A | 1.5729015 | Tumor | fatty_acid_score_low | 1.586049 |
| TCGA-38-4625-01A | 1.5731987 | Tumor | fatty_acid_score_low | 1.586049 |
| TCGA-4B-A93V-01A | 1.57389118 | Tumor | fatty_acid_score_low | 1.586049 |
| TCGA-NJ-A7XG-01A | 1.57417631 | Tumor | fatty_acid_score_low | 1.586049 |
| TCGA-75-7027-01A | 1.574194572 | Tumor | fatty_acid_score_low | 1.586049 |
| TCGA-55-6981-01A | 1.574735265 | Tumor | fatty_acid_score_low | 1.586049 |
| TCGA-55-8208-01A | 1.577039837 | Tumor | fatty_acid_score_low | 1.586049 |
| TCGA-55-7727-01A | 1.577639619 | Tumor | fatty_acid_score_low | 1.586049 |
| TCGA-86-6562-01A | 1.578653948 | Tumor | fatty_acid_score_low | 1.586049 |
| TCGA-55-6543-01A | 1.578873494 | Tumor | fatty_acid_score_low | 1.586049 |
| TCGA-49-4505-01A | 1.578898272 | Tumor | fatty_acid_score_low | 1.586049 |
| TCGA-55-8091-01A | 1.579322684 | Tumor | fatty_acid_score_low | 1.586049 |
| TCGA-05-4395-01A | 1.57939942 | Tumor | fatty_acid_score_low | 1.586049 |
| TCGA-55-8087-01A | 1.579951519 | Tumor | fatty_acid_score_low | 1.586049 |
| TCGA-99-7458-01A | 1.580832136 | Tumor | fatty_acid_score_low | 1.586049 |
| TCGA-91-6828-01A | 1.582157346 | Tumor | fatty_acid_score_low | 1.586049 |
| TCGA-67-6216-01A | 1.582427573 | Tumor | fatty_acid_score_low | 1.586049 |
| TCGA-55-8092-01A | 1.583046676 | Tumor | fatty_acid_score_low | 1.586049 |
| TCGA-69-7763-01A | 1.584286286 | Tumor | fatty_acid_score_low | 1.586049 |
| TCGA-44-2656-01A | 1.585902713 | Tumor | fatty_acid_score_low | 1.586049 |
| TCGA-64-5778-01A | 1.586194906 | Tumor | fatty_acid_score_high | 1.586049 |
| TCGA-78-8662-01A | 1.586300693 | Tumor | fatty_acid_score_high | 1.586049 |
| TCGA-55-7815-01A | 1.58649742 | Tumor | fatty_acid_score_high | 1.586049 |
| TCGA-78-8660-01A | 1.587214536 | Tumor | fatty_acid_score_high | 1.586049 |
| TCGA-75-7030-01A | 1.587388319 | Tumor | fatty_acid_score_high | 1.586049 |
| TCGA-78-7537-01A | 1.587402279 | Tumor | fatty_acid_score_high | 1.586049 |
| TCGA-44-6145-01A | 1.587479883 | Tumor | fatty_acid_score_high | 1.586049 |
| TCGA-93-7348-01A | 1.588142132 | Tumor | fatty_acid_score_high | 1.586049 |
| TCGA-38-4627-01A | 1.588937089 | Tumor | fatty_acid_score_high | 1.586049 |
| TCGA-55-7907-01A | 1.591577539 | Tumor | fatty_acid_score_high | 1.586049 |
| TCGA-55-8207-01A | 1.592999186 | Tumor | fatty_acid_score_high | 1.586049 |
| TCGA-J2-A4AD-01A | 1.593688182 | Tumor | fatty_acid_score_high | 1.586049 |
| TCGA-49-AARR-01A | 1.593733613 | Tumor | fatty_acid_score_high | 1.586049 |
| TCGA-78-7145-01A | 1.595562139 | Tumor | fatty_acid_score_high | 1.586049 |
| TCGA-91-6835-11A | 1.595666339 | Normal | fatty_acid_score_high | 1.586049 |
| TCGA-55-6983-01A | 1.596039341 | Tumor | fatty_acid_score_high | 1.586049 |
| TCGA-50-5930-01A | 1.596248467 | Tumor | fatty_acid_score_high | 1.586049 |
| TCGA-55-8514-01A | 1.596874249 | Tumor | fatty_acid_score_high | 1.586049 |
| TCGA-95-8039-01A | 1.597906142 | Tumor | fatty_acid_score_high | 1.586049 |
| TCGA-50-5936-01A | 1.598090631 | Tumor | fatty_acid_score_high | 1.586049 |
| TCGA-97-A4M6-01A | 1.598771433 | Tumor | fatty_acid_score_high | 1.586049 |
| TCGA-78-7147-01A | 1.600134938 | Tumor | fatty_acid_score_high | 1.586049 |
| TCGA-55-6982-01A | 1.600817626 | Tumor | fatty_acid_score_high | 1.586049 |
| TCGA-44-2657-01A | 1.601388207 | Tumor | fatty_acid_score_high | 1.586049 |
| TCGA-67-6217-01A | 1.602734017 | Tumor | fatty_acid_score_high | 1.586049 |
| TCGA-95-7039-01A | 1.603527318 | Tumor | fatty_acid_score_high | 1.586049 |
| TCGA-05-4402-01A | 1.603938307 | Tumor | fatty_acid_score_high | 1.586049 |
| TCGA-75-6211-01A | 1.604092731 | Tumor | fatty_acid_score_high | 1.586049 |
| TCGA-44-6148-01A | 1.60509154 | Tumor | fatty_acid_score_high | 1.586049 |
| TCGA-05-4244-01A | 1.605744856 | Tumor | fatty_acid_score_high | 1.586049 |
| TCGA-99-AA5R-01A | 1.605934754 | Tumor | fatty_acid_score_high | 1.586049 |
| TCGA-50-5939-01A | 1.606654444 | Tumor | fatty_acid_score_high | 1.586049 |
| TCGA-73-4670-01A | 1.609131724 | Tumor | fatty_acid_score_high | 1.586049 |
| TCGA-86-A4D0-01A | 1.609724473 | Tumor | fatty_acid_score_high | 1.586049 |
| TCGA-75-5126-01A | 1.610398744 | Tumor | fatty_acid_score_high | 1.586049 |
| TCGA-86-8056-01A | 1.615338619 | Tumor | fatty_acid_score_high | 1.586049 |
| TCGA-49-4506-01A | 1.616463305 | Tumor | fatty_acid_score_high | 1.586049 |
| TCGA-62-A46P-01A | 1.617596999 | Tumor | fatty_acid_score_high | 1.586049 |
| TCGA-44-A47G-01A | 1.618979418 | Tumor | fatty_acid_score_high | 1.586049 |
| TCGA-86-8280-01A | 1.621661968 | Tumor | fatty_acid_score_high | 1.586049 |
| TCGA-78-7152-01A | 1.621783266 | Tumor | fatty_acid_score_high | 1.586049 |
| TCGA-50-5045-01A | 1.621883065 | Tumor | fatty_acid_score_high | 1.586049 |
| TCGA-49-6744-01A | 1.622029622 | Tumor | fatty_acid_score_high | 1.586049 |
| TCGA-MP-A4TA-01A | 1.622650836 | Tumor | fatty_acid_score_high | 1.586049 |
| TCGA-62-8394-01A | 1.622786945 | Tumor | fatty_acid_score_high | 1.586049 |
| TCGA-55-6986-11A | 1.623667395 | Normal | fatty_acid_score_high | 1.586049 |
| TCGA-86-8076-01A | 1.623814538 | Tumor | fatty_acid_score_high | 1.586049 |
| TCGA-80-5611-01A | 1.625618239 | Tumor | fatty_acid_score_high | 1.586049 |
| TCGA-55-6982-11A | 1.626020372 | Normal | fatty_acid_score_high | 1.586049 |
| TCGA-78-8640-01A | 1.626112143 | Tumor | fatty_acid_score_high | 1.586049 |
| TCGA-64-1678-01A | 1.628202425 | Tumor | fatty_acid_score_high | 1.586049 |
| TCGA-80-5607-01A | 1.628688513 | Tumor | fatty_acid_score_high | 1.586049 |
| TCGA-44-6778-11A | 1.62897447 | Normal | fatty_acid_score_high | 1.586049 |
| TCGA-50-6592-01A | 1.629361081 | Tumor | fatty_acid_score_high | 1.586049 |
| TCGA-78-7539-01A | 1.630012746 | Tumor | fatty_acid_score_high | 1.586049 |
| TCGA-55-8616-01A | 1.631101078 | Tumor | fatty_acid_score_high | 1.586049 |
| TCGA-44-7659-01A | 1.633718239 | Tumor | fatty_acid_score_high | 1.586049 |
| TCGA-55-8085-01A | 1.634647626 | Tumor | fatty_acid_score_high | 1.586049 |
| TCGA-05-4410-01A | 1.636481551 | Tumor | fatty_acid_score_high | 1.586049 |
| TCGA-55-8510-01A | 1.637388015 | Tumor | fatty_acid_score_high | 1.586049 |
| TCGA-67-6215-01A | 1.638845702 | Tumor | fatty_acid_score_high | 1.586049 |
| TCGA-97-7552-01A | 1.639238007 | Tumor | fatty_acid_score_high | 1.586049 |
| TCGA-55-6972-11A | 1.639691121 | Normal | fatty_acid_score_high | 1.586049 |
| TCGA-55-5899-01A | 1.640111785 | Tumor | fatty_acid_score_high | 1.586049 |
| TCGA-78-7158-01A | 1.640331188 | Tumor | fatty_acid_score_high | 1.586049 |
| TCGA-55-7728-01A | 1.642338946 | Tumor | fatty_acid_score_high | 1.586049 |
| TCGA-55-7914-01A | 1.642410537 | Tumor | fatty_acid_score_high | 1.586049 |
| TCGA-55-8206-01A | 1.643579156 | Tumor | fatty_acid_score_high | 1.586049 |
| TCGA-38-A44F-01A | 1.645017074 | Tumor | fatty_acid_score_high | 1.586049 |
| TCGA-64-1676-01A | 1.646799636 | Tumor | fatty_acid_score_high | 1.586049 |
| TCGA-50-5051-01A | 1.647606904 | Tumor | fatty_acid_score_high | 1.586049 |
| TCGA-50-5932-01A | 1.647729408 | Tumor | fatty_acid_score_high | 1.586049 |
| TCGA-73-7499-01A | 1.650998668 | Tumor | fatty_acid_score_high | 1.586049 |
| TCGA-50-6673-01A | 1.653037626 | Tumor | fatty_acid_score_high | 1.586049 |
| TCGA-86-A4P8-01A | 1.653650061 | Tumor | fatty_acid_score_high | 1.586049 |
| TCGA-55-6981-11A | 1.655023121 | Normal | fatty_acid_score_high | 1.586049 |
| TCGA-50-6595-11A | 1.655655434 | Normal | fatty_acid_score_high | 1.586049 |
| TCGA-44-6146-11A | 1.65613535 | Normal | fatty_acid_score_high | 1.586049 |
| TCGA-97-7547-01A | 1.658095691 | Tumor | fatty_acid_score_high | 1.586049 |
| TCGA-62-A46Y-01A | 1.658495743 | Tumor | fatty_acid_score_high | 1.586049 |
| TCGA-38-4632-01A | 1.659279088 | Tumor | fatty_acid_score_high | 1.586049 |
| TCGA-35-4122-01A | 1.660934974 | Tumor | fatty_acid_score_high | 1.586049 |
| TCGA-50-5935-01A | 1.662607314 | Tumor | fatty_acid_score_high | 1.586049 |
| TCGA-38-4631-01A | 1.663518057 | Tumor | fatty_acid_score_high | 1.586049 |
| TCGA-55-7283-01A | 1.664515074 | Tumor | fatty_acid_score_high | 1.586049 |
| TCGA-50-5944-01A | 1.665054651 | Tumor | fatty_acid_score_high | 1.586049 |
| TCGA-55-7995-01A | 1.665976044 | Tumor | fatty_acid_score_high | 1.586049 |
| TCGA-67-3771-01A | 1.666685135 | Tumor | fatty_acid_score_high | 1.586049 |
| TCGA-NJ-A4YG-01A | 1.66769574 | Tumor | fatty_acid_score_high | 1.586049 |
| TCGA-95-7043-01A | 1.66896732 | Tumor | fatty_acid_score_high | 1.586049 |
| TCGA-44-2661-11A | 1.669122002 | Normal | fatty_acid_score_high | 1.586049 |
| TCGA-78-7540-01A | 1.669137094 | Tumor | fatty_acid_score_high | 1.586049 |
| TCGA-55-8094-01A | 1.669181968 | Tumor | fatty_acid_score_high | 1.586049 |
| TCGA-MP-A4SW-01A | 1.670902081 | Tumor | fatty_acid_score_high | 1.586049 |
| TCGA-49-4488-01A | 1.671072456 | Tumor | fatty_acid_score_high | 1.586049 |
| TCGA-55-A48Y-01A | 1.671086974 | Tumor | fatty_acid_score_high | 1.586049 |
| TCGA-97-A4M5-01A | 1.671145448 | Tumor | fatty_acid_score_high | 1.586049 |
| TCGA-91-6830-01A | 1.671533339 | Tumor | fatty_acid_score_high | 1.586049 |
| TCGA-L9-A50W-01A | 1.672526146 | Tumor | fatty_acid_score_high | 1.586049 |
| TCGA-44-2655-11A | 1.672586878 | Normal | fatty_acid_score_high | 1.586049 |
| TCGA-97-8174-01A | 1.674334668 | Tumor | fatty_acid_score_high | 1.586049 |
| TCGA-49-4494-01A | 1.675727617 | Tumor | fatty_acid_score_high | 1.586049 |
| TCGA-49-AAR0-01A | 1.676431502 | Tumor | fatty_acid_score_high | 1.586049 |
| TCGA-44-3396-11A | 1.676909544 | Normal | fatty_acid_score_high | 1.586049 |
| TCGA-44-6148-11A | 1.677318512 | Normal | fatty_acid_score_high | 1.586049 |
| TCGA-86-8669-01A | 1.678131301 | Tumor | fatty_acid_score_high | 1.586049 |
| TCGA-95-A4VK-01A | 1.678820287 | Tumor | fatty_acid_score_high | 1.586049 |
| TCGA-05-5715-01A | 1.679375299 | Tumor | fatty_acid_score_high | 1.586049 |
| TCGA-78-7163-01A | 1.679405859 | Tumor | fatty_acid_score_high | 1.586049 |
| TCGA-55-8619-01A | 1.681146592 | Tumor | fatty_acid_score_high | 1.586049 |
| TCGA-55-6978-11A | 1.683800374 | Normal | fatty_acid_score_high | 1.586049 |
| TCGA-97-7553-01A | 1.685628244 | Tumor | fatty_acid_score_high | 1.586049 |
| TCGA-55-8513-01A | 1.685779971 | Tumor | fatty_acid_score_high | 1.586049 |
| TCGA-93-A4JP-01A | 1.686044339 | Tumor | fatty_acid_score_high | 1.586049 |
| TCGA-05-4434-01A | 1.686104103 | Tumor | fatty_acid_score_high | 1.586049 |
| TCGA-80-5608-01A | 1.686769451 | Tumor | fatty_acid_score_high | 1.586049 |
| TCGA-55-6980-11A | 1.687910071 | Normal | fatty_acid_score_high | 1.586049 |
| TCGA-50-5939-11A | 1.68844861 | Normal | fatty_acid_score_high | 1.586049 |
| TCGA-05-4433-01A | 1.688736096 | Tumor | fatty_acid_score_high | 1.586049 |
| TCGA-91-8497-01A | 1.689429977 | Tumor | fatty_acid_score_high | 1.586049 |
| TCGA-38-4628-01A | 1.689730918 | Tumor | fatty_acid_score_high | 1.586049 |
| TCGA-86-8073-01A | 1.691503389 | Tumor | fatty_acid_score_high | 1.586049 |
| TCGA-91-6831-11A | 1.691593193 | Normal | fatty_acid_score_high | 1.586049 |
| TCGA-62-A471-01A | 1.693541656 | Tumor | fatty_acid_score_high | 1.586049 |
| TCGA-91-7771-01A | 1.694172531 | Tumor | fatty_acid_score_high | 1.586049 |
| TCGA-53-A4EZ-01A | 1.694647934 | Tumor | fatty_acid_score_high | 1.586049 |
| TCGA-91-6849-11A | 1.694871606 | Normal | fatty_acid_score_high | 1.586049 |
| TCGA-50-5932-11A | 1.694989727 | Normal | fatty_acid_score_high | 1.586049 |
| TCGA-55-6970-11A | 1.696205554 | Normal | fatty_acid_score_high | 1.586049 |
| TCGA-97-7938-01A | 1.696283922 | Tumor | fatty_acid_score_high | 1.586049 |
| TCGA-50-8460-01A | 1.698142157 | Tumor | fatty_acid_score_high | 1.586049 |
| TCGA-97-A4M3-01A | 1.698746513 | Tumor | fatty_acid_score_high | 1.586049 |
| TCGA-49-AAR2-01A | 1.699823672 | Tumor | fatty_acid_score_high | 1.586049 |
| TCGA-55-1596-01A | 1.700439337 | Tumor | fatty_acid_score_high | 1.586049 |
| TCGA-50-5066-01A | 1.701910293 | Tumor | fatty_acid_score_high | 1.586049 |
| TCGA-93-7347-01A | 1.702718868 | Tumor | fatty_acid_score_high | 1.586049 |
| TCGA-05-4425-01A | 1.703134989 | Tumor | fatty_acid_score_high | 1.586049 |
| TCGA-55-7724-01A | 1.703408535 | Tumor | fatty_acid_score_high | 1.586049 |
| TCGA-86-7714-01A | 1.704364758 | Tumor | fatty_acid_score_high | 1.586049 |
| TCGA-50-5942-01A | 1.705146104 | Tumor | fatty_acid_score_high | 1.586049 |
| TCGA-44-6146-01A | 1.706299921 | Tumor | fatty_acid_score_high | 1.586049 |
| TCGA-55-7725-01A | 1.706396802 | Tumor | fatty_acid_score_high | 1.586049 |
| TCGA-L4-A4E6-01A | 1.707549927 | Tumor | fatty_acid_score_high | 1.586049 |
| TCGA-64-1681-01A | 1.707617334 | Tumor | fatty_acid_score_high | 1.586049 |
| TCGA-55-6969-11A | 1.708576714 | Normal | fatty_acid_score_high | 1.586049 |
| TCGA-97-8176-01A | 1.709392469 | Tumor | fatty_acid_score_high | 1.586049 |
| TCGA-44-2665-11A | 1.709434365 | Normal | fatty_acid_score_high | 1.586049 |
| TCGA-MP-A4TE-01A | 1.710258752 | Tumor | fatty_acid_score_high | 1.586049 |
| TCGA-50-6593-01A | 1.710672458 | Tumor | fatty_acid_score_high | 1.586049 |
| TCGA-35-5375-01A | 1.710913208 | Tumor | fatty_acid_score_high | 1.586049 |
| TCGA-78-7167-01A | 1.711890351 | Tumor | fatty_acid_score_high | 1.586049 |
| TCGA-97-8552-01A | 1.712936246 | Tumor | fatty_acid_score_high | 1.586049 |
| TCGA-NJ-A55O-01A | 1.714578912 | Tumor | fatty_acid_score_high | 1.586049 |
| TCGA-55-8621-01A | 1.714983812 | Tumor | fatty_acid_score_high | 1.586049 |
| TCGA-73-4675-01A | 1.715708061 | Tumor | fatty_acid_score_high | 1.586049 |
| TCGA-50-5072-01A | 1.717380352 | Tumor | fatty_acid_score_high | 1.586049 |
| TCGA-93-8067-01A | 1.71751448 | Tumor | fatty_acid_score_high | 1.586049 |
| TCGA-44-5645-11A | 1.718000432 | Normal | fatty_acid_score_high | 1.586049 |
| TCGA-44-2661-01A | 1.718331922 | Tumor | fatty_acid_score_high | 1.586049 |
| TCGA-50-5935-11A | 1.719006245 | Normal | fatty_acid_score_high | 1.586049 |
| TCGA-50-6597-01A | 1.719328522 | Tumor | fatty_acid_score_high | 1.586049 |
| TCGA-05-4396-01A | 1.722005889 | Tumor | fatty_acid_score_high | 1.586049 |
| TCGA-MP-A4TD-01A | 1.723619986 | Tumor | fatty_acid_score_high | 1.586049 |
| TCGA-75-5147-01A | 1.724623567 | Tumor | fatty_acid_score_high | 1.586049 |
| TCGA-55-6970-01A | 1.724638114 | Tumor | fatty_acid_score_high | 1.586049 |
| TCGA-05-4424-01A | 1.725866128 | Tumor | fatty_acid_score_high | 1.586049 |
| TCGA-62-A46V-01A | 1.727368343 | Tumor | fatty_acid_score_high | 1.586049 |
| TCGA-49-4510-01A | 1.729043677 | Tumor | fatty_acid_score_high | 1.586049 |
| TCGA-49-4501-01A | 1.730469135 | Tumor | fatty_acid_score_high | 1.586049 |
| TCGA-78-7150-01A | 1.730744399 | Tumor | fatty_acid_score_high | 1.586049 |
| TCGA-J2-8194-01A | 1.731214625 | Tumor | fatty_acid_score_high | 1.586049 |
| TCGA-64-1680-01A | 1.734455975 | Tumor | fatty_acid_score_high | 1.586049 |
| TCGA-J2-A4AE-01A | 1.734525659 | Tumor | fatty_acid_score_high | 1.586049 |
| TCGA-78-7166-01A | 1.736811961 | Tumor | fatty_acid_score_high | 1.586049 |
| TCGA-44-A4SS-01A | 1.736920221 | Tumor | fatty_acid_score_high | 1.586049 |
| TCGA-75-5146-01A | 1.737715755 | Tumor | fatty_acid_score_high | 1.586049 |
| TCGA-69-8253-01A | 1.739307821 | Tumor | fatty_acid_score_high | 1.586049 |
| TCGA-05-4397-01A | 1.739379982 | Tumor | fatty_acid_score_high | 1.586049 |
| TCGA-50-5933-11A | 1.739974561 | Normal | fatty_acid_score_high | 1.586049 |
| TCGA-55-A4DG-01A | 1.740950272 | Tumor | fatty_acid_score_high | 1.586049 |
| TCGA-91-6828-11A | 1.741494348 | Normal | fatty_acid_score_high | 1.586049 |
| TCGA-05-4418-01A | 1.743371426 | Tumor | fatty_acid_score_high | 1.586049 |
| TCGA-67-3773-01A | 1.743418311 | Tumor | fatty_acid_score_high | 1.586049 |
| TCGA-69-8453-01A | 1.743707816 | Tumor | fatty_acid_score_high | 1.586049 |
| TCGA-05-5428-01A | 1.744125258 | Tumor | fatty_acid_score_high | 1.586049 |
| TCGA-75-6206-01A | 1.744196287 | Tumor | fatty_acid_score_high | 1.586049 |
| TCGA-50-5068-01A | 1.747545995 | Tumor | fatty_acid_score_high | 1.586049 |
| TCGA-73-7498-01A | 1.749522514 | Tumor | fatty_acid_score_high | 1.586049 |
| TCGA-55-6979-11A | 1.750220318 | Normal | fatty_acid_score_high | 1.586049 |
| TCGA-44-2657-11A | 1.750453408 | Normal | fatty_acid_score_high | 1.586049 |
| TCGA-O1-A52J-01A | 1.750459536 | Tumor | fatty_acid_score_high | 1.586049 |
| TCGA-67-3774-01A | 1.752069764 | Tumor | fatty_acid_score_high | 1.586049 |
| TCGA-44-6145-11A | 1.752575242 | Normal | fatty_acid_score_high | 1.586049 |
| TCGA-NJ-A55R-01A | 1.756256007 | Tumor | fatty_acid_score_high | 1.586049 |
| TCGA-62-A46S-01A | 1.757107889 | Tumor | fatty_acid_score_high | 1.586049 |
| TCGA-97-8171-01A | 1.758222841 | Tumor | fatty_acid_score_high | 1.586049 |
| TCGA-44-2662-11A | 1.758816848 | Normal | fatty_acid_score_high | 1.586049 |
| TCGA-50-5931-11A | 1.760158983 | Normal | fatty_acid_score_high | 1.586049 |
| TCGA-49-6744-11A | 1.762080374 | Normal | fatty_acid_score_high | 1.586049 |
| TCGA-62-8398-01A | 1.763428265 | Tumor | fatty_acid_score_high | 1.586049 |
| TCGA-78-7154-01A | 1.763779691 | Tumor | fatty_acid_score_high | 1.586049 |
| TCGA-73-4659-01A | 1.76472951 | Tumor | fatty_acid_score_high | 1.586049 |
| TCGA-91-6847-11A | 1.766645661 | Normal | fatty_acid_score_high | 1.586049 |
| TCGA-97-8177-01A | 1.76743772 | Tumor | fatty_acid_score_high | 1.586049 |
| TCGA-86-8674-01A | 1.770912598 | Tumor | fatty_acid_score_high | 1.586049 |
| TCGA-67-3772-01A | 1.772595951 | Tumor | fatty_acid_score_high | 1.586049 |
| TCGA-44-3398-01A | 1.772728089 | Tumor | fatty_acid_score_high | 1.586049 |
| TCGA-44-2666-01A | 1.773231723 | Tumor | fatty_acid_score_high | 1.586049 |
| TCGA-MP-A5C7-01A | 1.775343422 | Tumor | fatty_acid_score_high | 1.586049 |
| TCGA-55-6975-11A | 1.776474253 | Normal | fatty_acid_score_high | 1.586049 |
| TCGA-75-6203-01A | 1.776540884 | Tumor | fatty_acid_score_high | 1.586049 |
| TCGA-05-5425-01A | 1.777638241 | Tumor | fatty_acid_score_high | 1.586049 |
| TCGA-49-6743-11A | 1.778167482 | Normal | fatty_acid_score_high | 1.586049 |
| TCGA-97-A4M1-01A | 1.779254805 | Tumor | fatty_acid_score_high | 1.586049 |
| TCGA-78-7220-01A | 1.780273363 | Tumor | fatty_acid_score_high | 1.586049 |
| TCGA-55-1592-01A | 1.780416718 | Tumor | fatty_acid_score_high | 1.586049 |
| TCGA-55-8512-01A | 1.780911262 | Tumor | fatty_acid_score_high | 1.586049 |
| TCGA-73-4676-01A | 1.782503923 | Tumor | fatty_acid_score_high | 1.586049 |
| TCGA-44-6777-11A | 1.783433973 | Normal | fatty_acid_score_high | 1.586049 |
| TCGA-50-5936-11A | 1.786953971 | Normal | fatty_acid_score_high | 1.586049 |
| TCGA-86-A456-01A | 1.78695836 | Tumor | fatty_acid_score_high | 1.586049 |
| TCGA-62-8397-01A | 1.787718188 | Tumor | fatty_acid_score_high | 1.586049 |
| TCGA-75-7031-01A | 1.788105892 | Tumor | fatty_acid_score_high | 1.586049 |
| TCGA-38-4626-01A | 1.789571651 | Tumor | fatty_acid_score_high | 1.586049 |
| TCGA-78-7149-01A | 1.79100205 | Tumor | fatty_acid_score_high | 1.586049 |
| TCGA-97-7941-01A | 1.79435445 | Tumor | fatty_acid_score_high | 1.586049 |
| TCGA-55-8505-01A | 1.795015737 | Tumor | fatty_acid_score_high | 1.586049 |
| TCGA-05-4403-01A | 1.795847307 | Tumor | fatty_acid_score_high | 1.586049 |
| TCGA-55-6971-11A | 1.797042767 | Normal | fatty_acid_score_high | 1.586049 |
| TCGA-MP-A4T7-01A | 1.798463026 | Tumor | fatty_acid_score_high | 1.586049 |
| TCGA-55-6984-11A | 1.798779082 | Normal | fatty_acid_score_high | 1.586049 |
| TCGA-35-3615-01A | 1.799223438 | Tumor | fatty_acid_score_high | 1.586049 |
| TCGA-49-4514-01A | 1.799635432 | Tumor | fatty_acid_score_high | 1.586049 |
| TCGA-55-6968-11A | 1.799887042 | Normal | fatty_acid_score_high | 1.586049 |
| TCGA-78-7148-01A | 1.802310859 | Tumor | fatty_acid_score_high | 1.586049 |
| TCGA-78-7633-01A | 1.808091206 | Tumor | fatty_acid_score_high | 1.586049 |
| TCGA-78-7153-01A | 1.809407798 | Tumor | fatty_acid_score_high | 1.586049 |
| TCGA-97-8179-01A | 1.810261901 | Tumor | fatty_acid_score_high | 1.586049 |
| TCGA-62-A470-01A | 1.81096672 | Tumor | fatty_acid_score_high | 1.586049 |
| TCGA-49-6742-11A | 1.813522216 | Normal | fatty_acid_score_high | 1.586049 |
| TCGA-05-5423-01A | 1.814919342 | Tumor | fatty_acid_score_high | 1.586049 |
| TCGA-49-6761-11A | 1.815880253 | Normal | fatty_acid_score_high | 1.586049 |
| TCGA-38-4627-11A | 1.817314166 | Normal | fatty_acid_score_high | 1.586049 |
| TCGA-44-2668-11A | 1.818675844 | Normal | fatty_acid_score_high | 1.586049 |
| TCGA-86-8281-01A | 1.819342472 | Tumor | fatty_acid_score_high | 1.586049 |
| TCGA-55-6972-01A | 1.820988205 | Tumor | fatty_acid_score_high | 1.586049 |
| TCGA-97-A4M2-01A | 1.821247467 | Tumor | fatty_acid_score_high | 1.586049 |
| TCGA-91-6836-11A | 1.823142233 | Normal | fatty_acid_score_high | 1.586049 |
| TCGA-78-7160-01A | 1.82355328 | Tumor | fatty_acid_score_high | 1.586049 |
| TCGA-69-8254-01A | 1.825551947 | Tumor | fatty_acid_score_high | 1.586049 |
| TCGA-50-5930-11A | 1.828153843 | Normal | fatty_acid_score_high | 1.586049 |
| TCGA-05-4384-01A | 1.828194552 | Tumor | fatty_acid_score_high | 1.586049 |
| TCGA-69-8255-01A | 1.828969478 | Tumor | fatty_acid_score_high | 1.586049 |
| TCGA-05-5420-01A | 1.830683141 | Tumor | fatty_acid_score_high | 1.586049 |
| TCGA-44-6144-11A | 1.832826143 | Normal | fatty_acid_score_high | 1.586049 |
| TCGA-44-6776-11A | 1.837518812 | Normal | fatty_acid_score_high | 1.586049 |
| TCGA-75-6212-01A | 1.838563698 | Tumor | fatty_acid_score_high | 1.586049 |
| TCGA-62-8395-01A | 1.844052704 | Tumor | fatty_acid_score_high | 1.586049 |
| TCGA-55-6983-11A | 1.850074257 | Normal | fatty_acid_score_high | 1.586049 |
| TCGA-55-6985-11A | 1.850465974 | Normal | fatty_acid_score_high | 1.586049 |
| TCGA-49-4490-11A | 1.851017296 | Normal | fatty_acid_score_high | 1.586049 |
| TCGA-91-A4BD-01A | 1.851148366 | Tumor | fatty_acid_score_high | 1.586049 |
| TCGA-05-5429-01A | 1.851629025 | Tumor | fatty_acid_score_high | 1.586049 |
| TCGA-49-6745-11A | 1.8533916 | Normal | fatty_acid_score_high | 1.586049 |
| TCGA-05-4389-01A | 1.857124904 | Tumor | fatty_acid_score_high | 1.586049 |
| TCGA-44-6776-01A | 1.863645794 | Tumor | fatty_acid_score_high | 1.586049 |
| TCGA-49-4512-11A | 1.867397732 | Normal | fatty_acid_score_high | 1.586049 |
| TCGA-49-6742-01A | 1.8683095 | Tumor | fatty_acid_score_high | 1.586049 |
| TCGA-78-7162-01A | 1.870113806 | Tumor | fatty_acid_score_high | 1.586049 |
| TCGA-91-8496-01A | 1.890737776 | Tumor | fatty_acid_score_high | 1.586049 |
| TCGA-73-4676-11A | 1.890947089 | Normal | fatty_acid_score_high | 1.586049 |
| TCGA-44-7671-01A | 1.892992376 | Tumor | fatty_acid_score_high | 1.586049 |
| TCGA-73-4677-01A | 1.893353647 | Tumor | fatty_acid_score_high | 1.586049 |
| TCGA-44-2655-01A | 1.904586892 | Tumor | fatty_acid_score_high | 1.586049 |
| TCGA-78-7156-01A | 1.911457194 | Tumor | fatty_acid_score_high | 1.586049 |
| TCGA-91-6829-11A | 1.914785187 | Normal | fatty_acid_score_high | 1.586049 |
| TCGA-91-6849-01A | 1.91715873 | Tumor | fatty_acid_score_high | 1.586049 |
| TCGA-67-3770-01A | 1.917559232 | Tumor | fatty_acid_score_high | 1.586049 |
| TCGA-05-4422-01A | 1.925040114 | Tumor | fatty_acid_score_high | 1.586049 |
| TCGA-38-4632-11A | 1.930996727 | Normal | fatty_acid_score_high | 1.586049 |
| TCGA-38-4625-11A | 1.938485971 | Normal | fatty_acid_score_high | 1.586049 |
| TCGA-38-4626-11A | 1.950086392 | Normal | fatty_acid_score_high | 1.586049 |
| TCGA-55-A492-01A | 1.971291932 | Tumor | fatty_acid_score_high | 1.586049 |
| TCGA-49-4486-01A | 2.02630199 | Tumor | fatty_acid_score_high | 1.586049 |
