# Supplementary material for: Prognostic value of lncRNAs related to fatty acid metabolism in lung adenocarcinoma and their correlation with tumor microenvironment based on bioinformatics analysis
Source: Front Oncol. 2022 Oct 10;12:1022097. doi: 10.3389/fonc.2022.1022097 (PMC9590110; doi:10.3389/fonc.2022.1022097)
Supplement: Supplementary Table 1 — All samples were divided into high and low fatty acid metabolism score groups based on the median value of this score. [file DataSheet_1.zip › raw data and R code for checking/raw data/10.docx]

| miRNA | lnc | type.x | type.y |
| --- | --- | --- | --- |
| hsa-let-7a-5p | CTA-384D8.35 | up.lnc | down.mi |
| hsa-let-7b-5p | CTA-384D8.35 | up.lnc | down.mi |
| hsa-let-7c-5p | CTA-384D8.35 | up.lnc | down.mi |
| hsa-let-7e-5p | CTA-384D8.35 | up.lnc | down.mi |
| hsa-let-7f-5p | CTA-384D8.35 | up.lnc | down.mi |
| hsa-let-7g-5p | CTA-384D8.35 | up.lnc | down.mi |
| hsa-miR-15b-5p | Z83851.4 | up.lnc | down.mi |
| hsa-miR-195-5p | Z83851.4 | up.lnc | down.mi |
| hsa-miR-218-5p | CTA-384D8.35 | up.lnc | down.mi |
| hsa-miR-30a-3p | CTA-384D8.35 | up.lnc | down.mi |
| hsa-miR-30b-3p | RP5-1059L7.1 | up.lnc | down.mi |
| hsa-miR-30c-2-3p | CTA-384D8.35 | up.lnc | down.mi |
| hsa-miR-486-3p | CTA-384D8.35 | up.lnc | down.mi |
| hsa-miR-486-5p | RP5-1059L7.1 | up.lnc | down.mi |
| hsa-miR-605-5p | Z83851.4 | up.lnc | down.mi |
| hsa-let-7a-2-3p | RP11-401P9.4 | down.lnc | up.mi |
| hsa-let-7c-3p | RP11-401P9.4 | down.lnc | up.mi |
| hsa-let-7g-3p | RP11-401P9.4 | down.lnc | up.mi |
| hsa-miR-130b-5p | RP11-401P9.4 | down.lnc | up.mi |
| hsa-miR-134-5p | RP11-401P9.4 | down.lnc | up.mi |
| hsa-miR-135b-3p | RP11-401P9.4 | down.lnc | up.mi |
| hsa-miR-142-5p | RP11-401P9.4 | down.lnc | up.mi |
| hsa-miR-148a-5p | RP11-401P9.4 | down.lnc | up.mi |
| hsa-miR-148b-5p | RP11-401P9.4 | down.lnc | up.mi |
| hsa-miR-15a-5p | RP11-259K15.2 | down.lnc | up.mi |
| hsa-miR-193a-3p | RP11-259K15.2 | down.lnc | up.mi |
| hsa-miR-193b-3p | RP11-259K15.2 | down.lnc | up.mi |
| hsa-miR-20b-3p | RP11-401P9.4 | down.lnc | up.mi |
| hsa-miR-21-5p | RP11-401P9.4 | down.lnc | up.mi |
| hsa-miR-224-5p | RP11-401P9.4 | down.lnc | up.mi |
| hsa-miR-3136-5p | RP11-401P9.4 | down.lnc | up.mi |
| hsa-miR-323a-3p | RP11-401P9.4 | down.lnc | up.mi |
| hsa-miR-324-3p | RP11-401P9.4 | down.lnc | up.mi |
| hsa-miR-339-5p | RP11-401P9.4 | down.lnc | up.mi |
| hsa-miR-34a-3p | RP11-259K15.2 | down.lnc | up.mi |
| hsa-miR-361-3p | RP11-401P9.4 | down.lnc | up.mi |
| hsa-miR-3677-5p | RP11-401P9.4 | down.lnc | up.mi |
| hsa-miR-382-5p | RP11-401P9.4 | down.lnc | up.mi |
| hsa-miR-3913-5p | RP11-259K15.2 | down.lnc | up.mi |
| hsa-miR-409-3p | RP11-401P9.4 | down.lnc | up.mi |
| hsa-miR-425-5p | RP11-401P9.4 | down.lnc | up.mi |
| hsa-miR-431-5p | RP11-259K15.2 | down.lnc | up.mi |
| hsa-miR-4668-3p | RP11-401P9.4 | down.lnc | up.mi |
| hsa-miR-493-5p | RP11-401P9.4 | down.lnc | up.mi |
| hsa-miR-503-5p | RP11-259K15.2 | down.lnc | up.mi |
| hsa-miR-590-3p | RP11-259K15.2 | down.lnc | up.mi |
| hsa-miR-590-5p | RP11-401P9.4 | down.lnc | up.mi |
| hsa-miR-651-5p | RP11-259K15.2 | down.lnc | up.mi |
| hsa-miR-767-3p | RP11-4B16.3 | down.lnc | up.mi |
