# Supplementary material for: Prognostic value of lncRNAs related to fatty acid metabolism in lung adenocarcinoma and their correlation with tumor microenvironment based on bioinformatics analysis
Source: Front Oncol. 2022 Oct 10;12:1022097. doi: 10.3389/fonc.2022.1022097 (PMC9590110; doi:10.3389/fonc.2022.1022097)
Supplement: Supplementary Table 1 — All samples were divided into high and low fatty acid metabolism score groups based on the median value of this score. [file DataSheet_1.zip › raw data and R code for checking/raw data/11.docx]

| mRNA | miRNA | type.y | type.x |
| --- | --- | --- | --- |
| 6-Mar | hsa-miR-30c-2-3p | down.mi | up.m |
| 6-Mar | hsa-miR-30d-5p | down.mi | up.m |
| 6-Mar | hsa-let-7b-5p | down.mi | up.m |
| 6-Mar | hsa-miR-30a-5p | down.mi | up.m |
| 6-Mar | hsa-miR-30b-3p | down.mi | up.m |
| 9-Sep | hsa-miR-423-5p | down.mi | up.m |
| 9-Sep | hsa-miR-3614-5p | down.mi | up.m |
| AARS2 | hsa-miR-378a-5p | down.mi | up.m |
| AARSD1 | hsa-let-7b-5p | down.mi | up.m |
| AATF | hsa-let-7b-5p | down.mi | up.m |
| ABCB6 | hsa-miR-1-3p | down.mi | up.m |
| ABCC2 | hsa-miR-490-3p | down.mi | up.m |
| ABCC5 | hsa-miR-423-5p | down.mi | up.m |
| ABCF2 | hsa-miR-486-5p | down.mi | up.m |
| ABCF2 | hsa-miR-584-5p | down.mi | up.m |
| ABCF2 | hsa-miR-218-5p | down.mi | up.m |
| ABHD11 | hsa-miR-1-3p | down.mi | up.m |
| ABHD11 | hsa-miR-218-5p | down.mi | up.m |
| ABHD12 | hsa-miR-1-3p | down.mi | up.m |
| ABHD12 | hsa-miR-1247-3p | down.mi | up.m |
| ABRACL | hsa-miR-145-3p | down.mi | up.m |
| ABRACL | hsa-miR-378a-5p | down.mi | up.m |
| ACAD8 | hsa-let-7a-5p | down.mi | up.m |
| ACP1 | hsa-let-7b-3p | down.mi | up.m |
| ACTL6A | hsa-miR-206 | down.mi | up.m |
| ACVR1B | hsa-let-7c-5p | down.mi | up.m |
| ACVR1B | hsa-let-7a-5p | down.mi | up.m |
| ADAM12 | hsa-miR-30b-3p | down.mi | up.m |
| ADAM12 | hsa-miR-1-3p | down.mi | up.m |
| ADAM12 | hsa-miR-218-5p | down.mi | up.m |
| ADAMTS14 | hsa-miR-423-5p | down.mi | up.m |
| ADAR | hsa-miR-30b-3p | down.mi | up.m |
| ADAR | hsa-miR-1-3p | down.mi | up.m |
| ADORA2B | hsa-miR-30a-3p | down.mi | up.m |
| ADPRHL1 | hsa-miR-30a-5p | down.mi | up.m |
| ADPRHL1 | hsa-miR-30d-5p | down.mi | up.m |
| ADRM1 | hsa-miR-30b-3p | down.mi | up.m |
| ADRM1 | hsa-let-7b-5p | down.mi | up.m |
| AFMID | hsa-miR-374a-5p | down.mi | up.m |
| AGMAT | hsa-miR-195-3p | down.mi | up.m |
| AGMAT | hsa-miR-1-3p | down.mi | up.m |
| AGMAT | hsa-let-7e-5p | down.mi | up.m |
| AGRN | hsa-miR-1-3p | down.mi | up.m |
| AHCY | hsa-miR-140-3p | down.mi | up.m |
| AHCY | hsa-miR-340-5p | down.mi | up.m |
| AHDC1 | hsa-miR-486-3p | down.mi | up.m |
| AHDC1 | hsa-miR-423-5p | down.mi | up.m |
| AHDC1 | hsa-miR-30b-3p | down.mi | up.m |
| AHNAK2 | hsa-miR-195-5p | down.mi | up.m |
| AHNAK2 | hsa-miR-1-3p | down.mi | up.m |
| AHNAK2 | hsa-miR-15b-5p | down.mi | up.m |
| AHSA2 | hsa-miR-374a-5p | down.mi | up.m |
| AIFM2 | hsa-miR-140-3p | down.mi | up.m |
| AIFM2 | hsa-miR-1-3p | down.mi | up.m |
| AIFM2 | hsa-miR-15b-5p | down.mi | up.m |
| AIM2 | hsa-miR-1-3p | down.mi | up.m |
| AK2 | hsa-miR-204-5p | down.mi | up.m |
| AK2 | hsa-miR-423-5p | down.mi | up.m |
| AK2 | hsa-miR-30a-5p | down.mi | up.m |
| AK4 | hsa-let-7f-5p | down.mi | up.m |
| AK4 | hsa-let-7c-5p | down.mi | up.m |
| AK4 | hsa-let-7g-5p | down.mi | up.m |
| AK4 | hsa-let-7b-5p | down.mi | up.m |
| AK4 | hsa-let-7e-5p | down.mi | up.m |
| AK4 | hsa-let-7a-5p | down.mi | up.m |
| AKR1B10 | hsa-miR-195-5p | down.mi | up.m |
| AKR1B10 | hsa-miR-15b-5p | down.mi | up.m |
| ALDOA | hsa-miR-378a-5p | down.mi | up.m |
| ALDOA | hsa-miR-6720-3p | down.mi | up.m |
| ALDOA | hsa-miR-30b-3p | down.mi | up.m |
| ALG3 | hsa-let-7b-5p | down.mi | up.m |
| ALG3 | hsa-miR-1-3p | down.mi | up.m |
| ALYREF | hsa-miR-605-5p | down.mi | up.m |
| AMPD2 | hsa-let-7a-5p | down.mi | up.m |
| AMPD2 | hsa-let-7e-5p | down.mi | up.m |
| ANGPTL4 | hsa-miR-1-3p | down.mi | up.m |
| ANKIB1 | hsa-miR-1-3p | down.mi | up.m |
| ANKRD13B | hsa-miR-423-5p | down.mi | up.m |
| ANKRD13B | hsa-miR-195-5p | down.mi | up.m |
| ANKRD13B | hsa-miR-15b-5p | down.mi | up.m |
| ANKZF1 | hsa-let-7b-5p | down.mi | up.m |
| ANP32E | hsa-miR-1-3p | down.mi | up.m |
| ANP32E | hsa-miR-195-3p | down.mi | up.m |
| ANP32E | hsa-miR-206 | down.mi | up.m |
| ANP32E | hsa-let-7b-5p | down.mi | up.m |
| AP1S1 | hsa-let-7g-5p | down.mi | up.m |
| AP1S1 | hsa-let-7a-5p | down.mi | up.m |
| AP1S1 | hsa-miR-1-3p | down.mi | up.m |
| AP1S1 | hsa-let-7f-5p | down.mi | up.m |
| AP1S1 | hsa-let-7c-5p | down.mi | up.m |
| AP1S1 | hsa-let-7b-5p | down.mi | up.m |
| AP1S1 | hsa-miR-204-5p | down.mi | up.m |
| AP1S1 | hsa-let-7e-5p | down.mi | up.m |
| AP5Z1 | hsa-miR-15b-5p | down.mi | up.m |
| AP5Z1 | hsa-miR-195-5p | down.mi | up.m |
| AP5Z1 | hsa-miR-140-3p | down.mi | up.m |
| APEX1 | hsa-miR-27a-5p | down.mi | up.m |
| APEX1 | hsa-miR-423-5p | down.mi | up.m |
| APEX1 | hsa-miR-99a-5p | down.mi | up.m |
| APRT | hsa-miR-15b-5p | down.mi | up.m |
| APRT | hsa-let-7b-5p | down.mi | up.m |
| ARF4 | hsa-miR-1-3p | down.mi | up.m |
| ARG2 | hsa-let-7a-5p | down.mi | up.m |
| ARHGEF39 | hsa-miR-378a-5p | down.mi | up.m |
| ARL4C | hsa-miR-486-3p | down.mi | up.m |
| ARL6IP1 | hsa-miR-195-3p | down.mi | up.m |
| ARL6IP1 | hsa-miR-1247-5p | down.mi | up.m |
| ARL6IP1 | hsa-miR-133a-3p | down.mi | up.m |
| ARL6IP1 | hsa-let-7b-5p | down.mi | up.m |
| ARL6IP1 | hsa-let-7c-5p | down.mi | up.m |
| ARL6IP1 | hsa-miR-30a-3p | down.mi | up.m |
| ARL6IP1 | hsa-miR-218-5p | down.mi | up.m |
| ARL6IP1 | hsa-miR-423-5p | down.mi | up.m |
| ARL6IP1 | hsa-let-7f-5p | down.mi | up.m |
| ARL9 | hsa-miR-605-5p | down.mi | up.m |
| ARMC10 | hsa-miR-1-3p | down.mi | up.m |
| ARNTL2 | hsa-miR-204-5p | down.mi | up.m |
| ARPC1A | hsa-miR-423-5p | down.mi | up.m |
| ARSE | hsa-miR-204-5p | down.mi | up.m |
| ARTN | hsa-miR-223-3p | down.mi | up.m |
| ASCC3 | hsa-let-7b-5p | down.mi | up.m |
| ASCC3 | hsa-let-7a-5p | down.mi | up.m |
| ASCC3 | hsa-miR-30a-5p | down.mi | up.m |
| ASCC3 | hsa-let-7f-5p | down.mi | up.m |
| ASPH | hsa-miR-423-5p | down.mi | up.m |
| ASPH | hsa-miR-1-3p | down.mi | up.m |
| ASPM | hsa-let-7a-5p | down.mi | up.m |
| ASPM | hsa-miR-218-5p | down.mi | up.m |
| ATAD3B | hsa-let-7b-5p | down.mi | up.m |
| ATAD5 | hsa-miR-30c-2-3p | down.mi | up.m |
| ATAD5 | hsa-miR-195-5p | down.mi | up.m |
| ATAD5 | hsa-miR-15b-5p | down.mi | up.m |
| ATF6B | hsa-miR-204-5p | down.mi | up.m |
| ATL2 | hsa-miR-30a-5p | down.mi | up.m |
| ATL2 | hsa-miR-218-5p | down.mi | up.m |
| ATP13A1 | hsa-miR-1-3p | down.mi | up.m |
| ATP1B1 | hsa-miR-218-5p | down.mi | up.m |
| ATP2A2 | hsa-let-7b-5p | down.mi | up.m |
| ATP2A2 | hsa-miR-15b-5p | down.mi | up.m |
| ATP2A2 | hsa-let-7f-5p | down.mi | up.m |
| ATP2A2 | hsa-miR-30a-5p | down.mi | up.m |
| ATP2A2 | hsa-let-7a-5p | down.mi | up.m |
| ATP2A2 | hsa-let-7c-5p | down.mi | up.m |
| ATP2A2 | hsa-miR-30d-5p | down.mi | up.m |
| ATP5G3 | hsa-miR-195-5p | down.mi | up.m |
| ATP5G3 | hsa-miR-15b-5p | down.mi | up.m |
| ATXN2L | hsa-let-7f-5p | down.mi | up.m |
| ATXN2L | hsa-let-7a-5p | down.mi | up.m |
| ATXN2L | hsa-let-7e-5p | down.mi | up.m |
| ATXN2L | hsa-let-7b-5p | down.mi | up.m |
| ATXN2L | hsa-miR-340-5p | down.mi | up.m |
| ATXN7L3 | hsa-let-7a-5p | down.mi | up.m |
| ATXN7L3 | hsa-let-7f-5p | down.mi | up.m |
| ATXN7L3 | hsa-let-7g-5p | down.mi | up.m |
| ATXN7L3 | hsa-let-7c-5p | down.mi | up.m |
| ATXN7L3 | hsa-miR-486-3p | down.mi | up.m |
| ATXN7L3 | hsa-let-7b-5p | down.mi | up.m |
| ATXN7L3 | hsa-let-7e-5p | down.mi | up.m |
| AUP1 | hsa-let-7b-5p | down.mi | up.m |
| AURKA | hsa-let-7b-5p | down.mi | up.m |
| AURKAIP1 | hsa-miR-195-5p | down.mi | up.m |
| AURKAIP1 | hsa-miR-15b-5p | down.mi | up.m |
| AURKAIP1 | hsa-miR-218-5p | down.mi | up.m |
| AURKB | hsa-let-7e-5p | down.mi | up.m |
| AURKB | hsa-let-7a-5p | down.mi | up.m |
| AURKB | hsa-let-7b-5p | down.mi | up.m |
| AVEN | hsa-miR-30a-5p | down.mi | up.m |
| AVL9 | hsa-miR-195-5p | down.mi | up.m |
| AVL9 | hsa-miR-1247-3p | down.mi | up.m |
| AVL9 | hsa-miR-30a-5p | down.mi | up.m |
| AVL9 | hsa-miR-218-5p | down.mi | up.m |
| AVL9 | hsa-miR-15b-5p | down.mi | up.m |
| AVL9 | hsa-miR-30d-5p | down.mi | up.m |
| AXIN1 | hsa-miR-218-5p | down.mi | up.m |
| AZIN2 | hsa-miR-30d-5p | down.mi | up.m |
| B3GALNT2 | hsa-miR-139-5p | down.mi | up.m |
| B3GALNT2 | hsa-miR-144-3p | down.mi | up.m |
| B3GALNT2 | hsa-miR-1247-3p | down.mi | up.m |
| B3GNT6 | hsa-miR-3614-5p | down.mi | up.m |
| B4GALNT3 | hsa-let-7a-5p | down.mi | up.m |
| B4GALNT3 | hsa-miR-204-5p | down.mi | up.m |
| B4GALNT3 | hsa-miR-378a-3p | down.mi | up.m |
| B4GALNT4 | hsa-miR-423-5p | down.mi | up.m |
| B4GALNT4 | hsa-miR-486-3p | down.mi | up.m |
| B4GALT7 | hsa-miR-1247-3p | down.mi | up.m |
| BACE2 | hsa-miR-378a-5p | down.mi | up.m |
| BACE2 | hsa-miR-4732-3p | down.mi | up.m |
| BAK1 | hsa-miR-221-5p | down.mi | up.m |
| BAK1 | hsa-miR-30c-2-3p | down.mi | up.m |
| BAK1 | hsa-miR-423-5p | down.mi | up.m |
| BAK1 | hsa-miR-30b-3p | down.mi | up.m |
| BASP1 | hsa-miR-486-5p | down.mi | up.m |
| BBC3 | hsa-miR-143-3p | down.mi | up.m |
| BBC3 | hsa-miR-378a-5p | down.mi | up.m |
| BCCIP | hsa-miR-423-5p | down.mi | up.m |
| BCCIP | hsa-let-7c-5p | down.mi | up.m |
| BCKDK | hsa-miR-6720-3p | down.mi | up.m |
| BCL2L12 | hsa-miR-15b-5p | down.mi | up.m |
| BCL2L12 | hsa-miR-195-5p | down.mi | up.m |
| BCL2L15 | hsa-miR-139-5p | down.mi | up.m |
| BCL9 | hsa-miR-30c-2-3p | down.mi | up.m |
| BCL9 | hsa-miR-30a-5p | down.mi | up.m |
| BCL9 | hsa-miR-218-5p | down.mi | up.m |
| BCL9 | hsa-miR-605-5p | down.mi | up.m |
| BCL9 | hsa-miR-30d-5p | down.mi | up.m |
| BIRC5 | hsa-miR-1247-3p | down.mi | up.m |
| BIRC5 | hsa-let-7b-5p | down.mi | up.m |
| BIRC5 | hsa-miR-218-5p | down.mi | up.m |
| BIRC5 | hsa-miR-195-5p | down.mi | up.m |
| BOLA3 | hsa-miR-374a-5p | down.mi | up.m |
| BPNT1 | hsa-miR-30c-2-3p | down.mi | up.m |
| BPNT1 | hsa-miR-30b-3p | down.mi | up.m |
| BPNT1 | hsa-miR-1247-3p | down.mi | up.m |
| BRAT1 | hsa-miR-15b-5p | down.mi | up.m |
| BRDT | hsa-miR-1-3p | down.mi | up.m |
| BRI3BP | hsa-miR-30b-3p | down.mi | up.m |
| BRI3BP | hsa-miR-30c-2-3p | down.mi | up.m |
| BRI3BP | hsa-let-7e-5p | down.mi | up.m |
| BRI3BP | hsa-let-7b-5p | down.mi | up.m |
| BRI3BP | hsa-miR-1-3p | down.mi | up.m |
| BRI3BP | hsa-let-7f-5p | down.mi | up.m |
| BRI3BP | hsa-let-7g-5p | down.mi | up.m |
| BRI3BP | hsa-let-7c-5p | down.mi | up.m |
| BRI3BP | hsa-let-7b-3p | down.mi | up.m |
| BRI3BP | hsa-let-7a-5p | down.mi | up.m |
| BRIP1 | hsa-miR-490-3p | down.mi | up.m |
| BRIP1 | hsa-miR-34b-3p | down.mi | up.m |
| BRIP1 | hsa-miR-378a-5p | down.mi | up.m |
| BRIP1 | hsa-miR-3614-5p | down.mi | up.m |
| BRIX1 | hsa-miR-340-5p | down.mi | up.m |
| BRIX1 | hsa-miR-3614-5p | down.mi | up.m |
| BRIX1 | hsa-miR-34b-3p | down.mi | up.m |
| BRMS1 | hsa-miR-221-5p | down.mi | up.m |
| BROX | hsa-miR-34b-3p | down.mi | up.m |
| BRPF3 | hsa-let-7b-3p | down.mi | up.m |
| BRPF3 | hsa-miR-1-3p | down.mi | up.m |
| BRPF3 | hsa-let-7b-5p | down.mi | up.m |
| BSPRY | hsa-miR-15b-5p | down.mi | up.m |
| BSPRY | hsa-miR-195-5p | down.mi | up.m |
| BUB1 | hsa-miR-145-3p | down.mi | up.m |
| BUB1 | hsa-miR-30c-2-3p | down.mi | up.m |
| BUB1 | hsa-miR-340-5p | down.mi | up.m |
| BUB3 | hsa-miR-605-5p | down.mi | up.m |
| BYSL | hsa-miR-378a-3p | down.mi | up.m |
| BZW1 | hsa-let-7f-5p | down.mi | up.m |
| BZW1 | hsa-let-7g-5p | down.mi | up.m |
| BZW1 | hsa-let-7a-5p | down.mi | up.m |
| BZW1 | hsa-let-7c-5p | down.mi | up.m |
| BZW1 | hsa-let-7e-5p | down.mi | up.m |
| BZW1 | hsa-miR-204-5p | down.mi | up.m |
| BZW1 | hsa-miR-195-5p | down.mi | up.m |
| BZW1 | hsa-miR-15b-5p | down.mi | up.m |
| BZW1 | hsa-miR-34c-3p | down.mi | up.m |
| BZW1 | hsa-let-7b-5p | down.mi | up.m |
| BZW1 | hsa-miR-144-3p | down.mi | up.m |
| BZW2 | hsa-let-7b-5p | down.mi | up.m |
| C11orf24 | hsa-miR-195-5p | down.mi | up.m |
| C11orf24 | hsa-miR-15b-5p | down.mi | up.m |
| C11orf24 | hsa-miR-3614-5p | down.mi | up.m |
| C11orf24 | hsa-miR-133b | down.mi | up.m |
| C11orf24 | hsa-miR-133a-3p | down.mi | up.m |
| C11orf84 | hsa-miR-34b-3p | down.mi | up.m |
| C11orf98 | hsa-miR-490-3p | down.mi | up.m |
| C11orf98 | hsa-miR-30b-3p | down.mi | up.m |
| C16orf58 | hsa-miR-517b-3p | down.mi | up.m |
| C16orf58 | hsa-miR-195-5p | down.mi | up.m |
| C16orf58 | hsa-miR-517a-3p | down.mi | up.m |
| C16orf58 | hsa-miR-125b-2-3p | down.mi | up.m |
| C16orf58 | hsa-miR-15b-5p | down.mi | up.m |
| C19orf48 | hsa-let-7e-5p | down.mi | up.m |
| C1GALT1 | hsa-let-7a-5p | down.mi | up.m |
| C1GALT1 | hsa-let-7b-5p | down.mi | up.m |
| C1orf159 | hsa-miR-218-5p | down.mi | up.m |
| C1orf226 | hsa-miR-15b-5p | down.mi | up.m |
| C1orf226 | hsa-miR-195-5p | down.mi | up.m |
| C1orf27 | hsa-miR-1-3p | down.mi | up.m |
| C1orf27 | hsa-let-7b-5p | down.mi | up.m |
| C1orf43 | hsa-miR-340-5p | down.mi | up.m |
| C1orf43 | hsa-miR-15b-5p | down.mi | up.m |
| C1orf43 | hsa-miR-221-5p | down.mi | up.m |
| C1orf56 | hsa-miR-1-3p | down.mi | up.m |
| C1QBP | hsa-miR-30a-3p | down.mi | up.m |
| C1QBP | hsa-miR-378a-5p | down.mi | up.m |
| C1QTNF6 | hsa-miR-6720-3p | down.mi | up.m |
| C2CD4A | hsa-let-7a-5p | down.mi | up.m |
| C2orf15 | hsa-miR-143-3p | down.mi | up.m |
| C5orf51 | hsa-let-7a-5p | down.mi | up.m |
| C5orf51 | hsa-let-7e-5p | down.mi | up.m |
| C5orf51 | hsa-let-7f-5p | down.mi | up.m |
| C5orf51 | hsa-miR-1-3p | down.mi | up.m |
| C5orf51 | hsa-let-7g-5p | down.mi | up.m |
| C5orf51 | hsa-let-7c-5p | down.mi | up.m |
| C5orf51 | hsa-let-7b-5p | down.mi | up.m |
| C5orf51 | hsa-miR-374a-5p | down.mi | up.m |
| C5orf51 | hsa-miR-144-5p | down.mi | up.m |
| C5orf51 | hsa-miR-206 | down.mi | up.m |
| C5orf51 | hsa-miR-30a-3p | down.mi | up.m |
| C7orf43 | hsa-miR-30a-5p | down.mi | up.m |
| C7orf43 | hsa-miR-30d-5p | down.mi | up.m |
| C8orf76 | hsa-miR-30d-5p | down.mi | up.m |
| C8orf76 | hsa-miR-30a-5p | down.mi | up.m |
| CAD | hsa-miR-486-3p | down.mi | up.m |
| CAD | hsa-miR-1-3p | down.mi | up.m |
| CALR | hsa-miR-1-3p | down.mi | up.m |
| CALR | hsa-miR-516a-5p | down.mi | up.m |
| CALR | hsa-miR-486-3p | down.mi | up.m |
| CALU | hsa-miR-15b-5p | down.mi | up.m |
| CALU | hsa-let-7e-5p | down.mi | up.m |
| CALU | hsa-let-7g-5p | down.mi | up.m |
| CALU | hsa-let-7c-5p | down.mi | up.m |
| CALU | hsa-let-7a-5p | down.mi | up.m |
| CALU | hsa-miR-195-5p | down.mi | up.m |
| CALU | hsa-let-7b-5p | down.mi | up.m |
| CALU | hsa-let-7f-5p | down.mi | up.m |
| CANT1 | hsa-miR-4732-3p | down.mi | up.m |
| CAPN15 | hsa-miR-423-5p | down.mi | up.m |
| CAPN15 | hsa-miR-486-3p | down.mi | up.m |
| CAPN15 | hsa-miR-140-3p | down.mi | up.m |
| CAPN15 | hsa-let-7d-3p | down.mi | up.m |
| CARD11 | hsa-miR-218-5p | down.mi | up.m |
| CASP2 | hsa-miR-6892-5p | down.mi | up.m |
| CASP3 | hsa-let-7a-5p | down.mi | up.m |
| CASP3 | hsa-miR-30d-5p | down.mi | up.m |
| CASP3 | hsa-miR-138-5p | down.mi | up.m |
| CASP3 | hsa-let-7g-5p | down.mi | up.m |
| CASP3 | hsa-let-7b-3p | down.mi | up.m |
| CASP3 | hsa-miR-30a-5p | down.mi | up.m |
| CASP3 | hsa-miR-374a-5p | down.mi | up.m |
| CASP3 | hsa-let-7c-5p | down.mi | up.m |
| CBX1 | hsa-miR-218-5p | down.mi | up.m |
| CBX2 | hsa-miR-30a-5p | down.mi | up.m |
| CBX2 | hsa-miR-195-5p | down.mi | up.m |
| CBX2 | hsa-miR-30d-5p | down.mi | up.m |
| CBX2 | hsa-miR-1-3p | down.mi | up.m |
| CBX2 | hsa-miR-15b-5p | down.mi | up.m |
| CBX3 | hsa-miR-30a-5p | down.mi | up.m |
| CBX3 | hsa-miR-30d-5p | down.mi | up.m |
| CBX4 | hsa-miR-374a-5p | down.mi | up.m |
| CBX4 | hsa-miR-195-5p | down.mi | up.m |
| CBX4 | hsa-miR-15b-5p | down.mi | up.m |
| CBX8 | hsa-miR-140-3p | down.mi | up.m |
| CBX8 | hsa-miR-184 | down.mi | up.m |
| CBX8 | hsa-miR-423-5p | down.mi | up.m |
| CBX8 | hsa-miR-218-5p | down.mi | up.m |
| CBX8 | hsa-miR-2110 | down.mi | up.m |
| CCDC134 | hsa-miR-1-3p | down.mi | up.m |
| CCDC134 | hsa-let-7b-5p | down.mi | up.m |
| CCDC137 | hsa-miR-2110 | down.mi | up.m |
| CCDC43 | hsa-miR-204-5p | down.mi | up.m |
| CCDC86 | hsa-miR-30a-3p | down.mi | up.m |
| CCL22 | hsa-miR-30b-3p | down.mi | up.m |
| CCL22 | hsa-miR-30c-2-3p | down.mi | up.m |
| CCL7 | hsa-let-7f-5p | down.mi | up.m |
| CCNA2 | hsa-let-7b-5p | down.mi | up.m |
| CCNB1 | hsa-let-7b-5p | down.mi | up.m |
| CCNB2 | hsa-let-7a-5p | down.mi | up.m |
| CCNB2 | hsa-let-7c-5p | down.mi | up.m |
| CCNB2 | hsa-let-7b-5p | down.mi | up.m |
| CCNB2 | hsa-let-7f-5p | down.mi | up.m |
| CCNE1 | hsa-miR-195-5p | down.mi | up.m |
| CCNE1 | hsa-miR-15b-5p | down.mi | up.m |
| CCNE1 | hsa-miR-144-5p | down.mi | up.m |
| CCNE1 | hsa-miR-30c-2-3p | down.mi | up.m |
| CCNE2 | hsa-miR-30a-5p | down.mi | up.m |
| CCNE2 | hsa-miR-195-5p | down.mi | up.m |
| CCNE2 | hsa-miR-15b-5p | down.mi | up.m |
| CCNE2 | hsa-miR-144-5p | down.mi | up.m |
| CCNF | hsa-miR-30d-5p | down.mi | up.m |
| CCNF | hsa-let-7b-5p | down.mi | up.m |
| CCNF | hsa-miR-30b-3p | down.mi | up.m |
| CCNF | hsa-let-7c-5p | down.mi | up.m |
| CCNF | hsa-miR-218-5p | down.mi | up.m |
| CCNF | hsa-miR-30c-2-3p | down.mi | up.m |
| CCNF | hsa-miR-423-5p | down.mi | up.m |
| CCNF | hsa-miR-30a-5p | down.mi | up.m |
| CCNL2 | hsa-miR-218-5p | down.mi | up.m |
| CCT2 | hsa-miR-144-3p | down.mi | up.m |
| CCT4 | hsa-miR-34c-3p | down.mi | up.m |
| CCT4 | hsa-miR-195-3p | down.mi | up.m |
| CCT5 | hsa-miR-139-5p | down.mi | up.m |
| CCT7 | hsa-miR-598-3p | down.mi | up.m |
| CD276 | hsa-let-7a-5p | down.mi | up.m |
| CD276 | hsa-miR-486-3p | down.mi | up.m |
| CD2AP | hsa-miR-195-5p | down.mi | up.m |
| CD2AP | hsa-miR-15b-5p | down.mi | up.m |
| CD2AP | hsa-miR-30d-5p | down.mi | up.m |
| CD2AP | hsa-let-7a-5p | down.mi | up.m |
| CD2AP | hsa-miR-1-3p | down.mi | up.m |
| CD2AP | hsa-miR-30a-5p | down.mi | up.m |
| CD3EAP | hsa-miR-140-3p | down.mi | up.m |
| CD46 | hsa-miR-340-5p | down.mi | up.m |
| CD46 | hsa-miR-150-3p | down.mi | up.m |
| CDC123 | hsa-miR-30a-5p | down.mi | up.m |
| CDC20 | hsa-miR-30a-5p | down.mi | up.m |
| CDC25A | hsa-let-7c-5p | down.mi | up.m |
| CDC25A | hsa-miR-140-3p | down.mi | up.m |
| CDC25A | hsa-miR-15b-5p | down.mi | up.m |
| CDC25A | hsa-miR-195-3p | down.mi | up.m |
| CDC25A | hsa-miR-195-5p | down.mi | up.m |
| CDC25A | hsa-let-7b-5p | down.mi | up.m |
| CDC34 | hsa-let-7b-5p | down.mi | up.m |
| CDC34 | hsa-let-7a-5p | down.mi | up.m |
| CDC7 | hsa-miR-30a-5p | down.mi | up.m |
| CDC7 | hsa-let-7a-5p | down.mi | up.m |
| CDC7 | hsa-miR-30d-5p | down.mi | up.m |
| CDCA3 | hsa-let-7e-5p | down.mi | up.m |
| CDCA3 | hsa-miR-3154 | down.mi | up.m |
| CDCA3 | hsa-miR-144-5p | down.mi | up.m |
| CDCA4 | hsa-miR-195-5p | down.mi | up.m |
| CDCA4 | hsa-miR-15b-5p | down.mi | up.m |
| CDCA4 | hsa-miR-140-3p | down.mi | up.m |
| CDCA7 | hsa-miR-340-5p | down.mi | up.m |
| CDCA7 | hsa-let-7b-5p | down.mi | up.m |
| CDCA8 | hsa-let-7b-5p | down.mi | up.m |
| CDCA8 | hsa-miR-140-3p | down.mi | up.m |
| CDCP1 | hsa-miR-30c-2-3p | down.mi | up.m |
| CDCP1 | hsa-miR-30b-3p | down.mi | up.m |
| CDCP1 | hsa-miR-30a-5p | down.mi | up.m |
| CDCP1 | hsa-miR-1-3p | down.mi | up.m |
| CDH1 | hsa-miR-30a-5p | down.mi | up.m |
| CDH1 | hsa-miR-204-5p | down.mi | up.m |
| CDH1 | hsa-miR-138-5p | down.mi | up.m |
| CDK1 | hsa-miR-195-5p | down.mi | up.m |
| CDK1 | hsa-miR-15b-5p | down.mi | up.m |
| CDK16 | hsa-miR-139-3p | down.mi | up.m |
| CDK4 | hsa-miR-378a-5p | down.mi | up.m |
| CDK4 | hsa-miR-15b-5p | down.mi | up.m |
| CDK4 | hsa-miR-1-3p | down.mi | up.m |
| CDK4 | hsa-miR-195-5p | down.mi | up.m |
| CDK4 | hsa-miR-34b-3p | down.mi | up.m |
| CDK4 | hsa-miR-486-5p | down.mi | up.m |
| CDK4 | hsa-miR-206 | down.mi | up.m |
| CDK5R1 | hsa-miR-133a-3p | down.mi | up.m |
| CDK5R1 | hsa-miR-133b | down.mi | up.m |
| CDKN2A | hsa-miR-423-5p | down.mi | up.m |
| CDKN2A | hsa-let-7g-5p | down.mi | up.m |
| CDKN2C | hsa-miR-218-5p | down.mi | up.m |
| CDT1 | hsa-miR-2110 | down.mi | up.m |
| CEACAM6 | hsa-miR-423-5p | down.mi | up.m |
| CELSR3 | hsa-miR-30d-5p | down.mi | up.m |
| CELSR3 | hsa-miR-30a-5p | down.mi | up.m |
| CELSR3 | hsa-miR-3614-5p | down.mi | up.m |
| CENPF | hsa-miR-1-3p | down.mi | up.m |
| CENPH | hsa-miR-605-5p | down.mi | up.m |
| CENPI | hsa-miR-1247-3p | down.mi | up.m |
| CENPK | hsa-miR-374a-5p | down.mi | up.m |
| CENPM | hsa-miR-34b-3p | down.mi | up.m |
| CENPM | hsa-miR-3614-5p | down.mi | up.m |
| CENPN | hsa-miR-486-5p | down.mi | up.m |
| CENPO | hsa-miR-218-5p | down.mi | up.m |
| CENPO | hsa-miR-7704 | down.mi | up.m |
| CEP170B | hsa-let-7f-5p | down.mi | up.m |
| CEP170B | hsa-let-7c-5p | down.mi | up.m |
| CEP170B | hsa-miR-184 | down.mi | up.m |
| CEP170B | hsa-let-7a-5p | down.mi | up.m |
| CEP55 | hsa-miR-195-5p | down.mi | up.m |
| CEP55 | hsa-miR-605-5p | down.mi | up.m |
| CEP55 | hsa-miR-15b-5p | down.mi | up.m |
| CEP72 | hsa-miR-30a-5p | down.mi | up.m |
| CERCAM | hsa-miR-340-5p | down.mi | up.m |
| CHAC1 | hsa-miR-195-5p | down.mi | up.m |
| CHAC1 | hsa-miR-144-3p | down.mi | up.m |
| CHAC1 | hsa-miR-15b-5p | down.mi | up.m |
| CHAC1 | hsa-miR-3154 | down.mi | up.m |
| CHAF1A | hsa-let-7b-5p | down.mi | up.m |
| CHAF1B | hsa-miR-1-3p | down.mi | up.m |
| CHCHD2 | hsa-miR-218-5p | down.mi | up.m |
| CHEK1 | hsa-miR-195-5p | down.mi | up.m |
| CHEK1 | hsa-miR-139-3p | down.mi | up.m |
| CHEK1 | hsa-miR-15b-5p | down.mi | up.m |
| CHML | hsa-miR-206 | down.mi | up.m |
| CHML | hsa-miR-1-3p | down.mi | up.m |
| CHMP4B | hsa-miR-15b-5p | down.mi | up.m |
| CHMP4B | hsa-miR-195-5p | down.mi | up.m |
| CHMP4B | hsa-miR-340-5p | down.mi | up.m |
| CHMP4C | hsa-miR-30c-2-3p | down.mi | up.m |
| CHORDC1 | hsa-miR-605-5p | down.mi | up.m |
| CHORDC1 | hsa-miR-30b-3p | down.mi | up.m |
| CHORDC1 | hsa-miR-34b-3p | down.mi | up.m |
| CHORDC1 | hsa-miR-204-5p | down.mi | up.m |
| CHORDC1 | hsa-miR-144-3p | down.mi | up.m |
| CHORDC1 | hsa-miR-30c-2-3p | down.mi | up.m |
| CHPF | hsa-miR-15b-5p | down.mi | up.m |
| CHPF2 | hsa-let-7b-5p | down.mi | up.m |
| CHST15 | hsa-miR-30d-5p | down.mi | up.m |
| CHST15 | hsa-miR-30a-5p | down.mi | up.m |
| CHTF18 | hsa-miR-378a-5p | down.mi | up.m |
| CHTOP | hsa-let-7a-5p | down.mi | up.m |
| CHTOP | hsa-let-7f-5p | down.mi | up.m |
| CHTOP | hsa-let-7c-5p | down.mi | up.m |
| CHTOP | hsa-let-7e-5p | down.mi | up.m |
| CHTOP | hsa-let-7g-5p | down.mi | up.m |
| CHTOP | hsa-let-7b-5p | down.mi | up.m |
| CIB2 | hsa-miR-4732-3p | down.mi | up.m |
| CIT | hsa-miR-486-5p | down.mi | up.m |
| CKAP2 | hsa-miR-490-3p | down.mi | up.m |
| CKAP2 | hsa-let-7b-5p | down.mi | up.m |
| CKAP2L | hsa-miR-221-5p | down.mi | up.m |
| CKAP4 | hsa-miR-1247-3p | down.mi | up.m |
| CKAP5 | hsa-miR-15b-5p | down.mi | up.m |
| CKS2 | hsa-let-7b-5p | down.mi | up.m |
| CLCF1 | hsa-miR-218-5p | down.mi | up.m |
| CLCN7 | hsa-miR-423-5p | down.mi | up.m |
| CLDN1 | hsa-miR-338-5p | down.mi | up.m |
| CLDN10 | hsa-miR-486-5p | down.mi | up.m |
| CLDN12 | hsa-let-7a-5p | down.mi | up.m |
| CLDN12 | hsa-let-7b-5p | down.mi | up.m |
| CLDN12 | hsa-let-7f-5p | down.mi | up.m |
| CLDN12 | hsa-let-7c-5p | down.mi | up.m |
| CLDN12 | hsa-let-7e-5p | down.mi | up.m |
| CLDN12 | hsa-let-7g-5p | down.mi | up.m |
| CLDN12 | hsa-miR-4732-3p | down.mi | up.m |
| CLDN12 | hsa-miR-1-3p | down.mi | up.m |
| CLDN12 | hsa-miR-206 | down.mi | up.m |
| CLDN4 | hsa-miR-2110 | down.mi | up.m |
| CLDN4 | hsa-let-7e-5p | down.mi | up.m |
| CLDN7 | hsa-miR-140-3p | down.mi | up.m |
| CLSPN | hsa-miR-423-5p | down.mi | up.m |
| CLSPN | hsa-miR-30b-3p | down.mi | up.m |
| CLSPN | hsa-miR-490-3p | down.mi | up.m |
| CLSPN | hsa-miR-15b-5p | down.mi | up.m |
| CLSPN | hsa-miR-195-5p | down.mi | up.m |
| CLSTN1 | hsa-miR-30a-3p | down.mi | up.m |
| CLSTN1 | hsa-miR-218-5p | down.mi | up.m |
| CLSTN1 | hsa-miR-30b-3p | down.mi | up.m |
| CLSTN1 | hsa-miR-30c-2-3p | down.mi | up.m |
| CLSTN1 | hsa-miR-423-5p | down.mi | up.m |
| CLTC | hsa-miR-1-3p | down.mi | up.m |
| CLTC | hsa-let-7e-5p | down.mi | up.m |
| CLTC | hsa-miR-195-3p | down.mi | up.m |
| CMBL | hsa-miR-378a-5p | down.mi | up.m |
| CMSS1 | hsa-miR-486-5p | down.mi | up.m |
| CNP | hsa-miR-30a-5p | down.mi | up.m |
| COA1 | hsa-miR-218-5p | down.mi | up.m |
| COASY | hsa-miR-144-3p | down.mi | up.m |
| COIL | hsa-let-7c-5p | down.mi | up.m |
| COIL | hsa-let-7a-5p | down.mi | up.m |
| COIL | hsa-miR-1-3p | down.mi | up.m |
| COIL | hsa-let-7g-5p | down.mi | up.m |
| COIL | hsa-let-7f-5p | down.mi | up.m |
| COIL | hsa-let-7e-5p | down.mi | up.m |
| COIL | hsa-let-7b-5p | down.mi | up.m |
| COIL | hsa-miR-15b-5p | down.mi | up.m |
| COL18A1 | hsa-miR-486-3p | down.mi | up.m |
| COL1A1 | hsa-miR-143-3p | down.mi | up.m |
| COL1A1 | hsa-miR-133a-3p | down.mi | up.m |
| COL1A2 | hsa-let-7g-5p | down.mi | up.m |
| COL3A1 | hsa-let-7b-5p | down.mi | up.m |
| COL3A1 | hsa-miR-143-3p | down.mi | up.m |
| COL5A1 | hsa-miR-30b-3p | down.mi | up.m |
| COL5A1 | hsa-miR-143-3p | down.mi | up.m |
| COL5A2 | hsa-miR-143-3p | down.mi | up.m |
| COL9A2 | hsa-miR-490-3p | down.mi | up.m |
| COPB2 | hsa-let-7b-3p | down.mi | up.m |
| COPG1 | hsa-let-7e-5p | down.mi | up.m |
| COPG1 | hsa-miR-1-3p | down.mi | up.m |
| COPG1 | hsa-let-7b-5p | down.mi | up.m |
| COPS6 | hsa-let-7c-5p | down.mi | up.m |
| COPS6 | hsa-let-7f-5p | down.mi | up.m |
| COPS7B | hsa-miR-605-5p | down.mi | up.m |
| COPS7B | hsa-miR-30a-5p | down.mi | up.m |
| COX6A1 | hsa-miR-30c-2-3p | down.mi | up.m |
| COX6B1 | hsa-let-7a-5p | down.mi | up.m |
| COX6B1 | hsa-let-7g-5p | down.mi | up.m |
| COX6B1 | hsa-let-7c-5p | down.mi | up.m |
| COX6B1 | hsa-miR-30b-3p | down.mi | up.m |
| COX6B1 | hsa-let-7b-5p | down.mi | up.m |
| COX6B1 | hsa-let-7e-5p | down.mi | up.m |
| COX6B1 | hsa-let-7f-5p | down.mi | up.m |
| COX6B1 | hsa-miR-30c-2-3p | down.mi | up.m |
| COX6B1 | hsa-miR-143-3p | down.mi | up.m |
| CPNE1 | hsa-miR-378a-3p | down.mi | up.m |
| CPNE1 | hsa-miR-195-5p | down.mi | up.m |
| CPNE1 | hsa-miR-15b-5p | down.mi | up.m |
| CPNE5 | hsa-miR-7704 | down.mi | up.m |
| CPOX | hsa-miR-218-5p | down.mi | up.m |
| CPOX | hsa-miR-15b-5p | down.mi | up.m |
| CPOX | hsa-miR-1-3p | down.mi | up.m |
| CPOX | hsa-miR-30a-5p | down.mi | up.m |
| CPS1 | hsa-miR-144-3p | down.mi | up.m |
| CPSF1 | hsa-miR-218-5p | down.mi | up.m |
| CPSF1 | hsa-miR-30a-5p | down.mi | up.m |
| CPSF1 | hsa-miR-1-3p | down.mi | up.m |
| CPSF1 | hsa-let-7b-5p | down.mi | up.m |
| CPSF3 | hsa-miR-30a-5p | down.mi | up.m |
| CPSF3 | hsa-miR-1-3p | down.mi | up.m |
| CPSF4 | hsa-miR-30a-5p | down.mi | up.m |
| CPSF6 | hsa-miR-605-5p | down.mi | up.m |
| CPTP | hsa-let-7b-5p | down.mi | up.m |
| CRABP2 | hsa-miR-486-3p | down.mi | up.m |
| CREB3L2 | hsa-miR-1-3p | down.mi | up.m |
| CREB3L2 | hsa-miR-374a-5p | down.mi | up.m |
| CREB3L2 | hsa-miR-206 | down.mi | up.m |
| CREBZF | hsa-miR-340-5p | down.mi | up.m |
| CREBZF | hsa-let-7b-3p | down.mi | up.m |
| CREBZF | hsa-miR-221-5p | down.mi | up.m |
| CREBZF | hsa-miR-490-3p | down.mi | up.m |
| CREBZF | hsa-miR-150-3p | down.mi | up.m |
| CRELD2 | hsa-miR-1-3p | down.mi | up.m |
| CRIPT | hsa-miR-30b-3p | down.mi | up.m |
| CRIPT | hsa-miR-486-3p | down.mi | up.m |
| CRIPT | hsa-miR-486-5p | down.mi | up.m |
| CRIPT | hsa-miR-30c-2-3p | down.mi | up.m |
| CRIPT | hsa-miR-1247-3p | down.mi | up.m |
| CSE1L | hsa-miR-3614-5p | down.mi | up.m |
| CSTF2 | hsa-let-7b-3p | down.mi | up.m |
| CSTF3 | hsa-miR-1-3p | down.mi | up.m |
| CSTF3 | hsa-miR-378a-3p | down.mi | up.m |
| CTHRC1 | hsa-let-7b-5p | down.mi | up.m |
| CTPS1 | hsa-let-7b-5p | down.mi | up.m |
| CTPS1 | hsa-let-7f-5p | down.mi | up.m |
| CTPS1 | hsa-let-7e-5p | down.mi | up.m |
| CTPS1 | hsa-let-7c-5p | down.mi | up.m |
| CTPS1 | hsa-let-7g-5p | down.mi | up.m |
| CTPS1 | hsa-let-7a-5p | down.mi | up.m |
| CTPS2 | hsa-miR-6892-5p | down.mi | up.m |
| CTSA | hsa-let-7e-5p | down.mi | up.m |
| CTSA | hsa-miR-486-3p | down.mi | up.m |
| CTSA | hsa-miR-221-5p | down.mi | up.m |
| CTSV | hsa-miR-1247-3p | down.mi | up.m |
| CTTN | hsa-miR-338-5p | down.mi | up.m |
| CTTN | hsa-miR-1-3p | down.mi | up.m |
| CTTN | hsa-miR-340-5p | down.mi | up.m |
| CYCS | hsa-miR-34b-3p | down.mi | up.m |
| CYCS | hsa-miR-378a-5p | down.mi | up.m |
| CYCS | hsa-miR-3614-5p | down.mi | up.m |
| CYP24A1 | hsa-miR-218-5p | down.mi | up.m |
| CYP27C1 | hsa-miR-4732-3p | down.mi | up.m |
| CYP2J2 | hsa-let-7b-5p | down.mi | up.m |
| CYP4F11 | hsa-miR-374a-5p | down.mi | up.m |
| DARS2 | hsa-miR-3614-5p | down.mi | up.m |
| DARS2 | hsa-miR-30a-5p | down.mi | up.m |
| DARS2 | hsa-miR-34b-3p | down.mi | up.m |
| DBF4 | hsa-miR-30a-5p | down.mi | up.m |
| DBF4 | hsa-miR-30d-5p | down.mi | up.m |
| DBN1 | hsa-miR-517a-3p | down.mi | up.m |
| DBN1 | hsa-miR-1-3p | down.mi | up.m |
| DBN1 | hsa-miR-517b-3p | down.mi | up.m |
| DCPS | hsa-miR-3614-5p | down.mi | up.m |
| DCPS | hsa-miR-34b-3p | down.mi | up.m |
| DCTPP1 | hsa-miR-378a-3p | down.mi | up.m |
| DCTPP1 | hsa-miR-1-3p | down.mi | up.m |
| DCTPP1 | hsa-let-7b-5p | down.mi | up.m |
| DCTPP1 | hsa-miR-378c | down.mi | up.m |
| DCUN1D5 | hsa-miR-3614-5p | down.mi | up.m |
| DDR1 | hsa-let-7g-5p | down.mi | up.m |
| DDX39A | hsa-miR-378a-3p | down.mi | up.m |
| DDX39B | hsa-miR-486-3p | down.mi | up.m |
| DDX41 | hsa-let-7b-5p | down.mi | up.m |
| DDX52 | hsa-miR-374a-5p | down.mi | up.m |
| DDX54 | hsa-miR-423-5p | down.mi | up.m |
| DDX55 | hsa-miR-374a-5p | down.mi | up.m |
| DENR | hsa-let-7b-5p | down.mi | up.m |
| DENR | hsa-miR-15b-5p | down.mi | up.m |
| DENR | hsa-miR-204-5p | down.mi | up.m |
| DEPDC1 | hsa-miR-340-5p | down.mi | up.m |
| DHFR | hsa-miR-15b-5p | down.mi | up.m |
| DHTKD1 | hsa-let-7b-5p | down.mi | up.m |
| DHTKD1 | hsa-miR-378a-5p | down.mi | up.m |
| DHX37 | hsa-miR-15b-5p | down.mi | up.m |
| DIRAS1 | hsa-miR-27a-5p | down.mi | up.m |
| DNA2 | hsa-let-7b-5p | down.mi | up.m |
| DNA2 | hsa-let-7c-5p | down.mi | up.m |
| DNA2 | hsa-let-7f-5p | down.mi | up.m |
| DNA2 | hsa-let-7a-5p | down.mi | up.m |
| DNA2 | hsa-let-7e-5p | down.mi | up.m |
| DNA2 | hsa-let-7g-5p | down.mi | up.m |
| DNAAF5 | hsa-miR-30a-5p | down.mi | up.m |
| DNAAF5 | hsa-miR-1-3p | down.mi | up.m |
| DNAAF5 | hsa-let-7b-5p | down.mi | up.m |
| DNAJA3 | hsa-miR-99a-5p | down.mi | up.m |
| DNAJC10 | hsa-miR-195-5p | down.mi | up.m |
| DNAJC10 | hsa-miR-30c-2-3p | down.mi | up.m |
| DNAJC10 | hsa-miR-1-3p | down.mi | up.m |
| DNAJC10 | hsa-miR-145-3p | down.mi | up.m |
| DNAJC10 | hsa-miR-30b-3p | down.mi | up.m |
| DNAJC10 | hsa-miR-15b-5p | down.mi | up.m |
| DNAJC10 | hsa-miR-221-5p | down.mi | up.m |
| DNAJC2 | hsa-miR-218-5p | down.mi | up.m |
| DNAJC2 | hsa-miR-30a-5p | down.mi | up.m |
| DNAJC9 | hsa-miR-140-3p | down.mi | up.m |
| DNAJC9 | hsa-miR-15b-5p | down.mi | up.m |
| DNAJC9 | hsa-miR-195-5p | down.mi | up.m |
| DNMT1 | hsa-miR-30a-5p | down.mi | up.m |
| DNMT1 | hsa-let-7c-5p | down.mi | up.m |
| DNMT1 | hsa-miR-218-5p | down.mi | up.m |
| DNMT3A | hsa-miR-143-3p | down.mi | up.m |
| DNMT3A | hsa-miR-340-5p | down.mi | up.m |
| DOLPP1 | hsa-miR-1-3p | down.mi | up.m |
| DONSON | hsa-miR-340-5p | down.mi | up.m |
| DPH2 | hsa-miR-30b-3p | down.mi | up.m |
| DPM2 | hsa-miR-218-5p | down.mi | up.m |
| DPM2 | hsa-miR-30b-3p | down.mi | up.m |
| DPM2 | hsa-miR-30c-2-3p | down.mi | up.m |
| DPY19L1 | hsa-miR-30a-5p | down.mi | up.m |
| DPY19L1 | hsa-miR-1-3p | down.mi | up.m |
| DPY19L1 | hsa-miR-374a-5p | down.mi | up.m |
| DPY30 | hsa-miR-1-3p | down.mi | up.m |
| DRAP1 | hsa-miR-423-5p | down.mi | up.m |
| DRAP1 | hsa-miR-1-3p | down.mi | up.m |
| DROSHA | hsa-miR-195-3p | down.mi | up.m |
| DROSHA | hsa-miR-1-3p | down.mi | up.m |
| DROSHA | hsa-miR-30d-5p | down.mi | up.m |
| DROSHA | hsa-miR-30a-5p | down.mi | up.m |
| DSG2 | hsa-let-7b-3p | down.mi | up.m |
| DSG2 | hsa-miR-15b-5p | down.mi | up.m |
| DSG2 | hsa-miR-1-3p | down.mi | up.m |
| DSG2 | hsa-let-7b-5p | down.mi | up.m |
| DSG2 | hsa-miR-30a-5p | down.mi | up.m |
| DSN1 | hsa-miR-30c-2-3p | down.mi | up.m |
| DSN1 | hsa-miR-340-5p | down.mi | up.m |
| DSN1 | hsa-miR-1247-3p | down.mi | up.m |
| DSN1 | hsa-miR-374a-5p | down.mi | up.m |
| DSN1 | hsa-miR-605-5p | down.mi | up.m |
| DSN1 | hsa-miR-30b-3p | down.mi | up.m |
| DSP | hsa-let-7b-5p | down.mi | up.m |
| DSP | hsa-let-7e-5p | down.mi | up.m |
| DTL | hsa-miR-30a-5p | down.mi | up.m |
| DUSP12 | hsa-let-7b-5p | down.mi | up.m |
| DUSP23 | hsa-let-7b-5p | down.mi | up.m |
| DUSP4 | hsa-miR-30b-3p | down.mi | up.m |
| DVL1 | hsa-miR-378a-3p | down.mi | up.m |
| DVL3 | hsa-let-7b-5p | down.mi | up.m |
| DVL3 | hsa-let-7g-5p | down.mi | up.m |
| DVL3 | hsa-miR-204-5p | down.mi | up.m |
| DVL3 | hsa-let-7f-5p | down.mi | up.m |
| DVL3 | hsa-let-7e-5p | down.mi | up.m |
| DVL3 | hsa-miR-1247-5p | down.mi | up.m |
| DVL3 | hsa-let-7c-5p | down.mi | up.m |
| DVL3 | hsa-let-7a-5p | down.mi | up.m |
| DYRK2 | hsa-miR-378a-3p | down.mi | up.m |
| DYRK2 | hsa-miR-30c-2-3p | down.mi | up.m |
| DYRK2 | hsa-miR-4732-3p | down.mi | up.m |
| DYRK2 | hsa-let-7f-5p | down.mi | up.m |
| DYRK2 | hsa-miR-30b-3p | down.mi | up.m |
| DYRK2 | hsa-miR-195-3p | down.mi | up.m |
| DYRK2 | hsa-let-7e-5p | down.mi | up.m |
| E2F1 | hsa-miR-223-3p | down.mi | up.m |
| E2F1 | hsa-let-7a-5p | down.mi | up.m |
| E2F2 | hsa-miR-218-5p | down.mi | up.m |
| E2F2 | hsa-let-7b-5p | down.mi | up.m |
| E2F2 | hsa-let-7a-5p | down.mi | up.m |
| E2F3 | hsa-miR-2110 | down.mi | up.m |
| E2F3 | hsa-miR-195-5p | down.mi | up.m |
| E2F3 | hsa-let-7b-5p | down.mi | up.m |
| E2F3 | hsa-miR-15b-5p | down.mi | up.m |
| E2F3 | hsa-miR-4732-3p | down.mi | up.m |
| E2F3 | hsa-miR-139-3p | down.mi | up.m |
| E2F3 | hsa-miR-423-5p | down.mi | up.m |
| E2F5 | hsa-let-7b-5p | down.mi | up.m |
| E2F5 | hsa-miR-378a-3p | down.mi | up.m |
| E2F5 | hsa-miR-584-5p | down.mi | up.m |
| E2F5 | hsa-miR-1-3p | down.mi | up.m |
| E2F5 | hsa-let-7c-5p | down.mi | up.m |
| E2F7 | hsa-miR-15b-5p | down.mi | up.m |
| E2F7 | hsa-miR-195-5p | down.mi | up.m |
| E2F7 | hsa-let-7b-5p | down.mi | up.m |
| E2F8 | hsa-let-7b-3p | down.mi | up.m |
| E2F8 | hsa-miR-6892-5p | down.mi | up.m |
| E2F8 | hsa-miR-1258 | down.mi | up.m |
| E4F1 | hsa-miR-99a-5p | down.mi | up.m |
| EARS2 | hsa-miR-218-5p | down.mi | up.m |
| EBNA1BP2 | hsa-miR-30c-2-3p | down.mi | up.m |
| EBNA1BP2 | hsa-miR-34b-3p | down.mi | up.m |
| EBNA1BP2 | hsa-miR-490-3p | down.mi | up.m |
| EBNA1BP2 | hsa-miR-30b-3p | down.mi | up.m |
| EBNA1BP2 | hsa-miR-3614-5p | down.mi | up.m |
| EBNA1BP2 | hsa-miR-218-1-3p | down.mi | up.m |
| ECHS1 | hsa-miR-1-3p | down.mi | up.m |
| ECHS1 | hsa-miR-15b-5p | down.mi | up.m |
| ECM1 | hsa-miR-486-3p | down.mi | up.m |
| ECT2 | hsa-miR-223-3p | down.mi | up.m |
| EED | hsa-miR-30d-5p | down.mi | up.m |
| EED | hsa-miR-138-5p | down.mi | up.m |
| EED | hsa-miR-30a-5p | down.mi | up.m |
| EEF1D | hsa-miR-218-5p | down.mi | up.m |
| EEF1E1 | hsa-let-7b-5p | down.mi | up.m |
| EFTUD2 | hsa-miR-195-5p | down.mi | up.m |
| EFTUD2 | hsa-miR-1-3p | down.mi | up.m |
| EFTUD2 | hsa-miR-15b-5p | down.mi | up.m |
| EFTUD2 | hsa-miR-218-5p | down.mi | up.m |
| EHMT2 | hsa-miR-1-3p | down.mi | up.m |
| EIF2AK1 | hsa-let-7b-5p | down.mi | up.m |
| EIF2B4 | hsa-miR-30a-5p | down.mi | up.m |
| EIF2S3 | hsa-miR-218-5p | down.mi | up.m |
| EIF2S3 | hsa-miR-486-3p | down.mi | up.m |
| EIF2S3 | hsa-miR-374a-5p | down.mi | up.m |
| EIF2S3 | hsa-miR-144-3p | down.mi | up.m |
| EIF4A3 | hsa-let-7g-5p | down.mi | up.m |
| EIF4A3 | hsa-let-7a-5p | down.mi | up.m |
| EIF4A3 | hsa-let-7f-5p | down.mi | up.m |
| EIF4A3 | hsa-let-7e-5p | down.mi | up.m |
| EIF4A3 | hsa-miR-30b-3p | down.mi | up.m |
| EIF4A3 | hsa-let-7b-5p | down.mi | up.m |
| EIF4A3 | hsa-let-7c-5p | down.mi | up.m |
| EIF4EBP1 | hsa-miR-138-5p | down.mi | up.m |
| EIF4G3 | hsa-miR-218-5p | down.mi | up.m |
| EIF5A | hsa-miR-99a-5p | down.mi | up.m |
| ELOVL6 | hsa-miR-204-5p | down.mi | up.m |
| ELOVL6 | hsa-miR-218-5p | down.mi | up.m |
| EMB | hsa-miR-218-5p | down.mi | up.m |
| ENAH | hsa-miR-378a-5p | down.mi | up.m |
| ENAH | hsa-miR-204-5p | down.mi | up.m |
| ENAH | hsa-miR-3154 | down.mi | up.m |
| ENO1 | hsa-miR-378a-3p | down.mi | up.m |
| ENO1 | hsa-miR-204-5p | down.mi | up.m |
| ENTPD4 | hsa-miR-378a-5p | down.mi | up.m |
| ENTPD4 | hsa-miR-30a-3p | down.mi | up.m |
| ENTPD4 | hsa-let-7b-5p | down.mi | up.m |
| ENTPD6 | hsa-let-7b-5p | down.mi | up.m |
| ENTPD6 | hsa-miR-195-5p | down.mi | up.m |
| ENTPD6 | hsa-miR-15b-5p | down.mi | up.m |
| ENTPD7 | hsa-miR-15b-5p | down.mi | up.m |
| ENTPD7 | hsa-miR-195-5p | down.mi | up.m |
| EPHB2 | hsa-miR-30b-3p | down.mi | up.m |
| EPHB2 | hsa-miR-218-5p | down.mi | up.m |
| EPRS | hsa-miR-140-3p | down.mi | up.m |
| ERBB2 | hsa-miR-486-3p | down.mi | up.m |
| ERBB2 | hsa-miR-133a-3p | down.mi | up.m |
| ERBB3 | hsa-miR-143-3p | down.mi | up.m |
| ERCC6L | hsa-miR-221-5p | down.mi | up.m |
| ERGIC2 | hsa-let-7b-5p | down.mi | up.m |
| ERGIC2 | hsa-miR-195-3p | down.mi | up.m |
| ERGIC2 | hsa-miR-338-5p | down.mi | up.m |
| ERGIC3 | hsa-miR-490-3p | down.mi | up.m |
| ERGIC3 | hsa-let-7b-5p | down.mi | up.m |
| ERH | hsa-miR-3154 | down.mi | up.m |
| ERH | hsa-miR-340-5p | down.mi | up.m |
| ERRFI1 | hsa-miR-140-3p | down.mi | up.m |
| ERRFI1 | hsa-miR-423-5p | down.mi | up.m |
| ERRFI1 | hsa-miR-125b-2-3p | down.mi | up.m |
| ERRFI1 | hsa-miR-378a-3p | down.mi | up.m |
| ESCO2 | hsa-miR-30b-3p | down.mi | up.m |
| ESCO2 | hsa-miR-30c-2-3p | down.mi | up.m |
| ESCO2 | hsa-miR-1247-3p | down.mi | up.m |
| ESPL1 | hsa-let-7e-5p | down.mi | up.m |
| ESPL1 | hsa-let-7f-5p | down.mi | up.m |
| ESPL1 | hsa-let-7b-5p | down.mi | up.m |
| ESPL1 | hsa-let-7g-5p | down.mi | up.m |
| ESPL1 | hsa-let-7c-5p | down.mi | up.m |
| ESPL1 | hsa-let-7a-5p | down.mi | up.m |
| ETNK2 | hsa-let-7c-5p | down.mi | up.m |
| ETV6 | hsa-miR-30a-3p | down.mi | up.m |
| ETV7 | hsa-miR-1-3p | down.mi | up.m |
| EXOSC2 | hsa-miR-206 | down.mi | up.m |
| EXOSC2 | hsa-miR-1-3p | down.mi | up.m |
| EXOSC2 | hsa-miR-30c-2-3p | down.mi | up.m |
| EXOSC2 | hsa-miR-30b-3p | down.mi | up.m |
| EXOSC2 | hsa-miR-30a-3p | down.mi | up.m |
| EYA2 | hsa-miR-30a-5p | down.mi | up.m |
| EZH2 | hsa-miR-144-3p | down.mi | up.m |
| EZH2 | hsa-let-7c-5p | down.mi | up.m |
| EZH2 | hsa-miR-138-5p | down.mi | up.m |
| EZH2 | hsa-let-7b-5p | down.mi | up.m |
| EZH2 | hsa-miR-30d-5p | down.mi | up.m |
| EZH2 | hsa-let-7a-5p | down.mi | up.m |
| EZH2 | hsa-let-7e-5p | down.mi | up.m |
| F2RL1 | hsa-miR-1-3p | down.mi | up.m |
| FADD | hsa-miR-15b-5p | down.mi | up.m |
| FADD | hsa-miR-1-3p | down.mi | up.m |
| FAHD1 | hsa-miR-30a-5p | down.mi | up.m |
| FAHD1 | hsa-miR-490-3p | down.mi | up.m |
| FAHD1 | hsa-miR-30b-3p | down.mi | up.m |
| FAM102A | hsa-miR-1-3p | down.mi | up.m |
| FAM102A | hsa-miR-143-3p | down.mi | up.m |
| FAM122B | hsa-miR-144-3p | down.mi | up.m |
| FAM122B | hsa-miR-195-5p | down.mi | up.m |
| FAM122B | hsa-miR-15b-5p | down.mi | up.m |
| FAM136A | hsa-let-7b-5p | down.mi | up.m |
| FAM136A | hsa-miR-490-3p | down.mi | up.m |
| FAM162A | hsa-miR-139-5p | down.mi | up.m |
| FAM162A | hsa-miR-1247-3p | down.mi | up.m |
| FAM162A | hsa-miR-139-3p | down.mi | up.m |
| FAM171A2 | hsa-let-7a-5p | down.mi | up.m |
| FAM189B | hsa-miR-423-5p | down.mi | up.m |
| FAM208B | hsa-miR-340-5p | down.mi | up.m |
| FAM213A | hsa-let-7b-5p | down.mi | up.m |
| FAM222B | hsa-let-7e-5p | down.mi | up.m |
| FAM222B | hsa-let-7f-5p | down.mi | up.m |
| FAM222B | hsa-let-7b-5p | down.mi | up.m |
| FAM222B | hsa-let-7c-5p | down.mi | up.m |
| FAM222B | hsa-let-7g-5p | down.mi | up.m |
| FAM222B | hsa-let-7a-5p | down.mi | up.m |
| FAM57A | hsa-miR-1-3p | down.mi | up.m |
| FAM57A | hsa-let-7b-5p | down.mi | up.m |
| FAM69A | hsa-miR-144-3p | down.mi | up.m |
| FAM83A | hsa-miR-486-3p | down.mi | up.m |
| FAM83H | hsa-miR-423-5p | down.mi | up.m |
| FAM83H | hsa-miR-139-3p | down.mi | up.m |
| FAM83H | hsa-miR-3154 | down.mi | up.m |
| FAM98A | hsa-miR-423-5p | down.mi | up.m |
| FANCA | hsa-miR-140-3p | down.mi | up.m |
| FANCA | hsa-miR-1247-3p | down.mi | up.m |
| FANCD2 | hsa-let-7b-5p | down.mi | up.m |
| FANCD2 | hsa-miR-34c-3p | down.mi | up.m |
| FANCF | hsa-miR-30a-5p | down.mi | up.m |
| FANCF | hsa-miR-30d-5p | down.mi | up.m |
| FANCI | hsa-let-7c-5p | down.mi | up.m |
| FANCI | hsa-miR-1-3p | down.mi | up.m |
| FARSA | hsa-let-7a-5p | down.mi | up.m |
| FAT1 | hsa-miR-218-5p | down.mi | up.m |
| FBLIM1 | hsa-miR-378a-5p | down.mi | up.m |
| FBXL19 | hsa-miR-3154 | down.mi | up.m |
| FBXO41 | hsa-miR-218-5p | down.mi | up.m |
| FBXO45 | hsa-miR-30b-3p | down.mi | up.m |
| FBXO45 | hsa-miR-30a-5p | down.mi | up.m |
| FBXO45 | hsa-miR-30d-5p | down.mi | up.m |
| FBXO45 | hsa-miR-30c-2-3p | down.mi | up.m |
| FBXO45 | hsa-miR-1-3p | down.mi | up.m |
| FBXO45 | hsa-miR-125b-2-3p | down.mi | up.m |
| FEN1 | hsa-let-7b-5p | down.mi | up.m |
| FEN1 | hsa-miR-378a-5p | down.mi | up.m |
| FGB | hsa-miR-144-3p | down.mi | up.m |
| FGD6 | hsa-miR-143-3p | down.mi | up.m |
| FGFRL1 | hsa-let-7b-5p | down.mi | up.m |
| FHL2 | hsa-miR-34c-3p | down.mi | up.m |
| FKBP10 | hsa-let-7c-5p | down.mi | up.m |
| FKBP10 | hsa-miR-218-5p | down.mi | up.m |
| FKBP14 | hsa-miR-144-3p | down.mi | up.m |
| FKBP4 | hsa-miR-423-5p | down.mi | up.m |
| FLAD1 | hsa-let-7b-5p | down.mi | up.m |
| FLVCR1 | hsa-miR-378a-5p | down.mi | up.m |
| FOXA1 | hsa-let-7a-5p | down.mi | up.m |
| FOXA1 | hsa-miR-30d-5p | down.mi | up.m |
| FOXA1 | hsa-miR-584-5p | down.mi | up.m |
| FOXA1 | hsa-miR-30a-5p | down.mi | up.m |
| FOXM1 | hsa-miR-204-5p | down.mi | up.m |
| FOXRED1 | hsa-let-7b-5p | down.mi | up.m |
| FOXRED2 | hsa-miR-378a-3p | down.mi | up.m |
| FOXRED2 | hsa-miR-99a-5p | down.mi | up.m |
| FOXRED2 | hsa-let-7b-5p | down.mi | up.m |
| FRK | hsa-miR-2110 | down.mi | up.m |
| FRK | hsa-miR-30c-2-3p | down.mi | up.m |
| FRK | hsa-miR-6892-5p | down.mi | up.m |
| FRK | hsa-miR-4529-3p | down.mi | up.m |
| FRK | hsa-miR-195-3p | down.mi | up.m |
| FSCN1 | hsa-miR-133a-3p | down.mi | up.m |
| FSCN1 | hsa-miR-1247-3p | down.mi | up.m |
| FSCN1 | hsa-miR-30a-5p | down.mi | up.m |
| FSCN1 | hsa-miR-143-3p | down.mi | up.m |
| FSCN1 | hsa-miR-133b | down.mi | up.m |
| FSCN1 | hsa-miR-184 | down.mi | up.m |
| FSTL4 | hsa-miR-204-5p | down.mi | up.m |
| FTSJ1 | hsa-miR-1-3p | down.mi | up.m |
| FTSJ3 | hsa-miR-27a-5p | down.mi | up.m |
| FUBP1 | hsa-miR-218-5p | down.mi | up.m |
| FUBP1 | hsa-miR-1-3p | down.mi | up.m |
| FURIN | hsa-miR-30b-3p | down.mi | up.m |
| FURIN | hsa-miR-195-5p | down.mi | up.m |
| FURIN | hsa-miR-140-3p | down.mi | up.m |
| FURIN | hsa-miR-15b-5p | down.mi | up.m |
| FUT2 | hsa-miR-15b-5p | down.mi | up.m |
| FUT2 | hsa-miR-30c-2-3p | down.mi | up.m |
| FUT2 | hsa-miR-30b-3p | down.mi | up.m |
| G6PD | hsa-miR-1-3p | down.mi | up.m |
| G6PD | hsa-miR-206 | down.mi | up.m |
| GALNT2 | hsa-let-7b-5p | down.mi | up.m |
| GALNT3 | hsa-miR-340-5p | down.mi | up.m |
| GALNT6 | hsa-miR-143-3p | down.mi | up.m |
| GALNT6 | hsa-miR-378a-5p | down.mi | up.m |
| GALNT7 | hsa-miR-34b-3p | down.mi | up.m |
| GALNT7 | hsa-miR-30a-5p | down.mi | up.m |
| GALNT7 | hsa-miR-378a-3p | down.mi | up.m |
| GALNT7 | hsa-miR-374a-5p | down.mi | up.m |
| GALNT7 | hsa-miR-125b-2-3p | down.mi | up.m |
| GAPDH | hsa-let-7b-5p | down.mi | up.m |
| GAPDH | hsa-miR-423-5p | down.mi | up.m |
| GAPDH | hsa-miR-218-5p | down.mi | up.m |
| GARS | hsa-let-7c-5p | down.mi | up.m |
| GCLC | hsa-miR-30a-5p | down.mi | up.m |
| GCLC | hsa-miR-30d-5p | down.mi | up.m |
| GCNT3 | hsa-miR-490-3p | down.mi | up.m |
| GCNT3 | hsa-miR-140-3p | down.mi | up.m |
| GCNT3 | hsa-miR-30b-3p | down.mi | up.m |
| GDF15 | hsa-miR-140-3p | down.mi | up.m |
| GEMIN6 | hsa-miR-1247-3p | down.mi | up.m |
| GEMIN7 | hsa-let-7b-5p | down.mi | up.m |
| GEN1 | hsa-miR-490-3p | down.mi | up.m |
| GEN1 | hsa-miR-605-5p | down.mi | up.m |
| GEN1 | hsa-miR-30b-3p | down.mi | up.m |
| GFER | hsa-miR-486-3p | down.mi | up.m |
| GFPT1 | hsa-miR-34b-3p | down.mi | up.m |
| GFPT1 | hsa-miR-140-3p | down.mi | up.m |
| GFPT1 | hsa-miR-218-5p | down.mi | up.m |
| GGCT | hsa-miR-30a-5p | down.mi | up.m |
| GGCT | hsa-let-7b-5p | down.mi | up.m |
| GGCX | hsa-miR-30c-2-3p | down.mi | up.m |
| GGCX | hsa-miR-145-3p | down.mi | up.m |
| GGCX | hsa-miR-3154 | down.mi | up.m |
| GGCX | hsa-miR-1247-3p | down.mi | up.m |
| GGCX | hsa-miR-30b-3p | down.mi | up.m |
| GID8 | hsa-miR-340-5p | down.mi | up.m |
| GJB1 | hsa-miR-423-5p | down.mi | up.m |
| GJB3 | hsa-miR-1-3p | down.mi | up.m |
| GLMP | hsa-miR-140-3p | down.mi | up.m |
| GLRX3 | hsa-miR-30a-5p | down.mi | up.m |
| GMDS | hsa-miR-218-5p | down.mi | up.m |
| GMPS | hsa-let-7e-5p | down.mi | up.m |
| GNG4 | hsa-miR-30c-2-3p | down.mi | up.m |
| GNG4 | hsa-miR-30b-3p | down.mi | up.m |
| GNL3 | hsa-miR-374a-5p | down.mi | up.m |
| GNPNAT1 | hsa-miR-1-3p | down.mi | up.m |
| GOLGA3 | hsa-miR-490-3p | down.mi | up.m |
| GOLGA3 | hsa-miR-30b-3p | down.mi | up.m |
| GOLGA3 | hsa-miR-30c-2-3p | down.mi | up.m |
| GOLGA3 | hsa-miR-99a-5p | down.mi | up.m |
| GOLGA3 | hsa-miR-218-5p | down.mi | up.m |
| GOLPH3L | hsa-miR-218-5p | down.mi | up.m |
| GOLT1B | hsa-miR-338-5p | down.mi | up.m |
| GORASP2 | hsa-let-7c-5p | down.mi | up.m |
| GORASP2 | hsa-let-7a-5p | down.mi | up.m |
| GPAA1 | hsa-miR-1-3p | down.mi | up.m |
| GPATCH4 | hsa-let-7b-5p | down.mi | up.m |
| GPI | hsa-let-7b-5p | down.mi | up.m |
| GPI | hsa-miR-378a-5p | down.mi | up.m |
| GPN1 | hsa-let-7a-5p | down.mi | up.m |
| GPRIN1 | hsa-miR-184 | down.mi | up.m |
| GPRIN1 | hsa-miR-423-5p | down.mi | up.m |
| GPS1 | hsa-let-7c-5p | down.mi | up.m |
| GPS1 | hsa-let-7f-5p | down.mi | up.m |
| GPSM2 | hsa-miR-378a-5p | down.mi | up.m |
| GPX8 | hsa-miR-340-5p | down.mi | up.m |
| GREB1 | hsa-miR-490-3p | down.mi | up.m |
| GREB1 | hsa-miR-125b-2-3p | down.mi | up.m |
| GREB1 | hsa-miR-340-5p | down.mi | up.m |
| GREM1 | hsa-miR-27a-5p | down.mi | up.m |
| GREM1 | hsa-let-7b-3p | down.mi | up.m |
| GRHL1 | hsa-miR-99a-5p | down.mi | up.m |
| GSKIP | hsa-miR-374a-5p | down.mi | up.m |
| GSR | hsa-miR-4732-3p | down.mi | up.m |
| GSR | hsa-let-7b-5p | down.mi | up.m |
| GTF2E2 | hsa-miR-30a-5p | down.mi | up.m |
| GTF2E2 | hsa-miR-30d-5p | down.mi | up.m |
| GTF3C6 | hsa-miR-1247-3p | down.mi | up.m |
| GTF3C6 | hsa-miR-1-3p | down.mi | up.m |
| GTF3C6 | hsa-miR-221-5p | down.mi | up.m |
| GTPBP2 | hsa-miR-2110 | down.mi | up.m |
| GTPBP3 | hsa-let-7b-5p | down.mi | up.m |
| GTPBP4 | hsa-miR-2110 | down.mi | up.m |
| GUF1 | hsa-miR-34c-3p | down.mi | up.m |
| GUF1 | hsa-miR-34b-3p | down.mi | up.m |
| H1F0 | hsa-let-7a-5p | down.mi | up.m |
| H2AFX | hsa-miR-218-5p | down.mi | up.m |
| H2AFX | hsa-miR-138-5p | down.mi | up.m |
| HAUS5 | hsa-miR-3614-5p | down.mi | up.m |
| HAUS5 | hsa-miR-221-5p | down.mi | up.m |
| HAVCR1 | hsa-miR-490-3p | down.mi | up.m |
| HAX1 | hsa-miR-223-3p | down.mi | up.m |
| HCAR1 | hsa-miR-1247-3p | down.mi | up.m |
| HCAR1 | hsa-miR-378a-5p | down.mi | up.m |
| HDAC1 | hsa-miR-30a-5p | down.mi | up.m |
| HDGF | hsa-miR-195-5p | down.mi | up.m |
| HDGF | hsa-miR-15b-5p | down.mi | up.m |
| HDGF | hsa-miR-423-5p | down.mi | up.m |
| HDGF | hsa-miR-218-5p | down.mi | up.m |
| HDGF | hsa-miR-30a-5p | down.mi | up.m |
| HDGF | hsa-miR-30c-2-3p | down.mi | up.m |
| HDHD3 | hsa-miR-30d-5p | down.mi | up.m |
| HEATR1 | hsa-miR-218-5p | down.mi | up.m |
| HELLS | hsa-let-7b-5p | down.mi | up.m |
| HES1 | hsa-let-7b-5p | down.mi | up.m |
| HES4 | hsa-miR-423-5p | down.mi | up.m |
| HES6 | hsa-miR-30b-3p | down.mi | up.m |
| HGH1 | hsa-let-7b-5p | down.mi | up.m |
| HGS | hsa-let-7b-5p | down.mi | up.m |
| HID1 | hsa-miR-4732-3p | down.mi | up.m |
| HIF1A | hsa-let-7b-5p | down.mi | up.m |
| HIF1A | hsa-miR-138-5p | down.mi | up.m |
| HILPDA | hsa-miR-378a-5p | down.mi | up.m |
| HIST1H1C | hsa-miR-423-5p | down.mi | up.m |
| HIST1H1C | hsa-let-7b-5p | down.mi | up.m |
| HIST1H1D | hsa-let-7a-5p | down.mi | up.m |
| HIST1H1E | hsa-miR-423-5p | down.mi | up.m |
| HIST1H1E | hsa-miR-27a-5p | down.mi | up.m |
| HIST1H2AG | hsa-miR-1247-3p | down.mi | up.m |
| HIST1H2AG | hsa-miR-4529-3p | down.mi | up.m |
| HIST1H2AI | hsa-miR-218-1-3p | down.mi | up.m |
| HIST1H2BD | hsa-miR-378a-3p | down.mi | up.m |
| HIST1H2BD | hsa-let-7a-5p | down.mi | up.m |
| HIST1H2BD | hsa-let-7e-5p | down.mi | up.m |
| HIST1H2BD | hsa-miR-30b-3p | down.mi | up.m |
| HIST1H2BD | hsa-let-7g-5p | down.mi | up.m |
| HIST1H2BD | hsa-let-7f-5p | down.mi | up.m |
| HIST1H2BD | hsa-miR-490-3p | down.mi | up.m |
| HIST1H2BD | hsa-let-7c-5p | down.mi | up.m |
| HIST1H2BD | hsa-let-7b-5p | down.mi | up.m |
| HIST1H2BG | hsa-miR-143-3p | down.mi | up.m |
| HIST1H2BJ | hsa-miR-138-5p | down.mi | up.m |
| HIST1H2BK | hsa-let-7c-5p | down.mi | up.m |
| HIST1H2BK | hsa-let-7b-5p | down.mi | up.m |
| HIST1H2BK | hsa-let-7g-5p | down.mi | up.m |
| HIST1H2BK | hsa-let-7f-5p | down.mi | up.m |
| HIST1H2BK | hsa-miR-218-5p | down.mi | up.m |
| HIST1H2BK | hsa-let-7a-5p | down.mi | up.m |
| HIST1H2BK | hsa-let-7e-5p | down.mi | up.m |
| HIST1H2BK | hsa-miR-423-5p | down.mi | up.m |
| HIST1H2BK | hsa-miR-584-5p | down.mi | up.m |
| HIST1H2BK | hsa-miR-138-5p | down.mi | up.m |
| HIST1H3H | hsa-miR-378a-3p | down.mi | up.m |
| HIST1H4E | hsa-miR-598-3p | down.mi | up.m |
| HIST2H2AC | hsa-miR-133b | down.mi | up.m |
| HIST2H2AC | hsa-miR-1-3p | down.mi | up.m |
| HIST2H2AC | hsa-miR-133a-3p | down.mi | up.m |
| HIST2H2BE | hsa-miR-423-5p | down.mi | up.m |
| HIST2H2BE | hsa-miR-15b-5p | down.mi | up.m |
| HIST2H2BE | hsa-let-7a-5p | down.mi | up.m |
| HIST2H2BE | hsa-miR-195-5p | down.mi | up.m |
| HIST2H2BF | hsa-let-7a-5p | down.mi | up.m |
| HIST2H2BF | hsa-miR-423-5p | down.mi | up.m |
| HIST2H2BF | hsa-let-7e-5p | down.mi | up.m |
| HJURP | hsa-miR-1247-3p | down.mi | up.m |
| HJURP | hsa-miR-6720-3p | down.mi | up.m |
| HJURP | hsa-miR-218-5p | down.mi | up.m |
| HM13 | hsa-miR-1247-3p | down.mi | up.m |
| HM13 | hsa-miR-378a-5p | down.mi | up.m |
| HMGA1 | hsa-miR-15b-5p | down.mi | up.m |
| HMGA1 | hsa-let-7f-5p | down.mi | up.m |
| HMGA1 | hsa-let-7e-5p | down.mi | up.m |
| HMGA1 | hsa-miR-138-5p | down.mi | up.m |
| HMGA1 | hsa-let-7a-5p | down.mi | up.m |
| HMGA1 | hsa-let-7g-5p | down.mi | up.m |
| HMGA1 | hsa-let-7b-5p | down.mi | up.m |
| HMGA1 | hsa-miR-195-5p | down.mi | up.m |
| HMGA1 | hsa-miR-30c-2-3p | down.mi | up.m |
| HMGA1 | hsa-let-7c-5p | down.mi | up.m |
| HMGA1 | hsa-miR-486-3p | down.mi | up.m |
| HMGA1 | hsa-miR-486-5p | down.mi | up.m |
| HMGA1 | hsa-miR-218-5p | down.mi | up.m |
| HMGA1 | hsa-miR-30b-3p | down.mi | up.m |
| HMGA2 | hsa-let-7a-5p | down.mi | up.m |
| HMGA2 | hsa-let-7c-5p | down.mi | up.m |
| HMGA2 | hsa-miR-490-3p | down.mi | up.m |
| HMGA2 | hsa-let-7f-5p | down.mi | up.m |
| HMGA2 | hsa-let-7g-5p | down.mi | up.m |
| HMGA2 | hsa-let-7b-5p | down.mi | up.m |
| HMGA2 | hsa-let-7e-5p | down.mi | up.m |
| HMGA2 | hsa-miR-204-5p | down.mi | up.m |
| HMGA2 | hsa-miR-30a-3p | down.mi | up.m |
| HMGB2 | hsa-miR-374a-5p | down.mi | up.m |
| HMGB2 | hsa-miR-139-5p | down.mi | up.m |
| HMGB2 | hsa-miR-218-5p | down.mi | up.m |
| HMGB2 | hsa-let-7b-3p | down.mi | up.m |
| HMGN1 | hsa-miR-140-3p | down.mi | up.m |
| HNF4G | hsa-miR-30a-5p | down.mi | up.m |
| HNRNPAB | hsa-miR-144-3p | down.mi | up.m |
| HNRNPC | hsa-let-7e-5p | down.mi | up.m |
| HNRNPC | hsa-miR-139-3p | down.mi | up.m |
| HOOK1 | hsa-miR-584-5p | down.mi | up.m |
| HOOK1 | hsa-miR-1-3p | down.mi | up.m |
| HOOK1 | hsa-miR-139-5p | down.mi | up.m |
| HOXA10 | hsa-miR-144-3p | down.mi | up.m |
| HOXA10 | hsa-miR-15b-5p | down.mi | up.m |
| HOXA10 | hsa-miR-340-5p | down.mi | up.m |
| HOXA10 | hsa-miR-195-3p | down.mi | up.m |
| HOXA10 | hsa-miR-338-5p | down.mi | up.m |
| HOXA10 | hsa-miR-195-5p | down.mi | up.m |
| HOXA10 | hsa-miR-204-5p | down.mi | up.m |
| HOXB2 | hsa-miR-605-5p | down.mi | up.m |
| HOXB3 | hsa-miR-218-5p | down.mi | up.m |
| HOXB8 | hsa-miR-3154 | down.mi | up.m |
| HS3ST1 | hsa-miR-144-5p | down.mi | up.m |
| HS3ST1 | hsa-miR-30a-3p | down.mi | up.m |
| HS6ST2 | hsa-let-7e-5p | down.mi | up.m |
| HS6ST2 | hsa-miR-218-5p | down.mi | up.m |
| HSD3B7 | hsa-miR-1-3p | down.mi | up.m |
| HSF1 | hsa-let-7c-5p | down.mi | up.m |
| HSP90AB1 | hsa-miR-15b-5p | down.mi | up.m |
| HSP90B1 | hsa-miR-3154 | down.mi | up.m |
| HSP90B1 | hsa-miR-223-3p | down.mi | up.m |
| HSP90B1 | hsa-miR-1-3p | down.mi | up.m |
| HSP90B1 | hsa-miR-218-5p | down.mi | up.m |
| HSP90B1 | hsa-miR-206 | down.mi | up.m |
| HSPA1B | hsa-let-7b-5p | down.mi | up.m |
| HSPA1B | hsa-miR-30d-5p | down.mi | up.m |
| HSPA1B | hsa-miR-15b-5p | down.mi | up.m |
| HSPA1B | hsa-miR-195-5p | down.mi | up.m |
| HSPA1B | hsa-miR-340-5p | down.mi | up.m |
| HSPA1B | hsa-miR-6788-3p | down.mi | up.m |
| HSPA1B | hsa-miR-378a-5p | down.mi | up.m |
| HSPA1B | hsa-miR-378a-3p | down.mi | up.m |
| HSPA5 | hsa-miR-584-5p | down.mi | up.m |
| HSPA5 | hsa-miR-30a-5p | down.mi | up.m |
| HSPA6 | hsa-miR-1247-3p | down.mi | up.m |
| HSPA6 | hsa-miR-30a-3p | down.mi | up.m |
| HSPD1 | hsa-miR-1-3p | down.mi | up.m |
| HYOU1 | hsa-miR-195-5p | down.mi | up.m |
| HYOU1 | hsa-miR-15b-5p | down.mi | up.m |
| IARS | hsa-let-7a-5p | down.mi | up.m |
| IARS2 | hsa-let-7b-5p | down.mi | up.m |
| IDE | hsa-miR-378a-5p | down.mi | up.m |
| IDH1 | hsa-miR-30a-5p | down.mi | up.m |
| IFNGR2 | hsa-miR-15b-5p | down.mi | up.m |
| IGF2BP3 | hsa-let-7g-5p | down.mi | up.m |
| IGF2BP3 | hsa-let-7b-5p | down.mi | up.m |
| IGF2BP3 | hsa-let-7f-5p | down.mi | up.m |
| IGF2BP3 | hsa-let-7e-5p | down.mi | up.m |
| IGF2BP3 | hsa-let-7a-5p | down.mi | up.m |
| IGF2BP3 | hsa-let-7c-5p | down.mi | up.m |
| IGFBP2 | hsa-miR-204-5p | down.mi | up.m |
| IL20RB | hsa-miR-144-3p | down.mi | up.m |
| IL31RA | hsa-miR-486-3p | down.mi | up.m |
| IMP4 | hsa-miR-221-5p | down.mi | up.m |
| IMPDH2 | hsa-miR-218-5p | down.mi | up.m |
| IMPDH2 | hsa-let-7b-5p | down.mi | up.m |
| IMPDH2 | hsa-miR-378a-5p | down.mi | up.m |
| INPPL1 | hsa-miR-184 | down.mi | up.m |
| INPPL1 | hsa-let-7b-5p | down.mi | up.m |
| INTS1 | hsa-miR-218-5p | down.mi | up.m |
| INTS1 | hsa-let-7b-5p | down.mi | up.m |
| INTS7 | hsa-let-7g-5p | down.mi | up.m |
| INTS7 | hsa-let-7e-5p | down.mi | up.m |
| INTS7 | hsa-let-7b-5p | down.mi | up.m |
| INTS7 | hsa-let-7c-5p | down.mi | up.m |
| INTS7 | hsa-miR-30b-3p | down.mi | up.m |
| INTS7 | hsa-miR-6720-3p | down.mi | up.m |
| INTS7 | hsa-let-7a-5p | down.mi | up.m |
| INTS7 | hsa-let-7f-5p | down.mi | up.m |
| INTS8 | hsa-miR-340-5p | down.mi | up.m |
| IPO4 | hsa-let-7b-5p | down.mi | up.m |
| IQGAP3 | hsa-miR-1-3p | down.mi | up.m |
| IRAK2 | hsa-miR-3614-5p | down.mi | up.m |
| IRAK2 | hsa-miR-34b-3p | down.mi | up.m |
| ISG15 | hsa-miR-1-3p | down.mi | up.m |
| ISG20L2 | hsa-miR-378a-5p | down.mi | up.m |
| ISOC2 | hsa-let-7b-5p | down.mi | up.m |
| ITGA2 | hsa-miR-15b-5p | down.mi | up.m |
| ITGA2 | hsa-miR-1247-3p | down.mi | up.m |
| ITGA2 | hsa-miR-30a-5p | down.mi | up.m |
| ITGA2 | hsa-miR-195-5p | down.mi | up.m |
| ITGAV | hsa-miR-218-5p | down.mi | up.m |
| ITGB4 | hsa-miR-143-3p | down.mi | up.m |
| ITGB4 | hsa-miR-1-3p | down.mi | up.m |
| ITGB4 | hsa-miR-30a-5p | down.mi | up.m |
| ITM2C | hsa-miR-218-5p | down.mi | up.m |
| JMJD4 | hsa-miR-486-3p | down.mi | up.m |
| JUP | hsa-miR-1-3p | down.mi | up.m |
| JUP | hsa-miR-30a-5p | down.mi | up.m |
| KAT2A | hsa-miR-1-3p | down.mi | up.m |
| KCNK1 | hsa-miR-340-5p | down.mi | up.m |
| KCNK5 | hsa-miR-30c-2-3p | down.mi | up.m |
| KCNK5 | hsa-miR-34b-3p | down.mi | up.m |
| KCNK5 | hsa-miR-30b-3p | down.mi | up.m |
| KCNN4 | hsa-miR-30a-5p | down.mi | up.m |
| KCNN4 | hsa-miR-1-3p | down.mi | up.m |
| KCTD5 | hsa-miR-30d-5p | down.mi | up.m |
| KCTD5 | hsa-miR-30a-5p | down.mi | up.m |
| KDELR1 | hsa-miR-340-5p | down.mi | up.m |
| KDELR1 | hsa-miR-34c-3p | down.mi | up.m |
| KDELR1 | hsa-miR-150-3p | down.mi | up.m |
| KDELR1 | hsa-let-7b-5p | down.mi | up.m |
| KDELR1 | hsa-miR-1-3p | down.mi | up.m |
| KIAA0895L | hsa-miR-2110 | down.mi | up.m |
| KIAA0907 | hsa-miR-140-3p | down.mi | up.m |
| KIAA1522 | hsa-miR-1-3p | down.mi | up.m |
| KIAA1522 | hsa-miR-218-5p | down.mi | up.m |
| KIF11 | hsa-miR-30a-5p | down.mi | up.m |
| KIF11 | hsa-miR-30d-5p | down.mi | up.m |
| KIF11 | hsa-miR-340-5p | down.mi | up.m |
| KIF15 | hsa-miR-218-5p | down.mi | up.m |
| KIF18B | hsa-miR-6788-3p | down.mi | up.m |
| KIF1A | hsa-miR-423-5p | down.mi | up.m |
| KIF23 | hsa-miR-340-5p | down.mi | up.m |
| KIF23 | hsa-miR-195-5p | down.mi | up.m |
| KIF23 | hsa-miR-15b-5p | down.mi | up.m |
| KIF2A | hsa-miR-1-3p | down.mi | up.m |
| KIF2A | hsa-let-7b-5p | down.mi | up.m |
| KIF2C | hsa-miR-1-3p | down.mi | up.m |
| KIF2C | hsa-miR-3154 | down.mi | up.m |
| KIF4A | hsa-miR-1-3p | down.mi | up.m |
| KIFC1 | hsa-let-7b-5p | down.mi | up.m |
| KLC2 | hsa-miR-1247-5p | down.mi | up.m |
| KLC2 | hsa-miR-30b-3p | down.mi | up.m |
| KLC2 | hsa-miR-195-5p | down.mi | up.m |
| KLC2 | hsa-miR-15b-5p | down.mi | up.m |
| KLC2 | hsa-miR-184 | down.mi | up.m |
| KLK12 | hsa-miR-1-3p | down.mi | up.m |
| KLK6 | hsa-let-7f-5p | down.mi | up.m |
| KMT2B | hsa-miR-7704 | down.mi | up.m |
| KMT2B | hsa-miR-3154 | down.mi | up.m |
| KMT2B | hsa-miR-423-5p | down.mi | up.m |
| KNSTRN | hsa-miR-1247-3p | down.mi | up.m |
| KNTC1 | hsa-miR-204-5p | down.mi | up.m |
| KPNA2 | hsa-miR-144-3p | down.mi | up.m |
| KPNA2 | hsa-miR-139-5p | down.mi | up.m |
| KPNA2 | hsa-miR-30a-3p | down.mi | up.m |
| KPNB1 | hsa-miR-30d-5p | down.mi | up.m |
| KRAS | hsa-let-7g-5p | down.mi | up.m |
| KRAS | hsa-miR-206 | down.mi | up.m |
| KRAS | hsa-miR-30a-3p | down.mi | up.m |
| KRAS | hsa-miR-143-3p | down.mi | up.m |
| KRAS | hsa-let-7a-5p | down.mi | up.m |
| KRAS | hsa-miR-1-3p | down.mi | up.m |
| KRAS | hsa-miR-340-5p | down.mi | up.m |
| KRT6B | hsa-miR-30a-5p | down.mi | up.m |
| KRT8 | hsa-miR-4529-3p | down.mi | up.m |
| KRT8 | hsa-miR-30b-3p | down.mi | up.m |
| KRT8 | hsa-miR-486-3p | down.mi | up.m |
| KRT80 | hsa-miR-486-3p | down.mi | up.m |
| KRT80 | hsa-miR-139-3p | down.mi | up.m |
| L2HGDH | hsa-miR-195-5p | down.mi | up.m |
| L2HGDH | hsa-miR-15b-5p | down.mi | up.m |
| L2HGDH | hsa-miR-1247-3p | down.mi | up.m |
| LACTB2 | hsa-let-7b-5p | down.mi | up.m |
| LAMB3 | hsa-miR-218-5p | down.mi | up.m |
| LARP1 | hsa-miR-30a-5p | down.mi | up.m |
| LARP1 | hsa-miR-30d-5p | down.mi | up.m |
| LARP1 | hsa-let-7f-5p | down.mi | up.m |
| LARP1 | hsa-let-7b-5p | down.mi | up.m |
| LARP1 | hsa-miR-374a-5p | down.mi | up.m |
| LARP1 | hsa-let-7a-5p | down.mi | up.m |
| LARP1 | hsa-miR-423-5p | down.mi | up.m |
| LBR | hsa-miR-340-5p | down.mi | up.m |
| LBR | hsa-miR-144-5p | down.mi | up.m |
| LBR | hsa-let-7b-5p | down.mi | up.m |
| LCLAT1 | hsa-miR-30a-5p | down.mi | up.m |
| LCLAT1 | hsa-miR-340-5p | down.mi | up.m |
| LCLAT1 | hsa-miR-30d-5p | down.mi | up.m |
| LCLAT1 | hsa-miR-378a-5p | down.mi | up.m |
| LCN2 | hsa-miR-138-5p | down.mi | up.m |
| LCOR | hsa-miR-30a-5p | down.mi | up.m |
| LCOR | hsa-miR-139-5p | down.mi | up.m |
| LCOR | hsa-let-7b-3p | down.mi | up.m |
| LCOR | hsa-miR-30d-5p | down.mi | up.m |
| LCOR | hsa-miR-144-3p | down.mi | up.m |
| LCOR | hsa-miR-2110 | down.mi | up.m |
| LDHA | hsa-let-7c-5p | down.mi | up.m |
| LDHA | hsa-miR-378a-3p | down.mi | up.m |
| LDHA | hsa-miR-374a-5p | down.mi | up.m |
| LDHA | hsa-miR-34b-3p | down.mi | up.m |
| LDHA | hsa-miR-190a-5p | down.mi | up.m |
| LDOC1 | hsa-miR-221-5p | down.mi | up.m |
| LGALS3BP | hsa-miR-1-3p | down.mi | up.m |
| LGR4 | hsa-let-7b-5p | down.mi | up.m |
| LGR4 | hsa-miR-218-5p | down.mi | up.m |
| LGSN | hsa-miR-378c | down.mi | up.m |
| LGSN | hsa-miR-378a-3p | down.mi | up.m |
| LGSN | hsa-miR-221-5p | down.mi | up.m |
| LGSN | hsa-miR-486-3p | down.mi | up.m |
| LIF | hsa-miR-223-3p | down.mi | up.m |
| LIG3 | hsa-miR-30a-5p | down.mi | up.m |
| LIMD2 | hsa-miR-139-3p | down.mi | up.m |
| LIMD2 | hsa-miR-6892-5p | down.mi | up.m |
| LIMD2 | hsa-let-7e-5p | down.mi | up.m |
| LIMD2 | hsa-let-7a-5p | down.mi | up.m |
| LIMD2 | hsa-let-7f-5p | down.mi | up.m |
| LIMD2 | hsa-let-7b-5p | down.mi | up.m |
| LIMD2 | hsa-let-7g-5p | down.mi | up.m |
| LIMD2 | hsa-let-7c-5p | down.mi | up.m |
| LIMK1 | hsa-miR-30c-2-3p | down.mi | up.m |
| LIMK1 | hsa-miR-143-3p | down.mi | up.m |
| LMAN2 | hsa-miR-99a-5p | down.mi | up.m |
| LMAN2 | hsa-let-7b-5p | down.mi | up.m |
| LMAN2 | hsa-miR-374a-5p | down.mi | up.m |
| LMNB1 | hsa-miR-30a-3p | down.mi | up.m |
| LMNB1 | hsa-miR-1-3p | down.mi | up.m |
| LMNB1 | hsa-miR-218-5p | down.mi | up.m |
| LMNB2 | hsa-miR-143-3p | down.mi | up.m |
| LMNB2 | hsa-miR-378c | down.mi | up.m |
| LMNB2 | hsa-miR-144-3p | down.mi | up.m |
| LMNB2 | hsa-miR-490-3p | down.mi | up.m |
| LMNB2 | hsa-miR-423-5p | down.mi | up.m |
| LMNB2 | hsa-miR-27a-5p | down.mi | up.m |
| LMNB2 | hsa-miR-145-3p | down.mi | up.m |
| LMNB2 | hsa-miR-30a-5p | down.mi | up.m |
| LMNB2 | hsa-miR-378a-3p | down.mi | up.m |
| LOXL2 | hsa-miR-218-5p | down.mi | up.m |
| LPAR2 | hsa-miR-598-3p | down.mi | up.m |
| LPAR2 | hsa-miR-490-3p | down.mi | up.m |
| LPAR2 | hsa-miR-6720-3p | down.mi | up.m |
| LPGAT1 | hsa-let-7b-5p | down.mi | up.m |
| LPIN3 | hsa-miR-1247-3p | down.mi | up.m |
| LRIF1 | hsa-miR-1258 | down.mi | up.m |
| LRIF1 | hsa-miR-195-5p | down.mi | up.m |
| LRIF1 | hsa-miR-15b-5p | down.mi | up.m |
| LRIG3 | hsa-miR-3614-5p | down.mi | up.m |
| LRIG3 | hsa-let-7g-5p | down.mi | up.m |
| LRIG3 | hsa-let-7f-5p | down.mi | up.m |
| LRIG3 | hsa-let-7c-5p | down.mi | up.m |
| LRIG3 | hsa-let-7e-5p | down.mi | up.m |
| LRIG3 | hsa-let-7a-5p | down.mi | up.m |
| LRIG3 | hsa-let-7b-5p | down.mi | up.m |
| LRIG3 | hsa-miR-99a-5p | down.mi | up.m |
| LRP8 | hsa-miR-374a-5p | down.mi | up.m |
| LRPPRC | hsa-miR-340-5p | down.mi | up.m |
| LRPPRC | hsa-miR-195-5p | down.mi | up.m |
| LRPPRC | hsa-miR-139-3p | down.mi | up.m |
| LRPPRC | hsa-miR-15b-5p | down.mi | up.m |
| LRRC20 | hsa-let-7e-5p | down.mi | up.m |
| LRRC20 | hsa-let-7a-5p | down.mi | up.m |
| LRRC20 | hsa-let-7b-5p | down.mi | up.m |
| LRRC20 | hsa-let-7c-5p | down.mi | up.m |
| LRRC20 | hsa-miR-140-3p | down.mi | up.m |
| LRRC20 | hsa-let-7f-5p | down.mi | up.m |
| LRRC20 | hsa-miR-30b-3p | down.mi | up.m |
| LRRC20 | hsa-let-7g-5p | down.mi | up.m |
| LRRC42 | hsa-let-7a-5p | down.mi | up.m |
| LRRC42 | hsa-miR-30a-3p | down.mi | up.m |
| LRRC45 | hsa-miR-139-3p | down.mi | up.m |
| LRRC59 | hsa-miR-206 | down.mi | up.m |
| LRRC59 | hsa-miR-1-3p | down.mi | up.m |
| LSM12 | hsa-miR-30a-5p | down.mi | up.m |
| LSR | hsa-let-7e-5p | down.mi | up.m |
| LUC7L3 | hsa-miR-15b-5p | down.mi | up.m |
| LUC7L3 | hsa-miR-195-5p | down.mi | up.m |
| LY6K | hsa-miR-30a-3p | down.mi | up.m |
| LYPD6B | hsa-miR-218-5p | down.mi | up.m |
| MAD2L1 | hsa-miR-1-3p | down.mi | up.m |
| MAGEA12 | hsa-let-7c-5p | down.mi | up.m |
| MAGEA12 | hsa-let-7g-5p | down.mi | up.m |
| MAGEA12 | hsa-let-7e-5p | down.mi | up.m |
| MAGEA12 | hsa-let-7f-5p | down.mi | up.m |
| MAGEA12 | hsa-let-7a-5p | down.mi | up.m |
| MAGEA12 | hsa-let-7b-5p | down.mi | up.m |
| MAGEA3 | hsa-let-7b-5p | down.mi | up.m |
| MAGEA3 | hsa-let-7g-5p | down.mi | up.m |
| MAGEA3 | hsa-let-7f-5p | down.mi | up.m |
| MAGEA3 | hsa-let-7a-5p | down.mi | up.m |
| MAGEA3 | hsa-let-7c-5p | down.mi | up.m |
| MAGEA3 | hsa-let-7e-5p | down.mi | up.m |
| MAGEA6 | hsa-let-7a-5p | down.mi | up.m |
| MAGEA6 | hsa-let-7f-5p | down.mi | up.m |
| MAGEA6 | hsa-let-7e-5p | down.mi | up.m |
| MAGEA6 | hsa-let-7b-5p | down.mi | up.m |
| MAGEA6 | hsa-let-7g-5p | down.mi | up.m |
| MAGEA6 | hsa-let-7c-5p | down.mi | up.m |
| MANEAL | hsa-miR-378a-5p | down.mi | up.m |
| MAP10 | hsa-miR-140-3p | down.mi | up.m |
| MAPK8IP3 | hsa-miR-378a-5p | down.mi | up.m |
| MAPK8IP3 | hsa-miR-6720-3p | down.mi | up.m |
| MAPK8IP3 | hsa-miR-423-5p | down.mi | up.m |
| MARCKSL1 | hsa-miR-30c-2-3p | down.mi | up.m |
| MARCKSL1 | hsa-let-7e-5p | down.mi | up.m |
| MARCKSL1 | hsa-miR-2110 | down.mi | up.m |
| MARCKSL1 | hsa-let-7g-5p | down.mi | up.m |
| MARCKSL1 | hsa-miR-140-3p | down.mi | up.m |
| MARCKSL1 | hsa-let-7f-5p | down.mi | up.m |
| MARCKSL1 | hsa-miR-30a-5p | down.mi | up.m |
| MARCKSL1 | hsa-let-7c-5p | down.mi | up.m |
| MARCKSL1 | hsa-let-7a-5p | down.mi | up.m |
| MARCKSL1 | hsa-miR-30d-5p | down.mi | up.m |
| MARCKSL1 | hsa-let-7b-5p | down.mi | up.m |
| MARK2 | hsa-miR-190a-5p | down.mi | up.m |
| MARK2 | hsa-miR-2110 | down.mi | up.m |
| MARK2 | hsa-miR-99a-5p | down.mi | up.m |
| MARS | hsa-let-7b-5p | down.mi | up.m |
| MARS2 | hsa-let-7c-5p | down.mi | up.m |
| MARS2 | hsa-let-7g-5p | down.mi | up.m |
| MARS2 | hsa-let-7a-5p | down.mi | up.m |
| MARS2 | hsa-let-7b-5p | down.mi | up.m |
| MARS2 | hsa-let-7f-5p | down.mi | up.m |
| MARS2 | hsa-let-7e-5p | down.mi | up.m |
| MAZ | hsa-miR-27a-5p | down.mi | up.m |
| MAZ | hsa-miR-30b-3p | down.mi | up.m |
| MAZ | hsa-miR-7704 | down.mi | up.m |
| MAZ | hsa-miR-30c-2-3p | down.mi | up.m |
| MAZ | hsa-miR-30d-5p | down.mi | up.m |
| MCCC2 | hsa-miR-3614-5p | down.mi | up.m |
| MCM2 | hsa-miR-1-3p | down.mi | up.m |
| MCM3 | hsa-miR-1-3p | down.mi | up.m |
| MCM4 | hsa-let-7b-5p | down.mi | up.m |
| MCM4 | hsa-miR-1-3p | down.mi | up.m |
| MCM5 | hsa-miR-423-5p | down.mi | up.m |
| MCM5 | hsa-miR-1-3p | down.mi | up.m |
| MCM6 | hsa-miR-1-3p | down.mi | up.m |
| MCM7 | hsa-miR-30c-2-3p | down.mi | up.m |
| MCM7 | hsa-miR-423-5p | down.mi | up.m |
| MCM7 | hsa-miR-1-3p | down.mi | up.m |
| MCM7 | hsa-let-7b-5p | down.mi | up.m |
| MCM8 | hsa-miR-99a-5p | down.mi | up.m |
| MCM8 | hsa-miR-1247-3p | down.mi | up.m |
| MCM8 | hsa-miR-374a-5p | down.mi | up.m |
| MCM8 | hsa-miR-144-3p | down.mi | up.m |
| MCTS1 | hsa-miR-378a-5p | down.mi | up.m |
| MCU | hsa-miR-139-3p | down.mi | up.m |
| MDFI | hsa-miR-204-5p | down.mi | up.m |
| ME3 | hsa-miR-30a-5p | down.mi | up.m |
| MEA1 | hsa-miR-218-5p | down.mi | up.m |
| MED14 | hsa-let-7b-5p | down.mi | up.m |
| MED8 | hsa-miR-204-5p | down.mi | up.m |
| MELK | hsa-miR-378a-5p | down.mi | up.m |
| MEN1 | hsa-miR-486-3p | down.mi | up.m |
| METAP1 | hsa-miR-143-3p | down.mi | up.m |
| METTL1 | hsa-miR-3154 | down.mi | up.m |
| METTL21A | hsa-miR-6720-3p | down.mi | up.m |
| METTL23 | hsa-miR-99a-5p | down.mi | up.m |
| MEX3A | hsa-miR-423-5p | down.mi | up.m |
| MEX3A | hsa-miR-138-5p | down.mi | up.m |
| MEX3A | hsa-miR-204-5p | down.mi | up.m |
| MFSD3 | hsa-let-7b-5p | down.mi | up.m |
| MFSD9 | hsa-miR-4732-3p | down.mi | up.m |
| MGME1 | hsa-let-7b-5p | down.mi | up.m |
| MIF | hsa-miR-451a | down.mi | up.m |
| MIOX | hsa-miR-4732-3p | down.mi | up.m |
| MIS18A | hsa-miR-374a-5p | down.mi | up.m |
| MKI67 | hsa-miR-218-5p | down.mi | up.m |
| MKI67 | hsa-let-7e-5p | down.mi | up.m |
| MKI67 | hsa-miR-1-3p | down.mi | up.m |
| MLEC | hsa-miR-139-5p | down.mi | up.m |
| MLEC | hsa-miR-133a-3p | down.mi | up.m |
| MLEC | hsa-miR-144-3p | down.mi | up.m |
| MLLT11 | hsa-miR-30a-5p | down.mi | up.m |
| MMACHC | hsa-let-7b-5p | down.mi | up.m |
| MMP11 | hsa-miR-139-5p | down.mi | up.m |
| MMP11 | hsa-miR-139-3p | down.mi | up.m |
| MMP13 | hsa-miR-143-3p | down.mi | up.m |
| MMP14 | hsa-miR-133b | down.mi | up.m |
| MMP14 | hsa-miR-143-3p | down.mi | up.m |
| MMP14 | hsa-miR-133a-3p | down.mi | up.m |
| MMP17 | hsa-miR-423-5p | down.mi | up.m |
| MMP3 | hsa-miR-138-5p | down.mi | up.m |
| MMP9 | hsa-miR-15b-5p | down.mi | up.m |
| MMP9 | hsa-miR-143-3p | down.mi | up.m |
| MMP9 | hsa-let-7e-5p | down.mi | up.m |
| MMP9 | hsa-miR-133b | down.mi | up.m |
| MMP9 | hsa-miR-204-5p | down.mi | up.m |
| MMP9 | hsa-miR-451a | down.mi | up.m |
| MOGS | hsa-miR-378a-5p | down.mi | up.m |
| MORC2 | hsa-miR-218-5p | down.mi | up.m |
| MOV10 | hsa-let-7b-5p | down.mi | up.m |
| MOV10 | hsa-miR-1-3p | down.mi | up.m |
| MPG | hsa-let-7b-5p | down.mi | up.m |
| MRM1 | hsa-let-7b-5p | down.mi | up.m |
| MRPL12 | hsa-miR-2110 | down.mi | up.m |
| MRPL12 | hsa-miR-125b-2-3p | down.mi | up.m |
| MRPL12 | hsa-let-7b-5p | down.mi | up.m |
| MRPL12 | hsa-let-7f-5p | down.mi | up.m |
| MRPL12 | hsa-miR-30b-3p | down.mi | up.m |
| MRPL12 | hsa-let-7g-5p | down.mi | up.m |
| MRPL12 | hsa-let-7c-5p | down.mi | up.m |
| MRPL12 | hsa-let-7e-5p | down.mi | up.m |
| MRPL12 | hsa-miR-30c-2-3p | down.mi | up.m |
| MRPL12 | hsa-let-7a-5p | down.mi | up.m |
| MRPL13 | hsa-miR-218-5p | down.mi | up.m |
| MRPL15 | hsa-let-7a-5p | down.mi | up.m |
| MRPL17 | hsa-miR-30c-2-3p | down.mi | up.m |
| MRPL17 | hsa-miR-6892-5p | down.mi | up.m |
| MRPL19 | hsa-miR-1-3p | down.mi | up.m |
| MRPL28 | hsa-miR-30a-5p | down.mi | up.m |
| MRPL36 | hsa-miR-1247-3p | down.mi | up.m |
| MRPL36 | hsa-miR-340-5p | down.mi | up.m |
| MRPL37 | hsa-let-7b-5p | down.mi | up.m |
| MRPL37 | hsa-miR-378a-3p | down.mi | up.m |
| MRPL47 | hsa-let-7b-5p | down.mi | up.m |
| MRPL9 | hsa-miR-139-3p | down.mi | up.m |
| MRPS10 | hsa-miR-378a-5p | down.mi | up.m |
| MRPS10 | hsa-miR-490-3p | down.mi | up.m |
| MRPS15 | hsa-miR-204-5p | down.mi | up.m |
| MRPS16 | hsa-miR-30b-3p | down.mi | up.m |
| MRPS16 | hsa-miR-30c-2-3p | down.mi | up.m |
| MRPS16 | hsa-miR-221-5p | down.mi | up.m |
| MRPS18A | hsa-miR-218-5p | down.mi | up.m |
| MRPS2 | hsa-miR-218-5p | down.mi | up.m |
| MRPS2 | hsa-let-7a-5p | down.mi | up.m |
| MRPS2 | hsa-let-7e-5p | down.mi | up.m |
| MRPS21 | hsa-miR-34b-3p | down.mi | up.m |
| MRPS21 | hsa-miR-3614-5p | down.mi | up.m |
| MRPS23 | hsa-miR-490-3p | down.mi | up.m |
| MRPS23 | hsa-miR-30b-3p | down.mi | up.m |
| MRPS24 | hsa-let-7b-5p | down.mi | up.m |
| MRPS25 | hsa-miR-150-3p | down.mi | up.m |
| MRPS30 | hsa-miR-195-3p | down.mi | up.m |
| MRPS33 | hsa-let-7b-5p | down.mi | up.m |
| MRPS33 | hsa-miR-99a-5p | down.mi | up.m |
| MRPS5 | hsa-miR-30a-5p | down.mi | up.m |
| MSC | hsa-miR-378a-3p | down.mi | up.m |
| MSC | hsa-miR-218-5p | down.mi | up.m |
| MSH2 | hsa-miR-1-3p | down.mi | up.m |
| MSH6 | hsa-let-7b-3p | down.mi | up.m |
| MSH6 | hsa-miR-1-3p | down.mi | up.m |
| MSI1 | hsa-let-7b-5p | down.mi | up.m |
| MSI1 | hsa-miR-2110 | down.mi | up.m |
| MSI2 | hsa-let-7c-5p | down.mi | up.m |
| MSI2 | hsa-miR-338-5p | down.mi | up.m |
| MSI2 | hsa-let-7f-5p | down.mi | up.m |
| MSI2 | hsa-let-7g-5p | down.mi | up.m |
| MSI2 | hsa-let-7b-5p | down.mi | up.m |
| MSI2 | hsa-let-7a-5p | down.mi | up.m |
| MSI2 | hsa-let-7e-5p | down.mi | up.m |
| MTA1 | hsa-miR-486-3p | down.mi | up.m |
| MTA1 | hsa-miR-423-5p | down.mi | up.m |
| MTA3 | hsa-miR-204-5p | down.mi | up.m |
| MTA3 | hsa-miR-143-3p | down.mi | up.m |
| MTCH2 | hsa-miR-423-5p | down.mi | up.m |
| MTCH2 | hsa-let-7e-5p | down.mi | up.m |
| MTFP1 | hsa-let-7b-5p | down.mi | up.m |
| MTFR1 | hsa-let-7e-5p | down.mi | up.m |
| MTFR1 | hsa-let-7b-3p | down.mi | up.m |
| MTHFD1 | hsa-miR-3614-5p | down.mi | up.m |
| MTHFD1 | hsa-miR-34b-3p | down.mi | up.m |
| MTHFD1 | hsa-miR-139-3p | down.mi | up.m |
| MTHFD1 | hsa-let-7a-5p | down.mi | up.m |
| MTHFD1 | hsa-miR-378a-5p | down.mi | up.m |
| MTHFD1L | hsa-miR-138-5p | down.mi | up.m |
| MTHFD1L | hsa-miR-218-5p | down.mi | up.m |
| MTHFD2 | hsa-miR-1-3p | down.mi | up.m |
| MTHFD2 | hsa-miR-30d-5p | down.mi | up.m |
| MTHFD2 | hsa-miR-30a-3p | down.mi | up.m |
| MTMR4 | hsa-miR-195-5p | down.mi | up.m |
| MTMR4 | hsa-miR-218-5p | down.mi | up.m |
| MTMR4 | hsa-miR-15b-5p | down.mi | up.m |
| MTX1 | hsa-miR-1-3p | down.mi | up.m |
| MUC4 | hsa-miR-204-5p | down.mi | up.m |
| MUC4 | hsa-miR-378a-5p | down.mi | up.m |
| MYBBP1A | hsa-miR-30b-3p | down.mi | up.m |
| MYBL2 | hsa-miR-30a-5p | down.mi | up.m |
| MYBL2 | hsa-miR-30d-5p | down.mi | up.m |
| MYBL2 | hsa-miR-423-5p | down.mi | up.m |
| MYBL2 | hsa-miR-138-5p | down.mi | up.m |
| MYCN | hsa-let-7e-5p | down.mi | up.m |
| MYCN | hsa-miR-144-3p | down.mi | up.m |
| MYCN | hsa-miR-34b-3p | down.mi | up.m |
| MYO1E | hsa-let-7b-5p | down.mi | up.m |
| NAA10 | hsa-let-7b-5p | down.mi | up.m |
| NAA25 | hsa-miR-15b-5p | down.mi | up.m |
| NAA25 | hsa-let-7b-5p | down.mi | up.m |
| NAA25 | hsa-miR-195-5p | down.mi | up.m |
| NAA40 | hsa-let-7b-5p | down.mi | up.m |
| NABP2 | hsa-let-7b-5p | down.mi | up.m |
| NACC1 | hsa-miR-486-3p | down.mi | up.m |
| NACC1 | hsa-miR-3614-5p | down.mi | up.m |
| NACC1 | hsa-miR-218-5p | down.mi | up.m |
| NACC1 | hsa-miR-195-3p | down.mi | up.m |
| NACC1 | hsa-miR-30c-2-3p | down.mi | up.m |
| NACC1 | hsa-miR-2110 | down.mi | up.m |
| NACC1 | hsa-miR-138-5p | down.mi | up.m |
| NACC1 | hsa-miR-423-5p | down.mi | up.m |
| NACC1 | hsa-miR-144-3p | down.mi | up.m |
| NACC1 | hsa-miR-30b-3p | down.mi | up.m |
| NAPRT | hsa-miR-218-5p | down.mi | up.m |
| NARF | hsa-miR-1247-3p | down.mi | up.m |
| NAT9 | hsa-miR-3154 | down.mi | up.m |
| NAV1 | hsa-miR-30b-3p | down.mi | up.m |
| NAV1 | hsa-miR-423-5p | down.mi | up.m |
| NAV1 | hsa-miR-218-5p | down.mi | up.m |
| NAV1 | hsa-miR-30c-2-3p | down.mi | up.m |
| NAV1 | hsa-miR-486-3p | down.mi | up.m |
| NCAPD2 | hsa-let-7b-5p | down.mi | up.m |
| NCAPD3 | hsa-miR-1-3p | down.mi | up.m |
| NCAPG | hsa-miR-1-3p | down.mi | up.m |
| NCAPG2 | hsa-let-7b-5p | down.mi | up.m |
| NCL | hsa-miR-30a-5p | down.mi | up.m |
| NCL | hsa-miR-338-5p | down.mi | up.m |
| NCL | hsa-miR-598-3p | down.mi | up.m |
| NCS1 | hsa-miR-423-5p | down.mi | up.m |
| NCS1 | hsa-miR-1-3p | down.mi | up.m |
| NCS1 | hsa-miR-486-3p | down.mi | up.m |
| NDE1 | hsa-miR-423-5p | down.mi | up.m |
| NDE1 | hsa-miR-378a-3p | down.mi | up.m |
| NDE1 | hsa-miR-99a-5p | down.mi | up.m |
| NDE1 | hsa-miR-30a-5p | down.mi | up.m |
| NDUFB11 | hsa-miR-2110 | down.mi | up.m |
| NDUFS2 | hsa-let-7a-5p | down.mi | up.m |
| NDUFS5 | hsa-let-7e-5p | down.mi | up.m |
| NDUFS5 | hsa-miR-30b-3p | down.mi | up.m |
| NDUFS5 | hsa-miR-30c-2-3p | down.mi | up.m |
| NELFCD | hsa-miR-1-3p | down.mi | up.m |
| NET1 | hsa-miR-340-5p | down.mi | up.m |
| NETO2 | hsa-miR-1-3p | down.mi | up.m |
| NFYA | hsa-miR-140-3p | down.mi | up.m |
| NHP2 | hsa-miR-218-5p | down.mi | up.m |
| NHS | hsa-miR-338-5p | down.mi | up.m |
| NIF3L1 | hsa-miR-1247-3p | down.mi | up.m |
| NIFK | hsa-miR-1-3p | down.mi | up.m |
| NIFK | hsa-miR-30a-5p | down.mi | up.m |
| NIP7 | hsa-miR-30a-5p | down.mi | up.m |
| NKIRAS2 | hsa-miR-2110 | down.mi | up.m |
| NKIRAS2 | hsa-let-7a-5p | down.mi | up.m |
| NLE1 | hsa-let-7b-5p | down.mi | up.m |
| NLGN2 | hsa-miR-7704 | down.mi | up.m |
| NLGN2 | hsa-miR-378a-3p | down.mi | up.m |
| NLGN2 | hsa-miR-3154 | down.mi | up.m |
| NLN | hsa-miR-144-3p | down.mi | up.m |
| NLN | hsa-miR-374a-5p | down.mi | up.m |
| NLN | hsa-miR-378a-5p | down.mi | up.m |
| NME1-NME2 | hsa-miR-1247-3p | down.mi | up.m |
| NME4 | hsa-let-7b-5p | down.mi | up.m |
| NME4 | hsa-miR-378a-3p | down.mi | up.m |
| NME4 | hsa-let-7a-5p | down.mi | up.m |
| NME4 | hsa-let-7c-5p | down.mi | up.m |
| NME4 | hsa-let-7e-5p | down.mi | up.m |
| NMNAT2 | hsa-miR-223-3p | down.mi | up.m |
| NMRAL1 | hsa-miR-218-5p | down.mi | up.m |
| NOB1 | hsa-miR-139-3p | down.mi | up.m |
| NOC2L | hsa-miR-1-3p | down.mi | up.m |
| NOL11 | hsa-miR-340-5p | down.mi | up.m |
| NOL6 | hsa-let-7b-5p | down.mi | up.m |
| NOLC1 | hsa-let-7b-5p | down.mi | up.m |
| NOLC1 | hsa-miR-378a-3p | down.mi | up.m |
| NOLC1 | hsa-let-7a-5p | down.mi | up.m |
| NOLC1 | hsa-miR-423-5p | down.mi | up.m |
| NOLC1 | hsa-let-7b-3p | down.mi | up.m |
| NOLC1 | hsa-let-7e-5p | down.mi | up.m |
| NOLC1 | hsa-miR-195-5p | down.mi | up.m |
| NONO | hsa-miR-1247-3p | down.mi | up.m |
| NONO | hsa-miR-30c-2-3p | down.mi | up.m |
| NOP14 | hsa-miR-30a-5p | down.mi | up.m |
| NOP14 | hsa-let-7b-5p | down.mi | up.m |
| NOTCH3 | hsa-miR-206 | down.mi | up.m |
| NOTCH3 | hsa-miR-1-3p | down.mi | up.m |
| NPEPPS | hsa-miR-516a-5p | down.mi | up.m |
| NPEPPS | hsa-miR-218-1-3p | down.mi | up.m |
| NPLOC4 | hsa-miR-423-5p | down.mi | up.m |
| NPLOC4 | hsa-miR-30c-2-3p | down.mi | up.m |
| NPLOC4 | hsa-miR-138-5p | down.mi | up.m |
| NPLOC4 | hsa-miR-374a-5p | down.mi | up.m |
| NPLOC4 | hsa-miR-218-5p | down.mi | up.m |
| NPLOC4 | hsa-miR-2110 | down.mi | up.m |
| NPM1 | hsa-miR-15b-5p | down.mi | up.m |
| NPM1 | hsa-miR-598-3p | down.mi | up.m |
| NPM1 | hsa-miR-144-3p | down.mi | up.m |
| NPM3 | hsa-miR-374a-5p | down.mi | up.m |
| NPM3 | hsa-miR-99a-5p | down.mi | up.m |
| NPTX1 | hsa-miR-27a-5p | down.mi | up.m |
| NPTX1 | hsa-miR-204-5p | down.mi | up.m |
| NPTX1 | hsa-let-7a-5p | down.mi | up.m |
| NPTX1 | hsa-let-7c-5p | down.mi | up.m |
| NR2F6 | hsa-miR-30a-3p | down.mi | up.m |
| NR2F6 | hsa-miR-30a-5p | down.mi | up.m |
| NR2F6 | hsa-miR-30d-5p | down.mi | up.m |
| NRIP3 | hsa-miR-30c-2-3p | down.mi | up.m |
| NRIP3 | hsa-miR-30b-3p | down.mi | up.m |
| NRSN2 | hsa-let-7e-5p | down.mi | up.m |
| NSF | hsa-miR-218-5p | down.mi | up.m |
| NSUN2 | hsa-let-7b-5p | down.mi | up.m |
| NT5C3A | hsa-miR-144-3p | down.mi | up.m |
| NT5C3A | hsa-miR-30a-5p | down.mi | up.m |
| NT5DC2 | hsa-let-7b-5p | down.mi | up.m |
| NT5DC2 | hsa-let-7e-5p | down.mi | up.m |
| NT5E | hsa-miR-30a-5p | down.mi | up.m |
| NT5E | hsa-miR-1-3p | down.mi | up.m |
| NUAK2 | hsa-miR-218-5p | down.mi | up.m |
| NUBP2 | hsa-let-7b-5p | down.mi | up.m |
| NUDT8 | hsa-let-7e-5p | down.mi | up.m |
| NUP155 | hsa-let-7e-5p | down.mi | up.m |
| NUP155 | hsa-let-7f-5p | down.mi | up.m |
| NUP155 | hsa-miR-30c-2-3p | down.mi | up.m |
| NUP155 | hsa-let-7g-5p | down.mi | up.m |
| NUP155 | hsa-miR-378a-5p | down.mi | up.m |
| NUP155 | hsa-let-7b-5p | down.mi | up.m |
| NUP155 | hsa-let-7c-5p | down.mi | up.m |
| NUP155 | hsa-let-7a-5p | down.mi | up.m |
| NUP155 | hsa-miR-30b-3p | down.mi | up.m |
| NUP205 | hsa-miR-490-3p | down.mi | up.m |
| NUP205 | hsa-miR-3614-5p | down.mi | up.m |
| NUP210 | hsa-miR-218-5p | down.mi | up.m |
| NUP210 | hsa-miR-15b-5p | down.mi | up.m |
| NUP210 | hsa-miR-1-3p | down.mi | up.m |
| NUP37 | hsa-miR-4732-3p | down.mi | up.m |
| NUP43 | hsa-miR-34b-3p | down.mi | up.m |
| NUP43 | hsa-miR-30c-2-3p | down.mi | up.m |
| NUP43 | hsa-miR-30b-3p | down.mi | up.m |
| NUP62 | hsa-miR-378a-5p | down.mi | up.m |
| NUPL2 | hsa-miR-7704 | down.mi | up.m |
| NUPL2 | hsa-miR-517a-3p | down.mi | up.m |
| NUPL2 | hsa-miR-517b-3p | down.mi | up.m |
| NUPL2 | hsa-miR-204-5p | down.mi | up.m |
| NUPL2 | hsa-miR-30a-3p | down.mi | up.m |
| NUPL2 | hsa-miR-1247-3p | down.mi | up.m |
| NUSAP1 | hsa-let-7b-5p | down.mi | up.m |
| NVL | hsa-let-7b-5p | down.mi | up.m |
| NVL | hsa-miR-138-5p | down.mi | up.m |
| OAS3 | hsa-miR-143-3p | down.mi | up.m |
| OAT | hsa-miR-1-3p | down.mi | up.m |
| OCIAD2 | hsa-miR-30b-3p | down.mi | up.m |
| OCIAD2 | hsa-miR-1-3p | down.mi | up.m |
| OGT | hsa-miR-15b-5p | down.mi | up.m |
| OGT | hsa-miR-195-5p | down.mi | up.m |
| OGT | hsa-miR-486-3p | down.mi | up.m |
| OGT | hsa-miR-3154 | down.mi | up.m |
| OGT | hsa-miR-34c-3p | down.mi | up.m |
| OIP5 | hsa-miR-139-5p | down.mi | up.m |
| OIP5 | hsa-miR-218-5p | down.mi | up.m |
| OIP5 | hsa-miR-15b-5p | down.mi | up.m |
| OLA1 | hsa-miR-584-5p | down.mi | up.m |
| ORC5 | hsa-miR-99a-5p | down.mi | up.m |
| OSMR | hsa-miR-2110 | down.mi | up.m |
| OVOL1 | hsa-miR-190a-5p | down.mi | up.m |
| P3H1 | hsa-miR-3154 | down.mi | up.m |
| P3H3 | hsa-miR-1-3p | down.mi | up.m |
| P3H4 | hsa-miR-1-3p | down.mi | up.m |
| P4HB | hsa-miR-378c | down.mi | up.m |
| P4HB | hsa-miR-378a-3p | down.mi | up.m |
| PA2G4 | hsa-let-7b-5p | down.mi | up.m |
| PA2G4 | hsa-miR-423-5p | down.mi | up.m |
| PA2G4 | hsa-let-7e-5p | down.mi | up.m |
| PABPC1 | hsa-miR-221-5p | down.mi | up.m |
| PABPC1 | hsa-miR-423-5p | down.mi | up.m |
| PABPC1 | hsa-let-7b-5p | down.mi | up.m |
| PABPC1 | hsa-miR-30a-5p | down.mi | up.m |
| PABPC4 | hsa-miR-218-5p | down.mi | up.m |
| PABPN1 | hsa-miR-140-3p | down.mi | up.m |
| PABPN1 | hsa-miR-144-3p | down.mi | up.m |
| PACSIN3 | hsa-miR-1-3p | down.mi | up.m |
| PAFAH1B3 | hsa-miR-1-3p | down.mi | up.m |
| PAFAH1B3 | hsa-let-7b-5p | down.mi | up.m |
| PAICS | hsa-miR-378a-5p | down.mi | up.m |
| PAICS | hsa-miR-30a-5p | down.mi | up.m |
| PAIP1 | hsa-miR-30a-5p | down.mi | up.m |
| PAK1 | hsa-miR-34b-3p | down.mi | up.m |
| PAK1 | hsa-miR-423-5p | down.mi | up.m |
| PAK1 | hsa-let-7a-5p | down.mi | up.m |
| PAK1 | hsa-let-7b-5p | down.mi | up.m |
| PAK1IP1 | hsa-miR-30a-5p | down.mi | up.m |
| PARP1 | hsa-miR-221-5p | down.mi | up.m |
| PARP1 | hsa-miR-223-3p | down.mi | up.m |
| PARP1 | hsa-let-7a-5p | down.mi | up.m |
| PARP2 | hsa-miR-125b-2-3p | down.mi | up.m |
| PARP2 | hsa-miR-34b-3p | down.mi | up.m |
| PARP2 | hsa-miR-3614-5p | down.mi | up.m |
| PARP2 | hsa-miR-378a-5p | down.mi | up.m |
| PCCB | hsa-let-7b-5p | down.mi | up.m |
| PCCB | hsa-miR-30b-3p | down.mi | up.m |
| PCCB | hsa-miR-144-3p | down.mi | up.m |
| PCCB | hsa-miR-378a-3p | down.mi | up.m |
| PCCB | hsa-miR-490-3p | down.mi | up.m |
| PCDH7 | hsa-miR-1-3p | down.mi | up.m |
| PCGF3 | hsa-let-7g-5p | down.mi | up.m |
| PCGF3 | hsa-let-7c-5p | down.mi | up.m |
| PCGF3 | hsa-let-7e-5p | down.mi | up.m |
| PCGF3 | hsa-let-7a-5p | down.mi | up.m |
| PCGF3 | hsa-let-7f-5p | down.mi | up.m |
| PCGF3 | hsa-let-7b-5p | down.mi | up.m |
| PCGF3 | hsa-miR-30c-2-3p | down.mi | up.m |
| PCNA | hsa-miR-30a-5p | down.mi | up.m |
| PDAP1 | hsa-miR-30c-2-3p | down.mi | up.m |
| PDCD1 | hsa-miR-195-5p | down.mi | up.m |
| PDCD1 | hsa-miR-15b-5p | down.mi | up.m |
| PDCD11 | hsa-let-7b-5p | down.mi | up.m |
| PDCD11 | hsa-let-7e-5p | down.mi | up.m |
| PDCD6 | hsa-miR-218-5p | down.mi | up.m |
| PDCL3 | hsa-miR-1247-3p | down.mi | up.m |
| PDCL3 | hsa-let-7b-5p | down.mi | up.m |
| PDF | hsa-miR-204-5p | down.mi | up.m |
| PDIA3 | hsa-miR-1-3p | down.mi | up.m |
| PDIA4 | hsa-miR-218-5p | down.mi | up.m |
| PDIA6 | hsa-miR-15b-5p | down.mi | up.m |
| PDIA6 | hsa-miR-195-5p | down.mi | up.m |
| PDIA6 | hsa-miR-145-3p | down.mi | up.m |
| PDIA6 | hsa-miR-218-5p | down.mi | up.m |
| PDIK1L | hsa-miR-338-5p | down.mi | up.m |
| PDIK1L | hsa-miR-195-5p | down.mi | up.m |
| PDIK1L | hsa-miR-15b-5p | down.mi | up.m |
| PDK1 | hsa-let-7b-5p | down.mi | up.m |
| PDLIM7 | hsa-miR-1-3p | down.mi | up.m |
| PDLIM7 | hsa-miR-218-5p | down.mi | up.m |
| PDLIM7 | hsa-miR-30a-5p | down.mi | up.m |
| PES1 | hsa-let-7e-5p | down.mi | up.m |
| PES1 | hsa-miR-30a-5p | down.mi | up.m |
| PEX11B | hsa-let-7c-5p | down.mi | up.m |
| PEX11B | hsa-let-7b-5p | down.mi | up.m |
| PEX11B | hsa-let-7f-5p | down.mi | up.m |
| PEX11B | hsa-let-7e-5p | down.mi | up.m |
| PEX11B | hsa-miR-6892-5p | down.mi | up.m |
| PEX11B | hsa-let-7g-5p | down.mi | up.m |
| PEX11B | hsa-miR-30a-5p | down.mi | up.m |
| PEX11B | hsa-let-7a-5p | down.mi | up.m |
| PEX6 | hsa-let-7c-5p | down.mi | up.m |
| PEX6 | hsa-let-7b-5p | down.mi | up.m |
| PFAS | hsa-let-7e-5p | down.mi | up.m |
| PFDN2 | hsa-miR-338-5p | down.mi | up.m |
| PFN2 | hsa-miR-138-5p | down.mi | up.m |
| PFN2 | hsa-miR-218-5p | down.mi | up.m |
| PGAM5 | hsa-miR-30c-2-3p | down.mi | up.m |
| PGAM5 | hsa-miR-30b-3p | down.mi | up.m |
| PGM2L1 | hsa-let-7b-5p | down.mi | up.m |
| PGM2L1 | hsa-let-7a-5p | down.mi | up.m |
| PGM2L1 | hsa-let-7e-5p | down.mi | up.m |
| PGM2L1 | hsa-miR-30b-3p | down.mi | up.m |
| PGM2L1 | hsa-let-7c-5p | down.mi | up.m |
| PGM2L1 | hsa-let-7f-5p | down.mi | up.m |
| PGM2L1 | hsa-let-7g-5p | down.mi | up.m |
| PGM2L1 | hsa-miR-221-5p | down.mi | up.m |
| PHF12 | hsa-miR-486-3p | down.mi | up.m |
| PHF12 | hsa-miR-30b-3p | down.mi | up.m |
| PHF12 | hsa-miR-34c-3p | down.mi | up.m |
| PHF19 | hsa-miR-223-3p | down.mi | up.m |
| PHF19 | hsa-miR-15b-5p | down.mi | up.m |
| PHF19 | hsa-miR-218-5p | down.mi | up.m |
| PHF19 | hsa-miR-195-5p | down.mi | up.m |
| PHF19 | hsa-miR-218-1-3p | down.mi | up.m |
| PHF6 | hsa-let-7a-5p | down.mi | up.m |
| PHKA1 | hsa-miR-195-5p | down.mi | up.m |
| PHKA1 | hsa-let-7b-5p | down.mi | up.m |
| PHKA1 | hsa-miR-15b-5p | down.mi | up.m |
| PHKA1 | hsa-let-7e-5p | down.mi | up.m |
| PHKA1 | hsa-let-7b-3p | down.mi | up.m |
| PHKG2 | hsa-miR-138-5p | down.mi | up.m |
| PHLDA2 | hsa-miR-30a-5p | down.mi | up.m |
| PHLDB3 | hsa-miR-15b-5p | down.mi | up.m |
| PIGM | hsa-miR-190a-5p | down.mi | up.m |
| PIGT | hsa-miR-1-3p | down.mi | up.m |
| PIGU | hsa-let-7b-5p | down.mi | up.m |
| PIGW | hsa-miR-1247-3p | down.mi | up.m |
| PIGW | hsa-miR-374a-5p | down.mi | up.m |
| PIGW | hsa-miR-584-5p | down.mi | up.m |
| PIP4K2C | hsa-miR-340-5p | down.mi | up.m |
| PITX2 | hsa-miR-374a-5p | down.mi | up.m |
| PKM | hsa-miR-218-5p | down.mi | up.m |
| PKM | hsa-miR-133a-3p | down.mi | up.m |
| PKM | hsa-miR-184 | down.mi | up.m |
| PKM | hsa-let-7a-5p | down.mi | up.m |
| PKM | hsa-miR-133b | down.mi | up.m |
| PKM | hsa-miR-30a-5p | down.mi | up.m |
| PKM | hsa-miR-423-5p | down.mi | up.m |
| PLA2G4A | hsa-miR-30a-5p | down.mi | up.m |
| PLAT | hsa-miR-204-5p | down.mi | up.m |
| PLEK2 | hsa-miR-138-5p | down.mi | up.m |
| PLEKHG2 | hsa-miR-221-5p | down.mi | up.m |
| PLEKHG2 | hsa-miR-1247-3p | down.mi | up.m |
| PLEKHG2 | hsa-miR-1-3p | down.mi | up.m |
| PLEKHG2 | hsa-miR-133a-3p | down.mi | up.m |
| PLIN3 | hsa-miR-30a-5p | down.mi | up.m |
| PLK1 | hsa-let-7e-5p | down.mi | up.m |
| PLK1 | hsa-let-7b-5p | down.mi | up.m |
| PLOD1 | hsa-let-7a-5p | down.mi | up.m |
| PLS1 | hsa-miR-144-3p | down.mi | up.m |
| PLS1 | hsa-miR-1-3p | down.mi | up.m |
| PLXNA3 | hsa-miR-490-3p | down.mi | up.m |
| PM20D2 | hsa-let-7e-5p | down.mi | up.m |
| PM20D2 | hsa-let-7g-5p | down.mi | up.m |
| PM20D2 | hsa-let-7f-5p | down.mi | up.m |
| PM20D2 | hsa-let-7a-5p | down.mi | up.m |
| PM20D2 | hsa-let-7b-5p | down.mi | up.m |
| PM20D2 | hsa-miR-190a-5p | down.mi | up.m |
| PM20D2 | hsa-let-7c-5p | down.mi | up.m |
| PMAIP1 | hsa-let-7a-5p | down.mi | up.m |
| PMAIP1 | hsa-let-7f-5p | down.mi | up.m |
| PMAIP1 | hsa-let-7c-5p | down.mi | up.m |
| PMAIP1 | hsa-let-7g-5p | down.mi | up.m |
| PMAIP1 | hsa-let-7e-5p | down.mi | up.m |
| PMAIP1 | hsa-let-7b-5p | down.mi | up.m |
| PNN | hsa-miR-1-3p | down.mi | up.m |
| POFUT2 | hsa-miR-486-3p | down.mi | up.m |
| POLA2 | hsa-let-7b-3p | down.mi | up.m |
| POLA2 | hsa-miR-145-3p | down.mi | up.m |
| POLA2 | hsa-miR-1-3p | down.mi | up.m |
| POLD1 | hsa-miR-1-3p | down.mi | up.m |
| POLD1 | hsa-let-7e-5p | down.mi | up.m |
| POLD2 | hsa-let-7b-5p | down.mi | up.m |
| POLM | hsa-miR-218-5p | down.mi | up.m |
| POLM | hsa-miR-378a-5p | down.mi | up.m |
| POLR1B | hsa-let-7b-5p | down.mi | up.m |
| POLR1B | hsa-miR-374a-5p | down.mi | up.m |
| POLR2D | hsa-let-7e-5p | down.mi | up.m |
| POLR2D | hsa-let-7a-5p | down.mi | up.m |
| POLR2D | hsa-let-7c-5p | down.mi | up.m |
| POLR2D | hsa-let-7b-5p | down.mi | up.m |
| POLR2D | hsa-let-7g-5p | down.mi | up.m |
| POLR2D | hsa-let-7f-5p | down.mi | up.m |
| POLR2H | hsa-let-7b-5p | down.mi | up.m |
| POLR2I | hsa-miR-1-3p | down.mi | up.m |
| POLR3E | hsa-miR-30d-5p | down.mi | up.m |
| POLR3E | hsa-miR-30a-5p | down.mi | up.m |
| POLR3E | hsa-miR-584-5p | down.mi | up.m |
| POLR3K | hsa-miR-30c-2-3p | down.mi | up.m |
| POLR3K | hsa-miR-30b-3p | down.mi | up.m |
| POP1 | hsa-miR-125b-2-3p | down.mi | up.m |
| POP1 | hsa-let-7b-5p | down.mi | up.m |
| POSTN | hsa-let-7f-5p | down.mi | up.m |
| POU2AF1 | hsa-miR-195-5p | down.mi | up.m |
| POU2AF1 | hsa-miR-15b-5p | down.mi | up.m |
| PPIA | hsa-miR-423-5p | down.mi | up.m |
| PPIA | hsa-miR-218-5p | down.mi | up.m |
| PPIA | hsa-miR-1-3p | down.mi | up.m |
| PPIB | hsa-miR-1-3p | down.mi | up.m |
| PPIB | hsa-miR-423-5p | down.mi | up.m |
| PPIB | hsa-miR-206 | down.mi | up.m |
| PPIF | hsa-miR-338-5p | down.mi | up.m |
| PPIF | hsa-miR-195-3p | down.mi | up.m |
| PPIL1 | hsa-miR-15b-5p | down.mi | up.m |
| PPIL1 | hsa-miR-584-5p | down.mi | up.m |
| PPIL1 | hsa-miR-195-5p | down.mi | up.m |
| PPIL2 | hsa-miR-15b-5p | down.mi | up.m |
| PPM1G | hsa-let-7b-5p | down.mi | up.m |
| PPM1H | hsa-miR-1-3p | down.mi | up.m |
| PPP1R16A | hsa-miR-1-3p | down.mi | up.m |
| PPP1R3B | hsa-miR-378a-5p | down.mi | up.m |
| PRAF2 | hsa-let-7c-5p | down.mi | up.m |
| PRAF2 | hsa-let-7b-5p | down.mi | up.m |
| PRDX2 | hsa-miR-1-3p | down.mi | up.m |
| PRDX3 | hsa-miR-374a-5p | down.mi | up.m |
| PRDX3 | hsa-miR-15b-5p | down.mi | up.m |
| PREB | hsa-let-7a-5p | down.mi | up.m |
| PRELID1 | hsa-miR-218-5p | down.mi | up.m |
| PRELID1 | hsa-miR-133a-3p | down.mi | up.m |
| PRIM1 | hsa-miR-221-5p | down.mi | up.m |
| PRIM1 | hsa-miR-30a-5p | down.mi | up.m |
| PRIM1 | hsa-let-7b-5p | down.mi | up.m |
| PRIM1 | hsa-miR-3614-5p | down.mi | up.m |
| PRIM2 | hsa-let-7g-5p | down.mi | up.m |
| PRIM2 | hsa-miR-30c-2-3p | down.mi | up.m |
| PRIM2 | hsa-miR-30b-3p | down.mi | up.m |
| PRIM2 | hsa-let-7a-5p | down.mi | up.m |
| PRIM2 | hsa-let-7c-5p | down.mi | up.m |
| PRIM2 | hsa-let-7b-5p | down.mi | up.m |
| PRIM2 | hsa-let-7e-5p | down.mi | up.m |
| PRIM2 | hsa-miR-3614-5p | down.mi | up.m |
| PRIM2 | hsa-let-7f-5p | down.mi | up.m |
| PRKDC | hsa-miR-218-5p | down.mi | up.m |
| PRPF19 | hsa-miR-30a-5p | down.mi | up.m |
| PRPF19 | hsa-let-7b-5p | down.mi | up.m |
| PRPF4 | hsa-miR-1247-3p | down.mi | up.m |
| PRR11 | hsa-miR-378a-5p | down.mi | up.m |
| PRR3 | hsa-miR-3154 | down.mi | up.m |
| PRRC2A | hsa-let-7b-5p | down.mi | up.m |
| PRRC2A | hsa-miR-218-5p | down.mi | up.m |
| PRRC2A | hsa-let-7c-5p | down.mi | up.m |
| PRRC2A | hsa-miR-605-5p | down.mi | up.m |
| PRRC2A | hsa-let-7e-5p | down.mi | up.m |
| PRRC2A | hsa-miR-423-5p | down.mi | up.m |
| PRRC2C | hsa-miR-195-5p | down.mi | up.m |
| PRRC2C | hsa-miR-218-5p | down.mi | up.m |
| PRRC2C | hsa-miR-221-5p | down.mi | up.m |
| PRRC2C | hsa-miR-223-3p | down.mi | up.m |
| PRRC2C | hsa-miR-15b-5p | down.mi | up.m |
| PRSS22 | hsa-let-7c-5p | down.mi | up.m |
| PRSS22 | hsa-let-7g-5p | down.mi | up.m |
| PRSS22 | hsa-let-7e-5p | down.mi | up.m |
| PRSS22 | hsa-let-7a-5p | down.mi | up.m |
| PRSS22 | hsa-let-7f-5p | down.mi | up.m |
| PRSS22 | hsa-let-7b-5p | down.mi | up.m |
| PSAT1 | hsa-miR-140-3p | down.mi | up.m |
| PSAT1 | hsa-miR-195-5p | down.mi | up.m |
| PSAT1 | hsa-miR-15b-5p | down.mi | up.m |
| PSAT1 | hsa-miR-1-3p | down.mi | up.m |
| PSD3 | hsa-miR-218-5p | down.mi | up.m |
| PSD3 | hsa-let-7b-5p | down.mi | up.m |
| PSD3 | hsa-let-7e-5p | down.mi | up.m |
| PSENEN | hsa-miR-30c-2-3p | down.mi | up.m |
| PSMA1 | hsa-miR-218-1-3p | down.mi | up.m |
| PSMA6 | hsa-let-7e-5p | down.mi | up.m |
| PSMB2 | hsa-let-7c-5p | down.mi | up.m |
| PSMB2 | hsa-miR-6720-3p | down.mi | up.m |
| PSMB2 | hsa-miR-1247-3p | down.mi | up.m |
| PSMB5 | hsa-miR-15b-5p | down.mi | up.m |
| PSMB5 | hsa-miR-218-5p | down.mi | up.m |
| PSMB5 | hsa-miR-195-5p | down.mi | up.m |
| PSMC4 | hsa-miR-133a-3p | down.mi | up.m |
| PSMD11 | hsa-miR-423-5p | down.mi | up.m |
| PSMD11 | hsa-miR-30c-2-3p | down.mi | up.m |
| PSMD2 | hsa-let-7e-5p | down.mi | up.m |
| PSMD3 | hsa-miR-378a-5p | down.mi | up.m |
| PSMD7 | hsa-miR-15b-5p | down.mi | up.m |
| PSMD7 | hsa-miR-338-5p | down.mi | up.m |
| PSMD7 | hsa-miR-30a-5p | down.mi | up.m |
| PSMD7 | hsa-miR-30d-5p | down.mi | up.m |
| PSME4 | hsa-miR-30a-5p | down.mi | up.m |
| PSME4 | hsa-miR-218-5p | down.mi | up.m |
| PSME4 | hsa-let-7e-5p | down.mi | up.m |
| PSMG1 | hsa-miR-133a-3p | down.mi | up.m |
| PSMG1 | hsa-miR-140-3p | down.mi | up.m |
| PSMG1 | hsa-miR-34c-3p | down.mi | up.m |
| PSMG1 | hsa-miR-1-3p | down.mi | up.m |
| PSPH | hsa-miR-340-5p | down.mi | up.m |
| PTBP1 | hsa-miR-133b | down.mi | up.m |
| PTBP1 | hsa-miR-15b-5p | down.mi | up.m |
| PTBP1 | hsa-miR-340-5p | down.mi | up.m |
| PTBP1 | hsa-miR-1-3p | down.mi | up.m |
| PTBP1 | hsa-miR-378a-3p | down.mi | up.m |
| PTBP1 | hsa-miR-374a-5p | down.mi | up.m |
| PTBP1 | hsa-miR-4732-3p | down.mi | up.m |
| PTBP1 | hsa-miR-30a-5p | down.mi | up.m |
| PTGES | hsa-miR-486-3p | down.mi | up.m |
| PTGES2 | hsa-miR-7704 | down.mi | up.m |
| PTGES2 | hsa-miR-1247-3p | down.mi | up.m |
| PTGES2 | hsa-let-7a-5p | down.mi | up.m |
| PTGES2 | hsa-let-7c-5p | down.mi | up.m |
| PTGES2 | hsa-let-7b-5p | down.mi | up.m |
| PTGFRN | hsa-let-7b-5p | down.mi | up.m |
| PTGFRN | hsa-miR-218-5p | down.mi | up.m |
| PTGFRN | hsa-miR-139-5p | down.mi | up.m |
| PTGFRN | hsa-miR-30a-5p | down.mi | up.m |
| PTK6 | hsa-miR-30c-2-3p | down.mi | up.m |
| PTK6 | hsa-miR-34b-3p | down.mi | up.m |
| PTK6 | hsa-miR-3614-5p | down.mi | up.m |
| PTK6 | hsa-miR-30b-3p | down.mi | up.m |
| PTK6 | hsa-miR-1247-3p | down.mi | up.m |
| PTK7 | hsa-miR-218-5p | down.mi | up.m |
| PTK7 | hsa-miR-3154 | down.mi | up.m |
| PTK7 | hsa-miR-2110 | down.mi | up.m |
| PTMA | hsa-miR-1-3p | down.mi | up.m |
| PTMA | hsa-miR-206 | down.mi | up.m |
| PTMA | hsa-miR-374a-5p | down.mi | up.m |
| PTMA | hsa-miR-423-5p | down.mi | up.m |
| PTMA | hsa-miR-125b-2-3p | down.mi | up.m |
| PTMA | hsa-miR-598-3p | down.mi | up.m |
| PTMA | hsa-miR-133b | down.mi | up.m |
| PTMA | hsa-miR-133a-3p | down.mi | up.m |
| PTMA | hsa-miR-378a-3p | down.mi | up.m |
| PTRH2 | hsa-miR-30b-3p | down.mi | up.m |
| PTRH2 | hsa-miR-30c-2-3p | down.mi | up.m |
| PTTG1 | hsa-let-7b-5p | down.mi | up.m |
| PTTG1 | hsa-miR-423-5p | down.mi | up.m |
| PUF60 | hsa-miR-218-5p | down.mi | up.m |
| PUS1 | hsa-let-7b-5p | down.mi | up.m |
| PXDN | hsa-let-7b-5p | down.mi | up.m |
| PXDN | hsa-miR-1-3p | down.mi | up.m |
| PYCR1 | hsa-let-7e-5p | down.mi | up.m |
| PYCR1 | hsa-miR-2110 | down.mi | up.m |
| PYCR2 | hsa-miR-30a-5p | down.mi | up.m |
| PYGB | hsa-miR-378a-5p | down.mi | up.m |
| PYGB | hsa-miR-1-3p | down.mi | up.m |
| PYGO2 | hsa-miR-218-1-3p | down.mi | up.m |
| QPCTL | hsa-miR-30b-3p | down.mi | up.m |
| QPCTL | hsa-miR-1247-3p | down.mi | up.m |
| QPCTL | hsa-miR-30c-2-3p | down.mi | up.m |
| QSOX2 | hsa-miR-340-5p | down.mi | up.m |
| RAB15 | hsa-miR-15b-5p | down.mi | up.m |
| RAB15 | hsa-miR-195-5p | down.mi | up.m |
| RAB19 | hsa-let-7e-5p | down.mi | up.m |
| RAB19 | hsa-let-7g-5p | down.mi | up.m |
| RAB19 | hsa-let-7f-5p | down.mi | up.m |
| RAB19 | hsa-let-7a-5p | down.mi | up.m |
| RAB19 | hsa-let-7b-5p | down.mi | up.m |
| RAB19 | hsa-let-7c-5p | down.mi | up.m |
| RAB34 | hsa-miR-30a-5p | down.mi | up.m |
| RAB34 | hsa-miR-1-3p | down.mi | up.m |
| RAB34 | hsa-let-7c-5p | down.mi | up.m |
| RAB38 | hsa-let-7b-5p | down.mi | up.m |
| RAB38 | hsa-miR-30a-5p | down.mi | up.m |
| RAB40C | hsa-let-7f-5p | down.mi | up.m |
| RAB40C | hsa-let-7a-5p | down.mi | up.m |
| RAB40C | hsa-let-7e-5p | down.mi | up.m |
| RAB40C | hsa-let-7c-5p | down.mi | up.m |
| RAB40C | hsa-let-7g-5p | down.mi | up.m |
| RAB40C | hsa-let-7b-5p | down.mi | up.m |
| RABEP2 | hsa-miR-378a-3p | down.mi | up.m |
| RAC3 | hsa-miR-30c-2-3p | down.mi | up.m |
| RACGAP1 | hsa-miR-140-3p | down.mi | up.m |
| RACGAP1 | hsa-miR-15b-5p | down.mi | up.m |
| RACGAP1 | hsa-miR-204-5p | down.mi | up.m |
| RACGAP1 | hsa-let-7e-5p | down.mi | up.m |
| RACGAP1 | hsa-miR-195-5p | down.mi | up.m |
| RAD51 | hsa-miR-378a-5p | down.mi | up.m |
| RAD51 | hsa-miR-30b-3p | down.mi | up.m |
| RAD51 | hsa-miR-490-3p | down.mi | up.m |
| RAE1 | hsa-miR-15b-5p | down.mi | up.m |
| RANGAP1 | hsa-miR-223-3p | down.mi | up.m |
| RAP2B | hsa-miR-30c-2-3p | down.mi | up.m |
| RAP2B | hsa-miR-30b-3p | down.mi | up.m |
| RAP2B | hsa-miR-490-3p | down.mi | up.m |
| RAP2B | hsa-miR-378a-5p | down.mi | up.m |
| RAPGEFL1 | hsa-let-7c-5p | down.mi | up.m |
| RARS | hsa-miR-99a-5p | down.mi | up.m |
| RASD1 | hsa-miR-139-3p | down.mi | up.m |
| RBBP4 | hsa-let-7e-5p | down.mi | up.m |
| RBBP4 | hsa-miR-30c-2-3p | down.mi | up.m |
| RBBP4 | hsa-miR-30b-3p | down.mi | up.m |
| RBBP4 | hsa-miR-378a-5p | down.mi | up.m |
| RBM12B | hsa-let-7e-5p | down.mi | up.m |
| RBM12B | hsa-let-7g-5p | down.mi | up.m |
| RBM12B | hsa-let-7b-5p | down.mi | up.m |
| RBM12B | hsa-miR-1-3p | down.mi | up.m |
| RBM12B | hsa-let-7a-5p | down.mi | up.m |
| RBM12B | hsa-let-7b-3p | down.mi | up.m |
| RBM12B | hsa-let-7c-5p | down.mi | up.m |
| RBM12B | hsa-let-7f-5p | down.mi | up.m |
| RBM12B | hsa-miR-486-5p | down.mi | up.m |
| RBM38 | hsa-miR-490-3p | down.mi | up.m |
| RCC2 | hsa-miR-378a-3p | down.mi | up.m |
| RCC2 | hsa-miR-378c | down.mi | up.m |
| RCC2 | hsa-miR-1-3p | down.mi | up.m |
| RCC2 | hsa-miR-4529-3p | down.mi | up.m |
| RCE1 | hsa-miR-423-5p | down.mi | up.m |
| RCOR2 | hsa-miR-1-3p | down.mi | up.m |
| RDH10 | hsa-miR-30b-3p | down.mi | up.m |
| RDH10 | hsa-let-7b-5p | down.mi | up.m |
| RDH10 | hsa-miR-218-5p | down.mi | up.m |
| REPIN1 | hsa-miR-486-3p | down.mi | up.m |
| REPIN1 | hsa-miR-4529-3p | down.mi | up.m |
| RER1 | hsa-miR-143-3p | down.mi | up.m |
| RFC2 | hsa-let-7b-5p | down.mi | up.m |
| RFC2 | hsa-miR-1-3p | down.mi | up.m |
| RFC2 | hsa-let-7e-5p | down.mi | up.m |
| RFC2 | hsa-miR-3614-5p | down.mi | up.m |
| RFC2 | hsa-let-7f-5p | down.mi | up.m |
| RFC2 | hsa-let-7c-5p | down.mi | up.m |
| RFC2 | hsa-let-7a-5p | down.mi | up.m |
| RFC2 | hsa-let-7g-5p | down.mi | up.m |
| RFC5 | hsa-miR-1-3p | down.mi | up.m |
| RFWD3 | hsa-miR-486-3p | down.mi | up.m |
| RFWD3 | hsa-let-7c-5p | down.mi | up.m |
| RFWD3 | hsa-miR-340-5p | down.mi | up.m |
| RFWD3 | hsa-miR-218-5p | down.mi | up.m |
| RGS1 | hsa-miR-374a-5p | down.mi | up.m |
| RGS17 | hsa-miR-1-3p | down.mi | up.m |
| RHBDF2 | hsa-let-7g-5p | down.mi | up.m |
| RHBDF2 | hsa-let-7f-5p | down.mi | up.m |
| RHBDF2 | hsa-let-7a-5p | down.mi | up.m |
| RHBDF2 | hsa-let-7c-5p | down.mi | up.m |
| RHBDF2 | hsa-let-7b-5p | down.mi | up.m |
| RHBDF2 | hsa-let-7e-5p | down.mi | up.m |
| RHNO1 | hsa-miR-195-3p | down.mi | up.m |
| RHOV | hsa-miR-15b-5p | down.mi | up.m |
| RHOV | hsa-miR-195-5p | down.mi | up.m |
| RHOV | hsa-let-7c-5p | down.mi | up.m |
| RNASEH1 | hsa-miR-340-5p | down.mi | up.m |
| RNF187 | hsa-miR-30b-3p | down.mi | up.m |
| RNF187 | hsa-miR-486-3p | down.mi | up.m |
| RNF187 | hsa-miR-423-5p | down.mi | up.m |
| RNF187 | hsa-miR-99a-5p | down.mi | up.m |
| RNF213 | hsa-miR-1-3p | down.mi | up.m |
| RNPS1 | hsa-miR-15b-5p | down.mi | up.m |
| RNPS1 | hsa-miR-30b-3p | down.mi | up.m |
| RNPS1 | hsa-miR-195-5p | down.mi | up.m |
| RNPS1 | hsa-miR-218-5p | down.mi | up.m |
| RNPS1 | hsa-miR-378a-3p | down.mi | up.m |
| RPL12 | hsa-let-7g-5p | down.mi | up.m |
| RPL12 | hsa-let-7b-5p | down.mi | up.m |
| RPL12 | hsa-let-7e-5p | down.mi | up.m |
| RPL12 | hsa-miR-218-5p | down.mi | up.m |
| RPL22L1 | hsa-miR-3614-5p | down.mi | up.m |
| RPL23A | hsa-miR-423-5p | down.mi | up.m |
| RPL30 | hsa-let-7a-5p | down.mi | up.m |
| RPL36 | hsa-miR-15b-5p | down.mi | up.m |
| RPL36 | hsa-miR-195-5p | down.mi | up.m |
| RPL36A | hsa-miR-99a-5p | down.mi | up.m |
| RPL37 | hsa-miR-378a-5p | down.mi | up.m |
| RPL37 | hsa-miR-338-5p | down.mi | up.m |
| RPL37A | hsa-miR-99a-5p | down.mi | up.m |
| RPL37A | hsa-miR-340-5p | down.mi | up.m |
| RPL38 | hsa-miR-374a-5p | down.mi | up.m |
| RPL7L1 | hsa-miR-144-3p | down.mi | up.m |
| RPL7L1 | hsa-miR-30b-3p | down.mi | up.m |
| RPL7L1 | hsa-miR-30c-2-3p | down.mi | up.m |
| RPL7L1 | hsa-miR-423-5p | down.mi | up.m |
| RPL7L1 | hsa-miR-99a-5p | down.mi | up.m |
| RPL8 | hsa-miR-218-5p | down.mi | up.m |
| RPLP0 | hsa-miR-15b-5p | down.mi | up.m |
| RPLP0 | hsa-miR-30d-5p | down.mi | up.m |
| RPN2 | hsa-let-7e-5p | down.mi | up.m |
| RPN2 | hsa-miR-30d-5p | down.mi | up.m |
| RPN2 | hsa-miR-30a-5p | down.mi | up.m |
| RPP40 | hsa-miR-204-5p | down.mi | up.m |
| RPP40 | hsa-miR-30a-5p | down.mi | up.m |
| RPP40 | hsa-miR-1-3p | down.mi | up.m |
| RPS16 | hsa-miR-27a-5p | down.mi | up.m |
| RPS16 | hsa-let-7b-3p | down.mi | up.m |
| RPS18 | hsa-miR-218-5p | down.mi | up.m |
| RPS19 | hsa-miR-143-3p | down.mi | up.m |
| RPS2 | hsa-miR-423-5p | down.mi | up.m |
| RPS26 | hsa-miR-139-5p | down.mi | up.m |
| RPS26 | hsa-miR-99a-5p | down.mi | up.m |
| RPS5 | hsa-miR-15b-5p | down.mi | up.m |
| RPS5 | hsa-miR-195-5p | down.mi | up.m |
| RPS7 | hsa-miR-378a-3p | down.mi | up.m |
| RPS7 | hsa-miR-378c | down.mi | up.m |
| RRBP1 | hsa-let-7b-5p | down.mi | up.m |
| RRBP1 | hsa-miR-1-3p | down.mi | up.m |
| RRM1 | hsa-let-7e-5p | down.mi | up.m |
| RRM1 | hsa-let-7g-5p | down.mi | up.m |
| RRM1 | hsa-let-7f-5p | down.mi | up.m |
| RRM1 | hsa-miR-1-3p | down.mi | up.m |
| RRM1 | hsa-let-7b-5p | down.mi | up.m |
| RRM1 | hsa-let-7a-5p | down.mi | up.m |
| RRM1 | hsa-let-7c-5p | down.mi | up.m |
| RRM2 | hsa-let-7g-5p | down.mi | up.m |
| RRM2 | hsa-miR-423-5p | down.mi | up.m |
| RRM2 | hsa-let-7c-5p | down.mi | up.m |
| RRM2 | hsa-let-7f-5p | down.mi | up.m |
| RRM2 | hsa-let-7b-5p | down.mi | up.m |
| RRM2 | hsa-let-7e-5p | down.mi | up.m |
| RRM2 | hsa-miR-30a-5p | down.mi | up.m |
| RRM2 | hsa-miR-30d-5p | down.mi | up.m |
| RRM2 | hsa-let-7a-5p | down.mi | up.m |
| RRP1B | hsa-let-7b-5p | down.mi | up.m |
| RRP36 | hsa-miR-30a-5p | down.mi | up.m |
| RRP36 | hsa-miR-1-3p | down.mi | up.m |
| RRS1 | hsa-miR-3614-5p | down.mi | up.m |
| RSL1D1 | hsa-miR-30a-3p | down.mi | up.m |
| RSL1D1 | hsa-let-7e-5p | down.mi | up.m |
| RSL1D1 | hsa-miR-218-5p | down.mi | up.m |
| RUNX2 | hsa-miR-30a-3p | down.mi | up.m |
| RUNX2 | hsa-miR-30d-5p | down.mi | up.m |
| RUNX2 | hsa-miR-221-5p | down.mi | up.m |
| RUNX2 | hsa-miR-30a-5p | down.mi | up.m |
| RUNX2 | hsa-miR-204-5p | down.mi | up.m |
| RUNX2 | hsa-miR-195-5p | down.mi | up.m |
| RUNX2 | hsa-miR-218-5p | down.mi | up.m |
| S100A11 | hsa-miR-1-3p | down.mi | up.m |
| S100A11 | hsa-miR-218-5p | down.mi | up.m |
| S100A16 | hsa-miR-1247-3p | down.mi | up.m |
| S100A16 | hsa-miR-1-3p | down.mi | up.m |
| SAC3D1 | hsa-miR-1-3p | down.mi | up.m |
| SAMD1 | hsa-miR-204-5p | down.mi | up.m |
| SAMD10 | hsa-miR-3154 | down.mi | up.m |
| SAPCD2 | hsa-miR-486-5p | down.mi | up.m |
| SBK1 | hsa-miR-486-3p | down.mi | up.m |
| SBK1 | hsa-miR-423-5p | down.mi | up.m |
| SCAMP3 | hsa-let-7b-5p | down.mi | up.m |
| SCAMP5 | hsa-miR-3154 | down.mi | up.m |
| SCAMP5 | hsa-miR-15b-5p | down.mi | up.m |
| SCAMP5 | hsa-miR-195-5p | down.mi | up.m |
| SCIN | hsa-miR-2110 | down.mi | up.m |
| SCNM1 | hsa-miR-30b-3p | down.mi | up.m |
| SCNM1 | hsa-miR-30c-2-3p | down.mi | up.m |
| SCRN1 | hsa-let-7b-5p | down.mi | up.m |
| SCRN1 | hsa-miR-218-5p | down.mi | up.m |
| SCUBE3 | hsa-miR-30b-3p | down.mi | up.m |
| SCUBE3 | hsa-miR-30c-2-3p | down.mi | up.m |
| SCYL1 | hsa-let-7b-5p | down.mi | up.m |
| SCYL1 | hsa-let-7e-5p | down.mi | up.m |
| SCYL1 | hsa-miR-30a-5p | down.mi | up.m |
| SDC1 | hsa-miR-143-3p | down.mi | up.m |
| SEC11C | hsa-miR-30a-5p | down.mi | up.m |
| SEC11C | hsa-miR-1-3p | down.mi | up.m |
| SEC13 | hsa-miR-99a-5p | down.mi | up.m |
| SEC24A | hsa-miR-15b-5p | down.mi | up.m |
| SEC24A | hsa-miR-374a-5p | down.mi | up.m |
| SEC24A | hsa-miR-30a-5p | down.mi | up.m |
| SEC24A | hsa-let-7b-3p | down.mi | up.m |
| SEC24A | hsa-miR-195-5p | down.mi | up.m |
| SEC24A | hsa-miR-30d-5p | down.mi | up.m |
| SEC24A | hsa-miR-218-5p | down.mi | up.m |
| SEC24D | hsa-miR-605-5p | down.mi | up.m |
| SEC24D | hsa-miR-378a-5p | down.mi | up.m |
| SEC61A1 | hsa-miR-1-3p | down.mi | up.m |
| SEC61A1 | hsa-miR-195-5p | down.mi | up.m |
| SEC61A1 | hsa-miR-30c-2-3p | down.mi | up.m |
| SEC61A1 | hsa-miR-15b-5p | down.mi | up.m |
| SEC61A2 | hsa-miR-30d-5p | down.mi | up.m |
| SEC61A2 | hsa-miR-30a-5p | down.mi | up.m |
| SEL1L3 | hsa-miR-486-5p | down.mi | up.m |
| SEL1L3 | hsa-let-7a-5p | down.mi | up.m |
| SEMA3A | hsa-miR-223-3p | down.mi | up.m |
| SEMA4B | hsa-let-7e-5p | down.mi | up.m |
| SEMA4B | hsa-miR-4732-3p | down.mi | up.m |
| SEMA4C | hsa-let-7b-5p | down.mi | up.m |
| SEMA4C | hsa-let-7c-5p | down.mi | up.m |
| SEMA4C | hsa-let-7f-5p | down.mi | up.m |
| SEMA4C | hsa-miR-138-5p | down.mi | up.m |
| SEMA4C | hsa-let-7e-5p | down.mi | up.m |
| SEMA4C | hsa-let-7g-5p | down.mi | up.m |
| SEMA4C | hsa-let-7a-5p | down.mi | up.m |
| SEMA4C | hsa-miR-4732-3p | down.mi | up.m |
| SERPINB5 | hsa-miR-1-3p | down.mi | up.m |
| SERPINB5 | hsa-miR-30a-5p | down.mi | up.m |
| SERPINE2 | hsa-miR-30a-5p | down.mi | up.m |
| SERPINH1 | hsa-miR-378a-5p | down.mi | up.m |
| SERPINH1 | hsa-miR-133b | down.mi | up.m |
| SERPINH1 | hsa-miR-133a-3p | down.mi | up.m |
| SETDB1 | hsa-let-7c-5p | down.mi | up.m |
| SF3B3 | hsa-miR-30c-2-3p | down.mi | up.m |
| SF3B3 | hsa-miR-30a-3p | down.mi | up.m |
| SF3B3 | hsa-miR-423-5p | down.mi | up.m |
| SF3B3 | hsa-miR-15b-5p | down.mi | up.m |
| SF3B3 | hsa-miR-30b-3p | down.mi | up.m |
| SF3B3 | hsa-miR-30a-5p | down.mi | up.m |
| SF3B4 | hsa-let-7c-5p | down.mi | up.m |
| SFN | hsa-miR-1-3p | down.mi | up.m |
| SFN | hsa-miR-486-3p | down.mi | up.m |
| SFRP2 | hsa-miR-218-5p | down.mi | up.m |
| SFXN1 | hsa-miR-1-3p | down.mi | up.m |
| SFXN1 | hsa-miR-150-3p | down.mi | up.m |
| SFXN1 | hsa-miR-30d-5p | down.mi | up.m |
| SFXN1 | hsa-miR-30a-5p | down.mi | up.m |
| SFXN4 | hsa-miR-144-3p | down.mi | up.m |
| SGPL1 | hsa-miR-139-5p | down.mi | up.m |
| SGPL1 | hsa-miR-4732-3p | down.mi | up.m |
| SGPL1 | hsa-miR-143-3p | down.mi | up.m |
| SGPL1 | hsa-miR-2110 | down.mi | up.m |
| SGPL1 | hsa-miR-218-5p | down.mi | up.m |
| SGPL1 | hsa-miR-204-5p | down.mi | up.m |
| SGPL1 | hsa-miR-144-3p | down.mi | up.m |
| SGPP2 | hsa-miR-1247-3p | down.mi | up.m |
| SGPP2 | hsa-miR-99a-5p | down.mi | up.m |
| SH3GL1 | hsa-miR-30a-5p | down.mi | up.m |
| SH3GL1 | hsa-miR-30d-5p | down.mi | up.m |
| SH3GL1 | hsa-miR-218-5p | down.mi | up.m |
| SH3GL1 | hsa-miR-2110 | down.mi | up.m |
| SH3PXD2B | hsa-miR-1-3p | down.mi | up.m |
| SHCBP1 | hsa-miR-3614-5p | down.mi | up.m |
| SHCBP1 | hsa-miR-340-5p | down.mi | up.m |
| SHMT2 | hsa-let-7a-5p | down.mi | up.m |
| SIGMAR1 | hsa-let-7b-5p | down.mi | up.m |
| SIGMAR1 | hsa-miR-190a-5p | down.mi | up.m |
| SIGMAR1 | hsa-miR-1-3p | down.mi | up.m |
| SIX1 | hsa-miR-4732-3p | down.mi | up.m |
| SIX1 | hsa-miR-204-5p | down.mi | up.m |
| SIX1 | hsa-miR-605-5p | down.mi | up.m |
| SIX4 | hsa-miR-30a-5p | down.mi | up.m |
| SIX4 | hsa-miR-30d-5p | down.mi | up.m |
| SIX4 | hsa-miR-605-5p | down.mi | up.m |
| SIX4 | hsa-miR-144-3p | down.mi | up.m |
| SKA3 | hsa-let-7a-5p | down.mi | up.m |
| SKIV2L | hsa-let-7e-5p | down.mi | up.m |
| SKP2 | hsa-miR-340-5p | down.mi | up.m |
| SKP2 | hsa-miR-30a-5p | down.mi | up.m |
| SLBP | hsa-miR-1-3p | down.mi | up.m |
| SLC12A7 | hsa-let-7a-5p | down.mi | up.m |
| SLC12A7 | hsa-miR-605-5p | down.mi | up.m |
| SLC12A7 | hsa-miR-223-3p | down.mi | up.m |
| SLC12A7 | hsa-let-7f-5p | down.mi | up.m |
| SLC12A7 | hsa-let-7c-5p | down.mi | up.m |
| SLC12A7 | hsa-let-7b-5p | down.mi | up.m |
| SLC12A7 | hsa-let-7e-5p | down.mi | up.m |
| SLC12A7 | hsa-let-7g-5p | down.mi | up.m |
| SLC16A14 | hsa-let-7b-3p | down.mi | up.m |
| SLC16A14 | hsa-miR-340-5p | down.mi | up.m |
| SLC16A9 | hsa-miR-1-3p | down.mi | up.m |
| SLC16A9 | hsa-let-7c-5p | down.mi | up.m |
| SLC16A9 | hsa-let-7e-5p | down.mi | up.m |
| SLC16A9 | hsa-miR-378a-5p | down.mi | up.m |
| SLC16A9 | hsa-let-7g-5p | down.mi | up.m |
| SLC16A9 | hsa-let-7a-5p | down.mi | up.m |
| SLC16A9 | hsa-let-7b-5p | down.mi | up.m |
| SLC16A9 | hsa-let-7f-5p | down.mi | up.m |
| SLC1A4 | hsa-miR-140-3p | down.mi | up.m |
| SLC1A4 | hsa-let-7b-5p | down.mi | up.m |
| SLC1A5 | hsa-miR-30b-3p | down.mi | up.m |
| SLC1A5 | hsa-miR-30c-2-3p | down.mi | up.m |
| SLC1A5 | hsa-miR-378a-5p | down.mi | up.m |
| SLC1A5 | hsa-miR-15b-5p | down.mi | up.m |
| SLC1A5 | hsa-miR-218-5p | down.mi | up.m |
| SLC1A7 | hsa-miR-221-5p | down.mi | up.m |
| SLC20A1 | hsa-let-7c-5p | down.mi | up.m |
| SLC20A1 | hsa-let-7a-5p | down.mi | up.m |
| SLC20A1 | hsa-let-7g-5p | down.mi | up.m |
| SLC20A1 | hsa-let-7b-5p | down.mi | up.m |
| SLC20A1 | hsa-let-7f-5p | down.mi | up.m |
| SLC20A1 | hsa-let-7e-5p | down.mi | up.m |
| SLC25A10 | hsa-miR-1-3p | down.mi | up.m |
| SLC25A13 | hsa-let-7b-5p | down.mi | up.m |
| SLC25A13 | hsa-miR-605-5p | down.mi | up.m |
| SLC25A15 | hsa-miR-6720-3p | down.mi | up.m |
| SLC25A15 | hsa-miR-218-5p | down.mi | up.m |
| SLC25A22 | hsa-miR-15b-5p | down.mi | up.m |
| SLC25A22 | hsa-miR-486-3p | down.mi | up.m |
| SLC25A22 | hsa-miR-195-5p | down.mi | up.m |
| SLC25A22 | hsa-miR-1-3p | down.mi | up.m |
| SLC25A22 | hsa-miR-206 | down.mi | up.m |
| SLC25A37 | hsa-miR-30a-3p | down.mi | up.m |
| SLC27A2 | hsa-let-7b-5p | down.mi | up.m |
| SLC27A2 | hsa-miR-204-5p | down.mi | up.m |
| SLC27A4 | hsa-miR-1-3p | down.mi | up.m |
| SLC29A4 | hsa-miR-30c-2-3p | down.mi | up.m |
| SLC29A4 | hsa-miR-30b-3p | down.mi | up.m |
| SLC2A5 | hsa-miR-140-3p | down.mi | up.m |
| SLC2A5 | hsa-miR-1247-3p | down.mi | up.m |
| SLC2A5 | hsa-miR-423-5p | down.mi | up.m |
| SLC30A1 | hsa-let-7b-5p | down.mi | up.m |
| SLC35F5 | hsa-miR-144-3p | down.mi | up.m |
| SLC35F5 | hsa-miR-378a-5p | down.mi | up.m |
| SLC35F5 | hsa-miR-1247-3p | down.mi | up.m |
| SLC35F5 | hsa-miR-218-5p | down.mi | up.m |
| SLC38A7 | hsa-let-7a-5p | down.mi | up.m |
| SLC38A7 | hsa-let-7g-5p | down.mi | up.m |
| SLC38A7 | hsa-miR-30a-5p | down.mi | up.m |
| SLC38A7 | hsa-let-7c-5p | down.mi | up.m |
| SLC38A7 | hsa-let-7e-5p | down.mi | up.m |
| SLC38A7 | hsa-miR-204-5p | down.mi | up.m |
| SLC38A7 | hsa-miR-30d-5p | down.mi | up.m |
| SLC38A7 | hsa-miR-30c-2-3p | down.mi | up.m |
| SLC38A7 | hsa-miR-516a-5p | down.mi | up.m |
| SLC38A7 | hsa-let-7f-5p | down.mi | up.m |
| SLC38A7 | hsa-let-7b-5p | down.mi | up.m |
| SLC38A7 | hsa-miR-30b-3p | down.mi | up.m |
| SLC39A10 | hsa-miR-140-3p | down.mi | up.m |
| SLC39A10 | hsa-miR-221-5p | down.mi | up.m |
| SLC39A10 | hsa-miR-218-5p | down.mi | up.m |
| SLC39A11 | hsa-miR-423-5p | down.mi | up.m |
| SLC39A11 | hsa-miR-204-5p | down.mi | up.m |
| SLC39A14 | hsa-miR-1-3p | down.mi | up.m |
| SLC39A14 | hsa-miR-15b-5p | down.mi | up.m |
| SLC39A6 | hsa-miR-139-5p | down.mi | up.m |
| SLC39A6 | hsa-miR-144-3p | down.mi | up.m |
| SLC39A7 | hsa-miR-340-5p | down.mi | up.m |
| SLC41A2 | hsa-miR-3614-5p | down.mi | up.m |
| SLC41A2 | hsa-miR-34b-3p | down.mi | up.m |
| SLC5A6 | hsa-miR-139-3p | down.mi | up.m |
| SLC5A6 | hsa-let-7f-5p | down.mi | up.m |
| SLC5A6 | hsa-let-7b-5p | down.mi | up.m |
| SLC5A6 | hsa-let-7e-5p | down.mi | up.m |
| SLC5A6 | hsa-let-7g-5p | down.mi | up.m |
| SLC5A6 | hsa-miR-423-5p | down.mi | up.m |
| SLC5A6 | hsa-let-7a-5p | down.mi | up.m |
| SLC5A6 | hsa-let-7c-5p | down.mi | up.m |
| SLC6A8 | hsa-miR-340-5p | down.mi | up.m |
| SLC7A11 | hsa-miR-30a-5p | down.mi | up.m |
| SLC7A11 | hsa-miR-1247-3p | down.mi | up.m |
| SLC7A11 | hsa-miR-218-5p | down.mi | up.m |
| SLC7A11 | hsa-miR-340-5p | down.mi | up.m |
| SLC7A5 | hsa-miR-3154 | down.mi | up.m |
| SLC7A5 | hsa-miR-184 | down.mi | up.m |
| SLC7A5 | hsa-miR-223-3p | down.mi | up.m |
| SLC7A5 | hsa-miR-30b-3p | down.mi | up.m |
| SLC7A5 | hsa-miR-15b-5p | down.mi | up.m |
| SLC7A5 | hsa-miR-195-5p | down.mi | up.m |
| SLC7A5 | hsa-miR-140-3p | down.mi | up.m |
| SLC7A5 | hsa-miR-30a-5p | down.mi | up.m |
| SLC7A5 | hsa-miR-598-3p | down.mi | up.m |
| SLC7A5 | hsa-miR-30d-5p | down.mi | up.m |
| SLC9A3R1 | hsa-miR-423-5p | down.mi | up.m |
| SLC9A3R1 | hsa-let-7b-5p | down.mi | up.m |
| SMARCB1 | hsa-let-7b-5p | down.mi | up.m |
| SMARCB1 | hsa-miR-1-3p | down.mi | up.m |
| SMARCB1 | hsa-miR-206 | down.mi | up.m |
| SMARCB1 | hsa-miR-218-5p | down.mi | up.m |
| SMARCC1 | hsa-let-7b-5p | down.mi | up.m |
| SMARCC1 | hsa-let-7f-5p | down.mi | up.m |
| SMARCC1 | hsa-miR-1-3p | down.mi | up.m |
| SMARCC1 | hsa-miR-34b-3p | down.mi | up.m |
| SMARCC1 | hsa-let-7c-5p | down.mi | up.m |
| SMC4 | hsa-miR-1-3p | down.mi | up.m |
| SMG7 | hsa-let-7b-5p | down.mi | up.m |
| SMG7 | hsa-let-7a-5p | down.mi | up.m |
| SMKR1 | hsa-miR-206 | down.mi | up.m |
| SMKR1 | hsa-miR-1-3p | down.mi | up.m |
| SMOC1 | hsa-miR-204-5p | down.mi | up.m |
| SMPDL3B | hsa-miR-99a-5p | down.mi | up.m |
| SMPDL3B | hsa-let-7a-5p | down.mi | up.m |
| SMS | hsa-miR-30b-3p | down.mi | up.m |
| SMS | hsa-miR-490-3p | down.mi | up.m |
| SNAPIN | hsa-miR-218-5p | down.mi | up.m |
| SNAPIN | hsa-miR-1-3p | down.mi | up.m |
| SND1 | hsa-miR-184 | down.mi | up.m |
| SNRNP200 | hsa-miR-99a-5p | down.mi | up.m |
| SNRPA | hsa-let-7b-5p | down.mi | up.m |
| SNRPA1 | hsa-miR-374a-5p | down.mi | up.m |
| SNRPC | hsa-let-7f-5p | down.mi | up.m |
| SNRPC | hsa-let-7a-5p | down.mi | up.m |
| SNRPD1 | hsa-miR-1247-3p | down.mi | up.m |
| SNRPD1 | hsa-miR-30b-3p | down.mi | up.m |
| SNRPD1 | hsa-miR-30c-2-3p | down.mi | up.m |
| SNRPE | hsa-let-7b-5p | down.mi | up.m |
| SNX9 | hsa-miR-204-5p | down.mi | up.m |
| SOD1 | hsa-miR-378a-3p | down.mi | up.m |
| SOD1 | hsa-miR-206 | down.mi | up.m |
| SORD | hsa-miR-378a-5p | down.mi | up.m |
| SOX12 | hsa-miR-423-5p | down.mi | up.m |
| SOX12 | hsa-miR-30d-5p | down.mi | up.m |
| SOX12 | hsa-miR-30a-5p | down.mi | up.m |
| SOX2 | hsa-miR-340-5p | down.mi | up.m |
| SOX2 | hsa-miR-34b-3p | down.mi | up.m |
| SOX4 | hsa-miR-138-5p | down.mi | up.m |
| SOX4 | hsa-miR-139-3p | down.mi | up.m |
| SOX4 | hsa-miR-30d-5p | down.mi | up.m |
| SOX4 | hsa-miR-30a-5p | down.mi | up.m |
| SOX4 | hsa-miR-340-5p | down.mi | up.m |
| SOX4 | hsa-miR-204-5p | down.mi | up.m |
| SOX4 | hsa-miR-133a-3p | down.mi | up.m |
| SOX9 | hsa-miR-138-5p | down.mi | up.m |
| SOX9 | hsa-miR-338-5p | down.mi | up.m |
| SOX9 | hsa-miR-1247-5p | down.mi | up.m |
| SOX9 | hsa-miR-1-3p | down.mi | up.m |
| SOX9 | hsa-let-7b-5p | down.mi | up.m |
| SOX9 | hsa-miR-206 | down.mi | up.m |
| SPATS2 | hsa-miR-3154 | down.mi | up.m |
| SPATS2 | hsa-let-7a-5p | down.mi | up.m |
| SPC24 | hsa-miR-1-3p | down.mi | up.m |
| SPC25 | hsa-miR-374a-5p | down.mi | up.m |
| SPDEF | hsa-miR-204-5p | down.mi | up.m |
| SPDEF | hsa-let-7c-5p | down.mi | up.m |
| SPINK1 | hsa-miR-1-3p | down.mi | up.m |
| SPIRE2 | hsa-miR-1247-3p | down.mi | up.m |
| SPIRE2 | hsa-miR-378a-5p | down.mi | up.m |
| SPR | hsa-let-7b-5p | down.mi | up.m |
| SPSB1 | hsa-miR-30c-2-3p | down.mi | up.m |
| SPTBN2 | hsa-let-7b-5p | down.mi | up.m |
| SRCIN1 | hsa-miR-374a-5p | down.mi | up.m |
| SRCIN1 | hsa-miR-486-3p | down.mi | up.m |
| SRD5A1 | hsa-miR-30c-2-3p | down.mi | up.m |
| SRD5A3 | hsa-miR-30c-2-3p | down.mi | up.m |
| SRM | hsa-miR-423-5p | down.mi | up.m |
| SRM | hsa-miR-490-3p | down.mi | up.m |
| SRP54 | hsa-miR-423-5p | down.mi | up.m |
| SRPK1 | hsa-miR-15b-5p | down.mi | up.m |
| SRPK1 | hsa-miR-195-5p | down.mi | up.m |
| SRPK1 | hsa-miR-139-5p | down.mi | up.m |
| SRPK1 | hsa-miR-99a-5p | down.mi | up.m |
| SRPRB | hsa-miR-195-5p | down.mi | up.m |
| SRPRB | hsa-miR-15b-5p | down.mi | up.m |
| SRPRB | hsa-miR-6892-5p | down.mi | up.m |
| SRRT | hsa-miR-218-5p | down.mi | up.m |
| SRSF9 | hsa-miR-1-3p | down.mi | up.m |
| SRSF9 | hsa-miR-218-5p | down.mi | up.m |
| SRSF9 | hsa-miR-30d-5p | down.mi | up.m |
| SSB | hsa-let-7e-5p | down.mi | up.m |
| SSR1 | hsa-miR-1-3p | down.mi | up.m |
| SSR1 | hsa-let-7b-5p | down.mi | up.m |
| SSR1 | hsa-miR-30b-3p | down.mi | up.m |
| SSR1 | hsa-miR-30c-2-3p | down.mi | up.m |
| SSR1 | hsa-miR-218-5p | down.mi | up.m |
| SSR3 | hsa-miR-125b-2-3p | down.mi | up.m |
| SSR3 | hsa-miR-30c-2-3p | down.mi | up.m |
| SSR3 | hsa-miR-30b-3p | down.mi | up.m |
| SSRP1 | hsa-miR-15b-5p | down.mi | up.m |
| SSRP1 | hsa-miR-195-5p | down.mi | up.m |
| SSSCA1 | hsa-miR-221-5p | down.mi | up.m |
| ST6GALNAC1 | hsa-miR-605-5p | down.mi | up.m |
| STAT1 | hsa-miR-605-5p | down.mi | up.m |
| STAT1 | hsa-miR-223-3p | down.mi | up.m |
| STAT2 | hsa-let-7b-5p | down.mi | up.m |
| STAT2 | hsa-miR-221-5p | down.mi | up.m |
| STAT2 | hsa-let-7c-5p | down.mi | up.m |
| STAT2 | hsa-let-7e-5p | down.mi | up.m |
| STAT2 | hsa-let-7a-5p | down.mi | up.m |
| STAT2 | hsa-let-7f-5p | down.mi | up.m |
| STAT2 | hsa-let-7g-5p | down.mi | up.m |
| STEAP2 | hsa-miR-1247-3p | down.mi | up.m |
| STEAP3 | hsa-let-7b-5p | down.mi | up.m |
| STIP1 | hsa-let-7b-5p | down.mi | up.m |
| STIP1 | hsa-miR-218-5p | down.mi | up.m |
| STIP1 | hsa-miR-486-3p | down.mi | up.m |
| STK38 | hsa-miR-30c-2-3p | down.mi | up.m |
| STK38 | hsa-miR-195-5p | down.mi | up.m |
| STK38 | hsa-miR-15b-5p | down.mi | up.m |
| STK38 | hsa-miR-30b-3p | down.mi | up.m |
| STK38 | hsa-let-7b-5p | down.mi | up.m |
| STK38 | hsa-miR-145-3p | down.mi | up.m |
| STMN1 | hsa-miR-223-3p | down.mi | up.m |
| STMN1 | hsa-miR-30a-3p | down.mi | up.m |
| STMN1 | hsa-miR-30a-5p | down.mi | up.m |
| STRBP | hsa-miR-34b-3p | down.mi | up.m |
| STRBP | hsa-miR-34c-3p | down.mi | up.m |
| STRBP | hsa-miR-204-5p | down.mi | up.m |
| STRIP2 | hsa-miR-423-5p | down.mi | up.m |
| STRIP2 | hsa-miR-30c-2-3p | down.mi | up.m |
| STRIP2 | hsa-miR-30b-3p | down.mi | up.m |
| STX16 | hsa-miR-30a-5p | down.mi | up.m |
| STX16 | hsa-miR-30d-5p | down.mi | up.m |
| STX16 | hsa-miR-144-3p | down.mi | up.m |
| STX16 | hsa-miR-374a-5p | down.mi | up.m |
| STX1A | hsa-miR-3154 | down.mi | up.m |
| STX6 | hsa-miR-206 | down.mi | up.m |
| STX6 | hsa-miR-1-3p | down.mi | up.m |
| SURF4 | hsa-let-7g-5p | down.mi | up.m |
| SURF4 | hsa-let-7a-5p | down.mi | up.m |
| SURF4 | hsa-let-7e-5p | down.mi | up.m |
| SURF4 | hsa-let-7b-5p | down.mi | up.m |
| SURF4 | hsa-let-7c-5p | down.mi | up.m |
| SURF4 | hsa-let-7f-5p | down.mi | up.m |
| SUSD4 | hsa-miR-218-5p | down.mi | up.m |
| SYNJ2 | hsa-miR-30b-3p | down.mi | up.m |
| SYNJ2 | hsa-miR-30c-2-3p | down.mi | up.m |
| SYT7 | hsa-miR-139-3p | down.mi | up.m |
| SYT7 | hsa-miR-3154 | down.mi | up.m |
| SYT7 | hsa-miR-143-3p | down.mi | up.m |
| SYTL2 | hsa-miR-1-3p | down.mi | up.m |
| SYVN1 | hsa-let-7a-5p | down.mi | up.m |
| TACO1 | hsa-miR-30b-3p | down.mi | up.m |
| TACO1 | hsa-miR-184 | down.mi | up.m |
| TACO1 | hsa-miR-30c-2-3p | down.mi | up.m |
| TAF1D | hsa-miR-143-3p | down.mi | up.m |
| TAF1D | hsa-miR-139-5p | down.mi | up.m |
| TAF1D | hsa-miR-3614-5p | down.mi | up.m |
| TALDO1 | hsa-miR-378a-5p | down.mi | up.m |
| TARBP2 | hsa-miR-195-5p | down.mi | up.m |
| TARBP2 | hsa-miR-15b-5p | down.mi | up.m |
| TARS | hsa-let-7a-5p | down.mi | up.m |
| TARS2 | hsa-miR-1247-3p | down.mi | up.m |
| TAZ | hsa-let-7c-5p | down.mi | up.m |
| TBL1XR1 | hsa-miR-3154 | down.mi | up.m |
| TBL1XR1 | hsa-miR-195-5p | down.mi | up.m |
| TBL1XR1 | hsa-miR-15b-5p | down.mi | up.m |
| TBRG4 | hsa-let-7b-5p | down.mi | up.m |
| TBRG4 | hsa-miR-15b-5p | down.mi | up.m |
| TCERG1 | hsa-let-7a-5p | down.mi | up.m |
| TCF3 | hsa-miR-15b-5p | down.mi | up.m |
| TCF3 | hsa-miR-195-5p | down.mi | up.m |
| TCOF1 | hsa-miR-218-5p | down.mi | up.m |
| TCOF1 | hsa-let-7b-5p | down.mi | up.m |
| TDRKH | hsa-miR-423-5p | down.mi | up.m |
| TELO2 | hsa-miR-1-3p | down.mi | up.m |
| TELO2 | hsa-miR-30a-5p | down.mi | up.m |
| TFAP2A | hsa-miR-195-5p | down.mi | up.m |
| TFAP2A | hsa-miR-15b-5p | down.mi | up.m |
| TFAP2A | hsa-miR-338-5p | down.mi | up.m |
| TFAP4 | hsa-miR-144-3p | down.mi | up.m |
| TFAP4 | hsa-miR-30c-2-3p | down.mi | up.m |
| TFDP1 | hsa-miR-374a-5p | down.mi | up.m |
| TFDP1 | hsa-miR-30a-5p | down.mi | up.m |
| TFDP1 | hsa-miR-30d-5p | down.mi | up.m |
| TFF1 | hsa-miR-218-5p | down.mi | up.m |
| TFF1 | hsa-miR-423-5p | down.mi | up.m |
| TGFA | hsa-miR-30a-5p | down.mi | up.m |
| TGFA | hsa-miR-490-3p | down.mi | up.m |
| TGFA | hsa-miR-30d-5p | down.mi | up.m |
| THAP8 | hsa-miR-6788-3p | down.mi | up.m |
| THBS2 | hsa-miR-133a-3p | down.mi | up.m |
| THEM6 | hsa-let-7f-5p | down.mi | up.m |
| THEM6 | hsa-let-7e-5p | down.mi | up.m |
| THEM6 | hsa-let-7g-5p | down.mi | up.m |
| THEM6 | hsa-let-7b-5p | down.mi | up.m |
| THEM6 | hsa-let-7a-5p | down.mi | up.m |
| THEM6 | hsa-let-7c-5p | down.mi | up.m |
| THY1 | hsa-miR-1-3p | down.mi | up.m |
| THY1 | hsa-miR-486-3p | down.mi | up.m |
| THY1 | hsa-miR-139-3p | down.mi | up.m |
| TIGD1 | hsa-miR-378a-3p | down.mi | up.m |
| TIGD2 | hsa-miR-340-5p | down.mi | up.m |
| TIMM10 | hsa-miR-30a-5p | down.mi | up.m |
| TIMM10 | hsa-miR-378a-5p | down.mi | up.m |
| TIMM17A | hsa-miR-30a-5p | down.mi | up.m |
| TIMM23 | hsa-let-7b-5p | down.mi | up.m |
| TIMM50 | hsa-let-7b-5p | down.mi | up.m |
| TIMM50 | hsa-miR-30c-2-3p | down.mi | up.m |
| TIMM50 | hsa-miR-378a-5p | down.mi | up.m |
| TIMM50 | hsa-miR-30b-3p | down.mi | up.m |
| TIMM50 | hsa-let-7e-5p | down.mi | up.m |
| TIMM50 | hsa-miR-486-3p | down.mi | up.m |
| TIMM8A | hsa-let-7b-5p | down.mi | up.m |
| TIMM8A | hsa-miR-30a-5p | down.mi | up.m |
| TIMM8A | hsa-miR-143-3p | down.mi | up.m |
| TIMM8A | hsa-miR-378a-5p | down.mi | up.m |
| TIMM8A | hsa-miR-6720-3p | down.mi | up.m |
| TIMM9 | hsa-let-7b-5p | down.mi | up.m |
| TIMM9 | hsa-miR-30a-5p | down.mi | up.m |
| TJP3 | hsa-miR-184 | down.mi | up.m |
| TKT | hsa-miR-1-3p | down.mi | up.m |
| TKT | hsa-miR-206 | down.mi | up.m |
| TM4SF5 | hsa-miR-378a-5p | down.mi | up.m |
| TM4SF5 | hsa-miR-1247-3p | down.mi | up.m |
| TM9SF4 | hsa-miR-184 | down.mi | up.m |
| TM9SF4 | hsa-miR-125b-2-3p | down.mi | up.m |
| TMCO1 | hsa-miR-30a-5p | down.mi | up.m |
| TMCO1 | hsa-miR-3614-5p | down.mi | up.m |
| TMCO1 | hsa-miR-34b-3p | down.mi | up.m |
| TMED2 | hsa-miR-30d-5p | down.mi | up.m |
| TMED2 | hsa-miR-30a-5p | down.mi | up.m |
| TMED3 | hsa-miR-30a-5p | down.mi | up.m |
| TMED4 | hsa-let-7e-5p | down.mi | up.m |
| TMED4 | hsa-let-7f-5p | down.mi | up.m |
| TMED4 | hsa-let-7g-5p | down.mi | up.m |
| TMED4 | hsa-miR-1247-3p | down.mi | up.m |
| TMED4 | hsa-let-7b-5p | down.mi | up.m |
| TMED4 | hsa-miR-2110 | down.mi | up.m |
| TMED4 | hsa-let-7c-5p | down.mi | up.m |
| TMED4 | hsa-let-7a-5p | down.mi | up.m |
| TMEM106B | hsa-miR-145-3p | down.mi | up.m |
| TMEM106B | hsa-miR-1247-3p | down.mi | up.m |
| TMEM106C | hsa-miR-1-3p | down.mi | up.m |
| TMEM14A | hsa-miR-218-5p | down.mi | up.m |
| TMEM156 | hsa-miR-204-5p | down.mi | up.m |
| TMEM165 | hsa-let-7a-5p | down.mi | up.m |
| TMEM165 | hsa-let-7c-5p | down.mi | up.m |
| TMEM214 | hsa-miR-143-3p | down.mi | up.m |
| TMEM216 | hsa-miR-34b-3p | down.mi | up.m |
| TMEM216 | hsa-miR-3614-5p | down.mi | up.m |
| TMEM241 | hsa-miR-374a-5p | down.mi | up.m |
| TMEM241 | hsa-let-7a-5p | down.mi | up.m |
| TMEM263 | hsa-miR-340-5p | down.mi | up.m |
| TMEM263 | hsa-miR-584-5p | down.mi | up.m |
| TMEM63C | hsa-miR-30b-3p | down.mi | up.m |
| TMEM69 | hsa-miR-2110 | down.mi | up.m |
| TMEM69 | hsa-miR-15b-5p | down.mi | up.m |
| TMEM69 | hsa-miR-195-5p | down.mi | up.m |
| TMEM81 | hsa-miR-125b-2-3p | down.mi | up.m |
| TMEM81 | hsa-miR-218-1-3p | down.mi | up.m |
| TMPRSS4 | hsa-miR-3614-5p | down.mi | up.m |
| TMPRSS4 | hsa-miR-34b-3p | down.mi | up.m |
| TMUB1 | hsa-miR-423-5p | down.mi | up.m |
| TNFRSF10A | hsa-miR-340-5p | down.mi | up.m |
| TNFRSF13C | hsa-miR-1247-3p | down.mi | up.m |
| TNFRSF13C | hsa-miR-30c-2-3p | down.mi | up.m |
| TNFRSF13C | hsa-miR-30b-3p | down.mi | up.m |
| TNFRSF13C | hsa-miR-184 | down.mi | up.m |
| TNFRSF21 | hsa-miR-584-5p | down.mi | up.m |
| TNS4 | hsa-miR-30b-3p | down.mi | up.m |
| TNS4 | hsa-miR-30c-2-3p | down.mi | up.m |
| TNS4 | hsa-miR-1-3p | down.mi | up.m |
| TOMM40 | hsa-miR-221-5p | down.mi | up.m |
| TOMM40L | hsa-let-7e-5p | down.mi | up.m |
| TOMM40L | hsa-let-7a-5p | down.mi | up.m |
| TOMM40L | hsa-let-7c-5p | down.mi | up.m |
| TOMM40L | hsa-let-7b-5p | down.mi | up.m |
| TOMM40L | hsa-let-7f-5p | down.mi | up.m |
| TOMM40L | hsa-let-7g-5p | down.mi | up.m |
| TOP2A | hsa-miR-218-5p | down.mi | up.m |
| TOX3 | hsa-let-7b-5p | down.mi | up.m |
| TPBG | hsa-miR-30a-5p | down.mi | up.m |
| TPBG | hsa-let-7b-5p | down.mi | up.m |
| TPBG | hsa-miR-516a-5p | down.mi | up.m |
| TPD52 | hsa-miR-139-5p | down.mi | up.m |
| TPD52 | hsa-miR-218-5p | down.mi | up.m |
| TPD52 | hsa-miR-15b-5p | down.mi | up.m |
| TPD52L2 | hsa-let-7b-5p | down.mi | up.m |
| TPD52L2 | hsa-miR-1-3p | down.mi | up.m |
| TPI1 | hsa-miR-195-5p | down.mi | up.m |
| TPI1 | hsa-miR-140-3p | down.mi | up.m |
| TPI1 | hsa-miR-30a-5p | down.mi | up.m |
| TPI1 | hsa-miR-30c-2-3p | down.mi | up.m |
| TRABD | hsa-let-7b-5p | down.mi | up.m |
| TRADD | hsa-miR-30c-2-3p | down.mi | up.m |
| TRAF1 | hsa-miR-378a-3p | down.mi | up.m |
| TRAF4 | hsa-miR-605-5p | down.mi | up.m |
| TRAF5 | hsa-miR-3614-5p | down.mi | up.m |
| TRAF7 | hsa-miR-423-5p | down.mi | up.m |
| TRAF7 | hsa-let-7a-5p | down.mi | up.m |
| TRIM2 | hsa-miR-374a-5p | down.mi | up.m |
| TRIM2 | hsa-miR-1258 | down.mi | up.m |
| TRIM2 | hsa-miR-1-3p | down.mi | up.m |
| TRIM28 | hsa-miR-423-5p | down.mi | up.m |
| TRIM28 | hsa-miR-30a-5p | down.mi | up.m |
| TRIM28 | hsa-let-7b-5p | down.mi | up.m |
| TRIM31 | hsa-miR-140-3p | down.mi | up.m |
| TRIM59 | hsa-miR-340-5p | down.mi | up.m |
| TRIM59 | hsa-miR-1247-3p | down.mi | up.m |
| TRIO | hsa-miR-30a-5p | down.mi | up.m |
| TRIO | hsa-let-7a-5p | down.mi | up.m |
| TRMT1 | hsa-let-7b-5p | down.mi | up.m |
| TRMU | hsa-miR-30b-3p | down.mi | up.m |
| TRRAP | hsa-miR-30a-5p | down.mi | up.m |
| TRRAP | hsa-let-7e-5p | down.mi | up.m |
| TRRAP | hsa-let-7c-5p | down.mi | up.m |
| TRRAP | hsa-let-7a-5p | down.mi | up.m |
| TRUB2 | hsa-let-7b-5p | down.mi | up.m |
| TRUB2 | hsa-miR-3614-5p | down.mi | up.m |
| TRUB2 | hsa-miR-30b-3p | down.mi | up.m |
| TRUB2 | hsa-miR-34b-3p | down.mi | up.m |
| TRUB2 | hsa-miR-30c-2-3p | down.mi | up.m |
| TSEN34 | hsa-miR-490-3p | down.mi | up.m |
| TSKU | hsa-miR-3154 | down.mi | up.m |
| TSKU | hsa-miR-30b-3p | down.mi | up.m |
| TSPAN6 | hsa-miR-144-3p | down.mi | up.m |
| TTC22 | hsa-let-7a-5p | down.mi | up.m |
| TTC39C | hsa-miR-140-3p | down.mi | up.m |
| TTC9C | hsa-miR-490-3p | down.mi | up.m |
| TTC9C | hsa-miR-30c-2-3p | down.mi | up.m |
| TTC9C | hsa-miR-30b-3p | down.mi | up.m |
| TTC9C | hsa-miR-1247-3p | down.mi | up.m |
| TTC9C | hsa-let-7b-5p | down.mi | up.m |
| TTI1 | hsa-miR-218-5p | down.mi | up.m |
| TTLL12 | hsa-let-7b-5p | down.mi | up.m |
| TTLL12 | hsa-miR-140-3p | down.mi | up.m |
| TTLL12 | hsa-let-7e-5p | down.mi | up.m |
| TTLL12 | hsa-miR-423-5p | down.mi | up.m |
| TTPAL | hsa-miR-378a-5p | down.mi | up.m |
| TTYH3 | hsa-miR-143-3p | down.mi | up.m |
| TTYH3 | hsa-miR-218-5p | down.mi | up.m |
| TUBA1C | hsa-let-7b-5p | down.mi | up.m |
| TUBB | hsa-miR-6892-5p | down.mi | up.m |
| TUBB | hsa-miR-486-3p | down.mi | up.m |
| TUBB | hsa-miR-195-5p | down.mi | up.m |
| TUBB | hsa-let-7b-5p | down.mi | up.m |
| TUBB | hsa-miR-15b-5p | down.mi | up.m |
| TUBB | hsa-miR-423-5p | down.mi | up.m |
| TUBB2A | hsa-miR-27a-5p | down.mi | up.m |
| TUBB2A | hsa-miR-195-5p | down.mi | up.m |
| TUBB2A | hsa-miR-6892-5p | down.mi | up.m |
| TUBB2A | hsa-miR-486-3p | down.mi | up.m |
| TUBB2A | hsa-miR-30a-3p | down.mi | up.m |
| TUBB2A | hsa-let-7a-5p | down.mi | up.m |
| TUBB2A | hsa-let-7b-5p | down.mi | up.m |
| TUBB2A | hsa-miR-195-3p | down.mi | up.m |
| TUBB2A | hsa-let-7c-5p | down.mi | up.m |
| TUBB2A | hsa-let-7e-5p | down.mi | up.m |
| TUBB2A | hsa-miR-143-3p | down.mi | up.m |
| TUBB2A | hsa-let-7f-5p | down.mi | up.m |
| TUBB2A | hsa-miR-15b-5p | down.mi | up.m |
| TUBB2A | hsa-let-7g-5p | down.mi | up.m |
| TUBB2B | hsa-miR-1-3p | down.mi | up.m |
| TUBB3 | hsa-miR-340-5p | down.mi | up.m |
| TUBB3 | hsa-miR-30a-5p | down.mi | up.m |
| TUBG1 | hsa-miR-99a-5p | down.mi | up.m |
| TUFT1 | hsa-miR-195-5p | down.mi | up.m |
| TUT1 | hsa-let-7b-5p | down.mi | up.m |
| TUT1 | hsa-let-7c-5p | down.mi | up.m |
| TWF1 | hsa-miR-1-3p | down.mi | up.m |
| TWF1 | hsa-miR-30a-5p | down.mi | up.m |
| TWF1 | hsa-miR-30d-5p | down.mi | up.m |
| TWF1 | hsa-miR-206 | down.mi | up.m |
| TWF1 | hsa-miR-223-3p | down.mi | up.m |
| TWIST1 | hsa-miR-1-3p | down.mi | up.m |
| TWIST1 | hsa-miR-206 | down.mi | up.m |
| TXNDC5 | hsa-miR-30a-5p | down.mi | up.m |
| TXNDC5 | hsa-miR-30d-5p | down.mi | up.m |
| TXNRD1 | hsa-let-7e-5p | down.mi | up.m |
| TYMS | hsa-miR-99a-5p | down.mi | up.m |
| TYMS | hsa-let-7b-5p | down.mi | up.m |
| U2AF2 | hsa-miR-423-5p | down.mi | up.m |
| U2AF2 | hsa-miR-486-3p | down.mi | up.m |
| U2SURP | hsa-miR-27a-5p | down.mi | up.m |
| U2SURP | hsa-miR-340-5p | down.mi | up.m |
| U2SURP | hsa-miR-195-5p | down.mi | up.m |
| U2SURP | hsa-miR-15b-5p | down.mi | up.m |
| UAP1 | hsa-miR-30a-5p | down.mi | up.m |
| UBAC2 | hsa-miR-1-3p | down.mi | up.m |
| UBAP2L | hsa-let-7a-5p | down.mi | up.m |
| UBAP2L | hsa-let-7b-5p | down.mi | up.m |
| UBAP2L | hsa-let-7f-5p | down.mi | up.m |
| UBAP2L | hsa-let-7e-5p | down.mi | up.m |
| UBE2C | hsa-miR-140-3p | down.mi | up.m |
| UBE2Q1 | hsa-miR-15b-5p | down.mi | up.m |
| UBE2Q1 | hsa-miR-195-5p | down.mi | up.m |
| UBE2Q1 | hsa-let-7a-5p | down.mi | up.m |
| UBE2Q1 | hsa-let-7b-5p | down.mi | up.m |
| UBE2S | hsa-miR-486-5p | down.mi | up.m |
| UBE2S | hsa-miR-7704 | down.mi | up.m |
| UBFD1 | hsa-miR-423-5p | down.mi | up.m |
| UBQLN4 | hsa-miR-7704 | down.mi | up.m |
| UCHL1 | hsa-miR-218-5p | down.mi | up.m |
| UCK2 | hsa-miR-3154 | down.mi | up.m |
| UCK2 | hsa-miR-218-5p | down.mi | up.m |
| UCK2 | hsa-let-7b-5p | down.mi | up.m |
| UCK2 | hsa-miR-140-3p | down.mi | up.m |
| UCK2 | hsa-miR-143-3p | down.mi | up.m |
| UGDH | hsa-miR-221-5p | down.mi | up.m |
| UGDH | hsa-miR-30a-5p | down.mi | up.m |
| UGGT1 | hsa-miR-1-3p | down.mi | up.m |
| UGGT1 | hsa-let-7e-5p | down.mi | up.m |
| UGGT1 | hsa-miR-1247-3p | down.mi | up.m |
| UGGT1 | hsa-miR-30b-3p | down.mi | up.m |
| UGGT1 | hsa-miR-30c-2-3p | down.mi | up.m |
| UGT8 | hsa-let-7b-5p | down.mi | up.m |
| UGT8 | hsa-miR-378a-5p | down.mi | up.m |
| UGT8 | hsa-miR-1-3p | down.mi | up.m |
| UHMK1 | hsa-miR-1-3p | down.mi | up.m |
| UHMK1 | hsa-miR-139-5p | down.mi | up.m |
| UHMK1 | hsa-miR-340-5p | down.mi | up.m |
| UHRF1 | hsa-let-7a-5p | down.mi | up.m |
| UHRF1 | hsa-let-7b-5p | down.mi | up.m |
| UHRF1 | hsa-miR-1-3p | down.mi | up.m |
| ULK1 | hsa-miR-423-5p | down.mi | up.m |
| UQCRQ | hsa-miR-30c-2-3p | down.mi | up.m |
| UQCRQ | hsa-miR-30b-3p | down.mi | up.m |
| URB2 | hsa-miR-15b-5p | down.mi | up.m |
| USP14 | hsa-miR-204-5p | down.mi | up.m |
| USP14 | hsa-let-7a-5p | down.mi | up.m |
| USP14 | hsa-miR-140-3p | down.mi | up.m |
| USP14 | hsa-let-7b-5p | down.mi | up.m |
| USP28 | hsa-miR-340-5p | down.mi | up.m |
| UTP14A | hsa-miR-204-5p | down.mi | up.m |
| UTP6 | hsa-let-7b-5p | down.mi | up.m |
| UTP6 | hsa-miR-30a-5p | down.mi | up.m |
| VANGL1 | hsa-miR-6892-5p | down.mi | up.m |
| VARS | hsa-let-7e-5p | down.mi | up.m |
| VCAN | hsa-miR-218-5p | down.mi | up.m |
| VDR | hsa-miR-7704 | down.mi | up.m |
| VDR | hsa-let-7a-5p | down.mi | up.m |
| VKORC1 | hsa-miR-133a-3p | down.mi | up.m |
| VMP1 | hsa-miR-1-3p | down.mi | up.m |
| VMP1 | hsa-miR-150-3p | down.mi | up.m |
| VPS45 | hsa-miR-99a-5p | down.mi | up.m |
| VPS72 | hsa-miR-2110 | down.mi | up.m |
| VWA1 | hsa-miR-139-3p | down.mi | up.m |
| WASF1 | hsa-let-7b-5p | down.mi | up.m |
| WDHD1 | hsa-miR-218-5p | down.mi | up.m |
| WDR12 | hsa-miR-30a-5p | down.mi | up.m |
| WDR12 | hsa-miR-378a-5p | down.mi | up.m |
| WDR3 | hsa-miR-204-5p | down.mi | up.m |
| WDR3 | hsa-let-7b-5p | down.mi | up.m |
| WDR3 | hsa-let-7c-5p | down.mi | up.m |
| WDR4 | hsa-let-7b-5p | down.mi | up.m |
| WDR4 | hsa-let-7e-5p | down.mi | up.m |
| WDR45B | hsa-miR-139-3p | down.mi | up.m |
| WDR45B | hsa-miR-423-5p | down.mi | up.m |
| WDR46 | hsa-let-7a-5p | down.mi | up.m |
| WDR74 | hsa-miR-30a-5p | down.mi | up.m |
| WDR74 | hsa-let-7b-5p | down.mi | up.m |
| WDR75 | hsa-miR-30c-2-3p | down.mi | up.m |
| WDR75 | hsa-miR-30b-3p | down.mi | up.m |
| WDR75 | hsa-let-7b-5p | down.mi | up.m |
| WDR75 | hsa-miR-30a-5p | down.mi | up.m |
| WISP1 | hsa-miR-204-5p | down.mi | up.m |
| WNT10A | hsa-miR-378a-3p | down.mi | up.m |
| WNT10A | hsa-miR-27a-5p | down.mi | up.m |
| XKR9 | hsa-miR-374a-5p | down.mi | up.m |
| XKR9 | hsa-miR-15b-5p | down.mi | up.m |
| XPO1 | hsa-miR-30a-5p | down.mi | up.m |
| XPO1 | hsa-miR-30d-5p | down.mi | up.m |
| XPO5 | hsa-let-7b-5p | down.mi | up.m |
| XPO6 | hsa-miR-1-3p | down.mi | up.m |
| XPOT | hsa-miR-30a-5p | down.mi | up.m |
| XPOT | hsa-miR-1-3p | down.mi | up.m |
| XPOT | hsa-miR-378a-5p | down.mi | up.m |
| XPOT | hsa-miR-140-3p | down.mi | up.m |
| XYLT2 | hsa-let-7b-5p | down.mi | up.m |
| YAE1D1 | hsa-miR-486-5p | down.mi | up.m |
| YAE1D1 | hsa-let-7c-5p | down.mi | up.m |
| YAE1D1 | hsa-let-7f-5p | down.mi | up.m |
| YAE1D1 | hsa-let-7a-5p | down.mi | up.m |
| YAE1D1 | hsa-let-7g-5p | down.mi | up.m |
| YAE1D1 | hsa-miR-30b-3p | down.mi | up.m |
| YAE1D1 | hsa-let-7b-5p | down.mi | up.m |
| YAE1D1 | hsa-let-7e-5p | down.mi | up.m |
| YAE1D1 | hsa-miR-139-3p | down.mi | up.m |
| YARS | hsa-miR-423-5p | down.mi | up.m |
| YARS | hsa-miR-143-3p | down.mi | up.m |
| YARS | hsa-miR-3154 | down.mi | up.m |
| YARS2 | hsa-let-7f-5p | down.mi | up.m |
| YIPF2 | hsa-miR-340-5p | down.mi | up.m |
| YIPF3 | hsa-let-7b-5p | down.mi | up.m |
| YKT6 | hsa-miR-30d-5p | down.mi | up.m |
| YTHDF1 | hsa-miR-218-5p | down.mi | up.m |
| YTHDF1 | hsa-miR-139-5p | down.mi | up.m |
| YTHDF1 | hsa-miR-374a-5p | down.mi | up.m |
| YWHAG | hsa-miR-338-5p | down.mi | up.m |
| YWHAG | hsa-let-7e-5p | down.mi | up.m |
| YWHAG | hsa-miR-30a-3p | down.mi | up.m |
| YWHAG | hsa-miR-195-3p | down.mi | up.m |
| YWHAZ | hsa-miR-34b-3p | down.mi | up.m |
| YWHAZ | hsa-miR-30b-3p | down.mi | up.m |
| YWHAZ | hsa-let-7c-5p | down.mi | up.m |
| YWHAZ | hsa-miR-378a-3p | down.mi | up.m |
| YWHAZ | hsa-let-7g-5p | down.mi | up.m |
| YWHAZ | hsa-miR-1-3p | down.mi | up.m |
| YWHAZ | hsa-let-7f-5p | down.mi | up.m |
| YWHAZ | hsa-let-7b-5p | down.mi | up.m |
| YWHAZ | hsa-miR-423-5p | down.mi | up.m |
| YWHAZ | hsa-let-7e-5p | down.mi | up.m |
| YWHAZ | hsa-miR-30a-5p | down.mi | up.m |
| YWHAZ | hsa-let-7a-5p | down.mi | up.m |
| YWHAZ | hsa-miR-30a-3p | down.mi | up.m |
| ZC3HAV1L | hsa-let-7c-5p | down.mi | up.m |
| ZC3HAV1L | hsa-let-7b-5p | down.mi | up.m |
| ZC3HAV1L | hsa-miR-3154 | down.mi | up.m |
| ZC3HAV1L | hsa-miR-584-5p | down.mi | up.m |
| ZC3HAV1L | hsa-let-7g-5p | down.mi | up.m |
| ZC3HAV1L | hsa-let-7e-5p | down.mi | up.m |
| ZC3HAV1L | hsa-let-7a-5p | down.mi | up.m |
| ZC3HAV1L | hsa-miR-143-3p | down.mi | up.m |
| ZC3HAV1L | hsa-miR-486-3p | down.mi | up.m |
| ZC3HAV1L | hsa-let-7f-5p | down.mi | up.m |
| ZFP62 | hsa-let-7e-5p | down.mi | up.m |
| ZFP62 | hsa-miR-605-5p | down.mi | up.m |
| ZFP64 | hsa-miR-3614-5p | down.mi | up.m |
| ZFP69B | hsa-miR-30b-3p | down.mi | up.m |
| ZMIZ2 | hsa-miR-139-3p | down.mi | up.m |
| ZMYM3 | hsa-let-7a-5p | down.mi | up.m |
| ZNF107 | hsa-miR-340-5p | down.mi | up.m |
| ZNF117 | hsa-miR-490-3p | down.mi | up.m |
| ZNF117 | hsa-miR-30b-3p | down.mi | up.m |
| ZNF117 | hsa-miR-584-5p | down.mi | up.m |
| ZNF121 | hsa-miR-584-5p | down.mi | up.m |
| ZNF207 | hsa-miR-1-3p | down.mi | up.m |
| ZNF207 | hsa-let-7b-5p | down.mi | up.m |
| ZNF207 | hsa-miR-140-3p | down.mi | up.m |
| ZNF207 | hsa-miR-34c-3p | down.mi | up.m |
| ZNF207 | hsa-miR-144-3p | down.mi | up.m |
| ZNF207 | hsa-miR-378a-5p | down.mi | up.m |
| ZNF217 | hsa-miR-378a-5p | down.mi | up.m |
| ZNF217 | hsa-miR-15b-5p | down.mi | up.m |
| ZNF217 | hsa-miR-374a-5p | down.mi | up.m |
| ZNF239 | hsa-miR-378a-3p | down.mi | up.m |
| ZNF251 | hsa-miR-30c-2-3p | down.mi | up.m |
| ZNF260 | hsa-miR-374a-5p | down.mi | up.m |
| ZNF260 | hsa-let-7a-5p | down.mi | up.m |
| ZNF281 | hsa-miR-340-5p | down.mi | up.m |
| ZNF281 | hsa-miR-2110 | down.mi | up.m |
| ZNF281 | hsa-miR-1258 | down.mi | up.m |
| ZNF3 | hsa-miR-218-5p | down.mi | up.m |
| ZNF3 | hsa-let-7b-5p | down.mi | up.m |
| ZNF367 | hsa-miR-99a-5p | down.mi | up.m |
| ZNF367 | hsa-miR-195-5p | down.mi | up.m |
| ZNF367 | hsa-miR-139-5p | down.mi | up.m |
| ZNF367 | hsa-miR-15b-5p | down.mi | up.m |
| ZNF367 | hsa-miR-338-5p | down.mi | up.m |
| ZNF48 | hsa-miR-204-5p | down.mi | up.m |
| ZNF48 | hsa-miR-1-3p | down.mi | up.m |
| ZNF485 | hsa-miR-378a-5p | down.mi | up.m |
| ZNF512B | hsa-miR-584-5p | down.mi | up.m |
| ZNF581 | hsa-let-7b-5p | down.mi | up.m |
| ZNF581 | hsa-let-7c-5p | down.mi | up.m |
| ZNF587 | hsa-miR-1247-3p | down.mi | up.m |
| ZNF587 | hsa-miR-30c-2-3p | down.mi | up.m |
| ZNF587 | hsa-let-7f-5p | down.mi | up.m |
| ZNF587 | hsa-let-7e-5p | down.mi | up.m |
| ZNF587 | hsa-let-7c-5p | down.mi | up.m |
| ZNF587 | hsa-let-7a-5p | down.mi | up.m |
| ZNF587 | hsa-miR-30b-3p | down.mi | up.m |
| ZNF587 | hsa-let-7g-5p | down.mi | up.m |
| ZNF587 | hsa-let-7b-5p | down.mi | up.m |
| ZNF622 | hsa-miR-15b-5p | down.mi | up.m |
| ZNF622 | hsa-miR-1-3p | down.mi | up.m |
| ZNF622 | hsa-miR-195-5p | down.mi | up.m |
| ZNF623 | hsa-miR-30c-2-3p | down.mi | up.m |
| ZNF623 | hsa-miR-204-5p | down.mi | up.m |
| ZNF687 | hsa-let-7b-5p | down.mi | up.m |
| ZNF691 | hsa-miR-15b-5p | down.mi | up.m |
| ZNF691 | hsa-miR-195-5p | down.mi | up.m |
| ZNF786 | hsa-miR-378a-5p | down.mi | up.m |
| ZNHIT3 | hsa-miR-144-5p | down.mi | up.m |
| ZNHIT3 | hsa-miR-378a-3p | down.mi | up.m |
| ZSCAN16 | hsa-miR-145-3p | down.mi | up.m |
| ZSCAN2 | hsa-miR-378a-5p | down.mi | up.m |
| ZSWIM1 | hsa-miR-6720-3p | down.mi | up.m |
| ZSWIM1 | hsa-miR-6892-5p | down.mi | up.m |
| ZWILCH | hsa-miR-204-5p | down.mi | up.m |
| ZYG11A | hsa-miR-30b-3p | down.mi | up.m |
| ZYG11A | hsa-miR-30c-2-3p | down.mi | up.m |
| ZYG11A | hsa-miR-490-3p | down.mi | up.m |
| 10-Sep | hsa-miR-215-5p | up.mi | down.m |
| 10-Sep | hsa-miR-192-5p | up.mi | down.m |
| 2-Mar | hsa-miR-377-3p | up.mi | down.m |
| 2-Mar | hsa-miR-148b-3p | up.mi | down.m |
| 2-Mar | hsa-miR-141-5p | up.mi | down.m |
| 2-Mar | hsa-miR-155-5p | up.mi | down.m |
| 4-Sep | hsa-miR-2355-3p | up.mi | down.m |
| A2M | hsa-miR-128-3p | up.mi | down.m |
| AADAC | hsa-miR-323a-3p | up.mi | down.m |
| AADAC | hsa-miR-4668-3p | up.mi | down.m |
| AAED1 | hsa-miR-766-3p | up.mi | down.m |
| AAED1 | hsa-miR-92b-3p | up.mi | down.m |
| AAED1 | hsa-miR-616-5p | up.mi | down.m |
| AAED1 | hsa-miR-940 | up.mi | down.m |
| AAED1 | hsa-miR-92a-3p | up.mi | down.m |
| ABCA1 | hsa-miR-33b-5p | up.mi | down.m |
| ABCA1 | hsa-miR-19a-3p | up.mi | down.m |
| ABCA1 | hsa-miR-33a-5p | up.mi | down.m |
| ABCA1 | hsa-miR-93-5p | up.mi | down.m |
| ABCA1 | hsa-miR-17-5p | up.mi | down.m |
| ABCA1 | hsa-miR-20a-5p | up.mi | down.m |
| ABCA1 | hsa-miR-148a-5p | up.mi | down.m |
| ABCA1 | hsa-miR-20b-5p | up.mi | down.m |
| ABCA1 | hsa-miR-106b-5p | up.mi | down.m |
| ABCA1 | hsa-miR-128-3p | up.mi | down.m |
| ABCA3 | hsa-miR-20a-5p | up.mi | down.m |
| ABCA3 | hsa-miR-92b-3p | up.mi | down.m |
| ABCA3 | hsa-miR-409-5p | up.mi | down.m |
| ABCA3 | hsa-miR-92a-3p | up.mi | down.m |
| ABCA6 | hsa-miR-96-5p | up.mi | down.m |
| ABCA8 | hsa-miR-192-5p | up.mi | down.m |
| ABCB1 | hsa-miR-21-5p | up.mi | down.m |
| ABCB1 | hsa-miR-186-5p | up.mi | down.m |
| ABCB1 | hsa-miR-9-3p | up.mi | down.m |
| ABCB1 | hsa-miR-495-3p | up.mi | down.m |
| ABCC6 | hsa-miR-424-5p | up.mi | down.m |
| ABCC6 | hsa-miR-625-5p | up.mi | down.m |
| ABCC6 | hsa-miR-5698 | up.mi | down.m |
| ABCC6 | hsa-miR-15a-5p | up.mi | down.m |
| ABCC9 | hsa-miR-142-3p | up.mi | down.m |
| ABCG1 | hsa-miR-128-3p | up.mi | down.m |
| ABCG2 | hsa-miR-212-3p | up.mi | down.m |
| ABCG2 | hsa-miR-16-2-3p | up.mi | down.m |
| ABCG2 | hsa-miR-142-3p | up.mi | down.m |
| ABCG2 | hsa-miR-192-5p | up.mi | down.m |
| ABCG2 | hsa-miR-4724-5p | up.mi | down.m |
| ABCG2 | hsa-miR-889-3p | up.mi | down.m |
| ABCG2 | hsa-miR-369-3p | up.mi | down.m |
| ABHD2 | hsa-miR-92a-3p | up.mi | down.m |
| ABHD2 | hsa-miR-196b-5p | up.mi | down.m |
| ABHD2 | hsa-miR-629-3p | up.mi | down.m |
| ABHD2 | hsa-miR-493-3p | up.mi | down.m |
| ABHD2 | hsa-miR-532-3p | up.mi | down.m |
| ABHD2 | hsa-miR-340-3p | up.mi | down.m |
| ABHD2 | hsa-miR-186-5p | up.mi | down.m |
| ABHD2 | hsa-miR-15a-5p | up.mi | down.m |
| ABHD2 | hsa-miR-33b-5p | up.mi | down.m |
| ABHD2 | hsa-miR-1307-3p | up.mi | down.m |
| ABHD2 | hsa-miR-561-5p | up.mi | down.m |
| ABHD2 | hsa-miR-17-5p | up.mi | down.m |
| ABHD2 | hsa-miR-33a-5p | up.mi | down.m |
| ABHD2 | hsa-miR-93-5p | up.mi | down.m |
| ABHD2 | hsa-miR-20a-5p | up.mi | down.m |
| ABHD2 | hsa-miR-106b-5p | up.mi | down.m |
| ABHD2 | hsa-miR-20b-5p | up.mi | down.m |
| ABHD2 | hsa-miR-424-5p | up.mi | down.m |
| ABHD2 | hsa-miR-127-5p | up.mi | down.m |
| ABHD2 | hsa-miR-196a-5p | up.mi | down.m |
| ABHD5 | hsa-miR-19b-3p | up.mi | down.m |
| ABHD5 | hsa-miR-19a-3p | up.mi | down.m |
| ABLIM1 | hsa-miR-148b-3p | up.mi | down.m |
| ABLIM1 | hsa-miR-148a-3p | up.mi | down.m |
| ABLIM1 | hsa-miR-331-3p | up.mi | down.m |
| ABLIM1 | hsa-miR-1306-5p | up.mi | down.m |
| ABLIM1 | hsa-miR-34a-5p | up.mi | down.m |
| ABLIM1 | hsa-miR-153-3p | up.mi | down.m |
| ABLIM1 | hsa-miR-615-3p | up.mi | down.m |
| ACAA2 | hsa-miR-3913-5p | up.mi | down.m |
| ACAA2 | hsa-miR-143-5p | up.mi | down.m |
| ACAA2 | hsa-miR-9-5p | up.mi | down.m |
| ACAA2 | hsa-miR-186-5p | up.mi | down.m |
| ACACB | hsa-miR-425-5p | up.mi | down.m |
| ACACB | hsa-miR-148b-3p | up.mi | down.m |
| ACACB | hsa-miR-766-3p | up.mi | down.m |
| ACADL | hsa-miR-642a-5p | up.mi | down.m |
| ACADL | hsa-miR-3913-5p | up.mi | down.m |
| ACER3 | hsa-miR-1301-3p | up.mi | down.m |
| ACER3 | hsa-miR-153-5p | up.mi | down.m |
| ACER3 | hsa-miR-186-5p | up.mi | down.m |
| ACER3 | hsa-miR-142-5p | up.mi | down.m |
| ACER3 | hsa-miR-130b-5p | up.mi | down.m |
| ACER3 | hsa-miR-450b-5p | up.mi | down.m |
| ACER3 | hsa-miR-335-3p | up.mi | down.m |
| ACSL1 | hsa-miR-154-5p | up.mi | down.m |
| ACSL1 | hsa-miR-205-5p | up.mi | down.m |
| ACSL1 | hsa-miR-192-5p | up.mi | down.m |
| ACSL1 | hsa-miR-142-3p | up.mi | down.m |
| ACSL1 | hsa-miR-93-3p | up.mi | down.m |
| ACSL1 | hsa-miR-34a-5p | up.mi | down.m |
| ACSL1 | hsa-miR-191-5p | up.mi | down.m |
| ACSL4 | hsa-miR-454-3p | up.mi | down.m |
| ACSL4 | hsa-miR-130a-3p | up.mi | down.m |
| ACSL4 | hsa-miR-450b-5p | up.mi | down.m |
| ACSL4 | hsa-miR-106a-5p | up.mi | down.m |
| ACSL4 | hsa-miR-200b-3p | up.mi | down.m |
| ACSL4 | hsa-miR-130b-3p | up.mi | down.m |
| ACSL4 | hsa-miR-19a-3p | up.mi | down.m |
| ACSL4 | hsa-miR-33a-5p | up.mi | down.m |
| ACSL4 | hsa-miR-429 | up.mi | down.m |
| ACSL4 | hsa-miR-301a-3p | up.mi | down.m |
| ACSL4 | hsa-miR-93-5p | up.mi | down.m |
| ACSL4 | hsa-miR-205-5p | up.mi | down.m |
| ACSL4 | hsa-miR-19b-3p | up.mi | down.m |
| ACSL4 | hsa-miR-16-1-3p | up.mi | down.m |
| ACSL4 | hsa-miR-186-5p | up.mi | down.m |
| ACSL4 | hsa-miR-106b-5p | up.mi | down.m |
| ACSL4 | hsa-miR-212-3p | up.mi | down.m |
| ACSL4 | hsa-miR-34a-5p | up.mi | down.m |
| ACSL4 | hsa-miR-20a-5p | up.mi | down.m |
| ACSL4 | hsa-miR-301b-3p | up.mi | down.m |
| ACSL4 | hsa-miR-200c-3p | up.mi | down.m |
| ACSL4 | hsa-miR-17-5p | up.mi | down.m |
| ACSL4 | hsa-miR-33b-5p | up.mi | down.m |
| ACSL4 | hsa-miR-20b-5p | up.mi | down.m |
| ACSL4 | hsa-miR-142-3p | up.mi | down.m |
| ACSL4 | hsa-miR-369-3p | up.mi | down.m |
| ACSS3 | hsa-miR-450b-5p | up.mi | down.m |
| ACSS3 | hsa-miR-130a-5p | up.mi | down.m |
| ACSS3 | hsa-miR-153-5p | up.mi | down.m |
| ACVRL1 | hsa-miR-940 | up.mi | down.m |
| ADAMTS1 | hsa-miR-1180-3p | up.mi | down.m |
| ADAMTS1 | hsa-miR-92a-3p | up.mi | down.m |
| ADAMTS1 | hsa-miR-615-3p | up.mi | down.m |
| ADAMTS8 | hsa-miR-335-3p | up.mi | down.m |
| ADARB1 | hsa-miR-93-5p | up.mi | down.m |
| ADARB1 | hsa-miR-148a-3p | up.mi | down.m |
| ADARB1 | hsa-miR-130b-3p | up.mi | down.m |
| ADARB1 | hsa-miR-34a-5p | up.mi | down.m |
| ADARB1 | hsa-miR-193b-3p | up.mi | down.m |
| ADARB1 | hsa-miR-17-5p | up.mi | down.m |
| ADCY9 | hsa-miR-33b-5p | up.mi | down.m |
| ADCY9 | hsa-miR-331-3p | up.mi | down.m |
| ADCY9 | hsa-miR-181b-5p | up.mi | down.m |
| ADCY9 | hsa-miR-5698 | up.mi | down.m |
| ADCY9 | hsa-miR-1301-3p | up.mi | down.m |
| ADCY9 | hsa-miR-29b-2-5p | up.mi | down.m |
| ADCY9 | hsa-miR-3189-3p | up.mi | down.m |
| ADCY9 | hsa-miR-193b-3p | up.mi | down.m |
| ADCY9 | hsa-miR-143-5p | up.mi | down.m |
| ADCY9 | hsa-miR-181d-5p | up.mi | down.m |
| ADD1 | hsa-miR-3170 | up.mi | down.m |
| ADD1 | hsa-miR-320b | up.mi | down.m |
| ADD1 | hsa-miR-20b-5p | up.mi | down.m |
| ADD1 | hsa-miR-93-5p | up.mi | down.m |
| ADD1 | hsa-miR-17-5p | up.mi | down.m |
| ADD1 | hsa-miR-128-3p | up.mi | down.m |
| ADD1 | hsa-miR-20a-5p | up.mi | down.m |
| ADD1 | hsa-miR-193b-5p | up.mi | down.m |
| ADD1 | hsa-miR-106b-5p | up.mi | down.m |
| ADH1B | hsa-miR-381-3p | up.mi | down.m |
| ADIPOR2 | hsa-miR-127-5p | up.mi | down.m |
| ADIPOR2 | hsa-miR-186-5p | up.mi | down.m |
| ADIPOR2 | hsa-miR-19b-3p | up.mi | down.m |
| ADIPOR2 | hsa-miR-501-5p | up.mi | down.m |
| ADIPOR2 | hsa-miR-19a-3p | up.mi | down.m |
| ADPRH | hsa-miR-301a-5p | up.mi | down.m |
| ADPRH | hsa-miR-130b-5p | up.mi | down.m |
| ADRB1 | hsa-miR-188-5p | up.mi | down.m |
| ADRB1 | hsa-miR-19a-3p | up.mi | down.m |
| ADRB1 | hsa-miR-141-5p | up.mi | down.m |
| ADRB1 | hsa-miR-431-5p | up.mi | down.m |
| AFAP1L1 | hsa-miR-192-5p | up.mi | down.m |
| AFF3 | hsa-miR-766-3p | up.mi | down.m |
| AGPAT4 | hsa-miR-141-5p | up.mi | down.m |
| AGPAT4 | hsa-miR-20a-3p | up.mi | down.m |
| AGPAT4 | hsa-miR-766-3p | up.mi | down.m |
| AGTPBP1 | hsa-miR-4728-3p | up.mi | down.m |
| AGTPBP1 | hsa-miR-576-5p | up.mi | down.m |
| AGTPBP1 | hsa-miR-493-5p | up.mi | down.m |
| AGTPBP1 | hsa-miR-590-3p | up.mi | down.m |
| AGTR1 | hsa-miR-155-5p | up.mi | down.m |
| AGTR1 | hsa-miR-34a-5p | up.mi | down.m |
| AGTR2 | hsa-miR-148b-3p | up.mi | down.m |
| AGTR2 | hsa-miR-142-3p | up.mi | down.m |
| AHCYL2 | hsa-miR-940 | up.mi | down.m |
| AHCYL2 | hsa-miR-92a-3p | up.mi | down.m |
| AHCYL2 | hsa-miR-185-5p | up.mi | down.m |
| AHCYL2 | hsa-miR-1277-3p | up.mi | down.m |
| AHNAK | hsa-miR-324-5p | up.mi | down.m |
| AHNAK | hsa-miR-34a-5p | up.mi | down.m |
| AK1 | hsa-miR-431-5p | up.mi | down.m |
| AK1 | hsa-miR-17-5p | up.mi | down.m |
| AKAP11 | hsa-miR-17-5p | up.mi | down.m |
| AKAP11 | hsa-miR-20a-5p | up.mi | down.m |
| AKAP11 | hsa-miR-93-5p | up.mi | down.m |
| AKAP11 | hsa-miR-142-5p | up.mi | down.m |
| AKAP11 | hsa-miR-130b-3p | up.mi | down.m |
| AKAP11 | hsa-miR-215-5p | up.mi | down.m |
| AKAP11 | hsa-miR-106a-5p | up.mi | down.m |
| AKAP11 | hsa-miR-106b-5p | up.mi | down.m |
| AKAP11 | hsa-miR-20b-5p | up.mi | down.m |
| AKAP11 | hsa-miR-186-5p | up.mi | down.m |
| AKAP11 | hsa-miR-148b-3p | up.mi | down.m |
| AKAP11 | hsa-miR-615-3p | up.mi | down.m |
| AKAP11 | hsa-miR-192-5p | up.mi | down.m |
| AKAP11 | hsa-miR-15a-5p | up.mi | down.m |
| AKAP11 | hsa-miR-33a-5p | up.mi | down.m |
| AKAP12 | hsa-miR-29b-1-5p | up.mi | down.m |
| AKAP12 | hsa-miR-186-5p | up.mi | down.m |
| AKAP12 | hsa-miR-20b-3p | up.mi | down.m |
| AKAP12 | hsa-miR-183-5p | up.mi | down.m |
| AKAP12 | hsa-miR-592 | up.mi | down.m |
| AKAP12 | hsa-miR-760 | up.mi | down.m |
| AKAP12 | hsa-miR-577 | up.mi | down.m |
| AKAP13 | hsa-miR-193b-3p | up.mi | down.m |
| AKAP13 | hsa-miR-149-5p | up.mi | down.m |
| AKAP13 | hsa-miR-183-5p | up.mi | down.m |
| AKAP2 | hsa-miR-19b-3p | up.mi | down.m |
| AKAP2 | hsa-miR-629-3p | up.mi | down.m |
| AKAP2 | hsa-miR-432-5p | up.mi | down.m |
| AKAP2 | hsa-miR-130b-5p | up.mi | down.m |
| AKAP2 | hsa-miR-642a-5p | up.mi | down.m |
| AKAP2 | hsa-miR-651-5p | up.mi | down.m |
| AKAP2 | hsa-miR-19a-3p | up.mi | down.m |
| AKAP7 | hsa-miR-215-5p | up.mi | down.m |
| AKAP7 | hsa-miR-192-5p | up.mi | down.m |
| AKT3 | hsa-miR-20b-5p | up.mi | down.m |
| AKT3 | hsa-miR-29b-3p | up.mi | down.m |
| AKT3 | hsa-miR-15a-5p | up.mi | down.m |
| AKT3 | hsa-miR-503-5p | up.mi | down.m |
| AKT3 | hsa-miR-708-3p | up.mi | down.m |
| AKT3 | hsa-miR-424-5p | up.mi | down.m |
| AKT3 | hsa-miR-29b-1-5p | up.mi | down.m |
| ALCAM | hsa-miR-192-5p | up.mi | down.m |
| ALCAM | hsa-miR-148a-3p | up.mi | down.m |
| ALCAM | hsa-miR-215-5p | up.mi | down.m |
| ALCAM | hsa-miR-148b-3p | up.mi | down.m |
| ALCAM | hsa-miR-9-5p | up.mi | down.m |
| ALDH1A2 | hsa-miR-615-3p | up.mi | down.m |
| ALDH1A2 | hsa-miR-155-5p | up.mi | down.m |
| ALDH1A2 | hsa-miR-16-2-3p | up.mi | down.m |
| ALDH1A2 | hsa-miR-193b-3p | up.mi | down.m |
| ALDH1A2 | hsa-miR-2355-5p | up.mi | down.m |
| ALDH2 | hsa-miR-615-3p | up.mi | down.m |
| ALDH3B1 | hsa-miR-15a-5p | up.mi | down.m |
| ALDH3B1 | hsa-miR-424-5p | up.mi | down.m |
| ALOX15 | hsa-miR-940 | up.mi | down.m |
| ALOX5 | hsa-miR-19a-3p | up.mi | down.m |
| ALOX5AP | hsa-miR-5698 | up.mi | down.m |
| ALOX5AP | hsa-miR-143-5p | up.mi | down.m |
| ALOX5AP | hsa-miR-590-3p | up.mi | down.m |
| ALOX5AP | hsa-miR-744-3p | up.mi | down.m |
| AMIGO1 | hsa-miR-92b-3p | up.mi | down.m |
| AMOTL1 | hsa-miR-9-5p | up.mi | down.m |
| AMOTL1 | hsa-let-7c-3p | up.mi | down.m |
| AMOTL1 | hsa-miR-16-2-3p | up.mi | down.m |
| AMOTL1 | hsa-miR-5698 | up.mi | down.m |
| AMOTL1 | hsa-miR-4326 | up.mi | down.m |
| AMOTL1 | hsa-miR-616-5p | up.mi | down.m |
| AMOTL1 | hsa-miR-15a-5p | up.mi | down.m |
| AMOTL1 | hsa-miR-424-5p | up.mi | down.m |
| AMOTL1 | hsa-miR-92a-3p | up.mi | down.m |
| AMOTL1 | hsa-miR-2277-5p | up.mi | down.m |
| AMOTL1 | hsa-miR-339-5p | up.mi | down.m |
| AMOTL2 | hsa-miR-590-3p | up.mi | down.m |
| AMOTL2 | hsa-miR-135b-5p | up.mi | down.m |
| AMOTL2 | hsa-miR-4668-3p | up.mi | down.m |
| AMOTL2 | hsa-miR-532-3p | up.mi | down.m |
| AMOTL2 | hsa-miR-376b-3p | up.mi | down.m |
| AMOTL2 | hsa-miR-185-5p | up.mi | down.m |
| ANGPT4 | hsa-miR-542-3p | up.mi | down.m |
| ANGPT4 | hsa-miR-616-5p | up.mi | down.m |
| ANGPT4 | hsa-miR-3913-5p | up.mi | down.m |
| ANGPT4 | hsa-miR-940 | up.mi | down.m |
| ANGPT4 | hsa-miR-6510-3p | up.mi | down.m |
| ANGPT4 | hsa-miR-766-3p | up.mi | down.m |
| ANGPTL7 | hsa-miR-141-3p | up.mi | down.m |
| ANGPTL7 | hsa-miR-200a-3p | up.mi | down.m |
| ANGPTL7 | hsa-miR-205-5p | up.mi | down.m |
| ANGPTL7 | hsa-miR-188-3p | up.mi | down.m |
| ANK3 | hsa-miR-34a-5p | up.mi | down.m |
| ANK3 | hsa-miR-503-5p | up.mi | down.m |
| ANKRD29 | hsa-miR-93-5p | up.mi | down.m |
| ANKRD29 | hsa-miR-33a-5p | up.mi | down.m |
| ANKRD29 | hsa-miR-130b-3p | up.mi | down.m |
| ANKRD33B | hsa-miR-106b-5p | up.mi | down.m |
| ANKRD33B | hsa-miR-200b-3p | up.mi | down.m |
| ANKRD33B | hsa-miR-3170 | up.mi | down.m |
| ANKRD33B | hsa-miR-542-3p | up.mi | down.m |
| ANKRD33B | hsa-miR-4724-5p | up.mi | down.m |
| ANKRD33B | hsa-miR-17-5p | up.mi | down.m |
| ANKRD33B | hsa-miR-429 | up.mi | down.m |
| ANKRD33B | hsa-miR-93-5p | up.mi | down.m |
| ANKRD33B | hsa-miR-20b-5p | up.mi | down.m |
| ANKRD33B | hsa-miR-655-3p | up.mi | down.m |
| ANKRD33B | hsa-miR-874-3p | up.mi | down.m |
| ANKRD33B | hsa-miR-142-5p | up.mi | down.m |
| ANKRD33B | hsa-miR-20a-5p | up.mi | down.m |
| ANKRD33B | hsa-miR-3677-3p | up.mi | down.m |
| ANKRD33B | hsa-miR-200c-3p | up.mi | down.m |
| ANKRD33B | hsa-miR-106a-5p | up.mi | down.m |
| ANKRD33B | hsa-miR-193b-5p | up.mi | down.m |
| ANKRD44 | hsa-miR-335-3p | up.mi | down.m |
| ANKS1A | hsa-miR-34a-5p | up.mi | down.m |
| ANKS1A | hsa-miR-449a | up.mi | down.m |
| ANKS1A | hsa-miR-744-3p | up.mi | down.m |
| ANKS1A | hsa-miR-92a-3p | up.mi | down.m |
| ANO6 | hsa-miR-192-5p | up.mi | down.m |
| ANO6 | hsa-miR-615-3p | up.mi | down.m |
| ANO6 | hsa-miR-182-5p | up.mi | down.m |
| ANPEP | hsa-miR-155-5p | up.mi | down.m |
| ANXA1 | hsa-miR-196a-5p | up.mi | down.m |
| ANXA6 | hsa-miR-324-5p | up.mi | down.m |
| AOC3 | hsa-miR-335-3p | up.mi | down.m |
| AP1S2 | hsa-miR-215-5p | up.mi | down.m |
| AP1S2 | hsa-miR-192-5p | up.mi | down.m |
| AP1S2 | hsa-miR-542-3p | up.mi | down.m |
| APBB1 | hsa-miR-148b-3p | up.mi | down.m |
| APBB1 | hsa-miR-331-3p | up.mi | down.m |
| APBB2 | hsa-miR-424-5p | up.mi | down.m |
| APBB2 | hsa-miR-148b-3p | up.mi | down.m |
| APBB2 | hsa-miR-93-5p | up.mi | down.m |
| APBB2 | hsa-miR-9-5p | up.mi | down.m |
| APCDD1 | hsa-miR-193b-3p | up.mi | down.m |
| APCDD1 | hsa-miR-1306-5p | up.mi | down.m |
| APCDD1 | hsa-miR-382-5p | up.mi | down.m |
| APLN | hsa-miR-148b-3p | up.mi | down.m |
| APLN | hsa-miR-629-3p | up.mi | down.m |
| APLN | hsa-miR-550a-5p | up.mi | down.m |
| APLN | hsa-miR-361-3p | up.mi | down.m |
| APLN | hsa-miR-148a-3p | up.mi | down.m |
| APLN | hsa-miR-224-5p | up.mi | down.m |
| APOA1 | hsa-miR-135b-5p | up.mi | down.m |
| APOA1 | hsa-miR-431-5p | up.mi | down.m |
| APOBEC3A | hsa-miR-1307-3p | up.mi | down.m |
| APOBEC3A | hsa-miR-143-5p | up.mi | down.m |
| APOBEC4 | hsa-miR-181b-3p | up.mi | down.m |
| APOH | hsa-miR-103a-2-5p | up.mi | down.m |
| APOH | hsa-miR-106b-5p | up.mi | down.m |
| APOH | hsa-miR-93-5p | up.mi | down.m |
| APOH | hsa-miR-106a-5p | up.mi | down.m |
| APOH | hsa-miR-17-5p | up.mi | down.m |
| APOH | hsa-miR-20b-5p | up.mi | down.m |
| APOH | hsa-miR-20a-5p | up.mi | down.m |
| APOLD1 | hsa-miR-324-3p | up.mi | down.m |
| APOLD1 | hsa-miR-92b-3p | up.mi | down.m |
| APOLD1 | hsa-miR-92a-3p | up.mi | down.m |
| APOLD1 | hsa-miR-193b-3p | up.mi | down.m |
| APOLD1 | hsa-miR-21-5p | up.mi | down.m |
| APOLD1 | hsa-miR-215-5p | up.mi | down.m |
| APOLD1 | hsa-miR-192-5p | up.mi | down.m |
| AQP4 | hsa-miR-29b-3p | up.mi | down.m |
| ARAP1 | hsa-miR-874-3p | up.mi | down.m |
| ARAP2 | hsa-miR-93-5p | up.mi | down.m |
| ARAP2 | hsa-miR-767-3p | up.mi | down.m |
| ARAP2 | hsa-miR-20a-5p | up.mi | down.m |
| ARAP2 | hsa-miR-17-5p | up.mi | down.m |
| ARAP2 | hsa-miR-335-3p | up.mi | down.m |
| ARAP2 | hsa-miR-20b-5p | up.mi | down.m |
| ARAP2 | hsa-miR-19a-3p | up.mi | down.m |
| ARAP2 | hsa-miR-106b-5p | up.mi | down.m |
| ARAP2 | hsa-miR-381-3p | up.mi | down.m |
| ARAP2 | hsa-miR-19b-3p | up.mi | down.m |
| ARAP2 | hsa-miR-582-5p | up.mi | down.m |
| ARC | hsa-miR-4668-3p | up.mi | down.m |
| ARC | hsa-miR-185-5p | up.mi | down.m |
| ARC | hsa-miR-19a-3p | up.mi | down.m |
| ARC | hsa-miR-135b-5p | up.mi | down.m |
| ARC | hsa-miR-19b-3p | up.mi | down.m |
| ARC | hsa-miR-455-5p | up.mi | down.m |
| AREG | hsa-miR-200a-3p | up.mi | down.m |
| AREG | hsa-miR-34a-5p | up.mi | down.m |
| ARHGAP18 | hsa-miR-3136-5p | up.mi | down.m |
| ARHGAP18 | hsa-miR-134-5p | up.mi | down.m |
| ARHGAP18 | hsa-miR-339-5p | up.mi | down.m |
| ARHGAP18 | hsa-miR-15b-3p | up.mi | down.m |
| ARHGAP29 | hsa-miR-215-5p | up.mi | down.m |
| ARHGAP29 | hsa-miR-192-5p | up.mi | down.m |
| ARHGAP31 | hsa-miR-33b-5p | up.mi | down.m |
| ARHGAP31 | hsa-miR-33a-5p | up.mi | down.m |
| ARHGAP31 | hsa-miR-576-5p | up.mi | down.m |
| ARHGAP31 | hsa-miR-93-3p | up.mi | down.m |
| ARHGAP6 | hsa-miR-369-3p | up.mi | down.m |
| ARHGAP6 | hsa-miR-655-3p | up.mi | down.m |
| ARHGEF10 | hsa-miR-192-5p | up.mi | down.m |
| ARHGEF10 | hsa-miR-9-5p | up.mi | down.m |
| ARHGEF17 | hsa-miR-361-3p | up.mi | down.m |
| ARHGEF17 | hsa-miR-452-3p | up.mi | down.m |
| ARHGEF26 | hsa-miR-7705 | up.mi | down.m |
| ARHGEF26 | hsa-miR-454-3p | up.mi | down.m |
| ARHGEF26 | hsa-miR-192-5p | up.mi | down.m |
| ARHGEF26 | hsa-miR-19b-3p | up.mi | down.m |
| ARHGEF26 | hsa-miR-22-3p | up.mi | down.m |
| ARHGEF26 | hsa-miR-130b-3p | up.mi | down.m |
| ARHGEF26 | hsa-miR-215-5p | up.mi | down.m |
| ARHGEF26 | hsa-miR-19a-3p | up.mi | down.m |
| ARHGEF26 | hsa-miR-301a-3p | up.mi | down.m |
| ARHGEF26 | hsa-miR-188-3p | up.mi | down.m |
| ARHGEF26 | hsa-miR-130a-3p | up.mi | down.m |
| ARHGEF26 | hsa-miR-301b-3p | up.mi | down.m |
| ARHGEF3 | hsa-miR-450b-5p | up.mi | down.m |
| ARHGEF3 | hsa-miR-889-3p | up.mi | down.m |
| ARHGEF6 | hsa-miR-185-5p | up.mi | down.m |
| ARID5A | hsa-miR-324-5p | up.mi | down.m |
| ARL11 | hsa-miR-642a-5p | up.mi | down.m |
| ARL13B | hsa-miR-106b-5p | up.mi | down.m |
| ARRB1 | hsa-miR-642a-5p | up.mi | down.m |
| ARRB1 | hsa-miR-92a-3p | up.mi | down.m |
| ARRB1 | hsa-miR-186-5p | up.mi | down.m |
| ARRB2 | hsa-miR-361-3p | up.mi | down.m |
| ARRB2 | hsa-miR-181d-5p | up.mi | down.m |
| ARRB2 | hsa-miR-181b-5p | up.mi | down.m |
| ARRB2 | hsa-miR-149-5p | up.mi | down.m |
| ARRB2 | hsa-miR-615-3p | up.mi | down.m |
| ARRDC4 | hsa-miR-324-3p | up.mi | down.m |
| ART4 | hsa-miR-212-3p | up.mi | down.m |
| ART4 | hsa-miR-193b-5p | up.mi | down.m |
| ART4 | hsa-miR-188-5p | up.mi | down.m |
| ASAH1 | hsa-miR-92a-3p | up.mi | down.m |
| ASGR1 | hsa-miR-501-5p | up.mi | down.m |
| ASGR1 | hsa-miR-362-5p | up.mi | down.m |
| ASGR1 | hsa-miR-4668-3p | up.mi | down.m |
| ASPA | hsa-miR-455-3p | up.mi | down.m |
| ASPA | hsa-miR-940 | up.mi | down.m |
| ASPA | hsa-miR-629-3p | up.mi | down.m |
| ATF3 | hsa-miR-590-5p | up.mi | down.m |
| ATF3 | hsa-miR-331-3p | up.mi | down.m |
| ATF3 | hsa-miR-377-3p | up.mi | down.m |
| ATF3 | hsa-miR-505-5p | up.mi | down.m |
| ATF3 | hsa-miR-17-5p | up.mi | down.m |
| ATOH8 | hsa-miR-17-3p | up.mi | down.m |
| ATOH8 | hsa-miR-193b-3p | up.mi | down.m |
| ATP13A4 | hsa-miR-940 | up.mi | down.m |
| ATP13A4 | hsa-miR-1287-3p | up.mi | down.m |
| ATP13A4 | hsa-miR-9-3p | up.mi | down.m |
| ATP13A4 | hsa-miR-660-5p | up.mi | down.m |
| ATP2B4 | hsa-miR-642a-5p | up.mi | down.m |
| ATP2B4 | hsa-miR-452-3p | up.mi | down.m |
| ATP2B4 | hsa-miR-92b-3p | up.mi | down.m |
| ATP2B4 | hsa-miR-21-5p | up.mi | down.m |
| ATP2B4 | hsa-miR-92a-3p | up.mi | down.m |
| ATP6V1B2 | hsa-miR-301a-3p | up.mi | down.m |
| ATP6V1B2 | hsa-miR-454-3p | up.mi | down.m |
| ATP6V1B2 | hsa-miR-130b-3p | up.mi | down.m |
| ATP6V1B2 | hsa-miR-301b-3p | up.mi | down.m |
| ATP6V1B2 | hsa-miR-19a-3p | up.mi | down.m |
| ATP6V1B2 | hsa-miR-19b-3p | up.mi | down.m |
| ATP6V1B2 | hsa-miR-186-5p | up.mi | down.m |
| ATP6V1B2 | hsa-miR-130a-3p | up.mi | down.m |
| ATP6V1B2 | hsa-miR-196a-5p | up.mi | down.m |
| ATP6V1B2 | hsa-miR-503-5p | up.mi | down.m |
| ATP6V1B2 | hsa-miR-323b-3p | up.mi | down.m |
| ATP8A1 | hsa-miR-128-3p | up.mi | down.m |
| AVPI1 | hsa-miR-148b-3p | up.mi | down.m |
| AXIN2 | hsa-miR-34b-5p | up.mi | down.m |
| AXIN2 | hsa-miR-424-5p | up.mi | down.m |
| AXIN2 | hsa-miR-15a-5p | up.mi | down.m |
| AXIN2 | hsa-miR-34a-3p | up.mi | down.m |
| AXIN2 | hsa-miR-582-3p | up.mi | down.m |
| AXIN2 | hsa-miR-34a-5p | up.mi | down.m |
| AXL | hsa-miR-34a-5p | up.mi | down.m |
| AXL | hsa-miR-339-5p | up.mi | down.m |
| AXL | hsa-miR-155-5p | up.mi | down.m |
| B2M | hsa-miR-142-5p | up.mi | down.m |
| B2M | hsa-miR-17-5p | up.mi | down.m |
| B2M | hsa-miR-93-5p | up.mi | down.m |
| B2M | hsa-miR-20a-5p | up.mi | down.m |
| B2M | hsa-miR-106a-5p | up.mi | down.m |
| B2M | hsa-miR-106b-5p | up.mi | down.m |
| B2M | hsa-miR-20b-5p | up.mi | down.m |
| B2M | hsa-miR-186-5p | up.mi | down.m |
| B2M | hsa-miR-501-3p | up.mi | down.m |
| B2M | hsa-miR-16-2-3p | up.mi | down.m |
| B3GALNT1 | hsa-miR-192-5p | up.mi | down.m |
| B3GALNT1 | hsa-miR-215-5p | up.mi | down.m |
| B3GALNT1 | hsa-miR-21-5p | up.mi | down.m |
| B3GALNT1 | hsa-miR-130b-5p | up.mi | down.m |
| B3GNT2 | hsa-miR-183-5p | up.mi | down.m |
| B3GNT2 | hsa-miR-424-5p | up.mi | down.m |
| B3GNT2 | hsa-miR-15a-5p | up.mi | down.m |
| B3GNT2 | hsa-miR-590-3p | up.mi | down.m |
| BANK1 | hsa-miR-590-3p | up.mi | down.m |
| BCL2A1 | hsa-miR-34a-5p | up.mi | down.m |
| BCL2L2 | hsa-miR-361-3p | up.mi | down.m |
| BCL2L2 | hsa-miR-20b-5p | up.mi | down.m |
| BCL2L2 | hsa-miR-17-5p | up.mi | down.m |
| BCL2L2 | hsa-miR-106b-5p | up.mi | down.m |
| BCL2L2 | hsa-miR-20a-5p | up.mi | down.m |
| BCL2L2 | hsa-miR-106a-5p | up.mi | down.m |
| BCL2L2 | hsa-miR-93-5p | up.mi | down.m |
| BDNF | hsa-miR-210-3p | up.mi | down.m |
| BDNF | hsa-miR-182-5p | up.mi | down.m |
| BDNF | hsa-miR-15a-5p | up.mi | down.m |
| BDNF | hsa-miR-96-5p | up.mi | down.m |
| BDNF | hsa-miR-22-3p | up.mi | down.m |
| BEX4 | hsa-miR-615-3p | up.mi | down.m |
| BEX4 | hsa-miR-425-5p | up.mi | down.m |
| BHLHE41 | hsa-miR-186-5p | up.mi | down.m |
| BMP2 | hsa-miR-17-5p | up.mi | down.m |
| BMP2 | hsa-miR-576-5p | up.mi | down.m |
| BMP2 | hsa-miR-20a-5p | up.mi | down.m |
| BMP2 | hsa-miR-20b-5p | up.mi | down.m |
| BMP2 | hsa-miR-106b-5p | up.mi | down.m |
| BMP2 | hsa-miR-93-5p | up.mi | down.m |
| BMP2 | hsa-miR-106a-5p | up.mi | down.m |
| BMP2 | hsa-miR-505-3p | up.mi | down.m |
| BMP2 | hsa-miR-106b-3p | up.mi | down.m |
| BMP3 | hsa-miR-19a-3p | up.mi | down.m |
| BMP3 | hsa-miR-148a-3p | up.mi | down.m |
| BMP3 | hsa-miR-766-3p | up.mi | down.m |
| BMP3 | hsa-miR-454-3p | up.mi | down.m |
| BMP3 | hsa-miR-130b-3p | up.mi | down.m |
| BMP3 | hsa-miR-301b-3p | up.mi | down.m |
| BMP3 | hsa-miR-449a | up.mi | down.m |
| BMP3 | hsa-miR-19b-3p | up.mi | down.m |
| BMP3 | hsa-miR-148b-3p | up.mi | down.m |
| BMP3 | hsa-miR-130a-3p | up.mi | down.m |
| BMP3 | hsa-miR-34a-5p | up.mi | down.m |
| BMP3 | hsa-miR-455-3p | up.mi | down.m |
| BMP3 | hsa-miR-301a-3p | up.mi | down.m |
| BMP6 | hsa-miR-22-3p | up.mi | down.m |
| BMPER | hsa-miR-212-3p | up.mi | down.m |
| BMPR2 | hsa-miR-128-3p | up.mi | down.m |
| BMPR2 | hsa-miR-106b-5p | up.mi | down.m |
| BMPR2 | hsa-miR-21-5p | up.mi | down.m |
| BMPR2 | hsa-miR-215-5p | up.mi | down.m |
| BMPR2 | hsa-miR-92a-3p | up.mi | down.m |
| BMPR2 | hsa-miR-181d-5p | up.mi | down.m |
| BMPR2 | hsa-miR-19b-3p | up.mi | down.m |
| BMPR2 | hsa-miR-93-3p | up.mi | down.m |
| BMPR2 | hsa-miR-153-3p | up.mi | down.m |
| BMPR2 | hsa-miR-93-5p | up.mi | down.m |
| BMPR2 | hsa-miR-19a-3p | up.mi | down.m |
| BMPR2 | hsa-miR-92b-3p | up.mi | down.m |
| BMPR2 | hsa-miR-130a-3p | up.mi | down.m |
| BMPR2 | hsa-miR-135b-5p | up.mi | down.m |
| BMPR2 | hsa-miR-103a-2-5p | up.mi | down.m |
| BMPR2 | hsa-miR-192-5p | up.mi | down.m |
| BMPR2 | hsa-miR-20a-5p | up.mi | down.m |
| BMPR2 | hsa-miR-20b-5p | up.mi | down.m |
| BMPR2 | hsa-miR-17-5p | up.mi | down.m |
| BMPR2 | hsa-miR-181b-5p | up.mi | down.m |
| BNIP2 | hsa-miR-20b-5p | up.mi | down.m |
| BNIP2 | hsa-miR-17-5p | up.mi | down.m |
| BNIP2 | hsa-miR-21-5p | up.mi | down.m |
| BNIP2 | hsa-miR-106a-5p | up.mi | down.m |
| BNIP2 | hsa-miR-143-5p | up.mi | down.m |
| BNIP2 | hsa-miR-20a-5p | up.mi | down.m |
| BNIP2 | hsa-miR-455-3p | up.mi | down.m |
| BNIP2 | hsa-miR-194-5p | up.mi | down.m |
| BNIP2 | hsa-miR-106b-5p | up.mi | down.m |
| BNIP2 | hsa-miR-130b-3p | up.mi | down.m |
| BNIP2 | hsa-miR-93-5p | up.mi | down.m |
| BNIP2 | hsa-miR-34a-5p | up.mi | down.m |
| BTG2 | hsa-miR-34b-5p | up.mi | down.m |
| BTG2 | hsa-miR-339-5p | up.mi | down.m |
| BTG2 | hsa-miR-186-5p | up.mi | down.m |
| BTG2 | hsa-miR-3934-3p | up.mi | down.m |
| BTG2 | hsa-miR-106a-5p | up.mi | down.m |
| BTG2 | hsa-miR-92b-3p | up.mi | down.m |
| BTG2 | hsa-miR-15a-5p | up.mi | down.m |
| BTG2 | hsa-miR-92a-3p | up.mi | down.m |
| BTG2 | hsa-miR-21-5p | up.mi | down.m |
| BTG2 | hsa-miR-185-5p | up.mi | down.m |
| BTG2 | hsa-miR-17-5p | up.mi | down.m |
| BTG2 | hsa-miR-5698 | up.mi | down.m |
| BTG2 | hsa-miR-93-5p | up.mi | down.m |
| BTG2 | hsa-miR-193b-5p | up.mi | down.m |
| BTG2 | hsa-miR-210-5p | up.mi | down.m |
| BTG2 | hsa-miR-29b-3p | up.mi | down.m |
| BTG2 | hsa-miR-20a-5p | up.mi | down.m |
| BTG2 | hsa-miR-590-5p | up.mi | down.m |
| BTG2 | hsa-miR-1266-5p | up.mi | down.m |
| BTG2 | hsa-miR-106b-5p | up.mi | down.m |
| BTG2 | hsa-miR-17-3p | up.mi | down.m |
| BTG2 | hsa-miR-142-3p | up.mi | down.m |
| BTG2 | hsa-miR-188-3p | up.mi | down.m |
| BTG2 | hsa-miR-505-5p | up.mi | down.m |
| BTG2 | hsa-miR-20b-5p | up.mi | down.m |
| BTK | hsa-miR-425-5p | up.mi | down.m |
| BTK | hsa-miR-210-3p | up.mi | down.m |
| BTN3A3 | hsa-miR-106b-5p | up.mi | down.m |
| BTN3A3 | hsa-miR-20a-5p | up.mi | down.m |
| BTN3A3 | hsa-miR-15a-5p | up.mi | down.m |
| BTN3A3 | hsa-miR-93-5p | up.mi | down.m |
| BTN3A3 | hsa-miR-17-5p | up.mi | down.m |
| BTN3A3 | hsa-miR-424-5p | up.mi | down.m |
| BTN3A3 | hsa-miR-503-5p | up.mi | down.m |
| BTN3A3 | hsa-miR-21-5p | up.mi | down.m |
| BTN3A3 | hsa-miR-615-3p | up.mi | down.m |
| BTN3A3 | hsa-miR-186-5p | up.mi | down.m |
| BTN3A3 | hsa-miR-20b-5p | up.mi | down.m |
| BTN3A3 | hsa-miR-22-3p | up.mi | down.m |
| BTNL9 | hsa-miR-4326 | up.mi | down.m |
| BTNL9 | hsa-miR-192-5p | up.mi | down.m |
| C10orf10 | hsa-miR-141-5p | up.mi | down.m |
| C10orf10 | hsa-miR-361-3p | up.mi | down.m |
| C10orf67 | hsa-miR-582-3p | up.mi | down.m |
| C11orf16 | hsa-miR-142-3p | up.mi | down.m |
| C11orf96 | hsa-miR-19b-3p | up.mi | down.m |
| C12orf49 | hsa-miR-193b-3p | up.mi | down.m |
| C12orf49 | hsa-miR-493-5p | up.mi | down.m |
| C12orf49 | hsa-miR-340-3p | up.mi | down.m |
| C12orf49 | hsa-miR-3934-3p | up.mi | down.m |
| C12orf49 | hsa-miR-3170 | up.mi | down.m |
| C12orf49 | hsa-miR-193b-5p | up.mi | down.m |
| C12orf49 | hsa-miR-224-5p | up.mi | down.m |
| C15orf52 | hsa-miR-21-5p | up.mi | down.m |
| C15orf52 | hsa-miR-27b-5p | up.mi | down.m |
| C15orf52 | hsa-miR-590-5p | up.mi | down.m |
| C15orf52 | hsa-miR-31-5p | up.mi | down.m |
| C15orf52 | hsa-miR-3127-5p | up.mi | down.m |
| C15orf59 | hsa-miR-7705 | up.mi | down.m |
| C1orf115 | hsa-miR-542-5p | up.mi | down.m |
| C1orf158 | hsa-miR-181b-3p | up.mi | down.m |
| C1orf198 | hsa-miR-192-5p | up.mi | down.m |
| C1orf198 | hsa-miR-31-3p | up.mi | down.m |
| C1orf198 | hsa-miR-215-5p | up.mi | down.m |
| C1orf21 | hsa-miR-15a-5p | up.mi | down.m |
| C1orf21 | hsa-miR-424-5p | up.mi | down.m |
| C1orf21 | hsa-miR-708-3p | up.mi | down.m |
| C1orf21 | hsa-miR-503-5p | up.mi | down.m |
| C1orf21 | hsa-miR-136-3p | up.mi | down.m |
| C1orf21 | hsa-miR-93-3p | up.mi | down.m |
| C1orf21 | hsa-miR-186-5p | up.mi | down.m |
| C1orf87 | hsa-miR-22-3p | up.mi | down.m |
| C1orf87 | hsa-miR-301a-5p | up.mi | down.m |
| C1QTNF2 | hsa-miR-193b-3p | up.mi | down.m |
| C2 | hsa-miR-142-3p | up.mi | down.m |
| C20orf194 | hsa-miR-21-5p | up.mi | down.m |
| C20orf194 | hsa-miR-576-5p | up.mi | down.m |
| C3 | hsa-miR-589-3p | up.mi | down.m |
| C3 | hsa-miR-1306-5p | up.mi | down.m |
| C3 | hsa-miR-766-3p | up.mi | down.m |
| C3 | hsa-miR-940 | up.mi | down.m |
| C3 | hsa-miR-455-3p | up.mi | down.m |
| C3 | hsa-miR-1307-3p | up.mi | down.m |
| C3 | hsa-miR-127-5p | up.mi | down.m |
| C3 | hsa-miR-5698 | up.mi | down.m |
| C3orf58 | hsa-miR-155-5p | up.mi | down.m |
| C3orf58 | hsa-miR-148b-3p | up.mi | down.m |
| C5orf38 | hsa-miR-5698 | up.mi | down.m |
| CAB39L | hsa-miR-155-5p | up.mi | down.m |
| CAB39L | hsa-miR-192-5p | up.mi | down.m |
| CABLES1 | hsa-miR-93-5p | up.mi | down.m |
| CABLES1 | hsa-miR-20b-5p | up.mi | down.m |
| CABLES1 | hsa-miR-128-3p | up.mi | down.m |
| CABLES1 | hsa-miR-20a-5p | up.mi | down.m |
| CABLES1 | hsa-miR-106a-5p | up.mi | down.m |
| CABLES1 | hsa-miR-7-1-3p | up.mi | down.m |
| CABLES1 | hsa-miR-17-5p | up.mi | down.m |
| CABLES1 | hsa-miR-106b-5p | up.mi | down.m |
| CACHD1 | hsa-miR-185-5p | up.mi | down.m |
| CACHD1 | hsa-miR-324-5p | up.mi | down.m |
| CADM1 | hsa-miR-182-5p | up.mi | down.m |
| CADM1 | hsa-let-7g-3p | up.mi | down.m |
| CADM1 | hsa-miR-361-3p | up.mi | down.m |
| CADM1 | hsa-miR-15a-5p | up.mi | down.m |
| CADM1 | hsa-miR-192-5p | up.mi | down.m |
| CADM1 | hsa-miR-185-5p | up.mi | down.m |
| CADM1 | hsa-miR-21-5p | up.mi | down.m |
| CADM1 | hsa-let-7a-2-3p | up.mi | down.m |
| CADM3 | hsa-miR-377-3p | up.mi | down.m |
| CALD1 | hsa-miR-149-5p | up.mi | down.m |
| CALD1 | hsa-miR-21-5p | up.mi | down.m |
| CALD1 | hsa-miR-15a-5p | up.mi | down.m |
| CALD1 | hsa-miR-34a-5p | up.mi | down.m |
| CALM1 | hsa-let-7c-3p | up.mi | down.m |
| CALM1 | hsa-miR-142-3p | up.mi | down.m |
| CALM1 | hsa-miR-664a-3p | up.mi | down.m |
| CALM1 | hsa-miR-450b-5p | up.mi | down.m |
| CALM1 | hsa-miR-539-5p | up.mi | down.m |
| CALM1 | hsa-miR-382-5p | up.mi | down.m |
| CALM1 | hsa-miR-19a-3p | up.mi | down.m |
| CALM1 | hsa-miR-196a-5p | up.mi | down.m |
| CALM1 | hsa-miR-142-5p | up.mi | down.m |
| CALM1 | hsa-miR-196b-5p | up.mi | down.m |
| CALM1 | hsa-miR-625-5p | up.mi | down.m |
| CALM1 | hsa-miR-19b-3p | up.mi | down.m |
| CALM1 | hsa-miR-493-3p | up.mi | down.m |
| CALM1 | hsa-miR-21-3p | up.mi | down.m |
| CAMK2N1 | hsa-miR-452-3p | up.mi | down.m |
| CAMK2N1 | hsa-miR-224-3p | up.mi | down.m |
| CAMK2N1 | hsa-miR-590-3p | up.mi | down.m |
| CAMK2N1 | hsa-miR-2355-3p | up.mi | down.m |
| CAMK2N1 | hsa-miR-22-3p | up.mi | down.m |
| CAPS | hsa-miR-542-3p | up.mi | down.m |
| CARD6 | hsa-miR-92b-3p | up.mi | down.m |
| CARD6 | hsa-miR-92a-3p | up.mi | down.m |
| CARD6 | hsa-miR-766-3p | up.mi | down.m |
| CARD6 | hsa-miR-940 | up.mi | down.m |
| CASKIN2 | hsa-miR-1266-5p | up.mi | down.m |
| CASKIN2 | hsa-miR-31-5p | up.mi | down.m |
| CASKIN2 | hsa-miR-3127-5p | up.mi | down.m |
| CASP1 | hsa-miR-337-3p | up.mi | down.m |
| CASP1 | hsa-miR-34a-5p | up.mi | down.m |
| CASQ2 | hsa-miR-128-3p | up.mi | down.m |
| CASS4 | hsa-miR-766-3p | up.mi | down.m |
| CASS4 | hsa-miR-542-3p | up.mi | down.m |
| CASZ1 | hsa-miR-19b-3p | up.mi | down.m |
| CASZ1 | hsa-miR-151a-5p | up.mi | down.m |
| CASZ1 | hsa-miR-19a-3p | up.mi | down.m |
| CAT | hsa-miR-181b-5p | up.mi | down.m |
| CAT | hsa-miR-155-5p | up.mi | down.m |
| CAV1 | hsa-miR-17-5p | up.mi | down.m |
| CAV1 | hsa-miR-106a-5p | up.mi | down.m |
| CAV1 | hsa-miR-20a-5p | up.mi | down.m |
| CAV1 | hsa-miR-93-5p | up.mi | down.m |
| CAV1 | hsa-miR-192-5p | up.mi | down.m |
| CAV1 | hsa-miR-199a-5p | up.mi | down.m |
| CAV1 | hsa-miR-106b-5p | up.mi | down.m |
| CAV1 | hsa-miR-194-5p | up.mi | down.m |
| CAV1 | hsa-miR-20b-5p | up.mi | down.m |
| CAV1 | hsa-miR-34b-5p | up.mi | down.m |
| CAV2 | hsa-miR-93-5p | up.mi | down.m |
| CAV2 | hsa-miR-199a-3p | up.mi | down.m |
| CBFA2T3 | hsa-miR-15b-3p | up.mi | down.m |
| CBFA2T3 | hsa-miR-15a-5p | up.mi | down.m |
| CBFA2T3 | hsa-miR-937-3p | up.mi | down.m |
| CBX6 | hsa-miR-424-5p | up.mi | down.m |
| CBX6 | hsa-miR-1269b | up.mi | down.m |
| CBX6 | hsa-miR-539-5p | up.mi | down.m |
| CBX6 | hsa-miR-29b-3p | up.mi | down.m |
| CBX6 | hsa-miR-182-5p | up.mi | down.m |
| CBX6 | hsa-miR-5698 | up.mi | down.m |
| CBX6 | hsa-miR-615-3p | up.mi | down.m |
| CBX6 | hsa-miR-664a-3p | up.mi | down.m |
| CBX6 | hsa-miR-1269a | up.mi | down.m |
| CBX6 | hsa-miR-103a-2-5p | up.mi | down.m |
| CBX6 | hsa-miR-874-3p | up.mi | down.m |
| CBX6 | hsa-miR-185-5p | up.mi | down.m |
| CBX6 | hsa-miR-151a-5p | up.mi | down.m |
| CBX6 | hsa-miR-361-3p | up.mi | down.m |
| CBX6 | hsa-miR-15a-5p | up.mi | down.m |
| CBX6 | hsa-miR-2355-5p | up.mi | down.m |
| CBX6 | hsa-miR-92a-3p | up.mi | down.m |
| CBX6 | hsa-miR-642a-5p | up.mi | down.m |
| CBX7 | hsa-miR-9-3p | up.mi | down.m |
| CBX7 | hsa-miR-19b-3p | up.mi | down.m |
| CBX7 | hsa-miR-766-3p | up.mi | down.m |
| CBX7 | hsa-miR-19a-3p | up.mi | down.m |
| CBX7 | hsa-miR-181b-5p | up.mi | down.m |
| CBX7 | hsa-miR-9-5p | up.mi | down.m |
| CC2D2A | hsa-miR-92a-3p | up.mi | down.m |
| CC2D2A | hsa-miR-142-5p | up.mi | down.m |
| CC2D2A | hsa-miR-186-5p | up.mi | down.m |
| CCBE1 | hsa-miR-4326 | up.mi | down.m |
| CCBE1 | hsa-miR-2355-3p | up.mi | down.m |
| CCDC141 | hsa-miR-495-3p | up.mi | down.m |
| CCDC141 | hsa-miR-7-1-3p | up.mi | down.m |
| CCDC152 | hsa-miR-532-3p | up.mi | down.m |
| CCDC170 | hsa-miR-744-3p | up.mi | down.m |
| CCDC170 | hsa-miR-143-5p | up.mi | down.m |
| CCDC170 | hsa-miR-1307-3p | up.mi | down.m |
| CCDC50 | hsa-miR-5698 | up.mi | down.m |
| CCDC65 | hsa-miR-616-5p | up.mi | down.m |
| CCDC68 | hsa-miR-940 | up.mi | down.m |
| CCDC69 | hsa-miR-377-3p | up.mi | down.m |
| CCDC8 | hsa-miR-766-3p | up.mi | down.m |
| CCDC8 | hsa-miR-193a-3p | up.mi | down.m |
| CCL2 | hsa-miR-155-5p | up.mi | down.m |
| CCL2 | hsa-let-7g-3p | up.mi | down.m |
| CCL2 | hsa-miR-495-3p | up.mi | down.m |
| CCND2 | hsa-miR-19a-3p | up.mi | down.m |
| CCND2 | hsa-miR-503-5p | up.mi | down.m |
| CCND2 | hsa-miR-185-5p | up.mi | down.m |
| CCND2 | hsa-miR-29b-3p | up.mi | down.m |
| CCND2 | hsa-miR-424-5p | up.mi | down.m |
| CCND2 | hsa-miR-17-5p | up.mi | down.m |
| CCND2 | hsa-miR-4668-3p | up.mi | down.m |
| CCND2 | hsa-miR-16-1-3p | up.mi | down.m |
| CCND2 | hsa-miR-20a-5p | up.mi | down.m |
| CCND2 | hsa-miR-615-3p | up.mi | down.m |
| CCND2 | hsa-miR-15a-5p | up.mi | down.m |
| CCND2 | hsa-miR-301a-3p | up.mi | down.m |
| CCND2 | hsa-miR-19b-3p | up.mi | down.m |
| CCND2 | hsa-miR-96-5p | up.mi | down.m |
| CCND2 | hsa-miR-196a-5p | up.mi | down.m |
| CCND2 | hsa-miR-106b-5p | up.mi | down.m |
| CCND2 | hsa-miR-382-5p | up.mi | down.m |
| CCND2 | hsa-miR-192-3p | up.mi | down.m |
| CCND2 | hsa-miR-182-5p | up.mi | down.m |
| CCND2 | hsa-miR-301b-3p | up.mi | down.m |
| CCND2 | hsa-miR-130b-3p | up.mi | down.m |
| CCND2 | hsa-miR-324-3p | up.mi | down.m |
| CCND2 | hsa-miR-454-3p | up.mi | down.m |
| CCND2 | hsa-miR-154-5p | up.mi | down.m |
| CCND2 | hsa-miR-155-5p | up.mi | down.m |
| CCND2 | hsa-miR-744-3p | up.mi | down.m |
| CCND2 | hsa-miR-130a-3p | up.mi | down.m |
| CCND2 | hsa-miR-505-3p | up.mi | down.m |
| CCND2 | hsa-miR-191-5p | up.mi | down.m |
| CCND2 | hsa-miR-340-3p | up.mi | down.m |
| CCND3 | hsa-miR-615-3p | up.mi | down.m |
| CCND3 | hsa-miR-34a-5p | up.mi | down.m |
| CCND3 | hsa-miR-708-5p | up.mi | down.m |
| CCND3 | hsa-miR-15b-3p | up.mi | down.m |
| CCND3 | hsa-miR-28-5p | up.mi | down.m |
| CCND3 | hsa-miR-324-5p | up.mi | down.m |
| CCND3 | hsa-miR-424-5p | up.mi | down.m |
| CCND3 | hsa-miR-503-5p | up.mi | down.m |
| CCR1 | hsa-miR-21-5p | up.mi | down.m |
| CCRL2 | hsa-miR-1306-5p | up.mi | down.m |
| CD1C | hsa-miR-381-3p | up.mi | down.m |
| CD274 | hsa-miR-708-5p | up.mi | down.m |
| CD274 | hsa-miR-940 | up.mi | down.m |
| CD274 | hsa-miR-324-5p | up.mi | down.m |
| CD274 | hsa-miR-34a-3p | up.mi | down.m |
| CD274 | hsa-miR-194-5p | up.mi | down.m |
| CD274 | hsa-miR-15a-5p | up.mi | down.m |
| CD274 | hsa-miR-34b-5p | up.mi | down.m |
| CD274 | hsa-miR-424-5p | up.mi | down.m |
| CD274 | hsa-miR-200b-3p | up.mi | down.m |
| CD274 | hsa-miR-103a-2-5p | up.mi | down.m |
| CD300E | hsa-miR-744-3p | up.mi | down.m |
| CD300E | hsa-miR-615-3p | up.mi | down.m |
| CD300E | hsa-miR-301a-5p | up.mi | down.m |
| CD302 | hsa-miR-16-2-3p | up.mi | down.m |
| CD34 | hsa-miR-9-5p | up.mi | down.m |
| CD34 | hsa-miR-106b-5p | up.mi | down.m |
| CD34 | hsa-miR-377-3p | up.mi | down.m |
| CD36 | hsa-miR-455-5p | up.mi | down.m |
| CD36 | hsa-miR-155-5p | up.mi | down.m |
| CD4 | hsa-miR-769-3p | up.mi | down.m |
| CD40 | hsa-miR-224-5p | up.mi | down.m |
| CD40 | hsa-miR-503-5p | up.mi | down.m |
| CD40 | hsa-miR-34a-5p | up.mi | down.m |
| CD44 | hsa-miR-199a-3p | up.mi | down.m |
| CD44 | hsa-miR-34a-5p | up.mi | down.m |
| CD44 | hsa-miR-199a-5p | up.mi | down.m |
| CD44 | hsa-miR-708-5p | up.mi | down.m |
| CD44 | hsa-miR-130b-5p | up.mi | down.m |
| CD44 | hsa-miR-188-5p | up.mi | down.m |
| CD44 | hsa-miR-192-3p | up.mi | down.m |
| CD47 | hsa-miR-17-5p | up.mi | down.m |
| CD47 | hsa-miR-505-3p | up.mi | down.m |
| CD47 | hsa-miR-21-5p | up.mi | down.m |
| CD47 | hsa-miR-20a-5p | up.mi | down.m |
| CD47 | hsa-miR-142-5p | up.mi | down.m |
| CD47 | hsa-miR-106b-5p | up.mi | down.m |
| CD47 | hsa-miR-192-5p | up.mi | down.m |
| CD47 | hsa-miR-625-3p | up.mi | down.m |
| CD47 | hsa-miR-93-5p | up.mi | down.m |
| CD47 | hsa-miR-20b-5p | up.mi | down.m |
| CD55 | hsa-miR-3200-3p | up.mi | down.m |
| CD55 | hsa-miR-542-3p | up.mi | down.m |
| CD55 | hsa-miR-136-5p | up.mi | down.m |
| CD55 | hsa-miR-192-3p | up.mi | down.m |
| CD55 | hsa-miR-142-5p | up.mi | down.m |
| CD55 | hsa-miR-130b-5p | up.mi | down.m |
| CD55 | hsa-miR-664a-3p | up.mi | down.m |
| CD59 | hsa-miR-92a-3p | up.mi | down.m |
| CD68 | hsa-miR-155-5p | up.mi | down.m |
| CD68 | hsa-miR-629-3p | up.mi | down.m |
| CD69 | hsa-miR-92b-3p | up.mi | down.m |
| CD69 | hsa-miR-92a-3p | up.mi | down.m |
| CD81 | hsa-miR-155-5p | up.mi | down.m |
| CD81 | hsa-miR-582-5p | up.mi | down.m |
| CD81 | hsa-miR-769-3p | up.mi | down.m |
| CD82 | hsa-miR-130b-5p | up.mi | down.m |
| CD83 | hsa-miR-215-5p | up.mi | down.m |
| CD83 | hsa-miR-192-5p | up.mi | down.m |
| CD86 | hsa-miR-130b-5p | up.mi | down.m |
| CD9 | hsa-miR-3677-5p | up.mi | down.m |
| CD9 | hsa-miR-495-3p | up.mi | down.m |
| CD93 | hsa-miR-377-3p | up.mi | down.m |
| CDC14A | hsa-miR-192-5p | up.mi | down.m |
| CDC14A | hsa-miR-628-3p | up.mi | down.m |
| CDC14A | hsa-miR-301a-3p | up.mi | down.m |
| CDC14A | hsa-miR-424-5p | up.mi | down.m |
| CDC14A | hsa-miR-503-5p | up.mi | down.m |
| CDC14A | hsa-miR-215-5p | up.mi | down.m |
| CDH13 | hsa-miR-31-3p | up.mi | down.m |
| CDH13 | hsa-miR-155-5p | up.mi | down.m |
| CDH5 | hsa-miR-128-3p | up.mi | down.m |
| CDHR3 | hsa-miR-331-3p | up.mi | down.m |
| CDK14 | hsa-miR-185-5p | up.mi | down.m |
| CDK14 | hsa-miR-215-5p | up.mi | down.m |
| CDK14 | hsa-miR-192-5p | up.mi | down.m |
| CDK14 | hsa-miR-493-5p | up.mi | down.m |
| CDKN1A | hsa-miR-505-5p | up.mi | down.m |
| CDKN1A | hsa-miR-17-5p | up.mi | down.m |
| CDKN1A | hsa-miR-589-3p | up.mi | down.m |
| CDKN1A | hsa-miR-22-3p | up.mi | down.m |
| CDKN1A | hsa-miR-93-5p | up.mi | down.m |
| CDKN1A | hsa-miR-96-5p | up.mi | down.m |
| CDKN1A | hsa-miR-20a-5p | up.mi | down.m |
| CDKN1A | hsa-miR-182-5p | up.mi | down.m |
| CDKN1A | hsa-miR-582-5p | up.mi | down.m |
| CDKN1A | hsa-miR-106b-5p | up.mi | down.m |
| CDKN1A | hsa-miR-106a-5p | up.mi | down.m |
| CDKN1A | hsa-miR-423-3p | up.mi | down.m |
| CDKN1A | hsa-miR-212-3p | up.mi | down.m |
| CDKN1A | hsa-miR-493-5p | up.mi | down.m |
| CDKN1A | hsa-miR-503-5p | up.mi | down.m |
| CDKN1A | hsa-miR-15a-5p | up.mi | down.m |
| CDKN1A | hsa-miR-148b-3p | up.mi | down.m |
| CDKN1A | hsa-miR-3170 | up.mi | down.m |
| CDKN1A | hsa-miR-455-3p | up.mi | down.m |
| CDKN1A | hsa-miR-616-5p | up.mi | down.m |
| CDKN1A | hsa-miR-20b-5p | up.mi | down.m |
| CDKN1A | hsa-miR-708-5p | up.mi | down.m |
| CDKN1A | hsa-miR-2355-5p | up.mi | down.m |
| CDKN1A | hsa-miR-767-3p | up.mi | down.m |
| CDKN1A | hsa-miR-542-3p | up.mi | down.m |
| CDKN1A | hsa-miR-148a-3p | up.mi | down.m |
| CDKN1A | hsa-miR-4724-5p | up.mi | down.m |
| CDKN1A | hsa-miR-196a-5p | up.mi | down.m |
| CDKN1A | hsa-miR-576-5p | up.mi | down.m |
| CDKN1A | hsa-miR-28-5p | up.mi | down.m |
| CDKN1A | hsa-miR-345-5p | up.mi | down.m |
| CDKN1A | hsa-miR-370-3p | up.mi | down.m |
| CDKN1C | hsa-miR-199a-5p | up.mi | down.m |
| CDKN1C | hsa-miR-92b-3p | up.mi | down.m |
| CDKN2B | hsa-miR-15a-5p | up.mi | down.m |
| CDON | hsa-miR-96-5p | up.mi | down.m |
| CDON | hsa-miR-192-5p | up.mi | down.m |
| CDON | hsa-miR-34a-5p | up.mi | down.m |
| CDON | hsa-miR-889-3p | up.mi | down.m |
| CDON | hsa-miR-130b-5p | up.mi | down.m |
| CEBPD | hsa-miR-455-3p | up.mi | down.m |
| CEBPD | hsa-miR-103a-2-5p | up.mi | down.m |
| CELF2 | hsa-miR-425-5p | up.mi | down.m |
| CELF2 | hsa-miR-642a-5p | up.mi | down.m |
| CELF2 | hsa-miR-192-3p | up.mi | down.m |
| CELF2 | hsa-miR-324-5p | up.mi | down.m |
| CFAP43 | hsa-miR-331-3p | up.mi | down.m |
| CFL2 | hsa-miR-20a-5p | up.mi | down.m |
| CFL2 | hsa-miR-17-5p | up.mi | down.m |
| CFL2 | hsa-miR-130b-3p | up.mi | down.m |
| CFL2 | hsa-miR-155-5p | up.mi | down.m |
| CFL2 | hsa-miR-106b-5p | up.mi | down.m |
| CFL2 | hsa-miR-130a-3p | up.mi | down.m |
| CFL2 | hsa-miR-93-5p | up.mi | down.m |
| CFL2 | hsa-miR-215-5p | up.mi | down.m |
| CFL2 | hsa-miR-19b-3p | up.mi | down.m |
| CFL2 | hsa-miR-106a-5p | up.mi | down.m |
| CFL2 | hsa-miR-19a-3p | up.mi | down.m |
| CFL2 | hsa-miR-20b-5p | up.mi | down.m |
| CFL2 | hsa-miR-301b-3p | up.mi | down.m |
| CFL2 | hsa-miR-200c-3p | up.mi | down.m |
| CFL2 | hsa-miR-301a-3p | up.mi | down.m |
| CFL2 | hsa-miR-940 | up.mi | down.m |
| CFL2 | hsa-miR-454-3p | up.mi | down.m |
| CFL2 | hsa-miR-192-5p | up.mi | down.m |
| CFLAR | hsa-miR-34a-5p | up.mi | down.m |
| CFLAR | hsa-miR-629-5p | up.mi | down.m |
| CGNL1 | hsa-miR-215-5p | up.mi | down.m |
| CGNL1 | hsa-miR-3913-5p | up.mi | down.m |
| CGNL1 | hsa-miR-340-3p | up.mi | down.m |
| CGNL1 | hsa-miR-130b-5p | up.mi | down.m |
| CGNL1 | hsa-miR-940 | up.mi | down.m |
| CGNL1 | hsa-miR-192-5p | up.mi | down.m |
| CHPT1 | hsa-miR-192-5p | up.mi | down.m |
| CHPT1 | hsa-miR-215-5p | up.mi | down.m |
| CHRDL1 | hsa-miR-185-5p | up.mi | down.m |
| CHRDL1 | hsa-miR-155-5p | up.mi | down.m |
| CHRDL1 | hsa-miR-4677-3p | up.mi | down.m |
| CHRDL1 | hsa-miR-758-3p | up.mi | down.m |
| CHST9 | hsa-miR-92a-3p | up.mi | down.m |
| CHURC1 | hsa-miR-17-5p | up.mi | down.m |
| CHURC1 | hsa-miR-93-5p | up.mi | down.m |
| CHURC1 | hsa-miR-20a-5p | up.mi | down.m |
| CHURC1 | hsa-miR-20b-5p | up.mi | down.m |
| CHURC1 | hsa-miR-155-5p | up.mi | down.m |
| CHURC1 | hsa-miR-106b-5p | up.mi | down.m |
| CITED2 | hsa-miR-493-5p | up.mi | down.m |
| CITED2 | hsa-let-7g-3p | up.mi | down.m |
| CITED2 | hsa-let-7a-2-3p | up.mi | down.m |
| CITED2 | hsa-miR-182-5p | up.mi | down.m |
| CITED2 | hsa-miR-493-3p | up.mi | down.m |
| CITED2 | hsa-let-7c-3p | up.mi | down.m |
| CITED2 | hsa-miR-590-3p | up.mi | down.m |
| CKB | hsa-miR-324-5p | up.mi | down.m |
| CKB | hsa-miR-92a-3p | up.mi | down.m |
| CKB | hsa-miR-196b-5p | up.mi | down.m |
| CLDN5 | hsa-miR-128-3p | up.mi | down.m |
| CLEC12A | hsa-miR-154-3p | up.mi | down.m |
| CLEC14A | hsa-miR-577 | up.mi | down.m |
| CLEC4E | hsa-miR-655-3p | up.mi | down.m |
| CLEC4E | hsa-let-7c-3p | up.mi | down.m |
| CLIC4 | hsa-miR-493-3p | up.mi | down.m |
| CLIC4 | hsa-miR-301a-3p | up.mi | down.m |
| CLIC4 | hsa-miR-19a-3p | up.mi | down.m |
| CLIC4 | hsa-miR-181b-3p | up.mi | down.m |
| CLIC4 | hsa-miR-155-5p | up.mi | down.m |
| CLIC4 | hsa-miR-19b-3p | up.mi | down.m |
| CLIC4 | hsa-miR-577 | up.mi | down.m |
| CLIC4 | hsa-miR-142-3p | up.mi | down.m |
| CLIC4 | hsa-miR-106b-5p | up.mi | down.m |
| CLIC4 | hsa-miR-93-5p | up.mi | down.m |
| CLIC4 | hsa-miR-454-3p | up.mi | down.m |
| CLIC4 | hsa-miR-455-3p | up.mi | down.m |
| CLIC4 | hsa-miR-20b-5p | up.mi | down.m |
| CLIC4 | hsa-miR-106a-5p | up.mi | down.m |
| CLIC4 | hsa-miR-17-5p | up.mi | down.m |
| CLIC4 | hsa-miR-539-5p | up.mi | down.m |
| CLIC4 | hsa-miR-130b-3p | up.mi | down.m |
| CLIC4 | hsa-miR-193b-3p | up.mi | down.m |
| CLIC4 | hsa-miR-130a-3p | up.mi | down.m |
| CLIC4 | hsa-miR-20a-5p | up.mi | down.m |
| CLIC4 | hsa-miR-301b-3p | up.mi | down.m |
| CLIC5 | hsa-miR-532-3p | up.mi | down.m |
| CLIC5 | hsa-miR-642a-5p | up.mi | down.m |
| CLIC5 | hsa-miR-34a-5p | up.mi | down.m |
| CLIC5 | hsa-miR-449a | up.mi | down.m |
| CLIC5 | hsa-miR-105-5p | up.mi | down.m |
| CLIC5 | hsa-miR-539-5p | up.mi | down.m |
| CLIC5 | hsa-miR-628-5p | up.mi | down.m |
| CLIC5 | hsa-miR-335-3p | up.mi | down.m |
| CLIP4 | hsa-miR-93-5p | up.mi | down.m |
| CLIP4 | hsa-miR-215-5p | up.mi | down.m |
| CLIP4 | hsa-miR-323b-3p | up.mi | down.m |
| CLIP4 | hsa-miR-15a-5p | up.mi | down.m |
| CLIP4 | hsa-miR-21-5p | up.mi | down.m |
| CLIP4 | hsa-miR-192-5p | up.mi | down.m |
| CLIP4 | hsa-miR-424-5p | up.mi | down.m |
| CLIP4 | hsa-miR-577 | up.mi | down.m |
| CLIP4 | hsa-miR-106a-5p | up.mi | down.m |
| CLIP4 | hsa-miR-106b-5p | up.mi | down.m |
| CLIP4 | hsa-miR-20b-5p | up.mi | down.m |
| CLIP4 | hsa-miR-20a-5p | up.mi | down.m |
| CLIP4 | hsa-miR-21-3p | up.mi | down.m |
| CLIP4 | hsa-miR-17-5p | up.mi | down.m |
| CLU | hsa-miR-17-5p | up.mi | down.m |
| CLU | hsa-miR-425-5p | up.mi | down.m |
| CLU | hsa-miR-15a-5p | up.mi | down.m |
| CLU | hsa-miR-424-5p | up.mi | down.m |
| CLU | hsa-miR-21-5p | up.mi | down.m |
| CMKLR1 | hsa-miR-629-3p | up.mi | down.m |
| CMKLR1 | hsa-miR-532-3p | up.mi | down.m |
| CMKLR1 | hsa-miR-1269a | up.mi | down.m |
| CMKLR1 | hsa-miR-1269b | up.mi | down.m |
| CMKLR1 | hsa-miR-9-5p | up.mi | down.m |
| CMTM2 | hsa-miR-9-5p | up.mi | down.m |
| CNKSR2 | hsa-miR-381-3p | up.mi | down.m |
| CNKSR2 | hsa-miR-758-3p | up.mi | down.m |
| CNN3 | hsa-miR-651-5p | up.mi | down.m |
| CNN3 | hsa-miR-142-3p | up.mi | down.m |
| CNN3 | hsa-miR-577 | up.mi | down.m |
| CNN3 | hsa-miR-28-5p | up.mi | down.m |
| CNN3 | hsa-miR-15a-5p | up.mi | down.m |
| CNN3 | hsa-miR-337-3p | up.mi | down.m |
| CNTFR | hsa-miR-708-5p | up.mi | down.m |
| COBLL1 | hsa-miR-200a-5p | up.mi | down.m |
| COBLL1 | hsa-miR-192-5p | up.mi | down.m |
| COBLL1 | hsa-miR-215-5p | up.mi | down.m |
| COBLL1 | hsa-miR-200b-5p | up.mi | down.m |
| COBLL1 | hsa-miR-21-5p | up.mi | down.m |
| COBLL1 | hsa-miR-224-3p | up.mi | down.m |
| COL13A1 | hsa-miR-940 | up.mi | down.m |
| COL13A1 | hsa-miR-744-3p | up.mi | down.m |
| COL4A3BP | hsa-miR-194-5p | up.mi | down.m |
| COL4A3BP | hsa-miR-660-5p | up.mi | down.m |
| COL4A3BP | hsa-miR-629-3p | up.mi | down.m |
| COL4A3BP | hsa-miR-455-3p | up.mi | down.m |
| COL4A3BP | hsa-miR-3913-5p | up.mi | down.m |
| COL4A4 | hsa-miR-130b-5p | up.mi | down.m |
| COL4A5 | hsa-miR-199b-3p | up.mi | down.m |
| COL4A5 | hsa-miR-29b-3p | up.mi | down.m |
| COL4A5 | hsa-miR-199a-3p | up.mi | down.m |
| COLEC10 | hsa-miR-29b-3p | up.mi | down.m |
| COLEC10 | hsa-miR-450b-5p | up.mi | down.m |
| COLEC10 | hsa-miR-628-5p | up.mi | down.m |
| COLEC10 | hsa-miR-664a-3p | up.mi | down.m |
| COLEC10 | hsa-miR-103a-2-5p | up.mi | down.m |
| COLEC12 | hsa-miR-148b-3p | up.mi | down.m |
| COLEC12 | hsa-miR-335-3p | up.mi | down.m |
| COLEC12 | hsa-miR-148a-3p | up.mi | down.m |
| COLEC12 | hsa-miR-29b-2-5p | up.mi | down.m |
| COLEC12 | hsa-miR-9-5p | up.mi | down.m |
| CORO1C | hsa-miR-203b-3p | up.mi | down.m |
| CORO1C | hsa-miR-19b-1-5p | up.mi | down.m |
| CORO1C | hsa-miR-1301-3p | up.mi | down.m |
| CORO1C | hsa-miR-671-5p | up.mi | down.m |
| CORO2B | hsa-miR-215-5p | up.mi | down.m |
| CORO2B | hsa-miR-185-5p | up.mi | down.m |
| CORO2B | hsa-miR-192-5p | up.mi | down.m |
| COTL1 | hsa-miR-193b-3p | up.mi | down.m |
| COTL1 | hsa-miR-501-5p | up.mi | down.m |
| COTL1 | hsa-miR-331-3p | up.mi | down.m |
| CPA3 | hsa-miR-148b-3p | up.mi | down.m |
| CPM | hsa-miR-340-3p | up.mi | down.m |
| CPM | hsa-miR-550a-5p | up.mi | down.m |
| CPM | hsa-miR-2355-5p | up.mi | down.m |
| CPM | hsa-miR-3913-5p | up.mi | down.m |
| CPM | hsa-miR-616-5p | up.mi | down.m |
| CPM | hsa-miR-21-3p | up.mi | down.m |
| CPM | hsa-miR-455-3p | up.mi | down.m |
| CPPED1 | hsa-miR-625-3p | up.mi | down.m |
| CPPED1 | hsa-miR-130b-3p | up.mi | down.m |
| CPPED1 | hsa-miR-93-5p | up.mi | down.m |
| CPPED1 | hsa-miR-19b-3p | up.mi | down.m |
| CPT1A | hsa-miR-106b-5p | up.mi | down.m |
| CPT1A | hsa-miR-370-3p | up.mi | down.m |
| CPT1A | hsa-miR-214-5p | up.mi | down.m |
| CPT1A | hsa-miR-33a-5p | up.mi | down.m |
| CPT1A | hsa-miR-106a-5p | up.mi | down.m |
| CPT1A | hsa-miR-153-5p | up.mi | down.m |
| CPT1A | hsa-miR-155-5p | up.mi | down.m |
| CPT1A | hsa-miR-20a-5p | up.mi | down.m |
| CPT1A | hsa-miR-17-5p | up.mi | down.m |
| CPT1A | hsa-miR-93-5p | up.mi | down.m |
| CPT1A | hsa-miR-20b-5p | up.mi | down.m |
| CRBN | hsa-miR-92a-3p | up.mi | down.m |
| CREBL2 | hsa-miR-18a-5p | up.mi | down.m |
| CREBL2 | hsa-miR-19a-3p | up.mi | down.m |
| CREBL2 | hsa-miR-192-5p | up.mi | down.m |
| CREBL2 | hsa-miR-503-5p | up.mi | down.m |
| CREBL2 | hsa-miR-183-3p | up.mi | down.m |
| CREBL2 | hsa-miR-15a-5p | up.mi | down.m |
| CREBL2 | hsa-miR-424-5p | up.mi | down.m |
| CREBL2 | hsa-miR-215-5p | up.mi | down.m |
| CREBL2 | hsa-miR-19b-3p | up.mi | down.m |
| CREBL2 | hsa-miR-105-5p | up.mi | down.m |
| CREBL2 | hsa-miR-186-5p | up.mi | down.m |
| CREBRF | hsa-miR-369-3p | up.mi | down.m |
| CREBRF | hsa-miR-15a-5p | up.mi | down.m |
| CREBRF | hsa-let-7c-3p | up.mi | down.m |
| CREBRF | hsa-miR-136-3p | up.mi | down.m |
| CREBRF | hsa-miR-577 | up.mi | down.m |
| CREBRF | hsa-miR-19b-3p | up.mi | down.m |
| CREBRF | hsa-miR-655-3p | up.mi | down.m |
| CREBRF | hsa-miR-381-3p | up.mi | down.m |
| CREBRF | hsa-miR-186-5p | up.mi | down.m |
| CREBRF | hsa-miR-19a-3p | up.mi | down.m |
| CREBRF | hsa-miR-155-3p | up.mi | down.m |
| CREBRF | hsa-miR-320b | up.mi | down.m |
| CREBRF | hsa-miR-93-5p | up.mi | down.m |
| CREBRF | hsa-miR-142-5p | up.mi | down.m |
| CREBRF | hsa-miR-503-5p | up.mi | down.m |
| CREBRF | hsa-miR-224-3p | up.mi | down.m |
| CREBRF | hsa-miR-424-5p | up.mi | down.m |
| CREBRF | hsa-miR-20b-3p | up.mi | down.m |
| CREBRF | hsa-miR-9-3p | up.mi | down.m |
| CREBRF | hsa-miR-155-5p | up.mi | down.m |
| CRIM1 | hsa-miR-337-3p | up.mi | down.m |
| CRIM1 | hsa-miR-20b-5p | up.mi | down.m |
| CRIM1 | hsa-miR-377-3p | up.mi | down.m |
| CRIM1 | hsa-miR-92b-3p | up.mi | down.m |
| CRIM1 | hsa-miR-424-5p | up.mi | down.m |
| CRIM1 | hsa-miR-153-5p | up.mi | down.m |
| CRIM1 | hsa-miR-15a-5p | up.mi | down.m |
| CRIM1 | hsa-miR-148b-5p | up.mi | down.m |
| CRIM1 | hsa-miR-455-3p | up.mi | down.m |
| CRIM1 | hsa-miR-193b-5p | up.mi | down.m |
| CRIM1 | hsa-miR-20a-5p | up.mi | down.m |
| CRIM1 | hsa-miR-18a-5p | up.mi | down.m |
| CRIM1 | hsa-miR-183-5p | up.mi | down.m |
| CRIP2 | hsa-miR-449a | up.mi | down.m |
| CRLS1 | hsa-miR-7-1-3p | up.mi | down.m |
| CRLS1 | hsa-miR-26b-3p | up.mi | down.m |
| CRLS1 | hsa-miR-495-3p | up.mi | down.m |
| CRLS1 | hsa-miR-130a-5p | up.mi | down.m |
| CRTAP | hsa-miR-493-3p | up.mi | down.m |
| CRTAP | hsa-miR-940 | up.mi | down.m |
| CRTAP | hsa-miR-539-5p | up.mi | down.m |
| CRY2 | hsa-miR-106b-5p | up.mi | down.m |
| CRY2 | hsa-miR-20b-5p | up.mi | down.m |
| CRY2 | hsa-miR-130b-5p | up.mi | down.m |
| CRY2 | hsa-miR-642a-5p | up.mi | down.m |
| CRY2 | hsa-miR-93-5p | up.mi | down.m |
| CRY2 | hsa-miR-20a-5p | up.mi | down.m |
| CRY2 | hsa-miR-17-5p | up.mi | down.m |
| CRY2 | hsa-miR-33a-5p | up.mi | down.m |
| CRY2 | hsa-miR-576-5p | up.mi | down.m |
| CRY2 | hsa-miR-130b-3p | up.mi | down.m |
| CRY2 | hsa-miR-532-3p | up.mi | down.m |
| CSRP1 | hsa-miR-1306-5p | up.mi | down.m |
| CSRP1 | hsa-miR-767-5p | up.mi | down.m |
| CSRP1 | hsa-miR-192-3p | up.mi | down.m |
| CSRP1 | hsa-miR-501-3p | up.mi | down.m |
| CTDSPL | hsa-miR-92b-3p | up.mi | down.m |
| CTDSPL | hsa-miR-183-5p | up.mi | down.m |
| CTDSPL | hsa-miR-92a-3p | up.mi | down.m |
| CTDSPL | hsa-miR-18a-5p | up.mi | down.m |
| CTDSPL | hsa-miR-15a-5p | up.mi | down.m |
| CTDSPL | hsa-miR-767-5p | up.mi | down.m |
| CTDSPL | hsa-miR-503-5p | up.mi | down.m |
| CTDSPL | hsa-miR-424-5p | up.mi | down.m |
| CTGF | hsa-miR-18a-5p | up.mi | down.m |
| CTGF | hsa-miR-199a-5p | up.mi | down.m |
| CTGF | hsa-miR-493-5p | up.mi | down.m |
| CTGF | hsa-miR-16-2-3p | up.mi | down.m |
| CTGF | hsa-miR-577 | up.mi | down.m |
| CTGF | hsa-miR-205-5p | up.mi | down.m |
| CTIF | hsa-miR-708-5p | up.mi | down.m |
| CTIF | hsa-miR-449a | up.mi | down.m |
| CTIF | hsa-miR-28-5p | up.mi | down.m |
| CTIF | hsa-miR-539-5p | up.mi | down.m |
| CTIF | hsa-miR-34a-5p | up.mi | down.m |
| CTSS | hsa-miR-17-5p | up.mi | down.m |
| CTSS | hsa-miR-106b-5p | up.mi | down.m |
| CTSS | hsa-miR-93-5p | up.mi | down.m |
| CTSS | hsa-miR-20a-5p | up.mi | down.m |
| CTSS | hsa-miR-20b-5p | up.mi | down.m |
| CXCL12 | hsa-miR-454-3p | up.mi | down.m |
| CXCL12 | hsa-miR-31-5p | up.mi | down.m |
| CXCL16 | hsa-miR-92a-3p | up.mi | down.m |
| CXCL16 | hsa-miR-455-3p | up.mi | down.m |
| CXCL5 | hsa-miR-186-5p | up.mi | down.m |
| CXCL5 | hsa-miR-616-3p | up.mi | down.m |
| CXCL5 | hsa-miR-28-5p | up.mi | down.m |
| CXCL5 | hsa-miR-708-5p | up.mi | down.m |
| CXCL5 | hsa-miR-148b-3p | up.mi | down.m |
| CXCL5 | hsa-miR-155-3p | up.mi | down.m |
| CXCL5 | hsa-miR-93-3p | up.mi | down.m |
| CXCL5 | hsa-miR-369-3p | up.mi | down.m |
| CXCL5 | hsa-miR-495-3p | up.mi | down.m |
| CXCR2 | hsa-miR-22-3p | up.mi | down.m |
| CXCR2 | hsa-miR-29a-5p | up.mi | down.m |
| CXorf36 | hsa-miR-203b-3p | up.mi | down.m |
| CXorf36 | hsa-miR-590-3p | up.mi | down.m |
| CXorf36 | hsa-miR-5698 | up.mi | down.m |
| CXorf36 | hsa-miR-193b-5p | up.mi | down.m |
| CXorf36 | hsa-miR-3170 | up.mi | down.m |
| CXorf57 | hsa-miR-192-5p | up.mi | down.m |
| CXorf57 | hsa-miR-215-5p | up.mi | down.m |
| CYB5A | hsa-miR-93-5p | up.mi | down.m |
| CYB5A | hsa-miR-106a-5p | up.mi | down.m |
| CYB5A | hsa-miR-20b-5p | up.mi | down.m |
| CYB5A | hsa-miR-106b-5p | up.mi | down.m |
| CYB5A | hsa-miR-17-5p | up.mi | down.m |
| CYB5A | hsa-miR-20a-5p | up.mi | down.m |
| CYB5R3 | hsa-miR-92a-3p | up.mi | down.m |
| CYB5R3 | hsa-miR-767-3p | up.mi | down.m |
| CYBB | hsa-miR-148b-3p | up.mi | down.m |
| CYBB | hsa-miR-34a-5p | up.mi | down.m |
| CYBB | hsa-miR-106b-5p | up.mi | down.m |
| CYBRD1 | hsa-miR-92a-3p | up.mi | down.m |
| CYBRD1 | hsa-miR-93-5p | up.mi | down.m |
| CYBRD1 | hsa-miR-4652-5p | up.mi | down.m |
| CYBRD1 | hsa-miR-20a-5p | up.mi | down.m |
| CYBRD1 | hsa-miR-301a-3p | up.mi | down.m |
| CYBRD1 | hsa-miR-616-5p | up.mi | down.m |
| CYBRD1 | hsa-miR-589-5p | up.mi | down.m |
| CYBRD1 | hsa-miR-17-5p | up.mi | down.m |
| CYBRD1 | hsa-miR-106b-5p | up.mi | down.m |
| CYBRD1 | hsa-miR-20b-5p | up.mi | down.m |
| CYBRD1 | hsa-miR-21-5p | up.mi | down.m |
| CYGB | hsa-miR-7705 | up.mi | down.m |
| CYP1A1 | hsa-miR-429 | up.mi | down.m |
| CYP1A1 | hsa-miR-4677-3p | up.mi | down.m |
| CYP1A1 | hsa-miR-655-3p | up.mi | down.m |
| CYP1A1 | hsa-miR-200b-3p | up.mi | down.m |
| CYP1A1 | hsa-miR-200c-3p | up.mi | down.m |
| CYP1A1 | hsa-miR-155-5p | up.mi | down.m |
| CYP1A2 | hsa-miR-766-3p | up.mi | down.m |
| CYP2U1 | hsa-miR-192-3p | up.mi | down.m |
| CYP2U1 | hsa-miR-19b-3p | up.mi | down.m |
| CYP2U1 | hsa-miR-155-5p | up.mi | down.m |
| CYP2U1 | hsa-miR-19a-3p | up.mi | down.m |
| CYP2U1 | hsa-miR-501-5p | up.mi | down.m |
| CYP4V2 | hsa-miR-192-5p | up.mi | down.m |
| CYP4V2 | hsa-miR-92a-3p | up.mi | down.m |
| CYP4V2 | hsa-miR-21-5p | up.mi | down.m |
| CYP51A1 | hsa-miR-155-5p | up.mi | down.m |
| CYP51A1 | hsa-miR-3913-5p | up.mi | down.m |
| CYP51A1 | hsa-miR-214-5p | up.mi | down.m |
| CYP51A1 | hsa-miR-34a-5p | up.mi | down.m |
| CYR61 | hsa-miR-155-5p | up.mi | down.m |
| CYR61 | hsa-miR-142-3p | up.mi | down.m |
| CYR61 | hsa-miR-22-3p | up.mi | down.m |
| CYR61 | hsa-miR-455-3p | up.mi | down.m |
| CYR61 | hsa-miR-205-5p | up.mi | down.m |
| CYR61 | hsa-miR-20a-3p | up.mi | down.m |
| CYR61 | hsa-miR-29a-5p | up.mi | down.m |
| CYSTM1 | hsa-miR-183-3p | up.mi | down.m |
| CYYR1 | hsa-miR-130b-5p | up.mi | down.m |
| CYYR1 | hsa-miR-629-3p | up.mi | down.m |
| DAAM2 | hsa-miR-18a-5p | up.mi | down.m |
| DAAM2 | hsa-miR-589-3p | up.mi | down.m |
| DAAM2 | hsa-miR-19b-3p | up.mi | down.m |
| DAAM2 | hsa-miR-3136-5p | up.mi | down.m |
| DAB2 | hsa-miR-874-3p | up.mi | down.m |
| DAB2 | hsa-miR-106b-5p | up.mi | down.m |
| DAB2 | hsa-miR-93-5p | up.mi | down.m |
| DAB2 | hsa-miR-4677-3p | up.mi | down.m |
| DAB2 | hsa-miR-93-3p | up.mi | down.m |
| DAB2IP | hsa-miR-92b-3p | up.mi | down.m |
| DAB2IP | hsa-miR-4652-5p | up.mi | down.m |
| DAB2IP | hsa-miR-92a-3p | up.mi | down.m |
| DACT3 | hsa-miR-31-5p | up.mi | down.m |
| DAPK1 | hsa-miR-130b-3p | up.mi | down.m |
| DAPK1 | hsa-miR-34a-5p | up.mi | down.m |
| DAPK1 | hsa-miR-301a-3p | up.mi | down.m |
| DAPK1 | hsa-miR-301b-3p | up.mi | down.m |
| DAPK1 | hsa-miR-454-3p | up.mi | down.m |
| DAPK1 | hsa-miR-130a-3p | up.mi | down.m |
| DAPK2 | hsa-miR-576-5p | up.mi | down.m |
| DCC | hsa-miR-19b-3p | up.mi | down.m |
| DCC | hsa-miR-889-3p | up.mi | down.m |
| DCC | hsa-miR-19a-3p | up.mi | down.m |
| DCC | hsa-miR-105-5p | up.mi | down.m |
| DCC | hsa-miR-17-3p | up.mi | down.m |
| DCDC2 | hsa-miR-501-5p | up.mi | down.m |
| DCDC2 | hsa-miR-616-5p | up.mi | down.m |
| DCDC2 | hsa-miR-592 | up.mi | down.m |
| DCDC2 | hsa-miR-362-5p | up.mi | down.m |
| DDO | hsa-miR-153-5p | up.mi | down.m |
| DDR2 | hsa-miR-21-5p | up.mi | down.m |
| DDR2 | hsa-miR-3136-5p | up.mi | down.m |
| DENND2A | hsa-miR-185-5p | up.mi | down.m |
| DENND4C | hsa-miR-590-3p | up.mi | down.m |
| DENND5A | hsa-miR-92a-3p | up.mi | down.m |
| DHCR24 | hsa-miR-642a-5p | up.mi | down.m |
| DHCR24 | hsa-miR-155-5p | up.mi | down.m |
| DHCR24 | hsa-miR-532-3p | up.mi | down.m |
| DHCR24 | hsa-miR-92a-3p | up.mi | down.m |
| DHCR24 | hsa-miR-215-5p | up.mi | down.m |
| DHCR24 | hsa-miR-192-5p | up.mi | down.m |
| DHCR24 | hsa-miR-130b-5p | up.mi | down.m |
| DHCR24 | hsa-miR-191-5p | up.mi | down.m |
| DHCR24 | hsa-miR-205-5p | up.mi | down.m |
| DIAPH2 | hsa-miR-17-3p | up.mi | down.m |
| DIAPH2 | hsa-miR-20a-5p | up.mi | down.m |
| DIXDC1 | hsa-miR-582-5p | up.mi | down.m |
| DIXDC1 | hsa-miR-582-3p | up.mi | down.m |
| DIXDC1 | hsa-miR-615-3p | up.mi | down.m |
| DKK3 | hsa-miR-582-3p | up.mi | down.m |
| DKK3 | hsa-miR-642a-5p | up.mi | down.m |
| DKK3 | hsa-miR-183-5p | up.mi | down.m |
| DKK3 | hsa-miR-590-3p | up.mi | down.m |
| DKK3 | hsa-miR-92b-3p | up.mi | down.m |
| DKK3 | hsa-miR-2355-5p | up.mi | down.m |
| DLC1 | hsa-miR-130b-3p | up.mi | down.m |
| DLC1 | hsa-miR-29b-2-5p | up.mi | down.m |
| DLC1 | hsa-miR-744-3p | up.mi | down.m |
| DLC1 | hsa-let-7g-3p | up.mi | down.m |
| DLC1 | hsa-miR-429 | up.mi | down.m |
| DLC1 | hsa-let-7a-2-3p | up.mi | down.m |
| DLC1 | hsa-miR-200c-3p | up.mi | down.m |
| DLC1 | hsa-miR-130a-3p | up.mi | down.m |
| DLC1 | hsa-miR-301b-3p | up.mi | down.m |
| DLC1 | hsa-miR-301a-3p | up.mi | down.m |
| DLC1 | hsa-miR-495-3p | up.mi | down.m |
| DLC1 | hsa-miR-200b-3p | up.mi | down.m |
| DLC1 | hsa-miR-20a-5p | up.mi | down.m |
| DLC1 | hsa-miR-186-5p | up.mi | down.m |
| DLC1 | hsa-miR-454-3p | up.mi | down.m |
| DLC1 | hsa-miR-142-5p | up.mi | down.m |
| DLC1 | hsa-miR-200a-3p | up.mi | down.m |
| DLL1 | hsa-miR-34a-5p | up.mi | down.m |
| DLL1 | hsa-miR-130b-3p | up.mi | down.m |
| DLL4 | hsa-miR-130a-3p | up.mi | down.m |
| DMD | hsa-miR-19b-1-5p | up.mi | down.m |
| DMD | hsa-miR-9-3p | up.mi | down.m |
| DMD | hsa-miR-31-5p | up.mi | down.m |
| DMD | hsa-miR-155-5p | up.mi | down.m |
| DMD | hsa-miR-21-5p | up.mi | down.m |
| DMTN | hsa-miR-93-3p | up.mi | down.m |
| DMTN | hsa-miR-149-5p | up.mi | down.m |
| DMTN | hsa-miR-3127-5p | up.mi | down.m |
| DNAH9 | hsa-miR-455-3p | up.mi | down.m |
| DNAH9 | hsa-miR-3913-5p | up.mi | down.m |
| DNAJB1 | hsa-miR-155-5p | up.mi | down.m |
| DNAJB4 | hsa-miR-431-5p | up.mi | down.m |
| DNAJB4 | hsa-miR-135b-3p | up.mi | down.m |
| DNAJB4 | hsa-miR-744-3p | up.mi | down.m |
| DNAJB4 | hsa-miR-106b-5p | up.mi | down.m |
| DNAJB4 | hsa-miR-17-5p | up.mi | down.m |
| DNAJB4 | hsa-miR-93-5p | up.mi | down.m |
| DNAJB4 | hsa-miR-20b-5p | up.mi | down.m |
| DNAJB4 | hsa-miR-192-5p | up.mi | down.m |
| DNAJB4 | hsa-miR-142-3p | up.mi | down.m |
| DNAJB4 | hsa-miR-7-1-3p | up.mi | down.m |
| DNAJB4 | hsa-miR-20a-5p | up.mi | down.m |
| DNAJB4 | hsa-miR-148b-3p | up.mi | down.m |
| DNAJB4 | hsa-miR-215-5p | up.mi | down.m |
| DNAJB4 | hsa-miR-577 | up.mi | down.m |
| DNAJB4 | hsa-miR-105-5p | up.mi | down.m |
| DNAJB4 | hsa-miR-31-3p | up.mi | down.m |
| DNAJB4 | hsa-miR-15b-3p | up.mi | down.m |
| DNAJB4 | hsa-miR-183-3p | up.mi | down.m |
| DNAJB4 | hsa-miR-224-3p | up.mi | down.m |
| DNAJB4 | hsa-miR-148a-3p | up.mi | down.m |
| DNAJB4 | hsa-miR-106a-5p | up.mi | down.m |
| DNALI1 | hsa-miR-539-5p | up.mi | down.m |
| DOCK11 | hsa-miR-143-5p | up.mi | down.m |
| DOCK11 | hsa-miR-15a-5p | up.mi | down.m |
| DOCK11 | hsa-miR-424-5p | up.mi | down.m |
| DOCK11 | hsa-miR-5698 | up.mi | down.m |
| DOCK11 | hsa-miR-455-3p | up.mi | down.m |
| DOCK11 | hsa-miR-92b-3p | up.mi | down.m |
| DOCK4 | hsa-miR-155-5p | up.mi | down.m |
| DOCK4 | hsa-miR-21-5p | up.mi | down.m |
| DOCK4 | hsa-miR-130a-5p | up.mi | down.m |
| DOCK9 | hsa-miR-889-3p | up.mi | down.m |
| DOCK9 | hsa-miR-92a-3p | up.mi | down.m |
| DOCK9 | hsa-miR-92b-3p | up.mi | down.m |
| DOK2 | hsa-miR-155-5p | up.mi | down.m |
| DOK2 | hsa-miR-193b-3p | up.mi | down.m |
| DPCR1 | hsa-miR-24-2-5p | up.mi | down.m |
| DPCR1 | hsa-miR-24-1-5p | up.mi | down.m |
| DPH3 | hsa-miR-153-5p | up.mi | down.m |
| DPT | hsa-miR-148b-3p | up.mi | down.m |
| DPYSL2 | hsa-miR-19a-3p | up.mi | down.m |
| DPYSL2 | hsa-miR-93-5p | up.mi | down.m |
| DPYSL2 | hsa-miR-130a-3p | up.mi | down.m |
| DPYSL2 | hsa-miR-301a-3p | up.mi | down.m |
| DPYSL2 | hsa-miR-106b-5p | up.mi | down.m |
| DPYSL2 | hsa-miR-20a-5p | up.mi | down.m |
| DPYSL2 | hsa-miR-17-5p | up.mi | down.m |
| DPYSL2 | hsa-miR-454-3p | up.mi | down.m |
| DPYSL2 | hsa-miR-539-5p | up.mi | down.m |
| DPYSL2 | hsa-miR-224-5p | up.mi | down.m |
| DPYSL2 | hsa-miR-106a-5p | up.mi | down.m |
| DPYSL2 | hsa-miR-301b-3p | up.mi | down.m |
| DPYSL2 | hsa-miR-130b-3p | up.mi | down.m |
| DPYSL2 | hsa-miR-425-5p | up.mi | down.m |
| DPYSL2 | hsa-miR-20b-5p | up.mi | down.m |
| DRAM1 | hsa-miR-199a-5p | up.mi | down.m |
| DRAM1 | hsa-miR-181b-5p | up.mi | down.m |
| DRAM1 | hsa-miR-181d-5p | up.mi | down.m |
| DST | hsa-miR-370-3p | up.mi | down.m |
| DST | hsa-miR-136-5p | up.mi | down.m |
| DST | hsa-miR-432-5p | up.mi | down.m |
| DST | hsa-miR-127-5p | up.mi | down.m |
| DST | hsa-miR-215-5p | up.mi | down.m |
| DST | hsa-miR-615-3p | up.mi | down.m |
| DST | hsa-miR-1301-3p | up.mi | down.m |
| DST | hsa-miR-192-5p | up.mi | down.m |
| DTHD1 | hsa-miR-369-3p | up.mi | down.m |
| DTHD1 | hsa-miR-130b-5p | up.mi | down.m |
| DUSP1 | hsa-miR-22-5p | up.mi | down.m |
| DUSP1 | hsa-miR-192-3p | up.mi | down.m |
| DUSP1 | hsa-miR-200c-3p | up.mi | down.m |
| DUSP1 | hsa-miR-940 | up.mi | down.m |
| DUSP3 | hsa-miR-4677-3p | up.mi | down.m |
| DUSP3 | hsa-miR-148b-5p | up.mi | down.m |
| DUSP3 | hsa-miR-143-5p | up.mi | down.m |
| DUSP8 | hsa-miR-21-5p | up.mi | down.m |
| DUSP8 | hsa-miR-93-5p | up.mi | down.m |
| DUSP8 | hsa-miR-369-3p | up.mi | down.m |
| DUSP8 | hsa-miR-33b-5p | up.mi | down.m |
| DUSP8 | hsa-miR-130b-3p | up.mi | down.m |
| DUSP8 | hsa-miR-186-5p | up.mi | down.m |
| DUSP8 | hsa-miR-33a-5p | up.mi | down.m |
| DYNC2H1 | hsa-miR-331-3p | up.mi | down.m |
| DYNC2H1 | hsa-miR-155-5p | up.mi | down.m |
| ECHDC1 | hsa-miR-182-5p | up.mi | down.m |
| ECHDC1 | hsa-miR-15a-5p | up.mi | down.m |
| ECHDC1 | hsa-miR-196a-5p | up.mi | down.m |
| ECHDC1 | hsa-miR-31-5p | up.mi | down.m |
| EDEM1 | hsa-miR-616-5p | up.mi | down.m |
| EDEM1 | hsa-miR-92a-3p | up.mi | down.m |
| EDEM1 | hsa-miR-92b-3p | up.mi | down.m |
| EDEM1 | hsa-miR-19b-1-5p | up.mi | down.m |
| EDEM1 | hsa-miR-1301-3p | up.mi | down.m |
| EDEM1 | hsa-miR-96-5p | up.mi | down.m |
| EDIL3 | hsa-miR-21-5p | up.mi | down.m |
| EDIL3 | hsa-miR-377-3p | up.mi | down.m |
| EDN1 | hsa-miR-33b-5p | up.mi | down.m |
| EDN1 | hsa-miR-155-5p | up.mi | down.m |
| EDN1 | hsa-miR-664a-3p | up.mi | down.m |
| EDN1 | hsa-miR-454-3p | up.mi | down.m |
| EDN1 | hsa-miR-301b-3p | up.mi | down.m |
| EDN1 | hsa-miR-450b-5p | up.mi | down.m |
| EDN1 | hsa-miR-130b-3p | up.mi | down.m |
| EDN1 | hsa-miR-199a-5p | up.mi | down.m |
| EDN1 | hsa-miR-19b-1-5p | up.mi | down.m |
| EDN1 | hsa-miR-301a-3p | up.mi | down.m |
| EDN1 | hsa-miR-33a-5p | up.mi | down.m |
| EDN1 | hsa-miR-130a-3p | up.mi | down.m |
| EDNRA | hsa-miR-224-5p | up.mi | down.m |
| EDNRA | hsa-miR-200c-3p | up.mi | down.m |
| EFEMP2 | hsa-miR-128-3p | up.mi | down.m |
| EFHB | hsa-miR-200c-5p | up.mi | down.m |
| EFHB | hsa-miR-550a-3p | up.mi | down.m |
| EFNB1 | hsa-miR-92b-3p | up.mi | down.m |
| EFNB1 | hsa-miR-92a-3p | up.mi | down.m |
| EFNB1 | hsa-miR-449a | up.mi | down.m |
| EFNB1 | hsa-miR-34b-5p | up.mi | down.m |
| EFNB1 | hsa-miR-34a-5p | up.mi | down.m |
| EFNB1 | hsa-miR-31-5p | up.mi | down.m |
| EFNB2 | hsa-miR-339-5p | up.mi | down.m |
| EFNB2 | hsa-miR-424-5p | up.mi | down.m |
| EFNB2 | hsa-miR-192-5p | up.mi | down.m |
| EFNB2 | hsa-miR-215-5p | up.mi | down.m |
| EFNB2 | hsa-miR-503-5p | up.mi | down.m |
| EFNB2 | hsa-miR-96-5p | up.mi | down.m |
| EFNB2 | hsa-miR-582-5p | up.mi | down.m |
| EFNB2 | hsa-miR-15a-5p | up.mi | down.m |
| EFNB2 | hsa-miR-20b-5p | up.mi | down.m |
| EFNB2 | hsa-miR-331-3p | up.mi | down.m |
| EFR3B | hsa-miR-22-3p | up.mi | down.m |
| EFR3B | hsa-miR-409-3p | up.mi | down.m |
| EGR1 | hsa-miR-183-5p | up.mi | down.m |
| EGR1 | hsa-miR-377-3p | up.mi | down.m |
| EGR1 | hsa-miR-215-5p | up.mi | down.m |
| EGR1 | hsa-miR-192-5p | up.mi | down.m |
| EGR1 | hsa-miR-191-5p | up.mi | down.m |
| EGR2 | hsa-miR-20a-3p | up.mi | down.m |
| EGR2 | hsa-miR-130b-3p | up.mi | down.m |
| EGR2 | hsa-miR-17-5p | up.mi | down.m |
| EGR2 | hsa-miR-93-5p | up.mi | down.m |
| EGR2 | hsa-miR-20a-5p | up.mi | down.m |
| EGR2 | hsa-miR-142-3p | up.mi | down.m |
| EGR3 | hsa-miR-503-3p | up.mi | down.m |
| EGR3 | hsa-miR-377-3p | up.mi | down.m |
| EHD1 | hsa-miR-155-5p | up.mi | down.m |
| EHD1 | hsa-miR-19a-3p | up.mi | down.m |
| EHD1 | hsa-miR-19b-3p | up.mi | down.m |
| EHD1 | hsa-miR-34a-5p | up.mi | down.m |
| EHD2 | hsa-miR-3913-5p | up.mi | down.m |
| EHD2 | hsa-miR-210-3p | up.mi | down.m |
| EHD2 | hsa-miR-214-5p | up.mi | down.m |
| EID1 | hsa-miR-377-3p | up.mi | down.m |
| ELF5 | hsa-miR-192-3p | up.mi | down.m |
| ELMO1 | hsa-miR-21-3p | up.mi | down.m |
| ELN | hsa-miR-181b-5p | up.mi | down.m |
| EMCN | hsa-miR-592 | up.mi | down.m |
| EMCN | hsa-miR-431-5p | up.mi | down.m |
| EMCN | hsa-miR-616-5p | up.mi | down.m |
| EML1 | hsa-miR-215-5p | up.mi | down.m |
| EML1 | hsa-miR-192-5p | up.mi | down.m |
| EMP1 | hsa-miR-361-3p | up.mi | down.m |
| EMP1 | hsa-miR-29b-3p | up.mi | down.m |
| EMP1 | hsa-miR-34a-5p | up.mi | down.m |
| EMP2 | hsa-miR-130b-5p | up.mi | down.m |
| EMP2 | hsa-miR-153-5p | up.mi | down.m |
| EMP2 | hsa-miR-501-3p | up.mi | down.m |
| EMP2 | hsa-miR-767-5p | up.mi | down.m |
| EMP2 | hsa-miR-214-5p | up.mi | down.m |
| EMP2 | hsa-miR-203b-3p | up.mi | down.m |
| ENPP2 | hsa-miR-767-5p | up.mi | down.m |
| ENPP2 | hsa-miR-29b-3p | up.mi | down.m |
| ENPP2 | hsa-miR-17-3p | up.mi | down.m |
| ENPP4 | hsa-miR-301b-3p | up.mi | down.m |
| ENPP4 | hsa-miR-19b-3p | up.mi | down.m |
| ENPP4 | hsa-miR-454-3p | up.mi | down.m |
| ENPP4 | hsa-miR-30e-5p | up.mi | down.m |
| ENPP4 | hsa-miR-19a-3p | up.mi | down.m |
| ENPP4 | hsa-miR-192-5p | up.mi | down.m |
| ENPP4 | hsa-miR-130b-3p | up.mi | down.m |
| ENPP4 | hsa-miR-215-5p | up.mi | down.m |
| ENPP4 | hsa-miR-130a-3p | up.mi | down.m |
| ENPP4 | hsa-miR-301a-3p | up.mi | down.m |
| ENPP4 | hsa-miR-205-5p | up.mi | down.m |
| EOGT | hsa-miR-130b-3p | up.mi | down.m |
| EOGT | hsa-miR-301a-3p | up.mi | down.m |
| EOGT | hsa-miR-301b-3p | up.mi | down.m |
| EOGT | hsa-miR-19b-3p | up.mi | down.m |
| EOGT | hsa-miR-19a-3p | up.mi | down.m |
| EOGT | hsa-miR-425-5p | up.mi | down.m |
| EOGT | hsa-miR-148b-3p | up.mi | down.m |
| EOGT | hsa-miR-130a-3p | up.mi | down.m |
| EOGT | hsa-miR-148a-3p | up.mi | down.m |
| EOGT | hsa-miR-9-5p | up.mi | down.m |
| EOGT | hsa-miR-450b-5p | up.mi | down.m |
| EOGT | hsa-miR-155-5p | up.mi | down.m |
| EOGT | hsa-miR-19b-1-5p | up.mi | down.m |
| EOGT | hsa-miR-454-3p | up.mi | down.m |
| EOGT | hsa-miR-192-5p | up.mi | down.m |
| EPAS1 | hsa-miR-20a-5p | up.mi | down.m |
| EPAS1 | hsa-miR-185-5p | up.mi | down.m |
| EPAS1 | hsa-miR-17-5p | up.mi | down.m |
| EPB41L2 | hsa-miR-34a-5p | up.mi | down.m |
| EPB41L2 | hsa-miR-331-3p | up.mi | down.m |
| EPB41L2 | hsa-miR-155-5p | up.mi | down.m |
| EPB41L2 | hsa-miR-17-5p | up.mi | down.m |
| EPB41L3 | hsa-miR-92a-3p | up.mi | down.m |
| EPB41L5 | hsa-miR-17-5p | up.mi | down.m |
| EPDR1 | hsa-miR-215-5p | up.mi | down.m |
| EPDR1 | hsa-miR-192-5p | up.mi | down.m |
| EPDR1 | hsa-miR-615-3p | up.mi | down.m |
| EPDR1 | hsa-miR-193b-3p | up.mi | down.m |
| EPDR1 | hsa-miR-369-3p | up.mi | down.m |
| EPDR1 | hsa-miR-629-3p | up.mi | down.m |
| EPHX2 | hsa-miR-130b-5p | up.mi | down.m |
| EPHX2 | hsa-miR-1306-5p | up.mi | down.m |
| EPHX2 | hsa-miR-29b-3p | up.mi | down.m |
| EPHX2 | hsa-miR-26b-3p | up.mi | down.m |
| EPM2A | hsa-miR-192-5p | up.mi | down.m |
| EPM2A | hsa-miR-21-5p | up.mi | down.m |
| EPM2A | hsa-miR-215-5p | up.mi | down.m |
| ERBB4 | hsa-miR-19a-3p | up.mi | down.m |
| ERBB4 | hsa-miR-193a-3p | up.mi | down.m |
| ETF1 | hsa-miR-1306-5p | up.mi | down.m |
| ETF1 | hsa-miR-20b-5p | up.mi | down.m |
| ETF1 | hsa-miR-205-5p | up.mi | down.m |
| ETF1 | hsa-miR-20a-5p | up.mi | down.m |
| ETF1 | hsa-miR-128-3p | up.mi | down.m |
| ETF1 | hsa-miR-106b-5p | up.mi | down.m |
| ETF1 | hsa-miR-93-5p | up.mi | down.m |
| ETF1 | hsa-miR-653-5p | up.mi | down.m |
| ETF1 | hsa-miR-34b-5p | up.mi | down.m |
| ETF1 | hsa-miR-17-5p | up.mi | down.m |
| ETF1 | hsa-miR-423-3p | up.mi | down.m |
| ETF1 | hsa-miR-940 | up.mi | down.m |
| ETS1 | hsa-miR-200c-3p | up.mi | down.m |
| ETS1 | hsa-miR-193b-3p | up.mi | down.m |
| ETS1 | hsa-miR-155-5p | up.mi | down.m |
| ETS1 | hsa-miR-31-5p | up.mi | down.m |
| ETS1 | hsa-miR-377-3p | up.mi | down.m |
| ETS1 | hsa-miR-450b-5p | up.mi | down.m |
| ETS1 | hsa-miR-181b-5p | up.mi | down.m |
| ETS1 | hsa-miR-19b-1-5p | up.mi | down.m |
| ETS1 | hsa-miR-9-5p | up.mi | down.m |
| ETS1 | hsa-miR-181d-5p | up.mi | down.m |
| ETS1 | hsa-miR-324-5p | up.mi | down.m |
| ETS1 | hsa-miR-199a-5p | up.mi | down.m |
| ETS1 | hsa-miR-200b-3p | up.mi | down.m |
| ETS2 | hsa-miR-149-5p | up.mi | down.m |
| ETS2 | hsa-miR-199a-5p | up.mi | down.m |
| ETS2 | hsa-miR-199a-3p | up.mi | down.m |
| ETS2 | hsa-miR-582-5p | up.mi | down.m |
| ETS2 | hsa-miR-196b-5p | up.mi | down.m |
| ETS2 | hsa-miR-18a-5p | up.mi | down.m |
| ETS2 | hsa-miR-455-5p | up.mi | down.m |
| ETV1 | hsa-miR-17-5p | up.mi | down.m |
| ETV1 | hsa-miR-20a-5p | up.mi | down.m |
| ETV5 | hsa-miR-200b-3p | up.mi | down.m |
| EVI2B | hsa-miR-142-3p | up.mi | down.m |
| EYA4 | hsa-miR-128-3p | up.mi | down.m |
| EYA4 | hsa-miR-148b-3p | up.mi | down.m |
| EYA4 | hsa-miR-224-5p | up.mi | down.m |
| F2RL3 | hsa-miR-106a-5p | up.mi | down.m |
| F2RL3 | hsa-miR-93-5p | up.mi | down.m |
| F2RL3 | hsa-miR-20a-5p | up.mi | down.m |
| F2RL3 | hsa-miR-20b-5p | up.mi | down.m |
| F2RL3 | hsa-miR-17-5p | up.mi | down.m |
| F2RL3 | hsa-miR-106b-5p | up.mi | down.m |
| F3 | hsa-miR-106a-5p | up.mi | down.m |
| F3 | hsa-miR-17-5p | up.mi | down.m |
| F3 | hsa-miR-224-5p | up.mi | down.m |
| F3 | hsa-miR-20a-5p | up.mi | down.m |
| F3 | hsa-miR-106b-5p | up.mi | down.m |
| F3 | hsa-miR-20b-5p | up.mi | down.m |
| F3 | hsa-miR-93-5p | up.mi | down.m |
| F3 | hsa-miR-18a-5p | up.mi | down.m |
| F3 | hsa-miR-130b-3p | up.mi | down.m |
| FADS3 | hsa-miR-3127-5p | up.mi | down.m |
| FAM105A | hsa-miR-127-5p | up.mi | down.m |
| FAM105A | hsa-miR-455-3p | up.mi | down.m |
| FAM105A | hsa-miR-136-5p | up.mi | down.m |
| FAM105A | hsa-miR-382-5p | up.mi | down.m |
| FAM107A | hsa-miR-324-3p | up.mi | down.m |
| FAM107A | hsa-miR-874-3p | up.mi | down.m |
| FAM129B | hsa-miR-3136-5p | up.mi | down.m |
| FAM129B | hsa-miR-92a-3p | up.mi | down.m |
| FAM129B | hsa-miR-134-5p | up.mi | down.m |
| FAM13B | hsa-miR-19b-1-5p | up.mi | down.m |
| FAM13B | hsa-miR-136-5p | up.mi | down.m |
| FAM13B | hsa-miR-532-3p | up.mi | down.m |
| FAM166B | hsa-miR-3934-3p | up.mi | down.m |
| FAM171A1 | hsa-miR-96-5p | up.mi | down.m |
| FAM174B | hsa-miR-93-3p | up.mi | down.m |
| FAM189A2 | hsa-miR-215-5p | up.mi | down.m |
| FAM189A2 | hsa-miR-192-5p | up.mi | down.m |
| FAM212B | hsa-miR-193b-3p | up.mi | down.m |
| FAM212B | hsa-miR-148a-3p | up.mi | down.m |
| FAM212B | hsa-miR-148b-3p | up.mi | down.m |
| FAM212B | hsa-miR-766-3p | up.mi | down.m |
| FAM212B | hsa-miR-1306-5p | up.mi | down.m |
| FAM212B | hsa-miR-2355-5p | up.mi | down.m |
| FAM216B | hsa-miR-429 | up.mi | down.m |
| FAM216B | hsa-miR-200c-3p | up.mi | down.m |
| FAM216B | hsa-miR-200b-3p | up.mi | down.m |
| FAM229B | hsa-miR-503-3p | up.mi | down.m |
| FAM229B | hsa-miR-15a-5p | up.mi | down.m |
| FAM229B | hsa-miR-501-5p | up.mi | down.m |
| FAM229B | hsa-miR-629-3p | up.mi | down.m |
| FAM229B | hsa-miR-503-5p | up.mi | down.m |
| FAM229B | hsa-miR-136-5p | up.mi | down.m |
| FAM229B | hsa-miR-335-3p | up.mi | down.m |
| FAM229B | hsa-miR-455-5p | up.mi | down.m |
| FAM229B | hsa-miR-424-5p | up.mi | down.m |
| FAM229B | hsa-miR-143-5p | up.mi | down.m |
| FAM46B | hsa-miR-769-3p | up.mi | down.m |
| FAM46B | hsa-miR-320b | up.mi | down.m |
| FAM49A | hsa-miR-92a-3p | up.mi | down.m |
| FAM49A | hsa-miR-92b-3p | up.mi | down.m |
| FAM53B | hsa-miR-130b-5p | up.mi | down.m |
| FAM53B | hsa-miR-615-3p | up.mi | down.m |
| FAM81B | hsa-miR-30e-5p | up.mi | down.m |
| FAM89A | hsa-miR-20a-5p | up.mi | down.m |
| FAM89A | hsa-miR-335-3p | up.mi | down.m |
| FAM89A | hsa-miR-505-3p | up.mi | down.m |
| FAM89A | hsa-miR-425-5p | up.mi | down.m |
| FAM89A | hsa-miR-128-3p | up.mi | down.m |
| FAM89A | hsa-miR-1287-5p | up.mi | down.m |
| FAM89A | hsa-miR-20b-5p | up.mi | down.m |
| FAM89A | hsa-miR-17-5p | up.mi | down.m |
| FAM89A | hsa-miR-106b-5p | up.mi | down.m |
| FAM89A | hsa-miR-22-5p | up.mi | down.m |
| FAM89A | hsa-miR-106a-5p | up.mi | down.m |
| FAM89A | hsa-miR-93-5p | up.mi | down.m |
| FAR2 | hsa-miR-616-5p | up.mi | down.m |
| FAS | hsa-miR-18a-5p | up.mi | down.m |
| FAS | hsa-miR-106b-5p | up.mi | down.m |
| FAS | hsa-miR-17-5p | up.mi | down.m |
| FAS | hsa-miR-196b-5p | up.mi | down.m |
| FAS | hsa-miR-21-5p | up.mi | down.m |
| FAS | hsa-miR-19b-3p | up.mi | down.m |
| FAS | hsa-miR-19a-3p | up.mi | down.m |
| FAS | hsa-miR-106a-5p | up.mi | down.m |
| FASN | hsa-miR-92a-3p | up.mi | down.m |
| FASN | hsa-miR-615-3p | up.mi | down.m |
| FASN | hsa-miR-93-5p | up.mi | down.m |
| FASN | hsa-miR-423-3p | up.mi | down.m |
| FASN | hsa-miR-424-5p | up.mi | down.m |
| FASN | hsa-miR-15b-3p | up.mi | down.m |
| FASN | hsa-miR-324-3p | up.mi | down.m |
| FASN | hsa-miR-15a-5p | up.mi | down.m |
| FASN | hsa-miR-4668-3p | up.mi | down.m |
| FASN | hsa-miR-130b-5p | up.mi | down.m |
| FASN | hsa-miR-331-3p | up.mi | down.m |
| FASN | hsa-miR-186-5p | up.mi | down.m |
| FASN | hsa-miR-17-5p | up.mi | down.m |
| FASN | hsa-miR-193b-3p | up.mi | down.m |
| FASN | hsa-miR-92b-3p | up.mi | down.m |
| FAXDC2 | hsa-miR-21-5p | up.mi | down.m |
| FBLN5 | hsa-miR-200c-3p | up.mi | down.m |
| FBLN5 | hsa-miR-128-3p | up.mi | down.m |
| FBLN5 | hsa-miR-542-3p | up.mi | down.m |
| FBXL3 | hsa-let-7c-3p | up.mi | down.m |
| FBXL3 | hsa-let-7g-3p | up.mi | down.m |
| FBXL3 | hsa-miR-18a-5p | up.mi | down.m |
| FBXL3 | hsa-miR-93-5p | up.mi | down.m |
| FBXL3 | hsa-miR-340-3p | up.mi | down.m |
| FBXL3 | hsa-miR-4724-5p | up.mi | down.m |
| FBXL3 | hsa-miR-493-5p | up.mi | down.m |
| FBXL3 | hsa-let-7a-2-3p | up.mi | down.m |
| FBXL3 | hsa-miR-186-5p | up.mi | down.m |
| FBXL5 | hsa-miR-93-5p | up.mi | down.m |
| FBXL5 | hsa-miR-130b-3p | up.mi | down.m |
| FBXL5 | hsa-miR-20a-5p | up.mi | down.m |
| FBXL5 | hsa-miR-17-5p | up.mi | down.m |
| FBXL5 | hsa-miR-106a-5p | up.mi | down.m |
| FBXL5 | hsa-miR-3934-3p | up.mi | down.m |
| FBXL5 | hsa-miR-767-3p | up.mi | down.m |
| FBXL5 | hsa-miR-20b-5p | up.mi | down.m |
| FBXL5 | hsa-miR-106b-5p | up.mi | down.m |
| FBXL5 | hsa-miR-31-3p | up.mi | down.m |
| FBXL5 | hsa-miR-331-3p | up.mi | down.m |
| FBXL5 | hsa-miR-186-5p | up.mi | down.m |
| FBXL7 | hsa-miR-20a-5p | up.mi | down.m |
| FBXL7 | hsa-miR-31-3p | up.mi | down.m |
| FBXL7 | hsa-miR-20b-5p | up.mi | down.m |
| FBXL7 | hsa-miR-106a-5p | up.mi | down.m |
| FBXL7 | hsa-miR-106b-5p | up.mi | down.m |
| FBXL7 | hsa-miR-17-5p | up.mi | down.m |
| FBXL7 | hsa-miR-93-5p | up.mi | down.m |
| FCGR2A | hsa-miR-29b-2-5p | up.mi | down.m |
| FCGR3B | hsa-miR-136-5p | up.mi | down.m |
| FERMT2 | hsa-miR-940 | up.mi | down.m |
| FERMT2 | hsa-miR-4728-3p | up.mi | down.m |
| FERMT2 | hsa-miR-21-5p | up.mi | down.m |
| FERMT2 | hsa-miR-200b-3p | up.mi | down.m |
| FERMT2 | hsa-miR-34b-5p | up.mi | down.m |
| FFAR4 | hsa-miR-501-5p | up.mi | down.m |
| FFAR4 | hsa-miR-7-1-3p | up.mi | down.m |
| FFAR4 | hsa-miR-142-5p | up.mi | down.m |
| FGD4 | hsa-miR-339-5p | up.mi | down.m |
| FGD4 | hsa-miR-431-5p | up.mi | down.m |
| FGD4 | hsa-miR-335-3p | up.mi | down.m |
| FGF10 | hsa-miR-7705 | up.mi | down.m |
| FGF10 | hsa-miR-335-3p | up.mi | down.m |
| FGF2 | hsa-miR-215-5p | up.mi | down.m |
| FGF2 | hsa-miR-424-5p | up.mi | down.m |
| FGF2 | hsa-miR-503-5p | up.mi | down.m |
| FGF2 | hsa-miR-582-5p | up.mi | down.m |
| FGF2 | hsa-miR-92a-3p | up.mi | down.m |
| FGF2 | hsa-miR-92b-3p | up.mi | down.m |
| FGF2 | hsa-miR-181c-3p | up.mi | down.m |
| FGF2 | hsa-miR-7-1-3p | up.mi | down.m |
| FGF2 | hsa-miR-376b-3p | up.mi | down.m |
| FGF2 | hsa-miR-199b-3p | up.mi | down.m |
| FGF2 | hsa-miR-495-3p | up.mi | down.m |
| FGF2 | hsa-miR-708-3p | up.mi | down.m |
| FGF2 | hsa-miR-192-5p | up.mi | down.m |
| FGF2 | hsa-miR-186-5p | up.mi | down.m |
| FGF2 | hsa-miR-3913-5p | up.mi | down.m |
| FGF2 | hsa-miR-155-5p | up.mi | down.m |
| FGF2 | hsa-miR-26b-3p | up.mi | down.m |
| FGF2 | hsa-miR-199a-3p | up.mi | down.m |
| FGF2 | hsa-miR-15a-5p | up.mi | down.m |
| FGF2 | hsa-miR-577 | up.mi | down.m |
| FGF7 | hsa-miR-155-5p | up.mi | down.m |
| FGF7 | hsa-miR-192-3p | up.mi | down.m |
| FGF7 | hsa-miR-20a-5p | up.mi | down.m |
| FGF7 | hsa-miR-15a-5p | up.mi | down.m |
| FGFR2 | hsa-miR-19b-1-5p | up.mi | down.m |
| FGFR2 | hsa-miR-186-5p | up.mi | down.m |
| FGFR3 | hsa-miR-425-5p | up.mi | down.m |
| FGFR4 | hsa-miR-5698 | up.mi | down.m |
| FGFR4 | hsa-miR-424-5p | up.mi | down.m |
| FGFR4 | hsa-miR-15a-5p | up.mi | down.m |
| FGL2 | hsa-miR-155-5p | up.mi | down.m |
| FHDC1 | hsa-miR-93-5p | up.mi | down.m |
| FHDC1 | hsa-miR-17-5p | up.mi | down.m |
| FHDC1 | hsa-miR-192-5p | up.mi | down.m |
| FHDC1 | hsa-miR-20a-5p | up.mi | down.m |
| FHDC1 | hsa-miR-106a-5p | up.mi | down.m |
| FHDC1 | hsa-miR-106b-5p | up.mi | down.m |
| FHDC1 | hsa-miR-1287-5p | up.mi | down.m |
| FHDC1 | hsa-miR-20b-5p | up.mi | down.m |
| FIG4 | hsa-miR-130a-3p | up.mi | down.m |
| FIG4 | hsa-miR-744-3p | up.mi | down.m |
| FIG4 | hsa-miR-301a-3p | up.mi | down.m |
| FIG4 | hsa-miR-377-3p | up.mi | down.m |
| FIG4 | hsa-miR-301b-3p | up.mi | down.m |
| FIG4 | hsa-miR-130b-3p | up.mi | down.m |
| FIG4 | hsa-miR-454-3p | up.mi | down.m |
| FILIP1L | hsa-miR-21-5p | up.mi | down.m |
| FKBP1B | hsa-miR-34a-5p | up.mi | down.m |
| FLI1 | hsa-miR-155-5p | up.mi | down.m |
| FLNA | hsa-miR-331-3p | up.mi | down.m |
| FLNA | hsa-miR-196a-5p | up.mi | down.m |
| FLNA | hsa-miR-92b-3p | up.mi | down.m |
| FLNA | hsa-miR-149-5p | up.mi | down.m |
| FLNA | hsa-miR-20a-5p | up.mi | down.m |
| FLNA | hsa-miR-193b-3p | up.mi | down.m |
| FLNA | hsa-miR-92a-3p | up.mi | down.m |
| FLNA | hsa-miR-324-5p | up.mi | down.m |
| FLNA | hsa-miR-200c-3p | up.mi | down.m |
| FLNA | hsa-miR-615-3p | up.mi | down.m |
| FLNA | hsa-miR-155-5p | up.mi | down.m |
| FLNA | hsa-miR-31-5p | up.mi | down.m |
| FLT4 | hsa-miR-331-3p | up.mi | down.m |
| FLVCR2 | hsa-let-7c-3p | up.mi | down.m |
| FNIP2 | hsa-miR-450b-5p | up.mi | down.m |
| FNIP2 | hsa-miR-186-5p | up.mi | down.m |
| FOLR1 | hsa-miR-29b-1-5p | up.mi | down.m |
| FOS | hsa-miR-155-5p | up.mi | down.m |
| FOS | hsa-miR-548v | up.mi | down.m |
| FOS | hsa-miR-29b-3p | up.mi | down.m |
| FOS | hsa-miR-215-5p | up.mi | down.m |
| FOS | hsa-miR-192-5p | up.mi | down.m |
| FOS | hsa-miR-196b-5p | up.mi | down.m |
| FOS | hsa-miR-493-5p | up.mi | down.m |
| FOS | hsa-miR-19b-1-5p | up.mi | down.m |
| FOS | hsa-miR-34a-5p | up.mi | down.m |
| FOS | hsa-miR-323b-3p | up.mi | down.m |
| FOS | hsa-miR-181b-5p | up.mi | down.m |
| FOS | hsa-miR-449a | up.mi | down.m |
| FOSB | hsa-miR-224-5p | up.mi | down.m |
| FOXA2 | hsa-miR-200a-3p | up.mi | down.m |
| FOXA2 | hsa-miR-199a-3p | up.mi | down.m |
| FOXA2 | hsa-miR-187-3p | up.mi | down.m |
| FOXF2 | hsa-miR-377-3p | up.mi | down.m |
| FOXF2 | hsa-miR-182-5p | up.mi | down.m |
| FOXF2 | hsa-miR-335-3p | up.mi | down.m |
| FOXN3 | hsa-miR-16-1-3p | up.mi | down.m |
| FOXN3 | hsa-miR-210-3p | up.mi | down.m |
| FOXN3 | hsa-miR-34a-5p | up.mi | down.m |
| FOXN3 | hsa-miR-92b-3p | up.mi | down.m |
| FOXN3 | hsa-miR-409-3p | up.mi | down.m |
| FOXN3 | hsa-miR-29a-5p | up.mi | down.m |
| FOXN3 | hsa-miR-449a | up.mi | down.m |
| FOXN3 | hsa-miR-425-5p | up.mi | down.m |
| FOXN3 | hsa-miR-21-5p | up.mi | down.m |
| FOXN3 | hsa-miR-335-3p | up.mi | down.m |
| FOXO1 | hsa-miR-196a-5p | up.mi | down.m |
| FOXO1 | hsa-miR-153-3p | up.mi | down.m |
| FOXO1 | hsa-miR-15a-5p | up.mi | down.m |
| FOXO1 | hsa-miR-324-5p | up.mi | down.m |
| FOXO1 | hsa-miR-183-5p | up.mi | down.m |
| FOXO1 | hsa-miR-200c-3p | up.mi | down.m |
| FOXO1 | hsa-miR-96-5p | up.mi | down.m |
| FOXO1 | hsa-miR-182-5p | up.mi | down.m |
| FOXO1 | hsa-miR-9-5p | up.mi | down.m |
| FOXO1 | hsa-miR-9-3p | up.mi | down.m |
| FOXO1 | hsa-miR-186-5p | up.mi | down.m |
| FOXO1 | hsa-miR-21-5p | up.mi | down.m |
| FOXO1 | hsa-miR-130b-5p | up.mi | down.m |
| FOXO1 | hsa-miR-370-3p | up.mi | down.m |
| FOXO1 | hsa-miR-135b-5p | up.mi | down.m |
| FOXO1 | hsa-let-7c-3p | up.mi | down.m |
| FOXO1 | hsa-miR-106b-3p | up.mi | down.m |
| FOXO3 | hsa-miR-155-5p | up.mi | down.m |
| FOXO3 | hsa-miR-9-5p | up.mi | down.m |
| FOXO3 | hsa-miR-142-5p | up.mi | down.m |
| FOXO3 | hsa-miR-671-5p | up.mi | down.m |
| FOXO3 | hsa-miR-96-5p | up.mi | down.m |
| FOXO3 | hsa-miR-590-5p | up.mi | down.m |
| FOXO3 | hsa-miR-592 | up.mi | down.m |
| FOXO3 | hsa-miR-31-5p | up.mi | down.m |
| FOXO3 | hsa-miR-21-5p | up.mi | down.m |
| FOXO3 | hsa-miR-182-5p | up.mi | down.m |
| FOXO3 | hsa-miR-93-5p | up.mi | down.m |
| FOXO3 | hsa-miR-615-3p | up.mi | down.m |
| FOXO4 | hsa-miR-615-3p | up.mi | down.m |
| FOXP1 | hsa-miR-335-3p | up.mi | down.m |
| FOXP1 | hsa-miR-105-5p | up.mi | down.m |
| FOXP1 | hsa-miR-148b-3p | up.mi | down.m |
| FOXP1 | hsa-miR-33a-5p | up.mi | down.m |
| FOXP1 | hsa-miR-19a-3p | up.mi | down.m |
| FOXP1 | hsa-miR-148a-3p | up.mi | down.m |
| FOXP1 | hsa-miR-34a-5p | up.mi | down.m |
| FOXP1 | hsa-let-7c-3p | up.mi | down.m |
| FOXP1 | hsa-miR-577 | up.mi | down.m |
| FOXP1 | hsa-miR-22-3p | up.mi | down.m |
| FOXP1 | hsa-miR-9-5p | up.mi | down.m |
| FOXP1 | hsa-miR-19b-3p | up.mi | down.m |
| FOXP1 | hsa-miR-431-5p | up.mi | down.m |
| FOXP1 | hsa-miR-27b-5p | up.mi | down.m |
| FPR1 | hsa-miR-26b-3p | up.mi | down.m |
| FPR1 | hsa-miR-337-3p | up.mi | down.m |
| FPR1 | hsa-miR-103a-2-5p | up.mi | down.m |
| FPR1 | hsa-miR-188-3p | up.mi | down.m |
| FPR1 | hsa-miR-1306-5p | up.mi | down.m |
| FPR2 | hsa-miR-616-5p | up.mi | down.m |
| FREM2 | hsa-miR-493-5p | up.mi | down.m |
| FREM2 | hsa-miR-616-5p | up.mi | down.m |
| FREM2 | hsa-miR-615-3p | up.mi | down.m |
| FRMD3 | hsa-miR-377-3p | up.mi | down.m |
| FRMD4A | hsa-miR-34a-5p | up.mi | down.m |
| FRMD4A | hsa-miR-423-3p | up.mi | down.m |
| FRMD4B | hsa-miR-9-5p | up.mi | down.m |
| FRY | hsa-miR-148b-3p | up.mi | down.m |
| FRY | hsa-miR-186-5p | up.mi | down.m |
| FRY | hsa-miR-425-5p | up.mi | down.m |
| FSTL3 | hsa-miR-9-5p | up.mi | down.m |
| FSTL3 | hsa-miR-455-3p | up.mi | down.m |
| FSTL3 | hsa-miR-361-3p | up.mi | down.m |
| FUT1 | hsa-miR-339-5p | up.mi | down.m |
| FUT1 | hsa-miR-766-3p | up.mi | down.m |
| FXYD6 | hsa-miR-377-3p | up.mi | down.m |
| FYN | hsa-let-7g-3p | up.mi | down.m |
| FYN | hsa-miR-128-3p | up.mi | down.m |
| FYN | hsa-miR-431-5p | up.mi | down.m |
| FYN | hsa-miR-106b-5p | up.mi | down.m |
| FYN | hsa-let-7a-2-3p | up.mi | down.m |
| FYN | hsa-miR-93-3p | up.mi | down.m |
| FZD4 | hsa-miR-455-3p | up.mi | down.m |
| FZD4 | hsa-miR-493-3p | up.mi | down.m |
| FZD4 | hsa-miR-199a-5p | up.mi | down.m |
| FZD4 | hsa-miR-215-5p | up.mi | down.m |
| FZD4 | hsa-miR-192-5p | up.mi | down.m |
| FZD4 | hsa-miR-20b-3p | up.mi | down.m |
| FZD5 | hsa-miR-369-3p | up.mi | down.m |
| FZD5 | hsa-miR-381-3p | up.mi | down.m |
| FZD5 | hsa-miR-7-1-3p | up.mi | down.m |
| FZD5 | hsa-miR-148b-3p | up.mi | down.m |
| FZD5 | hsa-miR-149-5p | up.mi | down.m |
| FZD5 | hsa-miR-505-3p | up.mi | down.m |
| FZD5 | hsa-miR-148a-3p | up.mi | down.m |
| GAB1 | hsa-miR-30e-5p | up.mi | down.m |
| GAB1 | hsa-miR-93-5p | up.mi | down.m |
| GAB1 | hsa-miR-409-3p | up.mi | down.m |
| GAB1 | hsa-miR-17-5p | up.mi | down.m |
| GAB1 | hsa-miR-181d-5p | up.mi | down.m |
| GAB1 | hsa-miR-20a-5p | up.mi | down.m |
| GAB1 | hsa-miR-20b-5p | up.mi | down.m |
| GAB1 | hsa-miR-106b-5p | up.mi | down.m |
| GAB2 | hsa-miR-192-5p | up.mi | down.m |
| GAB2 | hsa-miR-215-5p | up.mi | down.m |
| GAB2 | hsa-miR-186-5p | up.mi | down.m |
| GABARAPL1 | hsa-miR-15a-5p | up.mi | down.m |
| GABARAPL1 | hsa-miR-424-5p | up.mi | down.m |
| GABARAPL1 | hsa-miR-155-5p | up.mi | down.m |
| GABARAPL1 | hsa-miR-629-3p | up.mi | down.m |
| GALC | hsa-miR-155-5p | up.mi | down.m |
| GALNT13 | hsa-miR-130b-5p | up.mi | down.m |
| GALNT5 | hsa-miR-4668-3p | up.mi | down.m |
| GAS1 | hsa-miR-148b-3p | up.mi | down.m |
| GAS1 | hsa-miR-34a-5p | up.mi | down.m |
| GAS1 | hsa-miR-33b-5p | up.mi | down.m |
| GAS1 | hsa-miR-33a-5p | up.mi | down.m |
| GAS1 | hsa-miR-449a | up.mi | down.m |
| GAS1 | hsa-miR-505-3p | up.mi | down.m |
| GAS1 | hsa-miR-148a-3p | up.mi | down.m |
| GAS1 | hsa-miR-130b-3p | up.mi | down.m |
| GAS1 | hsa-miR-21-3p | up.mi | down.m |
| GAS1 | hsa-miR-148a-5p | up.mi | down.m |
| GAS1 | hsa-miR-183-5p | up.mi | down.m |
| GAS7 | hsa-miR-337-3p | up.mi | down.m |
| GAS7 | hsa-miR-450b-5p | up.mi | down.m |
| GAS7 | hsa-miR-1287-5p | up.mi | down.m |
| GAS7 | hsa-miR-128-3p | up.mi | down.m |
| GATA2 | hsa-miR-671-5p | up.mi | down.m |
| GATA6 | hsa-miR-744-3p | up.mi | down.m |
| GATA6 | hsa-miR-183-5p | up.mi | down.m |
| GATA6 | hsa-miR-5698 | up.mi | down.m |
| GATA6 | hsa-miR-17-5p | up.mi | down.m |
| GATA6 | hsa-miR-20a-5p | up.mi | down.m |
| GATA6 | hsa-miR-200a-3p | up.mi | down.m |
| GATA6 | hsa-miR-185-5p | up.mi | down.m |
| GATA6 | hsa-miR-106a-5p | up.mi | down.m |
| GATA6 | hsa-miR-181b-5p | up.mi | down.m |
| GATA6 | hsa-miR-196a-5p | up.mi | down.m |
| GATA6 | hsa-miR-196b-5p | up.mi | down.m |
| GATA6 | hsa-miR-154-5p | up.mi | down.m |
| GATA6 | hsa-miR-429 | up.mi | down.m |
| GATA6 | hsa-miR-106b-5p | up.mi | down.m |
| GATA6 | hsa-miR-20b-5p | up.mi | down.m |
| GATA6 | hsa-miR-331-5p | up.mi | down.m |
| GATA6 | hsa-miR-93-5p | up.mi | down.m |
| GATA6 | hsa-miR-135b-5p | up.mi | down.m |
| GATA6 | hsa-miR-1307-3p | up.mi | down.m |
| GATA6 | hsa-miR-141-3p | up.mi | down.m |
| GATA6 | hsa-miR-92a-3p | up.mi | down.m |
| GATA6 | hsa-miR-200b-3p | up.mi | down.m |
| GATA6 | hsa-miR-92b-3p | up.mi | down.m |
| GATA6 | hsa-miR-199b-5p | up.mi | down.m |
| GATA6 | hsa-miR-891a-5p | up.mi | down.m |
| GATA6 | hsa-miR-425-3p | up.mi | down.m |
| GATA6 | hsa-miR-128-3p | up.mi | down.m |
| GATA6 | hsa-miR-199a-5p | up.mi | down.m |
| GATA6 | hsa-miR-200c-3p | up.mi | down.m |
| GBP4 | hsa-miR-92a-3p | up.mi | down.m |
| GBP4 | hsa-miR-1307-3p | up.mi | down.m |
| GBP4 | hsa-miR-340-3p | up.mi | down.m |
| GCHFR | hsa-miR-92a-3p | up.mi | down.m |
| GCNT4 | hsa-miR-3913-5p | up.mi | down.m |
| GFOD1 | hsa-miR-215-5p | up.mi | down.m |
| GFOD1 | hsa-miR-130b-3p | up.mi | down.m |
| GFOD1 | hsa-miR-9-5p | up.mi | down.m |
| GFOD1 | hsa-miR-19b-3p | up.mi | down.m |
| GFRA1 | hsa-miR-542-3p | up.mi | down.m |
| GFRA1 | hsa-miR-335-3p | up.mi | down.m |
| GFRA1 | hsa-miR-148b-3p | up.mi | down.m |
| GFRA1 | hsa-miR-589-5p | up.mi | down.m |
| GGA2 | hsa-miR-17-3p | up.mi | down.m |
| GGA2 | hsa-miR-224-5p | up.mi | down.m |
| GGA2 | hsa-miR-92a-3p | up.mi | down.m |
| GGA2 | hsa-miR-539-5p | up.mi | down.m |
| GGT6 | hsa-miR-501-5p | up.mi | down.m |
| GIMAP2 | hsa-miR-193b-3p | up.mi | down.m |
| GIMAP4 | hsa-miR-369-3p | up.mi | down.m |
| GJA1 | hsa-miR-651-5p | up.mi | down.m |
| GJA1 | hsa-miR-381-3p | up.mi | down.m |
| GJA1 | hsa-miR-20a-5p | up.mi | down.m |
| GJA1 | hsa-miR-186-5p | up.mi | down.m |
| GJA1 | hsa-miR-200a-3p | up.mi | down.m |
| GJA1 | hsa-miR-130a-3p | up.mi | down.m |
| GLDN | hsa-miR-29b-3p | up.mi | down.m |
| GLIPR2 | hsa-miR-155-5p | up.mi | down.m |
| GLRX | hsa-miR-128-3p | up.mi | down.m |
| GMPR | hsa-miR-766-3p | up.mi | down.m |
| GMPR | hsa-miR-106b-3p | up.mi | down.m |
| GMPR | hsa-miR-210-5p | up.mi | down.m |
| GNA11 | hsa-miR-224-5p | up.mi | down.m |
| GNAI2 | hsa-miR-625-5p | up.mi | down.m |
| GNAI2 | hsa-miR-92b-3p | up.mi | down.m |
| GNAI2 | hsa-miR-148b-3p | up.mi | down.m |
| GNAI2 | hsa-miR-30e-5p | up.mi | down.m |
| GNAI2 | hsa-miR-455-3p | up.mi | down.m |
| GNAQ | hsa-miR-4668-3p | up.mi | down.m |
| GNAQ | hsa-miR-21-5p | up.mi | down.m |
| GNAQ | hsa-miR-767-5p | up.mi | down.m |
| GNAQ | hsa-miR-653-5p | up.mi | down.m |
| GNAQ | hsa-miR-590-3p | up.mi | down.m |
| GNAQ | hsa-miR-92b-3p | up.mi | down.m |
| GNAQ | hsa-miR-92a-3p | up.mi | down.m |
| GNAQ | hsa-miR-501-5p | up.mi | down.m |
| GNAQ | hsa-miR-128-3p | up.mi | down.m |
| GNAQ | hsa-miR-142-3p | up.mi | down.m |
| GNB4 | hsa-miR-224-5p | up.mi | down.m |
| GNB4 | hsa-miR-5698 | up.mi | down.m |
| GNB4 | hsa-miR-93-5p | up.mi | down.m |
| GNB4 | hsa-miR-143-5p | up.mi | down.m |
| GNB4 | hsa-miR-1307-3p | up.mi | down.m |
| GNB4 | hsa-miR-21-5p | up.mi | down.m |
| GNB4 | hsa-miR-155-5p | up.mi | down.m |
| GNG2 | hsa-miR-16-1-3p | up.mi | down.m |
| GNG2 | hsa-miR-629-3p | up.mi | down.m |
| GNG7 | hsa-miR-92a-3p | up.mi | down.m |
| GPC5 | hsa-miR-186-5p | up.mi | down.m |
| GPC5 | hsa-miR-642a-5p | up.mi | down.m |
| GPC5 | hsa-miR-130b-5p | up.mi | down.m |
| GPC5 | hsa-miR-20a-3p | up.mi | down.m |
| GPD1 | hsa-miR-361-3p | up.mi | down.m |
| GPD1L | hsa-miR-21-5p | up.mi | down.m |
| GPD1L | hsa-miR-210-3p | up.mi | down.m |
| GPD1L | hsa-miR-142-3p | up.mi | down.m |
| GPER1 | hsa-miR-151a-5p | up.mi | down.m |
| GPER1 | hsa-miR-505-5p | up.mi | down.m |
| GPM6A | hsa-miR-106b-5p | up.mi | down.m |
| GPM6A | hsa-miR-17-5p | up.mi | down.m |
| GPM6B | hsa-miR-155-5p | up.mi | down.m |
| GPM6B | hsa-miR-501-5p | up.mi | down.m |
| GPR146 | hsa-miR-103a-2-5p | up.mi | down.m |
| GPR146 | hsa-miR-370-3p | up.mi | down.m |
| GPR162 | hsa-miR-93-3p | up.mi | down.m |
| GPRC5A | hsa-miR-424-5p | up.mi | down.m |
| GPRC5A | hsa-miR-301b-3p | up.mi | down.m |
| GPRC5A | hsa-miR-148a-3p | up.mi | down.m |
| GPRC5A | hsa-miR-130a-3p | up.mi | down.m |
| GPRC5A | hsa-miR-362-5p | up.mi | down.m |
| GPRC5A | hsa-miR-940 | up.mi | down.m |
| GPRC5A | hsa-miR-542-3p | up.mi | down.m |
| GPRC5A | hsa-miR-4652-5p | up.mi | down.m |
| GPRC5A | hsa-miR-767-3p | up.mi | down.m |
| GPRC5A | hsa-miR-3189-3p | up.mi | down.m |
| GPRC5A | hsa-miR-454-3p | up.mi | down.m |
| GPRC5A | hsa-miR-148b-3p | up.mi | down.m |
| GPRC5A | hsa-miR-301a-3p | up.mi | down.m |
| GPRC5A | hsa-miR-130b-3p | up.mi | down.m |
| GPRC5A | hsa-miR-105-5p | up.mi | down.m |
| GPRC5A | hsa-miR-15a-5p | up.mi | down.m |
| GPRIN2 | hsa-miR-337-3p | up.mi | down.m |
| GPRIN2 | hsa-miR-589-5p | up.mi | down.m |
| GPRIN2 | hsa-miR-93-3p | up.mi | down.m |
| GPRIN2 | hsa-miR-148b-5p | up.mi | down.m |
| GPRIN3 | hsa-miR-4724-5p | up.mi | down.m |
| GPRIN3 | hsa-miR-17-5p | up.mi | down.m |
| GPRIN3 | hsa-miR-106b-5p | up.mi | down.m |
| GPRIN3 | hsa-miR-20a-5p | up.mi | down.m |
| GPRIN3 | hsa-miR-20b-5p | up.mi | down.m |
| GPRIN3 | hsa-miR-93-5p | up.mi | down.m |
| GPRIN3 | hsa-miR-767-3p | up.mi | down.m |
| GPRIN3 | hsa-miR-106a-5p | up.mi | down.m |
| GPX3 | hsa-miR-92a-3p | up.mi | down.m |
| GRIA1 | hsa-miR-192-5p | up.mi | down.m |
| GRN | hsa-miR-9-5p | up.mi | down.m |
| GRN | hsa-miR-29b-3p | up.mi | down.m |
| GSN | hsa-miR-767-3p | up.mi | down.m |
| GSTA4 | hsa-miR-185-5p | up.mi | down.m |
| GUCY1A2 | hsa-miR-7705 | up.mi | down.m |
| GUCY1A2 | hsa-miR-625-3p | up.mi | down.m |
| HAS3 | hsa-miR-143-5p | up.mi | down.m |
| HAS3 | hsa-miR-215-5p | up.mi | down.m |
| HAS3 | hsa-miR-192-5p | up.mi | down.m |
| HAVCR2 | hsa-miR-3913-5p | up.mi | down.m |
| HAVCR2 | hsa-miR-940 | up.mi | down.m |
| HBB | hsa-miR-92a-3p | up.mi | down.m |
| HBEGF | hsa-miR-194-5p | up.mi | down.m |
| HBEGF | hsa-miR-215-5p | up.mi | down.m |
| HBEGF | hsa-miR-335-3p | up.mi | down.m |
| HBEGF | hsa-miR-192-5p | up.mi | down.m |
| HCAR2 | hsa-miR-409-3p | up.mi | down.m |
| HECA | hsa-miR-20b-5p | up.mi | down.m |
| HECA | hsa-miR-93-5p | up.mi | down.m |
| HECA | hsa-miR-20a-5p | up.mi | down.m |
| HECA | hsa-miR-17-5p | up.mi | down.m |
| HECA | hsa-miR-106b-5p | up.mi | down.m |
| HECW2 | hsa-miR-181d-5p | up.mi | down.m |
| HECW2 | hsa-miR-181b-5p | up.mi | down.m |
| HECW2 | hsa-miR-455-3p | up.mi | down.m |
| HECW2 | hsa-miR-19a-3p | up.mi | down.m |
| HECW2 | hsa-miR-19b-3p | up.mi | down.m |
| HEG1 | hsa-miR-93-5p | up.mi | down.m |
| HEG1 | hsa-miR-33a-5p | up.mi | down.m |
| HEG1 | hsa-miR-450b-5p | up.mi | down.m |
| HEG1 | hsa-miR-19b-3p | up.mi | down.m |
| HEG1 | hsa-miR-33b-5p | up.mi | down.m |
| HEG1 | hsa-miR-130b-3p | up.mi | down.m |
| HEY2 | hsa-miR-183-3p | up.mi | down.m |
| HEY2 | hsa-miR-4326 | up.mi | down.m |
| HEY2 | hsa-miR-148b-3p | up.mi | down.m |
| HEY2 | hsa-miR-128-3p | up.mi | down.m |
| HEYL | hsa-miR-5698 | up.mi | down.m |
| HEYL | hsa-miR-1307-3p | up.mi | down.m |
| HEYL | hsa-miR-642a-5p | up.mi | down.m |
| HEYL | hsa-miR-15a-5p | up.mi | down.m |
| HEYL | hsa-miR-135b-5p | up.mi | down.m |
| HEYL | hsa-miR-940 | up.mi | down.m |
| HEYL | hsa-miR-93-3p | up.mi | down.m |
| HEYL | hsa-miR-424-5p | up.mi | down.m |
| HEYL | hsa-miR-143-5p | up.mi | down.m |
| HGF | hsa-miR-200a-3p | up.mi | down.m |
| HGF | hsa-miR-199a-3p | up.mi | down.m |
| HHEX | hsa-miR-19b-3p | up.mi | down.m |
| HHEX | hsa-miR-130b-3p | up.mi | down.m |
| HHIP | hsa-miR-200b-5p | up.mi | down.m |
| HHIP | hsa-miR-5698 | up.mi | down.m |
| HHIP | hsa-miR-185-5p | up.mi | down.m |
| HHIP | hsa-miR-339-5p | up.mi | down.m |
| HHIP | hsa-miR-155-5p | up.mi | down.m |
| HHIP | hsa-miR-186-5p | up.mi | down.m |
| HHIP | hsa-miR-200a-5p | up.mi | down.m |
| HHIP | hsa-miR-369-3p | up.mi | down.m |
| HHIP | hsa-miR-130b-5p | up.mi | down.m |
| HIF3A | hsa-miR-210-3p | up.mi | down.m |
| HINT3 | hsa-miR-192-5p | up.mi | down.m |
| HINT3 | hsa-miR-215-5p | up.mi | down.m |
| HIPK3 | hsa-miR-767-3p | up.mi | down.m |
| HIPK3 | hsa-miR-181c-3p | up.mi | down.m |
| HIPK3 | hsa-miR-92a-3p | up.mi | down.m |
| HIPK3 | hsa-miR-655-3p | up.mi | down.m |
| HIPK3 | hsa-miR-106a-5p | up.mi | down.m |
| HIPK3 | hsa-miR-224-5p | up.mi | down.m |
| HIPK3 | hsa-miR-21-5p | up.mi | down.m |
| HIPK3 | hsa-miR-187-3p | up.mi | down.m |
| HIPK3 | hsa-miR-19b-3p | up.mi | down.m |
| HIPK3 | hsa-miR-19a-3p | up.mi | down.m |
| HIPK3 | hsa-miR-20b-5p | up.mi | down.m |
| HLA-DOA | hsa-miR-301a-5p | up.mi | down.m |
| HLA-DOA | hsa-miR-130b-5p | up.mi | down.m |
| HLA-DPA1 | hsa-miR-155-5p | up.mi | down.m |
| HLA-DRA | hsa-miR-128-3p | up.mi | down.m |
| HLA-DRB1 | hsa-miR-148a-5p | up.mi | down.m |
| HLA-DRB1 | hsa-miR-141-5p | up.mi | down.m |
| HLA-DRB1 | hsa-miR-22-5p | up.mi | down.m |
| HLA-DRB1 | hsa-miR-153-5p | up.mi | down.m |
| HLA-DRB1 | hsa-miR-335-3p | up.mi | down.m |
| HLA-DRB5 | hsa-miR-589-5p | up.mi | down.m |
| HLA-DRB5 | hsa-miR-576-5p | up.mi | down.m |
| HLA-DRB5 | hsa-miR-141-5p | up.mi | down.m |
| HLA-DRB5 | hsa-miR-136-5p | up.mi | down.m |
| HLA-E | hsa-miR-455-3p | up.mi | down.m |
| HLA-E | hsa-miR-339-5p | up.mi | down.m |
| HLA-E | hsa-miR-143-5p | up.mi | down.m |
| HLA-E | hsa-miR-92a-3p | up.mi | down.m |
| HLF | hsa-miR-29a-5p | up.mi | down.m |
| HLF | hsa-miR-664a-3p | up.mi | down.m |
| HLF | hsa-miR-377-3p | up.mi | down.m |
| HMGCLL1 | hsa-miR-370-3p | up.mi | down.m |
| HMGCR | hsa-miR-92a-3p | up.mi | down.m |
| HMGCR | hsa-miR-92b-3p | up.mi | down.m |
| HMGCR | hsa-miR-29b-3p | up.mi | down.m |
| HMGCR | hsa-miR-193b-3p | up.mi | down.m |
| HMGCR | hsa-miR-615-3p | up.mi | down.m |
| HMGCS1 | hsa-miR-210-3p | up.mi | down.m |
| HMGCS1 | hsa-miR-186-5p | up.mi | down.m |
| HMGCS1 | hsa-miR-769-3p | up.mi | down.m |
| HMGCS1 | hsa-miR-192-5p | up.mi | down.m |
| HMGCS1 | hsa-miR-1301-3p | up.mi | down.m |
| HMGCS1 | hsa-miR-96-5p | up.mi | down.m |
| HMGCS1 | hsa-miR-130a-5p | up.mi | down.m |
| HMGCS1 | hsa-miR-155-5p | up.mi | down.m |
| HMGCS1 | hsa-miR-19b-3p | up.mi | down.m |
| HMGCS1 | hsa-miR-92a-3p | up.mi | down.m |
| HMGCS1 | hsa-miR-18a-5p | up.mi | down.m |
| HMOX1 | hsa-miR-196a-5p | up.mi | down.m |
| HMOX1 | hsa-miR-128-3p | up.mi | down.m |
| HMOX1 | hsa-miR-148b-3p | up.mi | down.m |
| HOPX | hsa-miR-377-3p | up.mi | down.m |
| HOPX | hsa-miR-337-3p | up.mi | down.m |
| HOPX | hsa-miR-576-5p | up.mi | down.m |
| HOXA5 | hsa-miR-301b-3p | up.mi | down.m |
| HOXA5 | hsa-miR-196a-5p | up.mi | down.m |
| HOXA5 | hsa-miR-130a-3p | up.mi | down.m |
| HOXA5 | hsa-miR-130b-3p | up.mi | down.m |
| HOXA5 | hsa-miR-19a-3p | up.mi | down.m |
| HOXA5 | hsa-miR-301a-3p | up.mi | down.m |
| HOXA5 | hsa-miR-19b-3p | up.mi | down.m |
| HOXA5 | hsa-miR-96-5p | up.mi | down.m |
| HOXA5 | hsa-miR-92a-3p | up.mi | down.m |
| HOXA5 | hsa-miR-183-5p | up.mi | down.m |
| HOXA5 | hsa-miR-200c-3p | up.mi | down.m |
| HOXA5 | hsa-miR-196b-5p | up.mi | down.m |
| HOXA5 | hsa-miR-454-3p | up.mi | down.m |
| HPGD | hsa-miR-21-5p | up.mi | down.m |
| HPGD | hsa-miR-664a-3p | up.mi | down.m |
| HPGD | hsa-miR-21-3p | up.mi | down.m |
| HPS5 | hsa-miR-21-5p | up.mi | down.m |
| HSD17B13 | hsa-miR-940 | up.mi | down.m |
| HSD17B4 | hsa-miR-142-3p | up.mi | down.m |
| HSD17B4 | hsa-miR-324-5p | up.mi | down.m |
| HSDL2 | hsa-miR-577 | up.mi | down.m |
| HSDL2 | hsa-miR-15a-5p | up.mi | down.m |
| HSPA12B | hsa-miR-550a-5p | up.mi | down.m |
| HSPA12B | hsa-miR-377-3p | up.mi | down.m |
| HSPA12B | hsa-miR-651-5p | up.mi | down.m |
| HSPB2 | hsa-miR-17-5p | up.mi | down.m |
| HSPB2 | hsa-miR-577 | up.mi | down.m |
| HSPB6 | hsa-miR-29b-1-5p | up.mi | down.m |
| HSPB8 | hsa-miR-3170 | up.mi | down.m |
| HSPB8 | hsa-miR-671-5p | up.mi | down.m |
| HSPB8 | hsa-miR-128-3p | up.mi | down.m |
| HSPB8 | hsa-miR-193b-5p | up.mi | down.m |
| ICAM1 | hsa-miR-21-5p | up.mi | down.m |
| ICAM1 | hsa-miR-155-5p | up.mi | down.m |
| ICAM1 | hsa-miR-31-5p | up.mi | down.m |
| ICAM1 | hsa-miR-17-3p | up.mi | down.m |
| ICAM1 | hsa-miR-324-3p | up.mi | down.m |
| ICAM1 | hsa-miR-17-5p | up.mi | down.m |
| ICAM1 | hsa-miR-92b-3p | up.mi | down.m |
| ICAM1 | hsa-miR-141-5p | up.mi | down.m |
| ICAM1 | hsa-miR-92a-3p | up.mi | down.m |
| ICAM1 | hsa-miR-93-5p | up.mi | down.m |
| ID1 | hsa-miR-192-5p | up.mi | down.m |
| ID1 | hsa-miR-381-3p | up.mi | down.m |
| ID1 | hsa-miR-215-5p | up.mi | down.m |
| ID2 | hsa-miR-199a-3p | up.mi | down.m |
| ID2 | hsa-miR-9-5p | up.mi | down.m |
| ID2 | hsa-miR-192-5p | up.mi | down.m |
| ID2 | hsa-miR-335-3p | up.mi | down.m |
| ID2 | hsa-miR-199b-3p | up.mi | down.m |
| ID2 | hsa-miR-215-5p | up.mi | down.m |
| ID3 | hsa-miR-29b-3p | up.mi | down.m |
| ID3 | hsa-miR-766-3p | up.mi | down.m |
| ID4 | hsa-miR-324-3p | up.mi | down.m |
| ID4 | hsa-miR-181d-5p | up.mi | down.m |
| ID4 | hsa-let-7c-3p | up.mi | down.m |
| ID4 | hsa-miR-186-5p | up.mi | down.m |
| ID4 | hsa-miR-215-5p | up.mi | down.m |
| ID4 | hsa-miR-192-5p | up.mi | down.m |
| ID4 | hsa-miR-181b-5p | up.mi | down.m |
| ID4 | hsa-miR-590-3p | up.mi | down.m |
| ID4 | hsa-miR-432-5p | up.mi | down.m |
| ID4 | hsa-miR-505-3p | up.mi | down.m |
| ID4 | hsa-miR-9-5p | up.mi | down.m |
| ID4 | hsa-miR-18a-5p | up.mi | down.m |
| ID4 | hsa-miR-148a-5p | up.mi | down.m |
| IDI1 | hsa-miR-193b-3p | up.mi | down.m |
| IDI1 | hsa-miR-192-5p | up.mi | down.m |
| IDI1 | hsa-miR-215-5p | up.mi | down.m |
| IER2 | hsa-miR-539-5p | up.mi | down.m |
| IER2 | hsa-miR-15a-5p | up.mi | down.m |
| IER2 | hsa-miR-455-3p | up.mi | down.m |
| IER2 | hsa-miR-424-5p | up.mi | down.m |
| IFIT2 | hsa-miR-193b-5p | up.mi | down.m |
| IFIT3 | hsa-miR-92b-3p | up.mi | down.m |
| IFIT3 | hsa-miR-337-3p | up.mi | down.m |
| IFNGR1 | hsa-miR-181b-5p | up.mi | down.m |
| IFNGR1 | hsa-miR-196a-5p | up.mi | down.m |
| IFNGR1 | hsa-miR-155-5p | up.mi | down.m |
| IGSF10 | hsa-miR-148b-3p | up.mi | down.m |
| IGSF6 | hsa-miR-3913-5p | up.mi | down.m |
| IHH | hsa-miR-431-5p | up.mi | down.m |
| IKZF1 | hsa-miR-19b-3p | up.mi | down.m |
| IKZF1 | hsa-miR-92a-3p | up.mi | down.m |
| IKZF1 | hsa-miR-19a-3p | up.mi | down.m |
| IL10RA | hsa-miR-15a-5p | up.mi | down.m |
| IL10RA | hsa-miR-185-5p | up.mi | down.m |
| IL17RA | hsa-miR-92a-3p | up.mi | down.m |
| IL17RA | hsa-miR-135b-5p | up.mi | down.m |
| IL1A | hsa-miR-30e-5p | up.mi | down.m |
| IL1A | hsa-miR-191-5p | up.mi | down.m |
| IL1A | hsa-miR-142-3p | up.mi | down.m |
| IL1A | hsa-miR-181d-5p | up.mi | down.m |
| IL1A | hsa-miR-181b-5p | up.mi | down.m |
| IL1B | hsa-miR-106a-5p | up.mi | down.m |
| IL1B | hsa-miR-21-5p | up.mi | down.m |
| IL1R1 | hsa-miR-192-5p | up.mi | down.m |
| IL1R1 | hsa-miR-215-5p | up.mi | down.m |
| IL34 | hsa-miR-28-5p | up.mi | down.m |
| IL5RA | hsa-miR-31-5p | up.mi | down.m |
| IL5RA | hsa-miR-505-5p | up.mi | down.m |
| IL6 | hsa-miR-155-5p | up.mi | down.m |
| IL6 | hsa-miR-136-5p | up.mi | down.m |
| IL6 | hsa-miR-106a-5p | up.mi | down.m |
| IL6 | hsa-miR-149-5p | up.mi | down.m |
| IL6 | hsa-miR-142-3p | up.mi | down.m |
| IL6 | hsa-miR-9-5p | up.mi | down.m |
| IL6R | hsa-miR-1307-3p | up.mi | down.m |
| IL6R | hsa-miR-653-5p | up.mi | down.m |
| IL6R | hsa-miR-34a-5p | up.mi | down.m |
| IL6R | hsa-miR-192-5p | up.mi | down.m |
| IL6R | hsa-miR-215-5p | up.mi | down.m |
| IL6R | hsa-miR-1277-3p | up.mi | down.m |
| IL6R | hsa-miR-495-3p | up.mi | down.m |
| IL6R | hsa-let-7c-3p | up.mi | down.m |
| IL6R | hsa-miR-331-5p | up.mi | down.m |
| IL6R | hsa-miR-335-3p | up.mi | down.m |
| IL6ST | hsa-miR-590-3p | up.mi | down.m |
| IL6ST | hsa-miR-2355-3p | up.mi | down.m |
| IL6ST | hsa-miR-92b-3p | up.mi | down.m |
| IL6ST | hsa-miR-92a-3p | up.mi | down.m |
| IL6ST | hsa-miR-4661-5p | up.mi | down.m |
| IL6ST | hsa-miR-134-5p | up.mi | down.m |
| IL6ST | hsa-miR-192-5p | up.mi | down.m |
| IL6ST | hsa-miR-142-3p | up.mi | down.m |
| IL6ST | hsa-miR-20b-3p | up.mi | down.m |
| IL6ST | hsa-miR-215-5p | up.mi | down.m |
| IL6ST | hsa-miR-615-3p | up.mi | down.m |
| IL7R | hsa-miR-377-3p | up.mi | down.m |
| INHBA | hsa-miR-495-3p | up.mi | down.m |
| INHBA | hsa-miR-214-5p | up.mi | down.m |
| INHBA | hsa-miR-148b-3p | up.mi | down.m |
| INHBA | hsa-miR-4728-3p | up.mi | down.m |
| INHBA | hsa-miR-31-3p | up.mi | down.m |
| INHBA | hsa-miR-675-3p | up.mi | down.m |
| INMT | hsa-miR-760 | up.mi | down.m |
| INMT | hsa-miR-3913-5p | up.mi | down.m |
| INMT | hsa-miR-331-3p | up.mi | down.m |
| INPP5A | hsa-miR-210-3p | up.mi | down.m |
| INPP5A | hsa-miR-339-5p | up.mi | down.m |
| INPP5A | hsa-miR-155-5p | up.mi | down.m |
| INPP5A | hsa-miR-142-3p | up.mi | down.m |
| INSIG1 | hsa-miR-29b-3p | up.mi | down.m |
| INSIG1 | hsa-miR-192-5p | up.mi | down.m |
| INSIG1 | hsa-miR-29b-2-5p | up.mi | down.m |
| INSIG1 | hsa-miR-215-5p | up.mi | down.m |
| INSIG1 | hsa-miR-92a-3p | up.mi | down.m |
| INSIG1 | hsa-miR-20a-5p | up.mi | down.m |
| INSIG1 | hsa-miR-381-3p | up.mi | down.m |
| INSIG1 | hsa-miR-193b-3p | up.mi | down.m |
| INSIG1 | hsa-miR-92b-3p | up.mi | down.m |
| INSIG1 | hsa-miR-335-3p | up.mi | down.m |
| INSIG1 | hsa-miR-22-3p | up.mi | down.m |
| INSIG1 | hsa-miR-210-5p | up.mi | down.m |
| INSIG1 | hsa-miR-324-3p | up.mi | down.m |
| INSIG1 | hsa-miR-183-5p | up.mi | down.m |
| INSIG1 | hsa-miR-106b-5p | up.mi | down.m |
| INSIG1 | hsa-miR-542-3p | up.mi | down.m |
| INSIG1 | hsa-miR-210-3p | up.mi | down.m |
| INSIG1 | hsa-miR-767-5p | up.mi | down.m |
| IQCA1 | hsa-miR-193b-3p | up.mi | down.m |
| IQSEC1 | hsa-miR-20a-5p | up.mi | down.m |
| IQSEC1 | hsa-miR-2355-3p | up.mi | down.m |
| IQSEC1 | hsa-miR-106b-5p | up.mi | down.m |
| IQSEC1 | hsa-miR-17-5p | up.mi | down.m |
| IQSEC1 | hsa-miR-642a-5p | up.mi | down.m |
| IQSEC1 | hsa-miR-20b-5p | up.mi | down.m |
| IQSEC1 | hsa-miR-589-3p | up.mi | down.m |
| IQSEC1 | hsa-miR-93-5p | up.mi | down.m |
| IQSEC3 | hsa-miR-625-5p | up.mi | down.m |
| IRAK3 | hsa-miR-3913-5p | up.mi | down.m |
| IRAK3 | hsa-miR-940 | up.mi | down.m |
| IRAK3 | hsa-miR-155-3p | up.mi | down.m |
| IRF1 | hsa-miR-93-5p | up.mi | down.m |
| IRF1 | hsa-miR-130a-3p | up.mi | down.m |
| IRF1 | hsa-miR-301b-3p | up.mi | down.m |
| IRF1 | hsa-miR-130b-3p | up.mi | down.m |
| IRF1 | hsa-miR-1307-3p | up.mi | down.m |
| IRF1 | hsa-miR-193a-3p | up.mi | down.m |
| IRF1 | hsa-miR-454-3p | up.mi | down.m |
| IRF1 | hsa-miR-193b-3p | up.mi | down.m |
| IRF1 | hsa-miR-142-3p | up.mi | down.m |
| IRF1 | hsa-miR-301a-3p | up.mi | down.m |
| IRF1 | hsa-miR-143-5p | up.mi | down.m |
| IRF1 | hsa-miR-20b-3p | up.mi | down.m |
| IRX2 | hsa-miR-335-3p | up.mi | down.m |
| IRX2 | hsa-miR-542-3p | up.mi | down.m |
| IRX2 | hsa-miR-758-3p | up.mi | down.m |
| ITGA1 | hsa-miR-130b-5p | up.mi | down.m |
| ITGA1 | hsa-miR-7-1-3p | up.mi | down.m |
| ITGA1 | hsa-miR-576-5p | up.mi | down.m |
| ITGA5 | hsa-miR-92a-3p | up.mi | down.m |
| ITGA5 | hsa-miR-31-5p | up.mi | down.m |
| ITGA5 | hsa-miR-148b-3p | up.mi | down.m |
| ITGA5 | hsa-miR-183-5p | up.mi | down.m |
| ITGA5 | hsa-miR-17-3p | up.mi | down.m |
| ITGA5 | hsa-miR-205-5p | up.mi | down.m |
| ITGA5 | hsa-miR-148a-3p | up.mi | down.m |
| ITGA6 | hsa-miR-29b-3p | up.mi | down.m |
| ITGA6 | hsa-miR-92b-3p | up.mi | down.m |
| ITGA6 | hsa-miR-34a-5p | up.mi | down.m |
| ITGA8 | hsa-miR-615-3p | up.mi | down.m |
| ITGA8 | hsa-miR-183-5p | up.mi | down.m |
| ITGA9 | hsa-miR-452-3p | up.mi | down.m |
| ITGA9 | hsa-miR-194-5p | up.mi | down.m |
| ITGAL | hsa-miR-3189-3p | up.mi | down.m |
| ITGAX | hsa-miR-455-3p | up.mi | down.m |
| ITIH5 | hsa-miR-1307-3p | up.mi | down.m |
| ITM2B | hsa-miR-186-5p | up.mi | down.m |
| ITM2B | hsa-miR-320b | up.mi | down.m |
| ITPKC | hsa-miR-301a-5p | up.mi | down.m |
| ITPR1 | hsa-miR-92a-3p | up.mi | down.m |
| ITPR1 | hsa-miR-424-5p | up.mi | down.m |
| ITPR1 | hsa-miR-92b-3p | up.mi | down.m |
| ITPR1 | hsa-miR-19a-3p | up.mi | down.m |
| ITPR1 | hsa-miR-19b-3p | up.mi | down.m |
| ITPR1 | hsa-miR-128-3p | up.mi | down.m |
| ITPR1 | hsa-miR-15a-5p | up.mi | down.m |
| ITPR1 | hsa-miR-449a | up.mi | down.m |
| ITPRIP | hsa-miR-93-3p | up.mi | down.m |
| ITPRIP | hsa-miR-1301-3p | up.mi | down.m |
| JAG1 | hsa-miR-199b-5p | up.mi | down.m |
| JAG1 | hsa-miR-21-5p | up.mi | down.m |
| JAG1 | hsa-miR-192-3p | up.mi | down.m |
| JAG1 | hsa-miR-34a-5p | up.mi | down.m |
| JAG1 | hsa-miR-199a-5p | up.mi | down.m |
| JAG1 | hsa-miR-200c-3p | up.mi | down.m |
| JAM2 | hsa-miR-455-3p | up.mi | down.m |
| JAZF1 | hsa-miR-19a-3p | up.mi | down.m |
| JAZF1 | hsa-miR-96-5p | up.mi | down.m |
| JAZF1 | hsa-miR-31-5p | up.mi | down.m |
| JAZF1 | hsa-miR-19b-3p | up.mi | down.m |
| JAZF1 | hsa-miR-377-3p | up.mi | down.m |
| JAZF1 | hsa-miR-200c-3p | up.mi | down.m |
| JAZF1 | hsa-let-7c-3p | up.mi | down.m |
| JDP2 | hsa-miR-30e-5p | up.mi | down.m |
| JPH1 | hsa-miR-21-5p | up.mi | down.m |
| JPH2 | hsa-miR-5698 | up.mi | down.m |
| JPH2 | hsa-miR-17-3p | up.mi | down.m |
| JPH2 | hsa-miR-143-5p | up.mi | down.m |
| JUN | hsa-miR-200b-3p | up.mi | down.m |
| JUN | hsa-miR-655-3p | up.mi | down.m |
| JUN | hsa-miR-429 | up.mi | down.m |
| JUN | hsa-miR-4724-5p | up.mi | down.m |
| JUN | hsa-miR-93-5p | up.mi | down.m |
| JUN | hsa-miR-200c-3p | up.mi | down.m |
| JUN | hsa-miR-15a-5p | up.mi | down.m |
| JUN | hsa-miR-149-5p | up.mi | down.m |
| JUN | hsa-miR-155-5p | up.mi | down.m |
| JUNB | hsa-miR-199a-5p | up.mi | down.m |
| JUNB | hsa-miR-155-5p | up.mi | down.m |
| JUNB | hsa-miR-199b-5p | up.mi | down.m |
| JUNB | hsa-miR-199a-3p | up.mi | down.m |
| JUND | hsa-miR-625-5p | up.mi | down.m |
| JUND | hsa-miR-29b-1-5p | up.mi | down.m |
| JUND | hsa-miR-186-5p | up.mi | down.m |
| JUND | hsa-miR-2277-5p | up.mi | down.m |
| KANK1 | hsa-miR-424-5p | up.mi | down.m |
| KANK1 | hsa-miR-503-5p | up.mi | down.m |
| KANK1 | hsa-miR-15a-5p | up.mi | down.m |
| KANK1 | hsa-miR-3677-5p | up.mi | down.m |
| KANK2 | hsa-miR-370-3p | up.mi | down.m |
| KANK2 | hsa-miR-155-5p | up.mi | down.m |
| KANK2 | hsa-miR-193b-3p | up.mi | down.m |
| KANK2 | hsa-miR-106b-5p | up.mi | down.m |
| KANK2 | hsa-miR-664a-3p | up.mi | down.m |
| KANK2 | hsa-miR-92a-3p | up.mi | down.m |
| KANK4 | hsa-miR-153-3p | up.mi | down.m |
| KANK4 | hsa-miR-450b-5p | up.mi | down.m |
| KANK4 | hsa-miR-103a-2-5p | up.mi | down.m |
| KAT2B | hsa-miR-192-5p | up.mi | down.m |
| KAT2B | hsa-miR-539-5p | up.mi | down.m |
| KAT2B | hsa-miR-92a-3p | up.mi | down.m |
| KAT2B | hsa-miR-106b-5p | up.mi | down.m |
| KAT2B | hsa-miR-215-5p | up.mi | down.m |
| KAT2B | hsa-miR-19b-3p | up.mi | down.m |
| KAT2B | hsa-miR-127-5p | up.mi | down.m |
| KAT2B | hsa-miR-92b-3p | up.mi | down.m |
| KAT2B | hsa-miR-93-5p | up.mi | down.m |
| KAT2B | hsa-miR-181b-5p | up.mi | down.m |
| KAT2B | hsa-miR-19a-3p | up.mi | down.m |
| KAT2B | hsa-miR-17-5p | up.mi | down.m |
| KAT2B | hsa-miR-142-3p | up.mi | down.m |
| KCNA4 | hsa-miR-377-3p | up.mi | down.m |
| KCNJ15 | hsa-miR-340-3p | up.mi | down.m |
| KCNJ15 | hsa-miR-9-5p | up.mi | down.m |
| KCNJ2 | hsa-miR-19b-3p | up.mi | down.m |
| KCNJ2 | hsa-miR-196a-5p | up.mi | down.m |
| KCNJ2 | hsa-miR-7-1-3p | up.mi | down.m |
| KCNJ2 | hsa-miR-19a-3p | up.mi | down.m |
| KCNJ2 | hsa-miR-212-3p | up.mi | down.m |
| KCNJ2 | hsa-miR-196b-5p | up.mi | down.m |
| KCNJ2 | hsa-miR-331-5p | up.mi | down.m |
| KCNJ2 | hsa-miR-9-5p | up.mi | down.m |
| KCNJ5 | hsa-miR-214-5p | up.mi | down.m |
| KCNJ8 | hsa-miR-20b-5p | up.mi | down.m |
| KCNJ8 | hsa-miR-106a-5p | up.mi | down.m |
| KCNJ8 | hsa-miR-106b-5p | up.mi | down.m |
| KCNJ8 | hsa-miR-20a-5p | up.mi | down.m |
| KCNJ8 | hsa-miR-93-5p | up.mi | down.m |
| KCNJ8 | hsa-miR-17-5p | up.mi | down.m |
| KCNT2 | hsa-miR-183-3p | up.mi | down.m |
| KCNT2 | hsa-miR-335-3p | up.mi | down.m |
| KCTD10 | hsa-miR-22-3p | up.mi | down.m |
| KCTD10 | hsa-miR-301a-3p | up.mi | down.m |
| KCTD10 | hsa-miR-301b-3p | up.mi | down.m |
| KCTD10 | hsa-miR-130a-3p | up.mi | down.m |
| KCTD10 | hsa-miR-130b-3p | up.mi | down.m |
| KCTD10 | hsa-miR-487b-3p | up.mi | down.m |
| KCTD10 | hsa-miR-19b-3p | up.mi | down.m |
| KCTD10 | hsa-miR-193b-3p | up.mi | down.m |
| KCTD10 | hsa-miR-29b-2-5p | up.mi | down.m |
| KCTD10 | hsa-miR-19a-3p | up.mi | down.m |
| KCTD10 | hsa-miR-454-3p | up.mi | down.m |
| KCTD10 | hsa-miR-874-3p | up.mi | down.m |
| KCTD10 | hsa-miR-323a-3p | up.mi | down.m |
| KCTD12 | hsa-miR-22-3p | up.mi | down.m |
| KCTD12 | hsa-miR-142-3p | up.mi | down.m |
| KCTD12 | hsa-miR-215-5p | up.mi | down.m |
| KCTD12 | hsa-miR-183-3p | up.mi | down.m |
| KCTD12 | hsa-miR-29b-1-5p | up.mi | down.m |
| KCTD12 | hsa-miR-454-3p | up.mi | down.m |
| KCTD12 | hsa-miR-186-5p | up.mi | down.m |
| KCTD12 | hsa-miR-192-5p | up.mi | down.m |
| KDR | hsa-miR-199a-3p | up.mi | down.m |
| KDR | hsa-miR-200b-3p | up.mi | down.m |
| KDR | hsa-miR-17-3p | up.mi | down.m |
| KDR | hsa-miR-19b-1-5p | up.mi | down.m |
| KDR | hsa-miR-200c-3p | up.mi | down.m |
| KHDRBS2 | hsa-miR-148b-3p | up.mi | down.m |
| KIAA0040 | hsa-miR-3136-5p | up.mi | down.m |
| KIAA0040 | hsa-miR-134-5p | up.mi | down.m |
| KIAA0355 | hsa-miR-149-5p | up.mi | down.m |
| KIAA0355 | hsa-miR-370-3p | up.mi | down.m |
| KIAA0355 | hsa-miR-20a-3p | up.mi | down.m |
| KIAA0355 | hsa-miR-93-3p | up.mi | down.m |
| KIF1C | hsa-miR-769-3p | up.mi | down.m |
| KIF1C | hsa-miR-149-5p | up.mi | down.m |
| KIF1C | hsa-miR-550a-5p | up.mi | down.m |
| KIF1C | hsa-miR-29b-2-5p | up.mi | down.m |
| KIF1C | hsa-miR-590-3p | up.mi | down.m |
| KIF1C | hsa-miR-323b-3p | up.mi | down.m |
| KIF1C | hsa-miR-9-5p | up.mi | down.m |
| KIF1C | hsa-miR-214-5p | up.mi | down.m |
| KIF1C | hsa-miR-337-3p | up.mi | down.m |
| KIF1C | hsa-miR-3913-5p | up.mi | down.m |
| KL | hsa-miR-199a-5p | up.mi | down.m |
| KLB | hsa-miR-1306-5p | up.mi | down.m |
| KLB | hsa-miR-93-5p | up.mi | down.m |
| KLB | hsa-miR-34a-5p | up.mi | down.m |
| KLF10 | hsa-miR-200b-5p | up.mi | down.m |
| KLF10 | hsa-miR-20a-5p | up.mi | down.m |
| KLF10 | hsa-miR-17-5p | up.mi | down.m |
| KLF10 | hsa-miR-130a-5p | up.mi | down.m |
| KLF10 | hsa-miR-19b-3p | up.mi | down.m |
| KLF10 | hsa-miR-20b-5p | up.mi | down.m |
| KLF10 | hsa-miR-192-5p | up.mi | down.m |
| KLF10 | hsa-miR-215-5p | up.mi | down.m |
| KLF10 | hsa-miR-30e-5p | up.mi | down.m |
| KLF10 | hsa-miR-93-5p | up.mi | down.m |
| KLF10 | hsa-miR-143-5p | up.mi | down.m |
| KLF10 | hsa-miR-106a-5p | up.mi | down.m |
| KLF10 | hsa-miR-766-3p | up.mi | down.m |
| KLF10 | hsa-miR-106b-5p | up.mi | down.m |
| KLF10 | hsa-miR-200a-5p | up.mi | down.m |
| KLF10 | hsa-miR-381-3p | up.mi | down.m |
| KLF11 | hsa-miR-200a-3p | up.mi | down.m |
| KLF11 | hsa-miR-429 | up.mi | down.m |
| KLF11 | hsa-miR-141-3p | up.mi | down.m |
| KLF11 | hsa-miR-200c-3p | up.mi | down.m |
| KLF11 | hsa-miR-200b-3p | up.mi | down.m |
| KLF11 | hsa-miR-93-5p | up.mi | down.m |
| KLF11 | hsa-miR-130b-3p | up.mi | down.m |
| KLF13 | hsa-miR-539-5p | up.mi | down.m |
| KLF13 | hsa-miR-148a-5p | up.mi | down.m |
| KLF13 | hsa-miR-31-5p | up.mi | down.m |
| KLF13 | hsa-miR-1266-5p | up.mi | down.m |
| KLF13 | hsa-miR-19a-3p | up.mi | down.m |
| KLF13 | hsa-miR-19b-3p | up.mi | down.m |
| KLF13 | hsa-miR-940 | up.mi | down.m |
| KLF2 | hsa-miR-92a-3p | up.mi | down.m |
| KLF2 | hsa-miR-377-3p | up.mi | down.m |
| KLF2 | hsa-miR-616-5p | up.mi | down.m |
| KLF2 | hsa-miR-940 | up.mi | down.m |
| KLF2 | hsa-miR-1266-5p | up.mi | down.m |
| KLF2 | hsa-miR-532-3p | up.mi | down.m |
| KLF2 | hsa-miR-4668-3p | up.mi | down.m |
| KLF2 | hsa-miR-455-3p | up.mi | down.m |
| KLF4 | hsa-miR-130a-3p | up.mi | down.m |
| KLF4 | hsa-miR-135b-5p | up.mi | down.m |
| KLF4 | hsa-miR-34a-5p | up.mi | down.m |
| KLF4 | hsa-miR-128-3p | up.mi | down.m |
| KLF4 | hsa-miR-92a-3p | up.mi | down.m |
| KLF4 | hsa-miR-15a-5p | up.mi | down.m |
| KLF6 | hsa-miR-20a-5p | up.mi | down.m |
| KLF6 | hsa-miR-15a-5p | up.mi | down.m |
| KLF6 | hsa-miR-454-3p | up.mi | down.m |
| KLF6 | hsa-miR-142-5p | up.mi | down.m |
| KLF6 | hsa-miR-148a-3p | up.mi | down.m |
| KLF6 | hsa-miR-93-5p | up.mi | down.m |
| KLF6 | hsa-miR-148b-3p | up.mi | down.m |
| KLF6 | hsa-miR-130a-3p | up.mi | down.m |
| KLF6 | hsa-miR-106b-5p | up.mi | down.m |
| KLF6 | hsa-miR-301a-3p | up.mi | down.m |
| KLF6 | hsa-miR-9-5p | up.mi | down.m |
| KLF6 | hsa-miR-17-5p | up.mi | down.m |
| KLF6 | hsa-miR-653-5p | up.mi | down.m |
| KLF6 | hsa-miR-7-1-3p | up.mi | down.m |
| KLF6 | hsa-miR-550a-5p | up.mi | down.m |
| KLF6 | hsa-miR-106a-5p | up.mi | down.m |
| KLF6 | hsa-miR-301b-3p | up.mi | down.m |
| KLF6 | hsa-miR-130b-3p | up.mi | down.m |
| KLF6 | hsa-miR-20b-5p | up.mi | down.m |
| KLF9 | hsa-miR-431-5p | up.mi | down.m |
| KLF9 | hsa-miR-33a-5p | up.mi | down.m |
| KLF9 | hsa-miR-155-5p | up.mi | down.m |
| KLF9 | hsa-miR-21-5p | up.mi | down.m |
| KLF9 | hsa-miR-200c-3p | up.mi | down.m |
| KLF9 | hsa-miR-130b-3p | up.mi | down.m |
| KLF9 | hsa-miR-93-5p | up.mi | down.m |
| KLK10 | hsa-miR-224-5p | up.mi | down.m |
| KMO | hsa-miR-501-3p | up.mi | down.m |
| LAMA2 | hsa-miR-29b-3p | up.mi | down.m |
| LAMA2 | hsa-miR-183-3p | up.mi | down.m |
| LAMA2 | hsa-miR-539-5p | up.mi | down.m |
| LAMA4 | hsa-miR-7705 | up.mi | down.m |
| LAMA4 | hsa-miR-93-3p | up.mi | down.m |
| LAMB2 | hsa-miR-196b-5p | up.mi | down.m |
| LAMC3 | hsa-miR-766-3p | up.mi | down.m |
| LAMP3 | hsa-miR-369-3p | up.mi | down.m |
| LAPTM5 | hsa-miR-3127-5p | up.mi | down.m |
| LAT2 | hsa-miR-155-5p | up.mi | down.m |
| LAT2 | hsa-miR-455-3p | up.mi | down.m |
| LATS2 | hsa-miR-135b-3p | up.mi | down.m |
| LATS2 | hsa-miR-93-5p | up.mi | down.m |
| LATS2 | hsa-miR-181b-5p | up.mi | down.m |
| LATS2 | hsa-miR-183-5p | up.mi | down.m |
| LATS2 | hsa-miR-31-5p | up.mi | down.m |
| LAYN | hsa-miR-744-3p | up.mi | down.m |
| LCP1 | hsa-miR-30e-5p | up.mi | down.m |
| LDLR | hsa-miR-301a-3p | up.mi | down.m |
| LDLR | hsa-miR-130b-3p | up.mi | down.m |
| LDLR | hsa-miR-495-3p | up.mi | down.m |
| LDLR | hsa-miR-19a-3p | up.mi | down.m |
| LDLR | hsa-miR-212-3p | up.mi | down.m |
| LDLR | hsa-miR-106a-5p | up.mi | down.m |
| LDLR | hsa-miR-454-3p | up.mi | down.m |
| LDLR | hsa-miR-381-3p | up.mi | down.m |
| LDLR | hsa-miR-92a-3p | up.mi | down.m |
| LDLR | hsa-miR-93-5p | up.mi | down.m |
| LDLR | hsa-miR-106b-5p | up.mi | down.m |
| LDLR | hsa-miR-130a-3p | up.mi | down.m |
| LDLR | hsa-miR-20b-5p | up.mi | down.m |
| LDLR | hsa-miR-17-5p | up.mi | down.m |
| LDLR | hsa-miR-148a-3p | up.mi | down.m |
| LDLR | hsa-miR-7-1-3p | up.mi | down.m |
| LDLR | hsa-miR-20a-5p | up.mi | down.m |
| LDLR | hsa-miR-148b-3p | up.mi | down.m |
| LDLR | hsa-miR-26b-3p | up.mi | down.m |
| LDLR | hsa-miR-203b-3p | up.mi | down.m |
| LDLR | hsa-miR-128-3p | up.mi | down.m |
| LDLR | hsa-miR-19b-3p | up.mi | down.m |
| LDLR | hsa-miR-4668-3p | up.mi | down.m |
| LDLR | hsa-miR-181b-5p | up.mi | down.m |
| LDLR | hsa-miR-30e-5p | up.mi | down.m |
| LDLR | hsa-miR-377-3p | up.mi | down.m |
| LDLR | hsa-miR-539-5p | up.mi | down.m |
| LDLR | hsa-miR-1307-3p | up.mi | down.m |
| LDLR | hsa-miR-301b-3p | up.mi | down.m |
| LDLR | hsa-miR-450b-5p | up.mi | down.m |
| LDLR | hsa-miR-149-5p | up.mi | down.m |
| LDLR | hsa-miR-181d-5p | up.mi | down.m |
| LDLR | hsa-miR-199a-5p | up.mi | down.m |
| LDLR | hsa-miR-92b-3p | up.mi | down.m |
| LEPR | hsa-miR-200c-3p | up.mi | down.m |
| LEPROT | hsa-miR-376b-3p | up.mi | down.m |
| LEPROT | hsa-miR-106b-5p | up.mi | down.m |
| LEPROT | hsa-miR-20b-5p | up.mi | down.m |
| LEPROT | hsa-miR-17-5p | up.mi | down.m |
| LEPROT | hsa-miR-455-3p | up.mi | down.m |
| LEPROT | hsa-miR-142-5p | up.mi | down.m |
| LEPROT | hsa-miR-20a-5p | up.mi | down.m |
| LEPROT | hsa-miR-93-5p | up.mi | down.m |
| LEPROT | hsa-miR-106a-5p | up.mi | down.m |
| LGALSL | hsa-miR-503-3p | up.mi | down.m |
| LHFPL3 | hsa-miR-361-3p | up.mi | down.m |
| LHFPL3 | hsa-miR-1269b | up.mi | down.m |
| LHFPL3 | hsa-miR-1269a | up.mi | down.m |
| LIFR | hsa-miR-331-5p | up.mi | down.m |
| LIFR | hsa-miR-128-3p | up.mi | down.m |
| LIFR | hsa-miR-7-1-3p | up.mi | down.m |
| LIFR | hsa-miR-21-5p | up.mi | down.m |
| LIFR | hsa-miR-493-3p | up.mi | down.m |
| LIFR | hsa-miR-30e-5p | up.mi | down.m |
| LIFR | hsa-miR-26b-3p | up.mi | down.m |
| LIFR | hsa-miR-212-3p | up.mi | down.m |
| LIFR | hsa-miR-103a-2-5p | up.mi | down.m |
| LILRA2 | hsa-miR-301a-3p | up.mi | down.m |
| LILRA2 | hsa-miR-455-3p | up.mi | down.m |
| LILRA2 | hsa-miR-130b-3p | up.mi | down.m |
| LILRA2 | hsa-miR-92b-3p | up.mi | down.m |
| LILRA2 | hsa-miR-744-3p | up.mi | down.m |
| LILRA2 | hsa-miR-454-3p | up.mi | down.m |
| LILRA2 | hsa-miR-31-5p | up.mi | down.m |
| LILRA2 | hsa-miR-301b-3p | up.mi | down.m |
| LILRA2 | hsa-miR-130a-3p | up.mi | down.m |
| LILRA2 | hsa-miR-92a-3p | up.mi | down.m |
| LILRA5 | hsa-miR-455-3p | up.mi | down.m |
| LILRB2 | hsa-miR-29a-5p | up.mi | down.m |
| LILRB2 | hsa-miR-377-3p | up.mi | down.m |
| LILRB2 | hsa-miR-335-3p | up.mi | down.m |
| LILRB2 | hsa-miR-301a-5p | up.mi | down.m |
| LIMCH1 | hsa-miR-30e-5p | up.mi | down.m |
| LIMCH1 | hsa-miR-192-5p | up.mi | down.m |
| LIMCH1 | hsa-miR-452-3p | up.mi | down.m |
| LIMCH1 | hsa-miR-21-5p | up.mi | down.m |
| LIMD1 | hsa-miR-92a-3p | up.mi | down.m |
| LIMD1 | hsa-miR-766-3p | up.mi | down.m |
| LIMD1 | hsa-miR-339-5p | up.mi | down.m |
| LIMD1 | hsa-miR-455-3p | up.mi | down.m |
| LIMD1 | hsa-miR-7-1-3p | up.mi | down.m |
| LIMD1 | hsa-miR-550a-5p | up.mi | down.m |
| LIN7A | hsa-miR-199a-5p | up.mi | down.m |
| LIPA | hsa-miR-1301-3p | up.mi | down.m |
| LIPA | hsa-miR-454-3p | up.mi | down.m |
| LIPA | hsa-miR-153-5p | up.mi | down.m |
| LIPA | hsa-miR-192-5p | up.mi | down.m |
| LIPA | hsa-miR-130a-3p | up.mi | down.m |
| LIPA | hsa-miR-301b-3p | up.mi | down.m |
| LIPA | hsa-miR-301a-3p | up.mi | down.m |
| LIPA | hsa-miR-130b-3p | up.mi | down.m |
| LIPA | hsa-miR-215-5p | up.mi | down.m |
| LMCD1 | hsa-miR-181b-5p | up.mi | down.m |
| LMCD1 | hsa-miR-193b-3p | up.mi | down.m |
| LNX2 | hsa-miR-155-5p | up.mi | down.m |
| LONRF1 | hsa-miR-181b-5p | up.mi | down.m |
| LONRF1 | hsa-miR-19b-3p | up.mi | down.m |
| LONRF1 | hsa-miR-19a-3p | up.mi | down.m |
| LONRF1 | hsa-miR-128-3p | up.mi | down.m |
| LONRF1 | hsa-miR-301a-3p | up.mi | down.m |
| LONRF1 | hsa-miR-181d-5p | up.mi | down.m |
| LONRF3 | hsa-miR-92b-3p | up.mi | down.m |
| LONRF3 | hsa-miR-92a-3p | up.mi | down.m |
| LPCAT3 | hsa-miR-625-5p | up.mi | down.m |
| LPCAT3 | hsa-miR-5698 | up.mi | down.m |
| LPCAT3 | hsa-miR-185-5p | up.mi | down.m |
| LPL | hsa-miR-155-5p | up.mi | down.m |
| LPL | hsa-miR-148b-3p | up.mi | down.m |
| LPL | hsa-miR-767-5p | up.mi | down.m |
| LPXN | hsa-miR-9-5p | up.mi | down.m |
| LPXN | hsa-miR-4668-3p | up.mi | down.m |
| LPXN | hsa-miR-940 | up.mi | down.m |
| LRP1 | hsa-miR-9-5p | up.mi | down.m |
| LRP1 | hsa-miR-205-5p | up.mi | down.m |
| LRP2 | hsa-miR-196a-5p | up.mi | down.m |
| LRP2BP | hsa-miR-889-3p | up.mi | down.m |
| LRP2BP | hsa-miR-501-5p | up.mi | down.m |
| LRRC10B | hsa-miR-331-3p | up.mi | down.m |
| LRRC2 | hsa-miR-744-3p | up.mi | down.m |
| LRRC32 | hsa-miR-377-3p | up.mi | down.m |
| LRRC32 | hsa-miR-142-3p | up.mi | down.m |
| LRRC32 | hsa-miR-505-5p | up.mi | down.m |
| LRRC4 | hsa-miR-182-5p | up.mi | down.m |
| LRRC4 | hsa-miR-495-3p | up.mi | down.m |
| LRRC8C | hsa-miR-192-5p | up.mi | down.m |
| LRRFIP1 | hsa-miR-186-5p | up.mi | down.m |
| LRRFIP1 | hsa-miR-130b-5p | up.mi | down.m |
| LRRFIP1 | hsa-miR-149-5p | up.mi | down.m |
| LRRFIP1 | hsa-miR-192-5p | up.mi | down.m |
| LRRFIP1 | hsa-miR-21-5p | up.mi | down.m |
| LRRFIP1 | hsa-miR-215-5p | up.mi | down.m |
| LRRFIP1 | hsa-miR-34a-5p | up.mi | down.m |
| LRRK2 | hsa-miR-205-5p | up.mi | down.m |
| LRRK2 | hsa-miR-582-5p | up.mi | down.m |
| LRRK2 | hsa-miR-582-3p | up.mi | down.m |
| LSAMP | hsa-miR-629-3p | up.mi | down.m |
| LSAMP | hsa-miR-361-3p | up.mi | down.m |
| LSAMP | hsa-miR-192-3p | up.mi | down.m |
| LSAMP | hsa-miR-29b-2-5p | up.mi | down.m |
| LSAMP | hsa-miR-103a-2-5p | up.mi | down.m |
| LSAMP | hsa-miR-369-3p | up.mi | down.m |
| LSP1 | hsa-miR-196a-5p | up.mi | down.m |
| LTBP2 | hsa-miR-532-3p | up.mi | down.m |
| LTBP4 | hsa-miR-505-5p | up.mi | down.m |
| LTBP4 | hsa-miR-505-3p | up.mi | down.m |
| LURAP1L | hsa-miR-424-5p | up.mi | down.m |
| LURAP1L | hsa-miR-503-5p | up.mi | down.m |
| LURAP1L | hsa-miR-15a-5p | up.mi | down.m |
| LURAP1L | hsa-miR-381-3p | up.mi | down.m |
| LYVE1 | hsa-miR-34a-3p | up.mi | down.m |
| LYVE1 | hsa-miR-503-3p | up.mi | down.m |
| LYZ | hsa-miR-766-3p | up.mi | down.m |
| LYZ | hsa-miR-616-5p | up.mi | down.m |
| LYZ | hsa-miR-455-3p | up.mi | down.m |
| MACF1 | hsa-miR-215-5p | up.mi | down.m |
| MACF1 | hsa-miR-615-3p | up.mi | down.m |
| MACF1 | hsa-miR-19b-3p | up.mi | down.m |
| MACF1 | hsa-miR-92a-3p | up.mi | down.m |
| MACF1 | hsa-miR-19a-3p | up.mi | down.m |
| MACF1 | hsa-miR-192-5p | up.mi | down.m |
| MACROD2 | hsa-miR-153-5p | up.mi | down.m |
| MAFF | hsa-miR-744-3p | up.mi | down.m |
| MAFF | hsa-miR-5698 | up.mi | down.m |
| MAFF | hsa-miR-143-5p | up.mi | down.m |
| MAFF | hsa-miR-31-3p | up.mi | down.m |
| MAGI1 | hsa-miR-324-3p | up.mi | down.m |
| MAGI1 | hsa-miR-34a-5p | up.mi | down.m |
| MAGI3 | hsa-miR-153-5p | up.mi | down.m |
| MAGI3 | hsa-miR-455-3p | up.mi | down.m |
| MAGI3 | hsa-miR-766-3p | up.mi | down.m |
| MAMDC2 | hsa-miR-196a-5p | up.mi | down.m |
| MAML2 | hsa-miR-205-5p | up.mi | down.m |
| MAOA | hsa-miR-205-5p | up.mi | down.m |
| MAOA | hsa-miR-22-3p | up.mi | down.m |
| MAOB | hsa-miR-671-5p | up.mi | down.m |
| MAOB | hsa-miR-628-5p | up.mi | down.m |
| MAOB | hsa-miR-361-3p | up.mi | down.m |
| MAP2 | hsa-miR-493-3p | up.mi | down.m |
| MAP3K3 | hsa-miR-20b-5p | up.mi | down.m |
| MAP3K3 | hsa-miR-96-5p | up.mi | down.m |
| MAP3K3 | hsa-miR-19b-1-5p | up.mi | down.m |
| MAP3K3 | hsa-miR-106b-5p | up.mi | down.m |
| MAP3K3 | hsa-miR-20a-5p | up.mi | down.m |
| MAP3K3 | hsa-miR-193b-3p | up.mi | down.m |
| MAP3K3 | hsa-miR-183-5p | up.mi | down.m |
| MAP3K3 | hsa-miR-9-5p | up.mi | down.m |
| MAP3K3 | hsa-miR-181d-5p | up.mi | down.m |
| MAP3K3 | hsa-miR-188-5p | up.mi | down.m |
| MAP3K3 | hsa-miR-193a-3p | up.mi | down.m |
| MAP3K3 | hsa-miR-182-5p | up.mi | down.m |
| MAP3K3 | hsa-miR-93-5p | up.mi | down.m |
| MAP3K3 | hsa-miR-181b-5p | up.mi | down.m |
| MAP3K3 | hsa-miR-17-5p | up.mi | down.m |
| MAP3K8 | hsa-miR-589-5p | up.mi | down.m |
| MAP3K8 | hsa-miR-370-3p | up.mi | down.m |
| MAP3K8 | hsa-miR-9-5p | up.mi | down.m |
| MAP3K8 | hsa-miR-17-5p | up.mi | down.m |
| MAP4 | hsa-miR-324-3p | up.mi | down.m |
| MAP4 | hsa-miR-34a-5p | up.mi | down.m |
| MAP4 | hsa-miR-449a | up.mi | down.m |
| MAP4 | hsa-miR-34b-5p | up.mi | down.m |
| MAP4 | hsa-miR-92a-3p | up.mi | down.m |
| MAP4 | hsa-miR-331-3p | up.mi | down.m |
| MAP4 | hsa-miR-339-5p | up.mi | down.m |
| MAP4 | hsa-miR-551b-3p | up.mi | down.m |
| MAP4 | hsa-miR-196a-5p | up.mi | down.m |
| MAPK10 | hsa-miR-3170 | up.mi | down.m |
| MAPRE2 | hsa-miR-455-3p | up.mi | down.m |
| MAPRE2 | hsa-miR-370-3p | up.mi | down.m |
| MAPRE2 | hsa-miR-192-3p | up.mi | down.m |
| MATN3 | hsa-miR-940 | up.mi | down.m |
| MATN3 | hsa-miR-148b-3p | up.mi | down.m |
| MATN3 | hsa-miR-3913-5p | up.mi | down.m |
| MB21D2 | hsa-miR-301b-3p | up.mi | down.m |
| MB21D2 | hsa-miR-130b-3p | up.mi | down.m |
| MB21D2 | hsa-miR-301a-3p | up.mi | down.m |
| MB21D2 | hsa-miR-19a-3p | up.mi | down.m |
| MB21D2 | hsa-miR-22-5p | up.mi | down.m |
| MB21D2 | hsa-miR-454-3p | up.mi | down.m |
| MB21D2 | hsa-miR-19b-3p | up.mi | down.m |
| MB21D2 | hsa-miR-130a-3p | up.mi | down.m |
| MBNL2 | hsa-miR-30e-5p | up.mi | down.m |
| MBNL2 | hsa-miR-369-3p | up.mi | down.m |
| MBNL2 | hsa-miR-19a-3p | up.mi | down.m |
| MBNL2 | hsa-miR-19b-3p | up.mi | down.m |
| MBP | hsa-miR-127-5p | up.mi | down.m |
| MCAM | hsa-miR-155-5p | up.mi | down.m |
| MCAM | hsa-miR-5698 | up.mi | down.m |
| MCAM | hsa-miR-335-3p | up.mi | down.m |
| MCAM | hsa-miR-744-3p | up.mi | down.m |
| MCAM | hsa-miR-143-5p | up.mi | down.m |
| MCAM | hsa-miR-192-5p | up.mi | down.m |
| MCAM | hsa-miR-142-5p | up.mi | down.m |
| MCAM | hsa-miR-215-5p | up.mi | down.m |
| MCC | hsa-miR-2355-5p | up.mi | down.m |
| MCC | hsa-miR-93-5p | up.mi | down.m |
| MCC | hsa-miR-19a-3p | up.mi | down.m |
| MCC | hsa-miR-454-3p | up.mi | down.m |
| MCC | hsa-miR-301a-3p | up.mi | down.m |
| MCC | hsa-miR-130b-3p | up.mi | down.m |
| MCC | hsa-miR-20a-5p | up.mi | down.m |
| MCC | hsa-miR-106a-5p | up.mi | down.m |
| MCC | hsa-miR-301b-3p | up.mi | down.m |
| MCC | hsa-miR-17-5p | up.mi | down.m |
| MCC | hsa-miR-1306-5p | up.mi | down.m |
| MCC | hsa-miR-20b-5p | up.mi | down.m |
| MCC | hsa-miR-9-5p | up.mi | down.m |
| MCC | hsa-miR-19b-3p | up.mi | down.m |
| MCC | hsa-miR-106b-5p | up.mi | down.m |
| MCC | hsa-miR-130a-3p | up.mi | down.m |
| MDGA1 | hsa-miR-210-3p | up.mi | down.m |
| MDGA1 | hsa-miR-324-3p | up.mi | down.m |
| MECOM | hsa-miR-22-3p | up.mi | down.m |
| MEF2A | hsa-miR-155-5p | up.mi | down.m |
| MEF2A | hsa-miR-21-5p | up.mi | down.m |
| MEF2A | hsa-miR-19a-3p | up.mi | down.m |
| MEF2A | hsa-miR-19b-3p | up.mi | down.m |
| MEGF9 | hsa-miR-21-5p | up.mi | down.m |
| MEGF9 | hsa-miR-432-5p | up.mi | down.m |
| MEIS1 | hsa-miR-155-5p | up.mi | down.m |
| MEIS1 | hsa-miR-495-3p | up.mi | down.m |
| MEIS1 | hsa-miR-196b-5p | up.mi | down.m |
| MEIS1 | hsa-miR-4668-3p | up.mi | down.m |
| MEIS1 | hsa-miR-21-5p | up.mi | down.m |
| MEOX2 | hsa-miR-301a-3p | up.mi | down.m |
| MEOX2 | hsa-miR-130a-3p | up.mi | down.m |
| METTL7A | hsa-miR-589-5p | up.mi | down.m |
| METTL7A | hsa-miR-200a-5p | up.mi | down.m |
| METTL7A | hsa-miR-155-5p | up.mi | down.m |
| METTL7A | hsa-miR-200b-5p | up.mi | down.m |
| METTL7A | hsa-miR-339-5p | up.mi | down.m |
| MFAP4 | hsa-miR-449a | up.mi | down.m |
| MFSD2A | hsa-miR-106b-5p | up.mi | down.m |
| MFSD2A | hsa-miR-324-3p | up.mi | down.m |
| MFSD2A | hsa-miR-17-5p | up.mi | down.m |
| MFSD2A | hsa-miR-20b-5p | up.mi | down.m |
| MFSD2A | hsa-miR-106a-5p | up.mi | down.m |
| MFSD2A | hsa-miR-93-5p | up.mi | down.m |
| MFSD2A | hsa-miR-20a-5p | up.mi | down.m |
| MGLL | hsa-miR-142-3p | up.mi | down.m |
| MGLL | hsa-miR-93-5p | up.mi | down.m |
| MGLL | hsa-miR-205-5p | up.mi | down.m |
| MGLL | hsa-miR-324-3p | up.mi | down.m |
| MICALCL | hsa-miR-505-3p | up.mi | down.m |
| MID1IP1 | hsa-miR-615-3p | up.mi | down.m |
| MID1IP1 | hsa-miR-454-3p | up.mi | down.m |
| MID1IP1 | hsa-miR-19a-3p | up.mi | down.m |
| MID1IP1 | hsa-miR-130b-3p | up.mi | down.m |
| MID1IP1 | hsa-miR-19b-3p | up.mi | down.m |
| MID1IP1 | hsa-miR-301b-3p | up.mi | down.m |
| MID1IP1 | hsa-miR-4652-5p | up.mi | down.m |
| MID1IP1 | hsa-miR-301a-3p | up.mi | down.m |
| MID1IP1 | hsa-miR-130a-3p | up.mi | down.m |
| MID1IP1 | hsa-miR-210-3p | up.mi | down.m |
| MITF | hsa-miR-182-5p | up.mi | down.m |
| MITF | hsa-miR-616-5p | up.mi | down.m |
| MITF | hsa-miR-155-5p | up.mi | down.m |
| MITF | hsa-miR-210-3p | up.mi | down.m |
| MITF | hsa-miR-224-5p | up.mi | down.m |
| MITF | hsa-miR-96-5p | up.mi | down.m |
| MMP24 | hsa-miR-29b-3p | up.mi | down.m |
| MOAP1 | hsa-miR-92a-3p | up.mi | down.m |
| MOAP1 | hsa-miR-449a | up.mi | down.m |
| MOAP1 | hsa-miR-192-5p | up.mi | down.m |
| MOAP1 | hsa-miR-92b-3p | up.mi | down.m |
| MOAP1 | hsa-miR-21-5p | up.mi | down.m |
| MOAP1 | hsa-miR-215-5p | up.mi | down.m |
| MOAP1 | hsa-miR-34a-5p | up.mi | down.m |
| MOAP1 | hsa-miR-224-3p | up.mi | down.m |
| MOB3B | hsa-miR-128-3p | up.mi | down.m |
| MOB3B | hsa-miR-192-3p | up.mi | down.m |
| MOB3B | hsa-miR-19b-1-5p | up.mi | down.m |
| MOB3B | hsa-miR-148a-5p | up.mi | down.m |
| MOB3B | hsa-miR-409-3p | up.mi | down.m |
| MOB3B | hsa-miR-455-5p | up.mi | down.m |
| MPP1 | hsa-miR-92b-3p | up.mi | down.m |
| MPP1 | hsa-miR-92a-3p | up.mi | down.m |
| MRAS | hsa-miR-192-3p | up.mi | down.m |
| MRAS | hsa-miR-185-5p | up.mi | down.m |
| MRGPRF | hsa-miR-766-3p | up.mi | down.m |
| MSMO1 | hsa-miR-20b-5p | up.mi | down.m |
| MSMO1 | hsa-miR-93-5p | up.mi | down.m |
| MSMO1 | hsa-miR-454-3p | up.mi | down.m |
| MSMO1 | hsa-miR-203b-3p | up.mi | down.m |
| MSMO1 | hsa-miR-106a-5p | up.mi | down.m |
| MSMO1 | hsa-miR-20a-5p | up.mi | down.m |
| MSMO1 | hsa-miR-106b-5p | up.mi | down.m |
| MSMO1 | hsa-miR-301a-3p | up.mi | down.m |
| MSMO1 | hsa-miR-19a-3p | up.mi | down.m |
| MSMO1 | hsa-miR-130b-3p | up.mi | down.m |
| MSMO1 | hsa-miR-130a-3p | up.mi | down.m |
| MSMO1 | hsa-miR-17-5p | up.mi | down.m |
| MSMO1 | hsa-miR-455-3p | up.mi | down.m |
| MSMO1 | hsa-miR-582-5p | up.mi | down.m |
| MSMO1 | hsa-miR-19b-3p | up.mi | down.m |
| MSMO1 | hsa-miR-766-3p | up.mi | down.m |
| MSMO1 | hsa-miR-381-3p | up.mi | down.m |
| MSMO1 | hsa-miR-301b-3p | up.mi | down.m |
| MSN | hsa-miR-615-3p | up.mi | down.m |
| MSN | hsa-miR-766-3p | up.mi | down.m |
| MSN | hsa-miR-192-5p | up.mi | down.m |
| MSN | hsa-miR-4652-5p | up.mi | down.m |
| MSN | hsa-miR-215-5p | up.mi | down.m |
| MSN | hsa-miR-200b-3p | up.mi | down.m |
| MSN | hsa-miR-200c-3p | up.mi | down.m |
| MSN | hsa-miR-625-5p | up.mi | down.m |
| MSR1 | hsa-miR-9-5p | up.mi | down.m |
| MSRB3 | hsa-miR-193b-5p | up.mi | down.m |
| MSRB3 | hsa-miR-192-5p | up.mi | down.m |
| MT1A | hsa-miR-744-3p | up.mi | down.m |
| MT1A | hsa-miR-542-3p | up.mi | down.m |
| MT1A | hsa-miR-143-5p | up.mi | down.m |
| MT1A | hsa-miR-130b-5p | up.mi | down.m |
| MT1E | hsa-miR-4668-3p | up.mi | down.m |
| MT1E | hsa-miR-143-5p | up.mi | down.m |
| MT1E | hsa-miR-503-5p | up.mi | down.m |
| MT2A | hsa-let-7a-2-3p | up.mi | down.m |
| MT2A | hsa-miR-4668-3p | up.mi | down.m |
| MT2A | hsa-let-7g-3p | up.mi | down.m |
| MT2A | hsa-miR-493-5p | up.mi | down.m |
| MT2A | hsa-let-7c-3p | up.mi | down.m |
| MTMR10 | hsa-miR-148b-3p | up.mi | down.m |
| MTMR10 | hsa-miR-940 | up.mi | down.m |
| MTMR10 | hsa-miR-1307-3p | up.mi | down.m |
| MTMR10 | hsa-miR-92a-3p | up.mi | down.m |
| MTMR10 | hsa-miR-3913-5p | up.mi | down.m |
| MTMR10 | hsa-miR-455-3p | up.mi | down.m |
| MTMR10 | hsa-miR-92b-3p | up.mi | down.m |
| MTMR6 | hsa-miR-19a-3p | up.mi | down.m |
| MTMR6 | hsa-miR-1306-5p | up.mi | down.m |
| MTMR6 | hsa-miR-19b-3p | up.mi | down.m |
| MTMR6 | hsa-miR-3136-5p | up.mi | down.m |
| MUC15 | hsa-miR-15b-3p | up.mi | down.m |
| MVB12B | hsa-miR-2355-3p | up.mi | down.m |
| MXRA7 | hsa-miR-455-3p | up.mi | down.m |
| MXRA7 | hsa-miR-31-5p | up.mi | down.m |
| MXRA7 | hsa-miR-193b-3p | up.mi | down.m |
| MXRA7 | hsa-miR-940 | up.mi | down.m |
| MXRA7 | hsa-miR-6510-3p | up.mi | down.m |
| MXRA7 | hsa-miR-15b-3p | up.mi | down.m |
| MXRA7 | hsa-miR-616-5p | up.mi | down.m |
| MXRA7 | hsa-let-7i-3p | up.mi | down.m |
| MYADM | hsa-miR-642a-5p | up.mi | down.m |
| MYADM | hsa-miR-455-3p | up.mi | down.m |
| MYADM | hsa-miR-495-3p | up.mi | down.m |
| MYADM | hsa-miR-616-5p | up.mi | down.m |
| MYADM | hsa-miR-324-5p | up.mi | down.m |
| MYH10 | hsa-miR-200a-3p | up.mi | down.m |
| MYH10 | hsa-miR-505-3p | up.mi | down.m |
| MYH10 | hsa-miR-106b-3p | up.mi | down.m |
| MYH10 | hsa-miR-92b-3p | up.mi | down.m |
| MYH11 | hsa-miR-130a-3p | up.mi | down.m |
| MYH11 | hsa-miR-660-5p | up.mi | down.m |
| MYH11 | hsa-miR-454-3p | up.mi | down.m |
| MYH11 | hsa-miR-301b-3p | up.mi | down.m |
| MYH11 | hsa-miR-301a-3p | up.mi | down.m |
| MYH11 | hsa-miR-130b-3p | up.mi | down.m |
| MYH11 | hsa-miR-744-3p | up.mi | down.m |
| MYH14 | hsa-miR-185-5p | up.mi | down.m |
| MYH14 | hsa-miR-3127-5p | up.mi | down.m |
| MYH14 | hsa-miR-193b-3p | up.mi | down.m |
| MYH14 | hsa-miR-625-5p | up.mi | down.m |
| MYH14 | hsa-miR-615-3p | up.mi | down.m |
| MYH14 | hsa-miR-5698 | up.mi | down.m |
| MYLIP | hsa-miR-20a-5p | up.mi | down.m |
| MYLIP | hsa-miR-106a-5p | up.mi | down.m |
| MYLIP | hsa-miR-20b-5p | up.mi | down.m |
| MYLIP | hsa-miR-548v | up.mi | down.m |
| MYLIP | hsa-miR-542-3p | up.mi | down.m |
| MYLIP | hsa-miR-1287-3p | up.mi | down.m |
| MYLIP | hsa-miR-30e-5p | up.mi | down.m |
| MYLIP | hsa-miR-17-5p | up.mi | down.m |
| MYLIP | hsa-miR-93-5p | up.mi | down.m |
| MYLIP | hsa-miR-153-5p | up.mi | down.m |
| MYLIP | hsa-miR-143-5p | up.mi | down.m |
| MYLIP | hsa-miR-92a-3p | up.mi | down.m |
| MYLIP | hsa-miR-369-3p | up.mi | down.m |
| MYLIP | hsa-miR-92b-3p | up.mi | down.m |
| MYLIP | hsa-miR-106b-5p | up.mi | down.m |
| MYLIP | hsa-miR-301a-3p | up.mi | down.m |
| MYLIP | hsa-miR-454-3p | up.mi | down.m |
| MYLIP | hsa-miR-19b-3p | up.mi | down.m |
| MYLIP | hsa-miR-186-5p | up.mi | down.m |
| MYLIP | hsa-miR-224-5p | up.mi | down.m |
| MYLIP | hsa-miR-889-3p | up.mi | down.m |
| MYLIP | hsa-miR-130b-3p | up.mi | down.m |
| MYLIP | hsa-miR-323a-3p | up.mi | down.m |
| MYLIP | hsa-miR-455-5p | up.mi | down.m |
| MYLIP | hsa-miR-130a-3p | up.mi | down.m |
| MYLIP | hsa-miR-301b-3p | up.mi | down.m |
| MYLK | hsa-miR-589-5p | up.mi | down.m |
| MYLK | hsa-miR-1301-3p | up.mi | down.m |
| MYLK | hsa-miR-136-3p | up.mi | down.m |
| MYLK | hsa-miR-9-5p | up.mi | down.m |
| MYLK | hsa-miR-18a-5p | up.mi | down.m |
| MYLK | hsa-miR-200c-3p | up.mi | down.m |
| MYLK | hsa-miR-193b-3p | up.mi | down.m |
| MYLK | hsa-miR-155-5p | up.mi | down.m |
| MYO1C | hsa-miR-34a-5p | up.mi | down.m |
| MYO1C | hsa-miR-9-5p | up.mi | down.m |
| MYO1C | hsa-miR-3170 | up.mi | down.m |
| MYO1C | hsa-miR-193b-5p | up.mi | down.m |
| MYO1C | hsa-miR-181b-5p | up.mi | down.m |
| MYO1C | hsa-miR-324-5p | up.mi | down.m |
| MYO1C | hsa-miR-582-3p | up.mi | down.m |
| MYO1C | hsa-miR-92a-3p | up.mi | down.m |
| MYO1C | hsa-miR-1287-5p | up.mi | down.m |
| MYO1F | hsa-miR-592 | up.mi | down.m |
| MYO1F | hsa-miR-17-5p | up.mi | down.m |
| MYO1F | hsa-miR-1307-3p | up.mi | down.m |
| MYO1F | hsa-miR-629-3p | up.mi | down.m |
| MYO1F | hsa-miR-128-3p | up.mi | down.m |
| MYO1F | hsa-miR-20a-5p | up.mi | down.m |
| MYO1F | hsa-miR-532-3p | up.mi | down.m |
| MYO1F | hsa-miR-106b-5p | up.mi | down.m |
| MYO1F | hsa-miR-20b-5p | up.mi | down.m |
| MYO1F | hsa-miR-324-3p | up.mi | down.m |
| MYO1F | hsa-miR-106a-5p | up.mi | down.m |
| MYO1F | hsa-miR-93-3p | up.mi | down.m |
| MYO1F | hsa-miR-93-5p | up.mi | down.m |
| MYO1F | hsa-miR-1306-5p | up.mi | down.m |
| MYO9A | hsa-miR-615-3p | up.mi | down.m |
| MYO9A | hsa-miR-128-3p | up.mi | down.m |
| MYO9A | hsa-miR-21-5p | up.mi | down.m |
| MYOCD | hsa-let-7i-3p | up.mi | down.m |
| MYOCD | hsa-miR-629-3p | up.mi | down.m |
| MYOCD | hsa-miR-9-5p | up.mi | down.m |
| MYOCD | hsa-miR-660-5p | up.mi | down.m |
| MYOCD | hsa-miR-642a-5p | up.mi | down.m |
| MYOCD | hsa-miR-141-5p | up.mi | down.m |
| MYOCD | hsa-miR-130b-5p | up.mi | down.m |
| MYOCD | hsa-miR-335-3p | up.mi | down.m |
| MYRF | hsa-miR-376b-3p | up.mi | down.m |
| MYRF | hsa-miR-17-3p | up.mi | down.m |
| MYZAP | hsa-miR-655-3p | up.mi | down.m |
| MYZAP | hsa-miR-590-3p | up.mi | down.m |
| MYZAP | hsa-miR-92b-3p | up.mi | down.m |
| MYZAP | hsa-miR-92a-3p | up.mi | down.m |
| N4BP1 | hsa-miR-503-5p | up.mi | down.m |
| N4BP1 | hsa-miR-28-5p | up.mi | down.m |
| N4BP1 | hsa-miR-151a-5p | up.mi | down.m |
| N4BP1 | hsa-miR-708-3p | up.mi | down.m |
| N4BP1 | hsa-miR-20a-5p | up.mi | down.m |
| N4BP1 | hsa-miR-424-5p | up.mi | down.m |
| N4BP1 | hsa-miR-766-3p | up.mi | down.m |
| N4BP1 | hsa-miR-15a-5p | up.mi | down.m |
| N4BP1 | hsa-miR-320b | up.mi | down.m |
| N4BP1 | hsa-miR-93-5p | up.mi | down.m |
| N4BP1 | hsa-miR-155-5p | up.mi | down.m |
| N4BP1 | hsa-miR-17-5p | up.mi | down.m |
| N4BP1 | hsa-miR-106b-5p | up.mi | down.m |
| N4BP1 | hsa-miR-20b-5p | up.mi | down.m |
| NACC2 | hsa-miR-130a-3p | up.mi | down.m |
| NACC2 | hsa-miR-20a-5p | up.mi | down.m |
| NACC2 | hsa-miR-3189-3p | up.mi | down.m |
| NACC2 | hsa-miR-30e-5p | up.mi | down.m |
| NACC2 | hsa-miR-93-5p | up.mi | down.m |
| NACC2 | hsa-miR-17-5p | up.mi | down.m |
| NACC2 | hsa-miR-29b-2-5p | up.mi | down.m |
| NACC2 | hsa-miR-423-3p | up.mi | down.m |
| NACC2 | hsa-miR-454-3p | up.mi | down.m |
| NACC2 | hsa-miR-106a-5p | up.mi | down.m |
| NACC2 | hsa-miR-324-3p | up.mi | down.m |
| NACC2 | hsa-miR-148b-5p | up.mi | down.m |
| NACC2 | hsa-miR-130b-3p | up.mi | down.m |
| NACC2 | hsa-miR-19b-3p | up.mi | down.m |
| NACC2 | hsa-miR-92a-3p | up.mi | down.m |
| NACC2 | hsa-miR-301b-3p | up.mi | down.m |
| NACC2 | hsa-miR-106b-5p | up.mi | down.m |
| NACC2 | hsa-miR-19a-3p | up.mi | down.m |
| NACC2 | hsa-miR-301a-3p | up.mi | down.m |
| NACC2 | hsa-miR-92b-3p | up.mi | down.m |
| NACC2 | hsa-miR-20b-5p | up.mi | down.m |
| NACC2 | hsa-miR-210-5p | up.mi | down.m |
| NAP1L2 | hsa-miR-369-3p | up.mi | down.m |
| NAP1L2 | hsa-miR-142-3p | up.mi | down.m |
| NAP1L5 | hsa-miR-381-3p | up.mi | down.m |
| NCEH1 | hsa-miR-192-3p | up.mi | down.m |
| NCEH1 | hsa-miR-192-5p | up.mi | down.m |
| NCEH1 | hsa-miR-215-5p | up.mi | down.m |
| NCF1 | hsa-miR-20b-3p | up.mi | down.m |
| NCKAP1L | hsa-miR-22-5p | up.mi | down.m |
| NCKAP5 | hsa-miR-130b-3p | up.mi | down.m |
| NCKAP5 | hsa-miR-19b-3p | up.mi | down.m |
| NCMAP | hsa-miR-3677-5p | up.mi | down.m |
| NCMAP | hsa-miR-214-5p | up.mi | down.m |
| NCOA4 | hsa-miR-93-3p | up.mi | down.m |
| NCOA4 | hsa-miR-616-5p | up.mi | down.m |
| NCOA4 | hsa-miR-34b-5p | up.mi | down.m |
| NCOA7 | hsa-miR-181d-5p | up.mi | down.m |
| NCOA7 | hsa-miR-181b-5p | up.mi | down.m |
| NDN | hsa-miR-148b-3p | up.mi | down.m |
| NDNF | hsa-miR-224-3p | up.mi | down.m |
| NDNF | hsa-miR-452-3p | up.mi | down.m |
| NDNF | hsa-miR-335-3p | up.mi | down.m |
| NDRG2 | hsa-miR-301a-5p | up.mi | down.m |
| NDST1 | hsa-miR-331-3p | up.mi | down.m |
| NDST1 | hsa-miR-191-5p | up.mi | down.m |
| NDST1 | hsa-miR-128-3p | up.mi | down.m |
| NDST1 | hsa-miR-92a-3p | up.mi | down.m |
| NDST1 | hsa-miR-1306-5p | up.mi | down.m |
| NDST1 | hsa-miR-193b-3p | up.mi | down.m |
| NEBL | hsa-miR-324-5p | up.mi | down.m |
| NECAB1 | hsa-let-7c-3p | up.mi | down.m |
| NEDD4L | hsa-miR-93-3p | up.mi | down.m |
| NEDD9 | hsa-miR-642a-5p | up.mi | down.m |
| NEDD9 | hsa-miR-18a-5p | up.mi | down.m |
| NEDD9 | hsa-miR-29b-3p | up.mi | down.m |
| NEGR1 | hsa-miR-505-3p | up.mi | down.m |
| NEGR1 | hsa-miR-15a-5p | up.mi | down.m |
| NEGR1 | hsa-miR-183-3p | up.mi | down.m |
| NEGR1 | hsa-miR-424-5p | up.mi | down.m |
| NEGR1 | hsa-miR-335-3p | up.mi | down.m |
| NEGR1 | hsa-miR-629-3p | up.mi | down.m |
| NEGR1 | hsa-miR-576-5p | up.mi | down.m |
| NELL2 | hsa-miR-331-3p | up.mi | down.m |
| NEO1 | hsa-miR-18a-5p | up.mi | down.m |
| NEO1 | hsa-miR-590-3p | up.mi | down.m |
| NEO1 | hsa-miR-183-5p | up.mi | down.m |
| NES | hsa-miR-432-5p | up.mi | down.m |
| NES | hsa-miR-155-5p | up.mi | down.m |
| NFAM1 | hsa-miR-377-3p | up.mi | down.m |
| NFAM1 | hsa-miR-539-5p | up.mi | down.m |
| NFASC | hsa-miR-181b-3p | up.mi | down.m |
| NFASC | hsa-miR-370-3p | up.mi | down.m |
| NFASC | hsa-miR-5698 | up.mi | down.m |
| NFATC1 | hsa-miR-324-5p | up.mi | down.m |
| NFATC3 | hsa-miR-337-3p | up.mi | down.m |
| NFATC3 | hsa-miR-103a-2-5p | up.mi | down.m |
| NFATC3 | hsa-miR-324-3p | up.mi | down.m |
| NFATC3 | hsa-miR-493-3p | up.mi | down.m |
| NFATC3 | hsa-miR-361-3p | up.mi | down.m |
| NFATC3 | hsa-miR-185-5p | up.mi | down.m |
| NFATC3 | hsa-miR-9-5p | up.mi | down.m |
| NFIA | hsa-miR-21-5p | up.mi | down.m |
| NFIA | hsa-miR-335-3p | up.mi | down.m |
| NFIA | hsa-miR-19b-3p | up.mi | down.m |
| NFIA | hsa-miR-193b-3p | up.mi | down.m |
| NFIA | hsa-miR-424-5p | up.mi | down.m |
| NFIA | hsa-miR-19a-3p | up.mi | down.m |
| NFIA | hsa-miR-382-5p | up.mi | down.m |
| NFIA | hsa-miR-181b-5p | up.mi | down.m |
| NFIB | hsa-miR-301b-3p | up.mi | down.m |
| NFIB | hsa-miR-17-5p | up.mi | down.m |
| NFIB | hsa-miR-106b-5p | up.mi | down.m |
| NFIB | hsa-miR-130a-3p | up.mi | down.m |
| NFIB | hsa-miR-20a-5p | up.mi | down.m |
| NFIB | hsa-miR-19b-3p | up.mi | down.m |
| NFIB | hsa-miR-4668-3p | up.mi | down.m |
| NFIB | hsa-miR-106a-5p | up.mi | down.m |
| NFIB | hsa-miR-301a-3p | up.mi | down.m |
| NFIB | hsa-miR-331-3p | up.mi | down.m |
| NFIB | hsa-miR-20b-5p | up.mi | down.m |
| NFIB | hsa-miR-335-3p | up.mi | down.m |
| NFIB | hsa-miR-130b-3p | up.mi | down.m |
| NFIB | hsa-miR-192-5p | up.mi | down.m |
| NFIB | hsa-miR-19a-3p | up.mi | down.m |
| NFIB | hsa-miR-370-3p | up.mi | down.m |
| NFIB | hsa-miR-93-5p | up.mi | down.m |
| NFIB | hsa-miR-454-3p | up.mi | down.m |
| NFIB | hsa-miR-21-5p | up.mi | down.m |
| NFIB | hsa-miR-339-5p | up.mi | down.m |
| NFIB | hsa-miR-431-5p | up.mi | down.m |
| NFIB | hsa-miR-590-3p | up.mi | down.m |
| NFIB | hsa-miR-92a-3p | up.mi | down.m |
| NFIC | hsa-miR-424-5p | up.mi | down.m |
| NFIC | hsa-miR-708-5p | up.mi | down.m |
| NFIC | hsa-miR-210-3p | up.mi | down.m |
| NFIC | hsa-miR-28-5p | up.mi | down.m |
| NFIC | hsa-miR-210-5p | up.mi | down.m |
| NFIC | hsa-miR-182-5p | up.mi | down.m |
| NFIC | hsa-miR-2277-5p | up.mi | down.m |
| NFIC | hsa-miR-432-5p | up.mi | down.m |
| NFIC | hsa-miR-31-5p | up.mi | down.m |
| NFIC | hsa-miR-324-3p | up.mi | down.m |
| NFIC | hsa-miR-592 | up.mi | down.m |
| NFIC | hsa-miR-15a-5p | up.mi | down.m |
| NFIC | hsa-miR-940 | up.mi | down.m |
| NFIC | hsa-miR-339-5p | up.mi | down.m |
| NFIC | hsa-miR-96-5p | up.mi | down.m |
| NFIC | hsa-miR-153-5p | up.mi | down.m |
| NFIL3 | hsa-miR-183-5p | up.mi | down.m |
| NFIX | hsa-miR-324-5p | up.mi | down.m |
| NFIX | hsa-miR-24-2-5p | up.mi | down.m |
| NFIX | hsa-let-7g-3p | up.mi | down.m |
| NFIX | hsa-let-7a-2-3p | up.mi | down.m |
| NFIX | hsa-miR-155-3p | up.mi | down.m |
| NFIX | hsa-miR-149-5p | up.mi | down.m |
| NFIX | hsa-miR-4788 | up.mi | down.m |
| NFIX | hsa-miR-24-1-5p | up.mi | down.m |
| NFIX | hsa-miR-625-5p | up.mi | down.m |
| NFKBIA | hsa-miR-381-3p | up.mi | down.m |
| NFKBIA | hsa-miR-196a-5p | up.mi | down.m |
| NFKBIA | hsa-miR-891a-5p | up.mi | down.m |
| NFKBIA | hsa-miR-93-5p | up.mi | down.m |
| NFKBIZ | hsa-miR-193b-3p | up.mi | down.m |
| NFKBIZ | hsa-miR-215-5p | up.mi | down.m |
| NFKBIZ | hsa-miR-192-5p | up.mi | down.m |
| NHLRC4 | hsa-miR-324-3p | up.mi | down.m |
| NHSL1 | hsa-miR-192-5p | up.mi | down.m |
| NHSL1 | hsa-miR-92a-3p | up.mi | down.m |
| NKD1 | hsa-miR-501-5p | up.mi | down.m |
| NKD2 | hsa-miR-127-5p | up.mi | down.m |
| NKD2 | hsa-miR-130b-3p | up.mi | down.m |
| NME5 | hsa-miR-148b-3p | up.mi | down.m |
| NMUR1 | hsa-miR-940 | up.mi | down.m |
| NOTCH1 | hsa-miR-34a-5p | up.mi | down.m |
| NOTCH1 | hsa-miR-9-5p | up.mi | down.m |
| NOTCH1 | hsa-miR-1277-3p | up.mi | down.m |
| NOTCH1 | hsa-miR-449a | up.mi | down.m |
| NOTCH1 | hsa-miR-335-3p | up.mi | down.m |
| NOTCH1 | hsa-miR-200b-3p | up.mi | down.m |
| NOTCH1 | hsa-miR-30e-5p | up.mi | down.m |
| NOTCH1 | hsa-miR-200c-3p | up.mi | down.m |
| NPC2 | hsa-miR-92a-3p | up.mi | down.m |
| NPNT | hsa-miR-425-5p | up.mi | down.m |
| NPNT | hsa-miR-17-5p | up.mi | down.m |
| NPNT | hsa-miR-20a-5p | up.mi | down.m |
| NPNT | hsa-miR-93-5p | up.mi | down.m |
| NPNT | hsa-miR-20b-5p | up.mi | down.m |
| NPNT | hsa-miR-192-5p | up.mi | down.m |
| NPNT | hsa-miR-106b-5p | up.mi | down.m |
| NPNT | hsa-miR-106a-5p | up.mi | down.m |
| NPR1 | hsa-miR-590-3p | up.mi | down.m |
| NPR1 | hsa-miR-3913-5p | up.mi | down.m |
| NPR1 | hsa-miR-455-3p | up.mi | down.m |
| NPR1 | hsa-miR-940 | up.mi | down.m |
| NPR1 | hsa-miR-550a-5p | up.mi | down.m |
| NR0B2 | hsa-miR-141-3p | up.mi | down.m |
| NR2F1 | hsa-miR-149-5p | up.mi | down.m |
| NR2F1 | hsa-miR-181b-5p | up.mi | down.m |
| NR3C1 | hsa-miR-106b-5p | up.mi | down.m |
| NR3C1 | hsa-miR-590-3p | up.mi | down.m |
| NR3C1 | hsa-miR-369-3p | up.mi | down.m |
| NR3C1 | hsa-miR-2355-3p | up.mi | down.m |
| NR3C1 | hsa-miR-17-5p | up.mi | down.m |
| NR3C1 | hsa-miR-93-5p | up.mi | down.m |
| NR3C1 | hsa-miR-106a-5p | up.mi | down.m |
| NR3C1 | hsa-miR-130b-3p | up.mi | down.m |
| NR3C1 | hsa-miR-18a-5p | up.mi | down.m |
| NR3C1 | hsa-miR-155-5p | up.mi | down.m |
| NR3C1 | hsa-miR-20b-5p | up.mi | down.m |
| NR3C1 | hsa-miR-127-5p | up.mi | down.m |
| NR3C1 | hsa-miR-20a-5p | up.mi | down.m |
| NR3C1 | hsa-miR-377-3p | up.mi | down.m |
| NR3C1 | hsa-miR-183-5p | up.mi | down.m |
| NR3C1 | hsa-miR-22-3p | up.mi | down.m |
| NR3C1 | hsa-miR-182-5p | up.mi | down.m |
| NR3C1 | hsa-miR-19b-3p | up.mi | down.m |
| NR3C1 | hsa-miR-192-3p | up.mi | down.m |
| NR3C2 | hsa-miR-301b-3p | up.mi | down.m |
| NR3C2 | hsa-miR-148b-3p | up.mi | down.m |
| NR3C2 | hsa-miR-19b-3p | up.mi | down.m |
| NR4A1 | hsa-miR-196a-5p | up.mi | down.m |
| NR4A1 | hsa-miR-93-5p | up.mi | down.m |
| NR4A2 | hsa-miR-19a-3p | up.mi | down.m |
| NR4A2 | hsa-miR-34a-3p | up.mi | down.m |
| NR4A2 | hsa-miR-34a-5p | up.mi | down.m |
| NR4A3 | hsa-miR-671-5p | up.mi | down.m |
| NR4A3 | hsa-miR-20a-3p | up.mi | down.m |
| NR4A3 | hsa-miR-19b-1-5p | up.mi | down.m |
| NRGN | hsa-miR-5698 | up.mi | down.m |
| NRGN | hsa-miR-542-3p | up.mi | down.m |
| NRGN | hsa-miR-940 | up.mi | down.m |
| NRN1 | hsa-miR-130b-5p | up.mi | down.m |
| NRN1 | hsa-miR-182-5p | up.mi | down.m |
| NRN1 | hsa-miR-28-5p | up.mi | down.m |
| NT5DC1 | hsa-miR-2355-5p | up.mi | down.m |
| NT5DC1 | hsa-miR-15a-5p | up.mi | down.m |
| NT5DC1 | hsa-miR-335-3p | up.mi | down.m |
| NTM | hsa-miR-501-5p | up.mi | down.m |
| NTM | hsa-miR-182-5p | up.mi | down.m |
| NTM | hsa-miR-362-5p | up.mi | down.m |
| NTN4 | hsa-miR-17-5p | up.mi | down.m |
| NTN4 | hsa-miR-20a-5p | up.mi | down.m |
| NTN4 | hsa-miR-196a-5p | up.mi | down.m |
| NTRK2 | hsa-miR-200c-3p | up.mi | down.m |
| NTRK2 | hsa-miR-22-3p | up.mi | down.m |
| NUPR1 | hsa-miR-615-3p | up.mi | down.m |
| NXN | hsa-miR-5698 | up.mi | down.m |
| NXN | hsa-miR-6510-3p | up.mi | down.m |
| NXN | hsa-miR-616-5p | up.mi | down.m |
| NXN | hsa-miR-186-5p | up.mi | down.m |
| NXN | hsa-miR-92a-3p | up.mi | down.m |
| NXN | hsa-miR-1287-3p | up.mi | down.m |
| NXPE3 | hsa-miR-744-3p | up.mi | down.m |
| NXPE3 | hsa-miR-196b-5p | up.mi | down.m |
| NXPE3 | hsa-miR-148b-3p | up.mi | down.m |
| NXPE3 | hsa-miR-874-3p | up.mi | down.m |
| NXPE3 | hsa-miR-431-5p | up.mi | down.m |
| NXPE3 | hsa-miR-4668-3p | up.mi | down.m |
| NXPE3 | hsa-miR-337-3p | up.mi | down.m |
| NXPE3 | hsa-miR-196a-5p | up.mi | down.m |
| NXPH3 | hsa-miR-625-5p | up.mi | down.m |
| NXPH3 | hsa-miR-493-3p | up.mi | down.m |
| OLFML2A | hsa-miR-212-3p | up.mi | down.m |
| OLFML2A | hsa-miR-940 | up.mi | down.m |
| OLFML2A | hsa-miR-2355-3p | up.mi | down.m |
| OLFML2A | hsa-miR-766-3p | up.mi | down.m |
| OLFML2A | hsa-miR-214-5p | up.mi | down.m |
| OLR1 | hsa-let-7g-3p | up.mi | down.m |
| OLR1 | hsa-let-7c-3p | up.mi | down.m |
| OLR1 | hsa-miR-21-5p | up.mi | down.m |
| OLR1 | hsa-miR-493-5p | up.mi | down.m |
| OLR1 | hsa-miR-642a-5p | up.mi | down.m |
| OLR1 | hsa-let-7a-2-3p | up.mi | down.m |
| OLR1 | hsa-miR-651-5p | up.mi | down.m |
| OLR1 | hsa-miR-889-3p | up.mi | down.m |
| OLR1 | hsa-miR-616-5p | up.mi | down.m |
| OLR1 | hsa-miR-590-5p | up.mi | down.m |
| OLR1 | hsa-miR-155-5p | up.mi | down.m |
| OLR1 | hsa-miR-590-3p | up.mi | down.m |
| OMD | hsa-miR-130a-3p | up.mi | down.m |
| OMD | hsa-miR-301b-3p | up.mi | down.m |
| OMD | hsa-miR-301a-3p | up.mi | down.m |
| OMD | hsa-miR-616-5p | up.mi | down.m |
| OMD | hsa-miR-454-3p | up.mi | down.m |
| OMD | hsa-miR-130b-3p | up.mi | down.m |
| OSBPL11 | hsa-miR-615-3p | up.mi | down.m |
| OSBPL11 | hsa-miR-148b-3p | up.mi | down.m |
| OSBPL6 | hsa-miR-192-5p | up.mi | down.m |
| OSBPL6 | hsa-miR-33a-5p | up.mi | down.m |
| OSBPL6 | hsa-miR-215-5p | up.mi | down.m |
| OSCAR | hsa-miR-424-5p | up.mi | down.m |
| OSCAR | hsa-miR-503-5p | up.mi | down.m |
| OSCAR | hsa-miR-15a-5p | up.mi | down.m |
| OSCP1 | hsa-miR-33b-5p | up.mi | down.m |
| OSCP1 | hsa-miR-199a-5p | up.mi | down.m |
| OSCP1 | hsa-miR-33a-5p | up.mi | down.m |
| OSTF1 | hsa-miR-4668-3p | up.mi | down.m |
| OSTF1 | hsa-miR-429 | up.mi | down.m |
| OSTF1 | hsa-miR-136-5p | up.mi | down.m |
| OSTF1 | hsa-miR-616-5p | up.mi | down.m |
| OSTM1 | hsa-miR-17-5p | up.mi | down.m |
| OSTM1 | hsa-miR-155-5p | up.mi | down.m |
| OSTM1 | hsa-miR-369-3p | up.mi | down.m |
| OSTM1 | hsa-miR-760 | up.mi | down.m |
| OSTM1 | hsa-miR-200b-3p | up.mi | down.m |
| OSTM1 | hsa-miR-106b-5p | up.mi | down.m |
| OSTM1 | hsa-miR-429 | up.mi | down.m |
| OSTM1 | hsa-miR-200c-3p | up.mi | down.m |
| OSTM1 | hsa-miR-149-5p | up.mi | down.m |
| OSTM1 | hsa-miR-106a-5p | up.mi | down.m |
| OSTM1 | hsa-miR-20b-5p | up.mi | down.m |
| OSTM1 | hsa-miR-93-5p | up.mi | down.m |
| OSTM1 | hsa-miR-20a-5p | up.mi | down.m |
| OTUD1 | hsa-miR-19b-3p | up.mi | down.m |
| OTUD1 | hsa-miR-21-5p | up.mi | down.m |
| OTUD1 | hsa-miR-539-5p | up.mi | down.m |
| OTUD1 | hsa-miR-19a-3p | up.mi | down.m |
| OTUD1 | hsa-miR-181b-5p | up.mi | down.m |
| OTUD1 | hsa-miR-192-5p | up.mi | down.m |
| OTUD1 | hsa-miR-181d-5p | up.mi | down.m |
| P2RX7 | hsa-miR-130b-5p | up.mi | down.m |
| P2RX7 | hsa-miR-301a-5p | up.mi | down.m |
| P2RX7 | hsa-miR-9-3p | up.mi | down.m |
| P2RX7 | hsa-miR-186-5p | up.mi | down.m |
| P2RY1 | hsa-miR-19b-1-5p | up.mi | down.m |
| P2RY1 | hsa-miR-450b-5p | up.mi | down.m |
| P2RY1 | hsa-miR-153-3p | up.mi | down.m |
| P3H2 | hsa-miR-625-5p | up.mi | down.m |
| P3H2 | hsa-miR-5698 | up.mi | down.m |
| P3H2 | hsa-miR-203b-3p | up.mi | down.m |
| PALD1 | hsa-miR-155-5p | up.mi | down.m |
| PALD1 | hsa-miR-196b-5p | up.mi | down.m |
| PALM | hsa-miR-324-5p | up.mi | down.m |
| PAPSS2 | hsa-miR-18a-5p | up.mi | down.m |
| PAPSS2 | hsa-miR-335-3p | up.mi | down.m |
| PAQR5 | hsa-miR-199a-3p | up.mi | down.m |
| PAQR5 | hsa-miR-143-5p | up.mi | down.m |
| PAQR5 | hsa-miR-199b-3p | up.mi | down.m |
| PAQR5 | hsa-miR-28-5p | up.mi | down.m |
| PAQR5 | hsa-miR-20a-5p | up.mi | down.m |
| PAQR5 | hsa-miR-183-3p | up.mi | down.m |
| PAQR5 | hsa-miR-708-5p | up.mi | down.m |
| PAQR5 | hsa-miR-3913-5p | up.mi | down.m |
| PAQR8 | hsa-miR-136-5p | up.mi | down.m |
| PAQR8 | hsa-miR-192-3p | up.mi | down.m |
| PAQR8 | hsa-miR-7705 | up.mi | down.m |
| PAQR8 | hsa-miR-153-5p | up.mi | down.m |
| PAQR8 | hsa-miR-361-3p | up.mi | down.m |
| PARD6B | hsa-miR-93-5p | up.mi | down.m |
| PARD6B | hsa-miR-106b-5p | up.mi | down.m |
| PARD6B | hsa-miR-542-3p | up.mi | down.m |
| PARD6B | hsa-miR-6510-3p | up.mi | down.m |
| PARD6B | hsa-miR-205-5p | up.mi | down.m |
| PARD6B | hsa-miR-224-3p | up.mi | down.m |
| PARD6B | hsa-miR-429 | up.mi | down.m |
| PARD6B | hsa-miR-369-3p | up.mi | down.m |
| PARD6B | hsa-miR-17-5p | up.mi | down.m |
| PARD6B | hsa-miR-92b-3p | up.mi | down.m |
| PARD6B | hsa-miR-181d-5p | up.mi | down.m |
| PARD6B | hsa-miR-18a-5p | up.mi | down.m |
| PARD6B | hsa-miR-200b-3p | up.mi | down.m |
| PARD6B | hsa-miR-376b-3p | up.mi | down.m |
| PARD6B | hsa-miR-20b-5p | up.mi | down.m |
| PARD6B | hsa-miR-20a-5p | up.mi | down.m |
| PARD6B | hsa-miR-589-5p | up.mi | down.m |
| PARD6B | hsa-miR-616-5p | up.mi | down.m |
| PARD6B | hsa-miR-92a-3p | up.mi | down.m |
| PARD6B | hsa-miR-200c-3p | up.mi | down.m |
| PARD6B | hsa-miR-9-3p | up.mi | down.m |
| PARD6B | hsa-miR-760 | up.mi | down.m |
| PARVB | hsa-miR-370-3p | up.mi | down.m |
| PARVB | hsa-miR-5698 | up.mi | down.m |
| PARVB | hsa-miR-3934-3p | up.mi | down.m |
| PARVB | hsa-miR-143-5p | up.mi | down.m |
| PARVB | hsa-miR-616-5p | up.mi | down.m |
| PARVB | hsa-miR-1306-5p | up.mi | down.m |
| PARVB | hsa-miR-2355-5p | up.mi | down.m |
| PARVB | hsa-miR-340-3p | up.mi | down.m |
| PARVB | hsa-miR-501-5p | up.mi | down.m |
| PARVB | hsa-miR-1287-5p | up.mi | down.m |
| PARVG | hsa-miR-92b-3p | up.mi | down.m |
| PBX1 | hsa-miR-196b-5p | up.mi | down.m |
| PBX1 | hsa-miR-149-5p | up.mi | down.m |
| PBX1 | hsa-miR-21-5p | up.mi | down.m |
| PCDH17 | hsa-miR-452-3p | up.mi | down.m |
| PCOLCE2 | hsa-miR-215-5p | up.mi | down.m |
| PCOLCE2 | hsa-miR-182-5p | up.mi | down.m |
| PCOLCE2 | hsa-miR-192-5p | up.mi | down.m |
| PCSK9 | hsa-miR-215-5p | up.mi | down.m |
| PCSK9 | hsa-miR-744-3p | up.mi | down.m |
| PCSK9 | hsa-miR-192-5p | up.mi | down.m |
| PCYOX1 | hsa-miR-28-5p | up.mi | down.m |
| PCYOX1 | hsa-miR-155-5p | up.mi | down.m |
| PCYOX1 | hsa-miR-708-5p | up.mi | down.m |
| PCYOX1 | hsa-miR-590-3p | up.mi | down.m |
| PDE1B | hsa-miR-149-5p | up.mi | down.m |
| PDE2A | hsa-miR-192-5p | up.mi | down.m |
| PDE3B | hsa-miR-130b-3p | up.mi | down.m |
| PDE3B | hsa-miR-128-3p | up.mi | down.m |
| PDE3B | hsa-miR-93-5p | up.mi | down.m |
| PDE4B | hsa-miR-34a-3p | up.mi | down.m |
| PDE4D | hsa-miR-15a-5p | up.mi | down.m |
| PDE4D | hsa-miR-18a-5p | up.mi | down.m |
| PDE4D | hsa-miR-31-3p | up.mi | down.m |
| PDE4D | hsa-miR-19b-3p | up.mi | down.m |
| PDE4D | hsa-miR-424-3p | up.mi | down.m |
| PDE4D | hsa-miR-3136-5p | up.mi | down.m |
| PDE4D | hsa-miR-199a-5p | up.mi | down.m |
| PDE4D | hsa-miR-424-5p | up.mi | down.m |
| PDE4D | hsa-miR-142-5p | up.mi | down.m |
| PDGFA | hsa-miR-29b-3p | up.mi | down.m |
| PDGFA | hsa-miR-130b-5p | up.mi | down.m |
| PDGFB | hsa-miR-625-5p | up.mi | down.m |
| PDGFB | hsa-miR-93-5p | up.mi | down.m |
| PDGFB | hsa-miR-17-5p | up.mi | down.m |
| PDGFB | hsa-miR-20b-5p | up.mi | down.m |
| PDGFB | hsa-miR-106a-5p | up.mi | down.m |
| PDGFB | hsa-miR-106b-5p | up.mi | down.m |
| PDGFB | hsa-miR-20a-5p | up.mi | down.m |
| PDGFB | hsa-miR-29b-3p | up.mi | down.m |
| PDK4 | hsa-miR-182-5p | up.mi | down.m |
| PDLIM1 | hsa-miR-92a-3p | up.mi | down.m |
| PDLIM3 | hsa-miR-1307-3p | up.mi | down.m |
| PDLIM3 | hsa-miR-203b-3p | up.mi | down.m |
| PDLIM3 | hsa-miR-192-5p | up.mi | down.m |
| PDLIM3 | hsa-miR-215-5p | up.mi | down.m |
| PDPN | hsa-miR-940 | up.mi | down.m |
| PDZD2 | hsa-miR-377-3p | up.mi | down.m |
| PDZD4 | hsa-miR-185-5p | up.mi | down.m |
| PEAK1 | hsa-miR-92a-3p | up.mi | down.m |
| PEAK1 | hsa-miR-93-5p | up.mi | down.m |
| PEAK1 | hsa-miR-17-5p | up.mi | down.m |
| PEAK1 | hsa-miR-92b-3p | up.mi | down.m |
| PEAK1 | hsa-miR-20a-5p | up.mi | down.m |
| PEAK1 | hsa-miR-106b-5p | up.mi | down.m |
| PEAK1 | hsa-miR-183-5p | up.mi | down.m |
| PEAK1 | hsa-miR-20b-5p | up.mi | down.m |
| PEAK1 | hsa-miR-769-3p | up.mi | down.m |
| PEAK1 | hsa-miR-34b-5p | up.mi | down.m |
| PEAR1 | hsa-miR-93-3p | up.mi | down.m |
| PEAR1 | hsa-miR-505-5p | up.mi | down.m |
| PEAR1 | hsa-miR-370-3p | up.mi | down.m |
| PEBP1 | hsa-miR-539-5p | up.mi | down.m |
| PEBP1 | hsa-miR-224-5p | up.mi | down.m |
| PEBP1 | hsa-miR-181b-5p | up.mi | down.m |
| PEBP1 | hsa-miR-155-5p | up.mi | down.m |
| PEBP1 | hsa-miR-92a-3p | up.mi | down.m |
| PEBP1 | hsa-miR-370-3p | up.mi | down.m |
| PEBP1 | hsa-miR-103a-2-5p | up.mi | down.m |
| PEBP1 | hsa-miR-142-5p | up.mi | down.m |
| PEBP1 | hsa-miR-181d-5p | up.mi | down.m |
| PEG10 | hsa-miR-148b-5p | up.mi | down.m |
| PEG10 | hsa-miR-767-5p | up.mi | down.m |
| PEG10 | hsa-miR-128-3p | up.mi | down.m |
| PEG10 | hsa-miR-186-5p | up.mi | down.m |
| PEG10 | hsa-miR-105-5p | up.mi | down.m |
| PEG10 | hsa-miR-501-3p | up.mi | down.m |
| PEG10 | hsa-miR-769-3p | up.mi | down.m |
| PEG10 | hsa-miR-449a | up.mi | down.m |
| PEG10 | hsa-miR-34a-5p | up.mi | down.m |
| PEG10 | hsa-miR-30e-5p | up.mi | down.m |
| PEG10 | hsa-miR-193b-3p | up.mi | down.m |
| PEG10 | hsa-miR-625-3p | up.mi | down.m |
| PER1 | hsa-miR-28-3p | up.mi | down.m |
| PER1 | hsa-miR-17-5p | up.mi | down.m |
| PER1 | hsa-miR-2277-5p | up.mi | down.m |
| PER1 | hsa-miR-4677-3p | up.mi | down.m |
| PER1 | hsa-miR-34a-5p | up.mi | down.m |
| PER1 | hsa-miR-136-5p | up.mi | down.m |
| PER1 | hsa-miR-29b-3p | up.mi | down.m |
| PER1 | hsa-miR-34a-3p | up.mi | down.m |
| PF4 | hsa-miR-628-3p | up.mi | down.m |
| PFKFB2 | hsa-miR-93-5p | up.mi | down.m |
| PFKFB2 | hsa-miR-21-5p | up.mi | down.m |
| PFKFB2 | hsa-miR-382-5p | up.mi | down.m |
| PFKFB2 | hsa-miR-20a-5p | up.mi | down.m |
| PFKFB2 | hsa-miR-377-3p | up.mi | down.m |
| PFKFB2 | hsa-miR-106a-5p | up.mi | down.m |
| PFKFB2 | hsa-miR-17-5p | up.mi | down.m |
| PFKFB2 | hsa-miR-106b-5p | up.mi | down.m |
| PFKFB2 | hsa-miR-20b-5p | up.mi | down.m |
| PFKFB3 | hsa-miR-183-5p | up.mi | down.m |
| PFKFB3 | hsa-miR-185-5p | up.mi | down.m |
| PFKFB3 | hsa-miR-96-5p | up.mi | down.m |
| PFKFB3 | hsa-miR-182-5p | up.mi | down.m |
| PHACTR2 | hsa-miR-148a-3p | up.mi | down.m |
| PHACTR2 | hsa-miR-655-3p | up.mi | down.m |
| PHACTR2 | hsa-miR-148b-3p | up.mi | down.m |
| PHACTR2 | hsa-miR-183-3p | up.mi | down.m |
| PHACTR2 | hsa-miR-708-3p | up.mi | down.m |
| PHACTR2 | hsa-miR-21-5p | up.mi | down.m |
| PHACTR2 | hsa-miR-369-3p | up.mi | down.m |
| PHACTR2 | hsa-miR-29b-3p | up.mi | down.m |
| PHACTR2 | hsa-miR-185-5p | up.mi | down.m |
| PHACTR2 | hsa-miR-590-3p | up.mi | down.m |
| PHACTR2 | hsa-miR-136-5p | up.mi | down.m |
| PHACTR2 | hsa-miR-155-5p | up.mi | down.m |
| PHLDB2 | hsa-miR-432-5p | up.mi | down.m |
| PHLDB2 | hsa-miR-148b-3p | up.mi | down.m |
| PHLDB2 | hsa-miR-148a-3p | up.mi | down.m |
| PIAS1 | hsa-miR-424-5p | up.mi | down.m |
| PIAS1 | hsa-let-7g-3p | up.mi | down.m |
| PIAS1 | hsa-let-7a-2-3p | up.mi | down.m |
| PIGR | hsa-miR-766-3p | up.mi | down.m |
| PIGR | hsa-miR-940 | up.mi | down.m |
| PIGR | hsa-miR-642a-5p | up.mi | down.m |
| PIGR | hsa-miR-532-3p | up.mi | down.m |
| PIGR | hsa-miR-3913-5p | up.mi | down.m |
| PIH1D3 | hsa-miR-148b-3p | up.mi | down.m |
| PIK3AP1 | hsa-miR-92b-3p | up.mi | down.m |
| PIK3AP1 | hsa-miR-92a-3p | up.mi | down.m |
| PIK3AP1 | hsa-miR-106b-5p | up.mi | down.m |
| PIK3R1 | hsa-miR-15a-5p | up.mi | down.m |
| PIK3R1 | hsa-miR-455-5p | up.mi | down.m |
| PIK3R1 | hsa-miR-503-5p | up.mi | down.m |
| PIK3R1 | hsa-miR-155-5p | up.mi | down.m |
| PIK3R1 | hsa-miR-542-3p | up.mi | down.m |
| PIK3R1 | hsa-miR-424-5p | up.mi | down.m |
| PIK3R1 | hsa-miR-29b-3p | up.mi | down.m |
| PIK3R1 | hsa-miR-128-3p | up.mi | down.m |
| PIK3R1 | hsa-miR-21-5p | up.mi | down.m |
| PIK3R3 | hsa-miR-148b-3p | up.mi | down.m |
| PIK3R3 | hsa-miR-19b-3p | up.mi | down.m |
| PIK3R3 | hsa-miR-331-3p | up.mi | down.m |
| PIK3R3 | hsa-miR-19a-3p | up.mi | down.m |
| PJA2 | hsa-miR-505-3p | up.mi | down.m |
| PKDCC | hsa-miR-92a-3p | up.mi | down.m |
| PKHD1L1 | hsa-miR-642a-5p | up.mi | down.m |
| PKIA | hsa-miR-155-5p | up.mi | down.m |
| PKIA | hsa-miR-20a-3p | up.mi | down.m |
| PKIA | hsa-miR-210-3p | up.mi | down.m |
| PKIA | hsa-miR-550a-3p | up.mi | down.m |
| PKIA | hsa-miR-200c-5p | up.mi | down.m |
| PKIG | hsa-miR-331-5p | up.mi | down.m |
| PKIG | hsa-miR-331-3p | up.mi | down.m |
| PKNOX2 | hsa-miR-671-5p | up.mi | down.m |
| PKNOX2 | hsa-miR-5698 | up.mi | down.m |
| PKNOX2 | hsa-miR-625-5p | up.mi | down.m |
| PKNOX2 | hsa-miR-185-5p | up.mi | down.m |
| PLA2G4F | hsa-miR-92a-3p | up.mi | down.m |
| PLA2G4F | hsa-miR-331-3p | up.mi | down.m |
| PLA2G5 | hsa-miR-192-3p | up.mi | down.m |
| PLA2G5 | hsa-miR-128-3p | up.mi | down.m |
| PLAC8 | hsa-miR-29a-5p | up.mi | down.m |
| PLAC8 | hsa-miR-185-5p | up.mi | down.m |
| PLAC8 | hsa-miR-542-3p | up.mi | down.m |
| PLCB4 | hsa-miR-9-5p | up.mi | down.m |
| PLCD1 | hsa-miR-17-3p | up.mi | down.m |
| PLCD1 | hsa-miR-185-5p | up.mi | down.m |
| PLCE1 | hsa-miR-215-5p | up.mi | down.m |
| PLCE1 | hsa-miR-324-3p | up.mi | down.m |
| PLCE1 | hsa-miR-192-5p | up.mi | down.m |
| PLCE1 | hsa-miR-940 | up.mi | down.m |
| PLCL1 | hsa-miR-130b-5p | up.mi | down.m |
| PLCXD2 | hsa-miR-205-5p | up.mi | down.m |
| PLCXD2 | hsa-miR-503-3p | up.mi | down.m |
| PLCXD2 | hsa-miR-92a-3p | up.mi | down.m |
| PLEK | hsa-miR-153-5p | up.mi | down.m |
| PLEK | hsa-miR-183-3p | up.mi | down.m |
| PLEKHA1 | hsa-miR-92b-3p | up.mi | down.m |
| PLEKHA1 | hsa-miR-92a-3p | up.mi | down.m |
| PLEKHA1 | hsa-miR-616-5p | up.mi | down.m |
| PLEKHA1 | hsa-miR-188-5p | up.mi | down.m |
| PLEKHA1 | hsa-miR-3913-5p | up.mi | down.m |
| PLEKHA1 | hsa-miR-93-5p | up.mi | down.m |
| PLEKHA1 | hsa-miR-503-5p | up.mi | down.m |
| PLEKHA1 | hsa-miR-15a-5p | up.mi | down.m |
| PLEKHA1 | hsa-miR-424-5p | up.mi | down.m |
| PLEKHA1 | hsa-miR-590-3p | up.mi | down.m |
| PLEKHA1 | hsa-miR-21-5p | up.mi | down.m |
| PLEKHA2 | hsa-miR-590-3p | up.mi | down.m |
| PLEKHA2 | hsa-miR-155-5p | up.mi | down.m |
| PLEKHA2 | hsa-miR-651-5p | up.mi | down.m |
| PLEKHA2 | hsa-miR-193a-3p | up.mi | down.m |
| PLEKHA2 | hsa-miR-590-5p | up.mi | down.m |
| PLEKHA2 | hsa-miR-193b-3p | up.mi | down.m |
| PLEKHA2 | hsa-miR-34a-3p | up.mi | down.m |
| PLEKHA2 | hsa-miR-1287-5p | up.mi | down.m |
| PLEKHA2 | hsa-miR-21-5p | up.mi | down.m |
| PLEKHH2 | hsa-miR-186-5p | up.mi | down.m |
| PLEKHO2 | hsa-miR-20b-5p | up.mi | down.m |
| PLEKHO2 | hsa-miR-106b-5p | up.mi | down.m |
| PLEKHO2 | hsa-miR-17-5p | up.mi | down.m |
| PLEKHO2 | hsa-miR-30e-5p | up.mi | down.m |
| PLEKHO2 | hsa-miR-93-5p | up.mi | down.m |
| PLEKHO2 | hsa-miR-20a-5p | up.mi | down.m |
| PLIN2 | hsa-miR-148a-5p | up.mi | down.m |
| PLLP | hsa-miR-940 | up.mi | down.m |
| PLLP | hsa-miR-3913-5p | up.mi | down.m |
| PLSCR4 | hsa-miR-3934-3p | up.mi | down.m |
| PLSCR4 | hsa-miR-425-5p | up.mi | down.m |
| PLSCR4 | hsa-miR-382-5p | up.mi | down.m |
| PLXDC2 | hsa-miR-7705 | up.mi | down.m |
| PLXDC2 | hsa-miR-5698 | up.mi | down.m |
| PLXDC2 | hsa-miR-148b-3p | up.mi | down.m |
| PLXDC2 | hsa-miR-744-3p | up.mi | down.m |
| PLXDC2 | hsa-miR-143-5p | up.mi | down.m |
| PLXDC2 | hsa-miR-136-3p | up.mi | down.m |
| PNPLA2 | hsa-miR-2277-5p | up.mi | down.m |
| PNPLA2 | hsa-miR-148b-3p | up.mi | down.m |
| PNPLA6 | hsa-miR-424-5p | up.mi | down.m |
| PNPLA6 | hsa-miR-5698 | up.mi | down.m |
| PNPLA6 | hsa-miR-34a-5p | up.mi | down.m |
| PNPLA6 | hsa-miR-503-5p | up.mi | down.m |
| PNPLA6 | hsa-miR-15a-5p | up.mi | down.m |
| PNPLA6 | hsa-miR-455-3p | up.mi | down.m |
| PNRC1 | hsa-miR-20b-5p | up.mi | down.m |
| PNRC1 | hsa-miR-106a-5p | up.mi | down.m |
| PNRC1 | hsa-miR-301b-3p | up.mi | down.m |
| PNRC1 | hsa-miR-20a-5p | up.mi | down.m |
| PNRC1 | hsa-miR-19a-3p | up.mi | down.m |
| PNRC1 | hsa-miR-454-3p | up.mi | down.m |
| PNRC1 | hsa-miR-199a-3p | up.mi | down.m |
| PNRC1 | hsa-miR-19b-3p | up.mi | down.m |
| PNRC1 | hsa-miR-130b-3p | up.mi | down.m |
| PNRC1 | hsa-miR-3677-5p | up.mi | down.m |
| PNRC1 | hsa-miR-17-5p | up.mi | down.m |
| PNRC1 | hsa-miR-93-5p | up.mi | down.m |
| PNRC1 | hsa-miR-301a-3p | up.mi | down.m |
| PNRC1 | hsa-miR-335-3p | up.mi | down.m |
| PNRC1 | hsa-miR-199b-3p | up.mi | down.m |
| PNRC1 | hsa-miR-106b-5p | up.mi | down.m |
| PNRC1 | hsa-miR-130a-3p | up.mi | down.m |
| PODXL | hsa-miR-96-5p | up.mi | down.m |
| PODXL | hsa-miR-199b-5p | up.mi | down.m |
| PODXL | hsa-miR-192-5p | up.mi | down.m |
| PODXL | hsa-miR-671-5p | up.mi | down.m |
| PODXL | hsa-miR-155-5p | up.mi | down.m |
| PODXL | hsa-miR-3677-3p | up.mi | down.m |
| PODXL | hsa-miR-449a | up.mi | down.m |
| PODXL | hsa-miR-182-5p | up.mi | down.m |
| PODXL | hsa-miR-199a-5p | up.mi | down.m |
| PODXL | hsa-miR-215-5p | up.mi | down.m |
| PODXL | hsa-miR-34a-5p | up.mi | down.m |
| PODXL | hsa-miR-186-5p | up.mi | down.m |
| PPARG | hsa-miR-130b-3p | up.mi | down.m |
| PPARG | hsa-miR-130a-3p | up.mi | down.m |
| PPARG | hsa-miR-215-5p | up.mi | down.m |
| PPARG | hsa-miR-20b-5p | up.mi | down.m |
| PPARG | hsa-miR-192-5p | up.mi | down.m |
| PPARG | hsa-miR-20a-5p | up.mi | down.m |
| PPARGC1B | hsa-miR-361-3p | up.mi | down.m |
| PPARGC1B | hsa-miR-193b-3p | up.mi | down.m |
| PPARGC1B | hsa-miR-30e-5p | up.mi | down.m |
| PPARGC1B | hsa-miR-766-3p | up.mi | down.m |
| PPARGC1B | hsa-miR-186-5p | up.mi | down.m |
| PPFIBP1 | hsa-miR-339-5p | up.mi | down.m |
| PPFIBP1 | hsa-miR-155-5p | up.mi | down.m |
| PPFIBP1 | hsa-miR-3913-5p | up.mi | down.m |
| PPFIBP1 | hsa-miR-340-3p | up.mi | down.m |
| PPFIBP1 | hsa-miR-616-5p | up.mi | down.m |
| PPIL6 | hsa-miR-766-3p | up.mi | down.m |
| PPIL6 | hsa-miR-455-3p | up.mi | down.m |
| PPM1D | hsa-miR-153-3p | up.mi | down.m |
| PPM1D | hsa-miR-3913-5p | up.mi | down.m |
| PPM1F | hsa-miR-769-3p | up.mi | down.m |
| PPM1F | hsa-miR-186-5p | up.mi | down.m |
| PPM1F | hsa-miR-149-5p | up.mi | down.m |
| PPM1F | hsa-miR-324-5p | up.mi | down.m |
| PPP1R14C | hsa-miR-548v | up.mi | down.m |
| PPP1R14C | hsa-miR-181b-3p | up.mi | down.m |
| PPP1R14C | hsa-miR-130a-3p | up.mi | down.m |
| PPP1R14C | hsa-miR-191-5p | up.mi | down.m |
| PPP1R14C | hsa-miR-142-3p | up.mi | down.m |
| PPP1R14C | hsa-miR-301a-3p | up.mi | down.m |
| PPP1R14C | hsa-miR-454-3p | up.mi | down.m |
| PPP1R14C | hsa-miR-128-3p | up.mi | down.m |
| PPP1R14C | hsa-miR-301b-3p | up.mi | down.m |
| PPP1R14C | hsa-miR-130b-3p | up.mi | down.m |
| PPP1R15A | hsa-miR-17-5p | up.mi | down.m |
| PPP1R15A | hsa-miR-136-5p | up.mi | down.m |
| PPP1R15A | hsa-miR-148b-5p | up.mi | down.m |
| PPP1R15A | hsa-miR-539-5p | up.mi | down.m |
| PPP1R16B | hsa-miR-34a-3p | up.mi | down.m |
| PPP1R16B | hsa-miR-501-5p | up.mi | down.m |
| PPP1R16B | hsa-miR-3934-3p | up.mi | down.m |
| PPP1R16B | hsa-miR-3913-5p | up.mi | down.m |
| PPP2CB | hsa-miR-425-5p | up.mi | down.m |
| PPP2CB | hsa-miR-183-5p | up.mi | down.m |
| PPP2CB | hsa-miR-130b-5p | up.mi | down.m |
| PPP2R5A | hsa-miR-590-3p | up.mi | down.m |
| PRDM11 | hsa-miR-17-3p | up.mi | down.m |
| PRDM16 | hsa-miR-96-5p | up.mi | down.m |
| PRDM16 | hsa-miR-615-3p | up.mi | down.m |
| PRELP | hsa-miR-625-5p | up.mi | down.m |
| PRELP | hsa-miR-185-5p | up.mi | down.m |
| PRELP | hsa-miR-5698 | up.mi | down.m |
| PRICKLE1 | hsa-miR-17-5p | up.mi | down.m |
| PRICKLE1 | hsa-miR-203b-3p | up.mi | down.m |
| PRICKLE1 | hsa-miR-192-5p | up.mi | down.m |
| PRICKLE1 | hsa-miR-4668-3p | up.mi | down.m |
| PRICKLE1 | hsa-miR-214-5p | up.mi | down.m |
| PRICKLE1 | hsa-miR-331-3p | up.mi | down.m |
| PRICKLE1 | hsa-miR-2355-5p | up.mi | down.m |
| PRICKLE1 | hsa-miR-3913-5p | up.mi | down.m |
| PRICKLE1 | hsa-miR-215-5p | up.mi | down.m |
| PRICKLE1 | hsa-miR-455-3p | up.mi | down.m |
| PRICKLE2 | hsa-miR-15a-5p | up.mi | down.m |
| PRICKLE2 | hsa-miR-21-5p | up.mi | down.m |
| PRICKLE2 | hsa-miR-424-5p | up.mi | down.m |
| PRICKLE2 | hsa-miR-19a-3p | up.mi | down.m |
| PRICKLE2 | hsa-miR-505-3p | up.mi | down.m |
| PRICKLE2 | hsa-miR-4724-5p | up.mi | down.m |
| PRICKLE2 | hsa-miR-19b-3p | up.mi | down.m |
| PRKCB | hsa-miR-17-5p | up.mi | down.m |
| PRKCB | hsa-miR-130b-5p | up.mi | down.m |
| PRKCB | hsa-miR-20b-5p | up.mi | down.m |
| PRKCB | hsa-miR-106a-5p | up.mi | down.m |
| PRKCB | hsa-miR-381-3p | up.mi | down.m |
| PRKCB | hsa-miR-20a-5p | up.mi | down.m |
| PRKCB | hsa-miR-33a-5p | up.mi | down.m |
| PRKCB | hsa-miR-93-5p | up.mi | down.m |
| PRKCB | hsa-miR-142-5p | up.mi | down.m |
| PRKCB | hsa-miR-106b-5p | up.mi | down.m |
| PRKCE | hsa-miR-21-5p | up.mi | down.m |
| PRKCE | hsa-miR-31-5p | up.mi | down.m |
| PRKCE | hsa-miR-96-5p | up.mi | down.m |
| PRKCE | hsa-miR-205-5p | up.mi | down.m |
| PRKCH | hsa-miR-432-5p | up.mi | down.m |
| PRKCH | hsa-miR-493-3p | up.mi | down.m |
| PRKCZ | hsa-miR-200c-3p | up.mi | down.m |
| PRKD1 | hsa-miR-766-3p | up.mi | down.m |
| PRKD1 | hsa-miR-34a-5p | up.mi | down.m |
| PRKG1 | hsa-miR-20a-5p | up.mi | down.m |
| PRKG2 | hsa-miR-452-3p | up.mi | down.m |
| PRNP | hsa-miR-409-3p | up.mi | down.m |
| PRNP | hsa-miR-215-5p | up.mi | down.m |
| PRNP | hsa-miR-615-3p | up.mi | down.m |
| PRNP | hsa-miR-301b-3p | up.mi | down.m |
| PRNP | hsa-miR-106b-5p | up.mi | down.m |
| PRNP | hsa-miR-301a-3p | up.mi | down.m |
| PRNP | hsa-miR-20b-5p | up.mi | down.m |
| PRNP | hsa-miR-93-5p | up.mi | down.m |
| PRNP | hsa-miR-148b-3p | up.mi | down.m |
| PRNP | hsa-miR-20a-5p | up.mi | down.m |
| PRNP | hsa-miR-148a-3p | up.mi | down.m |
| PRNP | hsa-miR-17-5p | up.mi | down.m |
| PRNP | hsa-miR-7-1-3p | up.mi | down.m |
| PRNP | hsa-miR-130b-3p | up.mi | down.m |
| PRNP | hsa-miR-193b-3p | up.mi | down.m |
| PRNP | hsa-miR-192-5p | up.mi | down.m |
| PRNP | hsa-miR-495-3p | up.mi | down.m |
| PRNP | hsa-miR-106a-5p | up.mi | down.m |
| PRNP | hsa-miR-382-5p | up.mi | down.m |
| PRNP | hsa-miR-454-3p | up.mi | down.m |
| PRNP | hsa-miR-130a-3p | up.mi | down.m |
| PRNP | hsa-miR-188-3p | up.mi | down.m |
| PRR5L | hsa-miR-27b-5p | up.mi | down.m |
| PRRG1 | hsa-miR-93-5p | up.mi | down.m |
| PRRG1 | hsa-miR-17-5p | up.mi | down.m |
| PRRG1 | hsa-miR-106b-5p | up.mi | down.m |
| PRRG1 | hsa-miR-20a-5p | up.mi | down.m |
| PRX | hsa-miR-3127-5p | up.mi | down.m |
| PRX | hsa-miR-5698 | up.mi | down.m |
| PTAFR | hsa-miR-532-3p | up.mi | down.m |
| PTAFR | hsa-miR-149-5p | up.mi | down.m |
| PTAFR | hsa-miR-188-3p | up.mi | down.m |
| PTAFR | hsa-miR-1307-3p | up.mi | down.m |
| PTGER4 | hsa-miR-93-5p | up.mi | down.m |
| PTGER4 | hsa-miR-92b-3p | up.mi | down.m |
| PTGER4 | hsa-miR-20a-5p | up.mi | down.m |
| PTGER4 | hsa-miR-17-5p | up.mi | down.m |
| PTGER4 | hsa-miR-92a-3p | up.mi | down.m |
| PTGER4 | hsa-miR-20b-5p | up.mi | down.m |
| PTGER4 | hsa-miR-106b-5p | up.mi | down.m |
| PTGFR | hsa-miR-21-5p | up.mi | down.m |
| PTGIS | hsa-miR-20b-5p | up.mi | down.m |
| PTGIS | hsa-miR-106a-5p | up.mi | down.m |
| PTGIS | hsa-miR-340-3p | up.mi | down.m |
| PTGIS | hsa-miR-93-5p | up.mi | down.m |
| PTGIS | hsa-miR-106b-5p | up.mi | down.m |
| PTGIS | hsa-miR-4668-3p | up.mi | down.m |
| PTGIS | hsa-miR-642a-5p | up.mi | down.m |
| PTGIS | hsa-miR-20a-5p | up.mi | down.m |
| PTGIS | hsa-miR-17-5p | up.mi | down.m |
| PTGS2 | hsa-miR-199a-5p | up.mi | down.m |
| PTGS2 | hsa-miR-128-3p | up.mi | down.m |
| PTGS2 | hsa-miR-199a-3p | up.mi | down.m |
| PTGS2 | hsa-miR-589-5p | up.mi | down.m |
| PTK2B | hsa-miR-940 | up.mi | down.m |
| PTMS | hsa-miR-34a-5p | up.mi | down.m |
| PTMS | hsa-miR-155-5p | up.mi | down.m |
| PTMS | hsa-miR-423-3p | up.mi | down.m |
| PTMS | hsa-miR-193b-3p | up.mi | down.m |
| PTMS | hsa-miR-940 | up.mi | down.m |
| PTMS | hsa-miR-22-3p | up.mi | down.m |
| PTN | hsa-miR-155-5p | up.mi | down.m |
| PTPN1 | hsa-miR-210-3p | up.mi | down.m |
| PTPN13 | hsa-miR-186-5p | up.mi | down.m |
| PTPN13 | hsa-miR-331-3p | up.mi | down.m |
| PTPN13 | hsa-miR-194-5p | up.mi | down.m |
| PTPN13 | hsa-miR-200c-3p | up.mi | down.m |
| PTPN13 | hsa-miR-92a-3p | up.mi | down.m |
| PTPN14 | hsa-miR-130a-5p | up.mi | down.m |
| PTPN14 | hsa-miR-105-5p | up.mi | down.m |
| PTPN14 | hsa-miR-4668-3p | up.mi | down.m |
| PTPN14 | hsa-miR-21-5p | up.mi | down.m |
| PTPN14 | hsa-miR-629-5p | up.mi | down.m |
| PTPN14 | hsa-miR-16-2-3p | up.mi | down.m |
| PTPN14 | hsa-miR-642a-5p | up.mi | down.m |
| PTPN14 | hsa-miR-590-5p | up.mi | down.m |
| PTPN14 | hsa-miR-324-3p | up.mi | down.m |
| PTPN14 | hsa-miR-940 | up.mi | down.m |
| PTPN21 | hsa-miR-615-3p | up.mi | down.m |
| PTPRB | hsa-miR-455-5p | up.mi | down.m |
| PTPRB | hsa-miR-629-3p | up.mi | down.m |
| PTPRB | hsa-miR-629-5p | up.mi | down.m |
| PTPRB | hsa-miR-561-5p | up.mi | down.m |
| PTPRB | hsa-miR-19a-3p | up.mi | down.m |
| PTPRB | hsa-miR-19b-3p | up.mi | down.m |
| PTPRD | hsa-miR-200c-3p | up.mi | down.m |
| PTPRD | hsa-miR-424-5p | up.mi | down.m |
| PTPRD | hsa-miR-503-5p | up.mi | down.m |
| PTPRD | hsa-miR-141-3p | up.mi | down.m |
| PTPRD | hsa-miR-200b-3p | up.mi | down.m |
| PTPRD | hsa-miR-200a-3p | up.mi | down.m |
| PTPRD | hsa-miR-429 | up.mi | down.m |
| PTPRD | hsa-miR-15a-5p | up.mi | down.m |
| PTPRG | hsa-miR-301a-3p | up.mi | down.m |
| PTPRG | hsa-miR-19b-3p | up.mi | down.m |
| PTPRG | hsa-miR-130a-3p | up.mi | down.m |
| PTPRG | hsa-miR-130b-3p | up.mi | down.m |
| PTPRG | hsa-miR-19a-3p | up.mi | down.m |
| PTPRG | hsa-miR-454-3p | up.mi | down.m |
| PTPRG | hsa-miR-301b-3p | up.mi | down.m |
| PTPRG | hsa-miR-193b-3p | up.mi | down.m |
| PTPRG | hsa-miR-642a-5p | up.mi | down.m |
| PTPRM | hsa-miR-205-5p | up.mi | down.m |
| PTPRM | hsa-miR-130b-5p | up.mi | down.m |
| PTPRM | hsa-miR-450b-5p | up.mi | down.m |
| PTPRM | hsa-miR-452-3p | up.mi | down.m |
| PTPRN2 | hsa-miR-539-5p | up.mi | down.m |
| PTPRN2 | hsa-miR-616-5p | up.mi | down.m |
| PTPRO | hsa-miR-17-5p | up.mi | down.m |
| PTPRO | hsa-miR-20a-5p | up.mi | down.m |
| PTX3 | hsa-miR-21-5p | up.mi | down.m |
| PTX3 | hsa-miR-224-5p | up.mi | down.m |
| PTX3 | hsa-miR-9-5p | up.mi | down.m |
| PXDC1 | hsa-miR-642a-5p | up.mi | down.m |
| PXDC1 | hsa-miR-192-5p | up.mi | down.m |
| PXMP4 | hsa-miR-629-3p | up.mi | down.m |
| PXMP4 | hsa-miR-2355-5p | up.mi | down.m |
| PXMP4 | hsa-miR-143-5p | up.mi | down.m |
| PXMP4 | hsa-miR-455-3p | up.mi | down.m |
| PXMP4 | hsa-miR-200c-5p | up.mi | down.m |
| PXMP4 | hsa-miR-616-5p | up.mi | down.m |
| PXMP4 | hsa-miR-550a-3p | up.mi | down.m |
| PXMP4 | hsa-miR-6510-3p | up.mi | down.m |
| PXMP4 | hsa-miR-361-3p | up.mi | down.m |
| PXMP4 | hsa-miR-1307-3p | up.mi | down.m |
| PXMP4 | hsa-miR-532-3p | up.mi | down.m |
| QDPR | hsa-miR-582-5p | up.mi | down.m |
| QDPR | hsa-miR-34a-5p | up.mi | down.m |
| QDPR | hsa-miR-940 | up.mi | down.m |
| QKI | hsa-miR-19b-3p | up.mi | down.m |
| QKI | hsa-miR-17-5p | up.mi | down.m |
| QKI | hsa-miR-224-5p | up.mi | down.m |
| QKI | hsa-miR-106b-5p | up.mi | down.m |
| QKI | hsa-miR-301b-3p | up.mi | down.m |
| QKI | hsa-miR-93-5p | up.mi | down.m |
| QKI | hsa-miR-106a-5p | up.mi | down.m |
| QKI | hsa-miR-301a-3p | up.mi | down.m |
| QKI | hsa-miR-20a-5p | up.mi | down.m |
| QKI | hsa-miR-19a-3p | up.mi | down.m |
| QKI | hsa-miR-199a-3p | up.mi | down.m |
| QKI | hsa-miR-200b-3p | up.mi | down.m |
| QKI | hsa-miR-181b-3p | up.mi | down.m |
| QKI | hsa-miR-4668-3p | up.mi | down.m |
| QKI | hsa-miR-200a-3p | up.mi | down.m |
| QKI | hsa-miR-454-3p | up.mi | down.m |
| QKI | hsa-miR-141-3p | up.mi | down.m |
| QKI | hsa-miR-130b-3p | up.mi | down.m |
| QKI | hsa-miR-425-5p | up.mi | down.m |
| QKI | hsa-miR-130a-3p | up.mi | down.m |
| QKI | hsa-miR-369-3p | up.mi | down.m |
| QKI | hsa-miR-331-3p | up.mi | down.m |
| QKI | hsa-miR-199b-3p | up.mi | down.m |
| QKI | hsa-miR-20b-5p | up.mi | down.m |
| QKI | hsa-miR-200c-3p | up.mi | down.m |
| QKI | hsa-miR-455-5p | up.mi | down.m |
| QKI | hsa-miR-148a-3p | up.mi | down.m |
| QKI | hsa-miR-429 | up.mi | down.m |
| RAB11FIP1 | hsa-miR-93-5p | up.mi | down.m |
| RAB11FIP1 | hsa-miR-106a-5p | up.mi | down.m |
| RAB11FIP1 | hsa-miR-20b-5p | up.mi | down.m |
| RAB11FIP1 | hsa-miR-20a-5p | up.mi | down.m |
| RAB11FIP1 | hsa-miR-194-5p | up.mi | down.m |
| RAB11FIP1 | hsa-miR-324-5p | up.mi | down.m |
| RAB11FIP1 | hsa-miR-205-5p | up.mi | down.m |
| RAB11FIP1 | hsa-miR-324-3p | up.mi | down.m |
| RAB11FIP1 | hsa-miR-1306-5p | up.mi | down.m |
| RAB11FIP1 | hsa-miR-106b-5p | up.mi | down.m |
| RAB11FIP1 | hsa-miR-130a-3p | up.mi | down.m |
| RAB11FIP1 | hsa-miR-17-5p | up.mi | down.m |
| RAB11FIP1 | hsa-miR-301a-3p | up.mi | down.m |
| RAB11FIP1 | hsa-miR-149-5p | up.mi | down.m |
| RAB11FIP1 | hsa-miR-130b-3p | up.mi | down.m |
| RAB11FIP1 | hsa-miR-454-3p | up.mi | down.m |
| RAB11FIP1 | hsa-miR-615-3p | up.mi | down.m |
| RAB11FIP1 | hsa-miR-301b-3p | up.mi | down.m |
| RAB11FIP1 | hsa-miR-744-3p | up.mi | down.m |
| RAB11FIP1 | hsa-miR-29b-3p | up.mi | down.m |
| RAB11FIP2 | hsa-miR-215-5p | up.mi | down.m |
| RAB11FIP2 | hsa-miR-155-5p | up.mi | down.m |
| RAB11FIP2 | hsa-miR-192-5p | up.mi | down.m |
| RAB11FIP2 | hsa-miR-15a-5p | up.mi | down.m |
| RAB11FIP2 | hsa-miR-21-5p | up.mi | down.m |
| RAB14 | hsa-miR-19a-3p | up.mi | down.m |
| RAB14 | hsa-miR-181c-3p | up.mi | down.m |
| RAB14 | hsa-miR-301b-3p | up.mi | down.m |
| RAB14 | hsa-miR-409-5p | up.mi | down.m |
| RAB14 | hsa-miR-19b-3p | up.mi | down.m |
| RAB14 | hsa-miR-27b-5p | up.mi | down.m |
| RAB14 | hsa-miR-151a-5p | up.mi | down.m |
| RAB14 | hsa-miR-155-5p | up.mi | down.m |
| RAB14 | hsa-miR-148a-3p | up.mi | down.m |
| RAB14 | hsa-miR-148b-3p | up.mi | down.m |
| RAB14 | hsa-miR-130a-3p | up.mi | down.m |
| RAB14 | hsa-miR-130b-3p | up.mi | down.m |
| RAB14 | hsa-miR-454-3p | up.mi | down.m |
| RAB14 | hsa-miR-301a-3p | up.mi | down.m |
| RAB31 | hsa-miR-196a-5p | up.mi | down.m |
| RAB31 | hsa-miR-331-5p | up.mi | down.m |
| RAB31 | hsa-miR-425-5p | up.mi | down.m |
| RAB31 | hsa-miR-493-3p | up.mi | down.m |
| RAB31 | hsa-miR-7-1-3p | up.mi | down.m |
| RAB31 | hsa-miR-495-3p | up.mi | down.m |
| RAB32 | hsa-miR-21-3p | up.mi | down.m |
| RAB32 | hsa-miR-1287-5p | up.mi | down.m |
| RAB32 | hsa-miR-369-3p | up.mi | down.m |
| RAB32 | hsa-miR-193a-3p | up.mi | down.m |
| RAB32 | hsa-miR-185-5p | up.mi | down.m |
| RAB32 | hsa-miR-181c-3p | up.mi | down.m |
| RAB32 | hsa-miR-339-5p | up.mi | down.m |
| RAB32 | hsa-miR-539-5p | up.mi | down.m |
| RAB32 | hsa-miR-193b-3p | up.mi | down.m |
| RAB40A | hsa-miR-766-3p | up.mi | down.m |
| RAB40A | hsa-miR-1301-3p | up.mi | down.m |
| RAB8B | hsa-miR-128-3p | up.mi | down.m |
| RAB8B | hsa-miR-215-5p | up.mi | down.m |
| RAB8B | hsa-miR-192-5p | up.mi | down.m |
| RAB8B | hsa-miR-92a-3p | up.mi | down.m |
| RAB8B | hsa-miR-93-5p | up.mi | down.m |
| RAB8B | hsa-miR-369-3p | up.mi | down.m |
| RAB8B | hsa-miR-9-5p | up.mi | down.m |
| RAB8B | hsa-miR-92b-3p | up.mi | down.m |
| RAB8B | hsa-miR-200a-3p | up.mi | down.m |
| RAB8B | hsa-miR-19b-3p | up.mi | down.m |
| RAB8B | hsa-miR-19a-3p | up.mi | down.m |
| RAB8B | hsa-miR-141-3p | up.mi | down.m |
| RADIL | hsa-miR-215-5p | up.mi | down.m |
| RADIL | hsa-miR-192-5p | up.mi | down.m |
| RADIL | hsa-miR-766-3p | up.mi | down.m |
| RADIL | hsa-miR-615-3p | up.mi | down.m |
| RANBP10 | hsa-miR-185-5p | up.mi | down.m |
| RANBP10 | hsa-miR-615-3p | up.mi | down.m |
| RANBP10 | hsa-miR-186-5p | up.mi | down.m |
| RAP1A | hsa-miR-19b-3p | up.mi | down.m |
| RAP1A | hsa-miR-19a-3p | up.mi | down.m |
| RAP1A | hsa-miR-337-3p | up.mi | down.m |
| RAP1A | hsa-miR-340-3p | up.mi | down.m |
| RAP1A | hsa-miR-18a-5p | up.mi | down.m |
| RAPGEF1 | hsa-miR-561-5p | up.mi | down.m |
| RAPGEF1 | hsa-miR-766-3p | up.mi | down.m |
| RAPGEF1 | hsa-miR-615-3p | up.mi | down.m |
| RAPGEF1 | hsa-miR-339-5p | up.mi | down.m |
| RAPGEF1 | hsa-miR-185-5p | up.mi | down.m |
| RAPGEF1 | hsa-miR-193b-3p | up.mi | down.m |
| RAPGEF1 | hsa-miR-3127-5p | up.mi | down.m |
| RAPGEF2 | hsa-miR-615-3p | up.mi | down.m |
| RAPGEF2 | hsa-miR-149-5p | up.mi | down.m |
| RAPGEF2 | hsa-miR-19b-3p | up.mi | down.m |
| RAPGEF2 | hsa-miR-155-5p | up.mi | down.m |
| RAPGEF2 | hsa-miR-339-5p | up.mi | down.m |
| RAPGEF2 | hsa-miR-335-3p | up.mi | down.m |
| RAPGEF2 | hsa-miR-29b-2-5p | up.mi | down.m |
| RAPGEF2 | hsa-miR-19a-3p | up.mi | down.m |
| RAPGEF4 | hsa-miR-106b-5p | up.mi | down.m |
| RAPGEF4 | hsa-miR-19b-3p | up.mi | down.m |
| RAPGEF4 | hsa-miR-20b-5p | up.mi | down.m |
| RAPGEF4 | hsa-miR-20a-5p | up.mi | down.m |
| RAPGEF4 | hsa-miR-19a-3p | up.mi | down.m |
| RAPGEF4 | hsa-miR-93-5p | up.mi | down.m |
| RAPGEF4 | hsa-miR-17-5p | up.mi | down.m |
| RASGEF1B | hsa-miR-1287-3p | up.mi | down.m |
| RASL11A | hsa-miR-148a-5p | up.mi | down.m |
| RASSF2 | hsa-miR-141-3p | up.mi | down.m |
| RASSF2 | hsa-miR-429 | up.mi | down.m |
| RASSF2 | hsa-miR-424-5p | up.mi | down.m |
| RASSF2 | hsa-miR-455-5p | up.mi | down.m |
| RASSF2 | hsa-miR-19b-3p | up.mi | down.m |
| RASSF2 | hsa-miR-200a-3p | up.mi | down.m |
| RASSF2 | hsa-miR-200c-3p | up.mi | down.m |
| RASSF2 | hsa-miR-19a-3p | up.mi | down.m |
| RASSF2 | hsa-miR-200b-3p | up.mi | down.m |
| RASSF2 | hsa-miR-15a-5p | up.mi | down.m |
| RASSF3 | hsa-miR-193b-3p | up.mi | down.m |
| RASSF3 | hsa-miR-18a-5p | up.mi | down.m |
| RASSF3 | hsa-miR-20a-3p | up.mi | down.m |
| RASSF5 | hsa-miR-15a-5p | up.mi | down.m |
| RASSF5 | hsa-miR-19a-3p | up.mi | down.m |
| RASSF5 | hsa-miR-193b-3p | up.mi | down.m |
| RASSF5 | hsa-miR-19b-3p | up.mi | down.m |
| RASSF5 | hsa-miR-214-5p | up.mi | down.m |
| RASSF8 | hsa-miR-19b-1-5p | up.mi | down.m |
| RASSF8 | hsa-miR-429 | up.mi | down.m |
| RASSF8 | hsa-miR-4724-5p | up.mi | down.m |
| RASSF8 | hsa-miR-200c-3p | up.mi | down.m |
| RASSF8 | hsa-miR-224-5p | up.mi | down.m |
| RASSF8 | hsa-miR-192-5p | up.mi | down.m |
| RASSF8 | hsa-miR-9-5p | up.mi | down.m |
| RASSF8 | hsa-miR-200b-3p | up.mi | down.m |
| RASSF8 | hsa-miR-130b-3p | up.mi | down.m |
| RASSF8 | hsa-miR-148a-3p | up.mi | down.m |
| RASSF8 | hsa-miR-629-5p | up.mi | down.m |
| RASSF9 | hsa-miR-766-3p | up.mi | down.m |
| RASSF9 | hsa-miR-340-3p | up.mi | down.m |
| RASSF9 | hsa-miR-561-5p | up.mi | down.m |
| RASSF9 | hsa-miR-940 | up.mi | down.m |
| RASSF9 | hsa-miR-3913-5p | up.mi | down.m |
| RAVER2 | hsa-miR-615-3p | up.mi | down.m |
| RAVER2 | hsa-miR-149-5p | up.mi | down.m |
| RBMS2 | hsa-miR-1277-3p | up.mi | down.m |
| RBMS2 | hsa-miR-143-5p | up.mi | down.m |
| RBMS2 | hsa-miR-550a-5p | up.mi | down.m |
| RBMS2 | hsa-miR-92a-3p | up.mi | down.m |
| RBMS2 | hsa-miR-92b-3p | up.mi | down.m |
| RBMS2 | hsa-miR-455-3p | up.mi | down.m |
| RBMS2 | hsa-miR-3913-5p | up.mi | down.m |
| RBMS2 | hsa-miR-194-5p | up.mi | down.m |
| RBMS2 | hsa-miR-214-5p | up.mi | down.m |
| RBP2 | hsa-miR-34a-5p | up.mi | down.m |
| RBP2 | hsa-miR-212-3p | up.mi | down.m |
| RBP7 | hsa-miR-192-5p | up.mi | down.m |
| RBP7 | hsa-miR-215-5p | up.mi | down.m |
| RBPMS | hsa-miR-21-3p | up.mi | down.m |
| RCAN1 | hsa-miR-767-3p | up.mi | down.m |
| RCAN1 | hsa-miR-26b-3p | up.mi | down.m |
| RCAN1 | hsa-miR-339-5p | up.mi | down.m |
| RCAN1 | hsa-miR-134-5p | up.mi | down.m |
| RCAN1 | hsa-miR-92a-3p | up.mi | down.m |
| RCAN1 | hsa-miR-130b-5p | up.mi | down.m |
| RCAN1 | hsa-miR-4668-3p | up.mi | down.m |
| RCAN1 | hsa-miR-22-5p | up.mi | down.m |
| RCAN1 | hsa-miR-34a-5p | up.mi | down.m |
| RCAN2 | hsa-miR-142-5p | up.mi | down.m |
| RCAN2 | hsa-miR-148a-5p | up.mi | down.m |
| RCBTB2 | hsa-miR-192-5p | up.mi | down.m |
| RDX | hsa-miR-331-3p | up.mi | down.m |
| RDX | hsa-miR-196b-5p | up.mi | down.m |
| RDX | hsa-miR-409-3p | up.mi | down.m |
| RDX | hsa-miR-196a-5p | up.mi | down.m |
| RDX | hsa-miR-3934-3p | up.mi | down.m |
| RDX | hsa-miR-31-5p | up.mi | down.m |
| RECK | hsa-miR-409-3p | up.mi | down.m |
| RECK | hsa-miR-590-5p | up.mi | down.m |
| RECK | hsa-miR-21-5p | up.mi | down.m |
| RECK | hsa-miR-96-5p | up.mi | down.m |
| RECK | hsa-miR-15a-5p | up.mi | down.m |
| RECK | hsa-miR-29b-2-5p | up.mi | down.m |
| RECK | hsa-miR-92b-3p | up.mi | down.m |
| RECK | hsa-miR-135b-5p | up.mi | down.m |
| RECK | hsa-miR-940 | up.mi | down.m |
| RECK | hsa-miR-182-5p | up.mi | down.m |
| RECK | hsa-miR-183-5p | up.mi | down.m |
| RECK | hsa-miR-503-5p | up.mi | down.m |
| RECK | hsa-miR-424-5p | up.mi | down.m |
| RECK | hsa-miR-15b-3p | up.mi | down.m |
| REEP1 | hsa-miR-450b-5p | up.mi | down.m |
| REEP1 | hsa-miR-192-5p | up.mi | down.m |
| REEP1 | hsa-miR-33b-5p | up.mi | down.m |
| REEP1 | hsa-miR-215-5p | up.mi | down.m |
| REEP1 | hsa-miR-33a-5p | up.mi | down.m |
| REM1 | hsa-miR-19b-3p | up.mi | down.m |
| REV3L | hsa-miR-92a-3p | up.mi | down.m |
| REV3L | hsa-miR-30e-5p | up.mi | down.m |
| REV3L | hsa-miR-21-5p | up.mi | down.m |
| REV3L | hsa-miR-92b-3p | up.mi | down.m |
| REV3L | hsa-miR-183-5p | up.mi | down.m |
| REV3L | hsa-miR-93-5p | up.mi | down.m |
| RFX2 | hsa-miR-151a-5p | up.mi | down.m |
| RGL1 | hsa-miR-19b-3p | up.mi | down.m |
| RGL1 | hsa-miR-19a-3p | up.mi | down.m |
| RGL1 | hsa-miR-155-5p | up.mi | down.m |
| RGS16 | hsa-miR-153-5p | up.mi | down.m |
| RGS16 | hsa-miR-2355-3p | up.mi | down.m |
| RGS16 | hsa-miR-181b-5p | up.mi | down.m |
| RGS16 | hsa-miR-181d-5p | up.mi | down.m |
| RGS2 | hsa-miR-21-3p | up.mi | down.m |
| RGS2 | hsa-miR-182-5p | up.mi | down.m |
| RGS2 | hsa-miR-17-3p | up.mi | down.m |
| RGS2 | hsa-miR-183-5p | up.mi | down.m |
| RGS2 | hsa-miR-505-3p | up.mi | down.m |
| RGS2 | hsa-miR-96-5p | up.mi | down.m |
| RGS2 | hsa-miR-191-5p | up.mi | down.m |
| RGS2 | hsa-miR-22-3p | up.mi | down.m |
| RGS5 | hsa-miR-92a-3p | up.mi | down.m |
| RGS5 | hsa-miR-142-3p | up.mi | down.m |
| RGS5 | hsa-miR-642a-5p | up.mi | down.m |
| RGS5 | hsa-miR-9-3p | up.mi | down.m |
| RGS5 | hsa-miR-20a-5p | up.mi | down.m |
| RHOB | hsa-miR-19b-3p | up.mi | down.m |
| RHOB | hsa-miR-7-1-3p | up.mi | down.m |
| RHOB | hsa-miR-21-5p | up.mi | down.m |
| RHOB | hsa-miR-19a-3p | up.mi | down.m |
| RHOB | hsa-miR-590-3p | up.mi | down.m |
| RHOB | hsa-miR-186-5p | up.mi | down.m |
| RHOB | hsa-miR-153-5p | up.mi | down.m |
| RHOB | hsa-miR-642a-5p | up.mi | down.m |
| RHOBTB2 | hsa-miR-106b-5p | up.mi | down.m |
| RIMS4 | hsa-miR-142-5p | up.mi | down.m |
| RIMS4 | hsa-miR-130b-5p | up.mi | down.m |
| RIMS4 | hsa-miR-505-3p | up.mi | down.m |
| RIMS4 | hsa-miR-186-5p | up.mi | down.m |
| RIN2 | hsa-miR-200c-3p | up.mi | down.m |
| RIN2 | hsa-miR-429 | up.mi | down.m |
| RIN2 | hsa-miR-200b-3p | up.mi | down.m |
| RIN2 | hsa-miR-141-3p | up.mi | down.m |
| RIN2 | hsa-miR-200a-3p | up.mi | down.m |
| RMDN3 | hsa-miR-505-5p | up.mi | down.m |
| RMDN3 | hsa-miR-193b-3p | up.mi | down.m |
| RMDN3 | hsa-miR-93-5p | up.mi | down.m |
| RNASE1 | hsa-miR-19b-3p | up.mi | down.m |
| RND1 | hsa-miR-199a-5p | up.mi | down.m |
| RND1 | hsa-miR-128-3p | up.mi | down.m |
| RND3 | hsa-miR-200b-3p | up.mi | down.m |
| RND3 | hsa-miR-106a-5p | up.mi | down.m |
| RND3 | hsa-miR-200c-3p | up.mi | down.m |
| RND3 | hsa-miR-17-5p | up.mi | down.m |
| RND3 | hsa-miR-93-5p | up.mi | down.m |
| RNF11 | hsa-miR-590-3p | up.mi | down.m |
| RNF11 | hsa-miR-19b-3p | up.mi | down.m |
| RNF11 | hsa-miR-130a-3p | up.mi | down.m |
| RNF11 | hsa-miR-130b-3p | up.mi | down.m |
| RNF11 | hsa-miR-455-3p | up.mi | down.m |
| RNF11 | hsa-miR-17-3p | up.mi | down.m |
| RNF11 | hsa-miR-454-3p | up.mi | down.m |
| RNF11 | hsa-miR-199a-5p | up.mi | down.m |
| RNF11 | hsa-miR-19a-3p | up.mi | down.m |
| RNF11 | hsa-miR-21-5p | up.mi | down.m |
| RNF11 | hsa-miR-301a-3p | up.mi | down.m |
| RNF11 | hsa-miR-381-3p | up.mi | down.m |
| RNF11 | hsa-miR-199b-5p | up.mi | down.m |
| RNF11 | hsa-miR-105-5p | up.mi | down.m |
| RNF11 | hsa-miR-1307-3p | up.mi | down.m |
| RNF11 | hsa-miR-301b-3p | up.mi | down.m |
| RNF122 | hsa-miR-30e-5p | up.mi | down.m |
| RNF128 | hsa-miR-629-3p | up.mi | down.m |
| RNF128 | hsa-miR-92a-3p | up.mi | down.m |
| RNF128 | hsa-miR-335-3p | up.mi | down.m |
| RNF144B | hsa-miR-128-3p | up.mi | down.m |
| RNF182 | hsa-miR-106a-5p | up.mi | down.m |
| RNF182 | hsa-miR-128-3p | up.mi | down.m |
| RNF38 | hsa-miR-653-5p | up.mi | down.m |
| RNF38 | hsa-miR-4668-3p | up.mi | down.m |
| RNF38 | hsa-miR-15a-5p | up.mi | down.m |
| RNF38 | hsa-miR-409-3p | up.mi | down.m |
| RNF38 | hsa-miR-616-5p | up.mi | down.m |
| RNF38 | hsa-miR-142-3p | up.mi | down.m |
| RNF38 | hsa-miR-130a-5p | up.mi | down.m |
| ROBO4 | hsa-miR-337-3p | up.mi | down.m |
| ROPN1L | hsa-miR-215-5p | up.mi | down.m |
| ROPN1L | hsa-miR-128-3p | up.mi | down.m |
| ROPN1L | hsa-miR-192-5p | up.mi | down.m |
| ROPN1L | hsa-miR-148b-3p | up.mi | down.m |
| ROR1 | hsa-miR-19b-3p | up.mi | down.m |
| RORA | hsa-miR-186-5p | up.mi | down.m |
| RORA | hsa-miR-106a-5p | up.mi | down.m |
| RORA | hsa-miR-93-5p | up.mi | down.m |
| RORA | hsa-miR-758-3p | up.mi | down.m |
| RORA | hsa-miR-154-3p | up.mi | down.m |
| RORA | hsa-miR-19a-3p | up.mi | down.m |
| RORA | hsa-miR-381-3p | up.mi | down.m |
| RORA | hsa-miR-20a-5p | up.mi | down.m |
| RORA | hsa-miR-18a-5p | up.mi | down.m |
| RORA | hsa-miR-20b-5p | up.mi | down.m |
| RORA | hsa-miR-19b-3p | up.mi | down.m |
| RORA | hsa-miR-155-5p | up.mi | down.m |
| RORA | hsa-miR-127-5p | up.mi | down.m |
| RORA | hsa-miR-1277-3p | up.mi | down.m |
| RORA | hsa-miR-17-5p | up.mi | down.m |
| RORA | hsa-miR-377-3p | up.mi | down.m |
| RORA | hsa-miR-92a-3p | up.mi | down.m |
| RORA | hsa-miR-106b-5p | up.mi | down.m |
| RORA | hsa-miR-153-5p | up.mi | down.m |
| RORA | hsa-miR-33b-5p | up.mi | down.m |
| ROS1 | hsa-miR-33a-5p | up.mi | down.m |
| RP2 | hsa-miR-92a-3p | up.mi | down.m |
| RP2 | hsa-miR-181b-5p | up.mi | down.m |
| RP2 | hsa-miR-181d-5p | up.mi | down.m |
| RP2 | hsa-miR-155-5p | up.mi | down.m |
| RP2 | hsa-miR-92b-3p | up.mi | down.m |
| RP2 | hsa-miR-21-5p | up.mi | down.m |
| RRAD | hsa-miR-193b-5p | up.mi | down.m |
| RRAD | hsa-miR-3913-5p | up.mi | down.m |
| RRAGD | hsa-miR-106b-5p | up.mi | down.m |
| RRAGD | hsa-miR-449a | up.mi | down.m |
| RRAGD | hsa-miR-20a-5p | up.mi | down.m |
| RRAGD | hsa-miR-19b-3p | up.mi | down.m |
| RRAGD | hsa-miR-301b-3p | up.mi | down.m |
| RRAGD | hsa-miR-130a-3p | up.mi | down.m |
| RRAGD | hsa-miR-130b-3p | up.mi | down.m |
| RRAGD | hsa-miR-19a-3p | up.mi | down.m |
| RRAGD | hsa-let-7c-3p | up.mi | down.m |
| RRAGD | hsa-miR-454-3p | up.mi | down.m |
| RRAGD | hsa-miR-93-5p | up.mi | down.m |
| RRAGD | hsa-miR-301a-3p | up.mi | down.m |
| RRAGD | hsa-miR-20b-5p | up.mi | down.m |
| RRAGD | hsa-miR-106a-5p | up.mi | down.m |
| RRAGD | hsa-miR-34a-5p | up.mi | down.m |
| RRAGD | hsa-miR-744-3p | up.mi | down.m |
| RRAGD | hsa-miR-17-5p | up.mi | down.m |
| RRAS | hsa-miR-449a | up.mi | down.m |
| RRAS | hsa-miR-34a-5p | up.mi | down.m |
| RS1 | hsa-miR-15a-5p | up.mi | down.m |
| RS1 | hsa-miR-503-5p | up.mi | down.m |
| RS1 | hsa-miR-664a-3p | up.mi | down.m |
| RS1 | hsa-miR-376b-3p | up.mi | down.m |
| RS1 | hsa-miR-424-5p | up.mi | down.m |
| RSPH4A | hsa-miR-141-5p | up.mi | down.m |
| RSPH4A | hsa-miR-452-3p | up.mi | down.m |
| RSPO1 | hsa-miR-24-2-5p | up.mi | down.m |
| RSPO1 | hsa-miR-24-1-5p | up.mi | down.m |
| RTKN2 | hsa-miR-193b-3p | up.mi | down.m |
| RTKN2 | hsa-miR-215-5p | up.mi | down.m |
| RTKN2 | hsa-miR-92b-3p | up.mi | down.m |
| RTKN2 | hsa-miR-192-5p | up.mi | down.m |
| RUSC2 | hsa-miR-324-5p | up.mi | down.m |
| RUSC2 | hsa-miR-642a-5p | up.mi | down.m |
| RXRA | hsa-miR-128-3p | up.mi | down.m |
| RXRA | hsa-miR-423-3p | up.mi | down.m |
| S100A4 | hsa-miR-187-3p | up.mi | down.m |
| S1PR1 | hsa-miR-155-5p | up.mi | down.m |
| S1PR1 | hsa-miR-148a-3p | up.mi | down.m |
| S1PR1 | hsa-miR-532-3p | up.mi | down.m |
| SACM1L | hsa-miR-92a-3p | up.mi | down.m |
| SACM1L | hsa-miR-21-5p | up.mi | down.m |
| SACM1L | hsa-miR-324-3p | up.mi | down.m |
| SACM1L | hsa-miR-141-5p | up.mi | down.m |
| SACM1L | hsa-miR-155-5p | up.mi | down.m |
| SAMD4A | hsa-miR-301a-5p | up.mi | down.m |
| SAMD4A | hsa-miR-345-5p | up.mi | down.m |
| SAMD4A | hsa-miR-93-3p | up.mi | down.m |
| SAMD4A | hsa-miR-377-3p | up.mi | down.m |
| SAMD5 | hsa-miR-153-5p | up.mi | down.m |
| SAMD5 | hsa-miR-21-5p | up.mi | down.m |
| SAMD5 | hsa-miR-1306-5p | up.mi | down.m |
| SAMHD1 | hsa-miR-215-5p | up.mi | down.m |
| SAMHD1 | hsa-miR-642a-5p | up.mi | down.m |
| SAMHD1 | hsa-miR-130b-5p | up.mi | down.m |
| SAMHD1 | hsa-miR-155-5p | up.mi | down.m |
| SAMHD1 | hsa-miR-192-5p | up.mi | down.m |
| SAP30L | hsa-miR-155-5p | up.mi | down.m |
| SASH1 | hsa-miR-21-5p | up.mi | down.m |
| SASH1 | hsa-miR-92b-3p | up.mi | down.m |
| SASH1 | hsa-miR-93-5p | up.mi | down.m |
| SASH1 | hsa-miR-92a-3p | up.mi | down.m |
| SASH1 | hsa-miR-186-5p | up.mi | down.m |
| SASH1 | hsa-miR-766-3p | up.mi | down.m |
| SATB1 | hsa-miR-450b-5p | up.mi | down.m |
| SATB1 | hsa-miR-153-3p | up.mi | down.m |
| SATB1 | hsa-miR-655-3p | up.mi | down.m |
| SATB1 | hsa-miR-19b-3p | up.mi | down.m |
| SATB1 | hsa-miR-191-5p | up.mi | down.m |
| SATB1 | hsa-miR-19a-3p | up.mi | down.m |
| SATB1 | hsa-miR-21-5p | up.mi | down.m |
| SBDS | hsa-miR-590-3p | up.mi | down.m |
| SCAI | hsa-miR-625-5p | up.mi | down.m |
| SCAI | hsa-miR-455-3p | up.mi | down.m |
| SCARA3 | hsa-miR-615-3p | up.mi | down.m |
| SCARF1 | hsa-miR-455-3p | up.mi | down.m |
| SCARF1 | hsa-miR-940 | up.mi | down.m |
| SCARF1 | hsa-miR-766-3p | up.mi | down.m |
| SCD | hsa-miR-155-5p | up.mi | down.m |
| SCD | hsa-miR-331-3p | up.mi | down.m |
| SCD | hsa-miR-142-3p | up.mi | down.m |
| SCD | hsa-miR-130b-3p | up.mi | down.m |
| SCD | hsa-miR-3136-5p | up.mi | down.m |
| SCD | hsa-miR-4668-3p | up.mi | down.m |
| SCD | hsa-miR-548v | up.mi | down.m |
| SCD | hsa-miR-105-5p | up.mi | down.m |
| SCD | hsa-miR-106a-5p | up.mi | down.m |
| SCD | hsa-miR-181b-5p | up.mi | down.m |
| SCD | hsa-miR-19a-3p | up.mi | down.m |
| SCD | hsa-miR-493-5p | up.mi | down.m |
| SCD | hsa-miR-424-3p | up.mi | down.m |
| SCD | hsa-miR-181d-5p | up.mi | down.m |
| SCD | hsa-miR-625-3p | up.mi | down.m |
| SCD | hsa-miR-20b-5p | up.mi | down.m |
| SCD | hsa-miR-92a-3p | up.mi | down.m |
| SCD | hsa-miR-188-3p | up.mi | down.m |
| SCD | hsa-miR-106b-5p | up.mi | down.m |
| SCD | hsa-miR-455-3p | up.mi | down.m |
| SCD | hsa-miR-142-5p | up.mi | down.m |
| SCD | hsa-miR-215-5p | up.mi | down.m |
| SCD | hsa-miR-17-5p | up.mi | down.m |
| SCD | hsa-miR-19b-3p | up.mi | down.m |
| SCD | hsa-miR-22-3p | up.mi | down.m |
| SCD | hsa-miR-214-5p | up.mi | down.m |
| SCD | hsa-miR-185-5p | up.mi | down.m |
| SCD | hsa-miR-143-5p | up.mi | down.m |
| SCD | hsa-miR-192-5p | up.mi | down.m |
| SCD | hsa-miR-93-5p | up.mi | down.m |
| SCD | hsa-miR-1269b | up.mi | down.m |
| SCD | hsa-miR-505-3p | up.mi | down.m |
| SCD | hsa-miR-20a-5p | up.mi | down.m |
| SCD | hsa-miR-149-5p | up.mi | down.m |
| SCD | hsa-miR-324-5p | up.mi | down.m |
| SCD | hsa-miR-1269a | up.mi | down.m |
| SCD5 | hsa-miR-92a-3p | up.mi | down.m |
| SCD5 | hsa-miR-9-5p | up.mi | down.m |
| SCD5 | hsa-miR-141-3p | up.mi | down.m |
| SCD5 | hsa-miR-455-3p | up.mi | down.m |
| SCD5 | hsa-miR-200a-3p | up.mi | down.m |
| SCD5 | hsa-miR-324-5p | up.mi | down.m |
| SCD5 | hsa-miR-3913-5p | up.mi | down.m |
| SCIMP | hsa-miR-340-3p | up.mi | down.m |
| SCIMP | hsa-miR-495-3p | up.mi | down.m |
| SCN1A | hsa-miR-576-5p | up.mi | down.m |
| SCN1A | hsa-miR-4668-3p | up.mi | down.m |
| SCN1A | hsa-miR-93-3p | up.mi | down.m |
| SCN4B | hsa-miR-582-3p | up.mi | down.m |
| SCN7A | hsa-miR-210-5p | up.mi | down.m |
| SCN7A | hsa-miR-93-3p | up.mi | down.m |
| SCN7A | hsa-miR-370-3p | up.mi | down.m |
| SCUBE1 | hsa-miR-671-5p | up.mi | down.m |
| SCUBE1 | hsa-miR-3189-3p | up.mi | down.m |
| SCUBE1 | hsa-miR-181b-3p | up.mi | down.m |
| SDC2 | hsa-miR-495-3p | up.mi | down.m |
| SDC2 | hsa-miR-7-1-3p | up.mi | down.m |
| SDC2 | hsa-miR-577 | up.mi | down.m |
| SDC4 | hsa-miR-31-5p | up.mi | down.m |
| SDC4 | hsa-miR-18a-5p | up.mi | down.m |
| SDCBP | hsa-miR-155-5p | up.mi | down.m |
| SDCBP | hsa-miR-629-5p | up.mi | down.m |
| SEC14L1 | hsa-miR-708-5p | up.mi | down.m |
| SEC14L1 | hsa-miR-92a-3p | up.mi | down.m |
| SEC14L1 | hsa-let-7c-3p | up.mi | down.m |
| SEC14L3 | hsa-miR-203b-3p | up.mi | down.m |
| SEC14L4 | hsa-miR-505-5p | up.mi | down.m |
| SEC14L4 | hsa-miR-153-5p | up.mi | down.m |
| SEC14L4 | hsa-miR-192-3p | up.mi | down.m |
| SEC14L4 | hsa-miR-370-3p | up.mi | down.m |
| SEC14L4 | hsa-miR-767-3p | up.mi | down.m |
| SEC14L4 | hsa-miR-5698 | up.mi | down.m |
| SEC14L4 | hsa-miR-744-3p | up.mi | down.m |
| SEC14L4 | hsa-miR-155-3p | up.mi | down.m |
| SEC14L4 | hsa-miR-136-5p | up.mi | down.m |
| SEC14L4 | hsa-miR-93-3p | up.mi | down.m |
| SEC14L4 | hsa-miR-628-5p | up.mi | down.m |
| SEC14L4 | hsa-miR-143-5p | up.mi | down.m |
| SEC14L4 | hsa-miR-432-5p | up.mi | down.m |
| SEC14L6 | hsa-miR-183-5p | up.mi | down.m |
| SEC14L6 | hsa-miR-767-5p | up.mi | down.m |
| SEC22C | hsa-miR-660-5p | up.mi | down.m |
| SEC22C | hsa-miR-7705 | up.mi | down.m |
| SEC22C | hsa-miR-6510-3p | up.mi | down.m |
| SEC22C | hsa-miR-143-5p | up.mi | down.m |
| SEC22C | hsa-miR-616-5p | up.mi | down.m |
| SECISBP2L | hsa-miR-200c-3p | up.mi | down.m |
| SECISBP2L | hsa-miR-130b-3p | up.mi | down.m |
| SECISBP2L | hsa-miR-148b-3p | up.mi | down.m |
| SECISBP2L | hsa-miR-409-3p | up.mi | down.m |
| SECISBP2L | hsa-miR-429 | up.mi | down.m |
| SECISBP2L | hsa-miR-130a-3p | up.mi | down.m |
| SECISBP2L | hsa-miR-148a-3p | up.mi | down.m |
| SECISBP2L | hsa-miR-21-5p | up.mi | down.m |
| SECISBP2L | hsa-miR-454-3p | up.mi | down.m |
| SECISBP2L | hsa-miR-19b-3p | up.mi | down.m |
| SECISBP2L | hsa-miR-19a-3p | up.mi | down.m |
| SECISBP2L | hsa-miR-301a-3p | up.mi | down.m |
| SECISBP2L | hsa-miR-200b-3p | up.mi | down.m |
| SECISBP2L | hsa-miR-301b-3p | up.mi | down.m |
| SECISBP2L | hsa-miR-33b-5p | up.mi | down.m |
| SECISBP2L | hsa-miR-33a-5p | up.mi | down.m |
| SECTM1 | hsa-miR-615-3p | up.mi | down.m |
| SELE | hsa-miR-155-5p | up.mi | down.m |
| SELE | hsa-miR-31-5p | up.mi | down.m |
| SELE | hsa-miR-17-3p | up.mi | down.m |
| SELE | hsa-miR-17-5p | up.mi | down.m |
| SELENBP1 | hsa-miR-20a-5p | up.mi | down.m |
| SELENBP1 | hsa-miR-92a-3p | up.mi | down.m |
| SEMA3D | hsa-miR-193a-3p | up.mi | down.m |
| SEMA3D | hsa-miR-628-5p | up.mi | down.m |
| SEMA3D | hsa-miR-590-3p | up.mi | down.m |
| SEMA3E | hsa-miR-642a-5p | up.mi | down.m |
| SEMA3E | hsa-miR-1307-3p | up.mi | down.m |
| SEMA3E | hsa-miR-664a-3p | up.mi | down.m |
| SEMA3E | hsa-miR-153-5p | up.mi | down.m |
| SEMA3E | hsa-miR-615-3p | up.mi | down.m |
| SEMA3E | hsa-miR-17-3p | up.mi | down.m |
| SEMA5A | hsa-miR-21-5p | up.mi | down.m |
| SEMA6A | hsa-miR-1301-3p | up.mi | down.m |
| SEMA6A | hsa-miR-30e-5p | up.mi | down.m |
| SEMA6A | hsa-miR-450b-5p | up.mi | down.m |
| SEMA6D | hsa-miR-130a-5p | up.mi | down.m |
| SEMA6D | hsa-miR-589-3p | up.mi | down.m |
| SERINC1 | hsa-miR-136-5p | up.mi | down.m |
| SERINC1 | hsa-miR-93-5p | up.mi | down.m |
| SERINC1 | hsa-miR-149-5p | up.mi | down.m |
| SERINC1 | hsa-miR-106a-5p | up.mi | down.m |
| SERINC1 | hsa-miR-106b-5p | up.mi | down.m |
| SERINC1 | hsa-miR-92a-3p | up.mi | down.m |
| SERINC1 | hsa-miR-20a-5p | up.mi | down.m |
| SERINC1 | hsa-miR-17-5p | up.mi | down.m |
| SERINC1 | hsa-miR-20b-5p | up.mi | down.m |
| SERPING1 | hsa-miR-629-3p | up.mi | down.m |
| SERPING1 | hsa-miR-128-3p | up.mi | down.m |
| SERPING1 | hsa-miR-766-3p | up.mi | down.m |
| SERTAD1 | hsa-miR-940 | up.mi | down.m |
| SERTAD1 | hsa-miR-3913-5p | up.mi | down.m |
| SERTM1 | hsa-miR-210-3p | up.mi | down.m |
| SESN1 | hsa-miR-200c-3p | up.mi | down.m |
| SESN1 | hsa-miR-200b-3p | up.mi | down.m |
| SESN1 | hsa-miR-93-5p | up.mi | down.m |
| SESN1 | hsa-miR-21-5p | up.mi | down.m |
| SESN1 | hsa-miR-940 | up.mi | down.m |
| SESN1 | hsa-miR-377-3p | up.mi | down.m |
| SESN1 | hsa-miR-20b-5p | up.mi | down.m |
| SESN1 | hsa-miR-20a-5p | up.mi | down.m |
| SESN1 | hsa-miR-429 | up.mi | down.m |
| SESN1 | hsa-miR-501-3p | up.mi | down.m |
| SESN1 | hsa-miR-17-5p | up.mi | down.m |
| SESN1 | hsa-miR-154-5p | up.mi | down.m |
| SESN1 | hsa-miR-4652-5p | up.mi | down.m |
| SESN1 | hsa-miR-106b-5p | up.mi | down.m |
| SESN1 | hsa-miR-106a-5p | up.mi | down.m |
| SESTD1 | hsa-miR-15a-5p | up.mi | down.m |
| SESTD1 | hsa-miR-503-5p | up.mi | down.m |
| SESTD1 | hsa-miR-19b-3p | up.mi | down.m |
| SESTD1 | hsa-miR-130b-3p | up.mi | down.m |
| SESTD1 | hsa-miR-148b-3p | up.mi | down.m |
| SESTD1 | hsa-miR-424-5p | up.mi | down.m |
| SESTD1 | hsa-miR-4668-3p | up.mi | down.m |
| SESTD1 | hsa-miR-21-5p | up.mi | down.m |
| SESTD1 | hsa-miR-148a-3p | up.mi | down.m |
| SETBP1 | hsa-miR-4668-3p | up.mi | down.m |
| SETBP1 | hsa-miR-432-5p | up.mi | down.m |
| SFRP1 | hsa-miR-582-3p | up.mi | down.m |
| SFTPA1 | hsa-miR-19b-3p | up.mi | down.m |
| SFTPA1 | hsa-miR-130a-3p | up.mi | down.m |
| SFTPA1 | hsa-miR-19a-3p | up.mi | down.m |
| SFTPA1 | hsa-miR-130b-3p | up.mi | down.m |
| SFTPA1 | hsa-miR-301b-3p | up.mi | down.m |
| SFTPA1 | hsa-miR-301a-3p | up.mi | down.m |
| SFTPA1 | hsa-miR-454-3p | up.mi | down.m |
| SGCB | hsa-miR-192-5p | up.mi | down.m |
| SGCB | hsa-miR-21-5p | up.mi | down.m |
| SGCB | hsa-miR-590-3p | up.mi | down.m |
| SGCB | hsa-miR-215-5p | up.mi | down.m |
| SGK1 | hsa-miR-19a-3p | up.mi | down.m |
| SGK1 | hsa-miR-29b-3p | up.mi | down.m |
| SGK1 | hsa-miR-21-3p | up.mi | down.m |
| SGK1 | hsa-miR-19b-3p | up.mi | down.m |
| SGMS1 | hsa-miR-381-3p | up.mi | down.m |
| SGMS1 | hsa-miR-20b-5p | up.mi | down.m |
| SGMS1 | hsa-miR-106a-5p | up.mi | down.m |
| SGMS1 | hsa-miR-93-5p | up.mi | down.m |
| SGMS1 | hsa-miR-130b-3p | up.mi | down.m |
| SGMS1 | hsa-miR-142-3p | up.mi | down.m |
| SGMS1 | hsa-miR-33a-5p | up.mi | down.m |
| SGMS1 | hsa-miR-20a-5p | up.mi | down.m |
| SGMS1 | hsa-miR-17-5p | up.mi | down.m |
| SGMS1 | hsa-miR-106b-5p | up.mi | down.m |
| SGMS2 | hsa-miR-192-5p | up.mi | down.m |
| SGMS2 | hsa-miR-615-3p | up.mi | down.m |
| SH2B3 | hsa-miR-92a-3p | up.mi | down.m |
| SH2B3 | hsa-miR-767-5p | up.mi | down.m |
| SH2B3 | hsa-miR-766-3p | up.mi | down.m |
| SH2B3 | hsa-miR-92b-3p | up.mi | down.m |
| SH2B3 | hsa-miR-452-3p | up.mi | down.m |
| SH2B3 | hsa-miR-215-5p | up.mi | down.m |
| SH2B3 | hsa-miR-192-5p | up.mi | down.m |
| SH3BGRL | hsa-miR-210-3p | up.mi | down.m |
| SH3BGRL | hsa-miR-128-3p | up.mi | down.m |
| SH3BP5 | hsa-miR-16-2-3p | up.mi | down.m |
| SH3BP5 | hsa-miR-17-5p | up.mi | down.m |
| SH3BP5 | hsa-miR-323b-3p | up.mi | down.m |
| SH3BP5 | hsa-miR-106a-5p | up.mi | down.m |
| SH3BP5 | hsa-miR-193b-3p | up.mi | down.m |
| SH3BP5 | hsa-miR-106b-5p | up.mi | down.m |
| SH3BP5 | hsa-miR-20b-5p | up.mi | down.m |
| SH3BP5 | hsa-miR-130b-3p | up.mi | down.m |
| SH3BP5 | hsa-miR-93-5p | up.mi | down.m |
| SH3BP5 | hsa-miR-20a-5p | up.mi | down.m |
| SH3D19 | hsa-miR-128-3p | up.mi | down.m |
| SH3D19 | hsa-miR-766-3p | up.mi | down.m |
| SH3D19 | hsa-miR-616-5p | up.mi | down.m |
| SH3D19 | hsa-miR-183-5p | up.mi | down.m |
| SH3GL3 | hsa-miR-196a-5p | up.mi | down.m |
| SHC3 | hsa-miR-376b-3p | up.mi | down.m |
| SHC3 | hsa-miR-92a-3p | up.mi | down.m |
| SHC3 | hsa-miR-2355-3p | up.mi | down.m |
| SHC3 | hsa-miR-155-3p | up.mi | down.m |
| SHE | hsa-miR-92b-3p | up.mi | down.m |
| SHE | hsa-miR-616-5p | up.mi | down.m |
| SHE | hsa-miR-769-3p | up.mi | down.m |
| SHE | hsa-miR-766-3p | up.mi | down.m |
| SHE | hsa-miR-92a-3p | up.mi | down.m |
| SHISA2 | hsa-miR-106b-5p | up.mi | down.m |
| SHISA2 | hsa-miR-183-5p | up.mi | down.m |
| SHISA2 | hsa-miR-3913-5p | up.mi | down.m |
| SHROOM4 | hsa-miR-4661-5p | up.mi | down.m |
| SHROOM4 | hsa-miR-1306-5p | up.mi | down.m |
| SIDT2 | hsa-miR-424-5p | up.mi | down.m |
| SIDT2 | hsa-miR-15a-5p | up.mi | down.m |
| SIGLEC14 | hsa-miR-186-5p | up.mi | down.m |
| SIGLEC14 | hsa-miR-455-3p | up.mi | down.m |
| SIGLEC14 | hsa-let-7c-3p | up.mi | down.m |
| SIGLEC14 | hsa-miR-425-5p | up.mi | down.m |
| SIGLEC14 | hsa-miR-495-3p | up.mi | down.m |
| SIGLEC14 | hsa-miR-590-3p | up.mi | down.m |
| SIGLEC6 | hsa-miR-5698 | up.mi | down.m |
| SIGLEC6 | hsa-miR-744-3p | up.mi | down.m |
| SIGLEC6 | hsa-miR-143-5p | up.mi | down.m |
| SIGLEC9 | hsa-miR-130b-3p | up.mi | down.m |
| SIGLEC9 | hsa-miR-455-3p | up.mi | down.m |
| SIGLEC9 | hsa-miR-130a-3p | up.mi | down.m |
| SIGLEC9 | hsa-miR-142-5p | up.mi | down.m |
| SIGLEC9 | hsa-miR-766-3p | up.mi | down.m |
| SIGLEC9 | hsa-miR-183-5p | up.mi | down.m |
| SIGLEC9 | hsa-miR-301b-3p | up.mi | down.m |
| SIGLEC9 | hsa-miR-454-3p | up.mi | down.m |
| SIGLEC9 | hsa-miR-301a-3p | up.mi | down.m |
| SIGLEC9 | hsa-miR-186-5p | up.mi | down.m |
| SIK2 | hsa-miR-5698 | up.mi | down.m |
| SIK2 | hsa-miR-143-5p | up.mi | down.m |
| SIK2 | hsa-miR-142-3p | up.mi | down.m |
| SIRPA | hsa-miR-106a-5p | up.mi | down.m |
| SIRPA | hsa-miR-615-3p | up.mi | down.m |
| SIRPA | hsa-miR-1287-3p | up.mi | down.m |
| SIRPA | hsa-miR-17-5p | up.mi | down.m |
| SIRPA | hsa-miR-141-5p | up.mi | down.m |
| SIRPA | hsa-miR-20a-5p | up.mi | down.m |
| SIRPA | hsa-miR-2355-5p | up.mi | down.m |
| SIRPB2 | hsa-miR-19b-3p | up.mi | down.m |
| SIRPB2 | hsa-miR-616-5p | up.mi | down.m |
| SKI | hsa-miR-15a-5p | up.mi | down.m |
| SKI | hsa-miR-149-5p | up.mi | down.m |
| SKI | hsa-miR-369-3p | up.mi | down.m |
| SKI | hsa-miR-455-3p | up.mi | down.m |
| SKI | hsa-miR-203b-3p | up.mi | down.m |
| SKI | hsa-miR-449a | up.mi | down.m |
| SKI | hsa-miR-181b-3p | up.mi | down.m |
| SKI | hsa-miR-503-5p | up.mi | down.m |
| SKI | hsa-miR-92a-3p | up.mi | down.m |
| SKI | hsa-miR-106b-5p | up.mi | down.m |
| SKI | hsa-miR-17-5p | up.mi | down.m |
| SKI | hsa-miR-93-5p | up.mi | down.m |
| SKI | hsa-miR-92b-3p | up.mi | down.m |
| SKI | hsa-miR-450b-5p | up.mi | down.m |
| SKI | hsa-miR-424-5p | up.mi | down.m |
| SKI | hsa-miR-155-5p | up.mi | down.m |
| SKI | hsa-miR-655-3p | up.mi | down.m |
| SKI | hsa-miR-20a-5p | up.mi | down.m |
| SKI | hsa-miR-616-5p | up.mi | down.m |
| SKI | hsa-miR-3136-5p | up.mi | down.m |
| SKI | hsa-miR-34a-5p | up.mi | down.m |
| SKI | hsa-miR-20b-5p | up.mi | down.m |
| SKI | hsa-miR-196a-5p | up.mi | down.m |
| SKI | hsa-miR-590-3p | up.mi | down.m |
| SLC11A1 | hsa-miR-339-5p | up.mi | down.m |
| SLC11A1 | hsa-miR-182-5p | up.mi | down.m |
| SLC12A4 | hsa-miR-155-5p | up.mi | down.m |
| SLC12A4 | hsa-miR-92a-3p | up.mi | down.m |
| SLC16A5 | hsa-miR-766-3p | up.mi | down.m |
| SLC16A5 | hsa-miR-5698 | up.mi | down.m |
| SLC16A5 | hsa-miR-143-5p | up.mi | down.m |
| SLC16A6 | hsa-miR-215-5p | up.mi | down.m |
| SLC16A6 | hsa-miR-192-5p | up.mi | down.m |
| SLC16A6 | hsa-miR-4724-5p | up.mi | down.m |
| SLC19A1 | hsa-miR-93-5p | up.mi | down.m |
| SLC19A3 | hsa-miR-203b-3p | up.mi | down.m |
| SLC19A3 | hsa-miR-9-5p | up.mi | down.m |
| SLC19A3 | hsa-miR-3913-5p | up.mi | down.m |
| SLC19A3 | hsa-miR-548v | up.mi | down.m |
| SLC19A3 | hsa-miR-135b-5p | up.mi | down.m |
| SLC19A3 | hsa-miR-616-5p | up.mi | down.m |
| SLC1A1 | hsa-miR-629-3p | up.mi | down.m |
| SLC1A1 | hsa-miR-96-5p | up.mi | down.m |
| SLC1A1 | hsa-miR-186-5p | up.mi | down.m |
| SLC1A1 | hsa-miR-758-3p | up.mi | down.m |
| SLC22A3 | hsa-miR-452-3p | up.mi | down.m |
| SLC22A3 | hsa-miR-130b-5p | up.mi | down.m |
| SLC22A3 | hsa-miR-9-5p | up.mi | down.m |
| SLC22A3 | hsa-miR-148b-3p | up.mi | down.m |
| SLC24A3 | hsa-let-7c-3p | up.mi | down.m |
| SLC25A25 | hsa-miR-501-5p | up.mi | down.m |
| SLC25A25 | hsa-miR-181d-5p | up.mi | down.m |
| SLC25A25 | hsa-miR-205-5p | up.mi | down.m |
| SLC25A25 | hsa-miR-183-5p | up.mi | down.m |
| SLC25A25 | hsa-miR-181b-5p | up.mi | down.m |
| SLC25A25 | hsa-miR-96-5p | up.mi | down.m |
| SLC26A2 | hsa-miR-193b-5p | up.mi | down.m |
| SLC26A2 | hsa-miR-21-5p | up.mi | down.m |
| SLC26A2 | hsa-miR-215-5p | up.mi | down.m |
| SLC26A2 | hsa-miR-192-5p | up.mi | down.m |
| SLC26A2 | hsa-miR-199b-5p | up.mi | down.m |
| SLC26A2 | hsa-miR-128-3p | up.mi | down.m |
| SLC26A2 | hsa-miR-199a-5p | up.mi | down.m |
| SLC26A2 | hsa-miR-324-5p | up.mi | down.m |
| SLC26A9 | hsa-miR-185-5p | up.mi | down.m |
| SLC26A9 | hsa-miR-5698 | up.mi | down.m |
| SLC2A3 | hsa-miR-539-5p | up.mi | down.m |
| SLC2A3 | hsa-miR-15a-5p | up.mi | down.m |
| SLC2A3 | hsa-miR-148a-3p | up.mi | down.m |
| SLC2A3 | hsa-miR-34a-5p | up.mi | down.m |
| SLC2A3 | hsa-miR-106a-5p | up.mi | down.m |
| SLC2A3 | hsa-miR-503-5p | up.mi | down.m |
| SLC2A3 | hsa-miR-183-5p | up.mi | down.m |
| SLC2A3 | hsa-miR-424-5p | up.mi | down.m |
| SLC35A1 | hsa-miR-15a-5p | up.mi | down.m |
| SLC35A1 | hsa-miR-155-5p | up.mi | down.m |
| SLC35A1 | hsa-miR-224-3p | up.mi | down.m |
| SLC39A8 | hsa-miR-215-5p | up.mi | down.m |
| SLC39A8 | hsa-miR-192-5p | up.mi | down.m |
| SLC39A8 | hsa-miR-331-3p | up.mi | down.m |
| SLC47A1 | hsa-miR-5698 | up.mi | down.m |
| SLC47A1 | hsa-miR-625-5p | up.mi | down.m |
| SLC47A1 | hsa-miR-1307-3p | up.mi | down.m |
| SLC47A1 | hsa-miR-3189-3p | up.mi | down.m |
| SLC6A4 | hsa-miR-141-5p | up.mi | down.m |
| SLC6A4 | hsa-miR-93-5p | up.mi | down.m |
| SLC6A4 | hsa-miR-17-5p | up.mi | down.m |
| SLC6A4 | hsa-miR-20b-5p | up.mi | down.m |
| SLC6A4 | hsa-miR-106b-5p | up.mi | down.m |
| SLC6A4 | hsa-miR-142-5p | up.mi | down.m |
| SLC6A4 | hsa-miR-20a-5p | up.mi | down.m |
| SLC6A4 | hsa-miR-17-3p | up.mi | down.m |
| SLC6A4 | hsa-miR-106a-5p | up.mi | down.m |
| SLC7A7 | hsa-miR-196a-5p | up.mi | down.m |
| SLC7A8 | hsa-miR-185-5p | up.mi | down.m |
| SLC9A3R2 | hsa-miR-5698 | up.mi | down.m |
| SLC9A3R2 | hsa-miR-155-5p | up.mi | down.m |
| SLC9A3R2 | hsa-miR-345-5p | up.mi | down.m |
| SLC9A3R2 | hsa-miR-301a-5p | up.mi | down.m |
| SLCO3A1 | hsa-miR-424-5p | up.mi | down.m |
| SLCO3A1 | hsa-miR-149-5p | up.mi | down.m |
| SLCO3A1 | hsa-miR-15a-5p | up.mi | down.m |
| SLCO3A1 | hsa-miR-210-3p | up.mi | down.m |
| SLCO3A1 | hsa-miR-616-5p | up.mi | down.m |
| SLCO3A1 | hsa-miR-192-5p | up.mi | down.m |
| SLCO3A1 | hsa-miR-181b-3p | up.mi | down.m |
| SLCO3A1 | hsa-miR-215-5p | up.mi | down.m |
| SLCO4C1 | hsa-miR-615-3p | up.mi | down.m |
| SLCO4C1 | hsa-miR-148b-3p | up.mi | down.m |
| SLFN11 | hsa-miR-577 | up.mi | down.m |
| SLPI | hsa-miR-151a-5p | up.mi | down.m |
| SMAD6 | hsa-miR-134-5p | up.mi | down.m |
| SMAD6 | hsa-miR-17-5p | up.mi | down.m |
| SMAD6 | hsa-miR-93-5p | up.mi | down.m |
| SMAD6 | hsa-miR-20a-5p | up.mi | down.m |
| SMAD6 | hsa-miR-92b-3p | up.mi | down.m |
| SMAD6 | hsa-miR-20b-5p | up.mi | down.m |
| SMAD6 | hsa-miR-106b-5p | up.mi | down.m |
| SMAD6 | hsa-miR-92a-3p | up.mi | down.m |
| SMAD7 | hsa-miR-21-5p | up.mi | down.m |
| SMAD7 | hsa-miR-15a-5p | up.mi | down.m |
| SMAD7 | hsa-miR-503-5p | up.mi | down.m |
| SMAD7 | hsa-miR-424-5p | up.mi | down.m |
| SMAD7 | hsa-miR-590-5p | up.mi | down.m |
| SMAD7 | hsa-miR-92a-3p | up.mi | down.m |
| SMAD7 | hsa-miR-106b-5p | up.mi | down.m |
| SMAD7 | hsa-miR-92b-3p | up.mi | down.m |
| SMAD7 | hsa-miR-20a-5p | up.mi | down.m |
| SMAD7 | hsa-miR-185-5p | up.mi | down.m |
| SMAD7 | hsa-miR-93-5p | up.mi | down.m |
| SMAD9 | hsa-miR-940 | up.mi | down.m |
| SMAD9 | hsa-miR-106b-5p | up.mi | down.m |
| SMAD9 | hsa-miR-653-5p | up.mi | down.m |
| SMARCA2 | hsa-miR-19b-3p | up.mi | down.m |
| SMARCA2 | hsa-miR-615-3p | up.mi | down.m |
| SMARCA2 | hsa-miR-199a-5p | up.mi | down.m |
| SMARCA2 | hsa-miR-199a-3p | up.mi | down.m |
| SMARCA2 | hsa-miR-192-5p | up.mi | down.m |
| SMARCA2 | hsa-miR-215-5p | up.mi | down.m |
| SMARCA2 | hsa-miR-423-3p | up.mi | down.m |
| SMARCA2 | hsa-miR-19a-3p | up.mi | down.m |
| SMARCA2 | hsa-miR-186-5p | up.mi | down.m |
| SMARCA5 | hsa-let-7a-2-3p | up.mi | down.m |
| SMARCA5 | hsa-miR-542-3p | up.mi | down.m |
| SMARCA5 | hsa-miR-424-5p | up.mi | down.m |
| SMARCA5 | hsa-miR-92b-3p | up.mi | down.m |
| SMARCA5 | hsa-let-7g-3p | up.mi | down.m |
| SMARCA5 | hsa-miR-493-5p | up.mi | down.m |
| SMARCA5 | hsa-miR-186-5p | up.mi | down.m |
| SMARCA5 | hsa-miR-767-5p | up.mi | down.m |
| SMARCA5 | hsa-let-7c-3p | up.mi | down.m |
| SMARCA5 | hsa-miR-92a-3p | up.mi | down.m |
| SMARCA5 | hsa-miR-766-3p | up.mi | down.m |
| SMARCA5 | hsa-miR-151a-5p | up.mi | down.m |
| SMIM10 | hsa-miR-431-5p | up.mi | down.m |
| SMIM10 | hsa-miR-153-5p | up.mi | down.m |
| SMTNL2 | hsa-miR-744-3p | up.mi | down.m |
| SMTNL2 | hsa-miR-301a-3p | up.mi | down.m |
| SMTNL2 | hsa-miR-3913-5p | up.mi | down.m |
| SMTNL2 | hsa-miR-6510-3p | up.mi | down.m |
| SMTNL2 | hsa-miR-130b-3p | up.mi | down.m |
| SMTNL2 | hsa-miR-454-3p | up.mi | down.m |
| SMTNL2 | hsa-miR-301b-3p | up.mi | down.m |
| SMTNL2 | hsa-miR-940 | up.mi | down.m |
| SMTNL2 | hsa-miR-766-3p | up.mi | down.m |
| SMTNL2 | hsa-miR-130a-3p | up.mi | down.m |
| SNCA | hsa-miR-153-3p | up.mi | down.m |
| SNRK | hsa-miR-193b-3p | up.mi | down.m |
| SNRK | hsa-miR-192-3p | up.mi | down.m |
| SNRK | hsa-miR-21-5p | up.mi | down.m |
| SNRK | hsa-miR-153-5p | up.mi | down.m |
| SNRK | hsa-miR-154-5p | up.mi | down.m |
| SNRK | hsa-miR-128-3p | up.mi | down.m |
| SNTN | hsa-miR-940 | up.mi | down.m |
| SNTN | hsa-miR-758-3p | up.mi | down.m |
| SNTN | hsa-miR-3677-5p | up.mi | down.m |
| SNTN | hsa-miR-142-3p | up.mi | down.m |
| SNX1 | hsa-miR-28-5p | up.mi | down.m |
| SNX1 | hsa-miR-28-3p | up.mi | down.m |
| SNX1 | hsa-miR-3913-5p | up.mi | down.m |
| SNX10 | hsa-miR-192-5p | up.mi | down.m |
| SNX10 | hsa-miR-215-5p | up.mi | down.m |
| SNX10 | hsa-miR-92b-3p | up.mi | down.m |
| SNX10 | hsa-miR-331-5p | up.mi | down.m |
| SNX10 | hsa-miR-127-5p | up.mi | down.m |
| SNX10 | hsa-miR-27b-5p | up.mi | down.m |
| SNX10 | hsa-miR-92a-3p | up.mi | down.m |
| SNX2 | hsa-miR-19b-1-5p | up.mi | down.m |
| SNX2 | hsa-miR-203b-3p | up.mi | down.m |
| SNX2 | hsa-miR-744-3p | up.mi | down.m |
| SNX22 | hsa-miR-143-5p | up.mi | down.m |
| SNX22 | hsa-miR-940 | up.mi | down.m |
| SNX22 | hsa-miR-1307-3p | up.mi | down.m |
| SNX22 | hsa-miR-5698 | up.mi | down.m |
| SNX22 | hsa-miR-744-3p | up.mi | down.m |
| SNX30 | hsa-miR-21-5p | up.mi | down.m |
| SOBP | hsa-miR-324-5p | up.mi | down.m |
| SOBP | hsa-miR-432-5p | up.mi | down.m |
| SOBP | hsa-miR-30e-5p | up.mi | down.m |
| SOBP | hsa-miR-487b-3p | up.mi | down.m |
| SOBP | hsa-miR-15b-3p | up.mi | down.m |
| SOCS2 | hsa-miR-424-5p | up.mi | down.m |
| SOCS2 | hsa-miR-194-5p | up.mi | down.m |
| SOCS3 | hsa-miR-19a-3p | up.mi | down.m |
| SOCS3 | hsa-miR-455-5p | up.mi | down.m |
| SOCS3 | hsa-miR-155-5p | up.mi | down.m |
| SOCS3 | hsa-miR-4724-5p | up.mi | down.m |
| SOCS3 | hsa-miR-30e-5p | up.mi | down.m |
| SOCS3 | hsa-miR-335-3p | up.mi | down.m |
| SOCS3 | hsa-miR-19b-3p | up.mi | down.m |
| SOD3 | hsa-miR-21-5p | up.mi | down.m |
| SORT1 | hsa-miR-34a-5p | up.mi | down.m |
| SORT1 | hsa-miR-181b-5p | up.mi | down.m |
| SORT1 | hsa-miR-181d-5p | up.mi | down.m |
| SOX17 | hsa-miR-134-5p | up.mi | down.m |
| SOX17 | hsa-miR-758-3p | up.mi | down.m |
| SOX17 | hsa-miR-1287-5p | up.mi | down.m |
| SOX17 | hsa-miR-151a-5p | up.mi | down.m |
| SOX17 | hsa-miR-200a-5p | up.mi | down.m |
| SOX17 | hsa-miR-200b-5p | up.mi | down.m |
| SOX17 | hsa-miR-671-5p | up.mi | down.m |
| SOX5 | hsa-miR-15a-5p | up.mi | down.m |
| SOX5 | hsa-miR-21-5p | up.mi | down.m |
| SOX5 | hsa-miR-96-5p | up.mi | down.m |
| SOX5 | hsa-miR-194-5p | up.mi | down.m |
| SOX5 | hsa-miR-136-3p | up.mi | down.m |
| SOX5 | hsa-miR-130a-3p | up.mi | down.m |
| SOX7 | hsa-miR-1306-5p | up.mi | down.m |
| SOX7 | hsa-miR-664a-3p | up.mi | down.m |
| SOX7 | hsa-miR-9-5p | up.mi | down.m |
| SPEF1 | hsa-miR-151a-5p | up.mi | down.m |
| SPI1 | hsa-miR-155-5p | up.mi | down.m |
| SPI1 | hsa-miR-424-5p | up.mi | down.m |
| SPI1 | hsa-miR-9-5p | up.mi | down.m |
| SPI1 | hsa-miR-339-5p | up.mi | down.m |
| SPI1 | hsa-miR-34a-5p | up.mi | down.m |
| SPN | hsa-miR-324-3p | up.mi | down.m |
| SPN | hsa-miR-590-3p | up.mi | down.m |
| SPN | hsa-miR-337-3p | up.mi | down.m |
| SPN | hsa-miR-335-3p | up.mi | down.m |
| SPOCK2 | hsa-miR-92a-3p | up.mi | down.m |
| SPOCK2 | hsa-miR-92b-3p | up.mi | down.m |
| SPRY2 | hsa-miR-21-5p | up.mi | down.m |
| SPRY2 | hsa-miR-148a-3p | up.mi | down.m |
| SPRY2 | hsa-miR-24-2-5p | up.mi | down.m |
| SPRY4 | hsa-miR-215-5p | up.mi | down.m |
| SPRY4 | hsa-miR-769-3p | up.mi | down.m |
| SPRY4 | hsa-miR-590-3p | up.mi | down.m |
| SPRY4 | hsa-miR-31-5p | up.mi | down.m |
| SPRY4 | hsa-miR-625-5p | up.mi | down.m |
| SPRY4 | hsa-miR-21-5p | up.mi | down.m |
| SPRY4 | hsa-miR-2355-5p | up.mi | down.m |
| SPRY4 | hsa-miR-590-5p | up.mi | down.m |
| SPRY4 | hsa-miR-192-5p | up.mi | down.m |
| SPRY4 | hsa-miR-194-5p | up.mi | down.m |
| SPRY4 | hsa-miR-548v | up.mi | down.m |
| SPRY4 | hsa-miR-192-3p | up.mi | down.m |
| SPTAN1 | hsa-miR-34a-5p | up.mi | down.m |
| SPTAN1 | hsa-miR-128-3p | up.mi | down.m |
| SPTAN1 | hsa-miR-331-3p | up.mi | down.m |
| SPTAN1 | hsa-miR-93-3p | up.mi | down.m |
| SPTBN1 | hsa-miR-615-3p | up.mi | down.m |
| SPTBN1 | hsa-miR-34a-5p | up.mi | down.m |
| SPTBN1 | hsa-miR-93-5p | up.mi | down.m |
| SPTBN1 | hsa-miR-331-3p | up.mi | down.m |
| SPTBN1 | hsa-miR-589-3p | up.mi | down.m |
| SPTBN1 | hsa-miR-192-5p | up.mi | down.m |
| SPTBN1 | hsa-miR-9-5p | up.mi | down.m |
| SPTBN1 | hsa-miR-1301-3p | up.mi | down.m |
| SPTBN1 | hsa-miR-423-3p | up.mi | down.m |
| SPTBN1 | hsa-miR-193b-3p | up.mi | down.m |
| SPTBN1 | hsa-miR-215-5p | up.mi | down.m |
| SREBF2 | hsa-miR-92a-3p | up.mi | down.m |
| SREBF2 | hsa-miR-185-5p | up.mi | down.m |
| SREBF2 | hsa-miR-128-3p | up.mi | down.m |
| SREBF2 | hsa-miR-582-5p | up.mi | down.m |
| SREBF2 | hsa-miR-331-3p | up.mi | down.m |
| SREBF2 | hsa-miR-28-5p | up.mi | down.m |
| SRF | hsa-miR-92a-3p | up.mi | down.m |
| SRF | hsa-miR-324-3p | up.mi | down.m |
| SRF | hsa-miR-28-5p | up.mi | down.m |
| SRF | hsa-miR-940 | up.mi | down.m |
| SRF | hsa-miR-200a-3p | up.mi | down.m |
| SRF | hsa-miR-5698 | up.mi | down.m |
| SRF | hsa-miR-708-5p | up.mi | down.m |
| SRF | hsa-miR-9-5p | up.mi | down.m |
| SRF | hsa-miR-331-3p | up.mi | down.m |
| SRGN | hsa-miR-181d-5p | up.mi | down.m |
| SRGN | hsa-miR-181b-5p | up.mi | down.m |
| SSBP2 | hsa-miR-449a | up.mi | down.m |
| SSBP2 | hsa-miR-34a-5p | up.mi | down.m |
| SSBP2 | hsa-miR-940 | up.mi | down.m |
| SSBP2 | hsa-miR-590-3p | up.mi | down.m |
| SSBP2 | hsa-miR-629-3p | up.mi | down.m |
| SSBP2 | hsa-miR-542-3p | up.mi | down.m |
| SSBP2 | hsa-miR-766-3p | up.mi | down.m |
| SSBP2 | hsa-miR-455-3p | up.mi | down.m |
| SSBP2 | hsa-miR-493-3p | up.mi | down.m |
| SSFA2 | hsa-miR-92a-3p | up.mi | down.m |
| SSFA2 | hsa-miR-92b-3p | up.mi | down.m |
| SSFA2 | hsa-miR-128-3p | up.mi | down.m |
| SSFA2 | hsa-miR-15b-3p | up.mi | down.m |
| SSFA2 | hsa-miR-136-5p | up.mi | down.m |
| SSFA2 | hsa-miR-21-5p | up.mi | down.m |
| SSH2 | hsa-miR-106b-5p | up.mi | down.m |
| SSH2 | hsa-miR-20a-5p | up.mi | down.m |
| SSH2 | hsa-miR-766-3p | up.mi | down.m |
| SSH2 | hsa-miR-93-5p | up.mi | down.m |
| SSH2 | hsa-miR-155-5p | up.mi | down.m |
| SSH2 | hsa-miR-17-5p | up.mi | down.m |
| SSH2 | hsa-miR-130b-5p | up.mi | down.m |
| SSH2 | hsa-miR-20b-5p | up.mi | down.m |
| SSTR1 | hsa-miR-501-3p | up.mi | down.m |
| ST3GAL6 | hsa-miR-215-5p | up.mi | down.m |
| ST3GAL6 | hsa-miR-192-5p | up.mi | down.m |
| ST6GALNAC3 | hsa-miR-501-5p | up.mi | down.m |
| ST6GALNAC3 | hsa-miR-4668-3p | up.mi | down.m |
| ST6GALNAC3 | hsa-miR-2355-5p | up.mi | down.m |
| ST6GALNAC5 | hsa-miR-377-3p | up.mi | down.m |
| ST6GALNAC5 | hsa-miR-148b-3p | up.mi | down.m |
| STARD13 | hsa-miR-183-3p | up.mi | down.m |
| STARD13 | hsa-miR-148a-3p | up.mi | down.m |
| STARD13 | hsa-miR-21-3p | up.mi | down.m |
| STARD13 | hsa-miR-301a-3p | up.mi | down.m |
| STARD13 | hsa-miR-9-5p | up.mi | down.m |
| STARD13 | hsa-miR-130a-3p | up.mi | down.m |
| STARD13 | hsa-miR-454-3p | up.mi | down.m |
| STARD13 | hsa-miR-148b-3p | up.mi | down.m |
| STARD13 | hsa-miR-301b-3p | up.mi | down.m |
| STARD13 | hsa-miR-130b-3p | up.mi | down.m |
| STARD4 | hsa-miR-20b-3p | up.mi | down.m |
| STARD8 | hsa-miR-186-5p | up.mi | down.m |
| STARD8 | hsa-miR-3913-5p | up.mi | down.m |
| STARD8 | hsa-let-7a-2-3p | up.mi | down.m |
| STARD8 | hsa-let-7g-3p | up.mi | down.m |
| STAT5A | hsa-miR-141-3p | up.mi | down.m |
| STAT5A | hsa-miR-130b-5p | up.mi | down.m |
| STEAP4 | hsa-miR-148b-3p | up.mi | down.m |
| STK10 | hsa-miR-4652-5p | up.mi | down.m |
| STOM | hsa-miR-340-3p | up.mi | down.m |
| STOML3 | hsa-miR-96-5p | up.mi | down.m |
| STOML3 | hsa-miR-182-5p | up.mi | down.m |
| STOML3 | hsa-miR-5698 | up.mi | down.m |
| STOML3 | hsa-miR-625-5p | up.mi | down.m |
| STX11 | hsa-miR-136-3p | up.mi | down.m |
| STX11 | hsa-miR-15b-3p | up.mi | down.m |
| STX12 | hsa-miR-19a-3p | up.mi | down.m |
| STX12 | hsa-miR-106b-5p | up.mi | down.m |
| STX12 | hsa-miR-18a-5p | up.mi | down.m |
| STX12 | hsa-miR-30e-5p | up.mi | down.m |
| STX12 | hsa-miR-19b-3p | up.mi | down.m |
| STX2 | hsa-miR-142-3p | up.mi | down.m |
| STX2 | hsa-miR-181d-5p | up.mi | down.m |
| STX2 | hsa-miR-181b-5p | up.mi | down.m |
| STX2 | hsa-miR-455-3p | up.mi | down.m |
| STX2 | hsa-miR-3200-3p | up.mi | down.m |
| STX7 | hsa-miR-940 | up.mi | down.m |
| STX7 | hsa-miR-192-5p | up.mi | down.m |
| STX7 | hsa-miR-455-3p | up.mi | down.m |
| STX7 | hsa-miR-153-5p | up.mi | down.m |
| STX7 | hsa-miR-377-3p | up.mi | down.m |
| STX7 | hsa-miR-192-3p | up.mi | down.m |
| STX7 | hsa-miR-193b-3p | up.mi | down.m |
| STX7 | hsa-miR-215-5p | up.mi | down.m |
| SULT1C4 | hsa-miR-130b-5p | up.mi | down.m |
| SULT1C4 | hsa-miR-335-3p | up.mi | down.m |
| SUN2 | hsa-miR-454-3p | up.mi | down.m |
| SUN2 | hsa-miR-301a-3p | up.mi | down.m |
| SUN2 | hsa-miR-542-3p | up.mi | down.m |
| SUN2 | hsa-miR-130b-3p | up.mi | down.m |
| SUN2 | hsa-miR-301b-3p | up.mi | down.m |
| SUN2 | hsa-miR-758-3p | up.mi | down.m |
| SUN2 | hsa-miR-130a-3p | up.mi | down.m |
| SUSD2 | hsa-miR-1266-5p | up.mi | down.m |
| SUSD2 | hsa-miR-3934-3p | up.mi | down.m |
| SUSD6 | hsa-miR-5698 | up.mi | down.m |
| SUSD6 | hsa-miR-17-5p | up.mi | down.m |
| SUSD6 | hsa-miR-106b-5p | up.mi | down.m |
| SUSD6 | hsa-miR-3677-5p | up.mi | down.m |
| SUSD6 | hsa-miR-20a-5p | up.mi | down.m |
| SUSD6 | hsa-miR-93-5p | up.mi | down.m |
| SUSD6 | hsa-miR-20b-5p | up.mi | down.m |
| SUSD6 | hsa-miR-625-5p | up.mi | down.m |
| SVEP1 | hsa-miR-130b-5p | up.mi | down.m |
| SVEP1 | hsa-miR-501-5p | up.mi | down.m |
| SVEP1 | hsa-miR-20a-3p | up.mi | down.m |
| SYDE2 | hsa-miR-136-5p | up.mi | down.m |
| SYDE2 | hsa-miR-6510-3p | up.mi | down.m |
| SYDE2 | hsa-miR-616-5p | up.mi | down.m |
| SYDE2 | hsa-miR-31-5p | up.mi | down.m |
| SYDE2 | hsa-miR-182-5p | up.mi | down.m |
| SYNC | hsa-miR-3913-5p | up.mi | down.m |
| SYNE1 | hsa-miR-9-5p | up.mi | down.m |
| SYNE1 | hsa-miR-148b-3p | up.mi | down.m |
| SYNJ2BP | hsa-miR-20b-5p | up.mi | down.m |
| SYNJ2BP | hsa-miR-766-3p | up.mi | down.m |
| SYNJ2BP | hsa-miR-20a-5p | up.mi | down.m |
| SYNJ2BP | hsa-miR-17-5p | up.mi | down.m |
| SYNJ2BP | hsa-miR-183-5p | up.mi | down.m |
| SYNJ2BP | hsa-miR-4668-3p | up.mi | down.m |
| SYNJ2BP | hsa-miR-106a-5p | up.mi | down.m |
| SYNJ2BP | hsa-miR-616-5p | up.mi | down.m |
| SYNJ2BP | hsa-miR-431-5p | up.mi | down.m |
| SYNJ2BP | hsa-miR-542-3p | up.mi | down.m |
| SYNJ2BP | hsa-miR-340-3p | up.mi | down.m |
| SYNJ2BP | hsa-miR-532-3p | up.mi | down.m |
| SYNJ2BP | hsa-miR-940 | up.mi | down.m |
| SYNJ2BP | hsa-miR-186-5p | up.mi | down.m |
| SYNJ2BP | hsa-miR-106b-5p | up.mi | down.m |
| SYNJ2BP | hsa-miR-93-5p | up.mi | down.m |
| SYNM | hsa-miR-215-5p | up.mi | down.m |
| SYNM | hsa-miR-224-3p | up.mi | down.m |
| SYNM | hsa-miR-320b | up.mi | down.m |
| SYNM | hsa-miR-18a-5p | up.mi | down.m |
| SYNM | hsa-miR-192-5p | up.mi | down.m |
| SYNM | hsa-miR-331-3p | up.mi | down.m |
| SYNM | hsa-miR-96-5p | up.mi | down.m |
| SYNM | hsa-miR-182-5p | up.mi | down.m |
| SYNPO | hsa-miR-130b-5p | up.mi | down.m |
| SYNPO | hsa-miR-339-5p | up.mi | down.m |
| SYP | hsa-miR-369-3p | up.mi | down.m |
| SYT15 | hsa-miR-143-5p | up.mi | down.m |
| SYT15 | hsa-miR-940 | up.mi | down.m |
| TACC1 | hsa-miR-369-3p | up.mi | down.m |
| TACC1 | hsa-miR-758-3p | up.mi | down.m |
| TACC1 | hsa-miR-501-5p | up.mi | down.m |
| TACC1 | hsa-miR-4668-3p | up.mi | down.m |
| TACC1 | hsa-miR-148b-3p | up.mi | down.m |
| TACC1 | hsa-miR-193b-3p | up.mi | down.m |
| TACC1 | hsa-miR-92b-3p | up.mi | down.m |
| TACC1 | hsa-miR-22-3p | up.mi | down.m |
| TACC1 | hsa-miR-92a-3p | up.mi | down.m |
| TAGLN | hsa-miR-34b-5p | up.mi | down.m |
| TAGLN | hsa-miR-128-3p | up.mi | down.m |
| TAGLN | hsa-miR-9-5p | up.mi | down.m |
| TAL1 | hsa-miR-561-5p | up.mi | down.m |
| TAL1 | hsa-miR-577 | up.mi | down.m |
| TAL1 | hsa-miR-320b | up.mi | down.m |
| TAL1 | hsa-miR-340-3p | up.mi | down.m |
| TAL1 | hsa-miR-9-5p | up.mi | down.m |
| TAL1 | hsa-miR-377-3p | up.mi | down.m |
| TAPT1 | hsa-miR-155-5p | up.mi | down.m |
| TAPT1 | hsa-miR-193a-3p | up.mi | down.m |
| TBC1D1 | hsa-miR-34b-5p | up.mi | down.m |
| TBC1D1 | hsa-miR-193b-3p | up.mi | down.m |
| TBC1D1 | hsa-miR-7-1-3p | up.mi | down.m |
| TBC1D2 | hsa-miR-17-5p | up.mi | down.m |
| TBC1D2B | hsa-miR-128-3p | up.mi | down.m |
| TBC1D4 | hsa-miR-19b-3p | up.mi | down.m |
| TBC1D4 | hsa-miR-29b-2-5p | up.mi | down.m |
| TBC1D9 | hsa-miR-9-5p | up.mi | down.m |
| TBC1D9 | hsa-miR-106a-5p | up.mi | down.m |
| TBC1D9 | hsa-miR-381-3p | up.mi | down.m |
| TBC1D9 | hsa-miR-495-3p | up.mi | down.m |
| TBC1D9 | hsa-miR-106b-5p | up.mi | down.m |
| TBCEL | hsa-miR-92a-3p | up.mi | down.m |
| TBX2 | hsa-miR-331-3p | up.mi | down.m |
| TBX2 | hsa-miR-4652-5p | up.mi | down.m |
| TBX3 | hsa-miR-128-3p | up.mi | down.m |
| TBX3 | hsa-miR-22-3p | up.mi | down.m |
| TBX4 | hsa-miR-142-3p | up.mi | down.m |
| TBX4 | hsa-miR-20b-3p | up.mi | down.m |
| TCEAL7 | hsa-miR-182-5p | up.mi | down.m |
| TCF21 | hsa-miR-21-5p | up.mi | down.m |
| TCF4 | hsa-miR-17-5p | up.mi | down.m |
| TCF4 | hsa-miR-20b-5p | up.mi | down.m |
| TCF4 | hsa-miR-20a-5p | up.mi | down.m |
| TCF4 | hsa-miR-106b-5p | up.mi | down.m |
| TCF4 | hsa-miR-130b-3p | up.mi | down.m |
| TCF4 | hsa-miR-19b-3p | up.mi | down.m |
| TCF4 | hsa-miR-155-5p | up.mi | down.m |
| TCF4 | hsa-miR-93-5p | up.mi | down.m |
| TCF7L1 | hsa-miR-429 | up.mi | down.m |
| TCF7L1 | hsa-miR-200a-3p | up.mi | down.m |
| TCF7L1 | hsa-miR-200b-3p | up.mi | down.m |
| TCF7L1 | hsa-miR-200c-3p | up.mi | down.m |
| TCF7L1 | hsa-miR-141-3p | up.mi | down.m |
| TEF | hsa-miR-186-5p | up.mi | down.m |
| TEF | hsa-miR-92a-3p | up.mi | down.m |
| TEF | hsa-miR-181b-5p | up.mi | down.m |
| TEF | hsa-miR-92b-3p | up.mi | down.m |
| TEF | hsa-miR-181d-5p | up.mi | down.m |
| TEF | hsa-miR-532-3p | up.mi | down.m |
| TEF | hsa-miR-642a-5p | up.mi | down.m |
| TFEC | hsa-miR-335-3p | up.mi | down.m |
| TFPI | hsa-miR-92a-3p | up.mi | down.m |
| TFPI | hsa-miR-339-5p | up.mi | down.m |
| TFPI | hsa-miR-542-3p | up.mi | down.m |
| TFPI | hsa-miR-432-5p | up.mi | down.m |
| TFPI | hsa-miR-155-5p | up.mi | down.m |
| TFPI | hsa-miR-766-3p | up.mi | down.m |
| TFPI | hsa-miR-653-5p | up.mi | down.m |
| TFRC | hsa-miR-34a-5p | up.mi | down.m |
| TFRC | hsa-miR-181b-5p | up.mi | down.m |
| TFRC | hsa-miR-1301-3p | up.mi | down.m |
| TFRC | hsa-miR-136-5p | up.mi | down.m |
| TFRC | hsa-miR-210-3p | up.mi | down.m |
| TFRC | hsa-miR-200a-3p | up.mi | down.m |
| TFRC | hsa-miR-17-3p | up.mi | down.m |
| TFRC | hsa-miR-22-3p | up.mi | down.m |
| TFRC | hsa-miR-9-5p | up.mi | down.m |
| TFRC | hsa-miR-130b-5p | up.mi | down.m |
| TFRC | hsa-miR-181d-5p | up.mi | down.m |
| TGFB2 | hsa-miR-141-3p | up.mi | down.m |
| TGFB2 | hsa-miR-21-5p | up.mi | down.m |
| TGFB2 | hsa-miR-29b-3p | up.mi | down.m |
| TGFB2 | hsa-miR-142-5p | up.mi | down.m |
| TGFB2 | hsa-miR-193a-3p | up.mi | down.m |
| TGFB2 | hsa-miR-200a-3p | up.mi | down.m |
| TGFB2 | hsa-miR-153-5p | up.mi | down.m |
| TGFB2 | hsa-miR-148a-3p | up.mi | down.m |
| TGFB2 | hsa-miR-199a-5p | up.mi | down.m |
| TGFBR2 | hsa-miR-17-5p | up.mi | down.m |
| TGFBR2 | hsa-miR-93-5p | up.mi | down.m |
| TGFBR2 | hsa-miR-19b-3p | up.mi | down.m |
| TGFBR2 | hsa-miR-106b-5p | up.mi | down.m |
| TGFBR2 | hsa-miR-142-5p | up.mi | down.m |
| TGFBR2 | hsa-miR-21-5p | up.mi | down.m |
| TGFBR2 | hsa-miR-19a-3p | up.mi | down.m |
| TGFBR2 | hsa-miR-130a-3p | up.mi | down.m |
| TGFBR2 | hsa-miR-454-3p | up.mi | down.m |
| TGFBR2 | hsa-miR-20b-5p | up.mi | down.m |
| TGFBR2 | hsa-miR-130b-3p | up.mi | down.m |
| TGFBR2 | hsa-miR-590-5p | up.mi | down.m |
| TGFBR2 | hsa-miR-301a-3p | up.mi | down.m |
| TGFBR2 | hsa-miR-301b-3p | up.mi | down.m |
| TGFBR2 | hsa-miR-20a-5p | up.mi | down.m |
| TGFBR2 | hsa-miR-106a-5p | up.mi | down.m |
| TGFBR2 | hsa-miR-9-5p | up.mi | down.m |
| TGFBR2 | hsa-miR-34a-5p | up.mi | down.m |
| TGFBR2 | hsa-miR-196b-5p | up.mi | down.m |
| TGFBR2 | hsa-miR-34b-5p | up.mi | down.m |
| TGFBR2 | hsa-miR-582-5p | up.mi | down.m |
| TGFBR2 | hsa-miR-18a-5p | up.mi | down.m |
| TGFBR2 | hsa-miR-370-3p | up.mi | down.m |
| TGFBR2 | hsa-miR-92a-3p | up.mi | down.m |
| TGFBR2 | hsa-miR-495-3p | up.mi | down.m |
| TGFBR2 | hsa-miR-409-3p | up.mi | down.m |
| TGFBR2 | hsa-miR-505-3p | up.mi | down.m |
| TGFBR2 | hsa-miR-449a | up.mi | down.m |
| TGFBR2 | hsa-miR-196a-5p | up.mi | down.m |
| TGFBR2 | hsa-miR-655-3p | up.mi | down.m |
| TGFBR2 | hsa-miR-940 | up.mi | down.m |
| TGFBR3 | hsa-miR-136-3p | up.mi | down.m |
| TGFBR3 | hsa-miR-130b-5p | up.mi | down.m |
| TGFBR3 | hsa-miR-128-3p | up.mi | down.m |
| TGFBR3 | hsa-miR-186-5p | up.mi | down.m |
| TGFBR3 | hsa-miR-193b-3p | up.mi | down.m |
| TGFBR3 | hsa-miR-196a-5p | up.mi | down.m |
| TGFBR3 | hsa-miR-582-5p | up.mi | down.m |
| TGFBR3 | hsa-miR-21-5p | up.mi | down.m |
| TGFBR3 | hsa-miR-196b-5p | up.mi | down.m |
| TGFBR3 | hsa-miR-424-5p | up.mi | down.m |
| TGFBR3 | hsa-miR-15a-5p | up.mi | down.m |
| TGM2 | hsa-miR-155-5p | up.mi | down.m |
| TGM2 | hsa-miR-34a-5p | up.mi | down.m |
| THBD | hsa-miR-3127-5p | up.mi | down.m |
| THBD | hsa-miR-192-5p | up.mi | down.m |
| THBD | hsa-miR-215-5p | up.mi | down.m |
| THBS1 | hsa-miR-155-5p | up.mi | down.m |
| THBS1 | hsa-miR-455-3p | up.mi | down.m |
| THBS1 | hsa-miR-19a-3p | up.mi | down.m |
| THBS1 | hsa-miR-7-1-3p | up.mi | down.m |
| THBS1 | hsa-miR-675-3p | up.mi | down.m |
| THBS1 | hsa-miR-20a-5p | up.mi | down.m |
| THBS1 | hsa-miR-182-5p | up.mi | down.m |
| THBS1 | hsa-miR-487b-3p | up.mi | down.m |
| THBS1 | hsa-miR-19b-3p | up.mi | down.m |
| THBS1 | hsa-miR-17-5p | up.mi | down.m |
| THBS1 | hsa-miR-92a-3p | up.mi | down.m |
| THRA | hsa-miR-135b-3p | up.mi | down.m |
| THRA | hsa-miR-5698 | up.mi | down.m |
| THRA | hsa-miR-18a-5p | up.mi | down.m |
| THRA | hsa-miR-130a-3p | up.mi | down.m |
| THRA | hsa-miR-301b-3p | up.mi | down.m |
| THRA | hsa-miR-301a-3p | up.mi | down.m |
| THRA | hsa-miR-130b-3p | up.mi | down.m |
| THRA | hsa-miR-708-5p | up.mi | down.m |
| THRA | hsa-miR-143-5p | up.mi | down.m |
| THRA | hsa-miR-28-5p | up.mi | down.m |
| THRA | hsa-miR-454-3p | up.mi | down.m |
| THRB | hsa-miR-9-3p | up.mi | down.m |
| THRB | hsa-miR-425-5p | up.mi | down.m |
| THRB | hsa-miR-15b-3p | up.mi | down.m |
| THRB | hsa-miR-200a-3p | up.mi | down.m |
| THRB | hsa-miR-4677-3p | up.mi | down.m |
| THRB | hsa-miR-320b | up.mi | down.m |
| THRB | hsa-miR-155-5p | up.mi | down.m |
| THSD4 | hsa-miR-5698 | up.mi | down.m |
| THSD4 | hsa-miR-625-5p | up.mi | down.m |
| THSD4 | hsa-miR-766-3p | up.mi | down.m |
| TIMP2 | hsa-miR-429 | up.mi | down.m |
| TIMP2 | hsa-miR-106a-5p | up.mi | down.m |
| TIMP2 | hsa-miR-301a-3p | up.mi | down.m |
| TIMP2 | hsa-miR-20a-5p | up.mi | down.m |
| TIMP2 | hsa-miR-200c-3p | up.mi | down.m |
| TIMP3 | hsa-miR-21-5p | up.mi | down.m |
| TIMP3 | hsa-miR-17-3p | up.mi | down.m |
| TIMP3 | hsa-miR-181b-5p | up.mi | down.m |
| TIMP3 | hsa-miR-17-5p | up.mi | down.m |
| TIPARP | hsa-miR-142-3p | up.mi | down.m |
| TJP1 | hsa-miR-615-3p | up.mi | down.m |
| TJP1 | hsa-miR-194-5p | up.mi | down.m |
| TJP1 | hsa-miR-105-5p | up.mi | down.m |
| TJP1 | hsa-miR-155-5p | up.mi | down.m |
| TJP1 | hsa-miR-17-5p | up.mi | down.m |
| TJP1 | hsa-miR-324-3p | up.mi | down.m |
| TJP1 | hsa-miR-212-3p | up.mi | down.m |
| TJP2 | hsa-miR-215-5p | up.mi | down.m |
| TJP2 | hsa-miR-192-5p | up.mi | down.m |
| TJP2 | hsa-miR-193b-3p | up.mi | down.m |
| TJP2 | hsa-miR-34a-5p | up.mi | down.m |
| TLCD2 | hsa-miR-767-5p | up.mi | down.m |
| TLCD2 | hsa-miR-590-3p | up.mi | down.m |
| TLCD2 | hsa-miR-455-3p | up.mi | down.m |
| TLCD2 | hsa-miR-501-3p | up.mi | down.m |
| TLCD2 | hsa-miR-542-3p | up.mi | down.m |
| TLCD2 | hsa-miR-377-3p | up.mi | down.m |
| TLCD2 | hsa-miR-766-3p | up.mi | down.m |
| TLCD2 | hsa-miR-361-3p | up.mi | down.m |
| TLE4 | hsa-miR-193b-3p | up.mi | down.m |
| TLE4 | hsa-miR-155-5p | up.mi | down.m |
| TLE4 | hsa-miR-15a-5p | up.mi | down.m |
| TLE4 | hsa-miR-186-5p | up.mi | down.m |
| TLL1 | hsa-miR-589-5p | up.mi | down.m |
| TLL1 | hsa-miR-424-5p | up.mi | down.m |
| TLL1 | hsa-miR-503-5p | up.mi | down.m |
| TLL1 | hsa-miR-15a-5p | up.mi | down.m |
| TLN1 | hsa-miR-625-5p | up.mi | down.m |
| TLN1 | hsa-miR-186-5p | up.mi | down.m |
| TLN1 | hsa-miR-940 | up.mi | down.m |
| TLN1 | hsa-miR-193b-3p | up.mi | down.m |
| TLN1 | hsa-miR-616-5p | up.mi | down.m |
| TLN1 | hsa-miR-92b-3p | up.mi | down.m |
| TLN1 | hsa-miR-193a-3p | up.mi | down.m |
| TLN1 | hsa-miR-340-3p | up.mi | down.m |
| TLN1 | hsa-miR-92a-3p | up.mi | down.m |
| TLN1 | hsa-miR-561-5p | up.mi | down.m |
| TLN1 | hsa-miR-5698 | up.mi | down.m |
| TLN1 | hsa-miR-3913-5p | up.mi | down.m |
| TLN1 | hsa-miR-760 | up.mi | down.m |
| TLR2 | hsa-miR-105-5p | up.mi | down.m |
| TLR2 | hsa-miR-19b-3p | up.mi | down.m |
| TLR2 | hsa-miR-154-5p | up.mi | down.m |
| TLR2 | hsa-miR-19a-3p | up.mi | down.m |
| TLR2 | hsa-miR-106b-5p | up.mi | down.m |
| TLR3 | hsa-miR-92b-3p | up.mi | down.m |
| TLR3 | hsa-miR-758-3p | up.mi | down.m |
| TLR3 | hsa-miR-21-5p | up.mi | down.m |
| TLR3 | hsa-miR-92a-3p | up.mi | down.m |
| TLR4 | hsa-miR-21-5p | up.mi | down.m |
| TLR7 | hsa-miR-20b-5p | up.mi | down.m |
| TLR7 | hsa-miR-17-5p | up.mi | down.m |
| TLR7 | hsa-miR-20a-5p | up.mi | down.m |
| TLR7 | hsa-miR-758-3p | up.mi | down.m |
| TLR7 | hsa-miR-106a-5p | up.mi | down.m |
| TLR7 | hsa-miR-93-5p | up.mi | down.m |
| TLR7 | hsa-miR-106b-5p | up.mi | down.m |
| TLR7 | hsa-miR-19a-3p | up.mi | down.m |
| TM6SF1 | hsa-miR-590-3p | up.mi | down.m |
| TM6SF1 | hsa-miR-155-5p | up.mi | down.m |
| TMCC3 | hsa-miR-92a-3p | up.mi | down.m |
| TMEM100 | hsa-miR-93-5p | up.mi | down.m |
| TMEM100 | hsa-miR-20a-5p | up.mi | down.m |
| TMEM100 | hsa-miR-15a-5p | up.mi | down.m |
| TMEM100 | hsa-miR-424-5p | up.mi | down.m |
| TMEM100 | hsa-miR-503-5p | up.mi | down.m |
| TMEM100 | hsa-miR-20b-5p | up.mi | down.m |
| TMEM100 | hsa-miR-106a-5p | up.mi | down.m |
| TMEM100 | hsa-miR-17-5p | up.mi | down.m |
| TMEM100 | hsa-miR-106b-5p | up.mi | down.m |
| TMEM109 | hsa-miR-31-5p | up.mi | down.m |
| TMEM109 | hsa-miR-130b-5p | up.mi | down.m |
| TMEM109 | hsa-miR-331-3p | up.mi | down.m |
| TMEM109 | hsa-miR-1306-5p | up.mi | down.m |
| TMEM109 | hsa-miR-130b-3p | up.mi | down.m |
| TMEM109 | hsa-miR-143-5p | up.mi | down.m |
| TMEM109 | hsa-miR-493-3p | up.mi | down.m |
| TMEM109 | hsa-miR-34a-5p | up.mi | down.m |
| TMEM109 | hsa-miR-2355-5p | up.mi | down.m |
| TMEM109 | hsa-miR-185-5p | up.mi | down.m |
| TMEM109 | hsa-miR-3127-5p | up.mi | down.m |
| TMEM109 | hsa-miR-15a-5p | up.mi | down.m |
| TMEM164 | hsa-miR-193b-3p | up.mi | down.m |
| TMEM164 | hsa-miR-215-5p | up.mi | down.m |
| TMEM164 | hsa-miR-4668-3p | up.mi | down.m |
| TMEM164 | hsa-miR-192-5p | up.mi | down.m |
| TMEM170B | hsa-miR-188-5p | up.mi | down.m |
| TMEM170B | hsa-miR-128-3p | up.mi | down.m |
| TMEM170B | hsa-miR-7-1-3p | up.mi | down.m |
| TMEM170B | hsa-miR-33a-5p | up.mi | down.m |
| TMEM170B | hsa-miR-495-3p | up.mi | down.m |
| TMEM170B | hsa-miR-182-5p | up.mi | down.m |
| TMEM170B | hsa-miR-96-5p | up.mi | down.m |
| TMEM170B | hsa-miR-183-5p | up.mi | down.m |
| TMEM170B | hsa-miR-33b-5p | up.mi | down.m |
| TMEM2 | hsa-miR-192-5p | up.mi | down.m |
| TMEM2 | hsa-miR-551b-3p | up.mi | down.m |
| TMEM2 | hsa-miR-21-5p | up.mi | down.m |
| TMEM2 | hsa-miR-495-3p | up.mi | down.m |
| TMEM2 | hsa-miR-196a-5p | up.mi | down.m |
| TMEM2 | hsa-miR-130a-3p | up.mi | down.m |
| TMEM2 | hsa-miR-141-5p | up.mi | down.m |
| TMEM2 | hsa-miR-454-3p | up.mi | down.m |
| TMEM2 | hsa-miR-548v | up.mi | down.m |
| TMEM2 | hsa-miR-19b-3p | up.mi | down.m |
| TMEM2 | hsa-miR-19a-3p | up.mi | down.m |
| TMEM2 | hsa-miR-215-5p | up.mi | down.m |
| TMEM2 | hsa-miR-18a-5p | up.mi | down.m |
| TMEM2 | hsa-miR-186-5p | up.mi | down.m |
| TMEM2 | hsa-miR-130b-3p | up.mi | down.m |
| TMEM2 | hsa-miR-7-1-3p | up.mi | down.m |
| TMEM2 | hsa-miR-331-3p | up.mi | down.m |
| TMEM2 | hsa-miR-301a-3p | up.mi | down.m |
| TMEM2 | hsa-miR-301b-3p | up.mi | down.m |
| TMEM204 | hsa-miR-193b-3p | up.mi | down.m |
| TMEM231 | hsa-miR-24-2-5p | up.mi | down.m |
| TMEM231 | hsa-miR-24-1-5p | up.mi | down.m |
| TMEM231 | hsa-miR-561-5p | up.mi | down.m |
| TMEM245 | hsa-miR-17-5p | up.mi | down.m |
| TMEM245 | hsa-miR-376b-3p | up.mi | down.m |
| TMEM245 | hsa-miR-769-3p | up.mi | down.m |
| TMEM245 | hsa-miR-424-5p | up.mi | down.m |
| TMEM245 | hsa-miR-21-5p | up.mi | down.m |
| TMEM245 | hsa-miR-19b-1-5p | up.mi | down.m |
| TMEM245 | hsa-miR-186-5p | up.mi | down.m |
| TMEM245 | hsa-miR-3170 | up.mi | down.m |
| TMEM245 | hsa-miR-20b-5p | up.mi | down.m |
| TMEM245 | hsa-miR-193b-5p | up.mi | down.m |
| TMEM245 | hsa-miR-15a-5p | up.mi | down.m |
| TMEM245 | hsa-miR-450b-5p | up.mi | down.m |
| TMEM245 | hsa-miR-27b-5p | up.mi | down.m |
| TMEM245 | hsa-miR-106b-5p | up.mi | down.m |
| TMEM245 | hsa-miR-9-5p | up.mi | down.m |
| TMEM245 | hsa-miR-93-5p | up.mi | down.m |
| TMEM245 | hsa-miR-3913-5p | up.mi | down.m |
| TMEM245 | hsa-miR-503-5p | up.mi | down.m |
| TMEM245 | hsa-miR-183-5p | up.mi | down.m |
| TMEM245 | hsa-miR-3189-3p | up.mi | down.m |
| TMEM245 | hsa-miR-361-3p | up.mi | down.m |
| TMEM245 | hsa-miR-142-3p | up.mi | down.m |
| TMEM245 | hsa-miR-93-3p | up.mi | down.m |
| TMEM245 | hsa-miR-20a-5p | up.mi | down.m |
| TMEM246 | hsa-miR-215-5p | up.mi | down.m |
| TMEM246 | hsa-miR-148b-3p | up.mi | down.m |
| TMEM246 | hsa-miR-34a-5p | up.mi | down.m |
| TMEM246 | hsa-miR-192-5p | up.mi | down.m |
| TMEM246 | hsa-miR-148a-3p | up.mi | down.m |
| TMEM246 | hsa-miR-34b-5p | up.mi | down.m |
| TMEM246 | hsa-miR-2355-5p | up.mi | down.m |
| TMEM246 | hsa-miR-449a | up.mi | down.m |
| TMEM37 | hsa-miR-505-3p | up.mi | down.m |
| TMEM47 | hsa-miR-148b-3p | up.mi | down.m |
| TMEM64 | hsa-miR-20b-5p | up.mi | down.m |
| TMEM64 | hsa-miR-655-3p | up.mi | down.m |
| TMEM64 | hsa-miR-19b-3p | up.mi | down.m |
| TMEM64 | hsa-miR-106b-5p | up.mi | down.m |
| TMEM64 | hsa-miR-19a-3p | up.mi | down.m |
| TMEM64 | hsa-miR-331-3p | up.mi | down.m |
| TMEM64 | hsa-miR-186-5p | up.mi | down.m |
| TMEM64 | hsa-miR-17-5p | up.mi | down.m |
| TMEM64 | hsa-miR-20a-5p | up.mi | down.m |
| TMEM64 | hsa-miR-323b-3p | up.mi | down.m |
| TMEM64 | hsa-miR-93-5p | up.mi | down.m |
| TMEM64 | hsa-miR-106a-5p | up.mi | down.m |
| TMEM74B | hsa-miR-766-3p | up.mi | down.m |
| TMEM88 | hsa-miR-708-5p | up.mi | down.m |
| TMOD2 | hsa-miR-203b-3p | up.mi | down.m |
| TMOD2 | hsa-miR-1306-5p | up.mi | down.m |
| TMOD2 | hsa-miR-199b-5p | up.mi | down.m |
| TMOD2 | hsa-miR-34a-5p | up.mi | down.m |
| TMOD2 | hsa-miR-199a-5p | up.mi | down.m |
| TMOD3 | hsa-miR-17-5p | up.mi | down.m |
| TMOD3 | hsa-miR-19a-3p | up.mi | down.m |
| TMOD3 | hsa-miR-301a-3p | up.mi | down.m |
| TMOD3 | hsa-miR-34a-5p | up.mi | down.m |
| TMOD3 | hsa-miR-20b-5p | up.mi | down.m |
| TMOD3 | hsa-miR-20a-5p | up.mi | down.m |
| TMOD3 | hsa-miR-141-3p | up.mi | down.m |
| TMOD3 | hsa-miR-301b-3p | up.mi | down.m |
| TMOD3 | hsa-miR-29a-5p | up.mi | down.m |
| TMOD3 | hsa-miR-106b-5p | up.mi | down.m |
| TMOD3 | hsa-miR-130a-3p | up.mi | down.m |
| TMOD3 | hsa-miR-130b-3p | up.mi | down.m |
| TMOD3 | hsa-miR-200a-3p | up.mi | down.m |
| TMOD3 | hsa-miR-3677-5p | up.mi | down.m |
| TMOD3 | hsa-miR-454-3p | up.mi | down.m |
| TMOD3 | hsa-miR-505-3p | up.mi | down.m |
| TMOD3 | hsa-miR-106a-5p | up.mi | down.m |
| TMOD3 | hsa-miR-19b-3p | up.mi | down.m |
| TMOD3 | hsa-miR-93-5p | up.mi | down.m |
| TMOD3 | hsa-miR-155-5p | up.mi | down.m |
| TMOD3 | hsa-miR-185-5p | up.mi | down.m |
| TMOD3 | hsa-miR-767-5p | up.mi | down.m |
| TMSB4X | hsa-miR-148a-5p | up.mi | down.m |
| TMSB4X | hsa-miR-29a-5p | up.mi | down.m |
| TMSB4X | hsa-miR-183-5p | up.mi | down.m |
| TMSB4X | hsa-miR-205-5p | up.mi | down.m |
| TMTC1 | hsa-miR-335-3p | up.mi | down.m |
| TMTC1 | hsa-miR-19a-3p | up.mi | down.m |
| TMTC1 | hsa-miR-369-3p | up.mi | down.m |
| TMTC1 | hsa-miR-130b-3p | up.mi | down.m |
| TMTC1 | hsa-miR-454-3p | up.mi | down.m |
| TMTC1 | hsa-miR-130a-3p | up.mi | down.m |
| TMTC1 | hsa-miR-193b-3p | up.mi | down.m |
| TMTC1 | hsa-miR-188-5p | up.mi | down.m |
| TMTC1 | hsa-miR-301b-3p | up.mi | down.m |
| TMTC1 | hsa-miR-655-3p | up.mi | down.m |
| TMTC1 | hsa-miR-19b-3p | up.mi | down.m |
| TMTC1 | hsa-miR-301a-3p | up.mi | down.m |
| TNFAIP3 | hsa-miR-18a-5p | up.mi | down.m |
| TNFAIP3 | hsa-miR-19a-3p | up.mi | down.m |
| TNFAIP3 | hsa-miR-21-5p | up.mi | down.m |
| TNFAIP3 | hsa-miR-29b-3p | up.mi | down.m |
| TNFAIP3 | hsa-miR-130a-5p | up.mi | down.m |
| TNFAIP3 | hsa-miR-19b-3p | up.mi | down.m |
| TNFAIP3 | hsa-miR-505-3p | up.mi | down.m |
| TNFAIP3 | hsa-miR-335-3p | up.mi | down.m |
| TNFAIP3 | hsa-miR-141-5p | up.mi | down.m |
| TNFAIP8L3 | hsa-miR-505-5p | up.mi | down.m |
| TNFRSF1B | hsa-miR-193a-3p | up.mi | down.m |
| TNFRSF1B | hsa-miR-193b-3p | up.mi | down.m |
| TNFRSF1B | hsa-miR-19b-3p | up.mi | down.m |
| TNFRSF1B | hsa-miR-19a-3p | up.mi | down.m |
| TNFSF12 | hsa-miR-17-5p | up.mi | down.m |
| TNFSF12 | hsa-miR-215-5p | up.mi | down.m |
| TNFSF13 | hsa-miR-192-5p | up.mi | down.m |
| TNNC1 | hsa-miR-20b-3p | up.mi | down.m |
| TNS1 | hsa-miR-130b-5p | up.mi | down.m |
| TNS1 | hsa-miR-148a-5p | up.mi | down.m |
| TNS1 | hsa-miR-142-5p | up.mi | down.m |
| TNS1 | hsa-miR-4326 | up.mi | down.m |
| TNS1 | hsa-miR-33b-5p | up.mi | down.m |
| TNS1 | hsa-miR-33a-5p | up.mi | down.m |
| TNS1 | hsa-miR-22-5p | up.mi | down.m |
| TNS3 | hsa-miR-377-3p | up.mi | down.m |
| TNS3 | hsa-miR-21-5p | up.mi | down.m |
| TNXB | hsa-miR-1301-3p | up.mi | down.m |
| TOM1L2 | hsa-miR-224-5p | up.mi | down.m |
| TOM1L2 | hsa-miR-148b-3p | up.mi | down.m |
| TOM1L2 | hsa-miR-19b-3p | up.mi | down.m |
| TOM1L2 | hsa-miR-105-5p | up.mi | down.m |
| TOM1L2 | hsa-miR-130b-3p | up.mi | down.m |
| TOM1L2 | hsa-miR-4728-3p | up.mi | down.m |
| TOM1L2 | hsa-miR-616-5p | up.mi | down.m |
| TP53INP2 | hsa-miR-629-5p | up.mi | down.m |
| TP53INP2 | hsa-miR-128-3p | up.mi | down.m |
| TPPP | hsa-miR-92a-3p | up.mi | down.m |
| TPPP | hsa-miR-128-3p | up.mi | down.m |
| TPPP | hsa-miR-760 | up.mi | down.m |
| TPPP | hsa-miR-34a-5p | up.mi | down.m |
| TPPP | hsa-miR-92b-3p | up.mi | down.m |
| TPPP | hsa-miR-377-3p | up.mi | down.m |
| TPST2 | hsa-miR-615-3p | up.mi | down.m |
| TPST2 | hsa-miR-148a-5p | up.mi | down.m |
| TRAK2 | hsa-miR-19a-3p | up.mi | down.m |
| TRAK2 | hsa-miR-193b-3p | up.mi | down.m |
| TRAK2 | hsa-miR-9-5p | up.mi | down.m |
| TRAK2 | hsa-miR-19b-3p | up.mi | down.m |
| TREM1 | hsa-miR-505-3p | up.mi | down.m |
| TREM1 | hsa-miR-766-3p | up.mi | down.m |
| TREM2 | hsa-miR-34a-5p | up.mi | down.m |
| TRPV2 | hsa-miR-449a | up.mi | down.m |
| TRPV2 | hsa-miR-141-5p | up.mi | down.m |
| TRPV2 | hsa-miR-382-5p | up.mi | down.m |
| TRPV2 | hsa-miR-337-3p | up.mi | down.m |
| TRPV2 | hsa-miR-3170 | up.mi | down.m |
| TRPV2 | hsa-miR-3136-5p | up.mi | down.m |
| TRPV2 | hsa-miR-34a-5p | up.mi | down.m |
| TRPV2 | hsa-miR-3127-5p | up.mi | down.m |
| TRPV2 | hsa-miR-760 | up.mi | down.m |
| TRPV2 | hsa-miR-192-3p | up.mi | down.m |
| TRPV2 | hsa-miR-455-5p | up.mi | down.m |
| TRPV2 | hsa-miR-193b-5p | up.mi | down.m |
| TSC22D1 | hsa-miR-590-3p | up.mi | down.m |
| TSC22D1 | hsa-miR-708-5p | up.mi | down.m |
| TSC22D1 | hsa-miR-199b-5p | up.mi | down.m |
| TSC22D1 | hsa-miR-9-3p | up.mi | down.m |
| TSC22D1 | hsa-miR-28-5p | up.mi | down.m |
| TSC22D1 | hsa-miR-199a-5p | up.mi | down.m |
| TSC22D3 | hsa-miR-19b-3p | up.mi | down.m |
| TSC22D3 | hsa-miR-505-3p | up.mi | down.m |
| TSC22D3 | hsa-miR-182-5p | up.mi | down.m |
| TSC22D3 | hsa-miR-18a-5p | up.mi | down.m |
| TSPAN12 | hsa-miR-212-3p | up.mi | down.m |
| TSPAN12 | hsa-miR-196a-5p | up.mi | down.m |
| TSPAN13 | hsa-miR-548v | up.mi | down.m |
| TSPAN13 | hsa-miR-128-3p | up.mi | down.m |
| TSPAN18 | hsa-miR-92a-3p | up.mi | down.m |
| TSPAN2 | hsa-miR-664a-3p | up.mi | down.m |
| TSPAN2 | hsa-miR-452-3p | up.mi | down.m |
| TSPAN9 | hsa-miR-1180-3p | up.mi | down.m |
| TSPYL1 | hsa-miR-382-5p | up.mi | down.m |
| TSPYL1 | hsa-miR-142-3p | up.mi | down.m |
| TSPYL1 | hsa-miR-940 | up.mi | down.m |
| TSPYL1 | hsa-miR-539-5p | up.mi | down.m |
| TSPYL1 | hsa-miR-455-3p | up.mi | down.m |
| TSPYL1 | hsa-miR-16-1-3p | up.mi | down.m |
| TSPYL1 | hsa-miR-92b-3p | up.mi | down.m |
| TSPYL1 | hsa-miR-128-3p | up.mi | down.m |
| TSPYL1 | hsa-miR-30e-5p | up.mi | down.m |
| TTC28 | hsa-miR-550a-5p | up.mi | down.m |
| TTC28 | hsa-miR-20b-3p | up.mi | down.m |
| TTC28 | hsa-miR-182-5p | up.mi | down.m |
| TTC28 | hsa-miR-214-5p | up.mi | down.m |
| TTLL7 | hsa-miR-4677-3p | up.mi | down.m |
| TTLL7 | hsa-miR-136-3p | up.mi | down.m |
| TTLL7 | hsa-miR-92a-3p | up.mi | down.m |
| TTLL7 | hsa-miR-135b-5p | up.mi | down.m |
| TUBA1A | hsa-miR-324-5p | up.mi | down.m |
| TUBA1A | hsa-miR-615-3p | up.mi | down.m |
| TUBB1 | hsa-miR-183-5p | up.mi | down.m |
| TUBB6 | hsa-miR-92b-3p | up.mi | down.m |
| TXNIP | hsa-miR-128-3p | up.mi | down.m |
| TXNIP | hsa-miR-629-5p | up.mi | down.m |
| TXNIP | hsa-miR-17-3p | up.mi | down.m |
| TXNIP | hsa-miR-183-5p | up.mi | down.m |
| TXNIP | hsa-miR-449a | up.mi | down.m |
| TXNIP | hsa-miR-20b-5p | up.mi | down.m |
| TXNIP | hsa-miR-130a-5p | up.mi | down.m |
| TXNIP | hsa-miR-130b-3p | up.mi | down.m |
| TXNIP | hsa-miR-18a-5p | up.mi | down.m |
| TXNIP | hsa-miR-148a-3p | up.mi | down.m |
| TXNIP | hsa-miR-135b-5p | up.mi | down.m |
| TXNIP | hsa-miR-31-3p | up.mi | down.m |
| TXNIP | hsa-miR-301a-3p | up.mi | down.m |
| TXNIP | hsa-miR-224-5p | up.mi | down.m |
| TXNIP | hsa-miR-301b-3p | up.mi | down.m |
| TXNIP | hsa-miR-424-5p | up.mi | down.m |
| TXNIP | hsa-miR-454-3p | up.mi | down.m |
| TXNIP | hsa-miR-29b-1-5p | up.mi | down.m |
| TXNIP | hsa-miR-323a-3p | up.mi | down.m |
| TXNIP | hsa-miR-106a-5p | up.mi | down.m |
| TXNIP | hsa-miR-134-5p | up.mi | down.m |
| TXNIP | hsa-miR-3136-5p | up.mi | down.m |
| TXNIP | hsa-miR-20a-5p | up.mi | down.m |
| TXNIP | hsa-miR-93-5p | up.mi | down.m |
| TXNIP | hsa-miR-34a-5p | up.mi | down.m |
| TXNIP | hsa-miR-130a-3p | up.mi | down.m |
| TXNIP | hsa-miR-16-1-3p | up.mi | down.m |
| TXNIP | hsa-miR-155-3p | up.mi | down.m |
| TXNIP | hsa-miR-3189-3p | up.mi | down.m |
| TXNIP | hsa-miR-455-3p | up.mi | down.m |
| TXNIP | hsa-miR-4326 | up.mi | down.m |
| TXNIP | hsa-miR-106b-5p | up.mi | down.m |
| TXNIP | hsa-miR-17-5p | up.mi | down.m |
| TXNIP | hsa-miR-15a-5p | up.mi | down.m |
| TXNIP | hsa-miR-495-3p | up.mi | down.m |
| TXNIP | hsa-miR-148b-3p | up.mi | down.m |
| TYRP1 | hsa-miR-155-5p | up.mi | down.m |
| TYRP1 | hsa-miR-4668-3p | up.mi | down.m |
| TYRP1 | hsa-miR-889-3p | up.mi | down.m |
| UACA | hsa-miR-192-5p | up.mi | down.m |
| UACA | hsa-miR-940 | up.mi | down.m |
| UACA | hsa-miR-215-5p | up.mi | down.m |
| UBASH3B | hsa-miR-215-5p | up.mi | down.m |
| UBASH3B | hsa-miR-200a-3p | up.mi | down.m |
| UBASH3B | hsa-let-7a-2-3p | up.mi | down.m |
| UBASH3B | hsa-miR-192-5p | up.mi | down.m |
| UBASH3B | hsa-miR-192-3p | up.mi | down.m |
| UBASH3B | hsa-let-7g-3p | up.mi | down.m |
| UBASH3B | hsa-miR-455-5p | up.mi | down.m |
| UBE2E2 | hsa-miR-149-5p | up.mi | down.m |
| UBE2E2 | hsa-miR-186-5p | up.mi | down.m |
| UBL3 | hsa-miR-19a-3p | up.mi | down.m |
| UBL3 | hsa-miR-192-5p | up.mi | down.m |
| UBL3 | hsa-miR-194-5p | up.mi | down.m |
| UBL3 | hsa-miR-192-3p | up.mi | down.m |
| UBL3 | hsa-miR-19b-3p | up.mi | down.m |
| UBL3 | hsa-miR-186-5p | up.mi | down.m |
| UBL3 | hsa-miR-215-5p | up.mi | down.m |
| UBL3 | hsa-miR-155-5p | up.mi | down.m |
| ULK2 | hsa-miR-532-3p | up.mi | down.m |
| ULK2 | hsa-miR-214-5p | up.mi | down.m |
| ULK2 | hsa-miR-455-3p | up.mi | down.m |
| ULK2 | hsa-miR-301a-5p | up.mi | down.m |
| ULK2 | hsa-miR-3127-5p | up.mi | down.m |
| ULK2 | hsa-miR-33b-5p | up.mi | down.m |
| UNC13B | hsa-miR-92a-3p | up.mi | down.m |
| USP12 | hsa-miR-215-5p | up.mi | down.m |
| USP12 | hsa-miR-192-5p | up.mi | down.m |
| USP12 | hsa-miR-625-5p | up.mi | down.m |
| USP2 | hsa-miR-493-5p | up.mi | down.m |
| USP54 | hsa-miR-215-5p | up.mi | down.m |
| USP54 | hsa-miR-192-5p | up.mi | down.m |
| UST | hsa-miR-449a | up.mi | down.m |
| UST | hsa-miR-34a-5p | up.mi | down.m |
| UST | hsa-miR-149-5p | up.mi | down.m |
| UST | hsa-miR-154-5p | up.mi | down.m |
| UST | hsa-miR-369-3p | up.mi | down.m |
| UTRN | hsa-miR-505-3p | up.mi | down.m |
| UTRN | hsa-miR-455-3p | up.mi | down.m |
| UTRN | hsa-miR-331-3p | up.mi | down.m |
| UTRN | hsa-miR-21-5p | up.mi | down.m |
| VAMP2 | hsa-miR-34a-5p | up.mi | down.m |
| VAMP3 | hsa-miR-19a-3p | up.mi | down.m |
| VAMP3 | hsa-miR-19b-3p | up.mi | down.m |
| VAMP3 | hsa-miR-155-5p | up.mi | down.m |
| VAMP3 | hsa-miR-382-5p | up.mi | down.m |
| VAMP3 | hsa-miR-29a-5p | up.mi | down.m |
| VASH1 | hsa-miR-30e-5p | up.mi | down.m |
| VAT1 | hsa-miR-642a-5p | up.mi | down.m |
| VAT1 | hsa-miR-301a-5p | up.mi | down.m |
| VAT1 | hsa-miR-4652-5p | up.mi | down.m |
| VAT1 | hsa-miR-93-5p | up.mi | down.m |
| VEGFC | hsa-miR-128-3p | up.mi | down.m |
| VEGFC | hsa-miR-577 | up.mi | down.m |
| VEGFC | hsa-miR-766-3p | up.mi | down.m |
| VGLL3 | hsa-miR-224-5p | up.mi | down.m |
| VGLL3 | hsa-miR-192-5p | up.mi | down.m |
| VGLL3 | hsa-miR-766-3p | up.mi | down.m |
| VIM | hsa-miR-17-3p | up.mi | down.m |
| VIM | hsa-miR-134-5p | up.mi | down.m |
| VIM | hsa-miR-1287-5p | up.mi | down.m |
| VIM | hsa-miR-615-3p | up.mi | down.m |
| VIM | hsa-miR-1301-3p | up.mi | down.m |
| VIM | hsa-miR-9-5p | up.mi | down.m |
| VIPR1 | hsa-miR-532-3p | up.mi | down.m |
| VLDLR | hsa-miR-17-5p | up.mi | down.m |
| VLDLR | hsa-miR-767-3p | up.mi | down.m |
| VLDLR | hsa-miR-664a-3p | up.mi | down.m |
| VLDLR | hsa-miR-493-3p | up.mi | down.m |
| VLDLR | hsa-miR-130b-3p | up.mi | down.m |
| VLDLR | hsa-miR-93-5p | up.mi | down.m |
| VLDLR | hsa-miR-2355-3p | up.mi | down.m |
| VLDLR | hsa-miR-409-5p | up.mi | down.m |
| VLDLR | hsa-miR-616-5p | up.mi | down.m |
| VSIG10 | hsa-miR-335-3p | up.mi | down.m |
| VSIG10 | hsa-miR-1306-5p | up.mi | down.m |
| VSIG2 | hsa-miR-1307-3p | up.mi | down.m |
| VSIG2 | hsa-miR-154-5p | up.mi | down.m |
| VSTM4 | hsa-miR-153-5p | up.mi | down.m |
| VSTM4 | hsa-miR-1307-3p | up.mi | down.m |
| WARS | hsa-miR-92b-3p | up.mi | down.m |
| WASF3 | hsa-miR-542-5p | up.mi | down.m |
| WASF3 | hsa-miR-130b-5p | up.mi | down.m |
| WASF3 | hsa-miR-4652-5p | up.mi | down.m |
| WASF3 | hsa-miR-200b-3p | up.mi | down.m |
| WASF3 | hsa-miR-31-5p | up.mi | down.m |
| WASF3 | hsa-miR-188-5p | up.mi | down.m |
| WASF3 | hsa-miR-429 | up.mi | down.m |
| WASF3 | hsa-miR-200a-3p | up.mi | down.m |
| WASF3 | hsa-miR-141-5p | up.mi | down.m |
| WASF3 | hsa-miR-539-5p | up.mi | down.m |
| WFDC6 | hsa-miR-22-5p | up.mi | down.m |
| WFDC6 | hsa-miR-3189-3p | up.mi | down.m |
| WFDC6 | hsa-miR-769-3p | up.mi | down.m |
| WFS1 | hsa-miR-21-5p | up.mi | down.m |
| WISP2 | hsa-miR-449a | up.mi | down.m |
| WLS | hsa-miR-186-5p | up.mi | down.m |
| WLS | hsa-miR-331-5p | up.mi | down.m |
| WNT2 | hsa-miR-199a-5p | up.mi | down.m |
| WNT3A | hsa-miR-15a-5p | up.mi | down.m |
| WNT3A | hsa-miR-128-3p | up.mi | down.m |
| WNT7A | hsa-miR-664a-3p | up.mi | down.m |
| WWC1 | hsa-miR-155-5p | up.mi | down.m |
| WWC1 | hsa-miR-106a-5p | up.mi | down.m |
| WWC1 | hsa-miR-17-5p | up.mi | down.m |
| WWC1 | hsa-miR-20b-5p | up.mi | down.m |
| WWC1 | hsa-miR-106b-5p | up.mi | down.m |
| WWC1 | hsa-miR-324-3p | up.mi | down.m |
| WWC1 | hsa-miR-93-5p | up.mi | down.m |
| WWC1 | hsa-miR-5698 | up.mi | down.m |
| WWC1 | hsa-miR-20a-5p | up.mi | down.m |
| WWC1 | hsa-miR-22-3p | up.mi | down.m |
| WWC1 | hsa-miR-143-5p | up.mi | down.m |
| WWC2 | hsa-miR-561-5p | up.mi | down.m |
| WWC2 | hsa-miR-21-5p | up.mi | down.m |
| WWC2 | hsa-miR-215-5p | up.mi | down.m |
| WWC2 | hsa-miR-766-3p | up.mi | down.m |
| WWC2 | hsa-miR-192-5p | up.mi | down.m |
| WWC3 | hsa-miR-192-5p | up.mi | down.m |
| WWC3 | hsa-miR-215-5p | up.mi | down.m |
| WWTR1 | hsa-miR-3913-5p | up.mi | down.m |
| WWTR1 | hsa-miR-193b-3p | up.mi | down.m |
| WWTR1 | hsa-miR-369-3p | up.mi | down.m |
| WWTR1 | hsa-miR-200b-5p | up.mi | down.m |
| WWTR1 | hsa-miR-18a-5p | up.mi | down.m |
| WWTR1 | hsa-miR-200a-5p | up.mi | down.m |
| WWTR1 | hsa-miR-29b-3p | up.mi | down.m |
| ZBED2 | hsa-miR-889-3p | up.mi | down.m |
| ZBED3 | hsa-miR-127-5p | up.mi | down.m |
| ZBED3 | hsa-miR-17-5p | up.mi | down.m |
| ZBED3 | hsa-miR-9-5p | up.mi | down.m |
| ZBED3 | hsa-miR-369-3p | up.mi | down.m |
| ZBED3 | hsa-miR-431-5p | up.mi | down.m |
| ZBTB16 | hsa-miR-301b-3p | up.mi | down.m |
| ZBTB16 | hsa-miR-424-5p | up.mi | down.m |
| ZBTB16 | hsa-miR-452-3p | up.mi | down.m |
| ZBTB16 | hsa-miR-361-3p | up.mi | down.m |
| ZBTB16 | hsa-miR-1306-5p | up.mi | down.m |
| ZBTB16 | hsa-miR-15a-5p | up.mi | down.m |
| ZBTB4 | hsa-miR-130b-3p | up.mi | down.m |
| ZBTB4 | hsa-miR-20a-5p | up.mi | down.m |
| ZBTB4 | hsa-miR-671-5p | up.mi | down.m |
| ZBTB4 | hsa-miR-106a-5p | up.mi | down.m |
| ZBTB4 | hsa-miR-454-3p | up.mi | down.m |
| ZBTB4 | hsa-miR-93-5p | up.mi | down.m |
| ZBTB4 | hsa-miR-106b-5p | up.mi | down.m |
| ZBTB4 | hsa-miR-431-5p | up.mi | down.m |
| ZBTB4 | hsa-miR-301a-3p | up.mi | down.m |
| ZBTB4 | hsa-miR-331-3p | up.mi | down.m |
| ZBTB4 | hsa-miR-20b-5p | up.mi | down.m |
| ZBTB4 | hsa-miR-17-5p | up.mi | down.m |
| ZBTB4 | hsa-miR-181b-5p | up.mi | down.m |
| ZBTB4 | hsa-miR-19b-3p | up.mi | down.m |
| ZBTB4 | hsa-miR-130a-3p | up.mi | down.m |
| ZBTB4 | hsa-miR-19a-3p | up.mi | down.m |
| ZBTB4 | hsa-miR-186-5p | up.mi | down.m |
| ZBTB4 | hsa-miR-301b-3p | up.mi | down.m |
| ZBTB4 | hsa-miR-615-3p | up.mi | down.m |
| ZBTB4 | hsa-miR-181d-5p | up.mi | down.m |
| ZBTB4 | hsa-miR-381-3p | up.mi | down.m |
| ZBTB47 | hsa-miR-18a-5p | up.mi | down.m |
| ZBTB47 | hsa-miR-629-5p | up.mi | down.m |
| ZBTB47 | hsa-miR-7-1-3p | up.mi | down.m |
| ZBTB47 | hsa-miR-19b-3p | up.mi | down.m |
| ZBTB47 | hsa-miR-19a-3p | up.mi | down.m |
| ZBTB47 | hsa-miR-542-5p | up.mi | down.m |
| ZBTB47 | hsa-miR-21-5p | up.mi | down.m |
| ZBTB47 | hsa-miR-193b-5p | up.mi | down.m |
| ZBTB47 | hsa-miR-495-3p | up.mi | down.m |
| ZBTB47 | hsa-miR-28-5p | up.mi | down.m |
| ZBTB47 | hsa-miR-106a-5p | up.mi | down.m |
| ZBTB7C | hsa-miR-106b-3p | up.mi | down.m |
| ZC3H12C | hsa-miR-20a-5p | up.mi | down.m |
| ZC3H12C | hsa-miR-106b-5p | up.mi | down.m |
| ZC3H12C | hsa-miR-550a-3p | up.mi | down.m |
| ZC3H12C | hsa-miR-20b-5p | up.mi | down.m |
| ZC3H12C | hsa-miR-93-5p | up.mi | down.m |
| ZC3H12C | hsa-miR-106a-5p | up.mi | down.m |
| ZC3H12C | hsa-miR-17-5p | up.mi | down.m |
| ZC3H12C | hsa-miR-192-5p | up.mi | down.m |
| ZC3H12C | hsa-miR-889-3p | up.mi | down.m |
| ZC3H12C | hsa-miR-767-5p | up.mi | down.m |
| ZC3H12C | hsa-miR-576-5p | up.mi | down.m |
| ZC3H12C | hsa-miR-33a-5p | up.mi | down.m |
| ZC3H12C | hsa-miR-501-3p | up.mi | down.m |
| ZC3H12C | hsa-miR-532-3p | up.mi | down.m |
| ZC3H12C | hsa-miR-31-5p | up.mi | down.m |
| ZC3H12C | hsa-miR-200c-5p | up.mi | down.m |
| ZCCHC24 | hsa-miR-629-3p | up.mi | down.m |
| ZCCHC24 | hsa-miR-532-3p | up.mi | down.m |
| ZCCHC24 | hsa-miR-188-3p | up.mi | down.m |
| ZCCHC24 | hsa-miR-1301-3p | up.mi | down.m |
| ZCCHC24 | hsa-miR-335-3p | up.mi | down.m |
| ZCCHC24 | hsa-miR-29a-5p | up.mi | down.m |
| ZDHHC2 | hsa-miR-127-5p | up.mi | down.m |
| ZDHHC2 | hsa-miR-154-3p | up.mi | down.m |
| ZDHHC2 | hsa-miR-192-5p | up.mi | down.m |
| ZDHHC2 | hsa-miR-215-5p | up.mi | down.m |
| ZEB1 | hsa-miR-200c-3p | up.mi | down.m |
| ZEB1 | hsa-miR-590-3p | up.mi | down.m |
| ZEB1 | hsa-miR-539-5p | up.mi | down.m |
| ZEB1 | hsa-miR-200b-3p | up.mi | down.m |
| ZEB1 | hsa-miR-409-3p | up.mi | down.m |
| ZEB1 | hsa-miR-19b-1-5p | up.mi | down.m |
| ZEB1 | hsa-miR-141-3p | up.mi | down.m |
| ZEB1 | hsa-miR-33b-5p | up.mi | down.m |
| ZEB1 | hsa-miR-200a-3p | up.mi | down.m |
| ZEB1 | hsa-miR-130b-3p | up.mi | down.m |
| ZEB1 | hsa-miR-429 | up.mi | down.m |
| ZEB1 | hsa-miR-455-3p | up.mi | down.m |
| ZEB1 | hsa-miR-183-5p | up.mi | down.m |
| ZEB1 | hsa-miR-205-5p | up.mi | down.m |
| ZEB1 | hsa-miR-629-5p | up.mi | down.m |
| ZEB1 | hsa-miR-96-5p | up.mi | down.m |
| ZEB1 | hsa-miR-655-3p | up.mi | down.m |
| ZEB1 | hsa-miR-142-5p | up.mi | down.m |
| ZEB2 | hsa-miR-141-3p | up.mi | down.m |
| ZEB2 | hsa-miR-200a-3p | up.mi | down.m |
| ZEB2 | hsa-miR-200b-3p | up.mi | down.m |
| ZEB2 | hsa-miR-429 | up.mi | down.m |
| ZEB2 | hsa-miR-200c-3p | up.mi | down.m |
| ZEB2 | hsa-miR-205-5p | up.mi | down.m |
| ZEB2 | hsa-miR-708-5p | up.mi | down.m |
| ZEB2 | hsa-miR-590-3p | up.mi | down.m |
| ZEB2 | hsa-miR-153-3p | up.mi | down.m |
| ZEB2 | hsa-miR-34a-5p | up.mi | down.m |
| ZEB2 | hsa-miR-153-5p | up.mi | down.m |
| ZEB2 | hsa-miR-30e-5p | up.mi | down.m |
| ZEB2 | hsa-miR-154-5p | up.mi | down.m |
| ZEB2 | hsa-miR-215-5p | up.mi | down.m |
| ZFP36 | hsa-miR-155-5p | up.mi | down.m |
| ZFP36 | hsa-miR-5698 | up.mi | down.m |
| ZFP36 | hsa-miR-625-5p | up.mi | down.m |
| ZFP36 | hsa-miR-17-3p | up.mi | down.m |
| ZFP36L2 | hsa-miR-582-5p | up.mi | down.m |
| ZFP36L2 | hsa-miR-142-3p | up.mi | down.m |
| ZFP36L2 | hsa-miR-181d-5p | up.mi | down.m |
| ZFP36L2 | hsa-miR-186-5p | up.mi | down.m |
| ZFP36L2 | hsa-miR-671-5p | up.mi | down.m |
| ZFP36L2 | hsa-miR-181b-5p | up.mi | down.m |
| ZFP36L2 | hsa-miR-18a-5p | up.mi | down.m |
| ZFP36L2 | hsa-miR-2355-5p | up.mi | down.m |
| ZFYVE9 | hsa-miR-93-5p | up.mi | down.m |
| ZFYVE9 | hsa-miR-301a-3p | up.mi | down.m |
| ZFYVE9 | hsa-miR-106a-5p | up.mi | down.m |
| ZFYVE9 | hsa-miR-301b-3p | up.mi | down.m |
| ZFYVE9 | hsa-miR-106b-5p | up.mi | down.m |
| ZFYVE9 | hsa-miR-130b-3p | up.mi | down.m |
| ZFYVE9 | hsa-miR-19a-3p | up.mi | down.m |
| ZFYVE9 | hsa-miR-20a-5p | up.mi | down.m |
| ZFYVE9 | hsa-miR-503-3p | up.mi | down.m |
| ZFYVE9 | hsa-miR-130a-3p | up.mi | down.m |
| ZFYVE9 | hsa-miR-20b-5p | up.mi | down.m |
| ZFYVE9 | hsa-miR-214-5p | up.mi | down.m |
| ZFYVE9 | hsa-miR-454-3p | up.mi | down.m |
| ZFYVE9 | hsa-miR-19b-3p | up.mi | down.m |
| ZFYVE9 | hsa-miR-141-5p | up.mi | down.m |
| ZFYVE9 | hsa-miR-17-5p | up.mi | down.m |
| ZFYVE9 | hsa-miR-615-3p | up.mi | down.m |
| ZHX3 | hsa-miR-425-5p | up.mi | down.m |
| ZMIZ1 | hsa-miR-96-5p | up.mi | down.m |
| ZMIZ1 | hsa-miR-1180-3p | up.mi | down.m |
| ZMIZ1 | hsa-miR-493-5p | up.mi | down.m |
| ZMIZ1 | hsa-let-7g-3p | up.mi | down.m |
| ZMIZ1 | hsa-let-7a-2-3p | up.mi | down.m |
| ZMIZ1 | hsa-miR-1301-3p | up.mi | down.m |
| ZMIZ1 | hsa-miR-331-3p | up.mi | down.m |
| ZMIZ1 | hsa-miR-93-5p | up.mi | down.m |
| ZMIZ1 | hsa-let-7c-3p | up.mi | down.m |
| ZMIZ1 | hsa-miR-29b-2-5p | up.mi | down.m |
| ZNF106 | hsa-miR-199b-3p | up.mi | down.m |
| ZNF106 | hsa-miR-199a-3p | up.mi | down.m |
| ZNF106 | hsa-miR-3136-5p | up.mi | down.m |
| ZNF106 | hsa-miR-5698 | up.mi | down.m |
| ZNF106 | hsa-miR-28-5p | up.mi | down.m |
| ZNF106 | hsa-miR-143-5p | up.mi | down.m |
| ZNF106 | hsa-miR-134-5p | up.mi | down.m |
| ZNF106 | hsa-miR-345-5p | up.mi | down.m |
| ZNF25 | hsa-miR-431-5p | up.mi | down.m |
| ZNF25 | hsa-miR-153-5p | up.mi | down.m |
| ZNF25 | hsa-miR-450b-5p | up.mi | down.m |
| ZNF25 | hsa-miR-1301-3p | up.mi | down.m |
| ZNF331 | hsa-miR-20a-5p | up.mi | down.m |
| ZNF331 | hsa-miR-31-5p | up.mi | down.m |
| ZNF331 | hsa-miR-766-3p | up.mi | down.m |
| ZNF331 | hsa-miR-6510-3p | up.mi | down.m |
| ZNF365 | hsa-miR-193b-3p | up.mi | down.m |
| ZNF366 | hsa-miR-193b-5p | up.mi | down.m |
| ZNF366 | hsa-miR-19b-1-5p | up.mi | down.m |
| ZNF366 | hsa-miR-590-3p | up.mi | down.m |
| ZNF385B | hsa-miR-192-5p | up.mi | down.m |
| ZNF385B | hsa-miR-215-5p | up.mi | down.m |
| ZNF423 | hsa-miR-17-3p | up.mi | down.m |
| ZNF423 | hsa-miR-19b-3p | up.mi | down.m |
| ZNF423 | hsa-miR-19a-3p | up.mi | down.m |
| ZNF423 | hsa-miR-135b-3p | up.mi | down.m |
| ZNF704 | hsa-miR-3934-3p | up.mi | down.m |
| ZNF704 | hsa-miR-9-5p | up.mi | down.m |
| ZNF704 | hsa-miR-130a-5p | up.mi | down.m |
| ZNF704 | hsa-let-7g-3p | up.mi | down.m |
| ZNF704 | hsa-miR-192-5p | up.mi | down.m |
| ZNF704 | hsa-miR-130b-5p | up.mi | down.m |
| ZNF704 | hsa-miR-2355-5p | up.mi | down.m |
| ZNF704 | hsa-let-7a-2-3p | up.mi | down.m |
| ZNF704 | hsa-miR-203b-3p | up.mi | down.m |
| ZNF704 | hsa-miR-424-5p | up.mi | down.m |
| ZNF704 | hsa-miR-15a-5p | up.mi | down.m |
| ZNF704 | hsa-miR-431-5p | up.mi | down.m |
| ZNF704 | hsa-miR-503-5p | up.mi | down.m |
| ZNF704 | hsa-miR-675-3p | up.mi | down.m |
| ZYX | hsa-miR-96-5p | up.mi | down.m |
| ZYX | hsa-miR-16-1-3p | up.mi | down.m |
| ZYX | hsa-miR-193b-3p | up.mi | down.m |
