# Supplementary material for: Prognostic value of lncRNAs related to fatty acid metabolism in lung adenocarcinoma and their correlation with tumor microenvironment based on bioinformatics analysis
Source: Front Oncol. 2022 Oct 10;12:1022097. doi: 10.3389/fonc.2022.1022097 (PMC9590110; doi:10.3389/fonc.2022.1022097)
Supplement: Supplementary Table 1 — All samples were divided into high and low fatty acid metabolism score groups based on the median value of this score. [file DataSheet_1.zip › raw data and R code for checking/raw data/12.docx]

| lnc | m | r | p.value | qvalue |
| --- | --- | --- | --- | --- |
| RP11-259K15.2 | 2-Mar | 0.520775 | 8.39E-41 | 2.73E-39 |
| RP11-401P9.4 | 2-Mar | 0.474575 | 3.05E-33 | 5.83E-32 |
| RP11-4B16.3 | 2-Mar | 0.417556 | 2.26E-25 | 2.29E-24 |
| RP11-401P9.4 | 4-Sep | 0.661171 | 1.16E-72 | 3.87E-70 |
| RP11-4B16.3 | 4-Sep | 0.519168 | 1.61E-40 | 5.16E-39 |
| RP11-401P9.4 | A2M | 0.623199 | 2.02E-62 | 3.12E-60 |
| RP11-4B16.3 | A2M | 0.424373 | 3.09E-26 | 3.37E-25 |
| RP11-259K15.2 | ABCA3 | 0.545554 | 2.32E-45 | 1.04E-43 |
| RP11-401P9.4 | ABCA3 | 0.489122 | 1.68E-35 | 3.80E-34 |
| RP11-401P9.4 | ABCA6 | 0.573217 | 6.39E-51 | 4.30E-49 |
| RP11-4B16.3 | ABCA6 | 0.444812 | 5.99E-29 | 8.22E-28 |
| RP11-259K15.2 | ABCA8 | 0.445122 | 5.43E-29 | 7.48E-28 |
| RP11-401P9.4 | ABCA8 | 0.701663 | 2.28E-85 | 3.00E-82 |
| RP11-4B16.3 | ABCA8 | 0.587764 | 4.61E-54 | 3.89E-52 |
| RP11-401P9.4 | ABCA9 | 0.631394 | 1.64E-64 | 2.94E-62 |
| RP11-4B16.3 | ABCA9 | 0.512865 | 2.00E-39 | 5.95E-38 |
| RP11-401P9.4 | ABCB1 | 0.424819 | 2.71E-26 | 2.96E-25 |
| RP11-259K15.2 | ABCC6 | 0.520277 | 1.03E-40 | 3.34E-39 |
| RP11-401P9.4 | ABCG2 | 0.444555 | 6.49E-29 | 8.89E-28 |
| RP11-401P9.4 | ABHD6 | 0.472373 | 6.55E-33 | 1.23E-31 |
| RP11-4B16.3 | ABHD6 | 0.424972 | 2.59E-26 | 2.84E-25 |
| RP11-401P9.4 | ABI3BP | 0.632895 | 6.70E-65 | 1.29E-62 |
| RP11-4B16.3 | ABI3BP | 0.527639 | 4.99E-42 | 1.80E-40 |
| RP11-401P9.4 | ABLIM3 | 0.438517 | 4.29E-28 | 5.54E-27 |
| RP11-4B16.3 | ABLIM3 | 0.45605 | 1.61E-30 | 2.48E-29 |
| RP11-401P9.4 | ABTB1 | 0.411683 | 1.21E-24 | 1.15E-23 |
| RP11-4B16.3 | AC011380.1 | 0.452768 | 4.68E-30 | 6.98E-29 |
| RP11-401P9.4 | ACACB | 0.614617 | 2.67E-60 | 3.57E-58 |
| RP11-4B16.3 | ACACB | 0.446989 | 3.00E-29 | 4.21E-28 |
| RP11-259K15.2 | ACADL | 0.466371 | 5.12E-32 | 8.97E-31 |
| RP11-401P9.4 | ACADL | 0.605893 | 3.30E-58 | 3.69E-56 |
| RP11-4B16.3 | ACADL | 0.5876 | 5.01E-54 | 4.20E-52 |
| RP11-401P9.4 | ACE | 0.501823 | 1.46E-37 | 3.86E-36 |
| RP11-4B16.3 | ACE | 0.559698 | 3.87E-48 | 2.13E-46 |
| RP11-401P9.4 | ACKR1 | 0.519656 | 1.32E-40 | 4.25E-39 |
| RP11-401P9.4 | ACKR4 | 0.480246 | 4.14E-34 | 8.42E-33 |
| RP11-4B16.3 | ACKR4 | 0.577661 | 7.28E-52 | 5.30E-50 |
| RP11-259K15.2 | ACOXL | 0.515989 | 5.78E-40 | 1.77E-38 |
| RP11-401P9.4 | ACOXL | 0.501123 | 1.91E-37 | 4.99E-36 |
| RP11-4B16.3 | ACOXL | 0.41353 | 7.16E-25 | 6.93E-24 |
| RP11-4B16.3 | ACP5 | 0.405251 | 7.32E-24 | 6.40E-23 |
| RP11-401P9.4 | ACSS3 | 0.562267 | 1.17E-48 | 6.73E-47 |
| RP11-401P9.4 | ACTG2 | 0.435233 | 1.18E-27 | 1.46E-26 |
| RP11-401P9.4 | ACTN2 | 0.615326 | 1.79E-60 | 2.44E-58 |
| RP11-4B16.3 | ACTN2 | 0.578812 | 4.13E-52 | 3.08E-50 |
| RP11-401P9.4 | ACVRL1 | 0.59535 | 9.14E-56 | 8.80E-54 |
| RP11-4B16.3 | ACVRL1 | 0.692983 | 1.82E-82 | 1.59E-79 |
| RP5-1059L7.1 | ADAM12 | 0.43063 | 4.77E-27 | 5.56E-26 |
| RP11-401P9.4 | ADAMTS1 | 0.501203 | 1.85E-37 | 4.85E-36 |
| RP11-4B16.3 | ADAMTS1 | 0.420603 | 9.33E-26 | 9.75E-25 |
| RP5-1059L7.1 | ADAMTS12 | 0.411177 | 1.40E-24 | 1.31E-23 |
| RP11-259K15.2 | ADAMTS8 | 0.442506 | 1.24E-28 | 1.66E-27 |
| RP11-401P9.4 | ADAMTS8 | 0.75279 | ####### | ####### |
| RP11-4B16.3 | ADAMTS8 | 0.624959 | 7.26E-63 | 1.17E-60 |
| RP11-401P9.4 | ADAMTSL3 | 0.620465 | 9.72E-62 | 1.45E-59 |
| RP11-4B16.3 | ADAMTSL3 | 0.569137 | 4.56E-50 | 2.92E-48 |
| RP11-401P9.4 | ADAMTSL4 | 0.414042 | 6.19E-25 | 6.02E-24 |
| RP11-4B16.3 | ADAMTSL4 | 0.423758 | 3.70E-26 | 4.01E-25 |
| RP11-401P9.4 | ADARB1 | 0.661226 | 1.12E-72 | 3.77E-70 |
| RP11-4B16.3 | ADARB1 | 0.536824 | 1.03E-43 | 4.19E-42 |
| RP11-401P9.4 | ADCY4 | 0.601534 | 3.46E-57 | 3.64E-55 |
| RP11-4B16.3 | ADCY4 | 0.622492 | 3.03E-62 | 4.67E-60 |
| RP11-401P9.4 | ADCY8 | 0.450791 | 8.88E-30 | 1.29E-28 |
| RP11-4B16.3 | ADCY8 | 0.491291 | 7.60E-36 | 1.78E-34 |
| RP11-401P9.4 | ADCY9 | 0.460488 | 3.70E-31 | 6.04E-30 |
| RP11-401P9.4 | ADD1 | 0.490647 | 9.63E-36 | 2.23E-34 |
| RP11-259K15.2 | ADH1B | 0.492148 | 5.54E-36 | 1.31E-34 |
| RP11-401P9.4 | ADH1B | 0.66332 | 2.76E-73 | 1.00E-70 |
| RP11-4B16.3 | ADH1B | 0.526876 | 6.86E-42 | 2.43E-40 |
| RP11-259K15.2 | ADHFE1 | 0.456373 | 1.44E-30 | 2.24E-29 |
| RP11-401P9.4 | ADHFE1 | 0.442845 | 1.11E-28 | 1.50E-27 |
| CTA-384D8.35 | ADM2 | 0.42808 | 1.03E-26 | 1.16E-25 |
| Z83851.4 | ADM2 | 0.425898 | 1.97E-26 | 2.18E-25 |
| RP11-401P9.4 | ADPRH | 0.527632 | 5.01E-42 | 1.80E-40 |
| RP11-4B16.3 | ADPRH | 0.497407 | 7.79E-37 | 1.96E-35 |
| RP11-401P9.4 | ADRA1A | 0.61756 | 5.09E-61 | 7.20E-59 |
| RP11-4B16.3 | ADRA1A | 0.554841 | 3.60E-47 | 1.83E-45 |
| RP11-259K15.2 | ADRB1 | 0.412349 | 1.00E-24 | 9.57E-24 |
| RP11-401P9.4 | ADRB1 | 0.534534 | 2.75E-43 | 1.08E-41 |
| RP11-4B16.3 | ADRB1 | 0.574616 | 3.24E-51 | 2.22E-49 |
| RP11-259K15.2 | ADRB2 | 0.422912 | 4.75E-26 | 5.10E-25 |
| RP11-401P9.4 | ADRB2 | 0.602727 | 1.83E-57 | 1.96E-55 |
| RP11-4B16.3 | ADRB2 | 0.615042 | 2.10E-60 | 2.84E-58 |
| RP11-401P9.4 | ADTRP | 0.412193 | 1.05E-24 | 9.98E-24 |
| RP11-4B16.3 | ADTRP | 0.453186 | 4.09E-30 | 6.12E-29 |
| RP11-401P9.4 | AFAP1L1 | 0.501925 | 1.41E-37 | 3.72E-36 |
| RP11-4B16.3 | AFAP1L1 | 0.548483 | 6.32E-46 | 2.93E-44 |
| RP11-259K15.2 | AFF3 | 0.460522 | 3.66E-31 | 5.97E-30 |
| RP11-401P9.4 | AFF3 | 0.591913 | 5.47E-55 | 5.00E-53 |
| RP11-4B16.3 | AFF3 | 0.517277 | 3.45E-40 | 1.07E-38 |
| RP11-259K15.2 | AGER | 0.469958 | 1.51E-32 | 2.73E-31 |
| RP11-401P9.4 | AGER | 0.610173 | 3.16E-59 | 3.83E-57 |
| RP11-4B16.3 | AGER | 0.618208 | 3.52E-61 | 5.05E-59 |
| RP11-401P9.4 | AGPAT4 | 0.449841 | 1.21E-29 | 1.74E-28 |
| RP11-4B16.3 | AGPAT4 | 0.400433 | 2.75E-23 | 2.28E-22 |
| RP11-259K15.2 | AGRP | 0.441463 | 1.72E-28 | 2.29E-27 |
| RP11-401P9.4 | AGRP | 0.479613 | 5.18E-34 | 1.04E-32 |
| RP11-4B16.3 | AGRP | 0.530821 | 1.32E-42 | 4.96E-41 |
| RP11-4B16.3 | AGTPBP1 | 0.480062 | 4.42E-34 | 8.96E-33 |
| RP11-401P9.4 | AGTR1 | 0.462951 | 1.63E-31 | 2.74E-30 |
| RP11-4B16.3 | AGTR1 | 0.45666 | 1.31E-30 | 2.05E-29 |
| RP11-401P9.4 | AGTR2 | 0.424322 | 3.13E-26 | 3.42E-25 |
| RP11-4B16.3 | AGTR2 | 0.462857 | 1.68E-31 | 2.83E-30 |
| RP11-401P9.4 | AHNAK | 0.507405 | 1.70E-38 | 4.81E-37 |
| RP11-4B16.3 | AHNAK | 0.47746 | 1.11E-33 | 2.18E-32 |
| RP11-259K15.2 | AK1 | 0.624016 | 1.26E-62 | 1.98E-60 |
| RP11-401P9.4 | AKAP13 | 0.491496 | 7.05E-36 | 1.66E-34 |
| RP11-401P9.4 | AKAP2 | 0.550404 | 2.68E-46 | 1.28E-44 |
| RP11-4B16.3 | AKAP2 | 0.619578 | 1.61E-61 | 2.36E-59 |
| RP11-4B16.3 | AKAP7 | 0.406727 | 4.86E-24 | 4.33E-23 |
| RP11-401P9.4 | AKT3 | 0.429085 | 7.60E-27 | 8.68E-26 |
| RP11-4B16.3 | ALAS2 | 0.468659 | 2.35E-32 | 4.21E-31 |
| RP11-259K15.2 | ALDH2 | 0.478085 | 8.89E-34 | 1.76E-32 |
| RP11-401P9.4 | ALDH2 | 0.421115 | 8.04E-26 | 8.44E-25 |
| Z83851.4 | ALG3 | 0.44703 | 2.96E-29 | 4.16E-28 |
| Z83851.4 | ALG8 | 0.42198 | 6.24E-26 | 6.63E-25 |
| RP11-259K15.2 | ALOX15B | 0.504617 | 5.01E-38 | 1.36E-36 |
| RP11-401P9.4 | ALOX5 | 0.412575 | 9.40E-25 | 9.00E-24 |
| RP11-4B16.3 | ALOX5 | 0.400098 | 3.01E-23 | 2.49E-22 |
| RP11-259K15.2 | ALPL | 0.415079 | 4.60E-25 | 4.52E-24 |
| RP11-401P9.4 | AMICA1 | 0.486025 | 5.20E-35 | 1.13E-33 |
| RP11-4B16.3 | AMICA1 | 0.464226 | 1.06E-31 | 1.81E-30 |
| RP11-401P9.4 | AMIGO1 | 0.436667 | 7.59E-28 | 9.64E-27 |
| RP11-401P9.4 | AMOTL1 | 0.551006 | 2.04E-46 | 9.89E-45 |
| RP11-4B16.3 | AMOTL1 | 0.471897 | 7.72E-33 | 1.44E-31 |
| RP11-401P9.4 | ANGPT1 | 0.632537 | 8.30E-65 | 1.58E-62 |
| RP11-4B16.3 | ANGPT1 | 0.50031 | 2.60E-37 | 6.72E-36 |
| RP11-401P9.4 | ANGPT4 | 0.560207 | 3.06E-48 | 1.70E-46 |
| RP11-4B16.3 | ANGPT4 | 0.567612 | 9.43E-50 | 5.87E-48 |
| RP11-401P9.4 | ANGPTL1 | 0.61019 | 3.13E-59 | 3.81E-57 |
| RP11-4B16.3 | ANGPTL1 | 0.562132 | 1.25E-48 | 7.13E-47 |
| RP11-401P9.4 | ANGPTL7 | 0.616555 | 8.98E-61 | 1.24E-58 |
| RP11-4B16.3 | ANGPTL7 | 0.661042 | 1.27E-72 | 4.17E-70 |
| RP11-401P9.4 | ANKRD1 | 0.557431 | 1.10E-47 | 5.77E-46 |
| RP11-4B16.3 | ANKRD1 | 0.631785 | 1.30E-64 | 2.36E-62 |
| RP11-259K15.2 | ANKRD29 | 0.505979 | 2.96E-38 | 8.20E-37 |
| RP11-401P9.4 | ANKRD29 | 0.462819 | 1.70E-31 | 2.86E-30 |
| RP11-4B16.3 | ANKRD29 | 0.442854 | 1.11E-28 | 1.50E-27 |
| RP11-401P9.4 | ANKRD44 | 0.42211 | 6.01E-26 | 6.39E-25 |
| RP11-401P9.4 | ANKS1A | 0.432591 | 2.64E-27 | 3.15E-26 |
| RP5-1059L7.1 | ANLN | 0.421577 | 7.02E-26 | 7.43E-25 |
| Z83851.4 | ANLN | 0.452818 | 4.61E-30 | 6.88E-29 |
| RP11-4B16.3 | ANO6 | 0.400008 | 3.09E-23 | 2.55E-22 |
| RP11-4B16.3 | ANXA3 | 0.450504 | 9.74E-30 | 1.42E-28 |
| RP11-401P9.4 | ANXA6 | 0.403127 | 1.32E-23 | 1.12E-22 |
| RP11-401P9.4 | ANXA8L1 | 0.413596 | 7.03E-25 | 6.80E-24 |
| RP11-4B16.3 | ANXA8L1 | 0.454823 | 2.40E-30 | 3.66E-29 |
| RP11-401P9.4 | AOC3 | 0.68675 | 1.91E-80 | 1.43E-77 |
| RP11-4B16.3 | AOC3 | 0.523225 | 3.09E-41 | 1.05E-39 |
| RP11-401P9.4 | APBB1 | 0.452411 | 5.26E-30 | 7.81E-29 |
| RP11-401P9.4 | APBB2 | 0.493553 | 3.29E-36 | 7.93E-35 |
| RP11-401P9.4 | APH1B | 0.403901 | 1.06E-23 | 9.16E-23 |
| RP11-401P9.4 | APOBR | 0.443984 | 7.78E-29 | 1.06E-27 |
| RP11-4B16.3 | APOL3 | 0.438775 | 3.96E-28 | 5.13E-27 |
| RP11-259K15.2 | AQP4 | 0.517566 | 3.07E-40 | 9.64E-39 |
| RP11-401P9.4 | AQP4 | 0.460436 | 3.77E-31 | 6.14E-30 |
| RP11-4B16.3 | AQP4 | 0.454835 | 2.39E-30 | 3.65E-29 |
| RP11-4B16.3 | ARAP2 | 0.432841 | 2.45E-27 | 2.93E-26 |
| RP11-401P9.4 | ARAP3 | 0.567001 | 1.26E-49 | 7.77E-48 |
| RP11-4B16.3 | ARAP3 | 0.550313 | 2.79E-46 | 1.33E-44 |
| RP11-401P9.4 | ARC | 0.570857 | 2.00E-50 | 1.30E-48 |
| RP11-4B16.3 | ARC | 0.579578 | 2.82E-52 | 2.12E-50 |
| RP11-4B16.3 | ARHGAP18 | 0.464864 | 8.54E-32 | 1.47E-30 |
| RP11-401P9.4 | ARHGAP23 | 0.417873 | 2.06E-25 | 2.09E-24 |
| RP11-401P9.4 | ARHGAP24 | 0.563903 | 5.44E-49 | 3.20E-47 |
| RP11-4B16.3 | ARHGAP24 | 0.434155 | 1.64E-27 | 2.00E-26 |
| RP11-4B16.3 | ARHGAP29 | 0.445185 | 5.32E-29 | 7.34E-28 |
| RP11-401P9.4 | ARHGAP30 | 0.408662 | 2.83E-24 | 2.58E-23 |
| RP11-401P9.4 | ARHGAP31 | 0.605284 | 4.59E-58 | 5.07E-56 |
| RP11-4B16.3 | ARHGAP31 | 0.549648 | 3.76E-46 | 1.78E-44 |
| RP11-259K15.2 | ARHGAP44 | 0.432354 | 2.83E-27 | 3.37E-26 |
| RP11-401P9.4 | ARHGAP44 | 0.475424 | 2.26E-33 | 4.39E-32 |
| RP11-401P9.4 | ARHGAP6 | 0.700289 | 6.67E-85 | 7.80E-82 |
| RP11-4B16.3 | ARHGAP6 | 0.664784 | 1.03E-73 | 3.97E-71 |
| RP11-4B16.3 | ARHGEF10 | 0.42767 | 1.16E-26 | 1.31E-25 |
| RP11-401P9.4 | ARHGEF15 | 0.679501 | 3.69E-78 | 2.28E-75 |
| RP11-4B16.3 | ARHGEF15 | 0.671426 | 1.09E-75 | 5.15E-73 |
| RP11-401P9.4 | ARHGEF17 | 0.447345 | 2.68E-29 | 3.78E-28 |
| RP11-401P9.4 | ARHGEF26 | 0.532812 | 5.71E-43 | 2.20E-41 |
| RP11-4B16.3 | ARHGEF26 | 0.607353 | 1.49E-58 | 1.72E-56 |
| RP11-4B16.3 | ARHGEF3 | 0.412939 | 8.47E-25 | 8.15E-24 |
| RP11-401P9.4 | ARHGEF37 | 0.564118 | 4.92E-49 | 2.90E-47 |
| RP11-401P9.4 | ARHGEF6 | 0.501091 | 1.93E-37 | 5.04E-36 |
| RP11-4B16.3 | ARHGEF6 | 0.470137 | 1.42E-32 | 2.57E-31 |
| RP11-4B16.3 | ARL13B | 0.405577 | 6.69E-24 | 5.87E-23 |
| RP11-259K15.2 | ARRB1 | 0.411018 | 1.46E-24 | 1.37E-23 |
| RP11-401P9.4 | ARRB1 | 0.40608 | 5.82E-24 | 5.14E-23 |
| RP11-401P9.4 | ARRDC4 | 0.466106 | 5.61E-32 | 9.79E-31 |
| RP11-401P9.4 | ART4 | 0.563809 | 5.69E-49 | 3.33E-47 |
| RP11-4B16.3 | ART4 | 0.485001 | 7.54E-35 | 1.61E-33 |
| Z83851.4 | ASF1B | 0.410451 | 1.71E-24 | 1.59E-23 |
| RP11-401P9.4 | ASPA | 0.693558 | 1.18E-82 | 1.13E-79 |
| RP11-4B16.3 | ASPA | 0.634483 | 2.58E-65 | 5.09E-63 |
| Z83851.4 | ATAD3A | 0.418956 | 1.51E-25 | 1.54E-24 |
| Z83851.4 | ATIC | 0.520139 | 1.09E-40 | 3.52E-39 |
| RP11-259K15.2 | ATOH8 | 0.434458 | 1.49E-27 | 1.83E-26 |
| RP11-401P9.4 | ATOH8 | 0.429826 | 6.08E-27 | 7.02E-26 |
| RP11-259K15.2 | ATP13A4 | 0.509793 | 6.71E-39 | 1.93E-37 |
| RP11-401P9.4 | ATP13A4 | 0.410957 | 1.49E-24 | 1.39E-23 |
| RP11-401P9.4 | ATP1A2 | 0.653299 | 2.04E-70 | 5.70E-68 |
| RP11-4B16.3 | ATP1A2 | 0.51854 | 2.07E-40 | 6.60E-39 |
| RP11-401P9.4 | ATP1B2 | 0.573981 | 4.41E-51 | 3.00E-49 |
| RP11-4B16.3 | ATP1B2 | 0.400115 | 3.00E-23 | 2.48E-22 |
| RP11-259K15.2 | ATP8A1 | 0.46125 | 2.87E-31 | 4.75E-30 |
| RP11-401P9.4 | AVPR2 | 0.462726 | 1.75E-31 | 2.95E-30 |
| RP11-4B16.3 | AVPR2 | 0.460993 | 3.13E-31 | 5.15E-30 |
| RP11-401P9.4 | AXIN2 | 0.575102 | 2.55E-51 | 1.78E-49 |
| RP11-4B16.3 | B3GALNT1 | 0.405373 | 7.08E-24 | 6.20E-23 |
| Z83851.4 | B3GNT3 | 0.416468 | 3.09E-25 | 3.09E-24 |
| RP11-259K15.2 | B3GNT8 | 0.509733 | 6.87E-39 | 1.98E-37 |
| Z83851.4 | B4GALT2 | 0.416068 | 3.47E-25 | 3.44E-24 |
| Z83851.4 | BAIAP2L1 | 0.449262 | 1.45E-29 | 2.09E-28 |
| RP11-401P9.4 | BCHE | 0.629512 | 5.02E-64 | 8.80E-62 |
| RP11-4B16.3 | BCHE | 0.479342 | 5.70E-34 | 1.15E-32 |
| RP11-401P9.4 | BCL2L2 | 0.528835 | 3.03E-42 | 1.11E-40 |
| RP11-401P9.4 | BCL6B | 0.531055 | 1.20E-42 | 4.50E-41 |
| RP11-4B16.3 | BCL6B | 0.502411 | 1.17E-37 | 3.11E-36 |
| RP11-401P9.4 | BDNF | 0.437316 | 6.22E-28 | 7.95E-27 |
| RP11-4B16.3 | BDNF | 0.478119 | 8.79E-34 | 1.74E-32 |
| Z83851.4 | BIRC5 | 0.416278 | 3.26E-25 | 3.25E-24 |
| RP11-401P9.4 | BMP2 | 0.416644 | 2.94E-25 | 2.94E-24 |
| RP11-401P9.4 | BMP5 | 0.407458 | 3.97E-24 | 3.56E-23 |
| RP11-401P9.4 | BMPER | 0.444884 | 5.85E-29 | 8.04E-28 |
| RP11-4B16.3 | BMPER | 0.467463 | 3.54E-32 | 6.25E-31 |
| RP11-401P9.4 | BMPR2 | 0.505174 | 4.04E-38 | 1.11E-36 |
| RP11-4B16.3 | BMPR2 | 0.536407 | 1.24E-43 | 4.98E-42 |
| RP11-401P9.4 | BMX | 0.404604 | 8.76E-24 | 7.60E-23 |
| RP11-401P9.4 | BNIP2 | 0.486461 | 4.44E-35 | 9.75E-34 |
| RP11-4B16.3 | BNIP2 | 0.418687 | 1.63E-25 | 1.67E-24 |
| RP11-259K15.2 | BNIPL | 0.422525 | 5.32E-26 | 5.69E-25 |
| RP11-259K15.2 | BTG2 | 0.405361 | 7.10E-24 | 6.22E-23 |
| RP11-401P9.4 | BTG2 | 0.499441 | 3.62E-37 | 9.24E-36 |
| RP11-259K15.2 | BTNL9 | 0.435051 | 1.25E-27 | 1.54E-26 |
| RP11-401P9.4 | BTNL9 | 0.701028 | 3.75E-85 | 4.55E-82 |
| RP11-4B16.3 | BTNL9 | 0.693465 | 1.26E-82 | 1.14E-79 |
| Z83851.4 | BZW2 | 0.448967 | 1.60E-29 | 2.29E-28 |
| RP11-401P9.4 | C10orf128 | 0.423019 | 4.60E-26 | 4.94E-25 |
| RP11-4B16.3 | C10orf128 | 0.417659 | 2.19E-25 | 2.22E-24 |
| RP11-259K15.2 | C10orf32 | 0.488506 | 2.11E-35 | 4.74E-34 |
| RP11-401P9.4 | C10orf32 | 0.429281 | 7.16E-27 | 8.19E-26 |
| RP11-401P9.4 | C10orf54 | 0.479685 | 5.05E-34 | 1.02E-32 |
| RP11-4B16.3 | C10orf54 | 0.50871 | 1.03E-38 | 2.92E-37 |
| RP11-401P9.4 | C10orf67 | 0.599255 | 1.17E-56 | 1.19E-54 |
| RP11-4B16.3 | C10orf67 | 0.663593 | 2.30E-73 | 8.44E-71 |
| RP11-4B16.3 | C11orf21 | 0.427117 | 1.37E-26 | 1.53E-25 |
| RP11-401P9.4 | C11orf96 | 0.422698 | 5.06E-26 | 5.41E-25 |
| CTA-384D8.35 | C12orf45 | 0.436936 | 6.99E-28 | 8.89E-27 |
| RP11-401P9.4 | C15orf52 | 0.425461 | 2.24E-26 | 2.47E-25 |
| RP11-259K15.2 | C15orf59 | 0.421108 | 8.05E-26 | 8.46E-25 |
| RP11-401P9.4 | C15orf59 | 0.423533 | 3.96E-26 | 4.27E-25 |
| Z83851.4 | C16orf59 | 0.4205 | 9.62E-26 | 1.00E-24 |
| RP11-259K15.2 | C16orf89 | 0.658641 | 6.23E-72 | 1.91E-69 |
| CTA-384D8.35 | C17orf62 | 0.468604 | 2.39E-32 | 4.29E-31 |
| RP11-401P9.4 | C1orf115 | 0.475308 | 2.36E-33 | 4.56E-32 |
| RP11-259K15.2 | C1orf116 | 0.527742 | 4.79E-42 | 1.73E-40 |
| RP11-4B16.3 | C1orf162 | 0.446461 | 3.55E-29 | 4.95E-28 |
| RP11-401P9.4 | C1orf198 | 0.450894 | 8.59E-30 | 1.25E-28 |
| RP11-259K15.2 | C1orf21 | 0.432454 | 2.75E-27 | 3.28E-26 |
| RP11-401P9.4 | C1QTNF2 | 0.489592 | 1.42E-35 | 3.23E-34 |
| RP11-4B16.3 | C1QTNF2 | 0.449463 | 1.36E-29 | 1.96E-28 |
| RP5-1059L7.1 | C1QTNF6 | 0.461551 | 2.60E-31 | 4.31E-30 |
| Z83851.4 | C1QTNF6 | 0.459303 | 5.49E-31 | 8.82E-30 |
| RP11-259K15.2 | C1QTNF7 | 0.427202 | 1.33E-26 | 1.50E-25 |
| RP11-401P9.4 | C1QTNF7 | 0.732598 | 1.26E-96 | 5.67E-93 |
| RP11-4B16.3 | C1QTNF7 | 0.514194 | 1.18E-39 | 3.55E-38 |
| RP11-401P9.4 | C20orf194 | 0.619286 | 1.91E-61 | 2.77E-59 |
| RP11-4B16.3 | C20orf194 | 0.499959 | 2.97E-37 | 7.62E-36 |
| RP11-401P9.4 | C20orf202 | 0.527992 | 4.31E-42 | 1.56E-40 |
| RP11-4B16.3 | C20orf202 | 0.586603 | 8.32E-54 | 6.88E-52 |
| RP11-401P9.4 | C2orf40 | 0.546389 | 1.60E-45 | 7.22E-44 |
| RP11-4B16.3 | C2orf40 | 0.455942 | 1.66E-30 | 2.56E-29 |
| RP11-401P9.4 | C2orf91 | 0.504787 | 4.69E-38 | 1.28E-36 |
| RP11-4B16.3 | C2orf91 | 0.524999 | 1.49E-41 | 5.18E-40 |
| RP11-4B16.3 | C5AR1 | 0.403769 | 1.10E-23 | 9.48E-23 |
| RP11-259K15.2 | C5orf38 | 0.574346 | 3.69E-51 | 2.53E-49 |
| RP11-401P9.4 | C5orf38 | 0.401976 | 1.80E-23 | 1.52E-22 |
| RP11-401P9.4 | C7 | 0.548492 | 6.29E-46 | 2.93E-44 |
| RP11-401P9.4 | C8B | 0.45818 | 7.96E-31 | 1.26E-29 |
| CTA-384D8.35 | C8orf59 | 0.431901 | 3.25E-27 | 3.86E-26 |
| CTA-384D8.35 | C8orf76 | 0.429009 | 7.77E-27 | 8.87E-26 |
| RP11-259K15.2 | CA3 | 0.455057 | 2.22E-30 | 3.40E-29 |
| RP11-401P9.4 | CA3 | 0.516521 | 4.67E-40 | 1.45E-38 |
| RP11-259K15.2 | CA4 | 0.429869 | 6.00E-27 | 6.94E-26 |
| RP11-401P9.4 | CA4 | 0.619769 | 1.45E-61 | 2.13E-59 |
| RP11-4B16.3 | CA4 | 0.726898 | 1.95E-94 | 6.15E-91 |
| RP11-401P9.4 | CAB39L | 0.555308 | 2.91E-47 | 1.48E-45 |
| RP11-401P9.4 | CABLES1 | 0.47445 | 3.18E-33 | 6.08E-32 |
| RP11-401P9.4 | CACHD1 | 0.459968 | 4.40E-31 | 7.12E-30 |
| RP11-259K15.2 | CACNA2D2 | 0.568404 | 6.47E-50 | 4.09E-48 |
| RP11-401P9.4 | CACNA2D2 | 0.554273 | 4.66E-47 | 2.35E-45 |
| RP11-259K15.2 | CADM1 | 0.430333 | 5.22E-27 | 6.06E-26 |
| RP11-401P9.4 | CADM1 | 0.429546 | 6.61E-27 | 7.60E-26 |
| RP11-401P9.4 | CALCOCO1 | 0.615302 | 1.82E-60 | 2.46E-58 |
| RP11-4B16.3 | CALCOCO1 | 0.459439 | 5.25E-31 | 8.44E-30 |
| RP11-401P9.4 | CALCRL | 0.578708 | 4.34E-52 | 3.23E-50 |
| RP11-4B16.3 | CALCRL | 0.643869 | 8.16E-68 | 1.79E-65 |
| RP11-401P9.4 | CALM1 | 0.423187 | 4.38E-26 | 4.72E-25 |
| RP11-401P9.4 | CASKIN2 | 0.511089 | 4.03E-39 | 1.18E-37 |
| RP11-4B16.3 | CASKIN2 | 0.568755 | 5.47E-50 | 3.48E-48 |
| RP11-401P9.4 | CASQ2 | 0.562571 | 1.02E-48 | 5.88E-47 |
| RP11-4B16.3 | CASQ2 | 0.514336 | 1.12E-39 | 3.37E-38 |
| RP11-401P9.4 | CASS4 | 0.594221 | 1.65E-55 | 1.55E-53 |
| RP11-4B16.3 | CASS4 | 0.50974 | 6.85E-39 | 1.97E-37 |
| RP11-401P9.4 | CASZ1 | 0.542463 | 9.01E-45 | 3.91E-43 |
| RP11-259K15.2 | CAT | 0.513975 | 1.29E-39 | 3.86E-38 |
| RP11-401P9.4 | CAT | 0.511253 | 3.78E-39 | 1.11E-37 |
| RP11-4B16.3 | CAT | 0.462891 | 1.66E-31 | 2.80E-30 |
| RP11-401P9.4 | CAV1 | 0.503899 | 6.61E-38 | 1.78E-36 |
| RP11-4B16.3 | CAV1 | 0.601703 | 3.16E-57 | 3.34E-55 |
| RP11-4B16.3 | CAV2 | 0.513483 | 1.57E-39 | 4.68E-38 |
| RP11-401P9.4 | CBFA2T3 | 0.515476 | 7.09E-40 | 2.17E-38 |
| RP11-401P9.4 | CBX6 | 0.419057 | 1.46E-25 | 1.50E-24 |
| RP11-259K15.2 | CBX7 | 0.413254 | 7.75E-25 | 7.48E-24 |
| RP11-401P9.4 | CBX7 | 0.673355 | 2.85E-76 | 1.45E-73 |
| RP11-4B16.3 | CBX7 | 0.466836 | 4.38E-32 | 7.69E-31 |
| RP11-401P9.4 | CC2D2A | 0.401163 | 2.25E-23 | 1.88E-22 |
| RP11-4B16.3 | CCBE1 | 0.504864 | 4.56E-38 | 1.24E-36 |
| RP11-4B16.3 | CCDC102B | 0.400939 | 2.39E-23 | 2.00E-22 |
| Z83851.4 | CCDC137 | 0.454705 | 2.49E-30 | 3.80E-29 |
| RP11-401P9.4 | CCDC141 | 0.559357 | 4.53E-48 | 2.46E-46 |
| RP11-4B16.3 | CCDC141 | 0.574011 | 4.34E-51 | 2.96E-49 |
| RP11-259K15.2 | CCDC152 | 0.433737 | 1.86E-27 | 2.26E-26 |
| RP11-401P9.4 | CCDC152 | 0.485673 | 5.91E-35 | 1.28E-33 |
| RP11-4B16.3 | CCDC152 | 0.450811 | 8.82E-30 | 1.29E-28 |
| RP11-401P9.4 | CCDC50 | 0.452019 | 5.97E-30 | 8.84E-29 |
| RP11-4B16.3 | CCDC68 | 0.435792 | 9.93E-28 | 1.24E-26 |
| RP11-401P9.4 | CCDC69 | 0.459409 | 5.30E-31 | 8.52E-30 |
| RP11-4B16.3 | CCDC69 | 0.404654 | 8.64E-24 | 7.50E-23 |
| RP11-401P9.4 | CCDC8 | 0.416384 | 3.17E-25 | 3.16E-24 |
| RP11-401P9.4 | CCDC81 | 0.41546 | 4.13E-25 | 4.07E-24 |
| RP11-401P9.4 | CCDC85A | 0.534408 | 2.90E-43 | 1.14E-41 |
| RP11-4B16.3 | CCDC85A | 0.65266 | 3.09E-70 | 8.40E-68 |
| RP11-401P9.4 | CCL14 | 0.594731 | 1.26E-55 | 1.20E-53 |
| RP11-4B16.3 | CCL14 | 0.40401 | 1.03E-23 | 8.89E-23 |
| RP11-401P9.4 | CCL23 | 0.437943 | 5.12E-28 | 6.58E-27 |
| RP11-4B16.3 | CCL23 | 0.488362 | 2.22E-35 | 4.99E-34 |
| RP11-401P9.4 | CCM2L | 0.647809 | 6.85E-69 | 1.70E-66 |
| RP11-4B16.3 | CCM2L | 0.652139 | 4.32E-70 | 1.15E-67 |
| RP11-401P9.4 | CCND2 | 0.434718 | 1.38E-27 | 1.70E-26 |
| RP11-4B16.3 | CCRL2 | 0.463485 | 1.36E-31 | 2.30E-30 |
| RP11-401P9.4 | CD101 | 0.522435 | 4.27E-41 | 1.43E-39 |
| RP11-4B16.3 | CD101 | 0.486324 | 4.67E-35 | 1.02E-33 |
| RP11-259K15.2 | CD1C | 0.424325 | 3.13E-26 | 3.41E-25 |
| RP11-4B16.3 | CD300C | 0.459327 | 5.45E-31 | 8.76E-30 |
| RP11-4B16.3 | CD300LF | 0.422359 | 5.59E-26 | 5.96E-25 |
| RP11-401P9.4 | CD300LG | 0.6158 | 1.37E-60 | 1.89E-58 |
| RP11-4B16.3 | CD300LG | 0.733858 | 4.05E-97 | 2.13E-93 |
| RP11-259K15.2 | CD302 | 0.514347 | 1.11E-39 | 3.36E-38 |
| RP11-401P9.4 | CD302 | 0.473037 | 5.20E-33 | 9.80E-32 |
| RP11-401P9.4 | CD34 | 0.576437 | 1.33E-51 | 9.47E-50 |
| RP11-4B16.3 | CD34 | 0.481248 | 2.90E-34 | 5.98E-33 |
| RP11-259K15.2 | CD36 | 0.412556 | 9.45E-25 | 9.05E-24 |
| RP11-401P9.4 | CD36 | 0.586395 | 9.24E-54 | 7.58E-52 |
| RP11-4B16.3 | CD36 | 0.563147 | 7.76E-49 | 4.51E-47 |
| RP11-4B16.3 | CD52 | 0.409838 | 2.04E-24 | 1.88E-23 |
| RP11-401P9.4 | CD5L | 0.494162 | 2.62E-36 | 6.38E-35 |
| RP11-4B16.3 | CD5L | 0.500751 | 2.20E-37 | 5.72E-36 |
| RP11-259K15.2 | CD81 | 0.422707 | 5.04E-26 | 5.40E-25 |
| RP11-401P9.4 | CD81 | 0.42026 | 1.03E-25 | 1.07E-24 |
| RP11-401P9.4 | CD83 | 0.401381 | 2.12E-23 | 1.78E-22 |
| RP11-401P9.4 | CD93 | 0.561796 | 1.46E-48 | 8.26E-47 |
| RP11-4B16.3 | CD93 | 0.562071 | 1.28E-48 | 7.32E-47 |
| RP11-4B16.3 | CD97 | 0.403777 | 1.10E-23 | 9.47E-23 |
| RP11-401P9.4 | CDC14A | 0.428253 | 9.75E-27 | 1.10E-25 |
| Z83851.4 | CDC20 | 0.431785 | 3.37E-27 | 3.99E-26 |
| Z83851.4 | CDC45 | 0.437524 | 5.83E-28 | 7.48E-27 |
| Z83851.4 | CDC6 | 0.443266 | 9.75E-29 | 1.32E-27 |
| Z83851.4 | CDCA3 | 0.408949 | 2.61E-24 | 2.39E-23 |
| RP5-1059L7.1 | CDCA4 | 0.417473 | 2.31E-25 | 2.34E-24 |
| Z83851.4 | CDCA5 | 0.406725 | 4.86E-24 | 4.33E-23 |
| Z83851.4 | CDCA8 | 0.418758 | 1.60E-25 | 1.63E-24 |
| RP11-401P9.4 | CDH13 | 0.497102 | 8.74E-37 | 2.19E-35 |
| RP11-4B16.3 | CDH13 | 0.416093 | 3.44E-25 | 3.42E-24 |
| RP11-401P9.4 | CDH5 | 0.620752 | 8.25E-62 | 1.25E-59 |
| RP11-4B16.3 | CDH5 | 0.645794 | 2.44E-68 | 5.80E-66 |
| RP11-401P9.4 | CDKL5 | 0.441349 | 1.78E-28 | 2.37E-27 |
| RP11-401P9.4 | CDO1 | 0.629818 | 4.19E-64 | 7.42E-62 |
| RP11-4B16.3 | CDO1 | 0.543751 | 5.12E-45 | 2.25E-43 |
| RP11-401P9.4 | CELF2 | 0.441692 | 1.60E-28 | 2.14E-27 |
| Z83851.4 | CENPM | 0.521403 | 6.50E-41 | 2.13E-39 |
| RP11-4B16.3 | CETP | 0.445545 | 4.75E-29 | 6.57E-28 |
| RP11-259K15.2 | CFAP221 | 0.504587 | 5.07E-38 | 1.38E-36 |
| CTA-384D8.35 | CFB | 0.470331 | 1.32E-32 | 2.42E-31 |
| RP11-401P9.4 | CFD | 0.432478 | 2.73E-27 | 3.26E-26 |
| RP11-4B16.3 | CFD | 0.412239 | 1.03E-24 | 9.87E-24 |
| RP11-4B16.3 | CFL2 | 0.400521 | 2.68E-23 | 2.23E-22 |
| RP11-401P9.4 | CFP | 0.494573 | 2.25E-36 | 5.50E-35 |
| RP11-4B16.3 | CFP | 0.526861 | 6.90E-42 | 2.44E-40 |
| RP11-401P9.4 | CFTR | 0.430969 | 4.31E-27 | 5.05E-26 |
| RP11-259K15.2 | CGNL1 | 0.4454 | 4.97E-29 | 6.86E-28 |
| RP11-401P9.4 | CGNL1 | 0.675744 | 5.33E-77 | 2.90E-74 |
| RP11-4B16.3 | CGNL1 | 0.4983 | 5.57E-37 | 1.41E-35 |
| Z83851.4 | CHAF1B | 0.445304 | 5.12E-29 | 7.07E-28 |
| RP11-259K15.2 | CHIA | 0.410417 | 1.73E-24 | 1.61E-23 |
| RP11-401P9.4 | CHRDL1 | 0.605919 | 3.25E-58 | 3.65E-56 |
| RP11-4B16.3 | CHRDL1 | 0.442371 | 1.29E-28 | 1.74E-27 |
| RP11-401P9.4 | CHRM1 | 0.606891 | 1.92E-58 | 2.18E-56 |
| RP11-4B16.3 | CHRM1 | 0.77712 | ####### | ####### |
| RP11-401P9.4 | CIRBP | 0.455297 | 2.05E-30 | 3.15E-29 |
| RP11-259K15.2 | CITED2 | 0.423904 | 3.55E-26 | 3.85E-25 |
| RP11-401P9.4 | CLDN11 | 0.411858 | 1.15E-24 | 1.09E-23 |
| RP11-259K15.2 | CLDN18 | 0.447206 | 2.80E-29 | 3.94E-28 |
| RP11-401P9.4 | CLDN18 | 0.64052 | 6.51E-67 | 1.36E-64 |
| RP11-4B16.3 | CLDN18 | 0.54642 | 1.58E-45 | 7.13E-44 |
| RP11-401P9.4 | CLDN5 | 0.530471 | 1.53E-42 | 5.73E-41 |
| RP11-4B16.3 | CLDN5 | 0.572638 | 8.45E-51 | 5.61E-49 |
| RP11-4B16.3 | CLEC12A | 0.422604 | 5.20E-26 | 5.56E-25 |
| RP11-401P9.4 | CLEC14A | 0.639392 | 1.30E-66 | 2.67E-64 |
| RP11-4B16.3 | CLEC14A | 0.601097 | 4.37E-57 | 4.56E-55 |
| RP11-401P9.4 | CLEC1A | 0.596087 | 6.21E-56 | 6.03E-54 |
| RP11-4B16.3 | CLEC1A | 0.659065 | 4.71E-72 | 1.47E-69 |
| RP11-259K15.2 | CLEC3B | 0.478651 | 7.28E-34 | 1.45E-32 |
| RP11-401P9.4 | CLEC3B | 0.625706 | 4.70E-63 | 7.68E-61 |
| RP11-4B16.3 | CLEC3B | 0.628501 | 9.12E-64 | 1.56E-61 |
| RP11-259K15.2 | CLIC3 | 0.491048 | 8.31E-36 | 1.93E-34 |
| RP11-259K15.2 | CLIC5 | 0.475105 | 2.53E-33 | 4.89E-32 |
| RP11-401P9.4 | CLIC5 | 0.595336 | 9.21E-56 | 8.84E-54 |
| RP11-4B16.3 | CLIC5 | 0.566986 | 1.27E-49 | 7.81E-48 |
| RP11-401P9.4 | CMTM2 | 0.480909 | 3.27E-34 | 6.72E-33 |
| RP11-4B16.3 | CMTM2 | 0.509327 | 8.05E-39 | 2.31E-37 |
| RP11-401P9.4 | CNKSR2 | 0.524073 | 2.18E-41 | 7.47E-40 |
| RP11-401P9.4 | CNN1 | 0.488016 | 2.52E-35 | 5.65E-34 |
| CTA-384D8.35 | CNPY2 | 0.509145 | 8.65E-39 | 2.47E-37 |
| RP11-401P9.4 | CNRIP1 | 0.519402 | 1.46E-40 | 4.70E-39 |
| RP11-4B16.3 | CNRIP1 | 0.452966 | 4.39E-30 | 6.56E-29 |
| RP11-401P9.4 | CNTFR | 0.500123 | 2.79E-37 | 7.18E-36 |
| RP11-4B16.3 | CNTFR | 0.414873 | 4.88E-25 | 4.79E-24 |
| RP11-259K15.2 | CNTN6 | 0.427479 | 1.23E-26 | 1.38E-25 |
| RP11-401P9.4 | CNTN6 | 0.60991 | 3.66E-59 | 4.39E-57 |
| RP11-4B16.3 | CNTN6 | 0.614157 | 3.46E-60 | 4.53E-58 |
| CTA-384D8.35 | COA6 | 0.401935 | 1.82E-23 | 1.54E-22 |
| Z83851.4 | COASY | 0.428419 | 9.28E-27 | 1.05E-25 |
| RP11-401P9.4 | COBLL1 | 0.418546 | 1.70E-25 | 1.73E-24 |
| RP5-1059L7.1 | COL11A1 | 0.454511 | 2.66E-30 | 4.04E-29 |
| RP11-401P9.4 | COL13A1 | 0.494381 | 2.42E-36 | 5.90E-35 |
| RP5-1059L7.1 | COL1A1 | 0.428544 | 8.93E-27 | 1.02E-25 |
| RP5-1059L7.1 | COL3A1 | 0.402663 | 1.49E-23 | 1.27E-22 |
| RP11-259K15.2 | COL4A3 | 0.40295 | 1.38E-23 | 1.18E-22 |
| RP11-401P9.4 | COL4A3 | 0.463655 | 1.28E-31 | 2.18E-30 |
| RP11-401P9.4 | COL4A4 | 0.429453 | 6.80E-27 | 7.80E-26 |
| RP5-1059L7.1 | COL5A1 | 0.414261 | 5.82E-25 | 5.67E-24 |
| RP5-1059L7.1 | COL5A2 | 0.430688 | 4.69E-27 | 5.47E-26 |
| RP11-401P9.4 | COL6A5 | 0.502679 | 1.05E-37 | 2.81E-36 |
| RP11-401P9.4 | COL6A6 | 0.62392 | 1.33E-62 | 2.09E-60 |
| RP11-4B16.3 | COL6A6 | 0.595382 | 8.99E-56 | 8.68E-54 |
| RP11-401P9.4 | COLEC10 | 0.466225 | 5.38E-32 | 9.41E-31 |
| RP11-4B16.3 | COLEC10 | 0.515971 | 5.82E-40 | 1.79E-38 |
| RP11-401P9.4 | COLEC12 | 0.505621 | 3.40E-38 | 9.37E-37 |
| RP11-401P9.4 | COLGALT2 | 0.440445 | 2.36E-28 | 3.11E-27 |
| RP11-401P9.4 | CORO2B | 0.643377 | 1.11E-67 | 2.41E-65 |
| RP11-4B16.3 | CORO2B | 0.546468 | 1.55E-45 | 6.99E-44 |
| RP11-259K15.2 | COX4I2 | 0.42775 | 1.13E-26 | 1.28E-25 |
| RP11-401P9.4 | COX4I2 | 0.526592 | 7.71E-42 | 2.72E-40 |
| RP11-4B16.3 | COX4I2 | 0.56104 | 2.08E-48 | 1.17E-46 |
| RP11-401P9.4 | COX7A1 | 0.483153 | 1.47E-34 | 3.10E-33 |
| RP11-4B16.3 | COX7A1 | 0.471014 | 1.05E-32 | 1.93E-31 |
| RP11-401P9.4 | CPA3 | 0.442581 | 1.21E-28 | 1.63E-27 |
| RP11-259K15.2 | CPAMD8 | 0.411779 | 1.18E-24 | 1.12E-23 |
| RP11-401P9.4 | CPAMD8 | 0.460761 | 3.38E-31 | 5.53E-30 |
| RP11-259K15.2 | CPB2 | 0.410364 | 1.76E-24 | 1.63E-23 |
| RP11-401P9.4 | CPB2 | 0.507398 | 1.71E-38 | 4.82E-37 |
| RP11-4B16.3 | CPB2 | 0.450639 | 9.32E-30 | 1.36E-28 |
| RP11-401P9.4 | CPED1 | 0.589772 | 1.65E-54 | 1.47E-52 |
| RP11-4B16.3 | CPED1 | 0.519451 | 1.44E-40 | 4.61E-39 |
| RP11-259K15.2 | CPQ | 0.412026 | 1.10E-24 | 1.04E-23 |
| RP11-259K15.2 | CREBRF | 0.409161 | 2.46E-24 | 2.25E-23 |
| RP11-401P9.4 | CREBRF | 0.488914 | 1.82E-35 | 4.10E-34 |
| CTA-384D8.35 | CRELD2 | 0.434258 | 1.59E-27 | 1.94E-26 |
| RP11-401P9.4 | CRIM1 | 0.465611 | 6.63E-32 | 1.15E-30 |
| RP11-401P9.4 | CRTAC1 | 0.588067 | 3.95E-54 | 3.35E-52 |
| RP11-4B16.3 | CRTAC1 | 0.457229 | 1.09E-30 | 1.71E-29 |
| RP11-259K15.2 | CRY2 | 0.502511 | 1.12E-37 | 2.99E-36 |
| RP11-401P9.4 | CRY2 | 0.549418 | 4.16E-46 | 1.96E-44 |
| RP11-401P9.4 | CRYAB | 0.513467 | 1.58E-39 | 4.71E-38 |
| RP11-4B16.3 | CRYAB | 0.454275 | 2.87E-30 | 4.34E-29 |
| RP11-4B16.3 | CSF3 | 0.423968 | 3.48E-26 | 3.78E-25 |
| RP11-401P9.4 | CSRNP1 | 0.48405 | 1.06E-34 | 2.26E-33 |
| RP11-4B16.3 | CSRNP1 | 0.524524 | 1.81E-41 | 6.27E-40 |
| RP11-401P9.4 | CSRP1 | 0.473372 | 4.63E-33 | 8.78E-32 |
| RP11-259K15.2 | CST5 | 0.493828 | 2.97E-36 | 7.18E-35 |
| RP11-401P9.4 | CST5 | 0.449614 | 1.30E-29 | 1.87E-28 |
| RP11-401P9.4 | CTDSPL | 0.538315 | 5.45E-44 | 2.24E-42 |
| RP11-401P9.4 | CTGF | 0.444916 | 5.79E-29 | 7.97E-28 |
| RP5-1059L7.1 | CTHRC1 | 0.489162 | 1.66E-35 | 3.75E-34 |
| RP11-401P9.4 | CTIF | 0.604593 | 6.67E-58 | 7.32E-56 |
| RP11-4B16.3 | CTIF | 0.409332 | 2.35E-24 | 2.15E-23 |
| RP11-4B16.3 | CTNNAL1 | 0.491966 | 5.92E-36 | 1.40E-34 |
| RP11-259K15.2 | CTSH | 0.555156 | 3.12E-47 | 1.59E-45 |
| RP11-401P9.4 | CX3CR1 | 0.449991 | 1.15E-29 | 1.66E-28 |
| RP11-401P9.4 | CXCR2 | 0.457105 | 1.13E-30 | 1.78E-29 |
| RP11-4B16.3 | CXCR2 | 0.434666 | 1.40E-27 | 1.73E-26 |
| RP11-401P9.4 | CXorf36 | 0.543728 | 5.18E-45 | 2.27E-43 |
| RP11-4B16.3 | CXorf36 | 0.435856 | 9.74E-28 | 1.22E-26 |
| RP11-4B16.3 | CXorf57 | 0.401403 | 2.11E-23 | 1.77E-22 |
| RP11-259K15.2 | CYB5A | 0.543708 | 5.22E-45 | 2.29E-43 |
| RP11-401P9.4 | CYB5R3 | 0.405433 | 6.96E-24 | 6.11E-23 |
| RP11-259K15.2 | CYBRD1 | 0.412206 | 1.04E-24 | 9.95E-24 |
| RP11-401P9.4 | CYBRD1 | 0.491201 | 7.85E-36 | 1.83E-34 |
| RP11-401P9.4 | CYGB | 0.497756 | 6.83E-37 | 1.72E-35 |
| RP11-4B16.3 | CYP1A2 | 0.463494 | 1.35E-31 | 2.30E-30 |
| RP11-401P9.4 | CYP27A1 | 0.431551 | 3.61E-27 | 4.27E-26 |
| RP5-1059L7.1 | CYP27C1 | 0.414938 | 4.79E-25 | 4.70E-24 |
| RP11-401P9.4 | CYP2U1 | 0.413924 | 6.40E-25 | 6.22E-24 |
| RP11-259K15.2 | CYP4B1 | 0.585917 | 1.18E-53 | 9.51E-52 |
| RP11-401P9.4 | CYP4B1 | 0.50478 | 4.71E-38 | 1.28E-36 |
| RP11-4B16.3 | CYP4B1 | 0.416912 | 2.72E-25 | 2.72E-24 |
| RP11-259K15.2 | CYP4V2 | 0.40486 | 8.16E-24 | 7.10E-23 |
| RP11-401P9.4 | CYS1 | 0.407149 | 4.32E-24 | 3.87E-23 |
| RP11-401P9.4 | CYYR1 | 0.61663 | 8.61E-61 | 1.20E-58 |
| RP11-4B16.3 | CYYR1 | 0.576631 | 1.21E-51 | 8.63E-50 |
| RP11-259K15.2 | DAAM2 | 0.406944 | 4.58E-24 | 4.08E-23 |
| RP11-401P9.4 | DAB2 | 0.410551 | 1.67E-24 | 1.55E-23 |
| RP11-401P9.4 | DAB2IP | 0.468374 | 2.59E-32 | 4.62E-31 |
| RP11-401P9.4 | DACH1 | 0.596307 | 5.53E-56 | 5.39E-54 |
| RP11-4B16.3 | DACH1 | 0.558831 | 5.78E-48 | 3.11E-46 |
| RP11-401P9.4 | DACT3 | 0.487016 | 3.63E-35 | 8.02E-34 |
| Z83851.4 | DAK | 0.424879 | 2.66E-26 | 2.91E-25 |
| RP11-401P9.4 | DAPK1 | 0.431175 | 4.05E-27 | 4.76E-26 |
| RP11-259K15.2 | DAPK2 | 0.588329 | 3.45E-54 | 2.95E-52 |
| RP11-401P9.4 | DAPK2 | 0.530002 | 1.86E-42 | 6.93E-41 |
| RP11-4B16.3 | DAPK2 | 0.472397 | 6.49E-33 | 1.22E-31 |
| RP11-401P9.4 | DCC | 0.580944 | 1.43E-52 | 1.09E-50 |
| RP11-4B16.3 | DCC | 0.560298 | 2.93E-48 | 1.63E-46 |
| RP11-401P9.4 | DCHS1 | 0.430423 | 5.08E-27 | 5.90E-26 |
| RP11-401P9.4 | DCN | 0.409333 | 2.35E-24 | 2.15E-23 |
| RP11-4B16.3 | DCSTAMP | 0.41599 | 3.55E-25 | 3.52E-24 |
| RP11-401P9.4 | DDR2 | 0.479262 | 5.86E-34 | 1.18E-32 |
| Z83851.4 | DDX56 | 0.503487 | 7.74E-38 | 2.08E-36 |
| RP11-401P9.4 | DENND2A | 0.574142 | 4.08E-51 | 2.79E-49 |
| RP11-4B16.3 | DENND2A | 0.490922 | 8.70E-36 | 2.01E-34 |
| RP11-401P9.4 | DENND3 | 0.536037 | 1.45E-43 | 5.80E-42 |
| RP11-4B16.3 | DENND3 | 0.577265 | 8.85E-52 | 6.39E-50 |
| RP11-401P9.4 | DES | 0.617653 | 4.82E-61 | 6.86E-59 |
| RP11-4B16.3 | DES | 0.527753 | 4.76E-42 | 1.72E-40 |
| RP11-259K15.2 | DHCR24 | 0.405166 | 7.50E-24 | 6.54E-23 |
| RP11-401P9.4 | DIAPH2 | 0.419461 | 1.30E-25 | 1.34E-24 |
| RP11-401P9.4 | DISP1 | 0.485225 | 6.95E-35 | 1.49E-33 |
| RP11-401P9.4 | DIXDC1 | 0.576246 | 1.46E-51 | 1.03E-49 |
| RP11-4B16.3 | DIXDC1 | 0.426618 | 1.59E-26 | 1.77E-25 |
| RP11-401P9.4 | DKK2 | 0.524174 | 2.09E-41 | 7.19E-40 |
| RP11-4B16.3 | DKK2 | 0.56033 | 2.89E-48 | 1.62E-46 |
| RP11-401P9.4 | DKK3 | 0.495444 | 1.63E-36 | 4.00E-35 |
| RP11-259K15.2 | DLC1 | 0.528282 | 3.82E-42 | 1.39E-40 |
| RP11-401P9.4 | DLC1 | 0.551136 | 1.93E-46 | 9.36E-45 |
| RP11-4B16.3 | DLC1 | 0.457691 | 9.35E-31 | 1.48E-29 |
| RP11-401P9.4 | DNASE1L3 | 0.530272 | 1.66E-42 | 6.22E-41 |
| RP11-4B16.3 | DNASE1L3 | 0.467654 | 3.31E-32 | 5.87E-31 |
| RP11-401P9.4 | DNASE2B | 0.447769 | 2.34E-29 | 3.32E-28 |
| RP11-4B16.3 | DNASE2B | 0.470722 | 1.16E-32 | 2.13E-31 |
| RP11-4B16.3 | DOCK11 | 0.403392 | 1.22E-23 | 1.05E-22 |
| RP11-401P9.4 | DOCK4 | 0.534087 | 3.32E-43 | 1.30E-41 |
| RP11-4B16.3 | DOCK4 | 0.517299 | 3.42E-40 | 1.06E-38 |
| RP11-4B16.3 | DOK2 | 0.444802 | 6.01E-29 | 8.25E-28 |
| RP11-259K15.2 | DPEP2 | 0.406584 | 5.06E-24 | 4.49E-23 |
| RP11-401P9.4 | DPEP2 | 0.583596 | 3.80E-53 | 2.96E-51 |
| RP11-4B16.3 | DPEP2 | 0.541371 | 1.45E-44 | 6.20E-43 |
| RP11-259K15.2 | DPYSL2 | 0.446615 | 3.38E-29 | 4.72E-28 |
| RP11-401P9.4 | DPYSL2 | 0.544291 | 4.04E-45 | 1.78E-43 |
| RP11-4B16.3 | DPYSL2 | 0.438299 | 4.59E-28 | 5.91E-27 |
| RP11-259K15.2 | DRAM1 | 0.489671 | 1.38E-35 | 3.14E-34 |
| RP11-401P9.4 | DST | 0.411198 | 1.39E-24 | 1.30E-23 |
| Z83851.4 | DTX2 | 0.405779 | 6.32E-24 | 5.56E-23 |
| RP11-259K15.2 | DUOX1 | 0.506094 | 2.83E-38 | 7.87E-37 |
| RP11-401P9.4 | DUOX1 | 0.585582 | 1.40E-53 | 1.12E-51 |
| RP11-4B16.3 | DUOX1 | 0.491466 | 7.12E-36 | 1.67E-34 |
| RP11-259K15.2 | DUOXA1 | 0.537764 | 6.90E-44 | 2.81E-42 |
| RP11-401P9.4 | DUOXA1 | 0.538126 | 5.91E-44 | 2.42E-42 |
| RP11-4B16.3 | DUOXA1 | 0.430087 | 5.62E-27 | 6.51E-26 |
| CTA-384D8.35 | DUS1L | 0.402894 | 1.40E-23 | 1.19E-22 |
| RP11-401P9.4 | DYNC2H1 | 0.40231 | 1.65E-23 | 1.39E-22 |
| RP11-401P9.4 | ECM2 | 0.45916 | 5.76E-31 | 9.24E-30 |
| RP11-401P9.4 | ECSCR | 0.562012 | 1.32E-48 | 7.51E-47 |
| RP11-4B16.3 | ECSCR | 0.604032 | 9.04E-58 | 9.80E-56 |
| RP11-4B16.3 | EDN1 | 0.431016 | 4.25E-27 | 4.98E-26 |
| RP11-259K15.2 | EDNRB | 0.419617 | 1.24E-25 | 1.28E-24 |
| RP11-401P9.4 | EDNRB | 0.64796 | 6.23E-69 | 1.56E-66 |
| RP11-4B16.3 | EDNRB | 0.666731 | 2.75E-74 | 1.13E-71 |
| Z83851.4 | EEF2KMT | 0.417789 | 2.11E-25 | 2.14E-24 |
| RP11-259K15.2 | EFCC1 | 0.52707 | 6.33E-42 | 2.25E-40 |
| RP11-401P9.4 | EFCC1 | 0.725132 | 9.06E-94 | 2.60E-90 |
| RP11-4B16.3 | EFCC1 | 0.613043 | 6.44E-60 | 8.30E-58 |
| RP11-4B16.3 | EFNB2 | 0.408117 | 3.30E-24 | 2.98E-23 |
| RP11-401P9.4 | EGR1 | 0.412052 | 1.09E-24 | 1.04E-23 |
| RP11-401P9.4 | EGR2 | 0.452982 | 4.37E-30 | 6.53E-29 |
| Z83851.4 | EIF2AK1 | 0.421448 | 7.29E-26 | 7.69E-25 |
| Z83851.4 | EIF3B | 0.441238 | 1.84E-28 | 2.45E-27 |
| RP11-401P9.4 | EIF4E3 | 0.407259 | 4.19E-24 | 3.75E-23 |
| RP11-401P9.4 | ELMO1 | 0.418182 | 1.89E-25 | 1.92E-24 |
| RP11-401P9.4 | ELN | 0.534532 | 2.75E-43 | 1.08E-41 |
| RP11-259K15.2 | EMCN | 0.458223 | 7.85E-31 | 1.25E-29 |
| RP11-401P9.4 | EMCN | 0.598718 | 1.55E-56 | 1.56E-54 |
| RP11-4B16.3 | EMCN | 0.613673 | 4.53E-60 | 5.86E-58 |
| RP11-401P9.4 | EML1 | 0.538705 | 4.60E-44 | 1.90E-42 |
| RP11-4B16.3 | EML1 | 0.468008 | 2.94E-32 | 5.22E-31 |
| RP11-259K15.2 | EMP2 | 0.49691 | 9.39E-37 | 2.35E-35 |
| RP11-401P9.4 | EMP2 | 0.589803 | 1.62E-54 | 1.45E-52 |
| RP11-4B16.3 | EMP2 | 0.534856 | 2.40E-43 | 9.48E-42 |
| RP11-401P9.4 | EMR1 | 0.43151 | 3.66E-27 | 4.32E-26 |
| RP11-4B16.3 | EMR1 | 0.505121 | 4.13E-38 | 1.13E-36 |
| RP11-401P9.4 | EMR3 | 0.493331 | 3.57E-36 | 8.59E-35 |
| RP11-4B16.3 | EMR3 | 0.48286 | 1.63E-34 | 3.42E-33 |
| RP11-401P9.4 | ENG | 0.494631 | 2.20E-36 | 5.39E-35 |
| RP11-4B16.3 | ENG | 0.517895 | 2.69E-40 | 8.51E-39 |
| Z83851.4 | ENO1 | 0.403604 | 1.15E-23 | 9.90E-23 |
| RP11-401P9.4 | EOGT | 0.438878 | 3.84E-28 | 4.98E-27 |
| RP11-4B16.3 | EOGT | 0.462112 | 2.15E-31 | 3.60E-30 |
| RP11-401P9.4 | EPAS1 | 0.570566 | 2.30E-50 | 1.49E-48 |
| RP11-4B16.3 | EPAS1 | 0.610198 | 3.12E-59 | 3.80E-57 |
| RP11-401P9.4 | EPB41L2 | 0.514537 | 1.03E-39 | 3.12E-38 |
| RP11-4B16.3 | EPB41L2 | 0.478315 | 8.20E-34 | 1.63E-32 |
| RP11-259K15.2 | EPHX2 | 0.470686 | 1.17E-32 | 2.15E-31 |
| RP11-401P9.4 | EPM2A | 0.549528 | 3.96E-46 | 1.87E-44 |
| RP11-4B16.3 | EPM2A | 0.441498 | 1.70E-28 | 2.27E-27 |
| Z83851.4 | EPN3 | 0.450181 | 1.08E-29 | 1.57E-28 |
| RP11-401P9.4 | ERBB4 | 0.445626 | 4.63E-29 | 6.41E-28 |
| RP11-401P9.4 | ERG | 0.670958 | 1.51E-75 | 6.91E-73 |
| RP11-4B16.3 | ERG | 0.586374 | 9.34E-54 | 7.64E-52 |
| RP11-259K15.2 | ERICH2 | 0.480567 | 3.69E-34 | 7.55E-33 |
| RP5-1059L7.1 | ERO1L | 0.456717 | 1.29E-30 | 2.02E-29 |
| Z83851.4 | ERO1L | 0.408971 | 2.60E-24 | 2.37E-23 |
| RP11-259K15.2 | ESAM | 0.435385 | 1.13E-27 | 1.40E-26 |
| RP11-401P9.4 | ESAM | 0.517771 | 2.83E-40 | 8.92E-39 |
| RP11-4B16.3 | ESAM | 0.501666 | 1.55E-37 | 4.09E-36 |
| RP11-259K15.2 | ESYT3 | 0.521698 | 5.76E-41 | 1.91E-39 |
| RP11-401P9.4 | ESYT3 | 0.403765 | 1.10E-23 | 9.49E-23 |
| RP11-4B16.3 | ETS1 | 0.430749 | 4.61E-27 | 5.37E-26 |
| RP11-401P9.4 | ETV1 | 0.403864 | 1.07E-23 | 9.25E-23 |
| RP11-401P9.4 | ETV5 | 0.447956 | 2.20E-29 | 3.13E-28 |
| RP11-401P9.4 | EXOC3L1 | 0.470154 | 1.41E-32 | 2.56E-31 |
| RP11-4B16.3 | EXOC3L1 | 0.553218 | 7.53E-47 | 3.74E-45 |
| CTA-384D8.35 | EXOSC1 | 0.420069 | 1.09E-25 | 1.13E-24 |
| RP11-401P9.4 | F10 | 0.502161 | 1.29E-37 | 3.41E-36 |
| RP11-259K15.2 | F11 | 0.493992 | 2.79E-36 | 6.78E-35 |
| RP11-401P9.4 | F11 | 0.563188 | 7.62E-49 | 4.44E-47 |
| RP11-4B16.3 | F11 | 0.487704 | 2.83E-35 | 6.30E-34 |
| RP11-4B16.3 | F2RL3 | 0.436924 | 7.01E-28 | 8.92E-27 |
| RP11-259K15.2 | F8 | 0.408758 | 2.76E-24 | 2.51E-23 |
| RP11-401P9.4 | F8 | 0.500731 | 2.22E-37 | 5.76E-36 |
| RP11-4B16.3 | F8 | 0.471434 | 9.06E-33 | 1.68E-31 |
| RP11-401P9.4 | FABP4 | 0.597227 | 3.41E-56 | 3.41E-54 |
| RP11-4B16.3 | FABP4 | 0.588126 | 3.83E-54 | 3.26E-52 |
| RP11-401P9.4 | FAM101B | 0.458723 | 6.65E-31 | 1.06E-29 |
| RP11-401P9.4 | FAM107A | 0.646603 | 1.47E-68 | 3.56E-66 |
| RP11-4B16.3 | FAM107A | 0.648801 | 3.65E-69 | 9.22E-67 |
| RP11-401P9.4 | FAM110D | 0.585775 | 1.27E-53 | 1.02E-51 |
| RP11-4B16.3 | FAM110D | 0.524294 | 1.99E-41 | 6.86E-40 |
| RP11-401P9.4 | FAM124B | 0.478839 | 6.81E-34 | 1.36E-32 |
| RP11-4B16.3 | FAM124B | 0.577994 | 6.18E-52 | 4.53E-50 |
| Z83851.4 | FAM136A | 0.40744 | 3.98E-24 | 3.58E-23 |
| RP11-401P9.4 | FAM13B | 0.492727 | 4.47E-36 | 1.07E-34 |
| RP11-401P9.4 | FAM13C | 0.616747 | 8.06E-61 | 1.13E-58 |
| RP11-4B16.3 | FAM13C | 0.501119 | 1.91E-37 | 4.99E-36 |
| RP11-401P9.4 | FAM150B | 0.562472 | 1.06E-48 | 6.13E-47 |
| RP11-4B16.3 | FAM150B | 0.534354 | 2.97E-43 | 1.16E-41 |
| RP11-259K15.2 | FAM162B | 0.431764 | 3.39E-27 | 4.01E-26 |
| RP11-401P9.4 | FAM162B | 0.572779 | 7.90E-51 | 5.26E-49 |
| RP11-4B16.3 | FAM162B | 0.574683 | 3.13E-51 | 2.15E-49 |
| RP11-401P9.4 | FAM167A | 0.487177 | 3.43E-35 | 7.58E-34 |
| RP11-4B16.3 | FAM167A | 0.413655 | 6.91E-25 | 6.69E-24 |
| RP11-401P9.4 | FAM167B | 0.407181 | 4.28E-24 | 3.83E-23 |
| RP11-4B16.3 | FAM167B | 0.448147 | 2.07E-29 | 2.95E-28 |
| RP11-401P9.4 | FAM171A1 | 0.404404 | 9.25E-24 | 8.01E-23 |
| RP11-401P9.4 | FAM180A | 0.439714 | 2.96E-28 | 3.87E-27 |
| RP11-259K15.2 | FAM184A | 0.558328 | 7.29E-48 | 3.89E-46 |
| RP11-401P9.4 | FAM189A1 | 0.42793 | 1.07E-26 | 1.21E-25 |
| RP11-4B16.3 | FAM189A1 | 0.428957 | 7.89E-27 | 9.00E-26 |
| RP11-259K15.2 | FAM189A2 | 0.482515 | 1.84E-34 | 3.85E-33 |
| RP11-401P9.4 | FAM189A2 | 0.605811 | 3.45E-58 | 3.85E-56 |
| RP11-4B16.3 | FAM189A2 | 0.536859 | 1.02E-43 | 4.13E-42 |
| RP11-401P9.4 | FAM212A | 0.434424 | 1.51E-27 | 1.85E-26 |
| RP11-4B16.3 | FAM212A | 0.420569 | 9.43E-26 | 9.84E-25 |
| RP11-401P9.4 | FAM212B | 0.549284 | 4.42E-46 | 2.08E-44 |
| RP11-4B16.3 | FAM212B | 0.464203 | 1.07E-31 | 1.82E-30 |
| RP11-401P9.4 | FAM43A | 0.409976 | 1.96E-24 | 1.81E-23 |
| RP11-401P9.4 | FAM46B | 0.461767 | 2.42E-31 | 4.02E-30 |
| RP11-4B16.3 | FAM46B | 0.519678 | 1.31E-40 | 4.22E-39 |
| RP11-401P9.4 | FAM49A | 0.437175 | 6.49E-28 | 8.28E-27 |
| RP11-401P9.4 | FAM53B | 0.46706 | 4.06E-32 | 7.14E-31 |
| RP11-401P9.4 | FAM63B | 0.446471 | 3.54E-29 | 4.94E-28 |
| RP11-4B16.3 | FAM63B | 0.415985 | 3.55E-25 | 3.52E-24 |
| RP11-401P9.4 | FAM65A | 0.498005 | 6.22E-37 | 1.57E-35 |
| RP11-4B16.3 | FAM65A | 0.45428 | 2.86E-30 | 4.34E-29 |
| CTA-384D8.35 | FAM83A | 0.451933 | 6.14E-30 | 9.06E-29 |
| Z83851.4 | FAM86C1 | 0.485055 | 7.39E-35 | 1.58E-33 |
| Z83851.4 | FARSB | 0.414405 | 5.58E-25 | 5.45E-24 |
| RP11-401P9.4 | FAT4 | 0.546788 | 1.34E-45 | 6.09E-44 |
| RP11-4B16.3 | FAT4 | 0.408732 | 2.78E-24 | 2.53E-23 |
| RP11-401P9.4 | FBLN1 | 0.416027 | 3.51E-25 | 3.48E-24 |
| RP11-401P9.4 | FBLN5 | 0.589481 | 1.91E-54 | 1.69E-52 |
| RP11-4B16.3 | FBLN5 | 0.4058 | 6.29E-24 | 5.53E-23 |
| RP11-259K15.2 | FBP1 | 0.509329 | 8.05E-39 | 2.31E-37 |
| RP11-401P9.4 | FBXL7 | 0.440295 | 2.47E-28 | 3.25E-27 |
| RP11-259K15.2 | FCER1A | 0.463335 | 1.43E-31 | 2.42E-30 |
| RP11-259K15.2 | FCGRT | 0.474236 | 3.43E-33 | 6.54E-32 |
| RP11-401P9.4 | FCN3 | 0.506266 | 2.65E-38 | 7.39E-37 |
| RP11-4B16.3 | FCN3 | 0.692567 | 2.49E-82 | 2.12E-79 |
| RP11-401P9.4 | FERMT2 | 0.411419 | 1.30E-24 | 1.23E-23 |
| RP11-4B16.3 | FERMT2 | 0.43929 | 3.38E-28 | 4.40E-27 |
| RP11-401P9.4 | FEZ1 | 0.625366 | 5.73E-63 | 9.32E-61 |
| RP11-4B16.3 | FEZ1 | 0.555437 | 2.74E-47 | 1.40E-45 |
| RP11-401P9.4 | FFAR4 | 0.4804 | 3.92E-34 | 7.99E-33 |
| RP11-4B16.3 | FFAR4 | 0.454428 | 2.73E-30 | 4.15E-29 |
| RP11-401P9.4 | FGD3 | 0.423519 | 3.97E-26 | 4.29E-25 |
| RP11-401P9.4 | FGD4 | 0.411874 | 1.15E-24 | 1.09E-23 |
| RP11-401P9.4 | FGD5 | 0.655284 | 5.63E-71 | 1.68E-68 |
| RP11-4B16.3 | FGD5 | 0.617765 | 4.53E-61 | 6.47E-59 |
| RP11-401P9.4 | FGF10 | 0.56251 | 1.05E-48 | 6.04E-47 |
| RP11-4B16.3 | FGF10 | 0.465878 | 6.06E-32 | 1.05E-30 |
| RP11-401P9.4 | FGF2 | 0.527565 | 5.15E-42 | 1.85E-40 |
| RP11-4B16.3 | FGF2 | 0.48281 | 1.66E-34 | 3.48E-33 |
| RP11-401P9.4 | FGFBP2 | 0.520166 | 1.07E-40 | 3.48E-39 |
| RP11-4B16.3 | FGFBP2 | 0.634629 | 2.36E-65 | 4.72E-63 |
| RP11-401P9.4 | FGFR2 | 0.508411 | 1.15E-38 | 3.27E-37 |
| RP11-4B16.3 | FGFR2 | 0.400246 | 2.89E-23 | 2.40E-22 |
| RP11-401P9.4 | FGFR4 | 0.554165 | 4.90E-47 | 2.46E-45 |
| RP11-4B16.3 | FGFR4 | 0.489866 | 1.28E-35 | 2.93E-34 |
| RP11-401P9.4 | FGR | 0.439165 | 3.51E-28 | 4.57E-27 |
| RP11-4B16.3 | FGR | 0.497696 | 6.99E-37 | 1.76E-35 |
| RP11-401P9.4 | FHDC1 | 0.410377 | 1.75E-24 | 1.63E-23 |
| RP11-401P9.4 | FHL1 | 0.696685 | 1.08E-83 | 1.18E-80 |
| RP11-4B16.3 | FHL1 | 0.60625 | 2.72E-58 | 3.06E-56 |
| RP5-1059L7.1 | FHL2 | 0.421335 | 7.54E-26 | 7.94E-25 |
| RP11-259K15.2 | FHL5 | 0.412715 | 9.03E-25 | 8.68E-24 |
| RP11-401P9.4 | FHL5 | 0.641166 | 4.37E-67 | 9.19E-65 |
| RP11-4B16.3 | FHL5 | 0.629659 | 4.60E-64 | 8.11E-62 |
| RP11-401P9.4 | FIBIN | 0.477828 | 9.74E-34 | 1.92E-32 |
| RP11-4B16.3 | FIBIN | 0.420925 | 8.50E-26 | 8.90E-25 |
| RP11-259K15.2 | FIGF | 0.490562 | 9.94E-36 | 2.29E-34 |
| RP11-401P9.4 | FIGF | 0.696542 | 1.21E-83 | 1.27E-80 |
| RP11-4B16.3 | FIGF | 0.60452 | 6.94E-58 | 7.59E-56 |
| RP11-401P9.4 | FILIP1 | 0.434584 | 1.44E-27 | 1.77E-26 |
| CTA-384D8.35 | FKBP11 | 0.482201 | 2.06E-34 | 4.30E-33 |
| CTA-384D8.35 | FLAD1 | 0.451984 | 6.04E-30 | 8.92E-29 |
| RP11-401P9.4 | FLI1 | 0.553164 | 7.71E-47 | 3.82E-45 |
| RP11-4B16.3 | FLI1 | 0.517304 | 3.41E-40 | 1.06E-38 |
| RP11-401P9.4 | FLRT3 | 0.404209 | 9.76E-24 | 8.43E-23 |
| RP11-401P9.4 | FLT4 | 0.468591 | 2.41E-32 | 4.30E-31 |
| RP11-4B16.3 | FLT4 | 0.489572 | 1.43E-35 | 3.25E-34 |
| RP11-401P9.4 | FMO2 | 0.661335 | 1.04E-72 | 3.54E-70 |
| RP11-4B16.3 | FMO2 | 0.566159 | 1.88E-49 | 1.15E-47 |
| RP11-401P9.4 | FMO3 | 0.451073 | 8.11E-30 | 1.19E-28 |
| RP11-259K15.2 | FOLR1 | 0.535324 | 1.96E-43 | 7.82E-42 |
| RP11-401P9.4 | FOS | 0.429526 | 6.66E-27 | 7.64E-26 |
| RP11-401P9.4 | FOSB | 0.458714 | 6.67E-31 | 1.06E-29 |
| RP11-4B16.3 | FOSB | 0.411442 | 1.30E-24 | 1.22E-23 |
| RP11-259K15.2 | FOXA2 | 0.432677 | 2.57E-27 | 3.08E-26 |
| RP11-401P9.4 | FOXF1 | 0.703136 | 7.16E-86 | 1.02E-82 |
| RP11-4B16.3 | FOXF1 | 0.694593 | 5.36E-83 | 5.46E-80 |
| RP11-401P9.4 | FOXF2 | 0.557785 | 9.35E-48 | 4.95E-46 |
| Z83851.4 | FOXM1 | 0.435377 | 1.13E-27 | 1.40E-26 |
| RP11-401P9.4 | FOXN3 | 0.448961 | 1.60E-29 | 2.29E-28 |
| RP11-401P9.4 | FOXO1 | 0.405629 | 6.59E-24 | 5.79E-23 |
| RP11-401P9.4 | FOXO3 | 0.42063 | 9.26E-26 | 9.68E-25 |
| RP11-401P9.4 | FOXO4 | 0.490242 | 1.12E-35 | 2.56E-34 |
| RP11-401P9.4 | FOXP1 | 0.422023 | 6.16E-26 | 6.55E-25 |
| RP11-4B16.3 | FPR2 | 0.472406 | 6.47E-33 | 1.21E-31 |
| RP11-401P9.4 | FRAS1 | 0.499633 | 3.36E-37 | 8.61E-36 |
| RP11-401P9.4 | FREM2 | 0.422784 | 4.93E-26 | 5.29E-25 |
| RP11-401P9.4 | FRMD3 | 0.527282 | 5.79E-42 | 2.07E-40 |
| RP11-4B16.3 | FRMD3 | 0.536706 | 1.09E-43 | 4.40E-42 |
| RP11-401P9.4 | FRMD4A | 0.496208 | 1.22E-36 | 3.03E-35 |
| RP11-4B16.3 | FRMD4A | 0.468821 | 2.22E-32 | 3.99E-31 |
| RP11-401P9.4 | FRMD4B | 0.439006 | 3.69E-28 | 4.79E-27 |
| RP11-401P9.4 | FRY | 0.624254 | 1.09E-62 | 1.74E-60 |
| RP11-4B16.3 | FRY | 0.480688 | 3.54E-34 | 7.24E-33 |
| RP5-1059L7.1 | FSCN1 | 0.413655 | 6.91E-25 | 6.69E-24 |
| RP11-401P9.4 | FXYD1 | 0.687594 | 1.02E-80 | 8.02E-78 |
| RP11-4B16.3 | FXYD1 | 0.628412 | 9.61E-64 | 1.64E-61 |
| RP11-401P9.4 | FXYD6 | 0.582564 | 6.38E-53 | 4.94E-51 |
| RP11-4B16.3 | FXYD6 | 0.520333 | 1.00E-40 | 3.26E-39 |
| RP11-401P9.4 | FZD4 | 0.493484 | 3.38E-36 | 8.13E-35 |
| RP11-4B16.3 | FZD4 | 0.479978 | 4.55E-34 | 9.21E-33 |
| RP11-401P9.4 | GAB1 | 0.48781 | 2.72E-35 | 6.07E-34 |
| RP11-4B16.3 | GAB1 | 0.493329 | 3.58E-36 | 8.59E-35 |
| RP11-401P9.4 | GAB2 | 0.41131 | 1.34E-24 | 1.27E-23 |
| RP11-401P9.4 | GAB3 | 0.436881 | 7.11E-28 | 9.04E-27 |
| RP11-401P9.4 | GALNT18 | 0.450265 | 1.05E-29 | 1.53E-28 |
| RP11-4B16.3 | GALNT18 | 0.451815 | 6.38E-30 | 9.40E-29 |
| RP11-259K15.2 | GANC | 0.503163 | 8.76E-38 | 2.35E-36 |
| RP11-401P9.4 | GANC | 0.408668 | 2.83E-24 | 2.58E-23 |
| Z83851.4 | GAPDH | 0.447435 | 2.60E-29 | 3.68E-28 |
| RP11-401P9.4 | GAS6 | 0.589639 | 1.77E-54 | 1.57E-52 |
| RP11-4B16.3 | GAS6 | 0.44507 | 5.52E-29 | 7.60E-28 |
| RP11-401P9.4 | GAS7 | 0.478843 | 6.80E-34 | 1.36E-32 |
| RP11-401P9.4 | GATA2 | 0.465209 | 7.60E-32 | 1.31E-30 |
| RP11-4B16.3 | GATA2 | 0.486424 | 4.50E-35 | 9.88E-34 |
| RP11-401P9.4 | GATA5 | 0.525539 | 1.19E-41 | 4.18E-40 |
| RP11-401P9.4 | GATA6 | 0.597796 | 2.53E-56 | 2.53E-54 |
| RP11-4B16.3 | GATA6 | 0.457659 | 9.46E-31 | 1.49E-29 |
| RP11-4B16.3 | GBP4 | 0.409513 | 2.23E-24 | 2.05E-23 |
| RP11-401P9.4 | GDF10 | 0.697424 | 6.13E-84 | 6.92E-81 |
| RP11-4B16.3 | GDF10 | 0.588597 | 3.01E-54 | 2.62E-52 |
| RP11-401P9.4 | GFOD1 | 0.574856 | 2.88E-51 | 1.99E-49 |
| RP11-4B16.3 | GFOD1 | 0.496677 | 1.03E-36 | 2.56E-35 |
| RP11-401P9.4 | GFRA1 | 0.541772 | 1.22E-44 | 5.22E-43 |
| RP11-401P9.4 | GGA2 | 0.490547 | 9.99E-36 | 2.31E-34 |
| Z83851.4 | GGCT | 0.420334 | 1.01E-25 | 1.05E-24 |
| RP11-259K15.2 | GGTLC1 | 0.539623 | 3.10E-44 | 1.29E-42 |
| RP11-401P9.4 | GHR | 0.572699 | 8.21E-51 | 5.46E-49 |
| RP11-4B16.3 | GHR | 0.482227 | 2.04E-34 | 4.26E-33 |
| RP11-401P9.4 | GIMAP1 | 0.581801 | 9.34E-53 | 7.19E-51 |
| RP11-4B16.3 | GIMAP1 | 0.606813 | 2.00E-58 | 2.27E-56 |
| RP11-4B16.3 | GIMAP4 | 0.463991 | 1.15E-31 | 1.95E-30 |
| RP11-401P9.4 | GIMAP5 | 0.465022 | 8.09E-32 | 1.39E-30 |
| RP11-4B16.3 | GIMAP5 | 0.575362 | 2.25E-51 | 1.57E-49 |
| RP11-401P9.4 | GIMAP6 | 0.487317 | 3.26E-35 | 7.22E-34 |
| RP11-4B16.3 | GIMAP6 | 0.566048 | 1.98E-49 | 1.20E-47 |
| RP11-401P9.4 | GIMAP7 | 0.456242 | 1.51E-30 | 2.34E-29 |
| RP11-4B16.3 | GIMAP7 | 0.494946 | 1.96E-36 | 4.81E-35 |
| RP11-401P9.4 | GIMAP8 | 0.602037 | 2.64E-57 | 2.80E-55 |
| RP11-4B16.3 | GIMAP8 | 0.644853 | 4.41E-68 | 1.02E-65 |
| RP11-401P9.4 | GIPC3 | 0.435228 | 1.18E-27 | 1.46E-26 |
| RP11-4B16.3 | GIPC3 | 0.426294 | 1.75E-26 | 1.95E-25 |
| RP11-4B16.3 | GJA4 | 0.434536 | 1.46E-27 | 1.79E-26 |
| RP11-4B16.3 | GJA5 | 0.419144 | 1.43E-25 | 1.47E-24 |
| RP11-401P9.4 | GJC2 | 0.527324 | 5.69E-42 | 2.04E-40 |
| RP11-4B16.3 | GJC2 | 0.452064 | 5.88E-30 | 8.72E-29 |
| RP11-259K15.2 | GKN2 | 0.487908 | 2.62E-35 | 5.87E-34 |
| RP11-401P9.4 | GKN2 | 0.434723 | 1.38E-27 | 1.70E-26 |
| RP11-4B16.3 | GKN2 | 0.403762 | 1.10E-23 | 9.49E-23 |
| RP11-401P9.4 | GLDN | 0.493735 | 3.08E-36 | 7.42E-35 |
| RP11-4B16.3 | GLDN | 0.434067 | 1.68E-27 | 2.05E-26 |
| RP11-401P9.4 | GLIPR2 | 0.406141 | 5.72E-24 | 5.06E-23 |
| RP11-4B16.3 | GLIPR2 | 0.445979 | 4.14E-29 | 5.75E-28 |
| RP11-4B16.3 | GMFG | 0.400989 | 2.36E-23 | 1.97E-22 |
| CTA-384D8.35 | GMPPA | 0.455974 | 1.65E-30 | 2.54E-29 |
| Z83851.4 | GMPPA | 0.460615 | 3.55E-31 | 5.80E-30 |
| RP11-259K15.2 | GMPR | 0.440171 | 2.57E-28 | 3.37E-27 |
| RP11-4B16.3 | GNAI2 | 0.409277 | 2.38E-24 | 2.18E-23 |
| RP11-401P9.4 | GNAQ | 0.508554 | 1.09E-38 | 3.10E-37 |
| RP11-4B16.3 | GNAQ | 0.424927 | 2.62E-26 | 2.88E-25 |
| RP11-4B16.3 | GNG11 | 0.406135 | 5.73E-24 | 5.06E-23 |
| RP11-259K15.2 | GNG7 | 0.419318 | 1.36E-25 | 1.40E-24 |
| RP11-401P9.4 | GNG7 | 0.45837 | 7.48E-31 | 1.19E-29 |
| RP11-401P9.4 | GPA33 | 0.43573 | 1.01E-27 | 1.27E-26 |
| RP11-4B16.3 | GPA33 | 0.431315 | 3.88E-27 | 4.57E-26 |
| RP11-401P9.4 | GPBAR1 | 0.483012 | 1.54E-34 | 3.25E-33 |
| RP11-4B16.3 | GPBAR1 | 0.500326 | 2.59E-37 | 6.68E-36 |
| RP11-401P9.4 | GPC3 | 0.525365 | 1.28E-41 | 4.46E-40 |
| RP11-401P9.4 | GPD1 | 0.566115 | 1.92E-49 | 1.17E-47 |
| RP11-4B16.3 | GPD1 | 0.549645 | 3.76E-46 | 1.78E-44 |
| RP11-259K15.2 | GPD1L | 0.542091 | 1.06E-44 | 4.56E-43 |
| RP11-401P9.4 | GPER1 | 0.478498 | 7.69E-34 | 1.53E-32 |
| RP11-4B16.3 | GPER1 | 0.577167 | 9.28E-52 | 6.68E-50 |
| RP11-259K15.2 | GPIHBP1 | 0.40459 | 8.79E-24 | 7.63E-23 |
| RP11-401P9.4 | GPIHBP1 | 0.666384 | 3.48E-74 | 1.41E-71 |
| RP11-4B16.3 | GPIHBP1 | 0.646632 | 1.44E-68 | 3.53E-66 |
| RP11-401P9.4 | GPM6A | 0.612852 | 7.16E-60 | 9.16E-58 |
| RP11-4B16.3 | GPM6A | 0.653931 | 1.36E-70 | 3.86E-68 |
| RP11-401P9.4 | GPM6B | 0.536887 | 1.01E-43 | 4.09E-42 |
| RP11-4B16.3 | GPM6B | 0.453252 | 4.00E-30 | 6.00E-29 |
| RP5-1059L7.1 | GPR115 | 0.424028 | 3.42E-26 | 3.72E-25 |
| RP11-259K15.2 | GPR116 | 0.484459 | 9.16E-35 | 1.96E-33 |
| RP11-401P9.4 | GPR116 | 0.407291 | 4.15E-24 | 3.72E-23 |
| RP11-401P9.4 | GPR124 | 0.425821 | 2.01E-26 | 2.23E-25 |
| RP11-259K15.2 | GPR133 | 0.492767 | 4.40E-36 | 1.05E-34 |
| RP11-401P9.4 | GPR133 | 0.427935 | 1.07E-26 | 1.21E-25 |
| RP11-401P9.4 | GPR146 | 0.643203 | 1.23E-67 | 2.67E-65 |
| RP11-4B16.3 | GPR146 | 0.687568 | 1.04E-80 | 8.02E-78 |
| RP11-401P9.4 | GPR17 | 0.531945 | 8.23E-43 | 3.13E-41 |
| RP11-4B16.3 | GPR17 | 0.568119 | 7.41E-50 | 4.65E-48 |
| RP11-4B16.3 | GPR4 | 0.411845 | 1.16E-24 | 1.10E-23 |
| RP11-401P9.4 | GPRASP1 | 0.536715 | 1.08E-43 | 4.38E-42 |
| RP11-401P9.4 | GPRIN2 | 0.43972 | 2.95E-28 | 3.87E-27 |
| RP11-401P9.4 | GPX3 | 0.452558 | 5.01E-30 | 7.45E-29 |
| RP11-4B16.3 | GPX3 | 0.489872 | 1.28E-35 | 2.92E-34 |
| RP11-259K15.2 | GRAMD2 | 0.417512 | 2.29E-25 | 2.31E-24 |
| RP11-401P9.4 | GRAMD2 | 0.420555 | 9.47E-26 | 9.88E-25 |
| RP11-401P9.4 | GRASP | 0.584209 | 2.79E-53 | 2.19E-51 |
| RP11-4B16.3 | GRASP | 0.58786 | 4.39E-54 | 3.71E-52 |
| RP5-1059L7.1 | GREM1 | 0.452629 | 4.90E-30 | 7.29E-29 |
| RP11-259K15.2 | GRIA1 | 0.411022 | 1.46E-24 | 1.37E-23 |
| RP11-401P9.4 | GRIA1 | 0.715374 | 3.58E-90 | 7.53E-87 |
| RP11-4B16.3 | GRIA1 | 0.669887 | 3.16E-75 | 1.43E-72 |
| RP11-401P9.4 | GRK5 | 0.611314 | 1.68E-59 | 2.09E-57 |
| RP11-4B16.3 | GRK5 | 0.644442 | 5.70E-68 | 1.29E-65 |
| RP11-259K15.2 | GSAP | 0.478121 | 8.78E-34 | 1.74E-32 |
| RP11-401P9.4 | GSAP | 0.418405 | 1.77E-25 | 1.80E-24 |
| Z83851.4 | GSS | 0.462149 | 2.13E-31 | 3.56E-30 |
| RP11-401P9.4 | GSTM5 | 0.673006 | 3.64E-76 | 1.82E-73 |
| RP11-4B16.3 | GSTM5 | 0.54994 | 3.30E-46 | 1.57E-44 |
| Z83851.4 | GTSE1 | 0.470372 | 1.31E-32 | 2.39E-31 |
| RP11-401P9.4 | GUCY1A2 | 0.650839 | 9.94E-70 | 2.59E-67 |
| RP11-4B16.3 | GUCY1A2 | 0.596444 | 5.15E-56 | 5.03E-54 |
| RP11-401P9.4 | GYPC | 0.490339 | 1.08E-35 | 2.48E-34 |
| RP11-4B16.3 | GYPC | 0.456408 | 1.43E-30 | 2.22E-29 |
| RP11-401P9.4 | GYPE | 0.623202 | 2.01E-62 | 3.12E-60 |
| RP11-4B16.3 | GYPE | 0.632995 | 6.30E-65 | 1.22E-62 |
| RP11-401P9.4 | HBA1 | 0.478037 | 9.04E-34 | 1.79E-32 |
| RP11-4B16.3 | HBA1 | 0.603148 | 1.46E-57 | 1.57E-55 |
| RP11-401P9.4 | HBA2 | 0.499785 | 3.18E-37 | 8.13E-36 |
| RP11-4B16.3 | HBA2 | 0.559511 | 4.22E-48 | 2.32E-46 |
| RP11-401P9.4 | HBB | 0.433212 | 2.18E-27 | 2.63E-26 |
| RP11-4B16.3 | HBB | 0.492454 | 4.94E-36 | 1.18E-34 |
| RP11-401P9.4 | HBEGF | 0.437109 | 6.63E-28 | 8.44E-27 |
| RP11-4B16.3 | HBEGF | 0.521216 | 7.01E-41 | 2.30E-39 |
| RP11-401P9.4 | HDC | 0.410008 | 1.94E-24 | 1.80E-23 |
| RP11-401P9.4 | HECA | 0.461795 | 2.39E-31 | 3.99E-30 |
| RP11-401P9.4 | HECW2 | 0.41106 | 1.44E-24 | 1.35E-23 |
| RP11-4B16.3 | HECW2 | 0.43408 | 1.68E-27 | 2.04E-26 |
| RP11-401P9.4 | HEG1 | 0.559499 | 4.24E-48 | 2.33E-46 |
| RP11-4B16.3 | HEG1 | 0.530087 | 1.80E-42 | 6.70E-41 |
| RP11-401P9.4 | HEYL | 0.493882 | 2.91E-36 | 7.05E-35 |
| RP11-401P9.4 | HHIP | 0.539513 | 3.25E-44 | 1.35E-42 |
| RP11-4B16.3 | HHIP | 0.518089 | 2.49E-40 | 7.88E-39 |
| RP11-259K15.2 | HIGD1B | 0.515041 | 8.43E-40 | 2.57E-38 |
| RP11-401P9.4 | HIGD1B | 0.586533 | 8.62E-54 | 7.10E-52 |
| RP11-4B16.3 | HIGD1B | 0.590821 | 9.61E-55 | 8.64E-53 |
| RP11-4B16.3 | HK3 | 0.407488 | 3.93E-24 | 3.53E-23 |
| RP11-401P9.4 | HLA-E | 0.401651 | 1.97E-23 | 1.66E-22 |
| RP11-4B16.3 | HLA-E | 0.43503 | 1.25E-27 | 1.55E-26 |
| RP11-259K15.2 | HLF | 0.47058 | 1.22E-32 | 2.23E-31 |
| RP11-401P9.4 | HLF | 0.442758 | 1.14E-28 | 1.54E-27 |
| RP11-401P9.4 | HLX | 0.486052 | 5.15E-35 | 1.12E-33 |
| RP11-4B16.3 | HLX | 0.422775 | 4.94E-26 | 5.30E-25 |
| RP11-401P9.4 | HMCN1 | 0.492629 | 4.63E-36 | 1.11E-34 |
| CTA-384D8.35 | HMGB3 | 0.435803 | 9.90E-28 | 1.24E-26 |
| RP11-401P9.4 | HMGCLL1 | 0.464838 | 8.61E-32 | 1.48E-30 |
| CTA-384D8.35 | HN1 | 0.417901 | 2.04E-25 | 2.08E-24 |
| RP11-259K15.2 | HOPX | 0.571246 | 1.66E-50 | 1.08E-48 |
| RP11-401P9.4 | HOXA5 | 0.493882 | 2.91E-36 | 7.05E-35 |
| RP11-4B16.3 | HOXA5 | 0.463555 | 1.33E-31 | 2.25E-30 |
| RP11-4B16.3 | HPCAL1 | 0.410268 | 1.80E-24 | 1.67E-23 |
| RP11-259K15.2 | HPGDS | 0.447681 | 2.41E-29 | 3.41E-28 |
| RP11-401P9.4 | HPGDS | 0.419267 | 1.38E-25 | 1.42E-24 |
| RP11-4B16.3 | HPS5 | 0.461734 | 2.44E-31 | 4.07E-30 |
| RP11-401P9.4 | HPSE2 | 0.549137 | 4.72E-46 | 2.21E-44 |
| RP11-259K15.2 | HSD17B4 | 0.454422 | 2.73E-30 | 4.15E-29 |
| RP11-401P9.4 | HSD17B4 | 0.440322 | 2.45E-28 | 3.23E-27 |
| RP11-259K15.2 | HSD17B6 | 0.576154 | 1.53E-51 | 1.08E-49 |
| RP11-401P9.4 | HSD17B6 | 0.439491 | 3.17E-28 | 4.14E-27 |
| RP11-4B16.3 | HSD17B6 | 0.40272 | 1.47E-23 | 1.25E-22 |
| RP11-259K15.2 | HSDL2 | 0.503552 | 7.55E-38 | 2.04E-36 |
| CTA-384D8.35 | HSH2D | 0.498888 | 4.46E-37 | 1.13E-35 |
| RP11-401P9.4 | HSPA12B | 0.655169 | 6.07E-71 | 1.79E-68 |
| RP11-4B16.3 | HSPA12B | 0.713431 | 1.78E-89 | 3.52E-86 |
| RP11-401P9.4 | HSPB2 | 0.557315 | 1.16E-47 | 6.08E-46 |
| RP11-4B16.3 | HSPB2 | 0.500112 | 2.80E-37 | 7.21E-36 |
| RP11-401P9.4 | HSPB3 | 0.493851 | 2.95E-36 | 7.12E-35 |
| RP11-4B16.3 | HSPB3 | 0.401199 | 2.23E-23 | 1.87E-22 |
| RP11-401P9.4 | HSPB6 | 0.569192 | 4.44E-50 | 2.85E-48 |
| RP11-4B16.3 | HSPB6 | 0.482823 | 1.65E-34 | 3.46E-33 |
| RP11-401P9.4 | HSPB7 | 0.559328 | 4.59E-48 | 2.49E-46 |
| RP11-259K15.2 | HSPB8 | 0.436099 | 9.04E-28 | 1.14E-26 |
| RP11-401P9.4 | HSPB8 | 0.493166 | 3.80E-36 | 9.12E-35 |
| RP11-4B16.3 | HSPB8 | 0.431516 | 3.65E-27 | 4.31E-26 |
| CTA-384D8.35 | HSPE1 | 0.433887 | 1.78E-27 | 2.16E-26 |
| RP11-401P9.4 | HTR3C | 0.496034 | 1.30E-36 | 3.22E-35 |
| RP11-4B16.3 | HTR3C | 0.686217 | 2.82E-80 | 2.07E-77 |
| RP11-401P9.4 | HYAL1 | 0.404323 | 9.46E-24 | 8.18E-23 |
| RP11-4B16.3 | HYAL2 | 0.504116 | 6.08E-38 | 1.65E-36 |
| RP11-4B16.3 | ICAM2 | 0.485855 | 5.53E-35 | 1.20E-33 |
| CTA-384D8.35 | ICT1 | 0.402293 | 1.65E-23 | 1.40E-22 |
| RP11-401P9.4 | ID4 | 0.53639 | 1.24E-43 | 5.01E-42 |
| CTA-384D8.35 | IFITM1 | 0.415468 | 4.12E-25 | 4.06E-24 |
| RP11-259K15.2 | IFT57 | 0.430853 | 4.46E-27 | 5.22E-26 |
| RP11-259K15.2 | IGIP | 0.409796 | 2.06E-24 | 1.90E-23 |
| RP11-401P9.4 | IGIP | 0.514507 | 1.04E-39 | 3.16E-38 |
| RP11-401P9.4 | IGSF10 | 0.65635 | 2.81E-71 | 8.44E-69 |
| RP11-4B16.3 | IGSF10 | 0.556169 | 1.96E-47 | 1.02E-45 |
| RP11-401P9.4 | IHH | 0.491346 | 7.45E-36 | 1.74E-34 |
| RP11-401P9.4 | IL11RA | 0.446558 | 3.44E-29 | 4.81E-28 |
| RP11-401P9.4 | IL16 | 0.405304 | 7.21E-24 | 6.31E-23 |
| RP11-401P9.4 | IL17D | 0.434158 | 1.64E-27 | 2.00E-26 |
| RP11-4B16.3 | IL18R1 | 0.517634 | 2.99E-40 | 9.40E-39 |
| RP11-4B16.3 | IL1RL1 | 0.565436 | 2.64E-49 | 1.58E-47 |
| RP11-401P9.4 | IL33 | 0.531125 | 1.16E-42 | 4.37E-41 |
| RP11-4B16.3 | IL33 | 0.494335 | 2.46E-36 | 5.99E-35 |
| RP11-401P9.4 | IL3RA | 0.465688 | 6.46E-32 | 1.12E-30 |
| RP11-4B16.3 | IL3RA | 0.529998 | 1.87E-42 | 6.94E-41 |
| RP11-401P9.4 | IL5RA | 0.438065 | 4.93E-28 | 6.35E-27 |
| RP11-259K15.2 | IL6R | 0.429283 | 7.16E-27 | 8.19E-26 |
| RP11-401P9.4 | IL7R | 0.423514 | 3.98E-26 | 4.29E-25 |
| RP11-4B16.3 | IL7R | 0.494431 | 2.37E-36 | 5.79E-35 |
| Z83851.4 | IMP4 | 0.403429 | 1.21E-23 | 1.04E-22 |
| RP11-259K15.2 | INMT | 0.506958 | 2.03E-38 | 5.70E-37 |
| RP11-401P9.4 | INMT | 0.703093 | 7.40E-86 | 1.02E-82 |
| RP11-4B16.3 | INMT | 0.575662 | 1.94E-51 | 1.36E-49 |
| RP11-401P9.4 | INPP5A | 0.565755 | 2.27E-49 | 1.37E-47 |
| RP11-4B16.3 | INPP5A | 0.401391 | 2.12E-23 | 1.77E-22 |
| RP11-259K15.2 | INPP5K | 0.476783 | 1.41E-33 | 2.75E-32 |
| RP11-401P9.4 | INPP5K | 0.521394 | 6.52E-41 | 2.14E-39 |
| RP11-4B16.3 | INPP5K | 0.469146 | 1.99E-32 | 3.59E-31 |
| RP11-401P9.4 | IQSEC1 | 0.539937 | 2.70E-44 | 1.14E-42 |
| RP11-401P9.4 | IQSEC3 | 0.568269 | 6.89E-50 | 4.34E-48 |
| RP11-4B16.3 | IQSEC3 | 0.481428 | 2.72E-34 | 5.62E-33 |
| RP11-259K15.2 | IRX1 | 0.454511 | 2.66E-30 | 4.04E-29 |
| RP11-401P9.4 | IRX1 | 0.467855 | 3.09E-32 | 5.49E-31 |
| RP11-259K15.2 | IRX2 | 0.462728 | 1.75E-31 | 2.95E-30 |
| RP11-401P9.4 | ITGA10 | 0.42841 | 9.30E-27 | 1.06E-25 |
| RP5-1059L7.1 | ITGA11 | 0.415927 | 3.61E-25 | 3.58E-24 |
| RP11-401P9.4 | ITGA8 | 0.672552 | 4.99E-76 | 2.42E-73 |
| RP11-4B16.3 | ITGA8 | 0.505602 | 3.43E-38 | 9.44E-37 |
| RP11-401P9.4 | ITGA9 | 0.451744 | 6.53E-30 | 9.61E-29 |
| RP11-401P9.4 | ITIH5 | 0.610739 | 2.31E-59 | 2.83E-57 |
| RP11-4B16.3 | ITIH5 | 0.446509 | 3.50E-29 | 4.88E-28 |
| RP11-401P9.4 | ITLN2 | 0.625145 | 6.52E-63 | 1.06E-60 |
| RP11-4B16.3 | ITLN2 | 0.671341 | 1.16E-75 | 5.38E-73 |
| RP11-401P9.4 | ITM2A | 0.451339 | 7.44E-30 | 1.09E-28 |
| RP11-4B16.3 | ITM2A | 0.511238 | 3.81E-39 | 1.12E-37 |
| RP11-401P9.4 | ITPRIP | 0.409757 | 2.08E-24 | 1.92E-23 |
| RP11-4B16.3 | ITPRIP | 0.471637 | 8.44E-33 | 1.57E-31 |
| RP11-401P9.4 | JAM2 | 0.67605 | 4.30E-77 | 2.42E-74 |
| RP11-4B16.3 | JAM2 | 0.634491 | 2.57E-65 | 5.09E-63 |
| RP11-401P9.4 | JAM3 | 0.503938 | 6.51E-38 | 1.76E-36 |
| RP11-4B16.3 | JAM3 | 0.401443 | 2.09E-23 | 1.75E-22 |
| RP11-401P9.4 | JAZF1 | 0.410642 | 1.62E-24 | 1.51E-23 |
| RP11-4B16.3 | JDP2 | 0.453649 | 3.52E-30 | 5.29E-29 |
| RP11-401P9.4 | JPH2 | 0.462131 | 2.14E-31 | 3.58E-30 |
| RP11-401P9.4 | JPH4 | 0.587723 | 4.70E-54 | 3.96E-52 |
| RP11-4B16.3 | JPH4 | 0.610112 | 3.27E-59 | 3.94E-57 |
| CTA-384D8.35 | JTB | 0.413608 | 7.00E-25 | 6.78E-24 |
| RP11-401P9.4 | KAL1 | 0.567114 | 1.19E-49 | 7.38E-48 |
| RP11-4B16.3 | KAL1 | 0.500705 | 2.24E-37 | 5.80E-36 |
| RP11-401P9.4 | KANK1 | 0.425268 | 2.37E-26 | 2.61E-25 |
| RP11-401P9.4 | KANK2 | 0.619278 | 1.92E-61 | 2.77E-59 |
| RP11-4B16.3 | KANK2 | 0.525718 | 1.11E-41 | 3.88E-40 |
| RP11-401P9.4 | KANK3 | 0.664053 | 1.69E-73 | 6.34E-71 |
| RP11-4B16.3 | KANK3 | 0.708312 | 1.16E-87 | 1.92E-84 |
| CTA-384D8.35 | KAT2A | 0.425938 | 1.94E-26 | 2.16E-25 |
| RP11-401P9.4 | KAT2B | 0.466468 | 4.96E-32 | 8.69E-31 |
| RP11-4B16.3 | KAT2B | 0.439887 | 2.80E-28 | 3.68E-27 |
| RP11-401P9.4 | KAZN | 0.476686 | 1.45E-33 | 2.84E-32 |
| RP11-401P9.4 | KCNA4 | 0.538376 | 5.31E-44 | 2.19E-42 |
| RP11-4B16.3 | KCNA4 | 0.55032 | 2.78E-46 | 1.33E-44 |
| RP11-401P9.4 | KCNA5 | 0.553342 | 7.11E-47 | 3.54E-45 |
| RP11-4B16.3 | KCNA5 | 0.520557 | 9.16E-41 | 2.98E-39 |
| RP11-401P9.4 | KCNAB1 | 0.522284 | 4.54E-41 | 1.51E-39 |
| RP11-4B16.3 | KCNAB1 | 0.516084 | 5.56E-40 | 1.71E-38 |
| RP11-401P9.4 | KCNJ15 | 0.435092 | 1.23E-27 | 1.52E-26 |
| RP11-401P9.4 | KCNJ5 | 0.52227 | 4.56E-41 | 1.52E-39 |
| RP11-259K15.2 | KCNK17 | 0.406364 | 5.38E-24 | 4.76E-23 |
| RP11-401P9.4 | KCNK17 | 0.423138 | 4.44E-26 | 4.78E-25 |
| RP11-401P9.4 | KCNK3 | 0.580934 | 1.44E-52 | 1.09E-50 |
| RP11-4B16.3 | KCNK3 | 0.495135 | 1.83E-36 | 4.48E-35 |
| RP11-401P9.4 | KCNT2 | 0.491025 | 8.38E-36 | 1.95E-34 |
| RP11-4B16.3 | KCNT2 | 0.527281 | 5.79E-42 | 2.07E-40 |
| RP11-401P9.4 | KCTD10 | 0.403364 | 1.23E-23 | 1.06E-22 |
| RP11-401P9.4 | KCTD12 | 0.408314 | 3.12E-24 | 2.83E-23 |
| RP11-4B16.3 | KCTD12 | 0.400504 | 2.70E-23 | 2.24E-22 |
| RP11-401P9.4 | KIAA0040 | 0.480097 | 4.36E-34 | 8.86E-33 |
| RP11-4B16.3 | KIAA0040 | 0.426414 | 1.69E-26 | 1.88E-25 |
| Z83851.4 | KIAA0101 | 0.408993 | 2.58E-24 | 2.36E-23 |
| RP11-401P9.4 | KIAA1324L | 0.551042 | 2.01E-46 | 9.75E-45 |
| RP11-4B16.3 | KIAA1324L | 0.459934 | 4.45E-31 | 7.20E-30 |
| RP11-401P9.4 | KIAA1462 | 0.50228 | 1.23E-37 | 3.26E-36 |
| RP11-4B16.3 | KIAA1462 | 0.627725 | 1.44E-63 | 2.42E-61 |
| RP11-401P9.4 | KIAA1683 | 0.532065 | 7.82E-43 | 2.98E-41 |
| RP11-4B16.3 | KIAA1683 | 0.477979 | 9.23E-34 | 1.82E-32 |
| RP11-401P9.4 | KIF17 | 0.48549 | 6.31E-35 | 1.36E-33 |
| RP11-4B16.3 | KIF17 | 0.614545 | 2.78E-60 | 3.69E-58 |
| RP11-401P9.4 | KIF1C | 0.527246 | 5.88E-42 | 2.10E-40 |
| RP11-4B16.3 | KIF1C | 0.467336 | 3.69E-32 | 6.52E-31 |
| Z83851.4 | KIF2C | 0.409638 | 2.15E-24 | 1.98E-23 |
| RP5-1059L7.1 | KIF3C | 0.400238 | 2.90E-23 | 2.40E-22 |
| RP5-1059L7.1 | KIF4A | 0.400336 | 2.82E-23 | 2.34E-22 |
| Z83851.4 | KIF4A | 0.409379 | 2.32E-24 | 2.12E-23 |
| RP11-401P9.4 | KL | 0.559843 | 3.62E-48 | 1.99E-46 |
| RP11-4B16.3 | KL | 0.529556 | 2.24E-42 | 8.30E-41 |
| RP11-401P9.4 | KLF13 | 0.579232 | 3.35E-52 | 2.51E-50 |
| RP11-4B16.3 | KLF13 | 0.401194 | 2.23E-23 | 1.87E-22 |
| RP11-259K15.2 | KLF15 | 0.468804 | 2.24E-32 | 4.02E-31 |
| RP11-401P9.4 | KLF15 | 0.404177 | 9.85E-24 | 8.51E-23 |
| RP11-401P9.4 | KLF2 | 0.519908 | 1.19E-40 | 3.86E-39 |
| RP11-4B16.3 | KLF2 | 0.467195 | 3.87E-32 | 6.83E-31 |
| RP11-401P9.4 | KLF4 | 0.444386 | 6.85E-29 | 9.35E-28 |
| RP11-4B16.3 | KLF4 | 0.488436 | 2.16E-35 | 4.86E-34 |
| RP11-401P9.4 | KLF6 | 0.407285 | 4.16E-24 | 3.73E-23 |
| RP11-4B16.3 | KLF6 | 0.503344 | 8.18E-38 | 2.20E-36 |
| RP11-401P9.4 | KLF9 | 0.474806 | 2.81E-33 | 5.40E-32 |
| RP11-4B16.3 | KLF9 | 0.454341 | 2.81E-30 | 4.26E-29 |
| CTA-384D8.35 | KLHDC7B | 0.443496 | 9.07E-29 | 1.23E-27 |
| Z83851.4 | KNOP1 | 0.433357 | 2.09E-27 | 2.52E-26 |
| RP11-4B16.3 | KRT4 | 0.409883 | 2.01E-24 | 1.86E-23 |
| RP5-1059L7.1 | KRT80 | 0.420582 | 9.39E-26 | 9.80E-25 |
| CTA-384D8.35 | KRTCAP2 | 0.422735 | 5.00E-26 | 5.35E-25 |
| Z83851.4 | LAD1 | 0.404402 | 9.26E-24 | 8.01E-23 |
| RP11-401P9.4 | LAMA2 | 0.543451 | 5.84E-45 | 2.55E-43 |
| RP11-401P9.4 | LAMB2 | 0.432453 | 2.75E-27 | 3.28E-26 |
| RP11-401P9.4 | LAMC3 | 0.478443 | 7.83E-34 | 1.56E-32 |
| RP11-4B16.3 | LAMC3 | 0.497321 | 8.05E-37 | 2.02E-35 |
| RP11-259K15.2 | LAMP3 | 0.464661 | 9.14E-32 | 1.57E-30 |
| RP11-401P9.4 | LAMP3 | 0.486905 | 3.78E-35 | 8.34E-34 |
| RP11-4B16.3 | LAMP3 | 0.466294 | 5.26E-32 | 9.20E-31 |
| RP11-401P9.4 | LATS2 | 0.406473 | 5.22E-24 | 4.62E-23 |
| RP11-4B16.3 | LATS2 | 0.412586 | 9.37E-25 | 8.98E-24 |
| RP11-401P9.4 | LBH | 0.548999 | 5.02E-46 | 2.34E-44 |
| RP11-401P9.4 | LDB2 | 0.682813 | 3.39E-79 | 2.28E-76 |
| RP11-4B16.3 | LDB2 | 0.644171 | 6.75E-68 | 1.50E-65 |
| RP11-4B16.3 | LDLR | 0.437186 | 6.47E-28 | 8.25E-27 |
| RP11-401P9.4 | LEFTY2 | 0.573872 | 4.65E-51 | 3.16E-49 |
| RP11-401P9.4 | LEPR | 0.492278 | 5.28E-36 | 1.25E-34 |
| RP11-4B16.3 | LEPR | 0.431751 | 3.40E-27 | 4.02E-26 |
| RP11-401P9.4 | LGALSL | 0.442604 | 1.20E-28 | 1.62E-27 |
| RP11-259K15.2 | LGI3 | 0.478437 | 7.85E-34 | 1.56E-32 |
| RP11-401P9.4 | LGI3 | 0.616844 | 7.63E-61 | 1.07E-58 |
| RP11-4B16.3 | LGI3 | 0.61099 | 2.01E-59 | 2.48E-57 |
| RP11-401P9.4 | LGI4 | 0.554556 | 4.10E-47 | 2.07E-45 |
| RP11-4B16.3 | LGI4 | 0.555853 | 2.27E-47 | 1.17E-45 |
| RP11-401P9.4 | LHFP | 0.624359 | 1.03E-62 | 1.64E-60 |
| RP11-4B16.3 | LHFP | 0.49663 | 1.04E-36 | 2.60E-35 |
| RP11-259K15.2 | LHFPL3 | 0.406196 | 5.63E-24 | 4.98E-23 |
| RP11-401P9.4 | LHFPL3 | 0.483702 | 1.20E-34 | 2.56E-33 |
| RP11-4B16.3 | LHFPL3 | 0.417933 | 2.03E-25 | 2.06E-24 |
| RP11-401P9.4 | LIFR | 0.436587 | 7.78E-28 | 9.87E-27 |
| RP11-4B16.3 | LIFR | 0.415479 | 4.10E-25 | 4.05E-24 |
| RP11-4B16.3 | LILRA1 | 0.480386 | 3.94E-34 | 8.03E-33 |
| RP11-4B16.3 | LILRA5 | 0.417458 | 2.32E-25 | 2.35E-24 |
| RP11-4B16.3 | LILRB3 | 0.432198 | 2.97E-27 | 3.53E-26 |
| RP11-401P9.4 | LIMCH1 | 0.437423 | 6.01E-28 | 7.71E-27 |
| RP11-259K15.2 | LIMD1 | 0.429578 | 6.55E-27 | 7.53E-26 |
| RP11-401P9.4 | LIMD1 | 0.512913 | 1.96E-39 | 5.84E-38 |
| RP11-401P9.4 | LIMS2 | 0.685915 | 3.52E-80 | 2.53E-77 |
| RP11-4B16.3 | LIMS2 | 0.666731 | 2.75E-74 | 1.13E-71 |
| RP11-401P9.4 | LIN7A | 0.631648 | 1.41E-64 | 2.55E-62 |
| RP11-4B16.3 | LIN7A | 0.541599 | 1.31E-44 | 5.62E-43 |
| RP11-401P9.4 | LMCD1 | 0.606957 | 1.85E-58 | 2.11E-56 |
| RP11-4B16.3 | LMCD1 | 0.517787 | 2.81E-40 | 8.87E-39 |
| RP11-401P9.4 | LMO2 | 0.55846 | 6.86E-48 | 3.68E-46 |
| RP11-4B16.3 | LMO2 | 0.576313 | 1.41E-51 | 1.00E-49 |
| RP11-259K15.2 | LMO3 | 0.490967 | 8.56E-36 | 1.98E-34 |
| RP11-4B16.3 | LMO7 | 0.402622 | 1.51E-23 | 1.28E-22 |
| RP11-401P9.4 | LMOD1 | 0.639248 | 1.42E-66 | 2.90E-64 |
| RP11-4B16.3 | LMOD1 | 0.430994 | 4.28E-27 | 5.01E-26 |
| RP5-1059L7.1 | LOXL2 | 0.464171 | 1.08E-31 | 1.84E-30 |
| RP11-401P9.4 | LPHN2 | 0.462081 | 2.18E-31 | 3.64E-30 |
| RP11-4B16.3 | LPHN2 | 0.509036 | 9.02E-39 | 2.58E-37 |
| RP11-259K15.2 | LPL | 0.468447 | 2.53E-32 | 4.52E-31 |
| RP11-401P9.4 | LPL | 0.445413 | 4.95E-29 | 6.84E-28 |
| RP11-401P9.4 | LRCH1 | 0.466967 | 4.18E-32 | 7.36E-31 |
| RP11-4B16.3 | LRCH1 | 0.420102 | 1.08E-25 | 1.12E-24 |
| RP11-401P9.4 | LRP1 | 0.440998 | 1.98E-28 | 2.63E-27 |
| RP11-259K15.2 | LRP2BP | 0.458733 | 6.63E-31 | 1.06E-29 |
| RP11-401P9.4 | LRP2BP | 0.609576 | 4.40E-59 | 5.24E-57 |
| RP11-4B16.3 | LRP2BP | 0.469194 | 1.96E-32 | 3.53E-31 |
| RP11-401P9.4 | LRRC18 | 0.438729 | 4.02E-28 | 5.20E-27 |
| RP11-4B16.3 | LRRC18 | 0.404001 | 1.03E-23 | 8.91E-23 |
| RP11-401P9.4 | LRRC2 | 0.560323 | 2.90E-48 | 1.62E-46 |
| RP11-4B16.3 | LRRC2 | 0.552965 | 8.44E-47 | 4.17E-45 |
| RP11-401P9.4 | LRRC32 | 0.580965 | 1.42E-52 | 1.08E-50 |
| RP11-4B16.3 | LRRC32 | 0.570725 | 2.13E-50 | 1.38E-48 |
| RP11-259K15.2 | LRRC36 | 0.566382 | 1.69E-49 | 1.04E-47 |
| RP11-401P9.4 | LRRC36 | 0.664813 | 1.01E-73 | 3.96E-71 |
| RP11-4B16.3 | LRRC36 | 0.568383 | 6.53E-50 | 4.12E-48 |
| RP11-401P9.4 | LRRC48 | 0.403046 | 1.35E-23 | 1.15E-22 |
| RP11-4B16.3 | LRRC4B | 0.430554 | 4.88E-27 | 5.69E-26 |
| RP11-401P9.4 | LRRC8C | 0.403767 | 1.10E-23 | 9.49E-23 |
| RP11-401P9.4 | LRRFIP1 | 0.419577 | 1.26E-25 | 1.30E-24 |
| RP11-259K15.2 | LRRK2 | 0.51104 | 4.11E-39 | 1.20E-37 |
| RP11-401P9.4 | LRRK2 | 0.45774 | 9.21E-31 | 1.45E-29 |
| RP11-4B16.3 | LRRK2 | 0.416259 | 3.28E-25 | 3.27E-24 |
| RP11-401P9.4 | LRRN3 | 0.675766 | 5.25E-77 | 2.90E-74 |
| RP11-4B16.3 | LRRN3 | 0.667907 | 1.23E-74 | 5.33E-72 |
| RP11-401P9.4 | LSAMP | 0.602078 | 2.59E-57 | 2.75E-55 |
| RP11-401P9.4 | LTBP2 | 0.531195 | 1.13E-42 | 4.26E-41 |
| RP11-401P9.4 | LTBP4 | 0.615603 | 1.54E-60 | 2.11E-58 |
| RP11-4B16.3 | LTBP4 | 0.515545 | 6.90E-40 | 2.11E-38 |
| RP11-401P9.4 | LYVE1 | 0.51225 | 2.55E-39 | 7.55E-38 |
| RP11-4B16.3 | LYVE1 | 0.586778 | 7.61E-54 | 6.31E-52 |
| RP11-401P9.4 | MACF1 | 0.522587 | 4.01E-41 | 1.34E-39 |
| RP11-259K15.2 | MACROD2 | 0.405074 | 7.69E-24 | 6.71E-23 |
| RP11-401P9.4 | MACROD2 | 0.409585 | 2.19E-24 | 2.01E-23 |
| RP11-401P9.4 | MAGI1 | 0.545362 | 2.52E-45 | 1.12E-43 |
| RP11-259K15.2 | MAMDC2 | 0.451869 | 6.27E-30 | 9.24E-29 |
| RP11-401P9.4 | MAMDC2 | 0.66135 | 1.03E-72 | 3.54E-70 |
| RP11-4B16.3 | MAMDC2 | 0.506144 | 2.78E-38 | 7.73E-37 |
| CTA-384D8.35 | MANF | 0.483254 | 1.41E-34 | 2.99E-33 |
| RP11-259K15.2 | MAOA | 0.440734 | 2.15E-28 | 2.85E-27 |
| RP11-401P9.4 | MAOB | 0.595248 | 9.64E-56 | 9.17E-54 |
| RP11-401P9.4 | MAP3K3 | 0.514269 | 1.15E-39 | 3.45E-38 |
| RP11-4B16.3 | MAP3K3 | 0.441528 | 1.68E-28 | 2.25E-27 |
| RP11-401P9.4 | MAP6 | 0.490455 | 1.03E-35 | 2.38E-34 |
| RP11-259K15.2 | MAPK10 | 0.417813 | 2.10E-25 | 2.13E-24 |
| RP11-401P9.4 | MAPK10 | 0.424879 | 2.66E-26 | 2.91E-25 |
| RP11-401P9.4 | MAPRE2 | 0.403505 | 1.19E-23 | 1.02E-22 |
| RP11-401P9.4 | MARCO | 0.428545 | 8.93E-27 | 1.02E-25 |
| RP11-4B16.3 | MARCO | 0.475397 | 2.29E-33 | 4.43E-32 |
| RP11-401P9.4 | MASP1 | 0.552494 | 1.04E-46 | 5.14E-45 |
| RP11-4B16.3 | MASP1 | 0.569654 | 3.56E-50 | 2.29E-48 |
| Z83851.4 | MAST2 | 0.421573 | 7.03E-26 | 7.44E-25 |
| RP11-401P9.4 | MATN3 | 0.405896 | 6.12E-24 | 5.39E-23 |
| RP11-4B16.3 | MBP | 0.409129 | 2.48E-24 | 2.27E-23 |
| RP11-401P9.4 | MCC | 0.512153 | 2.65E-39 | 7.84E-38 |
| RP11-4B16.3 | MCC | 0.423293 | 4.24E-26 | 4.57E-25 |
| RP11-259K15.2 | MCEMP1 | 0.41665 | 2.93E-25 | 2.93E-24 |
| RP11-401P9.4 | MCEMP1 | 0.507557 | 1.61E-38 | 4.54E-37 |
| RP11-4B16.3 | MCEMP1 | 0.538149 | 5.85E-44 | 2.40E-42 |
| Z83851.4 | MCM5 | 0.42692 | 1.45E-26 | 1.62E-25 |
| RP11-401P9.4 | MDGA1 | 0.512499 | 2.31E-39 | 6.86E-38 |
| RP11-4B16.3 | MDGA1 | 0.473187 | 4.94E-33 | 9.32E-32 |
| RP11-401P9.4 | MEF2A | 0.489442 | 1.50E-35 | 3.40E-34 |
| RP11-4B16.3 | MEF2A | 0.442589 | 1.21E-28 | 1.62E-27 |
| RP11-4B16.3 | MEFV | 0.40026 | 2.88E-23 | 2.39E-22 |
| RP11-401P9.4 | MEIS1 | 0.585172 | 1.72E-53 | 1.37E-51 |
| RP11-4B16.3 | MEIS1 | 0.453647 | 3.52E-30 | 5.30E-29 |
| RP11-401P9.4 | MEOX2 | 0.442869 | 1.10E-28 | 1.49E-27 |
| Z83851.4 | METTL1 | 0.402318 | 1.64E-23 | 1.39E-22 |
| RP11-259K15.2 | METTL7A | 0.415931 | 3.61E-25 | 3.57E-24 |
| RP11-401P9.4 | METTL7A | 0.591994 | 5.24E-55 | 4.81E-53 |
| RP11-4B16.3 | METTL7A | 0.433404 | 2.06E-27 | 2.49E-26 |
| RP11-401P9.4 | MFAP3L | 0.439285 | 3.38E-28 | 4.40E-27 |
| RP11-4B16.3 | MFAP3L | 0.437304 | 6.24E-28 | 7.98E-27 |
| RP11-401P9.4 | MFAP4 | 0.659593 | 3.32E-72 | 1.06E-69 |
| RP11-4B16.3 | MFAP4 | 0.461618 | 2.54E-31 | 4.22E-30 |
| RP11-401P9.4 | MFNG | 0.546673 | 1.41E-45 | 6.40E-44 |
| RP11-4B16.3 | MFNG | 0.510126 | 5.89E-39 | 1.70E-37 |
| RP11-259K15.2 | MFSD2A | 0.494093 | 2.69E-36 | 6.54E-35 |
| RP11-401P9.4 | MFSD2A | 0.435161 | 1.21E-27 | 1.49E-26 |
| RP11-401P9.4 | MGAT3 | 0.651963 | 4.83E-70 | 1.28E-67 |
| RP11-4B16.3 | MGAT3 | 0.56029 | 2.94E-48 | 1.64E-46 |
| RP11-401P9.4 | MGP | 0.496273 | 1.19E-36 | 2.96E-35 |
| RP11-401P9.4 | MICU3 | 0.511167 | 3.91E-39 | 1.15E-37 |
| Z83851.4 | MMACHC | 0.411143 | 1.41E-24 | 1.32E-23 |
| RP11-401P9.4 | MME | 0.427197 | 1.34E-26 | 1.50E-25 |
| RP11-4B16.3 | MME | 0.444487 | 6.64E-29 | 9.08E-28 |
| RP5-1059L7.1 | MMP14 | 0.419339 | 1.35E-25 | 1.39E-24 |
| RP11-401P9.4 | MMP19 | 0.400332 | 2.83E-23 | 2.34E-22 |
| RP11-259K15.2 | MMP28 | 0.40809 | 3.32E-24 | 3.00E-23 |
| RP11-401P9.4 | MMRN1 | 0.52831 | 3.78E-42 | 1.38E-40 |
| RP11-401P9.4 | MMRN2 | 0.607334 | 1.50E-58 | 1.73E-56 |
| RP11-4B16.3 | MMRN2 | 0.57215 | 1.07E-50 | 7.05E-49 |
| RP11-259K15.2 | MOAP1 | 0.491459 | 7.14E-36 | 1.68E-34 |
| RP11-401P9.4 | MOCS1 | 0.577812 | 6.76E-52 | 4.93E-50 |
| Z83851.4 | MORC2 | 0.405541 | 6.76E-24 | 5.93E-23 |
| RP11-401P9.4 | MPDZ | 0.417966 | 2.01E-25 | 2.04E-24 |
| RP11-401P9.4 | MRC1 | 0.42405 | 3.40E-26 | 3.69E-25 |
| RP11-4B16.3 | MRC1 | 0.435524 | 1.08E-27 | 1.34E-26 |
| RP5-1059L7.1 | MRGBP | 0.423817 | 3.64E-26 | 3.94E-25 |
| Z83851.4 | MRM1 | 0.424865 | 2.67E-26 | 2.92E-25 |
| RP11-401P9.4 | MS4A14 | 0.417124 | 2.56E-25 | 2.57E-24 |
| RP11-4B16.3 | MS4A14 | 0.418604 | 1.67E-25 | 1.71E-24 |
| RP11-401P9.4 | MS4A15 | 0.421688 | 6.80E-26 | 7.20E-25 |
| RP11-401P9.4 | MS4A2 | 0.565998 | 2.03E-49 | 1.23E-47 |
| RP11-4B16.3 | MS4A2 | 0.408749 | 2.76E-24 | 2.52E-23 |
| RP11-401P9.4 | MS4A7 | 0.459759 | 4.72E-31 | 7.62E-30 |
| RP11-4B16.3 | MS4A7 | 0.4832 | 1.44E-34 | 3.05E-33 |
| RP11-4B16.3 | MSR1 | 0.436823 | 7.24E-28 | 9.19E-27 |
| RP11-401P9.4 | MSRB3 | 0.577182 | 9.22E-52 | 6.64E-50 |
| RP11-4B16.3 | MSRB3 | 0.479116 | 6.18E-34 | 1.24E-32 |
| RP11-401P9.4 | MT-ND5 | 0.424636 | 2.86E-26 | 3.12E-25 |
| RP11-401P9.4 | MT-ND6 | 0.447574 | 2.49E-29 | 3.52E-28 |
| RP11-4B16.3 | MT1M | 0.438742 | 4.00E-28 | 5.18E-27 |
| Z83851.4 | MTFP1 | 0.40058 | 2.64E-23 | 2.20E-22 |
| RP11-401P9.4 | MTMR10 | 0.618441 | 3.08E-61 | 4.45E-59 |
| RP11-4B16.3 | MTMR10 | 0.477455 | 1.11E-33 | 2.18E-32 |
| RP11-259K15.2 | MTURN | 0.491155 | 7.99E-36 | 1.86E-34 |
| RP11-401P9.4 | MTURN | 0.476328 | 1.65E-33 | 3.22E-32 |
| RP11-401P9.4 | MVB12B | 0.491266 | 7.67E-36 | 1.79E-34 |
| RP11-4B16.3 | MVB12B | 0.45033 | 1.03E-29 | 1.50E-28 |
| RP11-401P9.4 | MYADM | 0.451482 | 7.10E-30 | 1.04E-28 |
| RP11-4B16.3 | MYADM | 0.443189 | 9.99E-29 | 1.35E-27 |
| Z83851.4 | MYBL2 | 0.411133 | 1.41E-24 | 1.33E-23 |
| RP11-401P9.4 | MYCT1 | 0.591229 | 7.79E-55 | 7.05E-53 |
| RP11-4B16.3 | MYCT1 | 0.614776 | 2.44E-60 | 3.28E-58 |
| CTA-384D8.35 | MYDGF | 0.407664 | 3.74E-24 | 3.37E-23 |
| RP11-401P9.4 | MYH10 | 0.538474 | 5.09E-44 | 2.10E-42 |
| RP11-401P9.4 | MYH11 | 0.67375 | 2.16E-76 | 1.12E-73 |
| RP11-401P9.4 | MYL3 | 0.481938 | 2.26E-34 | 4.71E-33 |
| RP11-4B16.3 | MYL3 | 0.420464 | 9.72E-26 | 1.01E-24 |
| RP11-401P9.4 | MYL9 | 0.475295 | 2.37E-33 | 4.58E-32 |
| RP11-4B16.3 | MYL9 | 0.411853 | 1.15E-24 | 1.09E-23 |
| RP11-401P9.4 | MYLK | 0.471564 | 8.66E-33 | 1.61E-31 |
| RP11-4B16.3 | MYLK | 0.413067 | 8.17E-25 | 7.87E-24 |
| RP11-401P9.4 | MYO1C | 0.401465 | 2.07E-23 | 1.74E-22 |
| RP11-4B16.3 | MYO1C | 0.408766 | 2.75E-24 | 2.51E-23 |
| RP11-401P9.4 | MYO7B | 0.429518 | 6.67E-27 | 7.66E-26 |
| RP11-4B16.3 | MYO7B | 0.416396 | 3.16E-25 | 3.15E-24 |
| RP11-401P9.4 | MYO9A | 0.42991 | 5.93E-27 | 6.85E-26 |
| RP11-401P9.4 | MYOC | 0.569842 | 3.25E-50 | 2.10E-48 |
| RP11-4B16.3 | MYOC | 0.534591 | 2.68E-43 | 1.06E-41 |
| RP11-401P9.4 | MYOCD | 0.663839 | 1.95E-73 | 7.24E-71 |
| RP11-4B16.3 | MYOCD | 0.438544 | 4.25E-28 | 5.49E-27 |
| RP11-259K15.2 | MYOZ1 | 0.474447 | 3.18E-33 | 6.08E-32 |
| RP11-401P9.4 | MYOZ1 | 0.467944 | 3.00E-32 | 5.33E-31 |
| RP11-4B16.3 | MYOZ1 | 0.40573 | 6.41E-24 | 5.64E-23 |
| RP11-401P9.4 | MYRF | 0.460302 | 3.94E-31 | 6.41E-30 |
| RP11-4B16.3 | MYRF | 0.426758 | 1.52E-26 | 1.70E-25 |
| RP11-401P9.4 | MYZAP | 0.629332 | 5.58E-64 | 9.74E-62 |
| RP11-4B16.3 | MYZAP | 0.731941 | 2.26E-96 | 8.92E-93 |
| RP11-401P9.4 | N4BP1 | 0.533987 | 3.47E-43 | 1.35E-41 |
| RP11-4B16.3 | N4BP1 | 0.461594 | 2.56E-31 | 4.25E-30 |
| RP11-401P9.4 | N4BP2L1 | 0.459149 | 5.78E-31 | 9.27E-30 |
| RP11-4B16.3 | N4BP2L1 | 0.411176 | 1.40E-24 | 1.31E-23 |
| RP11-259K15.2 | NAPSA | 0.607654 | 1.26E-58 | 1.48E-56 |
| CTA-384D8.35 | NARF | 0.446715 | 3.27E-29 | 4.58E-28 |
| CTA-384D8.35 | NAT9 | 0.40447 | 9.09E-24 | 7.87E-23 |
| RP11-401P9.4 | NATD1 | 0.557645 | 9.98E-48 | 5.26E-46 |
| RP11-401P9.4 | NCALD | 0.435786 | 9.95E-28 | 1.25E-26 |
| RP11-401P9.4 | NCKAP5 | 0.609722 | 4.06E-59 | 4.85E-57 |
| RP11-4B16.3 | NCKAP5 | 0.605674 | 3.72E-58 | 4.13E-56 |
| RP11-401P9.4 | NDN | 0.439476 | 3.19E-28 | 4.16E-27 |
| RP11-259K15.2 | NDNF | 0.44224 | 1.35E-28 | 1.81E-27 |
| RP11-401P9.4 | NDNF | 0.448266 | 2.00E-29 | 2.85E-28 |
| RP11-401P9.4 | NDRG2 | 0.527224 | 5.93E-42 | 2.11E-40 |
| RP11-401P9.4 | NDRG4 | 0.400217 | 2.92E-23 | 2.41E-22 |
| RP11-4B16.3 | NDRG4 | 0.490477 | 1.03E-35 | 2.36E-34 |
| RP11-401P9.4 | NDST1 | 0.609416 | 4.80E-59 | 5.70E-57 |
| RP11-4B16.3 | NDST1 | 0.464246 | 1.05E-31 | 1.80E-30 |
| RP11-401P9.4 | NEBL | 0.431857 | 3.30E-27 | 3.90E-26 |
| RP11-401P9.4 | NECAB1 | 0.599657 | 9.42E-57 | 9.66E-55 |
| RP11-4B16.3 | NECAB1 | 0.626299 | 3.32E-63 | 5.52E-61 |
| RP11-259K15.2 | NEDD9 | 0.402671 | 1.49E-23 | 1.27E-22 |
| RP11-401P9.4 | NEDD9 | 0.46013 | 4.17E-31 | 6.78E-30 |
| RP11-401P9.4 | NEGR1 | 0.580375 | 1.90E-52 | 1.43E-50 |
| RP11-401P9.4 | NES | 0.485041 | 7.43E-35 | 1.59E-33 |
| RP11-4B16.3 | NES | 0.447128 | 2.87E-29 | 4.03E-28 |
| RP11-401P9.4 | NEXN | 0.466243 | 5.35E-32 | 9.36E-31 |
| RP11-4B16.3 | NEXN | 0.440873 | 2.06E-28 | 2.73E-27 |
| RP11-401P9.4 | NFASC | 0.535205 | 2.07E-43 | 8.20E-42 |
| RP11-401P9.4 | NFATC3 | 0.452239 | 5.56E-30 | 8.25E-29 |
| RP11-401P9.4 | NFIA | 0.428371 | 9.41E-27 | 1.07E-25 |
| RP11-259K15.2 | NFIX | 0.454328 | 2.82E-30 | 4.27E-29 |
| RP11-401P9.4 | NFIX | 0.596535 | 4.91E-56 | 4.81E-54 |
| RP11-401P9.4 | NHLRC4 | 0.402904 | 1.40E-23 | 1.19E-22 |
| RP11-401P9.4 | NHSL1 | 0.603968 | 9.36E-58 | 1.01E-55 |
| RP11-4B16.3 | NHSL1 | 0.412291 | 1.02E-24 | 9.72E-24 |
| RP11-4B16.3 | NIM1K | 0.457404 | 1.03E-30 | 1.62E-29 |
| RP11-259K15.2 | NINJ2 | 0.477432 | 1.12E-33 | 2.19E-32 |
| RP11-401P9.4 | NKD1 | 0.800607 | ####### | ####### |
| RP11-401P9.4 | NKD2 | 0.456015 | 1.62E-30 | 2.51E-29 |
| RP11-401P9.4 | NLRC4 | 0.470234 | 1.37E-32 | 2.50E-31 |
| RP11-4B16.3 | NLRC4 | 0.54128 | 1.51E-44 | 6.43E-43 |
| CTA-384D8.35 | NME1 | 0.466035 | 5.74E-32 | 1.00E-30 |
| RP11-401P9.4 | NMUR1 | 0.657935 | 9.92E-72 | 3.01E-69 |
| RP11-4B16.3 | NMUR1 | 0.693477 | 1.25E-82 | 1.14E-79 |
| Z83851.4 | NOP2 | 0.440394 | 2.39E-28 | 3.16E-27 |
| RP11-401P9.4 | NOSTRIN | 0.462856 | 1.68E-31 | 2.83E-30 |
| RP11-401P9.4 | NOTCH1 | 0.457392 | 1.03E-30 | 1.62E-29 |
| RP11-401P9.4 | NOTCH4 | 0.617138 | 6.46E-61 | 9.10E-59 |
| RP11-4B16.3 | NOTCH4 | 0.61155 | 1.48E-59 | 1.85E-57 |
| RP11-401P9.4 | NOVA2 | 0.62836 | 9.91E-64 | 1.68E-61 |
| RP11-4B16.3 | NOVA2 | 0.67161 | 9.61E-76 | 4.60E-73 |
| RP11-259K15.2 | NPC2 | 0.551665 | 1.52E-46 | 7.42E-45 |
| RP11-401P9.4 | NPNT | 0.463324 | 1.43E-31 | 2.43E-30 |
| RP11-259K15.2 | NPR1 | 0.429327 | 7.07E-27 | 8.09E-26 |
| RP11-401P9.4 | NPR1 | 0.586362 | 9.40E-54 | 7.67E-52 |
| RP11-4B16.3 | NPR1 | 0.562177 | 1.22E-48 | 7.00E-47 |
| RP11-401P9.4 | NPR2 | 0.52697 | 6.59E-42 | 2.34E-40 |
| RP11-401P9.4 | NPR3 | 0.546656 | 1.42E-45 | 6.44E-44 |
| CTA-384D8.35 | NPRL2 | 0.437083 | 6.68E-28 | 8.50E-27 |
| RP11-401P9.4 | NR2F1 | 0.455465 | 1.94E-30 | 2.98E-29 |
| RP11-401P9.4 | NR3C2 | 0.432363 | 2.83E-27 | 3.37E-26 |
| RP11-401P9.4 | NRN1 | 0.417717 | 2.16E-25 | 2.19E-24 |
| RP11-401P9.4 | NTN4 | 0.423818 | 3.64E-26 | 3.94E-25 |
| RP11-4B16.3 | NTN4 | 0.406834 | 4.72E-24 | 4.20E-23 |
| RP11-4B16.3 | NTNG1 | 0.400834 | 2.46E-23 | 2.05E-22 |
| Z83851.4 | NUDT1 | 0.425332 | 2.33E-26 | 2.56E-25 |
| RP11-401P9.4 | NXF3 | 0.435202 | 1.19E-27 | 1.47E-26 |
| RP11-4B16.3 | NXF3 | 0.506625 | 2.31E-38 | 6.46E-37 |
| RP11-401P9.4 | NXPH3 | 0.70794 | 1.56E-87 | 2.46E-84 |
| RP11-4B16.3 | NXPH3 | 0.588832 | 2.67E-54 | 2.34E-52 |
| RP11-401P9.4 | ODAM | 0.587597 | 5.02E-54 | 4.20E-52 |
| RP11-4B16.3 | ODAM | 0.547555 | 9.55E-46 | 4.37E-44 |
| RP11-401P9.4 | ODF3L1 | 0.426392 | 1.70E-26 | 1.89E-25 |
| RP11-4B16.3 | ODF3L1 | 0.435425 | 1.11E-27 | 1.38E-26 |
| RP11-401P9.4 | OGN | 0.523518 | 2.74E-41 | 9.32E-40 |
| RP11-4B16.3 | OGN | 0.412215 | 1.04E-24 | 9.93E-24 |
| RP11-401P9.4 | OLFML1 | 0.55669 | 1.55E-47 | 8.03E-46 |
| RP11-4B16.3 | OLFML1 | 0.435836 | 9.80E-28 | 1.23E-26 |
| RP11-401P9.4 | OLR1 | 0.425746 | 2.06E-26 | 2.28E-25 |
| RP11-4B16.3 | OLR1 | 0.440895 | 2.05E-28 | 2.71E-27 |
| RP11-401P9.4 | OSBPL6 | 0.471556 | 8.68E-33 | 1.61E-31 |
| RP11-401P9.4 | OSCAR | 0.417283 | 2.44E-25 | 2.46E-24 |
| RP11-4B16.3 | OSCAR | 0.46708 | 4.03E-32 | 7.10E-31 |
| RP11-401P9.4 | OTC | 0.550021 | 3.18E-46 | 1.52E-44 |
| RP11-4B16.3 | OTC | 0.538216 | 5.68E-44 | 2.34E-42 |
| RP11-401P9.4 | OTUD1 | 0.566485 | 1.61E-49 | 9.89E-48 |
| RP11-4B16.3 | OTUD1 | 0.568723 | 5.55E-50 | 3.53E-48 |
| RP11-401P9.4 | OVCH1 | 0.57298 | 7.17E-51 | 4.79E-49 |
| RP11-4B16.3 | OVCH1 | 0.486498 | 4.38E-35 | 9.63E-34 |
| RP11-401P9.4 | OVCH2 | 0.600729 | 5.32E-57 | 5.51E-55 |
| RP11-4B16.3 | OVCH2 | 0.607521 | 1.36E-58 | 1.58E-56 |
| RP11-401P9.4 | P2RY1 | 0.494931 | 1.97E-36 | 4.83E-35 |
| RP11-4B16.3 | P2RY1 | 0.534068 | 3.35E-43 | 1.31E-41 |
| RP11-4B16.3 | P2RY14 | 0.465919 | 5.97E-32 | 1.04E-30 |
| RP11-401P9.4 | P3H2 | 0.430728 | 4.63E-27 | 5.40E-26 |
| RP11-4B16.3 | P3H2 | 0.400869 | 2.44E-23 | 2.04E-22 |
| Z83851.4 | PAICS | 0.429466 | 6.78E-27 | 7.77E-26 |
| RP11-401P9.4 | PALD1 | 0.56114 | 1.98E-48 | 1.12E-46 |
| RP11-4B16.3 | PALD1 | 0.465765 | 6.29E-32 | 1.09E-30 |
| RP11-401P9.4 | PALMD | 0.586323 | 9.59E-54 | 7.80E-52 |
| RP11-4B16.3 | PALMD | 0.498572 | 5.02E-37 | 1.28E-35 |
| RP11-401P9.4 | PAPSS1 | 0.413251 | 7.76E-25 | 7.49E-24 |
| RP11-401P9.4 | PARD3B | 0.568323 | 6.72E-50 | 4.23E-48 |
| RP11-401P9.4 | PCDH12 | 0.521168 | 7.15E-41 | 2.34E-39 |
| RP11-4B16.3 | PCDH12 | 0.581616 | 1.02E-52 | 7.87E-51 |
| RP11-401P9.4 | PCDH17 | 0.507769 | 1.48E-38 | 4.19E-37 |
| RP11-4B16.3 | PCDH17 | 0.502181 | 1.28E-37 | 3.38E-36 |
| RP5-1059L7.1 | PCDH7 | 0.408418 | 3.03E-24 | 2.75E-23 |
| RP11-401P9.4 | PCDHGB7 | 0.568101 | 7.47E-50 | 4.68E-48 |
| RP11-4B16.3 | PCDHGB7 | 0.522622 | 3.95E-41 | 1.33E-39 |
| RP11-401P9.4 | PCDHGC3 | 0.528916 | 2.93E-42 | 1.08E-40 |
| RP11-4B16.3 | PCDHGC3 | 0.425888 | 1.97E-26 | 2.19E-25 |
| CTA-384D8.35 | PCGF1 | 0.436238 | 8.66E-28 | 1.09E-26 |
| RP11-401P9.4 | PCSK5 | 0.480152 | 4.28E-34 | 8.69E-33 |
| RP11-401P9.4 | PCYOX1 | 0.400407 | 2.77E-23 | 2.30E-22 |
| RP11-401P9.4 | PCYT1B | 0.430373 | 5.16E-27 | 5.99E-26 |
| RP11-401P9.4 | PDE1B | 0.460006 | 4.35E-31 | 7.04E-30 |
| RP11-401P9.4 | PDE2A | 0.470854 | 1.11E-32 | 2.03E-31 |
| RP11-401P9.4 | PDE5A | 0.66778 | 1.34E-74 | 5.74E-72 |
| RP11-4B16.3 | PDE5A | 0.520925 | 7.89E-41 | 2.58E-39 |
| RP11-401P9.4 | PDE8B | 0.60516 | 4.91E-58 | 5.40E-56 |
| RP11-4B16.3 | PDE8B | 0.45026 | 1.05E-29 | 1.53E-28 |
| RP11-401P9.4 | PDGFB | 0.404868 | 8.14E-24 | 7.09E-23 |
| RP11-4B16.3 | PDGFB | 0.438242 | 4.67E-28 | 6.01E-27 |
| RP11-401P9.4 | PDK4 | 0.478231 | 8.44E-34 | 1.67E-32 |
| RP11-4B16.3 | PDK4 | 0.475923 | 1.90E-33 | 3.70E-32 |
| RP11-259K15.2 | PDLIM2 | 0.423981 | 3.47E-26 | 3.76E-25 |
| RP11-401P9.4 | PDLIM2 | 0.564791 | 3.58E-49 | 2.13E-47 |
| RP11-4B16.3 | PDLIM2 | 0.555686 | 2.45E-47 | 1.26E-45 |
| RP5-1059L7.1 | PDLIM7 | 0.432743 | 2.52E-27 | 3.02E-26 |
| RP11-401P9.4 | PDZD2 | 0.613753 | 4.33E-60 | 5.63E-58 |
| RP11-4B16.3 | PDZD2 | 0.471058 | 1.03E-32 | 1.90E-31 |
| RP11-401P9.4 | PDZRN3 | 0.532653 | 6.10E-43 | 2.35E-41 |
| RP11-401P9.4 | PEAK1 | 0.661818 | 7.56E-73 | 2.68E-70 |
| RP11-4B16.3 | PEAK1 | 0.539569 | 3.17E-44 | 1.32E-42 |
| RP11-401P9.4 | PEAR1 | 0.644359 | 6.01E-68 | 1.34E-65 |
| RP11-4B16.3 | PEAR1 | 0.592163 | 4.80E-55 | 4.42E-53 |
| RP11-259K15.2 | PEBP4 | 0.601238 | 4.06E-57 | 4.25E-55 |
| RP11-401P9.4 | PEBP4 | 0.468581 | 2.41E-32 | 4.32E-31 |
| RP11-4B16.3 | PEBP4 | 0.438646 | 4.12E-28 | 5.33E-27 |
| RP11-401P9.4 | PECAM1 | 0.602692 | 1.86E-57 | 1.98E-55 |
| RP11-4B16.3 | PECAM1 | 0.614344 | 3.11E-60 | 4.11E-58 |
| Z83851.4 | PERM1 | 0.405915 | 6.09E-24 | 5.37E-23 |
| Z83851.4 | PES1 | 0.52548 | 1.22E-41 | 4.27E-40 |
| RP11-4B16.3 | PF4 | 0.408456 | 3.00E-24 | 2.73E-23 |
| CTA-384D8.35 | PFDN2 | 0.411408 | 1.31E-24 | 1.23E-23 |
| CTA-384D8.35 | PFDN6 | 0.403637 | 1.14E-23 | 9.82E-23 |
| RP11-259K15.2 | PGC | 0.528916 | 2.93E-42 | 1.08E-40 |
| RP11-259K15.2 | PGM5 | 0.435759 | 1.00E-27 | 1.26E-26 |
| RP11-401P9.4 | PGM5 | 0.595261 | 9.57E-56 | 9.13E-54 |
| RP11-259K15.2 | PHACTR1 | 0.498071 | 6.07E-37 | 1.54E-35 |
| RP11-401P9.4 | PHACTR1 | 0.622269 | 3.45E-62 | 5.28E-60 |
| RP11-4B16.3 | PHACTR1 | 0.509876 | 6.50E-39 | 1.87E-37 |
| RP11-401P9.4 | PHACTR2 | 0.569544 | 3.75E-50 | 2.41E-48 |
| RP11-4B16.3 | PHACTR2 | 0.486801 | 3.93E-35 | 8.65E-34 |
| RP11-401P9.4 | PHF2 | 0.425924 | 1.95E-26 | 2.17E-25 |
| RP11-401P9.4 | PI16 | 0.526361 | 8.49E-42 | 2.98E-40 |
| RP11-4B16.3 | PI16 | 0.471747 | 8.13E-33 | 1.51E-31 |
| RP11-401P9.4 | PIAS1 | 0.500291 | 2.62E-37 | 6.76E-36 |
| RP11-4B16.3 | PIAS1 | 0.410208 | 1.83E-24 | 1.70E-23 |
| RP11-401P9.4 | PID1 | 0.472652 | 5.94E-33 | 1.12E-31 |
| RP11-401P9.4 | PIEZO2 | 0.44982 | 1.21E-29 | 1.75E-28 |
| RP11-259K15.2 | PIGR | 0.408799 | 2.73E-24 | 2.49E-23 |
| RP11-401P9.4 | PIK3IP1 | 0.486284 | 4.74E-35 | 1.04E-33 |
| RP11-401P9.4 | PIK3R1 | 0.509336 | 8.02E-39 | 2.30E-37 |
| RP11-401P9.4 | PIK3R5 | 0.403302 | 1.25E-23 | 1.07E-22 |
| RP11-401P9.4 | PIP5K1B | 0.530808 | 1.33E-42 | 4.98E-41 |
| RP11-4B16.3 | PIP5K1B | 0.482564 | 1.81E-34 | 3.79E-33 |
| Z83851.4 | PKM | 0.413994 | 6.28E-25 | 6.10E-24 |
| Z83851.4 | PKMYT1 | 0.424201 | 3.25E-26 | 3.53E-25 |
| RP11-401P9.4 | PKNOX2 | 0.664709 | 1.08E-73 | 4.12E-71 |
| RP11-4B16.3 | PKNOX2 | 0.612095 | 1.09E-59 | 1.38E-57 |
| Z83851.4 | PKP3 | 0.407066 | 4.42E-24 | 3.95E-23 |
| RP11-259K15.2 | PLA2G1B | 0.62784 | 1.35E-63 | 2.27E-61 |
| RP11-401P9.4 | PLA2G1B | 0.544552 | 3.60E-45 | 1.60E-43 |
| RP11-259K15.2 | PLA2G4F | 0.58486 | 2.01E-53 | 1.60E-51 |
| RP11-401P9.4 | PLA2G4F | 0.625916 | 4.15E-63 | 6.83E-61 |
| RP11-4B16.3 | PLA2G4F | 0.486692 | 4.08E-35 | 8.99E-34 |
| RP11-401P9.4 | PLA2G5 | 0.407859 | 3.55E-24 | 3.20E-23 |
| RP11-259K15.2 | PLAC9 | 0.472617 | 6.02E-33 | 1.13E-31 |
| RP11-401P9.4 | PLAC9 | 0.627536 | 1.61E-63 | 2.69E-61 |
| RP11-4B16.3 | PLAC9 | 0.530159 | 1.74E-42 | 6.52E-41 |
| RP11-401P9.4 | PLAGL1 | 0.446044 | 4.05E-29 | 5.63E-28 |
| RP5-1059L7.1 | PLAU | 0.409546 | 2.21E-24 | 2.03E-23 |
| RP11-401P9.4 | PLCE1 | 0.506486 | 2.43E-38 | 6.81E-37 |
| RP11-401P9.4 | PLCL1 | 0.565049 | 3.17E-49 | 1.89E-47 |
| RP11-4B16.3 | PLCL1 | 0.4892 | 1.64E-35 | 3.71E-34 |
| RP11-401P9.4 | PLCXD2 | 0.413845 | 6.55E-25 | 6.35E-24 |
| RP11-4B16.3 | PLCXD2 | 0.476006 | 1.85E-33 | 3.60E-32 |
| Z83851.4 | PLEKHG6 | 0.454027 | 3.11E-30 | 4.70E-29 |
| RP11-401P9.4 | PLEKHH2 | 0.576939 | 1.04E-51 | 7.43E-50 |
| RP11-4B16.3 | PLEKHH2 | 0.405344 | 7.14E-24 | 6.25E-23 |
| Z83851.4 | PLEKHN1 | 0.413064 | 8.18E-25 | 7.88E-24 |
| Z83851.4 | PLK1 | 0.434915 | 1.30E-27 | 1.60E-26 |
| RP11-259K15.2 | PLLP | 0.500636 | 2.30E-37 | 5.95E-36 |
| RP11-401P9.4 | PLLP | 0.415952 | 3.58E-25 | 3.55E-24 |
| RP11-401P9.4 | PLSCR4 | 0.521864 | 5.39E-41 | 1.78E-39 |
| RP11-4B16.3 | PLSCR4 | 0.448061 | 2.13E-29 | 3.03E-28 |
| RP11-401P9.4 | PLXDC2 | 0.415813 | 3.73E-25 | 3.69E-24 |
| RP11-401P9.4 | PLXNA2 | 0.444645 | 6.31E-29 | 8.65E-28 |
| RP11-401P9.4 | PMP22 | 0.403561 | 1.17E-23 | 1.00E-22 |
| RP11-401P9.4 | PNPLA6 | 0.452455 | 5.19E-30 | 7.70E-29 |
| RP11-4B16.3 | PNPLA6 | 0.473648 | 4.21E-33 | 7.99E-32 |
| RP11-401P9.4 | PODXL | 0.442004 | 1.45E-28 | 1.94E-27 |
| Z83851.4 | POLD2 | 0.436355 | 8.36E-28 | 1.05E-26 |
| RP5-1059L7.1 | POSTN | 0.40453 | 8.94E-24 | 7.75E-23 |
| RP11-401P9.4 | PPAP2B | 0.546223 | 1.72E-45 | 7.75E-44 |
| RP11-4B16.3 | PPAP2B | 0.406971 | 4.54E-24 | 4.05E-23 |
| RP5-1059L7.1 | PPAPDC1A | 0.46479 | 8.75E-32 | 1.50E-30 |
| RP11-401P9.4 | PPARGC1B | 0.447846 | 2.28E-29 | 3.24E-28 |
| RP11-4B16.3 | PPARGC1B | 0.471089 | 1.02E-32 | 1.88E-31 |
| RP11-401P9.4 | PPM1F | 0.548881 | 5.29E-46 | 2.47E-44 |
| RP11-4B16.3 | PPM1F | 0.494164 | 2.62E-36 | 6.38E-35 |
| Z83851.4 | PPM1G | 0.412218 | 1.04E-24 | 9.92E-24 |
| RP11-401P9.4 | PPP1R14A | 0.487703 | 2.83E-35 | 6.30E-34 |
| RP11-4B16.3 | PPP1R14A | 0.460414 | 3.80E-31 | 6.18E-30 |
| CTA-384D8.35 | PPP1R14B | 0.431694 | 3.46E-27 | 4.09E-26 |
| CTA-384D8.35 | PPP1R14D | 0.432073 | 3.09E-27 | 3.66E-26 |
| RP11-401P9.4 | PPP1R15A | 0.436501 | 7.99E-28 | 1.01E-26 |
| RP11-4B16.3 | PPP1R15A | 0.509889 | 6.46E-39 | 1.87E-37 |
| RP11-401P9.4 | PPP1R16B | 0.455478 | 1.94E-30 | 2.97E-29 |
| CTA-384D8.35 | PPP1R35 | 0.412605 | 9.32E-25 | 8.93E-24 |
| RP11-259K15.2 | PPP2R5A | 0.481315 | 2.83E-34 | 5.84E-33 |
| RP11-401P9.4 | PRAM1 | 0.510997 | 4.18E-39 | 1.22E-37 |
| RP11-4B16.3 | PRAM1 | 0.520024 | 1.14E-40 | 3.68E-39 |
| Z83851.4 | PRC1 | 0.402695 | 1.48E-23 | 1.26E-22 |
| RP11-401P9.4 | PRDM11 | 0.539433 | 3.36E-44 | 1.40E-42 |
| RP11-4B16.3 | PRDM11 | 0.406665 | 4.95E-24 | 4.40E-23 |
| RP11-401P9.4 | PRDM16 | 0.417492 | 2.30E-25 | 2.32E-24 |
| RP11-401P9.4 | PRDM5 | 0.440277 | 2.48E-28 | 3.27E-27 |
| RP11-4B16.3 | PRDM5 | 0.443141 | 1.01E-28 | 1.37E-27 |
| CTA-384D8.35 | PRDX4 | 0.401626 | 1.99E-23 | 1.67E-22 |
| RP11-401P9.4 | PRELP | 0.57344 | 5.73E-51 | 3.87E-49 |
| RP11-4B16.3 | PREX1 | 0.462285 | 2.03E-31 | 3.41E-30 |
| RP11-401P9.4 | PREX2 | 0.643925 | 7.88E-68 | 1.74E-65 |
| RP11-4B16.3 | PREX2 | 0.544672 | 3.42E-45 | 1.52E-43 |
| RP11-401P9.4 | PRG4 | 0.552049 | 1.28E-46 | 6.27E-45 |
| RP11-401P9.4 | PRICKLE1 | 0.511411 | 3.55E-39 | 1.05E-37 |
| RP11-4B16.3 | PRICKLE1 | 0.434418 | 1.51E-27 | 1.85E-26 |
| RP11-401P9.4 | PRICKLE2 | 0.605474 | 4.14E-58 | 4.59E-56 |
| RP11-4B16.3 | PRICKLE2 | 0.516445 | 4.81E-40 | 1.49E-38 |
| RP11-401P9.4 | PRKCE | 0.539741 | 2.94E-44 | 1.24E-42 |
| RP11-4B16.3 | PRKCE | 0.629888 | 4.02E-64 | 7.16E-62 |
| RP11-401P9.4 | PRKCH | 0.457072 | 1.15E-30 | 1.80E-29 |
| RP11-4B16.3 | PRKCH | 0.460914 | 3.21E-31 | 5.28E-30 |
| RP11-401P9.4 | PRKCQ | 0.435779 | 9.97E-28 | 1.25E-26 |
| RP11-401P9.4 | PRKG1 | 0.560056 | 3.28E-48 | 1.82E-46 |
| RP11-4B16.3 | PRKG1 | 0.417335 | 2.41E-25 | 2.43E-24 |
| RP11-401P9.4 | PRKG2 | 0.480332 | 4.01E-34 | 8.17E-33 |
| RP11-4B16.3 | PRKG2 | 0.554123 | 4.99E-47 | 2.50E-45 |
| RP11-4B16.3 | PROS1 | 0.408012 | 3.40E-24 | 3.07E-23 |
| RP11-401P9.4 | PRSS35 | 0.503358 | 8.13E-38 | 2.19E-36 |
| RP11-4B16.3 | PRSS35 | 0.416415 | 3.14E-25 | 3.13E-24 |
| RP11-401P9.4 | PRX | 0.651782 | 5.43E-70 | 1.43E-67 |
| RP11-4B16.3 | PRX | 0.705239 | 1.35E-86 | 2.04E-83 |
| CTA-384D8.35 | PSMB4 | 0.440924 | 2.03E-28 | 2.69E-27 |
| CTA-384D8.35 | PSME2 | 0.46253 | 1.87E-31 | 3.14E-30 |
| CTA-384D8.35 | PSMG3 | 0.404609 | 8.74E-24 | 7.59E-23 |
| Z83851.4 | PSMG3 | 0.417149 | 2.54E-25 | 2.55E-24 |
| RP11-401P9.4 | PTCRA | 0.454245 | 2.90E-30 | 4.38E-29 |
| RP11-4B16.3 | PTCRA | 0.54547 | 2.41E-45 | 1.07E-43 |
| RP11-401P9.4 | PTGDS | 0.437114 | 6.62E-28 | 8.43E-27 |
| RP11-401P9.4 | PTGER4 | 0.478726 | 7.09E-34 | 1.42E-32 |
| RP11-4B16.3 | PTGER4 | 0.449147 | 1.51E-29 | 2.16E-28 |
| RP11-401P9.4 | PTGIR | 0.564098 | 4.97E-49 | 2.93E-47 |
| RP11-4B16.3 | PTGIR | 0.455154 | 2.15E-30 | 3.29E-29 |
| RP11-401P9.4 | PTGIS | 0.419825 | 1.17E-25 | 1.21E-24 |
| RP11-401P9.4 | PTH1R | 0.614 | 3.77E-60 | 4.92E-58 |
| RP11-4B16.3 | PTH1R | 0.516269 | 5.16E-40 | 1.59E-38 |
| RP11-4B16.3 | PTPLAD2 | 0.467759 | 3.20E-32 | 5.66E-31 |
| RP11-401P9.4 | PTPN21 | 0.649992 | 1.71E-69 | 4.42E-67 |
| RP11-4B16.3 | PTPN21 | 0.596616 | 4.70E-56 | 4.64E-54 |
| RP11-401P9.4 | PTPRB | 0.649724 | 2.03E-69 | 5.20E-67 |
| RP11-4B16.3 | PTPRB | 0.624556 | 9.18E-63 | 1.47E-60 |
| RP11-401P9.4 | PTPRD | 0.417581 | 2.24E-25 | 2.27E-24 |
| RP11-401P9.4 | PTPRM | 0.450787 | 8.89E-30 | 1.30E-28 |
| RP11-4B16.3 | PTPRM | 0.435902 | 9.60E-28 | 1.20E-26 |
| RP11-401P9.4 | PTPRQ | 0.560541 | 2.62E-48 | 1.47E-46 |
| RP11-4B16.3 | PTPRQ | 0.606967 | 1.84E-58 | 2.11E-56 |
| RP11-401P9.4 | PTRF | 0.522804 | 3.67E-41 | 1.24E-39 |
| RP11-4B16.3 | PTRF | 0.545509 | 2.36E-45 | 1.06E-43 |
| CTA-384D8.35 | PTRH2 | 0.423905 | 3.55E-26 | 3.85E-25 |
| Z83851.4 | PTRH2 | 0.451138 | 7.94E-30 | 1.16E-28 |
| CTA-384D8.35 | PTTG1 | 0.408068 | 3.34E-24 | 3.02E-23 |
| CTA-384D8.35 | PUS1 | 0.401737 | 1.93E-23 | 1.62E-22 |
| RP11-259K15.2 | PXMP4 | 0.53391 | 3.58E-43 | 1.39E-41 |
| CTA-384D8.35 | PYCR1 | 0.420131 | 1.07E-25 | 1.11E-24 |
| Z83851.4 | PYCR1 | 0.43323 | 2.17E-27 | 2.62E-26 |
| RP11-401P9.4 | QKI | 0.42096 | 8.41E-26 | 8.82E-25 |
| RP11-4B16.3 | QKI | 0.493104 | 3.89E-36 | 9.32E-35 |
| RP11-401P9.4 | RAB11FIP1 | 0.404131 | 9.98E-24 | 8.61E-23 |
| RP11-401P9.4 | RAB11FIP2 | 0.430184 | 5.46E-27 | 6.33E-26 |
| RP11-401P9.4 | RAB40A | 0.548564 | 6.09E-46 | 2.84E-44 |
| RP11-4B16.3 | RAB40A | 0.506843 | 2.12E-38 | 5.96E-37 |
| RP11-401P9.4 | RADIL | 0.499926 | 3.01E-37 | 7.71E-36 |
| RP11-4B16.3 | RADIL | 0.474962 | 2.66E-33 | 5.13E-32 |
| RP11-401P9.4 | RAI2 | 0.673935 | 1.90E-76 | 1.00E-73 |
| RP11-4B16.3 | RAI2 | 0.51751 | 3.14E-40 | 9.82E-39 |
| RP11-401P9.4 | RAMP2 | 0.583849 | 3.35E-53 | 2.62E-51 |
| RP11-4B16.3 | RAMP2 | 0.629312 | 5.65E-64 | 9.80E-62 |
| RP11-401P9.4 | RAMP3 | 0.57314 | 6.63E-51 | 4.44E-49 |
| RP11-4B16.3 | RAMP3 | 0.6602 | 2.22E-72 | 7.22E-70 |
| Z83851.4 | RANBP1 | 0.422771 | 4.95E-26 | 5.30E-25 |
| RP11-401P9.4 | RANBP10 | 0.467691 | 3.27E-32 | 5.79E-31 |
| RP11-401P9.4 | RANBP3L | 0.459623 | 4.94E-31 | 7.97E-30 |
| RP11-4B16.3 | RANBP3L | 0.45012 | 1.10E-29 | 1.60E-28 |
| Z83851.4 | RANGAP1 | 0.542249 | 9.89E-45 | 4.26E-43 |
| RP11-401P9.4 | RAPGEF2 | 0.42121 | 7.82E-26 | 8.23E-25 |
| RP11-401P9.4 | RAPGEF4 | 0.578345 | 5.20E-52 | 3.84E-50 |
| RP11-4B16.3 | RAPGEF4 | 0.539554 | 3.19E-44 | 1.33E-42 |
| RP11-401P9.4 | RAPGEF5 | 0.416544 | 3.02E-25 | 3.02E-24 |
| RP11-401P9.4 | RASGEF1B | 0.500256 | 2.66E-37 | 6.85E-36 |
| RP11-4B16.3 | RASGEF1B | 0.470928 | 1.08E-32 | 1.99E-31 |
| RP11-259K15.2 | RASGRF1 | 0.443983 | 7.78E-29 | 1.06E-27 |
| RP11-401P9.4 | RASGRF1 | 0.527768 | 4.73E-42 | 1.71E-40 |
| RP11-4B16.3 | RASGRF1 | 0.44587 | 4.28E-29 | 5.94E-28 |
| RP11-401P9.4 | RASGRP2 | 0.47561 | 2.12E-33 | 4.12E-32 |
| RP11-401P9.4 | RASGRP4 | 0.55562 | 2.52E-47 | 1.30E-45 |
| RP11-4B16.3 | RASGRP4 | 0.524697 | 1.69E-41 | 5.85E-40 |
| RP11-401P9.4 | RASIP1 | 0.568005 | 7.82E-50 | 4.89E-48 |
| RP11-4B16.3 | RASIP1 | 0.587307 | 5.81E-54 | 4.83E-52 |
| RP11-259K15.2 | RASL11A | 0.405339 | 7.15E-24 | 6.26E-23 |
| RP11-401P9.4 | RASL12 | 0.540175 | 2.44E-44 | 1.03E-42 |
| RP11-4B16.3 | RASL12 | 0.477972 | 9.25E-34 | 1.82E-32 |
| RP11-401P9.4 | RASSF2 | 0.510929 | 4.30E-39 | 1.25E-37 |
| RP11-401P9.4 | RBMS2 | 0.483313 | 1.38E-34 | 2.93E-33 |
| RP11-4B16.3 | RBMS2 | 0.453811 | 3.34E-30 | 5.03E-29 |
| RP11-401P9.4 | RBMS3 | 0.559478 | 4.28E-48 | 2.34E-46 |
| RP11-4B16.3 | RBMS3 | 0.464911 | 8.40E-32 | 1.44E-30 |
| RP11-401P9.4 | RBP2 | 0.511085 | 4.04E-39 | 1.18E-37 |
| RP11-4B16.3 | RBP2 | 0.535029 | 2.23E-43 | 8.83E-42 |
| RP11-401P9.4 | RCAN1 | 0.464434 | 9.87E-32 | 1.69E-30 |
| RP11-4B16.3 | RCAN1 | 0.4857 | 5.85E-35 | 1.27E-33 |
| RP11-401P9.4 | RCAN2 | 0.403231 | 1.28E-23 | 1.09E-22 |
| RP11-401P9.4 | RCBTB2 | 0.40443 | 9.19E-24 | 7.95E-23 |
| Z83851.4 | RCC1 | 0.437316 | 6.22E-28 | 7.95E-27 |
| Z83851.4 | RCC2 | 0.429618 | 6.47E-27 | 7.44E-26 |
| Z83851.4 | RCCD1 | 0.429042 | 7.70E-27 | 8.79E-26 |
| RP11-401P9.4 | RECK | 0.522883 | 3.55E-41 | 1.20E-39 |
| RP11-4B16.3 | RECK | 0.456314 | 1.47E-30 | 2.29E-29 |
| Z83851.4 | RECQL4 | 0.408148 | 3.27E-24 | 2.96E-23 |
| RP11-401P9.4 | REEP1 | 0.411502 | 1.27E-24 | 1.20E-23 |
| RP11-401P9.4 | REM1 | 0.566194 | 1.85E-49 | 1.13E-47 |
| RP11-4B16.3 | REM1 | 0.471381 | 9.22E-33 | 1.71E-31 |
| RP11-401P9.4 | REPS2 | 0.437673 | 5.57E-28 | 7.15E-27 |
| RP11-401P9.4 | RERG | 0.436661 | 7.61E-28 | 9.65E-27 |
| RP11-259K15.2 | RETN | 0.428779 | 8.33E-27 | 9.48E-26 |
| RP11-401P9.4 | RETN | 0.44428 | 7.08E-29 | 9.65E-28 |
| RP11-4B16.3 | RETN | 0.487872 | 2.66E-35 | 5.94E-34 |
| RP11-401P9.4 | REV3L | 0.477769 | 9.94E-34 | 1.95E-32 |
| RP11-259K15.2 | RFTN1 | 0.465667 | 6.51E-32 | 1.13E-30 |
| RP11-401P9.4 | RFX2 | 0.431394 | 3.79E-27 | 4.47E-26 |
| RP11-401P9.4 | RGAG4 | 0.448643 | 1.77E-29 | 2.53E-28 |
| RP11-259K15.2 | RGCC | 0.4598 | 4.66E-31 | 7.52E-30 |
| RP11-401P9.4 | RGCC | 0.612433 | 9.05E-60 | 1.15E-57 |
| RP11-4B16.3 | RGCC | 0.587313 | 5.80E-54 | 4.83E-52 |
| RP11-401P9.4 | RGS5 | 0.402061 | 1.76E-23 | 1.49E-22 |
| RP11-401P9.4 | RGS9 | 0.645712 | 2.57E-68 | 6.02E-66 |
| RP11-4B16.3 | RGS9 | 0.677488 | 1.55E-77 | 9.24E-75 |
| Z83851.4 | RHBDD3 | 0.44815 | 2.07E-29 | 2.95E-28 |
| Z83851.4 | RHBDL2 | 0.442036 | 1.43E-28 | 1.92E-27 |
| Z83851.4 | RHNO1 | 0.455645 | 1.83E-30 | 2.82E-29 |
| RP11-259K15.2 | RHOBTB2 | 0.471565 | 8.66E-33 | 1.61E-31 |
| RP11-401P9.4 | RHOJ | 0.632525 | 8.35E-65 | 1.58E-62 |
| RP11-4B16.3 | RHOJ | 0.561827 | 1.44E-48 | 8.15E-47 |
| RP11-259K15.2 | RILP | 0.533854 | 3.67E-43 | 1.42E-41 |
| RP11-401P9.4 | RILPL2 | 0.506135 | 2.79E-38 | 7.75E-37 |
| RP11-4B16.3 | RILPL2 | 0.470369 | 1.31E-32 | 2.39E-31 |
| RP11-401P9.4 | RMDN3 | 0.41368 | 6.86E-25 | 6.65E-24 |
| RP11-4B16.3 | RMDN3 | 0.42147 | 7.25E-26 | 7.65E-25 |
| Z83851.4 | RMI2 | 0.433093 | 2.26E-27 | 2.72E-26 |
| RP11-259K15.2 | RNASE1 | 0.588492 | 3.18E-54 | 2.75E-52 |
| RP11-401P9.4 | RNF144B | 0.492433 | 4.98E-36 | 1.19E-34 |
| RP11-4B16.3 | RNF144B | 0.469288 | 1.90E-32 | 3.42E-31 |
| RP11-401P9.4 | RNF180 | 0.411918 | 1.13E-24 | 1.08E-23 |
| RP11-401P9.4 | RNF182 | 0.403748 | 1.11E-23 | 9.53E-23 |
| RP11-401P9.4 | RNF38 | 0.404171 | 9.87E-24 | 8.52E-23 |
| RP11-401P9.4 | ROBO2 | 0.6751 | 8.39E-77 | 4.49E-74 |
| RP11-401P9.4 | ROBO4 | 0.636835 | 6.21E-66 | 1.26E-63 |
| RP11-4B16.3 | ROBO4 | 0.661662 | 8.38E-73 | 2.91E-70 |
| RP11-401P9.4 | ROR1 | 0.458257 | 7.76E-31 | 1.23E-29 |
| RP11-4B16.3 | ROR1 | 0.490289 | 1.10E-35 | 2.52E-34 |
| RP11-401P9.4 | RORA | 0.620301 | 1.07E-61 | 1.59E-59 |
| RP11-259K15.2 | ROS1 | 0.414126 | 6.04E-25 | 5.88E-24 |
| RP11-401P9.4 | RPGR | 0.485546 | 6.19E-35 | 1.34E-33 |
| RP11-259K15.2 | RPS6KA2 | 0.449281 | 1.44E-29 | 2.07E-28 |
| RP11-4B16.3 | RRAS | 0.427162 | 1.35E-26 | 1.51E-25 |
| Z83851.4 | RRP1 | 0.444463 | 6.69E-29 | 9.14E-28 |
| RP11-259K15.2 | RS1 | 0.468897 | 2.17E-32 | 3.89E-31 |
| RP11-401P9.4 | RS1 | 0.557139 | 1.26E-47 | 6.58E-46 |
| RP11-4B16.3 | RS1 | 0.623411 | 1.78E-62 | 2.79E-60 |
| RP11-401P9.4 | RSPO1 | 0.659798 | 2.90E-72 | 9.34E-70 |
| RP11-4B16.3 | RSPO1 | 0.591506 | 6.75E-55 | 6.14E-53 |
| RP11-259K15.2 | RSPO2 | 0.401317 | 2.16E-23 | 1.81E-22 |
| RP11-401P9.4 | RSPO2 | 0.644504 | 5.49E-68 | 1.25E-65 |
| RP11-4B16.3 | RSPO2 | 0.5326 | 6.24E-43 | 2.39E-41 |
| RP11-401P9.4 | RSPO4 | 0.490469 | 1.03E-35 | 2.37E-34 |
| RP11-4B16.3 | RSPO4 | 0.430109 | 5.58E-27 | 6.47E-26 |
| Z83851.4 | RTKN | 0.418298 | 1.82E-25 | 1.86E-24 |
| RP11-401P9.4 | RTKN2 | 0.646504 | 1.56E-68 | 3.77E-66 |
| RP11-4B16.3 | RTKN2 | 0.681266 | 1.04E-78 | 6.83E-76 |
| RP11-401P9.4 | RTN1 | 0.446198 | 3.86E-29 | 5.37E-28 |
| RP11-4B16.3 | RTN1 | 0.417524 | 2.28E-25 | 2.31E-24 |
| RP11-401P9.4 | RXFP1 | 0.626017 | 3.92E-63 | 6.48E-61 |
| RP11-4B16.3 | RXFP1 | 0.701357 | 2.90E-85 | 3.66E-82 |
| RP11-401P9.4 | RXRG | 0.461242 | 2.88E-31 | 4.76E-30 |
| RP11-4B16.3 | RXRG | 0.473458 | 4.49E-33 | 8.53E-32 |
| RP11-4B16.3 | S100A3 | 0.534037 | 3.39E-43 | 1.32E-41 |
| RP11-401P9.4 | S1PR1 | 0.594155 | 1.71E-55 | 1.60E-53 |
| RP11-4B16.3 | S1PR1 | 0.63258 | 8.08E-65 | 1.55E-62 |
| RP11-259K15.2 | S1PR4 | 0.423545 | 3.94E-26 | 4.26E-25 |
| RP11-401P9.4 | S1PR4 | 0.403347 | 1.24E-23 | 1.06E-22 |
| RP11-4B16.3 | S1PR4 | 0.435716 | 1.02E-27 | 1.27E-26 |
| RP11-259K15.2 | SACM1L | 0.452018 | 5.97E-30 | 8.84E-29 |
| RP11-401P9.4 | SAP30L | 0.501188 | 1.86E-37 | 4.87E-36 |
| RP11-401P9.4 | SASH1 | 0.550382 | 2.70E-46 | 1.29E-44 |
| RP11-4B16.3 | SASH1 | 0.565159 | 3.01E-49 | 1.80E-47 |
| RP11-401P9.4 | SATB1 | 0.478274 | 8.32E-34 | 1.65E-32 |
| RP11-401P9.4 | SBSPON | 0.565741 | 2.29E-49 | 1.38E-47 |
| RP11-4B16.3 | SBSPON | 0.487209 | 3.39E-35 | 7.50E-34 |
| RP11-401P9.4 | SCAI | 0.465686 | 6.47E-32 | 1.12E-30 |
| RP11-4B16.3 | SCAI | 0.413182 | 7.91E-25 | 7.62E-24 |
| RP11-401P9.4 | SCARA3 | 0.504147 | 6.01E-38 | 1.63E-36 |
| RP11-401P9.4 | SCARA5 | 0.505872 | 3.09E-38 | 8.53E-37 |
| RP11-401P9.4 | SCARF1 | 0.419782 | 1.19E-25 | 1.23E-24 |
| RP11-4B16.3 | SCARF1 | 0.439068 | 3.62E-28 | 4.70E-27 |
| RP11-401P9.4 | SCGB1A1 | 0.435104 | 1.23E-27 | 1.52E-26 |
| RP11-259K15.2 | SCGB3A1 | 0.444567 | 6.47E-29 | 8.86E-28 |
| RP11-259K15.2 | SCGB3A2 | 0.521441 | 6.40E-41 | 2.10E-39 |
| RP11-401P9.4 | SCN1A | 0.465617 | 6.62E-32 | 1.15E-30 |
| RP11-4B16.3 | SCN1A | 0.43 | 5.77E-27 | 6.67E-26 |
| RP11-401P9.4 | SCN2B | 0.494373 | 2.43E-36 | 5.91E-35 |
| RP11-259K15.2 | SCN4B | 0.441816 | 1.54E-28 | 2.06E-27 |
| RP11-401P9.4 | SCN4B | 0.723677 | 3.19E-93 | 8.38E-90 |
| RP11-4B16.3 | SCN4B | 0.574957 | 2.74E-51 | 1.90E-49 |
| RP11-259K15.2 | SCN7A | 0.440633 | 2.22E-28 | 2.94E-27 |
| RP11-401P9.4 | SCN7A | 0.691805 | 4.42E-82 | 3.67E-79 |
| RP11-4B16.3 | SCN7A | 0.490543 | 1.00E-35 | 2.31E-34 |
| CTA-384D8.35 | SCNM1 | 0.414726 | 5.09E-25 | 4.98E-24 |
| RP11-259K15.2 | SCNN1B | 0.498051 | 6.12E-37 | 1.55E-35 |
| CTA-384D8.35 | SCO2 | 0.53932 | 3.53E-44 | 1.46E-42 |
| RP11-259K15.2 | SCTR | 0.447143 | 2.86E-29 | 4.01E-28 |
| RP11-401P9.4 | SCUBE1 | 0.628823 | 7.54E-64 | 1.30E-61 |
| RP11-4B16.3 | SCUBE1 | 0.649714 | 2.04E-69 | 5.20E-67 |
| RP11-259K15.2 | SDPR | 0.434296 | 1.57E-27 | 1.92E-26 |
| RP11-401P9.4 | SDPR | 0.572894 | 7.47E-51 | 4.98E-49 |
| RP11-4B16.3 | SDPR | 0.575543 | 2.06E-51 | 1.44E-49 |
| RP11-401P9.4 | SEC14L1 | 0.405939 | 6.05E-24 | 5.33E-23 |
| RP11-401P9.4 | SEC14L3 | 0.42781 | 1.11E-26 | 1.26E-25 |
| RP11-401P9.4 | SEC14L4 | 0.41443 | 5.54E-25 | 5.41E-24 |
| RP11-259K15.2 | SEC14L6 | 0.474087 | 3.61E-33 | 6.88E-32 |
| RP11-401P9.4 | SEC14L6 | 0.510848 | 4.44E-39 | 1.29E-37 |
| RP11-401P9.4 | SEC22C | 0.401581 | 2.01E-23 | 1.69E-22 |
| RP11-401P9.4 | SECISBP2L | 0.505081 | 4.19E-38 | 1.15E-36 |
| RP11-4B16.3 | SECISBP2L | 0.40205 | 1.77E-23 | 1.49E-22 |
| RP11-259K15.2 | SELENBP1 | 0.658927 | 5.16E-72 | 1.60E-69 |
| RP11-401P9.4 | SELENBP1 | 0.418434 | 1.75E-25 | 1.79E-24 |
| RP11-401P9.4 | SELP | 0.596973 | 3.90E-56 | 3.88E-54 |
| RP11-4B16.3 | SELP | 0.503187 | 8.68E-38 | 2.33E-36 |
| RP11-401P9.4 | SEMA3G | 0.632191 | 1.02E-64 | 1.89E-62 |
| RP11-4B16.3 | SEMA3G | 0.738382 | 6.57E-99 | 4.15E-95 |
| RP11-401P9.4 | SEMA5A | 0.584236 | 2.75E-53 | 2.17E-51 |
| RP11-4B16.3 | SEMA5A | 0.50604 | 2.89E-38 | 8.02E-37 |
| RP11-401P9.4 | SEMA6A | 0.57806 | 5.98E-52 | 4.39E-50 |
| RP11-4B16.3 | SEMA6A | 0.646377 | 1.69E-68 | 4.05E-66 |
| RP11-401P9.4 | SEMA6D | 0.547047 | 1.20E-45 | 5.44E-44 |
| RP11-4B16.3 | SEMA6D | 0.490279 | 1.10E-35 | 2.53E-34 |
| RP11-259K15.2 | SEPP1 | 0.416915 | 2.72E-25 | 2.72E-24 |
| RP11-401P9.4 | SEPP1 | 0.411593 | 1.24E-24 | 1.17E-23 |
| RP11-401P9.4 | SERINC1 | 0.505118 | 4.13E-38 | 1.13E-36 |
| RP11-4B16.3 | SERINC1 | 0.428862 | 8.12E-27 | 9.25E-26 |
| RP11-401P9.4 | SERPING1 | 0.422201 | 5.85E-26 | 6.23E-25 |
| RP11-401P9.4 | SERTM1 | 0.614576 | 2.73E-60 | 3.64E-58 |
| RP11-4B16.3 | SERTM1 | 0.667479 | 1.65E-74 | 6.95E-72 |
| RP11-259K15.2 | SESN1 | 0.426578 | 1.61E-26 | 1.79E-25 |
| RP11-401P9.4 | SESN1 | 0.620673 | 8.63E-62 | 1.30E-59 |
| RP11-4B16.3 | SESN1 | 0.484923 | 7.75E-35 | 1.66E-33 |
| RP11-401P9.4 | SETBP1 | 0.469792 | 1.59E-32 | 2.89E-31 |
| RP11-401P9.4 | SFRP5 | 0.504928 | 4.45E-38 | 1.22E-36 |
| RP11-4B16.3 | SFRP5 | 0.434618 | 1.42E-27 | 1.75E-26 |
| RP11-259K15.2 | SFTA2 | 0.552215 | 1.18E-46 | 5.83E-45 |
| RP11-259K15.2 | SFTA3 | 0.631867 | 1.24E-64 | 2.26E-62 |
| RP11-259K15.2 | SFTPA1 | 0.473156 | 4.99E-33 | 9.41E-32 |
| RP11-259K15.2 | SFTPA2 | 0.459481 | 5.18E-31 | 8.33E-30 |
| RP11-259K15.2 | SFTPB | 0.535849 | 1.57E-43 | 6.27E-42 |
| RP11-259K15.2 | SFTPC | 0.48071 | 3.51E-34 | 7.20E-33 |
| RP11-401P9.4 | SFTPC | 0.526786 | 7.12E-42 | 2.51E-40 |
| RP11-4B16.3 | SFTPC | 0.491362 | 7.40E-36 | 1.73E-34 |
| RP11-259K15.2 | SFTPD | 0.572361 | 9.67E-51 | 6.39E-49 |
| RP11-401P9.4 | SFTPD | 0.418073 | 1.95E-25 | 1.98E-24 |
| CTA-384D8.35 | SFXN4 | 0.472747 | 5.75E-33 | 1.08E-31 |
| RP11-401P9.4 | SGCA | 0.631878 | 1.23E-64 | 2.26E-62 |
| RP11-4B16.3 | SGCA | 0.45114 | 7.93E-30 | 1.16E-28 |
| RP11-401P9.4 | SGCG | 0.666204 | 3.94E-74 | 1.57E-71 |
| RP11-4B16.3 | SGCG | 0.654462 | 9.61E-71 | 2.78E-68 |
| RP11-4B16.3 | SH2D1B | 0.458399 | 7.41E-31 | 1.18E-29 |
| RP11-401P9.4 | SH2D3C | 0.62125 | 6.20E-62 | 9.45E-60 |
| RP11-4B16.3 | SH2D3C | 0.672816 | 4.15E-76 | 2.05E-73 |
| RP11-401P9.4 | SH3BP5 | 0.433797 | 1.83E-27 | 2.22E-26 |
| RP11-4B16.3 | SH3BP5 | 0.425343 | 2.32E-26 | 2.56E-25 |
| RP11-401P9.4 | SH3D19 | 0.45657 | 1.35E-30 | 2.11E-29 |
| RP11-4B16.3 | SH3D19 | 0.417635 | 2.21E-25 | 2.24E-24 |
| RP11-401P9.4 | SH3GL3 | 0.59885 | 1.45E-56 | 1.46E-54 |
| RP11-4B16.3 | SH3GL3 | 0.643144 | 1.28E-67 | 2.75E-65 |
| RP11-401P9.4 | SHC3 | 0.523748 | 2.49E-41 | 8.49E-40 |
| RP11-401P9.4 | SHROOM4 | 0.550707 | 2.34E-46 | 1.13E-44 |
| RP11-4B16.3 | SHROOM4 | 0.475842 | 1.96E-33 | 3.81E-32 |
| RP11-401P9.4 | SIDT2 | 0.528466 | 3.54E-42 | 1.29E-40 |
| RP11-401P9.4 | SIGLEC11 | 0.434065 | 1.68E-27 | 2.05E-26 |
| RP11-4B16.3 | SIGLEC11 | 0.485571 | 6.13E-35 | 1.32E-33 |
| RP11-401P9.4 | SIGLEC6 | 0.424268 | 3.19E-26 | 3.47E-25 |
| RP11-401P9.4 | SIK2 | 0.406864 | 4.68E-24 | 4.17E-23 |
| RP11-401P9.4 | SIRPB1 | 0.473199 | 4.92E-33 | 9.29E-32 |
| RP11-4B16.3 | SIRPB1 | 0.497676 | 7.04E-37 | 1.77E-35 |
| RP11-401P9.4 | SKI | 0.450638 | 9.33E-30 | 1.36E-28 |
| RP11-4B16.3 | SLC11A1 | 0.421606 | 6.96E-26 | 7.37E-25 |
| RP11-401P9.4 | SLC12A4 | 0.518449 | 2.15E-40 | 6.84E-39 |
| RP11-4B16.3 | SLC12A4 | 0.428141 | 1.01E-26 | 1.14E-25 |
| RP11-401P9.4 | SLC14A1 | 0.501141 | 1.90E-37 | 4.96E-36 |
| RP11-4B16.3 | SLC14A1 | 0.654201 | 1.14E-70 | 3.27E-68 |
| RP11-259K15.2 | SLC15A2 | 0.469594 | 1.71E-32 | 3.09E-31 |
| RP11-401P9.4 | SLC15A2 | 0.441081 | 1.93E-28 | 2.57E-27 |
| RP11-259K15.2 | SLC16A11 | 0.557453 | 1.09E-47 | 5.73E-46 |
| RP11-401P9.4 | SLC16A11 | 0.550958 | 2.09E-46 | 1.01E-44 |
| RP11-401P9.4 | SLC19A3 | 0.585365 | 1.56E-53 | 1.25E-51 |
| RP11-4B16.3 | SLC19A3 | 0.546931 | 1.26E-45 | 5.72E-44 |
| RP11-401P9.4 | SLC1A1 | 0.455317 | 2.04E-30 | 3.13E-29 |
| RP11-4B16.3 | SLC1A1 | 0.474094 | 3.60E-33 | 6.87E-32 |
| RP11-259K15.2 | SLC22A3 | 0.538487 | 5.06E-44 | 2.09E-42 |
| RP11-401P9.4 | SLC22A3 | 0.405468 | 6.89E-24 | 6.05E-23 |
| RP11-401P9.4 | SLC24A3 | 0.401985 | 1.80E-23 | 1.52E-22 |
| Z83851.4 | SLC25A10 | 0.453705 | 3.45E-30 | 5.20E-29 |
| RP11-401P9.4 | SLC25A25 | 0.411799 | 1.17E-24 | 1.11E-23 |
| CTA-384D8.35 | SLC25A39 | 0.451968 | 6.07E-30 | 8.96E-29 |
| RP11-259K15.2 | SLC27A3 | 0.531588 | 9.57E-43 | 3.63E-41 |
| RP11-401P9.4 | SLC27A3 | 0.440611 | 2.24E-28 | 2.96E-27 |
| RP5-1059L7.1 | SLC2A1 | 0.432843 | 2.44E-27 | 2.93E-26 |
| RP11-259K15.2 | SLC34A2 | 0.436833 | 7.21E-28 | 9.17E-27 |
| CTA-384D8.35 | SLC35B1 | 0.442189 | 1.37E-28 | 1.83E-27 |
| Z83851.4 | SLC35F2 | 0.456291 | 1.48E-30 | 2.30E-29 |
| RP11-259K15.2 | SLC39A8 | 0.453188 | 4.09E-30 | 6.12E-29 |
| RP11-401P9.4 | SLC39A8 | 0.45308 | 4.23E-30 | 6.33E-29 |
| RP11-4B16.3 | SLC39A8 | 0.509628 | 7.16E-39 | 2.06E-37 |
| RP11-401P9.4 | SLC44A2 | 0.443976 | 7.80E-29 | 1.06E-27 |
| RP11-259K15.2 | SLC46A2 | 0.441286 | 1.81E-28 | 2.41E-27 |
| CTA-384D8.35 | SLC50A1 | 0.442603 | 1.20E-28 | 1.62E-27 |
| Z83851.4 | SLC5A6 | 0.40291 | 1.40E-23 | 1.19E-22 |
| RP11-401P9.4 | SLC5A9 | 0.545782 | 2.10E-45 | 9.41E-44 |
| RP11-4B16.3 | SLC5A9 | 0.548478 | 6.33E-46 | 2.94E-44 |
| RP11-401P9.4 | SLC6A4 | 0.635491 | 1.40E-65 | 2.82E-63 |
| RP11-4B16.3 | SLC6A4 | 0.773123 | ####### | ####### |
| RP11-4B16.3 | SLC9A3R2 | 0.408278 | 3.15E-24 | 2.86E-23 |
| RP11-401P9.4 | SLCO2A1 | 0.466794 | 4.44E-32 | 7.80E-31 |
| RP11-4B16.3 | SLCO2A1 | 0.447275 | 2.74E-29 | 3.86E-28 |
| RP11-401P9.4 | SLCO3A1 | 0.425975 | 1.92E-26 | 2.14E-25 |
| RP11-401P9.4 | SLIT2 | 0.680036 | 2.52E-78 | 1.59E-75 |
| RP11-4B16.3 | SLIT2 | 0.553891 | 5.55E-47 | 2.77E-45 |
| RP11-401P9.4 | SLIT3 | 0.668653 | 7.40E-75 | 3.29E-72 |
| RP11-4B16.3 | SLIT3 | 0.521993 | 5.11E-41 | 1.70E-39 |
| RP11-401P9.4 | SMAD6 | 0.644475 | 5.58E-68 | 1.27E-65 |
| RP11-4B16.3 | SMAD6 | 0.659148 | 4.46E-72 | 1.41E-69 |
| RP11-401P9.4 | SMAD7 | 0.518908 | 1.79E-40 | 5.71E-39 |
| RP11-4B16.3 | SMAD7 | 0.449245 | 1.46E-29 | 2.10E-28 |
| RP11-401P9.4 | SMAD9 | 0.523841 | 2.40E-41 | 8.20E-40 |
| RP11-401P9.4 | SMARCA2 | 0.46492 | 8.38E-32 | 1.44E-30 |
| RP11-401P9.4 | SMTNL2 | 0.457744 | 9.19E-31 | 1.45E-29 |
| CTA-384D8.35 | SMUG1 | 0.400101 | 3.01E-23 | 2.49E-22 |
| RP11-401P9.4 | SNRK | 0.56894 | 5.01E-50 | 3.20E-48 |
| RP11-4B16.3 | SNRK | 0.558079 | 8.17E-48 | 4.34E-46 |
| RP11-401P9.4 | SNX1 | 0.547932 | 8.08E-46 | 3.72E-44 |
| RP11-259K15.2 | SNX22 | 0.43049 | 4.98E-27 | 5.79E-26 |
| RP11-401P9.4 | SNX22 | 0.526898 | 6.79E-42 | 2.41E-40 |
| RP11-4B16.3 | SNX22 | 0.469482 | 1.77E-32 | 3.20E-31 |
| RP11-259K15.2 | SNX25 | 0.487518 | 3.03E-35 | 6.72E-34 |
| RP11-401P9.4 | SNX25 | 0.424084 | 3.36E-26 | 3.66E-25 |
| RP11-259K15.2 | SNX30 | 0.450627 | 9.36E-30 | 1.36E-28 |
| RP11-401P9.4 | SNX30 | 0.426045 | 1.88E-26 | 2.09E-25 |
| RP11-401P9.4 | SOBP | 0.501673 | 1.55E-37 | 4.08E-36 |
| RP11-401P9.4 | SORBS1 | 0.645773 | 2.48E-68 | 5.83E-66 |
| RP11-4B16.3 | SORBS1 | 0.452675 | 4.83E-30 | 7.19E-29 |
| RP11-401P9.4 | SORBS3 | 0.460889 | 3.24E-31 | 5.31E-30 |
| RP11-4B16.3 | SORBS3 | 0.439873 | 2.82E-28 | 3.69E-27 |
| RP11-401P9.4 | SORT1 | 0.425202 | 2.42E-26 | 2.66E-25 |
| RP11-401P9.4 | SOSTDC1 | 0.562705 | 9.54E-49 | 5.54E-47 |
| RP11-4B16.3 | SOSTDC1 | 0.607821 | 1.15E-58 | 1.36E-56 |
| RP11-401P9.4 | SOX13 | 0.443871 | 8.06E-29 | 1.10E-27 |
| RP11-401P9.4 | SOX17 | 0.549194 | 4.60E-46 | 2.16E-44 |
| RP11-4B16.3 | SOX17 | 0.590884 | 9.31E-55 | 8.39E-53 |
| RP11-401P9.4 | SOX18 | 0.456377 | 1.44E-30 | 2.24E-29 |
| RP11-401P9.4 | SOX5 | 0.588426 | 3.29E-54 | 2.83E-52 |
| RP11-4B16.3 | SOX5 | 0.449788 | 1.23E-29 | 1.77E-28 |
| RP11-401P9.4 | SOX7 | 0.590298 | 1.26E-54 | 1.13E-52 |
| RP11-4B16.3 | SOX7 | 0.599452 | 1.05E-56 | 1.07E-54 |
| CTA-384D8.35 | SPAG4 | 0.464993 | 8.17E-32 | 1.41E-30 |
| RP11-401P9.4 | SPARCL1 | 0.58853 | 3.12E-54 | 2.70E-52 |
| RP11-4B16.3 | SPARCL1 | 0.423685 | 3.78E-26 | 4.09E-25 |
| RP11-401P9.4 | SPATA18 | 0.448835 | 1.66E-29 | 2.38E-28 |
| RP11-401P9.4 | SPN | 0.544253 | 4.11E-45 | 1.81E-43 |
| RP11-4B16.3 | SPN | 0.497478 | 7.59E-37 | 1.91E-35 |
| RP5-1059L7.1 | SPOCK1 | 0.429404 | 6.90E-27 | 7.91E-26 |
| RP11-401P9.4 | SPOCK2 | 0.606482 | 2.39E-58 | 2.71E-56 |
| RP11-4B16.3 | SPOCK2 | 0.577522 | 7.79E-52 | 5.66E-50 |
| RP11-4B16.3 | SPRYD7 | 0.45368 | 3.48E-30 | 5.24E-29 |
| RP11-401P9.4 | SPTAN1 | 0.400594 | 2.63E-23 | 2.19E-22 |
| RP11-401P9.4 | SPTBN1 | 0.563804 | 5.70E-49 | 3.33E-47 |
| RP11-4B16.3 | SPTBN1 | 0.555961 | 2.16E-47 | 1.12E-45 |
| RP11-401P9.4 | SRGAP2C | 0.407647 | 3.76E-24 | 3.39E-23 |
| RP11-4B16.3 | SRGAP2C | 0.447754 | 2.35E-29 | 3.33E-28 |
| RP11-401P9.4 | SRPX | 0.460936 | 3.19E-31 | 5.24E-30 |
| RP11-4B16.3 | SRPX | 0.431644 | 3.51E-27 | 4.15E-26 |
| RP11-401P9.4 | SSFA2 | 0.415184 | 4.47E-25 | 4.39E-24 |
| RP11-4B16.3 | SSFA2 | 0.426128 | 1.84E-26 | 2.04E-25 |
| CTA-384D8.35 | SSR4 | 0.475086 | 2.55E-33 | 4.92E-32 |
| RP11-401P9.4 | SSTR1 | 0.600093 | 7.47E-57 | 7.68E-55 |
| RP11-4B16.3 | SSTR1 | 0.516818 | 4.14E-40 | 1.29E-38 |
| RP11-401P9.4 | ST6GALNAC3 | 0.432337 | 2.85E-27 | 3.39E-26 |
| RP11-4B16.3 | ST6GALNAC3 | 0.48036 | 3.97E-34 | 8.10E-33 |
| RP11-401P9.4 | ST6GALNAC5 | 0.417289 | 2.44E-25 | 2.46E-24 |
| RP11-4B16.3 | ST6GALNAC5 | 0.495414 | 1.65E-36 | 4.05E-35 |
| RP11-259K15.2 | ST6GALNAC6 | 0.557917 | 8.80E-48 | 4.66E-46 |
| RP11-401P9.4 | ST6GALNAC6 | 0.535711 | 1.66E-43 | 6.63E-42 |
| RP11-4B16.3 | ST6GALNAC6 | 0.468291 | 2.67E-32 | 4.75E-31 |
| RP11-259K15.2 | ST7 | 0.489169 | 1.66E-35 | 3.75E-34 |
| RP11-401P9.4 | ST8SIA6 | 0.588758 | 2.77E-54 | 2.42E-52 |
| RP11-4B16.3 | ST8SIA6 | 0.641605 | 3.33E-67 | 7.06E-65 |
| RP11-4B16.3 | STAC | 0.459151 | 5.77E-31 | 9.27E-30 |
| RP11-401P9.4 | STARD13 | 0.633135 | 5.80E-65 | 1.13E-62 |
| RP11-4B16.3 | STARD13 | 0.60781 | 1.16E-58 | 1.37E-56 |
| RP11-401P9.4 | STARD8 | 0.557982 | 8.54E-48 | 4.53E-46 |
| RP11-4B16.3 | STARD8 | 0.653753 | 1.52E-70 | 4.29E-68 |
| RP11-401P9.4 | STARD9 | 0.639674 | 1.09E-66 | 2.26E-64 |
| RP11-4B16.3 | STARD9 | 0.559936 | 3.47E-48 | 1.92E-46 |
| RP11-401P9.4 | STON1 | 0.420991 | 8.33E-26 | 8.74E-25 |
| RP11-401P9.4 | STX11 | 0.49654 | 1.08E-36 | 2.69E-35 |
| RP11-4B16.3 | STX11 | 0.607801 | 1.17E-58 | 1.37E-56 |
| RP11-259K15.2 | STX12 | 0.411843 | 1.16E-24 | 1.10E-23 |
| RP11-4B16.3 | STX12 | 0.469936 | 1.52E-32 | 2.75E-31 |
| RP11-4B16.3 | STX2 | 0.411106 | 1.42E-24 | 1.34E-23 |
| RP11-401P9.4 | STXBP6 | 0.615588 | 1.55E-60 | 2.12E-58 |
| RP11-4B16.3 | STXBP6 | 0.668076 | 1.10E-74 | 4.82E-72 |
| Z83851.4 | STYK1 | 0.406453 | 5.25E-24 | 4.65E-23 |
| RP5-1059L7.1 | SULF1 | 0.41876 | 1.59E-25 | 1.63E-24 |
| RP11-259K15.2 | SULT1A1 | 0.471093 | 1.02E-32 | 1.88E-31 |
| RP11-259K15.2 | SULT1A2 | 0.465281 | 7.41E-32 | 1.28E-30 |
| RP11-401P9.4 | SULT1C4 | 0.632004 | 1.14E-64 | 2.11E-62 |
| RP11-4B16.3 | SULT1C4 | 0.580707 | 1.61E-52 | 1.22E-50 |
| RP11-401P9.4 | SUN2 | 0.411528 | 1.26E-24 | 1.19E-23 |
| RP11-259K15.2 | SUSD2 | 0.595277 | 9.49E-56 | 9.08E-54 |
| RP11-401P9.4 | SUSD2 | 0.47002 | 1.47E-32 | 2.68E-31 |
| RP11-401P9.4 | SUSD6 | 0.471357 | 9.30E-33 | 1.72E-31 |
| RP11-401P9.4 | SVEP1 | 0.598544 | 1.70E-56 | 1.71E-54 |
| RP11-4B16.3 | SVEP1 | 0.461856 | 2.35E-31 | 3.91E-30 |
| RP11-4B16.3 | SYN2 | 0.467787 | 3.17E-32 | 5.61E-31 |
| RP11-401P9.4 | SYNC | 0.55156 | 1.59E-46 | 7.77E-45 |
| RP11-4B16.3 | SYNC | 0.523773 | 2.47E-41 | 8.42E-40 |
| RP11-259K15.2 | SYNDIG1L | 0.402095 | 1.75E-23 | 1.48E-22 |
| RP11-401P9.4 | SYNDIG1L | 0.440793 | 2.11E-28 | 2.80E-27 |
| RP11-259K15.2 | SYNE1 | 0.410471 | 1.70E-24 | 1.59E-23 |
| RP11-401P9.4 | SYNE1 | 0.488665 | 1.99E-35 | 4.48E-34 |
| RP11-401P9.4 | SYNE3 | 0.611661 | 1.39E-59 | 1.75E-57 |
| RP11-4B16.3 | SYNE3 | 0.47513 | 2.51E-33 | 4.85E-32 |
| RP11-401P9.4 | SYNJ2BP | 0.446343 | 3.69E-29 | 5.13E-28 |
| RP11-401P9.4 | SYNM | 0.524186 | 2.08E-41 | 7.16E-40 |
| RP11-4B16.3 | SYNM | 0.591538 | 6.64E-55 | 6.06E-53 |
| RP11-401P9.4 | SYNPO | 0.591352 | 7.31E-55 | 6.63E-53 |
| RP11-4B16.3 | SYNPO | 0.457663 | 9.44E-31 | 1.49E-29 |
| RP11-401P9.4 | SYNPO2 | 0.620083 | 1.21E-61 | 1.78E-59 |
| RP11-4B16.3 | SYNPO2 | 0.455358 | 2.01E-30 | 3.09E-29 |
| RP11-259K15.2 | SYT15 | 0.429721 | 6.28E-27 | 7.23E-26 |
| RP11-401P9.4 | SYT15 | 0.639747 | 1.05E-66 | 2.18E-64 |
| RP11-4B16.3 | SYT15 | 0.583366 | 4.27E-53 | 3.31E-51 |
| RP11-401P9.4 | TACC1 | 0.641985 | 2.63E-67 | 5.61E-65 |
| RP11-4B16.3 | TACC1 | 0.507342 | 1.75E-38 | 4.92E-37 |
| Z83851.4 | TACC3 | 0.400575 | 2.65E-23 | 2.20E-22 |
| RP11-401P9.4 | TAL1 | 0.677042 | 2.13E-77 | 1.24E-74 |
| RP11-4B16.3 | TAL1 | 0.720813 | 3.70E-92 | 8.98E-89 |
| RP11-259K15.2 | TAPT1 | 0.522634 | 3.93E-41 | 1.32E-39 |
| RP11-401P9.4 | TAPT1 | 0.471911 | 7.68E-33 | 1.43E-31 |
| RP11-401P9.4 | TBC1D2B | 0.432537 | 2.68E-27 | 3.20E-26 |
| RP11-401P9.4 | TBC1D4 | 0.436542 | 7.89E-28 | 1.00E-26 |
| Z83851.4 | TBRG4 | 0.447296 | 2.72E-29 | 3.83E-28 |
| CTA-384D8.35 | TBX15 | 0.440993 | 1.99E-28 | 2.64E-27 |
| RP11-401P9.4 | TBX2 | 0.708802 | 7.79E-88 | 1.37E-84 |
| RP11-4B16.3 | TBX2 | 0.54094 | 1.75E-44 | 7.42E-43 |
| RP11-401P9.4 | TBX3 | 0.676293 | 3.62E-77 | 2.08E-74 |
| RP11-4B16.3 | TBX3 | 0.614291 | 3.21E-60 | 4.22E-58 |
| RP11-259K15.2 | TBX4 | 0.423626 | 3.85E-26 | 4.16E-25 |
| RP11-401P9.4 | TBX4 | 0.71541 | 3.47E-90 | 7.53E-87 |
| RP11-4B16.3 | TBX4 | 0.528998 | 2.83E-42 | 1.05E-40 |
| RP11-401P9.4 | TBX5 | 0.661729 | 8.02E-73 | 2.81E-70 |
| RP11-4B16.3 | TBX5 | 0.469953 | 1.51E-32 | 2.74E-31 |
| RP11-401P9.4 | TCEAL2 | 0.508694 | 1.03E-38 | 2.94E-37 |
| RP11-4B16.3 | TCEAL2 | 0.496337 | 1.16E-36 | 2.89E-35 |
| CTA-384D8.35 | TCEB1 | 0.404614 | 8.73E-24 | 7.58E-23 |
| RP11-259K15.2 | TCF21 | 0.411534 | 1.26E-24 | 1.19E-23 |
| RP11-401P9.4 | TCF21 | 0.712241 | 4.74E-89 | 8.81E-86 |
| RP11-4B16.3 | TCF21 | 0.632459 | 8.69E-65 | 1.62E-62 |
| RP11-401P9.4 | TCF4 | 0.5281 | 4.12E-42 | 1.50E-40 |
| RP11-401P9.4 | TCP11L2 | 0.425933 | 1.95E-26 | 2.16E-25 |
| RP11-401P9.4 | TEF | 0.476863 | 1.37E-33 | 2.68E-32 |
| RP11-401P9.4 | TEK | 0.653106 | 2.31E-70 | 6.40E-68 |
| RP11-4B16.3 | TEK | 0.693928 | 8.88E-83 | 8.76E-80 |
| RP11-401P9.4 | TGFB2 | 0.412713 | 9.04E-25 | 8.68E-24 |
| RP11-401P9.4 | TGFBR2 | 0.547914 | 8.14E-46 | 3.74E-44 |
| RP11-4B16.3 | TGFBR2 | 0.503226 | 8.55E-38 | 2.30E-36 |
| RP11-401P9.4 | TGFBR3 | 0.682833 | 3.34E-79 | 2.28E-76 |
| RP11-4B16.3 | TGFBR3 | 0.589098 | 2.33E-54 | 2.05E-52 |
| RP11-401P9.4 | TGM1 | 0.46373 | 1.25E-31 | 2.13E-30 |
| RP11-4B16.3 | TGM1 | 0.47777 | 9.93E-34 | 1.95E-32 |
| RP11-401P9.4 | THBD | 0.511 | 4.18E-39 | 1.22E-37 |
| RP11-4B16.3 | THBD | 0.538033 | 6.15E-44 | 2.51E-42 |
| RP5-1059L7.1 | THBS2 | 0.424605 | 2.88E-26 | 3.15E-25 |
| RP11-401P9.4 | THRA | 0.521514 | 6.21E-41 | 2.05E-39 |
| RP11-401P9.4 | THSD1 | 0.662917 | 3.62E-73 | 1.30E-70 |
| RP11-4B16.3 | THSD1 | 0.65289 | 2.66E-70 | 7.30E-68 |
| RP11-401P9.4 | THSD4 | 0.43745 | 5.96E-28 | 7.65E-27 |
| RP11-401P9.4 | TIE1 | 0.588211 | 3.67E-54 | 3.13E-52 |
| RP11-4B16.3 | TIE1 | 0.589355 | 2.04E-54 | 1.80E-52 |
| RP11-401P9.4 | TIMP3 | 0.436541 | 7.89E-28 | 1.00E-26 |
| RP11-4B16.3 | TIMP3 | 0.419118 | 1.44E-25 | 1.48E-24 |
| RP11-401P9.4 | TJP1 | 0.407059 | 4.43E-24 | 3.96E-23 |
| RP11-4B16.3 | TJP1 | 0.416966 | 2.68E-25 | 2.68E-24 |
| Z83851.4 | TK1 | 0.492869 | 4.24E-36 | 1.02E-34 |
| Z83851.4 | TLCD1 | 0.410716 | 1.59E-24 | 1.48E-23 |
| RP11-401P9.4 | TLL1 | 0.459049 | 5.97E-31 | 9.58E-30 |
| RP11-4B16.3 | TLR4 | 0.407817 | 3.59E-24 | 3.23E-23 |
| RP11-401P9.4 | TM6SF1 | 0.409858 | 2.02E-24 | 1.87E-23 |
| RP11-4B16.3 | TM6SF1 | 0.458565 | 7.01E-31 | 1.12E-29 |
| RP11-259K15.2 | TMEM100 | 0.405985 | 5.97E-24 | 5.27E-23 |
| RP11-401P9.4 | TMEM100 | 0.579626 | 2.76E-52 | 2.07E-50 |
| RP11-4B16.3 | TMEM100 | 0.684493 | 9.98E-80 | 7.01E-77 |
| RP11-259K15.2 | TMEM125 | 0.647257 | 9.71E-69 | 2.40E-66 |
| RP11-4B16.3 | TMEM150B | 0.440262 | 2.50E-28 | 3.28E-27 |
| Z83851.4 | TMEM177 | 0.403304 | 1.25E-23 | 1.07E-22 |
| Z83851.4 | TMEM184A | 0.441312 | 1.80E-28 | 2.40E-27 |
| RP11-401P9.4 | TMEM204 | 0.5763 | 1.42E-51 | 1.01E-49 |
| RP11-4B16.3 | TMEM204 | 0.549938 | 3.30E-46 | 1.57E-44 |
| RP11-401P9.4 | TMEM220 | 0.460013 | 4.34E-31 | 7.03E-30 |
| RP11-259K15.2 | TMEM243 | 0.573675 | 5.11E-51 | 3.47E-49 |
| RP11-401P9.4 | TMEM255B | 0.421925 | 6.34E-26 | 6.73E-25 |
| RP11-401P9.4 | TMEM47 | 0.578535 | 4.73E-52 | 3.51E-50 |
| RP11-4B16.3 | TMEM47 | 0.443398 | 9.35E-29 | 1.27E-27 |
| RP11-401P9.4 | TMEM74B | 0.416138 | 3.40E-25 | 3.38E-24 |
| RP11-401P9.4 | TMEM88 | 0.517495 | 3.16E-40 | 9.87E-39 |
| RP11-4B16.3 | TMEM88 | 0.531872 | 8.49E-43 | 3.22E-41 |
| RP11-401P9.4 | TMOD1 | 0.441452 | 1.72E-28 | 2.30E-27 |
| RP11-401P9.4 | TMOD2 | 0.435018 | 1.26E-27 | 1.55E-26 |
| RP11-4B16.3 | TMOD2 | 0.411213 | 1.38E-24 | 1.30E-23 |
| RP11-259K15.2 | TMPRSS2 | 0.493657 | 3.17E-36 | 7.64E-35 |
| CTA-384D8.35 | TNFRSF18 | 0.466842 | 4.37E-32 | 7.68E-31 |
| RP11-401P9.4 | TNFRSF19 | 0.464552 | 9.48E-32 | 1.63E-30 |
| RP11-259K15.2 | TNFSF12 | 0.435587 | 1.06E-27 | 1.32E-26 |
| RP11-401P9.4 | TNFSF12 | 0.414431 | 5.54E-25 | 5.41E-24 |
| RP11-259K15.2 | TNFSF13 | 0.459611 | 4.96E-31 | 7.99E-30 |
| RP11-401P9.4 | TNFSF13 | 0.408875 | 2.67E-24 | 2.44E-23 |
| RP11-259K15.2 | TNNC1 | 0.496806 | 9.77E-37 | 2.44E-35 |
| RP11-401P9.4 | TNNC1 | 0.504009 | 6.33E-38 | 1.71E-36 |
| RP11-4B16.3 | TNNC1 | 0.536188 | 1.36E-43 | 5.45E-42 |
| RP11-401P9.4 | TNR | 0.528908 | 2.94E-42 | 1.08E-40 |
| RP11-4B16.3 | TNR | 0.655013 | 6.72E-71 | 1.96E-68 |
| RP11-259K15.2 | TNS1 | 0.422485 | 5.38E-26 | 5.75E-25 |
| RP11-401P9.4 | TNS1 | 0.68899 | 3.63E-81 | 2.94E-78 |
| RP11-4B16.3 | TNS1 | 0.488803 | 1.89E-35 | 4.26E-34 |
| RP11-259K15.2 | TNS2 | 0.429835 | 6.06E-27 | 7.00E-26 |
| RP11-401P9.4 | TNS2 | 0.577354 | 8.47E-52 | 6.13E-50 |
| RP11-4B16.3 | TNS2 | 0.43627 | 8.58E-28 | 1.08E-26 |
| RP11-401P9.4 | TNS3 | 0.430898 | 4.40E-27 | 5.15E-26 |
| RP11-4B16.3 | TNS3 | 0.406572 | 5.07E-24 | 4.51E-23 |
| RP11-401P9.4 | TNXB | 0.644728 | 4.77E-68 | 1.10E-65 |
| RP11-4B16.3 | TNXB | 0.524078 | 2.18E-41 | 7.47E-40 |
| RP11-401P9.4 | TOM1L2 | 0.601184 | 4.17E-57 | 4.36E-55 |
| RP11-4B16.3 | TOM1L2 | 0.468657 | 2.35E-32 | 4.21E-31 |
| RP11-401P9.4 | TOX2 | 0.457919 | 8.68E-31 | 1.37E-29 |
| RP11-4B16.3 | TOX2 | 0.472075 | 7.26E-33 | 1.35E-31 |
| RP11-259K15.2 | TPPP | 0.485885 | 5.47E-35 | 1.19E-33 |
| RP11-401P9.4 | TPPP | 0.515755 | 6.34E-40 | 1.94E-38 |
| RP11-401P9.4 | TPPP3 | 0.543583 | 5.52E-45 | 2.42E-43 |
| RP11-4B16.3 | TPPP3 | 0.413772 | 6.69E-25 | 6.48E-24 |
| RP11-401P9.4 | TPRG1 | 0.412356 | 1.00E-24 | 9.56E-24 |
| RP11-401P9.4 | TPSAB1 | 0.460613 | 3.55E-31 | 5.80E-30 |
| RP11-401P9.4 | TPSB2 | 0.401266 | 2.19E-23 | 1.83E-22 |
| RP5-1059L7.1 | TPX2 | 0.402641 | 1.50E-23 | 1.28E-22 |
| Z83851.4 | TPX2 | 0.415418 | 4.18E-25 | 4.11E-24 |
| RP11-401P9.4 | TRAK2 | 0.40476 | 8.39E-24 | 7.29E-23 |
| RP11-401P9.4 | TRHDE | 0.41012 | 1.88E-24 | 1.74E-23 |
| RP11-4B16.3 | TRHDE | 0.432673 | 2.57E-27 | 3.08E-26 |
| Z83851.4 | TRMU | 0.441683 | 1.60E-28 | 2.14E-27 |
| CTA-384D8.35 | TRPT1 | 0.414746 | 5.06E-25 | 4.96E-24 |
| RP11-401P9.4 | TRPV2 | 0.433999 | 1.72E-27 | 2.09E-26 |
| RP11-4B16.3 | TRPV2 | 0.489659 | 1.38E-35 | 3.15E-34 |
| RP11-259K15.2 | TSC22D3 | 0.400581 | 2.64E-23 | 2.20E-22 |
| RP11-401P9.4 | TSPAN18 | 0.561992 | 1.33E-48 | 7.56E-47 |
| RP11-4B16.3 | TSPAN18 | 0.541369 | 1.45E-44 | 6.20E-43 |
| RP11-259K15.2 | TSPAN4 | 0.429365 | 6.98E-27 | 8.00E-26 |
| RP11-401P9.4 | TSPAN7 | 0.461642 | 2.52E-31 | 4.19E-30 |
| RP11-4B16.3 | TSPAN7 | 0.406511 | 5.16E-24 | 4.58E-23 |
| RP11-401P9.4 | TSPAN9 | 0.417906 | 2.04E-25 | 2.07E-24 |
| RP11-401P9.4 | TSPYL1 | 0.481213 | 2.93E-34 | 6.05E-33 |
| RP11-401P9.4 | TTC28 | 0.514968 | 8.68E-40 | 2.64E-38 |
| Z83851.4 | TTLL12 | 0.434249 | 1.59E-27 | 1.94E-26 |
| RP11-401P9.4 | TTLL7 | 0.422203 | 5.85E-26 | 6.23E-25 |
| RP11-401P9.4 | TUBB1 | 0.482467 | 1.87E-34 | 3.92E-33 |
| RP11-4B16.3 | TUBB1 | 0.575808 | 1.81E-51 | 1.27E-49 |
| RP5-1059L7.1 | TUBB3 | 0.470573 | 1.22E-32 | 2.23E-31 |
| CTA-384D8.35 | TXNDC17 | 0.429399 | 6.91E-27 | 7.92E-26 |
| CTA-384D8.35 | TYMP | 0.558876 | 5.66E-48 | 3.05E-46 |
| RP11-401P9.4 | TYRP1 | 0.41992 | 1.14E-25 | 1.18E-24 |
| RP11-401P9.4 | UACA | 0.461855 | 2.35E-31 | 3.91E-30 |
| RP11-4B16.3 | UBASH3B | 0.400574 | 2.65E-23 | 2.20E-22 |
| CTA-384D8.35 | UBE2C | 0.402181 | 1.71E-23 | 1.44E-22 |
| RP5-1059L7.1 | UBE2C | 0.412158 | 1.06E-24 | 1.01E-23 |
| CTA-384D8.35 | UBE2T | 0.413536 | 7.15E-25 | 6.92E-24 |
| RP11-401P9.4 | UBL3 | 0.490999 | 8.46E-36 | 1.96E-34 |
| RP11-259K15.2 | UBTD1 | 0.547751 | 8.76E-46 | 4.01E-44 |
| RP11-401P9.4 | ULK2 | 0.433459 | 2.03E-27 | 2.45E-26 |
| RP11-259K15.2 | UNC13B | 0.424957 | 2.60E-26 | 2.85E-25 |
| RP11-401P9.4 | UPK3B | 0.568603 | 5.88E-50 | 3.73E-48 |
| RP11-4B16.3 | UPK3B | 0.602707 | 1.84E-57 | 1.97E-55 |
| CTA-384D8.35 | UQCC2 | 0.42482 | 2.71E-26 | 2.96E-25 |
| RP11-401P9.4 | USHBP1 | 0.677881 | 1.17E-77 | 7.12E-75 |
| RP11-4B16.3 | USHBP1 | 0.731741 | 2.70E-96 | 9.48E-93 |
| RP11-401P9.4 | USP12 | 0.505989 | 2.95E-38 | 8.17E-37 |
| RP11-4B16.3 | USP12 | 0.408325 | 3.11E-24 | 2.83E-23 |
| RP11-401P9.4 | UTRN | 0.534455 | 2.84E-43 | 1.12E-41 |
| RP11-4B16.3 | UTRN | 0.431096 | 4.15E-27 | 4.87E-26 |
| RP11-259K15.2 | VAMP2 | 0.471194 | 9.84E-33 | 1.82E-31 |
| RP11-401P9.4 | VAMP2 | 0.612908 | 6.95E-60 | 8.91E-58 |
| RP11-401P9.4 | VASH1 | 0.521651 | 5.87E-41 | 1.94E-39 |
| RP11-259K15.2 | VEPH1 | 0.50071 | 2.24E-37 | 5.80E-36 |
| RP11-401P9.4 | VEPH1 | 0.484948 | 7.68E-35 | 1.64E-33 |
| RP11-4B16.3 | VEPH1 | 0.481614 | 2.54E-34 | 5.27E-33 |
| RP11-401P9.4 | VGLL3 | 0.406768 | 4.81E-24 | 4.28E-23 |
| RP11-259K15.2 | VIPR1 | 0.470942 | 1.07E-32 | 1.98E-31 |
| RP11-401P9.4 | VIPR1 | 0.620247 | 1.10E-61 | 1.63E-59 |
| RP11-4B16.3 | VIPR1 | 0.664806 | 1.02E-73 | 3.96E-71 |
| CTA-384D8.35 | VMP1 | 0.4934 | 3.48E-36 | 8.38E-35 |
| RP11-259K15.2 | VSIG2 | 0.492166 | 5.50E-36 | 1.30E-34 |
| RP11-401P9.4 | VSIG2 | 0.465688 | 6.46E-32 | 1.12E-30 |
| RP11-4B16.3 | VSIG4 | 0.410281 | 1.80E-24 | 1.67E-23 |
| RP11-401P9.4 | VSTM4 | 0.467643 | 3.33E-32 | 5.88E-31 |
| RP11-401P9.4 | VWF | 0.593236 | 2.75E-55 | 2.57E-53 |
| RP11-4B16.3 | VWF | 0.482985 | 1.56E-34 | 3.28E-33 |
| RP11-401P9.4 | WASF3 | 0.578316 | 5.27E-52 | 3.88E-50 |
| RP11-4B16.3 | WASF3 | 0.401966 | 1.81E-23 | 1.53E-22 |
| Z83851.4 | WDR4 | 0.406285 | 5.50E-24 | 4.86E-23 |
| RP11-401P9.4 | WFDC1 | 0.607534 | 1.35E-58 | 1.57E-56 |
| RP11-4B16.3 | WFDC1 | 0.492633 | 4.63E-36 | 1.11E-34 |
| CTA-384D8.35 | WFDC3 | 0.409464 | 2.26E-24 | 2.08E-23 |
| RP11-401P9.4 | WFS1 | 0.542118 | 1.05E-44 | 4.51E-43 |
| RP11-4B16.3 | WFS1 | 0.486412 | 4.52E-35 | 9.91E-34 |
| RP11-259K15.2 | WIF1 | 0.453536 | 3.65E-30 | 5.48E-29 |
| RP11-401P9.4 | WIF1 | 0.551194 | 1.88E-46 | 9.14E-45 |
| RP11-4B16.3 | WIF1 | 0.408805 | 2.72E-24 | 2.48E-23 |
| RP11-401P9.4 | WISP2 | 0.432465 | 2.74E-27 | 3.27E-26 |
| RP11-401P9.4 | WNT11 | 0.51968 | 1.31E-40 | 4.22E-39 |
| RP11-401P9.4 | WNT3A | 0.634457 | 2.62E-65 | 5.14E-63 |
| RP11-4B16.3 | WNT3A | 0.652428 | 3.58E-70 | 9.67E-68 |
| RP11-401P9.4 | WNT7A | 0.402118 | 1.73E-23 | 1.47E-22 |
| RP11-4B16.3 | WNT7A | 0.440153 | 2.58E-28 | 3.39E-27 |
| RP11-401P9.4 | WWC2 | 0.556037 | 2.09E-47 | 1.08E-45 |
| RP11-4B16.3 | WWC2 | 0.596034 | 6.39E-56 | 6.19E-54 |
| RP11-401P9.4 | WWC3 | 0.432915 | 2.39E-27 | 2.87E-26 |
| Z83851.4 | YDJC | 0.458907 | 6.26E-31 | 1.00E-29 |
| Z83851.4 | YKT6 | 0.434222 | 1.61E-27 | 1.96E-26 |
| RP11-4B16.3 | ZBED2 | 0.448964 | 1.60E-29 | 2.29E-28 |
| RP11-401P9.4 | ZBTB16 | 0.448142 | 2.08E-29 | 2.96E-28 |
| RP11-4B16.3 | ZBTB16 | 0.406929 | 4.59E-24 | 4.10E-23 |
| RP11-259K15.2 | ZBTB4 | 0.416224 | 3.31E-25 | 3.30E-24 |
| RP11-401P9.4 | ZBTB4 | 0.518194 | 2.38E-40 | 7.56E-39 |
| RP11-401P9.4 | ZBTB47 | 0.480791 | 3.41E-34 | 7.00E-33 |
| RP11-4B16.3 | ZBTB47 | 0.415177 | 4.48E-25 | 4.40E-24 |
| RP11-401P9.4 | ZCCHC24 | 0.596803 | 4.26E-56 | 4.22E-54 |
| RP11-401P9.4 | ZEB1 | 0.59266 | 3.71E-55 | 3.45E-53 |
| RP11-4B16.3 | ZEB1 | 0.488599 | 2.04E-35 | 4.58E-34 |
| RP11-401P9.4 | ZEB2 | 0.522547 | 4.07E-41 | 1.36E-39 |
| RP11-4B16.3 | ZEB2 | 0.493885 | 2.91E-36 | 7.05E-35 |
| RP11-401P9.4 | ZFYVE9 | 0.461533 | 2.61E-31 | 4.34E-30 |
| RP11-401P9.4 | ZHX3 | 0.475062 | 2.57E-33 | 4.96E-32 |
| RP11-401P9.4 | ZMIZ1 | 0.505866 | 3.09E-38 | 8.54E-37 |
| RP11-259K15.2 | ZMYND15 | 0.460478 | 3.72E-31 | 6.06E-30 |
| RP11-401P9.4 | ZMYND15 | 0.426858 | 1.48E-26 | 1.65E-25 |
| RP11-401P9.4 | ZNF106 | 0.549156 | 4.68E-46 | 2.20E-44 |
| RP11-4B16.3 | ZNF106 | 0.44017 | 2.57E-28 | 3.37E-27 |
| RP11-401P9.4 | ZNF25 | 0.460332 | 3.90E-31 | 6.35E-30 |
| RP11-4B16.3 | ZNF365 | 0.406364 | 5.38E-24 | 4.76E-23 |
| RP11-401P9.4 | ZNF366 | 0.620632 | 8.83E-62 | 1.33E-59 |
| RP11-4B16.3 | ZNF366 | 0.594304 | 1.58E-55 | 1.49E-53 |
| RP11-259K15.2 | ZNF385B | 0.541989 | 1.11E-44 | 4.76E-43 |
| RP11-401P9.4 | ZNF423 | 0.600272 | 6.79E-57 | 7.01E-55 |
| RP11-4B16.3 | ZNF423 | 0.513332 | 1.66E-39 | 4.97E-38 |
| RP11-401P9.4 | ZNF704 | 0.523835 | 2.40E-41 | 8.22E-40 |
| Z83851.4 | ZWINT | 0.416294 | 3.25E-25 | 3.24E-24 |
