# Supplementary material for: Prognostic value of lncRNAs related to fatty acid metabolism in lung adenocarcinoma and their correlation with tumor microenvironment based on bioinformatics analysis
Source: Front Oncol. 2022 Oct 10;12:1022097. doi: 10.3389/fonc.2022.1022097 (PMC9590110; doi:10.3389/fonc.2022.1022097)
Supplement: Supplementary Table 1 — All samples were divided into high and low fatty acid metabolism score groups based on the median value of this score. [file DataSheet_1.zip › raw data and R code for checking/raw data/13.docx]

| lnc | miRNA | mRNA | type.lnc | type.mi | type.m |
| --- | --- | --- | --- | --- | --- |
| RP11-401P9.4 | hsa-let-7a-2-3p | STARD8 | down.lnc | up.mi | down.m |
| RP11-401P9.4 | hsa-let-7a-2-3p | ZMIZ1 | down.lnc | up.mi | down.m |
| RP11-401P9.4 | hsa-let-7a-2-3p | NFIX | down.lnc | up.mi | down.m |
| RP11-401P9.4 | hsa-let-7a-2-3p | OLR1 | down.lnc | up.mi | down.m |
| RP11-401P9.4 | hsa-let-7a-2-3p | PIAS1 | down.lnc | up.mi | down.m |
| RP11-401P9.4 | hsa-let-7a-2-3p | ZNF704 | down.lnc | up.mi | down.m |
| RP11-259K15.2 | hsa-miR-15a-5p | CREBRF | down.lnc | up.mi | down.m |
| RP11-401P9.4 | hsa-let-7a-2-3p | DLC1 | down.lnc | up.mi | down.m |
| CTA-384D8.35 | hsa-let-7b-5p | PTTG1 | up.lnc | down.mi | up.m |
| CTA-384D8.35 | hsa-let-7b-5p | PUS1 | up.lnc | down.mi | up.m |
| CTA-384D8.35 | hsa-let-7b-5p | FLAD1 | up.lnc | down.mi | up.m |
| RP11-401P9.4 | hsa-let-7c-3p | OLR1 | down.lnc | up.mi | down.m |
| RP11-401P9.4 | hsa-let-7c-3p | SLC24A3 | down.lnc | up.mi | down.m |
| RP11-401P9.4 | hsa-let-7c-3p | CALM1 | down.lnc | up.mi | down.m |
| RP11-401P9.4 | hsa-let-7c-3p | ZMIZ1 | down.lnc | up.mi | down.m |
| RP11-401P9.4 | hsa-let-7c-3p | FOXP1 | down.lnc | up.mi | down.m |
| RP11-259K15.2 | hsa-miR-15a-5p | CADM1 | down.lnc | up.mi | down.m |
| RP11-401P9.4 | hsa-let-7c-3p | FOXO1 | down.lnc | up.mi | down.m |
| RP11-401P9.4 | hsa-let-7c-3p | AMOTL1 | down.lnc | up.mi | down.m |
| RP11-401P9.4 | hsa-let-7c-3p | NECAB1 | down.lnc | up.mi | down.m |
| RP11-401P9.4 | hsa-let-7c-3p | JAZF1 | down.lnc | up.mi | down.m |
| RP11-401P9.4 | hsa-let-7c-3p | SEC14L1 | down.lnc | up.mi | down.m |
| RP11-401P9.4 | hsa-let-7c-3p | ID4 | down.lnc | up.mi | down.m |
| CTA-384D8.35 | hsa-let-7e-5p | PYCR1 | up.lnc | down.mi | up.m |
| RP11-401P9.4 | hsa-let-7g-3p | STARD8 | down.lnc | up.mi | down.m |
| RP11-259K15.2 | hsa-miR-15a-5p | BTG2 | down.lnc | up.mi | down.m |
| RP11-401P9.4 | hsa-let-7g-3p | OLR1 | down.lnc | up.mi | down.m |
| RP11-401P9.4 | hsa-let-7g-3p | PIAS1 | down.lnc | up.mi | down.m |
| RP11-401P9.4 | hsa-let-7g-3p | NFIX | down.lnc | up.mi | down.m |
| RP11-401P9.4 | hsa-let-7g-3p | ZNF704 | down.lnc | up.mi | down.m |
| RP11-401P9.4 | hsa-let-7g-3p | DLC1 | down.lnc | up.mi | down.m |
| RP11-401P9.4 | hsa-let-7g-3p | ZMIZ1 | down.lnc | up.mi | down.m |
| RP11-401P9.4 | hsa-miR-130b-5p | MYOCD | down.lnc | up.mi | down.m |
| RP11-401P9.4 | hsa-miR-130b-5p | COL4A4 | down.lnc | up.mi | down.m |
| RP11-401P9.4 | hsa-miR-130b-5p | CRY2 | down.lnc | up.mi | down.m |
| RP11-259K15.2 | hsa-miR-3913-5p | CGNL1 | down.lnc | up.mi | down.m |
| RP11-401P9.4 | hsa-miR-130b-5p | WASF3 | down.lnc | up.mi | down.m |
| RP11-401P9.4 | hsa-miR-130b-5p | PLCL1 | down.lnc | up.mi | down.m |
| RP11-401P9.4 | hsa-miR-130b-5p | FOXO1 | down.lnc | up.mi | down.m |
| RP11-401P9.4 | hsa-miR-130b-5p | RCAN1 | down.lnc | up.mi | down.m |
| RP11-401P9.4 | hsa-miR-130b-5p | PTPRM | down.lnc | up.mi | down.m |
| RP11-401P9.4 | hsa-miR-130b-5p | SULT1C4 | down.lnc | up.mi | down.m |
| RP11-401P9.4 | hsa-miR-130b-5p | FAM53B | down.lnc | up.mi | down.m |
| RP11-401P9.4 | hsa-miR-130b-5p | TNS1 | down.lnc | up.mi | down.m |
| RP11-401P9.4 | hsa-miR-130b-5p | CYYR1 | down.lnc | up.mi | down.m |
| RP11-401P9.4 | hsa-miR-130b-5p | NRN1 | down.lnc | up.mi | down.m |
| RP11-401P9.4 | hsa-miR-130b-5p | EMP2 | down.lnc | up.mi | down.m |
| RP11-401P9.4 | hsa-miR-130b-5p | LRRFIP1 | down.lnc | up.mi | down.m |
| RP11-401P9.4 | hsa-miR-130b-5p | ADPRH | down.lnc | up.mi | down.m |
| RP11-401P9.4 | hsa-miR-130b-5p | SYNPO | down.lnc | up.mi | down.m |
| RP11-401P9.4 | hsa-miR-130b-5p | SLC22A3 | down.lnc | up.mi | down.m |
| RP11-401P9.4 | hsa-miR-130b-5p | ZNF704 | down.lnc | up.mi | down.m |
| RP11-401P9.4 | hsa-miR-130b-5p | SVEP1 | down.lnc | up.mi | down.m |
| RP11-401P9.4 | hsa-miR-130b-5p | TGFBR3 | down.lnc | up.mi | down.m |
| RP11-401P9.4 | hsa-miR-130b-5p | AKAP2 | down.lnc | up.mi | down.m |
| RP11-401P9.4 | hsa-miR-130b-5p | HHIP | down.lnc | up.mi | down.m |
| RP11-401P9.4 | hsa-miR-134-5p | ZNF106 | down.lnc | up.mi | down.m |
| RP11-401P9.4 | hsa-miR-134-5p | KIAA0040 | down.lnc | up.mi | down.m |
| RP11-401P9.4 | hsa-miR-134-5p | SOX17 | down.lnc | up.mi | down.m |
| RP11-401P9.4 | hsa-miR-134-5p | SMAD6 | down.lnc | up.mi | down.m |
| RP11-401P9.4 | hsa-miR-134-5p | RCAN1 | down.lnc | up.mi | down.m |
| RP11-401P9.4 | hsa-miR-135b-3p | LATS2 | down.lnc | up.mi | down.m |
| RP11-401P9.4 | hsa-miR-135b-3p | ZNF423 | down.lnc | up.mi | down.m |
| RP11-401P9.4 | hsa-miR-135b-3p | THRA | down.lnc | up.mi | down.m |
| RP11-401P9.4 | hsa-miR-142-5p | CC2D2A | down.lnc | up.mi | down.m |
| RP11-401P9.4 | hsa-miR-142-5p | CALM1 | down.lnc | up.mi | down.m |
| RP11-259K15.2 | hsa-miR-503-5p | CREBRF | down.lnc | up.mi | down.m |
| RP11-401P9.4 | hsa-miR-142-5p | TNS1 | down.lnc | up.mi | down.m |
| RP11-401P9.4 | hsa-miR-142-5p | DLC1 | down.lnc | up.mi | down.m |
| RP11-401P9.4 | hsa-miR-142-5p | SLC6A4 | down.lnc | up.mi | down.m |
| RP11-401P9.4 | hsa-miR-142-5p | FOXO3 | down.lnc | up.mi | down.m |
| RP11-401P9.4 | hsa-miR-142-5p | TGFB2 | down.lnc | up.mi | down.m |
| RP11-401P9.4 | hsa-miR-142-5p | TGFBR2 | down.lnc | up.mi | down.m |
| RP11-401P9.4 | hsa-miR-142-5p | FFAR4 | down.lnc | up.mi | down.m |
| RP11-401P9.4 | hsa-miR-142-5p | ZEB1 | down.lnc | up.mi | down.m |
| RP11-401P9.4 | hsa-miR-142-5p | RCAN2 | down.lnc | up.mi | down.m |
| RP11-401P9.4 | hsa-miR-142-5p | KLF6 | down.lnc | up.mi | down.m |
| RP11-401P9.4 | hsa-miR-148a-5p | TNS1 | down.lnc | up.mi | down.m |
| RP11-401P9.4 | hsa-miR-148a-5p | ID4 | down.lnc | up.mi | down.m |
| RP11-401P9.4 | hsa-miR-148a-5p | KLF13 | down.lnc | up.mi | down.m |
| RP11-401P9.4 | hsa-miR-148a-5p | RCAN2 | down.lnc | up.mi | down.m |
| RP11-401P9.4 | hsa-miR-148b-5p | CRIM1 | down.lnc | up.mi | down.m |
| RP11-401P9.4 | hsa-miR-148b-5p | PPP1R15A | down.lnc | up.mi | down.m |
| RP11-401P9.4 | hsa-miR-148b-5p | GPRIN2 | down.lnc | up.mi | down.m |
| RP11-259K15.2 | hsa-miR-15a-5p | HSDL2 | down.lnc | up.mi | down.m |
| RP11-259K15.2 | hsa-miR-15a-5p | C1orf21 | down.lnc | up.mi | down.m |
| RP11-401P9.4 | hsa-let-7a-2-3p | CADM1 | down.lnc | up.mi | down.m |
| RP11-259K15.2 | hsa-miR-15a-5p | ABCC6 | down.lnc | up.mi | down.m |
| RP11-401P9.4 | hsa-let-7c-3p | CREBRF | down.lnc | up.mi | down.m |
| RP11-401P9.4 | hsa-let-7g-3p | CADM1 | down.lnc | up.mi | down.m |
| RP11-259K15.2 | hsa-miR-15a-5p | TMEM100 | down.lnc | up.mi | down.m |
| RP11-259K15.2 | hsa-miR-15a-5p | RS1 | down.lnc | up.mi | down.m |
| Z83851.4 | hsa-miR-15b-5p | TBRG4 | up.lnc | down.mi | up.m |
| RP11-259K15.2 | hsa-miR-193a-3p | TAPT1 | down.lnc | up.mi | down.m |
| RP11-259K15.2 | hsa-miR-193b-3p | ATOH8 | down.lnc | up.mi | down.m |
| Z83851.4 | hsa-miR-195-5p | BIRC5 | up.lnc | down.mi | up.m |
| RP11-401P9.4 | hsa-miR-20b-3p | TNNC1 | down.lnc | up.mi | down.m |
| RP11-401P9.4 | hsa-miR-20b-3p | FZD4 | down.lnc | up.mi | down.m |
| RP11-401P9.4 | hsa-miR-130b-5p | CGNL1 | down.lnc | up.mi | down.m |
| RP11-401P9.4 | hsa-miR-20b-3p | TBX4 | down.lnc | up.mi | down.m |
| RP11-401P9.4 | hsa-miR-20b-3p | TTC28 | down.lnc | up.mi | down.m |
| RP11-401P9.4 | hsa-miR-21-5p | DDR2 | down.lnc | up.mi | down.m |
| RP11-401P9.4 | hsa-miR-21-5p | SNX30 | down.lnc | up.mi | down.m |
| RP11-401P9.4 | hsa-miR-21-5p | SESN1 | down.lnc | up.mi | down.m |
| RP11-401P9.4 | hsa-miR-21-5p | FOXO3 | down.lnc | up.mi | down.m |
| RP11-401P9.4 | hsa-miR-21-5p | RECK | down.lnc | up.mi | down.m |
| RP11-401P9.4 | hsa-miR-21-5p | FOXO1 | down.lnc | up.mi | down.m |
| RP11-401P9.4 | hsa-miR-21-5p | TIMP3 | down.lnc | up.mi | down.m |
| RP11-401P9.4 | hsa-miR-142-5p | CREBRF | down.lnc | up.mi | down.m |
| RP11-401P9.4 | hsa-miR-21-5p | MYO9A | down.lnc | up.mi | down.m |
| RP11-401P9.4 | hsa-miR-21-5p | GNAQ | down.lnc | up.mi | down.m |
| RP11-401P9.4 | hsa-miR-21-5p | TGFBR3 | down.lnc | up.mi | down.m |
| RP11-401P9.4 | hsa-miR-21-5p | C20orf194 | down.lnc | up.mi | down.m |
| RP11-401P9.4 | hsa-miR-21-5p | EPM2A | down.lnc | up.mi | down.m |
| RP11-401P9.4 | hsa-miR-21-5p | OTUD1 | down.lnc | up.mi | down.m |
| RP11-401P9.4 | hsa-miR-21-5p | DOCK4 | down.lnc | up.mi | down.m |
| RP11-401P9.4 | hsa-miR-21-5p | SECISBP2L | down.lnc | up.mi | down.m |
| RP11-401P9.4 | hsa-miR-21-5p | LRRFIP1 | down.lnc | up.mi | down.m |
| RP11-401P9.4 | hsa-miR-21-5p | SSFA2 | down.lnc | up.mi | down.m |
| RP11-401P9.4 | hsa-miR-21-5p | SMAD7 | down.lnc | up.mi | down.m |
| RP11-401P9.4 | hsa-miR-21-5p | PIK3R1 | down.lnc | up.mi | down.m |
| RP11-401P9.4 | hsa-miR-21-5p | LIFR | down.lnc | up.mi | down.m |
| RP11-401P9.4 | hsa-miR-21-5p | KLF9 | down.lnc | up.mi | down.m |
| RP11-401P9.4 | hsa-miR-21-5p | BNIP2 | down.lnc | up.mi | down.m |
| RP11-401P9.4 | hsa-miR-21-5p | WFS1 | down.lnc | up.mi | down.m |
| RP11-401P9.4 | hsa-miR-21-5p | MEF2A | down.lnc | up.mi | down.m |
| RP11-401P9.4 | hsa-miR-21-5p | WWC2 | down.lnc | up.mi | down.m |
| RP11-401P9.4 | hsa-miR-21-5p | FERMT2 | down.lnc | up.mi | down.m |
| RP11-401P9.4 | hsa-miR-21-5p | SOX5 | down.lnc | up.mi | down.m |
| RP11-401P9.4 | hsa-miR-21-5p | PRKCE | down.lnc | up.mi | down.m |
| RP11-401P9.4 | hsa-miR-21-5p | ZBTB47 | down.lnc | up.mi | down.m |
| RP11-401P9.4 | hsa-miR-21-5p | REV3L | down.lnc | up.mi | down.m |
| RP11-401P9.4 | hsa-miR-21-5p | SATB1 | down.lnc | up.mi | down.m |
| RP11-401P9.4 | hsa-miR-21-5p | SASH1 | down.lnc | up.mi | down.m |
| RP11-401P9.4 | hsa-miR-21-5p | ABCB1 | down.lnc | up.mi | down.m |
| RP11-401P9.4 | hsa-miR-21-5p | TGFBR2 | down.lnc | up.mi | down.m |
| RP11-401P9.4 | hsa-miR-21-5p | RAB11FIP2 | down.lnc | up.mi | down.m |
| RP11-401P9.4 | hsa-miR-21-5p | MEIS1 | down.lnc | up.mi | down.m |
| RP11-401P9.4 | hsa-miR-21-5p | UTRN | down.lnc | up.mi | down.m |
| RP11-401P9.4 | hsa-miR-21-5p | FOXN3 | down.lnc | up.mi | down.m |
| RP11-401P9.4 | hsa-miR-20b-3p | CREBRF | down.lnc | up.mi | down.m |
| RP11-401P9.4 | hsa-miR-21-5p | COBLL1 | down.lnc | up.mi | down.m |
| RP11-401P9.4 | hsa-miR-21-5p | TNS3 | down.lnc | up.mi | down.m |
| RP11-401P9.4 | hsa-miR-21-5p | PRICKLE2 | down.lnc | up.mi | down.m |
| RP11-401P9.4 | hsa-miR-21-5p | LIMCH1 | down.lnc | up.mi | down.m |
| RP11-401P9.4 | hsa-miR-21-5p | OLR1 | down.lnc | up.mi | down.m |
| RP11-401P9.4 | hsa-miR-21-5p | PHACTR2 | down.lnc | up.mi | down.m |
| RP11-401P9.4 | hsa-miR-21-5p | SNRK | down.lnc | up.mi | down.m |
| RP11-401P9.4 | hsa-miR-21-5p | C15orf52 | down.lnc | up.mi | down.m |
| RP11-401P9.4 | hsa-miR-21-5p | BMPR2 | down.lnc | up.mi | down.m |
| RP11-401P9.4 | hsa-miR-21-5p | SEMA5A | down.lnc | up.mi | down.m |
| RP11-401P9.4 | hsa-miR-21-5p | TCF21 | down.lnc | up.mi | down.m |
| RP11-401P9.4 | hsa-miR-21-5p | NFIA | down.lnc | up.mi | down.m |
| RP11-401P9.4 | hsa-miR-21-5p | CYBRD1 | down.lnc | up.mi | down.m |
| RP11-401P9.4 | hsa-miR-21-5p | TGFB2 | down.lnc | up.mi | down.m |
| RP11-401P9.4 | hsa-miR-224-5p | DPYSL2 | down.lnc | up.mi | down.m |
| RP11-401P9.4 | hsa-miR-224-5p | TOM1L2 | down.lnc | up.mi | down.m |
| RP11-401P9.4 | hsa-miR-224-5p | GGA2 | down.lnc | up.mi | down.m |
| RP11-401P9.4 | hsa-miR-224-5p | FOSB | down.lnc | up.mi | down.m |
| RP11-401P9.4 | hsa-miR-224-5p | VGLL3 | down.lnc | up.mi | down.m |
| RP11-401P9.4 | hsa-miR-224-5p | QKI | down.lnc | up.mi | down.m |
| RP5-1059L7.1 | hsa-miR-30b-3p | ADAM12 | up.lnc | down.mi | up.m |
| RP5-1059L7.1 | hsa-miR-30b-3p | COL5A1 | up.lnc | down.mi | up.m |
| CTA-384D8.35 | hsa-miR-30c-2-3p | PTRH2 | up.lnc | down.mi | up.m |
| CTA-384D8.35 | hsa-miR-30c-2-3p | SCNM1 | up.lnc | down.mi | up.m |
| RP11-401P9.4 | hsa-miR-3136-5p | KIAA0040 | down.lnc | up.mi | down.m |
| RP11-401P9.4 | hsa-miR-3136-5p | TRPV2 | down.lnc | up.mi | down.m |
| RP11-401P9.4 | hsa-miR-3136-5p | DDR2 | down.lnc | up.mi | down.m |
| RP11-401P9.4 | hsa-miR-3136-5p | SKI | down.lnc | up.mi | down.m |
| RP11-401P9.4 | hsa-miR-3136-5p | ZNF106 | down.lnc | up.mi | down.m |
| RP11-401P9.4 | hsa-miR-323a-3p | KCTD10 | down.lnc | up.mi | down.m |
| RP11-401P9.4 | hsa-miR-324-3p | FAM107A | down.lnc | up.mi | down.m |
| RP11-401P9.4 | hsa-miR-324-3p | ARRDC4 | down.lnc | up.mi | down.m |
| RP11-401P9.4 | hsa-miR-324-3p | MFSD2A | down.lnc | up.mi | down.m |
| RP11-401P9.4 | hsa-miR-324-3p | RAB11FIP1 | down.lnc | up.mi | down.m |
| RP11-401P9.4 | hsa-miR-324-3p | TJP1 | down.lnc | up.mi | down.m |
| RP11-401P9.4 | hsa-miR-324-3p | PLCE1 | down.lnc | up.mi | down.m |
| RP11-401P9.4 | hsa-miR-324-3p | CCND2 | down.lnc | up.mi | down.m |
| RP11-401P9.4 | hsa-miR-324-3p | ID4 | down.lnc | up.mi | down.m |
| RP11-401P9.4 | hsa-miR-324-3p | SPN | down.lnc | up.mi | down.m |
| RP11-401P9.4 | hsa-miR-324-3p | MDGA1 | down.lnc | up.mi | down.m |
| RP11-401P9.4 | hsa-miR-324-3p | NHLRC4 | down.lnc | up.mi | down.m |
| RP11-401P9.4 | hsa-miR-324-3p | MAGI1 | down.lnc | up.mi | down.m |
| RP11-401P9.4 | hsa-miR-324-3p | NFATC3 | down.lnc | up.mi | down.m |
| RP11-401P9.4 | hsa-miR-339-5p | AMOTL1 | down.lnc | up.mi | down.m |
| RP11-401P9.4 | hsa-miR-339-5p | RCAN1 | down.lnc | up.mi | down.m |
| RP11-401P9.4 | hsa-miR-339-5p | RAPGEF2 | down.lnc | up.mi | down.m |
| RP11-401P9.4 | hsa-miR-339-5p | HLA-E | down.lnc | up.mi | down.m |
| RP11-401P9.4 | hsa-miR-339-5p | LIMD1 | down.lnc | up.mi | down.m |
| RP11-401P9.4 | hsa-miR-339-5p | HHIP | down.lnc | up.mi | down.m |
| RP11-401P9.4 | hsa-miR-21-5p | CADM1 | down.lnc | up.mi | down.m |
| RP11-401P9.4 | hsa-miR-339-5p | METTL7A | down.lnc | up.mi | down.m |
| RP11-401P9.4 | hsa-miR-339-5p | INPP5A | down.lnc | up.mi | down.m |
| RP11-401P9.4 | hsa-miR-339-5p | SYNPO | down.lnc | up.mi | down.m |
| RP11-401P9.4 | hsa-miR-339-5p | FGD4 | down.lnc | up.mi | down.m |
| RP11-401P9.4 | hsa-miR-361-3p | MAOB | down.lnc | up.mi | down.m |
| RP11-401P9.4 | hsa-miR-361-3p | LHFPL3 | down.lnc | up.mi | down.m |
| RP11-401P9.4 | hsa-miR-361-3p | CBX6 | down.lnc | up.mi | down.m |
| RP11-401P9.4 | hsa-miR-361-3p | ZBTB16 | down.lnc | up.mi | down.m |
| RP11-401P9.4 | hsa-miR-361-3p | NFATC3 | down.lnc | up.mi | down.m |
| RP11-401P9.4 | hsa-miR-361-3p | PPARGC1B | down.lnc | up.mi | down.m |
| RP11-401P9.4 | hsa-miR-361-3p | GPD1 | down.lnc | up.mi | down.m |
| RP11-401P9.4 | hsa-miR-361-3p | ARHGEF17 | down.lnc | up.mi | down.m |
| RP11-401P9.4 | hsa-miR-21-5p | BTG2 | down.lnc | up.mi | down.m |
| RP11-401P9.4 | hsa-miR-361-3p | LSAMP | down.lnc | up.mi | down.m |
| RP11-401P9.4 | hsa-miR-361-3p | BCL2L2 | down.lnc | up.mi | down.m |
| RP11-401P9.4 | hsa-miR-3677-5p | KANK1 | down.lnc | up.mi | down.m |
| RP11-401P9.4 | hsa-miR-3677-5p | SUSD6 | down.lnc | up.mi | down.m |
| RP11-401P9.4 | hsa-miR-382-5p | TSPYL1 | down.lnc | up.mi | down.m |
| RP11-401P9.4 | hsa-miR-382-5p | PLSCR4 | down.lnc | up.mi | down.m |
| RP11-401P9.4 | hsa-miR-382-5p | CALM1 | down.lnc | up.mi | down.m |
| RP11-401P9.4 | hsa-miR-382-5p | TRPV2 | down.lnc | up.mi | down.m |
| RP11-401P9.4 | hsa-miR-382-5p | CCND2 | down.lnc | up.mi | down.m |
| RP11-401P9.4 | hsa-miR-382-5p | NFIA | down.lnc | up.mi | down.m |
| RP11-259K15.2 | hsa-miR-3913-5p | INMT | down.lnc | up.mi | down.m |
| RP11-259K15.2 | hsa-miR-3913-5p | ACADL | down.lnc | up.mi | down.m |
| RP11-259K15.2 | hsa-miR-3913-5p | PIGR | down.lnc | up.mi | down.m |
| RP11-259K15.2 | hsa-miR-3913-5p | PLLP | down.lnc | up.mi | down.m |
| RP11-401P9.4 | hsa-miR-339-5p | BTG2 | down.lnc | up.mi | down.m |
| RP11-259K15.2 | hsa-miR-3913-5p | NPR1 | down.lnc | up.mi | down.m |
| RP11-401P9.4 | hsa-miR-409-3p | RECK | down.lnc | up.mi | down.m |
| RP11-401P9.4 | hsa-miR-409-3p | ZEB1 | down.lnc | up.mi | down.m |
| RP11-401P9.4 | hsa-miR-409-3p | RNF38 | down.lnc | up.mi | down.m |
| RP11-401P9.4 | hsa-miR-409-3p | GAB1 | down.lnc | up.mi | down.m |
| RP11-401P9.4 | hsa-miR-409-3p | SECISBP2L | down.lnc | up.mi | down.m |
| RP11-401P9.4 | hsa-miR-409-3p | FOXN3 | down.lnc | up.mi | down.m |
| RP11-401P9.4 | hsa-miR-409-3p | TGFBR2 | down.lnc | up.mi | down.m |
| RP11-401P9.4 | hsa-miR-425-5p | PLSCR4 | down.lnc | up.mi | down.m |
| RP11-401P9.4 | hsa-miR-425-5p | FRY | down.lnc | up.mi | down.m |
| RP11-401P9.4 | hsa-miR-425-5p | ZHX3 | down.lnc | up.mi | down.m |
| RP11-401P9.4 | hsa-miR-425-5p | QKI | down.lnc | up.mi | down.m |
| RP11-401P9.4 | hsa-miR-425-5p | ACACB | down.lnc | up.mi | down.m |
| RP11-401P9.4 | hsa-miR-425-5p | EOGT | down.lnc | up.mi | down.m |
| RP11-401P9.4 | hsa-miR-425-5p | CELF2 | down.lnc | up.mi | down.m |
| RP11-401P9.4 | hsa-miR-425-5p | DPYSL2 | down.lnc | up.mi | down.m |
| RP11-401P9.4 | hsa-miR-425-5p | NPNT | down.lnc | up.mi | down.m |
| RP11-401P9.4 | hsa-miR-425-5p | FOXN3 | down.lnc | up.mi | down.m |
| RP11-259K15.2 | hsa-miR-431-5p | EMCN | down.lnc | up.mi | down.m |
| RP11-259K15.2 | hsa-miR-431-5p | ZBTB4 | down.lnc | up.mi | down.m |
| RP11-259K15.2 | hsa-miR-431-5p | ADRB1 | down.lnc | up.mi | down.m |
| RP11-259K15.2 | hsa-miR-431-5p | AK1 | down.lnc | up.mi | down.m |
| RP11-401P9.4 | hsa-miR-4668-3p | MEIS1 | down.lnc | up.mi | down.m |
| RP11-401P9.4 | hsa-miR-4668-3p | SETBP1 | down.lnc | up.mi | down.m |
| RP11-401P9.4 | hsa-miR-4668-3p | TYRP1 | down.lnc | up.mi | down.m |
| RP11-401P9.4 | hsa-miR-4668-3p | KLF2 | down.lnc | up.mi | down.m |
| RP11-401P9.4 | hsa-miR-4668-3p | PRICKLE1 | down.lnc | up.mi | down.m |
| RP11-401P9.4 | hsa-miR-4668-3p | PTGIS | down.lnc | up.mi | down.m |
| RP11-401P9.4 | hsa-miR-4668-3p | RCAN1 | down.lnc | up.mi | down.m |
| RP11-401P9.4 | hsa-miR-4668-3p | SCN1A | down.lnc | up.mi | down.m |
| RP11-401P9.4 | hsa-miR-4668-3p | ST6GALNAC3 | down.lnc | up.mi | down.m |
| RP11-401P9.4 | hsa-miR-4668-3p | CCND2 | down.lnc | up.mi | down.m |
| RP11-401P9.4 | hsa-miR-4668-3p | TACC1 | down.lnc | up.mi | down.m |
| RP11-401P9.4 | hsa-miR-4668-3p | SYNJ2BP | down.lnc | up.mi | down.m |
| RP11-401P9.4 | hsa-miR-4668-3p | GNAQ | down.lnc | up.mi | down.m |
| RP11-401P9.4 | hsa-miR-4668-3p | RNF38 | down.lnc | up.mi | down.m |
| RP11-401P9.4 | hsa-miR-4668-3p | ARC | down.lnc | up.mi | down.m |
| RP11-401P9.4 | hsa-miR-4668-3p | QKI | down.lnc | up.mi | down.m |
| CTA-384D8.35 | hsa-miR-486-3p | FAM83A | up.lnc | down.mi | up.m |
| RP11-401P9.4 | hsa-miR-493-5p | FOS | down.lnc | up.mi | down.m |
| RP11-401P9.4 | hsa-miR-493-5p | CTGF | down.lnc | up.mi | down.m |
| RP11-401P9.4 | hsa-miR-493-5p | ZMIZ1 | down.lnc | up.mi | down.m |
| RP11-401P9.4 | hsa-miR-493-5p | OLR1 | down.lnc | up.mi | down.m |
| RP11-401P9.4 | hsa-miR-493-5p | FREM2 | down.lnc | up.mi | down.m |
| RP11-259K15.2 | hsa-miR-503-5p | RS1 | down.lnc | up.mi | down.m |
| RP11-259K15.2 | hsa-miR-503-5p | C1orf21 | down.lnc | up.mi | down.m |
| RP11-259K15.2 | hsa-miR-503-5p | TMEM100 | down.lnc | up.mi | down.m |
| RP11-401P9.4 | hsa-miR-361-3p | CADM1 | down.lnc | up.mi | down.m |
| RP11-259K15.2 | hsa-miR-590-3p | PPP2R5A | down.lnc | up.mi | down.m |
| RP11-259K15.2 | hsa-miR-590-3p | CITED2 | down.lnc | up.mi | down.m |
| RP11-259K15.2 | hsa-miR-590-3p | NPR1 | down.lnc | up.mi | down.m |
| RP11-401P9.4 | hsa-miR-590-5p | TGFBR2 | down.lnc | up.mi | down.m |
| RP11-401P9.4 | hsa-miR-590-5p | OLR1 | down.lnc | up.mi | down.m |
| RP11-401P9.4 | hsa-miR-590-5p | RECK | down.lnc | up.mi | down.m |
| RP11-401P9.4 | hsa-miR-590-5p | C15orf52 | down.lnc | up.mi | down.m |
| RP11-401P9.4 | hsa-miR-590-5p | FOXO3 | down.lnc | up.mi | down.m |
| RP11-401P9.4 | hsa-miR-590-5p | BTG2 | down.lnc | up.mi | down.m |
| RP11-401P9.4 | hsa-miR-590-5p | SMAD7 | down.lnc | up.mi | down.m |
| RP11-4B16.3 | hsa-miR-767-3p | ARAP2 | down.lnc | up.mi | down.m |
| RP11-4B16.3 | hsa-miR-767-3p | RCAN1 | down.lnc | up.mi | down.m |
