# Supplementary material for: Prognostic value of lncRNAs related to fatty acid metabolism in lung adenocarcinoma and their correlation with tumor microenvironment based on bioinformatics analysis
Source: Front Oncol. 2022 Oct 10;12:1022097. doi: 10.3389/fonc.2022.1022097 (PMC9590110; doi:10.3389/fonc.2022.1022097)
Supplement: Supplementary Table 1 — All samples were divided into high and low fatty acid metabolism score groups based on the median value of this score. [file DataSheet_1.zip › raw data and R code for checking/raw data/14.docx]

| Category | ID | Term | Count | % | PValue | Genes | List Total | Pop Hits | Pop Total | Fold Enrichment | Bonferroni | Benjamini | FDR |
| --- | --- | --- | --- | --- | --- | --- | --- | --- | --- | --- | --- | --- | --- |
| GOTERM_BP_DIRECT | GO:0000122 | negative regulation of transcription from RNA polymerase II promoter | 27 | 13.43284 | 1.19E-07 | BTG2, NFIX, CITED2, SATB1, WWC2, TCF21, FOXO3, ZBTB4, FOXO1, SOX17, JAZF1, MEF2A, MYOCD, CBX6, ZHX3, WFS1, ZBTB16, FOXP1, PIAS1, SMAD7, SKI, ZEB1, NFIA, CRY2, CREBRF, ID4, FOSB | 188 | 720 | 16792 | 3.349468 | 1.68E-04 | 1.68E-04 | 1.66E-04 |
| GOTERM_BP_DIRECT | GO:0001570 | vasculogenesis | 8 | 3.9801 | 2.78E-06 | TMEM100, MYOCD, SOX17, FZD4, CITED2, ZMIZ1, QKI, TGFBR2 | 188 | 56 | 16792 | 12.75988 | 0.003902 | 0.001954 | 0.001931 |
| GOTERM_BP_DIRECT | GO:0007179 | transforming growth factor beta receptor signaling pathway | 9 | 4.477612 | 8.53E-06 | SKI, TGFBR3, TGFB2, CITED2, FOS, SMAD6, FERMT2, TGFBR2, SMAD7 | 188 | 92 | 16792 | 8.737743 | 0.011944 | 0.004002 | 0.003957 |
| GOTERM_BP_DIRECT | GO:0060412 | ventricular septum morphogenesis | 6 | 2.985075 | 1.55E-05 | TGFBR3, TGFB2, BMPR2, CITED2, TGFBR2, SMAD7 | 188 | 29 | 16792 | 18.47982 | 0.021612 | 0.005458 | 0.005396 |
| GOTERM_BP_DIRECT | GO:0045892 | negative regulation of transcription, DNA-templated | 18 | 8.955224 | 4.43E-05 | ZHX3, THRA, CITED2, ZBTB16, PRICKLE1, FOXN3, LIMD1, ZBTB4, FOXO1, FOXP1, ZEB1, CRY2, ATOH8, ID4, BIRC5, LRRFIP1, ZNF423, PPARGC1B | 188 | 499 | 16792 | 3.221933 | 0.06046 | 0.012464 | 0.012322 |
| GOTERM_BP_DIRECT | GO:0045944 | positive regulation of transcription from RNA polymerase II promoter | 25 | 12.43781 | 2.38E-04 | BMPR2, NFIX, THRA, CITED2, TCF21, PIK3R1, FOXO3, FOXO1, SOX17, ZMIZ1, PPARGC1B, MEF2A, MYOCD, KLF13, NFATC3, FOS, KLF2, FOXP1, SMAD7, SKI, MEIS1, ZEB1, NFIA, ID4, FOSB | 188 | 981 | 16792 | 2.276227 | 0.284343 | 0.055713 | 0.055079 |
| GOTERM_BP_DIRECT | GO:0060389 | pathway-restricted SMAD protein phosphorylation | 4 | 1.99005 | 3.58E-04 | TGFBR3, TGFB2, TGFBR2, SMAD7 | 188 | 13 | 16792 | 27.48282 | 0.395996 | 0.071961 | 0.071142 |
| GOTERM_BP_DIRECT | GO:0003007 | heart morphogenesis | 5 | 2.487562 | 4.20E-04 | TGFBR3, TGFB2, COL5A1, ZMIZ1, DLC1 | 188 | 32 | 16792 | 13.95612 | 0.446208 | 0.073803 | 0.072963 |
| GOTERM_BP_DIRECT | GO:0042493 | response to drug | 12 | 5.970149 | 6.18E-04 | TJP1, TGFB2, ABCB1, MAOB, DPYSL2, ABCC6, FOSB, PTPRM, FOS, ACACB, SLC6A4, TGFBR2 | 188 | 304 | 16792 | 3.525756 | 0.581013 | 0.096233 | 0.095138 |
| GOTERM_BP_DIRECT | GO:0061036 | positive regulation of cartilage development | 4 | 1.99005 | 6.84E-04 | BMPR2, ZBTB16, TAPT1, SOX5 | 188 | 16 | 16792 | 22.32979 | 0.618384 | 0.096233 | 0.095138 |
| GOTERM_BP_DIRECT | GO:0051412 | response to corticosterone | 4 | 1.99005 | 9.80E-04 | MAOB, FOSB, FOS, CALM1 | 188 | 18 | 16792 | 19.8487 | 0.7487 | 0.121822 | 0.120437 |
| GOTERM_BP_DIRECT | GO:0007507 | heart development | 9 | 4.477612 | 0.001039 | MEF2A, TGFB2, CITED2, GNAQ, GAB1, PLCE1, CC2D2A, FREM2, TGFBR2 | 188 | 183 | 16792 | 4.392745 | 0.768613 | 0.121822 | 0.120437 |
| GOTERM_BP_DIRECT | GO:0030509 | BMP signaling pathway | 6 | 2.985075 | 0.001583 | SKI, TGFBR3, TMEM100, BMPR2, SMAD6, SMAD7 | 188 | 76 | 16792 | 7.051512 | 0.892526 | 0.158229 | 0.15643 |
| GOTERM_BP_DIRECT | GO:0001843 | neural tube closure | 6 | 2.985075 | 0.001678 | SKI, TGFB2, CITED2, DLC1, PRICKLE1, CC2D2A | 188 | 77 | 16792 | 6.959934 | 0.905986 | 0.158229 | 0.15643 |
| GOTERM_BP_DIRECT | GO:0003151 | outflow tract morphogenesis | 5 | 2.487562 | 0.001687 | TGFBR3, BMPR2, SOX17, CITED2, TGFBR2 | 188 | 46 | 16792 | 9.708603 | 0.907184 | 0.158229 | 0.15643 |
| GOTERM_BP_DIRECT | GO:0006366 | transcription from RNA polymerase II promoter | 15 | 7.462687 | 0.00185 | MEF2A, MYOCD, KLF13, NFIX, BMPR2, THRA, NFATC3, TCF21, FOS, FOXO3, MEIS1, NFIA, PTTG1, FOSB, SOX5 | 188 | 513 | 16792 | 2.611671 | 0.926293 | 0.158737 | 0.156932 |
| GOTERM_BP_DIRECT | GO:0045893 | positive regulation of transcription, DNA-templated | 15 | 7.462687 | 0.001918 | MYOCD, FZD4, CITED2, ZBTB16, NFATC3, FOS, FOXN3, FOXO3, FOXO1, KLF2, PIAS1, KLF6, SOX17, ATOH8, ZNF423 | 188 | 515 | 16792 | 2.601529 | 0.932998 | 0.158737 | 0.156932 |
| GOTERM_BP_DIRECT | GO:0003148 | outflow tract septum morphogenesis | 4 | 1.99005 | 0.002043 | TGFB2, BMPR2, SMAD6, TGFBR2 | 188 | 23 | 16792 | 15.53377 | 0.943802 | 0.15966 | 0.157845 |
| GOTERM_BP_DIRECT | GO:0030511 | positive regulation of transforming growth factor beta receptor signaling pathway | 4 | 1.99005 | 0.002315 | TGFBR3, MYOCD, CITED2, NPNT | 188 | 24 | 16792 | 14.88652 | 0.961758 | 0.171459 | 0.169509 |
| GOTERM_BP_DIRECT | GO:0006351 | transcription, DNA-templated | 36 | 17.91045 | 0.003 | KANK1, BTG2, NFIX, ZBTB47, THRA, CITED2, SATB1, LIMD1, ZBTB4, FOXO1, ZMIZ1, ATOH8, LRRFIP1, ZNF423, JAZF1, MEF2A, CBX6, ZHX3, ZBTB16, FOXN3, SMAD6, TBX4, KLF2, FOXP1, PIAS1, SMAD7, SKI, KLF6, ZEB1, NFIA, CRY2, KLF9, CREBRF, ID4, BIRC5, VGLL3 | 188 | 1955 | 16792 | 1.644752 | 0.985461 | 0.211078 | 0.208678 |
| GOTERM_BP_DIRECT | GO:0016477 | cell migration | 8 | 3.9801 | 0.003173 | TGFBR3, TGFB2, ARC, COL5A1, EMP2, LIMD1, TNS3, CTGF | 188 | 172 | 16792 | 4.154379 | 0.988608 | 0.2126 | 0.210182 |
| GOTERM_BP_DIRECT | GO:0007173 | epidermal growth factor receptor signaling pathway | 5 | 2.487562 | 0.003483 | PIGR, GAB1, PLCE1, PIK3R1, FAM83A | 188 | 56 | 16792 | 7.974924 | 0.992648 | 0.222762 | 0.220229 |
| GOTERM_BP_DIRECT | GO:0009612 | response to mechanical stimulus | 5 | 2.487562 | 0.004206 | RCAN1, BTG2, CITED2, FOSB, TGFBR2 | 188 | 59 | 16792 | 7.569419 | 0.997354 | 0.257307 | 0.254381 |
| GOTERM_BP_DIRECT | GO:0045669 | positive regulation of osteoblast differentiation | 5 | 2.487562 | 0.004468 | BMPR2, ZHX3, ID4, NPNT, DDR2 | 188 | 60 | 16792 | 7.443262 | 0.998173 | 0.261938 | 0.25896 |
| GOTERM_BP_DIRECT | GO:0007155 | cell adhesion | 13 | 6.467662 | 0.005419 | MAGI1, SEMA5A, PRKCE, LSAMP, EMP2, CTGF, RS1, COL5A1, ADAM12, OLR1, SVEP1, FREM2, DDR2 | 188 | 459 | 16792 | 2.529736 | 0.999524 | 0.302255 | 0.298818 |
| GOTERM_BP_DIRECT | GO:0030512 | negative regulation of transforming growth factor beta receptor signaling pathway | 5 | 2.487562 | 0.005625 | SKI, TGFBR3, SMAD6, TGFBR2, SMAD7 | 188 | 64 | 16792 | 6.978059 | 0.999645 | 0.302255 | 0.298818 |
| GOTERM_BP_DIRECT | GO:0010634 | positive regulation of epithelial cell migration | 4 | 1.99005 | 0.0058 | TGFB2, BMPR2, PRKCE, TGFBR2 | 188 | 33 | 16792 | 10.82656 | 0.999723 | 0.302255 | 0.298818 |
| GOTERM_BP_DIRECT | GO:0048286 | lung alveolus development | 4 | 1.99005 | 0.00631 | MYOCD, BMPR2, TCF21, TNS3 | 188 | 34 | 16792 | 10.50814 | 0.999865 | 0.308119 | 0.304616 |
| GOTERM_BP_DIRECT | GO:0003181 | atrioventricular valve morphogenesis | 3 | 1.492537 | 0.006351 | TGFB2, BMPR2, TGFBR2 | 188 | 11 | 16792 | 24.35977 | 0.999873 | 0.308119 | 0.304616 |
| GOTERM_BP_DIRECT | GO:0002089 | lens morphogenesis in camera-type eye | 3 | 1.492537 | 0.007565 | SKI, MEIS1, CITED2 | 188 | 12 | 16792 | 22.32979 | 0.999977 | 0.343368 | 0.339464 |
| GOTERM_BP_DIRECT | GO:0045778 | positive regulation of ossification | 3 | 1.492537 | 0.007565 | TGFB2, BMPR2, ZBTB16 | 188 | 12 | 16792 | 22.32979 | 0.999977 | 0.343368 | 0.339464 |
| GOTERM_BP_DIRECT | GO:0001657 | ureteric bud development | 4 | 1.99005 | 0.008612 | TCF21, SMAD6, NPNT, SMAD7 | 188 | 38 | 16792 | 9.402016 | 0.999995 | 0.367167 | 0.362992 |
| GOTERM_BP_DIRECT | GO:0001568 | blood vessel development | 4 | 1.99005 | 0.008612 | COL5A1, RAPGEF2, FOXO1, TGFBR2 | 188 | 38 | 16792 | 9.402016 | 0.999995 | 0.367167 | 0.362992 |
| GOTERM_BP_DIRECT | GO:0045444 | fat cell differentiation | 5 | 2.487562 | 0.008921 | FFAR4, ID4, SMAD6, FOXO1, PIAS1 | 188 | 73 | 16792 | 6.11775 | 0.999997 | 0.36918 | 0.364982 |
| GOTERM_BP_DIRECT | GO:0060021 | palate development | 5 | 2.487562 | 0.01025 | SKI, TGFBR3, TGFB2, TCF21, TGFBR2 | 188 | 76 | 16792 | 5.87626 | 0.999999 | 0.412032 | 0.407347 |
| GOTERM_BP_DIRECT | GO:0006357 | regulation of transcription from RNA polymerase II promoter | 12 | 5.970149 | 0.010623 | ZEB1, THRA, ZNF704, KLF9, NFATC3, ID4, FOSB, FOS, LRRFIP1, FOXO3, VGLL3, FOXP1 | 188 | 441 | 16792 | 2.430453 | 1 | 0.415178 | 0.410457 |
| GOTERM_BP_DIRECT | GO:0007566 | embryo implantation | 4 | 1.99005 | 0.011346 | PTGIS, EMP2, RECK, TGFBR2 | 188 | 42 | 16792 | 8.506586 | 1 | 0.431439 | 0.426533 |
| GOTERM_BP_DIRECT | GO:0001503 | ossification | 5 | 2.487562 | 0.012211 | THRA, PPARGC1B, TAPT1, CTGF, DDR2 | 188 | 80 | 16792 | 5.582447 | 1 | 0.452129 | 0.446987 |
| GOTERM_BP_DIRECT | GO:0001666 | response to hypoxia | 7 | 3.482587 | 0.012667 | TGFBR3, MYOCD, TGFB2, CITED2, LIMD1, SLC6A4, TGFBR2 | 188 | 172 | 16792 | 3.635082 | 1 | 0.456999 | 0.451802 |
| GOTERM_BP_DIRECT | GO:0010595 | positive regulation of endothelial cell migration | 4 | 1.99005 | 0.014525 | BMPR2, ATOH8, SASH1, FOXP1 | 188 | 46 | 16792 | 7.766883 | 1 | 0.510255 | 0.504452 |
| GOTERM_BP_DIRECT | GO:0035556 | intracellular signal transduction | 11 | 5.472637 | 0.015055 | TGFBR3, SNRK, LATS2, NPR1, PLCL1, PRKCE, RAPGEF2, TNS3, MYO9A, CTGF, TNS1 | 188 | 403 | 16792 | 2.437992 | 1 | 0.510255 | 0.504452 |
| GOTERM_BP_DIRECT | GO:0007399 | nervous system development | 9 | 4.477612 | 0.015231 | EPM2A, SEMA5A, NRN1, DPYSL2, ATOH8, CRIM1, LSAMP, FOS, ZNF423 | 188 | 287 | 16792 | 2.800949 | 1 | 0.510255 | 0.504452 |
| GOTERM_BP_DIRECT | GO:0051056 | regulation of small GTPase mediated signal transduction | 6 | 2.985075 | 0.017111 | FGD4, ARHGEF17, STARD8, DLC1, ARAP2, MYO9A | 188 | 134 | 16792 | 3.999365 | 1 | 0.55989 | 0.553523 |
| GOTERM_BP_DIRECT | GO:0001659 | temperature homeostasis | 3 | 1.492537 | 0.018626 | ACADL, ADRB1, FOXO1 | 188 | 19 | 16792 | 14.10302 | 1 | 0.595619 | 0.588845 |
| GOTERM_BP_DIRECT | GO:0030336 | negative regulation of cell migration | 5 | 2.487562 | 0.021636 | KANK1, CITED2, DLC1, RECK, SMAD7 | 188 | 95 | 16792 | 4.701008 | 1 | 0.674905 | 0.66723 |
| GOTERM_BP_DIRECT | GO:1904684 | negative regulation of metalloendopeptidase activity | 2 | 0.995025 | 0.022149 | TIMP3, RECK | 188 | 2 | 16792 | 89.31915 | 1 | 0.674905 | 0.66723 |
| GOTERM_BP_DIRECT | GO:0035307 | positive regulation of protein dephosphorylation | 3 | 1.492537 | 0.022545 | DLC1, PPP2R5A, CALM1 | 188 | 21 | 16792 | 12.75988 | 1 | 0.674905 | 0.66723 |
| GOTERM_BP_DIRECT | GO:0045665 | negative regulation of neuron differentiation | 4 | 1.99005 | 0.024474 | MEIS1, ID4, FOXO3, SLC6A4 | 188 | 56 | 16792 | 6.379939 | 1 | 0.692813 | 0.684934 |
| GOTERM_BP_DIRECT | GO:0043547 | positive regulation of GTPase activity | 13 | 6.467662 | 0.024554 | DOCK4, ARHGEF17, STARD8, ARAP2, ADRB1, MYO9A, FGD4, GNAQ, DLC1, BNIP2, PLCE1, RAPGEF2, CALM1 | 188 | 565 | 16792 | 2.055131 | 1 | 0.692813 | 0.684934 |
| GOTERM_BP_DIRECT | GO:0001502 | cartilage condensation | 3 | 1.492537 | 0.02462 | TGFB2, THRA, CTGF | 188 | 22 | 16792 | 12.17988 | 1 | 0.692813 | 0.684934 |
| GOTERM_BP_DIRECT | GO:0055010 | ventricular cardiac muscle tissue morphogenesis | 3 | 1.492537 | 0.031288 | TGFBR3, TNNC1, SMAD7 | 188 | 25 | 16792 | 10.7183 | 1 | 0.833554 | 0.824075 |
| GOTERM_BP_DIRECT | GO:0060395 | SMAD protein signal transduction | 4 | 1.99005 | 0.031826 | SKI, TGFB2, ATOH8, FOS | 188 | 62 | 16792 | 5.762526 | 1 | 0.833554 | 0.824075 |
| GOTERM_BP_DIRECT | GO:0001828 | inner cell mass cellular morphogenesis | 2 | 0.995025 | 0.03304 | LATS2, SOX17 | 188 | 3 | 16792 | 59.5461 | 1 | 0.833554 | 0.824075 |
| GOTERM_BP_DIRECT | GO:2000721 | positive regulation of transcription from RNA polymerase II promoter involved in smooth muscle cell differentiation | 2 | 0.995025 | 0.03304 | MYOCD, NPNT | 188 | 3 | 16792 | 59.5461 | 1 | 0.833554 | 0.824075 |
| GOTERM_BP_DIRECT | GO:0003274 | endocardial cushion fusion | 2 | 0.995025 | 0.03304 | TGFB2, TGFBR2 | 188 | 3 | 16792 | 59.5461 | 1 | 0.833554 | 0.824075 |
| GOTERM_BP_DIRECT | GO:0008285 | negative regulation of cell proliferation | 10 | 4.975124 | 0.033532 | SKI, MYOCD, TGFB2, BTG2, KLF13, ZEB1, ZBTB16, DLC1, RAPGEF2, SMAD6 | 188 | 396 | 16792 | 2.255534 | 1 | 0.833554 | 0.824075 |
| GOTERM_BP_DIRECT | GO:0007010 | cytoskeleton organization | 6 | 2.985075 | 0.034271 | FGD4, ARC, DPYSL2, PLCE1, LIMD1, WASF3 | 188 | 161 | 16792 | 3.328664 | 1 | 0.833554 | 0.824075 |
| GOTERM_BP_DIRECT | GO:0090090 | negative regulation of canonical Wnt signaling pathway | 6 | 2.985075 | 0.035853 | LATS2, SOX17, PRICKLE1, LIMD1, FOXO3, FOXO1 | 188 | 163 | 16792 | 3.287821 | 1 | 0.833554 | 0.824075 |
| GOTERM_BP_DIRECT | GO:0043627 | response to estrogen | 4 | 1.99005 | 0.035887 | RCAN1, CITED2, SMAD6, TGFBR2 | 188 | 65 | 16792 | 5.496563 | 1 | 0.833554 | 0.824075 |
| GOTERM_BP_DIRECT | GO:0035329 | hippo signaling | 3 | 1.492537 | 0.036082 | TJP1, LATS2, AMOTL1 | 188 | 27 | 16792 | 9.92435 | 1 | 0.833554 | 0.824075 |
| GOTERM_BP_DIRECT | GO:0008284 | positive regulation of cell proliferation | 11 | 5.472637 | 0.036138 | MYOCD, TGFB2, CCND2, ID4, BIRC5, LIFR, EMP2, TNS3, CTGF, TBRG4, TGFBR2 | 188 | 466 | 16792 | 2.108392 | 1 | 0.833554 | 0.824075 |
| GOTERM_BP_DIRECT | GO:0043388 | positive regulation of DNA binding | 3 | 1.492537 | 0.038579 | SKI, MYOCD, CALM1 | 188 | 28 | 16792 | 9.569909 | 1 | 0.867016 | 0.857157 |
| GOTERM_BP_DIRECT | GO:0001525 | angiogenesis | 7 | 3.482587 | 0.038822 | TMEM100, TGFB2, MEIS1, EMCN, SOX17, TBX4, CTGF | 188 | 223 | 16792 | 2.80374 | 1 | 0.867016 | 0.857157 |
| GOTERM_BP_DIRECT | GO:0001937 | negative regulation of endothelial cell proliferation | 3 | 1.492537 | 0.041139 | ATOH8, PTPRM, SYNJ2BP | 188 | 29 | 16792 | 9.239912 | 1 | 0.904421 | 0.894136 |
| GOTERM_BP_DIRECT | GO:0051138 | positive regulation of NK T cell differentiation | 2 | 0.995025 | 0.04381 | ZBTB16, TGFBR2 | 188 | 4 | 16792 | 44.65957 | 1 | 0.920016 | 0.909554 |
| GOTERM_BP_DIRECT | GO:0070884 | regulation of calcineurin-NFAT signaling cascade | 2 | 0.995025 | 0.04381 | RCAN1, RCAN2 | 188 | 4 | 16792 | 44.65957 | 1 | 0.920016 | 0.909554 |
| GOTERM_BP_DIRECT | GO:0003186 | tricuspid valve morphogenesis | 2 | 0.995025 | 0.04381 | BMPR2, TGFBR2 | 188 | 4 | 16792 | 44.65957 | 1 | 0.920016 | 0.909554 |
| GOTERM_CC_DIRECT | GO:0005667 | transcription factor complex | 9 | 4.477612 | 9.97E-04 | SKI, MEF2A, MEIS1, ZEB1, SOX17, FOS, LIMD1, SMAD6, SMAD7 | 192 | 193 | 18224 | 4.426166 | 0.191415 | 0.115138 | 0.112976 |
| GOTERM_CC_DIRECT | GO:0005634 | nucleus | 78 | 38.80597 | 0.001081 | THRA, CITED2, CELF2, FAM107A, TCF21, LIMD1, CCND2, PTTG1, SOX17, SESN1, REV3L, TIMP3, LRRFIP1, SYNPO, SOX5, EPM2A, MEF2A, MYOCD, ZHX3, KLF13, TSPYL1, PTGIS, PUS1, PRKCE, ABCC6, PPP2R5A, EMP2, FOS, FOXP1, PIAS1, ZEB1, SCNM1, LATS2, SETBP1, CRY2, BIRC5, VGLL3, KANK1, NECAB1, NFIX, ZBTB47, SATB1, RNF38, PRICKLE1, PIK3R1, FOXO3, ACACB, ZBTB4, FOXO1, SSFA2, ZNF704, ATOH8, ZNF423, PPARGC1B, JAZF1, CBX6, ZBTB16, NFATC3, FOXN3, SMAD6, TBX4, QKI, KLF2, SMAD7, TJP1, SKI, RCAN1, KLF6, SNRK, MEIS1, NFIA, DLC1, KLF9, ID4, FOSB, TACC1, CALM1, FERMT2 | 192 | 5415 | 18224 | 1.367221 | 0.205785 | 0.115138 | 0.112976 |
| GOTERM_CC_DIRECT | GO:0005829 | cytosol | 52 | 25.87065 | 0.002218 | DOCK4, BTG2, THRA, WWC2, SLC6A4, CTGF, CCND2, PTTG1, FLAD1, DPYSL2, SESN1, PLCE1, LRRFIP1, CC2D2A, SULT1C4, EPM2A, STARD8, PRKCE, TNNC1, ARHGEF17, GAB1, ARAP2, EMP2, FOS, INMT, TGFBR2, LATS2, PTRH2, GPD1, BIRC5, RAPGEF2, PPP1R15A, AK1, PRICKLE1, PIK3R1, FOXO3, ACACB, FOXO1, FGD4, BNIP2, ZNF106, ZBTB16, NFATC3, SMAD6, MYO9A, SMAD7, TJP1, DLC1, AMOTL1, CALM1, BCL2L2, FERMT2 | 192 | 3315 | 18224 | 1.488889 | 0.376835 | 0.121115 | 0.118841 |
| GOTERM_CC_DIRECT | GO:0005911 | cell-cell junction | 8 | 3.9801 | 0.002274 | MAGI1, BMPR2, CADM1, FZD4, PTPRM, RAPGEF2, PIK3R1, LIMD1 | 192 | 172 | 18224 | 4.414729 | 0.384311 | 0.121115 | 0.118841 |
| GOTERM_CC_DIRECT | GO:0005901 | caveola | 5 | 2.487562 | 0.004811 | BMPR2, PTGIS, DLC1, EMP2, TGFBR2 | 192 | 65 | 18224 | 7.301282 | 0.641995 | 0.174149 | 0.170879 |
| GOTERM_CC_DIRECT | GO:0005886 | plasma membrane | 60 | 29.85075 | 0.004906 | SEMA5A, PIGR, DOCK4, BMPR2, SLC6A4, CTGF, SPN, TMEM100, FLAD1, FFAR4, PHACTR2, OLR1, PLCE1, LRRFIP1, MFSD2A, SCN1A, MAGI1, EPM2A, ARRDC4, PRKCE, ABCC6, LIFR, EMP2, TGFBR2, HLA-E, ARC, EMCN, PLSCR4, ADAM12, RAPGEF2, UTRN, FREM2, DDR2, KANK1, SLC22A3, SLC24A3, NRN1, ABCB1, NPR1, AK1, ADRB1, PIK3R1, SSFA2, INPP5A, RAB11FIP2, CADM1, FZD4, ZBTB16, PLCL1, CRIM1, TRPV2, LSAMP, CYBRD1, SMAD7, TJP1, GNAQ, KLF9, CALM1, MDGA1, RECK | 192 | 4121 | 18224 | 1.381946 | 0.649174 | 0.174149 | 0.170879 |
| GOTERM_CC_DIRECT | GO:0005737 | cytoplasm | 72 | 35.8209 | 0.006469 | BMPR2, CITED2, HHIP, CELF2, PTPRM, LIMD1, TTC28, PTTG1, FLAD1, ZMIZ1, DPYSL2, SESN1, PLCE1, SVEP1, LRRFIP1, TNS3, TAPT1, MFSD2A, TNS1, MAGI1, EPM2A, MEF2A, PRKCE, PPP2R5A, EMP2, TGFBR3, ARC, ZEB1, LATS2, KCTD10, CRY2, BIRC5, RAPGEF2, UTRN, PPP1R15A, KANK1, NECAB1, RNF38, AK1, PRICKLE2, PIK3R1, FOXO3, FOXO1, SSFA2, FGD4, RAB11FIP1, SEC14L1, ATOH8, BNIP2, METTL7A, WASF3, JAZF1, FAM83A, PLCL1, NFATC3, SMAD6, QKI, SMAD7, TJP1, SKI, RCAN1, SNRK, DLC1, GNAQ, RCAN2, KLF9, CREBRF, ID4, TACC1, AMOTL1, CALM1, FERMT2 | 192 | 5222 | 18224 | 1.308694 | 0.748997 | 0.180183 | 0.176799 |
| GOTERM_CC_DIRECT | GO:0005923 | bicellular tight junction | 6 | 2.985075 | 0.006767 | MAGI1, TJP1, RAPGEF2, AMOTL1, SYNPO, CGNL1 | 192 | 113 | 18224 | 5.039823 | 0.764575 | 0.180183 | 0.176799 |
| GOTERM_CC_DIRECT | GO:0005604 | basement membrane | 5 | 2.487562 | 0.009537 | SPN, COL5A1, TIMP3, NPNT, FREM2 | 192 | 79 | 18224 | 6.007384 | 0.870111 | 0.225703 | 0.221465 |
| GOTERM_CC_DIRECT | GO:0043235 | receptor complex | 6 | 2.985075 | 0.010909 | TGFBR3, PIGR, NPR1, OLR1, LIFR, TGFBR2 | 192 | 127 | 18224 | 4.484252 | 0.903333 | 0.232372 | 0.228008 |
| GOTERM_CC_DIRECT | GO:0009986 | cell surface | 12 | 5.970149 | 0.02796 | SPN, TGFBR3, BMPR2, ABCB1, FZD4, HHIP, TRPV2, EMP2, SYNJ2BP, FERMT2, TNS1, HLA-E | 192 | 542 | 18224 | 2.101476 | 0.997619 | 0.541405 | 0.531238 |
| GOTERM_CC_DIRECT | GO:0016324 | apical plasma membrane | 8 | 3.9801 | 0.033983 | TJP1, BMPR2, ABCB1, ABCC6, RAPGEF2, EMP2, AMOTL1, DDR2 | 192 | 291 | 18224 | 2.609393 | 0.999366 | 0.603193 | 0.591866 |
| GOTERM_CC_DIRECT | GO:0016604 | nuclear body | 3 | 1.492537 | 0.046658 | SKI, ZBTB16, CREBRF | 192 | 33 | 18224 | 8.628788 | 0.999962 | 0.764471 | 0.750115 |
| GOTERM_MF_DIRECT | GO:0019901 | protein kinase binding | 15 | 7.462687 | 8.43E-05 | MEF2A, PPP1R15A, NPR1, EMP2, FOXO3, ZBTB4, SKI, TOM1L2, CCND2, DPYSL2, CALM1, UTRN, RAB11FIP2, SASH1, FAM83A | 189 | 376 | 16881 | 3.563197 | 0.03242 | 0.032956 | 0.032703 |
| GOTERM_MF_DIRECT | GO:0030165 | PDZ domain binding | 7 | 3.482587 | 3.96E-04 | TGFBR3, DOCK4, CADM1, FZD4, CRIM1, RAPGEF2, ADRB1 | 189 | 86 | 16881 | 7.270026 | 0.143402 | 0.057513 | 0.057072 |
| GOTERM_MF_DIRECT | GO:0003700 | sequence-specific DNA binding | 24 | 11.9403 | 4.41E-04 | MEF2A, ZHX3, NFIX, THRA, CITED2, ZBTB16, NFATC3, FOS, FOXN3, FOXO3, SMAD6, TBX4, KLF2, FOXP1, SMAD7, RCAN1, ZEB1, NFIA, PTTG1, KLF9, ATOH8, CREBRF, FOSB, SOX5 | 189 | 961 | 16881 | 2.230613 | 0.158506 | 0.057513 | 0.057072 |
| GOTERM_MF_DIRECT | GO:0003705 | RNA polymerase II distal enhancer sequence-specific binding | 6 | 2.985075 | 8.38E-04 | MEF2A, MEIS1, NFIX, SOX17, VGLL3, FOXP1 | 189 | 66 | 16881 | 8.119769 | 0.279397 | 0.081882 | 0.081254 |
| GOTERM_MF_DIRECT | GO:0005515 | protein binding | 120 | 59.70149 | 0.001134 | HHIP, CTGF, CCND2, PTTG1, SOX17, FLAD1, DPYSL2, SESN1, PLCE1, SYNPO, TNS3, SOX5, TNS1, MAGI1, ARRDC4, MEF2A, PRKCE, LIFR, PIAS1, FOXP1, TOM1L2, GPRIN2, KCTD10, ADPRH, UTRN, SLC22A3, ABCB1, ADRB1, PIK3R1, FOXO3, ACACB, ZBTB4, FOXO1, KIAA0040, INPP5A, SEC14L1, ZNF423, RAB11FIP2, FAM83A, TGFB2, CBX6, CADM1, WFS1, FZD4, ZBTB16, NFATC3, CYBRD1, PYCR1, FOXN3, SMAD6, QKI, SMAD7, GNAQ, ID4, CREBRF, AMOTL1, CALM1, BCL2L2, FERMT2, DOCK4, BTG2, BMPR2, THRA, CITED2, PTPRM, LIMD1, SLC6A4, TMEM100, REV3L, PHACTR2, OLR1, TIMP3, LRRFIP1, MYOCD, ZHX3, PTGIS, KLF13, PLLP, SECISBP2L, TNNC1, GAB1, PPP2R5A, EMP2, FOS, INMT, TGFBR2, TGFBR3, ZEB1, SCNM1, LATS2, SETBP1, PTRH2, PLSCR4, CRY2, BIRC5, RAPGEF2, DDR2, PPP1R15A, KANK1, NFIX, SATB1, TYRP1, PRICKLE1, SNX30, TBRG4, GGA2, BNIP2, SUSD6, LSAMP, C1ORF21, SYNJ2BP, MYO9A, KLF2, SKI, TJP1, KLF6, MEIS1, DLC1, TACC1, RECK | 189 | 8785 | 16881 | 1.220045 | 0.358217 | 0.088651 | 0.087971 |
| GOTERM_MF_DIRECT | GO:0001227 | transcriptional repressor activity | 5 | 2.487562 | 0.004207 | ZEB1, SATB1, NFATC3, FOXO3, ZBTB4 | 189 | 59 | 16881 | 7.569276 | 0.807671 | 0.178336 | 0.176967 |
| GOTERM_MF_DIRECT | GO:0034713 | type I transforming growth factor beta receptor binding | 3 | 1.492537 | 0.004219 | SMAD6, TGFBR2, SMAD7 | 189 | 9 | 16881 | 29.77249 | 0.808523 | 0.178336 | 0.176967 |
| GOTERM_MF_DIRECT | GO:0008022 | protein C-terminus binding | 8 | 3.9801 | 0.004331 | MAGI1, TJP1, ZBTB16, FOXN3, SYNJ2BP, SASH1, CTGF, PIAS1 | 189 | 182 | 16881 | 3.926042 | 0.81678 | 0.178336 | 0.176967 |
| GOTERM_MF_DIRECT | GO:0003682 | chromatin binding | 12 | 5.970149 | 0.004479 | SKI, MEF2A, MEIS1, ZEB1, NFIA, SATB1, CITED2, SVEP1, FOS, SMAD6, FOXO1, FOXP1 | 189 | 391 | 16881 | 2.741201 | 0.827109 | 0.178336 | 0.176967 |
| GOTERM_MF_DIRECT | GO:0008134 | transcription factor binding | 10 | 4.975124 | 0.004629 | MYOCD, ZEB1, SOX17, NFIA, THRA, FOSB, FOS, PIK3R1, PPARGC1B, PIAS1 | 189 | 284 | 16881 | 3.144981 | 0.837033 | 0.178336 | 0.176967 |
| GOTERM_MF_DIRECT | GO:0001077 | transcriptional activator activity, RNA polymerase II core promoter proximal region sequence-specific binding | 9 | 4.477612 | 0.005017 | MEF2A, MYOCD, MEIS1, KLF13, NFIA, NFATC3, FOSB, TCF21, FOS | 189 | 236 | 16881 | 3.406174 | 0.860072 | 0.178336 | 0.176967 |
| GOTERM_MF_DIRECT | GO:0046872 | metal ion binding | 36 | 17.91045 | 0.007381 | BMPR2, ZBTB47, ACACB, ZBTB4, SLC6A4, FGD4, ZNF704, REV3L, TIMP3, PLCE1, ZNF106, ZNF423, TNS3, JAZF1, ZHX3, KLF13, PRKCE, ZBTB16, ARAP2, CYBRD1, SMAD6, MYO9A, KLF2, TGFBR2, FOXP1, SMAD7, KLF6, ZEB1, SCNM1, LATS2, COL5A1, GNAQ, ADAM12, KLF9, BIRC5, FREM2 | 189 | 2069 | 16881 | 1.554098 | 0.944799 | 0.23284 | 0.231053 |
| GOTERM_MF_DIRECT | GO:0003714 | transcription corepressor activity | 8 | 3.9801 | 0.007741 | SKI, ZEB1, ZHX3, CITED2, ID4, LIMD1, JAZF1, PIAS1 | 189 | 203 | 16881 | 3.5199 | 0.952102 | 0.23284 | 0.231053 |
| GOTERM_MF_DIRECT | GO:0017124 | SH3 domain binding | 6 | 2.985075 | 0.010686 | DOCK4, PTTG1, PLSCR4, ADAM12, ZNF106, QKI | 189 | 119 | 16881 | 4.503401 | 0.985016 | 0.298449 | 0.296159 |
| GOTERM_MF_DIRECT | GO:0046332 | SMAD binding | 4 | 1.99005 | 0.012101 | SKI, TGFBR3, MEF2A, TGFBR2 | 189 | 43 | 16881 | 8.308601 | 0.991436 | 0.305422 | 0.303079 |
| GOTERM_MF_DIRECT | GO:0005096 | GTPase activator activity | 9 | 4.477612 | 0.013077 | DOCK4, GNAQ, STARD8, DLC1, ADPRH, ARAP2, BNIP2, RAPGEF2, MYO9A | 189 | 279 | 16881 | 2.881208 | 0.994182 | 0.305422 | 0.303079 |
| GOTERM_MF_DIRECT | GO:0008013 | beta-catenin binding | 5 | 2.487562 | 0.013279 | KANK1, SOX17, FOXO3, FOXO1, SMAD7 | 189 | 82 | 16881 | 5.446187 | 0.99463 | 0.305422 | 0.303079 |
| GOTERM_MF_DIRECT | GO:0046982 | protein heterodimerization activity | 12 | 5.970149 | 0.015268 | MEF2A, TGFB2, MEIS1, ZHX3, SOX17, FZD4, TYRP1, BIRC5, ADRB1, FOS, PIK3R1, BCL2L2 | 189 | 465 | 16881 | 2.304967 | 0.99756 | 0.331661 | 0.329116 |
| GOTERM_MF_DIRECT | GO:0000978 | RNA polymerase II core promoter proximal region sequence-specific DNA binding | 10 | 4.975124 | 0.018135 | SKI, MEF2A, MEIS1, KLF13, NFIA, NFATC3, FOSB, FOS, SMAD6, FOXP1 | 189 | 355 | 16881 | 2.515985 | 0.99922 | 0.373192 | 0.370329 |
| GOTERM_MF_DIRECT | GO:0030617 | transforming growth factor beta receptor, inhibitory cytoplasmic mediator activity | 2 | 0.995025 | 0.02215 | SMAD6, SMAD7 | 189 | 2 | 16881 | 89.31746 | 0.999843 | 0.419808 | 0.416587 |
| GOTERM_MF_DIRECT | GO:0070412 | R-SMAD binding | 3 | 1.492537 | 0.022547 | MYOCD, FOS, SMAD6 | 189 | 21 | 16881 | 12.75964 | 0.999866 | 0.419808 | 0.416587 |
| GOTERM_MF_DIRECT | GO:0033613 | activating transcription factor binding | 3 | 1.492537 | 0.024623 | MEF2A, WFS1, ATOH8 | 189 | 22 | 16881 | 12.17965 | 0.999942 | 0.437616 | 0.434258 |
| GOTERM_MF_DIRECT | GO:0005178 | integrin binding | 5 | 2.487562 | 0.029859 | COL5A1, EMP2, UTRN, NPNT, CTGF | 189 | 105 | 16881 | 4.253212 | 0.999993 | 0.500316 | 0.496477 |
| GOTERM_MF_DIRECT | GO:0043565 | sequence-specific DNA binding | 12 | 5.970149 | 0.03071 | MEF2A, SOX17, THRA, CREBRF, FOSB, FOS, FOXN3, FOXO3, ZBTB4, FOXO1, KLF2, FOXP1 | 189 | 518 | 16881 | 2.06913 | 0.999995 | 0.500316 | 0.496477 |
| GOTERM_MF_DIRECT | GO:0008597 | calcium-dependent protein serine/threonine phosphatase regulator activity | 2 | 0.995025 | 0.033042 | RCAN1, RCAN2 | 189 | 3 | 16881 | 59.54497 | 0.999998 | 0.510753 | 0.506834 |
| GOTERM_MF_DIRECT | GO:0042803 | protein homodimerization activity | 15 | 7.462687 | 0.033963 | TGFB2, ZHX3, MAOB, CADM1, FZD4, ZBTB16, TNNC1, TYRP1, ZBTB4, SLC6A4, GPD1, BIRC5, LRRFIP1, RAB11FIP2, BCL2L2 | 189 | 730 | 16881 | 1.83529 | 0.999999 | 0.510753 | 0.506834 |
| GOTERM_MF_DIRECT | GO:0003779 | actin binding | 8 | 3.9801 | 0.036355 | FGD4, LIMCH1, PHACTR2, SYNPO, UTRN, MYO9A, WASF3, TNS1 | 189 | 278 | 16881 | 2.570287 | 0.999999 | 0.52647 | 0.522431 |
| GOTERM_MF_DIRECT | GO:0035035 | histone acetyltransferase binding | 3 | 1.492537 | 0.038583 | MEF2A, MYOCD, CITED2 | 189 | 28 | 16881 | 9.569728 | 1 | 0.538782 | 0.534648 |
| GOTERM_MF_DIRECT | GO:0030546 | receptor activator activity | 2 | 0.995025 | 0.043812 | PRKCE, PPARGC1B | 189 | 4 | 16881 | 44.65873 | 1 | 0.57102 | 0.566639 |
| GOTERM_MF_DIRECT | GO:0034714 | type III transforming growth factor beta receptor binding | 2 | 0.995025 | 0.043812 | TGFB2, TGFBR2 | 189 | 4 | 16881 | 44.65873 | 1 | 0.57102 | 0.566639 |
| KEGG_PATHWAY | hsa04390 | Hippo signaling pathway | 9 | 4.477612 | 9.42E-04 | TGFB2, BMPR2, LATS2, CCND2, FZD4, BIRC5, LIMD1, CTGF, TGFBR2 | 94 | 151 | 6879 | 4.361773 | 0.152795 | 0.166676 | 0.165735 |
| KEGG_PATHWAY | hsa04350 | TGF-beta signaling pathway | 6 | 2.985075 | 0.005401 | TGFB2, BMPR2, ID4, SMAD6, TGFBR2, SMAD7 | 94 | 84 | 6879 | 5.227204 | 0.6145 | 0.421904 | 0.41952 |
| KEGG_PATHWAY | hsa04068 | FoxO signaling pathway | 7 | 3.482587 | 0.009327 | TGFB2, CCND2, PIK3R1, FOXO3, FOXO1, KLF2, TGFBR2 | 94 | 134 | 6879 | 3.82288 | 0.807812 | 0.421904 | 0.41952 |
| KEGG_PATHWAY | hsa05210 | Colorectal cancer | 5 | 2.487562 | 0.009609 | TGFB2, BIRC5, FOS, PIK3R1, TGFBR2 | 94 | 62 | 6879 | 5.901682 | 0.817187 | 0.421904 | 0.41952 |
| KEGG_PATHWAY | hsa05031 | Amphetamine addiction | 5 | 2.487562 | 0.011918 | ARC, MAOB, FOSB, FOS, CALM1 | 94 | 66 | 6879 | 5.544004 | 0.878786 | 0.421904 | 0.41952 |
| KEGG_PATHWAY | hsa04919 | Thyroid hormone signaling pathway | 6 | 2.985075 | 0.019394 | RCAN1, THRA, RCAN2, PLCE1, PIK3R1, FOXO1 | 94 | 115 | 6879 | 3.818131 | 0.968153 | 0.466594 | 0.463958 |
| KEGG_PATHWAY | hsa04022 | cGMP-PKG signaling pathway | 7 | 3.482587 | 0.019737 | MEF2A, NPR1, GNAQ, PRKCE, NFATC3, ADRB1, CALM1 | 94 | 158 | 6879 | 3.24219 | 0.970057 | 0.466594 | 0.463958 |
| KEGG_PATHWAY | hsa05166 | HTLV-I infection | 9 | 4.477612 | 0.021089 | TGFB2, CCND2, PTTG1, FZD4, NFATC3, FOS, PIK3R1, TGFBR2, HLA-E | 94 | 254 | 6879 | 2.593022 | 0.976514 | 0.466594 | 0.463958 |
| KEGG_PATHWAY | hsa04728 | Dopaminergic synapse | 6 | 2.985075 | 0.029192 | MAOB, GNAQ, PPP2R5A, FOS, CALM1, SCN1A | 94 | 128 | 6879 | 3.430352 | 0.994562 | 0.513325 | 0.510425 |
| KEGG_PATHWAY | hsa04310 | Wnt signaling pathway | 6 | 2.985075 | 0.038547 | CCND2, SOX17, FZD4, PRICKLE2, NFATC3, PRICKLE1 | 94 | 138 | 6879 | 3.181776 | 0.999011 | 0.513325 | 0.510425 |
| KEGG_PATHWAY | hsa05200 | Pathways in cancer | 11 | 5.472637 | 0.038684 | TGFB2, FZD4, COL4A4, ZBTB16, GNAQ, HHIP, BIRC5, FOS, PIK3R1, FOXO1, TGFBR2 | 94 | 393 | 6879 | 2.048319 | 0.999035 | 0.513325 | 0.510425 |
| KEGG_PATHWAY | hsa05206 | MicroRNAs in cancer | 9 | 4.477612 | 0.038952 | TGFB2, BMPR2, ZEB1, ABCB1, CCND2, PRKCE, TIMP3, RECK, BCL2L2 | 94 | 286 | 6879 | 2.302894 | 0.999081 | 0.513325 | 0.510425 |
| KEGG_PATHWAY | hsa04550 | Signaling pathways regulating pluripotency of stem cells | 6 | 2.985075 | 0.040615 | MEIS1, BMPR2, FZD4, ID4, LIFR, PIK3R1 | 94 | 140 | 6879 | 3.136322 | 0.999323 | 0.513325 | 0.510425 |
| KEGG_PATHWAY | hsa04750 | Inflammatory mediator regulation of TRP channels | 5 | 2.487562 | 0.043217 | GNAQ, PRKCE, TRPV2, PIK3R1, CALM1 | 94 | 98 | 6879 | 3.733717 | 0.99958 | 0.513325 | 0.510425 |
