# Supplementary material for: Prognostic value of lncRNAs related to fatty acid metabolism in lung adenocarcinoma and their correlation with tumor microenvironment based on bioinformatics analysis
Source: Front Oncol. 2022 Oct 10;12:1022097. doi: 10.3389/fonc.2022.1022097 (PMC9590110; doi:10.3389/fonc.2022.1022097)
Supplement: Supplementary Table 1 — All samples were divided into high and low fatty acid metabolism score groups based on the median value of this score. [file DataSheet_1.zip › raw data and R code for checking/raw data/2.docx]

| Ensembl_ID | gene_name | logFC | AveExpr | t | P.Value | adj.P.Val | B |
| --- | --- | --- | --- | --- | --- | --- | --- |
| ENSG00000118785.12 | SPP1 | 4.307017 | 6.506524 | 15.75961 | 1.15E-46 | 1.07E-44 | 95.09771 |
| ENSG00000143320.7 | CRABP2 | 4.222974 | 5.695204 | 13.98494 | 2.16E-38 | 1.31E-36 | 76.13 |
| ENSG00000147689.15 | FAM83A | 3.481368 | 3.385945 | 15.74718 | 1.32E-46 | 1.22E-44 | 94.9615 |
| ENSG00000164266.9 | SPINK1 | 3.418973 | 3.942328 | 8.644436 | 5.55E-17 | 8.23E-16 | 27.21832 |
| ENSG00000183010.15 | PYCR1 | 3.305164 | 4.911186 | 26.36124 | 1.50E-100 | 1.40E-97 | 218.7835 |
| ENSG00000105388.13 | CEACAM5 | 3.302294 | 5.292138 | 8.437433 | 2.71E-16 | 3.82E-15 | 25.65169 |
| ENSG00000164932.11 | CTHRC1 | 3.235979 | 4.559747 | 17.4186 | 9.77E-55 | 1.25E-52 | 113.6073 |
| ENSG00000179913.9 | B3GNT3 | 3.100256 | 3.654414 | 17.60775 | 1.13E-55 | 1.53E-53 | 115.7556 |
| ENSG00000175063.15 | UBE2C | 3.091057 | 4.053564 | 15.37932 | 7.38E-45 | 6.25E-43 | 90.95196 |
| ENSG00000196611.4 | MMP1 | 3.056704 | 3.706129 | 10.65117 | 2.85E-24 | 7.14E-23 | 43.82895 |
| ENSG00000163993.6 | S100P | 2.982606 | 5.046873 | 7.773987 | 3.60E-14 | 4.26E-13 | 20.83248 |
| ENSG00000029993.13 | HMGB3 | 2.910365 | 5.392101 | 15.81748 | 6.07E-47 | 5.75E-45 | 95.73206 |
| ENSG00000123500.8 | COL10A1 | 2.89384 | 3.153907 | 14.46214 | 1.42E-40 | 9.46E-39 | 81.13068 |
| ENSG00000099953.8 | MMP11 | 2.856332 | 2.972588 | 12.58826 | 3.25E-32 | 1.35E-30 | 61.98845 |
| ENSG00000108821.12 | COL1A1 | 2.849658 | 7.363984 | 13.26061 | 3.80E-35 | 1.89E-33 | 68.69862 |
| ENSG00000131747.13 | TOP2A | 2.813548 | 3.649943 | 17.07416 | 4.86E-53 | 5.84E-51 | 109.7134 |
| ENSG00000101057.14 | MYBL2 | 2.810747 | 3.476365 | 14.3728 | 3.66E-40 | 2.38E-38 | 80.18853 |
| ENSG00000110492.14 | MDK | 2.772574 | 6.522625 | 16.3119 | 2.54E-49 | 2.64E-47 | 101.1874 |
| ENSG00000117399.12 | CDC20 | 2.759097 | 3.520883 | 16.58229 | 1.24E-50 | 1.36E-48 | 104.1965 |
| ENSG00000101210.9 | EEF1A2 | 2.754256 | 2.90503 | 8.422471 | 3.04E-16 | 4.26E-15 | 25.5396 |
| ENSG00000047457.12 | CP | 2.726737 | 4.591787 | 11.044 | 8.13E-26 | 2.26E-24 | 47.35754 |
| ENSG00000117394.18 | SLC2A1 | 2.711146 | 4.355461 | 14.51402 | 8.19E-41 | 5.58E-39 | 81.67904 |
| ENSG00000170373.7 | CST1 | 2.695812 | 2.583827 | 9.628873 | 2.00E-20 | 3.81E-19 | 35.05667 |
| ENSG00000175832.11 | ETV4 | 2.673671 | 3.310688 | 17.78815 | 1.44E-56 | 2.03E-54 | 117.8108 |
| ENSG00000019186.8 | CYP24A1 | 2.673583 | 2.678771 | 9.575317 | 3.13E-20 | 5.90E-19 | 34.61433 |
| ENSG00000137648.15 | TMPRSS4 | 2.67259 | 2.86507 | 13.71079 | 3.75E-37 | 2.10E-35 | 73.29388 |
| ENSG00000077152.8 | UBE2T | 2.569938 | 3.711201 | 18.43976 | 7.98E-60 | 1.30E-57 | 125.2817 |
| ENSG00000088325.14 | TPX2 | 2.557552 | 3.386155 | 15.01555 | 3.81E-43 | 2.94E-41 | 87.0254 |
| ENSG00000165474.5 | GJB2 | 2.555722 | 2.943341 | 12.25133 | 8.89E-31 | 3.35E-29 | 58.69941 |
| ENSG00000166143.8 | PPP1R14D | 2.510022 | 2.537578 | 9.434296 | 1.01E-19 | 1.84E-18 | 33.45811 |
| ENSG00000176153.11 | GPX2 | 2.506199 | 3.22086 | 6.298284 | 6.04E-10 | 4.81E-09 | 11.28868 |
| ENSG00000262406.2 | MMP12 | 2.448817 | 2.984425 | 9.842451 | 3.31E-21 | 6.73E-20 | 36.83824 |
| ENSG00000168542.11 | COL3A1 | 2.441993 | 7.511967 | 12.29805 | 5.64E-31 | 2.15E-29 | 59.15242 |
| ENSG00000135052.15 | GOLM1 | 2.441646 | 5.734007 | 20.0237 | 7.57E-68 | 1.68E-65 | 143.6965 |
| ENSG00000089685.13 | BIRC5 | 2.385993 | 2.936982 | 14.7721 | 5.22E-42 | 3.83E-40 | 84.42013 |
| ENSG00000156234.7 | CXCL13 | 2.361963 | 3.498635 | 9.709399 | 1.02E-20 | 1.98E-19 | 35.72511 |
| ENSG00000186340.13 | THBS2 | 2.357212 | 4.307667 | 12.69773 | 1.10E-32 | 4.69E-31 | 63.06798 |
| ENSG00000135069.12 | PSAT1 | 2.34453 | 3.134798 | 13.96434 | 2.68E-38 | 1.61E-36 | 75.91594 |
| ENSG00000114631.10 | PODXL2 | 2.338115 | 3.84583 | 11.18902 | 2.14E-26 | 6.18E-25 | 48.68109 |
| ENSG00000124664.9 | SPDEF | 2.305097 | 3.648276 | 11.32794 | 5.91E-27 | 1.77E-25 | 49.95928 |
| ENSG00000167900.10 | TK1 | 2.304066 | 4.551557 | 15.43414 | 4.06E-45 | 3.49E-43 | 91.547 |
| ENSG00000142273.9 | CBLC | 2.30123 | 3.26004 | 15.945 | 1.49E-47 | 1.46E-45 | 97.13304 |
| ENSG00000104783.10 | KCNN4 | 2.292711 | 3.388979 | 11.63863 | 3.20E-28 | 1.04E-26 | 52.85345 |
| ENSG00000122133.15 | PAEP | 2.288225 | 2.358548 | 6.190694 | 1.15E-09 | 8.92E-09 | 10.65992 |
| ENSG00000181019.11 | NQO1 | 2.285025 | 5.481509 | 10.18339 | 1.77E-22 | 3.94E-21 | 39.73908 |
| ENSG00000134057.13 | CCNB1 | 2.278363 | 3.586801 | 16.07198 | 3.65E-48 | 3.65E-46 | 98.53228 |
| ENSG00000166920.9 | C15orf48 | 2.272444 | 4.377319 | 9.950397 | 1.32E-21 | 2.75E-20 | 37.74919 |
| ENSG00000171004.16 | HS6ST2 | 2.235539 | 2.674818 | 12.94946 | 8.85E-34 | 4.05E-32 | 65.56974 |
| ENSG00000171848.12 | RRM2 | 2.231101 | 3.003835 | 14.67878 | 1.42E-41 | 1.01E-39 | 83.4265 |
| ENSG00000145824.11 | CXCL14 | 2.227352 | 3.964135 | 6.814236 | 2.43E-11 | 2.22E-10 | 14.43341 |
| ENSG00000166123.12 | GPT2 | 2.18568 | 2.98788 | 14.35539 | 4.40E-40 | 2.84E-38 | 80.00515 |
| ENSG00000181649.5 | PHLDA2 | 2.18552 | 4.460235 | 13.67012 | 5.70E-37 | 3.17E-35 | 72.87562 |
| ENSG00000011426.9 | ANLN | 2.151877 | 2.548402 | 13.98485 | 2.17E-38 | 1.31E-36 | 76.12908 |
| ENSG00000141934.8 | PPAP2C | 2.149831 | 3.41339 | 17.93579 | 2.65E-57 | 3.94E-55 | 119.4973 |
| ENSG00000148344.10 | PTGES | 2.139585 | 3.819314 | 12.01664 | 8.65E-30 | 3.09E-28 | 56.43914 |
| ENSG00000158373.8 | HIST1H2BD | 2.130807 | 4.116907 | 14.53411 | 6.62E-41 | 4.53E-39 | 81.89164 |
| ENSG00000103257.7 | SLC7A5 | 2.126829 | 4.192953 | 12.44153 | 1.38E-31 | 5.52E-30 | 60.54991 |
| ENSG00000011347.8 | SYT7 | 2.121553 | 2.8859 | 11.49972 | 1.19E-27 | 3.70E-26 | 51.55343 |
| ENSG00000108176.13 | DNAJC12 | 2.118099 | 2.538127 | 9.375031 | 1.64E-19 | 2.95E-18 | 32.97593 |
| ENSG00000104140.6 | RHOV | 2.090925 | 2.980882 | 9.591125 | 2.74E-20 | 5.19E-19 | 34.74471 |
| ENSG00000107159.11 | CA9 | 2.090149 | 2.095005 | 8.091238 | 3.61E-15 | 4.66E-14 | 23.09797 |
| ENSG00000108846.14 | ABCC3 | 2.06817 | 4.199523 | 12.19334 | 1.56E-30 | 5.84E-29 | 58.13856 |
| ENSG00000160182.2 | TFF1 | 2.057292 | 2.031176 | 5.48495 | 6.24E-08 | 4.00E-07 | 6.773258 |
| ENSG00000173457.9 | PPP1R14B | 2.057108 | 5.577894 | 20.92377 | 1.86E-72 | 4.74E-70 | 154.2803 |
| ENSG00000122952.15 | ZWINT | 2.04854 | 3.41779 | 15.96026 | 1.26E-47 | 1.24E-45 | 97.301 |
| ENSG00000137309.18 | HMGA1 | 2.037832 | 6.328802 | 14.49022 | 1.05E-40 | 7.14E-39 | 81.42742 |
| ENSG00000062038.12 | CDH3 | 2.033461 | 3.529208 | 9.855574 | 2.96E-21 | 6.03E-20 | 36.9486 |
| ENSG00000142945.11 | KIF2C | 2.027822 | 2.398881 | 14.25743 | 1.24E-39 | 7.84E-38 | 78.97583 |
| ENSG00000134690.9 | CDCA8 | 2.027758 | 2.726453 | 15.41938 | 4.77E-45 | 4.08E-43 | 91.3867 |
| ENSG00000060718.17 | COL11A1 | 2.019109 | 1.943641 | 8.962798 | 4.57E-18 | 7.43E-17 | 29.68418 |
| ENSG00000171564.10 | FGB | 2.014638 | 1.986608 | 5.052978 | 5.88E-07 | 3.35E-06 | 4.603007 |
| ENSG00000198183.10 | BPIFA1 | 2.007362 | 3.954802 | 4.220648 | 2.84E-05 | 0.000128391 | 0.884567 |
| ENSG00000079462.6 | PAFAH1B3 | 1.999684 | 4.41537 | 17.85053 | 7.04E-57 | 1.02E-54 | 118.5229 |
| ENSG00000145247.10 | OCIAD2 | 1.999153 | 5.177599 | 17.9525 | 2.18E-57 | 3.26E-55 | 119.6884 |
| ENSG00000205213.12 | LGR4 | 1.998825 | 3.187196 | 16.60587 | 9.49E-51 | 1.06E-48 | 104.4597 |
| ENSG00000138180.14 | CEP55 | 1.997768 | 2.543312 | 14.36675 | 3.91E-40 | 2.53E-38 | 80.12475 |
| ENSG00000111206.11 | FOXM1 | 1.996189 | 2.508177 | 13.39086 | 1.01E-35 | 5.21E-34 | 70.02004 |
| ENSG00000155066.14 | PROM2 | 1.990797 | 3.467081 | 13.24581 | 4.42E-35 | 2.18E-33 | 68.54897 |
| ENSG00000143217.8 | PVRL4 | 1.983059 | 3.946182 | 14.47618 | 1.22E-40 | 8.23E-39 | 81.27899 |
| ENSG00000178999.11 | AURKB | 1.978795 | 2.364217 | 13.74974 | 2.50E-37 | 1.43E-35 | 73.69514 |
| ENSG00000164647.7 | STEAP1 | 1.978001 | 3.243944 | 10.78428 | 8.63E-25 | 2.25E-23 | 45.01514 |
| ENSG00000176920.11 | FUT2 | 1.975543 | 2.530843 | 15.10363 | 1.47E-43 | 1.16E-41 | 87.97251 |
| ENSG00000140297.11 | GCNT3 | 1.973677 | 2.048185 | 9.459833 | 8.15E-20 | 1.50E-18 | 33.66656 |
| ENSG00000239672.6 | NME1 | 1.966056 | 3.944363 | 18.86221 | 5.97E-62 | 1.04E-59 | 130.1611 |
| ENSG00000189366.8 | ALG1L | 1.964746 | 2.547526 | 11.19775 | 1.98E-26 | 5.72E-25 | 48.76109 |
| ENSG00000100196.9 | KDELR3 | 1.956578 | 4.415535 | 15.75725 | 1.18E-46 | 1.10E-44 | 95.0718 |
| ENSG00000100985.7 | MMP9 | 1.956426 | 4.330617 | 9.351007 | 1.99E-19 | 3.56E-18 | 32.7811 |
| ENSG00000100558.7 | PLEK2 | 1.952935 | 3.187813 | 14.71029 | 1.01E-41 | 7.31E-40 | 83.76171 |
| ENSG00000002726.18 | AOC1 | 1.946696 | 2.143474 | 7.700996 | 6.06E-14 | 7.02E-13 | 20.32157 |
| ENSG00000105664.9 | COMP | 1.945811 | 2.857501 | 8.416122 | 3.19E-16 | 4.47E-15 | 25.49208 |
| ENSG00000117983.16 | MUC5B | 1.945608 | 2.952736 | 5.728748 | 1.64E-08 | 1.12E-07 | 8.068704 |
| ENSG00000146674.13 | IGFBP3 | 1.94347 | 5.53272 | 10.40446 | 2.55E-23 | 5.98E-22 | 41.65634 |
| ENSG00000137573.12 | SULF1 | 1.934415 | 3.576443 | 10.74845 | 1.19E-24 | 3.08E-23 | 44.69488 |
| ENSG00000157456.6 | CCNB2 | 1.934312 | 2.583453 | 14.63035 | 2.37E-41 | 1.67E-39 | 82.9119 |
| ENSG00000198074.8 | AKR1B10 | 1.932147 | 1.992832 | 5.398628 | 9.89E-08 | 6.20E-07 | 6.326707 |
| ENSG00000175130.6 | MARCKSL1 | 1.931445 | 6.588961 | 15.80171 | 7.22E-47 | 6.81E-45 | 95.55902 |
| ENSG00000137804.11 | NUSAP1 | 1.931262 | 3.112841 | 15.19622 | 5.40E-44 | 4.35E-42 | 88.97061 |
| ENSG00000148346.10 | LCN2 | 1.928133 | 5.474716 | 6.884015 | 1.55E-11 | 1.44E-10 | 14.8749 |
| ENSG00000167767.12 | KRT80 | 1.919307 | 3.421378 | 12.45002 | 1.27E-31 | 5.08E-30 | 60.63284 |
| ENSG00000088992.16 | TESC | 1.917814 | 3.81926 | 6.699892 | 5.04E-11 | 4.48E-10 | 13.71821 |
| ENSG00000254709.5 | IGLL5 | 1.915485 | 4.759533 | 7.981975 | 8.04E-15 | 1.01E-13 | 22.30956 |
| ENSG00000243364.6 | EFNA4 | 1.914103 | 3.801743 | 19.34878 | 2.06E-64 | 4.10E-62 | 135.8117 |
| ENSG00000061656.8 | SPAG4 | 1.910117 | 2.629712 | 15.77307 | 9.89E-47 | 9.25E-45 | 95.24517 |
| ENSG00000168528.10 | SERINC2 | 1.90286 | 5.459771 | 15.35614 | 9.49E-45 | 7.99E-43 | 90.70056 |
| ENSG00000164855.14 | TMEM184A | 1.895487 | 2.40623 | 16.13436 | 1.83E-48 | 1.85E-46 | 99.22129 |
| ENSG00000117650.11 | NEK2 | 1.890554 | 2.134406 | 13.95014 | 3.11E-38 | 1.86E-36 | 75.76843 |
| ENSG00000137745.10 | MMP13 | 1.889177 | 1.876631 | 7.672997 | 7.38E-14 | 8.49E-13 | 20.12662 |
| ENSG00000254726.2 | MEX3A | 1.874361 | 2.01832 | 12.58496 | 3.36E-32 | 1.39E-30 | 61.95598 |
| ENSG00000166851.13 | PLK1 | 1.8701 | 2.298183 | 14.88126 | 1.62E-42 | 1.23E-40 | 85.58606 |
| ENSG00000122861.14 | PLAU | 1.864892 | 5.036356 | 8.959416 | 4.70E-18 | 7.63E-17 | 29.65763 |
| ENSG00000183856.9 | IQGAP3 | 1.858694 | 2.100287 | 15.08694 | 1.76E-43 | 1.38E-41 | 87.79288 |
| ENSG00000152766.5 | ANKRD22 | 1.858186 | 2.940469 | 10.88559 | 3.45E-25 | 9.26E-24 | 45.9245 |
| ENSG00000165304.6 | MELK | 1.856416 | 2.136145 | 13.26355 | 3.69E-35 | 1.83E-33 | 68.72843 |
| ENSG00000145386.8 | CCNA2 | 1.842883 | 2.588423 | 13.41 | 8.28E-36 | 4.30E-34 | 70.21478 |
| ENSG00000076382.15 | SPAG5 | 1.842011 | 2.363292 | 14.72539 | 8.60E-42 | 6.25E-40 | 83.92246 |
| ENSG00000146072.6 | TNFRSF21 | 1.841739 | 5.481243 | 15.07023 | 2.11E-43 | 1.64E-41 | 87.61313 |
| ENSG00000137825.9 | ITPKA | 1.833709 | 1.883217 | 8.878629 | 8.90E-18 | 1.41E-16 | 29.02568 |
| ENSG00000173702.6 | MUC13 | 1.832856 | 1.946893 | 6.03745 | 2.83E-09 | 2.11E-08 | 9.780717 |
| ENSG00000186193.8 | SAPCD2 | 1.830943 | 2.021176 | 14.82041 | 3.11E-42 | 2.30E-40 | 84.93566 |
| ENSG00000148773.11 | MKI67 | 1.828803 | 2.348426 | 13.14882 | 1.18E-34 | 5.71E-33 | 67.56991 |
| ENSG00000144354.12 | CDCA7 | 1.82712 | 2.439458 | 12.5101 | 7.03E-32 | 2.85E-30 | 61.22096 |
| ENSG00000154277.11 | UCHL1 | 1.822512 | 3.222166 | 6.428566 | 2.74E-10 | 2.26E-09 | 12.06263 |
| ENSG00000096696.12 | DSP | 1.819621 | 4.578812 | 9.254481 | 4.38E-19 | 7.67E-18 | 32.00199 |
| ENSG00000090889.11 | KIF4A | 1.816909 | 1.994899 | 13.72102 | 3.37E-37 | 1.90E-35 | 73.39928 |
| ENSG00000237649.6 | KIFC1 | 1.816818 | 2.663787 | 13.19231 | 7.61E-35 | 3.70E-33 | 68.00849 |
| ENSG00000160957.11 | RECQL4 | 1.811736 | 2.376699 | 14.48642 | 1.10E-40 | 7.42E-39 | 81.38718 |
| ENSG00000067057.15 | PFKP | 1.811372 | 4.663384 | 12.15468 | 2.28E-30 | 8.38E-29 | 57.76545 |
| ENSG00000101311.14 | FERMT1 | 1.803823 | 2.276117 | 13.96564 | 2.65E-38 | 1.59E-36 | 75.92947 |
| ENSG00000126787.11 | DLGAP5 | 1.802541 | 2.026986 | 13.46568 | 4.68E-36 | 2.47E-34 | 70.78218 |
| ENSG00000204544.5 | MUC21 | 1.800061 | 2.941048 | 6.150981 | 1.46E-09 | 1.11E-08 | 10.43022 |
| ENSG00000112984.10 | KIF20A | 1.796818 | 2.20936 | 15.10633 | 1.43E-43 | 1.12E-41 | 88.00154 |
| ENSG00000106541.10 | AGR2 | 1.789191 | 6.736165 | 7.495855 | 2.55E-13 | 2.82E-12 | 18.90666 |
| ENSG00000146670.8 | CDCA5 | 1.787864 | 2.351788 | 13.259 | 3.87E-35 | 1.91E-33 | 68.68236 |
| ENSG00000164611.11 | PTTG1 | 1.784532 | 3.277004 | 13.30113 | 2.52E-35 | 1.27E-33 | 69.10903 |
| ENSG00000159166.12 | LAD1 | 1.783114 | 5.177744 | 12.97675 | 6.72E-34 | 3.10E-32 | 65.84254 |
| ENSG00000183036.9 | PCP4 | 1.779066 | 1.95059 | 6.476166 | 2.04E-10 | 1.71E-09 | 12.34882 |
| ENSG00000182481.7 | KPNA2 | 1.772623 | 4.761324 | 13.81683 | 1.25E-37 | 7.16E-36 | 74.3876 |
| ENSG00000123485.10 | HJURP | 1.769017 | 1.924516 | 13.24684 | 4.37E-35 | 2.16E-33 | 68.55934 |
| ENSG00000105011.7 | ASF1B | 1.764486 | 2.870245 | 14.60054 | 3.26E-41 | 2.27E-39 | 82.59556 |
| ENSG00000087586.16 | AURKA | 1.763433 | 2.739697 | 13.0013 | 5.25E-34 | 2.44E-32 | 66.08828 |
| ENSG00000196754.9 | S100A2 | 1.76213 | 3.227638 | 7.444086 | 3.65E-13 | 3.98E-12 | 18.55453 |
| ENSG00000085552.15 | IGSF9 | 1.759517 | 2.030583 | 13.73864 | 2.81E-37 | 1.59E-35 | 73.58079 |
| ENSG00000006016.9 | CRLF1 | 1.756258 | 3.129802 | 5.383934 | 1.07E-07 | 6.67E-07 | 6.251329 |
| ENSG00000165905.15 | GYLTL1B | 1.75321 | 3.150148 | 11.20435 | 1.86E-26 | 5.39E-25 | 48.82165 |
| ENSG00000128165.8 | ADM2 | 1.752895 | 1.989616 | 15.71768 | 1.82E-46 | 1.67E-44 | 94.63858 |
| ENSG00000104738.15 | MCM4 | 1.748551 | 3.465075 | 14.37767 | 3.48E-40 | 2.27E-38 | 80.23979 |
| ENSG00000204262.10 | COL5A2 | 1.746844 | 4.284513 | 10.45153 | 1.69E-23 | 4.02E-22 | 42.06819 |
| ENSG00000117724.11 | CENPF | 1.738632 | 2.040119 | 13.31566 | 2.17E-35 | 1.10E-33 | 69.25633 |
| ENSG00000155660.9 | PDIA4 | 1.737836 | 6.950594 | 18.02521 | 9.48E-58 | 1.42E-55 | 120.5205 |
| ENSG00000065618.15 | COL17A1 | 1.733392 | 2.023776 | 7.310124 | 9.14E-13 | 9.63E-12 | 17.65267 |
| ENSG00000104760.15 | FGL1 | 1.733147 | 1.680879 | 5.898239 | 6.32E-09 | 4.53E-08 | 8.99884 |
| ENSG00000204019.4 | CT83 | 1.718813 | 1.637951 | 6.641227 | 7.30E-11 | 6.39E-10 | 13.35528 |
| ENSG00000124602.8 | UNC5CL | 1.71813 | 2.189428 | 12.42226 | 1.67E-31 | 6.63E-30 | 60.36161 |
| ENSG00000094804.8 | CDC6 | 1.717513 | 2.003427 | 13.67209 | 5.59E-37 | 3.12E-35 | 72.89581 |
| ENSG00000173898.10 | SPTBN2 | 1.714266 | 2.522474 | 15.65711 | 3.54E-46 | 3.22E-44 | 93.97626 |
| ENSG00000204839.7 | MROH6 | 1.709498 | 2.98106 | 11.28232 | 9.03E-27 | 2.67E-25 | 49.53839 |
| ENSG00000163923.8 | RPL39L | 1.708666 | 3.352371 | 10.39048 | 2.89E-23 | 6.75E-22 | 41.53431 |
| ENSG00000049283.16 | EPN3 | 1.70711 | 2.062505 | 14.90268 | 1.28E-42 | 9.78E-41 | 85.8152 |
| ENSG00000099958.13 | DERL3 | 1.705738 | 3.057812 | 10.85703 | 4.47E-25 | 1.19E-23 | 45.66761 |
| ENSG00000130635.14 | COL5A1 | 1.705287 | 4.337269 | 10.0924 | 3.89E-22 | 8.42E-21 | 38.95814 |
| ENSG00000197930.11 | ERO1L | 1.704861 | 4.579079 | 14.14079 | 4.23E-39 | 2.63E-37 | 77.75442 |
| ENSG00000170439.6 | METTL7B | 1.700999 | 2.916056 | 9.401466 | 1.32E-19 | 2.39E-18 | 33.19073 |
| ENSG00000132329.9 | RAMP1 | 1.696147 | 4.175988 | 9.109275 | 1.42E-18 | 2.39E-17 | 30.84123 |
| ENSG00000166923.9 | GREM1 | 1.691923 | 1.688555 | 10.19737 | 1.57E-22 | 3.51E-21 | 39.85943 |
| ENSG00000109255.10 | NMU | 1.687215 | 1.777808 | 8.887267 | 8.31E-18 | 1.32E-16 | 29.09305 |
| ENSG00000198488.9 | B3GNT6 | 1.684432 | 1.650994 | 7.460729 | 3.25E-13 | 3.57E-12 | 18.66752 |
| ENSG00000170312.14 | CDK1 | 1.681378 | 2.835632 | 12.18584 | 1.68E-30 | 6.26E-29 | 58.06615 |
| ENSG00000080031.8 | PTPRH | 1.678014 | 1.632195 | 10.56525 | 6.14E-24 | 1.51E-22 | 43.06844 |
| ENSG00000129521.12 | EGLN3 | 1.676817 | 2.510027 | 9.056516 | 2.16E-18 | 3.60E-17 | 30.42287 |
| ENSG00000111640.13 | GAPDH | 1.671086 | 9.22474 | 15.44499 | 3.60E-45 | 3.11E-43 | 91.66496 |
| ENSG00000087128.8 | TMPRSS11E | 1.664679 | 1.576981 | 7.375521 | 5.85E-13 | 6.26E-12 | 18.09125 |
| ENSG00000069011.14 | PITX1 | 1.664486 | 1.711325 | 9.635649 | 1.89E-20 | 3.60E-19 | 35.11277 |
| ENSG00000115641.17 | FHL2 | 1.663748 | 2.94677 | 11.24383 | 1.29E-26 | 3.77E-25 | 49.18421 |
| ENSG00000169241.16 | SLC50A1 | 1.662683 | 5.30063 | 15.93914 | 1.59E-47 | 1.54E-45 | 97.06859 |
| ENSG00000143228.11 | NUF2 | 1.661343 | 1.843645 | 12.89144 | 1.58E-33 | 7.14E-32 | 64.99077 |
| ENSG00000128050.7 | PAICS | 1.660765 | 3.969967 | 18.76519 | 1.84E-61 | 3.16E-59 | 129.0382 |
| ENSG00000168078.8 | PBK | 1.652297 | 1.951011 | 11.6342 | 3.34E-28 | 1.08E-26 | 52.81192 |
| ENSG00000138160.5 | KIF11 | 1.651677 | 2.329147 | 13.94728 | 3.21E-38 | 1.91E-36 | 75.73876 |
| ENSG00000141696.11 | P3H4 | 1.651394 | 3.27863 | 14.85292 | 2.19E-42 | 1.64E-40 | 85.28297 |
| ENSG00000167513.7 | CDT1 | 1.65046 | 2.229406 | 13.40348 | 8.85E-36 | 4.58E-34 | 70.14848 |
| ENSG00000165215.6 | CLDN3 | 1.64801 | 5.576031 | 8.282532 | 8.73E-16 | 1.18E-14 | 24.4987 |
| ENSG00000106089.10 | STX1A | 1.645476 | 2.11741 | 13.29786 | 2.60E-35 | 1.31E-33 | 69.07589 |
| ENSG00000151651.14 | ADAM8 | 1.63948 | 3.468505 | 11.328 | 5.91E-27 | 1.77E-25 | 49.95978 |
| ENSG00000166803.9 | KIAA0101 | 1.638908 | 2.150083 | 14.01698 | 1.55E-38 | 9.43E-37 | 76.46323 |
| ENSG00000170476.14 | MZB1 | 1.63793 | 3.537009 | 7.766551 | 3.80E-14 | 4.48E-13 | 20.78025 |
| ENSG00000102265.10 | TIMP1 | 1.637127 | 7.967693 | 13.56884 | 1.62E-36 | 8.83E-35 | 71.8365 |
| ENSG00000198901.12 | PRC1 | 1.633136 | 2.453041 | 13.69901 | 4.23E-37 | 2.37E-35 | 73.17268 |
| ENSG00000135451.11 | TROAP | 1.628544 | 1.767576 | 12.32454 | 4.35E-31 | 1.67E-29 | 59.40979 |
| ENSG00000124766.5 | SOX4 | 1.627667 | 5.115521 | 14.59794 | 3.35E-41 | 2.33E-39 | 82.56796 |
| ENSG00000100526.18 | CDKN3 | 1.626249 | 2.139164 | 11.94897 | 1.66E-29 | 5.83E-28 | 55.79212 |
| ENSG00000171124.11 | FUT3 | 1.612854 | 2.940691 | 10.86503 | 4.16E-25 | 1.11E-23 | 45.73955 |
| ENSG00000121152.8 | NCAPH | 1.609912 | 2.021191 | 12.849 | 2.42E-33 | 1.09E-31 | 64.56808 |
| ENSG00000158089.13 | GALNT14 | 1.607533 | 1.866237 | 9.788621 | 5.22E-21 | 1.05E-19 | 36.38659 |
| ENSG00000071539.12 | TRIP13 | 1.601605 | 2.38407 | 11.25671 | 1.15E-26 | 3.36E-25 | 49.30265 |
| ENSG00000267795.4 | SMIM22 | 1.59956 | 4.244736 | 10.03052 | 6.63E-22 | 1.41E-20 | 38.42989 |
| ENSG00000134827.6 | TCN1 | 1.599123 | 1.643445 | 5.523209 | 5.08E-08 | 3.29E-07 | 6.973211 |
| ENSG00000205420.9 | KRT6A | 1.591631 | 1.870611 | 5.044758 | 6.12E-07 | 3.48E-06 | 4.563282 |
| ENSG00000171631.13 | P2RY6 | 1.589847 | 2.051697 | 11.29596 | 7.96E-27 | 2.37E-25 | 49.66415 |
| ENSG00000107833.9 | NPM3 | 1.588716 | 4.425555 | 16.43847 | 6.18E-50 | 6.54E-48 | 102.5938 |
| ENSG00000183048.10 | SLC25A10 | 1.588598 | 2.708227 | 15.63414 | 4.55E-46 | 4.13E-44 | 93.72542 |
| ENSG00000110660.13 | SLC35F2 | 1.58734 | 3.382281 | 15.27545 | 2.28E-44 | 1.88E-42 | 89.82673 |
| ENSG00000119888.9 | EPCAM | 1.585454 | 7.301894 | 15.24634 | 3.13E-44 | 2.56E-42 | 89.51201 |
| ENSG00000163362.9 | C1orf106 | 1.585434 | 2.828819 | 12.84267 | 2.58E-33 | 1.16E-31 | 64.5051 |
| ENSG00000169750.7 | RAC3 | 1.584902 | 2.923092 | 11.00568 | 1.15E-25 | 3.19E-24 | 47.00961 |
| ENSG00000114346.12 | ECT2 | 1.584517 | 3.027972 | 13.23266 | 5.05E-35 | 2.48E-33 | 68.41597 |
| ENSG00000175643.8 | RMI2 | 1.583893 | 2.46756 | 15.4883 | 2.25E-45 | 1.96E-43 | 92.13584 |
| ENSG00000130768.13 | SMPDL3B | 1.583178 | 4.235206 | 10.84647 | 4.92E-25 | 1.30E-23 | 45.57274 |
| ENSG00000105369.8 | CD79A | 1.58238 | 3.623712 | 7.392116 | 5.22E-13 | 5.61E-12 | 18.20305 |
| ENSG00000180198.14 | RCC1 | 1.580479 | 3.960744 | 18.87542 | 5.12E-62 | 8.97E-60 | 130.3141 |
| ENSG00000189159.14 | HN1 | 1.575766 | 5.268729 | 14.86383 | 1.95E-42 | 1.46E-40 | 85.39963 |
| ENSG00000188322.4 | SBK1 | 1.574468 | 2.434316 | 10.34725 | 4.22E-23 | 9.76E-22 | 41.15751 |
| ENSG00000059573.7 | ALDH18A1 | 1.573242 | 4.755746 | 21.49328 | 2.18E-75 | 6.59E-73 | 161.0083 |
| ENSG00000156970.11 | BUB1B | 1.566602 | 1.786557 | 13.43829 | 6.20E-36 | 3.24E-34 | 70.50292 |
| ENSG00000185686.16 | PRAME | 1.565306 | 1.449702 | 6.49064 | 1.87E-10 | 1.57E-09 | 12.4362 |
| ENSG00000093009.8 | CDC45 | 1.563609 | 1.875829 | 12.7384 | 7.31E-33 | 3.16E-31 | 63.47033 |
| ENSG00000113368.10 | LMNB1 | 1.563169 | 3.457192 | 13.23986 | 4.70E-35 | 2.31E-33 | 68.48874 |
| ENSG00000198535.5 | C2CD4A | 1.562998 | 1.717875 | 9.017788 | 2.95E-18 | 4.86E-17 | 30.11692 |
| ENSG00000006625.16 | GGCT | 1.562621 | 4.843999 | 16.15593 | 1.44E-48 | 1.46E-46 | 99.45969 |
| ENSG00000101003.9 | GINS1 | 1.562262 | 1.932197 | 13.54763 | 2.02E-36 | 1.09E-34 | 71.61937 |
| ENSG00000073111.12 | MCM2 | 1.561279 | 3.259254 | 12.69431 | 1.13E-32 | 4.84E-31 | 63.03409 |
| ENSG00000106462.9 | EZH2 | 1.555433 | 2.205527 | 14.7097 | 1.02E-41 | 7.35E-40 | 83.75542 |
| ENSG00000108773.9 | KAT2A | 1.553312 | 4.038477 | 15.9448 | 1.49E-47 | 1.46E-45 | 97.13082 |
| ENSG00000072571.18 | HMMR | 1.548242 | 1.908583 | 12.94866 | 8.92E-34 | 4.08E-32 | 65.56177 |
| ENSG00000172927.6 | MYEOV | 1.546307 | 1.485796 | 7.398563 | 4.99E-13 | 5.37E-12 | 18.24654 |
| ENSG00000133110.13 | POSTN | 1.545287 | 5.004586 | 8.459909 | 2.29E-16 | 3.24E-15 | 25.82037 |
| ENSG00000105825.10 | TFPI2 | 1.544252 | 3.475517 | 4.693307 | 3.37E-06 | 1.74E-05 | 2.92033 |
| ENSG00000138271.5 | GPR87 | 1.54393 | 1.735292 | 6.813017 | 2.45E-11 | 2.24E-10 | 14.42573 |
| ENSG00000102359.5 | SRPX2 | 1.543855 | 3.105793 | 9.098891 | 1.54E-18 | 2.59E-17 | 30.75875 |
| ENSG00000186891.12 | TNFRSF18 | 1.538673 | 2.303608 | 10.15538 | 2.25E-22 | 4.98E-21 | 39.49817 |
| ENSG00000131668.12 | BARX1 | 1.535364 | 1.441078 | 5.776794 | 1.26E-08 | 8.70E-08 | 8.329923 |
| ENSG00000143324.12 | XPR1 | 1.535304 | 3.930376 | 12.76982 | 5.35E-33 | 2.35E-31 | 63.78165 |
| ENSG00000198125.11 | MB | 1.532556 | 1.849044 | 9.372829 | 1.67E-19 | 3.00E-18 | 32.95806 |
| ENSG00000144485.9 | HES6 | 1.532372 | 2.71818 | 8.415266 | 3.21E-16 | 4.50E-15 | 25.48568 |
| ENSG00000115163.13 | CENPA | 1.528322 | 1.687629 | 11.98558 | 1.17E-29 | 4.13E-28 | 56.14185 |
| ENSG00000160606.9 | TLCD1 | 1.520891 | 3.852692 | 13.87528 | 6.79E-38 | 3.97E-36 | 74.99222 |
| ENSG00000176945.15 | MUC20 | 1.516386 | 3.075448 | 8.423424 | 3.02E-16 | 4.23E-15 | 25.54673 |
| ENSG00000186907.6 | RTN4RL2 | 1.516058 | 2.187575 | 9.02679 | 2.75E-18 | 4.53E-17 | 30.18795 |
| ENSG00000101447.12 | FAM83D | 1.515539 | 2.409028 | 11.28004 | 9.22E-27 | 2.72E-25 | 49.51743 |
| ENSG00000151725.10 | CENPU | 1.514481 | 2.347396 | 12.96779 | 7.36E-34 | 3.38E-32 | 65.75295 |
| ENSG00000276043.3 | UHRF1 | 1.511982 | 1.712523 | 13.81643 | 1.25E-37 | 7.18E-36 | 74.38348 |
| ENSG00000125571.8 | IL37 | 1.510167 | 1.531295 | 5.701756 | 1.91E-08 | 1.30E-07 | 7.922802 |
| ENSG00000162849.14 | KIF26B | 1.509147 | 1.786226 | 13.66532 | 5.99E-37 | 3.33E-35 | 72.82622 |
| ENSG00000124116.17 | WFDC3 | 1.507896 | 1.715089 | 8.716757 | 3.17E-17 | 4.80E-16 | 27.77252 |
| ENSG00000079385.20 | CEACAM1 | 1.494695 | 3.093305 | 10.74361 | 1.24E-24 | 3.21E-23 | 44.65174 |
| ENSG00000124466.8 | LYPD3 | 1.492177 | 2.064528 | 7.680372 | 7.01E-14 | 8.08E-13 | 20.17791 |
| ENSG00000162482.4 | AKR7A3 | 1.491305 | 1.583445 | 7.040064 | 5.58E-12 | 5.43E-11 | 15.87596 |
| ENSG00000146166.15 | LGSN | 1.489404 | 1.530403 | 8.560612 | 1.06E-16 | 1.54E-15 | 26.5804 |
| ENSG00000003249.12 | DBNDD1 | 1.488145 | 2.733224 | 14.57488 | 4.29E-41 | 2.96E-39 | 82.32346 |
| ENSG00000105131.6 | EPHX3 | 1.484826 | 2.829651 | 9.329583 | 2.38E-19 | 4.23E-18 | 32.60767 |
| ENSG00000115828.14 | QPCT | 1.483636 | 3.158197 | 6.603211 | 9.27E-11 | 8.02E-10 | 13.12155 |
| ENSG00000131153.7 | GINS2 | 1.479525 | 1.888112 | 12.87766 | 1.82E-33 | 8.17E-32 | 64.85346 |
| ENSG00000186185.12 | KIF18B | 1.479322 | 1.580398 | 12.08262 | 4.58E-30 | 1.66E-28 | 57.07199 |
| ENSG00000103460.15 | TOX3 | 1.476826 | 2.13164 | 8.667528 | 4.64E-17 | 6.92E-16 | 27.39489 |
| ENSG00000101104.11 | PABPC1L | 1.476215 | 2.598767 | 10.10759 | 3.41E-22 | 7.41E-21 | 39.08815 |
| ENSG00000134285.9 | FKBP11 | 1.474862 | 3.244536 | 13.93976 | 3.47E-38 | 2.06E-36 | 75.66074 |
| ENSG00000169894.16 | MUC3A | 1.470622 | 1.613637 | 7.23804 | 1.49E-12 | 1.53E-11 | 17.17299 |
| ENSG00000006118.13 | TMEM132A | 1.47021 | 2.809042 | 11.30768 | 7.14E-27 | 2.13E-25 | 49.77218 |
| ENSG00000124143.9 | ARHGAP40 | 1.469059 | 1.628147 | 9.675723 | 1.35E-20 | 2.60E-19 | 35.44509 |
| ENSG00000261236.4 | BOP1 | 1.465496 | 3.872174 | 13.49634 | 3.42E-36 | 1.83E-34 | 71.09504 |
| ENSG00000176890.14 | TYMS | 1.46397 | 3.075729 | 12.51909 | 6.43E-32 | 2.62E-30 | 61.30908 |
| ENSG00000185033.13 | SEMA4B | 1.463279 | 4.582615 | 12.41986 | 1.71E-31 | 6.78E-30 | 60.33826 |
| ENSG00000126778.8 | SIX1 | 1.46243 | 2.127955 | 9.258234 | 4.25E-19 | 7.46E-18 | 32.03218 |
| ENSG00000105048.15 | TNNT1 | 1.462383 | 2.259903 | 5.957682 | 4.50E-09 | 3.27E-08 | 9.330733 |
| ENSG00000080986.11 | NDC80 | 1.461669 | 1.755379 | 12.26974 | 7.43E-31 | 2.82E-29 | 58.87781 |
| ENSG00000113924.10 | HGD | 1.457079 | 1.945025 | 6.466714 | 2.17E-10 | 1.81E-09 | 12.29185 |
| ENSG00000158125.8 | XDH | 1.455828 | 1.602501 | 9.840612 | 3.36E-21 | 6.83E-20 | 36.82278 |
| ENSG00000088882.7 | CPXM1 | 1.454965 | 2.37294 | 10.00304 | 8.39E-22 | 1.77E-20 | 38.19596 |
| ENSG00000166922.7 | SCG5 | 1.454661 | 1.964086 | 8.259395 | 1.04E-15 | 1.40E-14 | 24.32791 |
| ENSG00000164638.9 | SLC29A4 | 1.451145 | 1.921842 | 8.8514 | 1.10E-17 | 1.73E-16 | 28.81366 |
| ENSG00000169679.13 | BUB1 | 1.448837 | 1.981656 | 12.03194 | 7.47E-30 | 2.68E-28 | 56.58569 |
| ENSG00000158315.9 | RHBDL2 | 1.44667 | 1.948127 | 13.17546 | 9.03E-35 | 4.38E-33 | 67.83846 |
| ENSG00000149418.9 | ST14 | 1.445224 | 5.940692 | 13.36225 | 1.35E-35 | 6.94E-34 | 69.72923 |
| ENSG00000188761.10 | BCL2L15 | 1.444303 | 1.652589 | 10.69962 | 1.85E-24 | 4.69E-23 | 44.25957 |
| ENSG00000105173.12 | CCNE1 | 1.443883 | 1.730514 | 11.34487 | 5.05E-27 | 1.52E-25 | 50.11569 |
| ENSG00000129235.9 | TXNDC17 | 1.440518 | 4.110195 | 12.9905 | 5.85E-34 | 2.71E-32 | 65.98021 |
| ENSG00000151715.6 | TMEM45B | 1.437819 | 3.914819 | 9.56715 | 3.35E-20 | 6.30E-19 | 34.54703 |
| ENSG00000158106.11 | RHPN1 | 1.437368 | 2.926393 | 10.58755 | 5.04E-24 | 1.25E-22 | 43.26543 |
| ENSG00000143590.12 | EFNA3 | 1.437299 | 1.733391 | 13.91676 | 4.41E-38 | 2.60E-36 | 75.42208 |
| ENSG00000103089.7 | FA2H | 1.43496 | 2.54139 | 8.862593 | 1.01E-17 | 1.59E-16 | 28.90076 |
| ENSG00000105696.7 | TMEM59L | 1.433897 | 1.778563 | 5.861432 | 7.79E-09 | 5.53E-08 | 8.794808 |
| ENSG00000184363.8 | PKP3 | 1.433704 | 4.282566 | 13.95298 | 3.02E-38 | 1.81E-36 | 75.79799 |
| ENSG00000157778.7 | PSMG3 | 1.428972 | 4.248105 | 15.17424 | 6.85E-44 | 5.48E-42 | 88.73348 |
| ENSG00000109805.8 | NCAPG | 1.428223 | 1.671372 | 11.93061 | 1.98E-29 | 6.91E-28 | 55.61702 |
| ENSG00000141756.17 | FKBP10 | 1.426647 | 4.495254 | 8.469253 | 2.13E-16 | 3.02E-15 | 25.89061 |
| ENSG00000132821.10 | VSTM2L | 1.424556 | 4.316725 | 5.866718 | 7.56E-09 | 5.37E-08 | 8.824036 |
| ENSG00000070526.13 | ST6GALNAC1 | 1.424539 | 3.6556 | 6.766393 | 3.30E-11 | 2.99E-10 | 14.13291 |
| ENSG00000134873.8 | CLDN10 | 1.424337 | 1.677408 | 6.370594 | 3.90E-10 | 3.17E-09 | 11.71655 |
| ENSG00000131746.11 | TNS4 | 1.423179 | 1.818711 | 6.39485 | 3.36E-10 | 2.75E-09 | 11.86102 |
| ENSG00000156804.6 | FBXO32 | 1.412474 | 2.840525 | 10.99165 | 1.31E-25 | 3.61E-24 | 46.88246 |
| ENSG00000096063.13 | SRPK1 | 1.408987 | 3.675969 | 18.91399 | 3.27E-62 | 5.80E-60 | 130.7609 |
| ENSG00000101443.16 | WFDC2 | 1.408955 | 7.372328 | 6.572077 | 1.13E-10 | 9.66E-10 | 12.93099 |
| ENSG00000154096.12 | THY1 | 1.407081 | 3.632112 | 10.04458 | 5.87E-22 | 1.26E-20 | 38.54969 |
| ENSG00000147889.15 | CDKN2A | 1.403945 | 1.819797 | 7.062909 | 4.80E-12 | 4.70E-11 | 16.02409 |
| ENSG00000115255.9 | REEP6 | 1.401447 | 2.695863 | 8.320174 | 6.58E-16 | 8.99E-15 | 24.77735 |
| ENSG00000187583.9 | PLEKHN1 | 1.401022 | 1.627955 | 13.89484 | 5.54E-38 | 3.25E-36 | 75.19483 |
| ENSG00000182272.10 | B4GALNT4 | 1.400827 | 1.408545 | 8.312561 | 6.97E-16 | 9.50E-15 | 24.72091 |
| ENSG00000198753.10 | PLXNB3 | 1.396469 | 1.626102 | 9.813472 | 4.23E-21 | 8.53E-20 | 36.59487 |
| ENSG00000173207.11 | CKS1B | 1.393255 | 3.433374 | 12.30747 | 5.14E-31 | 1.97E-29 | 59.24391 |
| ENSG00000137809.15 | ITGA11 | 1.39316 | 1.819913 | 9.426162 | 1.08E-19 | 1.96E-18 | 33.3918 |
| ENSG00000133466.12 | C1QTNF6 | 1.392656 | 2.143265 | 12.70911 | 9.79E-33 | 4.19E-31 | 63.18041 |
| ENSG00000152669.8 | CCNO | 1.388302 | 2.185306 | 9.010495 | 3.13E-18 | 5.14E-17 | 30.05941 |
| ENSG00000109586.10 | GALNT7 | 1.385481 | 3.395311 | 14.24004 | 1.49E-39 | 9.40E-38 | 78.79339 |
| ENSG00000123131.11 | PRDX4 | 1.385006 | 5.79351 | 13.5442 | 2.09E-36 | 1.13E-34 | 71.58429 |
| ENSG00000105427.8 | CNFN | 1.382535 | 1.920638 | 10.11481 | 3.20E-22 | 6.99E-21 | 39.15001 |
| ENSG00000154040.19 | CABYR | 1.380194 | 1.435368 | 8.071465 | 4.18E-15 | 5.35E-14 | 22.95467 |
| ENSG00000168496.3 | FEN1 | 1.375352 | 3.622113 | 13.58929 | 1.31E-36 | 7.19E-35 | 72.04598 |
| ENSG00000185803.7 | SLC52A2 | 1.374353 | 4.683314 | 14.90853 | 1.21E-42 | 9.21E-41 | 85.87783 |
| ENSG00000198937.8 | CCDC167 | 1.374287 | 4.566055 | 14.06955 | 8.93E-39 | 5.49E-37 | 77.01077 |
| ENSG00000109814.10 | UGDH | 1.371208 | 4.766244 | 8.717865 | 3.14E-17 | 4.76E-16 | 27.78104 |
| ENSG00000145050.14 | MANF | 1.370603 | 5.219556 | 17.16238 | 1.79E-53 | 2.20E-51 | 110.7084 |
| ENSG00000073150.12 | PANX2 | 1.369333 | 2.171268 | 9.02342 | 2.82E-18 | 4.65E-17 | 30.16134 |
| ENSG00000162062.13 | C16orf59 | 1.366601 | 1.469288 | 15.19365 | 5.55E-44 | 4.47E-42 | 88.94284 |
| ENSG00000160161.8 | CILP2 | 1.365265 | 1.490425 | 9.898197 | 2.06E-21 | 4.24E-20 | 37.3078 |
| ENSG00000130762.13 | ARHGEF16 | 1.364506 | 2.813959 | 14.88152 | 1.61E-42 | 1.22E-40 | 85.58881 |
| ENSG00000182704.7 | TSKU | 1.364055 | 3.800029 | 9.665305 | 1.47E-20 | 2.83E-19 | 35.35859 |
| ENSG00000116711.9 | PLA2G4A | 1.359578 | 3.412085 | 6.466729 | 2.16E-10 | 1.81E-09 | 12.29194 |
| ENSG00000174939.9 | ASPHD1 | 1.359301 | 1.933322 | 8.280906 | 8.84E-16 | 1.20E-14 | 24.48668 |
| ENSG00000101194.16 | SLC17A9 | 1.359019 | 2.064068 | 9.426656 | 1.07E-19 | 1.95E-18 | 33.39583 |
| ENSG00000182199.9 | SHMT2 | 1.358982 | 4.35021 | 14.36464 | 3.99E-40 | 2.58E-38 | 80.10257 |
| ENSG00000100290.2 | BIK | 1.357605 | 2.72557 | 9.7591 | 6.69E-21 | 1.33E-19 | 36.13965 |
| ENSG00000163468.13 | CCT3 | 1.355918 | 6.212366 | 17.14435 | 2.20E-53 | 2.68E-51 | 110.5049 |
| ENSG00000142552.6 | RCN3 | 1.353391 | 3.91512 | 8.589132 | 8.50E-17 | 1.24E-15 | 26.79691 |
| ENSG00000075218.17 | GTSE1 | 1.35202 | 1.566891 | 12.72223 | 8.59E-33 | 3.70E-31 | 63.31025 |
| ENSG00000183248.10 | PRR36 | 1.347446 | 2.255291 | 10.09325 | 3.86E-22 | 8.37E-21 | 38.96539 |
| ENSG00000136295.13 | TTYH3 | 1.346024 | 4.480643 | 12.19236 | 1.58E-30 | 5.89E-29 | 58.12913 |
| ENSG00000189057.9 | FAM111B | 1.343648 | 1.726967 | 11.98892 | 1.13E-29 | 4.00E-28 | 56.17388 |
| ENSG00000161888.10 | SPC24 | 1.343426 | 1.736319 | 12.71894 | 8.88E-33 | 3.82E-31 | 63.27771 |
| ENSG00000152952.10 | PLOD2 | 1.339316 | 3.459361 | 8.039481 | 5.29E-15 | 6.71E-14 | 22.72345 |
| ENSG00000065911.10 | MTHFD2 | 1.339206 | 3.892857 | 11.28914 | 8.48E-27 | 2.51E-25 | 49.60127 |
| ENSG00000180879.12 | SSR4 | 1.337628 | 5.891015 | 13.08656 | 2.22E-34 | 1.06E-32 | 66.94356 |
| ENSG00000111602.10 | TIMELESS | 1.335957 | 2.940402 | 13.33377 | 1.80E-35 | 9.20E-34 | 69.44003 |
| ENSG00000104951.14 | IL4I1 | 1.333591 | 2.551291 | 8.93078 | 5.89E-18 | 9.51E-17 | 29.43314 |
| ENSG00000155265.9 | GOLGA7B | 1.333215 | 1.875571 | 9.226902 | 5.48E-19 | 9.52E-18 | 31.78049 |
| ENSG00000103269.12 | RHBDL1 | 1.325705 | 1.57355 | 10.6628 | 2.57E-24 | 6.46E-23 | 43.93219 |
| ENSG00000134333.12 | LDHA | 1.324826 | 6.85449 | 13.80713 | 1.38E-37 | 7.90E-36 | 74.28738 |
| ENSG00000103202.11 | NME4 | 1.324818 | 4.466938 | 13.78802 | 1.68E-37 | 9.62E-36 | 74.09002 |
| ENSG00000173599.12 | PC | 1.320275 | 2.696184 | 13.10446 | 1.85E-34 | 8.86E-33 | 67.12342 |
| ENSG00000142102.14 | ATHL1 | 1.319451 | 3.9809 | 6.057934 | 2.51E-09 | 1.88E-08 | 9.89712 |
| ENSG00000164109.12 | MAD2L1 | 1.318767 | 1.826989 | 11.62269 | 3.72E-28 | 1.20E-26 | 52.70379 |
| ENSG00000043039.6 | BARX2 | 1.315654 | 1.440271 | 8.644239 | 5.56E-17 | 8.24E-16 | 27.21681 |
| ENSG00000157992.11 | KRTCAP3 | 1.314249 | 4.829816 | 9.489618 | 6.37E-20 | 1.18E-18 | 33.91021 |
| ENSG00000170421.10 | KRT8 | 1.314034 | 7.515621 | 10.86489 | 4.16E-25 | 1.11E-23 | 45.73828 |
| ENSG00000171346.12 | KRT15 | 1.311798 | 2.20812 | 6.808116 | 2.53E-11 | 2.31E-10 | 14.39487 |
| ENSG00000076604.13 | TRAF4 | 1.311745 | 4.517152 | 16.19499 | 9.31E-49 | 9.50E-47 | 99.89181 |
| ENSG00000186832.7 | KRT16 | 1.309606 | 1.374204 | 6.325488 | 5.13E-10 | 4.12E-09 | 11.44915 |
| ENSG00000174938.13 | SEZ6L2 | 1.309586 | 4.138773 | 7.474211 | 2.96E-13 | 3.26E-12 | 18.7592 |
| ENSG00000243649.7 | CFB | 1.30747 | 2.504603 | 8.816256 | 1.45E-17 | 2.27E-16 | 28.54074 |
| ENSG00000169469.8 | SPRR1B | 1.305802 | 1.363072 | 4.888982 | 1.32E-06 | 7.19E-06 | 3.821615 |
| ENSG00000013810.17 | TACC3 | 1.305711 | 2.898792 | 12.42803 | 1.58E-31 | 6.28E-30 | 60.41796 |
| ENSG00000174371.15 | EXO1 | 1.305253 | 1.414705 | 12.04529 | 6.56E-30 | 2.36E-28 | 56.71369 |
| ENSG00000164692.16 | COL1A2 | 1.304334 | 6.808059 | 7.188218 | 2.08E-12 | 2.10E-11 | 16.84376 |
| ENSG00000182489.8 | XKRX | 1.303396 | 1.862087 | 7.888882 | 1.58E-14 | 1.93E-13 | 21.64457 |
| ENSG00000111344.10 | RASAL1 | 1.302915 | 1.34723 | 11.2587 | 1.12E-26 | 3.30E-25 | 49.32094 |
| ENSG00000151012.12 | SLC7A11 | 1.300475 | 1.706282 | 7.416523 | 4.41E-13 | 4.78E-12 | 18.36787 |
| ENSG00000180535.3 | BHLHA15 | 1.299673 | 1.750602 | 8.709045 | 3.36E-17 | 5.08E-16 | 27.71325 |
| ENSG00000106077.17 | ABHD11 | 1.297987 | 4.30405 | 12.40701 | 1.94E-31 | 7.68E-30 | 60.21278 |
| ENSG00000156802.11 | ATAD2 | 1.297775 | 2.671905 | 11.5155 | 1.02E-27 | 3.20E-26 | 51.70064 |
| ENSG00000198648.9 | STK39 | 1.29692 | 3.747466 | 12.02004 | 8.38E-30 | 2.99E-28 | 56.47167 |
| ENSG00000165449.10 | SLC16A9 | 1.294328 | 1.845691 | 6.645545 | 7.11E-11 | 6.22E-10 | 13.3819 |
| ENSG00000104341.15 | LAPTM4B | 1.293329 | 6.388122 | 9.976933 | 1.05E-21 | 2.21E-20 | 37.97419 |
| ENSG00000162433.13 | AK4 | 1.293169 | 1.775229 | 9.477141 | 7.06E-20 | 1.30E-18 | 33.80808 |
| ENSG00000120875.7 | DUSP4 | 1.290403 | 3.008931 | 6.295933 | 6.13E-10 | 4.88E-09 | 11.27484 |
| ENSG00000105699.15 | LSR | 1.289299 | 6.190793 | 11.67835 | 2.20E-28 | 7.23E-27 | 53.22697 |
| ENSG00000171163.14 | ZNF692 | 1.289018 | 2.86638 | 11.65972 | 2.62E-28 | 8.60E-27 | 53.0517 |
| ENSG00000068489.11 | PRR11 | 1.288721 | 2.058793 | 10.38528 | 3.02E-23 | 7.05E-22 | 41.48889 |
| ENSG00000127564.15 | PKMYT1 | 1.287634 | 1.504891 | 12.65939 | 1.60E-32 | 6.80E-31 | 62.68932 |
| ENSG00000146242.7 | TPBG | 1.285203 | 2.591525 | 12.36032 | 3.07E-31 | 1.19E-29 | 59.7578 |
| ENSG00000104522.14 | TSTA3 | 1.285025 | 4.795818 | 13.14142 | 1.28E-34 | 6.15E-33 | 67.49544 |
| ENSG00000165480.14 | SKA3 | 1.283058 | 1.460651 | 12.47091 | 1.03E-31 | 4.16E-30 | 60.83716 |
| ENSG00000187840.4 | EIF4EBP1 | 1.282638 | 4.887282 | 8.731582 | 2.82E-17 | 4.29E-16 | 27.88656 |
| ENSG00000173227.12 | SYT12 | 1.282483 | 1.226375 | 7.613038 | 1.13E-13 | 1.28E-12 | 19.71109 |
| ENSG00000128039.9 | SRD5A3 | 1.281798 | 3.883202 | 9.714182 | 9.77E-21 | 1.91E-19 | 35.76494 |
| ENSG00000181218.5 | HIST3H2A | 1.280656 | 2.541088 | 6.222843 | 9.50E-10 | 7.42E-09 | 10.84681 |
| ENSG00000163082.9 | SGPP2 | 1.279394 | 4.232947 | 7.946487 | 1.04E-14 | 1.29E-13 | 22.05532 |
| ENSG00000056998.17 | GYG2 | 1.278442 | 1.884319 | 11.0825 | 5.71E-26 | 1.60E-24 | 47.70784 |
| ENSG00000183605.15 | SFXN4 | 1.278271 | 3.7403 | 16.68969 | 3.71E-51 | 4.17E-49 | 105.3965 |
| ENSG00000160688.17 | FLAD1 | 1.277609 | 3.712648 | 16.73089 | 2.33E-51 | 2.65E-49 | 105.8575 |
| ENSG00000135127.10 | CCDC64 | 1.277008 | 2.051876 | 10.55608 | 6.67E-24 | 1.63E-22 | 42.98749 |
| ENSG00000258947.5 | TUBB3 | 1.276956 | 1.238397 | 11.77341 | 8.90E-29 | 3.00E-27 | 54.12404 |
| ENSG00000175793.11 | SFN | 1.274409 | 6.664542 | 8.041642 | 5.20E-15 | 6.61E-14 | 22.73905 |
| ENSG00000145358.5 | DDIT4L | 1.27328 | 1.610697 | 7.787879 | 3.26E-14 | 3.88E-13 | 20.93016 |
| ENSG00000130590.12 | SAMD10 | 1.271893 | 2.463712 | 14.59057 | 3.63E-41 | 2.51E-39 | 82.48989 |
| ENSG00000115541.9 | HSPE1 | 1.270026 | 5.314398 | 14.66208 | 1.69E-41 | 1.21E-39 | 83.24899 |
| ENSG00000167747.12 | C19orf48 | 1.268699 | 3.654119 | 12.90706 | 1.35E-33 | 6.13E-32 | 65.14647 |
| ENSG00000198286.8 | CARD11 | 1.268143 | 2.589606 | 8.733553 | 2.78E-17 | 4.23E-16 | 27.90174 |
| ENSG00000144381.15 | HSPD1 | 1.265888 | 6.126438 | 14.52559 | 7.24E-41 | 4.96E-39 | 81.80145 |
| ENSG00000136261.13 | BZW2 | 1.265345 | 4.592658 | 15.11297 | 1.33E-43 | 1.05E-41 | 88.07309 |
| ENSG00000163918.9 | RFC4 | 1.264791 | 2.598693 | 12.0746 | 4.95E-30 | 1.79E-28 | 56.99493 |
| ENSG00000076003.4 | MCM6 | 1.262136 | 3.617115 | 12.19619 | 1.52E-30 | 5.68E-29 | 58.16609 |
| ENSG00000205336.10 | GPR56 | 1.26056 | 4.198919 | 9.867481 | 2.67E-21 | 5.46E-20 | 37.04884 |
| ENSG00000074800.12 | ENO1 | 1.260076 | 8.736737 | 14.83892 | 2.55E-42 | 1.90E-40 | 85.13343 |
| ENSG00000145113.20 | MUC4 | 1.25957 | 2.123911 | 6.336846 | 4.79E-10 | 3.85E-09 | 11.51633 |
| ENSG00000187017.13 | ESPN | 1.258039 | 1.813937 | 8.407364 | 3.41E-16 | 4.76E-15 | 25.42658 |
| ENSG00000132000.10 | PODNL1 | 1.256929 | 1.719899 | 8.423351 | 3.02E-16 | 4.23E-15 | 25.54619 |
| ENSG00000163568.12 | AIM2 | 1.255363 | 1.863384 | 7.064819 | 4.74E-12 | 4.64E-11 | 16.03649 |
| ENSG00000132680.9 | KIAA0907 | 1.254908 | 3.244949 | 13.58469 | 1.38E-36 | 7.53E-35 | 71.99889 |
| ENSG00000167656.4 | LY6D | 1.254054 | 1.261786 | 5.575109 | 3.83E-08 | 2.52E-07 | 7.246449 |
| ENSG00000142632.15 | ARHGEF19 | 1.2526 | 3.225322 | 10.62974 | 3.46E-24 | 8.61E-23 | 43.63884 |
| ENSG00000013306.14 | SLC25A39 | 1.25207 | 5.563326 | 16.58463 | 1.20E-50 | 1.33E-48 | 104.2227 |
| ENSG00000188486.3 | H2AFX | 1.249036 | 4.5984 | 12.33277 | 4.01E-31 | 1.55E-29 | 59.4898 |
| ENSG00000112742.8 | TTK | 1.24843 | 1.413948 | 11.19485 | 2.03E-26 | 5.87E-25 | 48.73451 |
| ENSG00000185090.13 | MANEAL | 1.247432 | 2.556856 | 9.747356 | 7.39E-21 | 1.46E-19 | 36.04156 |
| ENSG00000021826.13 | CPS1 | 1.247379 | 1.410909 | 3.876151 | 0.000118543 | 0.00048781 | -0.47128 |
| ENSG00000137807.12 | KIF23 | 1.245197 | 1.677728 | 11.35324 | 4.67E-27 | 1.41E-25 | 50.19305 |
| ENSG00000138363.13 | ATIC | 1.239593 | 4.599287 | 19.11858 | 3.02E-63 | 5.68E-61 | 133.1346 |
| ENSG00000158406.3 | HIST1H4H | 1.238921 | 2.46541 | 7.506928 | 2.36E-13 | 2.62E-12 | 18.98223 |
| ENSG00000092607.12 | TBX15 | 1.238271 | 1.373641 | 8.451599 | 2.44E-16 | 3.44E-15 | 25.75797 |
| ENSG00000128059.7 | PPAT | 1.238113 | 2.098202 | 15.416 | 4.94E-45 | 4.22E-43 | 91.35003 |
| ENSG00000148848.13 | ADAM12 | 1.237661 | 1.516004 | 9.249186 | 4.58E-19 | 7.99E-18 | 31.95943 |
| ENSG00000060140.7 | STYK1 | 1.236903 | 1.664673 | 11.69433 | 1.89E-28 | 6.23E-27 | 53.37749 |
| ENSG00000100162.13 | CENPM | 1.236698 | 2.252922 | 11.27199 | 9.94E-27 | 2.93E-25 | 49.44327 |
| ENSG00000163053.9 | SLC16A14 | 1.236008 | 2.052322 | 6.149746 | 1.47E-09 | 1.12E-08 | 10.4231 |
| ENSG00000167772.10 | ANGPTL4 | 1.235023 | 3.53855 | 5.487947 | 6.14E-08 | 3.94E-07 | 6.788877 |
| ENSG00000164694.15 | FNDC1 | 1.234771 | 2.075956 | 7.226644 | 1.61E-12 | 1.65E-11 | 17.09752 |
| ENSG00000169258.6 | GPRIN1 | 1.233969 | 1.556137 | 12.14368 | 2.53E-30 | 9.28E-29 | 57.6595 |
| ENSG00000137288.8 | UQCC2 | 1.233075 | 3.147555 | 14.07188 | 8.71E-39 | 5.37E-37 | 77.03511 |
| ENSG00000140525.16 | FANCI | 1.232491 | 2.074326 | 12.78096 | 4.78E-33 | 2.11E-31 | 63.89216 |
| ENSG00000129195.14 | FAM64A | 1.231841 | 1.41499 | 10.75473 | 1.13E-24 | 2.91E-23 | 44.751 |
| ENSG00000029153.13 | ARNTL2 | 1.23169 | 2.023699 | 8.186873 | 1.78E-15 | 2.35E-14 | 23.79502 |
| ENSG00000137285.9 | TUBB2B | 1.229043 | 1.568041 | 5.681412 | 2.14E-08 | 1.45E-07 | 7.81324 |
| ENSG00000070669.15 | ASNS | 1.228598 | 3.017287 | 10.98766 | 1.36E-25 | 3.73E-24 | 46.84636 |
| ENSG00000132746.13 | ALDH3B2 | 1.22767 | 1.70152 | 7.730501 | 4.91E-14 | 5.74E-13 | 20.52762 |
| ENSG00000170608.2 | FOXA3 | 1.227063 | 1.572944 | 7.292663 | 1.03E-12 | 1.08E-11 | 17.53611 |
| ENSG00000196407.10 | THEM5 | 1.226397 | 2.538091 | 8.7966 | 1.70E-17 | 2.63E-16 | 28.38845 |
| ENSG00000035499.11 | DEPDC1B | 1.225459 | 1.314747 | 11.70167 | 1.76E-28 | 5.83E-27 | 53.44662 |
| ENSG00000134028.13 | ADAMDEC1 | 1.225254 | 1.909354 | 7.049803 | 5.23E-12 | 5.10E-11 | 15.93906 |
| ENSG00000101457.11 | DNTTIP1 | 1.222454 | 4.793288 | 9.787682 | 5.26E-21 | 1.05E-19 | 36.37872 |
| ENSG00000160949.15 | TONSL | 1.222359 | 1.87306 | 13.01626 | 4.52E-34 | 2.11E-32 | 66.2381 |
| ENSG00000188706.11 | ZDHHC9 | 1.221576 | 5.054447 | 11.01317 | 1.08E-25 | 2.99E-24 | 47.07762 |
| ENSG00000165434.7 | PGM2L1 | 1.221392 | 2.300228 | 11.93839 | 1.84E-29 | 6.44E-28 | 55.69117 |
| ENSG00000184216.10 | IRAK1 | 1.22082 | 5.344928 | 14.6947 | 1.19E-41 | 8.59E-40 | 83.59585 |
| ENSG00000132744.6 | ACY3 | 1.220346 | 1.552617 | 9.019757 | 2.90E-18 | 4.78E-17 | 30.13245 |
| ENSG00000181617.5 | FDCSP | 1.219647 | 2.018674 | 5.346671 | 1.30E-07 | 8.04E-07 | 6.06101 |
| ENSG00000176788.8 | BASP1 | 1.218208 | 4.67469 | 6.760512 | 3.43E-11 | 3.10E-10 | 14.09609 |
| ENSG00000005001.8 | PRSS22 | 1.21502 | 3.303557 | 7.948519 | 1.03E-14 | 1.27E-13 | 22.06986 |
| ENSG00000167123.17 | CERCAM | 1.214701 | 3.147457 | 10.44122 | 1.85E-23 | 4.39E-22 | 41.9779 |
| ENSG00000132470.12 | ITGB4 | 1.2128 | 4.524272 | 6.298938 | 6.02E-10 | 4.79E-09 | 11.29253 |
| ENSG00000062716.9 | VMP1 | 1.212729 | 5.083806 | 13.11775 | 1.62E-34 | 7.78E-33 | 67.25712 |
| ENSG00000149925.15 | ALDOA | 1.21218 | 7.629679 | 13.53561 | 2.28E-36 | 1.23E-34 | 71.49642 |
| ENSG00000108106.12 | UBE2S | 1.211716 | 2.665561 | 9.774994 | 5.85E-21 | 1.16E-19 | 36.27253 |
| ENSG00000038427.14 | VCAN | 1.210886 | 3.577353 | 7.282567 | 1.10E-12 | 1.15E-11 | 17.46883 |
| ENSG00000173848.17 | NET1 | 1.210549 | 5.116422 | 10.83968 | 5.23E-25 | 1.39E-23 | 45.51175 |
| ENSG00000066279.15 | ASPM | 1.210446 | 1.28859 | 11.80754 | 6.43E-29 | 2.18E-27 | 54.44716 |
| ENSG00000178773.13 | CPNE7 | 1.210138 | 1.432487 | 9.11713 | 1.33E-18 | 2.24E-17 | 30.90368 |
| ENSG00000166736.10 | HTR3A | 1.210037 | 1.154284 | 7.091337 | 3.97E-12 | 3.91E-11 | 16.20899 |
| ENSG00000123989.12 | CHPF | 1.210003 | 5.353334 | 11.37789 | 3.71E-27 | 1.12E-25 | 50.42125 |
| ENSG00000164466.11 | SFXN1 | 1.20903 | 2.736437 | 18.24326 | 7.71E-59 | 1.21E-56 | 123.0214 |
| ENSG00000121211.6 | MND1 | 1.20753 | 1.432864 | 13.26721 | 3.56E-35 | 1.77E-33 | 68.76547 |
| ENSG00000155792.8 | DEPTOR | 1.205457 | 3.224214 | 9.032739 | 2.62E-18 | 4.33E-17 | 30.23491 |
| ENSG00000180596.7 | HIST1H2BC | 1.204333 | 2.599692 | 7.170254 | 2.35E-12 | 2.36E-11 | 16.72552 |
| ENSG00000130821.14 | SLC6A8 | 1.204164 | 2.656285 | 8.247325 | 1.14E-15 | 1.52E-14 | 24.23896 |
| ENSG00000277075.1 | HIST1H2AE | 1.203558 | 1.474586 | 6.504528 | 1.71E-10 | 1.45E-09 | 12.52021 |
| ENSG00000185567.6 | AHNAK2 | 1.202019 | 2.165326 | 7.142437 | 2.83E-12 | 2.82E-11 | 16.54291 |
| ENSG00000106484.13 | MEST | 1.199748 | 3.784313 | 11.06472 | 6.73E-26 | 1.88E-24 | 47.54593 |
| ENSG00000149260.13 | CAPN5 | 1.199587 | 2.769126 | 7.872107 | 1.78E-14 | 2.17E-13 | 21.5254 |
| ENSG00000117122.12 | MFAP2 | 1.198816 | 3.05854 | 8.445688 | 2.55E-16 | 3.59E-15 | 25.71361 |
| ENSG00000188483.7 | IER5L | 1.197398 | 2.417275 | 9.737648 | 8.02E-21 | 1.58E-19 | 35.96054 |
| ENSG00000104415.12 | WISP1 | 1.195589 | 1.80977 | 10.31037 | 5.84E-23 | 1.34E-21 | 40.8369 |
| ENSG00000101842.12 | VSIG1 | 1.195353 | 1.858851 | 4.421737 | 1.17E-05 | 5.62E-05 | 1.726053 |
| ENSG00000150753.10 | CCT5 | 1.19352 | 5.187912 | 12.74732 | 6.69E-33 | 2.91E-31 | 63.55865 |
| ENSG00000042980.11 | ADAM28 | 1.193477 | 1.890287 | 9.856653 | 2.93E-21 | 5.98E-20 | 36.95769 |
| ENSG00000144120.11 | TMEM177 | 1.193354 | 2.324462 | 20.12823 | 2.21E-68 | 5.01E-66 | 144.9221 |
| ENSG00000111247.13 | RAD51AP1 | 1.19269 | 1.968517 | 10.35496 | 3.95E-23 | 9.14E-22 | 41.22459 |
| ENSG00000111665.10 | CDCA3 | 1.19003 | 1.350141 | 11.98958 | 1.12E-29 | 3.98E-28 | 56.18019 |
| ENSG00000085999.10 | RAD54L | 1.189962 | 1.321973 | 11.79547 | 7.22E-29 | 2.44E-27 | 54.33275 |
| ENSG00000158825.5 | CDA | 1.188549 | 2.965697 | 4.912805 | 1.18E-06 | 6.44E-06 | 3.933655 |
| ENSG00000188910.7 | GJB3 | 1.188543 | 1.664357 | 5.91322 | 5.80E-09 | 4.18E-08 | 9.082204 |
| ENSG00000117411.15 | B4GALT2 | 1.188522 | 4.013502 | 16.17864 | 1.12E-48 | 1.13E-46 | 99.71091 |
| ENSG00000085840.11 | ORC1 | 1.188252 | 1.473575 | 11.5795 | 5.60E-28 | 1.78E-26 | 52.29892 |
| ENSG00000240972.1 | MIF | 1.183879 | 5.177513 | 10.42172 | 2.19E-23 | 5.17E-22 | 41.80721 |
| ENSG00000169607.11 | CKAP2L | 1.183781 | 1.349366 | 11.61815 | 3.89E-28 | 1.25E-26 | 52.66124 |
| ENSG00000100522.7 | GNPNAT1 | 1.183641 | 3.256386 | 12.21863 | 1.22E-30 | 4.58E-29 | 58.383 |
| ENSG00000001084.9 | GCLC | 1.183121 | 2.942855 | 6.109827 | 1.86E-09 | 1.41E-08 | 10.19356 |
| ENSG00000133195.10 | SLC39A11 | 1.183039 | 3.601966 | 14.21421 | 1.95E-39 | 1.23E-37 | 78.52271 |
| ENSG00000129173.11 | E2F8 | 1.178373 | 1.32156 | 12.81439 | 3.43E-33 | 1.52E-31 | 64.22404 |
| ENSG00000134013.14 | LOXL2 | 1.178004 | 2.923178 | 7.594748 | 1.28E-13 | 1.45E-12 | 19.58486 |
| ENSG00000173137.10 | ADCK5 | 1.177065 | 2.404761 | 12.16108 | 2.14E-30 | 7.90E-29 | 57.82724 |
| ENSG00000185624.13 | P4HB | 1.176122 | 7.834733 | 16.91282 | 3.01E-52 | 3.51E-50 | 107.8979 |
| ENSG00000128298.15 | BAIAP2L2 | 1.175861 | 1.364546 | 7.811653 | 2.75E-14 | 3.29E-13 | 21.09765 |
| ENSG00000168393.11 | DTYMK | 1.175404 | 3.636176 | 13.25123 | 4.18E-35 | 2.07E-33 | 68.60372 |
| ENSG00000024526.15 | DEPDC1 | 1.174669 | 1.225491 | 10.98806 | 1.36E-25 | 3.72E-24 | 46.84999 |
| ENSG00000139629.14 | GALNT6 | 1.1745 | 2.936862 | 7.241511 | 1.45E-12 | 1.50E-11 | 17.196 |
| ENSG00000179750.14 | APOBEC3B | 1.173633 | 1.943336 | 7.72242 | 5.20E-14 | 6.06E-13 | 20.47113 |
| ENSG00000243449.5 | C4orf48 | 1.171781 | 2.680383 | 8.935464 | 5.68E-18 | 9.18E-17 | 29.46982 |
| ENSG00000273802.1 | HIST1H2BG | 1.171649 | 1.363828 | 6.759086 | 3.46E-11 | 3.12E-10 | 14.08717 |
| ENSG00000046604.11 | DSG2 | 1.171412 | 4.704848 | 8.611026 | 7.18E-17 | 1.06E-15 | 26.96349 |
| ENSG00000172724.10 | CCL19 | 1.169626 | 4.046054 | 4.97246 | 8.77E-07 | 4.88E-06 | 4.216413 |
| ENSG00000215788.8 | TNFRSF25 | 1.168933 | 1.969554 | 9.532307 | 4.47E-20 | 8.35E-19 | 34.26038 |
| ENSG00000101825.7 | MXRA5 | 1.167884 | 3.299705 | 7.00675 | 6.95E-12 | 6.70E-11 | 15.66067 |
| ENSG00000196684.11 | HSH2D | 1.167445 | 2.74663 | 12.3233 | 4.40E-31 | 1.69E-29 | 59.39769 |
| ENSG00000078098.12 | FAP | 1.166176 | 1.742898 | 9.947429 | 1.35E-21 | 2.82E-20 | 37.72405 |
| ENSG00000151632.15 | AKR1C2 | 1.165789 | 2.532322 | 3.077462 | 0.002188621 | 0.007091738 | -3.19039 |
| ENSG00000204394.11 | VARS | 1.16499 | 4.374808 | 15.03958 | 2.94E-43 | 2.28E-41 | 87.28362 |
| ENSG00000127831.9 | VIL1 | 1.164767 | 1.084668 | 5.578041 | 3.77E-08 | 2.48E-07 | 7.261955 |
| ENSG00000125319.13 | C17orf53 | 1.163372 | 1.448038 | 11.97127 | 1.34E-29 | 4.72E-28 | 56.00514 |
| ENSG00000278828.1 | HIST1H3H | 1.163129 | 1.682123 | 8.021135 | 6.04E-15 | 7.64E-14 | 22.59115 |
| ENSG00000189060.5 | H1F0 | 1.163077 | 6.93015 | 11.98579 | 1.16E-29 | 4.12E-28 | 56.14389 |
| ENSG00000152253.7 | SPC25 | 1.162956 | 1.606967 | 11.26429 | 1.07E-26 | 3.14E-25 | 49.37238 |
| ENSG00000037897.15 | METTL1 | 1.162462 | 3.190671 | 13.34695 | 1.58E-35 | 8.10E-34 | 69.57385 |
| ENSG00000154760.12 | SLFN13 | 1.162144 | 2.335061 | 10.59035 | 4.91E-24 | 1.22E-22 | 43.29015 |
| ENSG00000184697.6 | CLDN6 | 1.159673 | 1.27082 | 4.313346 | 1.90E-05 | 8.81E-05 | 1.26792 |
| ENSG00000128422.14 | KRT17 | 1.159414 | 3.647801 | 3.997766 | 7.24E-05 | 0.000308479 | -0.00508 |
| ENSG00000172061.8 | LRRC15 | 1.158908 | 1.843876 | 6.551563 | 1.28E-10 | 1.09E-09 | 12.80585 |
| ENSG00000262814.5 | MRPL12 | 1.157533 | 4.386823 | 11.51823 | 9.97E-28 | 3.13E-26 | 51.72612 |
| ENSG00000173894.9 | CBX2 | 1.157518 | 1.652164 | 8.502335 | 1.65E-16 | 2.37E-15 | 26.13972 |
| ENSG00000135916.14 | ITM2C | 1.157325 | 5.59795 | 10.02079 | 7.20E-22 | 1.53E-20 | 38.34703 |
| ENSG00000137203.9 | TFAP2A | 1.156706 | 1.225315 | 9.750566 | 7.19E-21 | 1.42E-19 | 36.06836 |
| ENSG00000160813.5 | PPP1R35 | 1.156398 | 3.849775 | 11.85927 | 3.92E-29 | 1.35E-27 | 54.93801 |
| ENSG00000135744.7 | AGT | 1.155648 | 2.322922 | 5.169002 | 3.27E-07 | 1.92E-06 | 5.170046 |
| ENSG00000126249.6 | PDCD2L | 1.153619 | 2.64678 | 13.98608 | 2.14E-38 | 1.30E-36 | 76.1418 |
| ENSG00000143476.16 | DTL | 1.152426 | 1.864771 | 11.28775 | 8.59E-27 | 2.54E-25 | 49.5884 |
| ENSG00000150551.10 | LYPD1 | 1.151787 | 1.219036 | 8.614414 | 7.00E-17 | 1.03E-15 | 26.9893 |
| ENSG00000272398.4 | CD24 | 1.150854 | 5.978274 | 6.032721 | 2.91E-09 | 2.16E-08 | 9.753892 |
| ENSG00000260027.4 | HOXB7 | 1.150499 | 2.411155 | 6.444045 | 2.49E-10 | 2.07E-09 | 12.1555 |
| ENSG00000169718.16 | DUS1L | 1.150022 | 4.126915 | 14.46192 | 1.42E-40 | 9.46E-39 | 81.12842 |
| ENSG00000182004.11 | SNRPE | 1.14946 | 5.014545 | 15.20513 | 4.90E-44 | 3.97E-42 | 89.06683 |
| ENSG00000146918.18 | NCAPG2 | 1.148241 | 2.199527 | 11.42454 | 2.40E-27 | 7.35E-26 | 50.85389 |
| ENSG00000115226.8 | FNDC4 | 1.145474 | 2.385597 | 7.501424 | 2.45E-13 | 2.72E-12 | 18.94466 |
| ENSG00000135318.10 | NT5E | 1.145281 | 3.39196 | 5.838272 | 8.89E-09 | 6.27E-08 | 8.667 |
| ENSG00000143850.11 | PLEKHA6 | 1.145167 | 2.722084 | 8.48036 | 1.96E-16 | 2.79E-15 | 25.97416 |
| ENSG00000141526.13 | SLC16A3 | 1.144583 | 4.12669 | 8.551073 | 1.14E-16 | 1.65E-15 | 26.50811 |
| ENSG00000185507.18 | IRF7 | 1.14321 | 4.166081 | 10.37645 | 3.27E-23 | 7.60E-22 | 41.41189 |
| ENSG00000112578.8 | BYSL | 1.141789 | 3.502734 | 13.48799 | 3.72E-36 | 1.98E-34 | 71.00985 |
| ENSG00000122966.12 | CIT | 1.14174 | 2.744262 | 6.104953 | 1.91E-09 | 1.44E-08 | 10.16562 |
| ENSG00000111669.13 | TPI1 | 1.140847 | 7.517442 | 13.43647 | 6.31E-36 | 3.30E-34 | 70.48437 |
| ENSG00000111057.9 | KRT18 | 1.140456 | 7.644946 | 10.11933 | 3.08E-22 | 6.74E-21 | 39.18877 |
| ENSG00000035141.6 | FAM136A | 1.139179 | 3.942792 | 17.61865 | 9.98E-56 | 1.36E-53 | 115.8796 |
| ENSG00000170779.10 | CDCA4 | 1.137929 | 2.924653 | 11.40757 | 2.81E-27 | 8.58E-26 | 50.69638 |
| ENSG00000206053.11 | HN1L | 1.137525 | 5.042926 | 15.3411 | 1.12E-44 | 9.37E-43 | 90.53756 |
| ENSG00000110400.9 | PVRL1 | 1.137047 | 2.664933 | 9.35578 | 1.92E-19 | 3.43E-18 | 32.81977 |
| ENSG00000173715.14 | C11orf80 | 1.136721 | 2.684422 | 14.46396 | 1.39E-40 | 9.30E-39 | 81.14989 |
| ENSG00000149257.12 | SERPINH1 | 1.136319 | 5.703023 | 12.1134 | 3.40E-30 | 1.24E-28 | 57.36792 |
| ENSG00000141682.11 | PMAIP1 | 1.134691 | 2.888746 | 7.20326 | 1.88E-12 | 1.91E-11 | 16.94296 |
| ENSG00000171241.7 | SHCBP1 | 1.134227 | 1.589674 | 11.9196 | 2.20E-29 | 7.66E-28 | 55.51205 |
| ENSG00000162063.11 | CCNF | 1.133796 | 1.856948 | 13.20392 | 6.77E-35 | 3.30E-33 | 68.12568 |
| ENSG00000164877.17 | MICALL2 | 1.132846 | 2.547687 | 10.15097 | 2.34E-22 | 5.17E-21 | 39.46029 |
| ENSG00000278259.3 | MYO19 | 1.131326 | 2.124603 | 15.2027 | 5.03E-44 | 4.07E-42 | 89.04055 |
| ENSG00000154839.8 | SKA1 | 1.131278 | 1.312288 | 10.77817 | 9.11E-25 | 2.37E-23 | 44.9605 |
| ENSG00000158435.6 | CNOT11 | 1.130025 | 4.755711 | 17.86126 | 6.22E-57 | 9.11E-55 | 118.6455 |
| ENSG00000106367.12 | AP1S1 | 1.129265 | 5.139101 | 13.60473 | 1.12E-36 | 6.16E-35 | 72.2043 |
| ENSG00000167476.9 | JSRP1 | 1.128434 | 1.422425 | 7.852461 | 2.05E-14 | 2.48E-13 | 21.38611 |
| ENSG00000143578.14 | CREB3L4 | 1.128362 | 3.115507 | 11.70827 | 1.65E-28 | 5.48E-27 | 53.50885 |
| ENSG00000137673.7 | MMP7 | 1.128345 | 4.18011 | 4.249066 | 2.51E-05 | 0.00011449 | 1.001257 |
| ENSG00000101213.6 | PTK6 | 1.127988 | 3.004526 | 7.311177 | 9.07E-13 | 9.57E-12 | 17.6597 |
| ENSG00000171940.12 | ZNF217 | 1.127576 | 3.749452 | 11.62323 | 3.70E-28 | 1.20E-26 | 52.70884 |
| ENSG00000161179.12 | YDJC | 1.127041 | 3.614206 | 12.36251 | 3.00E-31 | 1.17E-29 | 59.7791 |
| ENSG00000100625.8 | SIX4 | 1.12563 | 1.699004 | 12.00316 | 9.86E-30 | 3.50E-28 | 56.31004 |
| ENSG00000133216.15 | EPHB2 | 1.125388 | 1.716018 | 9.144881 | 1.06E-18 | 1.81E-17 | 31.12461 |
| ENSG00000189143.9 | CLDN4 | 1.125219 | 6.329806 | 9.162913 | 9.20E-19 | 1.57E-17 | 31.26843 |
| ENSG00000091651.7 | ORC6 | 1.125095 | 1.282755 | 12.83634 | 2.75E-33 | 1.23E-31 | 64.44218 |
| ENSG00000187608.7 | ISG15 | 1.124669 | 5.988007 | 6.146672 | 1.49E-09 | 1.14E-08 | 10.40538 |
| ENSG00000187837.3 | HIST1H1C | 1.12338 | 5.791413 | 6.366408 | 4.00E-10 | 3.25E-09 | 11.69166 |
| ENSG00000163882.8 | POLR2H | 1.122953 | 4.014487 | 15.55916 | 1.03E-45 | 9.16E-44 | 92.90742 |
| ENSG00000067177.13 | PHKA1 | 1.122502 | 2.157701 | 14.26863 | 1.10E-39 | 6.99E-38 | 79.09329 |
| ENSG00000163975.10 | MFI2 | 1.122405 | 1.501221 | 7.920616 | 1.26E-14 | 1.55E-13 | 21.87055 |
| ENSG00000103888.14 | CEMIP | 1.122066 | 2.551774 | 6.201145 | 1.08E-09 | 8.41E-09 | 10.72058 |
| ENSG00000104524.12 | PYCRL | 1.1214 | 2.866819 | 12.38308 | 2.45E-31 | 9.60E-30 | 59.97944 |
| ENSG00000146013.9 | GFRA3 | 1.121269 | 2.068164 | 4.336494 | 1.71E-05 | 8.01E-05 | 1.364866 |
| ENSG00000141562.16 | NARF | 1.1212 | 3.241683 | 15.89457 | 2.59E-47 | 2.49E-45 | 96.57851 |
| ENSG00000166508.16 | MCM7 | 1.120232 | 4.61388 | 10.38975 | 2.91E-23 | 6.79E-22 | 41.52796 |
| ENSG00000006453.12 | BAIAP2L1 | 1.119717 | 4.150995 | 12.05383 | 6.04E-30 | 2.18E-28 | 56.7956 |
| ENSG00000104889.4 | RNASEH2A | 1.118865 | 3.372995 | 11.60576 | 4.37E-28 | 1.40E-26 | 52.54497 |
| ENSG00000178401.13 | DNAJC22 | 1.118776 | 1.22635 | 9.0509 | 2.26E-18 | 3.76E-17 | 30.37844 |
| ENSG00000066248.13 | NGEF | 1.118643 | 1.328155 | 8.749038 | 2.46E-17 | 3.76E-16 | 28.02103 |
| ENSG00000077348.7 | EXOSC5 | 1.117945 | 3.723236 | 12.76727 | 5.48E-33 | 2.40E-31 | 63.75632 |
| ENSG00000116128.8 | BCL9 | 1.117245 | 3.032545 | 12.23742 | 1.02E-30 | 3.83E-29 | 58.5648 |
| ENSG00000185101.11 | ANO9 | 1.116772 | 2.504482 | 8.551647 | 1.13E-16 | 1.65E-15 | 26.51246 |
| ENSG00000145423.4 | SFRP2 | 1.116471 | 5.108919 | 4.48116 | 8.99E-06 | 4.38E-05 | 1.981719 |
| ENSG00000137868.17 | STRA6 | 1.11633 | 1.126107 | 8.099307 | 3.41E-15 | 4.40E-14 | 23.15654 |
| ENSG00000033170.15 | FUT8 | 1.116072 | 3.158567 | 10.81989 | 6.25E-25 | 1.65E-23 | 45.33422 |
| ENSG00000113758.12 | DBN1 | 1.115389 | 4.269989 | 10.48926 | 1.21E-23 | 2.90E-22 | 42.39924 |
| ENSG00000104213.11 | PDGFRL | 1.114649 | 2.645983 | 8.761185 | 2.24E-17 | 3.43E-16 | 28.11472 |
| ENSG00000135245.9 | HILPDA | 1.112943 | 3.285641 | 7.40577 | 4.75E-13 | 5.12E-12 | 18.2952 |
| ENSG00000163938.15 | GNL3 | 1.112621 | 4.598056 | 15.98529 | 9.52E-48 | 9.42E-46 | 97.57652 |
| ENSG00000147804.8 | SLC39A4 | 1.112084 | 3.231943 | 9.064734 | 2.03E-18 | 3.38E-17 | 30.48792 |
| ENSG00000164626.8 | KCNK5 | 1.109539 | 3.708878 | 6.824108 | 2.28E-11 | 2.09E-10 | 14.49564 |
| ENSG00000112242.13 | E2F3 | 1.109038 | 2.620129 | 14.61123 | 2.91E-41 | 2.04E-39 | 82.70904 |
| ENSG00000129514.5 | FOXA1 | 1.108601 | 3.989792 | 7.279611 | 1.12E-12 | 1.17E-11 | 17.44914 |
| ENSG00000182054.8 | IDH2 | 1.10827 | 5.559362 | 11.40927 | 2.77E-27 | 8.45E-26 | 50.71211 |
| ENSG00000149554.11 | CHEK1 | 1.108104 | 1.606979 | 11.6219 | 3.75E-28 | 1.21E-26 | 52.6964 |
| ENSG00000167702.10 | KIFC2 | 1.107973 | 2.143358 | 8.683259 | 4.11E-17 | 6.16E-16 | 27.51538 |
| ENSG00000105220.13 | GPI | 1.107102 | 5.215322 | 13.23949 | 4.71E-35 | 2.32E-33 | 68.48508 |
| ENSG00000073605.17 | GSDMB | 1.106012 | 2.200623 | 8.977597 | 4.06E-18 | 6.62E-17 | 29.80045 |
| ENSG00000162851.7 | TFB2M | 1.105647 | 3.682654 | 15.25661 | 2.80E-44 | 2.29E-42 | 89.62305 |
| ENSG00000196976.6 | LAGE3 | 1.105297 | 4.424717 | 10.98638 | 1.38E-25 | 3.78E-24 | 46.83476 |
| ENSG00000065328.15 | MCM10 | 1.105122 | 1.180331 | 10.90997 | 2.76E-25 | 7.47E-24 | 46.1442 |
| ENSG00000143621.15 | ILF2 | 1.104399 | 6.557119 | 14.61857 | 2.69E-41 | 1.89E-39 | 82.78685 |
| ENSG00000161800.11 | RACGAP1 | 1.102515 | 2.885776 | 9.787625 | 5.26E-21 | 1.05E-19 | 36.37825 |
| ENSG00000143882.8 | ATP6V1C2 | 1.100587 | 1.475342 | 7.610849 | 1.14E-13 | 1.30E-12 | 19.69597 |
| ENSG00000102109.8 | PCSK1N | 1.099862 | 2.232746 | 4.963386 | 9.18E-07 | 5.10E-06 | 4.173199 |
| ENSG00000101084.15 | C20orf24 | 1.099605 | 4.341646 | 11.82752 | 5.31E-29 | 1.81E-27 | 54.63653 |
| ENSG00000143512.11 | HHIPL2 | 1.099604 | 1.048929 | 6.067716 | 2.37E-09 | 1.78E-08 | 9.952832 |
| ENSG00000203760.7 | CENPW | 1.0991 | 2.961531 | 8.297517 | 7.80E-16 | 1.06E-14 | 24.6095 |
| ENSG00000137547.7 | MRPL15 | 1.097552 | 5.280517 | 11.77955 | 8.40E-29 | 2.84E-27 | 54.18205 |
| ENSG00000112699.9 | GMDS | 1.097525 | 2.812029 | 11.18081 | 2.31E-26 | 6.65E-25 | 48.60581 |
| ENSG00000213886.3 | UBD | 1.097306 | 2.879177 | 5.367593 | 1.17E-07 | 7.25E-07 | 6.167725 |
| ENSG00000184445.10 | KNTC1 | 1.097209 | 1.654352 | 12.38415 | 2.43E-31 | 9.51E-30 | 59.98988 |
| ENSG00000180921.6 | FAM83H | 1.097188 | 4.413981 | 10.22315 | 1.25E-22 | 2.82E-21 | 40.08176 |
| ENSG00000165548.9 | TMEM63C | 1.096357 | 1.138825 | 8.053316 | 4.78E-15 | 6.08E-14 | 22.82337 |
| ENSG00000131462.6 | TUBG1 | 1.095762 | 4.13991 | 12.73573 | 7.51E-33 | 3.24E-31 | 63.44386 |
| ENSG00000101412.12 | E2F1 | 1.094315 | 3.002075 | 8.544536 | 1.20E-16 | 1.73E-15 | 26.4586 |
| ENSG00000149428.17 | HYOU1 | 1.094231 | 4.983162 | 11.61911 | 3.85E-28 | 1.24E-26 | 52.67021 |
| ENSG00000105219.7 | CNTD2 | 1.093594 | 1.121721 | 7.989628 | 7.61E-15 | 9.52E-14 | 22.3645 |
| ENSG00000108179.12 | PPIF | 1.093525 | 4.457675 | 13.84917 | 8.91E-38 | 5.17E-36 | 74.72198 |
| ENSG00000240204.2 | SMKR1 | 1.091688 | 1.575244 | 6.677045 | 5.83E-11 | 5.15E-10 | 13.57654 |
| ENSG00000130826.14 | DKC1 | 1.090857 | 4.140636 | 15.2334 | 3.61E-44 | 2.94E-42 | 89.3721 |
| ENSG00000164087.6 | POC1A | 1.090151 | 2.21885 | 12.21567 | 1.26E-30 | 4.71E-29 | 58.35437 |
| ENSG00000177732.7 | SOX12 | 1.089869 | 2.840973 | 10.70617 | 1.74E-24 | 4.44E-23 | 44.31792 |
| ENSG00000162073.12 | PAQR4 | 1.085433 | 2.590198 | 10.89542 | 3.15E-25 | 8.49E-24 | 46.01304 |
| ENSG00000182134.14 | TDRKH | 1.083748 | 2.704517 | 12.65597 | 1.66E-32 | 7.02E-31 | 62.65549 |
| ENSG00000149269.8 | PAK1 | 1.083386 | 3.753928 | 14.37295 | 3.66E-40 | 2.38E-38 | 80.19008 |
| ENSG00000112312.8 | GMNN | 1.083157 | 2.8867 | 11.39565 | 3.14E-27 | 9.55E-26 | 50.58582 |
| ENSG00000126602.9 | TRAP1 | 1.083111 | 3.559722 | 16.55584 | 1.66E-50 | 1.82E-48 | 103.9014 |
| ENSG00000163382.10 | APOA1BP | 1.083093 | 5.852828 | 11.76955 | 9.24E-29 | 3.11E-27 | 54.08751 |
| ENSG00000119431.8 | HDHD3 | 1.082286 | 3.775198 | 12.53094 | 5.72E-32 | 2.34E-30 | 61.42534 |
| ENSG00000128228.4 | SDF2L1 | 1.080992 | 4.757046 | 10.90119 | 2.99E-25 | 8.08E-24 | 46.06506 |
| ENSG00000049089.12 | COL9A2 | 1.078485 | 2.417723 | 6.253685 | 7.90E-10 | 6.22E-09 | 11.0269 |
| ENSG00000116649.8 | SRM | 1.0772 | 4.993122 | 13.44853 | 5.58E-36 | 2.92E-34 | 70.60731 |
| ENSG00000197903.7 | HIST1H2BK | 1.075091 | 5.879802 | 7.423941 | 4.19E-13 | 4.55E-12 | 18.41805 |
| ENSG00000116191.16 | RALGPS2 | 1.074319 | 2.060787 | 12.92782 | 1.10E-33 | 5.01E-32 | 65.35361 |
| ENSG00000157399.13 | ARSE | 1.073938 | 2.683119 | 5.072576 | 5.33E-07 | 3.05E-06 | 4.697962 |
| ENSG00000159259.7 | CHAF1B | 1.073681 | 1.690229 | 13.27447 | 3.30E-35 | 1.65E-33 | 68.83897 |
| ENSG00000168275.13 | COA6 | 1.073102 | 4.16049 | 11.23312 | 1.43E-26 | 4.15E-25 | 49.08571 |
| ENSG00000164749.10 | HNF4G | 1.072497 | 1.175747 | 9.797855 | 4.82E-21 | 9.69E-20 | 36.46394 |
| ENSG00000184368.14 | MAP7D2 | 1.071075 | 1.242424 | 7.301686 | 9.68E-13 | 1.02E-11 | 17.59632 |
| ENSG00000110628.12 | SLC22A18 | 1.070382 | 2.737388 | 10.22054 | 1.28E-22 | 2.88E-21 | 40.05927 |
| ENSG00000214160.8 | ALG3 | 1.069807 | 3.970653 | 14.29923 | 7.97E-40 | 5.09E-38 | 79.4146 |
| ENSG00000235173.5 | HGH1 | 1.069621 | 3.629425 | 13.09951 | 1.95E-34 | 9.30E-33 | 67.07368 |
| ENSG00000183317.15 | EPHA10 | 1.069356 | 1.116405 | 11.70469 | 1.71E-28 | 5.67E-27 | 53.47512 |
| ENSG00000079616.11 | KIF22 | 1.069231 | 3.523837 | 13.49302 | 3.53E-36 | 1.89E-34 | 71.06121 |
| ENSG00000102878.14 | HSF4 | 1.068817 | 1.965354 | 7.623363 | 1.05E-13 | 1.19E-12 | 19.78246 |
| ENSG00000143314.11 | MRPL24 | 1.0687 | 5.101203 | 12.7445 | 6.88E-33 | 2.99E-31 | 63.53072 |
| ENSG00000070087.12 | PFN2 | 1.065691 | 4.282138 | 5.941132 | 4.94E-09 | 3.58E-08 | 9.238034 |
| ENSG00000158402.17 | CDC25C | 1.064921 | 1.107545 | 12.16118 | 2.14E-30 | 7.89E-29 | 57.82814 |
| ENSG00000139998.13 | RAB15 | 1.064418 | 3.474739 | 7.673867 | 7.34E-14 | 8.44E-13 | 20.13267 |
| ENSG00000134247.9 | PTGFRN | 1.064089 | 3.765235 | 10.43936 | 1.88E-23 | 4.46E-22 | 41.96165 |
| ENSG00000167157.10 | PRRX2 | 1.064056 | 1.813981 | 6.617344 | 8.49E-11 | 7.37E-10 | 13.20831 |
| ENSG00000135476.10 | ESPL1 | 1.063737 | 1.200915 | 10.69922 | 1.85E-24 | 4.71E-23 | 44.256 |
| ENSG00000153292.14 | GPR110 | 1.063692 | 2.009766 | 4.966426 | 9.04E-07 | 5.02E-06 | 4.187671 |
| ENSG00000131187.8 | F12 | 1.063105 | 1.280838 | 10.30072 | 6.35E-23 | 1.45E-21 | 40.75316 |
| ENSG00000188833.8 | ENTPD8 | 1.061979 | 1.050821 | 8.302923 | 7.49E-16 | 1.02E-14 | 24.64952 |
| ENSG00000242612.5 | DECR2 | 1.06176 | 2.567017 | 12.68971 | 1.19E-32 | 5.06E-31 | 62.98867 |
| ENSG00000164934.12 | DCAF13 | 1.061577 | 2.684034 | 14.02658 | 1.40E-38 | 8.56E-37 | 76.56313 |
| ENSG00000108582.10 | CPD | 1.060829 | 4.410692 | 7.903668 | 1.42E-14 | 1.74E-13 | 21.74977 |
| ENSG00000122565.17 | CBX3 | 1.060425 | 5.391514 | 14.54649 | 5.80E-41 | 3.98E-39 | 82.0227 |
| ENSG00000127586.15 | CHTF18 | 1.060123 | 1.799642 | 11.1778 | 2.38E-26 | 6.83E-25 | 48.57825 |
| ENSG00000184117.10 | NIPSNAP1 | 1.05915 | 4.636148 | 12.81985 | 3.24E-33 | 1.44E-31 | 64.27827 |
| ENSG00000159147.16 | DONSON | 1.058581 | 2.270355 | 13.58265 | 1.41E-36 | 7.68E-35 | 71.97795 |
| ENSG00000165802.18 | NSMF | 1.058002 | 3.419244 | 10.79957 | 7.51E-25 | 1.97E-23 | 45.15207 |
| ENSG00000132646.9 | PCNA | 1.0564 | 5.72915 | 10.78163 | 8.83E-25 | 2.30E-23 | 44.99147 |
| ENSG00000051180.15 | RAD51 | 1.055669 | 1.495428 | 11.52802 | 9.09E-28 | 2.86E-26 | 51.81753 |
| ENSG00000114686.7 | MRPL3 | 1.055511 | 5.006686 | 15.61258 | 5.76E-46 | 5.20E-44 | 93.49008 |
| ENSG00000136026.12 | CKAP4 | 1.055095 | 5.257619 | 11.58668 | 5.23E-28 | 1.67E-26 | 52.36617 |
| ENSG00000197785.12 | ATAD3A | 1.054653 | 3.148833 | 12.74331 | 6.96E-33 | 3.02E-31 | 63.51892 |
| ENSG00000143870.11 | PDIA6 | 1.053034 | 6.045488 | 12.30023 | 5.52E-31 | 2.11E-29 | 59.17359 |
| ENSG00000104413.14 | ESRP1 | 1.052363 | 4.499673 | 11.63174 | 3.42E-28 | 1.11E-26 | 52.7888 |
| ENSG00000108379.8 | WNT3 | 1.052246 | 1.602515 | 11.41145 | 2.71E-27 | 8.28E-26 | 50.73234 |
| ENSG00000148702.13 | HABP2 | 1.051607 | 2.017689 | 4.768421 | 2.36E-06 | 1.24E-05 | 3.26229 |
| ENSG00000125459.13 | MSTO1 | 1.050597 | 2.137887 | 15.93462 | 1.67E-47 | 1.62E-45 | 97.01886 |
| ENSG00000000003.13 | TSPAN6 | 1.050548 | 3.81846 | 11.04929 | 7.75E-26 | 2.16E-24 | 47.40558 |
| ENSG00000141582.13 | CBX4 | 1.04771 | 3.898191 | 13.82427 | 1.15E-37 | 6.63E-36 | 74.46447 |
| ENSG00000100479.11 | POLE2 | 1.047416 | 1.438549 | 11.69357 | 1.90E-28 | 6.27E-27 | 53.37031 |
| ENSG00000143321.17 | HDGF | 1.046865 | 6.625705 | 13.55138 | 1.94E-36 | 1.05E-34 | 71.65774 |
| ENSG00000138615.5 | CILP | 1.045705 | 1.553237 | 6.203541 | 1.07E-09 | 8.30E-09 | 10.7345 |
| ENSG00000180818.4 | HOXC10 | 1.045384 | 0.995357 | 5.3428 | 1.33E-07 | 8.20E-07 | 6.041309 |
| ENSG00000198380.11 | GFPT1 | 1.045191 | 4.304056 | 12.77468 | 5.09E-33 | 2.24E-31 | 63.82982 |
| ENSG00000106683.13 | LIMK1 | 1.044164 | 3.723603 | 10.69307 | 1.96E-24 | 4.96E-23 | 44.2013 |
| ENSG00000198826.9 | ARHGAP11A | 1.043097 | 1.651747 | 10.1671 | 2.04E-22 | 4.51E-21 | 39.59887 |
| ENSG00000149328.13 | GLB1L2 | 1.042534 | 2.398665 | 8.030351 | 5.65E-15 | 7.15E-14 | 22.65758 |
| ENSG00000123999.4 | INHA | 1.041555 | 1.053155 | 4.685612 | 3.50E-06 | 1.80E-05 | 2.885581 |
| ENSG00000091073.18 | DTX2 | 1.041261 | 3.032605 | 10.3271 | 5.04E-23 | 1.16E-21 | 40.98227 |
| ENSG00000163359.14 | COL6A3 | 1.040149 | 4.798639 | 6.61856 | 8.42E-11 | 7.32E-10 | 13.21578 |
| ENSG00000173432.9 | SAA1 | 1.039705 | 3.02805 | 3.835789 | 0.000139219 | 0.000566152 | -0.62299 |
| ENSG00000160180.15 | TFF3 | 1.038295 | 4.342701 | 2.962725 | 0.003177287 | 0.009926743 | -3.53154 |
| ENSG00000170369.3 | CST2 | 1.038131 | 1.478444 | 6.400884 | 3.24E-10 | 2.66E-09 | 11.89704 |
| ENSG00000109654.13 | TRIM2 | 1.03813 | 3.401787 | 7.732477 | 4.84E-14 | 5.66E-13 | 20.54145 |
| ENSG00000171617.12 | ENC1 | 1.036826 | 3.859132 | 8.702637 | 3.53E-17 | 5.32E-16 | 27.66405 |
| ENSG00000115009.10 | CCL20 | 1.036113 | 3.4456 | 3.969832 | 8.12E-05 | 0.000343513 | -0.11337 |
| ENSG00000087085.12 | ACHE | 1.035472 | 1.776207 | 5.824529 | 9.61E-09 | 6.74E-08 | 8.591377 |
| ENSG00000123473.14 | STIL | 1.034701 | 1.305372 | 12.96765 | 7.37E-34 | 3.38E-32 | 65.75157 |
| ENSG00000086232.11 | EIF2AK1 | 1.032078 | 5.403349 | 16.41022 | 8.47E-50 | 8.88E-48 | 102.2796 |
| ENSG00000113460.11 | BRIX1 | 1.032072 | 3.139293 | 12.39843 | 2.11E-31 | 8.33E-30 | 60.12912 |
| ENSG00000172731.12 | LRRC20 | 1.031947 | 3.100686 | 11.79926 | 6.96E-29 | 2.36E-27 | 54.36864 |
| ENSG00000169302.13 | STK32A | 1.031473 | 1.668292 | 8.547264 | 1.17E-16 | 1.70E-15 | 26.47926 |
| ENSG00000137331.11 | IER3 | 1.02935 | 5.60791 | 6.513031 | 1.63E-10 | 1.38E-09 | 12.57172 |
| ENSG00000139835.12 | GRTP1 | 1.028894 | 2.307777 | 10.94907 | 1.94E-25 | 5.27E-24 | 46.49721 |
| ENSG00000160753.14 | RUSC1 | 1.028167 | 3.357645 | 14.00195 | 1.81E-38 | 1.10E-36 | 76.30687 |
| ENSG00000137168.7 | PPIL1 | 1.027012 | 4.307236 | 12.10541 | 3.67E-30 | 1.33E-28 | 57.29109 |
| ENSG00000117593.9 | DARS2 | 1.026724 | 3.272078 | 12.2447 | 9.49E-31 | 3.57E-29 | 58.63522 |
| ENSG00000167553.13 | TUBA1C | 1.025748 | 4.673047 | 10.85338 | 4.62E-25 | 1.23E-23 | 45.63484 |
| ENSG00000135750.13 | KCNK1 | 1.025308 | 3.267511 | 6.890622 | 1.48E-11 | 1.39E-10 | 14.9169 |
| ENSG00000198732.9 | SMOC1 | 1.024672 | 1.375184 | 4.721329 | 2.96E-06 | 1.54E-05 | 3.047314 |
| ENSG00000070756.12 | PABPC1 | 1.02401 | 8.162562 | 11.13887 | 3.40E-26 | 9.66E-25 | 48.22216 |
| ENSG00000159055.3 | MIS18A | 1.023926 | 2.9283 | 12.32837 | 4.19E-31 | 1.61E-29 | 59.44699 |
| ENSG00000110680.11 | CALCA | 1.022436 | 1.236125 | 2.775895 | 0.005686945 | 0.01677298 | -4.06012 |
| ENSG00000121895.7 | TMEM156 | 1.022429 | 1.445132 | 7.808978 | 2.81E-14 | 3.35E-13 | 21.07878 |
| ENSG00000111012.8 | CYP27B1 | 1.021656 | 1.416835 | 10.12475 | 2.94E-22 | 6.44E-21 | 39.23524 |
| ENSG00000174564.11 | IL20RB | 1.02142 | 1.260126 | 5.520549 | 5.15E-08 | 3.34E-07 | 6.95927 |
| ENSG00000106105.12 | GARS | 1.020299 | 4.91361 | 12.89632 | 1.51E-33 | 6.81E-32 | 65.03941 |
| ENSG00000136240.8 | KDELR2 | 1.019684 | 6.470159 | 13.92034 | 4.25E-38 | 2.51E-36 | 75.45926 |
| ENSG00000188747.7 | NOXA1 | 1.018051 | 2.953173 | 7.537827 | 1.91E-13 | 2.13E-12 | 19.19361 |
| ENSG00000153294.10 | GPR115 | 1.017818 | 1.022528 | 7.478557 | 2.88E-13 | 3.17E-12 | 18.78877 |
| ENSG00000104147.7 | OIP5 | 1.017602 | 1.39554 | 10.09139 | 3.92E-22 | 8.50E-21 | 38.94946 |
| ENSG00000156127.6 | BATF | 1.017124 | 3.577409 | 7.812561 | 2.74E-14 | 3.27E-13 | 21.10406 |
| ENSG00000033100.13 | CHPF2 | 1.016991 | 4.592369 | 14.08693 | 7.44E-39 | 4.59E-37 | 77.19208 |
| ENSG00000256269.5 | HMBS | 1.016431 | 2.862041 | 13.36468 | 1.32E-35 | 6.78E-34 | 69.75397 |
| ENSG00000188372.13 | ZP3 | 1.015586 | 1.530705 | 9.666047 | 1.46E-20 | 2.81E-19 | 35.36475 |
| ENSG00000263639.4 | MSMB | 1.015334 | 3.068336 | 2.531023 | 0.011642472 | 0.031738028 | -4.70212 |
| ENSG00000157593.15 | SLC35B2 | 1.012434 | 5.251043 | 14.48139 | 1.16E-40 | 7.80E-39 | 81.33402 |
| ENSG00000101811.12 | CSTF2 | 1.011406 | 3.382778 | 15.74116 | 1.41E-46 | 1.30E-44 | 94.89559 |
| ENSG00000243678.10 | NME1-NME2 | 1.011108 | 4.537644 | 10.51835 | 9.32E-24 | 2.26E-22 | 42.65501 |
| ENSG00000170577.7 | SIX2 | 1.010879 | 1.24472 | 6.010696 | 3.31E-09 | 2.44E-08 | 9.629208 |
| ENSG00000123213.21 | NLN | 1.010749 | 2.086503 | 15.46594 | 2.87E-45 | 2.49E-43 | 91.89261 |
| ENSG00000278619.3 | MRM1 | 1.009913 | 2.427956 | 13.97198 | 2.48E-38 | 1.49E-36 | 75.99531 |
| ENSG00000146731.9 | CCT6A | 1.009665 | 5.682281 | 11.21609 | 1.67E-26 | 4.85E-25 | 48.92934 |
| ENSG00000163207.6 | IVL | 1.009594 | 1.160059 | 4.762622 | 2.43E-06 | 1.28E-05 | 3.235712 |
| ENSG00000166557.11 | TMED3 | 1.009576 | 2.915206 | 12.6588 | 1.61E-32 | 6.84E-31 | 62.68344 |
| ENSG00000132436.10 | FIGNL1 | 1.008629 | 1.998524 | 11.92412 | 2.11E-29 | 7.34E-28 | 55.55518 |
| ENSG00000124787.12 | RPP40 | 1.008297 | 1.917194 | 13.13307 | 1.39E-34 | 6.68E-33 | 67.41132 |
| ENSG00000109674.3 | NEIL3 | 1.007697 | 1.020514 | 8.842236 | 1.19E-17 | 1.86E-16 | 28.74242 |
| ENSG00000160886.12 | LY6K | 1.007249 | 1.163103 | 5.770198 | 1.30E-08 | 9.01E-08 | 8.29395 |
| ENSG00000206075.12 | SERPINB5 | 1.007231 | 1.130818 | 4.851848 | 1.58E-06 | 8.52E-06 | 3.647966 |
| ENSG00000050344.8 | NFE2L3 | 1.006768 | 3.23155 | 7.93693 | 1.12E-14 | 1.38E-13 | 21.98701 |
| ENSG00000117308.13 | GALE | 1.005721 | 4.227669 | 10.71855 | 1.56E-24 | 3.99E-23 | 44.42814 |
| ENSG00000138028.13 | CGREF1 | 1.005567 | 1.037879 | 7.672315 | 7.42E-14 | 8.53E-13 | 20.12188 |
| ENSG00000169903.6 | TM4SF4 | 1.004943 | 1.317111 | 3.667432 | 0.000268083 | 0.001037727 | -1.2395 |
| ENSG00000204616.9 | TRIM31 | 1.003678 | 1.134567 | 6.33102 | 4.96E-10 | 3.99E-09 | 11.48185 |
| ENSG00000205426.9 | KRT81 | 1.002647 | 1.10483 | 4.382379 | 1.40E-05 | 6.63E-05 | 1.558471 |
| ENSG00000198598.5 | MMP17 | 1.002537 | 1.288206 | 8.446466 | 2.53E-16 | 3.58E-15 | 25.71945 |
| ENSG00000221867.7 | MAGEA3 | 1.001245 | 0.921749 | 4.076086 | 5.23E-05 | 0.000228144 | 0.302353 |
| ENSG00000114270.14 | COL7A1 | 1.000718 | 1.244356 | 6.922093 | 1.21E-11 | 1.14E-10 | 15.11743 |
| ENSG00000139193.3 | CD27 | 1.000703 | 2.613489 | 6.757304 | 3.50E-11 | 3.16E-10 | 14.07602 |
| ENSG00000143452.14 | HORMAD1 | 1.000322 | 1.052813 | 5.876244 | 7.17E-09 | 5.10E-08 | 8.876779 |
| ENSG00000179051.12 | RCC2 | 1.000196 | 5.280972 | 12.5885 | 3.24E-32 | 1.34E-30 | 61.99084 |
| ENSG00000101189.6 | MRGBP | 0.9993 | 3.200688 | 13.71435 | 3.61E-37 | 2.03E-35 | 73.33058 |
| ENSG00000170689.9 | HOXB9 | 0.999203 | 0.939439 | 4.781641 | 2.22E-06 | 1.17E-05 | 3.322996 |
| ENSG00000102890.13 | ELMO3 | 0.998505 | 4.007254 | 9.241314 | 4.88E-19 | 8.51E-18 | 31.89618 |
| ENSG00000118193.10 | KIF14 | 0.997906 | 1.032349 | 11.23216 | 1.44E-26 | 4.19E-25 | 49.07692 |
| ENSG00000132698.12 | RAB25 | 0.997891 | 6.076951 | 8.512593 | 1.53E-16 | 2.19E-15 | 26.21713 |
| ENSG00000101255.9 | TRIB3 | 0.997405 | 3.230489 | 8.344325 | 5.49E-16 | 7.55E-15 | 24.95665 |
| ENSG00000156510.12 | HKDC1 | 0.997241 | 2.229703 | 6.494434 | 1.82E-10 | 1.53E-09 | 12.45914 |
| ENSG00000136270.12 | TBRG4 | 0.997198 | 3.481824 | 13.72703 | 3.17E-37 | 1.79E-35 | 73.46118 |
| ENSG00000128218.7 | VPREB3 | 0.996964 | 1.544631 | 6.994633 | 7.53E-12 | 7.23E-11 | 15.58257 |
| ENSG00000112559.12 | MDFI | 0.996188 | 2.959605 | 6.599266 | 9.50E-11 | 8.20E-10 | 13.09736 |
| ENSG00000184661.12 | CDCA2 | 0.994899 | 1.141458 | 10.92508 | 2.41E-25 | 6.53E-24 | 46.28049 |
| ENSG00000173156.5 | RHOD | 0.994839 | 4.091771 | 8.00752 | 6.68E-15 | 8.39E-14 | 22.49312 |
| ENSG00000276180.1 | HIST1H4I | 0.994201 | 3.142049 | 8.3534 | 5.12E-16 | 7.06E-15 | 25.02413 |
| ENSG00000113504.18 | SLC12A7 | 0.992863 | 4.610092 | 8.459337 | 2.30E-16 | 3.25E-15 | 25.81608 |
| ENSG00000049768.13 | FOXP3 | 0.991862 | 1.516463 | 11.00346 | 1.18E-25 | 3.25E-24 | 46.98953 |
| ENSG00000145194.16 | ECE2 | 0.991092 | 1.411469 | 12.62827 | 2.18E-32 | 9.18E-31 | 62.38241 |
| ENSG00000180900.15 | SCRIB | 0.9908 | 4.154885 | 9.140364 | 1.10E-18 | 1.87E-17 | 31.08861 |
| ENSG00000111641.9 | NOP2 | 0.99035 | 3.297184 | 12.24058 | 9.88E-31 | 3.71E-29 | 58.59537 |
| ENSG00000004478.7 | FKBP4 | 0.989545 | 4.347697 | 9.376452 | 1.62E-19 | 2.91E-18 | 32.98746 |
| ENSG00000099364.15 | FBXL19 | 0.988241 | 2.692581 | 11.6339 | 3.35E-28 | 1.09E-26 | 52.80905 |
| ENSG00000143153.11 | ATP1B1 | 0.987726 | 7.309794 | 6.706652 | 4.83E-11 | 4.30E-10 | 13.7602 |
| ENSG00000109065.10 | NAT9 | 0.986939 | 3.283575 | 14.343 | 5.02E-40 | 3.23E-38 | 79.87486 |
| ENSG00000066855.14 | MTFR1 | 0.986843 | 3.031892 | 10.82623 | 5.90E-25 | 1.56E-23 | 45.39108 |
| ENSG00000157227.11 | MMP14 | 0.986214 | 5.871026 | 7.237995 | 1.49E-12 | 1.53E-11 | 17.17269 |
| ENSG00000157224.14 | CLDN12 | 0.986071 | 3.399664 | 11.5466 | 7.63E-28 | 2.41E-26 | 51.99115 |
| ENSG00000112977.14 | DAP | 0.98599 | 6.141502 | 11.71181 | 1.60E-28 | 5.31E-27 | 53.54221 |
| ENSG00000188643.9 | S100A16 | 0.984847 | 6.513932 | 6.288739 | 6.40E-10 | 5.09E-09 | 11.23252 |
| ENSG00000167642.11 | SPINT2 | 0.984582 | 6.603957 | 9.942763 | 1.41E-21 | 2.93E-20 | 37.68454 |
| ENSG00000167964.11 | RAB26 | 0.983245 | 1.050804 | 9.520128 | 4.95E-20 | 9.21E-19 | 34.16036 |
| ENSG00000173272.12 | MZT2A | 0.982934 | 2.746221 | 12.53127 | 5.70E-32 | 2.33E-30 | 61.4286 |
| ENSG00000178814.14 | OPLAH | 0.982782 | 2.709416 | 8.566539 | 1.01E-16 | 1.47E-15 | 26.62535 |
| ENSG00000178896.6 | EXOSC4 | 0.982033 | 3.93171 | 10.58755 | 5.04E-24 | 1.25E-22 | 43.26539 |
| ENSG00000131050.9 | BPIFA2 | 0.981577 | 1.022962 | 5.095583 | 4.74E-07 | 2.73E-06 | 4.809868 |
| ENSG00000204220.8 | PFDN6 | 0.980931 | 3.674501 | 13.41148 | 8.15E-36 | 4.24E-34 | 70.2299 |
| ENSG00000144591.16 | GMPPA | 0.980444 | 3.417964 | 17.37418 | 1.62E-54 | 2.06E-52 | 113.1038 |
| ENSG00000196584.2 | XRCC2 | 0.980414 | 1.08011 | 11.65577 | 2.72E-28 | 8.90E-27 | 53.01451 |
| ENSG00000160072.18 | ATAD3B | 0.980198 | 2.036844 | 10.34462 | 4.32E-23 | 9.97E-22 | 41.1346 |
| ENSG00000162522.9 | KIAA1522 | 0.979658 | 5.10193 | 10.73043 | 1.40E-24 | 3.59E-23 | 44.53411 |
| ENSG00000147697.7 | GSDMC | 0.977911 | 1.364339 | 6.769282 | 3.24E-11 | 2.93E-10 | 14.151 |
| ENSG00000166105.14 | GLB1L3 | 0.977907 | 0.969627 | 5.552571 | 4.33E-08 | 2.83E-07 | 7.127511 |
| ENSG00000182871.13 | COL18A1 | 0.977856 | 4.329746 | 7.15598 | 2.58E-12 | 2.59E-11 | 16.63174 |
| ENSG00000167799.8 | NUDT8 | 0.977758 | 2.561222 | 9.900583 | 2.02E-21 | 4.16E-20 | 37.32793 |
| ENSG00000152049.6 | KCNE4 | 0.977368 | 1.85887 | 5.612109 | 3.13E-08 | 2.08E-07 | 7.442643 |
| ENSG00000165704.13 | HPRT1 | 0.977303 | 4.404416 | 10.56345 | 6.24E-24 | 1.53E-22 | 43.0525 |
| ENSG00000106305.8 | AIMP2 | 0.977195 | 3.591498 | 13.09778 | 1.98E-34 | 9.45E-33 | 67.05634 |
| ENSG00000176444.17 | CLK2 | 0.976586 | 3.736048 | 11.91706 | 2.26E-29 | 7.84E-28 | 55.48788 |
| ENSG00000003147.16 | ICA1 | 0.975867 | 3.289762 | 10.79371 | 7.92E-25 | 2.07E-23 | 45.09955 |
| ENSG00000166670.8 | MMP10 | 0.975846 | 1.204071 | 5.25512 | 2.10E-07 | 1.27E-06 | 5.598496 |
| ENSG00000131788.14 | PIAS3 | 0.975519 | 3.631109 | 11.85903 | 3.93E-29 | 1.35E-27 | 54.93569 |
| ENSG00000183971.5 | NPW | 0.97549 | 1.001303 | 5.465107 | 6.94E-08 | 4.43E-07 | 6.670044 |
| ENSG00000183486.11 | MX2 | 0.975087 | 2.266448 | 8.760775 | 2.25E-17 | 3.44E-16 | 28.11156 |
| ENSG00000113569.14 | NUP155 | 0.974984 | 2.628748 | 11.15826 | 2.85E-26 | 8.11E-25 | 48.39939 |
| ENSG00000182325.9 | FBXL6 | 0.97493 | 2.879412 | 10.90021 | 3.02E-25 | 8.15E-24 | 46.05626 |
| ENSG00000091844.6 | RGS17 | 0.974747 | 1.030805 | 9.668518 | 1.43E-20 | 2.76E-19 | 35.38526 |
| ENSG00000164171.9 | ITGA2 | 0.974221 | 3.297659 | 5.467058 | 6.87E-08 | 4.39E-07 | 6.680176 |
| ENSG00000179222.16 | MAGED1 | 0.973154 | 5.363193 | 11.19957 | 1.94E-26 | 5.63E-25 | 48.77781 |
| ENSG00000249915.6 | PDCD6 | 0.973008 | 3.710007 | 12.55501 | 4.51E-32 | 1.86E-30 | 61.66168 |
| ENSG00000184156.14 | KCNQ3 | 0.972625 | 1.59807 | 8.088586 | 3.69E-15 | 4.74E-14 | 23.07874 |
| ENSG00000169972.10 | PUSL1 | 0.972432 | 2.692217 | 12.49407 | 8.23E-32 | 3.32E-30 | 61.06389 |
| ENSG00000067225.16 | PKM | 0.971536 | 7.160308 | 12.60685 | 2.70E-32 | 1.13E-30 | 62.17139 |
| ENSG00000182379.9 | NXPH4 | 0.971496 | 1.057396 | 5.901942 | 6.19E-09 | 4.44E-08 | 9.019428 |
| ENSG00000158850.13 | B4GALT3 | 0.97078 | 4.146135 | 14.63431 | 2.28E-41 | 1.61E-39 | 82.95393 |
| ENSG00000196937.9 | FAM3C | 0.97057 | 4.100042 | 8.582002 | 8.98E-17 | 1.31E-15 | 26.74273 |
| ENSG00000130701.3 | RBBP8NL | 0.970544 | 1.36745 | 11.66728 | 2.44E-28 | 8.02E-27 | 53.12281 |
| ENSG00000171224.8 | C10orf35 | 0.97046 | 3.205986 | 11.3831 | 3.53E-27 | 1.07E-25 | 50.46948 |
| ENSG00000048462.9 | TNFRSF17 | 0.970455 | 1.696335 | 6.458105 | 2.28E-10 | 1.90E-09 | 12.24001 |
| ENSG00000078269.12 | SYNJ2 | 0.970333 | 1.851317 | 12.90044 | 1.45E-33 | 6.55E-32 | 65.08046 |
| ENSG00000138074.13 | SLC5A6 | 0.970119 | 2.796541 | 13.85663 | 8.25E-38 | 4.80E-36 | 74.79921 |
| ENSG00000143374.13 | TARS2 | 0.969405 | 3.377568 | 14.82961 | 2.82E-42 | 2.09E-40 | 85.03388 |
| ENSG00000281855.1 | AC105009.1 | 0.969396 | 1.114095 | 7.776132 | 3.55E-14 | 4.20E-13 | 20.84755 |
| ENSG00000185298.11 | CCDC137 | 0.969097 | 3.549713 | 12.77528 | 5.06E-33 | 2.23E-31 | 63.83579 |
| ENSG00000014914.18 | MTMR11 | 0.969045 | 2.500594 | 8.113377 | 3.07E-15 | 3.98E-14 | 23.25876 |
| ENSG00000146858.7 | ZC3HAV1L | 0.968464 | 1.846091 | 11.86505 | 3.71E-29 | 1.28E-27 | 54.99288 |
| ENSG00000088826.16 | SMOX | 0.968417 | 2.239436 | 8.386442 | 3.99E-16 | 5.55E-15 | 25.27031 |
| ENSG00000144452.13 | ABCA12 | 0.968165 | 0.916676 | 8.631201 | 6.15E-17 | 9.08E-16 | 27.11728 |
| ENSG00000145545.10 | SRD5A1 | 0.96709 | 2.041638 | 10.32973 | 4.93E-23 | 1.13E-21 | 41.00513 |
| ENSG00000171421.11 | MRPL36 | 0.966802 | 3.7692 | 10.99279 | 1.30E-25 | 3.57E-24 | 46.89285 |
| ENSG00000039068.17 | CDH1 | 0.966558 | 6.015877 | 8.644507 | 5.55E-17 | 8.23E-16 | 27.21886 |
| ENSG00000140263.12 | SORD | 0.966178 | 2.161273 | 11.24751 | 1.25E-26 | 3.65E-25 | 49.21803 |
| ENSG00000170775.2 | GPR37 | 0.965883 | 1.291155 | 6.806337 | 2.56E-11 | 2.34E-10 | 14.38367 |
| ENSG00000204536.12 | CCHCR1 | 0.965862 | 2.658778 | 11.48862 | 1.32E-27 | 4.09E-26 | 51.44999 |
| ENSG00000143436.9 | MRPL9 | 0.965787 | 4.785618 | 14.35981 | 4.20E-40 | 2.72E-38 | 80.05173 |
| ENSG00000171345.12 | KRT19 | 0.96574 | 7.902323 | 5.853201 | 8.17E-09 | 5.78E-08 | 8.749334 |
| ENSG00000117139.15 | KDM5B | 0.965223 | 3.324724 | 13.05932 | 2.93E-34 | 1.38E-32 | 66.66997 |
| ENSG00000084774.12 | CAD | 0.965197 | 3.062475 | 10.73986 | 1.29E-24 | 3.31E-23 | 44.61823 |
| ENSG00000198691.10 | ABCA4 | 0.964937 | 1.131769 | 5.617566 | 3.04E-08 | 2.02E-07 | 7.471679 |
| ENSG00000135549.13 | PKIB | 0.964815 | 1.868025 | 6.38405 | 3.59E-10 | 2.93E-09 | 11.79663 |
| ENSG00000044574.7 | HSPA5 | 0.964796 | 7.840126 | 14.4613 | 1.43E-40 | 9.51E-39 | 81.12184 |
| ENSG00000168209.4 | DDIT4 | 0.963956 | 5.490054 | 5.869905 | 7.43E-09 | 5.28E-08 | 8.841673 |
| ENSG00000101444.11 | AHCY | 0.963932 | 5.339249 | 11.4116 | 2.71E-27 | 8.27E-26 | 50.73382 |
| ENSG00000138018.16 | EPT1 | 0.963168 | 2.752446 | 11.147 | 3.16E-26 | 8.99E-25 | 48.2964 |
| ENSG00000091127.12 | PUS7 | 0.962747 | 2.488347 | 12.35241 | 3.31E-31 | 1.28E-29 | 59.68076 |
| ENSG00000176171.10 | BNIP3 | 0.962185 | 3.913194 | 8.642423 | 5.64E-17 | 8.35E-16 | 27.20294 |
| ENSG00000037474.13 | NSUN2 | 0.9621 | 4.165619 | 11.76523 | 9.63E-29 | 3.24E-27 | 54.04659 |
| ENSG00000136982.5 | DSCC1 | 0.961939 | 1.728418 | 9.356015 | 1.91E-19 | 3.43E-18 | 32.82168 |
| ENSG00000171067.9 | C11orf24 | 0.960966 | 3.900188 | 13.14976 | 1.17E-34 | 5.66E-33 | 67.57938 |
| ENSG00000132840.8 | BHMT2 | 0.959362 | 1.61356 | 4.465829 | 9.63E-06 | 4.67E-05 | 1.915454 |
| ENSG00000166598.11 | HSP90B1 | 0.959178 | 7.7118 | 11.41306 | 2.67E-27 | 8.16E-26 | 50.7473 |
| ENSG00000163808.15 | KIF15 | 0.958212 | 1.162677 | 10.98477 | 1.40E-25 | 3.83E-24 | 46.8202 |
| ENSG00000159377.9 | PSMB4 | 0.958206 | 6.720216 | 13.26942 | 3.48E-35 | 1.73E-33 | 68.78783 |
| ENSG00000263528.6 | IKBKE | 0.9578 | 2.546773 | 12.74673 | 6.73E-33 | 2.93E-31 | 63.55277 |
| ENSG00000184164.13 | CRELD2 | 0.956088 | 3.43144 | 11.18933 | 2.14E-26 | 6.17E-25 | 48.68388 |
| ENSG00000188505.4 | NCCRP1 | 0.956073 | 1.213538 | 5.107779 | 4.46E-07 | 2.58E-06 | 4.869373 |
| ENSG00000171792.9 | RHNO1 | 0.955605 | 3.732627 | 14.68946 | 1.26E-41 | 9.07E-40 | 83.54009 |
| ENSG00000185467.7 | KPNA7 | 0.955259 | 1.384602 | 8.378168 | 4.25E-16 | 5.89E-15 | 25.20859 |
| ENSG00000143799.11 | PARP1 | 0.955238 | 4.694769 | 12.57188 | 3.82E-32 | 1.58E-30 | 61.82743 |
| ENSG00000105655.17 | ISYNA1 | 0.954384 | 3.630329 | 7.703159 | 5.96E-14 | 6.92E-13 | 20.33665 |
| ENSG00000163348.3 | PYGO2 | 0.954073 | 4.454574 | 14.91869 | 1.08E-42 | 8.27E-41 | 85.9867 |
| ENSG00000147535.15 | PPAPDC1B | 0.952728 | 3.668859 | 10.12726 | 2.88E-22 | 6.31E-21 | 39.25672 |
| ENSG00000107815.6 | C10orf2 | 0.952703 | 2.250597 | 13.00743 | 4.94E-34 | 2.30E-32 | 66.14967 |
| ENSG00000013573.15 | DDX11 | 0.952603 | 1.867508 | 10.29547 | 6.65E-23 | 1.52E-21 | 40.7076 |
| ENSG00000204922.4 | UQCC3 | 0.952486 | 3.195576 | 10.84799 | 4.85E-25 | 1.29E-23 | 45.58637 |
| ENSG00000153012.10 | LGI2 | 0.950565 | 1.248229 | 7.606577 | 1.18E-13 | 1.34E-12 | 19.66647 |
| ENSG00000198554.10 | WDHD1 | 0.9505 | 1.636912 | 10.53057 | 8.36E-24 | 2.04E-22 | 42.76267 |
| ENSG00000153044.8 | CENPH | 0.950035 | 2.090529 | 10.86425 | 4.18E-25 | 1.12E-23 | 45.73254 |
| ENSG00000138346.13 | DNA2 | 0.949408 | 1.318226 | 11.73675 | 1.26E-28 | 4.21E-27 | 53.77752 |
| ENSG00000184349.11 | EFNA5 | 0.949319 | 2.561352 | 7.516326 | 2.21E-13 | 2.46E-12 | 19.04645 |
| ENSG00000141738.12 | GRB7 | 0.948896 | 3.604846 | 8.346485 | 5.40E-16 | 7.43E-15 | 24.97271 |
| ENSG00000127129.8 | EDN2 | 0.948326 | 1.598092 | 5.311177 | 1.57E-07 | 9.60E-07 | 5.88084 |
| ENSG00000221829.8 | FANCG | 0.948244 | 2.445034 | 12.16137 | 2.13E-30 | 7.88E-29 | 57.83001 |
| ENSG00000182580.2 | EPHB3 | 0.948207 | 2.660016 | 7.69009 | 6.54E-14 | 7.56E-13 | 20.24557 |
| ENSG00000183765.19 | CHEK2 | 0.947849 | 2.005932 | 10.86107 | 4.31E-25 | 1.15E-23 | 45.70397 |
| ENSG00000143401.13 | ANP32E | 0.947787 | 4.83305 | 7.964571 | 9.13E-15 | 1.14E-13 | 22.18476 |
| ENSG00000149380.10 | P4HA3 | 0.947783 | 1.282074 | 10.07342 | 4.58E-22 | 9.87E-21 | 38.79582 |
| ENSG00000143256.4 | PFDN2 | 0.947221 | 6.146805 | 11.45581 | 1.79E-27 | 5.53E-26 | 51.14453 |
| ENSG00000164294.12 | GPX8 | 0.946908 | 2.819353 | 7.935963 | 1.12E-14 | 1.39E-13 | 21.9801 |
| ENSG00000139880.18 | CDH24 | 0.946682 | 1.972597 | 8.68177 | 4.15E-17 | 6.24E-16 | 27.50398 |
| ENSG00000134398.11 | ERN2 | 0.946573 | 1.442607 | 4.455556 | 1.01E-05 | 4.88E-05 | 1.871169 |
| ENSG00000174607.9 | UGT8 | 0.945332 | 1.59377 | 6.345235 | 4.55E-10 | 3.68E-09 | 11.56601 |
| ENSG00000106483.10 | SFRP4 | 0.943986 | 3.597916 | 4.695453 | 3.34E-06 | 1.72E-05 | 2.930031 |
| ENSG00000008323.14 | PLEKHG6 | 0.943183 | 1.82198 | 8.54122 | 1.23E-16 | 1.77E-15 | 26.43351 |
| ENSG00000100442.9 | FKBP3 | 0.943 | 4.391894 | 11.63609 | 3.28E-28 | 1.07E-26 | 52.82962 |
| ENSG00000106263.16 | EIF3B | 0.942129 | 4.668649 | 12.27142 | 7.31E-31 | 2.78E-29 | 58.89408 |
| ENSG00000163584.16 | RPL22L1 | 0.94159 | 3.464905 | 8.266112 | 9.87E-16 | 1.33E-14 | 24.37745 |
| ENSG00000257727.4 | CNPY2 | 0.940871 | 3.50505 | 13.02453 | 4.16E-34 | 1.95E-32 | 66.32099 |
| ENSG00000168234.11 | TTC39C | 0.940689 | 2.187485 | 9.145281 | 1.06E-18 | 1.80E-17 | 31.1278 |
| ENSG00000137124.6 | ALDH1B1 | 0.940632 | 3.646024 | 9.661602 | 1.52E-20 | 2.91E-19 | 35.32787 |
| ENSG00000120509.9 | PDZD11 | 0.940181 | 4.788425 | 12.98758 | 6.03E-34 | 2.78E-32 | 65.95095 |
| ENSG00000166224.15 | SGPL1 | 0.939679 | 3.897533 | 13.23745 | 4.81E-35 | 2.36E-33 | 68.46436 |
| ENSG00000112473.15 | SLC39A7 | 0.939616 | 6.27784 | 12.17718 | 1.83E-30 | 6.78E-29 | 57.98253 |
| ENSG00000110777.10 | POU2AF1 | 0.939184 | 1.748006 | 6.424852 | 2.80E-10 | 2.31E-09 | 12.04038 |
| ENSG00000167755.12 | KLK6 | 0.937315 | 0.900493 | 4.453365 | 1.02E-05 | 4.92E-05 | 1.861737 |
| ENSG00000196793.12 | ZNF239 | 0.936996 | 1.644546 | 11.63944 | 3.18E-28 | 1.03E-26 | 52.86109 |
| ENSG00000132182.10 | NUP210 | 0.936609 | 3.471771 | 8.238419 | 1.21E-15 | 1.62E-14 | 24.1734 |
| ENSG00000134291.10 | TMEM106C | 0.936119 | 4.363269 | 9.07196 | 1.91E-18 | 3.19E-17 | 30.54514 |
| ENSG00000176619.9 | LMNB2 | 0.935864 | 3.736164 | 9.299501 | 3.04E-19 | 5.37E-18 | 32.36464 |
| ENSG00000139579.11 | NABP2 | 0.9351 | 3.989375 | 13.90647 | 4.91E-38 | 2.89E-36 | 75.31541 |
| ENSG00000142731.9 | PLK4 | 0.933957 | 1.396779 | 10.60737 | 4.22E-24 | 1.05E-22 | 43.44074 |
| ENSG00000123352.16 | SPATS2 | 0.93392 | 2.470296 | 13.37958 | 1.13E-35 | 5.84E-34 | 69.90534 |
| ENSG00000128595.15 | CALU | 0.933603 | 5.781886 | 9.57838 | 3.05E-20 | 5.76E-19 | 34.63958 |
| ENSG00000123975.4 | CKS2 | 0.933562 | 5.170947 | 6.858752 | 1.82E-11 | 1.69E-10 | 14.71463 |
| ENSG00000071564.13 | TCF3 | 0.933278 | 3.70994 | 11.45069 | 1.88E-27 | 5.79E-26 | 51.0969 |
| ENSG00000100473.14 | COCH | 0.932327 | 1.02622 | 6.794544 | 2.76E-11 | 2.51E-10 | 14.3095 |
| ENSG00000145494.10 | NDUFS6 | 0.931926 | 5.287759 | 10.35181 | 4.06E-23 | 9.39E-22 | 41.19717 |
| ENSG00000163463.10 | KRTCAP2 | 0.931619 | 3.008463 | 12.31052 | 4.99E-31 | 1.91E-29 | 59.27349 |
| ENSG00000144867.10 | SRPRB | 0.931494 | 4.256542 | 12.92744 | 1.10E-33 | 5.03E-32 | 65.34981 |
| ENSG00000108465.13 | CDK5RAP3 | 0.931486 | 4.128402 | 9.379873 | 1.57E-19 | 2.83E-18 | 33.01524 |
| ENSG00000115507.8 | OTX1 | 0.931417 | 0.998497 | 11.13549 | 3.51E-26 | 9.96E-25 | 48.19127 |
| ENSG00000149503.11 | INCENP | 0.930716 | 2.509865 | 9.908484 | 1.88E-21 | 3.90E-20 | 37.39466 |
| ENSG00000197409.7 | HIST1H3D | 0.930334 | 1.118174 | 6.65121 | 6.86E-11 | 6.02E-10 | 13.41684 |
| ENSG00000102100.13 | SLC35A2 | 0.930282 | 4.189502 | 11.52221 | 9.60E-28 | 3.02E-26 | 51.76329 |
| ENSG00000167105.6 | TMEM92 | 0.9302 | 2.665963 | 7.516003 | 2.22E-13 | 2.46E-12 | 19.04425 |
| ENSG00000132676.14 | DAP3 | 0.929963 | 4.659295 | 15.37627 | 7.62E-45 | 6.45E-43 | 90.91886 |
| ENSG00000186474.14 | KLK12 | 0.929918 | 1.171006 | 3.223445 | 0.001339422 | 0.004538757 | -2.73824 |
| ENSG00000253729.6 | PRKDC | 0.928976 | 3.758219 | 8.945957 | 5.22E-18 | 8.47E-17 | 29.55205 |
| ENSG00000149679.10 | CABLES2 | 0.928715 | 2.296143 | 10.83416 | 5.50E-25 | 1.45E-23 | 45.46221 |
| ENSG00000172031.6 | EPHX4 | 0.928679 | 1.160438 | 8.720437 | 3.08E-17 | 4.67E-16 | 27.80081 |
| ENSG00000132432.12 | SEC61G | 0.928331 | 5.058888 | 10.65478 | 2.76E-24 | 6.93E-23 | 43.86094 |
| ENSG00000108829.9 | LRRC59 | 0.928059 | 5.366826 | 13.59237 | 1.27E-36 | 6.98E-35 | 72.07752 |
| ENSG00000141570.9 | CBX8 | 0.927501 | 1.93533 | 14.47605 | 1.23E-40 | 8.23E-39 | 81.27765 |
| ENSG00000163472.17 | TMEM79 | 0.927016 | 2.06867 | 12.49674 | 8.02E-32 | 3.24E-30 | 61.09003 |
| ENSG00000160113.5 | NR2F6 | 0.926771 | 4.404149 | 10.52981 | 8.42E-24 | 2.05E-22 | 42.75598 |
| ENSG00000151498.10 | ACAD8 | 0.92647 | 2.880106 | 8.139111 | 2.54E-15 | 3.31E-14 | 23.44609 |
| ENSG00000164941.12 | INTS8 | 0.926409 | 2.650112 | 13.85598 | 8.30E-38 | 4.82E-36 | 74.79251 |
| ENSG00000034053.13 | APBA2 | 0.926169 | 1.564982 | 7.11693 | 3.35E-12 | 3.32E-11 | 16.37598 |
| ENSG00000109881.15 | CCDC34 | 0.925938 | 1.949006 | 9.926446 | 1.62E-21 | 3.36E-20 | 37.54646 |
| ENSG00000132481.5 | TRIM47 | 0.925309 | 3.833414 | 8.693003 | 3.81E-17 | 5.72E-16 | 27.59011 |
| ENSG00000078898.6 | BPIFB2 | 0.924739 | 1.170634 | 3.620798 | 0.000320027 | 0.001218445 | -1.4056 |
| ENSG00000053372.4 | MRTO4 | 0.924696 | 4.018154 | 13.44685 | 5.68E-36 | 2.97E-34 | 70.59018 |
| ENSG00000123684.11 | LPGAT1 | 0.924555 | 3.551195 | 10.97005 | 1.60E-25 | 4.37E-24 | 46.68694 |
| ENSG00000196465.9 | MYL6B | 0.923352 | 3.333101 | 11.18209 | 2.28E-26 | 6.58E-25 | 48.61756 |
| ENSG00000166823.5 | MESP1 | 0.922273 | 1.251368 | 8.859948 | 1.03E-17 | 1.63E-16 | 28.88017 |
| ENSG00000137474.18 | MYO7A | 0.921073 | 1.322576 | 12.19151 | 1.59E-30 | 5.93E-29 | 58.12089 |
| ENSG00000171302.15 | CANT1 | 0.920212 | 4.676203 | 11.86764 | 3.62E-29 | 1.25E-27 | 55.01749 |
| ENSG00000170571.10 | EMB | 0.919719 | 3.923114 | 6.311319 | 5.59E-10 | 4.46E-09 | 11.36549 |
| ENSG00000137842.5 | TMEM62 | 0.919231 | 3.298769 | 11.90797 | 2.46E-29 | 8.53E-28 | 55.40127 |
| ENSG00000179091.4 | CYC1 | 0.919144 | 5.669291 | 9.230524 | 5.32E-19 | 9.26E-18 | 31.80955 |
| ENSG00000004864.12 | SLC25A13 | 0.918397 | 3.310784 | 11.69846 | 1.82E-28 | 6.00E-27 | 53.41641 |
| ENSG00000130513.6 | GDF15 | 0.918078 | 4.60109 | 4.246911 | 2.53E-05 | 0.000115501 | 0.992384 |
| ENSG00000137310.10 | TCF19 | 0.917441 | 2.823283 | 9.175479 | 8.32E-19 | 1.43E-17 | 31.36878 |
| ENSG00000247077.5 | PGAM5 | 0.916849 | 3.741909 | 11.23515 | 1.40E-26 | 4.08E-25 | 49.10443 |
| ENSG00000152377.11 | SPOCK1 | 0.916596 | 1.165406 | 6.064591 | 2.42E-09 | 1.81E-08 | 9.935024 |
| ENSG00000143537.12 | ADAM15 | 0.916369 | 4.993244 | 9.598699 | 2.57E-20 | 4.88E-19 | 34.80724 |
| ENSG00000140832.8 | MARVELD3 | 0.914762 | 1.875342 | 12.37903 | 2.55E-31 | 9.97E-30 | 59.94004 |
| ENSG00000221978.10 | CCNL2 | 0.91467 | 4.002383 | 7.24111 | 1.46E-12 | 1.50E-11 | 17.19334 |
| ENSG00000006282.18 | SPATA20 | 0.912515 | 3.71511 | 9.889718 | 2.21E-21 | 4.55E-20 | 37.23626 |
| ENSG00000198736.10 | MSRB1 | 0.911805 | 4.596958 | 7.904232 | 1.41E-14 | 1.73E-13 | 21.75379 |
| ENSG00000070182.16 | SPTB | 0.911795 | 1.099848 | 6.153157 | 1.44E-09 | 1.10E-08 | 10.44277 |
| ENSG00000204060.5 | FOXO6 | 0.911459 | 1.294832 | 7.593856 | 1.29E-13 | 1.46E-12 | 19.57871 |
| ENSG00000103485.16 | QPRT | 0.910774 | 3.251265 | 4.637483 | 4.38E-06 | 2.23E-05 | 2.669445 |
| ENSG00000159352.14 | PSMD4 | 0.909901 | 5.716657 | 13.26466 | 3.65E-35 | 1.82E-33 | 68.73969 |
| ENSG00000137968.15 | SLC44A5 | 0.909502 | 1.2267 | 6.732956 | 4.09E-11 | 3.67E-10 | 13.92396 |
| ENSG00000123136.13 | DDX39A | 0.909167 | 3.885196 | 11.34174 | 5.20E-27 | 1.56E-25 | 50.08674 |
| ENSG00000146733.12 | PSPH | 0.909056 | 2.726633 | 8.311578 | 7.02E-16 | 9.57E-15 | 24.71362 |
| ENSG00000138794.8 | CASP6 | 0.908379 | 3.722261 | 12.67454 | 1.38E-32 | 5.86E-31 | 62.83884 |
| ENSG00000167771.5 | RCOR2 | 0.908226 | 1.14969 | 7.336791 | 7.62E-13 | 8.08E-12 | 17.83112 |
| ENSG00000182240.14 | BACE2 | 0.907672 | 3.12749 | 5.919369 | 5.60E-09 | 4.04E-08 | 9.116477 |
| ENSG00000167861.14 | HID1 | 0.907085 | 3.696491 | 8.09888 | 3.42E-15 | 4.41E-14 | 23.15343 |
| ENSG00000074071.12 | MRPS34 | 0.906165 | 5.749445 | 12.64779 | 1.80E-32 | 7.59E-31 | 62.57481 |
| ENSG00000154920.13 | EME1 | 0.906118 | 1.065945 | 11.16765 | 2.61E-26 | 7.46E-25 | 48.48535 |
| ENSG00000116771.5 | AGMAT | 0.905774 | 1.101051 | 10.95539 | 1.83E-25 | 4.98E-24 | 46.55429 |
| ENSG00000181924.6 | COA4 | 0.905622 | 4.385368 | 12.68371 | 1.26E-32 | 5.36E-31 | 62.92935 |
| ENSG00000136628.16 | EPRS | 0.905384 | 4.941386 | 12.61937 | 2.39E-32 | 9.99E-31 | 62.29474 |
| ENSG00000128626.10 | MRPS12 | 0.905378 | 3.876382 | 10.17217 | 1.95E-22 | 4.32E-21 | 39.64247 |
| ENSG00000041982.13 | TNC | 0.905326 | 4.224204 | 3.986998 | 7.57E-05 | 0.000321731 | -0.04691 |
| ENSG00000167700.7 | MFSD3 | 0.905276 | 3.380931 | 8.141054 | 2.50E-15 | 3.26E-14 | 23.46026 |
| ENSG00000008300.13 | CELSR3 | 0.905203 | 0.979953 | 9.565897 | 3.38E-20 | 6.36E-19 | 34.53671 |
| ENSG00000171877.18 | FRMD5 | 0.904032 | 0.94289 | 10.98231 | 1.43E-25 | 3.92E-24 | 46.7979 |
| ENSG00000165724.5 | ZMYND19 | 0.902493 | 3.071172 | 13.21881 | 5.82E-35 | 2.85E-33 | 68.27607 |
| ENSG00000151388.9 | ADAMTS12 | 0.901818 | 1.635356 | 7.821859 | 2.56E-14 | 3.07E-13 | 21.16968 |
| ENSG00000139263.10 | LRIG3 | 0.901808 | 3.105946 | 5.990659 | 3.72E-09 | 2.73E-08 | 9.516123 |
| ENSG00000173852.12 | DPY19L1 | 0.901785 | 3.976453 | 7.72802 | 5.00E-14 | 5.83E-13 | 20.51027 |
| ENSG00000130305.15 | NSUN5 | 0.901778 | 3.264539 | 12.65531 | 1.67E-32 | 7.06E-31 | 62.64906 |
| ENSG00000179041.3 | RRS1 | 0.901769 | 3.895745 | 9.622657 | 2.11E-20 | 4.00E-19 | 35.00524 |
| ENSG00000176490.4 | DIRAS1 | 0.900697 | 1.142563 | 7.056413 | 5.01E-12 | 4.89E-11 | 15.98193 |
| ENSG00000198856.11 | OSTC | 0.900527 | 5.848059 | 11.8867 | 3.02E-29 | 1.04E-27 | 55.19873 |
| ENSG00000115902.9 | SLC1A4 | 0.900071 | 2.804659 | 9.565326 | 3.40E-20 | 6.39E-19 | 34.532 |
| ENSG00000221955.9 | SLC12A8 | 0.899593 | 2.247642 | 9.47862 | 6.97E-20 | 1.29E-18 | 33.82018 |
| ENSG00000138430.14 | OLA1 | 0.899497 | 3.789508 | 13.11113 | 1.73E-34 | 8.30E-33 | 67.19055 |
| ENSG00000171208.8 | NETO2 | 0.899137 | 1.973178 | 8.545707 | 1.19E-16 | 1.72E-15 | 26.46747 |
| ENSG00000157214.12 | STEAP2 | 0.89828 | 2.046783 | 6.770301 | 3.22E-11 | 2.92E-10 | 14.15739 |
| ENSG00000175505.10 | CLCF1 | 0.897623 | 3.045401 | 6.547283 | 1.31E-10 | 1.12E-09 | 12.77979 |
| ENSG00000196747.4 | HIST1H2AI | 0.897606 | 0.918938 | 6.814434 | 2.43E-11 | 2.22E-10 | 14.43466 |
| ENSG00000187961.12 | KLHL17 | 0.896921 | 1.801742 | 10.00366 | 8.35E-22 | 1.77E-20 | 38.20122 |
| ENSG00000167004.11 | PDIA3 | 0.896441 | 7.16822 | 9.914673 | 1.79E-21 | 3.71E-20 | 37.44693 |
| ENSG00000138316.9 | ADAMTS14 | 0.896303 | 1.085937 | 9.818981 | 4.03E-21 | 8.15E-20 | 36.6411 |
| ENSG00000227057.6 | WDR46 | 0.896214 | 4.159341 | 15.71216 | 1.93E-46 | 1.77E-44 | 94.5782 |
| ENSG00000181192.10 | DHTKD1 | 0.896046 | 3.238612 | 11.11149 | 4.38E-26 | 1.24E-24 | 47.97207 |
| ENSG00000137343.16 | ATAT1 | 0.895615 | 2.111481 | 10.67736 | 2.26E-24 | 5.69E-23 | 44.06151 |
| ENSG00000198807.11 | PAX9 | 0.894962 | 1.219458 | 6.456534 | 2.31E-10 | 1.92E-09 | 12.23056 |
| ENSG00000184992.10 | BRI3BP | 0.89483 | 2.397649 | 11.03898 | 8.52E-26 | 2.36E-24 | 47.31195 |
| ENSG00000158716.7 | DUSP23 | 0.894683 | 5.726484 | 7.380032 | 5.67E-13 | 6.08E-12 | 18.12162 |
| ENSG00000172366.18 | FAM195A | 0.894246 | 2.695323 | 9.79254 | 5.05E-21 | 1.01E-19 | 36.41941 |
| ENSG00000186007.8 | LEMD1 | 0.894032 | 1.123503 | 7.181254 | 2.18E-12 | 2.20E-11 | 16.79789 |
| ENSG00000089127.11 | OAS1 | 0.893986 | 3.907807 | 6.226572 | 9.29E-10 | 7.26E-09 | 10.86854 |
| ENSG00000136271.9 | DDX56 | 0.893461 | 4.319857 | 14.4385 | 1.83E-40 | 1.21E-38 | 80.88111 |
| ENSG00000177192.12 | PUS1 | 0.892776 | 2.247212 | 12.02657 | 7.86E-30 | 2.81E-28 | 56.53418 |
| ENSG00000119969.13 | HELLS | 0.89227 | 1.171037 | 11.53709 | 8.35E-28 | 2.63E-26 | 51.90221 |
| ENSG00000163159.10 | VPS72 | 0.891896 | 3.733431 | 12.96124 | 7.86E-34 | 3.60E-32 | 65.68746 |
| ENSG00000127824.12 | TUBA4A | 0.891192 | 4.365133 | 7.710694 | 5.65E-14 | 6.57E-13 | 20.38923 |
| ENSG00000161981.9 | SNRNP25 | 0.890859 | 3.310394 | 12.36519 | 2.92E-31 | 1.14E-29 | 59.80524 |
| ENSG00000137274.11 | BPHL | 0.890376 | 2.057812 | 12.25721 | 8.40E-31 | 3.17E-29 | 58.7564 |
| ENSG00000232119.6 | MCTS1 | 0.890282 | 3.679449 | 14.19695 | 2.34E-39 | 1.47E-37 | 78.34192 |
| ENSG00000049656.12 | CLPTM1L | 0.890095 | 5.276066 | 9.422991 | 1.10E-19 | 2.01E-18 | 33.36596 |
| ENSG00000123415.13 | SMUG1 | 0.88942 | 2.846492 | 13.25742 | 3.93E-35 | 1.94E-33 | 68.66638 |
| ENSG00000115657.11 | ABCB6 | 0.888508 | 1.328038 | 9.961446 | 1.20E-21 | 2.51E-20 | 37.84282 |
| ENSG00000129810.13 | SGOL1 | 0.88814 | 0.966121 | 11.17208 | 2.51E-26 | 7.18E-25 | 48.52588 |
| ENSG00000180573.9 | HIST1H2AC | 0.885595 | 4.692856 | 5.805907 | 1.07E-08 | 7.45E-08 | 8.489154 |
| ENSG00000127191.16 | TRAF2 | 0.884864 | 3.142945 | 10.18191 | 1.79E-22 | 3.99E-21 | 39.72628 |
| ENSG00000100116.15 | GCAT | 0.884593 | 2.795355 | 8.379345 | 4.21E-16 | 5.84E-15 | 25.21737 |
| ENSG00000136231.12 | IGF2BP3 | 0.884474 | 0.924615 | 6.717347 | 4.51E-11 | 4.04E-10 | 13.82672 |
| ENSG00000092853.12 | CLSPN | 0.884432 | 1.060856 | 9.676038 | 1.35E-20 | 2.59E-19 | 35.4477 |
| ENSG00000277791.3 | PSMB3 | 0.884307 | 6.621297 | 10.77216 | 9.62E-25 | 2.49E-23 | 44.90676 |
| ENSG00000168439.15 | STIP1 | 0.884296 | 5.265927 | 12.69386 | 1.14E-32 | 4.86E-31 | 63.02964 |
| ENSG00000131778.16 | CHD1L | 0.883926 | 4.21029 | 11.52826 | 9.07E-28 | 2.85E-26 | 51.81978 |
| ENSG00000164695.4 | CHMP4C | 0.88386 | 3.760761 | 8.983991 | 3.86E-18 | 6.31E-17 | 29.85072 |
| ENSG00000110944.7 | IL23A | 0.883688 | 1.403541 | 8.315589 | 6.81E-16 | 9.29E-15 | 24.74335 |
| ENSG00000125247.14 | TMTC4 | 0.883615 | 2.350561 | 11.16982 | 2.56E-26 | 7.32E-25 | 48.5052 |
| ENSG00000136518.15 | ACTL6A | 0.882422 | 3.945339 | 11.05373 | 7.44E-26 | 2.07E-24 | 47.44597 |
| ENSG00000198431.14 | TXNRD1 | 0.882067 | 5.095544 | 4.549615 | 6.58E-06 | 3.27E-05 | 2.28018 |
| ENSG00000196917.5 | HCAR1 | 0.882038 | 1.187366 | 6.180085 | 1.23E-09 | 9.47E-09 | 10.59843 |
| ENSG00000010292.11 | NCAPD2 | 0.881726 | 3.6666 | 8.717829 | 3.14E-17 | 4.76E-16 | 27.78076 |
| ENSG00000130731.14 | C16orf13 | 0.881713 | 4.701001 | 10.15651 | 2.23E-22 | 4.94E-21 | 39.50784 |
| ENSG00000163507.12 | KIAA1524 | 0.88166 | 1.396358 | 9.131778 | 1.18E-18 | 2.00E-17 | 31.02023 |
| ENSG00000135632.10 | SMYD5 | 0.881091 | 3.49857 | 16.92198 | 2.71E-52 | 3.18E-50 | 108.0008 |
| ENSG00000124571.16 | XPO5 | 0.880969 | 2.929577 | 11.99858 | 1.03E-29 | 3.66E-28 | 56.2662 |
| ENSG00000071894.13 | CPSF1 | 0.880796 | 4.100556 | 9.264807 | 4.03E-19 | 7.08E-18 | 32.08506 |
| ENSG00000197172.9 | MAGEA6 | 0.880526 | 0.809011 | 3.86157 | 0.000125652 | 0.000514754 | -0.52626 |
| ENSG00000131941.6 | RHPN2 | 0.880525 | 2.80601 | 6.955691 | 9.71E-12 | 9.23E-11 | 15.33236 |
| ENSG00000149743.12 | TRPT1 | 0.879859 | 3.479872 | 12.63586 | 2.03E-32 | 8.53E-31 | 62.45719 |
| ENSG00000007376.6 | RPUSD1 | 0.879628 | 3.213204 | 11.28106 | 9.14E-27 | 2.70E-25 | 49.52676 |
| ENSG00000213937.3 | CLDN9 | 0.87942 | 1.569016 | 5.153836 | 3.53E-07 | 2.07E-06 | 5.095262 |
| ENSG00000151208.15 | DLG5 | 0.878762 | 2.34863 | 9.701519 | 1.09E-20 | 2.12E-19 | 35.65952 |
| ENSG00000134258.15 | VTCN1 | 0.878712 | 1.305878 | 4.769753 | 2.35E-06 | 1.24E-05 | 3.268401 |
| ENSG00000140511.10 | HAPLN3 | 0.878622 | 2.410549 | 7.578624 | 1.43E-13 | 1.62E-12 | 19.47379 |
| ENSG00000006634.6 | DBF4 | 0.87852 | 1.869802 | 10.86218 | 4.26E-25 | 1.14E-23 | 45.71396 |
| ENSG00000151131.8 | C12orf45 | 0.877848 | 2.909138 | 13.02869 | 3.98E-34 | 1.87E-32 | 66.3627 |
| ENSG00000225697.9 | SLC26A6 | 0.877848 | 1.925974 | 9.898482 | 2.05E-21 | 4.24E-20 | 37.3102 |
| ENSG00000149636.14 | DSN1 | 0.87748 | 3.112381 | 10.21714 | 1.32E-22 | 2.96E-21 | 40.02994 |
| ENSG00000113407.12 | TARS | 0.877371 | 4.116507 | 9.986318 | 9.69E-22 | 2.04E-20 | 38.05387 |
| ENSG00000213462.4 | ERV3-1 | 0.87657 | 2.731205 | 5.832793 | 9.17E-09 | 6.46E-08 | 8.636831 |
| ENSG00000172336.4 | POP7 | 0.876382 | 4.679946 | 10.06337 | 4.99E-22 | 1.07E-20 | 38.71005 |
| ENSG00000115718.16 | PROC | 0.87594 | 0.945252 | 6.493095 | 1.84E-10 | 1.55E-09 | 12.45104 |
| ENSG00000068120.13 | COASY | 0.875261 | 4.436097 | 14.67919 | 1.41E-41 | 1.01E-39 | 83.4309 |
| ENSG00000167535.6 | CACNB3 | 0.874979 | 2.48372 | 9.127425 | 1.23E-18 | 2.07E-17 | 30.98558 |
| ENSG00000087510.6 | TFAP2C | 0.874921 | 2.734148 | 7.132429 | 3.02E-12 | 3.01E-11 | 16.47735 |
| ENSG00000102996.4 | MMP15 | 0.873885 | 4.271618 | 6.776858 | 3.09E-11 | 2.80E-10 | 14.19848 |
| ENSG00000128654.12 | MTX2 | 0.873536 | 4.495484 | 14.74675 | 6.84E-42 | 4.99E-40 | 84.14996 |
| ENSG00000100908.12 | EMC9 | 0.87329 | 2.994281 | 10.76477 | 1.03E-24 | 2.66E-23 | 44.84069 |
| ENSG00000108551.4 | RASD1 | 0.87292 | 4.667417 | 3.507536 | 0.000488145 | 0.001799833 | -1.80057 |
| ENSG00000169220.16 | RGS14 | 0.87285 | 2.824801 | 10.57751 | 5.51E-24 | 1.36E-22 | 43.17669 |
| ENSG00000198715.10 | GLMP | 0.872807 | 4.217888 | 11.46447 | 1.65E-27 | 5.10E-26 | 51.22508 |
| ENSG00000123219.11 | CENPK | 0.871687 | 1.199037 | 10.44601 | 1.77E-23 | 4.21E-22 | 42.01989 |
| ENSG00000168758.9 | SEMA4C | 0.871414 | 3.488984 | 10.21521 | 1.34E-22 | 3.01E-21 | 40.0133 |
| ENSG00000129038.14 | LOXL1 | 0.870936 | 2.868408 | 7.396257 | 5.07E-13 | 5.46E-12 | 18.23098 |
| ENSG00000152256.12 | PDK1 | 0.870926 | 1.509066 | 11.58176 | 5.48E-28 | 1.74E-26 | 52.32005 |
| ENSG00000203805.9 | PPAPDC1A | 0.870801 | 0.885514 | 7.491369 | 2.63E-13 | 2.91E-12 | 18.87607 |
| ENSG00000178096.8 | BOLA1 | 0.87061 | 3.442487 | 9.697603 | 1.12E-20 | 2.18E-19 | 35.62694 |
| ENSG00000256525.5 | POLG2 | 0.870202 | 2.04422 | 12.4876 | 8.78E-32 | 3.54E-30 | 61.00058 |
| ENSG00000137142.4 | IGFBPL1 | 0.869876 | 1.29408 | 6.439156 | 2.56E-10 | 2.13E-09 | 12.12615 |
| ENSG00000112118.16 | MCM3 | 0.86969 | 4.684481 | 9.972344 | 1.09E-21 | 2.29E-20 | 37.93525 |
| ENSG00000126453.8 | BCL2L12 | 0.869465 | 2.994567 | 11.26727 | 1.04E-26 | 3.06E-25 | 49.39977 |
| ENSG00000135446.15 | CDK4 | 0.868799 | 4.568977 | 9.002185 | 3.34E-18 | 5.48E-17 | 29.99393 |
| ENSG00000203724.9 | C1orf53 | 0.868666 | 2.64787 | 7.739666 | 4.60E-14 | 5.39E-13 | 20.59176 |
| ENSG00000011009.9 | LYPLA2 | 0.868465 | 5.294399 | 11.6257 | 3.62E-28 | 1.17E-26 | 52.73205 |
| ENSG00000115806.11 | GORASP2 | 0.868249 | 4.873198 | 15.72488 | 1.68E-46 | 1.56E-44 | 94.71738 |
| ENSG00000112378.11 | PERP | 0.868194 | 5.714303 | 7.323196 | 8.36E-13 | 8.84E-12 | 17.74007 |
| ENSG00000213983.10 | AP1G2 | 0.867596 | 2.848388 | 9.199356 | 6.85E-19 | 1.18E-17 | 31.55973 |
| ENSG00000142583.16 | SLC2A5 | 0.867536 | 1.06681 | 9.100929 | 1.52E-18 | 2.55E-17 | 30.77493 |
| ENSG00000070501.10 | POLB | 0.867189 | 2.824632 | 9.7486 | 7.31E-21 | 1.44E-19 | 36.05195 |
| ENSG00000109113.16 | RAB34 | 0.867157 | 4.844289 | 10.67361 | 2.33E-24 | 5.88E-23 | 44.02825 |
| ENSG00000158042.8 | MRPL17 | 0.867144 | 4.535477 | 13.08341 | 2.29E-34 | 1.09E-32 | 66.91193 |
| ENSG00000204237.4 | OXLD1 | 0.865418 | 3.729599 | 10.19025 | 1.67E-22 | 3.72E-21 | 39.79811 |
| ENSG00000128578.8 | STRIP2 | 0.864766 | 1.170849 | 7.029633 | 5.98E-12 | 5.80E-11 | 15.80846 |
| ENSG00000179958.7 | DCTPP1 | 0.864745 | 4.455337 | 9.874887 | 2.51E-21 | 5.14E-20 | 37.11122 |
| ENSG00000026751.15 | SLAMF7 | 0.864614 | 2.97968 | 5.480167 | 6.40E-08 | 4.10E-07 | 6.748349 |
| ENSG00000198915.10 | RASGEF1A | 0.864332 | 1.35886 | 7.833535 | 2.35E-14 | 2.83E-13 | 21.25218 |
| ENSG00000134317.16 | GRHL1 | 0.863736 | 1.750996 | 7.710569 | 5.66E-14 | 6.58E-13 | 20.38836 |
| ENSG00000185100.9 | ADSSL1 | 0.862802 | 1.689514 | 7.544826 | 1.82E-13 | 2.03E-12 | 19.24159 |
| ENSG00000159063.11 | ALG8 | 0.862759 | 3.584744 | 13.08368 | 2.29E-34 | 1.09E-32 | 66.91459 |
| ENSG00000131037.13 | EPS8L1 | 0.862104 | 2.777428 | 6.61661 | 8.53E-11 | 7.40E-10 | 13.2038 |
| ENSG00000178078.10 | STAP2 | 0.86206 | 3.508401 | 9.1447 | 1.07E-18 | 1.81E-17 | 31.12317 |
| ENSG00000259680.5 | RP11-812E19.9 | 0.861476 | 1.208905 | 5.807251 | 1.06E-08 | 7.40E-08 | 8.496522 |
| ENSG00000171621.12 | SPSB1 | 0.861134 | 4.103126 | 8.890522 | 8.10E-18 | 1.29E-16 | 29.11845 |
| ENSG00000242372.5 | EIF6 | 0.861132 | 6.251208 | 12.51371 | 6.78E-32 | 2.76E-30 | 61.25633 |
| ENSG00000167130.16 | DOLPP1 | 0.861095 | 3.440001 | 14.27423 | 1.04E-39 | 6.60E-38 | 79.15206 |
| ENSG00000105639.17 | JAK3 | 0.860822 | 2.220286 | 8.869559 | 9.56E-18 | 1.51E-16 | 28.95501 |
| ENSG00000130005.10 | GAMT | 0.860776 | 2.79543 | 7.034967 | 5.77E-12 | 5.61E-11 | 15.84296 |
| ENSG00000160124.8 | CCDC58 | 0.860572 | 2.995707 | 10.96171 | 1.73E-25 | 4.71E-24 | 46.61148 |
| ENSG00000119285.9 | HEATR1 | 0.860239 | 2.589412 | 12.40687 | 1.94E-31 | 7.68E-30 | 60.21147 |
| ENSG00000153944.9 | MSI2 | 0.859644 | 2.138677 | 12.16982 | 1.97E-30 | 7.27E-29 | 57.9115 |
| ENSG00000138834.11 | MAPK8IP3 | 0.859329 | 2.627241 | 6.996087 | 7.45E-12 | 7.16E-11 | 15.59194 |
| ENSG00000109062.8 | SLC9A3R1 | 0.858964 | 4.694822 | 7.573188 | 1.49E-13 | 1.68E-12 | 19.43639 |
| ENSG00000168061.12 | SAC3D1 | 0.858411 | 2.736614 | 11.04913 | 7.76E-26 | 2.16E-24 | 47.40418 |
| ENSG00000101294.15 | HM13 | 0.857754 | 4.412557 | 14.86143 | 2.00E-42 | 1.50E-40 | 85.37396 |
| ENSG00000211448.10 | DIO2 | 0.857421 | 1.158284 | 7.13582 | 2.96E-12 | 2.94E-11 | 16.49955 |
| ENSG00000186665.8 | C17orf58 | 0.857383 | 2.941904 | 10.50845 | 1.02E-23 | 2.46E-22 | 42.56791 |
| ENSG00000099901.15 | RANBP1 | 0.857312 | 3.71139 | 10.81708 | 6.41E-25 | 1.69E-23 | 45.30897 |
| ENSG00000141552.16 | ANAPC11 | 0.856862 | 4.204967 | 10.05857 | 5.21E-22 | 1.12E-20 | 38.66901 |
| ENSG00000099256.17 | PRTFDC1 | 0.856515 | 2.196544 | 8.008516 | 6.63E-15 | 8.33E-14 | 22.50029 |
| ENSG00000142207.6 | URB1 | 0.85626 | 2.478029 | 10.44886 | 1.73E-23 | 4.11E-22 | 42.04484 |
| ENSG00000120053.10 | GOT1 | 0.856006 | 4.433051 | 8.87312 | 9.30E-18 | 1.47E-16 | 28.98275 |
| ENSG00000143443.9 | C1orf56 | 0.855672 | 2.361137 | 9.910534 | 1.85E-21 | 3.84E-20 | 37.41197 |
| ENSG00000173660.10 | UQCRH | 0.855395 | 6.042422 | 10.44348 | 1.81E-23 | 4.30E-22 | 41.9977 |
| ENSG00000204366.3 | ZBTB12 | 0.855383 | 2.045627 | 9.253987 | 4.40E-19 | 7.70E-18 | 31.99803 |
| ENSG00000105185.10 | PDCD5 | 0.854367 | 4.310817 | 10.51439 | 9.66E-24 | 2.34E-22 | 42.62022 |
| ENSG00000182472.7 | CAPN12 | 0.85389 | 1.36018 | 8.139184 | 2.54E-15 | 3.31E-14 | 23.44663 |
| ENSG00000085733.14 | CTTN | 0.853624 | 5.399783 | 11.01118 | 1.10E-25 | 3.04E-24 | 47.05954 |
| ENSG00000064547.12 | LPAR2 | 0.853002 | 2.80684 | 11.65791 | 2.67E-28 | 8.74E-27 | 53.03465 |
| ENSG00000275713.1 | HIST1H2BH | 0.852903 | 0.912116 | 5.5926 | 3.48E-08 | 2.30E-07 | 7.339053 |
| ENSG00000164818.14 | DNAAF5 | 0.852316 | 2.839937 | 12.58856 | 3.24E-32 | 1.34E-30 | 61.9914 |
| ENSG00000254986.6 | DPP3 | 0.852033 | 4.06487 | 12.38031 | 2.52E-31 | 9.85E-30 | 59.95253 |
| ENSG00000132768.12 | DPH2 | 0.851512 | 3.299651 | 12.91794 | 1.21E-33 | 5.52E-32 | 65.25498 |
| ENSG00000114993.14 | RTKN | 0.851474 | 3.083232 | 11.85461 | 4.10E-29 | 1.41E-27 | 54.89372 |
| ENSG00000108375.11 | RNF43 | 0.850626 | 2.459386 | 7.534157 | 1.96E-13 | 2.18E-12 | 19.16847 |
| ENSG00000131876.15 | SNRPA1 | 0.850581 | 3.102163 | 12.39248 | 2.24E-31 | 8.80E-30 | 60.0711 |
| ENSG00000147799.10 | ARHGAP39 | 0.850432 | 2.074338 | 9.045084 | 2.37E-18 | 3.93E-17 | 30.33245 |
| ENSG00000138755.5 | CXCL9 | 0.850425 | 4.184707 | 3.431052 | 0.000645062 | 0.00232137 | -2.06049 |
| ENSG00000182022.16 | CHST15 | 0.850242 | 3.522191 | 7.911768 | 1.34E-14 | 1.65E-13 | 21.80747 |
| ENSG00000187741.13 | FANCA | 0.850039 | 1.275806 | 11.71897 | 1.49E-28 | 4.97E-27 | 53.60969 |
| ENSG00000164251.4 | F2RL1 | 0.849876 | 3.069303 | 5.68465 | 2.10E-08 | 1.42E-07 | 7.830658 |
| ENSG00000088836.11 | SLC4A11 | 0.849454 | 1.525873 | 5.996142 | 3.60E-09 | 2.64E-08 | 9.547034 |
| ENSG00000166012.13 | TAF1D | 0.849087 | 3.054251 | 11.94709 | 1.69E-29 | 5.93E-28 | 55.7742 |
| ENSG00000118322.11 | ATP10B | 0.848731 | 0.805478 | 6.709859 | 4.73E-11 | 4.22E-10 | 13.78014 |
| ENSG00000134460.14 | IL2RA | 0.848598 | 1.769838 | 7.012334 | 6.70E-12 | 6.47E-11 | 15.69669 |
| ENSG00000148334.13 | PTGES2 | 0.848529 | 3.873217 | 12.32208 | 4.46E-31 | 1.71E-29 | 59.38585 |
| ENSG00000127952.15 | STYXL1 | 0.848425 | 4.190464 | 10.8359 | 5.41E-25 | 1.43E-23 | 45.47782 |
| ENSG00000110104.10 | CCDC86 | 0.847933 | 3.557168 | 11.19926 | 1.95E-26 | 5.64E-25 | 48.77496 |
| ENSG00000164924.16 | YWHAZ | 0.847274 | 6.717101 | 11.11843 | 4.11E-26 | 1.16E-24 | 48.03542 |
| ENSG00000224877.3 | C17orf89 | 0.846926 | 3.520451 | 11.46993 | 1.57E-27 | 4.85E-26 | 51.27591 |
| ENSG00000164896.18 | FASTK | 0.846911 | 4.304242 | 10.42781 | 2.08E-23 | 4.91E-22 | 41.86052 |
| ENSG00000102384.12 | CENPI | 0.846622 | 1.00244 | 10.65449 | 2.77E-24 | 6.94E-23 | 43.85841 |
| ENSG00000100968.12 | NFATC4 | 0.84643 | 2.292645 | 8.271128 | 9.51E-16 | 1.28E-14 | 24.41447 |
| ENSG00000167862.8 | ICT1 | 0.846148 | 4.421919 | 11.83757 | 4.83E-29 | 1.65E-27 | 54.73194 |
| ENSG00000179889.17 | PDXDC1 | 0.846116 | 4.164148 | 11.73893 | 1.24E-28 | 4.13E-27 | 53.7981 |
| ENSG00000166965.11 | RCCD1 | 0.846011 | 1.766431 | 12.88631 | 1.67E-33 | 7.51E-32 | 64.93959 |
| ENSG00000169684.12 | CHRNA5 | 0.845982 | 0.91396 | 7.827587 | 2.46E-14 | 2.95E-13 | 21.21014 |
| ENSG00000151239.12 | TWF1 | 0.845917 | 4.04406 | 9.959417 | 1.22E-21 | 2.56E-20 | 37.82563 |
| ENSG00000138778.10 | CENPE | 0.845711 | 1.032859 | 10.05149 | 5.53E-22 | 1.19E-20 | 38.60867 |
| ENSG00000117385.14 | P3H1 | 0.843976 | 2.764464 | 11.63643 | 3.27E-28 | 1.06E-26 | 52.83279 |
| ENSG00000076554.14 | TPD52 | 0.843975 | 3.704891 | 8.229029 | 1.30E-15 | 1.73E-14 | 24.10433 |
| ENSG00000181449.3 | SOX2 | 0.843956 | 2.241994 | 3.655007 | 0.00028109 | 0.001084119 | -1.28396 |
| ENSG00000106268.14 | NUDT1 | 0.843536 | 2.878723 | 10.16333 | 2.10E-22 | 4.66E-21 | 39.56646 |
| ENSG00000134709.9 | HOOK1 | 0.843167 | 2.667488 | 9.560744 | 3.53E-20 | 6.63E-19 | 34.49427 |
| ENSG00000163235.14 | TGFA | 0.842715 | 2.751753 | 5.581512 | 3.70E-08 | 2.44E-07 | 7.280318 |
| ENSG00000137267.5 | TUBB2A | 0.842577 | 3.169453 | 6.133045 | 1.62E-09 | 1.23E-08 | 10.32691 |
| ENSG00000124813.19 | RUNX2 | 0.842506 | 1.560783 | 9.702387 | 1.08E-20 | 2.10E-19 | 35.66674 |
| ENSG00000006047.11 | YBX2 | 0.842251 | 0.822273 | 6.867017 | 1.73E-11 | 1.60E-10 | 14.76701 |
| ENSG00000086015.19 | MAST2 | 0.842206 | 3.007956 | 10.67336 | 2.34E-24 | 5.89E-23 | 44.02599 |
| ENSG00000079112.8 | CDH17 | 0.842039 | 0.868916 | 4.395023 | 1.32E-05 | 6.29E-05 | 1.612156 |
| ENSG00000156413.12 | FUT6 | 0.842016 | 0.90168 | 5.953533 | 4.60E-09 | 3.35E-08 | 9.307473 |
| ENSG00000185885.14 | IFITM1 | 0.841971 | 6.50244 | 5.226963 | 2.43E-07 | 1.45E-06 | 5.457706 |
| ENSG00000124562.8 | SNRPC | 0.841955 | 5.809661 | 12.26449 | 7.82E-31 | 2.96E-29 | 58.82697 |
| ENSG00000166546.12 | BEAN1 | 0.841895 | 0.996788 | 8.319678 | 6.61E-16 | 9.02E-15 | 24.77367 |
| ENSG00000103353.14 | UBFD1 | 0.841696 | 3.120733 | 13.48411 | 3.87E-36 | 2.05E-34 | 70.97021 |
| ENSG00000007968.6 | E2F2 | 0.84161 | 1.0593 | 10.54168 | 7.58E-24 | 1.85E-22 | 42.86051 |
| ENSG00000067704.9 | IARS2 | 0.841109 | 5.148352 | 12.17962 | 1.79E-30 | 6.64E-29 | 58.00608 |
| ENSG00000162769.11 | FLVCR1 | 0.841081 | 2.106735 | 10.48923 | 1.21E-23 | 2.90E-22 | 42.39901 |
| ENSG00000140988.14 | RPS2 | 0.840912 | 8.283077 | 10.1375 | 2.63E-22 | 5.79E-21 | 39.3446 |
| ENSG00000173237.4 | C11orf86 | 0.840348 | 0.766292 | 5.316368 | 1.53E-07 | 9.37E-07 | 5.907122 |
| ENSG00000100263.12 | RHBDD3 | 0.84028 | 3.021058 | 10.7485 | 1.19E-24 | 3.08E-23 | 44.69534 |
| ENSG00000187260.14 | WDR86 | 0.839868 | 1.258886 | 6.453398 | 2.35E-10 | 1.96E-09 | 12.2117 |
| ENSG00000121039.8 | RDH10 | 0.839821 | 3.923311 | 4.866079 | 1.48E-06 | 7.97E-06 | 3.714368 |
| ENSG00000128610.10 | FEZF1 | 0.839756 | 0.793865 | 6.437587 | 2.59E-10 | 2.15E-09 | 12.11673 |
| ENSG00000146197.8 | SCUBE3 | 0.839327 | 1.302066 | 5.808696 | 1.05E-08 | 7.34E-08 | 8.504446 |
| ENSG00000169689.13 | STRA13 | 0.838641 | 4.622048 | 8.079977 | 3.93E-15 | 5.04E-14 | 23.01633 |
| ENSG00000134375.9 | TIMM17A | 0.838595 | 4.142736 | 13.32407 | 1.99E-35 | 1.01E-33 | 69.3416 |
| ENSG00000116161.16 | CACYBP | 0.838335 | 4.046962 | 11.27203 | 9.94E-27 | 2.93E-25 | 49.44363 |
| ENSG00000129925.9 | TMEM8A | 0.838333 | 4.868551 | 10.34565 | 4.28E-23 | 9.89E-22 | 41.14361 |
| ENSG00000083444.15 | PLOD1 | 0.837902 | 4.998811 | 10.38735 | 2.97E-23 | 6.93E-22 | 41.50694 |
| ENSG00000127922.8 | SHFM1 | 0.837814 | 3.023075 | 11.17444 | 2.45E-26 | 7.04E-25 | 48.54753 |
| ENSG00000125995.14 | ROMO1 | 0.837746 | 6.16756 | 8.302165 | 7.54E-16 | 1.02E-14 | 24.64391 |
| ENSG00000183763.7 | TRAIP | 0.837572 | 1.273218 | 12.59366 | 3.08E-32 | 1.28E-30 | 62.04159 |
| ENSG00000165376.9 | CLDN2 | 0.837489 | 1.943167 | 2.978799 | 0.003017731 | 0.009479172 | -3.48451 |
| ENSG00000145604.14 | SKP2 | 0.837318 | 2.417782 | 7.950035 | 1.01E-14 | 1.26E-13 | 22.0807 |
| ENSG00000172818.8 | OVOL1 | 0.836719 | 1.084433 | 9.267074 | 3.96E-19 | 6.95E-18 | 32.10331 |
| ENSG00000159131.15 | GART | 0.836695 | 3.284205 | 12.96551 | 7.53E-34 | 3.46E-32 | 65.73014 |
| ENSG00000157064.9 | NMNAT2 | 0.835899 | 1.255729 | 5.091218 | 4.85E-07 | 2.79E-06 | 4.788601 |
| ENSG00000136108.13 | CKAP2 | 0.83545 | 2.59613 | 9.10845 | 1.43E-18 | 2.40E-17 | 30.83468 |
| ENSG00000163435.14 | ELF3 | 0.834905 | 5.285621 | 6.538919 | 1.39E-10 | 1.18E-09 | 12.72889 |
| ENSG00000105778.16 | AVL9 | 0.834532 | 2.591185 | 12.37017 | 2.78E-31 | 1.08E-29 | 59.8537 |
| ENSG00000163013.10 | FBXO41 | 0.834103 | 1.365651 | 9.882024 | 2.36E-21 | 4.85E-20 | 37.17137 |
| ENSG00000160691.17 | SHC1 | 0.833743 | 5.272822 | 9.803688 | 4.59E-21 | 9.24E-20 | 36.51283 |
| ENSG00000120992.16 | LYPLA1 | 0.832772 | 4.328937 | 7.953956 | 9.86E-15 | 1.23E-13 | 22.10876 |
| ENSG00000197712.10 | FAM114A1 | 0.832653 | 4.098848 | 10.58034 | 5.37E-24 | 1.32E-22 | 43.20168 |
| ENSG00000186871.6 | ERCC6L | 0.83239 | 0.895635 | 11.4405 | 2.07E-27 | 6.36E-26 | 51.00212 |
| ENSG00000198794.10 | SCAMP5 | 0.831974 | 1.981535 | 8.320374 | 6.57E-16 | 8.98E-15 | 24.77883 |
| ENSG00000159596.6 | TMEM69 | 0.831572 | 3.521667 | 14.51223 | 8.35E-41 | 5.68E-39 | 81.66018 |
| ENSG00000167103.10 | PIP5KL1 | 0.831541 | 1.685009 | 7.568647 | 1.54E-13 | 1.73E-12 | 19.40516 |
| ENSG00000100304.12 | TTLL12 | 0.831399 | 3.621861 | 9.154839 | 9.82E-19 | 1.67E-17 | 31.204 |
| ENSG00000101199.11 | ARFGAP1 | 0.831329 | 3.922211 | 9.908916 | 1.88E-21 | 3.89E-20 | 37.39831 |
| ENSG00000141378.14 | PTRH2 | 0.83032 | 2.509125 | 13.48578 | 3.81E-36 | 2.02E-34 | 70.98731 |
| ENSG00000152455.14 | SUV39H2 | 0.830304 | 1.850055 | 12.15187 | 2.34E-30 | 8.60E-29 | 57.73836 |
| ENSG00000159231.5 | CBR3 | 0.829946 | 2.531018 | 5.284955 | 1.80E-07 | 1.09E-06 | 5.748427 |
| ENSG00000159228.11 | CBR1 | 0.829737 | 5.13063 | 4.588414 | 5.50E-06 | 2.76E-05 | 2.451211 |
| ENSG00000203668.1 | CHML | 0.829181 | 2.489696 | 6.81798 | 2.37E-11 | 2.17E-10 | 14.457 |
| ENSG00000104081.12 | BMF | 0.82898 | 3.081643 | 7.986483 | 7.78E-15 | 9.74E-14 | 22.34192 |
| ENSG00000119411.10 | BSPRY | 0.828803 | 3.794569 | 8.014217 | 6.36E-15 | 8.01E-14 | 22.54132 |
| ENSG00000125450.9 | NUP85 | 0.828293 | 3.213451 | 12.94424 | 9.32E-34 | 4.26E-32 | 65.51757 |
| ENSG00000144136.9 | SLC20A1 | 0.825324 | 3.745033 | 8.857844 | 1.05E-17 | 1.65E-16 | 28.8638 |
| ENSG00000116670.13 | MAD2L2 | 0.825124 | 3.17561 | 9.585258 | 2.88E-20 | 5.44E-19 | 34.6963 |
| ENSG00000242114.4 | MTFP1 | 0.824524 | 1.973326 | 11.49593 | 1.23E-27 | 3.83E-26 | 51.51813 |
| ENSG00000186222.4 | BLOC1S4 | 0.824457 | 3.50183 | 12.33177 | 4.05E-31 | 1.56E-29 | 59.48002 |
| ENSG00000143977.12 | SNRPG | 0.823955 | 4.526283 | 9.110969 | 1.40E-18 | 2.36E-17 | 30.8547 |
| ENSG00000204335.3 | SP5 | 0.823054 | 1.337407 | 5.602677 | 3.30E-08 | 2.18E-07 | 7.392522 |
| ENSG00000108515.16 | ENO3 | 0.822868 | 1.376203 | 5.17571 | 3.16E-07 | 1.86E-06 | 5.203188 |
| ENSG00000101470.8 | TNNC2 | 0.822473 | 1.928996 | 3.397167 | 0.000728648 | 0.00259775 | -2.17389 |
| ENSG00000126953.5 | TIMM8A | 0.822095 | 1.893125 | 13.53122 | 2.39E-36 | 1.29E-34 | 71.45152 |
| ENSG00000196636.7 | ACN9 | 0.822 | 2.82425 | 9.749097 | 7.28E-21 | 1.44E-19 | 36.05609 |
| ENSG00000143387.11 | CTSK | 0.8218 | 4.896996 | 5.213901 | 2.60E-07 | 1.55E-06 | 5.392624 |
| ENSG00000055044.9 | NOP58 | 0.821536 | 4.450323 | 13.2507 | 4.21E-35 | 2.08E-33 | 68.59839 |
| ENSG00000121900.17 | TMEM54 | 0.821403 | 5.36866 | 8.165561 | 2.09E-15 | 2.74E-14 | 23.63913 |
| ENSG00000125869.8 | LAMP5 | 0.821292 | 1.657126 | 6.650587 | 6.89E-11 | 6.04E-10 | 13.413 |
| ENSG00000086598.9 | TMED2 | 0.821148 | 6.573766 | 10.60003 | 4.51E-24 | 1.12E-22 | 43.3758 |
| ENSG00000082512.13 | TRAF5 | 0.820991 | 1.847107 | 9.321095 | 2.55E-19 | 4.52E-18 | 32.53903 |
| ENSG00000167635.10 | ZNF146 | 0.820758 | 4.498753 | 10.24006 | 1.08E-22 | 2.44E-21 | 40.22786 |
| ENSG00000166794.4 | PPIB | 0.820615 | 7.060152 | 9.904244 | 1.95E-21 | 4.04E-20 | 37.35884 |
| ENSG00000164045.10 | CDC25A | 0.820478 | 1.05273 | 10.15978 | 2.17E-22 | 4.80E-21 | 39.53592 |
| ENSG00000166145.13 | SPINT1 | 0.82046 | 6.156598 | 8.333066 | 5.97E-16 | 8.19E-15 | 24.87301 |
| ENSG00000137692.10 | DCUN1D5 | 0.820258 | 3.839581 | 10.89532 | 3.16E-25 | 8.50E-24 | 46.01218 |
| ENSG00000184575.10 | XPOT | 0.820204 | 3.817543 | 9.374569 | 1.64E-19 | 2.96E-18 | 32.97218 |
| ENSG00000189376.10 | C8orf76 | 0.820154 | 2.218591 | 10.62125 | 3.73E-24 | 9.28E-23 | 43.56361 |
| ENSG00000125871.12 | MGME1 | 0.820152 | 3.222339 | 11.92182 | 2.15E-29 | 7.50E-28 | 55.53324 |
| ENSG00000121073.12 | SLC35B1 | 0.820052 | 3.647977 | 14.87933 | 1.65E-42 | 1.25E-40 | 85.5654 |
| ENSG00000244038.8 | DDOST | 0.819772 | 6.737008 | 14.12087 | 5.21E-39 | 3.23E-37 | 77.54628 |
| ENSG00000141736.12 | ERBB2 | 0.818869 | 4.813216 | 6.943266 | 1.05E-11 | 9.97E-11 | 15.25277 |
| ENSG00000145592.12 | RPL37 | 0.81866 | 6.321087 | 9.260534 | 4.17E-19 | 7.32E-18 | 32.05068 |
| ENSG00000115598.8 | IL1RL2 | 0.818599 | 1.052399 | 10.29899 | 6.45E-23 | 1.48E-21 | 40.73811 |
| ENSG00000160211.14 | G6PD | 0.818296 | 4.991954 | 4.776298 | 2.28E-06 | 1.20E-05 | 3.298442 |
| ENSG00000051128.17 | HOMER3 | 0.817481 | 2.861697 | 8.234317 | 1.25E-15 | 1.67E-14 | 24.14322 |
| ENSG00000171453.16 | POLR1C | 0.817282 | 3.388877 | 12.60677 | 2.70E-32 | 1.13E-30 | 62.17059 |
| ENSG00000155438.10 | NIFK | 0.816708 | 4.042483 | 13.01192 | 4.72E-34 | 2.20E-32 | 66.19465 |
| ENSG00000183161.4 | FANCF | 0.816364 | 2.472999 | 14.31121 | 7.03E-40 | 4.51E-38 | 79.54048 |
| ENSG00000095002.11 | MSH2 | 0.815437 | 3.15799 | 10.01757 | 7.41E-22 | 1.57E-20 | 38.31956 |
| ENSG00000002587.8 | HS3ST1 | 0.815075 | 1.722359 | 6.898547 | 1.41E-11 | 1.32E-10 | 14.96733 |
| ENSG00000101361.13 | NOP56 | 0.814539 | 4.091385 | 10.52075 | 9.13E-24 | 2.21E-22 | 42.67619 |
| ENSG00000198018.6 | ENTPD7 | 0.813898 | 2.247867 | 10.03484 | 6.38E-22 | 1.36E-20 | 38.46665 |
| ENSG00000137563.10 | GGH | 0.813297 | 2.775837 | 5.167365 | 3.29E-07 | 1.94E-06 | 5.161967 |
| ENSG00000185000.8 | DGAT1 | 0.813153 | 3.789506 | 9.024 | 2.81E-18 | 4.63E-17 | 30.16592 |
| ENSG00000130827.6 | PLXNA3 | 0.813126 | 2.657908 | 8.031072 | 5.62E-15 | 7.11E-14 | 22.66278 |
| ENSG00000168411.12 | RFWD3 | 0.813016 | 2.771835 | 12.05576 | 5.93E-30 | 2.14E-28 | 56.81408 |
| ENSG00000130733.9 | YIPF2 | 0.812825 | 4.488831 | 10.99137 | 1.32E-25 | 3.62E-24 | 46.87994 |
| ENSG00000143543.13 | JTB | 0.812823 | 6.726118 | 11.14481 | 3.22E-26 | 9.16E-25 | 48.27641 |
| ENSG00000178927.15 | C17orf62 | 0.812316 | 3.819574 | 12.64806 | 1.80E-32 | 7.58E-31 | 62.57748 |
| ENSG00000197586.11 | ENTPD6 | 0.812268 | 4.183907 | 10.21737 | 1.32E-22 | 2.96E-21 | 40.03194 |
| ENSG00000184343.9 | SRPK3 | 0.812039 | 0.91654 | 7.862028 | 1.92E-14 | 2.33E-13 | 21.4539 |
| ENSG00000188976.9 | NOC2L | 0.812032 | 4.097681 | 11.09567 | 5.06E-26 | 1.42E-24 | 47.82785 |
| ENSG00000178397.11 | FAM220A | 0.811938 | 3.389367 | 11.42527 | 2.38E-27 | 7.30E-26 | 50.86066 |
| ENSG00000058804.11 | NDC1 | 0.810674 | 2.854594 | 10.10577 | 3.46E-22 | 7.52E-21 | 39.07257 |
| ENSG00000121742.14 | GJB6 | 0.810446 | 0.798699 | 5.222158 | 2.49E-07 | 1.49E-06 | 5.433747 |
| ENSG00000173801.15 | JUP | 0.810379 | 6.213085 | 7.759073 | 4.01E-14 | 4.71E-13 | 20.72778 |
| ENSG00000013275.6 | PSMC4 | 0.809484 | 5.251973 | 11.98052 | 1.23E-29 | 4.33E-28 | 56.09348 |
| ENSG00000184162.13 | NR2C2AP | 0.809028 | 3.398935 | 11.29524 | 8.01E-27 | 2.38E-25 | 49.6575 |
| ENSG00000126768.11 | TIMM17B | 0.808996 | 4.151843 | 9.649961 | 1.68E-20 | 3.20E-19 | 35.23134 |
| ENSG00000213160.8 | KLHL23 | 0.80889 | 2.322348 | 8.635859 | 5.93E-17 | 8.77E-16 | 27.15283 |
| ENSG00000100823.10 | APEX1 | 0.808792 | 6.200646 | 11.64548 | 3.00E-28 | 9.79E-27 | 52.91785 |
| ENSG00000136052.8 | SLC41A2 | 0.808454 | 2.781509 | 8.01233 | 6.45E-15 | 8.12E-14 | 22.52774 |
| ENSG00000131435.11 | PDLIM4 | 0.808407 | 2.452752 | 5.114675 | 4.31E-07 | 2.49E-06 | 4.903075 |
| ENSG00000048162.18 | NOP16 | 0.808178 | 2.753117 | 11.0289 | 9.34E-26 | 2.59E-24 | 47.22031 |
| ENSG00000203697.10 | CAPN8 | 0.807861 | 3.559524 | 3.899623 | 0.000107891 | 0.00044702 | -0.38237 |
| ENSG00000106628.9 | POLD2 | 0.807559 | 4.601755 | 10.37294 | 3.37E-23 | 7.83E-22 | 41.38132 |
| ENSG00000166986.11 | MARS | 0.80754 | 4.043603 | 10.78469 | 8.59E-25 | 2.24E-23 | 45.01889 |
| ENSG00000083857.12 | FAT1 | 0.807435 | 3.159164 | 6.039892 | 2.79E-09 | 2.08E-08 | 9.794577 |
| ENSG00000008394.11 | MGST1 | 0.807399 | 5.05805 | 6.029568 | 2.97E-09 | 2.20E-08 | 9.736019 |
| ENSG00000173621.8 | LRFN4 | 0.807348 | 2.471227 | 6.317122 | 5.39E-10 | 4.32E-09 | 11.39974 |
| ENSG00000147536.10 | GINS4 | 0.807062 | 1.033816 | 9.57307 | 3.18E-20 | 6.00E-19 | 34.59581 |
| ENSG00000166426.7 | CRABP1 | 0.807051 | 0.800168 | 4.659048 | 3.96E-06 | 2.02E-05 | 2.766034 |
| ENSG00000100983.8 | GSS | 0.806662 | 4.871795 | 12.14586 | 2.48E-30 | 9.09E-29 | 57.68047 |
| ENSG00000133422.11 | MORC2 | 0.806642 | 3.242324 | 13.02019 | 4.34E-34 | 2.03E-32 | 66.27747 |
| ENSG00000011638.9 | TMEM159 | 0.806594 | 4.453208 | 6.918524 | 1.24E-11 | 1.17E-10 | 15.09465 |
| ENSG00000130695.12 | CEP85 | 0.806539 | 2.261049 | 11.10578 | 4.61E-26 | 1.30E-24 | 47.92002 |
| ENSG00000163516.12 | ANKZF1 | 0.806258 | 2.8981 | 10.64748 | 2.95E-24 | 7.37E-23 | 43.79621 |
| ENSG00000182931.8 | WFDC10B | 0.806191 | 1.188432 | 6.070927 | 2.33E-09 | 1.75E-08 | 9.971135 |
| ENSG00000163155.10 | LYSMD1 | 0.805588 | 2.444157 | 10.49161 | 1.18E-23 | 2.84E-22 | 42.41989 |
| ENSG00000127252.5 | HRASLS | 0.805539 | 0.985447 | 6.980922 | 8.23E-12 | 7.87E-11 | 15.49434 |
| ENSG00000122884.11 | P4HA1 | 0.804744 | 5.029696 | 8.108144 | 3.19E-15 | 4.13E-14 | 23.22073 |
| ENSG00000075702.15 | WDR62 | 0.804644 | 1.048691 | 9.165395 | 9.02E-19 | 1.54E-17 | 31.28824 |
| ENSG00000143369.13 | ECM1 | 0.804402 | 3.722071 | 4.213715 | 2.92E-05 | 0.000132067 | 0.856206 |
| ENSG00000030110.11 | BAK1 | 0.803659 | 3.901045 | 10.52344 | 8.91E-24 | 2.17E-22 | 42.69984 |
| ENSG00000044090.7 | CUL7 | 0.803431 | 3.670415 | 10.16599 | 2.06E-22 | 4.55E-21 | 39.58938 |
| ENSG00000073969.17 | NSF | 0.802545 | 3.650965 | 12.97864 | 6.60E-34 | 3.04E-32 | 65.86144 |
| ENSG00000197008.8 | ZNF138 | 0.802367 | 2.065911 | 11.71359 | 1.57E-28 | 5.22E-27 | 53.55903 |
| ENSG00000144554.9 | FANCD2 | 0.802358 | 1.422451 | 12.07926 | 4.73E-30 | 1.71E-28 | 57.0397 |
| ENSG00000116478.10 | HDAC1 | 0.802274 | 5.023976 | 12.63538 | 2.04E-32 | 8.56E-31 | 62.45252 |
| ENSG00000108010.10 | GLRX3 | 0.801966 | 3.593086 | 11.11896 | 4.09E-26 | 1.16E-24 | 48.04027 |
| ENSG00000141527.15 | CARD14 | 0.801843 | 0.875095 | 7.610859 | 1.14E-13 | 1.30E-12 | 19.69604 |
| ENSG00000019505.6 | SYT13 | 0.801822 | 0.929786 | 4.219466 | 2.85E-05 | 0.000129007 | 0.879726 |
| ENSG00000187531.12 | SIRT7 | 0.80172 | 2.562293 | 11.56037 | 6.70E-28 | 2.13E-26 | 52.11983 |
| ENSG00000196700.6 | ZNF512B | 0.80139 | 3.10353 | 8.181774 | 1.85E-15 | 2.44E-14 | 23.7577 |
| ENSG00000143630.8 | HCN3 | 0.800844 | 1.283113 | 9.797021 | 4.86E-21 | 9.76E-20 | 36.45695 |
| ENSG00000000460.15 | C1orf112 | 0.798816 | 1.267011 | 13.5204 | 2.67E-36 | 1.44E-34 | 71.34095 |
| ENSG00000162961.12 | DPY30 | 0.798554 | 4.087627 | 11.48511 | 1.36E-27 | 4.22E-26 | 51.4173 |
| ENSG00000204580.10 | DDR1 | 0.797465 | 4.976148 | 8.39289 | 3.80E-16 | 5.29E-15 | 25.31843 |
| ENSG00000116120.9 | FARSB | 0.797138 | 3.790642 | 12.768 | 5.44E-33 | 2.39E-31 | 63.76364 |
| ENSG00000117632.19 | STMN1 | 0.796688 | 4.329215 | 6.063924 | 2.43E-09 | 1.82E-08 | 9.931223 |
| ENSG00000076248.9 | UNG | 0.796639 | 3.945155 | 9.931048 | 1.55E-21 | 3.24E-20 | 37.58539 |
| ENSG00000198952.8 | SMG5 | 0.796427 | 4.742171 | 10.61546 | 3.93E-24 | 9.76E-23 | 43.51233 |
| ENSG00000112667.11 | DNPH1 | 0.79625 | 4.451237 | 8.544538 | 1.20E-16 | 1.73E-15 | 26.45862 |
| ENSG00000137747.13 | TMPRSS13 | 0.795869 | 2.540785 | 6.421008 | 2.87E-10 | 2.36E-09 | 12.01736 |
| ENSG00000134910.11 | STT3A | 0.7958 | 4.662042 | 11.61761 | 3.91E-28 | 1.26E-26 | 52.65614 |
| ENSG00000173786.15 | CNP | 0.795411 | 3.93261 | 13.46595 | 4.67E-36 | 2.46E-34 | 70.78495 |
| ENSG00000204351.10 | SKIV2L | 0.795306 | 3.649104 | 12.39455 | 2.19E-31 | 8.63E-30 | 60.09132 |
| ENSG00000090520.9 | DNAJB11 | 0.795297 | 3.846753 | 11.76312 | 9.82E-29 | 3.30E-27 | 54.02668 |
| ENSG00000174851.13 | YIF1A | 0.795212 | 5.02713 | 10.57062 | 5.86E-24 | 1.44E-22 | 43.11585 |
| ENSG00000131653.11 | TRAF7 | 0.79514 | 4.45964 | 12.15532 | 2.26E-30 | 8.33E-29 | 57.77163 |
| ENSG00000106636.6 | YKT6 | 0.794727 | 5.006094 | 10.84336 | 5.06E-25 | 1.34E-23 | 45.54483 |
| ENSG00000134815.17 | DHX34 | 0.794499 | 2.419108 | 10.86502 | 4.16E-25 | 1.11E-23 | 45.73945 |
| ENSG00000178752.14 | FAM132B | 0.794315 | 0.914034 | 6.6122 | 8.76E-11 | 7.60E-10 | 13.17671 |
| ENSG00000164093.14 | PITX2 | 0.794074 | 0.719358 | 6.115106 | 1.80E-09 | 1.37E-08 | 10.22384 |
| ENSG00000117143.12 | UAP1 | 0.793656 | 4.455512 | 10.0562 | 5.31E-22 | 1.14E-20 | 38.64878 |
| ENSG00000135953.9 | MFSD9 | 0.793547 | 2.29826 | 12.55458 | 4.53E-32 | 1.86E-30 | 61.65737 |
| ENSG00000241343.8 | RPL36A | 0.793177 | 4.089351 | 9.190566 | 7.36E-19 | 1.27E-17 | 31.48939 |
| ENSG00000179403.11 | VWA1 | 0.792989 | 4.850961 | 7.278138 | 1.14E-12 | 1.18E-11 | 17.43933 |
| ENSG00000170417.13 | TMEM182 | 0.792274 | 1.368728 | 13.84723 | 9.09E-38 | 5.27E-36 | 74.7019 |
| ENSG00000136883.11 | KIF12 | 0.792149 | 1.870516 | 4.737166 | 2.74E-06 | 1.43E-05 | 3.119394 |
| ENSG00000204392.9 | LSM2 | 0.792102 | 5.087572 | 9.238111 | 5.01E-19 | 8.72E-18 | 31.87046 |
| ENSG00000171246.5 | NPTX1 | 0.791775 | 1.135048 | 3.958982 | 8.48E-05 | 0.000357852 | -0.15524 |
| ENSG00000164078.11 | MST1R | 0.791764 | 3.026785 | 6.715138 | 4.58E-11 | 4.09E-10 | 13.81298 |
| ENSG00000049449.7 | RCN1 | 0.79137 | 4.108633 | 8.844677 | 1.16E-17 | 1.83E-16 | 28.76139 |
| ENSG00000178623.10 | GPR35 | 0.791211 | 1.122538 | 6.22275 | 9.51E-10 | 7.43E-09 | 10.84626 |
| ENSG00000153406.12 | NMRAL1 | 0.790994 | 3.739578 | 11.99108 | 1.11E-29 | 3.93E-28 | 56.19453 |
| ENSG00000143845.13 | ETNK2 | 0.790946 | 1.732408 | 7.193981 | 2.00E-12 | 2.03E-11 | 16.88174 |
| ENSG00000071462.10 | WBSCR22 | 0.790798 | 3.672377 | 12.91106 | 1.30E-33 | 5.89E-32 | 65.18634 |
| ENSG00000061794.11 | MRPS35 | 0.790721 | 5.041689 | 8.91555 | 6.65E-18 | 1.07E-16 | 29.31396 |
| ENSG00000198743.5 | SLC5A3 | 0.790483 | 2.294025 | 7.443503 | 3.67E-13 | 3.99E-12 | 18.55058 |
| ENSG00000110583.11 | NAA40 | 0.790427 | 2.643383 | 10.77711 | 9.20E-25 | 2.39E-23 | 44.95103 |
| ENSG00000197299.9 | BLM | 0.789765 | 1.094863 | 10.92055 | 2.51E-25 | 6.80E-24 | 46.23963 |
| ENSG00000164237.7 | CMBL | 0.789716 | 3.579996 | 4.302297 | 1.99E-05 | 9.21E-05 | 1.221815 |
| ENSG00000138413.12 | IDH1 | 0.789681 | 5.086425 | 7.734289 | 4.78E-14 | 5.59E-13 | 20.55413 |
| ENSG00000141580.14 | WDR45B | 0.789017 | 5.326551 | 9.828777 | 3.71E-21 | 7.52E-20 | 36.72334 |
| ENSG00000161267.10 | BDH1 | 0.788688 | 1.673471 | 9.554489 | 3.72E-20 | 6.97E-19 | 34.44279 |
| ENSG00000125835.16 | SNRPB | 0.788583 | 6.891115 | 8.55782 | 1.08E-16 | 1.57E-15 | 26.55924 |
| ENSG00000168243.9 | GNG4 | 0.788517 | 0.837216 | 4.956252 | 9.51E-07 | 5.27E-06 | 4.139279 |
| ENSG00000138073.12 | PREB | 0.788431 | 4.46014 | 13.96254 | 2.73E-38 | 1.64E-36 | 75.89727 |
| ENSG00000123297.15 | TSFM | 0.788246 | 3.295951 | 10.27155 | 8.20E-23 | 1.87E-21 | 40.50027 |
| ENSG00000173171.13 | MTX1 | 0.78802 | 2.828368 | 12.76748 | 5.47E-33 | 2.40E-31 | 63.7584 |
| ENSG00000197982.13 | C1orf122 | 0.788004 | 4.00298 | 10.56337 | 6.25E-24 | 1.53E-22 | 43.05185 |
| ENSG00000165689.15 | SDCCAG3 | 0.787657 | 3.550413 | 12.89092 | 1.59E-33 | 7.18E-32 | 64.98557 |
| ENSG00000130294.13 | KIF1A | 0.787449 | 0.796297 | 4.044898 | 5.96E-05 | 0.000257517 | 0.179255 |
| ENSG00000110717.9 | NDUFS8 | 0.787013 | 4.091475 | 10.50633 | 1.04E-23 | 2.51E-22 | 42.54929 |
| ENSG00000185480.10 | PARPBP | 0.786945 | 1.10229 | 9.99064 | 9.33E-22 | 1.97E-20 | 38.09058 |
| ENSG00000198087.7 | CD2AP | 0.78684 | 4.210694 | 9.352236 | 1.97E-19 | 3.53E-18 | 32.79106 |
| ENSG00000184840.10 | TMED9 | 0.786814 | 6.567784 | 12.45439 | 1.22E-31 | 4.87E-30 | 60.67555 |
| ENSG00000159214.11 | CCDC24 | 0.786787 | 2.325691 | 8.944424 | 5.29E-18 | 8.56E-17 | 29.54004 |
| ENSG00000040275.15 | SPDL1 | 0.786747 | 1.703125 | 11.25931 | 1.12E-26 | 3.28E-25 | 49.32657 |
| ENSG00000182173.11 | TSEN54 | 0.786302 | 3.585098 | 11.50597 | 1.12E-27 | 3.50E-26 | 51.61169 |
| ENSG00000119333.10 | WDR34 | 0.786035 | 4.862761 | 8.642353 | 5.64E-17 | 8.35E-16 | 27.20241 |
| ENSG00000179218.12 | CALR | 0.785591 | 8.670503 | 13.4806 | 4.02E-36 | 2.13E-34 | 70.93438 |
| ENSG00000175931.11 | UBE2O | 0.78536 | 3.042439 | 11.93585 | 1.88E-29 | 6.59E-28 | 55.66701 |
| ENSG00000069482.6 | GAL | 0.785244 | 0.79018 | 4.901785 | 1.24E-06 | 6.77E-06 | 3.881763 |
| ENSG00000115233.10 | PSMD14 | 0.785213 | 3.353212 | 11.22124 | 1.59E-26 | 4.63E-25 | 48.97659 |
| ENSG00000119203.12 | CPSF3 | 0.784504 | 3.698279 | 13.69642 | 4.35E-37 | 2.43E-35 | 73.14602 |
| ENSG00000049541.9 | RFC2 | 0.784016 | 3.872811 | 9.945794 | 1.37E-21 | 2.86E-20 | 37.7102 |
| ENSG00000157833.11 | GAREML | 0.783464 | 1.151533 | 8.759967 | 2.26E-17 | 3.46E-16 | 28.10532 |
| ENSG00000183684.7 | ALYREF | 0.78311 | 5.214467 | 8.796744 | 1.69E-17 | 2.63E-16 | 28.38957 |
| ENSG00000140939.13 | NOL3 | 0.783098 | 3.695346 | 8.05907 | 4.58E-15 | 5.84E-14 | 22.86498 |
| ENSG00000101464.9 | PIGU | 0.783071 | 3.648485 | 11.64491 | 3.02E-28 | 9.84E-27 | 52.91246 |
| ENSG00000137135.16 | ARHGEF39 | 0.782905 | 0.963363 | 12.17065 | 1.95E-30 | 7.22E-29 | 57.91951 |
| ENSG00000178821.11 | TMEM52 | 0.782862 | 1.185547 | 8.156758 | 2.23E-15 | 2.92E-14 | 23.57483 |
| ENSG00000186603.5 | HPDL | 0.782454 | 0.851 | 7.178112 | 2.23E-12 | 2.24E-11 | 16.77721 |
| ENSG00000118705.15 | RPN2 | 0.781884 | 7.285979 | 10.96651 | 1.65E-25 | 4.51E-24 | 46.65483 |
| ENSG00000143179.11 | UCK2 | 0.781751 | 2.127253 | 6.326524 | 5.10E-10 | 4.09E-09 | 11.45527 |
| ENSG00000143793.11 | C1orf35 | 0.781134 | 2.573992 | 11.55212 | 7.24E-28 | 2.29E-26 | 52.04275 |
| ENSG00000176428.5 | VPS37D | 0.781104 | 1.511856 | 7.921907 | 1.24E-14 | 1.53E-13 | 21.87976 |
| ENSG00000100726.13 | TELO2 | 0.780644 | 3.305682 | 9.421902 | 1.11E-19 | 2.03E-18 | 33.35709 |
| ENSG00000160818.15 | GPATCH4 | 0.780054 | 3.147846 | 10.22189 | 1.26E-22 | 2.85E-21 | 40.07096 |
| ENSG00000106853.15 | PTGR1 | 0.77996 | 3.601357 | 5.400367 | 9.80E-08 | 6.15E-07 | 6.335641 |
| ENSG00000172172.6 | MRPL13 | 0.779949 | 3.140965 | 9.122421 | 1.28E-18 | 2.15E-17 | 30.94576 |
| ENSG00000105829.10 | BET1 | 0.778816 | 2.791876 | 9.407716 | 1.25E-19 | 2.27E-18 | 33.24158 |
| ENSG00000114354.11 | TFG | 0.778522 | 4.584125 | 11.61372 | 4.05E-28 | 1.30E-26 | 52.61961 |
| ENSG00000065154.10 | OAT | 0.778482 | 5.090356 | 6.539427 | 1.38E-10 | 1.18E-09 | 12.73198 |
| ENSG00000158286.11 | RNF207 | 0.77833 | 1.420482 | 8.262257 | 1.02E-15 | 1.37E-14 | 24.34901 |
| ENSG00000102096.9 | PIM2 | 0.778311 | 4.442098 | 5.662606 | 2.37E-08 | 1.60E-07 | 7.712273 |
| ENSG00000064102.13 | ASUN | 0.778168 | 2.981159 | 9.85517 | 2.97E-21 | 6.05E-20 | 36.9452 |
| ENSG00000097046.11 | CDC7 | 0.777979 | 1.804669 | 8.243023 | 1.17E-15 | 1.57E-14 | 24.20729 |
| ENSG00000080819.5 | CPOX | 0.7777 | 2.890096 | 11.33382 | 5.60E-27 | 1.68E-25 | 50.01355 |
| ENSG00000101158.11 | NELFCD | 0.77703 | 3.823457 | 11.18743 | 2.17E-26 | 6.27E-25 | 48.66646 |
| ENSG00000160767.19 | FAM189B | 0.776873 | 3.206931 | 10.65653 | 2.72E-24 | 6.82E-23 | 43.87653 |
| ENSG00000175707.8 | KDF1 | 0.775452 | 3.626312 | 10.64385 | 3.05E-24 | 7.61E-23 | 43.76401 |
| ENSG00000164032.10 | H2AFZ | 0.775316 | 5.952167 | 8.54789 | 1.17E-16 | 1.69E-15 | 26.484 |
| ENSG00000186212.3 | SOWAHB | 0.775181 | 2.045288 | 7.160388 | 2.51E-12 | 2.52E-11 | 16.66068 |
| ENSG00000150961.13 | SEC24D | 0.774179 | 3.288434 | 10.78495 | 8.57E-25 | 2.24E-23 | 45.02114 |
| ENSG00000164104.10 | HMGB2 | 0.773668 | 4.789575 | 7.236334 | 1.51E-12 | 1.55E-11 | 17.16169 |
| ENSG00000156709.12 | AIFM1 | 0.773182 | 3.903462 | 12.21956 | 1.21E-30 | 4.54E-29 | 58.39194 |
| ENSG00000162298.15 | SYVN1 | 0.773063 | 4.367787 | 10.92725 | 2.36E-25 | 6.41E-24 | 46.30008 |
| ENSG00000103550.12 | KNOP1 | 0.772985 | 1.777134 | 14.96836 | 6.33E-43 | 4.87E-41 | 86.51901 |
| ENSG00000205581.9 | HMGN1 | 0.772965 | 4.894556 | 12.48313 | 9.17E-32 | 3.69E-30 | 60.95678 |
| ENSG00000275395.3 | FCGBP | 0.772677 | 2.103026 | 4.057129 | 5.67E-05 | 0.000245675 | 0.227423 |
| ENSG00000160972.8 | PPP1R16A | 0.772574 | 2.615901 | 8.339522 | 5.69E-16 | 7.81E-15 | 24.92096 |
| ENSG00000130204.11 | TOMM40 | 0.772536 | 4.091492 | 9.364307 | 1.79E-19 | 3.21E-18 | 32.88892 |
| ENSG00000134809.7 | TIMM10 | 0.772361 | 4.752415 | 9.857728 | 2.90E-21 | 5.93E-20 | 36.96673 |
| ENSG00000108468.13 | CBX1 | 0.772247 | 4.581715 | 9.835788 | 3.50E-21 | 7.10E-20 | 36.78224 |
| ENSG00000162836.10 | ACP6 | 0.772127 | 1.616083 | 11.62965 | 3.49E-28 | 1.13E-26 | 52.76916 |
| ENSG00000167670.14 | CHAF1A | 0.772119 | 2.284607 | 9.415109 | 1.18E-19 | 2.14E-18 | 33.30176 |
| ENSG00000170175.9 | CHRNB1 | 0.771826 | 2.003467 | 9.207009 | 6.44E-19 | 1.11E-17 | 31.62102 |
| ENSG00000138798.10 | EGF | 0.771576 | 1.105005 | 6.933434 | 1.12E-11 | 1.06E-10 | 15.18988 |
| ENSG00000139146.12 | FAM60A | 0.771571 | 3.618628 | 7.538594 | 1.90E-13 | 2.12E-12 | 19.19887 |
| ENSG00000113249.11 | HAVCR1 | 0.77126 | 0.739434 | 5.962497 | 4.37E-09 | 3.19E-08 | 9.357744 |
| ENSG00000105677.10 | TMEM147 | 0.770665 | 5.286045 | 9.652746 | 1.64E-20 | 3.13E-19 | 35.25443 |
| ENSG00000273604.1 | C17orf96 | 0.770622 | 1.467693 | 8.868605 | 9.63E-18 | 1.52E-16 | 28.94757 |
| ENSG00000204713.9 | TRIM27 | 0.770044 | 3.5279 | 14.46206 | 1.42E-40 | 9.46E-39 | 81.12982 |
| ENSG00000102030.14 | NAA10 | 0.769668 | 3.085367 | 10.72969 | 1.41E-24 | 3.62E-23 | 44.52751 |
| ENSG00000100804.17 | PSMB5 | 0.769642 | 5.953321 | 10.05665 | 5.29E-22 | 1.14E-20 | 38.65267 |
| ENSG00000149541.8 | B3GAT3 | 0.769051 | 4.172156 | 9.976337 | 1.06E-21 | 2.22E-20 | 37.96913 |
| ENSG00000115368.8 | WDR75 | 0.768941 | 3.121631 | 14.0424 | 1.19E-38 | 7.29E-37 | 76.72788 |
| ENSG00000083307.9 | GRHL2 | 0.768683 | 3.244737 | 8.655232 | 5.10E-17 | 7.59E-16 | 27.30083 |
| ENSG00000006704.9 | GTF2IRD1 | 0.76865 | 2.560715 | 10.829 | 5.76E-25 | 1.52E-23 | 45.41592 |
| ENSG00000198331.9 | HYLS1 | 0.768504 | 1.603111 | 11.37512 | 3.81E-27 | 1.15E-25 | 50.39558 |
| ENSG00000103423.12 | DNAJA3 | 0.768499 | 3.232161 | 14.81587 | 3.26E-42 | 2.41E-40 | 84.88716 |
| ENSG00000135094.9 | SDS | 0.768292 | 1.809361 | 5.837245 | 8.94E-09 | 6.30E-08 | 8.661346 |
| ENSG00000169683.6 | LRRC45 | 0.768125 | 2.987962 | 8.895792 | 7.77E-18 | 1.24E-16 | 29.15958 |
| ENSG00000146701.10 | MDH2 | 0.76761 | 5.766225 | 11.16435 | 2.69E-26 | 7.69E-25 | 48.45512 |
| ENSG00000106565.16 | TMEM176B | 0.767219 | 5.677117 | 5.0719 | 5.34E-07 | 3.06E-06 | 4.694685 |
| ENSG00000197451.9 | HNRNPAB | 0.767125 | 5.943534 | 11.9735 | 1.31E-29 | 4.62E-28 | 56.02643 |
| ENSG00000143368.9 | SF3B4 | 0.766548 | 5.526996 | 10.55396 | 6.79E-24 | 1.66E-22 | 42.96883 |
| ENSG00000005075.14 | POLR2J | 0.766085 | 4.654238 | 9.759447 | 6.67E-21 | 1.32E-19 | 36.14255 |
| ENSG00000108671.8 | PSMD11 | 0.765962 | 4.115171 | 10.77947 | 9.01E-25 | 2.35E-23 | 44.97216 |
| ENSG00000128342.4 | LIF | 0.765717 | 2.890151 | 4.422583 | 1.17E-05 | 5.60E-05 | 1.729671 |
| ENSG00000171903.15 | CYP4F11 | 0.765193 | 1.149132 | 4.040416 | 6.07E-05 | 0.000261955 | 0.161638 |
| ENSG00000187642.8 | PERM1 | 0.76514 | 0.838675 | 11.07998 | 5.85E-26 | 1.64E-24 | 47.68483 |
| ENSG00000114859.13 | CLCN2 | 0.764921 | 1.483246 | 11.66389 | 2.52E-28 | 8.28E-27 | 53.09089 |
| ENSG00000214517.7 | PPME1 | 0.764489 | 3.159894 | 12.6292 | 2.16E-32 | 9.10E-31 | 62.3916 |
| ENSG00000160214.11 | RRP1 | 0.763596 | 3.095896 | 11.68114 | 2.14E-28 | 7.05E-27 | 53.25324 |
| ENSG00000087494.14 | PTHLH | 0.7635 | 1.04921 | 5.34126 | 1.34E-07 | 8.26E-07 | 6.033475 |
| ENSG00000096092.5 | TMEM14A | 0.763183 | 4.692395 | 8.351864 | 5.18E-16 | 7.14E-15 | 25.0127 |
| ENSG00000136943.9 | CTSV | 0.762769 | 1.128078 | 5.996687 | 3.59E-09 | 2.64E-08 | 9.55011 |
| ENSG00000143641.9 | GALNT2 | 0.76254 | 4.182739 | 9.689353 | 1.20E-20 | 2.33E-19 | 35.55834 |
| ENSG00000136810.11 | TXN | 0.762485 | 7.234624 | 6.978029 | 8.39E-12 | 8.01E-11 | 15.47574 |
| ENSG00000162458.11 | FBLIM1 | 0.761756 | 3.716609 | 7.757923 | 4.04E-14 | 4.75E-13 | 20.71971 |
| ENSG00000166140.16 | ZFYVE19 | 0.761533 | 3.001 | 10.49807 | 1.12E-23 | 2.69E-22 | 42.47669 |
| ENSG00000117616.16 | RSRP1 | 0.761174 | 2.831222 | 6.38302 | 3.62E-10 | 2.95E-09 | 11.7905 |
| ENSG00000168005.7 | C11orf84 | 0.761142 | 3.229259 | 9.010515 | 3.13E-18 | 5.14E-17 | 30.05957 |
| ENSG00000172244.7 | C5orf34 | 0.760986 | 1.087366 | 10.47992 | 1.31E-23 | 3.14E-22 | 42.31719 |
| ENSG00000137700.15 | SLC37A4 | 0.760916 | 2.674845 | 12.42448 | 1.63E-31 | 6.50E-30 | 60.38329 |
| ENSG00000140284.9 | SLC27A2 | 0.760282 | 2.173806 | 5.362503 | 1.20E-07 | 7.43E-07 | 6.141725 |
| ENSG00000162910.17 | MRPL55 | 0.760191 | 4.167274 | 9.430754 | 1.04E-19 | 1.89E-18 | 33.42923 |
| ENSG00000198930.11 | CSAG1 | 0.759635 | 0.711712 | 3.606841 | 0.000337323 | 0.001280106 | -1.45492 |
| ENSG00000133895.13 | MEN1 | 0.759033 | 3.502375 | 11.4347 | 2.18E-27 | 6.70E-26 | 50.94827 |
| ENSG00000167962.11 | ZNF598 | 0.758889 | 3.262397 | 9.775237 | 5.84E-21 | 1.16E-19 | 36.27457 |
| ENSG00000148335.13 | NTMT1 | 0.758556 | 2.765892 | 12.05197 | 6.15E-30 | 2.22E-28 | 56.77774 |
| ENSG00000110811.18 | P3H3 | 0.758448 | 2.428054 | 6.137664 | 1.57E-09 | 1.20E-08 | 10.35349 |
| ENSG00000129667.11 | RHBDF2 | 0.757987 | 2.929873 | 8.410253 | 3.33E-16 | 4.66E-15 | 25.44818 |
| ENSG00000179918.16 | SEPHS2 | 0.757844 | 5.289651 | 8.50462 | 1.63E-16 | 2.33E-15 | 26.15696 |
| ENSG00000117322.15 | CR2 | 0.757476 | 1.276408 | 4.886475 | 1.34E-06 | 7.27E-06 | 3.809852 |
| ENSG00000111845.4 | PAK1IP1 | 0.757257 | 3.610735 | 9.280447 | 3.55E-19 | 6.25E-18 | 32.21099 |
| ENSG00000160803.7 | UBQLN4 | 0.757146 | 4.09348 | 10.3951 | 2.77E-23 | 6.48E-22 | 41.57464 |
| ENSG00000106003.11 | LFNG | 0.756959 | 3.872116 | 4.938908 | 1.04E-06 | 5.71E-06 | 4.056994 |
| ENSG00000105767.2 | CADM4 | 0.756892 | 3.346353 | 6.007227 | 3.38E-09 | 2.49E-08 | 9.609607 |
| ENSG00000178882.12 | FAM101A | 0.756628 | 1.120978 | 5.914073 | 5.77E-09 | 4.16E-08 | 9.086958 |
| ENSG00000115241.10 | PPM1G | 0.756608 | 5.12679 | 12.46237 | 1.13E-31 | 4.51E-30 | 60.75366 |
| ENSG00000152082.12 | MZT2B | 0.756557 | 4.672595 | 8.331462 | 6.05E-16 | 8.28E-15 | 24.8611 |
| ENSG00000058262.8 | SEC61A1 | 0.756556 | 6.601729 | 12.80444 | 3.78E-33 | 1.67E-31 | 64.12515 |
| ENSG00000142252.9 | GEMIN7 | 0.75576 | 3.147607 | 10.73641 | 1.33E-24 | 3.41E-23 | 44.58745 |
| ENSG00000090447.10 | TFAP4 | 0.75561 | 1.508672 | 14.6072 | 3.04E-41 | 2.12E-39 | 82.66628 |
| ENSG00000125885.12 | MCM8 | 0.755302 | 1.563278 | 9.69603 | 1.14E-20 | 2.21E-19 | 35.61385 |
| ENSG00000164897.11 | TMUB1 | 0.755261 | 4.450391 | 8.780865 | 1.92E-17 | 2.96E-16 | 28.26673 |
| ENSG00000108448.19 | TRIM16L | 0.755099 | 1.676347 | 4.934218 | 1.06E-06 | 5.84E-06 | 4.034789 |
| ENSG00000116014.8 | KISS1R | 0.754975 | 0.698275 | 6.709796 | 4.73E-11 | 4.22E-10 | 13.77975 |
| ENSG00000141577.12 | CEP131 | 0.75451 | 2.613429 | 8.13953 | 2.53E-15 | 3.30E-14 | 23.44915 |
| ENSG00000133740.9 | E2F5 | 0.754411 | 1.777295 | 9.561426 | 3.51E-20 | 6.59E-19 | 34.49989 |
| ENSG00000104687.11 | GSR | 0.754121 | 4.991289 | 5.802261 | 1.09E-08 | 7.60E-08 | 8.469171 |
| ENSG00000162383.10 | SLC1A7 | 0.75333 | 1.408156 | 3.307181 | 0.001002108 | 0.003484565 | -2.46977 |
| ENSG00000113360.15 | DROSHA | 0.753218 | 3.18827 | 9.630265 | 1.98E-20 | 3.76E-19 | 35.0682 |
| ENSG00000071794.14 | HLTF | 0.752971 | 3.020308 | 8.12572 | 2.80E-15 | 3.64E-14 | 23.34856 |
| ENSG00000160284.13 | SPATC1L | 0.752881 | 1.974046 | 7.330761 | 7.94E-13 | 8.41E-12 | 17.79072 |
| ENSG00000181588.16 | MEX3D | 0.752784 | 3.25139 | 8.152866 | 2.29E-15 | 3.00E-14 | 23.54642 |
| ENSG00000101911.11 | PRPS2 | 0.752763 | 4.248425 | 9.538221 | 4.26E-20 | 7.96E-19 | 34.30898 |
| ENSG00000085760.13 | MTIF2 | 0.752748 | 3.503206 | 13.71244 | 3.68E-37 | 2.07E-35 | 73.31091 |
| ENSG00000125901.5 | MRPS26 | 0.752311 | 5.119744 | 7.853199 | 2.04E-14 | 2.47E-13 | 21.39133 |
| ENSG00000177455.10 | CD19 | 0.752126 | 1.149614 | 6.063762 | 2.43E-09 | 1.82E-08 | 9.9303 |
| ENSG00000181143.14 | MUC16 | 0.752075 | 0.885724 | 5.863455 | 7.71E-09 | 5.47E-08 | 8.805989 |
| ENSG00000175792.10 | RUVBL1 | 0.751899 | 3.113459 | 10.23624 | 1.12E-22 | 2.52E-21 | 40.19484 |
| ENSG00000174943.8 | KCTD13 | 0.751497 | 2.183814 | 10.62223 | 3.70E-24 | 9.20E-23 | 43.57231 |
| ENSG00000142185.15 | TRPM2 | 0.750601 | 1.652272 | 7.665361 | 7.79E-14 | 8.95E-13 | 20.07356 |
| ENSG00000147955.15 | SIGMAR1 | 0.750596 | 4.71727 | 10.24565 | 1.03E-22 | 2.33E-21 | 40.27613 |
| ENSG00000124635.8 | HIST1H2BJ | 0.750586 | 1.3269 | 5.438081 | 8.02E-08 | 5.09E-07 | 6.530012 |
| ENSG00000119408.15 | NEK6 | 0.750271 | 4.176363 | 9.842774 | 3.30E-21 | 6.71E-20 | 36.84095 |
| ENSG00000143493.11 | INTS7 | 0.749879 | 2.797743 | 11.75234 | 1.09E-28 | 3.64E-27 | 53.9248 |
| ENSG00000043514.14 | TRIT1 | 0.749458 | 3.175524 | 10.58125 | 5.33E-24 | 1.31E-22 | 43.20977 |
| ENSG00000128944.12 | KNSTRN | 0.749445 | 2.241171 | 8.30676 | 7.28E-16 | 9.90E-15 | 24.67793 |
| ENSG00000106211.8 | HSPB1 | 0.749333 | 7.854296 | 6.080323 | 2.21E-09 | 1.66E-08 | 10.02475 |
| ENSG00000065923.8 | SLC9A7 | 0.748925 | 1.862736 | 9.149187 | 1.03E-18 | 1.75E-17 | 31.15894 |
| ENSG00000174437.15 | ATP2A2 | 0.748489 | 4.853662 | 9.366658 | 1.75E-19 | 3.15E-18 | 32.90799 |
| ENSG00000139182.12 | CLSTN3 | 0.747993 | 3.695418 | 9.237039 | 5.05E-19 | 8.80E-18 | 31.86185 |
| ENSG00000146386.7 | ABRACL | 0.747989 | 4.780324 | 7.736365 | 4.71E-14 | 5.51E-13 | 20.56865 |
| ENSG00000234602.6 | MCIDAS | 0.747541 | 0.855839 | 7.548882 | 1.76E-13 | 1.98E-12 | 19.26942 |
| ENSG00000138448.10 | ITGAV | 0.747329 | 4.4289 | 6.649721 | 6.92E-11 | 6.07E-10 | 13.40766 |
| ENSG00000111445.12 | RFC5 | 0.746824 | 2.605578 | 9.53067 | 4.53E-20 | 8.46E-19 | 34.24693 |
| ENSG00000162078.10 | ZG16B | 0.746562 | 1.193443 | 4.677601 | 3.63E-06 | 1.87E-05 | 2.849462 |
| ENSG00000124772.10 | CPNE5 | 0.746494 | 1.53268 | 6.919349 | 1.23E-11 | 1.16E-10 | 15.09992 |
| ENSG00000048544.5 | MRPS10 | 0.74627 | 4.574763 | 10.47417 | 1.38E-23 | 3.30E-22 | 42.26678 |
| ENSG00000177542.9 | SLC25A22 | 0.745391 | 2.948497 | 9.682938 | 1.27E-20 | 2.46E-19 | 35.50502 |
| ENSG00000175216.13 | CKAP5 | 0.74534 | 3.632648 | 9.325446 | 2.46E-19 | 4.37E-18 | 32.57421 |
| ENSG00000129354.10 | AP1M2 | 0.745243 | 5.020072 | 8.384227 | 4.06E-16 | 5.64E-15 | 25.25378 |
| ENSG00000196812.4 | ZSCAN16 | 0.744908 | 2.610465 | 9.950781 | 1.31E-21 | 2.74E-20 | 37.75244 |
| ENSG00000096384.18 | HSP90AB1 | 0.744842 | 8.771738 | 10.41214 | 2.39E-23 | 5.61E-22 | 41.72348 |
| ENSG00000145912.7 | NHP2 | 0.744582 | 4.760272 | 10.12992 | 2.81E-22 | 6.17E-21 | 39.27954 |
| ENSG00000204371.10 | EHMT2 | 0.744535 | 3.435016 | 9.743645 | 7.63E-21 | 1.50E-19 | 36.01058 |
| ENSG00000170801.8 | HTRA3 | 0.744462 | 3.94655 | 4.427705 | 1.14E-05 | 5.48E-05 | 1.75159 |
| ENSG00000177685.15 | CRACR2B | 0.744313 | 3.022576 | 5.367568 | 1.17E-07 | 7.25E-07 | 6.167597 |
| ENSG00000174442.10 | ZWILCH | 0.743598 | 1.997799 | 10.63852 | 3.19E-24 | 7.97E-23 | 43.71673 |
| ENSG00000227507.2 | LTB | 0.742659 | 3.258212 | 4.621319 | 4.73E-06 | 2.39E-05 | 2.597317 |
| ENSG00000197713.13 | RPE | 0.742618 | 3.549149 | 12.14919 | 2.40E-30 | 8.81E-29 | 57.71255 |
| ENSG00000163931.14 | TKT | 0.742596 | 5.745582 | 7.669359 | 7.58E-14 | 8.71E-13 | 20.10133 |
| ENSG00000120334.14 | CENPL | 0.742477 | 1.408536 | 11.60094 | 4.57E-28 | 1.47E-26 | 52.49984 |
| ENSG00000117215.13 | PLA2G2D | 0.741564 | 1.681645 | 4.266008 | 2.33E-05 | 0.000106911 | 1.071176 |
| ENSG00000137812.18 | CASC5 | 0.741216 | 0.886668 | 10.44408 | 1.80E-23 | 4.28E-22 | 42.00297 |
| ENSG00000064787.11 | BCAS1 | 0.741011 | 1.448678 | 4.277918 | 2.21E-05 | 0.000101836 | 1.120484 |
| ENSG00000172590.17 | MRPL52 | 0.740373 | 3.617219 | 9.631491 | 1.96E-20 | 3.73E-19 | 35.07834 |
| ENSG00000198876.11 | DCAF12 | 0.740246 | 4.424044 | 9.727454 | 8.74E-21 | 1.71E-19 | 35.87553 |
| ENSG00000112290.11 | WASF1 | 0.740118 | 1.688778 | 7.684839 | 6.79E-14 | 7.84E-13 | 20.20901 |
| ENSG00000120254.14 | MTHFD1L | 0.740045 | 2.824928 | 10.50661 | 1.03E-23 | 2.50E-22 | 42.55172 |
| ENSG00000102144.12 | PGK1 | 0.739917 | 6.694477 | 7.341504 | 7.38E-13 | 7.83E-12 | 17.86272 |
| ENSG00000127423.9 | AUNIP | 0.739856 | 0.952858 | 9.244869 | 4.74E-19 | 8.27E-18 | 31.92474 |
| ENSG00000117360.11 | PRPF3 | 0.739715 | 3.436378 | 9.480466 | 6.87E-20 | 1.27E-18 | 33.83529 |
| ENSG00000101132.8 | PFDN4 | 0.739511 | 3.352121 | 8.709837 | 3.34E-17 | 5.05E-16 | 27.71934 |
| ENSG00000117597.16 | DIEXF | 0.739335 | 2.017979 | 13.11236 | 1.71E-34 | 8.20E-33 | 67.20295 |
| ENSG00000196866.2 | HIST1H2AD | 0.739055 | 0.824245 | 5.383797 | 1.07E-07 | 6.68E-07 | 6.250631 |
| ENSG00000139572.3 | GPR84 | 0.738666 | 1.142365 | 7.64806 | 8.80E-14 | 1.01E-12 | 19.95348 |
| ENSG00000001167.13 | NFYA | 0.737936 | 3.603236 | 10.43087 | 2.02E-23 | 4.79E-22 | 41.88729 |
| ENSG00000198931.9 | APRT | 0.73765 | 5.707065 | 8.625365 | 6.43E-17 | 9.49E-16 | 27.07276 |
| ENSG00000145354.8 | CISD2 | 0.737476 | 3.917272 | 12.59181 | 3.14E-32 | 1.30E-30 | 62.0234 |
| ENSG00000140650.10 | PMM2 | 0.737379 | 1.801378 | 14.31004 | 7.11E-40 | 4.56E-38 | 79.52823 |
| ENSG00000074181.7 | NOTCH3 | 0.736782 | 4.261705 | 6.154847 | 1.42E-09 | 1.09E-08 | 10.45253 |
| ENSG00000127561.13 | SYNGR3 | 0.736615 | 0.868019 | 7.136756 | 2.94E-12 | 2.93E-11 | 16.50568 |
| ENSG00000099899.13 | TRMT2A | 0.736548 | 3.185244 | 9.62864 | 2.00E-20 | 3.81E-19 | 35.05475 |
| ENSG00000068438.13 | FTSJ1 | 0.736424 | 3.96611 | 11.59995 | 4.61E-28 | 1.48E-26 | 52.49055 |
| ENSG00000084110.9 | HAL | 0.736289 | 1.071101 | 5.122087 | 4.15E-07 | 2.41E-06 | 4.939349 |
| ENSG00000134222.15 | PSRC1 | 0.736175 | 1.720737 | 8.060711 | 4.52E-15 | 5.78E-14 | 22.87684 |
| ENSG00000105486.12 | LIG1 | 0.736114 | 2.505271 | 10.01396 | 7.64E-22 | 1.62E-20 | 38.28882 |
| ENSG00000163535.16 | SGOL2 | 0.735953 | 1.307911 | 9.29962 | 3.03E-19 | 5.37E-18 | 32.3656 |
| ENSG00000204160.10 | ZDHHC18 | 0.735889 | 3.439876 | 10.61732 | 3.86E-24 | 9.61E-23 | 43.52881 |
| ENSG00000196230.11 | TUBB | 0.735618 | 7.498616 | 8.187393 | 1.78E-15 | 2.34E-14 | 23.79883 |
| ENSG00000184428.11 | TOP1MT | 0.735436 | 2.49029 | 9.492905 | 6.20E-20 | 1.15E-18 | 33.93713 |
| ENSG00000150756.12 | FAM173B | 0.735383 | 2.699062 | 10.69453 | 1.93E-24 | 4.90E-23 | 44.21425 |
| ENSG00000163156.10 | SCNM1 | 0.735124 | 3.302736 | 10.61211 | 4.05E-24 | 1.01E-22 | 43.4827 |
| ENSG00000074211.12 | PPP2R2C | 0.735112 | 0.712192 | 5.400768 | 9.78E-08 | 6.14E-07 | 6.337699 |
| ENSG00000115884.9 | SDC1 | 0.735072 | 7.262469 | 6.10068 | 1.96E-09 | 1.48E-08 | 10.14114 |
| ENSG00000143258.14 | USP21 | 0.734464 | 3.290554 | 11.50078 | 1.17E-27 | 3.66E-26 | 51.56331 |
| ENSG00000158055.14 | GRHL3 | 0.733937 | 0.764651 | 8.863325 | 1.00E-17 | 1.59E-16 | 28.90646 |
| ENSG00000156795.5 | WDYHV1 | 0.733936 | 2.702002 | 9.649459 | 1.68E-20 | 3.22E-19 | 35.22718 |
| ENSG00000116221.14 | MRPL37 | 0.733338 | 4.660796 | 12.33333 | 3.99E-31 | 1.54E-29 | 59.49523 |
| ENSG00000152926.13 | ZNF117 | 0.733337 | 2.245313 | 5.625391 | 2.91E-08 | 1.94E-07 | 7.513356 |
| ENSG00000130787.12 | HIP1R | 0.733308 | 3.161855 | 7.85413 | 2.03E-14 | 2.46E-13 | 21.39793 |
| ENSG00000073536.16 | NLE1 | 0.733207 | 2.144467 | 12.82825 | 2.98E-33 | 1.33E-31 | 64.36171 |
| ENSG00000088305.17 | DNMT3B | 0.733108 | 1.105095 | 7.929016 | 1.18E-14 | 1.46E-13 | 21.93049 |
| ENSG00000138442.8 | WDR12 | 0.732996 | 1.995084 | 12.77813 | 4.92E-33 | 2.17E-31 | 63.86408 |
| ENSG00000147592.7 | LACTB2 | 0.73269 | 4.022838 | 7.000281 | 7.25E-12 | 6.98E-11 | 15.61896 |
| ENSG00000225556.1 | C2CD4D | 0.732568 | 0.906353 | 8.786722 | 1.83E-17 | 2.84E-16 | 28.31202 |
| ENSG00000143742.11 | SRP9 | 0.732501 | 6.532461 | 10.11007 | 3.34E-22 | 7.26E-21 | 39.10943 |
| ENSG00000131584.17 | ACAP3 | 0.732134 | 2.444821 | 8.058389 | 4.60E-15 | 5.87E-14 | 22.86005 |
| ENSG00000136492.7 | BRIP1 | 0.731972 | 0.856264 | 10.91727 | 2.59E-25 | 7.00E-24 | 46.21006 |
| ENSG00000141101.11 | NOB1 | 0.731895 | 4.336315 | 11.58451 | 5.34E-28 | 1.70E-26 | 52.34588 |
| ENSG00000074201.7 | CLNS1A | 0.731705 | 3.980817 | 12.32207 | 4.46E-31 | 1.71E-29 | 59.38574 |
| ENSG00000115107.18 | STEAP3 | 0.73164 | 3.990206 | 5.885008 | 6.82E-09 | 4.87E-08 | 8.925366 |
| ENSG00000086548.8 | CEACAM6 | 0.730984 | 8.219457 | 2.627473 | 0.008835176 | 0.024854789 | -4.45615 |
| ENSG00000166451.12 | CENPN | 0.730665 | 1.827665 | 8.482027 | 1.93E-16 | 2.75E-15 | 25.98671 |
| ENSG00000168785.6 | TSPAN5 | 0.730213 | 2.219911 | 6.874228 | 1.65E-11 | 1.53E-10 | 14.81275 |
| ENSG00000108679.11 | LGALS3BP | 0.729984 | 8.188308 | 6.288015 | 6.43E-10 | 5.11E-09 | 11.22826 |
| ENSG00000185156.5 | MFSD6L | 0.729892 | 1.308176 | 5.113959 | 4.32E-07 | 2.50E-06 | 4.899573 |
| ENSG00000196305.16 | IARS | 0.729785 | 4.051017 | 10.81635 | 6.46E-25 | 1.70E-23 | 45.30247 |
| ENSG00000121578.11 | B4GALT4 | 0.729437 | 2.355971 | 8.819207 | 1.42E-17 | 2.22E-16 | 28.56363 |
| ENSG00000077232.15 | DNAJC10 | 0.729337 | 2.936276 | 9.693169 | 1.17E-20 | 2.26E-19 | 35.59006 |
| ENSG00000119772.15 | DNMT3A | 0.729183 | 2.330802 | 9.51588 | 5.12E-20 | 9.54E-19 | 34.12549 |
| ENSG00000213190.3 | MLLT11 | 0.728769 | 1.717658 | 5.293727 | 1.72E-07 | 1.05E-06 | 5.792657 |
| ENSG00000166226.11 | CCT2 | 0.728578 | 5.004144 | 7.157715 | 2.55E-12 | 2.56E-11 | 16.64312 |
| ENSG00000112877.7 | CEP72 | 0.728337 | 1.609983 | 9.441548 | 9.47E-20 | 1.73E-18 | 33.51726 |
| ENSG00000163950.11 | SLBP | 0.72825 | 4.76302 | 10.70694 | 1.73E-24 | 4.41E-23 | 44.32473 |
| ENSG00000143183.15 | TMCO1 | 0.727939 | 4.526117 | 10.75876 | 1.09E-24 | 2.81E-23 | 44.78701 |
| ENSG00000077312.7 | SNRPA | 0.727934 | 4.551632 | 12.88347 | 1.72E-33 | 7.72E-32 | 64.91135 |
| ENSG00000143297.17 | FCRL5 | 0.727765 | 0.8935 | 7.242745 | 1.44E-12 | 1.48E-11 | 17.20418 |
| ENSG00000115275.10 | MOGS | 0.727239 | 4.745323 | 12.25497 | 8.58E-31 | 3.24E-29 | 58.73466 |
| ENSG00000139410.13 | SDSL | 0.726585 | 2.873469 | 7.161791 | 2.49E-12 | 2.49E-11 | 16.6699 |
| ENSG00000204315.3 | FKBPL | 0.726384 | 2.70379 | 10.93181 | 2.27E-25 | 6.15E-24 | 46.34127 |
| ENSG00000069509.5 | FUNDC1 | 0.726112 | 3.563111 | 10.58179 | 5.30E-24 | 1.31E-22 | 43.21453 |
| ENSG00000146826.13 | C7orf43 | 0.725986 | 2.898228 | 9.716502 | 9.59E-21 | 1.87E-19 | 35.78426 |
| ENSG00000130935.8 | NOL11 | 0.725935 | 3.536697 | 12.03128 | 7.52E-30 | 2.69E-28 | 56.57931 |
| ENSG00000156738.16 | MS4A1 | 0.725865 | 1.374277 | 4.420248 | 1.18E-05 | 5.65E-05 | 1.719689 |
| ENSG00000090924.13 | PLEKHG2 | 0.725258 | 2.700853 | 5.883114 | 6.89E-09 | 4.92E-08 | 8.914856 |
| ENSG00000188158.13 | NHS | 0.725178 | 1.188011 | 7.546029 | 1.80E-13 | 2.01E-12 | 19.24984 |
| ENSG00000091490.9 | SEL1L3 | 0.72507 | 4.7233 | 7.94847 | 1.03E-14 | 1.27E-13 | 22.0695 |
| ENSG00000197261.10 | C6orf141 | 0.724617 | 1.137284 | 6.204019 | 1.06E-09 | 8.28E-09 | 10.73728 |
| ENSG00000115307.15 | AUP1 | 0.724092 | 5.827919 | 11.99622 | 1.05E-29 | 3.74E-28 | 56.24365 |
| ENSG00000146757.12 | ZNF92 | 0.723856 | 2.092033 | 10.24632 | 1.02E-22 | 2.32E-21 | 40.28197 |
| ENSG00000122218.13 | COPA | 0.723682 | 5.758184 | 11.17033 | 2.55E-26 | 7.30E-25 | 48.50984 |
| ENSG00000108424.8 | KPNB1 | 0.72361 | 5.108094 | 10.40848 | 2.46E-23 | 5.78E-22 | 41.69152 |
| ENSG00000124641.13 | MED20 | 0.723429 | 3.00567 | 10.71756 | 1.57E-24 | 4.02E-23 | 44.41938 |
| ENSG00000176731.10 | C8orf59 | 0.72334 | 3.601307 | 8.636615 | 5.89E-17 | 8.72E-16 | 27.1586 |
| ENSG00000162069.13 | CCDC64B | 0.723252 | 2.601921 | 5.892085 | 6.55E-09 | 4.69E-08 | 8.964644 |
| ENSG00000116586.10 | LAMTOR2 | 0.723086 | 5.115071 | 9.80982 | 4.36E-21 | 8.79E-20 | 36.56424 |
| ENSG00000160877.5 | NACC1 | 0.723038 | 4.157846 | 9.174836 | 8.36E-19 | 1.43E-17 | 31.36364 |
| ENSG00000087087.17 | SRRT | 0.722928 | 4.434321 | 10.77658 | 9.25E-25 | 2.40E-23 | 44.94631 |
| ENSG00000082458.10 | DLG3 | 0.722867 | 3.167841 | 10.5729 | 5.74E-24 | 1.41E-22 | 43.136 |
| ENSG00000160181.7 | TFF2 | 0.722444 | 0.671159 | 2.766869 | 0.005844606 | 0.017185188 | -4.08481 |
| ENSG00000163170.10 | BOLA3 | 0.721836 | 2.333023 | 9.869419 | 2.63E-21 | 5.38E-20 | 37.06516 |
| ENSG00000110107.7 | PRPF19 | 0.721833 | 5.408939 | 12.51721 | 6.55E-32 | 2.66E-30 | 61.29068 |
| ENSG00000156697.11 | UTP14A | 0.721742 | 2.865871 | 13.21806 | 5.86E-35 | 2.87E-33 | 68.26849 |
| ENSG00000168894.8 | RNF181 | 0.721578 | 5.94698 | 9.050272 | 2.28E-18 | 3.78E-17 | 30.37347 |
| ENSG00000054179.10 | ENTPD2 | 0.721084 | 1.059871 | 6.396257 | 3.34E-10 | 2.73E-09 | 11.86942 |
| ENSG00000167088.9 | SNRPD1 | 0.719729 | 3.261029 | 9.467405 | 7.65E-20 | 1.41E-18 | 33.72845 |
| ENSG00000123843.11 | C4BPB | 0.719382 | 1.383859 | 4.263963 | 2.35E-05 | 0.00010779 | 1.062725 |
| ENSG00000089157.14 | RPLP0 | 0.71922 | 8.218499 | 8.141187 | 2.50E-15 | 3.26E-14 | 23.46122 |
| ENSG00000164379.5 | FOXQ1 | 0.719062 | 2.974057 | 3.232359 | 0.001299065 | 0.004415114 | -2.70998 |
| ENSG00000161980.5 | POLR3K | 0.718631 | 3.314608 | 10.76626 | 1.01E-24 | 2.63E-23 | 44.85402 |
| ENSG00000107937.17 | GTPBP4 | 0.718349 | 3.289781 | 9.305883 | 2.88E-19 | 5.11E-18 | 32.41615 |
| ENSG00000204866.7 | IGFL2 | 0.717981 | 0.734119 | 6.500908 | 1.75E-10 | 1.48E-09 | 12.4983 |
| ENSG00000089199.8 | CHGB | 0.717853 | 0.842925 | 3.403433 | 0.000712468 | 0.002544564 | -2.153 |
| ENSG00000154582.15 | TCEB1 | 0.717821 | 3.518899 | 8.854667 | 1.08E-17 | 1.69E-16 | 28.83908 |
| ENSG00000164305.16 | CASP3 | 0.717104 | 3.788842 | 11.42386 | 2.41E-27 | 7.39E-26 | 50.84758 |
| ENSG00000064195.7 | DLX3 | 0.717024 | 1.021695 | 6.158395 | 1.39E-09 | 1.07E-08 | 10.47301 |
| ENSG00000115128.6 | SF3B6 | 0.716806 | 5.694963 | 11.32293 | 6.19E-27 | 1.85E-25 | 49.91294 |
| ENSG00000277363.3 | SRCIN1 | 0.71654 | 1.069719 | 7.819275 | 2.61E-14 | 3.12E-13 | 21.15144 |
| ENSG00000175600.14 | SUGCT | 0.716385 | 1.102319 | 7.301647 | 9.68E-13 | 1.02E-11 | 17.59605 |
| ENSG00000151135.8 | TMEM263 | 0.715549 | 3.959851 | 9.095748 | 1.58E-18 | 2.65E-17 | 30.73379 |
| ENSG00000136463.7 | TACO1 | 0.715458 | 3.367259 | 12.57179 | 3.82E-32 | 1.58E-30 | 61.82649 |
| ENSG00000169245.5 | CXCL10 | 0.715412 | 4.178356 | 3.191909 | 0.001491681 | 0.005003402 | -2.83763 |
| ENSG00000167920.7 | TMEM99 | 0.71522 | 2.709547 | 7.341583 | 7.38E-13 | 7.83E-12 | 17.86324 |
| ENSG00000169962.4 | TAS1R3 | 0.71512 | 1.014867 | 6.822875 | 2.30E-11 | 2.11E-10 | 14.48786 |
| ENSG00000141198.12 | TOM1L1 | 0.715062 | 2.679852 | 9.590687 | 2.75E-20 | 5.21E-19 | 34.7411 |
| ENSG00000198443.6 | KRTAP4-1 | 0.714875 | 0.648919 | 6.357348 | 4.23E-10 | 3.43E-09 | 11.63785 |
| ENSG00000198858.8 | R3HDM4 | 0.714779 | 4.91102 | 10.19189 | 1.64E-22 | 3.67E-21 | 39.81226 |
| ENSG00000114923.15 | SLC4A3 | 0.714533 | 1.151513 | 7.033526 | 5.83E-12 | 5.66E-11 | 15.83364 |
| ENSG00000023839.9 | ABCC2 | 0.714399 | 0.803807 | 3.638214 | 0.000299611 | 0.00114947 | -1.34381 |
| ENSG00000181885.17 | CLDN7 | 0.714188 | 5.848191 | 6.774474 | 3.14E-11 | 2.84E-10 | 14.18354 |
| ENSG00000215182.8 | MUC5AC | 0.713769 | 1.156536 | 2.576907 | 0.010220719 | 0.028294568 | -4.58623 |
| ENSG00000065361.13 | ERBB3 | 0.713272 | 4.756144 | 5.885077 | 6.81E-09 | 4.87E-08 | 8.925748 |
| ENSG00000053108.15 | FSTL4 | 0.712822 | 1.210002 | 4.301046 | 2.00E-05 | 9.26E-05 | 1.216605 |
| ENSG00000145623.11 | OSMR | 0.712792 | 4.690607 | 5.539688 | 4.64E-08 | 3.02E-07 | 7.059723 |
| ENSG00000165271.15 | NOL6 | 0.712105 | 3.692281 | 9.926961 | 1.61E-21 | 3.35E-20 | 37.55081 |
| ENSG00000174705.10 | SH3PXD2B | 0.711133 | 3.085291 | 6.537699 | 1.40E-10 | 1.19E-09 | 12.72147 |
| ENSG00000162396.5 | PARS2 | 0.711069 | 1.921538 | 12.97402 | 6.91E-34 | 3.18E-32 | 65.81526 |
| ENSG00000159199.12 | ATP5G1 | 0.711027 | 4.250134 | 8.395401 | 3.73E-16 | 5.20E-15 | 25.33718 |
| ENSG00000134825.12 | TMEM258 | 0.711022 | 4.637017 | 11.48101 | 1.41E-27 | 4.38E-26 | 51.37904 |
| ENSG00000160193.10 | WDR4 | 0.710986 | 2.257269 | 10.6454 | 3.00E-24 | 7.51E-23 | 43.77775 |
| ENSG00000182446.12 | NPLOC4 | 0.70996 | 4.143585 | 11.53644 | 8.40E-28 | 2.65E-26 | 51.89613 |
| ENSG00000149923.12 | PPP4C | 0.709924 | 5.344023 | 10.60609 | 4.27E-24 | 1.06E-22 | 43.4294 |
| ENSG00000147140.14 | NONO | 0.709844 | 6.069262 | 11.39299 | 3.22E-27 | 9.78E-26 | 50.56119 |
| ENSG00000278615.3 | C11orf98 | 0.709779 | 2.418921 | 10.1107 | 3.32E-22 | 7.23E-21 | 39.11481 |
| ENSG00000179526.15 | SHARPIN | 0.709516 | 4.7901 | 8.784528 | 1.86E-17 | 2.89E-16 | 28.29505 |
| ENSG00000011478.10 | QPCTL | 0.709266 | 2.824469 | 9.775576 | 5.82E-21 | 1.16E-19 | 36.2774 |
| ENSG00000012048.18 | BRCA1 | 0.709177 | 1.384555 | 8.307279 | 7.25E-16 | 9.86E-15 | 24.68178 |
| ENSG00000074582.11 | BCS1L | 0.708828 | 2.759954 | 13.22878 | 5.26E-35 | 2.58E-33 | 68.37681 |
| ENSG00000115761.14 | NOL10 | 0.708787 | 3.265336 | 13.08263 | 2.31E-34 | 1.09E-32 | 66.90409 |
| ENSG00000198088.9 | NUP62CL | 0.708376 | 1.324883 | 8.107654 | 3.20E-15 | 4.14E-14 | 23.21717 |
| ENSG00000120370.11 | GORAB | 0.708208 | 2.371649 | 11.30416 | 7.37E-27 | 2.20E-25 | 49.73978 |
| ENSG00000163347.5 | CLDN1 | 0.707839 | 3.985613 | 3.660643 | 0.000275118 | 0.001062715 | -1.26381 |
| ENSG00000186395.6 | KRT10 | 0.707454 | 3.631096 | 7.877124 | 1.72E-14 | 2.09E-13 | 21.56102 |
| ENSG00000122515.13 | ZMIZ2 | 0.707244 | 4.35015 | 7.703662 | 5.94E-14 | 6.90E-13 | 20.34017 |
| ENSG00000100373.8 | UPK3A | 0.707029 | 0.854682 | 5.406187 | 9.50E-08 | 5.98E-07 | 6.365556 |
| ENSG00000178222.11 | RNF212 | 0.706963 | 1.404384 | 5.23809 | 2.29E-07 | 1.38E-06 | 5.513263 |
| ENSG00000110074.9 | FOXRED1 | 0.70632 | 2.412821 | 10.70505 | 1.76E-24 | 4.47E-23 | 44.30793 |
| ENSG00000181610.11 | MRPS23 | 0.706247 | 3.298989 | 9.474903 | 7.19E-20 | 1.33E-18 | 33.78977 |
| ENSG00000155324.8 | GRAMD3 | 0.706121 | 2.773391 | 8.357943 | 4.95E-16 | 6.83E-15 | 25.05793 |
| ENSG00000154511.10 | FAM69A | 0.705933 | 2.475383 | 9.225591 | 5.54E-19 | 9.62E-18 | 31.76998 |
| ENSG00000010438.15 | PRSS3 | 0.705686 | 0.719013 | 4.345879 | 1.64E-05 | 7.71E-05 | 1.404312 |
| ENSG00000231500.5 | RPS18 | 0.705565 | 9.426545 | 6.180523 | 1.22E-09 | 9.45E-09 | 10.60096 |
| ENSG00000167112.9 | TRUB2 | 0.705548 | 2.832739 | 12.34355 | 3.61E-31 | 1.40E-29 | 59.59462 |
| ENSG00000178966.14 | RMI1 | 0.705484 | 2.315142 | 10.4245 | 2.14E-23 | 5.05E-22 | 41.83157 |
| ENSG00000140983.12 | RHOT2 | 0.704891 | 3.855189 | 8.544676 | 1.20E-16 | 1.73E-15 | 26.45967 |
| ENSG00000130487.5 | KLHDC7B | 0.704883 | 1.318421 | 5.75619 | 1.41E-08 | 9.71E-08 | 8.217663 |
| ENSG00000124228.13 | DDX27 | 0.704768 | 3.754823 | 10.528 | 8.56E-24 | 2.08E-22 | 42.74001 |
| ENSG00000161016.14 | RPL8 | 0.704748 | 9.437783 | 6.984108 | 8.06E-12 | 7.72E-11 | 15.51483 |
| ENSG00000100462.14 | PRMT5 | 0.704519 | 3.975813 | 9.188895 | 7.46E-19 | 1.28E-17 | 31.47603 |
| ENSG00000185504.15 | C17orf70 | 0.704462 | 3.266903 | 10.08427 | 4.17E-22 | 9.02E-21 | 38.88857 |
| ENSG00000124429.16 | POF1B | 0.70434 | 1.626655 | 4.27398 | 2.25E-05 | 0.000103485 | 1.104167 |
| ENSG00000185515.13 | BRCC3 | 0.704277 | 3.344032 | 10.78068 | 8.91E-25 | 2.32E-23 | 44.98292 |
| ENSG00000106009.14 | BRAT1 | 0.70407 | 3.252917 | 8.956263 | 4.81E-18 | 7.81E-17 | 29.63289 |
| ENSG00000198734.9 | F5 | 0.703809 | 1.184961 | 4.55229 | 6.50E-06 | 3.23E-05 | 2.291931 |
| ENSG00000124733.3 | MEA1 | 0.703281 | 5.798286 | 10.64348 | 3.06E-24 | 7.63E-23 | 43.76068 |
| ENSG00000128965.10 | CHAC1 | 0.703175 | 1.650116 | 7.007923 | 6.90E-12 | 6.65E-11 | 15.66823 |
| ENSG00000141543.8 | EIF4A3 | 0.702248 | 4.228027 | 9.775488 | 5.83E-21 | 1.16E-19 | 36.27667 |
| ENSG00000125877.11 | ITPA | 0.701856 | 4.448394 | 9.405279 | 1.28E-19 | 2.31E-18 | 33.22175 |
| ENSG00000168268.9 | NT5DC2 | 0.701829 | 3.6877 | 7.843387 | 2.19E-14 | 2.64E-13 | 21.32186 |
| ENSG00000115290.8 | GRB14 | 0.701457 | 1.64143 | 5.06386 | 5.57E-07 | 3.18E-06 | 4.655694 |
| ENSG00000196247.10 | ZNF107 | 0.701336 | 1.532393 | 9.991392 | 9.27E-22 | 1.96E-20 | 38.09697 |
| ENSG00000116212.13 | LRRC42 | 0.701006 | 3.784119 | 9.570145 | 3.26E-20 | 6.15E-19 | 34.57171 |
| ENSG00000157181.13 | C1orf27 | 0.700853 | 3.46968 | 10.68332 | 2.14E-24 | 5.40E-23 | 44.11455 |
| ENSG00000145536.14 | ADAMTS16 | 0.7006 | 0.856711 | 7.571666 | 1.50E-13 | 1.69E-12 | 19.42592 |
| ENSG00000146955.9 | RAB19 | 0.700483 | 1.246595 | 9.689005 | 1.21E-20 | 2.34E-19 | 35.55544 |
| ENSG00000181789.13 | COPG1 | 0.700382 | 5.951899 | 11.58749 | 5.19E-28 | 1.66E-26 | 52.37374 |
| ENSG00000106153.12 | CHCHD2 | 0.700375 | 7.43542 | 7.263625 | 1.25E-12 | 1.30E-11 | 17.34279 |
| ENSG00000239887.4 | C1orf226 | 0.700368 | 2.410114 | 6.628844 | 7.89E-11 | 6.88E-10 | 13.27902 |
| ENSG00000143633.11 | C1orf131 | 0.700025 | 1.765972 | 15.50424 | 1.89E-45 | 1.65E-43 | 92.30928 |
| ENSG00000172292.13 | CERS6 | 0.699389 | 2.388193 | 8.231176 | 1.28E-15 | 1.71E-14 | 24.12012 |
| ENSG00000106086.17 | PLEKHA8 | 0.699238 | 1.494621 | 11.95825 | 1.52E-29 | 5.33E-28 | 55.8808 |
| ENSG00000124279.10 | FASTKD3 | 0.699211 | 2.410576 | 9.846573 | 3.19E-21 | 6.50E-20 | 36.87289 |
| ENSG00000106638.14 | TBL2 | 0.698837 | 3.657777 | 11.45258 | 1.85E-27 | 5.69E-26 | 51.11447 |
| ENSG00000183155.4 | RABIF | 0.69854 | 3.257963 | 13.6048 | 1.12E-36 | 6.16E-35 | 72.20494 |
| ENSG00000139343.9 | SNRPF | 0.698056 | 3.700001 | 8.592233 | 8.30E-17 | 1.22E-15 | 26.82048 |
| ENSG00000089012.13 | SIRPG | 0.697944 | 1.25314 | 7.157066 | 2.57E-12 | 2.57E-11 | 16.63886 |
| ENSG00000180263.12 | FGD6 | 0.69771 | 2.560951 | 7.352248 | 6.86E-13 | 7.30E-12 | 17.9348 |
| ENSG00000002933.6 | TMEM176A | 0.697346 | 4.099989 | 4.534896 | 7.04E-06 | 3.48E-05 | 2.215651 |
| ENSG00000111364.14 | DDX55 | 0.697341 | 2.26684 | 10.93843 | 2.13E-25 | 5.80E-24 | 46.40106 |
| ENSG00000139726.9 | DENR | 0.697282 | 4.375138 | 11.17809 | 2.37E-26 | 6.81E-25 | 48.58091 |
| ENSG00000118894.13 | EEF2KMT | 0.697254 | 2.060514 | 13.92973 | 3.85E-38 | 2.28E-36 | 75.55659 |
| ENSG00000260428.2 | SCX | 0.697189 | 1.335111 | 5.999893 | 3.52E-09 | 2.59E-08 | 9.568198 |
| ENSG00000106460.17 | TMEM106B | 0.697095 | 3.067125 | 8.03882 | 5.31E-15 | 6.74E-14 | 22.71867 |
| ENSG00000136425.11 | CIB2 | 0.697032 | 1.722046 | 6.074268 | 2.29E-09 | 1.72E-08 | 9.990189 |
| ENSG00000131944.8 | C19orf40 | 0.696901 | 1.521741 | 9.332043 | 2.33E-19 | 4.15E-18 | 32.62757 |
| ENSG00000213585.9 | VDAC1 | 0.696745 | 5.7154 | 9.774107 | 5.90E-21 | 1.17E-19 | 36.26511 |
| ENSG00000172432.17 | GTPBP2 | 0.696708 | 4.033483 | 10.43884 | 1.89E-23 | 4.48E-22 | 41.95706 |
| ENSG00000156873.14 | PHKG2 | 0.696646 | 2.475898 | 10.60846 | 4.18E-24 | 1.04E-22 | 43.4504 |
| ENSG00000173065.12 | FAM222B | 0.696571 | 3.145143 | 10.08417 | 4.17E-22 | 9.03E-21 | 38.88773 |
| ENSG00000278677.1 | HIST1H2AM | 0.696344 | 0.697156 | 6.507161 | 1.69E-10 | 1.42E-09 | 12.53615 |
| ENSG00000105438.7 | KDELR1 | 0.696195 | 6.687122 | 12.36062 | 3.06E-31 | 1.19E-29 | 59.76073 |
| ENSG00000183386.8 | FHL3 | 0.696098 | 3.504906 | 8.622881 | 6.55E-17 | 9.66E-16 | 27.05382 |
| ENSG00000181392.13 | SYNE4 | 0.696069 | 2.492738 | 6.351529 | 4.38E-10 | 3.54E-09 | 11.60333 |
| ENSG00000128040.9 | SPINK2 | 0.696055 | 0.891806 | 5.68944 | 2.05E-08 | 1.39E-07 | 7.856437 |
| ENSG00000142197.11 | DOPEY2 | 0.695864 | 2.552855 | 9.234066 | 5.17E-19 | 9.00E-18 | 31.83799 |
| ENSG00000116898.10 | MRPS15 | 0.695804 | 4.501294 | 8.72711 | 2.92E-17 | 4.44E-16 | 27.85215 |
| ENSG00000196290.13 | NIF3L1 | 0.695689 | 3.714929 | 12.122 | 3.13E-30 | 1.14E-28 | 57.45063 |
| ENSG00000015532.8 | XYLT2 | 0.695606 | 3.226055 | 10.40547 | 2.53E-23 | 5.93E-22 | 41.66523 |
| ENSG00000136908.16 | DPM2 | 0.695545 | 4.293669 | 9.873801 | 2.53E-21 | 5.19E-20 | 37.10207 |
| ENSG00000197345.11 | MRPL21 | 0.695456 | 3.449618 | 9.693301 | 1.16E-20 | 2.26E-19 | 35.59116 |
| ENSG00000004142.10 | POLDIP2 | 0.695396 | 5.755699 | 12.8325 | 2.86E-33 | 1.27E-31 | 64.40402 |
| ENSG00000175087.8 | PDIK1L | 0.695221 | 2.381577 | 9.903834 | 1.96E-21 | 4.05E-20 | 37.35538 |
| ENSG00000134339.7 | SAA2 | 0.695056 | 1.271444 | 3.92844 | 9.61E-05 | 0.000401314 | -0.27251 |
| ENSG00000100883.10 | SRP54 | 0.694828 | 4.390283 | 8.336233 | 5.83E-16 | 8.00E-15 | 24.89653 |
| ENSG00000143643.11 | TTC13 | 0.694784 | 2.630128 | 10.11053 | 3.32E-22 | 7.24E-21 | 39.11335 |
| ENSG00000172239.12 | PAIP1 | 0.693829 | 4.286914 | 9.408932 | 1.24E-19 | 2.25E-18 | 33.25147 |
| ENSG00000171425.8 | ZNF581 | 0.693341 | 3.415352 | 9.051207 | 2.26E-18 | 3.75E-17 | 30.38087 |
| ENSG00000143457.9 | GOLPH3L | 0.693325 | 4.126046 | 8.591221 | 8.37E-17 | 1.22E-15 | 26.81279 |
| ENSG00000122691.11 | TWIST1 | 0.692972 | 0.906716 | 5.85439 | 8.11E-09 | 5.74E-08 | 8.755899 |
| ENSG00000065600.11 | TMEM206 | 0.692964 | 2.305051 | 9.090898 | 1.64E-18 | 2.75E-17 | 30.69531 |
| ENSG00000138182.13 | KIF20B | 0.692844 | 1.518263 | 8.922117 | 6.31E-18 | 1.02E-16 | 29.36533 |
| ENSG00000162702.7 | ZNF281 | 0.692626 | 3.403225 | 8.189597 | 1.75E-15 | 2.31E-14 | 23.81497 |
| ENSG00000104368.16 | PLAT | 0.692203 | 3.476696 | 3.193594 | 0.001483154 | 0.004977563 | -2.83234 |
| ENSG00000198563.12 | DDX39B | 0.692101 | 3.693262 | 5.638602 | 2.71E-08 | 1.81E-07 | 7.583833 |
| ENSG00000175305.15 | CCNE2 | 0.691978 | 0.999764 | 8.920103 | 6.41E-18 | 1.03E-16 | 29.34957 |
| ENSG00000158477.6 | CD1A | 0.691892 | 1.598287 | 3.570846 | 0.000386064 | 0.001450598 | -1.58128 |
| ENSG00000099377.12 | HSD3B7 | 0.691875 | 3.170844 | 7.407394 | 4.70E-13 | 5.07E-12 | 18.30617 |
| ENSG00000123427.14 | METTL21B | 0.691852 | 2.454292 | 9.198404 | 6.91E-19 | 1.19E-17 | 31.55212 |
| ENSG00000116957.11 | TBCE | 0.691568 | 3.634799 | 10.23144 | 1.16E-22 | 2.62E-21 | 40.15337 |
| ENSG00000185875.11 | THNSL1 | 0.690975 | 1.871831 | 10.38399 | 3.06E-23 | 7.13E-22 | 41.47764 |
| ENSG00000273045.4 | C2orf15 | 0.690615 | 1.526457 | 9.739495 | 7.90E-21 | 1.55E-19 | 35.97595 |
| ENSG00000101276.13 | SLC52A3 | 0.69059 | 1.805966 | 6.689323 | 5.39E-11 | 4.78E-10 | 13.65262 |
| ENSG00000197965.10 | MPZL1 | 0.690511 | 4.70225 | 11.71953 | 1.49E-28 | 4.94E-27 | 53.61498 |
| ENSG00000170638.8 | TRABD | 0.690499 | 3.984717 | 8.763411 | 2.20E-17 | 3.38E-16 | 28.1319 |
| ENSG00000102172.14 | SMS | 0.690393 | 5.109388 | 8.202845 | 1.58E-15 | 2.10E-14 | 23.91206 |
| ENSG00000109685.16 | WHSC1 | 0.690286 | 2.332729 | 8.762462 | 2.22E-17 | 3.40E-16 | 28.12458 |
| ENSG00000163814.6 | CDCP1 | 0.690172 | 3.360717 | 4.998808 | 7.70E-07 | 4.31E-06 | 4.342292 |
| ENSG00000171551.10 | ECEL1 | 0.6898 | 0.6899 | 4.863024 | 1.50E-06 | 8.08E-06 | 3.7001 |
| ENSG00000103512.13 | NOMO1 | 0.689743 | 3.329865 | 10.07392 | 4.56E-22 | 9.84E-21 | 38.80017 |
| ENSG00000082898.15 | XPO1 | 0.689368 | 4.00508 | 11.40232 | 2.95E-27 | 9.00E-26 | 50.64766 |
| ENSG00000147813.14 | NAPRT | 0.689189 | 3.978098 | 5.246874 | 2.19E-07 | 1.32E-06 | 5.557193 |
| ENSG00000105464.3 | GRIN2D | 0.688847 | 0.926336 | 5.665285 | 2.34E-08 | 1.58E-07 | 7.726642 |
| ENSG00000132780.15 | NASP | 0.688011 | 3.640602 | 8.182385 | 1.84E-15 | 2.43E-14 | 23.76217 |
| ENSG00000126456.14 | IRF3 | 0.687925 | 4.08876 | 9.897676 | 2.07E-21 | 4.26E-20 | 37.3034 |
| ENSG00000213551.4 | DNAJC9 | 0.687706 | 2.32758 | 10.96523 | 1.67E-25 | 4.56E-24 | 46.64328 |
| ENSG00000143748.16 | NVL | 0.68749 | 2.722651 | 12.43761 | 1.44E-31 | 5.73E-30 | 60.51155 |
| ENSG00000265354.3 | TIMM23 | 0.687244 | 5.279891 | 11.30745 | 7.15E-27 | 2.13E-25 | 49.7701 |
| ENSG00000135919.11 | SERPINE2 | 0.687144 | 1.823832 | 5.342773 | 1.33E-07 | 8.20E-07 | 6.041171 |
| ENSG00000177595.16 | PIDD1 | 0.686872 | 1.883137 | 7.996054 | 7.26E-15 | 9.11E-14 | 22.41067 |
| ENSG00000100575.12 | TIMM9 | 0.686651 | 3.923078 | 9.889985 | 2.21E-21 | 4.54E-20 | 37.23851 |
| ENSG00000117395.9 | EBNA1BP2 | 0.686586 | 4.088442 | 11.28693 | 8.65E-27 | 2.56E-25 | 49.58092 |
| ENSG00000092470.10 | WDR76 | 0.686529 | 1.721866 | 8.365372 | 4.68E-16 | 6.47E-15 | 25.11324 |
| ENSG00000159212.11 | CLIC6 | 0.68646 | 4.467292 | 2.804284 | 0.005215821 | 0.015509759 | -3.98196 |
| ENSG00000133048.11 | CHI3L1 | 0.686417 | 4.685474 | 2.992434 | 0.002888171 | 0.009119088 | -3.44441 |
| ENSG00000174177.11 | CTU2 | 0.686357 | 2.401106 | 11.16155 | 2.76E-26 | 7.88E-25 | 48.42948 |
| ENSG00000155363.17 | MOV10 | 0.686348 | 3.397667 | 9.326561 | 2.44E-19 | 4.33E-18 | 32.58323 |
| ENSG00000131188.10 | PRR7 | 0.686096 | 1.260643 | 8.026589 | 5.81E-15 | 7.35E-14 | 22.63045 |
| ENSG00000274750.1 | HIST1H3E | 0.686002 | 1.160215 | 5.457366 | 7.23E-08 | 4.61E-07 | 6.629872 |
| ENSG00000101166.14 | SLMO2 | 0.685731 | 4.980597 | 7.908927 | 1.37E-14 | 1.68E-13 | 21.78723 |
| ENSG00000143367.14 | TUFT1 | 0.685495 | 3.523953 | 7.346461 | 7.13E-13 | 7.58E-12 | 17.89596 |
| ENSG00000125743.9 | SNRPD2 | 0.685299 | 6.17824 | 7.941052 | 1.08E-14 | 1.34E-13 | 22.01647 |
| ENSG00000196878.11 | LAMB3 | 0.685204 | 5.985653 | 3.762736 | 0.000185568 | 0.000738498 | -0.89374 |
| ENSG00000103042.7 | SLC38A7 | 0.685106 | 2.254145 | 10.83896 | 5.26E-25 | 1.39E-23 | 45.50528 |
| ENSG00000212864.3 | RNF208 | 0.685077 | 2.87128 | 6.488367 | 1.89E-10 | 1.59E-09 | 12.42247 |
| ENSG00000168701.17 | TMEM208 | 0.684776 | 4.595167 | 8.612522 | 7.10E-17 | 1.04E-15 | 26.97488 |
| ENSG00000168488.17 | ATXN2L | 0.684421 | 4.473234 | 10.23599 | 1.12E-22 | 2.53E-21 | 40.19264 |
| ENSG00000132591.10 | ERAL1 | 0.684332 | 4.484195 | 12.46914 | 1.05E-31 | 4.22E-30 | 60.81985 |
| ENSG00000150787.6 | PTS | 0.684138 | 2.563177 | 9.805546 | 4.52E-21 | 9.10E-20 | 36.52841 |
| ENSG00000247626.4 | MARS2 | 0.683657 | 2.037047 | 11.18442 | 2.24E-26 | 6.44E-25 | 48.63889 |
| ENSG00000163462.16 | TRIM46 | 0.683566 | 1.073541 | 7.781232 | 3.42E-14 | 4.06E-13 | 20.88341 |
| ENSG00000116521.9 | SCAMP3 | 0.681845 | 5.246096 | 10.70324 | 1.79E-24 | 4.55E-23 | 44.29178 |
| ENSG00000249992.1 | TMEM158 | 0.681541 | 1.367154 | 5.560032 | 4.16E-08 | 2.72E-07 | 7.166838 |
| ENSG00000165644.9 | COMTD1 | 0.681411 | 2.954301 | 5.882832 | 6.90E-09 | 4.93E-08 | 8.913297 |
| ENSG00000165283.14 | STOML2 | 0.680809 | 5.402984 | 8.539421 | 1.25E-16 | 1.80E-15 | 26.41989 |
| ENSG00000090932.9 | DLL3 | 0.680785 | 0.630901 | 4.652016 | 4.10E-06 | 2.09E-05 | 2.73449 |
| ENSG00000154269.13 | ENPP3 | 0.680601 | 1.25043 | 4.176949 | 3.42E-05 | 0.000153181 | 0.706558 |
| ENSG00000165591.6 | FAAH2 | 0.680337 | 2.871207 | 7.10518 | 3.63E-12 | 3.58E-11 | 16.29925 |
| ENSG00000204991.9 | SPIRE2 | 0.680281 | 1.736359 | 7.003671 | 7.09E-12 | 6.83E-11 | 15.64081 |
| ENSG00000151503.11 | NCAPD3 | 0.680258 | 2.112093 | 8.675448 | 4.36E-17 | 6.53E-16 | 27.45554 |
| ENSG00000177156.9 | TALDO1 | 0.680227 | 6.176097 | 6.498246 | 1.78E-10 | 1.50E-09 | 12.48219 |
| ENSG00000181163.12 | NPM1 | 0.680194 | 6.802907 | 8.443847 | 2.58E-16 | 3.64E-15 | 25.6998 |
| ENSG00000177459.9 | ERICH5 | 0.680126 | 1.536045 | 4.472684 | 9.34E-06 | 4.54E-05 | 1.945057 |
| ENSG00000103356.14 | EARS2 | 0.680092 | 2.664496 | 11.55167 | 7.27E-28 | 2.30E-26 | 52.03855 |
| ENSG00000164885.11 | CDK5 | 0.680003 | 2.74133 | 10.13083 | 2.79E-22 | 6.12E-21 | 39.28735 |
| ENSG00000149761.7 | NUDT22 | 0.67988 | 2.931799 | 10.68929 | 2.03E-24 | 5.13E-23 | 44.16767 |
| ENSG00000187045.15 | TMPRSS6 | 0.679826 | 1.289435 | 3.874691 | 0.000119237 | 0.000490567 | -0.4768 |
| ENSG00000115486.10 | GGCX | 0.679802 | 3.494173 | 7.604187 | 1.20E-13 | 1.36E-12 | 19.64997 |
| ENSG00000213401.7 | MAGEA12 | 0.679683 | 0.630067 | 3.458825 | 0.000583315 | 0.002117559 | -1.96675 |
| ENSG00000130332.13 | LSM7 | 0.679332 | 4.249283 | 8.388744 | 3.92E-16 | 5.46E-15 | 25.28748 |
| ENSG00000143315.6 | PIGM | 0.679324 | 2.872615 | 9.937671 | 1.47E-21 | 3.06E-20 | 37.64143 |
| ENSG00000188042.7 | ARL4C | 0.679266 | 4.407335 | 5.717994 | 1.75E-08 | 1.19E-07 | 8.010503 |
| ENSG00000161682.13 | FAM171A2 | 0.678904 | 1.070321 | 6.495883 | 1.81E-10 | 1.52E-09 | 12.4679 |
| ENSG00000132341.10 | RAN | 0.67881 | 5.583383 | 8.156389 | 2.23E-15 | 2.93E-14 | 23.57213 |
| ENSG00000144231.9 | POLR2D | 0.678609 | 3.240618 | 12.39175 | 2.25E-31 | 8.85E-30 | 60.06403 |
| ENSG00000143373.16 | ZNF687 | 0.678472 | 3.124831 | 9.544496 | 4.04E-20 | 7.57E-19 | 34.36057 |
| ENSG00000143575.13 | HAX1 | 0.678338 | 5.39671 | 8.925904 | 6.13E-18 | 9.87E-17 | 29.39496 |
| ENSG00000121931.14 | LRIF1 | 0.678327 | 2.572301 | 8.70582 | 3.45E-17 | 5.20E-16 | 27.68849 |
| ENSG00000116455.12 | WDR77 | 0.678203 | 3.41831 | 11.30791 | 7.12E-27 | 2.12E-25 | 49.7743 |
| ENSG00000164815.9 | ORC5 | 0.677916 | 2.508354 | 10.58693 | 5.06E-24 | 1.25E-22 | 43.25994 |
| ENSG00000125991.17 | ERGIC3 | 0.677699 | 5.504444 | 11.46281 | 1.68E-27 | 5.18E-26 | 51.20963 |
| ENSG00000084623.10 | EIF3I | 0.677629 | 6.797892 | 11.76945 | 9.25E-29 | 3.11E-27 | 54.08655 |
| ENSG00000175634.13 | RPS6KB2 | 0.677579 | 3.278512 | 11.25147 | 1.20E-26 | 3.52E-25 | 49.25447 |
| ENSG00000072864.11 | NDE1 | 0.677478 | 2.201519 | 9.454706 | 8.50E-20 | 1.56E-18 | 33.62468 |
| ENSG00000167325.13 | RRM1 | 0.677375 | 3.927702 | 8.963345 | 4.55E-18 | 7.40E-17 | 29.68847 |
| ENSG00000116096.5 | SPR | 0.67707 | 4.801886 | 7.821748 | 2.56E-14 | 3.07E-13 | 21.1689 |
| ENSG00000171960.9 | PPIH | 0.676905 | 4.02667 | 9.215192 | 6.03E-19 | 1.04E-17 | 31.68658 |
| ENSG00000110697.11 | PITPNM1 | 0.675876 | 3.438395 | 7.509469 | 2.32E-13 | 2.57E-12 | 18.99959 |
| ENSG00000106355.8 | LSM5 | 0.675791 | 3.012998 | 9.863604 | 2.76E-21 | 5.64E-20 | 37.01619 |
| ENSG00000131368.6 | MRPS25 | 0.675779 | 3.345894 | 7.182946 | 2.16E-12 | 2.18E-11 | 16.80903 |
| ENSG00000137948.17 | BRDT | 0.675478 | 0.635207 | 4.493071 | 8.51E-06 | 4.16E-05 | 2.03335 |
| ENSG00000125850.9 | OVOL2 | 0.675288 | 2.354028 | 7.398722 | 4.99E-13 | 5.37E-12 | 18.24762 |
| ENSG00000214087.7 | ARL16 | 0.675098 | 3.369984 | 8.494014 | 1.76E-16 | 2.52E-15 | 26.07699 |
| ENSG00000173486.11 | FKBP2 | 0.674753 | 5.510317 | 7.368069 | 6.15E-13 | 6.58E-12 | 18.04111 |
| ENSG00000183309.10 | ZNF623 | 0.674655 | 2.605102 | 10.07364 | 4.57E-22 | 9.86E-21 | 38.79771 |
| ENSG00000160917.13 | CPSF4 | 0.674362 | 2.920348 | 9.221254 | 5.74E-19 | 9.95E-18 | 31.73519 |
| ENSG00000198363.14 | ASPH | 0.674102 | 3.986354 | 4.376631 | 1.44E-05 | 6.79E-05 | 1.534116 |
| ENSG00000108469.13 | RECQL5 | 0.674048 | 2.237736 | 9.800913 | 4.70E-21 | 9.45E-20 | 36.48957 |
| ENSG00000136718.8 | IMP4 | 0.674018 | 3.894833 | 12.61955 | 2.38E-32 | 9.98E-31 | 62.29651 |
| ENSG00000142319.17 | SLC6A3 | 0.673889 | 0.780517 | 4.237713 | 2.64E-05 | 0.000119838 | 0.954552 |
| ENSG00000159208.14 | CIART | 0.673883 | 1.636601 | 6.038052 | 2.82E-09 | 2.10E-08 | 9.784135 |
| ENSG00000103005.10 | USB1 | 0.673787 | 3.209393 | 11.1196 | 4.06E-26 | 1.15E-24 | 48.04609 |
| ENSG00000167536.12 | DHRS13 | 0.673201 | 1.438275 | 10.73471 | 1.35E-24 | 3.46E-23 | 44.57223 |
| ENSG00000162813.16 | BPNT1 | 0.673179 | 3.950157 | 9.429027 | 1.05E-19 | 1.92E-18 | 33.41515 |
| ENSG00000160307.8 | S100B | 0.67314 | 2.356789 | 3.759047 | 0.000188257 | 0.000748363 | -0.90728 |
| ENSG00000276966.1 | HIST1H4E | 0.672811 | 0.777671 | 4.373911 | 1.45E-05 | 6.86E-05 | 1.522599 |
| ENSG00000161996.16 | WDR90 | 0.672648 | 1.890666 | 7.246602 | 1.41E-12 | 1.45E-11 | 17.22976 |
| ENSG00000101134.10 | DOK5 | 0.672605 | 0.931056 | 6.421726 | 2.85E-10 | 2.35E-09 | 12.02165 |
| ENSG00000156469.7 | MTERF3 | 0.672521 | 3.38661 | 9.196294 | 7.03E-19 | 1.21E-17 | 31.53523 |
| ENSG00000125508.3 | SRMS | 0.671956 | 0.887032 | 6.265972 | 7.34E-10 | 5.79E-09 | 11.09886 |
| ENSG00000103254.8 | FAM173A | 0.671863 | 2.665771 | 7.90618 | 1.39E-14 | 1.71E-13 | 21.76766 |
| ENSG00000023909.8 | GCLM | 0.671595 | 3.086974 | 5.187238 | 2.98E-07 | 1.76E-06 | 5.260238 |
| ENSG00000101849.14 | TBL1X | 0.671457 | 2.745841 | 7.14064 | 2.86E-12 | 2.86E-11 | 16.53113 |
| ENSG00000150990.6 | DHX37 | 0.671212 | 2.392031 | 9.929926 | 1.57E-21 | 3.27E-20 | 37.57589 |
| ENSG00000108344.13 | PSMD3 | 0.671145 | 4.930637 | 10.4077 | 2.48E-23 | 5.82E-22 | 41.68466 |
| ENSG00000105281.11 | SLC1A5 | 0.670922 | 5.398083 | 7.86392 | 1.89E-14 | 2.30E-13 | 21.46732 |
| ENSG00000129991.11 | TNNI3 | 0.67078 | 0.897236 | 4.743139 | 2.67E-06 | 1.39E-05 | 3.146635 |
| ENSG00000221947.6 | XKR9 | 0.670584 | 0.937357 | 7.326835 | 8.16E-13 | 8.62E-12 | 17.76443 |
| ENSG00000124207.15 | CSE1L | 0.670581 | 4.589904 | 8.000767 | 7.01E-15 | 8.81E-14 | 22.44455 |
| ENSG00000184678.9 | HIST2H2BE | 0.670523 | 3.479547 | 4.395226 | 1.32E-05 | 6.29E-05 | 1.61302 |
| ENSG00000048991.15 | R3HDM1 | 0.670126 | 2.291837 | 11.7012 | 1.77E-28 | 5.85E-27 | 53.44216 |
| ENSG00000196542.7 | SPTSSB | 0.670123 | 0.694823 | 4.726605 | 2.88E-06 | 1.50E-05 | 3.071305 |
| ENSG00000085465.12 | OVGP1 | 0.669969 | 1.292993 | 6.898055 | 1.41E-11 | 1.32E-10 | 14.9642 |
| ENSG00000135372.7 | NAT10 | 0.66964 | 3.632194 | 11.37084 | 3.96E-27 | 1.19E-25 | 50.35598 |
| ENSG00000106399.10 | RPA3 | 0.669607 | 3.03844 | 8.206713 | 1.54E-15 | 2.04E-14 | 23.94044 |
| ENSG00000169857.6 | AVEN | 0.669564 | 2.737175 | 9.910098 | 1.86E-21 | 3.85E-20 | 37.40828 |
| ENSG00000175854.10 | SWI5 | 0.669408 | 3.43663 | 10.18361 | 1.76E-22 | 3.93E-21 | 39.74095 |
| ENSG00000100350.13 | FOXRED2 | 0.669037 | 2.520303 | 6.514762 | 1.61E-10 | 1.36E-09 | 12.58221 |
| ENSG00000065357.18 | DGKA | 0.668953 | 1.919141 | 8.066578 | 4.33E-15 | 5.54E-14 | 22.91929 |
| ENSG00000101146.11 | RAE1 | 0.668895 | 2.724233 | 10.55549 | 6.70E-24 | 1.64E-22 | 42.98226 |
| ENSG00000204618.7 | RNF39 | 0.668718 | 2.000957 | 5.001726 | 7.59E-07 | 4.25E-06 | 4.35627 |
| ENSG00000241468.6 | ATP5J2 | 0.668713 | 5.293705 | 7.227151 | 1.60E-12 | 1.64E-11 | 17.10087 |
| ENSG00000169474.4 | SPRR1A | 0.668529 | 0.842856 | 3.552431 | 0.000413478 | 0.001545401 | -1.64545 |
| ENSG00000115457.8 | IGFBP2 | 0.668239 | 4.314353 | 2.786558 | 0.005505641 | 0.016286692 | -4.03085 |
| ENSG00000051596.8 | THOC3 | 0.667495 | 1.738363 | 10.58744 | 5.04E-24 | 1.25E-22 | 43.26442 |
| ENSG00000180902.15 | D2HGDH | 0.667388 | 2.417402 | 6.60678 | 9.07E-11 | 7.84E-10 | 13.14345 |
| ENSG00000138376.9 | BARD1 | 0.667309 | 1.614273 | 9.802656 | 4.63E-21 | 9.31E-20 | 36.50418 |
| ENSG00000184432.8 | COPB2 | 0.667265 | 4.596403 | 11.03257 | 9.03E-26 | 2.51E-24 | 47.25366 |
| ENSG00000203995.8 | ZYG11A | 0.667065 | 0.624693 | 8.207272 | 1.53E-15 | 2.03E-14 | 23.94454 |
| ENSG00000004487.14 | KDM1A | 0.666783 | 4.046075 | 10.44988 | 1.71E-23 | 4.08E-22 | 42.05376 |
| ENSG00000059588.8 | TARBP1 | 0.666767 | 2.723092 | 7.459249 | 3.29E-13 | 3.60E-12 | 18.65746 |
| ENSG00000168569.7 | TMEM223 | 0.666553 | 3.200693 | 9.464952 | 7.81E-20 | 1.44E-18 | 33.7084 |
| ENSG00000130299.15 | GTPBP3 | 0.666479 | 2.158744 | 9.959757 | 1.22E-21 | 2.55E-20 | 37.82851 |
| ENSG00000103855.16 | CD276 | 0.666411 | 4.150105 | 9.370253 | 1.70E-19 | 3.06E-18 | 32.93715 |
| ENSG00000027847.12 | B4GALT7 | 0.666285 | 2.846938 | 10.80636 | 7.07E-25 | 1.86E-23 | 45.21286 |
| ENSG00000060339.12 | CCAR1 | 0.666227 | 3.635083 | 11.94593 | 1.71E-29 | 5.99E-28 | 55.76319 |
| ENSG00000125505.15 | MBOAT7 | 0.666204 | 4.195252 | 8.909698 | 6.96E-18 | 1.12E-16 | 29.26821 |
| ENSG00000142765.16 | SYTL1 | 0.666149 | 3.020804 | 5.621501 | 2.97E-08 | 1.98E-07 | 7.492627 |
| ENSG00000099804.7 | CDC34 | 0.665925 | 4.581968 | 8.243588 | 1.17E-15 | 1.56E-14 | 24.21145 |
| ENSG00000146410.10 | MTFR2 | 0.665877 | 1.215634 | 7.703909 | 5.93E-14 | 6.88E-13 | 20.34188 |
| ENSG00000196187.10 | TMEM63A | 0.665766 | 3.435877 | 6.884414 | 1.54E-11 | 1.44E-10 | 14.87744 |
| ENSG00000133055.7 | MYBPH | 0.665512 | 0.816277 | 4.310067 | 1.92E-05 | 8.93E-05 | 1.254226 |
| ENSG00000143106.11 | PSMA5 | 0.665009 | 4.124593 | 9.849475 | 3.11E-21 | 6.35E-20 | 36.89729 |
| ENSG00000241370.4 | RPP21 | 0.664892 | 2.417963 | 9.996711 | 8.86E-22 | 1.87E-20 | 38.14217 |
| ENSG00000100401.18 | RANGAP1 | 0.664547 | 4.260399 | 9.106687 | 1.45E-18 | 2.43E-17 | 30.82067 |
| ENSG00000090263.14 | MRPS33 | 0.664211 | 2.838196 | 9.251493 | 4.49E-19 | 7.85E-18 | 31.97797 |
| ENSG00000101407.11 | TTI1 | 0.664087 | 3.090278 | 10.383 | 3.08E-23 | 7.19E-22 | 41.469 |
| ENSG00000139546.9 | TARBP2 | 0.663488 | 2.978022 | 10.04807 | 5.70E-22 | 1.22E-20 | 38.57948 |
| ENSG00000064601.15 | CTSA | 0.66301 | 5.460963 | 8.088386 | 3.69E-15 | 4.75E-14 | 23.07729 |
| ENSG00000175352.9 | NRIP3 | 0.662978 | 1.295356 | 5.27419 | 1.90E-07 | 1.15E-06 | 5.69424 |
| ENSG00000127418.13 | FGFRL1 | 0.662925 | 3.365068 | 6.615754 | 8.57E-11 | 7.44E-10 | 13.19854 |
| ENSG00000217555.11 | CKLF | 0.662609 | 3.394466 | 6.986004 | 7.96E-12 | 7.63E-11 | 15.52703 |
| ENSG00000119630.12 | PGF | 0.662365 | 1.353511 | 6.761988 | 3.40E-11 | 3.07E-10 | 14.10533 |
| ENSG00000228474.4 | OST4 | 0.662128 | 7.482573 | 9.21265 | 6.16E-19 | 1.06E-17 | 31.66621 |
| ENSG00000213347.9 | MXD3 | 0.662007 | 1.223753 | 9.189317 | 7.44E-19 | 1.28E-17 | 31.47941 |
| ENSG00000081721.10 | DUSP12 | 0.661876 | 3.374692 | 11.44029 | 2.07E-27 | 6.37E-26 | 51.00024 |
| ENSG00000183258.10 | DDX41 | 0.661862 | 4.006497 | 12.73183 | 7.81E-33 | 3.37E-31 | 63.40528 |
| ENSG00000177888.7 | ZBTB41 | 0.66183 | 2.441672 | 10.23384 | 1.14E-22 | 2.57E-21 | 40.17409 |
| ENSG00000196155.11 | PLEKHG4 | 0.661645 | 1.815916 | 5.650462 | 2.54E-08 | 1.70E-07 | 7.647234 |
| ENSG00000157111.11 | TMEM171 | 0.661504 | 0.776658 | 6.606856 | 9.06E-11 | 7.84E-10 | 13.14391 |
| ENSG00000186283.12 | TOR3A | 0.661489 | 4.123041 | 10.83067 | 5.67E-25 | 1.50E-23 | 45.43086 |
| ENSG00000136280.14 | CCM2 | 0.661345 | 3.119018 | 8.068398 | 4.28E-15 | 5.47E-14 | 22.93246 |
| ENSG00000176749.7 | CDK5R1 | 0.661041 | 1.143629 | 7.888585 | 1.58E-14 | 1.93E-13 | 21.64246 |
| ENSG00000111716.11 | LDHB | 0.66098 | 7.021878 | 4.63382 | 4.46E-06 | 2.27E-05 | 2.653078 |
| ENSG00000123562.15 | MORF4L2 | 0.660965 | 6.383161 | 10.12126 | 3.03E-22 | 6.63E-21 | 39.20527 |
| ENSG00000130520.9 | LSM4 | 0.660858 | 4.733522 | 9.000322 | 3.39E-18 | 5.56E-17 | 29.97926 |
| ENSG00000082153.16 | BZW1 | 0.660583 | 4.244158 | 8.521977 | 1.42E-16 | 2.05E-15 | 26.288 |
| ENSG00000106028.9 | SSBP1 | 0.660204 | 3.666722 | 10.40526 | 2.54E-23 | 5.94E-22 | 41.6634 |
| ENSG00000135503.11 | ACVR1B | 0.659539 | 3.600554 | 6.915175 | 1.26E-11 | 1.19E-10 | 15.07328 |
| ENSG00000100416.11 | TRMU | 0.659349 | 1.862357 | 10.13934 | 2.59E-22 | 5.71E-21 | 39.36037 |
| ENSG00000120802.12 | TMPO | 0.659104 | 3.608541 | 7.193867 | 2.00E-12 | 2.03E-11 | 16.881 |
| ENSG00000141698.15 | NT5C3B | 0.658803 | 3.962747 | 8.678706 | 4.25E-17 | 6.37E-16 | 27.48049 |
| ENSG00000164880.14 | INTS1 | 0.658797 | 4.107368 | 6.738466 | 3.95E-11 | 3.55E-10 | 13.95833 |
| ENSG00000123364.4 | HOXC13 | 0.658707 | 0.600149 | 5.696313 | 1.97E-08 | 1.34E-07 | 7.893457 |
| ENSG00000133119.11 | RFC3 | 0.6587 | 2.429291 | 7.422655 | 4.23E-13 | 4.59E-12 | 18.40935 |
| ENSG00000172269.15 | DPAGT1 | 0.658663 | 3.386874 | 11.11842 | 4.11E-26 | 1.16E-24 | 48.03537 |
| ENSG00000122678.13 | POLM | 0.658659 | 2.622331 | 9.564813 | 3.41E-20 | 6.41E-19 | 34.52778 |
| ENSG00000124802.10 | EEF1E1 | 0.658289 | 2.533932 | 9.164592 | 9.08E-19 | 1.55E-17 | 31.28183 |
| ENSG00000089692.7 | LAG3 | 0.658277 | 1.645324 | 5.273277 | 1.91E-07 | 1.16E-06 | 5.689652 |
| ENSG00000136243.15 | NUPL2 | 0.658242 | 2.733054 | 10.53632 | 7.95E-24 | 1.94E-22 | 42.81329 |
| ENSG00000032389.11 | TSSC1 | 0.657932 | 2.559587 | 11.74215 | 1.20E-28 | 4.01E-27 | 53.82854 |
| ENSG00000154473.16 | BUB3 | 0.657882 | 3.419577 | 11.44241 | 2.03E-27 | 6.25E-26 | 51.01989 |
| ENSG00000166126.9 | AMN | 0.657706 | 1.316146 | 4.843158 | 1.65E-06 | 8.87E-06 | 3.607507 |
| ENSG00000266472.4 | MRPS21 | 0.656502 | 5.236578 | 7.535212 | 1.94E-13 | 2.16E-12 | 19.17569 |
| ENSG00000169851.14 | PCDH7 | 0.656178 | 1.342297 | 4.926749 | 1.10E-06 | 6.04E-06 | 3.99947 |
| ENSG00000197520.9 | FAM177B | 0.656045 | 0.840356 | 4.883688 | 1.36E-06 | 7.36E-06 | 3.796786 |
| ENSG00000134684.9 | YARS | 0.655998 | 3.930711 | 12.02748 | 7.80E-30 | 2.79E-28 | 56.54291 |
| ENSG00000101442.9 | ACTR5 | 0.655927 | 2.505454 | 11.16528 | 2.67E-26 | 7.62E-25 | 48.46366 |
| ENSG00000184260.5 | HIST2H2AC | 0.654892 | 1.237665 | 4.910207 | 1.19E-06 | 6.52E-06 | 3.921415 |
| ENSG00000029559.6 | IBSP | 0.654875 | 0.713183 | 5.110247 | 4.41E-07 | 2.55E-06 | 4.881428 |
| ENSG00000250571.5 | GLI4 | 0.654711 | 2.218075 | 7.118811 | 3.31E-12 | 3.28E-11 | 16.38827 |
| ENSG00000163545.7 | NUAK2 | 0.654695 | 2.881143 | 6.289665 | 6.36E-10 | 5.06E-09 | 11.23796 |
| ENSG00000173281.4 | PPP1R3B | 0.654618 | 3.51471 | 5.632536 | 2.80E-08 | 1.87E-07 | 7.551455 |
| ENSG00000134602.14 | STK26 | 0.654529 | 3.174395 | 6.397967 | 3.30E-10 | 2.71E-09 | 11.87962 |
| ENSG00000113643.7 | RARS | 0.654144 | 4.240271 | 11.94338 | 1.75E-29 | 6.14E-28 | 55.73883 |
| ENSG00000034510.5 | TMSB10 | 0.653852 | 11.27545 | 6.300913 | 5.95E-10 | 4.74E-09 | 11.30416 |
| ENSG00000163755.7 | HPS3 | 0.653762 | 2.883154 | 9.141225 | 1.10E-18 | 1.86E-17 | 31.09548 |
| ENSG00000170485.15 | NPAS2 | 0.653607 | 1.444641 | 6.898028 | 1.41E-11 | 1.32E-10 | 14.96402 |
| ENSG00000104635.12 | SLC39A14 | 0.65339 | 2.915629 | 6.079271 | 2.22E-09 | 1.67E-08 | 10.01874 |
| ENSG00000249115.7 | HAUS5 | 0.653343 | 2.274615 | 7.821758 | 2.56E-14 | 3.07E-13 | 21.16896 |
| ENSG00000111331.11 | OAS3 | 0.653338 | 3.761789 | 5.096538 | 4.72E-07 | 2.72E-06 | 4.81452 |
| ENSG00000185332.5 | TMEM105 | 0.653258 | 0.848608 | 9.417282 | 1.16E-19 | 2.10E-18 | 33.31945 |
| ENSG00000139044.9 | B4GALNT3 | 0.653224 | 3.268853 | 4.337101 | 1.71E-05 | 7.99E-05 | 1.367416 |
| ENSG00000177054.12 | ZDHHC13 | 0.653147 | 2.087312 | 9.782255 | 5.50E-21 | 1.10E-19 | 36.33329 |
| ENSG00000175756.12 | AURKAIP1 | 0.65303 | 5.370405 | 7.956218 | 9.70E-15 | 1.21E-13 | 22.12494 |
| ENSG00000125844.14 | RRBP1 | 0.653022 | 5.73153 | 6.132522 | 1.62E-09 | 1.24E-08 | 10.3239 |
| ENSG00000168040.4 | FADD | 0.652779 | 2.830911 | 11.21123 | 1.74E-26 | 5.07E-25 | 48.88475 |
| ENSG00000163218.13 | PGLYRP4 | 0.652638 | 0.6558 | 6.019585 | 3.14E-09 | 2.33E-08 | 9.679481 |
| ENSG00000143942.4 | CHAC2 | 0.652309 | 1.838156 | 7.871658 | 1.79E-14 | 2.18E-13 | 21.52221 |
| ENSG00000198720.11 | ANKRD13B | 0.651928 | 1.352617 | 7.230428 | 1.57E-12 | 1.61E-11 | 17.12256 |
| ENSG00000125398.5 | SOX9 | 0.65178 | 2.652986 | 3.291931 | 0.001056969 | 0.003657035 | -2.51916 |
| ENSG00000124155.15 | PIGT | 0.651768 | 6.173156 | 7.964446 | 9.14E-15 | 1.14E-13 | 22.18386 |
| ENSG00000116857.15 | TMEM9 | 0.651682 | 5.123899 | 7.049188 | 5.25E-12 | 5.12E-11 | 15.93507 |
| ENSG00000169136.7 | ATF5 | 0.651558 | 3.971433 | 7.101146 | 3.72E-12 | 3.67E-11 | 16.27293 |
| ENSG00000115084.11 | SLC35F5 | 0.651527 | 3.340299 | 9.109795 | 1.41E-18 | 2.38E-17 | 30.84537 |
| ENSG00000166197.15 | NOLC1 | 0.65148 | 4.839608 | 8.896871 | 7.71E-18 | 1.23E-16 | 29.16801 |
| ENSG00000137504.12 | CREBZF | 0.651322 | 3.542989 | 8.341636 | 5.60E-16 | 7.70E-15 | 24.93667 |
| ENSG00000163781.11 | TOPBP1 | 0.651196 | 2.808728 | 7.798525 | 3.02E-14 | 3.60E-13 | 21.00511 |
| ENSG00000156876.9 | SASS6 | 0.650998 | 1.440718 | 9.642345 | 1.79E-20 | 3.41E-19 | 35.16823 |
| ENSG00000217442.3 | SYCE3 | 0.650732 | 0.978783 | 6.62898 | 7.89E-11 | 6.87E-10 | 13.27986 |
| ENSG00000091483.6 | FH | 0.650641 | 4.864365 | 10.35475 | 3.95E-23 | 9.15E-22 | 41.22275 |
| ENSG00000109919.8 | MTCH2 | 0.650605 | 4.772596 | 10.79626 | 7.74E-25 | 2.03E-23 | 45.1224 |
| ENSG00000185414.18 | MRPL30 | 0.650468 | 3.43889 | 11.48841 | 1.32E-27 | 4.10E-26 | 51.448 |
| ENSG00000007306.13 | CEACAM7 | 0.650072 | 0.698164 | 4.307126 | 1.95E-05 | 9.03E-05 | 1.241951 |
| ENSG00000105197.9 | TIMM50 | 0.650023 | 3.199733 | 9.156699 | 9.68E-19 | 1.65E-17 | 31.21884 |
| ENSG00000145495.13 | 6-Mar | 0.650004 | 4.065982 | 8.331246 | 6.06E-16 | 8.29E-15 | 24.8595 |
| ENSG00000123892.10 | RAB38 | 0.649985 | 3.120669 | 5.051587 | 5.92E-07 | 3.37E-06 | 4.596279 |
| ENSG00000144191.10 | CNGA3 | 0.649985 | 0.660609 | 4.459413 | 9.91E-06 | 4.80E-05 | 1.887785 |
| ENSG00000171806.10 | METTL18 | 0.649657 | 2.682747 | 11.10385 | 4.70E-26 | 1.32E-24 | 47.9024 |
| ENSG00000004455.15 | AK2 | 0.649618 | 4.669954 | 9.559927 | 3.55E-20 | 6.67E-19 | 34.48755 |
| ENSG00000214022.10 | REPIN1 | 0.649254 | 4.489305 | 8.737605 | 2.69E-17 | 4.10E-16 | 27.93294 |
| ENSG00000204564.10 | C6orf136 | 0.649171 | 2.915566 | 8.926467 | 6.10E-18 | 9.83E-17 | 29.39937 |
| ENSG00000164024.10 | METAP1 | 0.648887 | 3.834523 | 11.5955 | 4.81E-28 | 1.54E-26 | 52.44882 |
| ENSG00000154370.12 | TRIM11 | 0.648826 | 2.319408 | 10.69334 | 1.96E-24 | 4.95E-23 | 44.20369 |
| ENSG00000090534.16 | THPO | 0.648782 | 0.780881 | 5.196868 | 2.83E-07 | 1.68E-06 | 5.307979 |
| ENSG00000141424.11 | SLC39A6 | 0.648631 | 4.396243 | 6.618284 | 8.44E-11 | 7.33E-10 | 13.21409 |
| ENSG00000130706.11 | ADRM1 | 0.648482 | 5.573712 | 8.823092 | 1.38E-17 | 2.15E-16 | 28.59377 |
| ENSG00000139405.14 | RITA1 | 0.648369 | 3.52456 | 10.71135 | 1.66E-24 | 4.25E-23 | 44.36402 |
| ENSG00000115415.17 | STAT1 | 0.647835 | 5.734622 | 5.23269 | 2.36E-07 | 1.41E-06 | 5.486285 |
| ENSG00000205937.10 | RNPS1 | 0.64773 | 3.837624 | 13.15303 | 1.13E-34 | 5.49E-33 | 67.61238 |
| ENSG00000213186.6 | TRIM59 | 0.647654 | 1.128161 | 10.57226 | 5.77E-24 | 1.42E-22 | 43.13029 |
| ENSG00000134851.11 | TMEM165 | 0.647402 | 4.267753 | 8.99004 | 3.68E-18 | 6.02E-17 | 29.89831 |
| ENSG00000087152.14 | ATXN7L3 | 0.646941 | 3.98537 | 11.43002 | 2.28E-27 | 6.99E-26 | 50.90483 |
| ENSG00000106615.8 | RHEB | 0.646904 | 4.693241 | 8.277448 | 9.07E-16 | 1.23E-14 | 24.46113 |
| ENSG00000278535.3 | DHRS11 | 0.646849 | 1.579789 | 9.72269 | 9.10E-21 | 1.78E-19 | 35.83582 |
| ENSG00000214193.8 | SH3D21 | 0.646814 | 1.580298 | 7.767816 | 3.77E-14 | 4.44E-13 | 20.78913 |
| ENSG00000126267.7 | COX6B1 | 0.646767 | 7.128544 | 7.412128 | 4.55E-13 | 4.92E-12 | 18.33816 |
| ENSG00000175426.9 | PCSK1 | 0.646419 | 0.703038 | 2.941971 | 0.003394694 | 0.010536321 | -3.59191 |
| ENSG00000138092.9 | CENPO | 0.645832 | 1.652062 | 9.351589 | 1.98E-19 | 3.55E-18 | 32.78582 |
| ENSG00000168101.13 | NUDT16L1 | 0.645729 | 3.972781 | 7.987859 | 7.71E-15 | 9.64E-14 | 22.35179 |
| ENSG00000130300.7 | PLVAP | 0.645693 | 5.039435 | 5.244601 | 2.22E-07 | 1.33E-06 | 5.545819 |
| ENSG00000100979.13 | PLTP | 0.645669 | 5.64588 | 4.370782 | 1.47E-05 | 6.95E-05 | 1.509358 |
| ENSG00000142675.16 | CNKSR1 | 0.645526 | 3.270002 | 7.32163 | 8.45E-13 | 8.93E-12 | 17.7296 |
| ENSG00000075618.16 | FSCN1 | 0.645407 | 4.450801 | 3.724741 | 0.000215086 | 0.000845896 | -1.0326 |
| ENSG00000115866.9 | DARS | 0.645172 | 4.59857 | 11.07683 | 6.02E-26 | 1.68E-24 | 47.65614 |
| ENSG00000204291.9 | COL15A1 | 0.644975 | 2.955321 | 4.143358 | 3.95E-05 | 0.00017512 | 0.570909 |
| ENSG00000196123.11 | KIAA0895L | 0.644963 | 2.152358 | 6.32106 | 5.27E-10 | 4.22E-09 | 11.42299 |
| ENSG00000103534.15 | TMC5 | 0.644626 | 4.165154 | 4.127222 | 4.22E-05 | 0.000186712 | 0.506116 |
| ENSG00000172830.11 | SSH3 | 0.644616 | 4.016 | 8.00886 | 6.61E-15 | 8.32E-14 | 22.50277 |
| ENSG00000103507.12 | BCKDK | 0.64382 | 4.080223 | 10.43484 | 1.95E-23 | 4.63E-22 | 41.92206 |
| ENSG00000054148.16 | PHPT1 | 0.643604 | 5.373387 | 6.602237 | 9.33E-11 | 8.06E-10 | 13.11558 |
| ENSG00000103326.9 | CAPN15 | 0.643341 | 2.864131 | 8.359102 | 4.91E-16 | 6.77E-15 | 25.06656 |
| ENSG00000162377.5 | COA7 | 0.643296 | 2.673536 | 9.665396 | 1.47E-20 | 2.82E-19 | 35.35935 |
| ENSG00000125775.13 | SDCBP2 | 0.643258 | 2.572579 | 3.52245 | 0.000462045 | 0.00170996 | -1.74925 |
| ENSG00000108561.7 | C1QBP | 0.643205 | 4.839972 | 7.275185 | 1.16E-12 | 1.20E-11 | 17.41968 |
| ENSG00000172009.13 | THOP1 | 0.643108 | 2.379097 | 9.456801 | 8.35E-20 | 1.53E-18 | 33.64179 |
| ENSG00000164919.9 | COX6C | 0.642815 | 4.912547 | 7.649418 | 8.72E-14 | 9.99E-13 | 19.9629 |
| ENSG00000146143.16 | PRIM2 | 0.642794 | 1.93975 | 9.942664 | 1.41E-21 | 2.93E-20 | 37.6837 |
| ENSG00000136731.11 | UGGT1 | 0.64276 | 3.4796 | 8.699296 | 3.63E-17 | 5.46E-16 | 27.63839 |
| ENSG00000143001.4 | TMEM61 | 0.642378 | 1.552145 | 5.169158 | 3.26E-07 | 1.92E-06 | 5.170816 |
| ENSG00000057294.12 | PKP2 | 0.642324 | 1.427511 | 4.332026 | 1.75E-05 | 8.16E-05 | 1.346117 |
| ENSG00000163083.5 | INHBB | 0.642118 | 2.743701 | 3.634715 | 0.000303612 | 0.001162754 | -1.35625 |
| ENSG00000177943.12 | MAMDC4 | 0.64196 | 1.183465 | 6.595505 | 9.73E-11 | 8.39E-10 | 13.07431 |
| ENSG00000006555.9 | TTC22 | 0.641878 | 1.940628 | 7.576257 | 1.46E-13 | 1.64E-12 | 19.4575 |
| ENSG00000108389.8 | MTMR4 | 0.641823 | 2.94097 | 9.833138 | 3.58E-21 | 7.25E-20 | 36.75997 |
| ENSG00000140451.11 | PIF1 | 0.641731 | 0.802311 | 8.647425 | 5.42E-17 | 8.05E-16 | 27.24115 |
| ENSG00000081181.6 | ARG2 | 0.64142 | 1.644752 | 5.493287 | 5.97E-08 | 3.84E-07 | 6.816722 |
| ENSG00000135624.14 | CCT7 | 0.641231 | 5.927186 | 11.28176 | 9.08E-27 | 2.68E-25 | 49.53328 |
| ENSG00000112651.10 | MRPL2 | 0.641205 | 3.568014 | 9.688781 | 1.21E-20 | 2.34E-19 | 35.55358 |
| ENSG00000158864.11 | NDUFS2 | 0.640897 | 4.843487 | 10.82455 | 6.00E-25 | 1.58E-23 | 45.37599 |
| ENSG00000196748.8 | CLPSL2 | 0.640828 | 0.600433 | 6.040697 | 2.78E-09 | 2.07E-08 | 9.799143 |
| ENSG00000198918.7 | RPL39 | 0.640766 | 5.802801 | 6.181358 | 1.22E-09 | 9.40E-09 | 10.6058 |
| ENSG00000227471.7 | AKR1B15 | 0.640725 | 0.602416 | 4.870451 | 1.45E-06 | 7.82E-06 | 3.734807 |
| ENSG00000119812.17 | FAM98A | 0.640595 | 3.271543 | 12.78674 | 4.52E-33 | 1.99E-31 | 63.94949 |
| ENSG00000100911.12 | PSME2 | 0.640478 | 5.333384 | 7.567984 | 1.54E-13 | 1.74E-12 | 19.4006 |
| ENSG00000187049.8 | TMEM216 | 0.640374 | 2.902526 | 11.34901 | 4.86E-27 | 1.46E-25 | 50.15398 |
| ENSG00000171793.12 | CTPS1 | 0.640244 | 2.792427 | 7.435584 | 3.87E-13 | 4.21E-12 | 18.49689 |
| ENSG00000143569.17 | UBAP2L | 0.64024 | 4.69352 | 9.583334 | 2.92E-20 | 5.53E-19 | 34.68043 |
| ENSG00000159128.13 | IFNGR2 | 0.640161 | 5.433782 | 8.173393 | 1.97E-15 | 2.59E-14 | 23.69638 |
| ENSG00000153531.11 | ADPRHL1 | 0.639712 | 1.571929 | 6.222168 | 9.54E-10 | 7.45E-09 | 10.84287 |
| ENSG00000116761.10 | CTH | 0.639682 | 1.808603 | 6.92219 | 1.21E-11 | 1.14E-10 | 15.11805 |
| ENSG00000204388.6 | HSPA1B | 0.639491 | 5.046763 | 5.782231 | 1.22E-08 | 8.45E-08 | 8.359607 |
| ENSG00000173239.12 | LIPM | 0.639323 | 0.767223 | 7.94705 | 1.04E-14 | 1.29E-13 | 22.05935 |
| ENSG00000132763.13 | MMACHC | 0.639211 | 1.516414 | 12.99378 | 5.66E-34 | 2.62E-32 | 66.013 |
| ENSG00000185619.16 | PCGF3 | 0.638582 | 3.097108 | 9.480394 | 6.87E-20 | 1.27E-18 | 33.8347 |
| ENSG00000175334.6 | BANF1 | 0.638553 | 6.004637 | 9.138826 | 1.12E-18 | 1.90E-17 | 31.07636 |
| ENSG00000174514.11 | MFSD4 | 0.638509 | 2.56016 | 3.133853 | 0.001814519 | 0.005972332 | -3.01813 |
| ENSG00000117877.9 | CD3EAP | 0.638155 | 1.326953 | 10.70568 | 1.75E-24 | 4.45E-23 | 44.31355 |
| ENSG00000138035.13 | PNPT1 | 0.638151 | 3.03485 | 9.000035 | 3.40E-18 | 5.57E-17 | 29.977 |
| ENSG00000124587.12 | PEX6 | 0.638086 | 3.480477 | 6.040805 | 2.78E-09 | 2.07E-08 | 9.799761 |
| ENSG00000177084.15 | POLE | 0.637719 | 1.729639 | 8.502505 | 1.65E-16 | 2.36E-15 | 26.14101 |
| ENSG00000185359.11 | HGS | 0.637359 | 3.467129 | 9.474002 | 7.25E-20 | 1.34E-18 | 33.7824 |
| ENSG00000105355.7 | PLIN3 | 0.637252 | 4.758398 | 7.366312 | 6.23E-13 | 6.65E-12 | 18.02929 |
| ENSG00000143319.15 | ISG20L2 | 0.637036 | 3.389967 | 11.3969 | 3.11E-27 | 9.46E-26 | 50.59744 |
| ENSG00000106927.10 | AMBP | 0.636975 | 1.129504 | 3.240336 | 0.001263908 | 0.004305545 | -2.68462 |
| ENSG00000158604.13 | TMED4 | 0.636962 | 5.321564 | 10.32072 | 5.33E-23 | 1.22E-21 | 40.92679 |
| ENSG00000101181.16 | MTG2 | 0.63646 | 2.766518 | 9.382208 | 1.54E-19 | 2.78E-18 | 33.0342 |
| ENSG00000144524.16 | COPS7B | 0.636423 | 2.867539 | 12.24229 | 9.71E-31 | 3.66E-29 | 58.61186 |
| ENSG00000004777.17 | ARHGAP33 | 0.636407 | 1.365458 | 6.321158 | 5.26E-10 | 4.22E-09 | 11.42357 |
| ENSG00000117153.14 | KLHL12 | 0.636272 | 3.970804 | 11.47962 | 1.43E-27 | 4.43E-26 | 51.36612 |
| ENSG00000198466.11 | ZNF587 | 0.636146 | 1.816138 | 9.317905 | 2.61E-19 | 4.64E-18 | 32.51325 |
| ENSG00000101928.11 | MOSPD1 | 0.635848 | 3.009109 | 6.885874 | 1.53E-11 | 1.43E-10 | 14.88672 |
| ENSG00000162222.12 | TTC9C | 0.635677 | 3.030008 | 12.11277 | 3.42E-30 | 1.24E-28 | 57.3618 |
| ENSG00000250479.7 | CHCHD10 | 0.635232 | 4.186679 | 6.364324 | 4.05E-10 | 3.29E-09 | 11.67928 |
| ENSG00000163216.6 | SPRR2D | 0.634991 | 0.713087 | 3.430569 | 0.000646189 | 0.002325151 | -2.06212 |
| ENSG00000092199.16 | HNRNPC | 0.634902 | 5.680865 | 12.51131 | 6.95E-32 | 2.82E-30 | 61.23286 |
| ENSG00000140905.8 | GCSH | 0.634514 | 1.555469 | 10.37603 | 3.28E-23 | 7.62E-22 | 41.40821 |
| ENSG00000185049.11 | NELFA | 0.634294 | 2.845058 | 10.58094 | 5.34E-24 | 1.32E-22 | 43.207 |
| ENSG00000132793.10 | LPIN3 | 0.634225 | 2.399403 | 6.616538 | 8.53E-11 | 7.40E-10 | 13.20336 |
| ENSG00000106236.3 | NPTX2 | 0.634154 | 0.996267 | 4.356619 | 1.57E-05 | 7.37E-05 | 1.449547 |
| ENSG00000196497.14 | IPO4 | 0.633863 | 1.147585 | 11.77039 | 9.16E-29 | 3.09E-27 | 54.09541 |
| ENSG00000170442.10 | KRT86 | 0.633835 | 1.134601 | 5.965851 | 4.29E-09 | 3.13E-08 | 9.376572 |
| ENSG00000139514.11 | SLC7A1 | 0.633345 | 2.798544 | 6.49656 | 1.80E-10 | 1.52E-09 | 12.47199 |
| ENSG00000185238.11 | PRMT3 | 0.633278 | 1.975532 | 11.52026 | 9.78E-28 | 3.07E-26 | 51.74506 |
| ENSG00000158483.14 | FAM86C1 | 0.632978 | 1.69707 | 12.18493 | 1.70E-30 | 6.31E-29 | 58.05738 |
| ENSG00000173992.7 | CCS | 0.632952 | 3.803045 | 9.036262 | 2.55E-18 | 4.21E-17 | 30.26274 |
| ENSG00000169727.11 | GPS1 | 0.632445 | 4.208317 | 10.26694 | 8.53E-23 | 1.94E-21 | 40.46036 |
| ENSG00000151881.13 | C5orf28 | 0.632306 | 2.632255 | 8.511759 | 1.54E-16 | 2.21E-15 | 26.21083 |
| ENSG00000157800.16 | SLC37A3 | 0.632235 | 2.485523 | 10.78231 | 8.78E-25 | 2.29E-23 | 44.99753 |
| ENSG00000115364.12 | MRPL19 | 0.632197 | 3.232971 | 11.48712 | 1.33E-27 | 4.14E-26 | 51.43602 |
| ENSG00000165501.15 | LRR1 | 0.632184 | 2.235843 | 8.036067 | 5.42E-15 | 6.87E-14 | 22.69881 |
| ENSG00000183527.10 | PSMG1 | 0.631849 | 3.215432 | 8.146036 | 2.41E-15 | 3.15E-14 | 23.49659 |
| ENSG00000048028.10 | USP28 | 0.631741 | 2.609256 | 8.431487 | 2.84E-16 | 3.99E-15 | 25.60713 |
| ENSG00000124541.6 | RRP36 | 0.631502 | 4.294123 | 11.00254 | 1.19E-25 | 3.27E-24 | 46.98121 |
| ENSG00000180806.4 | HOXC9 | 0.631472 | 0.696377 | 5.718235 | 1.74E-08 | 1.19E-07 | 8.011802 |
| ENSG00000132749.9 | MTL5 | 0.6311 | 0.748674 | 7.016603 | 6.51E-12 | 6.29E-11 | 15.72426 |
| ENSG00000075213.9 | SEMA3A | 0.631052 | 1.360686 | 4.386967 | 1.37E-05 | 6.51E-05 | 1.577937 |
| ENSG00000137501.15 | SYTL2 | 0.630217 | 2.092384 | 5.792222 | 1.15E-08 | 8.02E-08 | 8.414219 |
| ENSG00000186364.10 | NUDT17 | 0.630055 | 1.714142 | 8.768618 | 2.11E-17 | 3.25E-16 | 28.1721 |
| ENSG00000159210.8 | SNF8 | 0.630018 | 3.708383 | 11.80363 | 6.68E-29 | 2.26E-27 | 54.41008 |
| ENSG00000090615.11 | GOLGA3 | 0.630005 | 3.63646 | 8.024026 | 5.92E-15 | 7.48E-14 | 22.61198 |
| ENSG00000168090.8 | COPS6 | 0.629988 | 5.081588 | 9.047889 | 2.32E-18 | 3.85E-17 | 30.35463 |
| ENSG00000167106.10 | FAM102A | 0.629795 | 3.966688 | 7.00651 | 6.96E-12 | 6.71E-11 | 15.65912 |
| ENSG00000168298.5 | HIST1H1E | 0.629692 | 0.705098 | 4.427471 | 1.14E-05 | 5.49E-05 | 1.750586 |
| ENSG00000058799.12 | YIPF1 | 0.629675 | 4.32423 | 11.51507 | 1.03E-27 | 3.21E-26 | 51.69666 |
| ENSG00000167695.13 | FAM57A | 0.629508 | 3.278271 | 7.419826 | 4.31E-13 | 4.67E-12 | 18.39021 |
| ENSG00000165188.12 | RNF183 | 0.629033 | 0.642315 | 5.473174 | 6.65E-08 | 4.25E-07 | 6.711964 |
| ENSG00000071859.13 | FAM50A | 0.628984 | 5.046691 | 7.820656 | 2.58E-14 | 3.09E-13 | 21.16118 |
| ENSG00000168754.12 | FAM178B | 0.62863 | 0.720128 | 7.095345 | 3.87E-12 | 3.81E-11 | 16.23511 |
| ENSG00000174109.4 | C16orf91 | 0.628527 | 3.567676 | 8.504217 | 1.63E-16 | 2.33E-15 | 26.15392 |
| ENSG00000102225.14 | CDK16 | 0.628268 | 4.089471 | 8.888842 | 8.21E-18 | 1.31E-16 | 29.10534 |
| ENSG00000167085.10 | PHB | 0.628208 | 4.806924 | 10.45537 | 1.63E-23 | 3.89E-22 | 42.10185 |
| ENSG00000163467.10 | TSACC | 0.628165 | 0.683374 | 9.714595 | 9.74E-21 | 1.90E-19 | 35.76837 |
| ENSG00000164951.14 | PDP1 | 0.628014 | 3.074447 | 6.31007 | 5.63E-10 | 4.50E-09 | 11.35813 |
| ENSG00000131844.14 | MCCC2 | 0.62795 | 3.728569 | 9.129136 | 1.21E-18 | 2.05E-17 | 30.9992 |
| ENSG00000115677.15 | HDLBP | 0.627746 | 5.843657 | 9.415833 | 1.17E-19 | 2.13E-18 | 33.30765 |
| ENSG00000079393.19 | DUSP13 | 0.6276 | 0.585385 | 5.073114 | 5.31E-07 | 3.04E-06 | 4.700575 |
| ENSG00000178035.10 | IMPDH2 | 0.627587 | 5.530623 | 8.276041 | 9.17E-16 | 1.24E-14 | 24.45074 |
| ENSG00000149968.10 | MMP3 | 0.627578 | 0.656353 | 5.495145 | 5.91E-08 | 3.80E-07 | 6.82642 |
| ENSG00000164405.9 | UQCRQ | 0.627521 | 5.819067 | 7.012604 | 6.69E-12 | 6.45E-11 | 15.69844 |
| ENSG00000122034.11 | GTF3A | 0.627301 | 4.979378 | 7.844446 | 2.18E-14 | 2.63E-13 | 21.32936 |
| ENSG00000237289.8 | CKMT1B | 0.627089 | 0.717948 | 6.746303 | 3.75E-11 | 3.38E-10 | 14.00726 |
| ENSG00000104852.13 | SNRNP70 | 0.626874 | 4.999709 | 7.24821 | 1.39E-12 | 1.43E-11 | 17.24042 |
| ENSG00000138095.17 | LRPPRC | 0.626493 | 3.989618 | 9.427176 | 1.07E-19 | 1.94E-18 | 33.40007 |
| ENSG00000121621.6 | KIF18A | 0.626346 | 1.224378 | 8.283681 | 8.66E-16 | 1.17E-14 | 24.50718 |
| ENSG00000095932.6 | SMIM24 | 0.626339 | 0.686973 | 4.512314 | 7.80E-06 | 3.83E-05 | 2.117026 |
| ENSG00000131475.5 | VPS25 | 0.626104 | 4.448315 | 10.45192 | 1.68E-23 | 4.01E-22 | 42.07163 |
| ENSG00000197021.7 | CXorf40B | 0.626 | 2.883645 | 11.39565 | 3.14E-27 | 9.55E-26 | 50.58581 |
| ENSG00000149136.6 | SSRP1 | 0.625966 | 4.794609 | 9.71778 | 9.48E-21 | 1.85E-19 | 35.7949 |
| ENSG00000096070.18 | BRPF3 | 0.625653 | 3.451681 | 7.258416 | 1.30E-12 | 1.34E-11 | 17.30818 |
| ENSG00000116062.13 | MSH6 | 0.625398 | 2.780202 | 8.77615 | 1.99E-17 | 3.07E-16 | 28.23029 |
| ENSG00000115207.12 | GTF3C2 | 0.625218 | 3.655027 | 11.95889 | 1.51E-29 | 5.30E-28 | 55.88685 |
| ENSG00000120659.13 | TNFSF11 | 0.62512 | 0.638307 | 6.058982 | 2.50E-09 | 1.87E-08 | 9.903088 |
| ENSG00000132003.8 | ZSWIM4 | 0.625002 | 2.719186 | 7.495062 | 2.57E-13 | 2.83E-12 | 18.90125 |
| ENSG00000169783.11 | LINGO1 | 0.624458 | 0.810009 | 6.132686 | 1.62E-09 | 1.24E-08 | 10.32484 |
| ENSG00000100644.15 | HIF1A | 0.624375 | 5.359141 | 6.182892 | 1.21E-09 | 9.32E-09 | 10.61469 |
| ENSG00000108651.8 | UTP6 | 0.624316 | 3.052911 | 12.59411 | 3.06E-32 | 1.27E-30 | 62.04607 |
| ENSG00000188725.6 | SMIM15 | 0.62425 | 4.528075 | 10.88108 | 3.59E-25 | 9.64E-24 | 45.88395 |
| ENSG00000033050.6 | ABCF2 | 0.623949 | 3.96209 | 11.08417 | 5.63E-26 | 1.58E-24 | 47.723 |
| ENSG00000253293.4 | HOXA10 | 0.623936 | 0.62723 | 5.468449 | 6.82E-08 | 4.36E-07 | 6.687406 |
| ENSG00000228716.5 | DHFR | 0.623925 | 1.8188 | 9.135323 | 1.15E-18 | 1.95E-17 | 31.04845 |
| ENSG00000105821.13 | DNAJC2 | 0.62375 | 2.487601 | 9.740445 | 7.83E-21 | 1.54E-19 | 35.98387 |
| ENSG00000154380.15 | ENAH | 0.623468 | 3.431435 | 7.617191 | 1.09E-13 | 1.24E-12 | 19.73978 |
| ENSG00000143294.13 | PRCC | 0.623011 | 4.281884 | 11.08945 | 5.36E-26 | 1.50E-24 | 47.77113 |
| ENSG00000148843.12 | PDCD11 | 0.622955 | 3.024547 | 8.5436 | 1.21E-16 | 1.74E-15 | 26.45152 |
| ENSG00000088356.5 | PDRG1 | 0.622881 | 3.464809 | 8.729891 | 2.86E-17 | 4.35E-16 | 27.87355 |
| ENSG00000182180.12 | MRPS16 | 0.622688 | 4.764137 | 10.63472 | 3.31E-24 | 8.24E-23 | 43.683 |
| ENSG00000160256.11 | FAM207A | 0.622594 | 3.039127 | 8.534662 | 1.29E-16 | 1.86E-15 | 26.38389 |
| ENSG00000115484.13 | CCT4 | 0.622278 | 5.816137 | 8.592066 | 8.31E-17 | 1.22E-15 | 26.81921 |
| ENSG00000109854.12 | HTATIP2 | 0.622131 | 4.507708 | 6.833654 | 2.14E-11 | 1.97E-10 | 14.55588 |
| ENSG00000035687.9 | ADSS | 0.621975 | 4.448947 | 10.49955 | 1.10E-23 | 2.66E-22 | 42.48969 |
| ENSG00000116698.19 | SMG7 | 0.621555 | 3.850511 | 10.05457 | 5.39E-22 | 1.16E-20 | 38.63493 |
| ENSG00000179950.12 | PUF60 | 0.621417 | 4.824889 | 8.427777 | 2.92E-16 | 4.10E-15 | 25.57933 |
| ENSG00000177602.5 | GSG2 | 0.621391 | 0.873232 | 9.109444 | 1.42E-18 | 2.38E-17 | 30.84258 |
| ENSG00000087502.16 | ERGIC2 | 0.621227 | 3.389008 | 8.885777 | 8.41E-18 | 1.34E-16 | 29.08143 |
| ENSG00000169992.8 | NLGN2 | 0.620759 | 2.352746 | 6.433713 | 2.65E-10 | 2.19E-09 | 12.09349 |
| ENSG00000148248.12 | SURF4 | 0.620578 | 6.640021 | 11.65567 | 2.72E-28 | 8.91E-27 | 53.01357 |
| ENSG00000104907.11 | TRMT1 | 0.620569 | 3.166511 | 8.538298 | 1.26E-16 | 1.81E-15 | 26.41139 |
| ENSG00000110063.7 | DCPS | 0.620487 | 2.71472 | 10.07136 | 4.66E-22 | 1.00E-20 | 38.77826 |
| ENSG00000146232.13 | NFKBIE | 0.620482 | 3.434432 | 7.128666 | 3.10E-12 | 3.08E-11 | 16.45272 |
| ENSG00000213024.9 | NUP62 | 0.620454 | 3.709425 | 10.37724 | 3.24E-23 | 7.55E-22 | 41.41881 |
| ENSG00000115946.6 | PNO1 | 0.620313 | 3.490764 | 8.904125 | 7.28E-18 | 1.17E-16 | 29.22466 |
| ENSG00000167291.14 | TBC1D16 | 0.620302 | 2.718867 | 6.861523 | 1.79E-11 | 1.66E-10 | 14.73218 |
| ENSG00000140548.8 | ZNF710 | 0.620193 | 3.19561 | 4.939 | 1.03E-06 | 5.71E-06 | 4.057429 |
| ENSG00000082213.16 | C5orf22 | 0.620192 | 3.190808 | 8.828578 | 1.32E-17 | 2.07E-16 | 28.63634 |
| ENSG00000111639.6 | MRPL51 | 0.620151 | 5.529044 | 8.795415 | 1.71E-17 | 2.66E-16 | 28.37928 |
| ENSG00000197265.7 | GTF2E2 | 0.619992 | 3.617052 | 9.406721 | 1.26E-19 | 2.29E-18 | 33.23348 |
| ENSG00000138382.12 | METTL5 | 0.619931 | 3.2239 | 10.22259 | 1.26E-22 | 2.83E-21 | 40.07693 |
| ENSG00000146904.7 | EPHA1 | 0.619886 | 2.281599 | 6.164993 | 1.34E-09 | 1.03E-08 | 10.51112 |
| ENSG00000133316.14 | WDR74 | 0.619718 | 2.854294 | 11.92465 | 2.10E-29 | 7.31E-28 | 55.56017 |
| ENSG00000130816.13 | DNMT1 | 0.619626 | 3.260576 | 7.180452 | 2.19E-12 | 2.21E-11 | 16.79261 |
| ENSG00000119787.12 | ATL2 | 0.618703 | 4.164535 | 7.886964 | 1.60E-14 | 1.96E-13 | 21.63093 |
| ENSG00000139131.11 | YARS2 | 0.618525 | 3.087106 | 8.827989 | 1.33E-17 | 2.08E-16 | 28.63177 |
| ENSG00000173110.7 | HSPA6 | 0.618433 | 2.29242 | 4.200205 | 3.10E-05 | 0.000139486 | 0.801074 |
| ENSG00000074842.6 | MYDGF | 0.618395 | 6.027275 | 8.419364 | 3.11E-16 | 4.36E-15 | 25.51634 |
| ENSG00000147454.12 | SLC25A37 | 0.618131 | 3.168916 | 5.464231 | 6.97E-08 | 4.45E-07 | 6.665497 |
| ENSG00000090621.12 | PABPC4 | 0.618039 | 4.701969 | 9.620614 | 2.14E-20 | 4.07E-19 | 34.98835 |
| ENSG00000108826.14 | MRPL27 | 0.617874 | 3.697448 | 9.427651 | 1.06E-19 | 1.94E-18 | 33.40394 |
| ENSG00000111110.10 | PPM1H | 0.617681 | 2.097251 | 5.152242 | 3.56E-07 | 2.08E-06 | 5.087415 |
| ENSG00000171863.11 | RPS7 | 0.617602 | 6.311479 | 6.20239 | 1.07E-09 | 8.35E-09 | 10.72781 |
| ENSG00000084731.12 | KIF3C | 0.617524 | 1.809718 | 5.468032 | 6.83E-08 | 4.36E-07 | 6.685239 |
| ENSG00000111581.8 | NUP107 | 0.617402 | 2.599563 | 7.656852 | 8.27E-14 | 9.49E-13 | 20.01447 |
| ENSG00000135775.12 | COG2 | 0.617373 | 2.283344 | 13.14346 | 1.25E-34 | 6.03E-33 | 67.51596 |
| ENSG00000111671.8 | SPSB2 | 0.617339 | 3.3119 | 5.651628 | 2.52E-08 | 1.69E-07 | 7.653473 |
| ENSG00000177628.14 | GBA | 0.617104 | 4.164636 | 8.87463 | 9.19E-18 | 1.46E-16 | 28.99452 |
| ENSG00000163811.10 | WDR43 | 0.616965 | 3.635854 | 8.56867 | 9.95E-17 | 1.45E-15 | 26.64152 |
| ENSG00000135925.7 | WNT10A | 0.616916 | 1.110795 | 4.871422 | 1.44E-06 | 7.78E-06 | 3.739346 |
| ENSG00000148908.13 | RGS10 | 0.616869 | 4.427932 | 5.286202 | 1.79E-07 | 1.09E-06 | 5.754712 |
| ENSG00000170540.13 | ARL6IP1 | 0.616813 | 5.86103 | 8.449547 | 2.47E-16 | 3.49E-15 | 25.74257 |
| ENSG00000232434.2 | C9orf172 | 0.616627 | 1.025846 | 9.307673 | 2.84E-19 | 5.04E-18 | 32.4306 |
| ENSG00000230124.5 | LHX4-AS1 | 0.616383 | 2.549935 | 12.54971 | 4.75E-32 | 1.95E-30 | 61.60955 |
| ENSG00000063241.6 | ISOC2 | 0.616338 | 3.372144 | 8.605808 | 7.48E-17 | 1.10E-15 | 26.92376 |
| ENSG00000042286.13 | AIFM2 | 0.616244 | 2.72836 | 7.021282 | 6.32E-12 | 6.11E-11 | 15.75448 |
| ENSG00000161692.16 | DBF4B | 0.615634 | 1.151204 | 9.693422 | 1.16E-20 | 2.26E-19 | 35.59217 |
| ENSG00000135119.13 | RNFT2 | 0.615488 | 0.834854 | 7.178838 | 2.22E-12 | 2.23E-11 | 16.78199 |
| ENSG00000148735.13 | PLEKHS1 | 0.615484 | 1.855419 | 3.119534 | 0.001903478 | 0.006235609 | -3.06216 |
| ENSG00000105202.6 | FBL | 0.615229 | 5.525986 | 8.43547 | 2.75E-16 | 3.88E-15 | 25.63698 |
| ENSG00000179674.3 | ARL14 | 0.6152 | 0.660765 | 3.883477 | 0.000115116 | 0.000474668 | -0.44359 |
| ENSG00000100253.11 | MIOX | 0.61507 | 0.613335 | 5.76411 | 1.35E-08 | 9.32E-08 | 8.260776 |
| ENSG00000168653.9 | NDUFS5 | 0.614951 | 7.567657 | 7.95025 | 1.01E-14 | 1.26E-13 | 22.08224 |
| ENSG00000165238.15 | WNK2 | 0.614871 | 0.869641 | 6.518438 | 1.57E-10 | 1.33E-09 | 12.6045 |
| ENSG00000187735.11 | TCEA1 | 0.614657 | 4.247928 | 8.262002 | 1.02E-15 | 1.37E-14 | 24.34713 |
| ENSG00000062582.12 | MRPS24 | 0.61459 | 2.173152 | 7.783439 | 3.37E-14 | 3.99E-13 | 20.89892 |
| ENSG00000064655.17 | EYA2 | 0.614554 | 1.659277 | 3.166167 | 0.00162765 | 0.00541324 | -2.91806 |
| ENSG00000198522.12 | GPN1 | 0.614125 | 3.733305 | 9.117935 | 1.32E-18 | 2.23E-17 | 30.91008 |
| ENSG00000182158.13 | CREB3L2 | 0.614046 | 4.133345 | 6.880665 | 1.58E-11 | 1.47E-10 | 14.85362 |
| ENSG00000172613.6 | RAD9A | 0.614009 | 2.373906 | 7.895053 | 1.51E-14 | 1.85E-13 | 21.68846 |
| ENSG00000130054.4 | FAM155B | 0.61387 | 0.666345 | 7.310364 | 9.12E-13 | 9.61E-12 | 17.65427 |
| ENSG00000124574.13 | ABCC10 | 0.613765 | 2.406771 | 9.676865 | 1.34E-20 | 2.58E-19 | 35.45457 |
| ENSG00000147123.10 | NDUFB11 | 0.612674 | 5.818989 | 8.173937 | 1.96E-15 | 2.58E-14 | 23.70036 |
| ENSG00000134323.10 | MYCN | 0.61264 | 0.734945 | 3.896988 | 0.00010904 | 0.000451439 | -0.39238 |
| ENSG00000159479.15 | MED8 | 0.612602 | 4.140163 | 11.59469 | 4.85E-28 | 1.55E-26 | 52.44123 |
| ENSG00000157193.13 | LRP8 | 0.612463 | 1.352047 | 6.837096 | 2.10E-11 | 1.93E-10 | 14.57763 |
| ENSG00000083093.8 | PALB2 | 0.612423 | 2.079473 | 12.58158 | 3.47E-32 | 1.44E-30 | 61.92273 |
| ENSG00000134253.8 | TRIM45 | 0.612222 | 1.183519 | 8.509952 | 1.56E-16 | 2.24E-15 | 26.19719 |
| ENSG00000172115.7 | CYCS | 0.612182 | 4.704004 | 6.8651 | 1.75E-11 | 1.62E-10 | 14.75485 |
| ENSG00000182979.16 | MTA1 | 0.612053 | 3.03315 | 7.65499 | 8.38E-14 | 9.61E-13 | 20.00155 |
| ENSG00000196233.10 | LCOR | 0.61198 | 1.798502 | 9.384543 | 1.51E-19 | 2.73E-18 | 33.05317 |
| ENSG00000165209.17 | STRBP | 0.611856 | 2.361069 | 9.822804 | 3.91E-21 | 7.90E-20 | 36.67318 |
| ENSG00000162931.10 | TRIM17 | 0.611671 | 0.946042 | 5.258247 | 2.06E-07 | 1.25E-06 | 5.614173 |
| ENSG00000006747.13 | SCIN | 0.611506 | 1.781656 | 3.847152 | 0.000133077 | 0.000543294 | -0.58043 |
| ENSG00000141279.14 | NPEPPS | 0.611295 | 3.511064 | 9.21067 | 6.25E-19 | 1.08E-17 | 31.65035 |
| ENSG00000116985.9 | BMP8B | 0.611289 | 1.308564 | 7.806503 | 2.86E-14 | 3.41E-13 | 21.06134 |
| ENSG00000170260.7 | ZNF212 | 0.611135 | 2.772582 | 10.01929 | 7.30E-22 | 1.55E-20 | 38.33426 |
| ENSG00000243927.4 | MRPS6 | 0.61081 | 3.36411 | 6.899871 | 1.40E-11 | 1.31E-10 | 14.97576 |
| ENSG00000196262.12 | PPIA | 0.610753 | 6.293986 | 8.673036 | 4.45E-17 | 6.64E-16 | 27.43706 |
| ENSG00000172586.7 | CHCHD1 | 0.6107 | 4.401556 | 9.108937 | 1.42E-18 | 2.39E-17 | 30.83855 |
| ENSG00000025708.11 | TYMP | 0.610682 | 5.285926 | 5.17443 | 3.18E-07 | 1.87E-06 | 5.196865 |
| ENSG00000105865.9 | DUS4L | 0.610664 | 1.407802 | 11.08373 | 5.65E-26 | 1.58E-24 | 47.71905 |
| ENSG00000162613.15 | FUBP1 | 0.610642 | 4.121753 | 10.05898 | 5.19E-22 | 1.12E-20 | 38.67251 |
| ENSG00000197136.4 | PCNXL3 | 0.610548 | 3.566837 | 8.554149 | 1.11E-16 | 1.61E-15 | 26.53142 |
| ENSG00000147144.11 | CCDC120 | 0.610473 | 2.42202 | 7.685249 | 6.77E-14 | 7.82E-13 | 20.21186 |
| ENSG00000116661.9 | FBXO2 | 0.610328 | 2.77656 | 3.587034 | 0.000363379 | 0.001372182 | -1.5246 |
| ENSG00000093217.8 | XYLB | 0.609963 | 0.9919 | 10.93541 | 2.19E-25 | 5.96E-24 | 46.37375 |
| ENSG00000179546.4 | HTR1D | 0.609947 | 0.772273 | 5.424689 | 8.61E-08 | 5.44E-07 | 6.460849 |
| ENSG00000166908.16 | PIP4K2C | 0.609938 | 4.578829 | 7.626869 | 1.02E-13 | 1.16E-12 | 19.80671 |
| ENSG00000160685.12 | ZBTB7B | 0.609907 | 4.266492 | 7.640717 | 9.27E-14 | 1.06E-12 | 19.90258 |
| ENSG00000187801.13 | ZFP69B | 0.609903 | 0.93519 | 10.94104 | 2.08E-25 | 5.67E-24 | 46.4246 |
| ENSG00000197958.11 | RPL12 | 0.609414 | 7.856142 | 6.557171 | 1.24E-10 | 1.06E-09 | 12.84003 |
| ENSG00000184979.9 | USP18 | 0.609149 | 2.518709 | 6.6339 | 7.65E-11 | 6.67E-10 | 13.31014 |
| ENSG00000048140.16 | TSPAN17 | 0.608963 | 3.601507 | 9.213618 | 6.11E-19 | 1.06E-17 | 31.67397 |
| ENSG00000164509.12 | IL31RA | 0.60894 | 0.615513 | 6.437453 | 2.59E-10 | 2.15E-09 | 12.11592 |
| ENSG00000120526.9 | NUDCD1 | 0.608882 | 2.72588 | 7.548745 | 1.77E-13 | 1.98E-12 | 19.26847 |
| ENSG00000148362.9 | C9orf142 | 0.608879 | 4.233324 | 7.221435 | 1.67E-12 | 1.70E-11 | 17.06305 |
| ENSG00000108688.10 | CCL7 | 0.608766 | 0.855986 | 5.328271 | 1.43E-07 | 8.82E-07 | 5.967475 |
| ENSG00000012211.11 | PRICKLE3 | 0.608551 | 2.282219 | 9.627402 | 2.02E-20 | 3.85E-19 | 35.0445 |
| ENSG00000244005.11 | NFS1 | 0.608535 | 2.324308 | 12.123 | 3.10E-30 | 1.13E-28 | 57.46026 |
| ENSG00000143502.13 | SUSD4 | 0.608252 | 1.781401 | 4.277565 | 2.22E-05 | 0.000101961 | 1.119021 |
| ENSG00000196704.10 | AMZ2 | 0.608238 | 3.976505 | 10.77474 | 9.40E-25 | 2.44E-23 | 44.92987 |
| ENSG00000100941.7 | PNN | 0.608187 | 4.423822 | 6.489068 | 1.89E-10 | 1.58E-09 | 12.4267 |
| ENSG00000132541.9 | HRSP12 | 0.608172 | 3.430234 | 7.390986 | 5.26E-13 | 5.65E-12 | 18.19544 |
| ENSG00000167114.11 | SLC27A4 | 0.608102 | 3.691689 | 8.445665 | 2.55E-16 | 3.59E-15 | 25.71344 |
| ENSG00000165244.6 | ZNF367 | 0.608093 | 1.318371 | 7.623618 | 1.05E-13 | 1.19E-12 | 19.78422 |
| ENSG00000157483.7 | MYO1E | 0.608018 | 2.939217 | 6.151532 | 1.45E-09 | 1.11E-08 | 10.4334 |
| ENSG00000196839.11 | ADA | 0.607956 | 1.918817 | 6.394389 | 3.37E-10 | 2.76E-09 | 11.85827 |
| ENSG00000162745.9 | OLFML2B | 0.607721 | 3.174899 | 4.685401 | 3.50E-06 | 1.80E-05 | 2.884632 |
| ENSG00000153214.8 | TMEM87B | 0.6076 | 3.688308 | 10.32413 | 5.17E-23 | 1.19E-21 | 40.95639 |
| ENSG00000111711.8 | GOLT1B | 0.607383 | 4.043537 | 6.460508 | 2.25E-10 | 1.88E-09 | 12.25448 |
| ENSG00000147162.12 | OGT | 0.607327 | 4.087924 | 5.491044 | 6.04E-08 | 3.88E-07 | 6.805027 |
| ENSG00000172197.10 | MBOAT1 | 0.607182 | 3.034255 | 5.850532 | 8.29E-09 | 5.86E-08 | 8.734602 |
| ENSG00000197858.9 | GPAA1 | 0.606923 | 5.325776 | 7.29032 | 1.05E-12 | 1.09E-11 | 17.52049 |
| ENSG00000184857.7 | TMEM186 | 0.606917 | 2.674129 | 11.62991 | 3.48E-28 | 1.13E-26 | 52.77156 |
| ENSG00000189046.9 | ALKBH2 | 0.606622 | 2.494565 | 8.144075 | 2.45E-15 | 3.19E-14 | 23.48229 |
| ENSG00000196711.7 | FAM150A | 0.60644 | 0.88615 | 5.535033 | 4.76E-08 | 3.10E-07 | 7.035259 |
| ENSG00000213185.5 | FAM24B | 0.606244 | 1.200715 | 8.244372 | 1.16E-15 | 1.56E-14 | 24.21722 |
| ENSG00000174996.10 | KLC2 | 0.606068 | 2.887372 | 8.270328 | 9.57E-16 | 1.29E-14 | 24.40856 |
| ENSG00000117335.17 | CD46 | 0.605989 | 5.750968 | 7.179694 | 2.20E-12 | 2.22E-11 | 16.78762 |
| ENSG00000147684.6 | NDUFB9 | 0.605905 | 5.517279 | 6.261846 | 7.52E-10 | 5.94E-09 | 11.07468 |
| ENSG00000197362.12 | ZNF786 | 0.605796 | 1.823465 | 10.71653 | 1.59E-24 | 4.06E-23 | 44.41016 |
| ENSG00000119777.17 | TMEM214 | 0.60563 | 4.807152 | 10.58123 | 5.33E-24 | 1.31E-22 | 43.20955 |
| ENSG00000176208.7 | ATAD5 | 0.605546 | 1.019235 | 9.887589 | 2.25E-21 | 4.63E-20 | 37.2183 |
| ENSG00000105372.5 | RPS19 | 0.605513 | 7.453693 | 6.250022 | 8.08E-10 | 6.35E-09 | 11.00546 |
| ENSG00000156170.11 | NDUFAF6 | 0.605426 | 1.861895 | 8.557507 | 1.08E-16 | 1.57E-15 | 26.55687 |
| ENSG00000171858.16 | RPS21 | 0.604638 | 8.2852 | 5.611799 | 3.14E-08 | 2.08E-07 | 7.440996 |
| ENSG00000103495.12 | MAZ | 0.604605 | 3.852811 | 8.541315 | 1.23E-16 | 1.77E-15 | 26.43422 |
| ENSG00000008283.14 | CYB561 | 0.604546 | 4.853343 | 7.717508 | 5.39E-14 | 6.27E-13 | 20.4368 |
| ENSG00000144028.13 | SNRNP200 | 0.604461 | 4.726392 | 8.262633 | 1.01E-15 | 1.36E-14 | 24.35178 |
| ENSG00000165792.16 | METTL17 | 0.60421 | 3.004578 | 8.404099 | 3.49E-16 | 4.88E-15 | 25.40217 |
| ENSG00000186806.5 | VSIG10L | 0.604073 | 1.307621 | 4.60222 | 5.16E-06 | 2.60E-05 | 2.512395 |
| ENSG00000184831.12 | APOO | 0.604009 | 3.381784 | 7.873471 | 1.77E-14 | 2.15E-13 | 21.53509 |
| ENSG00000109103.10 | UNC119 | 0.603889 | 2.650562 | 10.60727 | 4.22E-24 | 1.05E-22 | 43.43981 |
| ENSG00000085978.20 | ATG16L1 | 0.603786 | 2.871601 | 11.91295 | 2.35E-29 | 8.15E-28 | 55.44869 |
| ENSG00000088682.12 | COQ9 | 0.603706 | 3.579614 | 9.679855 | 1.30E-20 | 2.52E-19 | 35.4794 |
| ENSG00000066379.13 | ZNRD1 | 0.603634 | 2.533449 | 10.1333 | 2.73E-22 | 6.00E-21 | 39.3086 |
| ENSG00000081692.11 | JMJD4 | 0.603525 | 2.307367 | 10.89244 | 3.24E-25 | 8.72E-24 | 45.98621 |
| ENSG00000051341.12 | POLQ | 0.603347 | 0.639159 | 9.484327 | 6.65E-20 | 1.23E-18 | 33.86689 |
| ENSG00000130340.13 | SNX9 | 0.603244 | 3.900119 | 8.213261 | 1.46E-15 | 1.95E-14 | 23.98849 |
| ENSG00000052749.12 | RRP12 | 0.603065 | 2.73794 | 8.187801 | 1.77E-15 | 2.34E-14 | 23.80182 |
| ENSG00000109511.9 | ANXA10 | 0.603006 | 0.592243 | 3.002872 | 0.002792446 | 0.008849846 | -3.4136 |
| ENSG00000102743.13 | SLC25A15 | 0.602883 | 2.005634 | 7.452411 | 3.45E-13 | 3.77E-12 | 18.61102 |
| ENSG00000116213.14 | WRAP73 | 0.602853 | 2.073053 | 12.00871 | 9.34E-30 | 3.33E-28 | 56.36315 |
| ENSG00000170515.12 | PA2G4 | 0.602827 | 5.053266 | 9.426309 | 1.07E-19 | 1.96E-18 | 33.393 |
| ENSG00000128203.6 | ASPHD2 | 0.602766 | 1.337774 | 6.716837 | 4.53E-11 | 4.05E-10 | 13.82354 |
| ENSG00000214078.10 | CPNE1 | 0.602652 | 4.970525 | 6.17364 | 1.27E-09 | 9.83E-09 | 10.56112 |
| ENSG00000143740.13 | SNAP47 | 0.602568 | 2.664979 | 12.62548 | 2.25E-32 | 9.43E-31 | 62.35487 |
| ENSG00000073578.15 | SDHA | 0.602279 | 3.910961 | 8.338875 | 5.72E-16 | 7.85E-15 | 24.91615 |
| ENSG00000101049.13 | SGK2 | 0.602158 | 0.756442 | 6.422121 | 2.85E-10 | 2.35E-09 | 12.02402 |
| ENSG00000038002.7 | AGA | 0.601961 | 3.67502 | 6.319447 | 5.32E-10 | 4.26E-09 | 11.41346 |
| ENSG00000130675.13 | MNX1 | 0.601918 | 0.584233 | 8.745973 | 2.52E-17 | 3.85E-16 | 27.99741 |
| ENSG00000130829.16 | DUSP9 | 0.601817 | 0.595967 | 5.286378 | 1.78E-07 | 1.09E-06 | 5.755598 |
| ENSG00000014138.7 | POLA2 | 0.601554 | 2.206827 | 8.834795 | 1.26E-17 | 1.97E-16 | 28.68461 |
| ENSG00000181038.12 | METTL23 | 0.601421 | 3.695687 | 11.84586 | 4.46E-29 | 1.53E-27 | 54.81062 |
| ENSG00000213918.9 | DNASE1 | 0.601051 | 1.297586 | 8.93303 | 5.79E-18 | 9.35E-17 | 29.45075 |
| ENSG00000131591.16 | C1orf159 | 0.600975 | 1.560321 | 9.401368 | 1.32E-19 | 2.39E-18 | 33.18994 |
| ENSG00000184402.13 | SS18L1 | 0.600972 | 2.398646 | 7.255243 | 1.33E-12 | 1.37E-11 | 17.28711 |
| ENSG00000177169.8 | ULK1 | 0.600925 | 3.243166 | 7.292828 | 1.03E-12 | 1.08E-11 | 17.53722 |
| ENSG00000103152.10 | MPG | 0.600789 | 4.276601 | 7.080909 | 4.26E-12 | 4.19E-11 | 16.14109 |
| ENSG00000183840.6 | GPR39 | 0.600769 | 2.269952 | 5.66907 | 2.29E-08 | 1.54E-07 | 7.746944 |
| ENSG00000100297.14 | MCM5 | 0.600656 | 3.068919 | 7.348811 | 7.02E-13 | 7.47E-12 | 17.91173 |
| ENSG00000102178.11 | UBL4A | 0.600537 | 4.574477 | 8.294544 | 7.98E-16 | 1.08E-14 | 24.58751 |
| ENSG00000162782.14 | TDRD5 | 0.600096 | 0.63063 | 5.746059 | 1.49E-08 | 1.02E-07 | 8.162598 |
| ENSG00000160679.11 | CHTOP | 0.599788 | 4.109888 | 11.04515 | 8.05E-26 | 2.24E-24 | 47.36799 |
| ENSG00000128563.12 | PRKRIP1 | 0.599771 | 3.116079 | 8.918331 | 6.50E-18 | 1.05E-16 | 29.33571 |
| ENSG00000111300.8 | NAA25 | 0.599661 | 1.946116 | 10.1091 | 3.37E-22 | 7.32E-21 | 39.1011 |
| ENSG00000111196.8 | MAGOHB | 0.599398 | 2.040727 | 9.684737 | 1.25E-20 | 2.42E-19 | 35.51997 |
| ENSG00000258429.1 | PDF | 0.59939 | 1.269806 | 10.80299 | 7.29E-25 | 1.91E-23 | 45.18268 |
| ENSG00000163479.12 | SSR2 | 0.599329 | 5.574112 | 8.216413 | 1.43E-15 | 1.90E-14 | 24.01163 |
| ENSG00000130876.10 | SLC7A10 | 0.599284 | 0.590341 | 4.922779 | 1.12E-06 | 6.16E-06 | 3.980714 |
| ENSG00000165458.12 | INPPL1 | 0.599068 | 4.552394 | 6.818238 | 2.37E-11 | 2.17E-10 | 14.45863 |
| ENSG00000123388.4 | HOXC11 | 0.599001 | 0.547138 | 5.213594 | 2.60E-07 | 1.55E-06 | 5.391094 |
| ENSG00000149658.16 | YTHDF1 | 0.598871 | 4.637072 | 9.79416 | 4.98E-21 | 9.99E-20 | 36.43298 |
| ENSG00000171311.11 | EXOSC1 | 0.598589 | 3.0221 | 12.34862 | 3.44E-31 | 1.33E-29 | 59.64392 |
| ENSG00000213339.7 | QTRT1 | 0.598473 | 3.031918 | 7.490387 | 2.65E-13 | 2.93E-12 | 18.86937 |
| ENSG00000120539.13 | MASTL | 0.598359 | 2.330652 | 7.811936 | 2.75E-14 | 3.28E-13 | 21.09965 |
| ENSG00000114956.18 | DGUOK | 0.598198 | 4.818913 | 9.922721 | 1.67E-21 | 3.47E-20 | 37.51497 |
| ENSG00000129484.12 | PARP2 | 0.597965 | 2.584205 | 8.45365 | 2.40E-16 | 3.39E-15 | 25.77337 |
| ENSG00000205208.4 | C4orf46 | 0.597644 | 1.924178 | 8.173152 | 1.97E-15 | 2.59E-14 | 23.69462 |
| ENSG00000198298.11 | ZNF485 | 0.597425 | 1.177692 | 11.93177 | 1.96E-29 | 6.84E-28 | 55.62809 |
| ENSG00000090861.14 | AARS | 0.597288 | 5.192008 | 7.824757 | 2.51E-14 | 3.01E-13 | 21.19015 |
| ENSG00000141367.10 | CLTC | 0.597163 | 5.579293 | 9.157587 | 9.61E-19 | 1.64E-17 | 31.22593 |
| ENSG00000100836.9 | PABPN1 | 0.597032 | 4.401808 | 7.501285 | 2.46E-13 | 2.72E-12 | 18.9437 |
| ENSG00000167977.7 | KCTD5 | 0.596884 | 3.806685 | 7.862977 | 1.90E-14 | 2.31E-13 | 21.46063 |
| ENSG00000196950.12 | SLC39A10 | 0.596773 | 2.754506 | 7.767126 | 3.78E-14 | 4.46E-13 | 20.78429 |
| ENSG00000132507.16 | EIF5A | 0.596732 | 6.88455 | 7.660901 | 8.04E-14 | 9.23E-13 | 20.04258 |
| ENSG00000111674.7 | ENO2 | 0.596606 | 3.853539 | 4.388853 | 1.36E-05 | 6.46E-05 | 1.585943 |
| ENSG00000014164.6 | ZC3H3 | 0.596589 | 3.258095 | 7.84026 | 2.24E-14 | 2.70E-13 | 21.29973 |
| ENSG00000205155.6 | PSENEN | 0.596536 | 4.803886 | 6.646711 | 7.06E-11 | 6.18E-10 | 13.38909 |
| ENSG00000198176.11 | TFDP1 | 0.596515 | 4.358807 | 7.303871 | 9.53E-13 | 1.00E-11 | 17.6109 |
| ENSG00000183682.7 | BMP8A | 0.59641 | 0.750977 | 8.132467 | 2.67E-15 | 3.47E-14 | 23.39769 |
| ENSG00000169957.9 | ZNF768 | 0.596099 | 4.187416 | 7.8204 | 2.59E-14 | 3.10E-13 | 21.15938 |
| ENSG00000134121.8 | CHL1 | 0.596076 | 1.504132 | 3.952052 | 8.73E-05 | 0.000367168 | -0.18192 |
| ENSG00000105258.7 | POLR2I | 0.595892 | 4.112899 | 7.420145 | 4.31E-13 | 4.66E-12 | 18.39237 |
| ENSG00000068796.15 | KIF2A | 0.59586 | 2.730102 | 7.221453 | 1.67E-12 | 1.70E-11 | 17.06317 |
| ENSG00000146281.5 | PM20D2 | 0.595512 | 2.526819 | 7.15292 | 2.64E-12 | 2.64E-11 | 16.61165 |
| ENSG00000180346.3 | TIGD2 | 0.595492 | 2.09971 | 8.385711 | 4.02E-16 | 5.58E-15 | 25.26485 |
| ENSG00000188368.8 | PRR19 | 0.595412 | 0.762701 | 9.460451 | 8.10E-20 | 1.49E-18 | 33.67161 |
| ENSG00000116001.14 | TIA1 | 0.595381 | 3.596998 | 7.135093 | 2.97E-12 | 2.96E-11 | 16.49479 |
| ENSG00000126067.10 | PSMB2 | 0.595112 | 4.724185 | 10.19713 | 1.57E-22 | 3.51E-21 | 39.85742 |
| ENSG00000111605.15 | CPSF6 | 0.595097 | 3.559378 | 10.99702 | 1.25E-25 | 3.44E-24 | 46.9311 |
| ENSG00000120253.12 | NUP43 | 0.594995 | 3.02734 | 10.78098 | 8.89E-25 | 2.32E-23 | 44.98569 |
| ENSG00000171604.10 | CXXC5 | 0.594951 | 3.821413 | 6.801379 | 2.64E-11 | 2.41E-10 | 14.35247 |
| ENSG00000121957.11 | GPSM2 | 0.594925 | 1.712181 | 7.740458 | 4.58E-14 | 5.36E-13 | 20.59731 |
| ENSG00000111775.2 | COX6A1 | 0.594899 | 6.155388 | 6.831802 | 2.17E-11 | 1.99E-10 | 14.54419 |
| ENSG00000171490.11 | RSL1D1 | 0.594884 | 4.794072 | 9.749139 | 7.28E-21 | 1.44E-19 | 36.05645 |
| ENSG00000144481.15 | TRPM8 | 0.594828 | 0.547156 | 4.694546 | 3.36E-06 | 1.73E-05 | 2.925929 |
| ENSG00000136699.18 | SMPD4 | 0.594766 | 3.270736 | 9.501196 | 5.79E-20 | 1.07E-18 | 34.00507 |
| ENSG00000140534.12 | TICRR | 0.594734 | 0.655159 | 9.496854 | 6.00E-20 | 1.11E-18 | 33.96948 |
| ENSG00000160710.14 | ADAR | 0.594272 | 5.748571 | 8.247774 | 1.13E-15 | 1.52E-14 | 24.24227 |
| ENSG00000111726.11 | CMAS | 0.593934 | 4.201606 | 6.80488 | 2.58E-11 | 2.36E-10 | 14.3745 |
| ENSG00000241685.7 | ARPC1A | 0.593847 | 5.763583 | 7.49834 | 2.51E-13 | 2.77E-12 | 18.92361 |
| ENSG00000135697.8 | BCO1 | 0.593709 | 0.751688 | 6.388207 | 3.50E-10 | 2.86E-09 | 11.8214 |
| ENSG00000204576.10 | PRR3 | 0.593605 | 2.221758 | 9.80594 | 4.51E-21 | 9.07E-20 | 36.53171 |
| ENSG00000126368.5 | NR1D1 | 0.593539 | 2.921875 | 5.023968 | 6.79E-07 | 3.84E-06 | 4.463064 |
| ENSG00000162757.4 | C1orf74 | 0.593508 | 1.168825 | 13.74125 | 2.73E-37 | 1.55E-35 | 73.60764 |
| ENSG00000143379.11 | SETDB1 | 0.593307 | 2.959564 | 9.07748 | 1.83E-18 | 3.06E-17 | 30.58889 |
| ENSG00000173465.6 | SSSCA1 | 0.593234 | 3.431242 | 8.912591 | 6.81E-18 | 1.09E-16 | 29.29083 |
| ENSG00000149657.18 | LSM14B | 0.593098 | 3.931521 | 8.06888 | 4.26E-15 | 5.45E-14 | 22.93595 |
| ENSG00000178952.7 | TUFM | 0.593087 | 6.497191 | 10.31545 | 5.58E-23 | 1.28E-21 | 40.88103 |
| ENSG00000183978.7 | COA3 | 0.593018 | 4.963236 | 6.728361 | 4.21E-11 | 3.77E-10 | 13.89532 |
| ENSG00000178860.8 | MSC | 0.592973 | 1.954136 | 5.061129 | 5.64E-07 | 3.22E-06 | 4.642458 |
| ENSG00000169231.12 | THBS3 | 0.59199 | 2.929912 | 6.774223 | 3.14E-11 | 2.85E-10 | 14.18197 |
| ENSG00000172932.13 | ANKRD13D | 0.591701 | 2.732506 | 8.045006 | 5.08E-15 | 6.46E-14 | 22.76333 |
| ENSG00000121690.8 | DEPDC7 | 0.591622 | 0.796543 | 6.111982 | 1.83E-09 | 1.39E-08 | 10.20592 |
| ENSG00000277203.1 | F8A1 | 0.591557 | 2.839894 | 5.764031 | 1.35E-08 | 9.32E-08 | 8.260345 |
| ENSG00000066455.11 | GOLGA5 | 0.591389 | 4.454405 | 10.30884 | 5.92E-23 | 1.36E-21 | 40.8236 |
| ENSG00000123572.15 | NRK | 0.59115 | 0.629392 | 5.192834 | 2.89E-07 | 1.71E-06 | 5.287972 |
| ENSG00000106327.11 | TFR2 | 0.591059 | 0.647458 | 8.719593 | 3.10E-17 | 4.70E-16 | 27.79432 |
| ENSG00000137364.4 | TPMT | 0.590896 | 3.762516 | 9.5117 | 5.30E-20 | 9.86E-19 | 34.0912 |
| ENSG00000115339.12 | GALNT3 | 0.590855 | 3.63793 | 4.01821 | 6.66E-05 | 0.000285205 | 0.074624 |
| ENSG00000178921.12 | PFAS | 0.590611 | 2.266276 | 8.456942 | 2.34E-16 | 3.31E-15 | 25.79809 |
| ENSG00000185615.14 | PDIA2 | 0.590511 | 0.628798 | 3.652648 | 0.000283625 | 0.001092782 | -1.29238 |
| ENSG00000125166.11 | GOT2 | 0.590477 | 4.59153 | 9.378575 | 1.59E-19 | 2.86E-18 | 33.0047 |
| ENSG00000092208.15 | GEMIN2 | 0.590359 | 2.172785 | 6.770896 | 3.21E-11 | 2.91E-10 | 14.16112 |
| ENSG00000160781.14 | PAQR6 | 0.590052 | 1.04736 | 5.896282 | 6.39E-09 | 4.58E-08 | 8.987959 |
| ENSG00000125841.11 | NRSN2 | 0.589716 | 3.884148 | 6.964853 | 9.15E-12 | 8.71E-11 | 15.39112 |
| ENSG00000106080.9 | FKBP14 | 0.589638 | 2.28245 | 7.821137 | 2.57E-14 | 3.08E-13 | 21.16458 |
| ENSG00000140474.11 | ULK3 | 0.589355 | 3.52882 | 8.095109 | 3.51E-15 | 4.53E-14 | 23.12606 |
| ENSG00000212916.4 | MAP10 | 0.58918 | 1.146313 | 7.387045 | 5.40E-13 | 5.80E-12 | 18.16887 |
| ENSG00000135845.8 | PIGC | 0.589029 | 3.001614 | 10.41985 | 2.23E-23 | 5.25E-22 | 41.7909 |
| ENSG00000172663.7 | TMEM134 | 0.588849 | 2.820506 | 8.721268 | 3.06E-17 | 4.64E-16 | 27.80721 |
| ENSG00000228300.12 | C19orf24 | 0.588791 | 3.827674 | 7.319307 | 8.58E-13 | 9.06E-12 | 17.71406 |
| ENSG00000156261.11 | CCT8 | 0.588756 | 5.626777 | 9.447641 | 9.01E-20 | 1.65E-18 | 33.56699 |
| ENSG00000159958.4 | TNFRSF13C | 0.588685 | 1.040966 | 5.367819 | 1.16E-07 | 7.24E-07 | 6.168878 |
| ENSG00000103253.16 | HAGHL | 0.588611 | 1.130874 | 7.150832 | 2.67E-12 | 2.67E-11 | 16.59795 |
| ENSG00000101115.11 | SALL4 | 0.588542 | 0.614025 | 8.398616 | 3.64E-16 | 5.08E-15 | 25.3612 |
| ENSG00000180035.9 | ZNF48 | 0.588485 | 2.303578 | 9.257058 | 4.29E-19 | 7.52E-18 | 32.02272 |
| ENSG00000145293.13 | ENOPH1 | 0.588482 | 4.545303 | 9.774226 | 5.89E-21 | 1.17E-19 | 36.26611 |
| ENSG00000164011.16 | ZNF691 | 0.588455 | 2.696035 | 10.21253 | 1.37E-22 | 3.08E-21 | 39.99013 |
| ENSG00000137210.12 | TMEM14B | 0.588383 | 3.764569 | 7.504215 | 2.41E-13 | 2.67E-12 | 18.96371 |
| ENSG00000101391.19 | CDK5RAP1 | 0.588151 | 2.899925 | 10.43422 | 1.96E-23 | 4.65E-22 | 41.91663 |
| ENSG00000169223.13 | LMAN2 | 0.588143 | 6.400017 | 8.340351 | 5.65E-16 | 7.77E-15 | 24.92712 |
| ENSG00000106049.7 | HIBADH | 0.588109 | 4.505968 | 7.69791 | 6.19E-14 | 7.17E-13 | 20.30006 |
| ENSG00000136193.15 | SCRN1 | 0.588011 | 4.03868 | 4.96184 | 9.25E-07 | 5.13E-06 | 4.165843 |
| ENSG00000223572.8 | CKMT1A | 0.587978 | 0.676858 | 6.843054 | 2.02E-11 | 1.86E-10 | 14.61528 |
| ENSG00000167863.10 | ATP5H | 0.587931 | 5.132287 | 9.245779 | 4.70E-19 | 8.21E-18 | 31.93205 |
| ENSG00000130508.9 | PXDN | 0.587922 | 3.313235 | 3.90466 | 0.000105726 | 0.000438561 | -0.36322 |
| ENSG00000170385.9 | SLC30A1 | 0.587762 | 3.416422 | 6.86957 | 1.70E-11 | 1.58E-10 | 14.7832 |
| ENSG00000132603.12 | NIP7 | 0.587542 | 3.396602 | 9.231727 | 5.27E-19 | 9.17E-18 | 31.81921 |
| ENSG00000118939.16 | UCHL3 | 0.587462 | 2.252747 | 8.838137 | 1.22E-17 | 1.92E-16 | 28.71057 |
| ENSG00000167815.10 | PRDX2 | 0.587416 | 5.916411 | 6.16189 | 1.36E-09 | 1.05E-08 | 10.49319 |
| ENSG00000143155.11 | TIPRL | 0.587265 | 4.177683 | 9.916581 | 1.76E-21 | 3.65E-20 | 37.46306 |
| ENSG00000166016.5 | ABTB2 | 0.587263 | 1.637432 | 6.8668 | 1.73E-11 | 1.61E-10 | 14.76563 |
| ENSG00000133805.14 | AMPD3 | 0.587238 | 2.060077 | 8.031515 | 5.60E-15 | 7.09E-14 | 22.66597 |
| ENSG00000157020.16 | SEC13 | 0.587112 | 4.43298 | 10.88026 | 3.62E-25 | 9.70E-24 | 45.87656 |
| ENSG00000173653.6 | RCE1 | 0.586977 | 3.053464 | 10.19743 | 1.56E-22 | 3.50E-21 | 39.85994 |
| ENSG00000119912.14 | IDE | 0.586892 | 2.929038 | 9.418587 | 1.14E-19 | 2.08E-18 | 33.33009 |
| ENSG00000102007.9 | PLP2 | 0.586773 | 6.59175 | 5.140463 | 3.78E-07 | 2.21E-06 | 5.029484 |
| ENSG00000100902.9 | PSMA6 | 0.58655 | 2.812125 | 6.306297 | 5.76E-10 | 4.59E-09 | 11.33588 |
| ENSG00000214530.6 | STARD10 | 0.586544 | 4.67251 | 5.02068 | 6.91E-07 | 3.90E-06 | 4.447251 |
| ENSG00000134058.9 | CDK7 | 0.586269 | 3.403136 | 9.369825 | 1.71E-19 | 3.07E-18 | 32.93368 |
| ENSG00000106290.13 | TAF6 | 0.586214 | 3.432687 | 8.617155 | 6.85E-17 | 1.01E-15 | 27.01018 |
| ENSG00000134697.11 | GNL2 | 0.586166 | 3.621826 | 10.31421 | 5.64E-23 | 1.29E-21 | 40.8702 |
| ENSG00000148824.17 | MTG1 | 0.586164 | 1.383054 | 8.734421 | 2.76E-17 | 4.20E-16 | 27.90842 |
| ENSG00000169297.7 | NR0B1 | 0.586137 | 0.568031 | 3.221 | 0.00135069 | 0.004572329 | -2.74598 |
| ENSG00000101193.7 | GID8 | 0.586068 | 4.23603 | 8.980819 | 3.96E-18 | 6.46E-17 | 29.82578 |
| ENSG00000125630.14 | POLR1B | 0.585881 | 2.485324 | 10.3448 | 4.32E-23 | 9.96E-22 | 41.13617 |
| ENSG00000198203.8 | SULT1C2 | 0.58588 | 1.308539 | 4.249636 | 2.50E-05 | 0.000114225 | 1.003606 |
| ENSG00000131779.9 | PEX11B | 0.585691 | 4.656096 | 8.94125 | 5.42E-18 | 8.78E-17 | 29.51515 |
| ENSG00000186684.11 | CYP27C1 | 0.585622 | 0.658924 | 6.351537 | 4.38E-10 | 3.54E-09 | 11.60337 |
| ENSG00000175711.7 | B3GNTL1 | 0.585518 | 1.169532 | 11.10709 | 4.56E-26 | 1.28E-24 | 47.93198 |
| ENSG00000105193.7 | RPS16 | 0.585284 | 8.083758 | 5.806955 | 1.06E-08 | 7.41E-08 | 8.494897 |
| ENSG00000122687.16 | FTSJ2 | 0.585165 | 3.710514 | 9.599404 | 2.56E-20 | 4.85E-19 | 34.81306 |
| ENSG00000188290.9 | HES4 | 0.58512 | 2.554641 | 4.807023 | 1.97E-06 | 1.05E-05 | 3.439978 |
| ENSG00000151465.12 | CDC123 | 0.585113 | 4.625859 | 9.330654 | 2.36E-19 | 4.19E-18 | 32.61632 |
| ENSG00000040341.16 | STAU2 | 0.585073 | 2.777868 | 7.852577 | 2.05E-14 | 2.48E-13 | 21.38693 |
| ENSG00000100994.10 | PYGB | 0.584905 | 5.690711 | 5.488552 | 6.12E-08 | 3.93E-07 | 6.792033 |
| ENSG00000167797.6 | CDK2AP2 | 0.584894 | 5.565469 | 5.355175 | 1.24E-07 | 7.70E-07 | 6.104342 |
| ENSG00000064313.10 | TAF2 | 0.584597 | 2.658123 | 8.732108 | 2.81E-17 | 4.27E-16 | 27.89061 |
| ENSG00000278888.1 | AC090154.1 | 0.584458 | 1.379574 | 6.098388 | 1.98E-09 | 1.50E-08 | 10.12802 |
| ENSG00000167930.14 | ITFG3 | 0.584369 | 3.839236 | 7.362523 | 6.39E-13 | 6.82E-12 | 18.00382 |
| ENSG00000057935.12 | MTA3 | 0.584343 | 2.331221 | 10.25962 | 9.10E-23 | 2.07E-21 | 40.39696 |
| ENSG00000091513.13 | TF | 0.583933 | 0.773948 | 3.126065 | 0.001862415 | 0.006115335 | -3.0421 |
| ENSG00000065057.6 | NTHL1 | 0.583915 | 3.278364 | 6.210943 | 1.02E-09 | 7.96E-09 | 10.77753 |
| ENSG00000117407.15 | ARTN | 0.583669 | 0.717872 | 6.160859 | 1.37E-09 | 1.06E-08 | 10.48724 |
| ENSG00000125520.12 | SLC2A4RG | 0.583663 | 4.850739 | 6.261268 | 7.55E-10 | 5.95E-09 | 11.07129 |
| ENSG00000163794.6 | UCN | 0.583537 | 1.119827 | 6.501858 | 1.74E-10 | 1.47E-09 | 12.50404 |
| ENSG00000135083.13 | CCNJL | 0.583433 | 3.010654 | 4.449971 | 1.03E-05 | 4.99E-05 | 1.847135 |
| ENSG00000156711.15 | MAPK13 | 0.583414 | 4.349974 | 7.415701 | 4.44E-13 | 4.80E-12 | 18.36231 |
| ENSG00000182362.12 | YBEY | 0.583257 | 2.568343 | 7.093177 | 3.93E-12 | 3.87E-11 | 16.22098 |
| ENSG00000196503.2 | ARL9 | 0.583182 | 0.736184 | 6.597921 | 9.59E-11 | 8.27E-10 | 13.08911 |
| ENSG00000105929.14 | ATP6V0A4 | 0.583112 | 1.090117 | 3.777761 | 0.000174984 | 0.000698954 | -0.83846 |
| ENSG00000132185.15 | FCRLA | 0.582878 | 0.966485 | 5.122119 | 4.15E-07 | 2.41E-06 | 4.939507 |
| ENSG00000169230.8 | PRELID1 | 0.582522 | 4.540443 | 8.784821 | 1.86E-17 | 2.88E-16 | 28.29732 |
| ENSG00000180329.12 | CCDC43 | 0.582507 | 3.454263 | 10.34937 | 4.15E-23 | 9.59E-22 | 41.17599 |
| ENSG00000116750.12 | UCHL5 | 0.582308 | 2.890247 | 10.52756 | 8.59E-24 | 2.09E-22 | 42.73609 |
| ENSG00000198169.7 | ZNF251 | 0.582223 | 2.138189 | 7.626078 | 1.03E-13 | 1.17E-12 | 19.80123 |
| ENSG00000163655.14 | GMPS | 0.581918 | 3.05277 | 7.883779 | 1.64E-14 | 2.00E-13 | 21.60829 |
| ENSG00000198276.12 | UCKL1 | 0.581725 | 3.583435 | 7.437615 | 3.82E-13 | 4.15E-12 | 18.51066 |
| ENSG00000152332.14 | UHMK1 | 0.581281 | 4.250187 | 8.35248 | 5.16E-16 | 7.11E-15 | 25.01728 |
| ENSG00000182195.7 | LDOC1 | 0.581152 | 4.347118 | 3.306449 | 0.001004679 | 0.003491295 | -2.47215 |
| ENSG00000179083.6 | FAM133A | 0.581058 | 0.564804 | 3.770488 | 0.000180034 | 0.00071794 | -0.86524 |
| ENSG00000010030.12 | ETV7 | 0.580984 | 2.403779 | 5.126278 | 4.06E-07 | 2.36E-06 | 4.95988 |
| ENSG00000099814.14 | CEP170B | 0.580652 | 3.566966 | 6.106618 | 1.89E-09 | 1.43E-08 | 10.17516 |
| ENSG00000127554.12 | GFER | 0.580126 | 2.966309 | 10.24297 | 1.05E-22 | 2.38E-21 | 40.25297 |
| ENSG00000171729.12 | TMEM51 | 0.579937 | 3.625457 | 6.258594 | 7.67E-10 | 6.05E-09 | 11.05563 |
| ENSG00000072506.11 | HSD17B10 | 0.579765 | 5.705333 | 8.473262 | 2.07E-16 | 2.94E-15 | 25.92075 |
| ENSG00000156521.12 | TYSND1 | 0.579462 | 2.695973 | 9.054118 | 2.21E-18 | 3.67E-17 | 30.40389 |
| ENSG00000113615.11 | SEC24A | 0.579415 | 3.021853 | 8.86991 | 9.53E-18 | 1.51E-16 | 28.95774 |
| ENSG00000239264.7 | TXNDC5 | 0.579238 | 1.87862 | 7.006007 | 6.98E-12 | 6.73E-11 | 15.65588 |
| ENSG00000150556.15 | LYPD6B | 0.579042 | 1.023273 | 4.280292 | 2.19E-05 | 0.000100866 | 1.130328 |
| ENSG00000116285.11 | ERRFI1 | 0.579009 | 5.864441 | 3.621372 | 0.000319335 | 0.001216114 | -1.40357 |
| ENSG00000065548.16 | ZC3H15 | 0.578534 | 4.466949 | 10.23625 | 1.12E-22 | 2.52E-21 | 40.1949 |
| ENSG00000074695.5 | LMAN1 | 0.578533 | 4.834818 | 7.327758 | 8.10E-13 | 8.57E-12 | 17.77061 |
| ENSG00000058600.14 | POLR3E | 0.578188 | 2.405626 | 10.84462 | 5.00E-25 | 1.33E-23 | 45.55608 |
| ENSG00000132382.13 | MYBBP1A | 0.57809 | 3.383161 | 7.599374 | 1.24E-13 | 1.40E-12 | 19.61677 |
| ENSG00000170581.12 | STAT2 | 0.577862 | 4.527363 | 7.529453 | 2.02E-13 | 2.25E-12 | 19.13626 |
| ENSG00000128534.6 | LSM8 | 0.57784 | 2.414043 | 10.25349 | 9.60E-23 | 2.18E-21 | 40.34397 |
| ENSG00000104442.8 | ARMC1 | 0.577774 | 3.80024 | 7.730548 | 4.91E-14 | 5.73E-13 | 20.52795 |
| ENSG00000152147.9 | GEMIN6 | 0.577643 | 2.08307 | 10.16711 | 2.04E-22 | 4.51E-21 | 39.59895 |
| ENSG00000135940.5 | COX5B | 0.577521 | 5.847249 | 6.692544 | 5.28E-11 | 4.69E-10 | 13.6726 |
| ENSG00000214336.4 | FOXI3 | 0.577472 | 0.532058 | 4.488265 | 8.70E-06 | 4.24E-05 | 2.012499 |
| ENSG00000168883.18 | USP39 | 0.577086 | 4.079768 | 11.00472 | 1.17E-25 | 3.21E-24 | 47.00095 |
| ENSG00000136122.14 | BORA | 0.577066 | 1.344118 | 8.548684 | 1.16E-16 | 1.68E-15 | 26.49002 |
| ENSG00000149256.13 | TENM4 | 0.576755 | 0.880595 | 7.582109 | 1.40E-13 | 1.58E-12 | 19.49778 |
| ENSG00000176102.10 | CSTF3 | 0.576117 | 2.880834 | 11.37548 | 3.79E-27 | 1.15E-25 | 50.39889 |
| ENSG00000065665.19 | SEC61A2 | 0.575977 | 1.4539 | 8.789659 | 1.79E-17 | 2.77E-16 | 28.33474 |
| ENSG00000142484.6 | TM4SF5 | 0.57562 | 0.531331 | 3.210327 | 0.001400922 | 0.004726499 | -2.7797 |
| ENSG00000155561.13 | NUP205 | 0.575518 | 3.229116 | 7.411115 | 4.58E-13 | 4.95E-12 | 18.33131 |
| ENSG00000177830.16 | CHID1 | 0.575455 | 3.931028 | 9.053224 | 2.22E-18 | 3.70E-17 | 30.39682 |
| ENSG00000197157.9 | SND1 | 0.575158 | 5.300746 | 10.17625 | 1.88E-22 | 4.18E-21 | 39.67759 |
| ENSG00000185043.9 | CIB1 | 0.575156 | 6.738366 | 6.434184 | 2.64E-10 | 2.19E-09 | 12.09632 |
| ENSG00000221944.4 | TIGD1 | 0.57513 | 1.555802 | 8.04633 | 5.03E-15 | 6.40E-14 | 22.7729 |
| ENSG00000134716.8 | CYP2J2 | 0.575067 | 1.744758 | 6.052551 | 2.59E-09 | 1.94E-08 | 9.866497 |
| ENSG00000172809.11 | RPL38 | 0.574846 | 6.338509 | 6.957273 | 9.61E-12 | 9.14E-11 | 15.3425 |
| ENSG00000130726.10 | TRIM28 | 0.574804 | 6.014775 | 8.064302 | 4.41E-15 | 5.63E-14 | 22.90282 |
| ENSG00000119705.8 | SLIRP | 0.574446 | 3.347398 | 8.260982 | 1.03E-15 | 1.38E-14 | 24.33961 |
| ENSG00000101441.4 | CST4 | 0.574393 | 0.528391 | 4.481136 | 8.99E-06 | 4.38E-05 | 1.981617 |
| ENSG00000142920.15 | AZIN2 | 0.574271 | 1.128252 | 8.324278 | 6.38E-16 | 8.73E-15 | 24.80778 |
| ENSG00000135763.8 | URB2 | 0.57421 | 1.960179 | 9.01917 | 2.92E-18 | 4.80E-17 | 30.12781 |
| ENSG00000137411.15 | VARS2 | 0.574073 | 2.539896 | 8.519751 | 1.45E-16 | 2.08E-15 | 26.27118 |
| ENSG00000160051.10 | IQCC | 0.573969 | 1.595165 | 8.896247 | 7.74E-18 | 1.24E-16 | 29.16314 |
| ENSG00000113013.11 | HSPA9 | 0.573928 | 5.626399 | 8.501125 | 1.67E-16 | 2.39E-15 | 26.1306 |
| ENSG00000136631.11 | VPS45 | 0.573894 | 3.031668 | 10.03412 | 6.42E-22 | 1.37E-20 | 38.46055 |
| ENSG00000274523.3 | WBSCR16 | 0.573844 | 3.893253 | 10.45165 | 1.68E-23 | 4.02E-22 | 42.06925 |
| ENSG00000196367.11 | TRRAP | 0.57379 | 2.997144 | 6.722118 | 4.38E-11 | 3.92E-10 | 13.85642 |
| ENSG00000235961.5 | PNMA6A | 0.573668 | 0.840862 | 5.26163 | 2.03E-07 | 1.23E-06 | 5.631148 |
| ENSG00000242802.5 | AP5Z1 | 0.573116 | 2.855581 | 7.471541 | 3.02E-13 | 3.32E-12 | 18.74103 |
| ENSG00000116337.14 | AMPD2 | 0.573099 | 2.837646 | 9.174153 | 8.40E-19 | 1.44E-17 | 31.35819 |
| ENSG00000244242.1 | IFITM10 | 0.573091 | 1.995832 | 4.698096 | 3.30E-06 | 1.70E-05 | 2.941982 |
| ENSG00000184640.16 | 9-Sep | 0.57297 | 4.798951 | 7.826065 | 2.48E-14 | 2.98E-13 | 21.19939 |
| ENSG00000105854.11 | PON2 | 0.572917 | 5.549948 | 4.840481 | 1.67E-06 | 8.97E-06 | 3.595058 |
| ENSG00000005156.10 | LIG3 | 0.572488 | 1.824571 | 10.13247 | 2.75E-22 | 6.04E-21 | 39.3014 |
| ENSG00000154174.7 | TOMM70A | 0.572417 | 3.952783 | 9.775433 | 5.83E-21 | 1.16E-19 | 36.2762 |
| ENSG00000185761.9 | ADAMTSL5 | 0.57222 | 0.99337 | 6.734129 | 4.06E-11 | 3.64E-10 | 13.93127 |
| ENSG00000136695.13 | IL36RN | 0.572172 | 0.531291 | 4.875454 | 1.41E-06 | 7.64E-06 | 3.758215 |
| ENSG00000127334.10 | DYRK2 | 0.572099 | 2.283329 | 7.278489 | 1.13E-12 | 1.18E-11 | 17.44167 |
| ENSG00000108883.11 | EFTUD2 | 0.571963 | 3.596024 | 10.46476 | 1.50E-23 | 3.59E-22 | 42.18418 |
| ENSG00000167165.17 | UGT1A6 | 0.571696 | 0.613621 | 3.448728 | 0.000605095 | 0.002190229 | -2.00091 |
| ENSG00000127884.4 | ECHS1 | 0.571471 | 6.147949 | 8.881762 | 8.68E-18 | 1.38E-16 | 29.05011 |
| ENSG00000065518.7 | NDUFB4 | 0.571363 | 5.348156 | 7.887929 | 1.59E-14 | 1.94E-13 | 21.6378 |
| ENSG00000170522.8 | ELOVL6 | 0.571105 | 1.533678 | 4.695203 | 3.34E-06 | 1.73E-05 | 2.928902 |
| ENSG00000115289.11 | PCGF1 | 0.571067 | 2.921837 | 10.86453 | 4.17E-25 | 1.12E-23 | 45.73507 |
| ENSG00000114315.3 | HES1 | 0.570956 | 4.470899 | 4.675032 | 3.68E-06 | 1.89E-05 | 2.837893 |
| ENSG00000197635.8 | DPP4 | 0.570726 | 4.000559 | 2.516074 | 0.012142296 | 0.032955015 | -4.73944 |
| ENSG00000185122.9 | HSF1 | 0.570311 | 4.263475 | 7.905779 | 1.40E-14 | 1.72E-13 | 21.7648 |
| ENSG00000135823.12 | STX6 | 0.570191 | 3.505189 | 10.49322 | 1.17E-23 | 2.80E-22 | 42.43409 |
| ENSG00000114023.14 | FAM162A | 0.570143 | 3.529741 | 6.817592 | 2.38E-11 | 2.18E-10 | 14.45455 |
| ENSG00000037241.6 | RPL26L1 | 0.570085 | 3.772591 | 7.448153 | 3.55E-13 | 3.87E-12 | 18.58212 |
| ENSG00000146070.15 | PLA2G7 | 0.570031 | 2.85804 | 4.0458 | 5.94E-05 | 0.000256629 | 0.182803 |
| ENSG00000196335.11 | STK31 | 0.569968 | 0.804349 | 7.476779 | 2.91E-13 | 3.20E-12 | 18.77667 |
| ENSG00000114405.9 | C3orf14 | 0.569806 | 1.808414 | 6.494784 | 1.82E-10 | 1.53E-09 | 12.46125 |
| ENSG00000112812.14 | PRSS16 | 0.569758 | 2.341583 | 6.883536 | 1.55E-11 | 1.45E-10 | 14.87186 |
| ENSG00000163599.13 | CTLA4 | 0.5695 | 1.389077 | 5.640107 | 2.69E-08 | 1.80E-07 | 7.591876 |
| ENSG00000100632.9 | ERH | 0.569494 | 6.001231 | 7.854614 | 2.02E-14 | 2.45E-13 | 21.40136 |
| ENSG00000170633.15 | RNF34 | 0.569377 | 2.883094 | 10.73652 | 1.33E-24 | 3.41E-23 | 44.5884 |
| ENSG00000065183.14 | WDR3 | 0.569035 | 2.272072 | 8.675307 | 4.37E-17 | 6.54E-16 | 27.45445 |
| ENSG00000198056.12 | PRIM1 | 0.568972 | 2.078015 | 6.145808 | 1.50E-09 | 1.15E-08 | 10.4004 |
| ENSG00000165898.12 | ISCA2 | 0.568477 | 2.975598 | 8.387636 | 3.96E-16 | 5.50E-15 | 25.27922 |
| ENSG00000124608.4 | AARS2 | 0.568409 | 2.540664 | 8.713053 | 3.26E-17 | 4.93E-16 | 27.74405 |
| ENSG00000124783.11 | SSR1 | 0.568406 | 4.321079 | 9.509296 | 5.41E-20 | 1.01E-18 | 34.07148 |
| ENSG00000106603.16 | COA1 | 0.568305 | 2.302702 | 9.952091 | 1.30E-21 | 2.71E-20 | 37.76354 |
| ENSG00000104884.13 | ERCC2 | 0.568264 | 2.375325 | 9.348053 | 2.04E-19 | 3.65E-18 | 32.75717 |
| ENSG00000197774.11 | EME2 | 0.568211 | 1.438596 | 6.337584 | 4.77E-10 | 3.84E-09 | 11.5207 |
| ENSG00000198758.9 | EPS8L3 | 0.568073 | 0.529465 | 3.469373 | 0.000561345 | 0.002045046 | -1.93095 |
| ENSG00000096080.10 | MRPS18A | 0.567911 | 4.052251 | 8.334311 | 5.92E-16 | 8.11E-15 | 24.88225 |
| ENSG00000113810.14 | SMC4 | 0.567875 | 2.712323 | 5.664857 | 2.34E-08 | 1.58E-07 | 7.724343 |
| ENSG00000122512.13 | PMS2 | 0.567722 | 1.959516 | 9.323012 | 2.51E-19 | 4.45E-18 | 32.55453 |
| ENSG00000101421.3 | CHMP4B | 0.56758 | 6.931544 | 9.553417 | 3.75E-20 | 7.03E-19 | 34.43396 |
| ENSG00000136522.12 | MRPL47 | 0.567347 | 4.456933 | 7.428397 | 4.07E-13 | 4.42E-12 | 18.44821 |
| ENSG00000174013.7 | FBXO45 | 0.566967 | 2.229938 | 7.610957 | 1.14E-13 | 1.30E-12 | 19.69671 |
| ENSG00000120211.4 | INSL4 | 0.566775 | 0.52088 | 3.153011 | 0.001701475 | 0.005630591 | -2.95892 |
| ENSG00000176125.4 | UFSP1 | 0.566655 | 1.40632 | 7.407749 | 4.69E-13 | 5.06E-12 | 18.30857 |
| ENSG00000197568.12 | HHLA3 | 0.566436 | 2.555936 | 5.789764 | 1.17E-08 | 8.12E-08 | 8.400776 |
| ENSG00000175166.15 | PSMD2 | 0.566301 | 5.361211 | 7.644102 | 9.05E-14 | 1.04E-12 | 19.92604 |
| ENSG00000104892.15 | KLC3 | 0.565823 | 1.019924 | 5.579297 | 3.75E-08 | 2.46E-07 | 7.2686 |
| ENSG00000025772.7 | TOMM34 | 0.565687 | 4.295749 | 7.862865 | 1.91E-14 | 2.31E-13 | 21.45984 |
| ENSG00000135249.6 | RINT1 | 0.565643 | 2.783571 | 9.296821 | 3.10E-19 | 5.49E-18 | 32.34301 |
| ENSG00000075399.11 | VPS9D1 | 0.565636 | 2.865607 | 6.874713 | 1.64E-11 | 1.53E-10 | 14.81583 |
| ENSG00000115539.12 | PDCL3 | 0.565534 | 3.707986 | 9.321621 | 2.54E-19 | 4.50E-18 | 32.54328 |
| ENSG00000156531.15 | PHF6 | 0.56549 | 2.255927 | 8.780798 | 1.92E-17 | 2.97E-16 | 28.26621 |
| ENSG00000100519.10 | PSMC6 | 0.56539 | 3.398972 | 8.704054 | 3.49E-17 | 5.27E-16 | 27.67492 |
| ENSG00000156482.9 | RPL30 | 0.565253 | 7.216647 | 6.938124 | 1.09E-11 | 1.03E-10 | 15.21987 |
| ENSG00000119878.5 | CRIPT | 0.565204 | 3.155921 | 8.427945 | 2.92E-16 | 4.10E-15 | 25.58059 |
| ENSG00000087302.7 | C14orf166 | 0.565178 | 3.731122 | 10.07723 | 4.43E-22 | 9.58E-21 | 38.82843 |
| ENSG00000278845.3 | MRPL45 | 0.56516 | 4.498135 | 8.741272 | 2.61E-17 | 3.99E-16 | 27.96118 |
| ENSG00000120093.10 | HOXB3 | 0.565034 | 1.476476 | 4.317837 | 1.86E-05 | 8.65E-05 | 1.28669 |
| ENSG00000149948.12 | HMGA2 | 0.564579 | 0.537474 | 4.251641 | 2.48E-05 | 0.000113307 | 1.011869 |
| ENSG00000028310.16 | BRD9 | 0.564162 | 2.604341 | 7.758642 | 4.02E-14 | 4.73E-13 | 20.72475 |
| ENSG00000164163.9 | ABCE1 | 0.564157 | 3.585122 | 8.933431 | 5.77E-18 | 9.32E-17 | 29.4539 |
| ENSG00000164920.8 | OSR2 | 0.564118 | 1.242949 | 5.189757 | 2.94E-07 | 1.74E-06 | 5.272716 |
| ENSG00000101400.5 | SNTA1 | 0.564005 | 3.396173 | 5.055912 | 5.79E-07 | 3.30E-06 | 4.6172 |
| ENSG00000125285.5 | SOX21 | 0.56382 | 0.800828 | 3.959516 | 8.47E-05 | 0.000357124 | -0.15318 |
| ENSG00000123080.9 | CDKN2C | 0.563771 | 2.254473 | 6.013807 | 3.25E-09 | 2.40E-08 | 9.646792 |
| ENSG00000163354.13 | DCST2 | 0.563639 | 0.706739 | 7.661271 | 8.02E-14 | 9.21E-13 | 20.04515 |
| ENSG00000171320.13 | ESCO2 | 0.563492 | 0.647684 | 9.712602 | 9.91E-21 | 1.93E-19 | 35.75177 |
| ENSG00000122711.7 | SPINK4 | 0.563478 | 0.570132 | 3.302736 | 0.001017819 | 0.003534115 | -2.48419 |
| ENSG00000170027.6 | YWHAG | 0.562914 | 5.956928 | 7.1828 | 2.16E-12 | 2.18E-11 | 16.80807 |
| ENSG00000141568.18 | FOXK2 | 0.562841 | 3.056227 | 9.334809 | 2.28E-19 | 4.06E-18 | 32.64994 |
| ENSG00000129455.14 | KLK8 | 0.562682 | 0.569511 | 3.625436 | 0.000314466 | 0.001199877 | -1.38917 |
| ENSG00000075188.7 | NUP37 | 0.562616 | 2.659137 | 8.1597 | 2.18E-15 | 2.86E-14 | 23.59631 |
| ENSG00000113649.10 | TCERG1 | 0.562572 | 2.672387 | 9.256592 | 4.31E-19 | 7.55E-18 | 32.01897 |
| ENSG00000149476.13 | DAK | 0.562298 | 2.644158 | 8.711335 | 3.30E-17 | 5.00E-16 | 27.73085 |
| ENSG00000099998.16 | GGT5 | 0.562264 | 2.803551 | 4.751852 | 2.56E-06 | 1.34E-05 | 3.18643 |
| ENSG00000130741.9 | EIF2S3 | 0.562214 | 5.790315 | 8.036404 | 5.41E-15 | 6.86E-14 | 22.70124 |
| ENSG00000107130.8 | NCS1 | 0.562172 | 2.217476 | 5.548485 | 4.43E-08 | 2.89E-07 | 7.105999 |
| ENSG00000171155.7 | C1GALT1C1 | 0.562122 | 4.274042 | 8.798174 | 1.68E-17 | 2.61E-16 | 28.40064 |
| ENSG00000151790.7 | TDO2 | 0.562102 | 0.898792 | 6.631718 | 7.75E-11 | 6.76E-10 | 13.29671 |
| ENSG00000130475.13 | FCHO1 | 0.562039 | 1.620928 | 6.157996 | 1.40E-09 | 1.07E-08 | 10.4707 |
| ENSG00000169562.9 | GJB1 | 0.561855 | 2.325244 | 2.364487 | 0.018391269 | 0.047585521 | -5.10564 |
| ENSG00000189280.3 | GJB5 | 0.561108 | 0.915743 | 3.628094 | 0.000311321 | 0.00118904 | -1.37975 |
| ENSG00000099956.16 | SMARCB1 | 0.560833 | 4.859848 | 9.730176 | 8.54E-21 | 1.68E-19 | 35.89822 |
| ENSG00000275700.3 | AATF | 0.560722 | 3.857728 | 8.933784 | 5.75E-18 | 9.30E-17 | 29.45666 |
| ENSG00000104689.8 | TNFRSF10A | 0.560657 | 2.800393 | 6.276982 | 6.87E-10 | 5.44E-09 | 11.16344 |
| ENSG00000127399.13 | LRRC61 | 0.560378 | 2.914624 | 6.623877 | 8.15E-11 | 7.09E-10 | 13.24846 |
| ENSG00000169733.10 | RFNG | 0.560074 | 3.554755 | 8.020555 | 6.07E-15 | 7.67E-14 | 22.58697 |
| ENSG00000004779.8 | NDUFAB1 | 0.559811 | 4.886307 | 8.48184 | 1.93E-16 | 2.76E-15 | 25.9853 |
| ENSG00000167543.14 | TP53I13 | 0.559388 | 3.072664 | 7.45252 | 3.44E-13 | 3.77E-12 | 18.61176 |
| ENSG00000095906.15 | NUBP2 | 0.55937 | 3.494321 | 7.785273 | 3.33E-14 | 3.95E-13 | 20.91183 |
| ENSG00000070814.16 | TCOF1 | 0.559194 | 2.628737 | 7.907959 | 1.38E-14 | 1.69E-13 | 21.78034 |
| ENSG00000139083.9 | ETV6 | 0.559166 | 3.302395 | 7.99171 | 7.49E-15 | 9.39E-14 | 22.37946 |
| ENSG00000166562.7 | SEC11C | 0.558862 | 4.070301 | 3.718316 | 0.000220495 | 0.000864918 | -1.05594 |
| ENSG00000007384.14 | RHBDF1 | 0.558618 | 3.225223 | 6.292895 | 6.24E-10 | 4.96E-09 | 11.25696 |
| ENSG00000103035.9 | PSMD7 | 0.55845 | 4.629594 | 9.11842 | 1.32E-18 | 2.22E-17 | 30.91394 |
| ENSG00000142700.10 | DMRTA2 | 0.558434 | 0.529435 | 5.315164 | 1.53E-07 | 9.42E-07 | 5.901025 |
| ENSG00000064932.14 | SBNO2 | 0.558393 | 4.123147 | 5.530082 | 4.89E-08 | 3.18E-07 | 7.009266 |
| ENSG00000143363.14 | PRUNE | 0.5583 | 3.826643 | 7.951963 | 1.00E-14 | 1.24E-13 | 22.09449 |
| ENSG00000141076.16 | CIRH1A | 0.558148 | 3.019746 | 10.14341 | 2.50E-22 | 5.51E-21 | 39.39533 |
| ENSG00000111786.7 | SRSF9 | 0.558082 | 4.457766 | 9.396632 | 1.37E-19 | 2.48E-18 | 33.15142 |
| ENSG00000180182.9 | MED14 | 0.557963 | 2.934783 | 8.872611 | 9.33E-18 | 1.48E-16 | 28.97878 |
| ENSG00000138399.16 | FASTKD1 | 0.557813 | 2.317257 | 9.482661 | 6.74E-20 | 1.25E-18 | 33.85325 |
| ENSG00000155506.15 | LARP1 | 0.557754 | 4.662433 | 7.006981 | 6.94E-12 | 6.69E-11 | 15.66215 |
| ENSG00000116830.10 | TTF2 | 0.557623 | 1.544158 | 10.28814 | 7.09E-23 | 1.62E-21 | 40.64401 |
| ENSG00000056558.9 | TRAF1 | 0.557585 | 2.053448 | 5.973077 | 4.11E-09 | 3.01E-08 | 9.417168 |
| ENSG00000187954.11 | CYHR1 | 0.557431 | 2.780067 | 7.817968 | 2.63E-14 | 3.15E-13 | 21.14221 |
| ENSG00000088386.14 | SLC15A1 | 0.557393 | 0.583185 | 4.950605 | 9.77E-07 | 5.41E-06 | 4.11246 |
| ENSG00000101150.16 | TPD52L2 | 0.557145 | 5.070813 | 8.909906 | 6.95E-18 | 1.11E-16 | 29.26983 |
| ENSG00000169188.4 | APEX2 | 0.55714 | 3.521349 | 9.654535 | 1.61E-20 | 3.09E-19 | 35.26926 |
| ENSG00000183340.6 | JRKL | 0.556993 | 2.575159 | 8.265907 | 9.89E-16 | 1.33E-14 | 24.37594 |
| ENSG00000116580.17 | GON4L | 0.556793 | 2.436414 | 8.074151 | 4.10E-15 | 5.25E-14 | 22.97412 |
| ENSG00000110917.6 | MLEC | 0.556653 | 5.226847 | 6.258034 | 7.70E-10 | 6.07E-09 | 11.05235 |
| ENSG00000174744.12 | BRMS1 | 0.556647 | 4.331887 | 9.255764 | 4.34E-19 | 7.60E-18 | 32.01232 |
| ENSG00000178445.8 | GLDC | 0.556082 | 0.731514 | 4.474893 | 9.24E-06 | 4.49E-05 | 1.954607 |
| ENSG00000129084.16 | PSMA1 | 0.556024 | 4.416476 | 10.06757 | 4.82E-22 | 1.04E-20 | 38.74588 |
| ENSG00000145979.16 | TBC1D7 | 0.55549 | 1.538182 | 9.071204 | 1.92E-18 | 3.21E-17 | 30.53915 |
| ENSG00000187566.4 | NHLRC1 | 0.555295 | 1.646648 | 6.957822 | 9.58E-12 | 9.11E-11 | 15.34602 |
| ENSG00000160714.8 | UBE2Q1 | 0.555131 | 4.799451 | 9.782544 | 5.49E-21 | 1.10E-19 | 36.33571 |
| ENSG00000103037.10 | SETD6 | 0.55484 | 1.915235 | 9.402075 | 1.31E-19 | 2.37E-18 | 33.19569 |
| ENSG00000111490.11 | TBC1D30 | 0.554783 | 1.697505 | 6.774302 | 3.14E-11 | 2.84E-10 | 14.18246 |
| ENSG00000197122.10 | SRC | 0.554581 | 3.843165 | 6.943454 | 1.05E-11 | 9.96E-11 | 15.25398 |
| ENSG00000167701.12 | GPT | 0.554401 | 0.864272 | 5.551067 | 4.37E-08 | 2.85E-07 | 7.119591 |
| ENSG00000168887.9 | C2orf68 | 0.554341 | 3.448569 | 9.014655 | 3.02E-18 | 4.98E-17 | 30.09221 |
| ENSG00000143498.16 | TAF1A | 0.554254 | 1.620241 | 9.794099 | 4.98E-21 | 9.99E-20 | 36.43247 |
| ENSG00000172725.12 | CORO1B | 0.554231 | 4.637542 | 8.648909 | 5.36E-17 | 7.96E-16 | 27.2525 |
| ENSG00000159111.11 | MRPL10 | 0.554169 | 4.168467 | 10.69623 | 1.91E-24 | 4.83E-23 | 44.22943 |
| ENSG00000143811.15 | PYCR2 | 0.554127 | 4.397424 | 8.801923 | 1.63E-17 | 2.53E-16 | 28.42967 |
| ENSG00000188095.4 | MESP2 | 0.553946 | 0.612387 | 6.313291 | 5.52E-10 | 4.41E-09 | 11.37713 |
| ENSG00000062822.11 | POLD1 | 0.553884 | 2.846818 | 7.160682 | 2.50E-12 | 2.51E-11 | 16.66261 |
| ENSG00000204348.8 | DXO | 0.553705 | 2.77977 | 7.53659 | 1.92E-13 | 2.14E-12 | 19.18513 |
| ENSG00000120756.11 | PLS1 | 0.553597 | 3.552258 | 5.587612 | 3.58E-08 | 2.36E-07 | 7.312617 |
| ENSG00000020256.18 | ZFP64 | 0.553583 | 1.887043 | 11.44781 | 1.93E-27 | 5.95E-26 | 51.07011 |
| ENSG00000166415.13 | WDR72 | 0.553517 | 0.837917 | 3.819882 | 0.000148268 | 0.000599845 | -0.68237 |
| ENSG00000124120.9 | TTPAL | 0.55344 | 2.166953 | 8.949632 | 5.07E-18 | 8.23E-17 | 29.58087 |
| ENSG00000141858.10 | SAMD1 | 0.552827 | 4.065919 | 7.648766 | 8.76E-14 | 1.00E-12 | 19.95838 |
| ENSG00000129910.6 | CDH15 | 0.552814 | 0.785677 | 5.702298 | 1.90E-08 | 1.30E-07 | 7.925724 |
| ENSG00000161013.15 | MGAT4B | 0.552682 | 4.621629 | 7.720281 | 5.28E-14 | 6.15E-13 | 20.45618 |
| ENSG00000107404.16 | DVL1 | 0.552667 | 4.520077 | 5.868389 | 7.49E-09 | 5.33E-08 | 8.833284 |
| ENSG00000167397.13 | VKORC1 | 0.552644 | 4.092392 | 7.21048 | 1.79E-12 | 1.82E-11 | 16.99064 |
| ENSG00000130713.14 | EXOSC2 | 0.552522 | 2.584305 | 9.675051 | 1.36E-20 | 2.61E-19 | 35.4395 |
| ENSG00000243279.3 | PRAF2 | 0.552392 | 3.98117 | 5.790251 | 1.17E-08 | 8.10E-08 | 8.403435 |
| ENSG00000138100.12 | TRIM54 | 0.552391 | 0.58751 | 5.137672 | 3.83E-07 | 2.24E-06 | 5.015774 |
| ENSG00000177398.17 | UMODL1 | 0.552383 | 0.566809 | 4.690072 | 3.43E-06 | 1.77E-05 | 2.905718 |
| ENSG00000152990.12 | GPR125 | 0.552234 | 2.126413 | 7.542845 | 1.84E-13 | 2.06E-12 | 19.22801 |
| ENSG00000167302.8 | ENTHD2 | 0.552221 | 1.856337 | 8.002785 | 6.91E-15 | 8.69E-14 | 22.45906 |
| ENSG00000173917.10 | HOXB2 | 0.552213 | 1.955541 | 3.33681 | 0.000903 | 0.003164135 | -2.37319 |
| ENSG00000068878.13 | PSME4 | 0.552161 | 3.298 | 8.782017 | 1.90E-17 | 2.94E-16 | 28.27564 |
| ENSG00000166788.8 | SAAL1 | 0.551589 | 2.059791 | 9.344091 | 2.11E-19 | 3.77E-18 | 32.72508 |
| ENSG00000125351.9 | UPF3B | 0.551549 | 2.775735 | 7.253147 | 1.34E-12 | 1.39E-11 | 17.27319 |
| ENSG00000087995.14 | METTL2A | 0.5515 | 2.619023 | 10.17137 | 1.96E-22 | 4.35E-21 | 39.63561 |
| ENSG00000278053.3 | DDX52 | 0.551382 | 2.33927 | 10.36298 | 3.68E-23 | 8.54E-22 | 41.29446 |
| ENSG00000166881.8 | TMEM194A | 0.551231 | 2.521024 | 6.502273 | 1.74E-10 | 1.46E-09 | 12.50655 |
| ENSG00000100591.6 | AHSA1 | 0.55118 | 4.969033 | 8.839132 | 1.21E-17 | 1.91E-16 | 28.7183 |
| ENSG00000165661.14 | QSOX2 | 0.550686 | 2.685308 | 7.702133 | 6.01E-14 | 6.97E-13 | 20.3295 |
| ENSG00000197457.8 | STMN3 | 0.550656 | 3.405452 | 3.48316 | 0.000533793 | 0.001952384 | -1.88401 |
| ENSG00000170448.10 | NFXL1 | 0.550615 | 1.939625 | 9.077122 | 1.84E-18 | 3.07E-17 | 30.58606 |
| ENSG00000100714.14 | MTHFD1 | 0.550553 | 3.034055 | 8.083071 | 3.84E-15 | 4.93E-14 | 23.03875 |
| ENSG00000103126.13 | AXIN1 | 0.55048 | 2.897178 | 8.317093 | 6.74E-16 | 9.19E-15 | 24.7545 |
| ENSG00000072518.19 | MARK2 | 0.550374 | 3.830779 | 8.461124 | 2.27E-16 | 3.21E-15 | 25.8295 |
| ENSG00000142937.10 | RPS8 | 0.549856 | 8.370395 | 6.255096 | 7.83E-10 | 6.17E-09 | 11.03515 |
| ENSG00000109534.15 | GAR1 | 0.549676 | 3.302003 | 9.081367 | 1.77E-18 | 2.97E-17 | 30.6197 |
| ENSG00000165973.16 | NELL1 | 0.549486 | 0.64958 | 3.380659 | 0.000772928 | 0.002743648 | -2.22874 |
| ENSG00000167967.14 | E4F1 | 0.549483 | 2.392122 | 8.5533 | 1.12E-16 | 1.63E-15 | 26.52498 |
| ENSG00000170498.8 | KISS1 | 0.549382 | 0.876554 | 5.003195 | 7.54E-07 | 4.22E-06 | 4.363311 |
| ENSG00000132819.15 | RBM38 | 0.549371 | 3.939325 | 5.185264 | 3.01E-07 | 1.78E-06 | 5.250458 |
| ENSG00000181847.10 | TIGIT | 0.549323 | 1.17912 | 6.11118 | 1.84E-09 | 1.40E-08 | 10.20131 |
| ENSG00000038382.16 | TRIO | 0.549212 | 3.003366 | 5.597866 | 3.38E-08 | 2.24E-07 | 7.366981 |
| ENSG00000111816.7 | FRK | 0.549153 | 1.78699 | 7.378385 | 5.73E-13 | 6.14E-12 | 18.11053 |
| ENSG00000143553.9 | SNAPIN | 0.549089 | 4.653331 | 7.980022 | 8.16E-15 | 1.02E-13 | 22.29554 |
| ENSG00000101337.14 | TM9SF4 | 0.549049 | 4.450709 | 9.120631 | 1.29E-18 | 2.19E-17 | 30.93152 |
| ENSG00000204435.12 | CSNK2B | 0.548995 | 4.176003 | 9.03424 | 2.59E-18 | 4.28E-17 | 30.24677 |
| ENSG00000184925.10 | LCN12 | 0.548291 | 0.690082 | 6.512411 | 1.63E-10 | 1.38E-09 | 12.56796 |
| ENSG00000147676.12 | MAL2 | 0.547691 | 6.726681 | 4.45332 | 1.02E-05 | 4.92E-05 | 1.861543 |
| ENSG00000111424.9 | VDR | 0.547676 | 3.260883 | 4.928134 | 1.09E-06 | 6.01E-06 | 4.006014 |
| ENSG00000108592.15 | FTSJ3 | 0.547662 | 3.731742 | 8.681497 | 4.16E-17 | 6.25E-16 | 27.50188 |
| ENSG00000277161.1 | PIGW | 0.547455 | 2.342274 | 8.503825 | 1.64E-16 | 2.34E-15 | 26.15096 |
| ENSG00000135912.9 | TTLL4 | 0.547423 | 2.177348 | 8.926837 | 6.08E-18 | 9.80E-17 | 29.40227 |
| ENSG00000105289.13 | TJP3 | 0.547294 | 3.383986 | 4.602505 | 5.16E-06 | 2.60E-05 | 2.513659 |
| ENSG00000143815.13 | LBR | 0.546884 | 3.827653 | 7.281417 | 1.11E-12 | 1.16E-11 | 17.46116 |
| ENSG00000183780.11 | SLC35F3 | 0.546867 | 0.768395 | 5.646039 | 2.60E-08 | 1.74E-07 | 7.623577 |
| ENSG00000105472.11 | CLEC11A | 0.546459 | 2.898012 | 5.078716 | 5.16E-07 | 2.96E-06 | 4.727781 |
| ENSG00000009709.10 | PAX7 | 0.546444 | 0.516623 | 3.376658 | 0.00078403 | 0.002778654 | -2.24199 |
| ENSG00000167792.10 | NDUFV1 | 0.546175 | 4.866097 | 8.63507 | 5.96E-17 | 8.81E-16 | 27.1468 |
| ENSG00000176371.12 | ZSCAN2 | 0.546069 | 1.290216 | 11.30983 | 6.99E-27 | 2.09E-25 | 49.79206 |
| ENSG00000108559.10 | NUP88 | 0.545927 | 3.03072 | 9.079412 | 1.80E-18 | 3.01E-17 | 30.6042 |
| ENSG00000277224.1 | HIST1H2BF | 0.54577 | 0.545468 | 4.665875 | 3.84E-06 | 1.96E-05 | 2.796696 |
| ENSG00000089154.9 | GCN1L1 | 0.545641 | 3.953641 | 7.058913 | 4.93E-12 | 4.81E-11 | 15.99815 |
| ENSG00000109118.12 | PHF12 | 0.545636 | 2.634062 | 9.361231 | 1.83E-19 | 3.29E-18 | 32.86397 |
| ENSG00000273542.1 | HIST1H4K | 0.545573 | 0.682993 | 6.504742 | 1.71E-10 | 1.44E-09 | 12.5215 |
| ENSG00000080839.10 | RBL1 | 0.545469 | 1.650097 | 7.761282 | 3.95E-14 | 4.64E-13 | 20.74327 |
| ENSG00000171865.8 | RNASEH1 | 0.545447 | 2.763642 | 9.197268 | 6.97E-19 | 1.20E-17 | 31.54302 |
| ENSG00000154102.9 | C16orf74 | 0.545235 | 1.155524 | 4.512794 | 7.78E-06 | 3.83E-05 | 2.119119 |
| ENSG00000100665.10 | SERPINA4 | 0.544832 | 0.495363 | 3.706119 | 0.000231117 | 0.000903889 | -1.10016 |
| ENSG00000163795.12 | ZNF513 | 0.544774 | 3.136929 | 7.136355 | 2.95E-12 | 2.93E-11 | 16.50305 |
| ENSG00000225526.4 | MKRN2OS | 0.54463 | 0.959982 | 6.979191 | 8.33E-12 | 7.96E-11 | 15.48321 |
| ENSG00000121058.4 | COIL | 0.544521 | 3.135338 | 10.28524 | 7.27E-23 | 1.66E-21 | 40.61886 |
| ENSG00000165891.14 | E2F7 | 0.544332 | 0.632666 | 7.150424 | 2.68E-12 | 2.68E-11 | 16.59528 |
| ENSG00000108352.10 | RAPGEFL1 | 0.544053 | 1.594992 | 5.074387 | 5.28E-07 | 3.02E-06 | 4.706758 |
| ENSG00000139372.13 | TDG | 0.543995 | 2.519915 | 7.414188 | 4.49E-13 | 4.85E-12 | 18.35208 |
| ENSG00000170425.3 | ADORA2B | 0.5439 | 2.254603 | 5.102814 | 4.57E-07 | 2.64E-06 | 4.845134 |
| ENSG00000165912.14 | PACSIN3 | 0.543709 | 3.630771 | 5.059649 | 5.68E-07 | 3.24E-06 | 4.635291 |
| ENSG00000197961.10 | ZNF121 | 0.543493 | 2.366652 | 7.56085 | 1.62E-13 | 1.82E-12 | 19.35158 |
| ENSG00000175197.9 | DDIT3 | 0.543334 | 3.957844 | 4.802633 | 2.01E-06 | 1.07E-05 | 3.419706 |
| ENSG00000015413.8 | DPEP1 | 0.543289 | 0.808632 | 5.153405 | 3.54E-07 | 2.07E-06 | 5.093136 |
| ENSG00000126001.14 | CEP250 | 0.542974 | 1.915041 | 7.221731 | 1.66E-12 | 1.70E-11 | 17.06501 |
| ENSG00000213563.5 | C8orf82 | 0.542755 | 3.032216 | 6.30359 | 5.85E-10 | 4.67E-09 | 11.31993 |
| ENSG00000131061.12 | ZNF341 | 0.542746 | 1.570737 | 9.025468 | 2.77E-18 | 4.58E-17 | 30.17751 |
| ENSG00000104835.13 | SARS2 | 0.542709 | 1.459426 | 8.31035 | 7.09E-16 | 9.65E-15 | 24.70453 |
| ENSG00000168612.4 | ZSWIM1 | 0.54267 | 2.574015 | 9.778125 | 5.70E-21 | 1.14E-19 | 36.29873 |
| ENSG00000203896.8 | LIME1 | 0.542255 | 0.976872 | 7.278431 | 1.13E-12 | 1.18E-11 | 17.44128 |
| ENSG00000185262.8 | UBALD2 | 0.542173 | 4.953402 | 6.811005 | 2.48E-11 | 2.27E-10 | 14.41306 |
| ENSG00000156011.15 | PSD3 | 0.542133 | 1.738596 | 6.854605 | 1.87E-11 | 1.73E-10 | 14.68836 |
| ENSG00000178828.6 | RNF186 | 0.541735 | 0.516409 | 5.157327 | 3.47E-07 | 2.04E-06 | 5.112459 |
| ENSG00000204469.11 | PRRC2A | 0.54168 | 5.094547 | 6.648943 | 6.96E-11 | 6.10E-10 | 13.40286 |
| ENSG00000174547.12 | MRPL11 | 0.541446 | 4.000544 | 7.045942 | 5.37E-12 | 5.23E-11 | 15.91404 |
| ENSG00000135722.7 | FBXL8 | 0.541345 | 1.780419 | 6.191319 | 1.15E-09 | 8.89E-09 | 10.66355 |
| ENSG00000213865.6 | C8orf44 | 0.541273 | 1.303062 | 8.194073 | 1.69E-15 | 2.23E-14 | 23.84776 |
| ENSG00000169180.10 | XPO6 | 0.541225 | 3.938182 | 9.418657 | 1.14E-19 | 2.08E-18 | 33.33066 |
| ENSG00000281126.1 | AC091180.1 | 0.541191 | 1.007161 | 5.556363 | 4.24E-08 | 2.78E-07 | 7.147496 |
| ENSG00000172954.12 | LCLAT1 | 0.540947 | 1.906776 | 9.777417 | 5.73E-21 | 1.14E-19 | 36.2928 |
| ENSG00000074855.9 | ANO8 | 0.54086 | 2.14941 | 5.939311 | 5.00E-09 | 3.62E-08 | 9.227849 |
| ENSG00000116922.13 | C1orf109 | 0.540818 | 2.21586 | 10.29453 | 6.71E-23 | 1.53E-21 | 40.69941 |
| ENSG00000272333.4 | KMT2B | 0.540594 | 2.905903 | 6.752712 | 3.60E-11 | 3.25E-10 | 14.04731 |
| ENSG00000161654.8 | LSM12 | 0.540282 | 2.256191 | 10.1926 | 1.63E-22 | 3.65E-21 | 39.81838 |
| ENSG00000105726.15 | ATP13A1 | 0.540156 | 3.416382 | 8.46444 | 2.21E-16 | 3.13E-15 | 25.85443 |
| ENSG00000197562.8 | RAB40C | 0.540067 | 3.734859 | 5.624626 | 2.92E-08 | 1.95E-07 | 7.509279 |
| ENSG00000135900.3 | MRPL44 | 0.53991 | 4.119232 | 10.06597 | 4.88E-22 | 1.05E-20 | 38.73218 |
| ENSG00000158793.12 | NIT1 | 0.539896 | 3.316885 | 9.593401 | 2.69E-20 | 5.10E-19 | 34.76349 |
| ENSG00000134070.4 | IRAK2 | 0.539808 | 2.205735 | 5.253783 | 2.11E-07 | 1.27E-06 | 5.591797 |
| ENSG00000178919.8 | FOXE1 | 0.539532 | 0.553065 | 4.442987 | 1.07E-05 | 5.14E-05 | 1.817117 |
| ENSG00000143303.10 | RRNAD1 | 0.539448 | 3.58248 | 7.815649 | 2.68E-14 | 3.20E-13 | 21.12585 |
| ENSG00000122140.9 | MRPS2 | 0.53942 | 3.948324 | 7.915008 | 1.31E-14 | 1.61E-13 | 21.83057 |
| ENSG00000184967.5 | NOC4L | 0.539229 | 3.450062 | 7.850334 | 2.09E-14 | 2.52E-13 | 21.37104 |
| ENSG00000088035.14 | ALG6 | 0.539103 | 2.015042 | 10.27987 | 7.62E-23 | 1.74E-21 | 40.57238 |
| ENSG00000138623.8 | SEMA7A | 0.539063 | 2.161317 | 4.337982 | 1.70E-05 | 7.96E-05 | 1.371115 |
| ENSG00000181652.17 | ATG9B | 0.538797 | 0.847291 | 5.617421 | 3.04E-08 | 2.02E-07 | 7.470907 |
| ENSG00000184207.8 | PGP | 0.538665 | 3.031492 | 7.67727 | 7.16E-14 | 8.25E-13 | 20.15634 |
| ENSG00000197728.8 | RPS26 | 0.538646 | 5.924942 | 4.455813 | 1.01E-05 | 4.87E-05 | 1.872274 |
| ENSG00000180185.10 | FAHD1 | 0.538531 | 3.236295 | 9.194218 | 7.15E-19 | 1.23E-17 | 31.51861 |
| ENSG00000174669.10 | SLC29A2 | 0.538453 | 3.290852 | 5.050688 | 5.95E-07 | 3.38E-06 | 4.591934 |
| ENSG00000107821.13 | KAZALD1 | 0.538439 | 1.735007 | 5.560183 | 4.16E-08 | 2.72E-07 | 7.167634 |
| ENSG00000161277.10 | THAP8 | 0.538389 | 2.290375 | 7.358682 | 6.56E-13 | 6.99E-12 | 17.97801 |
| ENSG00000134369.14 | NAV1 | 0.538337 | 1.733305 | 6.235942 | 8.79E-10 | 6.88E-09 | 10.9232 |
| ENSG00000033011.10 | ALG1 | 0.537968 | 2.696357 | 9.596468 | 2.62E-20 | 4.97E-19 | 34.78881 |
| ENSG00000108848.14 | LUC7L3 | 0.537921 | 3.798187 | 6.450261 | 2.40E-10 | 1.99E-09 | 12.19285 |
| ENSG00000023892.10 | DEF6 | 0.537889 | 3.435662 | 6.102941 | 1.93E-09 | 1.46E-08 | 10.15409 |
| ENSG00000126705.12 | AHDC1 | 0.53787 | 2.743205 | 5.768919 | 1.31E-08 | 9.07E-08 | 8.286976 |
| ENSG00000122643.17 | NT5C3A | 0.537658 | 3.271566 | 7.085104 | 4.14E-12 | 4.08E-11 | 16.1684 |
| ENSG00000100744.13 | GSKIP | 0.537619 | 2.969482 | 6.838096 | 2.08E-11 | 1.92E-10 | 14.58394 |
| ENSG00000103067.10 | ESRP2 | 0.537561 | 3.775669 | 5.462912 | 7.02E-08 | 4.48E-07 | 6.658646 |
| ENSG00000161202.16 | DVL3 | 0.537485 | 4.06396 | 7.305954 | 9.40E-13 | 9.89E-12 | 17.62481 |
| ENSG00000005206.15 | SPPL2B | 0.537479 | 2.710811 | 5.889724 | 6.64E-09 | 4.75E-08 | 8.951537 |
| ENSG00000163191.5 | S100A11 | 0.537442 | 9.546758 | 5.553436 | 4.31E-08 | 2.82E-07 | 7.132068 |
| ENSG00000158882.11 | TOMM40L | 0.537276 | 2.750165 | 7.863464 | 1.90E-14 | 2.30E-13 | 21.46409 |
| ENSG00000188707.5 | ZBED6CL | 0.5371 | 2.492888 | 5.839911 | 8.81E-09 | 6.22E-08 | 8.676033 |
| ENSG00000100426.6 | ZBED4 | 0.536512 | 2.527415 | 9.191232 | 7.32E-19 | 1.26E-17 | 31.49472 |
| ENSG00000094975.12 | SUCO | 0.536396 | 3.231887 | 7.888548 | 1.58E-14 | 1.93E-13 | 21.64219 |
| ENSG00000124222.20 | STX16 | 0.536216 | 3.858327 | 6.412519 | 3.02E-10 | 2.48E-09 | 11.96656 |
| ENSG00000196787.3 | HIST1H2AG | 0.536101 | 0.645305 | 5.542509 | 4.57E-08 | 2.98E-07 | 7.074556 |
| ENSG00000174529.7 | TMEM81 | 0.536064 | 1.561796 | 8.814426 | 1.48E-17 | 2.30E-16 | 28.52655 |
| ENSG00000110218.7 | PANX1 | 0.535963 | 3.265177 | 7.181441 | 2.18E-12 | 2.20E-11 | 16.79912 |
| ENSG00000067829.17 | IDH3G | 0.53594 | 4.471874 | 8.600387 | 7.80E-17 | 1.14E-15 | 26.8825 |
| ENSG00000144401.13 | METTL21A | 0.53583 | 1.741394 | 10.58492 | 5.16E-24 | 1.27E-22 | 43.24214 |
| ENSG00000167280.15 | ENGASE | 0.535749 | 2.456795 | 4.994026 | 7.89E-07 | 4.41E-06 | 4.319398 |
| ENSG00000141560.13 | FN3KRP | 0.535697 | 4.008016 | 8.717171 | 3.16E-17 | 4.78E-16 | 27.7757 |
| ENSG00000075643.5 | MOCOS | 0.535649 | 1.811506 | 5.681425 | 2.14E-08 | 1.45E-07 | 7.81331 |
| ENSG00000151806.12 | GUF1 | 0.535631 | 2.864344 | 9.009527 | 3.15E-18 | 5.18E-17 | 30.05178 |
| ENSG00000136490.7 | LIMD2 | 0.535323 | 2.746382 | 4.821761 | 1.83E-06 | 9.78E-06 | 3.508168 |
| ENSG00000105327.14 | BBC3 | 0.535297 | 2.041263 | 6.930253 | 1.15E-11 | 1.08E-10 | 15.16955 |
| ENSG00000106144.18 | CASP2 | 0.535132 | 2.750781 | 8.229004 | 1.30E-15 | 1.73E-14 | 24.10415 |
| ENSG00000168002.10 | POLR2G | 0.53487 | 4.774807 | 8.354439 | 5.08E-16 | 7.01E-15 | 25.03186 |
| ENSG00000114021.10 | NIT2 | 0.534466 | 2.431495 | 9.185509 | 7.67E-19 | 1.32E-17 | 31.44895 |
| ENSG00000007062.10 | PROM1 | 0.534258 | 1.583436 | 2.537591 | 0.011428735 | 0.031232793 | -4.68566 |
| ENSG00000143851.14 | PTPN7 | 0.53423 | 1.797118 | 5.367472 | 1.17E-07 | 7.25E-07 | 6.167104 |
| ENSG00000243317.6 | C7orf73 | 0.534184 | 3.76989 | 8.135176 | 2.61E-15 | 3.40E-14 | 23.41742 |
| ENSG00000117523.14 | PRRC2C | 0.53414 | 4.096236 | 6.418275 | 2.92E-10 | 2.40E-09 | 12.001 |
| ENSG00000204604.8 | ZNF468 | 0.534123 | 2.297865 | 7.51503 | 2.23E-13 | 2.48E-12 | 19.03759 |
| ENSG00000100167.18 | 3-Sep | 0.53382 | 1.033835 | 4.787628 | 2.16E-06 | 1.14E-05 | 3.35054 |
| ENSG00000007392.15 | LUC7L | 0.533697 | 2.810848 | 6.035916 | 2.86E-09 | 2.12E-08 | 9.772015 |
| ENSG00000204385.9 | SLC44A4 | 0.533689 | 4.930462 | 2.738418 | 0.006367754 | 0.018560112 | -4.16212 |
| ENSG00000125445.9 | MRPS7 | 0.53351 | 4.306579 | 8.381612 | 4.14E-16 | 5.75E-15 | 25.23427 |
| ENSG00000196208.12 | GREB1 | 0.5334 | 0.658679 | 5.819451 | 9.89E-09 | 6.93E-08 | 8.563471 |
| ENSG00000243989.6 | ACY1 | 0.53334 | 1.279005 | 9.151233 | 1.01E-18 | 1.72E-17 | 31.17524 |
| ENSG00000086504.14 | MRPL28 | 0.533277 | 4.446192 | 8.066399 | 4.34E-15 | 5.55E-14 | 22.918 |
| ENSG00000167608.10 | TMC4 | 0.533189 | 4.98312 | 4.554019 | 6.45E-06 | 3.20E-05 | 2.299528 |
| ENSG00000130489.11 | SCO2 | 0.533189 | 3.951466 | 6.231567 | 9.02E-10 | 7.06E-09 | 10.89767 |
| ENSG00000159184.7 | HOXB13 | 0.533048 | 0.489436 | 4.57563 | 5.84E-06 | 2.92E-05 | 2.394711 |
| ENSG00000187514.13 | PTMA | 0.532953 | 7.790625 | 9.262696 | 4.10E-19 | 7.20E-18 | 32.06807 |
| ENSG00000224051.5 | CPTP | 0.532825 | 3.830328 | 6.92405 | 1.19E-11 | 1.13E-10 | 15.12993 |
| ENSG00000124659.6 | TBCC | 0.532786 | 4.019013 | 8.143863 | 2.45E-15 | 3.20E-14 | 23.48074 |
| ENSG00000141985.8 | SH3GL1 | 0.532744 | 4.446861 | 7.861865 | 1.92E-14 | 2.33E-13 | 21.45275 |
| ENSG00000153046.16 | CDYL | 0.532667 | 3.026898 | 8.784084 | 1.87E-17 | 2.89E-16 | 28.29162 |
| ENSG00000143727.14 | ACP1 | 0.532583 | 4.393199 | 9.573264 | 3.18E-20 | 6.00E-19 | 34.5974 |
| ENSG00000120068.6 | HOXB8 | 0.532386 | 0.651104 | 3.608676 | 0.000335001 | 0.001272011 | -1.44845 |
| ENSG00000160208.12 | RRP1B | 0.531969 | 3.228383 | 8.185364 | 1.80E-15 | 2.38E-14 | 23.78397 |
| ENSG00000132581.8 | SDF2 | 0.531899 | 4.002586 | 8.888616 | 8.23E-18 | 1.31E-16 | 29.10358 |
| ENSG00000188157.12 | AGRN | 0.531892 | 5.212758 | 4.27767 | 2.22E-05 | 0.000101929 | 1.119456 |
| ENSG00000196670.12 | ZFP62 | 0.531847 | 2.594107 | 7.656834 | 8.28E-14 | 9.49E-13 | 20.01435 |
| ENSG00000112855.13 | HARS2 | 0.53161 | 3.426584 | 9.807348 | 4.45E-21 | 8.97E-20 | 36.54351 |
| ENSG00000100023.16 | PPIL2 | 0.531477 | 2.882262 | 7.879772 | 1.69E-14 | 2.06E-13 | 21.57983 |
| ENSG00000170892.9 | TSEN34 | 0.531123 | 4.014071 | 8.881081 | 8.73E-18 | 1.39E-16 | 29.0448 |
| ENSG00000010165.18 | METTL13 | 0.531088 | 3.691747 | 9.306516 | 2.87E-19 | 5.08E-18 | 32.42126 |
| ENSG00000170854.16 | MINA | 0.530915 | 2.167433 | 9.656565 | 1.59E-20 | 3.04E-19 | 35.28609 |
| ENSG00000001629.8 | ANKIB1 | 0.530709 | 3.32065 | 7.560995 | 1.62E-13 | 1.82E-12 | 19.35257 |
| ENSG00000163482.10 | STK36 | 0.530533 | 2.252085 | 6.350767 | 4.40E-10 | 3.56E-09 | 11.59881 |
| ENSG00000160959.6 | LRRC14 | 0.530249 | 2.588318 | 7.433373 | 3.93E-13 | 4.27E-12 | 18.48191 |
| ENSG00000178741.10 | COX5A | 0.530067 | 5.603183 | 7.068803 | 4.62E-12 | 4.53E-11 | 16.06237 |
| ENSG00000112029.8 | FBXO5 | 0.529789 | 1.659814 | 6.728121 | 4.21E-11 | 3.78E-10 | 13.89382 |
| ENSG00000178776.4 | C5orf46 | 0.52972 | 0.749059 | 5.535239 | 4.76E-08 | 3.09E-07 | 7.036344 |
| ENSG00000188389.9 | PDCD1 | 0.529513 | 1.324408 | 4.927303 | 1.10E-06 | 6.03E-06 | 4.002085 |
| ENSG00000189091.11 | SF3B3 | 0.52951 | 3.791308 | 8.613971 | 7.02E-17 | 1.03E-15 | 26.98592 |
| ENSG00000142168.13 | SOD1 | 0.52915 | 6.243646 | 7.021355 | 6.31E-12 | 6.10E-11 | 15.75494 |
| ENSG00000165672.6 | PRDX3 | 0.528842 | 5.375781 | 7.162979 | 2.47E-12 | 2.47E-11 | 16.6777 |
| ENSG00000167965.16 | MLST8 | 0.528597 | 3.327788 | 8.899765 | 7.53E-18 | 1.20E-16 | 29.1906 |
| ENSG00000184220.9 | CMSS1 | 0.528523 | 2.326056 | 7.122898 | 3.22E-12 | 3.20E-11 | 16.41499 |
| ENSG00000168958.18 | MFF | 0.528471 | 3.353647 | 8.168831 | 2.04E-15 | 2.68E-14 | 23.66303 |
| ENSG00000213420.6 | GPC2 | 0.528402 | 0.571748 | 5.942876 | 4.90E-09 | 3.55E-08 | 9.247791 |
| ENSG00000241127.6 | YAE1D1 | 0.528358 | 2.388561 | 7.767679 | 3.77E-14 | 4.45E-13 | 20.78818 |
| ENSG00000196365.10 | LONP1 | 0.528145 | 4.200258 | 7.546017 | 1.80E-13 | 2.01E-12 | 19.24976 |
| ENSG00000063244.11 | U2AF2 | 0.52813 | 5.156273 | 9.112089 | 1.39E-18 | 2.34E-17 | 30.8636 |
| ENSG00000047230.13 | CTPS2 | 0.527879 | 2.691236 | 8.12537 | 2.81E-15 | 3.65E-14 | 23.34601 |
| ENSG00000123064.11 | DDX54 | 0.527854 | 3.951862 | 7.349 | 7.01E-13 | 7.46E-12 | 17.913 |
| ENSG00000087269.14 | NOP14 | 0.527677 | 3.838689 | 8.896308 | 7.74E-18 | 1.24E-16 | 29.16361 |
| ENSG00000008735.13 | MAPK8IP2 | 0.527653 | 1.144204 | 4.576407 | 5.82E-06 | 2.91E-05 | 2.398139 |
| ENSG00000214510.8 | SPINK13 | 0.527639 | 0.651387 | 4.053611 | 5.75E-05 | 0.000249054 | 0.213555 |
| ENSG00000163714.16 | U2SURP | 0.52753 | 3.241026 | 8.057636 | 4.63E-15 | 5.90E-14 | 22.85461 |
| ENSG00000111321.9 | LTBR | 0.527512 | 4.564042 | 6.124028 | 1.71E-09 | 1.30E-08 | 10.27506 |
| ENSG00000005175.8 | RPAP3 | 0.527262 | 2.69202 | 9.880591 | 2.39E-21 | 4.91E-20 | 37.15929 |
| ENSG00000107949.15 | BCCIP | 0.527149 | 3.54319 | 8.662659 | 4.82E-17 | 7.17E-16 | 27.35763 |
| ENSG00000112079.8 | STK38 | 0.527008 | 4.397731 | 7.658613 | 8.17E-14 | 9.37E-13 | 20.0267 |
| ENSG00000143612.17 | C1orf43 | 0.526967 | 6.698111 | 6.921041 | 1.22E-11 | 1.15E-10 | 15.11072 |
| ENSG00000162723.8 | SLAMF9 | 0.52689 | 0.773386 | 5.731119 | 1.62E-08 | 1.11E-07 | 8.081549 |
| ENSG00000149016.14 | TUT1 | 0.526855 | 2.203641 | 8.586144 | 8.70E-17 | 1.27E-15 | 26.7742 |
| ENSG00000157916.17 | RER1 | 0.526851 | 4.330131 | 10.02932 | 6.70E-22 | 1.42E-20 | 38.41966 |
| ENSG00000170632.12 | ARMC10 | 0.526724 | 3.098355 | 8.601882 | 7.71E-17 | 1.13E-15 | 26.89388 |
| ENSG00000090104.10 | RGS1 | 0.526077 | 4.129392 | 3.097872 | 0.002045722 | 0.006664407 | -3.1284 |
| ENSG00000136247.13 | ZDHHC4 | 0.52606 | 3.904957 | 8.352238 | 5.17E-16 | 7.12E-15 | 25.01549 |
| ENSG00000176973.7 | FAM89B | 0.526012 | 4.138211 | 6.692578 | 5.28E-11 | 4.69E-10 | 13.67281 |
| ENSG00000101463.5 | SYNDIG1 | 0.525997 | 0.880881 | 5.554204 | 4.29E-08 | 2.81E-07 | 7.136117 |
| ENSG00000114770.15 | ABCC5 | 0.525824 | 2.222944 | 5.531926 | 4.84E-08 | 3.15E-07 | 7.018945 |
| ENSG00000078668.12 | VDAC3 | 0.525801 | 4.768646 | 5.940126 | 4.97E-09 | 3.60E-08 | 9.232405 |
| ENSG00000165171.9 | WBSCR27 | 0.525545 | 2.085136 | 3.908507 | 0.000104101 | 0.000432472 | -0.34858 |
| ENSG00000114850.5 | SSR3 | 0.525538 | 5.646034 | 6.945595 | 1.04E-11 | 9.83E-11 | 15.26768 |
| ENSG00000167645.15 | YIF1B | 0.525353 | 3.555632 | 7.183514 | 2.15E-12 | 2.17E-11 | 16.81277 |
| ENSG00000023572.7 | GLRX2 | 0.525238 | 2.647063 | 8.126751 | 2.78E-15 | 3.61E-14 | 23.35606 |
| ENSG00000155115.6 | GTF3C6 | 0.525179 | 5.153305 | 8.107325 | 3.21E-15 | 4.15E-14 | 23.21477 |
| ENSG00000166477.11 | LEO1 | 0.525113 | 3.139271 | 9.440561 | 9.55E-20 | 1.75E-18 | 33.50921 |
| ENSG00000177565.14 | TBL1XR1 | 0.524936 | 3.870033 | 8.257844 | 1.05E-15 | 1.41E-14 | 24.31648 |
| ENSG00000147130.13 | ZMYM3 | 0.524909 | 3.152965 | 7.751144 | 4.24E-14 | 4.97E-13 | 20.67217 |
| ENSG00000266967.5 | AARSD1 | 0.524617 | 1.265552 | 9.714405 | 9.76E-21 | 1.91E-19 | 35.76679 |
| ENSG00000100997.17 | ABHD12 | 0.524301 | 4.62208 | 7.098655 | 3.79E-12 | 3.73E-11 | 16.25668 |
| ENSG00000089195.13 | TRMT6 | 0.5238 | 2.801581 | 7.452702 | 3.44E-13 | 3.76E-12 | 18.61299 |
| ENSG00000134882.14 | UBAC2 | 0.523672 | 4.09779 | 9.159366 | 9.47E-19 | 1.62E-17 | 31.24012 |
| ENSG00000150779.10 | TIMM8B | 0.522629 | 4.532571 | 5.520121 | 5.16E-08 | 3.34E-07 | 6.957029 |
| ENSG00000140564.9 | FURIN | 0.52248 | 5.780014 | 3.379104 | 0.000777225 | 0.002757769 | -2.23389 |
| ENSG00000175550.6 | DRAP1 | 0.522188 | 5.237558 | 6.547462 | 1.31E-10 | 1.12E-09 | 12.78088 |
| ENSG00000122378.12 | FAM213A | 0.522183 | 3.530356 | 7.931058 | 1.16E-14 | 1.44E-13 | 21.94507 |
| ENSG00000166526.15 | ZNF3 | 0.521986 | 2.845989 | 8.58817 | 8.57E-17 | 1.25E-15 | 26.7896 |
| ENSG00000162006.8 | MSLNL | 0.521747 | 0.705787 | 3.765996 | 0.000183221 | 0.000729641 | -0.88176 |
| ENSG00000142186.15 | SCYL1 | 0.521736 | 4.670585 | 8.631882 | 6.11E-17 | 9.03E-16 | 27.12248 |
| ENSG00000169436.15 | COL22A1 | 0.521723 | 0.553352 | 5.76553 | 1.34E-08 | 9.24E-08 | 8.26851 |
| ENSG00000104859.13 | CLASRP | 0.521721 | 2.867355 | 6.170185 | 1.30E-09 | 1.00E-08 | 10.54114 |
| ENSG00000173821.18 | RNF213 | 0.521711 | 3.693553 | 4.900931 | 1.25E-06 | 6.80E-06 | 3.87775 |
| ENSG00000213337.7 | ANKRD39 | 0.521458 | 1.664081 | 10.62853 | 3.49E-24 | 8.70E-23 | 43.62817 |
| ENSG00000106244.11 | PDAP1 | 0.521457 | 5.276904 | 7.735301 | 4.75E-14 | 5.55E-13 | 20.56121 |
| ENSG00000151376.15 | ME3 | 0.521439 | 2.280577 | 5.932297 | 5.20E-09 | 3.76E-08 | 9.18864 |
| ENSG00000258890.5 | CEP95 | 0.521174 | 2.129699 | 8.381361 | 4.15E-16 | 5.76E-15 | 25.2324 |
| ENSG00000135074.14 | ADAM19 | 0.52105 | 2.111251 | 4.700275 | 3.27E-06 | 1.69E-05 | 2.951842 |
| ENSG00000138050.13 | THUMPD2 | 0.521022 | 1.990339 | 11.05775 | 7.17E-26 | 2.00E-24 | 47.48256 |
| ENSG00000065427.13 | KARS | 0.520986 | 5.110441 | 9.331534 | 2.34E-19 | 4.17E-18 | 32.62345 |
| ENSG00000114388.11 | NPRL2 | 0.520338 | 2.664308 | 9.640235 | 1.82E-20 | 3.47E-19 | 35.15075 |
| ENSG00000146555.17 | SDK1 | 0.520262 | 1.808836 | 3.720729 | 0.000218449 | 0.000857671 | -1.04718 |
| ENSG00000197217.11 | ENTPD4 | 0.519959 | 3.132274 | 6.686338 | 5.49E-11 | 4.87E-10 | 13.63411 |
| ENSG00000274641.1 | HIST1H2BO | 0.519815 | 0.542712 | 4.695944 | 3.33E-06 | 1.72E-05 | 2.932252 |
| ENSG00000157637.11 | SLC38A10 | 0.519808 | 4.679316 | 6.320506 | 5.28E-10 | 4.23E-09 | 11.41972 |
| ENSG00000106976.17 | DNM1 | 0.519737 | 1.166002 | 6.036319 | 2.85E-09 | 2.12E-08 | 9.7743 |
| ENSG00000196363.8 | WDR5 | 0.519705 | 3.676957 | 8.456978 | 2.34E-16 | 3.31E-15 | 25.79836 |
| ENSG00000144029.10 | MRPS5 | 0.519652 | 3.165468 | 10.19722 | 1.57E-22 | 3.51E-21 | 39.85816 |
| ENSG00000104879.4 | CKM | 0.518873 | 0.60112 | 4.801555 | 2.02E-06 | 1.07E-05 | 3.41473 |
| ENSG00000185730.6 | ZNF696 | 0.517955 | 1.556276 | 8.090149 | 3.64E-15 | 4.69E-14 | 23.09008 |
| ENSG00000181315.9 | ZNF322 | 0.517845 | 1.407981 | 5.351401 | 1.27E-07 | 7.85E-07 | 6.085103 |
| ENSG00000142684.7 | ZNF593 | 0.517485 | 1.655648 | 8.238309 | 1.22E-15 | 1.62E-14 | 24.17259 |
| ENSG00000108395.12 | TRIM37 | 0.517476 | 2.789414 | 7.357825 | 6.60E-13 | 7.03E-12 | 17.97226 |
| ENSG00000159069.12 | FBXW5 | 0.51745 | 5.154538 | 6.174851 | 1.26E-09 | 9.76E-09 | 10.56813 |
| ENSG00000175279.20 | APITD1 | 0.517244 | 1.664725 | 7.914133 | 1.32E-14 | 1.62E-13 | 21.82432 |
| ENSG00000158411.9 | MITD1 | 0.517201 | 2.263692 | 9.27138 | 3.82E-19 | 6.72E-18 | 32.13796 |
| ENSG00000172531.13 | PPP1CA | 0.517197 | 6.158263 | 8.929086 | 5.97E-18 | 9.64E-17 | 29.41988 |
| ENSG00000168374.9 | ARF4 | 0.517196 | 6.641182 | 8.433217 | 2.80E-16 | 3.94E-15 | 25.62009 |
| ENSG00000162542.12 | TMCO4 | 0.517192 | 3.225907 | 7.708885 | 5.73E-14 | 6.65E-13 | 20.3766 |
| ENSG00000173473.9 | SMARCC1 | 0.517146 | 4.061577 | 7.240219 | 1.47E-12 | 1.51E-11 | 17.18743 |
| ENSG00000112249.12 | ASCC3 | 0.516876 | 2.438825 | 7.639175 | 9.37E-14 | 1.07E-12 | 19.8919 |
| ENSG00000049249.7 | TNFRSF9 | 0.51662 | 0.954826 | 5.910377 | 5.90E-09 | 4.24E-08 | 9.066374 |
| ENSG00000179115.9 | FARSA | 0.516425 | 4.526724 | 8.674433 | 4.40E-17 | 6.57E-16 | 27.44776 |
| ENSG00000152382.5 | TADA1 | 0.51634 | 2.690078 | 9.035392 | 2.56E-18 | 4.24E-17 | 30.25586 |
| ENSG00000173545.4 | ZNF622 | 0.516213 | 4.471162 | 8.088947 | 3.68E-15 | 4.73E-14 | 23.08136 |
| ENSG00000136950.12 | ARPC5L | 0.516111 | 4.039283 | 8.67806 | 4.28E-17 | 6.40E-16 | 27.47554 |
| ENSG00000127838.12 | PNKD | 0.516088 | 4.651119 | 5.423165 | 8.68E-08 | 5.49E-07 | 6.452991 |
| ENSG00000197757.7 | HOXC6 | 0.515825 | 0.657385 | 5.461944 | 7.06E-08 | 4.50E-07 | 6.653624 |
| ENSG00000117450.12 | PRDX1 | 0.515566 | 7.735796 | 5.374166 | 1.13E-07 | 7.01E-07 | 6.201328 |
| ENSG00000121236.18 | TRIM6 | 0.515564 | 1.29858 | 5.357976 | 1.23E-07 | 7.60E-07 | 6.118625 |
| ENSG00000133398.3 | MED10 | 0.515515 | 3.958772 | 6.327908 | 5.05E-10 | 4.06E-09 | 11.46345 |
| ENSG00000119403.12 | PHF19 | 0.515513 | 2.387448 | 6.717544 | 4.51E-11 | 4.03E-10 | 13.82795 |
| ENSG00000131652.12 | THOC6 | 0.51525 | 4.058278 | 7.887298 | 1.60E-14 | 1.95E-13 | 21.63331 |
| ENSG00000154518.8 | ATP5G3 | 0.515138 | 4.415198 | 7.310972 | 9.09E-13 | 9.58E-12 | 17.65834 |
| ENSG00000130699.15 | TAF4 | 0.514998 | 1.855601 | 9.001006 | 3.37E-18 | 5.53E-17 | 29.98464 |
| ENSG00000112655.14 | PTK7 | 0.514568 | 4.276236 | 4.513081 | 7.77E-06 | 3.82E-05 | 2.120372 |
| ENSG00000183077.14 | AFMID | 0.514489 | 3.052004 | 6.979166 | 8.33E-12 | 7.96E-11 | 15.48305 |
| ENSG00000090539.14 | CHRD | 0.514198 | 1.284301 | 4.26886 | 2.30E-05 | 0.000105692 | 1.082973 |
| ENSG00000116288.11 | PARK7 | 0.514181 | 6.135058 | 9.325164 | 2.46E-19 | 4.38E-18 | 32.57193 |
| ENSG00000104356.9 | POP1 | 0.51415 | 1.468061 | 8.882209 | 8.65E-18 | 1.38E-16 | 29.0536 |
| ENSG00000083845.7 | RPS5 | 0.513809 | 7.121945 | 5.067571 | 5.46E-07 | 3.12E-06 | 4.673682 |
| ENSG00000100029.16 | PES1 | 0.513762 | 4.082929 | 8.981897 | 3.93E-18 | 6.41E-17 | 29.83425 |
| ENSG00000148399.10 | DPH7 | 0.513661 | 2.339454 | 7.844007 | 2.18E-14 | 2.63E-13 | 21.32625 |
| ENSG00000167393.15 | PPP2R3B | 0.513613 | 1.205901 | 7.868987 | 1.82E-14 | 2.22E-13 | 21.50327 |
| ENSG00000104529.16 | EEF1D | 0.51359 | 3.974626 | 7.115667 | 3.38E-12 | 3.35E-11 | 16.36772 |
| ENSG00000127054.17 | CPSF3L | 0.513586 | 3.483655 | 8.549087 | 1.16E-16 | 1.68E-15 | 26.49307 |
| ENSG00000006327.12 | TNFRSF12A | 0.513195 | 5.241911 | 3.288446 | 0.00106989 | 0.00369709 | -2.53042 |
| ENSG00000173531.14 | MST1 | 0.513168 | 1.017618 | 6.248335 | 8.16E-10 | 6.41E-09 | 10.9956 |
| ENSG00000204316.11 | MRPL38 | 0.513112 | 1.680721 | 9.416946 | 1.16E-19 | 2.11E-18 | 33.31672 |
| ENSG00000112996.8 | MRPS30 | 0.512842 | 2.800075 | 8.36922 | 4.55E-16 | 6.29E-15 | 25.1419 |
| ENSG00000254004.5 | ZNF260 | 0.512796 | 2.417324 | 7.365209 | 6.28E-13 | 6.70E-12 | 18.02188 |
| ENSG00000115053.14 | NCL | 0.512637 | 6.291215 | 7.57002 | 1.52E-13 | 1.71E-12 | 19.4146 |
| ENSG00000159593.13 | NAE1 | 0.512621 | 3.180554 | 9.244492 | 4.75E-19 | 8.30E-18 | 31.92171 |
| ENSG00000176531.9 | PHLDB3 | 0.512611 | 1.471462 | 7.496758 | 2.54E-13 | 2.80E-12 | 18.91282 |
| ENSG00000080608.9 | KIAA0020 | 0.512526 | 3.480646 | 6.606058 | 9.11E-11 | 7.88E-10 | 13.13902 |
| ENSG00000008130.14 | NADK | 0.512491 | 3.94293 | 7.691906 | 6.46E-14 | 7.47E-13 | 20.25822 |
| ENSG00000198892.6 | SHISA4 | 0.512365 | 3.421333 | 5.235139 | 2.33E-07 | 1.40E-06 | 5.498518 |
| ENSG00000100749.6 | VRK1 | 0.512352 | 2.430007 | 6.360116 | 4.16E-10 | 3.37E-09 | 11.65429 |
| ENSG00000136875.11 | PRPF4 | 0.512091 | 3.443269 | 8.545587 | 1.19E-16 | 1.72E-15 | 26.46656 |
| ENSG00000273611.3 | ZNHIT3 | 0.512003 | 2.403143 | 10.27735 | 7.79E-23 | 1.77E-21 | 40.55048 |
| ENSG00000081870.10 | HSPB11 | 0.511889 | 2.750985 | 7.555189 | 1.69E-13 | 1.89E-12 | 19.3127 |
| ENSG00000102962.4 | CCL22 | 0.511754 | 2.144415 | 3.345954 | 0.000874302 | 0.003073918 | -2.34322 |
| ENSG00000162819.10 | BROX | 0.511706 | 4.009317 | 8.103013 | 3.31E-15 | 4.28E-14 | 23.18345 |
| ENSG00000141401.10 | IMPA2 | 0.511675 | 4.181937 | 3.882208 | 0.000115703 | 0.000476812 | -0.44839 |
| ENSG00000108021.18 | FAM208B | 0.511501 | 2.656234 | 7.433305 | 3.93E-13 | 4.27E-12 | 18.48145 |
| ENSG00000174243.8 | DDX23 | 0.511404 | 4.676533 | 9.503896 | 5.66E-20 | 1.05E-18 | 34.0272 |
| ENSG00000102125.14 | TAZ | 0.510955 | 2.753902 | 6.747342 | 3.73E-11 | 3.36E-10 | 14.01375 |
| ENSG00000138385.14 | SSB | 0.510854 | 4.280861 | 8.868955 | 9.61E-18 | 1.52E-16 | 28.9503 |
| ENSG00000177679.15 | SRRM3 | 0.510841 | 0.625783 | 7.825888 | 2.49E-14 | 2.99E-13 | 21.19814 |
| ENSG00000160993.3 | ALKBH4 | 0.510711 | 2.618955 | 8.807842 | 1.55E-17 | 2.42E-16 | 28.47553 |
| ENSG00000196923.12 | PDLIM7 | 0.510635 | 3.654034 | 4.984771 | 8.26E-07 | 4.61E-06 | 4.275155 |
| ENSG00000162521.17 | RBBP4 | 0.510583 | 4.263032 | 8.896613 | 7.72E-18 | 1.23E-16 | 29.16599 |
| ENSG00000162885.11 | B3GALNT2 | 0.510574 | 1.573123 | 7.965479 | 9.07E-15 | 1.13E-13 | 22.19127 |
| ENSG00000137642.11 | SORL1 | 0.510524 | 2.721911 | 3.907998 | 0.000104315 | 0.0004333 | -0.35052 |
| ENSG00000075151.18 | EIF4G3 | 0.510451 | 3.228125 | 7.81193 | 2.75E-14 | 3.28E-13 | 21.0996 |
| ENSG00000168256.16 | NKIRAS2 | 0.510431 | 3.352465 | 8.779044 | 1.95E-17 | 3.00E-16 | 28.25266 |
| ENSG00000105707.12 | HPN | 0.510114 | 3.732756 | 2.829244 | 0.004831114 | 0.014491905 | -3.9126 |
| ENSG00000197756.8 | RPL37A | 0.509878 | 6.62006 | 6.515638 | 1.60E-10 | 1.35E-09 | 12.58752 |
| ENSG00000146223.13 | RPL7L1 | 0.509641 | 3.913411 | 8.806082 | 1.58E-17 | 2.45E-16 | 28.46189 |
| ENSG00000117595.9 | IRF6 | 0.50959 | 3.830953 | 5.220213 | 2.51E-07 | 1.50E-06 | 5.424055 |
| ENSG00000198131.12 | ZNF544 | 0.509324 | 2.16575 | 7.688722 | 6.61E-14 | 7.64E-13 | 20.23604 |
| ENSG00000117362.11 | APH1A | 0.509267 | 6.247001 | 8.781205 | 1.91E-17 | 2.96E-16 | 28.26936 |
| ENSG00000162576.15 | MXRA8 | 0.509245 | 4.482191 | 3.471439 | 0.000557132 | 0.002030675 | -1.92393 |
| ENSG00000114054.12 | PCCB | 0.508776 | 2.67087 | 7.309595 | 9.17E-13 | 9.66E-12 | 17.64913 |
| ENSG00000167775.9 | CD320 | 0.508762 | 3.964836 | 5.252962 | 2.12E-07 | 1.28E-06 | 5.587681 |
| ENSG00000177548.11 | RABEP2 | 0.508732 | 2.836561 | 6.722108 | 4.38E-11 | 3.92E-10 | 13.85636 |
| ENSG00000124575.6 | HIST1H1D | 0.508426 | 0.501203 | 3.970243 | 8.10E-05 | 0.000342986 | -0.11178 |
| ENSG00000173218.13 | VANGL1 | 0.508425 | 2.109681 | 7.217668 | 1.71E-12 | 1.74E-11 | 17.03814 |
| ENSG00000144115.15 | THNSL2 | 0.508339 | 2.398316 | 3.919094 | 9.97E-05 | 0.000415791 | -0.30822 |
| ENSG00000185479.5 | KRT6B | 0.508338 | 0.600486 | 3.233941 | 0.001292024 | 0.004393899 | -2.70496 |
| ENSG00000087299.10 | L2HGDH | 0.50822 | 1.319093 | 9.043248 | 2.41E-18 | 3.99E-17 | 30.31794 |
| ENSG00000115211.14 | EIF2B4 | 0.50768 | 3.04221 | 11.43175 | 2.24E-27 | 6.88E-26 | 50.92082 |
| ENSG00000039650.8 | PNKP | 0.507571 | 3.092561 | 7.612695 | 1.13E-13 | 1.28E-12 | 19.70872 |
| ENSG00000103249.16 | CLCN7 | 0.50739 | 3.373433 | 6.854053 | 1.88E-11 | 1.74E-10 | 14.68487 |
| ENSG00000100147.12 | CCDC134 | 0.507388 | 1.971705 | 8.790106 | 1.79E-17 | 2.77E-16 | 28.3382 |
| ENSG00000147789.14 | ZNF7 | 0.507257 | 1.707557 | 9.664577 | 1.48E-20 | 2.84E-19 | 35.35256 |
| ENSG00000124383.7 | MPHOSPH10 | 0.507196 | 3.252108 | 10.54224 | 7.54E-24 | 1.84E-22 | 42.86547 |
| ENSG00000176182.5 | MYPOP | 0.507195 | 2.247601 | 8.483143 | 1.92E-16 | 2.73E-15 | 25.99511 |
| ENSG00000205765.7 | C5orf51 | 0.506961 | 3.173629 | 7.332811 | 7.83E-13 | 8.29E-12 | 17.80445 |
| ENSG00000171603.15 | CLSTN1 | 0.506931 | 5.370276 | 5.800226 | 1.10E-08 | 7.68E-08 | 8.458026 |
| ENSG00000211450.8 | C11orf31 | 0.506764 | 4.800202 | 6.905171 | 1.35E-11 | 1.27E-10 | 15.00951 |
| ENSG00000102871.14 | TRADD | 0.50672 | 3.775585 | 6.493559 | 1.83E-10 | 1.54E-09 | 12.45385 |
| ENSG00000204356.10 | NELFE | 0.506515 | 4.821149 | 7.609956 | 1.15E-13 | 1.31E-12 | 19.6898 |
| ENSG00000091732.14 | ZC3HC1 | 0.506505 | 2.730096 | 9.305433 | 2.89E-19 | 5.13E-18 | 32.41252 |
| ENSG00000106392.9 | C1GALT1 | 0.505805 | 2.859992 | 5.400902 | 9.77E-08 | 6.14E-07 | 6.33839 |
| ENSG00000010244.15 | ZNF207 | 0.505772 | 3.434412 | 13.5928 | 1.27E-36 | 6.96E-35 | 72.08194 |
| ENSG00000110172.10 | CHORDC1 | 0.50567 | 1.738687 | 7.994888 | 7.32E-15 | 9.18E-14 | 22.40229 |
| ENSG00000213676.9 | ATF6B | 0.505556 | 5.07319 | 7.936121 | 1.12E-14 | 1.39E-13 | 21.98123 |
| ENSG00000188517.13 | COL25A1 | 0.505403 | 0.535328 | 3.476997 | 0.000545948 | 0.001993152 | -1.90501 |
| ENSG00000183808.10 | RBM12B | 0.505248 | 1.781417 | 9.007948 | 3.19E-18 | 5.24E-17 | 30.03933 |
| ENSG00000130707.16 | ASS1 | 0.504866 | 5.255064 | 3.060577 | 0.002313716 | 0.00745554 | -3.24138 |
| ENSG00000203814.6 | HIST2H2BF | 0.504838 | 0.647818 | 5.715282 | 1.77E-08 | 1.21E-07 | 7.995836 |
| ENSG00000103591.11 | AAGAB | 0.504793 | 3.777617 | 8.469175 | 2.13E-16 | 3.02E-15 | 25.89001 |
| ENSG00000124786.9 | SLC35B3 | 0.504318 | 3.712829 | 9.019377 | 2.91E-18 | 4.80E-17 | 30.12944 |
| ENSG00000186866.15 | POFUT2 | 0.504315 | 2.858034 | 7.223419 | 1.64E-12 | 1.68E-11 | 17.07618 |
| ENSG00000187522.12 | HSPA14 | 0.504312 | 2.640029 | 7.88633 | 1.61E-14 | 1.96E-13 | 21.62643 |
| ENSG00000101557.13 | USP14 | 0.504297 | 3.815772 | 7.825771 | 2.49E-14 | 2.99E-13 | 21.19731 |
| ENSG00000178295.13 | GEN1 | 0.504178 | 1.074195 | 9.566588 | 3.36E-20 | 6.33E-19 | 34.5424 |
| ENSG00000198242.12 | RPL23A | 0.504177 | 7.141414 | 6.874794 | 1.64E-11 | 1.53E-10 | 14.81634 |
| ENSG00000197114.10 | ZGPAT | 0.504051 | 1.854774 | 6.624232 | 8.13E-11 | 7.07E-10 | 13.25065 |
| ENSG00000130193.7 | THEM6 | 0.503982 | 3.764112 | 4.827344 | 1.78E-06 | 9.53E-06 | 3.53405 |
| ENSG00000100197.19 | CYP2D6 | 0.503885 | 0.760353 | 5.53529 | 4.76E-08 | 3.09E-07 | 7.036612 |
| ENSG00000168159.10 | RNF187 | 0.503871 | 5.247434 | 8.590728 | 8.40E-17 | 1.23E-15 | 26.80904 |
| ENSG00000101945.15 | SUV39H1 | 0.503667 | 2.029498 | 7.209876 | 1.80E-12 | 1.83E-11 | 16.98664 |
| ENSG00000165506.13 | DNAAF2 | 0.503609 | 2.685888 | 7.053484 | 5.11E-12 | 4.98E-11 | 15.96293 |
| ENSG00000130255.11 | RPL36 | 0.503128 | 6.992954 | 5.216049 | 2.57E-07 | 1.53E-06 | 5.403317 |
| ENSG00000127585.10 | FBXL16 | 0.503026 | 1.554305 | 3.438512 | 0.000627903 | 0.002265013 | -2.03538 |
| ENSG00000100105.16 | PATZ1 | 0.502853 | 3.314884 | 7.144995 | 2.78E-12 | 2.78E-11 | 16.55967 |
| ENSG00000196141.11 | SPATS2L | 0.502826 | 3.674935 | 5.005491 | 7.45E-07 | 4.18E-06 | 4.374318 |
| ENSG00000163444.10 | TMEM183A | 0.502624 | 3.361654 | 7.942544 | 1.07E-14 | 1.33E-13 | 22.02713 |
| ENSG00000156504.15 | FAM122B | 0.502624 | 3.303576 | 7.173853 | 2.29E-12 | 2.31E-11 | 16.74918 |
| ENSG00000135097.5 | MSI1 | 0.502457 | 0.794621 | 3.705451 | 0.000231712 | 0.0009061 | -1.10258 |
| ENSG00000205078.5 | SYCE1L | 0.502454 | 1.166208 | 5.664797 | 2.34E-08 | 1.58E-07 | 7.724021 |
| ENSG00000162572.18 | SCNN1D | 0.502209 | 0.890003 | 5.793378 | 1.15E-08 | 7.97E-08 | 8.420541 |
| ENSG00000137207.10 | YIPF3 | 0.502109 | 5.730283 | 8.272971 | 9.38E-16 | 1.27E-14 | 24.42807 |
| ENSG00000164465.17 | DCBLD1 | 0.502045 | 2.337801 | 5.558971 | 4.18E-08 | 2.74E-07 | 7.161241 |
| ENSG00000105290.10 | APLP1 | 0.502032 | 1.548349 | 3.26577 | 0.001157612 | 0.003974109 | -2.60337 |
| ENSG00000174276.6 | ZNHIT2 | 0.501717 | 3.335239 | 6.186515 | 1.18E-09 | 9.13E-09 | 10.63569 |
| ENSG00000168993.13 | CPLX1 | 0.501627 | 1.375818 | 4.592068 | 5.41E-06 | 2.72E-05 | 2.467388 |
| ENSG00000181467.3 | RAP2B | 0.501562 | 3.619471 | 5.929964 | 5.27E-09 | 3.81E-08 | 9.175606 |
| ENSG00000196436.8 | NPIPB15 | 0.501558 | 1.102455 | 3.654035 | 0.000282131 | 0.00108779 | -1.28743 |
| ENSG00000186141.7 | POLR3C | 0.501349 | 3.616457 | 8.93015 | 5.92E-18 | 9.56E-17 | 29.4282 |
| ENSG00000011304.15 | PTBP1 | 0.501259 | 5.253033 | 9.668161 | 1.44E-20 | 2.76E-19 | 35.3823 |
| ENSG00000184956.14 | MUC6 | 0.501214 | 0.597021 | 2.459614 | 0.014206284 | 0.037866469 | -4.87844 |
| ENSG00000074266.16 | EED | 0.501024 | 1.942099 | 9.991061 | 9.30E-22 | 1.96E-20 | 38.09416 |
| ENSG00000156026.13 | MCU | 0.500985 | 3.637996 | 5.755819 | 1.41E-08 | 9.73E-08 | 8.215649 |
| ENSG00000179528.14 | LBX2 | 0.500984 | 0.639313 | 9.721332 | 9.20E-21 | 1.80E-19 | 35.8245 |
| ENSG00000134490.12 | TMEM241 | 0.500837 | 2.132663 | 7.370666 | 6.05E-13 | 6.46E-12 | 18.05858 |
| ENSG00000173209.21 | AHSA2 | 0.500822 | 2.27886 | 4.243877 | 2.57E-05 | 0.00011692 | 0.979893 |
| ENSG00000159579.12 | RSPRY1 | 0.500764 | 3.059993 | 9.790473 | 5.14E-21 | 1.03E-19 | 36.4021 |
| ENSG00000134986.12 | NREP | 0.500695 | 2.523741 | 5.150467 | 3.59E-07 | 2.10E-06 | 5.078673 |
| ENSG00000133247.12 | SUV420H2 | 0.500528 | 1.657158 | 6.439251 | 2.56E-10 | 2.13E-09 | 12.12671 |
| ENSG00000120800.4 | UTP20 | 0.500169 | 2.147581 | 6.112064 | 1.83E-09 | 1.39E-08 | 10.20638 |
| ENSG00000184489.10 | PTP4A3 | 0.500159 | 4.562614 | 3.618602 | 0.000322692 | 0.001227895 | -1.41338 |
| ENSG00000133703.10 | KRAS | 0.500105 | 3.352759 | 5.714648 | 1.78E-08 | 1.21E-07 | 7.99241 |
| ENSG00000197587.9 | DMBX1 | 0.500091 | 0.458372 | 5.629471 | 2.85E-08 | 1.90E-07 | 7.535103 |
| ENSG00000140688.15 | C16orf58 | 0.500007 | 3.892301 | 6.700343 | 5.03E-11 | 4.47E-10 | 13.72101 |
| ENSG00000266412.4 | NCOA4 | -0.5003 | 5.695181 | -7.27227 | 1.18E-12 | 1.23E-11 | 17.40026 |
| ENSG00000243543.7 | WFDC6 | -0.50052 | 0.287584 | -6.87914 | 1.60E-11 | 1.49E-10 | 14.84393 |
| ENSG00000157110.14 | RBPMS | -0.5007 | 3.277274 | -4.41544 | 1.21E-05 | 5.77E-05 | 1.699149 |
| ENSG00000173846.11 | PLK3 | -0.50075 | 2.833343 | -5.94116 | 4.94E-09 | 3.58E-08 | 9.238167 |
| ENSG00000112379.8 | KIAA1244 | -0.5009 | 2.369959 | -4.42306 | 1.17E-05 | 5.59E-05 | 1.731732 |
| ENSG00000135362.12 | PRR5L | -0.50152 | 1.645273 | -5.34738 | 1.30E-07 | 8.01E-07 | 6.064631 |
| ENSG00000141084.9 | RANBP10 | -0.50175 | 2.211392 | -7.18819 | 2.08E-12 | 2.10E-11 | 16.8436 |
| ENSG00000132002.6 | DNAJB1 | -0.50177 | 5.161567 | -5.70874 | 1.84E-08 | 1.25E-07 | 7.960508 |
| ENSG00000185640.5 | KRT79 | -0.50185 | 0.157409 | -13.6476 | 7.20E-37 | 3.99E-35 | 72.64411 |
| ENSG00000086289.10 | EPDR1 | -0.50212 | 4.089048 | -3.13304 | 0.00181949 | 0.005987062 | -3.02065 |
| ENSG00000173221.12 | GLRX | -0.50246 | 2.749053 | -4.9107 | 1.19E-06 | 6.50E-06 | 3.923748 |
| ENSG00000102230.12 | PCYT1B | -0.50265 | 0.279236 | -10.5079 | 1.02E-23 | 2.47E-22 | 42.56268 |
| ENSG00000148411.6 | NACC2 | -0.50299 | 2.525975 | -6.84314 | 2.02E-11 | 1.86E-10 | 14.6158 |
| ENSG00000115295.18 | CLIP4 | -0.50332 | 2.459666 | -6.11471 | 1.80E-09 | 1.37E-08 | 10.22155 |
| ENSG00000124570.16 | SERPINB6 | -0.50341 | 4.705931 | -6.79566 | 2.74E-11 | 2.49E-10 | 14.31651 |
| ENSG00000184828.8 | ZBTB7C | -0.50351 | 1.362072 | -4.26085 | 2.38E-05 | 0.000109156 | 1.049869 |
| ENSG00000103187.7 | COTL1 | -0.50366 | 4.529085 | -4.28495 | 2.15E-05 | 9.90E-05 | 1.149655 |
| ENSG00000147862.13 | NFIB | -0.50392 | 3.030531 | -4.95637 | 9.50E-07 | 5.26E-06 | 4.139837 |
| ENSG00000164330.15 | EBF1 | -0.50447 | 0.850539 | -9.08545 | 1.72E-18 | 2.87E-17 | 30.65212 |
| ENSG00000145687.14 | SSBP2 | -0.50456 | 1.36185 | -7.64874 | 8.76E-14 | 1.00E-12 | 19.95819 |
| ENSG00000260286.2 | C6orf229 | -0.50458 | 0.131645 | -13.1238 | 1.52E-34 | 7.32E-33 | 67.31815 |
| ENSG00000147416.9 | ATP6V1B2 | -0.50459 | 4.003671 | -7.37555 | 5.85E-13 | 6.26E-12 | 18.09144 |
| ENSG00000164691.15 | TAGAP | -0.50465 | 2.05355 | -4.86516 | 1.48E-06 | 8.00E-06 | 3.710057 |
| ENSG00000134109.9 | EDEM1 | -0.50469 | 4.24047 | -5.51334 | 5.36E-08 | 3.46E-07 | 6.921507 |
| ENSG00000166501.11 | PRKCB | -0.50473 | 1.393649 | -5.77259 | 1.29E-08 | 8.90E-08 | 8.307002 |
| ENSG00000151689.11 | INPP1 | -0.50479 | 2.517534 | -6.51841 | 1.57E-10 | 1.33E-09 | 12.6043 |
| ENSG00000089220.4 | PEBP1 | -0.50508 | 7.031715 | -6.65156 | 6.84E-11 | 6.01E-10 | 13.41899 |
| ENSG00000241978.8 | AKAP2 | -0.50518 | 0.190691 | -26.8106 | 7.34E-103 | 7.79E-100 | 224.0916 |
| ENSG00000175556.15 | LONRF3 | -0.5052 | 1.593721 | -6.57071 | 1.14E-10 | 9.74E-10 | 12.92263 |
| ENSG00000087884.13 | AAMDC | -0.50524 | 2.80588 | -7.40559 | 4.76E-13 | 5.13E-12 | 18.29401 |
| ENSG00000197353.3 | LYPD2 | -0.50539 | 0.29084 | -5.58379 | 3.66E-08 | 2.41E-07 | 7.292386 |
| ENSG00000123091.4 | RNF11 | -0.50542 | 4.957501 | -8.32468 | 6.36E-16 | 8.71E-15 | 24.8108 |
| ENSG00000110237.3 | ARHGEF17 | -0.50591 | 2.942238 | -5.87822 | 7.09E-09 | 5.05E-08 | 8.887726 |
| ENSG00000157152.15 | SYN2 | -0.50592 | 0.21306 | -12.3514 | 3.35E-31 | 1.30E-29 | 59.67055 |
| ENSG00000197724.9 | PHF2 | -0.50609 | 3.349906 | -7.79658 | 3.07E-14 | 3.65E-13 | 20.99145 |
| ENSG00000163885.10 | CCDC37 | -0.50631 | 0.459862 | -5.44981 | 7.53E-08 | 4.79E-07 | 6.590726 |
| ENSG00000197380.9 | DACT3 | -0.50637 | 1.098034 | -7.49723 | 2.53E-13 | 2.79E-12 | 18.91603 |
| ENSG00000138400.11 | MDH1B | -0.50647 | 0.596617 | -6.20806 | 1.04E-09 | 8.09E-09 | 10.76077 |
| ENSG00000130700.6 | GATA5 | -0.50705 | 0.460906 | -9.72576 | 8.87E-21 | 1.74E-19 | 35.86136 |
| ENSG00000128815.16 | WDFY4 | -0.50708 | 1.475332 | -4.86809 | 1.46E-06 | 7.90E-06 | 3.723754 |
| ENSG00000174951.9 | FUT1 | -0.50755 | 1.632666 | -6.13157 | 1.63E-09 | 1.24E-08 | 10.31842 |
| ENSG00000077984.5 | CST7 | -0.50776 | 3.395557 | -3.45942 | 0.000582061 | 0.00211326 | -1.96474 |
| ENSG00000049245.11 | VAMP3 | -0.5078 | 5.410423 | -9.3276 | 2.41E-19 | 4.30E-18 | 32.59162 |
| ENSG00000145476.14 | CYP4V2 | -0.50803 | 1.96625 | -6.66526 | 6.28E-11 | 5.53E-10 | 13.50364 |
| ENSG00000078804.11 | TP53INP2 | -0.5081 | 3.629764 | -4.64434 | 4.25E-06 | 2.16E-05 | 2.700111 |
| ENSG00000152804.9 | HHEX | -0.50814 | 2.129131 | -5.82339 | 9.67E-09 | 6.78E-08 | 8.585132 |
| ENSG00000110090.11 | CPT1A | -0.50861 | 3.486904 | -5.64886 | 2.56E-08 | 1.72E-07 | 7.638669 |
| ENSG00000213463.4 | SYNJ2BP | -0.50877 | 2.732976 | -8.959 | 4.71E-18 | 7.65E-17 | 29.6544 |
| ENSG00000104332.10 | SFRP1 | -0.50897 | 0.874139 | -3.54498 | 0.000425087 | 0.001584723 | -1.67134 |
| ENSG00000153147.5 | SMARCA5 | -0.50912 | 3.941353 | -9.06043 | 2.10E-18 | 3.50E-17 | 30.45382 |
| ENSG00000162706.11 | CADM3 | -0.50935 | 0.797153 | -5.52567 | 5.01E-08 | 3.25E-07 | 6.986112 |
| ENSG00000185633.9 | NDUFA4L2 | -0.50936 | 2.908443 | -3.18576 | 0.001523181 | 0.005101703 | -2.85691 |
| ENSG00000140287.9 | HDC | -0.50943 | 0.776673 | -6.42677 | 2.77E-10 | 2.28E-09 | 12.05185 |
| ENSG00000154822.14 | PLCL2 | -0.50954 | 1.446274 | -6.56507 | 1.18E-10 | 1.01E-09 | 12.88824 |
| ENSG00000167202.10 | TBC1D2B | -0.50969 | 2.740899 | -6.84648 | 1.97E-11 | 1.82E-10 | 14.63691 |
| ENSG00000064309.13 | CDON | -0.50979 | 1.008835 | -6.04213 | 2.76E-09 | 2.05E-08 | 9.807256 |
| ENSG00000113448.15 | PDE4D | -0.50998 | 2.002264 | -3.9071 | 0.000104693 | 0.000434691 | -0.35394 |
| ENSG00000136999.4 | NOV | -0.51033 | 1.580255 | -5.02225 | 6.85E-07 | 3.87E-06 | 4.454784 |
| ENSG00000023516.8 | AKAP11 | -0.51034 | 2.661009 | -7.79569 | 3.09E-14 | 3.67E-13 | 20.98516 |
| ENSG00000117228.9 | GBP1 | -0.5105 | 3.800945 | -3.2725 | 0.001130905 | 0.003889265 | -2.58177 |
| ENSG00000013583.7 | HEBP1 | -0.51058 | 3.615119 | -8.31182 | 7.01E-16 | 9.55E-15 | 24.71544 |
| ENSG00000126878.11 | AIF1L | -0.51073 | 2.451109 | -3.77915 | 0.000174035 | 0.000695438 | -0.83333 |
| ENSG00000275302.1 | CCL4 | -0.51081 | 2.53914 | -3.70638 | 0.000230889 | 0.000903056 | -1.09924 |
| ENSG00000163075.11 | CFAP221 | -0.51126 | 1.250697 | -4.51912 | 7.56E-06 | 3.72E-05 | 2.146682 |
| ENSG00000101017.12 | CD40 | -0.5124 | 3.73714 | -3.94144 | 9.11E-05 | 0.000382181 | -0.22272 |
| ENSG00000187240.12 | DYNC2H1 | -0.51247 | 0.919276 | -7.23397 | 1.53E-12 | 1.57E-11 | 17.14599 |
| ENSG00000099622.12 | CIRBP | -0.51299 | 4.979724 | -5.69069 | 2.03E-08 | 1.38E-07 | 7.863169 |
| ENSG00000079950.12 | STX7 | -0.51313 | 2.307471 | -10.2354 | 1.12E-22 | 2.54E-21 | 40.18729 |
| ENSG00000198003.10 | CCDC151 | -0.51359 | 0.745438 | -5.37831 | 1.10E-07 | 6.86E-07 | 6.222541 |
| ENSG00000163701.17 | IL17RE | -0.51363 | 2.476001 | -4.36724 | 1.50E-05 | 7.06E-05 | 1.494396 |
| ENSG00000149201.8 | CCDC81 | -0.51458 | 0.331651 | -9.43817 | 9.74E-20 | 1.78E-18 | 33.48971 |
| ENSG00000088766.10 | CRLS1 | -0.51472 | 3.86158 | -5.64551 | 2.61E-08 | 1.75E-07 | 7.620757 |
| ENSG00000113163.14 | COL4A3BP | -0.51486 | 2.721322 | -7.86672 | 1.85E-14 | 2.25E-13 | 21.4872 |
| ENSG00000134321.10 | RSAD2 | -0.51531 | 2.283147 | -4.10717 | 4.60E-05 | 0.000201875 | 0.425942 |
| ENSG00000152213.3 | ARL11 | -0.51586 | 1.241216 | -6.78202 | 2.99E-11 | 2.72E-10 | 14.23086 |
| ENSG00000154813.8 | DPH3 | -0.51604 | 3.113963 | -8.86334 | 1.00E-17 | 1.59E-16 | 28.90654 |
| ENSG00000184613.9 | NELL2 | -0.51674 | 1.011703 | -5.27576 | 1.88E-07 | 1.14E-06 | 5.702152 |
| ENSG00000140105.16 | WARS | -0.51684 | 5.408515 | -3.8303 | 0.000142282 | 0.000577678 | -0.64352 |
| ENSG00000162004.15 | CCDC78 | -0.51717 | 1.152425 | -4.08953 | 4.95E-05 | 0.000216378 | 0.355685 |
| ENSG00000161714.10 | PLCD3 | -0.5173 | 1.737083 | -4.72658 | 2.88E-06 | 1.50E-05 | 3.071178 |
| ENSG00000147576.14 | ADHFE1 | -0.51731 | 1.083346 | -5.90426 | 6.11E-09 | 4.39E-08 | 9.032299 |
| ENSG00000144476.5 | ACKR3 | -0.51856 | 3.218344 | -3.27202 | 0.001132805 | 0.003895357 | -2.58332 |
| ENSG00000213625.7 | LEPROT | -0.51872 | 4.741228 | -8.99964 | 3.41E-18 | 5.59E-17 | 29.9739 |
| ENSG00000112658.7 | SRF | -0.51912 | 3.959266 | -8.32347 | 6.42E-16 | 8.78E-15 | 24.80181 |
| ENSG00000065882.14 | TBC1D1 | -0.51936 | 2.457386 | -6.03417 | 2.89E-09 | 2.15E-08 | 9.762126 |
| ENSG00000170525.17 | PFKFB3 | -0.51943 | 4.560098 | -4.08693 | 5.00E-05 | 0.000218541 | 0.345352 |
| ENSG00000180353.9 | HCLS1 | -0.51947 | 3.721474 | -4.33487 | 1.73E-05 | 8.06E-05 | 1.358065 |
| ENSG00000185811.15 | IKZF1 | -0.5195 | 1.740653 | -5.40449 | 9.59E-08 | 6.03E-07 | 6.356834 |
| ENSG00000137145.19 | DENND4C | -0.52003 | 2.632187 | -6.93487 | 1.11E-11 | 1.05E-10 | 15.19909 |
| ENSG00000101098.11 | RIMS4 | -0.52056 | 0.28846 | -8.41178 | 3.30E-16 | 4.61E-15 | 25.45961 |
| ENSG00000155368.15 | DBI | -0.52059 | 5.442068 | -6.83178 | 2.17E-11 | 1.99E-10 | 14.54403 |
| ENSG00000204020.5 | LIPN | -0.52077 | 0.186862 | -16.6503 | 5.77E-51 | 6.46E-49 | 104.9558 |
| ENSG00000147119.3 | CHST7 | -0.52094 | 1.459354 | -8.30714 | 7.26E-16 | 9.87E-15 | 24.68076 |
| ENSG00000139793.17 | MBNL2 | -0.52108 | 3.888007 | -6.96268 | 9.28E-12 | 8.83E-11 | 15.3772 |
| ENSG00000119938.8 | PPP1R3C | -0.52113 | 2.113306 | -4.02994 | 6.34E-05 | 0.000272714 | 0.120514 |
| ENSG00000155970.10 | MICU3 | -0.52274 | 0.756918 | -10.8965 | 3.12E-25 | 8.42E-24 | 46.02255 |
| ENSG00000152518.6 | ZFP36L2 | -0.52278 | 5.536305 | -5.39096 | 1.03E-07 | 6.45E-07 | 6.287371 |
| ENSG00000198680.4 | TUSC1 | -0.52305 | 2.575723 | -5.9685 | 4.22E-09 | 3.08E-08 | 9.391432 |
| ENSG00000125966.9 | MMP24 | -0.52362 | 2.20692 | -3.86838 | 0.000122281 | 0.000502168 | -0.50059 |
| ENSG00000152104.10 | PTPN14 | -0.52411 | 1.651596 | -6.99746 | 7.39E-12 | 7.10E-11 | 15.60076 |
| ENSG00000205086.6 | C2orf91 | -0.52447 | 0.16164 | -17.812 | 1.09E-56 | 1.57E-54 | 118.0825 |
| ENSG00000007944.13 | MYLIP | -0.52464 | 3.758396 | -5.66731 | 2.31E-08 | 1.56E-07 | 7.737515 |
| ENSG00000130592.12 | LSP1 | -0.52475 | 3.802906 | -4.137 | 4.05E-05 | 0.000179632 | 0.545344 |
| ENSG00000166681.12 | NGFRAP1 | -0.52478 | 5.87391 | -4.01676 | 6.70E-05 | 0.00028674 | 0.068953 |
| ENSG00000123700.4 | KCNJ2 | -0.52498 | 1.168254 | -5.87016 | 7.42E-09 | 5.28E-08 | 8.843065 |
| ENSG00000115221.9 | ITGB6 | -0.52523 | 4.483644 | -3.08894 | 0.002107158 | 0.006847953 | -3.15557 |
| ENSG00000134996.11 | OSTF1 | -0.52552 | 5.150394 | -9.1381 | 1.12E-18 | 1.91E-17 | 31.0706 |
| ENSG00000164116.15 | GUCY1A3 | -0.52577 | 2.486373 | -4.95657 | 9.49E-07 | 5.26E-06 | 4.140766 |
| ENSG00000162437.13 | RAVER2 | -0.52591 | 1.872711 | -6.87912 | 1.60E-11 | 1.49E-10 | 14.84383 |
| ENSG00000120907.16 | ADRA1A | -0.52596 | 0.118368 | -21.2268 | 5.13E-74 | 1.44E-71 | 157.8578 |
| ENSG00000036672.14 | USP2 | -0.526 | 0.900327 | -6.44437 | 2.48E-10 | 2.06E-09 | 12.15746 |
| ENSG00000189320.7 | FAM180A | -0.52688 | 0.556416 | -8.67109 | 4.51E-17 | 6.74E-16 | 27.42216 |
| ENSG00000258289.6 | CHURC1 | -0.52715 | 3.209804 | -7.64525 | 8.98E-14 | 1.03E-12 | 19.93402 |
| ENSG00000153993.12 | SEMA3D | -0.52725 | 0.54447 | -8.54662 | 1.18E-16 | 1.71E-15 | 26.47441 |
| ENSG00000167693.15 | NXN | -0.5273 | 3.434666 | -5.06161 | 5.63E-07 | 3.21E-06 | 4.644778 |
| ENSG00000196169.13 | KIF19 | -0.52735 | 0.610611 | -5.23143 | 2.37E-07 | 1.42E-06 | 5.479989 |
| ENSG00000187889.11 | C1orf168 | -0.52741 | 0.624895 | -6.10528 | 1.91E-09 | 1.44E-08 | 10.16747 |
| ENSG00000166398.11 | KIAA0355 | -0.52756 | 2.488375 | -8.13078 | 2.70E-15 | 3.51E-14 | 23.38542 |
| ENSG00000183273.5 | CCDC60 | -0.52782 | 0.380614 | -6.91446 | 1.27E-11 | 1.19E-10 | 15.0687 |
| ENSG00000177663.12 | IL17RA | -0.52786 | 2.454812 | -8.37048 | 4.50E-16 | 6.23E-15 | 25.15131 |
| ENSG00000138738.9 | PRDM5 | -0.52803 | 0.667121 | -13.74 | 2.77E-37 | 1.57E-35 | 73.59431 |
| ENSG00000126950.7 | TMEM35 | -0.52805 | 0.692364 | -5.38671 | 1.05E-07 | 6.58E-07 | 6.265575 |
| ENSG00000178425.12 | NT5DC1 | -0.52833 | 2.556633 | -9.38123 | 1.56E-19 | 2.80E-18 | 33.02628 |
| ENSG00000114450.8 | GNB4 | -0.52907 | 2.478003 | -5.7219 | 1.71E-08 | 1.17E-07 | 8.031618 |
| ENSG00000135547.7 | HEY2 | -0.52929 | 0.86287 | -7.81824 | 2.63E-14 | 3.14E-13 | 21.14413 |
| ENSG00000101384.10 | JAG1 | -0.52933 | 2.691884 | -4.5465 | 6.67E-06 | 3.31E-05 | 2.266497 |
| ENSG00000163531.14 | NFASC | -0.52949 | 0.526813 | -9.54477 | 4.03E-20 | 7.55E-19 | 34.36282 |
| ENSG00000146151.11 | HMGCLL1 | -0.52995 | 0.341621 | -14.4064 | 2.57E-40 | 1.69E-38 | 80.54215 |
| ENSG00000142449.11 | FBN3 | -0.53013 | 0.453594 | -6.18868 | 1.16E-09 | 9.02E-09 | 10.64826 |
| ENSG00000187091.12 | PLCD1 | -0.53052 | 2.309238 | -6.28631 | 6.49E-10 | 5.16E-09 | 11.21824 |
| ENSG00000154114.11 | TBCEL | -0.53053 | 2.121565 | -8.64623 | 5.47E-17 | 8.12E-16 | 27.23199 |
| ENSG00000152217.15 | SETBP1 | -0.53141 | 1.113715 | -6.55977 | 1.22E-10 | 1.04E-09 | 12.85587 |
| ENSG00000079739.14 | PGM1 | -0.5321 | 4.521671 | -6.61153 | 8.80E-11 | 7.63E-10 | 13.17258 |
| ENSG00000088256.7 | GNA11 | -0.53241 | 3.674701 | -7.99817 | 7.15E-15 | 8.97E-14 | 22.42589 |
| ENSG00000112981.4 | NME5 | -0.53267 | 1.176076 | -5.1949 | 2.86E-07 | 1.69E-06 | 5.298224 |
| ENSG00000110665.10 | C11orf21 | -0.53267 | 0.605078 | -9.07572 | 1.86E-18 | 3.10E-17 | 30.57492 |
| ENSG00000149575.5 | SCN2B | -0.53294 | 0.331457 | -12.8235 | 3.13E-33 | 1.39E-31 | 64.31496 |
| ENSG00000109072.12 | VTN | -0.53308 | 0.636987 | -4.40521 | 1.26E-05 | 6.03E-05 | 1.655533 |
| ENSG00000116885.17 | OSCP1 | -0.53329 | 2.152886 | -6.33928 | 4.72E-10 | 3.80E-09 | 11.53074 |
| ENSG00000165943.4 | MOAP1 | -0.53364 | 3.855843 | -6.92923 | 1.15E-11 | 1.09E-10 | 15.16304 |
| ENSG00000141052.16 | MYOCD | -0.53392 | 0.320771 | -12.7859 | 4.55E-33 | 2.01E-31 | 63.94122 |
| ENSG00000173918.13 | C1QTNF1 | -0.53443 | 2.404577 | -4.72848 | 2.86E-06 | 1.49E-05 | 3.079823 |
| ENSG00000175806.13 | MSRA | -0.53451 | 1.904369 | -7.86823 | 1.83E-14 | 2.23E-13 | 21.4979 |
| ENSG00000006831.9 | ADIPOR2 | -0.53459 | 3.894112 | -7.44179 | 3.71E-13 | 4.04E-12 | 18.53896 |
| ENSG00000171943.10 | SRGAP2C | -0.53558 | 0.860343 | -13.56 | 1.78E-36 | 9.64E-35 | 71.7459 |
| ENSG00000111796.3 | KLRB1 | -0.5358 | 2.035739 | -4.65737 | 3.99E-06 | 2.04E-05 | 2.758503 |
| ENSG00000127083.7 | OMD | -0.53645 | 1.17324 | -4.48481 | 8.84E-06 | 4.31E-05 | 1.997528 |
| ENSG00000004866.17 | ST7 | -0.53688 | 2.848796 | -8.68352 | 4.10E-17 | 6.15E-16 | 27.51741 |
| ENSG00000155846.15 | PPARGC1B | -0.53724 | 0.547167 | -12.2632 | 7.92E-31 | 3.00E-29 | 58.81479 |
| ENSG00000160808.8 | MYL3 | -0.53743 | 0.417527 | -13.9682 | 2.58E-38 | 1.55E-36 | 75.95598 |
| ENSG00000136161.11 | RCBTB2 | -0.53838 | 2.146359 | -7.60643 | 1.18E-13 | 1.34E-12 | 19.66545 |
| ENSG00000164342.11 | TLR3 | -0.53877 | 1.735457 | -5.72371 | 1.69E-08 | 1.16E-07 | 8.041422 |
| ENSG00000111911.6 | HINT3 | -0.53879 | 3.838308 | -7.52275 | 2.12E-13 | 2.36E-12 | 19.09039 |
| ENSG00000198932.11 | GPRASP1 | -0.53902 | 0.97413 | -7.9987 | 7.12E-15 | 8.94E-14 | 22.42966 |
| ENSG00000136783.9 | NIPSNAP3A | -0.53929 | 3.135012 | -7.34121 | 7.39E-13 | 7.85E-12 | 17.86077 |
| ENSG00000059377.14 | TBXAS1 | -0.53998 | 2.469632 | -5.59863 | 3.37E-08 | 2.23E-07 | 7.371047 |
| ENSG00000139505.11 | MTMR6 | -0.54021 | 2.973923 | -8.06487 | 4.39E-15 | 5.61E-14 | 22.90693 |
| ENSG00000173013.5 | CCDC96 | -0.54029 | 1.397997 | -5.62537 | 2.91E-08 | 1.94E-07 | 7.513225 |
| ENSG00000137098.12 | SPAG8 | -0.54083 | 0.726593 | -6.30584 | 5.77E-10 | 4.61E-09 | 11.33319 |
| ENSG00000105357.14 | MYH14 | -0.54231 | 4.378677 | -4.11315 | 4.48E-05 | 0.000197251 | 0.449808 |
| ENSG00000010810.16 | FYN | -0.54268 | 2.76267 | -5.64543 | 2.61E-08 | 1.75E-07 | 7.620347 |
| ENSG00000140465.12 | CYP1A1 | -0.54324 | 0.272116 | -4.56694 | 6.08E-06 | 3.03E-05 | 2.356374 |
| ENSG00000104974.9 | LILRA1 | -0.54329 | 0.472362 | -14.7188 | 9.23E-42 | 6.70E-40 | 83.85177 |
| ENSG00000186635.13 | ARAP1 | -0.54351 | 4.056824 | -7.19928 | 1.93E-12 | 1.96E-11 | 16.91667 |
| ENSG00000119396.9 | RAB14 | -0.54394 | 4.996008 | -10.1167 | 3.15E-22 | 6.88E-21 | 39.16631 |
| ENSG00000154262.11 | ABCA6 | -0.54397 | 0.545471 | -12.0825 | 4.58E-30 | 1.66E-28 | 57.07047 |
| ENSG00000198574.5 | SH2D1B | -0.54421 | 0.37002 | -11.8019 | 6.79E-29 | 2.30E-27 | 54.39339 |
| ENSG00000185477.4 | GPRIN3 | -0.54423 | 1.495927 | -6.33632 | 4.80E-10 | 3.87E-09 | 11.51319 |
| ENSG00000187323.10 | DCC | -0.54514 | 0.198289 | -21.1563 | 1.18E-73 | 3.21E-71 | 157.0254 |
| ENSG00000100170.8 | SLC5A1 | -0.54542 | 1.041255 | -3.53019 | 0.000449022 | 0.001665941 | -1.72254 |
| ENSG00000113580.13 | NR3C1 | -0.54564 | 3.40577 | -6.82608 | 2.25E-11 | 2.07E-10 | 14.5081 |
| ENSG00000107679.13 | PLEKHA1 | -0.54616 | 1.800467 | -8.93653 | 5.63E-18 | 9.11E-17 | 29.47816 |
| ENSG00000093183.12 | SEC22C | -0.5463 | 2.605074 | -12.9193 | 1.20E-33 | 5.45E-32 | 65.26834 |
| ENSG00000164096.12 | C4orf3 | -0.54705 | 5.322084 | -9.7548 | 6.94E-21 | 1.37E-19 | 36.10375 |
| ENSG00000167741.9 | GGT6 | -0.54711 | 1.902006 | -2.9922 | 0.002890384 | 0.009125123 | -3.44511 |
| ENSG00000184454.6 | NCMAP | -0.54731 | 1.941361 | -4.16336 | 3.62E-05 | 0.00016174 | 0.651557 |
| ENSG00000113851.12 | CRBN | -0.54735 | 2.351748 | -9.95824 | 1.23E-21 | 2.58E-20 | 37.81564 |
| ENSG00000028137.15 | TNFRSF1B | -0.54785 | 4.133965 | -5.22711 | 2.42E-07 | 1.45E-06 | 5.458446 |
| ENSG00000108861.7 | DUSP3 | -0.54789 | 4.340734 | -9.05018 | 2.28E-18 | 3.78E-17 | 30.37277 |
| ENSG00000110906.11 | KCTD10 | -0.54798 | 3.129727 | -11.7176 | 1.51E-28 | 5.03E-27 | 53.59708 |
| ENSG00000116729.12 | WLS | -0.54812 | 4.047764 | -3.69907 | 0.000237476 | 0.000927079 | -1.12566 |
| ENSG00000162591.14 | MEGF6 | -0.54823 | 2.680063 | -4.14643 | 3.89E-05 | 0.00017298 | 0.583293 |
| ENSG00000147852.14 | VLDLR | -0.5483 | 1.586552 | -6.57513 | 1.11E-10 | 9.49E-10 | 12.94961 |
| ENSG00000149596.6 | JPH2 | -0.5486 | 0.547644 | -10.3823 | 3.10E-23 | 7.23E-22 | 41.4626 |
| ENSG00000146192.13 | FGD2 | -0.54867 | 1.329236 | -6.88647 | 1.52E-11 | 1.42E-10 | 14.8905 |
| ENSG00000132846.5 | ZBED3 | -0.54875 | 1.82879 | -6.65094 | 6.87E-11 | 6.03E-10 | 13.41517 |
| ENSG00000197168.10 | NEK5 | -0.54921 | 0.49436 | -7.77758 | 3.51E-14 | 4.16E-13 | 20.85773 |
| ENSG00000154864.10 | PIEZO2 | -0.54931 | 0.851621 | -7.88465 | 1.63E-14 | 1.99E-13 | 21.61452 |
| ENSG00000180739.13 | S1PR5 | -0.54941 | 0.467109 | -9.18916 | 7.44E-19 | 1.28E-17 | 31.47812 |
| ENSG00000111269.2 | CREBL2 | -0.54952 | 4.260511 | -8.10024 | 3.38E-15 | 4.37E-14 | 23.16333 |
| ENSG00000122641.9 | INHBA | -0.54953 | 2.628722 | -3.76653 | 0.000182837 | 0.000728255 | -0.87979 |
| ENSG00000047849.20 | MAP4 | -0.5499 | 4.092007 | -8.2182 | 1.41E-15 | 1.88E-14 | 24.02473 |
| ENSG00000176029.12 | C11orf16 | -0.54991 | 0.765576 | -4.72694 | 2.88E-06 | 1.50E-05 | 3.07282 |
| ENSG00000122786.18 | CALD1 | -0.54996 | 4.167505 | -4.82122 | 1.84E-06 | 9.80E-06 | 3.505652 |
| ENSG00000102218.5 | RP2 | -0.55028 | 2.842642 | -8.13205 | 2.67E-15 | 3.48E-14 | 23.39462 |
| ENSG00000173868.10 | PHOSPHO1 | -0.55053 | 0.440928 | -12.6186 | 2.40E-32 | 1.01E-30 | 62.28717 |
| ENSG00000109182.10 | CWH43 | -0.55083 | 0.271107 | -7.83419 | 2.34E-14 | 2.82E-13 | 21.25683 |
| ENSG00000172602.8 | RND1 | -0.55095 | 3.094079 | -2.74343 | 0.006272667 | 0.018312967 | -4.14856 |
| ENSG00000019485.11 | PRDM11 | -0.55151 | 0.837161 | -13.0878 | 2.19E-34 | 1.05E-32 | 66.95587 |
| ENSG00000198947.13 | DMD | -0.55188 | 0.910768 | -8.03555 | 5.44E-15 | 6.90E-14 | 22.69507 |
| ENSG00000109943.7 | CRTAM | -0.55202 | 0.869658 | -7.64261 | 9.15E-14 | 1.05E-12 | 19.9157 |
| ENSG00000154678.15 | PDE1C | -0.55212 | 0.407558 | -9.28734 | 3.35E-19 | 5.92E-18 | 32.26656 |
| ENSG00000114115.8 | RBP1 | -0.55213 | 2.068728 | -4.51497 | 7.71E-06 | 3.79E-05 | 2.128594 |
| ENSG00000112319.16 | EYA4 | -0.55217 | 0.385448 | -7.9886 | 7.66E-15 | 9.59E-14 | 22.35712 |
| ENSG00000177666.14 | PNPLA2 | -0.55229 | 4.427816 | -6.02903 | 2.98E-09 | 2.21E-08 | 9.73295 |
| ENSG00000107104.17 | KANK1 | -0.55245 | 1.965615 | -7.38203 | 5.59E-13 | 6.00E-12 | 18.13506 |
| ENSG00000240891.5 | PLCXD2 | -0.55268 | 0.370978 | -15.1968 | 5.36E-44 | 4.33E-42 | 88.9773 |
| ENSG00000243710.6 | CFAP57 | -0.55281 | 0.759042 | -5.48688 | 6.18E-08 | 3.96E-07 | 6.783302 |
| ENSG00000175395.14 | ZNF25 | -0.55285 | 1.883054 | -10.2332 | 1.15E-22 | 2.59E-21 | 40.16882 |
| ENSG00000141574.6 | SECTM1 | -0.55288 | 3.608615 | -3.97995 | 7.79E-05 | 0.000330452 | -0.07423 |
| ENSG00000134532.14 | SOX5 | -0.5531 | 0.370094 | -15.2711 | 2.39E-44 | 1.96E-42 | 89.77973 |
| ENSG00000185442.11 | FAM174B | -0.55322 | 3.24024 | -4.69724 | 3.31E-06 | 1.71E-05 | 2.938127 |
| ENSG00000153707.14 | PTPRD | -0.55339 | 0.714785 | -8.91865 | 6.49E-18 | 1.04E-16 | 29.33822 |
| ENSG00000182809.9 | CRIP2 | -0.55357 | 4.653632 | -3.89623 | 0.000109372 | 0.000452752 | -0.39525 |
| ENSG00000106852.14 | LHX6 | -0.55411 | 0.665814 | -9.75294 | 7.05E-21 | 1.40E-19 | 36.08818 |
| ENSG00000026103.18 | FAS | -0.5544 | 2.842657 | -4.70718 | 3.16E-06 | 1.64E-05 | 2.983128 |
| ENSG00000103381.10 | CPPED1 | -0.55491 | 2.241609 | -6.77794 | 3.07E-11 | 2.78E-10 | 14.20527 |
| ENSG00000125347.12 | IRF1 | -0.55503 | 3.575106 | -4.9755 | 8.64E-07 | 4.82E-06 | 4.230896 |
| ENSG00000116774.10 | OLFML3 | -0.55513 | 4.027327 | -3.56714 | 0.000391438 | 0.001469239 | -1.59421 |
| ENSG00000154310.15 | TNIK | -0.55541 | 1.841531 | -4.68153 | 3.57E-06 | 1.83E-05 | 2.867153 |
| ENSG00000136830.10 | FAM129B | -0.55549 | 6.339807 | -6.3981 | 3.30E-10 | 2.70E-09 | 11.88044 |
| ENSG00000103313.10 | MEFV | -0.55641 | 0.414608 | -13.9077 | 4.84E-38 | 2.86E-36 | 75.32832 |
| ENSG00000138172.9 | CALHM2 | -0.55749 | 2.599106 | -6.71128 | 4.69E-11 | 4.19E-10 | 13.78897 |
| ENSG00000187231.12 | SESTD1 | -0.55807 | 2.489325 | -6.78131 | 3.00E-11 | 2.73E-10 | 14.22641 |
| ENSG00000086544.2 | ITPKC | -0.55832 | 4.317989 | -5.23949 | 2.27E-07 | 1.37E-06 | 5.520279 |
| ENSG00000205269.5 | TMEM170B | -0.55862 | 1.243859 | -8.79217 | 1.76E-17 | 2.72E-16 | 28.35419 |
| ENSG00000149970.13 | CNKSR2 | -0.55874 | 0.329782 | -14.456 | 1.52E-40 | 1.01E-38 | 81.06587 |
| ENSG00000221968.7 | FADS3 | -0.55887 | 2.237586 | -6.5595 | 1.22E-10 | 1.04E-09 | 12.8542 |
| ENSG00000091262.13 | ABCC6 | -0.55892 | 1.803015 | -5.20705 | 2.69E-07 | 1.60E-06 | 5.35857 |
| ENSG00000009413.14 | REV3L | -0.55917 | 1.601124 | -8.51617 | 1.49E-16 | 2.14E-15 | 26.24416 |
| ENSG00000148803.10 | FUOM | -0.5592 | 2.74843 | -4.77648 | 2.27E-06 | 1.20E-05 | 3.299261 |
| ENSG00000205363.5 | C15orf59 | -0.55936 | 0.435936 | -9.69768 | 1.12E-20 | 2.18E-19 | 35.62761 |
| ENSG00000113966.8 | ARL6 | -0.55964 | 1.539658 | -9.84127 | 3.34E-21 | 6.79E-20 | 36.82834 |
| ENSG00000135048.12 | TMEM2 | -0.55968 | 3.644863 | -6.14313 | 1.52E-09 | 1.16E-08 | 10.38496 |
| ENSG00000111912.17 | NCOA7 | -0.56007 | 4.144612 | -4.07952 | 5.16E-05 | 0.000225109 | 0.31595 |
| ENSG00000214013.8 | GANC | -0.56014 | 1.668787 | -8.51371 | 1.52E-16 | 2.18E-15 | 26.22554 |
| ENSG00000066933.14 | MYO9A | -0.56038 | 1.546479 | -9.94615 | 1.37E-21 | 2.85E-20 | 37.71323 |
| ENSG00000043462.10 | LCP2 | -0.56105 | 2.653352 | -5.40804 | 9.41E-08 | 5.92E-07 | 6.375099 |
| ENSG00000143127.11 | ITGA10 | -0.56135 | 0.778567 | -7.45841 | 3.31E-13 | 3.62E-12 | 18.65174 |
| ENSG00000110422.10 | HIPK3 | -0.56136 | 3.815513 | -8.13838 | 2.55E-15 | 3.33E-14 | 23.44074 |
| ENSG00000164463.11 | CREBRF | -0.56208 | 1.745907 | -9.52242 | 4.85E-20 | 9.05E-19 | 34.17916 |
| ENSG00000087274.15 | ADD1 | -0.56215 | 4.123548 | -10.7316 | 1.39E-24 | 3.56E-23 | 44.54428 |
| ENSG00000159899.13 | NPR2 | -0.56242 | 1.696593 | -8.09674 | 3.47E-15 | 4.48E-14 | 23.13788 |
| ENSG00000113396.11 | SLC27A6 | -0.56299 | 0.181175 | -12.5186 | 6.46E-32 | 2.63E-30 | 61.30432 |
| ENSG00000198553.7 | KCNRG | -0.56301 | 0.495607 | -8.36526 | 4.69E-16 | 6.47E-15 | 25.1124 |
| ENSG00000174080.9 | CTSF | -0.56326 | 4.065246 | -4.83921 | 1.68E-06 | 9.02E-06 | 3.589152 |
| ENSG00000198719.8 | DLL1 | -0.56332 | 1.590644 | -4.93505 | 1.06E-06 | 5.82E-06 | 4.038704 |
| ENSG00000186462.8 | NAP1L2 | -0.56356 | 0.81263 | -7.13583 | 2.96E-12 | 2.94E-11 | 16.4996 |
| ENSG00000105492.14 | SIGLEC6 | -0.56359 | 0.497097 | -9.24824 | 4.61E-19 | 8.05E-18 | 31.95182 |
| ENSG00000187244.9 | BCAM | -0.56419 | 5.74274 | -3.64087 | 0.000296612 | 0.001138973 | -1.33437 |
| ENSG00000128294.14 | TPST2 | -0.56423 | 2.751967 | -9.15426 | 9.87E-19 | 1.68E-17 | 31.19942 |
| ENSG00000184584.11 | TMEM173 | -0.56524 | 4.798293 | -4.83971 | 1.68E-06 | 9.00E-06 | 3.591456 |
| ENSG00000001630.14 | CYP51A1 | -0.56527 | 1.345865 | -8.75494 | 2.35E-17 | 3.60E-16 | 28.06654 |
| ENSG00000184497.11 | TMEM255B | -0.56607 | 1.003631 | -9.78362 | 5.44E-21 | 1.09E-19 | 36.34474 |
| ENSG00000120910.13 | PPP3CC | -0.56681 | 2.554988 | -9.86961 | 2.62E-21 | 5.37E-20 | 37.06679 |
| ENSG00000182534.12 | MXRA7 | -0.56686 | 3.046509 | -5.39058 | 1.03E-07 | 6.46E-07 | 6.285375 |
| ENSG00000138311.14 | ZNF365 | -0.56719 | 0.344429 | -14.4937 | 1.02E-40 | 6.89E-39 | 81.46412 |
| ENSG00000255302.3 | EID1 | -0.56759 | 5.826695 | -9.11802 | 1.32E-18 | 2.23E-17 | 30.91079 |
| ENSG00000169710.6 | FASN | -0.56766 | 5.074238 | -3.68309 | 0.0002525 | 0.000981048 | -1.18328 |
| ENSG00000128383.11 | APOBEC3A | -0.56838 | 0.753771 | -6.85548 | 1.86E-11 | 1.72E-10 | 14.69391 |
| ENSG00000100605.15 | ITPK1 | -0.56884 | 3.384667 | -7.88573 | 1.62E-14 | 1.97E-13 | 21.62214 |
| ENSG00000173546.7 | CSPG4 | -0.56895 | 1.768253 | -5.74622 | 1.49E-08 | 1.02E-07 | 8.163475 |
| ENSG00000205084.9 | TMEM231 | -0.56916 | 1.519461 | -5.90562 | 6.06E-09 | 4.35E-08 | 9.03989 |
| ENSG00000185630.17 | PBX1 | -0.56969 | 1.760046 | -6.03431 | 2.89E-09 | 2.14E-08 | 9.76293 |
| ENSG00000198961.8 | PJA2 | -0.57003 | 4.555918 | -8.16923 | 2.03E-15 | 2.67E-14 | 23.66598 |
| ENSG00000104903.4 | LYL1 | -0.57019 | 1.549896 | -7.79417 | 3.12E-14 | 3.71E-13 | 20.97445 |
| ENSG00000129951.17 | LPPR3 | -0.57023 | 0.604024 | -5.80404 | 1.08E-08 | 7.52E-08 | 8.47892 |
| ENSG00000114626.16 | ABTB1 | -0.57024 | 2.638224 | -7.53988 | 1.88E-13 | 2.10E-12 | 19.20769 |
| ENSG00000115963.12 | RND3 | -0.57056 | 3.19717 | -4.12058 | 4.34E-05 | 0.000191666 | 0.479511 |
| ENSG00000107165.11 | TYRP1 | -0.57066 | 0.682405 | -6.58776 | 1.02E-10 | 8.79E-10 | 13.0269 |
| ENSG00000130962.16 | PRRG1 | -0.571 | 1.189697 | -8.31823 | 6.68E-16 | 9.11E-15 | 24.7629 |
| ENSG00000152284.4 | TCF7L1 | -0.57116 | 2.633701 | -4.24217 | 2.59E-05 | 0.000117742 | 0.972858 |
| ENSG00000120899.16 | PTK2B | -0.57141 | 2.959207 | -6.33437 | 4.86E-10 | 3.91E-09 | 11.50165 |
| ENSG00000064225.11 | ST3GAL6 | -0.57198 | 1.314653 | -7.65401 | 8.44E-14 | 9.67E-13 | 19.99473 |
| ENSG00000168461.11 | RAB31 | -0.57209 | 4.624866 | -5.16334 | 3.36E-07 | 1.98E-06 | 5.14208 |
| ENSG00000163879.10 | DNALI1 | -0.57351 | 2.670036 | -3.86869 | 0.000122133 | 0.000501696 | -0.49945 |
| ENSG00000118564.13 | FBXL5 | -0.57355 | 4.519849 | -9.16877 | 8.78E-19 | 1.50E-17 | 31.31521 |
| ENSG00000095917.12 | TPSD1 | -0.57412 | 1.111914 | -4.045 | 5.96E-05 | 0.000257428 | 0.179652 |
| ENSG00000175463.10 | TBC1D10C | -0.57483 | 1.785581 | -5.42182 | 8.74E-08 | 5.52E-07 | 6.44604 |
| ENSG00000196405.11 | EVL | -0.57509 | 2.983839 | -6.41598 | 2.96E-10 | 2.43E-09 | 11.98724 |
| ENSG00000103044.9 | HAS3 | -0.57517 | 2.564949 | -2.98382 | 0.002969384 | 0.009345263 | -3.46975 |
| ENSG00000141905.16 | NFIC | -0.57592 | 3.941912 | -6.54082 | 1.37E-10 | 1.17E-09 | 12.74046 |
| ENSG00000183833.15 | MAATS1 | -0.57594 | 0.655371 | -6.65941 | 6.51E-11 | 5.73E-10 | 13.46746 |
| ENSG00000146278.10 | PNRC1 | -0.57605 | 5.057778 | -8.62326 | 6.53E-17 | 9.64E-16 | 27.05672 |
| ENSG00000181804.13 | SLC9A9 | -0.57615 | 1.593013 | -7.20536 | 1.86E-12 | 1.88E-11 | 16.95684 |
| ENSG00000114861.17 | FOXP1 | -0.57672 | 2.630323 | -8.41769 | 3.15E-16 | 4.42E-15 | 25.50384 |
| ENSG00000111913.14 | FAM65B | -0.57674 | 1.55064 | -5.55518 | 4.27E-08 | 2.79E-07 | 7.141246 |
| ENSG00000036473.6 | OTC | -0.57763 | 0.128872 | -24.4421 | 1.23E-90 | 7.27E-88 | 196.0238 |
| ENSG00000067141.15 | NEO1 | -0.57781 | 2.93166 | -5.12121 | 4.17E-07 | 2.42E-06 | 4.93503 |
| ENSG00000107281.8 | NPDC1 | -0.57829 | 3.777073 | -4.09867 | 4.76E-05 | 0.000208878 | 0.392052 |
| ENSG00000180611.6 | MB21D2 | -0.57911 | 1.551322 | -6.62604 | 8.03E-11 | 7.00E-10 | 13.26179 |
| ENSG00000172476.3 | RAB40A | -0.57959 | 0.408671 | -17.5734 | 1.67E-55 | 2.25E-53 | 115.3653 |
| ENSG00000164576.10 | SAP30L | -0.5803 | 2.501762 | -10.046 | 5.80E-22 | 1.24E-20 | 38.5618 |
| ENSG00000213085.8 | CFAP45 | -0.58056 | 1.563099 | -3.8994 | 0.00010799 | 0.000447397 | -0.38323 |
| ENSG00000100365.13 | NCF4 | -0.58069 | 3.432286 | -4.85865 | 1.53E-06 | 8.25E-06 | 3.679694 |
| ENSG00000020181.16 | GPR124 | -0.58069 | 2.272241 | -5.95639 | 4.53E-09 | 3.30E-08 | 9.323495 |
| ENSG00000025039.13 | RRAGD | -0.58119 | 2.571064 | -5.13211 | 3.94E-07 | 2.30E-06 | 4.988469 |
| ENSG00000136250.10 | AOAH | -0.58142 | 2.047157 | -5.13387 | 3.91E-07 | 2.28E-06 | 4.997122 |
| ENSG00000052795.11 | FNIP2 | -0.58149 | 2.821999 | -5.15232 | 3.56E-07 | 2.08E-06 | 5.087786 |
| ENSG00000172578.10 | KLHL6 | -0.58149 | 1.403868 | -6.39439 | 3.37E-10 | 2.76E-09 | 11.85829 |
| ENSG00000116147.15 | TNR | -0.58246 | 0.122985 | -19.7498 | 1.89E-66 | 4.02E-64 | 140.4901 |
| ENSG00000177707.9 | PVRL3 | -0.58282 | 1.580988 | -5.02523 | 6.75E-07 | 3.81E-06 | 4.469129 |
| ENSG00000010626.13 | LRRC23 | -0.58292 | 2.442555 | -5.68586 | 2.09E-08 | 1.41E-07 | 7.83714 |
| ENSG00000048342.14 | CC2D2A | -0.58321 | 1.641277 | -8.23703 | 1.23E-15 | 1.64E-14 | 24.16316 |
| ENSG00000170891.9 | CYTL1 | -0.58358 | 1.046588 | -5.55473 | 4.28E-08 | 2.80E-07 | 7.138886 |
| ENSG00000097096.8 | SYDE2 | -0.58378 | 0.974436 | -8.51465 | 1.51E-16 | 2.16E-15 | 26.23268 |
| ENSG00000169499.13 | PLEKHA2 | -0.58401 | 2.853137 | -6.77556 | 3.11E-11 | 2.82E-10 | 14.19033 |
| ENSG00000132561.12 | MATN2 | -0.58413 | 1.836459 | -4.36302 | 1.52E-05 | 7.18E-05 | 1.476552 |
| ENSG00000119655.7 | NPC2 | -0.58415 | 7.48013 | -3.76349 | 0.000185024 | 0.000736529 | -0.89098 |
| ENSG00000141298.16 | SSH2 | -0.58456 | 2.045004 | -9.98019 | 1.02E-21 | 2.15E-20 | 38.00184 |
| ENSG00000157933.9 | SKI | -0.58485 | 4.175231 | -7.25936 | 1.29E-12 | 1.33E-11 | 17.31447 |
| ENSG00000120051.13 | CFAP58 | -0.58522 | 0.405635 | -8.68487 | 4.06E-17 | 6.09E-16 | 27.52777 |
| ENSG00000131409.11 | LRRC4B | -0.58542 | 0.663884 | -9.2254 | 5.55E-19 | 9.64E-18 | 31.76846 |
| ENSG00000107560.9 | RAB11FIP2 | -0.58586 | 2.085603 | -9.10792 | 1.43E-18 | 2.41E-17 | 30.8305 |
| ENSG00000038295.6 | TLL1 | -0.58597 | 0.490773 | -12.1317 | 2.85E-30 | 1.04E-28 | 57.54367 |
| ENSG00000165030.3 | NFIL3 | -0.5861 | 4.072818 | -6.53785 | 1.39E-10 | 1.19E-09 | 12.72239 |
| ENSG00000162722.8 | TRIM58 | -0.58651 | 0.206167 | -11.1698 | 2.56E-26 | 7.32E-25 | 48.50527 |
| ENSG00000198964.12 | SGMS1 | -0.58676 | 3.008653 | -6.401 | 3.24E-10 | 2.66E-09 | 11.89772 |
| ENSG00000102003.9 | SYP | -0.58734 | 0.732348 | -6.31723 | 5.39E-10 | 4.31E-09 | 11.40036 |
| ENSG00000150681.8 | RGS18 | -0.58746 | 0.998606 | -8.00862 | 6.62E-15 | 8.33E-14 | 22.50105 |
| ENSG00000197971.13 | MBP | -0.58798 | 1.944107 | -8.9984 | 3.44E-18 | 5.64E-17 | 29.96411 |
| ENSG00000112425.12 | EPM2A | -0.588 | 0.822909 | -16.847 | 6.32E-52 | 7.29E-50 | 107.1586 |
| ENSG00000155850.7 | SLC26A2 | -0.58826 | 2.255137 | -5.00228 | 7.57E-07 | 4.24E-06 | 4.358945 |
| ENSG00000162571.12 | TTLL10 | -0.58831 | 0.415878 | -7.66763 | 7.67E-14 | 8.81E-13 | 20.08935 |
| ENSG00000189319.12 | FAM53B | -0.58857 | 2.675102 | -7.57432 | 1.48E-13 | 1.66E-12 | 19.44421 |
| ENSG00000137198.8 | GMPR | -0.58857 | 2.290082 | -5.27792 | 1.86E-07 | 1.13E-06 | 5.712986 |
| ENSG00000118137.8 | APOA1 | -0.58884 | 0.314996 | -7.13893 | 2.90E-12 | 2.89E-11 | 16.51992 |
| ENSG00000091436.15 | pk | -0.58944 | 2.385355 | -7.51425 | 2.25E-13 | 2.49E-12 | 19.03225 |
| ENSG00000120705.11 | ETF1 | -0.5896 | 4.766346 | -10.8021 | 7.34E-25 | 1.93E-23 | 45.175 |
| ENSG00000078124.10 | ACER3 | -0.58985 | 2.302176 | -10.3866 | 2.99E-23 | 6.97E-22 | 41.5004 |
| ENSG00000128872.8 | TMOD2 | -0.59041 | 1.037832 | -10.6394 | 3.17E-24 | 7.91E-23 | 43.72483 |
| ENSG00000196776.13 | CD47 | -0.59069 | 4.933421 | -5.80191 | 1.09E-08 | 7.61E-08 | 8.467251 |
| ENSG00000104059.4 | FAM189A1 | -0.59081 | 0.270914 | -13.3787 | 1.14E-35 | 5.89E-34 | 69.89687 |
| ENSG00000110852.4 | CLEC2B | -0.59132 | 2.6389 | -4.85609 | 1.55E-06 | 8.35E-06 | 3.667749 |
| ENSG00000175920.14 | DOK7 | -0.59135 | 0.794105 | -7.76573 | 3.82E-14 | 4.50E-13 | 20.77451 |
| ENSG00000170017.11 | ALCAM | -0.5914 | 4.960409 | -4.88954 | 1.32E-06 | 7.17E-06 | 3.824241 |
| ENSG00000101333.15 | PLCB4 | -0.59143 | 1.043722 | -5.59365 | 3.46E-08 | 2.29E-07 | 7.344619 |
| ENSG00000196664.4 | TLR7 | -0.59181 | 1.18464 | -7.09063 | 3.99E-12 | 3.93E-11 | 16.2044 |
| ENSG00000205038.10 | PKHD1L1 | -0.59182 | 0.191833 | -13.6801 | 5.15E-37 | 2.87E-35 | 72.97796 |
| ENSG00000242732.4 | RGAG4 | -0.59259 | 1.54528 | -6.30744 | 5.72E-10 | 4.56E-09 | 11.34263 |
| ENSG00000138594.11 | TMOD3 | -0.59264 | 3.402551 | -8.8902 | 8.12E-18 | 1.29E-16 | 29.11591 |
| ENSG00000081479.11 | LRP2 | -0.59268 | 1.010085 | -4.60778 | 5.03E-06 | 2.54E-05 | 2.537082 |
| ENSG00000087237.9 | CETP | -0.59319 | 0.901048 | -10.6848 | 2.11E-24 | 5.33E-23 | 44.12743 |
| ENSG00000147570.8 | DNAJC5B | -0.59331 | 0.906597 | -7.85619 | 2.00E-14 | 2.42E-13 | 21.41254 |
| ENSG00000109756.7 | RAPGEF2 | -0.59348 | 2.255456 | -8.91381 | 6.74E-18 | 1.08E-16 | 29.30033 |
| ENSG00000163576.16 | EFHB | -0.59391 | 0.501845 | -7.84827 | 2.12E-14 | 2.56E-13 | 21.35645 |
| ENSG00000171100.13 | MTM1 | -0.59407 | 2.37456 | -9.464 | 7.87E-20 | 1.45E-18 | 33.70063 |
| ENSG00000170915.8 | PAQR8 | -0.59444 | 2.072663 | -5.94657 | 4.79E-09 | 3.48E-08 | 9.26845 |
| ENSG00000281227.1 | AC011380.1 | -0.59485 | 0.114587 | -15.3399 | 1.13E-44 | 9.48E-43 | 90.52477 |
| ENSG00000102243.11 | VGLL1 | -0.59571 | 1.089955 | -3.70315 | 0.000233778 | 0.000913705 | -1.11092 |
| ENSG00000160219.10 | GAB3 | -0.59615 | 1.288814 | -8.3826 | 4.11E-16 | 5.71E-15 | 25.24162 |
| ENSG00000150630.3 | VEGFC | -0.59646 | 2.045001 | -4.79214 | 2.11E-06 | 1.12E-05 | 3.371321 |
| ENSG00000131042.12 | LILRB2 | -0.59733 | 1.860789 | -5.82327 | 9.68E-09 | 6.79E-08 | 8.584444 |
| ENSG00000138964.15 | PARVG | -0.5975 | 1.744653 | -6.57249 | 1.12E-10 | 9.64E-10 | 12.9335 |
| ENSG00000099139.12 | PCSK5 | -0.59851 | 0.972021 | -8.9284 | 6.01E-18 | 9.69E-17 | 29.41454 |
| ENSG00000122223.11 | CD244 | -0.59872 | 0.798327 | -9.22758 | 5.45E-19 | 9.48E-18 | 31.78594 |
| ENSG00000152763.15 | WDR78 | -0.59892 | 0.839628 | -6.91981 | 1.23E-11 | 1.16E-10 | 15.10286 |
| ENSG00000166165.11 | CKB | -0.59913 | 4.94198 | -3.3146 | 0.000976399 | 0.003402406 | -2.44568 |
| ENSG00000020577.12 | SAMD4A | -0.59924 | 1.683711 | -6.41689 | 2.94E-10 | 2.42E-09 | 11.99274 |
| ENSG00000110324.8 | IL10RA | -0.59924 | 2.78675 | -5.24682 | 2.19E-07 | 1.32E-06 | 5.556932 |
| ENSG00000176928.5 | GCNT4 | -0.59941 | 0.698211 | -8.63989 | 5.75E-17 | 8.51E-16 | 27.18361 |
| ENSG00000172059.9 | KLF11 | -0.59987 | 2.743088 | -7.39558 | 5.10E-13 | 5.48E-12 | 18.22639 |
| ENSG00000186350.9 | RXRA | -0.60007 | 3.161373 | -8.02038 | 6.08E-15 | 7.67E-14 | 22.58569 |
| ENSG00000105967.14 | TFEC | -0.60009 | 1.139989 | -7.37443 | 5.89E-13 | 6.30E-12 | 18.08392 |
| ENSG00000001561.6 | ENPP4 | -0.6007 | 3.426966 | -6.16661 | 1.33E-09 | 1.02E-08 | 10.52047 |
| ENSG00000067840.11 | PDZD4 | -0.60098 | 0.822228 | -6.67128 | 6.04E-11 | 5.33E-10 | 13.54088 |
| ENSG00000003402.18 | CFLAR | -0.60113 | 3.180551 | -7.45238 | 3.45E-13 | 3.77E-12 | 18.61082 |
| ENSG00000230873.7 | STMND1 | -0.60127 | 0.632267 | -5.87282 | 7.31E-09 | 5.20E-08 | 8.857821 |
| ENSG00000170275.13 | CRTAP | -0.60133 | 4.55639 | -8.95315 | 4.94E-18 | 8.01E-17 | 29.60844 |
| ENSG00000170955.9 | PRKCDBP | -0.60193 | 3.341226 | -5.00776 | 7.37E-07 | 4.14E-06 | 4.385191 |
| ENSG00000182247.8 | UBE2E2 | -0.60202 | 2.835461 | -7.13049 | 3.06E-12 | 3.04E-11 | 16.46469 |
| ENSG00000169994.17 | MYO7B | -0.60346 | 0.35382 | -10.1766 | 1.87E-22 | 4.16E-21 | 39.68101 |
| ENSG00000058668.13 | ATP2B4 | -0.60377 | 4.246369 | -5.52811 | 4.95E-08 | 3.21E-07 | 6.998919 |
| ENSG00000120915.12 | EPHX2 | -0.60415 | 1.960728 | -6.02191 | 3.10E-09 | 2.30E-08 | 9.692662 |
| ENSG00000048540.13 | LMO3 | -0.60465 | 3.144804 | -3.18172 | 0.001544218 | 0.005164157 | -2.86955 |
| ENSG00000072736.17 | NFATC3 | -0.60547 | 2.0201 | -9.33122 | 2.34E-19 | 4.18E-18 | 32.62088 |
| ENSG00000163817.14 | SLC6A20 | -0.60566 | 0.907394 | -4.61422 | 4.88E-06 | 2.47E-05 | 2.565727 |
| ENSG00000109339.17 | MAPK10 | -0.60586 | 0.721401 | -8.61485 | 6.97E-17 | 1.03E-15 | 26.99264 |
| ENSG00000152785.6 | BMP3 | -0.6072 | 2.015958 | -3.2754 | 0.001119579 | 0.003854259 | -2.57245 |
| ENSG00000115523.15 | GNLY | -0.60733 | 2.162994 | -3.56611 | 0.000392948 | 0.001474177 | -1.59782 |
| ENSG00000183741.10 | CBX6 | -0.60757 | 3.060383 | -5.38911 | 1.04E-07 | 6.51E-07 | 6.277845 |
| ENSG00000203734.10 | ECT2L | -0.60763 | 0.441588 | -7.07255 | 4.50E-12 | 4.42E-11 | 16.0867 |
| ENSG00000198846.5 | TOX | -0.60804 | 1.949757 | -4.98308 | 8.33E-07 | 4.64E-06 | 4.267074 |
| ENSG00000180447.6 | GAS1 | -0.60877 | 1.802887 | -4.70572 | 3.18E-06 | 1.65E-05 | 2.97648 |
| ENSG00000144959.8 | NCEH1 | -0.60931 | 3.422618 | -5.55897 | 4.18E-08 | 2.74E-07 | 7.161221 |
| ENSG00000188257.9 | PLA2G2A | -0.6094 | 1.407492 | -3.1291 | 0.001843592 | 0.006058791 | -3.03276 |
| ENSG00000198865.8 | CCDC152 | -0.60974 | 0.663822 | -14.2246 | 1.75E-39 | 1.10E-37 | 78.63193 |
| ENSG00000113645.12 | WWC1 | -0.61169 | 2.781148 | -7.01143 | 6.74E-12 | 6.50E-11 | 15.69089 |
| ENSG00000087086.12 | FTL | -0.61232 | 12.14329 | -5.26554 | 1.99E-07 | 1.20E-06 | 5.650756 |
| ENSG00000157330.8 | C1orf158 | -0.61235 | 0.297428 | -8.79793 | 1.68E-17 | 2.61E-16 | 28.39877 |
| ENSG00000140682.17 | TGFB1I1 | -0.61258 | 2.409058 | -6.80531 | 2.57E-11 | 2.35E-10 | 14.37718 |
| ENSG00000162643.11 | WDR63 | -0.61281 | 0.49466 | -7.70185 | 6.02E-14 | 6.98E-13 | 20.32752 |
| ENSG00000266094.5 | RASSF5 | -0.613 | 2.988624 | -6.62374 | 8.15E-11 | 7.09E-10 | 13.24765 |
| ENSG00000157168.17 | NRG1 | -0.61313 | 0.421025 | -11.1732 | 2.48E-26 | 7.11E-25 | 48.53634 |
| ENSG00000137075.16 | RNF38 | -0.61322 | 2.956231 | -9.18343 | 7.80E-19 | 1.34E-17 | 31.43236 |
| ENSG00000131069.18 | ACSS2 | -0.61338 | 3.815819 | -6.27533 | 6.94E-10 | 5.49E-09 | 11.15375 |
| ENSG00000140526.15 | ABHD2 | -0.61374 | 4.398228 | -4.87344 | 1.43E-06 | 7.71E-06 | 3.748776 |
| ENSG00000125730.15 | C3 | -0.61376 | 6.577911 | -3.02464 | 0.002602139 | 0.008297404 | -3.34902 |
| ENSG00000204711.7 | C9orf135 | -0.61457 | 1.027848 | -3.75981 | 0.000187695 | 0.000746229 | -0.90447 |
| ENSG00000124145.6 | SDC4 | -0.61494 | 7.587209 | -5.72952 | 1.64E-08 | 1.12E-07 | 8.072865 |
| ENSG00000198853.10 | RUSC2 | -0.61517 | 2.364173 | -8.47127 | 2.10E-16 | 2.98E-15 | 25.90574 |
| ENSG00000123977.8 | DAW1 | -0.61537 | 0.804983 | -5.52058 | 5.15E-08 | 3.34E-07 | 6.959424 |
| ENSG00000128917.6 | DLL4 | -0.61537 | 2.553922 | -6.44412 | 2.49E-10 | 2.07E-09 | 12.15596 |
| ENSG00000172458.4 | IL17D | -0.61568 | 0.5426 | -14.3082 | 7.25E-40 | 4.64E-38 | 79.50932 |
| ENSG00000005812.9 | FBXL3 | -0.61584 | 3.001463 | -10.0382 | 6.20E-22 | 1.32E-20 | 38.49515 |
| ENSG00000138135.6 | CH25H | -0.61709 | 2.540263 | -3.4308 | 0.000645652 | 0.002323355 | -2.06134 |
| ENSG00000130558.17 | OLFM1 | -0.61725 | 1.320252 | -5.40644 | 9.49E-08 | 5.97E-07 | 6.366869 |
| ENSG00000172426.14 | RSPH9 | -0.61766 | 0.9796 | -6.93468 | 1.11E-11 | 1.05E-10 | 15.19786 |
| ENSG00000156427.7 | FGF18 | -0.61776 | 0.94901 | -5.55334 | 4.31E-08 | 2.82E-07 | 7.131551 |
| ENSG00000159335.14 | PTMS | -0.61801 | 6.822941 | -6.0215 | 3.11E-09 | 2.30E-08 | 9.690338 |
| ENSG00000163251.3 | FZD5 | -0.61873 | 2.886388 | -6.82318 | 2.29E-11 | 2.10E-10 | 14.48976 |
| ENSG00000164088.16 | PPM1M | -0.61875 | 3.171293 | -8.23619 | 1.23E-15 | 1.65E-14 | 24.15702 |
| ENSG00000031003.9 | FAM13B | -0.61927 | 2.206546 | -9.38098 | 1.56E-19 | 2.81E-18 | 33.02423 |
| ENSG00000139304.11 | PTPRQ | -0.61941 | 0.122525 | -22.8133 | 3.31E-82 | 1.38E-79 | 176.6625 |
| ENSG00000184014.6 | DENND5A | -0.61944 | 3.182783 | -9.89726 | 2.07E-21 | 4.27E-20 | 37.29987 |
| ENSG00000077420.14 | APBB1IP | -0.62026 | 2.481991 | -5.87356 | 7.28E-09 | 5.18E-08 | 8.86193 |
| ENSG00000127472.9 | PLA2G5 | -0.62039 | 0.876906 | -10.7379 | 1.31E-24 | 3.37E-23 | 44.60112 |
| ENSG00000156313.11 | RPGR | -0.62071 | 1.258916 | -10.7954 | 7.80E-25 | 2.04E-23 | 45.11476 |
| ENSG00000172349.15 | IL16 | -0.62119 | 1.529361 | -7.20517 | 1.86E-12 | 1.89E-11 | 16.95554 |
| ENSG00000134198.8 | TSPAN2 | -0.6225 | 1.358905 | -7.30028 | 9.77E-13 | 1.02E-11 | 17.58691 |
| ENSG00000203778.6 | FAM229B | -0.62292 | 1.806399 | -6.39644 | 3.33E-10 | 2.73E-09 | 11.87052 |
| ENSG00000172243.16 | CLEC7A | -0.62352 | 2.634837 | -4.93918 | 1.03E-06 | 5.71E-06 | 4.05826 |
| ENSG00000184384.12 | MAML2 | -0.62489 | 2.279332 | -6.18967 | 1.16E-09 | 8.97E-09 | 10.65398 |
| ENSG00000211456.9 | SACM1L | -0.62492 | 2.954454 | -11.3975 | 3.09E-27 | 9.41E-26 | 50.60312 |
| ENSG00000170836.10 | PPM1D | -0.62493 | 2.159725 | -12.1351 | 2.75E-30 | 1.01E-28 | 57.57703 |
| ENSG00000140505.6 | CYP1A2 | -0.62506 | 0.122478 | -13.8618 | 7.81E-38 | 4.55E-36 | 74.85313 |
| ENSG00000181378.12 | CCDC108 | -0.62601 | 0.39698 | -7.85556 | 2.01E-14 | 2.43E-13 | 21.40809 |
| ENSG00000169313.9 | P2RY12 | -0.62627 | 0.830068 | -7.89326 | 1.53E-14 | 1.87E-13 | 21.67568 |
| ENSG00000163141.17 | BNIPL | -0.62653 | 2.000679 | -3.73363 | 0.000207807 | 0.000819777 | -1.00023 |
| ENSG00000160888.6 | IER2 | -0.62742 | 4.874926 | -5.88853 | 6.68E-09 | 4.78E-08 | 8.944931 |
| ENSG00000134443.8 | GRP | -0.62745 | 1.09487 | -3.70142 | 0.00023534 | 0.000919215 | -1.11717 |
| ENSG00000182118.5 | FAM89A | -0.62752 | 2.962839 | -5.39492 | 1.01E-07 | 6.32E-07 | 6.307645 |
| ENSG00000144230.15 | GPR17 | -0.62764 | 0.318885 | -13.5701 | 1.60E-36 | 8.72E-35 | 71.84978 |
| ENSG00000171798.16 | KNDC1 | -0.62831 | 1.182902 | -5.10619 | 4.50E-07 | 2.60E-06 | 4.861632 |
| ENSG00000126561.15 | STAT5A | -0.62853 | 2.805364 | -7.85647 | 2.00E-14 | 2.42E-13 | 21.41451 |
| ENSG00000197208.5 | SLC22A4 | -0.62908 | 1.176711 | -6.69084 | 5.34E-11 | 4.74E-10 | 13.66203 |
| ENSG00000163812.12 | ZDHHC3 | -0.62988 | 3.63563 | -8.54535 | 1.19E-16 | 1.72E-15 | 26.46477 |
| ENSG00000173627.7 | APOBEC4 | -0.63041 | 0.390193 | -7.44847 | 3.54E-13 | 3.87E-12 | 18.58427 |
| ENSG00000164188.7 | RANBP3L | -0.63056 | 0.391773 | -14.4266 | 2.07E-40 | 1.37E-38 | 80.75607 |
| ENSG00000154258.15 | ABCA9 | -0.63069 | 0.471633 | -14.6521 | 1.88E-41 | 1.34E-39 | 83.14256 |
| ENSG00000198911.10 | SREBF2 | -0.63077 | 4.597232 | -7.68407 | 6.83E-14 | 7.88E-13 | 20.20369 |
| ENSG00000073464.10 | CLCN4 | -0.631 | 1.386816 | -7.83733 | 2.29E-14 | 2.76E-13 | 21.27904 |
| ENSG00000100242.14 | SUN2 | -0.63129 | 4.497135 | -7.8709 | 1.80E-14 | 2.19E-13 | 21.51684 |
| ENSG00000136014.10 | USP44 | -0.6314 | 1.015568 | -7.53676 | 1.92E-13 | 2.14E-12 | 19.18627 |
| ENSG00000153814.10 | JAZF1 | -0.63164 | 2.095018 | -8.7189 | 3.11E-17 | 4.72E-16 | 27.78899 |
| ENSG00000145703.14 | IQGAP2 | -0.63187 | 1.850217 | -5.75727 | 1.40E-08 | 9.66E-08 | 8.223528 |
| ENSG00000105376.4 | ICAM5 | -0.63207 | 1.605957 | -4.7743 | 2.30E-06 | 1.21E-05 | 3.289274 |
| ENSG00000072657.7 | TRHDE | -0.63412 | 0.19024 | -16.8073 | 9.88E-52 | 1.14E-49 | 106.7139 |
| ENSG00000078018.18 | MAP2 | -0.63422 | 1.812607 | -4.58613 | 5.56E-06 | 2.79E-05 | 2.441096 |
| ENSG00000204381.10 | LAYN | -0.63423 | 1.917825 | -6.13895 | 1.56E-09 | 1.19E-08 | 10.36089 |
| ENSG00000072786.11 | STK10 | -0.63434 | 2.493708 | -7.74385 | 4.47E-14 | 5.23E-13 | 20.62107 |
| ENSG00000092068.17 | SLC7A8 | -0.63484 | 2.905214 | -4.25197 | 2.48E-05 | 0.000113163 | 1.013221 |
| ENSG00000125355.14 | TMEM255A | -0.63488 | 0.874496 | -7.78504 | 3.33E-14 | 3.95E-13 | 20.91021 |
| ENSG00000154080.11 | CHST9 | -0.63524 | 0.472079 | -6.19345 | 1.13E-09 | 8.78E-09 | 10.67592 |
| ENSG00000153902.12 | LGI4 | -0.63598 | 0.580856 | -15.8126 | 6.40E-47 | 6.05E-45 | 95.67849 |
| ENSG00000107263.17 | RAPGEF1 | -0.63604 | 3.926625 | -8.20942 | 1.51E-15 | 2.00E-14 | 23.96026 |
| ENSG00000184588.16 | PDE4B | -0.63633 | 1.776201 | -6.98166 | 8.19E-12 | 7.83E-11 | 15.49906 |
| ENSG00000113161.14 | HMGCR | -0.63662 | 3.270206 | -8.58469 | 8.80E-17 | 1.28E-15 | 26.76315 |
| ENSG00000115993.10 | TRAK2 | -0.63745 | 3.228108 | -9.6767 | 1.34E-20 | 2.58E-19 | 35.45324 |
| ENSG00000162599.14 | NFIA | -0.63749 | 2.008284 | -7.11854 | 3.32E-12 | 3.29E-11 | 16.38652 |
| ENSG00000144668.10 | ITGA9 | -0.63821 | 2.290993 | -5.3512 | 1.27E-07 | 7.86E-07 | 6.084104 |
| ENSG00000135205.13 | CCDC146 | -0.63829 | 1.33607 | -5.91341 | 5.80E-09 | 4.17E-08 | 9.08328 |
| ENSG00000177432.6 | NAP1L5 | -0.6388 | 1.401585 | -9.99348 | 9.11E-22 | 1.92E-20 | 38.11469 |
| ENSG00000205542.9 | TMSB4X | -0.63916 | 10.01523 | -6.24552 | 8.30E-10 | 6.52E-09 | 10.97915 |
| ENSG00000100599.14 | RIN3 | -0.63942 | 2.356253 | -8.03655 | 5.40E-15 | 6.85E-14 | 22.7023 |
| ENSG00000132554.18 | RGS22 | -0.63951 | 0.404429 | -9.62084 | 2.14E-20 | 4.06E-19 | 34.9902 |
| ENSG00000108798.7 | ABI3 | -0.63959 | 2.79758 | -6.59144 | 9.98E-11 | 8.60E-10 | 13.04944 |
| ENSG00000224383.6 | PRR29 | -0.63959 | 1.339496 | -5.91746 | 5.66E-09 | 4.08E-08 | 9.105829 |
| ENSG00000110108.8 | TMEM109 | -0.6408 | 5.668608 | -9.97874 | 1.03E-21 | 2.17E-20 | 37.98954 |
| ENSG00000186329.8 | TMEM212 | -0.64142 | 0.32138 | -8.58853 | 8.54E-17 | 1.25E-15 | 26.79232 |
| ENSG00000033800.12 | PIAS1 | -0.64143 | 2.183066 | -13.3045 | 2.43E-35 | 1.23E-33 | 69.14326 |
| ENSG00000197043.12 | ANXA6 | -0.6421 | 4.531886 | -6.86893 | 1.71E-11 | 1.59E-10 | 14.77912 |
| ENSG00000155016.16 | CYP2U1 | -0.6422 | 1.422515 | -10.285 | 7.29E-23 | 1.66E-21 | 40.61674 |
| ENSG00000101222.11 | SPEF1 | -0.64225 | 0.886853 | -5.69104 | 2.03E-08 | 1.37E-07 | 7.865041 |
| ENSG00000141505.10 | ASGR1 | -0.64227 | 0.956579 | -9.52222 | 4.86E-20 | 9.06E-19 | 34.17752 |
| ENSG00000198756.9 | COLGALT2 | -0.64239 | 0.477481 | -8.01568 | 6.29E-15 | 7.93E-14 | 22.55183 |
| ENSG00000188677.13 | PARVB | -0.64272 | 2.131901 | -8.46546 | 2.19E-16 | 3.11E-15 | 25.86209 |
| ENSG00000197273.3 | GUCA2A | -0.64274 | 0.35242 | -7.24254 | 1.44E-12 | 1.49E-11 | 17.20281 |
| ENSG00000143341.10 | HMCN1 | -0.64302 | 1.443634 | -6.29623 | 6.12E-10 | 4.87E-09 | 11.27657 |
| ENSG00000153064.10 | BANK1 | -0.64329 | 1.607707 | -7.09906 | 3.78E-12 | 3.72E-11 | 16.25935 |
| ENSG00000135636.12 | DYSF | -0.64351 | 2.451006 | -6.19242 | 1.14E-09 | 8.83E-09 | 10.66994 |
| ENSG00000007264.12 | MATK | -0.64413 | 1.117759 | -8.39051 | 3.87E-16 | 5.39E-15 | 25.30064 |
| ENSG00000174306.20 | ZHX3 | -0.64415 | 1.858437 | -10.6112 | 4.08E-24 | 1.01E-22 | 43.47478 |
| ENSG00000166974.11 | MAPRE2 | -0.64428 | 3.171559 | -8.26487 | 9.96E-16 | 1.34E-14 | 24.36829 |
| ENSG00000080493.12 | SLC4A4 | -0.64518 | 2.085808 | -4.44817 | 1.04E-05 | 5.03E-05 | 1.839392 |
| ENSG00000130037.4 | KCNA5 | -0.64556 | 0.35685 | -14.8031 | 3.74E-42 | 2.76E-40 | 84.75072 |
| ENSG00000150281.6 | CTF1 | -0.64587 | 2.648136 | -5.62548 | 2.91E-08 | 1.94E-07 | 7.513854 |
| ENSG00000108175.15 | ZMIZ1 | -0.64613 | 3.342047 | -7.8512 | 2.07E-14 | 2.51E-13 | 21.37716 |
| ENSG00000174502.17 | SLC26A9 | -0.64643 | 2.536581 | -2.87495 | 0.004192681 | 0.012735101 | -3.78402 |
| ENSG00000174898.14 | CATSPERD | -0.64656 | 0.281881 | -10.0588 | 5.20E-22 | 1.12E-20 | 38.67079 |
| ENSG00000178038.15 | ALS2CL | -0.64744 | 2.281337 | -5.06777 | 5.46E-07 | 3.12E-06 | 4.674639 |
| ENSG00000205302.5 | SNX2 | -0.64766 | 4.103903 | -11.1812 | 2.30E-26 | 6.62E-25 | 48.60966 |
| ENSG00000165804.14 | ZNF219 | -0.64789 | 2.243159 | -6.76744 | 3.28E-11 | 2.97E-10 | 14.13944 |
| ENSG00000123836.13 | PFKFB2 | -0.64805 | 2.466537 | -7.30468 | 9.48E-13 | 9.97E-12 | 17.61629 |
| ENSG00000186377.7 | CYP4X1 | -0.64808 | 2.305683 | -3.86554 | 0.000123678 | 0.000507388 | -0.51132 |
| ENSG00000166348.16 | USP54 | -0.64822 | 2.774452 | -7.24356 | 1.43E-12 | 1.48E-11 | 17.20955 |
| ENSG00000168216.9 | LMBRD1 | -0.64865 | 4.684124 | -9.32661 | 2.43E-19 | 4.33E-18 | 32.58362 |
| ENSG00000178568.12 | ERBB4 | -0.64866 | 0.355405 | -13.19 | 7.79E-35 | 3.79E-33 | 67.98503 |
| ENSG00000171962.16 | LRRC48 | -0.64885 | 0.894261 | -7.50813 | 2.34E-13 | 2.60E-12 | 18.99047 |
| ENSG00000222046.2 | DCDC2B | -0.64933 | 0.506082 | -7.93275 | 1.15E-14 | 1.42E-13 | 21.95712 |
| ENSG00000119698.10 | PPP4R4 | -0.65027 | 0.43502 | -11.5555 | 7.02E-28 | 2.22E-26 | 52.07394 |
| ENSG00000198189.9 | HSD17B11 | -0.65038 | 5.333326 | -6.01455 | 3.24E-09 | 2.39E-08 | 9.650994 |
| ENSG00000181826.8 | RELL1 | -0.65047 | 1.721458 | -7.18645 | 2.11E-12 | 2.13E-11 | 16.8321 |
| ENSG00000158467.15 | AHCYL2 | -0.65087 | 4.272061 | -3.74124 | 0.000201762 | 0.000797748 | -0.97246 |
| ENSG00000164197.10 | RNF180 | -0.65148 | 1.025771 | -8.81829 | 1.43E-17 | 2.23E-16 | 28.55654 |
| ENSG00000109321.9 | AREG | -0.65161 | 3.962018 | -2.43401 | 0.015240591 | 0.040261921 | -4.94046 |
| ENSG00000186469.7 | GNG2 | -0.65207 | 1.846948 | -8.36717 | 4.62E-16 | 6.39E-15 | 25.12663 |
| ENSG00000117461.13 | PIK3R3 | -0.65223 | 2.412821 | -6.48352 | 1.95E-10 | 1.64E-09 | 12.39322 |
| ENSG00000083290.18 | ULK2 | -0.6531 | 1.754421 | -10.3009 | 6.34E-23 | 1.45E-21 | 40.75504 |
| ENSG00000143842.13 | SOX13 | -0.65337 | 2.403 | -6.43487 | 2.63E-10 | 2.18E-09 | 12.10042 |
| ENSG00000169314.13 | C22orf15 | -0.65384 | 0.474585 | -7.38834 | 5.36E-13 | 5.75E-12 | 18.17758 |
| ENSG00000018236.13 | CNTN1 | -0.65408 | 0.913459 | -5.38842 | 1.04E-07 | 6.53E-07 | 6.274335 |
| ENSG00000118689.13 | FOXO3 | -0.65416 | 2.964857 | -8.0482 | 4.96E-15 | 6.31E-14 | 22.7864 |
| ENSG00000124762.12 | CDKN1A | -0.65422 | 5.27574 | -5.78636 | 1.19E-08 | 8.27E-08 | 8.382173 |
| ENSG00000158481.11 | CD1C | -0.6545 | 1.946227 | -4.25569 | 2.44E-05 | 0.000111446 | 1.028579 |
| ENSG00000169126.14 | ARMC4 | -0.65681 | 0.585863 | -6.86041 | 1.80E-11 | 1.67E-10 | 14.7251 |
| ENSG00000165886.4 | UBTD1 | -0.65713 | 4.217706 | -6.62026 | 8.33E-11 | 7.24E-10 | 13.22622 |
| ENSG00000096968.11 | JAK2 | -0.65738 | 2.057526 | -8.38097 | 4.16E-16 | 5.77E-15 | 25.22946 |
| ENSG00000108405.3 | P2RX1 | -0.65757 | 0.981661 | -8.76882 | 2.11E-17 | 3.25E-16 | 28.17366 |
| ENSG00000196924.13 | FLNA | -0.65804 | 6.606036 | -4.88841 | 1.33E-06 | 7.20E-06 | 3.818926 |
| ENSG00000150995.16 | ITPR1 | -0.65859 | 2.04469 | -6.77843 | 3.06E-11 | 2.78E-10 | 14.20836 |
| ENSG00000101162.3 | TUBB1 | -0.65974 | 0.256464 | -19.1379 | 2.41E-63 | 4.56E-61 | 133.3593 |
| ENSG00000148468.15 | FAM171A1 | -0.66007 | 2.178604 | -5.51737 | 5.24E-08 | 3.39E-07 | 6.942622 |
| ENSG00000182568.15 | SATB1 | -0.66014 | 1.533312 | -8.12557 | 2.81E-15 | 3.64E-14 | 23.34749 |
| ENSG00000188641.11 | DPYD | -0.66039 | 3.221012 | -5.11527 | 4.29E-07 | 2.49E-06 | 4.905973 |
| ENSG00000150636.14 | CCDC102B | -0.66057 | 1.195007 | -10.9909 | 1.32E-25 | 3.63E-24 | 46.87566 |
| ENSG00000019991.14 | HGF | -0.6608 | 1.22951 | -7.21208 | 1.77E-12 | 1.80E-11 | 17.00118 |
| ENSG00000155629.13 | PIK3AP1 | -0.66108 | 2.651733 | -5.63192 | 2.81E-08 | 1.87E-07 | 7.548146 |
| ENSG00000196872.9 | KIAA1211L | -0.66153 | 2.129799 | -6.46521 | 2.19E-10 | 1.82E-09 | 12.28277 |
| ENSG00000130940.13 | CASZ1 | -0.66191 | 1.401185 | -9.14279 | 1.08E-18 | 1.84E-17 | 31.10793 |
| ENSG00000085563.13 | ABCB1 | -0.66227 | 0.716677 | -9.79578 | 4.91E-21 | 9.86E-20 | 36.44658 |
| ENSG00000149289.9 | ZC3H12C | -0.66247 | 1.347227 | -9.95371 | 1.28E-21 | 2.68E-20 | 37.77722 |
| ENSG00000148541.11 | FAM13C | -0.66287 | 0.517767 | -15.6778 | 2.82E-46 | 2.57E-44 | 94.20222 |
| ENSG00000153179.10 | RASSF3 | -0.66313 | 4.018844 | -8.05607 | 4.68E-15 | 5.97E-14 | 22.84331 |
| ENSG00000166046.9 | TCP11L2 | -0.66358 | 1.299494 | -10.8863 | 3.43E-25 | 9.21E-24 | 45.93098 |
| ENSG00000154856.11 | APCDD1 | -0.6643 | 1.902953 | -6.24149 | 8.50E-10 | 6.67E-09 | 10.95559 |
| ENSG00000148180.15 | GSN | -0.6647 | 5.33748 | -6.24094 | 8.53E-10 | 6.69E-09 | 10.95241 |
| ENSG00000117009.10 | KMO | -0.66511 | 0.857869 | -10.7741 | 9.46E-25 | 2.45E-23 | 44.92374 |
| ENSG00000089250.17 | NOS1 | -0.66569 | 0.246056 | -10.4368 | 1.92E-23 | 4.55E-22 | 41.93962 |
| ENSG00000169064.11 | ZBBX | -0.6657 | 0.500658 | -6.97341 | 8.65E-12 | 8.25E-11 | 15.44606 |
| ENSG00000117519.14 | CNN3 | -0.66585 | 5.866508 | -6.62813 | 7.93E-11 | 6.91E-10 | 13.27464 |
| ENSG00000142611.15 | PRDM16 | -0.66619 | 1.207618 | -5.64431 | 2.62E-08 | 1.76E-07 | 7.614356 |
| ENSG00000081087.13 | OSTM1 | -0.6672 | 2.904344 | -9.71353 | 9.83E-21 | 1.92E-19 | 35.75948 |
| ENSG00000198668.9 | CALM1 | -0.66735 | 5.583965 | -11.5791 | 5.62E-28 | 1.79E-26 | 52.29506 |
| ENSG00000188001.8 | TPRG1 | -0.6675 | 0.576674 | -12.4233 | 1.65E-31 | 6.57E-30 | 60.37135 |
| ENSG00000143344.14 | RGL1 | -0.66776 | 3.187662 | -6.1761 | 1.25E-09 | 9.69E-09 | 10.57534 |
| ENSG00000137801.10 | THBS1 | -0.66782 | 5.023393 | -3.95457 | 8.64E-05 | 0.000363964 | -0.17225 |
| ENSG00000167315.16 | ACAA2 | -0.66788 | 3.377649 | -7.32115 | 8.48E-13 | 8.95E-12 | 17.72639 |
| ENSG00000152270.7 | PDE3B | -0.66794 | 1.165399 | -8.06572 | 4.36E-15 | 5.57E-14 | 22.91311 |
| ENSG00000026652.12 | AGPAT4 | -0.66809 | 1.02869 | -11.0428 | 8.22E-26 | 2.29E-24 | 47.34662 |
| ENSG00000085741.11 | WNT11 | -0.66816 | 0.909339 | -6.71636 | 4.54E-11 | 4.06E-10 | 13.82058 |
| ENSG00000106829.17 | TLE4 | -0.66874 | 1.661493 | -8.77733 | 1.97E-17 | 3.04E-16 | 28.23943 |
| ENSG00000129450.7 | SIGLEC9 | -0.66958 | 1.573601 | -7.48516 | 2.75E-13 | 3.03E-12 | 18.83375 |
| ENSG00000132321.15 | IQCA1 | -0.66959 | 0.975208 | -6.67822 | 5.78E-11 | 5.12E-10 | 13.58379 |
| ENSG00000163827.11 | LRRC2 | -0.6699 | 0.296464 | -19.562 | 1.70E-65 | 3.48E-63 | 138.2971 |
| ENSG00000112414.13 | GPR126 | -0.66993 | 2.382252 | -5.48142 | 6.36E-08 | 4.08E-07 | 6.754865 |
| ENSG00000106780.8 | MEGF9 | -0.6702 | 3.642135 | -5.77828 | 1.25E-08 | 8.63E-08 | 8.338013 |
| ENSG00000155897.8 | ADCY8 | -0.67045 | 0.139071 | -17.9019 | 3.90E-57 | 5.77E-55 | 119.1097 |
| ENSG00000237541.3 | HLA-DQA2 | -0.67046 | 4.505385 | -2.54488 | 0.011195681 | 0.030666564 | -4.66734 |
| ENSG00000155093.16 | PTPRN2 | -0.67054 | 1.970618 | -4.07147 | 5.34E-05 | 0.000232362 | 0.284085 |
| ENSG00000099769.5 | IGFALS | -0.67075 | 0.875974 | -5.97255 | 4.13E-09 | 3.02E-08 | 9.414219 |
| ENSG00000179855.5 | GIPC3 | -0.67103 | 1.107071 | -10.4211 | 2.20E-23 | 5.20E-22 | 41.80173 |
| ENSG00000152760.8 | TCTEX1D1 | -0.67106 | 0.512634 | -9.32075 | 2.55E-19 | 4.53E-18 | 32.53621 |
| ENSG00000175267.13 | VWA3A | -0.67148 | 0.529196 | -6.56179 | 1.20E-10 | 1.03E-09 | 12.8682 |
| ENSG00000095970.15 | TREM2 | -0.67185 | 4.215185 | -4.09808 | 4.77E-05 | 0.000209351 | 0.389698 |
| ENSG00000149591.15 | TAGLN | -0.67217 | 5.405952 | -4.78188 | 2.22E-06 | 1.17E-05 | 3.324099 |
| ENSG00000085117.10 | CD82 | -0.67232 | 4.202859 | -5.69514 | 1.98E-08 | 1.35E-07 | 7.887135 |
| ENSG00000065413.15 | ANKRD44 | -0.67248 | 1.564789 | -8.49831 | 1.71E-16 | 2.44E-15 | 26.10937 |
| ENSG00000188549.11 | C15orf52 | -0.67252 | 1.681304 | -6.84491 | 1.99E-11 | 1.84E-10 | 14.62703 |
| ENSG00000172260.12 | NEGR1 | -0.67258 | 0.488715 | -13.0094 | 4.84E-34 | 2.25E-32 | 66.1697 |
| ENSG00000117525.12 | F3 | -0.67365 | 5.093705 | -3.60906 | 0.000334522 | 0.001270353 | -1.44711 |
| ENSG00000173638.17 | SLC19A1 | -0.674 | 1.993248 | -9.57824 | 3.05E-20 | 5.76E-19 | 34.63844 |
| ENSG00000154099.16 | DNAAF1 | -0.67418 | 0.662818 | -6.30771 | 5.71E-10 | 4.56E-09 | 11.34424 |
| ENSG00000130830.13 | MPP1 | -0.67462 | 2.5092 | -6.91528 | 1.26E-11 | 1.19E-10 | 15.07396 |
| ENSG00000132718.8 | SYT11 | -0.67462 | 2.120694 | -6.98594 | 7.97E-12 | 7.63E-11 | 15.5266 |
| ENSG00000112576.11 | CCND3 | -0.6747 | 4.484095 | -6.44342 | 2.50E-10 | 2.07E-09 | 12.15174 |
| ENSG00000126860.10 | EVI2A | -0.67508 | 2.78062 | -5.58541 | 3.62E-08 | 2.39E-07 | 7.300974 |
| ENSG00000085276.16 | MECOM | -0.6751 | 2.868954 | -4.88695 | 1.34E-06 | 7.25E-06 | 3.812085 |
| ENSG00000187601.4 | MAGEH1 | -0.67529 | 3.849776 | -6.98638 | 7.94E-12 | 7.61E-11 | 15.52946 |
| ENSG00000160255.15 | ITGB2 | -0.67568 | 4.721427 | -4.39399 | 1.33E-05 | 6.32E-05 | 1.607781 |
| ENSG00000172379.17 | ARNT2 | -0.67614 | 1.760758 | -5.26314 | 2.01E-07 | 1.22E-06 | 5.638707 |
| ENSG00000125148.6 | MT2A | -0.67662 | 6.202506 | -3.70412 | 0.000232905 | 0.000910471 | -1.1074 |
| ENSG00000196329.9 | GIMAP5 | -0.67694 | 0.540642 | -14.091 | 7.13E-39 | 4.41E-37 | 77.23448 |
| ENSG00000162631.17 | NTNG1 | -0.67783 | 0.333062 | -11.4216 | 2.47E-27 | 7.54E-26 | 50.82666 |
| ENSG00000011198.6 | ABHD5 | -0.67802 | 2.785691 | -11.0619 | 6.90E-26 | 1.93E-24 | 47.52019 |
| ENSG00000168631.10 | DPCR1 | -0.67824 | 1.028543 | -3.60457 | 0.000340217 | 0.0012906 | -1.46292 |
| ENSG00000082438.14 | COBLL1 | -0.67861 | 1.826065 | -8.66915 | 4.58E-17 | 6.84E-16 | 27.40733 |
| ENSG00000139537.9 | CCDC65 | -0.67957 | 1.256608 | -7.29753 | 9.95E-13 | 1.04E-11 | 17.56856 |
| ENSG00000108828.14 | VAT1 | -0.68025 | 5.865765 | -7.73444 | 4.78E-14 | 5.59E-13 | 20.55515 |
| ENSG00000214128.9 | TMEM213 | -0.68095 | 0.780866 | -5.09237 | 4.82E-07 | 2.77E-06 | 4.794214 |
| ENSG00000173611.16 | SCAI | -0.68139 | 1.063355 | -13.8641 | 7.63E-38 | 4.45E-36 | 74.87648 |
| ENSG00000084710.12 | EFR3B | -0.68147 | 0.902045 | -7.91173 | 1.34E-14 | 1.65E-13 | 21.80724 |
| ENSG00000015285.9 | WAS | -0.68153 | 2.971916 | -6.2551 | 7.83E-10 | 6.17E-09 | 11.0352 |
| ENSG00000110934.9 | BIN2 | -0.68165 | 2.166399 | -6.78598 | 2.91E-11 | 2.65E-10 | 14.25571 |
| ENSG00000153714.5 | LURAP1L | -0.68212 | 3.196787 | -6.78089 | 3.01E-11 | 2.73E-10 | 14.2238 |
| ENSG00000102032.11 | RENBP | -0.68264 | 3.129101 | -5.57959 | 3.74E-08 | 2.46E-07 | 7.270124 |
| ENSG00000136235.14 | GPNMB | -0.6827 | 5.309612 | -4.12373 | 4.29E-05 | 0.000189251 | 0.492107 |
| ENSG00000171867.15 | PRNP | -0.68304 | 5.121098 | -6.84225 | 2.03E-11 | 1.87E-10 | 14.61019 |
| ENSG00000136158.9 | SPRY2 | -0.68318 | 3.431971 | -7.01292 | 6.67E-12 | 6.44E-11 | 15.70045 |
| ENSG00000169515.5 | CCDC8 | -0.68347 | 1.764569 | -6.01572 | 3.21E-09 | 2.38E-08 | 9.657603 |
| ENSG00000104870.11 | FCGRT | -0.68384 | 5.410748 | -6.95824 | 9.55E-12 | 9.08E-11 | 15.34872 |
| ENSG00000126524.8 | SBDS | -0.68386 | 5.57641 | -11.2285 | 1.49E-26 | 4.33E-25 | 49.04365 |
| ENSG00000118507.14 | AKAP7 | -0.68482 | 1.182854 | -12.3893 | 2.31E-31 | 9.06E-30 | 60.0398 |
| ENSG00000008086.9 | CDKL5 | -0.68525 | 1.217659 | -11.8558 | 4.05E-29 | 1.39E-27 | 54.90538 |
| ENSG00000142910.14 | TINAGL1 | -0.68554 | 3.12268 | -5.1137 | 4.33E-07 | 2.51E-06 | 4.898317 |
| ENSG00000105137.11 | SYDE1 | -0.68577 | 2.044173 | -8.03997 | 5.27E-15 | 6.69E-14 | 22.72696 |
| ENSG00000146859.6 | TMEM140 | -0.68594 | 3.60275 | -9.11455 | 1.36E-18 | 2.29E-17 | 30.88315 |
| ENSG00000163453.10 | IGFBP7 | -0.68664 | 6.989828 | -5.87506 | 7.21E-09 | 5.14E-08 | 8.870202 |
| ENSG00000270765.4 | GAS2L2 | -0.68686 | 0.73547 | -6.03035 | 2.95E-09 | 2.19E-08 | 9.740445 |
| ENSG00000100292.15 | HMOX1 | -0.68696 | 4.66111 | -5.34163 | 1.34E-07 | 8.24E-07 | 6.035334 |
| ENSG00000196569.10 | LAMA2 | -0.68718 | 1.889967 | -6.72091 | 4.41E-11 | 3.95E-10 | 13.8489 |
| ENSG00000131196.16 | NFATC1 | -0.68756 | 1.557319 | -9.97343 | 1.08E-21 | 2.27E-20 | 37.94448 |
| ENSG00000132357.12 | CARD6 | -0.68812 | 2.363017 | -7.99305 | 7.42E-15 | 9.30E-14 | 22.38907 |
| ENSG00000093144.17 | ECHDC1 | -0.68853 | 3.531574 | -8.32293 | 6.45E-16 | 8.81E-15 | 24.79782 |
| ENSG00000162148.9 | PPP1R32 | -0.68862 | 1.069599 | -7.24298 | 1.44E-12 | 1.48E-11 | 17.20576 |
| ENSG00000168913.6 | ENHO | -0.68991 | 0.673018 | -7.72364 | 5.16E-14 | 6.01E-13 | 20.47965 |
| ENSG00000189337.14 | KAZN | -0.69024 | 0.846789 | -11.1078 | 4.53E-26 | 1.28E-24 | 47.93815 |
| ENSG00000197057.7 | DTHD1 | -0.69091 | 0.511908 | -8.03458 | 5.48E-15 | 6.94E-14 | 22.68811 |
| ENSG00000164414.15 | SLC35A1 | -0.69096 | 3.214085 | -8.38328 | 4.09E-16 | 5.68E-15 | 25.24674 |
| ENSG00000154359.11 | LONRF1 | -0.69133 | 1.990163 | -10.4954 | 1.14E-23 | 2.75E-22 | 42.45351 |
| ENSG00000076706.13 | MCAM | -0.69229 | 3.627778 | -5.75236 | 1.44E-08 | 9.91E-08 | 8.196834 |
| ENSG00000066027.10 | PPP2R5A | -0.69284 | 4.657398 | -8.44591 | 2.54E-16 | 3.59E-15 | 25.71529 |
| ENSG00000068383.17 | INPP5A | -0.6929 | 2.893196 | -11.3762 | 3.77E-27 | 1.14E-25 | 50.40524 |
| ENSG00000184785.5 | SMIM10 | -0.69297 | 1.876538 | -7.58084 | 1.41E-13 | 1.59E-12 | 19.48903 |
| ENSG00000111801.14 | BTN3A3 | -0.69302 | 2.874731 | -7.50764 | 2.35E-13 | 2.61E-12 | 18.98709 |
| ENSG00000170381.11 | SEMA3E | -0.69366 | 1.148763 | -5.46849 | 6.82E-08 | 4.36E-07 | 6.687635 |
| ENSG00000137070.16 | IL11RA | -0.69428 | 1.750047 | -9.22075 | 5.76E-19 | 9.99E-18 | 31.73115 |
| ENSG00000174684.6 | B4GAT1 | -0.69436 | 3.596446 | -9.28201 | 3.50E-19 | 6.18E-18 | 32.22356 |
| ENSG00000107186.15 | MPDZ | -0.69466 | 1.535652 | -9.36078 | 1.84E-19 | 3.30E-18 | 32.86034 |
| ENSG00000111729.11 | CLEC4A | -0.69481 | 2.314033 | -6.07094 | 2.33E-09 | 1.75E-08 | 9.971219 |
| ENSG00000129993.13 | CBFA2T3 | -0.69489 | 1.060846 | -8.50469 | 1.62E-16 | 2.33E-15 | 26.1575 |
| ENSG00000104324.14 | CPQ | -0.6955 | 3.313578 | -6.64824 | 6.99E-11 | 6.13E-10 | 13.39849 |
| ENSG00000163492.12 | CCDC141 | -0.69557 | 0.186501 | -25.8778 | 4.66E-98 | 3.97E-95 | 213.062 |
| ENSG00000153347.8 | FAM81B | -0.69565 | 1.098134 | -5.27623 | 1.88E-07 | 1.14E-06 | 5.704517 |
| ENSG00000164684.12 | ZNF704 | -0.69567 | 1.727515 | -7.2325 | 1.55E-12 | 1.58E-11 | 17.13629 |
| ENSG00000132259.11 | CNGA4 | -0.69568 | 0.457914 | -8.28061 | 8.86E-16 | 1.20E-14 | 24.48451 |
| ENSG00000100079.6 | LGALS2 | -0.69571 | 2.051017 | -5.12037 | 4.18E-07 | 2.43E-06 | 4.930957 |
| ENSG00000151150.19 | ANK3 | -0.69699 | 1.386338 | -8.15527 | 2.25E-15 | 2.95E-14 | 23.56398 |
| ENSG00000168658.17 | VWA3B | -0.6977 | 0.540408 | -7.50329 | 2.42E-13 | 2.68E-12 | 18.95741 |
| ENSG00000132854.17 | KANK4 | -0.69771 | 0.55963 | -8.83271 | 1.28E-17 | 2.00E-16 | 28.66844 |
| ENSG00000242574.7 | HLA-DMB | -0.69777 | 4.251214 | -4.78583 | 2.18E-06 | 1.15E-05 | 3.342271 |
| ENSG00000091409.13 | ITGA6 | -0.69877 | 3.452503 | -4.63381 | 4.46E-06 | 2.27E-05 | 2.653024 |
| ENSG00000116473.13 | RAP1A | -0.69889 | 3.964305 | -11.8436 | 4.56E-29 | 1.56E-27 | 54.78945 |
| ENSG00000132669.11 | RIN2 | -0.7 | 3.857927 | -9.34341 | 2.12E-19 | 3.79E-18 | 32.71957 |
| ENSG00000077522.11 | ACTN2 | -0.70027 | 0.241514 | -20.5677 | 1.25E-70 | 2.97E-68 | 150.0852 |
| ENSG00000140067.6 | FAM181A | -0.70029 | 0.388783 | -8.8373 | 1.23E-17 | 1.93E-16 | 28.70409 |
| ENSG00000082996.18 | RNF13 | -0.70032 | 4.35839 | -10.829 | 5.76E-25 | 1.52E-23 | 45.41558 |
| ENSG00000135077.7 | HAVCR2 | -0.70073 | 2.830528 | -5.86781 | 7.52E-09 | 5.34E-08 | 8.830074 |
| ENSG00000182700.4 | IGIP | -0.70155 | 2.082668 | -9.22108 | 5.75E-19 | 9.96E-18 | 31.73383 |
| ENSG00000115594.10 | IL1R1 | -0.70171 | 4.358334 | -6.17857 | 1.24E-09 | 9.55E-09 | 10.58968 |
| ENSG00000079335.16 | CDC14A | -0.70176 | 1.312137 | -10.8513 | 4.71E-25 | 1.25E-23 | 45.61612 |
| ENSG00000140057.7 | AK7 | -0.70187 | 0.982141 | -7.48509 | 2.75E-13 | 3.03E-12 | 18.83327 |
| ENSG00000160285.13 | LSS | -0.70288 | 3.221837 | -8.48024 | 1.96E-16 | 2.79E-15 | 25.97327 |
| ENSG00000151552.10 | QDPR | -0.70321 | 3.305904 | -9.75628 | 6.86E-21 | 1.36E-19 | 36.1161 |
| ENSG00000197694.12 | SPTAN1 | -0.70329 | 4.314058 | -8.97761 | 4.06E-18 | 6.62E-17 | 29.80051 |
| ENSG00000158856.16 | DMTN | -0.70348 | 3.067193 | -6.08292 | 2.17E-09 | 1.63E-08 | 10.03957 |
| ENSG00000124780.12 | KCNK17 | -0.70377 | 0.89378 | -6.77658 | 3.09E-11 | 2.81E-10 | 14.19675 |
| ENSG00000214212.7 | C19orf38 | -0.70377 | 1.463847 | -8.4648 | 2.20E-16 | 3.12E-15 | 25.85716 |
| ENSG00000108932.10 | SLC16A6 | -0.70398 | 0.983712 | -8.92192 | 6.32E-18 | 1.02E-16 | 29.36381 |
| ENSG00000068078.16 | FGFR3 | -0.7044 | 2.560887 | -3.6623 | 0.000273384 | 0.001056623 | -1.25787 |
| ENSG00000250510.6 | GPR162 | -0.70448 | 1.223553 | -7.41753 | 4.38E-13 | 4.75E-12 | 18.37465 |
| ENSG00000171227.6 | TMEM37 | -0.70475 | 3.071038 | -5.00338 | 7.53E-07 | 4.22E-06 | 4.364209 |
| ENSG00000213949.7 | ITGA1 | -0.70481 | 2.349392 | -7.2841 | 1.09E-12 | 1.14E-11 | 17.47904 |
| ENSG00000110047.16 | EHD1 | -0.70496 | 3.710474 | -8.27556 | 9.20E-16 | 1.24E-14 | 24.44717 |
| ENSG00000028528.13 | SNX1 | -0.7052 | 3.652623 | -13.3086 | 2.33E-35 | 1.18E-33 | 69.1847 |
| ENSG00000106560.9 | GIMAP2 | -0.70632 | 2.681259 | -6.47644 | 2.04E-10 | 1.71E-09 | 12.35046 |
| ENSG00000133104.11 | SPG20 | -0.7064 | 3.004092 | -10.4752 | 1.37E-23 | 3.27E-22 | 42.27581 |
| ENSG00000166928.9 | MS4A14 | -0.70668 | 0.703306 | -11.0037 | 1.18E-25 | 3.24E-24 | 46.99178 |
| ENSG00000185482.6 | STAC3 | -0.70702 | 1.702876 | -9.59625 | 2.63E-20 | 4.98E-19 | 34.78705 |
| ENSG00000151090.16 | THRB | -0.70713 | 1.086692 | -9.77012 | 6.10E-21 | 1.21E-19 | 36.23175 |
| ENSG00000168995.12 | SIGLEC7 | -0.70818 | 1.310145 | -8.42992 | 2.87E-16 | 4.04E-15 | 25.59536 |
| ENSG00000213145.8 | CRIP1 | -0.709 | 2.090994 | -6.96957 | 8.87E-12 | 8.46E-11 | 15.4214 |
| ENSG00000054983.15 | GALC | -0.70931 | 2.860737 | -9.26057 | 4.17E-19 | 7.32E-18 | 32.05094 |
| ENSG00000104490.16 | NCALD | -0.70953 | 1.830904 | -6.9647 | 9.16E-12 | 8.72E-11 | 15.39017 |
| ENSG00000079156.15 | OSBPL6 | -0.70968 | 0.687591 | -10.1184 | 3.10E-22 | 6.79E-21 | 39.18083 |
| ENSG00000243244.4 | STON1 | -0.71015 | 1.064434 | -11.9102 | 2.41E-29 | 8.35E-28 | 55.42292 |
| ENSG00000239998.4 | LILRA2 | -0.71051 | 0.839566 | -6.81198 | 2.46E-11 | 2.25E-10 | 14.41922 |
| ENSG00000111450.12 | STX2 | -0.7116 | 2.404873 | -9.82849 | 3.72E-21 | 7.54E-20 | 36.72097 |
| ENSG00000100647.7 | SUSD6 | -0.71166 | 4.168664 | -8.98864 | 3.72E-18 | 6.09E-17 | 29.88731 |
| ENSG00000132514.12 | CLEC10A | -0.71192 | 2.003703 | -5.51343 | 5.35E-08 | 3.46E-07 | 6.922007 |
| ENSG00000114013.14 | CD86 | -0.71203 | 2.667629 | -6.42674 | 2.77E-10 | 2.28E-09 | 12.05166 |
| ENSG00000112406.4 | HECA | -0.71235 | 2.969777 | -9.77642 | 5.78E-21 | 1.15E-19 | 36.28444 |
| ENSG00000068305.16 | MEF2A | -0.71285 | 3.029032 | -12.6568 | 1.65E-32 | 6.97E-31 | 62.66346 |
| ENSG00000167083.5 | GNGT2 | -0.71321 | 1.310149 | -10.0036 | 8.35E-22 | 1.77E-20 | 38.20044 |
| ENSG00000140285.8 | FGF7 | -0.71326 | 1.379306 | -7.44738 | 3.57E-13 | 3.89E-12 | 18.57687 |
| ENSG00000169762.15 | TAPT1 | -0.71339 | 2.533599 | -9.09671 | 1.57E-18 | 2.63E-17 | 30.74141 |
| ENSG00000165029.14 | ABCA1 | -0.71402 | 2.536378 | -6.9634 | 9.23E-12 | 8.79E-11 | 15.38183 |
| ENSG00000196843.14 | ARID5A | -0.71426 | 3.070661 | -8.15608 | 2.24E-15 | 2.93E-14 | 23.56987 |
| ENSG00000011028.12 | MRC2 | -0.71443 | 4.203824 | -5.65772 | 2.44E-08 | 1.64E-07 | 7.686077 |
| ENSG00000172638.11 | EFEMP2 | -0.71459 | 3.104999 | -7.02302 | 6.24E-12 | 6.04E-11 | 15.76571 |
| ENSG00000197461.12 | PDGFA | -0.71484 | 2.948958 | -5.89021 | 6.62E-09 | 4.74E-08 | 8.954233 |
| ENSG00000166341.7 | DCHS1 | -0.71558 | 1.753291 | -7.87218 | 1.78E-14 | 2.17E-13 | 21.52595 |
| ENSG00000167779.6 | IGFBP6 | -0.71592 | 2.9909 | -3.96348 | 8.33E-05 | 0.000351774 | -0.13791 |
| ENSG00000143995.18 | MEIS1 | -0.71594 | 1.215547 | -11.039 | 8.52E-26 | 2.36E-24 | 47.31195 |
| ENSG00000102935.10 | ZNF423 | -0.71595 | 0.53954 | -16.4805 | 3.86E-50 | 4.14E-48 | 103.0614 |
| ENSG00000133169.5 | BEX1 | -0.71599 | 0.698986 | -4.47863 | 9.09E-06 | 4.42E-05 | 1.970787 |
| ENSG00000127863.14 | TNFRSF19 | -0.71656 | 2.614727 | -4.92467 | 1.11E-06 | 6.10E-06 | 3.989624 |
| ENSG00000084453.15 | SLCO1A2 | -0.71679 | 0.220008 | -14.131 | 4.69E-39 | 2.91E-37 | 77.65179 |
| ENSG00000179921.13 | GPBAR1 | -0.71688 | 0.525301 | -16.7269 | 2.44E-51 | 2.77E-49 | 105.8124 |
| ENSG00000154553.12 | PDLIM3 | -0.71739 | 2.10082 | -6.48884 | 1.89E-10 | 1.58E-09 | 12.42533 |
| ENSG00000142494.12 | SLC47A1 | -0.71753 | 1.567464 | -5.51311 | 5.36E-08 | 3.47E-07 | 6.920321 |
| ENSG00000198774.4 | RASSF9 | -0.71816 | 1.350979 | -6.40015 | 3.26E-10 | 2.67E-09 | 11.89264 |
| ENSG00000144711.12 | IQSEC1 | -0.7186 | 3.270061 | -9.5564 | 3.66E-20 | 6.87E-19 | 34.45849 |
| ENSG00000104219.11 | ZDHHC2 | -0.71928 | 2.766481 | -7.48354 | 2.78E-13 | 3.06E-12 | 18.82269 |
| ENSG00000112303.12 | VNN2 | -0.71943 | 1.580777 | -6.15933 | 1.39E-09 | 1.06E-08 | 10.47843 |
| ENSG00000106771.11 | TMEM245 | -0.71971 | 3.621484 | -9.08857 | 1.67E-18 | 2.81E-17 | 30.67687 |
| ENSG00000137880.5 | GCHFR | -0.71988 | 2.627897 | -7.96527 | 9.08E-15 | 1.13E-13 | 22.18977 |
| ENSG00000230062.5 | ANKRD66 | -0.71993 | 0.349917 | -9.45436 | 8.52E-20 | 1.56E-18 | 33.62187 |
| ENSG00000080572.11 | PIH1D3 | -0.72006 | 0.388306 | -8.74774 | 2.49E-17 | 3.80E-16 | 28.01101 |
| ENSG00000118508.4 | RAB32 | -0.72031 | 4.190679 | -6.60727 | 9.04E-11 | 7.82E-10 | 13.14643 |
| ENSG00000010278.10 | CD9 | -0.72058 | 6.669223 | -6.37378 | 3.83E-10 | 3.12E-09 | 11.73548 |
| ENSG00000069431.9 | ABCC9 | -0.72087 | 1.16228 | -8.76888 | 2.11E-17 | 3.25E-16 | 28.17415 |
| ENSG00000179399.12 | GPC5 | -0.7215 | 0.495354 | -8.94564 | 5.24E-18 | 8.48E-17 | 29.54957 |
| ENSG00000053254.14 | FOXN3 | -0.72288 | 2.649978 | -9.21316 | 6.13E-19 | 1.06E-17 | 31.67029 |
| ENSG00000136141.13 | LRCH1 | -0.72347 | 1.986098 | -11.3819 | 3.57E-27 | 1.08E-25 | 50.45849 |
| ENSG00000100450.11 | GZMH | -0.72369 | 2.293417 | -4.85863 | 1.53E-06 | 8.25E-06 | 3.679597 |
| ENSG00000146221.9 | TCTE1 | -0.72395 | 0.383376 | -9.08956 | 1.66E-18 | 2.78E-17 | 30.68465 |
| ENSG00000163947.10 | ARHGEF3 | -0.72489 | 2.810072 | -11.6638 | 2.52E-28 | 8.28E-27 | 53.08975 |
| ENSG00000175745.10 | NR2F1 | -0.72517 | 2.939902 | -6.09007 | 2.08E-09 | 1.57E-08 | 10.08045 |
| ENSG00000169282.16 | KCNAB1 | -0.72551 | 0.55403 | -19.8722 | 4.49E-67 | 9.62E-65 | 141.9222 |
| ENSG00000104695.11 | PPP2CB | -0.72561 | 4.389746 | -10.8806 | 3.61E-25 | 9.68E-24 | 45.87927 |
| ENSG00000137473.16 | TTC29 | -0.72577 | 0.412505 | -8.50891 | 1.57E-16 | 2.25E-15 | 26.18935 |
| ENSG00000139132.13 | FGD4 | -0.72632 | 1.808424 | -10.0742 | 4.55E-22 | 9.82E-21 | 38.80226 |
| ENSG00000156206.12 | C15orf26 | -0.72632 | 0.3947 | -10.4976 | 1.12E-23 | 2.70E-22 | 42.47266 |
| ENSG00000114353.15 | GNAI2 | -0.72681 | 5.440827 | -10.3613 | 3.73E-23 | 8.66E-22 | 41.27992 |
| ENSG00000180694.12 | TMEM64 | -0.727 | 2.443214 | -7.04839 | 5.28E-12 | 5.14E-11 | 15.92993 |
| ENSG00000144802.10 | NFKBIZ | -0.72746 | 3.171838 | -5.31563 | 1.53E-07 | 9.40E-07 | 5.903366 |
| ENSG00000135374.8 | ELF5 | -0.72754 | 1.444631 | -4.75349 | 2.54E-06 | 1.33E-05 | 3.193908 |
| ENSG00000144815.13 | NXPE3 | -0.72793 | 1.61856 | -9.89881 | 2.05E-21 | 4.22E-20 | 37.31298 |
| ENSG00000174749.5 | C4orf32 | -0.72821 | 1.341205 | -13.063 | 2.82E-34 | 1.33E-32 | 66.70735 |
| ENSG00000134216.17 | CHIA | -0.72822 | 1.1951 | -4.15709 | 3.72E-05 | 0.000165787 | 0.626242 |
| ENSG00000151023.15 | ENKUR | -0.72829 | 0.773429 | -6.58712 | 1.03E-10 | 8.82E-10 | 13.02295 |
| ENSG00000204257.13 | HLA-DMA | -0.72834 | 5.899151 | -5.04531 | 6.11E-07 | 3.47E-06 | 4.565931 |
| ENSG00000233608.3 | TWIST2 | -0.72844 | 1.116457 | -8.46592 | 2.18E-16 | 3.10E-15 | 25.86557 |
| ENSG00000183580.9 | FBXL7 | -0.72939 | 1.488893 | -8.77357 | 2.03E-17 | 3.13E-16 | 28.21037 |
| ENSG00000122986.12 | HVCN1 | -0.73054 | 1.823295 | -9.55399 | 3.73E-20 | 7.00E-19 | 34.43868 |
| ENSG00000111144.8 | LTA4H | -0.73142 | 4.968575 | -7.9147 | 1.31E-14 | 1.61E-13 | 21.82838 |
| ENSG00000150893.10 | FREM2 | -0.7315 | 0.96914 | -7.74097 | 4.56E-14 | 5.34E-13 | 20.60089 |
| ENSG00000100243.19 | CYB5R3 | -0.73154 | 5.313302 | -9.77163 | 6.02E-21 | 1.20E-19 | 36.24439 |
| ENSG00000109686.15 | SH3D19 | -0.73244 | 3.339371 | -10.4016 | 2.62E-23 | 6.13E-22 | 41.63166 |
| ENSG00000204815.7 | TTC25 | -0.73253 | 1.294748 | -6.37572 | 3.78E-10 | 3.08E-09 | 11.74705 |
| ENSG00000163378.12 | EOGT | -0.73257 | 2.471383 | -11.487 | 1.34E-27 | 4.14E-26 | 51.43491 |
| ENSG00000119139.15 | TJP2 | -0.7326 | 3.639584 | -8.91537 | 6.66E-18 | 1.07E-16 | 29.31257 |
| ENSG00000184481.15 | FOXO4 | -0.73319 | 2.67385 | -9.85726 | 2.91E-21 | 5.95E-20 | 36.96281 |
| ENSG00000069667.14 | RORA | -0.73343 | 1.109633 | -11.7375 | 1.25E-28 | 4.18E-27 | 53.78424 |
| ENSG00000137462.6 | TLR2 | -0.73459 | 3.394083 | -5.1834 | 3.04E-07 | 1.79E-06 | 5.241249 |
| ENSG00000030582.15 | GRN | -0.73631 | 7.527435 | -8.71543 | 3.20E-17 | 4.84E-16 | 27.76235 |
| ENSG00000188396.3 | TCTEX1D4 | -0.73678 | 0.641856 | -8.66724 | 4.65E-17 | 6.94E-16 | 27.39269 |
| ENSG00000007174.16 | DNAH9 | -0.73692 | 0.396605 | -8.05481 | 4.72E-15 | 6.02E-14 | 22.83419 |
| ENSG00000123360.10 | PDE1B | -0.73721 | 0.712182 | -12.9525 | 8.58E-34 | 3.93E-32 | 65.5998 |
| ENSG00000162444.11 | RBP7 | -0.7395 | 1.878712 | -6.34512 | 4.55E-10 | 3.68E-09 | 11.5653 |
| ENSG00000182916.7 | TCEAL7 | -0.7401 | 1.120111 | -10.0094 | 7.95E-22 | 1.68E-20 | 38.24971 |
| ENSG00000090674.14 | MCOLN1 | -0.74033 | 2.977198 | -11.2704 | 1.01E-26 | 2.97E-25 | 49.42845 |
| ENSG00000126895.12 | AVPR2 | -0.7408 | 0.434603 | -13.5098 | 2.98E-36 | 1.60E-34 | 71.23216 |
| ENSG00000137076.17 | TLN1 | -0.74149 | 5.286364 | -8.91078 | 6.90E-18 | 1.11E-16 | 29.27666 |
| ENSG00000091181.18 | IL5RA | -0.74188 | 0.4151 | -11.1137 | 4.29E-26 | 1.21E-24 | 47.99191 |
| ENSG00000140749.8 | IGSF6 | -0.74223 | 2.878857 | -5.75232 | 1.44E-08 | 9.91E-08 | 8.196618 |
| ENSG00000151892.13 | GFRA1 | -0.74335 | 0.585783 | -9.71744 | 9.51E-21 | 1.86E-19 | 35.79208 |
| ENSG00000159840.14 | ZYX | -0.74378 | 5.768602 | -7.80729 | 2.84E-14 | 3.39E-13 | 21.06689 |
| ENSG00000184304.13 | PRKD1 | -0.74381 | 1.529416 | -8.12818 | 2.75E-15 | 3.58E-14 | 23.36644 |
| ENSG00000172361.5 | CFAP53 | -0.74448 | 1.194064 | -6.38371 | 3.60E-10 | 2.94E-09 | 11.79462 |
| ENSG00000112139.13 | MDGA1 | -0.74505 | 0.580365 | -14.8676 | 1.87E-42 | 1.41E-40 | 85.44025 |
| ENSG00000076356.6 | PLXNA2 | -0.74536 | 2.508622 | -6.14487 | 1.51E-09 | 1.15E-08 | 10.395 |
| ENSG00000172987.11 | HPSE2 | -0.74614 | 0.332004 | -14.8475 | 2.32E-42 | 1.74E-40 | 85.22457 |
| ENSG00000121858.9 | TNFSF10 | -0.7463 | 5.074514 | -4.82873 | 1.77E-06 | 9.48E-06 | 3.540459 |
| ENSG00000198835.3 | GJC2 | -0.74668 | 0.856435 | -10.8877 | 3.38E-25 | 9.10E-24 | 45.9434 |
| ENSG00000196209.11 | SIRPB2 | -0.74702 | 1.155012 | -10.1269 | 2.89E-22 | 6.33E-21 | 39.25355 |
| ENSG00000089041.15 | P2RX7 | -0.74791 | 1.484913 | -8.41396 | 3.24E-16 | 4.54E-15 | 25.47587 |
| ENSG00000172752.13 | COL6A5 | -0.74792 | 0.610362 | -11.2866 | 8.68E-27 | 2.57E-25 | 49.57758 |
| ENSG00000164236.10 | ANKRD33B | -0.74883 | 0.555148 | -12.7537 | 6.28E-33 | 2.74E-31 | 63.62155 |
| ENSG00000115363.12 | EVA1A | -0.74917 | 3.209662 | -5.17387 | 3.19E-07 | 1.88E-06 | 5.19408 |
| ENSG00000148053.14 | NTRK2 | -0.74927 | 0.381634 | -9.74379 | 7.62E-21 | 1.50E-19 | 36.0118 |
| ENSG00000076555.14 | ACACB | -0.74978 | 1.016949 | -12.693 | 1.15E-32 | 4.89E-31 | 63.02163 |
| ENSG00000151474.18 | FRMD4A | -0.75003 | 1.04617 | -15.167 | 7.41E-44 | 5.92E-42 | 88.65514 |
| ENSG00000068831.17 | RASGRP2 | -0.75032 | 1.131787 | -8.78012 | 1.93E-17 | 2.98E-16 | 28.26094 |
| ENSG00000154309.8 | DISP1 | -0.75034 | 1.87996 | -9.21433 | 6.07E-19 | 1.05E-17 | 31.67967 |
| ENSG00000011105.10 | TSPAN9 | -0.75055 | 3.281054 | -8.24148 | 1.19E-15 | 1.59E-14 | 24.19593 |
| ENSG00000138613.12 | APH1B | -0.7506 | 1.964501 | -10.4106 | 2.42E-23 | 5.68E-22 | 41.7096 |
| ENSG00000198523.5 | PLN | -0.75182 | 2.031578 | -5.44036 | 7.92E-08 | 5.03E-07 | 6.54181 |
| ENSG00000143878.9 | RHOB | -0.75244 | 6.996161 | -5.47972 | 6.42E-08 | 4.11E-07 | 6.746038 |
| ENSG00000088002.10 | SULT2B1 | -0.75321 | 2.160835 | -5.15081 | 3.59E-07 | 2.10E-06 | 5.080349 |
| ENSG00000189241.6 | TSPYL1 | -0.7534 | 4.479375 | -11.1034 | 4.72E-26 | 1.33E-24 | 47.89801 |
| ENSG00000140030.5 | GPR65 | -0.7534 | 1.286125 | -9.76275 | 6.49E-21 | 1.29E-19 | 36.17014 |
| ENSG00000144285.14 | SCN1A | -0.75358 | 0.422902 | -13.7086 | 3.83E-37 | 2.15E-35 | 73.27095 |
| ENSG00000085063.13 | CD59 | -0.75411 | 6.292359 | -7.07569 | 4.41E-12 | 4.33E-11 | 16.10715 |
| ENSG00000185008.16 | ROBO2 | -0.75506 | 0.548599 | -12.2067 | 1.37E-30 | 5.14E-29 | 58.26724 |
| ENSG00000133687.14 | TMTC1 | -0.75536 | 0.910478 | -8.01477 | 6.33E-15 | 7.98E-14 | 22.5453 |
| ENSG00000119986.6 | AVPI1 | -0.75611 | 4.01699 | -5.65266 | 2.51E-08 | 1.68E-07 | 7.659018 |
| ENSG00000140299.10 | BNIP2 | -0.75632 | 3.094533 | -13.6484 | 7.14E-37 | 3.96E-35 | 72.65223 |
| ENSG00000116117.16 | PARD3B | -0.75663 | 1.371752 | -11.003 | 1.18E-25 | 3.26E-24 | 46.98572 |
| ENSG00000221869.4 | CEBPD | -0.75673 | 5.450375 | -5.45324 | 7.40E-08 | 4.71E-07 | 6.608469 |
| ENSG00000265190.5 | ANXA8 | -0.75679 | 0.47624 | -7.84115 | 2.23E-14 | 2.69E-13 | 21.30606 |
| ENSG00000172348.13 | RCAN2 | -0.75688 | 2.52483 | -5.51165 | 5.40E-08 | 3.49E-07 | 6.912657 |
| ENSG00000150337.12 | FCGR1A | -0.75697 | 1.715936 | -7.44936 | 3.52E-13 | 3.84E-12 | 18.59033 |
| ENSG00000147231.12 | CXorf57 | -0.75705 | 0.618005 | -12.8186 | 3.28E-33 | 1.45E-31 | 64.26607 |
| ENSG00000136156.11 | ITM2B | -0.75725 | 5.903819 | -9.3227 | 2.51E-19 | 4.46E-18 | 32.55201 |
| ENSG00000131378.12 | RFTN1 | -0.75727 | 3.846197 | -6.26141 | 7.54E-10 | 5.95E-09 | 11.07215 |
| ENSG00000176533.11 | GNG7 | -0.75772 | 1.506688 | -8.87555 | 9.12E-18 | 1.45E-16 | 29.00166 |
| ENSG00000158122.10 | AAED1 | -0.75795 | 2.690847 | -11.8401 | 4.71E-29 | 1.61E-27 | 54.75566 |
| ENSG00000119471.13 | HSDL2 | -0.7583 | 3.942492 | -9.57873 | 3.04E-20 | 5.74E-19 | 34.64247 |
| ENSG00000187720.13 | THSD4 | -0.75844 | 1.258549 | -9.64438 | 1.76E-20 | 3.35E-19 | 35.18512 |
| ENSG00000103365.14 | GGA2 | -0.75866 | 3.970485 | -11.2818 | 9.07E-27 | 2.68E-25 | 49.53359 |
| ENSG00000141480.16 | ARRB2 | -0.7598 | 3.849104 | -10.5241 | 8.85E-24 | 2.15E-22 | 42.70605 |
| ENSG00000124019.9 | FAM124B | -0.75983 | 0.597636 | -16.2985 | 2.94E-49 | 3.06E-47 | 101.0384 |
| ENSG00000197165.9 | SULT1A2 | -0.76036 | 1.547028 | -7.09497 | 3.88E-12 | 3.82E-11 | 16.23268 |
| ENSG00000134352.18 | IL6ST | -0.761 | 4.597191 | -7.81798 | 2.63E-14 | 3.15E-13 | 21.14226 |
| ENSG00000197467.12 | COL13A1 | -0.76165 | 1.058876 | -9.53808 | 4.26E-20 | 7.97E-19 | 34.30784 |
| ENSG00000120306.8 | CYSTM1 | -0.76185 | 4.862187 | -7.05476 | 5.06E-12 | 4.94E-11 | 15.97121 |
| ENSG00000134516.14 | DOCK2 | -0.76205 | 1.871764 | -7.74493 | 4.43E-14 | 5.19E-13 | 20.62863 |
| ENSG00000130988.11 | RGN | -0.76239 | 1.570255 | -7.27431 | 1.17E-12 | 1.21E-11 | 17.41382 |
| ENSG00000152484.12 | USP12 | -0.76258 | 3.066545 | -12.5121 | 6.89E-32 | 2.80E-30 | 61.24038 |
| ENSG00000057704.9 | TMCC3 | -0.76325 | 1.693298 | -8.87078 | 9.47E-18 | 1.50E-16 | 28.9645 |
| ENSG00000159433.10 | STARD9 | -0.76333 | 0.612308 | -15.1794 | 6.48E-44 | 5.20E-42 | 88.78874 |
| ENSG00000112367.9 | FIG4 | -0.7635 | 2.78999 | -13.2221 | 5.62E-35 | 2.76E-33 | 68.3096 |
| ENSG00000147202.16 | DIAPH2 | -0.76399 | 1.858286 | -11.519 | 9.89E-28 | 3.10E-26 | 51.73372 |
| ENSG00000177191.2 | B3GNT8 | -0.764 | 3.154913 | -4.72217 | 2.95E-06 | 1.53E-05 | 3.051116 |
| ENSG00000197748.11 | CFAP43 | -0.76505 | 0.774914 | -6.73255 | 4.10E-11 | 3.68E-10 | 13.92144 |
| ENSG00000109436.7 | TBC1D9 | -0.76521 | 3.231136 | -9.574 | 3.16E-20 | 5.96E-19 | 34.60351 |
| ENSG00000145335.14 | SNCA | -0.76524 | 0.786453 | -12.4049 | 1.98E-31 | 7.82E-30 | 60.19192 |
| ENSG00000104369.4 | JPH1 | -0.76568 | 1.79713 | -6.33552 | 4.83E-10 | 3.88E-09 | 11.50846 |
| ENSG00000144909.7 | OSBPL11 | -0.76591 | 2.854348 | -13.3711 | 1.23E-35 | 6.35E-34 | 69.8191 |
| ENSG00000180730.4 | SHISA2 | -0.7662 | 1.923163 | -3.86211 | 0.000125382 | 0.000513786 | -0.52423 |
| ENSG00000119782.12 | FKBP1B | -0.76621 | 1.706766 | -7.44955 | 3.52E-13 | 3.84E-12 | 18.59157 |
| ENSG00000177272.8 | KCNA3 | -0.7666 | 1.341345 | -6.91798 | 1.24E-11 | 1.17E-10 | 15.09115 |
| ENSG00000127951.5 | FGL2 | -0.76694 | 3.264113 | -5.30369 | 1.63E-07 | 9.97E-07 | 5.842961 |
| ENSG00000132274.14 | TRIM22 | -0.76696 | 3.748077 | -6.35004 | 4.42E-10 | 3.57E-09 | 11.59449 |
| ENSG00000176834.12 | VSIG10 | -0.76735 | 2.062268 | -8.64221 | 5.64E-17 | 8.36E-16 | 27.20128 |
| ENSG00000058091.15 | CDK14 | -0.7681 | 2.011603 | -8.1963 | 1.66E-15 | 2.20E-14 | 23.86407 |
| ENSG00000169715.13 | MT1E | -0.76833 | 4.624543 | -3.95352 | 8.68E-05 | 0.000365217 | -0.17629 |
| ENSG00000105519.11 | CAPS | -0.76833 | 3.166515 | -4.2872 | 2.13E-05 | 9.81E-05 | 1.159019 |
| ENSG00000121440.13 | PDZRN3 | -0.76856 | 1.396412 | -9.19369 | 7.18E-19 | 1.23E-17 | 31.51435 |
| ENSG00000112320.10 | SOBP | -0.76882 | 0.729043 | -11.7747 | 8.79E-29 | 2.97E-27 | 54.13646 |
| ENSG00000176463.12 | SLCO3A1 | -0.76911 | 2.221371 | -8.19191 | 1.72E-15 | 2.27E-14 | 23.83193 |
| ENSG00000198759.10 | EGFL6 | -0.76987 | 2.675507 | -6.63156 | 7.76E-11 | 6.76E-10 | 13.29574 |
| ENSG00000147872.8 | PLIN2 | -0.77021 | 3.930972 | -5.83984 | 8.81E-09 | 6.22E-08 | 8.675656 |
| ENSG00000035862.11 | TIMP2 | -0.77155 | 5.707834 | -6.14275 | 1.53E-09 | 1.17E-08 | 10.38279 |
| ENSG00000197852.9 | FAM212B | -0.77185 | 0.767159 | -17.8562 | 6.60E-57 | 9.61E-55 | 118.5872 |
| ENSG00000099864.16 | PALM | -0.77203 | 2.158267 | -7.37124 | 6.02E-13 | 6.44E-12 | 18.06242 |
| ENSG00000188176.10 | SMTNL2 | -0.77206 | 0.611838 | -11.7466 | 1.15E-28 | 3.84E-27 | 53.87071 |
| ENSG00000161638.9 | ITGA5 | -0.77289 | 3.717435 | -6.04154 | 2.77E-09 | 2.06E-08 | 9.803946 |
| ENSG00000104067.15 | TJP1 | -0.77299 | 3.536093 | -10.4503 | 1.70E-23 | 4.07E-22 | 42.05718 |
| ENSG00000179044.14 | EXOC3L1 | -0.77323 | 1.465495 | -12.3036 | 5.34E-31 | 2.04E-29 | 59.20629 |
| ENSG00000170340.10 | B3GNT2 | -0.7733 | 3.901722 | -10.4986 | 1.11E-23 | 2.68E-22 | 42.48132 |
| ENSG00000171488.13 | LRRC8C | -0.77413 | 1.447613 | -12.1156 | 3.32E-30 | 1.21E-28 | 57.3895 |
| ENSG00000185250.14 | PPIL6 | -0.77425 | 1.092014 | -8.07766 | 3.99E-15 | 5.12E-14 | 22.99955 |
| ENSG00000102921.6 | N4BP1 | -0.77433 | 3.309678 | -13.4642 | 4.75E-36 | 2.50E-34 | 70.76718 |
| ENSG00000244482.8 | LILRA6 | -0.77476 | 0.988256 | -10.4372 | 1.91E-23 | 4.54E-22 | 41.94249 |
| ENSG00000128923.9 | FAM63B | -0.77569 | 2.093703 | -12.7149 | 9.24E-33 | 3.96E-31 | 63.23766 |
| ENSG00000066735.13 | KIF26A | -0.77578 | 0.719305 | -10.2074 | 1.43E-22 | 3.22E-21 | 39.94615 |
| ENSG00000086730.15 | LAT2 | -0.77585 | 2.38307 | -7.80032 | 2.99E-14 | 3.56E-13 | 21.01775 |
| ENSG00000181754.6 | AMIGO1 | -0.77612 | 1.661787 | -9.90701 | 1.91E-21 | 3.95E-20 | 37.38217 |
| ENSG00000170190.14 | SLC16A5 | -0.77738 | 3.242907 | -6.24538 | 8.30E-10 | 6.52E-09 | 10.97832 |
| ENSG00000257108.1 | NHLRC4 | -0.77741 | 0.876294 | -8.91141 | 6.87E-18 | 1.10E-16 | 29.28155 |
| ENSG00000111666.9 | CHPT1 | -0.77782 | 2.733672 | -7.24897 | 1.38E-12 | 1.43E-11 | 17.24549 |
| ENSG00000171860.4 | C3AR1 | -0.77837 | 3.397421 | -5.75315 | 1.43E-08 | 9.87E-08 | 8.20111 |
| ENSG00000148400.9 | NOTCH1 | -0.77863 | 2.728625 | -8.40202 | 3.55E-16 | 4.96E-15 | 25.38661 |
| ENSG00000067064.9 | IDI1 | -0.78011 | 4.205982 | -8.30648 | 7.29E-16 | 9.91E-15 | 24.67584 |
| ENSG00000140678.15 | ITGAX | -0.78121 | 2.778496 | -6.32799 | 5.05E-10 | 4.06E-09 | 11.46396 |
| ENSG00000163219.10 | ARHGAP25 | -0.78187 | 2.210541 | -9.07926 | 1.80E-18 | 3.02E-17 | 30.60298 |
| ENSG00000181631.6 | P2RY13 | -0.78221 | 1.727189 | -7.27523 | 1.16E-12 | 1.20E-11 | 17.41995 |
| ENSG00000198786.2 | MT-ND5 | -0.78354 | 10.09222 | -5.22829 | 2.41E-07 | 1.44E-06 | 5.464312 |
| ENSG00000106123.10 | EPHB6 | -0.78425 | 1.064195 | -7.55641 | 1.67E-13 | 1.88E-12 | 19.32106 |
| ENSG00000126351.11 | THRA | -0.78429 | 2.821861 | -9.80524 | 4.53E-21 | 9.12E-20 | 36.52587 |
| ENSG00000182255.6 | KCNA4 | -0.78617 | 0.14798 | -22.2218 | 3.79E-79 | 1.31E-76 | 169.6403 |
| ENSG00000090376.7 | IRAK3 | -0.78707 | 1.872511 | -6.74048 | 3.89E-11 | 3.50E-10 | 13.97092 |
| ENSG00000168646.11 | AXIN2 | -0.78741 | 1.955698 | -7.0214 | 6.31E-12 | 6.10E-11 | 15.75522 |
| ENSG00000088387.16 | DOCK9 | -0.78806 | 2.865102 | -7.93919 | 1.10E-14 | 1.36E-13 | 22.00313 |
| ENSG00000158517.12 | NCF1 | -0.78812 | 1.303145 | -9.00463 | 3.28E-18 | 5.38E-17 | 30.0132 |
| ENSG00000104728.14 | ARHGEF10 | -0.78868 | 1.929887 | -10.0553 | 5.35E-22 | 1.15E-20 | 38.64122 |
| ENSG00000163909.7 | HEYL | -0.78977 | 2.697374 | -8.12515 | 2.81E-15 | 3.65E-14 | 23.34442 |
| ENSG00000176438.11 | SYNE3 | -0.79014 | 0.671172 | -16.4422 | 5.93E-50 | 6.30E-48 | 102.6349 |
| ENSG00000170276.5 | HSPB2 | -0.7904 | 0.660524 | -17.153 | 1.99E-53 | 2.44E-51 | 110.6024 |
| ENSG00000185561.9 | TLCD2 | -0.79169 | 1.797929 | -9.74891 | 7.30E-21 | 1.44E-19 | 36.05451 |
| ENSG00000171444.16 | MCC | -0.79185 | 1.175715 | -11.1189 | 4.09E-26 | 1.16E-24 | 48.03963 |
| ENSG00000163823.3 | CCR1 | -0.792 | 2.933186 | -6.35284 | 4.34E-10 | 3.52E-09 | 11.61112 |
| ENSG00000138801.7 | PAPSS1 | -0.79242 | 4.295021 | -13.4377 | 6.23E-36 | 3.26E-34 | 70.49691 |
| ENSG00000196260.3 | SFTA2 | -0.79274 | 7.075333 | -3.07489 | 0.002207263 | 0.007145634 | -3.19818 |
| ENSG00000129244.7 | ATP1B2 | -0.79311 | 0.779084 | -12.3927 | 2.23E-31 | 8.79E-30 | 60.07284 |
| ENSG00000168386.17 | FILIP1L | -0.79332 | 3.120948 | -6.34672 | 4.51E-10 | 3.65E-09 | 11.57481 |
| ENSG00000122735.14 | DNAI1 | -0.79386 | 0.631337 | -7.16807 | 2.38E-12 | 2.39E-11 | 16.71116 |
| ENSG00000119922.8 | IFIT2 | -0.79417 | 2.826381 | -6.67473 | 5.91E-11 | 5.22E-10 | 13.56219 |
| ENSG00000106537.7 | TSPAN13 | -0.79456 | 6.688446 | -7.24442 | 1.43E-12 | 1.47E-11 | 17.21526 |
| ENSG00000137824.14 | RMDN3 | -0.79481 | 3.534278 | -14.3272 | 5.93E-40 | 3.81E-38 | 79.7088 |
| ENSG00000159588.13 | CCDC17 | -0.79488 | 1.123576 | -5.6854 | 2.09E-08 | 1.42E-07 | 7.834675 |
| ENSG00000137941.15 | TTLL7 | -0.7962 | 0.677938 | -10.8995 | 3.04E-25 | 8.19E-24 | 46.04972 |
| ENSG00000137672.11 | TRPC6 | -0.79639 | 1.925937 | -5.63284 | 2.79E-08 | 1.86E-07 | 7.553076 |
| ENSG00000197446.7 | CYP2F1 | -0.79701 | 0.402648 | -8.85719 | 1.05E-17 | 1.66E-16 | 28.85868 |
| ENSG00000182287.12 | AP1S2 | -0.79719 | 2.319279 | -8.95969 | 4.69E-18 | 7.61E-17 | 29.65981 |
| ENSG00000143226.12 | FCGR2A | -0.79773 | 3.632692 | -6.54069 | 1.37E-10 | 1.17E-09 | 12.73968 |
| ENSG00000215595.1 | C20orf202 | -0.79816 | 0.342763 | -26.2371 | 6.56E-100 | 5.92E-97 | 217.3148 |
| ENSG00000110651.10 | CD81 | -0.79843 | 6.190351 | -9.88193 | 2.36E-21 | 4.85E-20 | 37.17058 |
| ENSG00000172716.15 | SLFN11 | -0.79862 | 3.045983 | -6.67698 | 5.83E-11 | 5.15E-10 | 13.57613 |
| ENSG00000196352.12 | CD55 | -0.79992 | 6.404104 | -5.06598 | 5.51E-07 | 3.15E-06 | 4.665946 |
| ENSG00000169379.14 | ARL13B | -0.79999 | 2.034393 | -13.4781 | 4.12E-36 | 2.18E-34 | 70.9092 |
| ENSG00000137575.10 | SDCBP | -0.80051 | 5.551214 | -11.0743 | 6.16E-26 | 1.72E-24 | 47.63305 |
| ENSG00000129473.8 | BCL2L2 | -0.8025 | 3.485737 | -11.2055 | 1.84E-26 | 5.34E-25 | 48.83208 |
| ENSG00000174004.5 | NRROS | -0.80297 | 2.002839 | -8.74512 | 2.54E-17 | 3.87E-16 | 27.99083 |
| ENSG00000074416.12 | MGLL | -0.80317 | 4.253876 | -5.93272 | 5.19E-09 | 3.75E-08 | 9.191027 |
| ENSG00000277758.3 | SYT15 | -0.80344 | 0.270425 | -23.8058 | 2.41E-87 | 1.25E-84 | 188.4602 |
| ENSG00000149798.4 | CDC42EP2 | -0.80441 | 2.746511 | -8.01465 | 6.34E-15 | 7.99E-14 | 22.54444 |
| ENSG00000124831.17 | LRRFIP1 | -0.80463 | 3.803308 | -12.4667 | 1.08E-31 | 4.33E-30 | 60.79562 |
| ENSG00000070193.4 | FGF10 | -0.8048 | 0.232137 | -20.0113 | 8.75E-68 | 1.93E-65 | 143.5513 |
| ENSG00000143768.10 | LEFTY2 | -0.80481 | 0.521008 | -11.599 | 4.66E-28 | 1.49E-26 | 52.48116 |
| ENSG00000136237.17 | RAPGEF5 | -0.805 | 2.481801 | -8.23473 | 1.25E-15 | 1.67E-14 | 24.14624 |
| ENSG00000184005.10 | ST6GALNAC3 | -0.80522 | 0.531897 | -17.1737 | 1.58E-53 | 1.94E-51 | 110.8365 |
| ENSG00000150045.10 | KLRF1 | -0.8061 | 0.825802 | -9.98494 | 9.80E-22 | 2.06E-20 | 38.04214 |
| ENSG00000003436.13 | TFPI | -0.80641 | 3.667096 | -4.83724 | 1.70E-06 | 9.11E-06 | 3.579973 |
| ENSG00000169271.2 | HSPB3 | -0.80642 | 0.350924 | -15.9088 | 2.22E-47 | 2.13E-45 | 96.7346 |
| ENSG00000153234.12 | NR4A2 | -0.80685 | 2.827458 | -4.70888 | 3.14E-06 | 1.62E-05 | 2.990821 |
| ENSG00000071242.10 | RPS6KA2 | -0.8071 | 3.903311 | -6.85302 | 1.89E-11 | 1.75E-10 | 14.67832 |
| ENSG00000133665.11 | DYDC2 | -0.80767 | 0.688391 | -8.03677 | 5.39E-15 | 6.84E-14 | 22.7039 |
| ENSG00000080293.8 | SCTR | -0.80778 | 2.16337 | -3.99956 | 7.19E-05 | 0.000306493 | 0.001899 |
| ENSG00000185924.6 | RTN4RL1 | -0.80921 | 1.045038 | -7.37647 | 5.81E-13 | 6.22E-12 | 18.09764 |
| ENSG00000160013.7 | PTGIR | -0.8094 | 1.31341 | -11.9365 | 1.87E-29 | 6.55E-28 | 55.67334 |
| ENSG00000166275.14 | C10orf32 | -0.81002 | 3.272834 | -11.5647 | 6.43E-28 | 2.04E-26 | 52.16065 |
| ENSG00000256043.2 | CTSO | -0.81035 | 4.202425 | -8.19602 | 1.67E-15 | 2.20E-14 | 23.86202 |
| ENSG00000130433.6 | CACNG6 | -0.81066 | 0.632747 | -6.60672 | 9.07E-11 | 7.85E-10 | 13.14309 |
| ENSG00000073756.10 | PTGS2 | -0.81136 | 3.450687 | -3.4106 | 0.000694379 | 0.002485683 | -2.12907 |
| ENSG00000163697.15 | APBB2 | -0.81136 | 2.011312 | -11.2577 | 1.14E-26 | 3.33E-25 | 49.31141 |
| ENSG00000111885.6 | MAN1A1 | -0.81147 | 3.830746 | -7.09602 | 3.85E-12 | 3.80E-11 | 16.23951 |
| ENSG00000186088.14 | GSAP | -0.81166 | 2.254218 | -9.45677 | 8.35E-20 | 1.53E-18 | 33.64157 |
| ENSG00000183378.10 | OVCH2 | -0.81207 | 0.23789 | -21.183 | 8.63E-74 | 2.36E-71 | 157.34 |
| ENSG00000139174.9 | PRICKLE1 | -0.81213 | 0.918939 | -12.6002 | 2.89E-32 | 1.20E-30 | 62.10597 |
| ENSG00000136111.11 | TBC1D4 | -0.81262 | 2.304434 | -10.2317 | 1.16E-22 | 2.62E-21 | 40.15593 |
| ENSG00000111684.9 | LPCAT3 | -0.81281 | 2.429599 | -11.5017 | 1.16E-27 | 3.63E-26 | 51.57168 |
| ENSG00000197576.12 | HOXA4 | -0.81282 | 0.676976 | -11.0802 | 5.83E-26 | 1.63E-24 | 47.68729 |
| ENSG00000196159.10 | FAT4 | -0.81336 | 0.979056 | -11.0506 | 7.65E-26 | 2.13E-24 | 47.41788 |
| ENSG00000151617.14 | EDNRA | -0.81376 | 2.768676 | -6.87127 | 1.68E-11 | 1.56E-10 | 14.79397 |
| ENSG00000155849.14 | ELMO1 | -0.81518 | 1.815015 | -9.1426 | 1.08E-18 | 1.84E-17 | 31.10642 |
| ENSG00000151276.22 | MAGI1 | -0.81554 | 1.759957 | -10.8152 | 6.52E-25 | 1.72E-23 | 45.29246 |
| ENSG00000198771.9 | RCSD1 | -0.81566 | 2.110612 | -8.28303 | 8.70E-16 | 1.18E-14 | 24.50235 |
| ENSG00000141497.12 | ZMYND15 | -0.81706 | 1.561102 | -10.3489 | 4.16E-23 | 9.62E-22 | 41.17225 |
| ENSG00000114019.13 | AMOTL2 | -0.81761 | 3.629547 | -7.7876 | 3.27E-14 | 3.88E-13 | 20.92822 |
| ENSG00000136630.12 | HLX | -0.81824 | 1.398689 | -11.6308 | 3.45E-28 | 1.12E-26 | 52.77957 |
| ENSG00000147065.15 | MSN | -0.81826 | 6.749746 | -7.83059 | 2.40E-14 | 2.89E-13 | 21.23134 |
| ENSG00000164211.11 | STARD4 | -0.81848 | 2.147383 | -9.76408 | 6.42E-21 | 1.27E-19 | 36.18128 |
| ENSG00000140932.8 | CMTM2 | -0.81856 | 0.468116 | -20.3418 | 1.79E-69 | 4.16E-67 | 147.4293 |
| ENSG00000053918.14 | KCNQ1 | -0.81865 | 3.223136 | -5.08432 | 5.02E-07 | 2.88E-06 | 4.755017 |
| ENSG00000120162.9 | MOB3B | -0.81965 | 1.675067 | -9.87684 | 2.47E-21 | 5.06E-20 | 37.12769 |
| ENSG00000169504.13 | CLIC4 | -0.82004 | 5.350078 | -8.78503 | 1.86E-17 | 2.88E-16 | 28.29896 |
| ENSG00000146250.6 | PRSS35 | -0.82007 | 0.395319 | -16.4646 | 4.61E-50 | 4.92E-48 | 102.8851 |
| ENSG00000170145.4 | SIK2 | -0.82088 | 2.803183 | -10.6994 | 1.85E-24 | 4.70E-23 | 44.25773 |
| ENSG00000181744.7 | C3orf58 | -0.82147 | 2.280814 | -10.0567 | 5.29E-22 | 1.14E-20 | 38.65337 |
| ENSG00000184545.9 | DUSP8 | -0.82171 | 2.081479 | -8.8833 | 8.58E-18 | 1.36E-16 | 29.06208 |
| ENSG00000164929.15 | BAALC | -0.82323 | 1.207382 | -5.09741 | 4.70E-07 | 2.70E-06 | 4.818793 |
| ENSG00000105383.13 | CD33 | -0.82324 | 1.018371 | -12.3764 | 2.62E-31 | 1.02E-29 | 59.9141 |
| ENSG00000118503.13 | TNFAIP3 | -0.82359 | 3.57851 | -6.44721 | 2.44E-10 | 2.03E-09 | 12.17451 |
| ENSG00000112964.12 | GHR | -0.82362 | 0.477895 | -16.9239 | 2.66E-52 | 3.12E-50 | 108.0226 |
| ENSG00000114853.12 | ZBTB47 | -0.82432 | 2.267391 | -11.5457 | 7.69E-28 | 2.43E-26 | 51.98282 |
| ENSG00000122756.13 | CNTFR | -0.82461 | 0.340165 | -13.9852 | 2.16E-38 | 1.31E-36 | 76.13285 |
| ENSG00000160712.11 | IL6R | -0.82469 | 3.235122 | -7.22027 | 1.68E-12 | 1.71E-11 | 17.05533 |
| ENSG00000047644.16 | WWC3 | -0.82542 | 3.593191 | -8.57021 | 9.83E-17 | 1.43E-15 | 26.65324 |
| ENSG00000130224.13 | LRCH2 | -0.82553 | 0.935825 | -9.70453 | 1.06E-20 | 2.06E-19 | 35.68459 |
| ENSG00000163069.11 | SGCB | -0.82609 | 2.880112 | -9.78585 | 5.34E-21 | 1.07E-19 | 36.36341 |
| ENSG00000102804.13 | TSC22D1 | -0.82615 | 4.757813 | -8.76778 | 2.13E-17 | 3.27E-16 | 28.16564 |
| ENSG00000150907.6 | FOXO1 | -0.82618 | 2.54072 | -8.91239 | 6.82E-18 | 1.09E-16 | 29.28927 |
| ENSG00000013297.9 | CLDN11 | -0.82635 | 0.814336 | -9.30093 | 3.00E-19 | 5.32E-18 | 32.37618 |
| ENSG00000115896.14 | PLCL1 | -0.82707 | 0.682945 | -18.9114 | 3.37E-62 | 5.96E-60 | 130.7308 |
| ENSG00000111339.9 | ART4 | -0.82757 | 0.378196 | -17.0776 | 4.68E-53 | 5.64E-51 | 109.7521 |
| ENSG00000169891.16 | REPS2 | -0.82769 | 1.488646 | -10.4735 | 1.39E-23 | 3.32E-22 | 42.2607 |
| ENSG00000067606.14 | PRKCZ | -0.82773 | 2.644817 | -7.84196 | 2.22E-14 | 2.67E-13 | 21.31176 |
| ENSG00000119917.12 | IFIT3 | -0.82793 | 4.130763 | -6.19883 | 1.10E-09 | 8.52E-09 | 10.70713 |
| ENSG00000088320.3 | REM1 | -0.82835 | 0.854466 | -14.275 | 1.03E-39 | 6.55E-38 | 79.16021 |
| ENSG00000120217.12 | CD274 | -0.82838 | 2.009134 | -5.91462 | 5.76E-09 | 4.14E-08 | 9.089999 |
| ENSG00000115956.9 | PLEK | -0.82853 | 3.541675 | -5.9405 | 4.96E-09 | 3.60E-08 | 9.234484 |
| ENSG00000203727.3 | SAMD5 | -0.82876 | 1.029824 | -8.89969 | 7.54E-18 | 1.20E-16 | 29.19004 |
| ENSG00000183578.5 | TNFAIP8L3 | -0.82941 | 1.526868 | -9.80324 | 4.61E-21 | 9.27E-20 | 36.50905 |
| ENSG00000113231.12 | PDE8B | -0.82945 | 0.872303 | -15.0763 | 1.98E-43 | 1.54E-41 | 87.67789 |
| ENSG00000091583.9 | APOH | -0.82988 | 1.761361 | -3.94801 | 8.87E-05 | 0.000372754 | -0.19746 |
| ENSG00000100012.10 | SEC14L3 | -0.83079 | 0.248561 | -12.6882 | 1.21E-32 | 5.13E-31 | 62.97346 |
| ENSG00000158578.17 | ALAS2 | -0.83178 | 0.193859 | -18.7047 | 3.71E-61 | 6.31E-59 | 128.3385 |
| ENSG00000171033.11 | PKIA | -0.83184 | 1.48452 | -7.11771 | 3.34E-12 | 3.30E-11 | 16.38109 |
| ENSG00000166710.16 | B2M | -0.83283 | 9.617595 | -7.58616 | 1.36E-13 | 1.54E-12 | 19.52571 |
| ENSG00000144724.17 | PTPRG | -0.83321 | 2.014757 | -8.97496 | 4.15E-18 | 6.76E-17 | 29.77973 |
| ENSG00000137831.13 | UACA | -0.83553 | 2.220448 | -9.95397 | 1.28E-21 | 2.67E-20 | 37.7795 |
| ENSG00000184374.2 | COLEC10 | -0.83577 | 0.29049 | -15.3906 | 6.52E-45 | 5.55E-43 | 91.07462 |
| ENSG00000104763.16 | ASAH1 | -0.83625 | 6.176445 | -8.30427 | 7.42E-16 | 1.01E-14 | 24.65949 |
| ENSG00000127603.22 | MACF1 | -0.83626 | 2.948613 | -8.81179 | 1.51E-17 | 2.35E-16 | 28.50609 |
| ENSG00000116741.7 | RGS2 | -0.83799 | 4.538576 | -5.52652 | 4.99E-08 | 3.23E-07 | 6.990573 |
| ENSG00000136867.9 | SLC31A2 | -0.83875 | 0.997749 | -14.126 | 4.94E-39 | 3.06E-37 | 77.60013 |
| ENSG00000197879.13 | MYO1C | -0.83894 | 4.909593 | -11.7377 | 1.25E-28 | 4.17E-27 | 53.78656 |
| ENSG00000171840.10 | NINJ2 | -0.83914 | 3.136067 | -6.5073 | 1.69E-10 | 1.42E-09 | 12.53698 |
| ENSG00000144278.13 | GALNT13 | -0.83935 | 0.552626 | -9.95834 | 1.23E-21 | 2.58E-20 | 37.81651 |
| ENSG00000204577.10 | LILRB3 | -0.84031 | 1.19392 | -12.4229 | 1.66E-31 | 6.59E-30 | 60.36793 |
| ENSG00000172935.8 | MRGPRF | -0.84102 | 1.628726 | -8.5899 | 8.45E-17 | 1.24E-15 | 26.80277 |
| ENSG00000111252.9 | SH2B3 | -0.84178 | 2.915267 | -9.92382 | 1.65E-21 | 3.44E-20 | 37.52427 |
| ENSG00000151490.12 | PTPRO | -0.84319 | 0.962664 | -12.3678 | 2.85E-31 | 1.11E-29 | 59.83022 |
| ENSG00000108381.9 | ASPA | -0.8433 | 0.33128 | -24.7014 | 5.58E-92 | 3.48E-89 | 199.1046 |
| ENSG00000124406.15 | ATP8A1 | -0.84418 | 2.367045 | -6.67026 | 6.08E-11 | 5.36E-10 | 13.53455 |
| ENSG00000069424.13 | KCNAB2 | -0.84447 | 2.265593 | -9.21582 | 6.00E-19 | 1.04E-17 | 31.69164 |
| ENSG00000174600.12 | CMKLR1 | -0.84498 | 2.14919 | -7.44881 | 3.53E-13 | 3.86E-12 | 18.58657 |
| ENSG00000100906.9 | NFKBIA | -0.84514 | 6.570025 | -7.51398 | 2.25E-13 | 2.50E-12 | 19.03043 |
| ENSG00000164176.11 | EDIL3 | -0.84577 | 2.993161 | -5.36419 | 1.19E-07 | 7.37E-07 | 6.150353 |
| ENSG00000188659.8 | FAM154B | -0.84637 | 0.781008 | -7.67894 | 7.08E-14 | 8.16E-13 | 20.16798 |
| ENSG00000160838.12 | LRRC71 | -0.84745 | 0.488721 | -8.75308 | 2.38E-17 | 3.65E-16 | 28.05216 |
| ENSG00000186407.6 | CD300E | -0.84834 | 1.025857 | -9.51962 | 4.97E-20 | 9.25E-19 | 34.15619 |
| ENSG00000115604.9 | IL18R1 | -0.84835 | 1.125055 | -10.7156 | 1.60E-24 | 4.09E-23 | 44.40231 |
| ENSG00000027075.12 | PRKCH | -0.84859 | 2.357156 | -12.8382 | 2.70E-33 | 1.21E-31 | 64.46108 |
| ENSG00000092051.15 | JPH4 | -0.84935 | 0.355891 | -27.8445 | 3.68E-108 | 4.73E-105 | 236.2622 |
| ENSG00000138449.9 | SLC40A1 | -0.84958 | 5.287527 | -5.58177 | 3.70E-08 | 2.44E-07 | 7.281705 |
| ENSG00000169896.15 | ITGAM | -0.85017 | 2.605417 | -6.42681 | 2.77E-10 | 2.28E-09 | 12.05213 |
| ENSG00000142347.15 | MYO1F | -0.85018 | 2.33285 | -8.706 | 3.44E-17 | 5.19E-16 | 27.68985 |
| ENSG00000110880.9 | CORO1C | -0.85051 | 4.493636 | -10.2489 | 9.99E-23 | 2.27E-21 | 40.3039 |
| ENSG00000135185.10 | TMEM243 | -0.85052 | 3.299798 | -8.37494 | 4.36E-16 | 6.03E-15 | 25.18452 |
| ENSG00000089356.15 | FXYD3 | -0.85054 | 4.578554 | -3.99238 | 7.40E-05 | 0.000315025 | -0.02604 |
| ENSG00000134243.10 | SORT1 | -0.85073 | 4.062572 | -9.64703 | 1.72E-20 | 3.28E-19 | 35.20703 |
| ENSG00000129657.13 | SEC14L1 | -0.85075 | 3.185096 | -10.0299 | 6.66E-22 | 1.42E-20 | 38.42491 |
| ENSG00000198909.6 | MAP3K3 | -0.85095 | 2.92002 | -11.9861 | 1.16E-29 | 4.11E-28 | 56.14726 |
| ENSG00000071246.9 | VASH1 | -0.85158 | 2.301641 | -9.74076 | 7.81E-21 | 1.54E-19 | 35.98652 |
| ENSG00000214063.9 | TSPAN4 | -0.85225 | 3.505999 | -8.30661 | 7.29E-16 | 9.90E-15 | 24.67685 |
| ENSG00000127241.15 | MASP1 | -0.85261 | 0.339113 | -21.9858 | 6.28E-78 | 2.09E-75 | 166.841 |
| ENSG00000134240.10 | HMGCS2 | -0.85303 | 0.679432 | -5.47977 | 6.42E-08 | 4.11E-07 | 6.746259 |
| ENSG00000180537.11 | RNF182 | -0.85316 | 0.420665 | -12.7821 | 4.73E-33 | 2.09E-31 | 63.90324 |
| ENSG00000163412.11 | EIF4E3 | -0.85396 | 1.895479 | -12.5255 | 6.04E-32 | 2.47E-30 | 61.37219 |
| ENSG00000105509.9 | HAS1 | -0.85447 | 0.439419 | -9.74055 | 7.83E-21 | 1.54E-19 | 35.98478 |
| ENSG00000124067.15 | SLC12A4 | -0.85484 | 2.845518 | -11.4392 | 2.09E-27 | 6.43E-26 | 50.99051 |
| ENSG00000122122.9 | SASH3 | -0.85498 | 3.392181 | -6.87978 | 1.59E-11 | 1.48E-10 | 14.84799 |
| ENSG00000159176.12 | CSRP1 | -0.85538 | 4.709923 | -12.697 | 1.10E-32 | 4.71E-31 | 63.06101 |
| ENSG00000102010.13 | BMX | -0.85659 | 0.532911 | -10.7262 | 1.46E-24 | 3.73E-23 | 44.496 |
| ENSG00000164683.15 | HEY1 | -0.85676 | 2.34252 | -6.93025 | 1.15E-11 | 1.08E-10 | 15.16951 |
| ENSG00000204950.3 | LRRC10B | -0.85722 | 1.354647 | -5.57063 | 3.93E-08 | 2.58E-07 | 7.222779 |
| ENSG00000178965.12 | ERICH3 | -0.85902 | 0.501067 | -8.13291 | 2.66E-15 | 3.46E-14 | 23.40091 |
| ENSG00000086300.14 | SNX10 | -0.85943 | 3.204889 | -7.04715 | 5.33E-12 | 5.19E-11 | 15.9219 |
| ENSG00000165424.6 | ZCCHC24 | -0.86028 | 2.77404 | -10.1548 | 2.27E-22 | 5.01E-21 | 39.49291 |
| ENSG00000122420.8 | PTGFR | -0.86032 | 0.626357 | -9.53381 | 4.41E-20 | 8.25E-19 | 34.27271 |
| ENSG00000141506.12 | PIK3R5 | -0.86118 | 1.577101 | -10.2126 | 1.37E-22 | 3.08E-21 | 39.99077 |
| ENSG00000115109.12 | EPB41L5 | -0.86151 | 2.700299 | -9.77698 | 5.76E-21 | 1.15E-19 | 36.28911 |
| ENSG00000119681.10 | LTBP2 | -0.86155 | 4.495337 | -5.84027 | 8.79E-09 | 6.20E-08 | 8.678006 |
| ENSG00000147206.15 | NXF3 | -0.8617 | 0.3949 | -11.5025 | 1.16E-27 | 3.61E-26 | 51.57904 |
| ENSG00000128052.8 | KDR | -0.86188 | 3.272289 | -5.77741 | 1.25E-08 | 8.67E-08 | 8.333263 |
| ENSG00000177119.14 | ANO6 | -0.86249 | 3.514093 | -11.1528 | 2.99E-26 | 8.52E-25 | 48.34933 |
| ENSG00000185112.5 | FAM43A | -0.86339 | 2.034198 | -8.69693 | 3.69E-17 | 5.55E-16 | 27.62025 |
| ENSG00000151414.13 | NEK7 | -0.8645 | 3.915451 | -11.7019 | 1.76E-28 | 5.82E-27 | 53.4488 |
| ENSG00000112769.17 | LAMA4 | -0.86495 | 2.662672 | -7.97623 | 8.39E-15 | 1.05E-13 | 22.2683 |
| ENSG00000128918.13 | ALDH1A2 | -0.86539 | 0.87031 | -6.70172 | 4.98E-11 | 4.43E-10 | 13.72957 |
| ENSG00000163659.11 | TIPARP | -0.86564 | 3.692134 | -6.14597 | 1.50E-09 | 1.15E-08 | 10.40131 |
| ENSG00000152642.9 | GPD1L | -0.86568 | 3.813799 | -7.60286 | 1.21E-13 | 1.37E-12 | 19.64083 |
| ENSG00000173947.12 | PIFO | -0.86701 | 1.977905 | -5.67671 | 2.19E-08 | 1.48E-07 | 7.787979 |
| ENSG00000112530.10 | PACRG | -0.86814 | 0.561918 | -9.98486 | 9.81E-22 | 2.06E-20 | 38.04147 |
| ENSG00000151726.12 | ACSL1 | -0.86903 | 4.355072 | -8.02442 | 5.90E-15 | 7.46E-14 | 22.61483 |
| ENSG00000117245.11 | KIF17 | -0.86923 | 0.616305 | -19.6988 | 3.43E-66 | 7.20E-64 | 139.895 |
| ENSG00000176046.8 | NUPR1 | -0.86934 | 3.82323 | -6.38708 | 3.53E-10 | 2.88E-09 | 11.81471 |
| ENSG00000149577.14 | SIDT2 | -0.86959 | 2.981728 | -12.5993 | 2.91E-32 | 1.21E-30 | 62.09703 |
| ENSG00000166106.3 | ADAMTS15 | -0.87038 | 0.891064 | -10.8246 | 5.99E-25 | 1.58E-23 | 45.37673 |
| ENSG00000122035.6 | RASL11A | -0.87102 | 2.648853 | -6.54652 | 1.32E-10 | 1.13E-09 | 12.77516 |
| ENSG00000196396.8 | PTPN1 | -0.87123 | 4.604851 | -11.4873 | 1.33E-27 | 4.14E-26 | 51.43804 |
| ENSG00000117594.8 | HSD11B1 | -0.872 | 2.321083 | -6.97157 | 8.75E-12 | 8.35E-11 | 15.43424 |
| ENSG00000178175.10 | ZNF366 | -0.87223 | 0.644904 | -17.4013 | 1.19E-54 | 1.52E-52 | 113.4106 |
| ENSG00000145861.7 | C1QTNF2 | -0.87252 | 0.711374 | -15.5545 | 1.09E-45 | 9.63E-44 | 92.85618 |
| ENSG00000174156.12 | GSTA3 | -0.8729 | 0.346302 | -12.3967 | 2.15E-31 | 8.46E-30 | 60.1122 |
| ENSG00000157600.10 | TMEM164 | -0.87372 | 3.404763 | -10.1269 | 2.88E-22 | 6.33E-21 | 39.25404 |
| ENSG00000206190.10 | ATP10A | -0.87383 | 1.87859 | -7.69567 | 6.29E-14 | 7.28E-13 | 20.28442 |
| ENSG00000197629.5 | MPEG1 | -0.87457 | 3.397491 | -6.68874 | 5.41E-11 | 4.80E-10 | 13.64898 |
| ENSG00000165383.9 | LRRC18 | -0.87498 | 0.427186 | -11.8294 | 5.22E-29 | 1.78E-27 | 54.65401 |
| ENSG00000170899.9 | GSTA4 | -0.8754 | 3.548613 | -6.89256 | 1.47E-11 | 1.37E-10 | 14.92923 |
| ENSG00000125538.10 | IL1B | -0.87556 | 1.710538 | -7.27089 | 1.19E-12 | 1.24E-11 | 17.3911 |
| ENSG00000171533.10 | MAP6 | -0.87616 | 1.096878 | -10.0405 | 6.08E-22 | 1.30E-20 | 38.51476 |
| ENSG00000166912.15 | MTMR10 | -0.87637 | 1.903493 | -15.5426 | 1.24E-45 | 1.10E-43 | 92.72661 |
| ENSG00000117020.15 | AKT3 | -0.87658 | 1.767565 | -9.56854 | 3.31E-20 | 6.23E-19 | 34.55847 |
| ENSG00000129353.13 | SLC44A2 | -0.8766 | 5.352352 | -12.3367 | 3.86E-31 | 1.49E-29 | 59.52771 |
| ENSG00000188883.4 | KLRG2 | -0.87668 | 1.017523 | -8.01959 | 6.11E-15 | 7.72E-14 | 22.58005 |
| ENSG00000163154.5 | TNFAIP8L2 | -0.87669 | 2.658256 | -7.71447 | 5.50E-14 | 6.40E-13 | 20.41559 |
| ENSG00000151623.13 | NR3C2 | -0.87687 | 1.754747 | -8.4723 | 2.08E-16 | 2.96E-15 | 25.9135 |
| ENSG00000106511.5 | MEOX2 | -0.87728 | 1.478225 | -8.39761 | 3.67E-16 | 5.12E-15 | 25.35368 |
| ENSG00000126861.4 | OMG | -0.87788 | 0.782051 | -8.17047 | 2.01E-15 | 2.65E-14 | 23.67501 |
| ENSG00000158186.11 | MRAS | -0.87859 | 2.17268 | -11.813 | 6.11E-29 | 2.08E-27 | 54.49872 |
| ENSG00000186471.11 | AKAP14 | -0.87941 | 0.568091 | -8.10594 | 3.24E-15 | 4.19E-14 | 23.20469 |
| ENSG00000112539.13 | C6orf118 | -0.87986 | 0.542244 | -8.29708 | 7.83E-16 | 1.06E-14 | 24.60626 |
| ENSG00000138411.9 | HECW2 | -0.88196 | 1.485657 | -11.2885 | 8.53E-27 | 2.53E-25 | 49.59533 |
| ENSG00000123384.12 | LRP1 | -0.88215 | 4.206857 | -7.25319 | 1.34E-12 | 1.39E-11 | 17.27346 |
| ENSG00000027697.11 | IFNGR1 | -0.88265 | 5.552733 | -9.48679 | 6.52E-20 | 1.21E-18 | 33.88702 |
| ENSG00000185905.3 | C16orf54 | -0.88287 | 1.818945 | -7.89834 | 1.48E-14 | 1.81E-13 | 21.71186 |
| ENSG00000150764.12 | DIXDC1 | -0.88313 | 1.572435 | -14.0217 | 1.47E-38 | 8.99E-37 | 76.51257 |
| ENSG00000185614.4 | FAM212A | -0.8836 | 1.40966 | -11.592 | 4.97E-28 | 1.59E-26 | 52.41633 |
| ENSG00000167705.10 | RILP | -0.88655 | 2.906606 | -10.4246 | 2.14E-23 | 5.05E-22 | 41.8326 |
| ENSG00000133835.13 | HSD17B4 | -0.88683 | 3.932966 | -12.0426 | 6.74E-30 | 2.42E-28 | 56.688 |
| ENSG00000187492.7 | CDHR4 | -0.88742 | 0.732814 | -6.784 | 2.95E-11 | 2.68E-10 | 14.24327 |
| ENSG00000065054.12 | SLC9A3R2 | -0.88836 | 4.997863 | -7.79864 | 3.02E-14 | 3.60E-13 | 21.0059 |
| ENSG00000162598.12 | C1orf87 | -0.88853 | 0.413342 | -10.0987 | 3.68E-22 | 7.99E-21 | 39.01242 |
| ENSG00000107968.8 | MAP3K8 | -0.88925 | 2.676851 | -9.77822 | 5.70E-21 | 1.14E-19 | 36.2995 |
| ENSG00000137752.21 | CASP1 | -0.88939 | 2.935858 | -8.16368 | 2.12E-15 | 2.78E-14 | 23.62538 |
| ENSG00000128641.16 | MYO1B | -0.88956 | 4.136242 | -11.2196 | 1.61E-26 | 4.70E-25 | 48.96157 |
| ENSG00000188153.11 | COL4A5 | -0.88988 | 1.372654 | -7.67661 | 7.20E-14 | 8.29E-13 | 20.15172 |
| ENSG00000163686.12 | ABHD6 | -0.89047 | 1.726326 | -14.5158 | 8.04E-41 | 5.49E-39 | 81.69805 |
| ENSG00000134533.5 | RERG | -0.89117 | 1.720756 | -8.51647 | 1.49E-16 | 2.13E-15 | 26.24637 |
| ENSG00000187950.7 | OVCH1 | -0.89118 | 0.190399 | -29.122 | 1.15E-114 | 1.97E-111 | 251.1988 |
| ENSG00000115008.5 | IL1A | -0.89135 | 0.692738 | -8.59125 | 8.36E-17 | 1.22E-15 | 26.81298 |
| ENSG00000204472.11 | AIF1 | -0.89193 | 4.539348 | -6.68401 | 5.57E-11 | 4.94E-10 | 13.61971 |
| ENSG00000197646.7 | PDCD1LG2 | -0.89209 | 1.725695 | -8.24371 | 1.17E-15 | 1.56E-14 | 24.21235 |
| ENSG00000117758.12 | STX12 | -0.89213 | 4.235026 | -17.2469 | 6.87E-54 | 8.59E-52 | 111.6634 |
| ENSG00000105989.7 | WNT2 | -0.89309 | 1.876453 | -7.43435 | 3.90E-13 | 4.24E-12 | 18.48853 |
| ENSG00000155090.13 | KLF10 | -0.8935 | 4.306311 | -10.2464 | 1.02E-22 | 2.32E-21 | 40.28273 |
| ENSG00000203797.8 | DDO | -0.89362 | 1.193261 | -10.0365 | 6.30E-22 | 1.34E-20 | 38.48041 |
| ENSG00000262655.3 | SPON1 | -0.89366 | 3.152089 | -6.06669 | 2.39E-09 | 1.79E-08 | 9.946994 |
| ENSG00000136960.11 | ENPP2 | -0.89408 | 3.160694 | -7.20171 | 1.90E-12 | 1.93E-11 | 16.93275 |
| ENSG00000114541.13 | FRMD4B | -0.89413 | 2.071681 | -12.7427 | 7.01E-33 | 3.04E-31 | 63.51275 |
| ENSG00000127084.16 | FGD3 | -0.89468 | 1.881797 | -10.2167 | 1.32E-22 | 2.97E-21 | 40.02645 |
| ENSG00000139517.7 | LNX2 | -0.895 | 3.165447 | -10.7179 | 1.57E-24 | 4.01E-23 | 44.42263 |
| ENSG00000100154.13 | TTC28 | -0.89567 | 1.599808 | -12.5575 | 4.40E-32 | 1.81E-30 | 61.68627 |
| ENSG00000196628.12 | TCF4 | -0.8957 | 1.690663 | -11.2942 | 8.09E-27 | 2.40E-25 | 49.64794 |
| ENSG00000198722.11 | UNC13B | -0.89636 | 4.165623 | -6.79853 | 2.69E-11 | 2.45E-10 | 14.33455 |
| ENSG00000064999.13 | ANKS1A | -0.89656 | 3.276361 | -11.967 | 1.40E-29 | 4.91E-28 | 55.96418 |
| ENSG00000161640.14 | SIGLEC11 | -0.89656 | 0.465783 | -19.6267 | 7.98E-66 | 1.65E-63 | 139.0522 |
| ENSG00000152492.12 | CCDC50 | -0.89662 | 2.760618 | -12.1836 | 1.72E-30 | 6.39E-29 | 58.04451 |
| ENSG00000135046.12 | ANXA1 | -0.89723 | 5.907262 | -4.75749 | 2.49E-06 | 1.31E-05 | 3.212204 |
| ENSG00000145675.13 | PIK3R1 | -0.89772 | 2.479032 | -11.3196 | 6.39E-27 | 1.91E-25 | 49.88219 |
| ENSG00000166128.11 | RAB8B | -0.89931 | 2.923566 | -10.6152 | 3.93E-24 | 9.78E-23 | 43.51017 |
| ENSG00000080503.18 | SMARCA2 | -0.89979 | 3.533609 | -9.78315 | 5.46E-21 | 1.09E-19 | 36.34077 |
| ENSG00000175489.9 | LRRC25 | -0.89991 | 2.346561 | -8.47807 | 1.99E-16 | 2.83E-15 | 25.95694 |
| ENSG00000130844.15 | ZNF331 | -0.90146 | 2.072648 | -10.0327 | 6.51E-22 | 1.39E-20 | 38.44809 |
| ENSG00000103175.9 | WFDC1 | -0.90195 | 0.719227 | -14.6601 | 1.73E-41 | 1.23E-39 | 83.22782 |
| ENSG00000049540.15 | ELN | -0.90432 | 3.694067 | -4.98366 | 8.30E-07 | 4.63E-06 | 4.269834 |
| ENSG00000108387.13 | 4-Sep | -0.90436 | 1.149601 | -15.7139 | 1.90E-46 | 1.74E-44 | 94.59694 |
| ENSG00000132376.18 | INPP5K | -0.90504 | 3.193513 | -15.9125 | 2.13E-47 | 2.05E-45 | 96.77515 |
| ENSG00000166510.12 | CCDC68 | -0.90504 | 1.687086 | -9.25731 | 4.28E-19 | 7.51E-18 | 32.02478 |
| ENSG00000047365.10 | ARAP2 | -0.90548 | 1.756818 | -12.0491 | 6.33E-30 | 2.28E-28 | 56.75002 |
| ENSG00000120645.10 | IQSEC3 | -0.90674 | 0.40191 | -20.1272 | 2.24E-68 | 5.06E-66 | 144.9099 |
| ENSG00000140807.5 | NKD1 | -0.90675 | 0.829234 | -9.52222 | 4.86E-20 | 9.06E-19 | 34.17751 |
| ENSG00000154479.11 | CCDC173 | -0.90697 | 0.674834 | -10.4921 | 1.18E-23 | 2.83E-22 | 42.42435 |
| ENSG00000107099.14 | DOCK8 | -0.90771 | 2.156039 | -9.20484 | 6.56E-19 | 1.13E-17 | 31.60365 |
| ENSG00000007237.17 | GAS7 | -0.90774 | 2.092672 | -8.97948 | 4.00E-18 | 6.53E-17 | 29.81523 |
| ENSG00000165996.12 | PTPLA | -0.90821 | 1.435956 | -8.66997 | 4.55E-17 | 6.80E-16 | 27.41359 |
| ENSG00000129226.12 | CD68 | -0.90851 | 1.138424 | -12.0173 | 8.60E-30 | 3.07E-28 | 56.44514 |
| ENSG00000118432.12 | CNR1 | -0.90909 | 0.864453 | -10.2449 | 1.03E-22 | 2.34E-21 | 40.27006 |
| ENSG00000172037.12 | LAMB2 | -0.90909 | 4.76915 | -9.95613 | 1.25E-21 | 2.62E-20 | 37.79779 |
| ENSG00000136848.15 | DAB2IP | -0.90944 | 3.158974 | -10.7917 | 8.07E-25 | 2.11E-23 | 45.08169 |
| ENSG00000275832.3 | ARHGAP23 | -0.91001 | 2.5645 | -9.13652 | 1.14E-18 | 1.93E-17 | 31.05798 |
| ENSG00000163661.3 | PTX3 | -0.91079 | 1.039128 | -8.43427 | 2.78E-16 | 3.91E-15 | 25.62796 |
| ENSG00000081026.17 | MAGI3 | -0.9109 | 2.633004 | -8.65906 | 4.95E-17 | 7.38E-16 | 27.3301 |
| ENSG00000138759.16 | FRAS1 | -0.91195 | 0.849054 | -10.7027 | 1.80E-24 | 4.57E-23 | 44.28667 |
| ENSG00000106078.16 | COBL | -0.91206 | 1.691368 | -8.7549 | 2.35E-17 | 3.60E-16 | 28.06621 |
| ENSG00000064763.9 | FAR2 | -0.91338 | 1.328535 | -10.3849 | 3.03E-23 | 7.07E-22 | 41.48577 |
| ENSG00000174282.10 | ZBTB4 | -0.91344 | 4.287656 | -11.6322 | 3.40E-28 | 1.10E-26 | 52.79268 |
| ENSG00000120594.15 | PLXDC2 | -0.91386 | 2.866135 | -8.3468 | 5.39E-16 | 7.41E-15 | 24.97504 |
| ENSG00000131171.11 | SH3BGRL | -0.91502 | 5.555418 | -11.0097 | 1.11E-25 | 3.08E-24 | 47.04573 |
| ENSG00000138193.13 | PLCE1 | -0.91591 | 1.240603 | -12.4088 | 1.90E-31 | 7.54E-30 | 60.23059 |
| ENSG00000010610.8 | CD4 | -0.91678 | 4.565488 | -7.20716 | 1.83E-12 | 1.86E-11 | 16.96868 |
| ENSG00000176697.17 | BDNF | -0.91901 | 0.376201 | -16.0771 | 3.44E-48 | 3.46E-46 | 98.5888 |
| ENSG00000134962.6 | KLB | -0.91906 | 0.503837 | -9.24953 | 4.56E-19 | 7.97E-18 | 31.96222 |
| ENSG00000170786.11 | SDR16C5 | -0.91958 | 3.882264 | -5.13638 | 3.86E-07 | 2.25E-06 | 5.009445 |
[truncated: 109,074 more chars]
