# Supplementary material for: Prognostic value of lncRNAs related to fatty acid metabolism in lung adenocarcinoma and their correlation with tumor microenvironment based on bioinformatics analysis
Source: Front Oncol. 2022 Oct 10;12:1022097. doi: 10.3389/fonc.2022.1022097 (PMC9590110; doi:10.3389/fonc.2022.1022097)
Supplement: Supplementary Table 1 — All samples were divided into high and low fatty acid metabolism score groups based on the median value of this score. [file DataSheet_1.zip › raw data and R code for checking/raw data/3.docx]

| miRNA | logFC | AveExpr | t | P.Value | adj.P.Val | B |
| --- | --- | --- | --- | --- | --- | --- |
| hsa-miR-210-3p | 5.272807 | 8.893332 | 19.02096 | 6.33E-61 | 7.00E-58 | 127.9145 |
| hsa-miR-9-5p | 4.350388 | 9.205928 | 12.14366 | 7.09E-30 | 5.23E-28 | 56.99179 |
| hsa-miR-196a-5p | 3.137568 | 3.82237 | 7.122609 | 3.77E-12 | 5.44E-11 | 16.72393 |
| hsa-miR-708-3p | 3.049883 | 5.744342 | 17.34202 | 5.80E-53 | 2.14E-50 | 109.718 |
| hsa-miR-21-5p | 2.92406 | 18.10083 | 23.10967 | 1.23E-80 | 2.71E-77 | 172.9622 |
| hsa-miR-96-5p | 2.738601 | 4.2218 | 17.43299 | 2.17E-53 | 1.20E-50 | 110.6949 |
| hsa-miR-153-5p | 2.727865 | 3.037313 | 11.92023 | 5.75E-29 | 3.97E-27 | 54.91915 |
| hsa-miR-135b-5p | 2.716438 | 5.181977 | 11.79752 | 1.80E-28 | 1.14E-26 | 53.78989 |
| hsa-miR-708-5p | 2.704267 | 4.499057 | 15.02291 | 2.78E-42 | 5.58E-40 | 85.31376 |
| hsa-miR-1269a | 2.702711 | 3.052722 | 5.344777 | 1.39E-07 | 1.24E-06 | 6.485925 |
| hsa-miR-142-3p | 2.687924 | 10.58947 | 13.18619 | 3.14E-34 | 3.47E-32 | 66.92417 |
| hsa-miR-1307-5p | 2.635736 | 7.959777 | 13.02847 | 1.47E-33 | 1.55E-31 | 65.39541 |
| hsa-miR-182-5p | 2.628906 | 14.03415 | 17.38155 | 3.78E-53 | 1.67E-50 | 110.1424 |
| hsa-miR-183-5p | 2.533935 | 12.95584 | 15.61848 | 5.58E-45 | 1.37E-42 | 91.4752 |
| hsa-miR-21-3p | 2.465657 | 11.36289 | 17.49218 | 1.14E-53 | 8.41E-51 | 111.3312 |
| hsa-miR-33a-5p | 2.409384 | 3.652316 | 12.60302 | 9.01E-32 | 8.66E-30 | 61.31693 |
| hsa-miR-200a-3p | 2.393127 | 7.829826 | 11.66885 | 5.91E-28 | 3.44E-26 | 52.61275 |
| hsa-miR-141-5p | 2.358326 | 8.918923 | 14.98178 | 4.25E-42 | 7.83E-40 | 84.89138 |
| hsa-let-7g-3p | 2.327091 | 4.66827 | 13.50395 | 1.37E-35 | 1.89E-33 | 70.03055 |
| hsa-miR-200a-5p | 2.311295 | 8.654286 | 13.56256 | 7.65E-36 | 1.13E-33 | 70.60723 |
| hsa-miR-1287-3p | 2.233031 | 2.724775 | 12.30065 | 1.61E-30 | 1.32E-28 | 58.4604 |
| hsa-miR-141-3p | 2.225448 | 9.382206 | 12.42127 | 5.12E-31 | 4.35E-29 | 59.59557 |
| hsa-miR-127-5p | 2.212203 | 6.327993 | 9.772058 | 9.74E-21 | 3.08E-19 | 36.18118 |
| hsa-miR-196b-5p | 2.070144 | 4.583489 | 5.833396 | 9.83E-09 | 9.71E-08 | 9.048717 |
| hsa-miR-29b-3p | 2.008023 | 9.425452 | 12.0626 | 1.52E-29 | 1.08E-27 | 56.23735 |
| hsa-miR-7-1-3p | 1.982988 | 3.776026 | 16.39841 | 1.46E-48 | 4.60E-46 | 99.66219 |
| hsa-miR-31-5p | 1.957949 | 2.120655 | 6.400759 | 3.60E-10 | 4.28E-09 | 12.26748 |
| hsa-miR-205-5p | 1.950577 | 6.287915 | 4.314025 | 1.94E-05 | 0.000118 | 1.744502 |
| hsa-miR-301a-3p | 1.929234 | 2.998626 | 12.21305 | 3.69E-30 | 2.91E-28 | 57.63965 |
| hsa-miR-429 | 1.914004 | 6.456518 | 9.818819 | 6.61E-21 | 2.12E-19 | 36.5637 |
| hsa-miR-130b-5p | 1.905633 | 3.612329 | 11.74239 | 3.00E-28 | 1.79E-26 | 53.28462 |
| hsa-miR-143-5p | 1.893941 | 5.019366 | 9.901317 | 3.33E-21 | 1.10E-19 | 37.24155 |
| hsa-miR-455-3p | 1.882673 | 6.706934 | 9.493544 | 9.55E-20 | 2.74E-18 | 33.9286 |
| hsa-miR-193b-3p | 1.878407 | 5.490309 | 8.743448 | 3.56E-17 | 8.29E-16 | 28.0904 |
| hsa-miR-577 | 1.864736 | 1.880476 | 7.867107 | 2.31E-14 | 4.18E-13 | 21.72133 |
| hsa-miR-345-5p | 1.859517 | 3.755486 | 9.932744 | 2.56E-21 | 8.85E-20 | 37.50077 |
| hsa-miR-301a-5p | 1.790797 | 1.905124 | 10.22841 | 2.11E-22 | 8.98E-21 | 39.96594 |
| hsa-miR-130b-3p | 1.771618 | 3.950787 | 11.84929 | 1.11E-28 | 7.24E-27 | 54.26547 |
| hsa-miR-224-5p | 1.751677 | 4.824711 | 6.907308 | 1.53E-11 | 2.06E-10 | 15.35358 |
| hsa-miR-503-5p | 1.720912 | 2.581874 | 9.914164 | 2.99E-21 | 1.00E-19 | 37.34745 |
| hsa-miR-409-5p | 1.71478 | 2.124892 | 8.612934 | 9.63E-17 | 2.11E-15 | 27.11006 |
| hsa-miR-744-3p | 1.707677 | 3.271241 | 10.04298 | 1.02E-21 | 3.80E-20 | 38.41435 |
| hsa-miR-105-5p | 1.70238 | 1.900318 | 3.782922 | 0.000174 | 0.00091 | -0.33218 |
| hsa-miR-26b-3p | 1.696162 | 4.309981 | 11.50766 | 2.59E-27 | 1.43E-25 | 51.14848 |
| hsa-miR-450a-5p | 1.695548 | 2.853815 | 10.04787 | 9.74E-22 | 3.71E-20 | 38.45501 |
| hsa-miR-136-5p | 1.690991 | 3.553938 | 7.962705 | 1.17E-14 | 2.13E-13 | 22.39122 |
| hsa-miR-148a-3p | 1.690278 | 15.06766 | 10.88716 | 6.90E-25 | 3.25E-23 | 45.62382 |
| hsa-miR-590-5p | 1.681684 | 3.367473 | 12.52717 | 1.86E-31 | 1.65E-29 | 60.59701 |
| hsa-miR-20a-5p | 1.627392 | 7.517362 | 10.33493 | 8.49E-23 | 3.68E-21 | 40.86565 |
| hsa-miR-33b-5p | 1.603126 | 1.793063 | 10.05905 | 8.87E-22 | 3.44E-20 | 38.54808 |
| hsa-miR-29a-5p | 1.596201 | 2.992863 | 11.76805 | 2.36E-28 | 1.45E-26 | 53.51965 |
| hsa-miR-424-5p | 1.594042 | 5.886762 | 8.808765 | 2.15E-17 | 5.07E-16 | 28.58506 |
| hsa-miR-192-5p | 1.588627 | 8.895106 | 5.140441 | 3.96E-07 | 3.28E-06 | 5.473272 |
| hsa-miR-19a-3p | 1.57721 | 4.002567 | 8.637574 | 7.99E-17 | 1.80E-15 | 27.29431 |
| hsa-miR-181b-3p | 1.561617 | 4.348748 | 10.45449 | 3.04E-23 | 1.34E-21 | 41.88255 |
| hsa-miR-324-5p | 1.555346 | 4.349593 | 9.424254 | 1.67E-19 | 4.62E-18 | 33.37514 |
| hsa-miR-1307-3p | 1.552974 | 9.705516 | 10.54275 | 1.41E-23 | 6.38E-22 | 42.63803 |
| hsa-miR-629-3p | 1.547409 | 2.363694 | 9.566251 | 5.28E-20 | 1.56E-18 | 34.51234 |
| hsa-miR-20a-3p | 1.544655 | 2.188174 | 11.48721 | 3.13E-27 | 1.69E-25 | 50.96359 |
| hsa-let-7a-2-3p | 1.542044 | 2.925385 | 7.364573 | 7.50E-13 | 1.18E-11 | 18.30465 |
| hsa-miR-671-5p | 1.533741 | 2.077824 | 13.20062 | 2.73E-34 | 3.17E-32 | 67.06452 |
| hsa-miR-31-3p | 1.528843 | 1.727518 | 5.776717 | 1.35E-08 | 1.31E-07 | 8.741363 |
| hsa-miR-93-5p | 1.522043 | 11.65607 | 10.90853 | 5.71E-25 | 2.75E-23 | 45.81106 |
| hsa-miR-17-5p | 1.515004 | 8.110919 | 10.1608 | 3.75E-22 | 1.56E-20 | 39.3981 |
| hsa-miR-2355-5p | 1.51071 | 5.50324 | 9.720642 | 1.49E-20 | 4.64E-19 | 35.762 |
| hsa-miR-187-3p | 1.474564 | 3.617741 | 4.843154 | 1.71E-06 | 1.27E-05 | 4.063515 |
| hsa-miR-151a-5p | 1.441764 | 6.335432 | 12.73326 | 2.57E-32 | 2.59E-30 | 62.55826 |
| hsa-miR-217-5p | 1.415166 | 4.138594 | 6.482262 | 2.19E-10 | 2.74E-09 | 12.75073 |
| hsa-miR-9-3p | 1.41416 | 1.583406 | 5.833601 | 9.82E-09 | 9.71E-08 | 9.049829 |
| hsa-miR-29b-1-5p | 1.410655 | 2.353436 | 9.714801 | 1.56E-20 | 4.80E-19 | 35.71448 |
| hsa-miR-1301-3p | 1.404487 | 3.601375 | 9.407861 | 1.91E-19 | 5.17E-18 | 33.24461 |
| hsa-miR-200c-5p | 1.394659 | 4.73558 | 9.185858 | 1.13E-18 | 2.93E-17 | 31.49261 |
| hsa-miR-331-3p | 1.391091 | 5.064154 | 9.095674 | 2.30E-18 | 5.72E-17 | 30.78933 |
| hsa-miR-93-3p | 1.367303 | 3.096349 | 9.541958 | 6.44E-20 | 1.87E-18 | 34.31696 |
| hsa-miR-199b-5p | 1.35286 | 6.84167 | 7.746312 | 5.41E-14 | 9.50E-13 | 20.88388 |
| hsa-miR-19b-1-5p | 1.349895 | 2.729688 | 11.2912 | 1.86E-26 | 9.55E-25 | 49.20071 |
| hsa-miR-199a-5p | 1.349118 | 9.241072 | 8.867531 | 1.37E-17 | 3.25E-16 | 29.03239 |
| hsa-miR-450b-5p | 1.342861 | 3.659227 | 7.499461 | 3.00E-13 | 5.02E-12 | 19.20421 |
| hsa-miR-767-5p | 1.335198 | 1.509714 | 3.397153 | 0.000736 | 0.00328 | -1.67914 |
| hsa-miR-425-5p | 1.325875 | 7.114973 | 9.572568 | 5.02E-20 | 1.50E-18 | 34.5632 |
| hsa-miR-200b-5p | 1.325429 | 4.044771 | 8.161803 | 2.77E-15 | 5.32E-14 | 23.80631 |
| hsa-miR-19b-3p | 1.324631 | 6.391616 | 8.267753 | 1.27E-15 | 2.53E-14 | 24.57019 |
| hsa-miR-339-5p | 1.311886 | 4.835391 | 7.484802 | 3.31E-13 | 5.51E-12 | 19.10582 |
| hsa-miR-199b-3p | 1.285763 | 10.66264 | 9.199951 | 1.01E-18 | 2.69E-17 | 31.60295 |
| hsa-miR-199a-3p | 1.284438 | 10.66647 | 9.197241 | 1.03E-18 | 2.71E-17 | 31.58173 |
| hsa-miR-375-3p | 1.260692 | 13.90441 | 4.39494 | 1.36E-05 | 8.43E-05 | 2.083136 |
| hsa-miR-539-5p | 1.240268 | 1.629982 | 6.172041 | 1.41E-09 | 1.53E-08 | 10.93912 |
| hsa-miR-675-3p | 1.239368 | 2.761545 | 3.608615 | 0.000339 | 0.001674 | -0.9578 |
| hsa-miR-194-5p | 1.229946 | 7.789737 | 4.182742 | 3.41E-05 | 0.000202 | 1.207506 |
| hsa-miR-455-5p | 1.225713 | 4.267497 | 8.618261 | 9.25E-17 | 2.06E-15 | 27.14986 |
| hsa-miR-629-5p | 1.216758 | 6.655059 | 10.07416 | 7.81E-22 | 3.08E-20 | 38.67396 |
| hsa-miR-200b-3p | 1.208469 | 9.123572 | 7.001143 | 8.33E-12 | 1.17E-10 | 15.94659 |
| hsa-miR-625-3p | 1.20121 | 7.770253 | 8.420299 | 4.11E-16 | 8.56E-15 | 25.68306 |
| hsa-miR-937-3p | 1.200648 | 2.217581 | 5.891438 | 7.09E-09 | 7.16E-08 | 9.366164 |
| hsa-miR-34a-5p | 1.196278 | 7.549118 | 8.951588 | 7.12E-18 | 1.73E-16 | 29.67599 |
| hsa-miR-454-3p | 1.195861 | 2.682079 | 10.03669 | 1.07E-21 | 3.94E-20 | 38.36205 |
| hsa-miR-542-3p | 1.192322 | 7.607397 | 7.061379 | 5.63E-12 | 7.92E-11 | 16.33072 |
| hsa-miR-323b-3p | 1.164101 | 1.517305 | 4.995033 | 8.18E-07 | 6.39E-06 | 4.77426 |
| hsa-miR-653-5p | 1.163825 | 3.494594 | 5.030329 | 6.87E-07 | 5.42E-06 | 4.942271 |
| hsa-miR-616-5p | 1.153773 | 1.34772 | 8.018879 | 7.80E-15 | 1.44E-13 | 22.78776 |
| hsa-miR-625-5p | 1.149701 | 2.157384 | 9.407287 | 1.92E-19 | 5.17E-18 | 33.24005 |
| hsa-miR-147b-3p | 1.140466 | 1.135712 | 7.242606 | 1.70E-12 | 2.61E-11 | 17.50253 |
| hsa-miR-4677-3p | 1.133664 | 3.028653 | 10.07516 | 7.74E-22 | 3.08E-20 | 38.68225 |
| hsa-miR-22-5p | 1.130254 | 4.518867 | 8.324372 | 8.37E-16 | 1.70E-14 | 24.98146 |
| hsa-miR-148a-5p | 1.125542 | 5.73966 | 6.783638 | 3.36E-11 | 4.42E-10 | 14.5821 |
| hsa-miR-106a-5p | 1.111605 | 3.284437 | 6.05335 | 2.81E-09 | 2.94E-08 | 10.26608 |
| hsa-miR-214-5p | 1.102992 | 4.016069 | 6.992583 | 8.81E-12 | 1.22E-10 | 15.89223 |
| hsa-miR-203b-3p | 1.099321 | 4.8434 | 4.161349 | 3.73E-05 | 0.000218 | 1.121465 |
| hsa-miR-758-3p | 1.077338 | 2.616069 | 5.325831 | 1.53E-07 | 1.35E-06 | 6.390547 |
| hsa-miR-135b-3p | 1.074832 | 1.170982 | 9.180575 | 1.18E-18 | 3.02E-17 | 31.45127 |
| hsa-miR-505-5p | 1.073516 | 2.542795 | 7.417603 | 5.24E-13 | 8.39E-12 | 18.65675 |
| hsa-miR-423-3p | 1.051407 | 6.582684 | 8.07087 | 5.36E-15 | 9.95E-14 | 23.15668 |
| hsa-miR-4668-3p | 1.043529 | 1.189933 | 9.964229 | 1.97E-21 | 7.02E-20 | 37.76101 |
| hsa-miR-505-3p | 1.030356 | 4.980987 | 7.21886 | 1.99E-12 | 3.00E-11 | 17.34762 |
| hsa-miR-891a-5p | 1.029271 | 1.934069 | 3.860455 | 0.000128 | 0.000686 | -0.04495 |
| hsa-miR-183-3p | 1.028369 | 1.124669 | 8.6458 | 7.50E-17 | 1.71E-15 | 27.3559 |
| hsa-miR-889-3p | 1.0211 | 3.220769 | 4.636102 | 4.55E-06 | 3.13E-05 | 3.126791 |
| hsa-miR-382-5p | 1.013786 | 3.133481 | 5.225544 | 2.57E-07 | 2.17E-06 | 5.890734 |
| hsa-miR-24-2-5p | 1.00968 | 4.150448 | 7.591589 | 1.59E-13 | 2.73E-12 | 19.82606 |
| hsa-miR-766-3p | 1.008442 | 3.187263 | 6.562963 | 1.34E-10 | 1.69E-09 | 13.23429 |
| hsa-miR-191-5p | 0.987231 | 8.583117 | 7.809772 | 3.46E-14 | 6.18E-13 | 21.32258 |
| hsa-miR-92b-3p | 0.982628 | 7.398493 | 6.395292 | 3.72E-10 | 4.38E-09 | 12.23525 |
| hsa-miR-4326 | 0.982604 | 1.961675 | 4.958459 | 9.79E-07 | 7.57E-06 | 4.601288 |
| hsa-miR-3677-3p | 0.977365 | 2.126856 | 6.203652 | 1.17E-09 | 1.30E-08 | 11.12025 |
| hsa-miR-409-3p | 0.976759 | 3.258691 | 4.542596 | 6.99E-06 | 4.62E-05 | 2.716047 |
| hsa-miR-548v | 0.971691 | 1.229517 | 7.213218 | 2.07E-12 | 3.09E-11 | 17.31087 |
| hsa-miR-592 | 0.969288 | 1.387079 | 6.985868 | 9.20E-12 | 1.27E-10 | 15.84961 |
| hsa-miR-18a-5p | 0.965207 | 3.350292 | 5.671519 | 2.41E-08 | 2.30E-07 | 8.177861 |
| hsa-miR-628-5p | 0.958253 | 3.321617 | 7.81295 | 3.39E-14 | 6.09E-13 | 21.34462 |
| hsa-miR-1269b | 0.957574 | 0.949266 | 3.069336 | 0.002263 | 0.00868 | -2.71502 |
| hsa-miR-642a-5p | 0.953969 | 2.111634 | 5.657677 | 2.60E-08 | 2.47E-07 | 8.104392 |
| hsa-miR-4652-5p | 0.947903 | 0.922894 | 5.145468 | 3.86E-07 | 3.21E-06 | 5.497757 |
| hsa-miR-301b-3p | 0.943864 | 0.96671 | 7.233298 | 1.81E-12 | 2.76E-11 | 17.44176 |
| hsa-miR-30e-5p | 0.942798 | 12.39112 | 8.188092 | 2.28E-15 | 4.43E-14 | 23.99515 |
| hsa-miR-660-5p | 0.939527 | 5.3401 | 6.240873 | 9.37E-10 | 1.06E-08 | 11.33455 |
| hsa-miR-5698 | 0.928188 | 1.857185 | 4.470439 | 9.69E-06 | 6.21E-05 | 2.404342 |
| hsa-miR-142-5p | 0.919406 | 6.3139 | 5.225969 | 2.56E-07 | 2.17E-06 | 5.892835 |
| hsa-miR-128-3p | 0.91921 | 6.984002 | 8.657198 | 6.88E-17 | 1.58E-15 | 27.44132 |
| hsa-miR-651-5p | 0.91592 | 1.790608 | 7.457201 | 4.00E-13 | 6.55E-12 | 18.92098 |
| hsa-miR-589-3p | 0.914687 | 1.543557 | 7.229743 | 1.85E-12 | 2.81E-11 | 17.41856 |
| hsa-miR-16-1-3p | 0.909467 | 2.001791 | 9.467311 | 1.18E-19 | 3.30E-18 | 33.71873 |
| hsa-miR-550a-5p | 0.890145 | 1.975522 | 6.472814 | 2.32E-10 | 2.85E-09 | 12.69444 |
| hsa-miR-590-3p | 0.887958 | 2.373471 | 7.437435 | 4.58E-13 | 7.44E-12 | 18.78894 |
| hsa-miR-22-3p | 0.884662 | 16.09178 | 10.08989 | 6.84E-22 | 2.80E-20 | 38.80512 |
| hsa-miR-149-5p | 0.873533 | 4.376785 | 3.490586 | 0.000525 | 0.00246 | -1.36553 |
| hsa-miR-137-3p | 0.873488 | 0.864222 | 4.648454 | 4.30E-06 | 2.97E-05 | 3.181624 |
| hsa-miR-1287-5p | 0.872032 | 5.448198 | 6.071855 | 2.52E-09 | 2.67E-08 | 10.37028 |
| hsa-miR-493-3p | 0.864405 | 2.027683 | 4.974132 | 9.06E-07 | 7.03E-06 | 4.675269 |
| hsa-miR-20b-5p | 0.859251 | 3.357256 | 3.38299 | 0.000774 | 0.003416 | -1.72597 |
| hsa-miR-3127-5p | 0.855531 | 2.533159 | 6.406899 | 3.47E-10 | 4.14E-09 | 12.3037 |
| hsa-miR-576-5p | 0.854436 | 3.182993 | 6.976741 | 9.76E-12 | 1.33E-10 | 15.79175 |
| hsa-miR-4661-5p | 0.853619 | 1.972401 | 5.848498 | 9.03E-09 | 9.00E-08 | 9.131046 |
| hsa-miR-337-3p | 0.852032 | 3.67765 | 4.706019 | 3.28E-06 | 2.32E-05 | 3.438924 |
| hsa-miR-551b-3p | 0.851691 | 1.544381 | 4.45511 | 1.04E-05 | 6.60E-05 | 2.338717 |
| hsa-miR-181a-3p | 0.838685 | 8.308034 | 6.837715 | 2.38E-11 | 3.16E-10 | 14.91803 |
| hsa-miR-193b-5p | 0.830724 | 2.329513 | 5.166745 | 3.46E-07 | 2.89E-06 | 5.601644 |
| hsa-miR-2355-3p | 0.826415 | 2.535336 | 6.447565 | 2.71E-10 | 3.26E-09 | 12.54437 |
| hsa-miR-493-5p | 0.816561 | 2.45075 | 4.584146 | 5.78E-06 | 3.89E-05 | 2.897616 |
| hsa-miR-27b-5p | 0.810253 | 4.000486 | 8.282022 | 1.15E-15 | 2.30E-14 | 24.67364 |
| hsa-miR-340-3p | 0.803532 | 2.711183 | 7.200038 | 2.26E-12 | 3.35E-11 | 17.22513 |
| hsa-miR-24-1-5p | 0.803084 | 3.627925 | 6.210661 | 1.12E-09 | 1.26E-08 | 11.16053 |
| hsa-miR-323a-3p | 0.800379 | 1.359394 | 3.793127 | 0.000167 | 0.000878 | -0.29469 |
| hsa-miR-106b-3p | 0.799634 | 8.292797 | 5.699608 | 2.07E-08 | 1.99E-07 | 8.327436 |
| hsa-miR-134-5p | 0.796025 | 6.851769 | 3.44436 | 0.000621 | 0.002826 | -1.5217 |
| hsa-miR-3934-3p | 0.795485 | 1.780733 | 6.452327 | 2.63E-10 | 3.18E-09 | 12.57264 |
| hsa-miR-335-3p | 0.791176 | 6.557971 | 5.723277 | 1.81E-08 | 1.75E-07 | 8.453973 |
| hsa-miR-542-5p | 0.789681 | 1.493888 | 6.593269 | 1.11E-10 | 1.43E-09 | 13.41719 |
| hsa-miR-874-3p | 0.789361 | 4.442911 | 5.367087 | 1.23E-07 | 1.11E-06 | 6.598619 |
| hsa-miR-589-5p | 0.781373 | 6.437381 | 6.109669 | 2.03E-09 | 2.17E-08 | 10.58404 |
| hsa-miR-188-3p | 0.776456 | 0.89806 | 7.13236 | 3.53E-12 | 5.14E-11 | 16.7868 |
| hsa-miR-3170 | 0.776153 | 0.900879 | 7.474324 | 3.56E-13 | 5.87E-12 | 19.03559 |
| hsa-miR-186-5p | 0.774019 | 7.933223 | 8.617035 | 9.34E-17 | 2.06E-15 | 27.14069 |
| hsa-miR-181b-5p | 0.771626 | 9.334962 | 6.380328 | 4.07E-10 | 4.76E-09 | 12.14715 |
| hsa-miR-940 | 0.770803 | 1.679581 | 5.270734 | 2.03E-07 | 1.76E-06 | 6.114904 |
| hsa-miR-6510-3p | 0.768478 | 1.227291 | 4.129377 | 4.27E-05 | 0.000247 | 0.993641 |
| hsa-miR-487b-3p | 0.764141 | 1.982426 | 3.989295 | 7.63E-05 | 0.000424 | 0.444459 |
| hsa-miR-15a-5p | 0.763671 | 6.955388 | 6.582286 | 1.19E-10 | 1.52E-09 | 13.35083 |
| hsa-miR-3913-5p | 0.763235 | 2.055661 | 7.41118 | 5.47E-13 | 8.64E-12 | 18.61399 |
| hsa-miR-3200-3p | 0.761387 | 1.942204 | 4.685893 | 3.61E-06 | 2.52E-05 | 3.348637 |
| hsa-miR-103a-2-5p | 0.754942 | 1.025422 | 6.475475 | 2.29E-10 | 2.84E-09 | 12.71029 |
| hsa-miR-215-5p | 0.751775 | 2.843913 | 2.545938 | 0.011201 | 0.034634 | -4.15847 |
| hsa-miR-181d-5p | 0.751539 | 4.460732 | 5.811901 | 1.11E-08 | 1.08E-07 | 8.931847 |
| hsa-miR-449a | 0.750126 | 1.451001 | 3.047566 | 0.002431 | 0.009187 | -2.78024 |
| hsa-miR-1306-5p | 0.750067 | 2.89722 | 4.471434 | 9.65E-06 | 6.20E-05 | 2.408611 |
| hsa-miR-148b-5p | 0.749627 | 1.454102 | 8.104048 | 4.21E-15 | 7.89E-14 | 23.39306 |
| hsa-miR-532-3p | 0.741662 | 4.965908 | 4.034778 | 6.33E-05 | 0.000357 | 0.620829 |
| hsa-miR-200c-3p | 0.731534 | 13.078 | 4.95356 | 1.00E-06 | 7.72E-06 | 4.578208 |
| hsa-miR-501-3p | 0.722261 | 5.051116 | 4.568122 | 6.22E-06 | 4.17E-05 | 2.827411 |
| hsa-miR-28-5p | 0.719481 | 6.651967 | 8.325952 | 8.28E-16 | 1.69E-14 | 24.99297 |
| hsa-miR-20b-3p | 0.712677 | 0.802556 | 4.589458 | 5.64E-06 | 3.80E-05 | 2.920937 |
| hsa-miR-34a-3p | 0.709722 | 0.984662 | 8.495989 | 2.33E-16 | 5.00E-15 | 26.2409 |
| hsa-miR-582-3p | 0.707534 | 7.611092 | 3.042953 | 0.002467 | 0.00931 | -2.794 |
| hsa-miR-331-5p | 0.706751 | 2.237038 | 7.68184 | 8.50E-14 | 1.47E-12 | 20.44106 |
| hsa-miR-320b | 0.705574 | 2.21492 | 5.365962 | 1.24E-07 | 1.11E-06 | 6.592926 |
| hsa-let-7c-3p | 0.704953 | 2.43352 | 3.984868 | 7.77E-05 | 0.000431 | 0.427392 |
| hsa-miR-212-3p | 0.704602 | 2.941559 | 5.013496 | 7.46E-07 | 5.87E-06 | 4.86201 |
| hsa-miR-582-5p | 0.698358 | 3.575295 | 3.494229 | 0.000518 | 0.002438 | -1.35314 |
| hsa-miR-431-3p | 0.692426 | 2.369624 | 3.053599 | 0.002383 | 0.009038 | -2.76221 |
| hsa-let-7i-3p | 0.692296 | 6.546322 | 4.414433 | 1.24E-05 | 7.78E-05 | 2.165582 |
| hsa-miR-148b-3p | 0.681034 | 7.477313 | 6.474332 | 2.30E-10 | 2.84E-09 | 12.70348 |
| hsa-miR-615-3p | 0.680614 | 1.032069 | 3.8012 | 0.000162 | 0.000853 | -0.26496 |
| hsa-miR-17-3p | 0.677899 | 8.292016 | 5.480693 | 6.77E-08 | 6.23E-07 | 7.178943 |
| hsa-miR-369-3p | 0.676991 | 2.16809 | 3.445394 | 0.000619 | 0.002821 | -1.51823 |
| hsa-miR-154-3p | 0.675576 | 1.095239 | 4.481949 | 9.20E-06 | 5.95E-05 | 2.453758 |
| hsa-miR-628-3p | 0.673825 | 1.380659 | 5.656744 | 2.62E-08 | 2.47E-07 | 8.099444 |
| hsa-miR-3677-5p | 0.672288 | 1.138905 | 6.277387 | 7.55E-10 | 8.69E-09 | 11.54584 |
| hsa-miR-155-5p | 0.668887 | 8.4753 | 4.208319 | 3.06E-05 | 0.000182 | 1.310914 |
| hsa-miR-424-3p | 0.668671 | 1.807519 | 5.129396 | 4.18E-07 | 3.44E-06 | 5.419544 |
| hsa-miR-153-3p | 0.668478 | 1.108106 | 5.089846 | 5.10E-07 | 4.13E-06 | 5.228001 |
| hsa-miR-324-3p | 0.662531 | 4.34172 | 5.296002 | 1.79E-07 | 1.56E-06 | 6.240999 |
| hsa-miR-362-5p | 0.661435 | 2.937476 | 4.120209 | 4.44E-05 | 0.000256 | 0.957157 |
| hsa-miR-381-3p | 0.660456 | 4.724034 | 3.132463 | 0.001836 | 0.007263 | -2.52338 |
| hsa-miR-7705 | 0.66021 | 1.01579 | 6.636078 | 8.49E-11 | 1.10E-09 | 13.67673 |
| hsa-miR-29b-2-5p | 0.651492 | 4.83098 | 3.838759 | 0.00014 | 0.000743 | -0.12588 |
| hsa-miR-34b-5p | 0.650266 | 1.912093 | 2.402412 | 0.016656 | 0.048264 | -4.50856 |
| hsa-miR-4788 | 0.64413 | 0.840224 | 2.696283 | 0.007251 | 0.023577 | -3.77056 |
| hsa-miR-154-5p | 0.639964 | 1.538002 | 3.921762 | 0.0001 | 0.000548 | 0.186047 |
| hsa-miR-192-3p | 0.638487 | 1.571205 | 2.717457 | 0.00681 | 0.022439 | -3.71419 |
| hsa-miR-1266-5p | 0.634537 | 3.869519 | 3.770085 | 0.000183 | 0.000949 | -0.3792 |
| hsa-miR-224-3p | 0.634078 | 1.704093 | 4.054803 | 5.83E-05 | 0.00033 | 0.699073 |
| hsa-miR-106b-5p | 0.629372 | 7.88439 | 4.922597 | 1.17E-06 | 8.89E-06 | 4.432799 |
| hsa-miR-188-5p | 0.623328 | 1.304154 | 5.861784 | 8.38E-09 | 8.42E-08 | 9.203636 |
| hsa-miR-3189-3p | 0.62204 | 0.635564 | 5.245693 | 2.31E-07 | 2.00E-06 | 5.990471 |
| hsa-miR-28-3p | 0.614331 | 11.81673 | 6.396725 | 3.69E-10 | 4.36E-09 | 12.24369 |
| hsa-miR-616-3p | 0.610592 | 0.846797 | 6.128992 | 1.81E-09 | 1.96E-08 | 10.69372 |
| hsa-miR-92a-3p | 0.609662 | 13.03995 | 4.533634 | 7.28E-06 | 4.78E-05 | 2.677083 |
| hsa-miR-181c-3p | 0.607509 | 5.584175 | 5.032141 | 6.80E-07 | 5.39E-06 | 4.950928 |
| hsa-miR-1277-3p | 0.606507 | 0.816126 | 6.063066 | 2.66E-09 | 2.80E-08 | 10.32076 |
| hsa-miR-431-5p | 0.605162 | 1.127746 | 3.016592 | 0.002688 | 0.009989 | -2.87226 |
| hsa-miR-370-3p | 0.603512 | 1.979998 | 3.028097 | 0.00259 | 0.009705 | -2.83818 |
| hsa-miR-412-5p | 0.600188 | 1.534432 | 3.103205 | 0.002024 | 0.007885 | -2.61266 |
| hsa-miR-4724-5p | 0.599414 | 0.826209 | 5.297064 | 1.78E-07 | 1.56E-06 | 6.246311 |
| hsa-miR-664a-3p | 0.599116 | 4.562311 | 4.859438 | 1.58E-06 | 1.18E-05 | 4.138767 |
| hsa-miR-760 | 0.599022 | 1.325933 | 3.861786 | 0.000128 | 0.000684 | -0.03997 |
| hsa-miR-501-5p | 0.595178 | 2.134185 | 4.725864 | 2.99E-06 | 2.13E-05 | 3.528296 |
| hsa-miR-377-3p | 0.592834 | 0.68111 | 5.058076 | 5.98E-07 | 4.77E-06 | 5.075102 |
| hsa-miR-29c-5p | 0.588705 | 4.456167 | 4.14225 | 4.05E-05 | 0.000236 | 1.044995 |
| hsa-miR-432-5p | 0.581445 | 2.315683 | 2.809523 | 0.005158 | 0.017655 | -3.46412 |
| hsa-miR-15b-3p | 0.568517 | 4.623881 | 3.935432 | 9.50E-05 | 0.000524 | 0.238023 |
| hsa-miR-425-3p | 0.56788 | 3.378314 | 5.105755 | 4.71E-07 | 3.84E-06 | 5.304885 |
| hsa-miR-452-3p | 0.557754 | 1.953116 | 3.445821 | 0.000618 | 0.002821 | -1.5168 |
| hsa-miR-193a-3p | 0.555605 | 2.723701 | 4.218393 | 2.93E-05 | 0.000175 | 1.351806 |
| hsa-miR-130a-3p | 0.554152 | 5.921565 | 3.635355 | 0.000307 | 0.001524 | -0.86364 |
| hsa-miR-16-2-3p | 0.546414 | 2.957289 | 4.356113 | 1.61E-05 | 9.88E-05 | 1.919916 |
| hsa-miR-376b-3p | 0.545051 | 0.662709 | 4.511215 | 8.06E-06 | 5.26E-05 | 2.57992 |
| hsa-miR-4728-3p | 0.543562 | 1.487934 | 3.872284 | 0.000122 | 0.00066 | -0.00065 |
| hsa-miR-550a-3p | 0.542354 | 1.679613 | 4.058456 | 5.74E-05 | 0.000326 | 0.713384 |
| hsa-miR-361-3p | 0.541226 | 7.681785 | 4.900804 | 1.30E-06 | 9.85E-06 | 4.330956 |
| hsa-miR-155-3p | 0.538986 | 0.528441 | 6.46704 | 2.41E-10 | 2.94E-09 | 12.66008 |
| hsa-miR-495-3p | 0.537539 | 2.149152 | 2.748288 | 0.00621 | 0.020709 | -3.63134 |
| hsa-miR-1180-3p | 0.531983 | 4.592006 | 3.421247 | 0.000675 | 0.00304 | -1.59904 |
| hsa-miR-185-5p | 0.527472 | 6.264649 | 5.071789 | 5.59E-07 | 4.51E-06 | 5.140993 |
| hsa-miR-503-3p | 0.526366 | 0.764892 | 4.854253 | 1.62E-06 | 1.21E-05 | 4.114781 |
| hsa-miR-136-3p | 0.525347 | 2.786709 | 2.839501 | 0.004705 | 0.016407 | -3.38095 |
| hsa-miR-3136-5p | 0.522644 | 0.583405 | 6.951222 | 1.15E-11 | 1.56E-10 | 15.63029 |
| hsa-miR-767-3p | 0.516437 | 0.474148 | 3.151292 | 0.001724 | 0.006919 | -2.46549 |
| hsa-miR-130a-5p | 0.515374 | 0.703208 | 5.825378 | 1.03E-08 | 1.01E-07 | 9.005075 |
| hsa-miR-2277-5p | 0.512486 | 0.766544 | 5.513279 | 5.69E-08 | 5.28E-07 | 7.347388 |
| hsa-miR-769-3p | 0.511712 | 1.30736 | 5.060827 | 5.90E-07 | 4.73E-06 | 5.088306 |
| hsa-miR-579-5p | 0.510467 | 0.602908 | 6.006226 | 3.69E-09 | 3.79E-08 | 10.00199 |
| hsa-miR-210-5p | 0.508136 | 1.115806 | 4.732315 | 2.90E-06 | 2.08E-05 | 3.55742 |
| hsa-miR-655-3p | 0.504338 | 0.984304 | 3.776605 | 0.000178 | 0.00093 | -0.35534 |
| hsa-miR-561-5p | 0.503913 | 0.790055 | 4.122525 | 4.40E-05 | 0.000254 | 0.966364 |
| hsa-miR-605-5p | -0.50169 | 0.379912 | -6.87424 | 1.89E-11 | 2.53E-10 | 15.14619 |
| hsa-miR-374a-5p | -0.50953 | 4.91015 | -3.86663 | 0.000125 | 0.000673 | -0.02186 |
| hsa-miR-4529-3p | -0.51125 | 0.152578 | -14.0459 | 6.05E-38 | 9.56E-36 | 75.40547 |
| hsa-miR-6788-3p | -0.51389 | 0.178519 | -8.24677 | 1.48E-15 | 2.93E-14 | 24.41831 |
| hsa-miR-340-5p | -0.52242 | 4.189061 | -3.11071 | 0.001974 | 0.007781 | -2.58983 |
| hsa-miR-3154 | -0.53267 | 0.186249 | -9.84392 | 5.37E-21 | 1.75E-19 | 36.76956 |
| hsa-miR-195-3p | -0.54264 | 2.045183 | -4.97535 | 9.01E-07 | 7.01E-06 | 4.681028 |
| hsa-miR-99a-5p | -0.54447 | 8.073219 | -3.53372 | 0.000448 | 0.002174 | -1.21802 |
| hsa-miR-3614-5p | -0.54658 | 2.126896 | -4.01272 | 6.93E-05 | 0.000387 | 0.535047 |
| hsa-miR-30b-3p | -0.56942 | 2.175941 | -4.5153 | 7.92E-06 | 5.18E-05 | 2.597573 |
| hsa-miR-15b-5p | -0.57606 | 7.551749 | -4.90021 | 1.30E-06 | 9.85E-06 | 4.328188 |
| hsa-miR-378a-5p | -0.58517 | 5.474883 | -3.90071 | 0.000109 | 0.000593 | 0.106348 |
| hsa-let-7g-5p | -0.5898 | 9.256629 | -5.62814 | 3.06E-08 | 2.88E-07 | 7.948118 |
| hsa-miR-486-3p | -0.59626 | 0.611433 | -5.77551 | 1.36E-08 | 1.32E-07 | 8.734845 |
| hsa-miR-204-5p | -0.61945 | 2.008218 | -3.25552 | 0.00121 | 0.005094 | -2.13904 |
| hsa-miR-1258 | -0.62189 | 0.658365 | -6.97693 | 9.75E-12 | 1.33E-10 | 15.79297 |
| hsa-miR-517b-3p | -0.65302 | 0.257786 | -4.45134 | 1.06E-05 | 6.67E-05 | 2.322594 |
| hsa-miR-517a-3p | -0.65337 | 0.257821 | -4.45279 | 1.05E-05 | 6.65E-05 | 2.328816 |
| hsa-miR-223-3p | -0.70501 | 7.858045 | -3.82453 | 0.000148 | 0.000782 | -0.17873 |
| hsa-let-7d-3p | -0.73216 | 8.31392 | -5.06713 | 5.72E-07 | 4.60E-06 | 5.118566 |
| hsa-miR-2110 | -0.75185 | 1.439869 | -6.49523 | 2.03E-10 | 2.54E-09 | 12.82811 |
| hsa-miR-30d-5p | -0.7754 | 13.42167 | -5.27652 | 1.97E-07 | 1.72E-06 | 6.14373 |
| hsa-miR-34b-3p | -0.81117 | 2.363343 | -2.9507 | 0.003321 | 0.011806 | -3.06499 |
| hsa-miR-138-5p | -0.82185 | 1.904206 | -4.28966 | 2.15E-05 | 0.000131 | 1.643663 |
| hsa-miR-150-3p | -0.83787 | 1.891334 | -5.22991 | 2.51E-07 | 2.14E-06 | 5.912323 |
| hsa-miR-206 | -0.8466 | 0.383206 | -7.14106 | 3.33E-12 | 4.88E-11 | 16.84293 |
| hsa-miR-6720-3p | -0.89035 | 1.085083 | -7.30232 | 1.14E-12 | 1.76E-11 | 17.89387 |
| hsa-miR-598-3p | -0.89552 | 3.88273 | -6.12019 | 1.91E-09 | 2.06E-08 | 10.64371 |
| hsa-miR-490-3p | -0.89788 | 0.404607 | -9.06163 | 3.01E-18 | 7.39E-17 | 30.52515 |
| hsa-miR-34c-3p | -0.90911 | 3.922143 | -2.98111 | 0.003014 | 0.010978 | -2.97655 |
| hsa-let-7b-5p | -0.93429 | 13.79659 | -7.07876 | 5.02E-12 | 7.16E-11 | 16.44207 |
| hsa-let-7e-5p | -0.93455 | 10.43549 | -7.42567 | 4.96E-13 | 8.00E-12 | 18.71046 |
| hsa-miR-27a-5p | -0.9371 | 3.551005 | -5.61944 | 3.21E-08 | 3.00E-07 | 7.902242 |
| hsa-let-7b-3p | -0.94021 | 4.45642 | -7.41115 | 5.47E-13 | 8.64E-12 | 18.61376 |
| hsa-miR-140-3p | -0.94829 | 9.858598 | -9.91781 | 2.90E-21 | 9.87E-20 | 37.37756 |
| hsa-miR-423-5p | -0.96644 | 5.856653 | -7.54867 | 2.14E-13 | 3.64E-12 | 19.53561 |
| hsa-miR-218-5p | -0.98064 | 5.55707 | -6.01381 | 3.53E-09 | 3.66E-08 | 10.04438 |
| hsa-miR-1247-5p | -0.99991 | 2.235962 | -4.80308 | 2.08E-06 | 1.52E-05 | 3.879297 |
| hsa-miR-378c | -1.04855 | 2.495346 | -8.5656 | 1.38E-16 | 2.99E-15 | 26.75721 |
| hsa-miR-190a-5p | -1.06364 | 1.733306 | -9.13181 | 1.73E-18 | 4.40E-17 | 31.07052 |
| hsa-miR-125b-2-3p | -1.06967 | 3.443485 | -7.07683 | 5.09E-12 | 7.21E-11 | 16.42965 |
| hsa-miR-145-3p | -1.07181 | 4.968789 | -8.34582 | 7.14E-16 | 1.48E-14 | 25.13779 |
| hsa-miR-218-1-3p | -1.09218 | 1.057667 | -11.5192 | 2.33E-27 | 1.32E-25 | 51.25314 |
| hsa-miR-221-5p | -1.12375 | 2.105021 | -6.46122 | 2.49E-10 | 3.03E-09 | 12.62545 |
| hsa-miR-516a-5p | -1.12641 | 0.954547 | -5.67922 | 2.31E-08 | 2.21E-07 | 8.218794 |
| hsa-let-7f-5p | -1.21263 | 13.24085 | -7.09928 | 4.39E-12 | 6.30E-11 | 16.57382 |
| hsa-miR-195-5p | -1.23709 | 5.118554 | -7.7122 | 6.88E-14 | 1.20E-12 | 20.64926 |
| hsa-miR-7704 | -1.30739 | 1.400454 | -8.12816 | 3.53E-15 | 6.74E-14 | 23.56529 |
| hsa-miR-6892-5p | -1.30815 | 1.681796 | -10.0338 | 1.10E-21 | 3.98E-20 | 38.3379 |
| hsa-miR-584-5p | -1.31012 | 4.692228 | -6.8486 | 2.22E-11 | 2.96E-10 | 14.9859 |
| hsa-miR-139-5p | -1.33227 | 5.104103 | -7.5424 | 2.23E-13 | 3.77E-12 | 19.49332 |
| hsa-miR-4732-3p | -1.41622 | 0.553094 | -13.2706 | 1.37E-34 | 1.78E-32 | 67.74592 |
| hsa-miR-133b | -1.42138 | 0.990612 | -11.9054 | 6.61E-29 | 4.43E-27 | 54.78224 |
| hsa-let-7a-5p | -1.42825 | 14.9342 | -12.1729 | 5.39E-30 | 4.11E-28 | 57.26414 |
| hsa-miR-30a-5p | -1.55034 | 13.53647 | -8.42232 | 4.04E-16 | 8.51E-15 | 25.69793 |
| hsa-miR-338-5p | -1.57226 | 3.245586 | -7.80026 | 3.70E-14 | 6.55E-13 | 21.25666 |
| hsa-miR-378a-3p | -1.61644 | 7.623553 | -10.9813 | 2.99E-25 | 1.47E-23 | 46.45014 |
| hsa-let-7c-5p | -1.76312 | 10.18401 | -11.2276 | 3.30E-26 | 1.66E-24 | 48.63264 |
| hsa-miR-133a-3p | -1.79195 | 2.635493 | -8.93627 | 8.02E-18 | 1.93E-16 | 29.55834 |
| hsa-miR-1-3p | -1.79629 | 3.223845 | -8.22321 | 1.76E-15 | 3.45E-14 | 24.24815 |
| hsa-miR-143-3p | -2.02339 | 16.20807 | -11.3482 | 1.11E-26 | 5.83E-25 | 49.71192 |
| hsa-miR-144-3p | -2.17317 | 3.067338 | -9.6758 | 2.15E-20 | 6.53E-19 | 35.39764 |
| hsa-miR-1247-3p | -2.27662 | 3.210498 | -9.11286 | 2.01E-18 | 5.05E-17 | 30.92293 |
| hsa-miR-451a | -2.4248 | 8.614892 | -9.47778 | 1.08E-19 | 3.07E-18 | 33.80243 |
| hsa-miR-30a-3p | -2.49361 | 11.78161 | -13.2284 | 2.08E-34 | 2.55E-32 | 67.33514 |
| hsa-miR-139-3p | -2.65721 | 3.678032 | -15.5741 | 8.88E-45 | 1.96E-42 | 91.01341 |
| hsa-miR-144-5p | -2.67147 | 6.291284 | -10.6706 | 4.64E-24 | 2.14E-22 | 43.73923 |
| hsa-miR-30c-2-3p | -2.70245 | 4.924497 | -15.6386 | 4.51E-45 | 1.25E-42 | 91.68512 |
| hsa-miR-184 | -2.90022 | 2.017879 | -12.5359 | 1.71E-31 | 1.58E-29 | 60.68 |
| hsa-miR-486-5p | -3.50515 | 6.860455 | -14.4293 | 1.24E-39 | 2.11E-37 | 79.26157 |
