# Supplementary material for: Prognostic value of lncRNAs related to fatty acid metabolism in lung adenocarcinoma and their correlation with tumor microenvironment based on bioinformatics analysis
Source: Front Oncol. 2022 Oct 10;12:1022097. doi: 10.3389/fonc.2022.1022097 (PMC9590110; doi:10.3389/fonc.2022.1022097)
Supplement: Supplementary Table 1 — All samples were divided into high and low fatty acid metabolism score groups based on the median value of this score. [file DataSheet_1.zip › raw data and R code for checking/raw data/4.docx]

| lncRNA | module |
| --- | --- |
| A2M-AS1 | yellow |
| AC000403.4 | yellow |
| AC002066.1 | yellow |
| AC002076.10 | yellow |
| AC002398.12 | yellow |
| AC002454.1 | magenta |
| AC003090.1 | yellow |
| AC004112.4 | yellow |
| AC004490.1 | yellow |
| AC004540.4 | yellow |
| AC004540.5 | yellow |
| AC004791.2 | yellow |
| AC004870.4 | yellow |
| AC004947.2 | yellow |
| AC005256.1 | magenta |
| AC005264.2 | yellow |
| AC005330.2 | yellow |
| AC005562.1 | yellow |
| AC005624.2 | yellow |
| AC006126.4 | yellow |
| AC006129.1 | yellow |
| AC006159.5 | yellow |
| AC006273.4 | yellow |
| AC006273.5 | yellow |
| AC007163.3 | yellow |
| AC007182.6 | yellow |
| AC007277.3 | yellow |
| AC007392.3 | yellow |
| AC007405.6 | yellow |
| AC007743.1 | yellow |
| AC007879.7 | magenta |
| AC008074.3 | yellow |
| AC008268.1 | yellow |
| AC009005.2 | yellow |
| AC009227.2 | yellow |
| AC010226.4 | yellow |
| AC010524.2 | yellow |
| AC011294.3 | yellow |
| AC012668.2 | yellow |
| AC016747.3 | yellow |
| AC016910.1 | yellow |
| AC018647.3 | yellow |
| AC018816.3 | yellow |
| AC020571.3 | magenta |
| AC023115.2 | yellow |
| AC023669.1 | yellow |
| AC025335.1 | yellow |
| AC067959.1 | yellow |
| AC072062.1 | yellow |
| AC073130.1 | magenta |
| AC078942.1 | yellow |
| AC079354.3 | yellow |
| AC079354.5 | yellow |
| AC079630.2 | yellow |
| AC079630.4 | yellow |
| AC079779.4 | yellow |
| AC090044.2 | yellow |
| AC090616.2 | yellow |
| AC092162.1 | yellow |
| AC092431.3 | yellow |
| AC092667.2 | yellow |
| AC093110.3 | yellow |
| AC093609.1 | yellow |
| AC093673.5 | magenta |
| AC093850.2 | magenta |
| AC096772.6 | yellow |
| AC099850.1 | yellow |
| AC103563.8 | yellow |
| AC104654.2 | yellow |
| AC104655.3 | magenta |
| AC105053.3 | yellow |
| AC106869.2 | yellow |
| AC108463.1 | magenta |
| AC109642.1 | yellow |
| AC112715.2 | magenta |
| AC112721.1 | magenta |
| AC112721.2 | magenta |
| AC115522.3 | magenta |
| AC116035.1 | yellow |
| AC123023.1 | yellow |
| AC123886.2 | yellow |
| AC124789.1 | yellow |
| AC124861.1 | yellow |
| AC128709.2 | yellow |
| AC128709.4 | yellow |
| AC133785.1 | yellow |
| AC135178.7 | yellow |
| AC141928.1 | yellow |
| AC142293.3 | magenta |
| AC144449.1 | yellow |
| AC144831.1 | yellow |
| AC144831.3 | yellow |
| AC145124.2 | yellow |
| AC145343.2 | yellow |
| ADAMTS9-AS1 | yellow |
| ADAMTS9-AS2 | yellow |
| ADD3-AS1 | yellow |
| AF064858.7 | yellow |
| AF131215.2 | yellow |
| AF131215.8 | yellow |
| AF131215.9 | yellow |
| AFAP1-AS1 | yellow |
| AGAP1-IT1 | yellow |
| AGAP11 | yellow |
| AGAP2-AS1 | magenta |
| AJ011932.1 | yellow |
| AL022344.7 | yellow |
| AL035610.1 | yellow |
| AL162759.1 | yellow |
| AL450992.2 | yellow |
| AP000223.42 | yellow |
| AP000438.2 | yellow |
| AP000662.4 | yellow |
| AP000695.4 | magenta |
| AP000695.6 | magenta |
| AP001189.4 | yellow |
| AP001434.2 | magenta |
| AP001626.1 | yellow |
| AP001631.9 | yellow |
| APCDD1L-AS1 | magenta |
| APOA1-AS | yellow |
| ATP13A4-AS1 | yellow |
| ATP6V1B1-AS1 | magenta |
| B4GALT1-AS1 | yellow |
| BANCR | yellow |
| C10orf25 | yellow |
| C10orf91 | yellow |
| C14orf132 | yellow |
| C1QTNF1-AS1 | yellow |
| C1orf140 | yellow |
| C22orf34 | yellow |
| C6orf3 | yellow |
| CADM3-AS1 | yellow |
| CASC9 | yellow |
| CERS3-AS1 | yellow |
| CH17-360D5.2 | yellow |
| CH17-360D5.3 | yellow |
| CH507-154B10.2 | yellow |
| CITF22-62D4.1 | yellow |
| CLDN10-AS1 | yellow |
| CMB9-22P13.1 | yellow |
| COL4A2-AS1 | yellow |
| CTA-384D8.34 | yellow |
| CTA-384D8.35 | yellow |
| CTA-989H11.1 | yellow |
| CTB-111H14.1 | magenta |
| CTB-151G24.1 | yellow |
| CTB-25B13.5 | yellow |
| CTB-30L5.1 | magenta |
| CTB-36H16.2 | yellow |
| CTB-39G8.2 | yellow |
| CTB-43E15.1 | yellow |
| CTB-66B24.1 | yellow |
| CTC-205M6.5 | yellow |
| CTC-232P5.3 | yellow |
| CTC-241F20.4 | yellow |
| CTC-265F19.3 | yellow |
| CTC-296K1.3 | yellow |
| CTC-296K1.4 | yellow |
| CTC-297N7.9 | yellow |
| CTC-321K16.1 | yellow |
| CTC-327F10.4 | magenta |
| CTC-327F10.5 | magenta |
| CTC-332L22.1 | yellow |
| CTC-431G16.2 | yellow |
| CTC-518P12.6 | magenta |
| CTC-526N19.1 | yellow |
| CTC-548K16.1 | yellow |
| CTC-558O2.1 | yellow |
| CTC-558O2.2 | yellow |
| CTC-786C10.1 | yellow |
| CTD-2006K23.1 | yellow |
| CTD-2008P7.9 | yellow |
| CTD-2012K14.8 | yellow |
| CTD-2014B16.3 | magenta |
| CTD-2014B16.5 | magenta |
| CTD-2015H6.3 | yellow |
| CTD-2016O11.1 | yellow |
| CTD-2021J15.1 | magenta |
| CTD-2037L6.2 | yellow |
| CTD-2054N24.2 | yellow |
| CTD-2066L21.3 | yellow |
| CTD-2078B5.2 | yellow |
| CTD-2091N23.1 | magenta |
| CTD-2130O13.1 | magenta |
| CTD-2135D7.5 | yellow |
| CTD-2135J3.3 | yellow |
| CTD-2171N6.1 | magenta |
| CTD-2201G3.1 | yellow |
| CTD-2207P18.2 | magenta |
| CTD-2235C13.3 | yellow |
| CTD-2269F5.1 | yellow |
| CTD-2298J14.2 | yellow |
| CTD-2313J17.6 | yellow |
| CTD-2319I12.5 | yellow |
| CTD-2334D19.1 | magenta |
| CTD-2357A8.3 | yellow |
| CTD-2369P2.8 | yellow |
| CTD-2373J6.1 | yellow |
| CTD-2510F5.4 | yellow |
| CTD-2515H24.2 | yellow |
| CTD-2523D13.2 | yellow |
| CTD-2524L6.3 | yellow |
| CTD-2530N21.5 | yellow |
| CTD-2532K18.2 | magenta |
| CTD-2547H18.1 | yellow |
| CTD-2547L16.1 | yellow |
| CTD-2553C6.1 | magenta |
| CTD-2562J17.4 | yellow |
| CTD-2562J17.7 | yellow |
| CTD-2566J3.1 | yellow |
| CTD-2587H24.5 | magenta |
| CTD-2588E21.1 | yellow |
| CTD-2589M5.4 | yellow |
| CTD-2595P9.4 | yellow |
| CTD-2636A23.2 | yellow |
| CTD-3010D24.3 | yellow |
| CTD-3035D6.2 | yellow |
| CTD-3060P21.1 | yellow |
| CTD-3064M3.3 | yellow |
| CTD-3076O17.1 | yellow |
| CTD-3116E22.8 | yellow |
| CTD-3179P9.1 | yellow |
| CTD-3193K9.11 | yellow |
| CTD-3224I3.3 | yellow |
| CTD-3247F14.2 | yellow |
| DEPDC1-AS1 | yellow |
| DIAPH2-AS1 | magenta |
| DKFZp779M0652 | yellow |
| DPH6-AS1 | yellow |
| ELFN1-AS1 | yellow |
| EP300-AS1 | yellow |
| EPB41L4A-AS2 | yellow |
| F11-AS1 | yellow |
| FAM212B-AS1 | yellow |
| FAM222A-AS1 | yellow |
| FAM225A | magenta |
| FAM225B | magenta |
| FAM66C | yellow |
| FAM83A-AS1 | yellow |
| FAM83H-AS1 | yellow |
| FAM85B | yellow |
| FENDRR | yellow |
| FGD5-AS1 | yellow |
| FGF14-AS2 | yellow |
| FLJ22447 | magenta |
| FLJ38122 | yellow |
| FLJ43879 | magenta |
| FNDC1-IT1 | magenta |
| FOXD3-AS1 | yellow |
| GAS5 | yellow |
| GAS6-AS2 | yellow |
| GATA2-AS1 | yellow |
| GATA6-AS1 | yellow |
| GLIDR | yellow |
| GRM5-AS1 | yellow |
| GS1-115G20.1 | yellow |
| GS1-166A23.1 | yellow |
| GS1-279B7.2 | yellow |
| GS1-421I3.2 | yellow |
| GS1-600G8.5 | yellow |
| HAND2-AS1 | yellow |
| HHIP-AS1 | yellow |
| HID1-AS1 | yellow |
| HIF1A-AS1 | magenta |
| HLX-AS1 | yellow |
| IGFBP7-AS1 | yellow |
| INAFM2 | yellow |
| INHBA-AS1 | yellow |
| ITGA9-AS1 | yellow |
| JAZF1-AS1 | yellow |
| KB-1448A5.1 | yellow |
| KB-1460A1.1 | magenta |
| KB-1958F4.1 | yellow |
| KB-68A7.1 | yellow |
| KCNC4-AS1 | yellow |
| KCNQ1-AS1 | yellow |
| KTN1-AS1 | yellow |
| LANCL1-AS1 | yellow |
| LBX1-AS1 | yellow |
| LDLRAD4-AS1 | yellow |
| LEF1-AS1 | magenta |
| LHFPL3-AS1 | yellow |
| LHFPL3-AS2 | yellow |
| LINC00028 | yellow |
| LINC00032 | yellow |
| LINC00087 | yellow |
| LINC00092 | yellow |
| LINC00114 | yellow |
| LINC00152 | magenta |
| LINC00160 | yellow |
| LINC00162 | yellow |
| LINC00163 | yellow |
| LINC00165 | yellow |
| LINC00211 | yellow |
| LINC00261 | yellow |
| LINC00315 | yellow |
| LINC00337 | yellow |
| LINC00346 | yellow |
| LINC00359 | yellow |
| LINC00365 | yellow |
| LINC00460 | magenta |
| LINC00472 | yellow |
| LINC00505 | yellow |
| LINC00511 | yellow |
| LINC00519 | magenta |
| LINC00524 | yellow |
| LINC00540 | yellow |
| LINC00551 | yellow |
| LINC00607 | yellow |
| LINC00619 | yellow |
| LINC00622 | yellow |
| LINC00632 | yellow |
| LINC00636 | yellow |
| LINC00639 | yellow |
| LINC00656 | yellow |
| LINC00670 | yellow |
| LINC00694 | yellow |
| LINC00702 | yellow |
| LINC00840 | yellow |
| LINC00844 | yellow |
| LINC00857 | yellow |
| LINC00862 | yellow |
| LINC00863 | yellow |
| LINC00880 | magenta |
| LINC00887 | yellow |
| LINC00890 | yellow |
| LINC00891 | yellow |
| LINC00920 | yellow |
| LINC00924 | yellow |
| LINC00935 | yellow |
| LINC00936 | yellow |
| LINC00959 | yellow |
| LINC00961 | yellow |
| LINC00968 | yellow |
| LINC00973 | magenta |
| LINC00987 | yellow |
| LINC00989 | yellow |
| LINC01016 | yellow |
| LINC01050 | magenta |
| LINC01060 | magenta |
| LINC01082 | yellow |
| LINC01096 | magenta |
| LINC01099 | yellow |
| LINC01105 | yellow |
| LINC01108 | yellow |
| LINC01116 | yellow |
| LINC01117 | yellow |
| LINC01140 | yellow |
| LINC01141 | magenta |
| LINC01165 | yellow |
| LINC01169 | yellow |
| LINC01197 | yellow |
| LINC01204 | magenta |
| LINC01207 | yellow |
| LINC01213 | magenta |
| LINC01214 | magenta |
| LINC01234 | yellow |
| LINC01266 | yellow |
| LINC01268 | yellow |
| LINC01269 | yellow |
| LINC01290 | yellow |
| LINC01314 | yellow |
| LINC01352 | yellow |
| LINC01354 | yellow |
| LINC01356 | yellow |
| LINC01366 | yellow |
| LINC01394 | yellow |
| LINC01412 | yellow |
| LINC01426 | yellow |
| LINC01429 | magenta |
| LINC01447 | yellow |
| LINC01500 | magenta |
| LINC01503 | yellow |
| LINC01504 | yellow |
| LINC01510 | yellow |
| LINC01537 | yellow |
| LINC01556 | yellow |
| LINC01561 | magenta |
| LINC01583 | magenta |
| LL0XNC01-250H12.3 | yellow |
| LMF1-AS1 | yellow |
| LUCAT1 | magenta |
| LYPLAL1-AS1 | yellow |
| MAFG-AS1 | yellow |
| MAGI2-AS3 | yellow |
| MBNL1-AS1 | yellow |
| MED4-AS1 | yellow |
| MGAT3-AS1 | yellow |
| MGC27382 | yellow |
| MID1IP1-AS1 | magenta |
| MIR100HG | magenta |
| MIR22HG | yellow |
| MIR31HG | magenta |
| MIR4435-1HG | magenta |
| MIR497HG | yellow |
| MKLN1-AS | yellow |
| MNX1-AS1 | yellow |
| MRGPRF-AS1 | yellow |
| MTHFS | yellow |
| MYHAS | magenta |
| MYO16-AS1 | yellow |
| NAALADL2-AS2 | magenta |
| NALCN-AS1 | yellow |
| NAV2-AS2 | yellow |
| NFE4 | magenta |
| NFIA-AS2 | yellow |
| NR2F2-AS1 | yellow |
| OGFRP1 | yellow |
| OVCH1-AS1 | yellow |
| P3H2-AS1 | yellow |
| P4HA2-AS1 | yellow |
| PCAT19 | yellow |
| PGM5-AS1 | yellow |
| PGM5P3-AS1 | yellow |
| PGM5P4-AS1 | yellow |
| PIK3CD-AS2 | yellow |
| PKI55 | yellow |
| POT1-AS1 | yellow |
| PRKCQ-AS1 | yellow |
| PRRT3-AS1 | yellow |
| PTPRD-AS1 | yellow |
| PTPRG-AS1 | yellow |
| RAMP2-AS1 | yellow |
| RASAL2-AS1 | magenta |
| RBMS3-AS3 | yellow |
| RBPMS-AS1 | yellow |
| RMST | yellow |
| RP1-117B12.4 | yellow |
| RP1-124C6.1 | yellow |
| RP1-137D17.1 | yellow |
| RP1-140C12.2 | yellow |
| RP1-142L7.5 | yellow |
| RP1-153G14.4 | yellow |
| RP1-15D23.2 | yellow |
| RP1-163G9.2 | yellow |
| RP1-167F1.2 | yellow |
| RP1-16A9.1 | magenta |
| RP1-18D14.7 | yellow |
| RP1-228H13.5 | yellow |
| RP1-27K12.2 | yellow |
| RP1-29C18.8 | yellow |
| RP1-29C18.9 | yellow |
| RP1-309I22.2 | yellow |
| RP1-310O13.7 | yellow |
| RP1-32I10.10 | yellow |
| RP1-34H18.1 | yellow |
| RP1-78O14.1 | yellow |
| RP1-79C4.4 | magenta |
| RP1-93H18.1 | magenta |
| RP11-1000B6.3 | yellow |
| RP11-1008C21.2 | yellow |
| RP11-100E13.1 | magenta |
| RP11-100L22.1 | yellow |
| RP11-1024P17.1 | yellow |
| RP11-102N12.3 | yellow |
| RP11-1060G2.1 | yellow |
| RP11-1060J15.9 | yellow |
| RP11-1069G10.2 | yellow |
| RP11-1090M7.1 | yellow |
| RP11-109N23.4 | yellow |
| RP11-10A14.5 | yellow |
| RP11-10C24.1 | yellow |
| RP11-10C24.2 | yellow |
| RP11-10C24.3 | yellow |
| RP11-111E14.1 | yellow |
| RP11-1149O23.2 | yellow |
| RP11-1149O23.3 | yellow |
| RP11-115C10.1 | magenta |
| RP11-115H18.1 | yellow |
| RP11-115N4.1 | yellow |
| RP11-119J18.1 | yellow |
| RP11-120D5.1 | yellow |
| RP11-124N14.3 | yellow |
| RP11-124O11.1 | yellow |
| RP11-125O18.1 | yellow |
| RP11-127I20.5 | yellow |
| RP11-1293J14.1 | yellow |
| RP11-12A2.3 | yellow |
| RP11-12D24.10 | yellow |
| RP11-12G12.7 | yellow |
| RP11-132A1.4 | yellow |
| RP11-133F8.2 | yellow |
| RP11-133L19.3 | yellow |
| RP11-136H19.1 | yellow |
| RP11-138I1.2 | yellow |
| RP11-13P5.2 | magenta |
| RP11-141J13.5 | yellow |
| RP11-141M1.3 | yellow |
| RP11-143J24.1 | yellow |
| RP11-145A3.1 | magenta |
| RP11-146D12.2 | yellow |
| RP11-148B18.3 | magenta |
| RP11-148B18.4 | magenta |
| RP11-14D22.1 | magenta |
| RP11-14N7.2 | magenta |
| RP11-150C16.1 | yellow |
| RP11-150O12.1 | magenta |
| RP11-150O12.6 | magenta |
| RP11-154H12.2 | yellow |
| RP11-161I6.2 | yellow |
| RP11-164O23.8 | yellow |
| RP11-166D19.1 | magenta |
| RP11-16K12.1 | yellow |
| RP11-175K6.1 | yellow |
| RP11-17A4.2 | yellow |
| RP11-180I4.4 | yellow |
| RP11-180N14.1 | yellow |
| RP11-182J1.1 | yellow |
| RP11-182J1.17 | yellow |
| RP11-187E13.2 | yellow |
| RP11-187O7.3 | yellow |
| RP11-192P3.4 | yellow |
| RP11-195F19.9 | yellow |
| RP11-203F10.5 | yellow |
| RP11-20D14.6 | yellow |
| RP11-20E24.1 | yellow |
| RP11-211C9.1 | yellow |
| RP11-213H15.1 | yellow |
| RP11-214F16.8 | yellow |
| RP11-217B1.2 | yellow |
| RP11-218M22.1 | yellow |
| RP11-21L23.2 | yellow |
| RP11-221N13.3 | magenta |
| RP11-229P13.19 | yellow |
| RP11-230G5.2 | magenta |
| RP11-236L14.2 | yellow |
| RP11-238K6.1 | yellow |
| RP11-23D24.2 | yellow |
| RP11-244M2.1 | yellow |
| RP11-246K15.1 | yellow |
| RP11-247C2.2 | magenta |
| RP11-251M1.1 | yellow |
| RP11-253E3.3 | yellow |
| RP11-254F19.2 | yellow |
| RP11-254F7.3 | yellow |
| RP11-254I22.3 | yellow |
| RP11-257O5.4 | yellow |
| RP11-259K15.2 | yellow |
| RP11-262A16.1 | yellow |
| RP11-264B14.1 | yellow |
| RP11-264E20.1 | magenta |
| RP11-264E20.2 | magenta |
| RP11-272L14.2 | magenta |
| RP11-276H19.1 | yellow |
| RP11-27M24.2 | yellow |
| RP11-27N21.3 | yellow |
| RP11-284F21.10 | yellow |
| RP11-284F21.7 | yellow |
| RP11-284F21.9 | yellow |
| RP11-286H15.1 | yellow |
| RP11-290O12.2 | magenta |
| RP11-293M10.6 | yellow |
| RP11-293P20.2 | yellow |
| RP11-295G20.2 | yellow |
| RP11-295M18.6 | yellow |
| RP11-2N1.2 | yellow |
| RP11-302L19.3 | yellow |
| RP11-303E16.2 | yellow |
| RP11-304L19.1 | yellow |
| RP11-304L19.3 | yellow |
| RP11-305D15.8 | yellow |
| RP11-305L7.6 | yellow |
| RP11-307C18.1 | yellow |
| RP11-307L14.1 | yellow |
| RP11-307L14.2 | yellow |
| RP11-308N19.1 | magenta |
| RP11-309M7.1 | magenta |
| RP11-30K9.5 | yellow |
| RP11-312J18.6 | yellow |
| RP11-314C16.1 | yellow |
| RP11-314O13.1 | yellow |
| RP11-317J10.2 | yellow |
| RP11-31F15.2 | yellow |
| RP11-31I22.3 | yellow |
| RP11-322E11.2 | yellow |
| RP11-322E11.5 | yellow |
| RP11-324L17.1 | yellow |
| RP11-325L12.6 | yellow |
| RP11-327J17.9 | yellow |
| RP11-327L3.3 | yellow |
| RP11-332J15.3 | yellow |
| RP11-335L23.5 | yellow |
| RP11-336A10.5 | magenta |
| RP11-336K24.5 | yellow |
| RP11-33A14.1 | yellow |
| RP11-342A23.2 | yellow |
| RP11-344B5.2 | yellow |
| RP11-345M22.2 | yellow |
| RP11-347E10.1 | yellow |
| RP11-348F1.2 | yellow |
| RP11-348F1.3 | yellow |
| RP11-350J20.12 | yellow |
| RP11-352D13.5 | yellow |
| RP11-352D13.6 | yellow |
| RP11-353N4.5 | yellow |
| RP11-354E11.2 | yellow |
| RP11-354P11.2 | yellow |
| RP11-354P11.4 | yellow |
| RP11-357D18.1 | yellow |
| RP11-359E10.1 | magenta |
| RP11-359G22.2 | magenta |
| RP11-359M6.1 | yellow |
| RP11-359N11.1 | yellow |
| RP11-35J10.7 | yellow |
| RP11-366L20.2 | magenta |
| RP11-366L5.1 | yellow |
| RP11-370I10.2 | yellow |
| RP11-371A19.2 | yellow |
| RP11-375I20.6 | yellow |
| RP11-378A13.1 | yellow |
| RP11-37L2.1 | yellow |
| RP11-380B4.3 | yellow |
| RP11-383H13.1 | magenta |
| RP11-383J24.1 | yellow |
| RP11-384F7.2 | yellow |
| RP11-384O8.1 | yellow |
| RP11-384P7.7 | yellow |
| RP11-387H17.6 | yellow |
| RP11-389C8.2 | yellow |
| RP11-38L15.3 | yellow |
| RP11-38M8.1 | yellow |
| RP11-394I13.1 | yellow |
| RP11-396F22.1 | yellow |
| RP11-397O8.7 | yellow |
| RP11-3B12.5 | yellow |
| RP11-400F19.6 | yellow |
| RP11-400K9.3 | yellow |
| RP11-400N13.3 | magenta |
| RP11-401O9.3 | magenta |
| RP11-401O9.4 | magenta |
| RP11-401P9.4 | yellow |
| RP11-401P9.6 | yellow |
| RP11-403A3.3 | yellow |
| RP11-414H23.3 | magenta |
| RP11-416I2.1 | yellow |
| RP11-417E7.1 | magenta |
| RP11-417E7.2 | magenta |
| RP11-419J16.1 | yellow |
| RP11-426C22.4 | magenta |
| RP11-428J1.4 | yellow |
| RP11-428J1.5 | yellow |
| RP11-429E11.3 | yellow |
| RP11-431J24.2 | magenta |
| RP11-432J24.2 | yellow |
| RP11-434D9.1 | yellow |
| RP11-434H14.1 | yellow |
| RP11-435O5.2 | yellow |
| RP11-438B23.2 | magenta |
| RP11-439L18.1 | yellow |
| RP11-439L18.2 | yellow |
| RP11-43F13.3 | yellow |
| RP11-440D17.3 | yellow |
| RP11-442O1.3 | yellow |
| RP11-443C10.1 | magenta |
| RP11-446H18.5 | yellow |
| RP11-449D8.1 | yellow |
| RP11-44B19.1 | yellow |
| RP11-44F14.2 | yellow |
| RP11-44F14.8 | yellow |
| RP11-452I5.2 | yellow |
| RP11-454K7.3 | yellow |
| RP11-455B3.1 | yellow |
| RP11-455O6.2 | yellow |
| RP11-456K23.1 | yellow |
| RP11-459E5.1 | yellow |
| RP11-462G12.1 | yellow |
| RP11-462G12.2 | yellow |
| RP11-473E2.4 | yellow |
| RP11-474I16.8 | yellow |
| RP11-475O23.2 | yellow |
| RP11-476D10.1 | yellow |
| RP11-479J7.2 | magenta |
| RP11-47I22.2 | magenta |
| RP11-484L8.1 | yellow |
| RP11-490M8.1 | yellow |
| RP11-492E3.2 | yellow |
| RP11-493L12.3 | magenta |
| RP11-493L12.5 | yellow |
| RP11-494M8.4 | yellow |
| RP11-49G2.3 | yellow |
| RP11-4B16.3 | yellow |
| RP11-500C11.3 | magenta |
| RP11-501J20.5 | yellow |
| RP11-506B6.6 | yellow |
| RP11-507K2.3 | yellow |
| RP11-512N21.3 | yellow |
| RP11-513M16.8 | yellow |
| RP11-513N24.1 | yellow |
| RP11-514D23.3 | yellow |
| RP11-517P14.2 | yellow |
| RP11-519G16.3 | yellow |
| RP11-519G16.5 | yellow |
| RP11-51B23.3 | yellow |
| RP11-521D12.5 | yellow |
| RP11-522B15.3 | yellow |
| RP11-522B15.4 | yellow |
| RP11-524D16__A.3 | magenta |
| RP11-527N22.2 | magenta |
| RP11-528A4.2 | yellow |
| RP11-532F6.3 | yellow |
| RP11-535A5.1 | yellow |
| RP11-536G4.2 | yellow |
| RP11-536O18.1 | yellow |
| RP11-538I12.3 | magenta |
| RP11-539E17.5 | yellow |
| RP11-53M11.3 | magenta |
| RP11-53O19.1 | yellow |
| RP11-540A21.2 | yellow |
| RP11-541N10.3 | yellow |
| RP11-543C4.1 | yellow |
| RP11-544L8__B.4 | yellow |
| RP11-544M22.1 | yellow |
| RP11-545I5.3 | yellow |
| RP11-547D24.1 | yellow |
| RP11-549B18.1 | yellow |
| RP11-54A9.1 | magenta |
| RP11-550A5.2 | yellow |
| RP11-554A11.4 | yellow |
| RP11-554I8.2 | yellow |
| RP11-561I11.3 | yellow |
| RP11-561I11.4 | yellow |
| RP11-567G11.1 | yellow |
| RP11-567J20.2 | yellow |
| RP11-571L19.8 | yellow |
| RP11-572M11.4 | yellow |
| RP11-573G6.6 | yellow |
| RP11-574O7.1 | yellow |
| RP11-576I22.2 | magenta |
| RP11-582J16.4 | yellow |
| RP11-588K22.2 | yellow |
| RP11-589C21.6 | yellow |
| RP11-594N15.3 | yellow |
| RP11-598F7.4 | yellow |
| RP11-598F7.5 | yellow |
| RP11-598F7.6 | yellow |
| RP11-59D5__B.2 | yellow |
| RP11-5C23.1 | yellow |
| RP11-613D13.4 | yellow |
| RP11-613D13.8 | yellow |
| RP11-626G11.1 | yellow |
| RP11-626H12.1 | yellow |
| RP11-627G18.1 | yellow |
| RP11-62F24.2 | yellow |
| RP11-635O16.2 | yellow |
| RP11-640L9.1 | yellow |
| RP11-641D5.2 | yellow |
| RP11-64B16.4 | yellow |
| RP11-64C12.8 | yellow |
| RP11-655M14.12 | yellow |
| RP11-65D17.1 | yellow |
| RP11-65J21.3 | yellow |
| RP11-662I13.2 | magenta |
| RP11-664D7.4 | yellow |
| RP11-671P2.1 | yellow |
| RP11-672A2.4 | yellow |
| RP11-672A2.5 | yellow |
| RP11-672A2.6 | yellow |
| RP11-673E1.1 | yellow |
| RP11-676J12.6 | yellow |
| RP11-677M14.3 | yellow |
| RP11-679B19.1 | yellow |
| RP11-67L2.2 | yellow |
| RP11-680F8.1 | magenta |
| RP11-680F8.3 | yellow |
| RP11-690D19.3 | yellow |
| RP11-696D21.2 | yellow |
| RP11-6F2.5 | magenta |
| RP11-710C12.1 | yellow |
| RP11-710F7.2 | yellow |
| RP11-713C5.1 | magenta |
| RP11-714G18.1 | yellow |
| RP11-716O23.2 | yellow |
| RP11-718B12.2 | yellow |
| RP11-71E19.1 | yellow |
| RP11-71E19.2 | yellow |
| RP11-723D22.3 | yellow |
| RP11-736K20.5 | yellow |
| RP11-739B23.1 | yellow |
| RP11-742B18.1 | yellow |
| RP11-753H16.3 | yellow |
| RP11-76E17.3 | yellow |
| RP11-770E5.1 | yellow |
| RP11-775C24.5 | yellow |
| RP11-779O18.1 | yellow |
| RP11-779O18.3 | yellow |
| RP11-783K16.10 | yellow |
| RP11-783K16.5 | yellow |
| RP11-789C1.1 | yellow |
| RP11-789C17.3 | yellow |
| RP11-789C17.5 | yellow |
| RP11-78O7.2 | yellow |
| RP11-78O7.3 | yellow |
| RP11-791G15.2 | yellow |
| RP11-79H23.3 | yellow |
| RP11-7F17.7 | magenta |
| RP11-800A3.7 | yellow |
| RP11-805I24.3 | yellow |
| RP11-817I4.1 | yellow |
| RP11-818F20.5 | magenta |
| RP11-830F9.6 | yellow |
| RP11-84A19.3 | magenta |
| RP11-84G21.1 | magenta |
| RP11-85G21.2 | yellow |
| RP11-85G21.3 | yellow |
| RP11-863K10.7 | yellow |
| RP11-863P13.3 | magenta |
| RP11-863P13.4 | yellow |
| RP11-864I4.4 | yellow |
| RP11-865I6.2 | magenta |
| RP11-867G23.10 | yellow |
| RP11-867G23.8 | yellow |
| RP11-875O11.1 | yellow |
| RP11-875O11.3 | yellow |
| RP11-879F14.2 | yellow |
| RP11-87E22.2 | yellow |
| RP11-887P2.6 | yellow |
| RP11-888D10.3 | yellow |
| RP11-88G17.6 | magenta |
| RP11-88I21.2 | yellow |
| RP11-890B15.2 | magenta |
| RP11-894P9.2 | yellow |
| RP11-895M11.3 | yellow |
| RP11-89B16.1 | yellow |
| RP11-89C3.3 | yellow |
| RP11-89C3.4 | yellow |
| RP11-89K21.1 | yellow |
| RP11-92C4.6 | yellow |
| RP11-930O11.1 | yellow |
| RP11-93H12.4 | magenta |
| RP11-93K22.13 | yellow |
| RP11-94A24.1 | magenta |
| RP11-958F21.1 | yellow |
| RP11-95H3.1 | yellow |
| RP11-95I16.2 | yellow |
| RP11-95I16.6 | yellow |
| RP11-95M15.1 | yellow |
| RP11-963H4.3 | yellow |
| RP11-96C23.13 | yellow |
| RP11-96C23.14 | yellow |
| RP11-96H19.1 | yellow |
| RP11-982M15.6 | yellow |
| RP11-993B23.3 | yellow |
| RP11-9N20.3 | yellow |
| RP13-1016M1.2 | yellow |
| RP13-152O15.5 | yellow |
| RP13-314C10.5 | yellow |
| RP13-463N16.6 | magenta |
| RP13-580F15.2 | yellow |
| RP13-631K18.3 | yellow |
| RP3-332B22.1 | yellow |
| RP3-340B19.3 | yellow |
| RP3-428L16.2 | magenta |
| RP3-431P23.5 | yellow |
| RP3-454B23.1 | yellow |
| RP3-483K16.4 | yellow |
| RP3-495K2.2 | magenta |
| RP3-525N10.2 | yellow |
| RP4-545L17.12 | yellow |
| RP4-564M11.2 | yellow |
| RP4-568C11.4 | yellow |
| RP4-575N6.4 | yellow |
| RP4-575N6.5 | yellow |
| RP4-621B10.8 | yellow |
| RP4-639F20.1 | yellow |
| RP4-655C5.4 | magenta |
| RP4-668J24.2 | yellow |
| RP4-694A7.2 | yellow |
| RP4-735C1.4 | yellow |
| RP4-737E23.7 | yellow |
| RP4-755D9.1 | yellow |
| RP4-782L23.1 | yellow |
| RP4-785G19.5 | yellow |
| RP5-1059L7.1 | yellow |
| RP5-1074L1.1 | yellow |
| RP5-1120P11.1 | magenta |
| RP5-1172A22.1 | magenta |
| RP5-1184F4.7 | yellow |
| RP5-1185I7.1 | yellow |
| RP5-1198O20.4 | yellow |
| RP5-826L7.1 | yellow |
| RP5-858L17.1 | yellow |
| RP5-884M6.1 | magenta |
| RP5-907D15.4 | magenta |
| RP5-933K21.3 | yellow |
| RP5-965F6.2 | yellow |
| RP6-191P20.4 | magenta |
| RP6-65G23.3 | yellow |
| SBF2-AS1 | yellow |
| SENCR | yellow |
| SFTA1P | yellow |
| SH3RF3-AS1 | yellow |
| SHANK3 | yellow |
| SLC22A18AS | yellow |
| SLC2A1-AS1 | yellow |
| SNHG18 | yellow |
| SPANXA2-OT1 | yellow |
| SPATA3-AS1 | magenta |
| ST20-AS1 | yellow |
| ST3GAL4-AS1 | yellow |
| ST3GAL6-AS1 | yellow |
| STXBP5-AS1 | magenta |
| SYNPR-AS1 | yellow |
| TARID | yellow |
| TBX2-AS1 | yellow |
| TBX5-AS1 | yellow |
| TGFB2-AS1 | yellow |
| TINCR | yellow |
| TMEM220-AS1 | yellow |
| TRHDE-AS1 | yellow |
| TRIM31-AS1 | yellow |
| TRIM52-AS1 | yellow |
| TSTD3 | yellow |
| UBA6-AS1 | yellow |
| UG0898H09 | magenta |
| VAC14-AS1 | magenta |
| WDFY3-AS2 | yellow |
| WDR11-AS1 | yellow |
| WWC2-AS2 | yellow |
| XXbac-BPG13B8.10 | yellow |
| XXbac-BPG252P9.10 | yellow |
| XXbac-BPG27H4.8 | yellow |
| XXyac-YM21GA2.7 | yellow |
| XXyac-YX65C7_A.2 | magenta |
| Z83851.4 | yellow |
| ZFPM2-AS1 | yellow |
| ZIM2-AS1 | magenta |
| ZNF582-AS1 | yellow |
