# Supplementary material for: Prognostic value of lncRNAs related to fatty acid metabolism in lung adenocarcinoma and their correlation with tumor microenvironment based on bioinformatics analysis
Source: Front Oncol. 2022 Oct 10;12:1022097. doi: 10.3389/fonc.2022.1022097 (PMC9590110; doi:10.3389/fonc.2022.1022097)
Supplement: Supplementary Table 1 — All samples were divided into high and low fatty acid metabolism score groups based on the median value of this score. [file DataSheet_1.zip › raw data and R code for checking/raw data/5.docx]

| Ensembl_ID | gene_name | logFC | AveExpr | t | P.Value | adj.P.Val | B |
| --- | --- | --- | --- | --- | --- | --- | --- |
| ENSG00000272620.1 | AFAP1-AS1 | 2.656892 | 2.549835 | 11.51014 | 1.08E-27 | 3.36E-26 | 51.65063 |
| ENSG00000203499.9 | FAM83H-AS1 | 1.842131 | 3.385684 | 15.44657 | 3.54E-45 | 3.06E-43 | 91.68215 |
| ENSG00000259153.1 | RP6-65G23.3 | 1.747486 | 2.367799 | 14.17533 | 2.94E-39 | 1.84E-37 | 78.11557 |
| ENSG00000272666.1 | CTA-384D8.35 | 1.711362 | 2.372794 | 12.04823 | 6.38E-30 | 2.30E-28 | 56.74181 |
| ENSG00000228288.5 | PCAT6 | 1.671491 | 2.316184 | 14.42349 | 2.14E-40 | 1.41E-38 | 80.72275 |
| ENSG00000227066.1 | RP3-340N1.2 | 1.643162 | 1.559721 | 9.122341 | 1.28E-18 | 2.16E-17 | 30.94512 |
| ENSG00000273132.1 | RP11-350J20.12 | 1.623193 | 2.587682 | 9.159009 | 9.50E-19 | 1.62E-17 | 31.23728 |
| ENSG00000259342.1 | RP11-519G16.5 | 1.57619 | 1.776668 | 8.585955 | 8.71E-17 | 1.27E-15 | 26.77276 |
| ENSG00000233461.4 | RP11-295G20.2 | 1.56786 | 2.467407 | 10.30805 | 5.96E-23 | 1.37E-21 | 40.81673 |
| ENSG00000204949.7 | FAM83A-AS1 | 1.463676 | 1.359613 | 10.40992 | 2.43E-23 | 5.71E-22 | 41.70406 |
| ENSG00000265415.1 | CTD-2510F5.4 | 1.463458 | 2.383214 | 10.58814 | 5.01E-24 | 1.24E-22 | 43.27062 |
| ENSG00000227036.5 | LINC00511 | 1.413125 | 1.651435 | 13.79096 | 1.63E-37 | 9.34E-36 | 74.1204 |
| ENSG00000272405.1 | RP11-284F21.10 | 1.39985 | 1.490693 | 7.520722 | 2.15E-13 | 2.39E-12 | 19.07651 |
| ENSG00000234614.1 | AL450992.2 | 1.356344 | 3.010091 | 9.438891 | 9.68E-20 | 1.77E-18 | 33.49559 |
| ENSG00000256940.1 | RP11-783K16.5 | 1.35037 | 1.478215 | 12.73533 | 7.54E-33 | 3.25E-31 | 63.43988 |
| ENSG00000255717.5 | SNHG1 | 1.300105 | 3.226326 | 12.26326 | 7.92E-31 | 3.00E-29 | 58.81507 |
| ENSG00000261373.1 | VPS9D1-AS1 | 1.294234 | 1.63476 | 10.92186 | 2.48E-25 | 6.72E-24 | 46.25149 |
| ENSG00000261123.1 | RP11-304L19.3 | 1.291371 | 1.426619 | 8.384016 | 4.07E-16 | 5.65E-15 | 25.25221 |
| ENSG00000230838.1 | AC093850.2 | 1.26575 | 1.426954 | 8.878776 | 8.89E-18 | 1.41E-16 | 29.02683 |
| ENSG00000259933.5 | RP11-304L19.1 | 1.2643 | 1.405656 | 8.549744 | 1.15E-16 | 1.67E-15 | 26.49805 |
| ENSG00000231106.2 | LINC01436 | 1.263363 | 1.941181 | 5.45057 | 7.50E-08 | 4.77E-07 | 6.594644 |
| ENSG00000249859.6 | PVT1 | 1.242927 | 1.590405 | 12.83764 | 2.71E-33 | 1.21E-31 | 64.45512 |
| ENSG00000255026.1 | RP11-326C3.2 | 1.238087 | 2.878193 | 5.183534 | 3.03E-07 | 1.79E-06 | 5.241896 |
| ENSG00000274605.1 | RP11-12G12.7 | 1.200802 | 2.550021 | 10.81169 | 6.73E-25 | 1.77E-23 | 45.26062 |
| ENSG00000229953.1 | RP11-284F21.7 | 1.19992 | 1.322224 | 7.888688 | 1.58E-14 | 1.93E-13 | 21.64319 |
| ENSG00000263893.2 | CTD-3010D24.3 | 1.185428 | 1.126858 | 9.207477 | 6.42E-19 | 1.11E-17 | 31.62477 |
| ENSG00000230316.5 | FEZF1-AS1 | 1.177414 | 1.102457 | 7.708919 | 5.73E-14 | 6.65E-13 | 20.37684 |
| ENSG00000273272.1 | CTA-384D8.34 | 1.164024 | 1.46885 | 8.96684 | 4.43E-18 | 7.20E-17 | 29.71592 |
| ENSG00000268307.1 | CTD-2619J13.13 | 1.161279 | 1.516794 | 7.325239 | 8.24E-13 | 8.71E-12 | 17.75375 |
| ENSG00000242125.3 | SNHG3 | 1.16021 | 2.806678 | 10.38855 | 2.94E-23 | 6.86E-22 | 41.51742 |
| ENSG00000243479.3 | MNX1-AS1 | 1.139784 | 1.081588 | 10.71051 | 1.68E-24 | 4.27E-23 | 44.35655 |
| ENSG00000254827.4 | SLC22A18AS | 1.098555 | 1.514554 | 9.688319 | 1.21E-20 | 2.35E-19 | 35.54974 |
| ENSG00000251003.6 | ZFPM2-AS1 | 1.096728 | 1.039794 | 9.610209 | 2.34E-20 | 4.44E-19 | 34.90232 |
| ENSG00000173727.10 | CMB9-22P13.1 | 1.093502 | 1.678036 | 8.653975 | 5.15E-17 | 7.66E-16 | 27.29122 |
| ENSG00000249395.2 | CASC9 | 1.091553 | 1.047966 | 6.238702 | 8.64E-10 | 6.78E-09 | 10.93931 |
| ENSG00000267751.4 | AC009005.2 | 1.086352 | 1.671476 | 10.04125 | 6.04E-22 | 1.29E-20 | 38.52128 |
| ENSG00000232677.5 | LINC00665 | 1.076638 | 1.665161 | 8.003393 | 6.88E-15 | 8.65E-14 | 22.46343 |
| ENSG00000265055.1 | AC145343.2 | 1.076356 | 1.239039 | 10.6865 | 2.08E-24 | 5.25E-23 | 44.14282 |
| ENSG00000223392.1 | CLDN10-AS1 | 1.075172 | 0.991394 | 7.409526 | 4.63E-13 | 5.00E-12 | 18.32057 |
| ENSG00000260265.1 | RP11-44F21.5 | 1.049844 | 1.596079 | 7.292232 | 1.03E-12 | 1.08E-11 | 17.53324 |
| ENSG00000249007.1 | RP11-510N19.5 | 1.03487 | 1.726272 | 7.033638 | 5.82E-12 | 5.66E-11 | 15.83437 |
| ENSG00000237523.1 | LINC00857 | 1.02829 | 1.380054 | 12.73304 | 7.71E-33 | 3.33E-31 | 63.4172 |
| ENSG00000281406.1 | BLACAT1 | 1.024282 | 1.201723 | 10.13548 | 2.68E-22 | 5.89E-21 | 39.32726 |
| ENSG00000260877.1 | RP11-211G23.2 | 1.023152 | 0.954762 | 6.471655 | 2.10E-10 | 1.76E-09 | 12.32162 |
| ENSG00000236345.1 | RP11-59D5__B.2 | 1.019125 | 1.071302 | 7.828469 | 2.44E-14 | 2.93E-13 | 21.21637 |
| ENSG00000248243.1 | RP11-93K22.13 | 1.019104 | 1.030437 | 12.62148 | 2.34E-32 | 9.79E-31 | 62.31554 |
| ENSG00000261716.1 | RP11-196G18.22 | 0.995969 | 1.655151 | 10.77658 | 9.25E-25 | 2.40E-23 | 44.9463 |
| ENSG00000234380.1 | LINC01426 | 0.97165 | 1.224888 | 10.41352 | 2.36E-23 | 5.55E-22 | 41.73558 |
| ENSG00000222041.9 | LINC00152 | 0.955003 | 2.177509 | 10.33058 | 4.89E-23 | 1.13E-21 | 41.01252 |
| ENSG00000250258.1 | CTC-431G16.2 | 0.950875 | 0.938711 | 7.214162 | 1.75E-12 | 1.78E-11 | 17.01497 |
| ENSG00000234741.6 | GAS5 | 0.94936 | 5.489661 | 7.327368 | 8.13E-13 | 8.59E-12 | 17.768 |
| ENSG00000234678.1 | RP11-465N4.4 | 0.94772 | 1.604781 | 11.55412 | 7.11E-28 | 2.25E-26 | 52.06145 |
| ENSG00000232445.1 | RP11-132A1.4 | 0.944616 | 1.512268 | 7.203955 | 1.87E-12 | 1.90E-11 | 16.94755 |
| ENSG00000254988.1 | CTD-2547H18.1 | 0.943242 | 0.982239 | 6.995838 | 7.47E-12 | 7.17E-11 | 15.59033 |
| ENSG00000272913.1 | RP11-440D17.3 | 0.941211 | 2.22805 | 11.51796 | 9.99E-28 | 3.13E-26 | 51.72362 |
| ENSG00000272141.1 | RP11-465B22.8 | 0.938752 | 1.632891 | 7.041998 | 5.51E-12 | 5.36E-11 | 15.88848 |
| ENSG00000245750.6 | RP11-279F6.1 | 0.931754 | 1.159546 | 4.747751 | 2.61E-06 | 1.37E-05 | 3.167692 |
| ENSG00000254560.4 | BBOX1-AS1 | 0.927951 | 0.874904 | 7.056776 | 5.00E-12 | 4.88E-11 | 15.98428 |
| ENSG00000231789.2 | PIK3CD-AS2 | 0.913959 | 1.786785 | 7.436719 | 3.84E-13 | 4.18E-12 | 18.50458 |
| ENSG00000235123.4 | DSCAM-AS1 | 0.910552 | 0.864668 | 3.63505 | 0.000303228 | 0.001161429 | -1.35506 |
| ENSG00000258914.1 | CTD-2134A5.3 | 0.905562 | 1.412548 | 9.695483 | 1.14E-20 | 2.22E-19 | 35.60931 |
| ENSG00000249746.1 | RP11-254I22.3 | 0.90449 | 0.963118 | 6.871652 | 1.68E-11 | 1.56E-10 | 14.7964 |
| ENSG00000232931.4 | LINC00342 | 0.894008 | 2.318623 | 4.57672 | 5.81E-06 | 2.91E-05 | 2.399522 |
| ENSG00000270933.1 | CTD-2227E11.1 | 0.893518 | 1.045916 | 8.230322 | 1.29E-15 | 1.72E-14 | 24.11384 |
| ENSG00000272825.1 | LL21NC02-1C16.2 | 0.886608 | 1.217456 | 6.007285 | 3.38E-09 | 2.49E-08 | 9.609933 |
| ENSG00000237643.1 | RP11-462G2.1 | 0.876354 | 1.887249 | 3.06424 | 0.002286035 | 0.007376167 | -3.23035 |
| ENSG00000228437.4 | RP11-400N13.2 | 0.866149 | 0.859915 | 6.395741 | 3.35E-10 | 2.74E-09 | 11.86634 |
| ENSG00000237686.5 | RP5-1120P11.1 | 0.860967 | 1.093745 | 7.144623 | 2.79E-12 | 2.78E-11 | 16.55723 |
| ENSG00000227719.1 | AC006042.6 | 0.8592 | 1.941583 | 7.204106 | 1.87E-12 | 1.90E-11 | 16.94854 |
| ENSG00000259772.5 | RP11-16E12.2 | 0.858872 | 1.410918 | 8.322207 | 6.48E-16 | 8.86E-15 | 24.79243 |
| ENSG00000249602.1 | RP11-98D18.3 | 0.857732 | 1.596359 | 9.706291 | 1.04E-20 | 2.04E-19 | 35.69923 |
| ENSG00000226738.1 | CTA-384D8.31 | 0.857502 | 1.273337 | 5.468757 | 6.81E-08 | 4.35E-07 | 6.689005 |
| ENSG00000234854.1 | LINC00676 | 0.854062 | 0.772427 | 3.263325 | 0.001167458 | 0.004004954 | -2.61121 |
| ENSG00000225490.1 | RP4-610C12.3 | 0.848933 | 1.347512 | 5.143272 | 3.73E-07 | 2.18E-06 | 5.043287 |
| ENSG00000197989.12 | SNHG12 | 0.847961 | 2.379653 | 8.109024 | 3.17E-15 | 4.10E-14 | 23.22712 |
| ENSG00000270761.1 | RP11-385F7.1 | 0.844444 | 1.744368 | 8.672599 | 4.46E-17 | 6.67E-16 | 27.43371 |
| ENSG00000265688.1 | MAFG-AS1 | 0.839019 | 2.040833 | 9.381624 | 1.55E-19 | 2.80E-18 | 33.02946 |
| ENSG00000272068.1 | RP11-284F21.9 | 0.838815 | 0.807354 | 5.880257 | 7.00E-09 | 4.99E-08 | 8.899016 |
| ENSG00000227619.1 | RP11-492E3.2 | 0.832066 | 0.837419 | 7.246091 | 1.41E-12 | 1.45E-11 | 17.22637 |
| ENSG00000249628.2 | LINC00942 | 0.823448 | 0.793341 | 4.259895 | 2.39E-05 | 0.000109568 | 1.045919 |
| ENSG00000237424.1 | FOXD2-AS1 | 0.819611 | 1.360167 | 11.24452 | 1.28E-26 | 3.75E-25 | 49.19052 |
| ENSG00000237978.4 | KCNMB2-AS1 | 0.818208 | 0.772649 | 6.481975 | 1.97E-10 | 1.65E-09 | 12.38387 |
| ENSG00000230082.1 | PRRT3-AS1 | 0.816285 | 1.424741 | 8.362763 | 4.78E-16 | 6.59E-15 | 25.09381 |
| ENSG00000233903.2 | Z83851.4 | 0.813225 | 1.591822 | 11.79557 | 7.21E-29 | 2.44E-27 | 54.33372 |
| ENSG00000237517.7 | DGCR5 | 0.812767 | 0.840988 | 8.820712 | 1.40E-17 | 2.19E-16 | 28.5753 |
| ENSG00000203999.7 | LINC01270 | 0.809813 | 0.92236 | 10.43146 | 2.01E-23 | 4.76E-22 | 41.89243 |
| ENSG00000232093.1 | RP11-307C12.11 | 0.807589 | 1.329153 | 9.992922 | 9.15E-22 | 1.93E-20 | 38.10997 |
| ENSG00000261189.1 | RP3-512B11.3 | 0.803915 | 1.200744 | 7.74888 | 4.31E-14 | 5.05E-13 | 20.6563 |
| ENSG00000272645.2 | RP11-504P24.8 | 0.800856 | 1.755254 | 7.523873 | 2.10E-13 | 2.34E-12 | 19.09807 |
| ENSG00000233532.4 | LINC00460 | 0.799085 | 0.869004 | 5.15187 | 3.57E-07 | 2.09E-06 | 5.085583 |
| ENSG00000272455.1 | RP4-758J18.13 | 0.798264 | 1.279936 | 11.09738 | 4.98E-26 | 1.40E-24 | 47.84344 |
| ENSG00000275180.1 | RP11-631N16.4 | 0.792656 | 1.390474 | 8.108522 | 3.18E-15 | 4.12E-14 | 23.22347 |
| ENSG00000228952.1 | RP11-567G11.1 | 0.791828 | 1.534243 | 4.812261 | 1.92E-06 | 1.02E-05 | 3.464193 |
| ENSG00000225138.6 | CTD-2228K2.7 | 0.791378 | 1.487981 | 6.225311 | 9.36E-10 | 7.32E-09 | 10.86119 |
| ENSG00000188242.4 | PP7080 | 0.785089 | 2.568319 | 9.735278 | 8.18E-21 | 1.61E-19 | 35.94077 |
| ENSG00000258534.1 | CTD-2134A5.4 | 0.780717 | 1.340983 | 9.714106 | 9.78E-21 | 1.91E-19 | 35.7643 |
| ENSG00000238042.4 | RP11-815M8.1 | 0.780392 | 1.32115 | 5.255726 | 2.09E-07 | 1.26E-06 | 5.601534 |
| ENSG00000232956.7 | SNHG15 | 0.780061 | 2.926353 | 7.398222 | 5.01E-13 | 5.39E-12 | 18.24424 |
| ENSG00000253339.1 | RP11-434I12.3 | 0.774959 | 0.792105 | 5.606682 | 3.23E-08 | 2.14E-07 | 7.413792 |
| ENSG00000277247.1 | RP11-785D18.3 | 0.774354 | 0.903538 | 3.781501 | 0.00017244 | 0.00068961 | -0.82466 |
| ENSG00000196756.10 | SNHG17 | 0.773369 | 2.434006 | 8.64305 | 5.61E-17 | 8.32E-16 | 27.20773 |
| ENSG00000263711.4 | RP11-169F17.1 | 0.772915 | 0.708924 | 5.892756 | 6.52E-09 | 4.67E-08 | 8.968375 |
| ENSG00000272556.1 | RP11-638I8.1 | 0.772596 | 1.500952 | 7.496812 | 2.53E-13 | 2.80E-12 | 18.91319 |
| ENSG00000241359.1 | SYNPR-AS1 | 0.772335 | 1.14291 | 6.915842 | 1.26E-11 | 1.18E-10 | 15.07754 |
| ENSG00000262772.1 | RP11-353N14.2 | 0.771981 | 0.734685 | 9.042237 | 2.43E-18 | 4.02E-17 | 30.30995 |
| ENSG00000248323.4 | LUCAT1 | 0.769071 | 0.911042 | 6.07818 | 2.23E-09 | 1.68E-08 | 10.01251 |
| ENSG00000281398.1 | SNHG4 | 0.768842 | 1.045339 | 9.256008 | 4.33E-19 | 7.58E-18 | 32.01427 |
| ENSG00000273478.1 | RP11-465N4.5 | 0.767731 | 1.962212 | 8.038666 | 5.32E-15 | 6.75E-14 | 22.71756 |
| ENSG00000250343.1 | CTC-255N20.1 | 0.759899 | 0.87765 | 6.83863 | 2.08E-11 | 1.91E-10 | 14.58732 |
| ENSG00000227039.5 | ITGB2-AS1 | 0.758718 | 1.396341 | 6.089833 | 2.09E-09 | 1.57E-08 | 10.07908 |
| ENSG00000248538.5 | RP11-10A14.5 | 0.75786 | 0.766008 | 6.055745 | 2.55E-09 | 1.90E-08 | 9.884664 |
| ENSG00000266402.3 | RP11-329L6.2 | 0.757644 | 1.421974 | 7.47959 | 2.86E-13 | 3.14E-12 | 18.79581 |
| ENSG00000230798.4 | FOXD3-AS1 | 0.755739 | 0.687039 | 6.916411 | 1.25E-11 | 1.18E-10 | 15.08117 |
| ENSG00000260920.2 | RP1-228H13.5 | 0.750473 | 1.121999 | 12.43714 | 1.44E-31 | 5.75E-30 | 60.50701 |
| ENSG00000261061.1 | RP11-303E16.2 | 0.746542 | 2.361781 | 8.079198 | 3.95E-15 | 5.07E-14 | 23.01068 |
| ENSG00000261039.2 | RP11-417E7.2 | 0.743671 | 0.783454 | 7.309498 | 9.18E-13 | 9.66E-12 | 17.64848 |
| ENSG00000236305.1 | RP11-126L15.4 | 0.738214 | 0.992267 | 9.536425 | 4.32E-20 | 8.08E-19 | 34.29421 |
| ENSG00000236618.2 | PITPNA-AS1 | 0.737194 | 2.421768 | 8.319982 | 6.59E-16 | 9.00E-15 | 24.77592 |
| ENSG00000257702.3 | LBX2-AS1 | 0.733492 | 2.269517 | 8.307328 | 7.25E-16 | 9.86E-15 | 24.68214 |
| ENSG00000259802.1 | CTD-2256P15.2 | 0.732383 | 1.433392 | 8.10985 | 3.15E-15 | 4.08E-14 | 23.23312 |
| ENSG00000248771.4 | LINC01207 | 0.729202 | 0.948404 | 5.320717 | 1.49E-07 | 9.16E-07 | 5.929156 |
| ENSG00000228109.1 | MFI2-AS1 | 0.728796 | 1.263492 | 7.972593 | 8.61E-15 | 1.08E-13 | 22.24225 |
| ENSG00000229647.1 | AC007879.7 | 0.726687 | 0.823336 | 6.176979 | 1.25E-09 | 9.64E-09 | 10.58044 |
| ENSG00000226950.5 | DANCR | 0.725921 | 3.440667 | 7.185116 | 2.13E-12 | 2.15E-11 | 16.82332 |
| ENSG00000223784.1 | RP11-554I8.2 | 0.725015 | 0.73699 | 5.579992 | 3.73E-08 | 2.46E-07 | 7.272275 |
| ENSG00000225986.1 | UBXN10-AS1 | 0.724309 | 1.250296 | 6.480051 | 1.99E-10 | 1.67E-09 | 12.37226 |
| ENSG00000224081.6 | LINC01057 | 0.723696 | 1.225877 | 12.19422 | 1.55E-30 | 5.79E-29 | 58.14709 |
| ENSG00000177788.5 | RP5-1061H20.4 | 0.722372 | 0.898785 | 12.26475 | 7.80E-31 | 2.96E-29 | 58.8295 |
| ENSG00000235026.4 | DPP10-AS1 | 0.715754 | 0.841885 | 5.126558 | 4.06E-07 | 2.36E-06 | 4.96125 |
| ENSG00000179066.7 | CTD-2527I21.15 | 0.715331 | 0.661128 | 7.732087 | 4.86E-14 | 5.67E-13 | 20.53872 |
| ENSG00000249199.1 | CTD-2139B15.5 | 0.714565 | 0.646859 | 4.507093 | 7.99E-06 | 3.92E-05 | 2.094292 |
| ENSG00000272138.1 | RP11-27N21.3 | 0.713693 | 0.892613 | 8.470448 | 2.11E-16 | 3.00E-15 | 25.89959 |
| ENSG00000258053.1 | CTD-2021H9.3 | 0.712902 | 0.702075 | 4.703888 | 3.21E-06 | 1.66E-05 | 2.968196 |
| ENSG00000235029.1 | MNX1-AS2 | 0.710618 | 0.798128 | 7.581778 | 1.40E-13 | 1.58E-12 | 19.4955 |
| ENSG00000272502.1 | RP11-713M15.2 | 0.708581 | 1.803419 | 4.907798 | 1.21E-06 | 6.59E-06 | 3.910068 |
| ENSG00000231226.1 | TRIM31-AS1 | 0.703702 | 0.906687 | 7.961282 | 9.35E-15 | 1.16E-13 | 22.16121 |
| ENSG00000270460.1 | RP11-796E10.1 | 0.702421 | 0.675664 | 7.34004 | 7.45E-13 | 7.91E-12 | 17.85289 |
| ENSG00000248367.1 | CTB-129O4.1 | 0.70082 | 1.340871 | 7.762752 | 3.90E-14 | 4.59E-13 | 20.75359 |
| ENSG00000257924.1 | RP11-493L12.5 | 0.697569 | 0.680467 | 8.860365 | 1.03E-17 | 1.62E-16 | 28.88341 |
| ENSG00000247095.2 | MIR210HG | 0.696463 | 1.881281 | 6.816071 | 2.40E-11 | 2.20E-10 | 14.44497 |
| ENSG00000228265.4 | RALY-AS1 | 0.69404 | 1.449607 | 10.52208 | 9.02E-24 | 2.19E-22 | 42.68783 |
| ENSG00000253665.1 | RP11-359E19.2 | 0.692247 | 0.792114 | 3.619706 | 0.00032135 | 0.00122325 | -1.40947 |
| ENSG00000228989.1 | AC133528.2 | 0.691975 | 3.043871 | 7.803382 | 2.92E-14 | 3.48E-13 | 21.03933 |
| ENSG00000265800.1 | RP11-649A18.5 | 0.691515 | 1.078564 | 7.978399 | 8.26E-15 | 1.03E-13 | 22.2839 |
| ENSG00000224251.5 | RP11-499O7.7 | 0.690835 | 0.781994 | 4.492913 | 8.52E-06 | 4.16E-05 | 2.032662 |
| ENSG00000262580.4 | RP11-334C17.5 | 0.689445 | 1.134799 | 7.822524 | 2.55E-14 | 3.05E-13 | 21.17438 |
| ENSG00000153363.11 | LINC00467 | 0.68801 | 1.945329 | 9.068692 | 1.96E-18 | 3.28E-17 | 30.51926 |
| ENSG00000267123.4 | CTD-2357A8.3 | 0.686983 | 0.794335 | 7.31322 | 8.95E-13 | 9.44E-12 | 17.67336 |
| ENSG00000262920.4 | RP11-1260E13.1 | 0.683459 | 0.957683 | 3.917755 | 0.00010029 | 0.000417843 | -0.31333 |
| ENSG00000262468.4 | LINC01569 | 0.683183 | 1.228901 | 8.842647 | 1.18E-17 | 1.85E-16 | 28.74561 |
| ENSG00000249042.4 | CTD-2015H6.3 | 0.682889 | 2.600868 | 8.417614 | 3.15E-16 | 4.42E-15 | 25.50324 |
| ENSG00000261116.1 | RP3-523K23.2 | 0.681046 | 0.790004 | 5.36998 | 1.15E-07 | 7.16E-07 | 6.17992 |
| ENSG00000245910.7 | SNHG6 | 0.680683 | 5.339329 | 6.366078 | 4.01E-10 | 3.26E-09 | 11.6897 |
| ENSG00000235899.1 | LINC01564 | 0.675452 | 0.824256 | 5.503953 | 5.63E-08 | 3.64E-07 | 6.872416 |
| ENSG00000273821.1 | RP5-963E22.6 | 0.674803 | 0.899779 | 9.869195 | 2.63E-21 | 5.39E-20 | 37.06327 |
| ENSG00000232442.1 | CTD-3184A7.4 | 0.672878 | 2.31739 | 6.018321 | 3.17E-09 | 2.34E-08 | 9.672328 |
| ENSG00000172965.13 | MIR4435-1HG | 0.67131 | 1.699603 | 9.197606 | 6.95E-19 | 1.20E-17 | 31.54573 |
| ENSG00000224536.1 | RP11-134G8.7 | 0.670962 | 1.617044 | 9.168476 | 8.80E-19 | 1.51E-17 | 31.31284 |
| ENSG00000233834.5 | AC005083.1 | 0.665664 | 2.262502 | 5.439853 | 7.94E-08 | 5.04E-07 | 6.539175 |
| ENSG00000246582.2 | RP11-1149O23.3 | 0.664569 | 1.15377 | 12.48739 | 8.79E-32 | 3.54E-30 | 60.9985 |
| ENSG00000225518.2 | RP11-396C23.2 | 0.664169 | 0.816026 | 10.57236 | 5.77E-24 | 1.42E-22 | 43.13119 |
| ENSG00000236017.6 | ASMTL-AS1 | 0.660053 | 1.612049 | 4.722214 | 2.94E-06 | 1.53E-05 | 3.051339 |
| ENSG00000224220.1 | AC104699.1 | 0.658856 | 0.906598 | 6.352135 | 4.36E-10 | 3.53E-09 | 11.60692 |
| ENSG00000203288.3 | RP11-98D18.9 | 0.658146 | 0.896314 | 10.405 | 2.54E-23 | 5.95E-22 | 41.6611 |
| ENSG00000253395.1 | KB-1460A1.1 | 0.658044 | 0.761037 | 8.118741 | 2.95E-15 | 3.83E-14 | 23.29777 |
| ENSG00000229043.2 | AC091729.9 | 0.657511 | 1.608723 | 8.464094 | 2.21E-16 | 3.14E-15 | 25.85182 |
| ENSG00000232640.1 | RP1-266L20.2 | 0.654658 | 1.922201 | 7.500639 | 2.47E-13 | 2.73E-12 | 18.9393 |
| ENSG00000214049.6 | UCA1 | 0.652097 | 0.658618 | 3.91607 | 0.000100974 | 0.000420463 | -0.31976 |
| ENSG00000183250.10 | LINC01547 | 0.651758 | 1.30829 | 6.624164 | 8.13E-11 | 7.08E-10 | 13.25023 |
| ENSG00000230177.1 | RP5-1112D6.4 | 0.651197 | 1.680804 | 7.920291 | 1.26E-14 | 1.55E-13 | 21.86823 |
| ENSG00000267670.1 | CTB-55O6.4 | 0.65106 | 0.806394 | 5.366831 | 1.17E-07 | 7.27E-07 | 6.163828 |
| ENSG00000254389.3 | RHPN1-AS1 | 0.648701 | 1.090935 | 9.067464 | 1.98E-18 | 3.31E-17 | 30.50953 |
| ENSG00000254343.2 | RP11-760H22.2 | 0.641163 | 2.127609 | 5.540324 | 4.63E-08 | 3.01E-07 | 7.063063 |
| ENSG00000267523.1 | CTD-2537I9.12 | 0.639843 | 1.675842 | 6.033816 | 2.89E-09 | 2.15E-08 | 9.760104 |
| ENSG00000267080.4 | ASB16-AS1 | 0.637801 | 2.32187 | 9.2812 | 3.53E-19 | 6.22E-18 | 32.21706 |
| ENSG00000249430.1 | CTD-2231H16.1 | 0.637221 | 1.109835 | 4.563611 | 6.17E-06 | 3.07E-05 | 2.341724 |
| ENSG00000266968.2 | RP11-116O18.1 | 0.632343 | 0.731118 | 3.003908 | 0.00278311 | 0.008823297 | -3.41054 |
| ENSG00000249096.5 | RP11-290F5.1 | 0.628822 | 0.763175 | 7.283428 | 1.10E-12 | 1.14E-11 | 17.47456 |
| ENSG00000273179.1 | RP11-20I20.4 | 0.627331 | 1.599475 | 4.779918 | 2.24E-06 | 1.18E-05 | 3.315073 |
| ENSG00000231483.1 | RP11-336A10.5 | 0.624614 | 0.605303 | 7.494346 | 2.58E-13 | 2.85E-12 | 18.89636 |
| ENSG00000242147.1 | RP13-463N16.6 | 0.624204 | 0.586899 | 6.859767 | 1.81E-11 | 1.68E-10 | 14.72105 |
| ENSG00000231683.5 | RP1-27K12.2 | 0.622775 | 0.600888 | 3.855354 | 0.000128804 | 0.000526987 | -0.54964 |
| ENSG00000225285.1 | RP4-758J18.10 | 0.622481 | 1.992623 | 5.447393 | 7.63E-08 | 4.85E-07 | 6.578191 |
| ENSG00000240731.1 | RP5-890O3.9 | 0.621655 | 2.65355 | 5.886147 | 6.77E-09 | 4.84E-08 | 8.931686 |
| ENSG00000253616.4 | RP11-875O11.3 | 0.620452 | 0.793552 | 8.452954 | 2.41E-16 | 3.41E-15 | 25.76814 |
| ENSG00000269867.1 | CTD-2583A14.8 | 0.619831 | 2.277194 | 7.201118 | 1.91E-12 | 1.94E-11 | 16.92882 |
| ENSG00000176912.3 | TYMSOS | 0.610503 | 0.737029 | 8.617929 | 6.81E-17 | 1.00E-15 | 27.01608 |
| ENSG00000257883.1 | RP11-497G19.1 | 0.609913 | 0.637666 | 3.702221 | 0.000234612 | 0.000916671 | -1.11427 |
| ENSG00000224093.4 | RP5-1033H22.2 | 0.609768 | 0.934328 | 6.949718 | 1.01E-11 | 9.58E-11 | 15.29409 |
| ENSG00000177410.11 | ZFAS1 | 0.609683 | 5.267801 | 5.575606 | 3.82E-08 | 2.51E-07 | 7.249081 |
| ENSG00000229152.2 | ANKRD10-IT1 | 0.608101 | 2.978327 | 4.995097 | 7.84E-07 | 4.39E-06 | 4.324525 |
| ENSG00000224817.1 | RP11-190J1.3 | 0.607214 | 0.56173 | 5.408849 | 9.37E-08 | 5.90E-07 | 6.37925 |
| ENSG00000236663.1 | AP001631.9 | 0.604787 | 0.610707 | 6.715367 | 4.57E-11 | 4.08E-10 | 13.8144 |
| ENSG00000257270.1 | RP11-521B24.5 | 0.601708 | 1.694663 | 5.432149 | 8.28E-08 | 5.24E-07 | 6.499357 |
| ENSG00000237857.2 | RP11-435O5.2 | 0.599015 | 0.941654 | 10.73562 | 1.34E-24 | 3.43E-23 | 44.5804 |
| ENSG00000275479.1 | RP11-334C17.6 | 0.598892 | 1.319814 | 6.841444 | 2.04E-11 | 1.88E-10 | 14.6051 |
| ENSG00000273568.1 | RP11-417L19.6 | 0.597933 | 1.192438 | 8.294699 | 7.97E-16 | 1.08E-14 | 24.58865 |
| ENSG00000251179.1 | TMEM92-AS1 | 0.596339 | 1.059518 | 6.912525 | 1.29E-11 | 1.21E-10 | 15.05639 |
| ENSG00000255545.6 | RP11-627G23.1 | 0.596197 | 0.694425 | 5.392593 | 1.02E-07 | 6.39E-07 | 6.295727 |
| ENSG00000260710.1 | RP11-616M22.7 | 0.594865 | 0.550241 | 5.493577 | 5.96E-08 | 3.83E-07 | 6.818237 |
| ENSG00000266869.1 | RP6-114E22.1 | 0.594307 | 0.569434 | 5.053168 | 5.87E-07 | 3.34E-06 | 4.603926 |
| ENSG00000253417.4 | RP11-109J4.1 | 0.591773 | 0.593439 | 6.338904 | 4.73E-10 | 3.81E-09 | 11.52851 |
| ENSG00000273619.1 | RP5-908M14.9 | 0.590999 | 1.466273 | 6.062432 | 2.45E-09 | 1.83E-08 | 9.922726 |
| ENSG00000270195.1 | RP11-572O17.1 | 0.589318 | 0.783288 | 8.665614 | 4.71E-17 | 7.02E-16 | 27.38025 |
| ENSG00000229512.1 | AC068580.5 | 0.588811 | 1.251395 | 6.622713 | 8.20E-11 | 7.14E-10 | 13.24131 |
| ENSG00000260051.1 | LA16c-390E6.4 | 0.587262 | 0.96275 | 6.71696 | 4.52E-11 | 4.04E-10 | 13.82431 |
| ENSG00000240476.1 | LINC00973 | 0.587162 | 0.559382 | 3.784311 | 0.000170551 | 0.000682916 | -0.81429 |
| ENSG00000246273.5 | SBF2-AS1 | 0.584916 | 1.128941 | 10.86529 | 4.15E-25 | 1.11E-23 | 45.74192 |
| ENSG00000269397.1 | CTB-92J24.2 | 0.583595 | 0.832568 | 7.956431 | 9.69E-15 | 1.21E-13 | 22.12647 |
| ENSG00000257654.1 | RP11-497G19.2 | 0.583581 | 0.625833 | 3.506108 | 0.000490716 | 0.001807879 | -1.80548 |
| ENSG00000267892.1 | CTD-2540F13.2 | 0.582957 | 1.008482 | 6.627189 | 7.98E-11 | 6.95E-10 | 13.26884 |
| ENSG00000236871.5 | LINC00106 | 0.581915 | 1.596893 | 4.378164 | 1.43E-05 | 6.74E-05 | 1.540607 |
| ENSG00000234072.1 | AC074117.10 | 0.581771 | 1.34961 | 8.76784 | 2.12E-17 | 3.27E-16 | 28.1661 |
| ENSG00000227925.1 | RP11-191N8.2 | 0.581169 | 1.012828 | 4.495078 | 8.44E-06 | 4.12E-05 | 2.042059 |
| ENSG00000223768.1 | LINC00205 | 0.581046 | 1.937672 | 6.828066 | 2.22E-11 | 2.04E-10 | 14.52061 |
| ENSG00000226465.1 | RP13-401N8.1 | 0.578919 | 1.273694 | 4.912091 | 1.18E-06 | 6.46E-06 | 3.930291 |
| ENSG00000273032.1 | DGCR9 | 0.576817 | 0.643196 | 6.952928 | 9.89E-12 | 9.39E-11 | 15.31465 |
| ENSG00000225489.5 | RP11-390F4.3 | 0.574351 | 0.642951 | 7.60606 | 1.18E-13 | 1.34E-12 | 19.6629 |
| ENSG00000261762.1 | RP11-650L12.2 | 0.574137 | 0.586371 | 6.556393 | 1.24E-10 | 1.06E-09 | 12.83529 |
| ENSG00000272954.1 | KB-1440D3.13 | 0.57384 | 0.642061 | 7.228978 | 1.58E-12 | 1.62E-11 | 17.11297 |
| ENSG00000261804.1 | RP11-44F14.2 | 0.572498 | 0.883532 | 7.745336 | 4.42E-14 | 5.18E-13 | 20.63147 |
| ENSG00000276900.1 | RP11-467L13.7 | 0.571867 | 1.459622 | 6.327783 | 5.06E-10 | 4.06E-09 | 11.46272 |
| ENSG00000223783.1 | AC124944.5 | 0.571365 | 0.595901 | 5.759551 | 1.38E-08 | 9.55E-08 | 8.235956 |
| ENSG00000273576.1 | RP11-390P24.1 | 0.570795 | 2.478297 | 5.398739 | 9.88E-08 | 6.20E-07 | 6.327278 |
| ENSG00000263325.1 | LA16c-325D7.1 | 0.570689 | 0.812906 | 6.198413 | 1.10E-09 | 8.54E-09 | 10.70471 |
| ENSG00000225783.5 | MIAT | 0.569714 | 1.105672 | 4.46762 | 9.55E-06 | 4.64E-05 | 1.923183 |
| ENSG00000223749.6 | MIR503HG | 0.566343 | 0.893853 | 6.006786 | 3.39E-09 | 2.49E-08 | 9.607116 |
| ENSG00000272183.1 | RP11-523H20.3 | 0.566194 | 0.772409 | 9.888168 | 2.24E-21 | 4.61E-20 | 37.22318 |
| ENSG00000272720.1 | CTA-228A9.3 | 0.566156 | 0.827791 | 6.571818 | 1.13E-10 | 9.68E-10 | 12.92941 |
| ENSG00000273142.1 | RP11-458F8.4 | 0.565611 | 1.808902 | 5.465531 | 6.93E-08 | 4.42E-07 | 6.672245 |
| ENSG00000278709.1 | RP5-1059L7.1 | 0.558968 | 1.248901 | 5.947599 | 4.76E-09 | 3.46E-08 | 9.274231 |
| ENSG00000272172.1 | RP13-582O9.7 | 0.557893 | 1.279352 | 6.752166 | 3.62E-11 | 3.26E-10 | 14.04389 |
| ENSG00000262251.1 | RP11-199F11.2 | 0.557527 | 0.840106 | 8.706636 | 3.42E-17 | 5.17E-16 | 27.69475 |
| ENSG00000261801.4 | LOXL1-AS1 | 0.557193 | 1.063298 | 8.750973 | 2.42E-17 | 3.71E-16 | 28.03595 |
| ENSG00000226696.4 | LENG8-AS1 | 0.554617 | 1.464699 | 7.344575 | 7.23E-13 | 7.68E-12 | 17.88331 |
| ENSG00000271032.1 | CTD-2527I21.14 | 0.554211 | 1.258702 | 5.616976 | 3.05E-08 | 2.03E-07 | 7.468537 |
| ENSG00000266490.1 | CTD-2349P21.9 | 0.551682 | 1.507057 | 7.237942 | 1.49E-12 | 1.53E-11 | 17.17234 |
| ENSG00000273759.1 | RP4-563E14.1 | 0.550259 | 0.949531 | 7.810239 | 2.78E-14 | 3.32E-13 | 21.08768 |
| ENSG00000249379.1 | RP1-27K12.4 | 0.549839 | 0.580905 | 5.08231 | 5.07E-07 | 2.91E-06 | 4.745254 |
| ENSG00000267077.1 | RP11-127I20.5 | 0.54925 | 0.63909 | 9.679243 | 1.31E-20 | 2.53E-19 | 35.47432 |
| ENSG00000182109.6 | RP11-69E11.4 | 0.548736 | 1.149032 | 6.833838 | 2.14E-11 | 1.97E-10 | 14.55705 |
| ENSG00000251169.2 | AC005355.2 | 0.545955 | 0.77651 | 5.39887 | 9.88E-08 | 6.20E-07 | 6.32795 |
| ENSG00000236481.1 | AC002331.1 | 0.545602 | 0.850753 | 5.180941 | 3.07E-07 | 1.81E-06 | 5.229061 |
| ENSG00000226328.5 | NUP50-AS1 | 0.545175 | 1.937054 | 6.626779 | 8.00E-11 | 6.97E-10 | 13.26631 |
| ENSG00000253716.4 | RP13-582O9.5 | 0.544735 | 1.60842 | 6.437409 | 2.59E-10 | 2.15E-09 | 12.11566 |
| ENSG00000257588.1 | RP11-469H8.6 | 0.544675 | 0.957587 | 3.637723 | 0.00030017 | 0.001151396 | -1.34556 |
| ENSG00000259038.1 | CTD-2325P2.4 | 0.544598 | 1.015136 | 5.264182 | 2.00E-07 | 1.21E-06 | 5.643956 |
| ENSG00000266970.1 | RP11-806H10.4 | 0.544235 | 1.006628 | 6.451578 | 2.38E-10 | 1.98E-09 | 12.20076 |
| ENSG00000230623.2 | RP11-469A15.2 | 0.54372 | 0.674765 | 4.912796 | 1.18E-06 | 6.44E-06 | 3.933612 |
| ENSG00000234771.3 | SLC25A25-AS1 | 0.543145 | 1.153331 | 6.247733 | 8.19E-10 | 6.43E-09 | 10.99208 |
| ENSG00000245970.2 | KB-1208A12.3 | 0.542404 | 1.499966 | 7.158478 | 2.54E-12 | 2.55E-11 | 16.64813 |
| ENSG00000176659.7 | C20orf197 | 0.540529 | 0.640686 | 4.89789 | 1.27E-06 | 6.90E-06 | 3.86345 |
| ENSG00000271936.1 | RP11-443B20.1 | 0.540212 | 1.014669 | 8.240921 | 1.19E-15 | 1.59E-14 | 24.19181 |
| ENSG00000272275.1 | RP11-791G15.2 | 0.539769 | 1.265368 | 5.602468 | 3.30E-08 | 2.18E-07 | 7.391409 |
| ENSG00000188185.10 | LINC00265 | 0.539172 | 1.288053 | 6.902901 | 1.37E-11 | 1.28E-10 | 14.99505 |
| ENSG00000256967.1 | RP11-273B20.1 | 0.537126 | 1.110579 | 6.74695 | 3.74E-11 | 3.37E-10 | 14.0113 |
| ENSG00000223396.2 | RP11-134G8.5 | 0.536927 | 1.155942 | 8.636073 | 5.92E-17 | 8.75E-16 | 27.15446 |
| ENSG00000260442.4 | ATP2A1-AS1 | 0.53434 | 0.845096 | 7.292574 | 1.03E-12 | 1.08E-11 | 17.53552 |
| ENSG00000256073.3 | URB1-AS1 | 0.53382 | 2.664773 | 5.74196 | 1.53E-08 | 1.05E-07 | 8.140343 |
| ENSG00000251022.5 | THAP9-AS1 | 0.531098 | 2.975301 | 6.902137 | 1.38E-11 | 1.29E-10 | 14.99019 |
| ENSG00000225742.4 | RP11-513G11.4 | 0.530224 | 0.806268 | 5.600541 | 3.34E-08 | 2.21E-07 | 7.381182 |
| ENSG00000254973.1 | RP11-429J17.7 | 0.526951 | 0.609057 | 8.794065 | 1.73E-17 | 2.68E-16 | 28.36883 |
| ENSG00000258837.1 | CTD-2566J3.1 | 0.526768 | 0.507388 | 3.695108 | 0.000241118 | 0.000940449 | -1.13996 |
| ENSG00000236144.5 | TMEM147-AS1 | 0.526369 | 1.237573 | 6.956129 | 9.68E-12 | 9.20E-11 | 15.33517 |
| ENSG00000273257.1 | RP11-177J6.1 | 0.524922 | 0.990116 | 5.276337 | 1.88E-07 | 1.14E-06 | 5.705042 |
| ENSG00000231439.4 | WASIR2 | 0.524204 | 0.534589 | 8.812079 | 1.50E-17 | 2.34E-16 | 28.50836 |
| ENSG00000262903.1 | RP11-235E17.6 | 0.523899 | 0.999506 | 5.895949 | 6.40E-09 | 4.59E-08 | 8.986109 |
| ENSG00000275888.1 | RP13-516M14.10 | 0.523741 | 1.457404 | 5.362333 | 1.20E-07 | 7.44E-07 | 6.140859 |
| ENSG00000231327.1 | AC016700.5 | 0.521915 | 0.969334 | 6.120631 | 1.74E-09 | 1.32E-08 | 10.25556 |
| ENSG00000254367.4 | RP11-211C9.1 | 0.52028 | 0.522195 | 6.464546 | 2.19E-10 | 1.83E-09 | 12.27879 |
| ENSG00000262714.1 | RP11-44F14.8 | 0.519002 | 0.864763 | 7.250019 | 1.37E-12 | 1.42E-11 | 17.25243 |
| ENSG00000258346.1 | RP11-148B3.2 | 0.517059 | 0.589891 | 3.865667 | 0.000123615 | 0.000507164 | -0.51084 |
| ENSG00000231563.1 | RP11-245P10.4 | 0.516462 | 0.982168 | 5.062408 | 5.61E-07 | 3.20E-06 | 4.648658 |
| ENSG00000281404.1 | LINC01176 | 0.516282 | 1.321815 | 5.954406 | 4.58E-09 | 3.33E-08 | 9.312363 |
| ENSG00000266088.4 | RP5-1028K7.2 | 0.516189 | 0.911428 | 5.977118 | 4.02E-09 | 2.94E-08 | 9.439889 |
| ENSG00000250742.1 | RP11-834C11.4 | 0.515806 | 1.945187 | 3.437931 | 0.000629224 | 0.002269102 | -2.03734 |
| ENSG00000253931.1 | RP11-909N17.2 | 0.514954 | 0.513062 | 4.961924 | 9.24E-07 | 5.13E-06 | 4.166242 |
| ENSG00000255224.1 | CTD-3065J16.9 | 0.51432 | 0.943089 | 8.276893 | 9.11E-16 | 1.23E-14 | 24.45703 |
| ENSG00000238058.1 | RP11-432J22.2 | 0.51255 | 1.481765 | 7.196123 | 1.97E-12 | 2.00E-11 | 16.89587 |
| ENSG00000264608.1 | RP11-192H23.8 | 0.512208 | 1.215324 | 5.967401 | 4.25E-09 | 3.10E-08 | 9.385276 |
| ENSG00000255100.1 | RP11-21L23.3 | 0.511366 | 0.736685 | 6.233165 | 8.93E-10 | 6.99E-09 | 10.90699 |
| ENSG00000272763.1 | RP11-357H14.17 | 0.511251 | 0.512037 | 4.410471 | 1.23E-05 | 5.90E-05 | 1.677942 |
| ENSG00000249592.4 | RP11-440L14.1 | 0.510644 | 1.079945 | 8.184217 | 1.82E-15 | 2.40E-14 | 23.77558 |
| ENSG00000267147.2 | CTC-548K16.1 | 0.510016 | 0.538038 | 5.889288 | 6.65E-09 | 4.76E-08 | 8.949114 |
| ENSG00000269352.1 | PTOV1-AS2 | 0.509826 | 1.774371 | 4.855423 | 1.56E-06 | 8.37E-06 | 3.664631 |
| ENSG00000277744.1 | CTC-435M10.12 | 0.507687 | 0.998248 | 6.314747 | 5.47E-10 | 4.38E-09 | 11.38572 |
| ENSG00000229312.2 | RP11-470P21.2 | 0.507062 | 0.470753 | 6.180306 | 1.22E-09 | 9.46E-09 | 10.59971 |
| ENSG00000214725.6 | CDIPT-AS1 | 0.506225 | 0.55607 | 5.305244 | 1.62E-07 | 9.89E-07 | 5.850828 |
| ENSG00000262979.1 | CTD-2047H16.2 | 0.504893 | 0.910839 | 5.29372 | 1.72E-07 | 1.05E-06 | 5.792622 |
| ENSG00000267121.4 | CTD-2020K17.1 | 0.504362 | 1.078529 | 5.165116 | 3.33E-07 | 1.96E-06 | 5.150868 |
| ENSG00000261295.1 | RP11-524D16__A.3 | 0.503577 | 1.684791 | 4.180821 | 3.36E-05 | 0.000150809 | 0.722262 |
| ENSG00000253563.2 | NKX2-1-AS1 | 0.503435 | 2.306544 | 3.426726 | 0.000655212 | 0.002355542 | -2.07503 |
| ENSG00000235939.1 | RP11-123B3.2 | 0.502542 | 0.469046 | 5.157368 | 3.47E-07 | 2.04E-06 | 5.112658 |
| ENSG00000273373.1 | RP5-1074L1.4 | 0.501754 | 1.142257 | 6.325369 | 5.13E-10 | 4.12E-09 | 11.44845 |
| ENSG00000271971.1 | CTD-2006H14.2 | 0.501659 | 1.859043 | 5.796516 | 1.12E-08 | 7.84E-08 | 8.437715 |
| ENSG00000223414.2 | LINC00473 | 0.501607 | 0.536677 | 3.475598 | 0.000548744 | 0.002002516 | -1.90978 |
| ENSG00000271781.1 | CTD-2589H19.6 | 0.500961 | 1.042673 | 5.417338 | 8.96E-08 | 5.65E-07 | 6.422953 |
| ENSG00000226330.1 | RP11-739N20.2 | 0.500805 | 0.746306 | 6.681288 | 5.67E-11 | 5.02E-10 | 13.60282 |
| ENSG00000259366.1 | CTD-2647L4.4 | -0.50196 | 1.430976 | -6.59052 | 1.00E-10 | 8.64E-10 | 13.04377 |
| ENSG00000271324.1 | RP11-10C24.2 | -0.50313 | 0.870471 | -9.76662 | 6.28E-21 | 1.25E-19 | 36.20253 |
| ENSG00000266283.1 | RP11-627G18.1 | -0.505 | 0.272731 | -14.4433 | 1.74E-40 | 1.15E-38 | 80.9319 |
| ENSG00000272221.1 | XXbac-BPG181B23.7 | -0.51313 | 1.381737 | -6.16876 | 1.31E-09 | 1.01E-08 | 10.5329 |
| ENSG00000259351.1 | RP11-111E14.1 | -0.51537 | 0.322063 | -13.3184 | 2.11E-35 | 1.07E-33 | 69.28412 |
| ENSG00000273664.1 | RP11-2N1.3 | -0.51593 | 0.094989 | -21.9817 | 6.59E-78 | 2.18E-75 | 166.7929 |
| ENSG00000224875.2 | AC083949.1 | -0.51724 | 0.487106 | -11.6764 | 2.24E-28 | 7.36E-27 | 53.2088 |
| ENSG00000225315.2 | RP11-293P20.2 | -0.51759 | 0.468899 | -6.56675 | 1.16E-10 | 9.97E-10 | 12.89846 |
| ENSG00000268001.1 | CARD8-AS1 | -0.51929 | 1.491125 | -7.51985 | 2.16E-13 | 2.40E-12 | 19.07056 |
| ENSG00000267100.1 | ILF3-AS1 | -0.52279 | 3.228001 | -6.50235 | 1.74E-10 | 1.46E-09 | 12.507 |
| ENSG00000259504.2 | RP11-352D13.5 | -0.52314 | 0.221229 | -16.0689 | 3.77E-48 | 3.77E-46 | 98.49856 |
| ENSG00000232480.1 | TGFB2-AS1 | -0.52479 | 0.891139 | -5.3485 | 1.29E-07 | 7.96E-07 | 6.070303 |
| ENSG00000274245.1 | RP11-357P18.2 | -0.52535 | 1.174159 | -4.79009 | 2.13E-06 | 1.13E-05 | 3.361867 |
| ENSG00000238123.1 | MID1IP1-AS1 | -0.52956 | 1.298295 | -6.65279 | 6.79E-11 | 5.96E-10 | 13.42659 |
| ENSG00000227188.1 | MGAT3-AS1 | -0.53096 | 0.188143 | -16.6738 | 4.43E-51 | 4.97E-49 | 105.2187 |
| ENSG00000248399.1 | RP11-503N18.4 | -0.53117 | 0.680372 | -4.8861 | 1.34E-06 | 7.28E-06 | 3.808095 |
| ENSG00000275894.1 | RP3-453C12.14 | -0.53209 | 0.721505 | -7.6303 | 9.98E-14 | 1.14E-12 | 19.83046 |
| ENSG00000244124.1 | ATP1B3-AS1 | -0.53609 | 0.763308 | -6.82492 | 2.27E-11 | 2.08E-10 | 14.50075 |
| ENSG00000271643.1 | RP11-10C24.3 | -0.5387 | 1.174825 | -11.3896 | 3.32E-27 | 1.01E-25 | 50.53015 |
| ENSG00000273156.1 | RP11-127B20.2 | -0.5395 | 0.813899 | -8.60815 | 7.34E-17 | 1.08E-15 | 26.94157 |
| ENSG00000272777.1 | RP11-571L19.8 | -0.54161 | 0.894875 | -11.4629 | 1.68E-27 | 5.18E-26 | 51.21047 |
| ENSG00000236914.3 | RP11-1008C21.2 | -0.54186 | 0.74309 | -13.501 | 3.26E-36 | 1.74E-34 | 71.14255 |
| ENSG00000259330.1 | INAFM2 | -0.54259 | 4.051268 | -6.58284 | 1.05E-10 | 9.05E-10 | 12.99676 |
| ENSG00000236054.1 | LL22NC03-104C7.1 | -0.546 | 0.124375 | -20.6523 | 4.59E-71 | 1.11E-68 | 151.0807 |
| ENSG00000250786.1 | SNHG18 | -0.54623 | 1.897269 | -4.12541 | 4.26E-05 | 0.000187996 | 0.498844 |
| ENSG00000237923.1 | XXbac-BPG27H4.8 | -0.54664 | 0.120543 | -19.392 | 1.24E-64 | 2.49E-62 | 136.3146 |
| ENSG00000249610.1 | CTC-441N14.1 | -0.54694 | 0.377208 | -7.36793 | 6.16E-13 | 6.58E-12 | 18.04015 |
| ENSG00000269916.1 | RP11-193M21.1 | -0.54979 | 0.418033 | -7.02475 | 6.17E-12 | 5.98E-11 | 15.77692 |
| ENSG00000230939.1 | RP11-314C16.1 | -0.55442 | 0.549341 | -11.2668 | 1.04E-26 | 3.07E-25 | 49.39528 |
| ENSG00000265752.2 | RP11-403A21.1 | -0.55655 | 0.42796 | -9.27057 | 3.85E-19 | 6.76E-18 | 32.13141 |
| ENSG00000255471.1 | RP11-736K20.5 | -0.56048 | 0.480609 | -11.4991 | 1.19E-27 | 3.71E-26 | 51.54767 |
| ENSG00000250041.2 | CTD-2003C8.2 | -0.56227 | 0.257526 | -16.8328 | 7.41E-52 | 8.54E-50 | 106.9999 |
| ENSG00000277351.1 | RP11-325L12.6 | -0.56363 | 0.473147 | -13.4804 | 4.03E-36 | 2.13E-34 | 70.93196 |
| ENSG00000272411.1 | RP11-44B19.1 | -0.56368 | 0.185666 | -19.1642 | 1.78E-63 | 3.39E-61 | 133.6648 |
| ENSG00000226822.1 | RP11-356N1.2 | -0.56454 | 0.322069 | -14.5031 | 9.20E-41 | 6.24E-39 | 81.56387 |
| ENSG00000270076.1 | AF131215.8 | -0.56837 | 0.258841 | -16.7961 | 1.12E-51 | 1.29E-49 | 106.588 |
| ENSG00000274002.1 | RP11-66N24.6 | -0.56957 | 1.927435 | -2.91778 | 0.003665187 | 0.01129808 | -3.66175 |
| ENSG00000273437.1 | RP11-434H6.7 | -0.57104 | 1.501605 | -7.09347 | 3.92E-12 | 3.86E-11 | 16.22291 |
| ENSG00000246016.2 | LINC01513 | -0.573 | 0.325631 | -8.44215 | 2.62E-16 | 3.69E-15 | 25.68707 |
| ENSG00000272760.1 | RP11-5C23.1 | -0.57638 | 1.819101 | -8.2439 | 1.17E-15 | 1.56E-14 | 24.21378 |
| ENSG00000197291.7 | RAMP2-AS1 | -0.58045 | 0.361473 | -10.9965 | 1.26E-25 | 3.45E-24 | 46.92652 |
| ENSG00000254847.1 | RP11-51B23.3 | -0.58097 | 0.256811 | -21.4704 | 2.86E-75 | 8.60E-73 | 160.7371 |
| ENSG00000233760.1 | AC004947.2 | -0.58489 | 0.179979 | -20.8069 | 7.39E-72 | 1.84E-69 | 152.9022 |
| ENSG00000264125.1 | RP11-354P11.4 | -0.58496 | 0.110435 | -17.7301 | 2.79E-56 | 3.90E-54 | 117.1484 |
| ENSG00000225194.2 | LINC00092 | -0.58544 | 0.389623 | -14.5934 | 3.52E-41 | 2.44E-39 | 82.51976 |
| ENSG00000165511.6 | C10orf25 | -0.58558 | 0.841032 | -13.8281 | 1.11E-37 | 6.38E-36 | 74.50446 |
| ENSG00000238078.1 | LINC01352 | -0.58619 | 0.294609 | -19.0233 | 9.17E-63 | 1.66E-60 | 132.0283 |
| ENSG00000254453.1 | NAV2-AS2 | -0.58893 | 0.214673 | -13.842 | 9.60E-38 | 5.55E-36 | 74.64804 |
| ENSG00000273650.1 | CTD-3193K9.11 | -0.58941 | 0.278314 | -13.8887 | 5.91E-38 | 3.46E-36 | 75.13124 |
| ENSG00000275830.1 | RP11-403A3.3 | -0.58964 | 0.206614 | -20.27 | 4.17E-69 | 9.63E-67 | 146.586 |
| ENSG00000275392.1 | RP11-164O23.8 | -0.5932 | 0.528041 | -7.82223 | 2.55E-14 | 3.06E-13 | 21.17229 |
| ENSG00000241158.4 | ADAMTS9-AS1 | -0.59361 | 0.193078 | -22.7021 | 1.25E-81 | 5.13E-79 | 175.3413 |
| ENSG00000225431.1 | AP001626.1 | -0.59374 | 1.288306 | -4.52508 | 7.36E-06 | 3.63E-05 | 2.172717 |
| ENSG00000234405.1 | LL0XNC01-250H12.3 | -0.59404 | 0.453905 | -15.2739 | 2.32E-44 | 1.91E-42 | 89.81026 |
| ENSG00000275764.1 | RP11-582E3.6 | -0.59517 | 1.993432 | -7.9815 | 8.07E-15 | 1.01E-13 | 22.30613 |
| ENSG00000233237.5 | LINC00472 | -0.59966 | 0.250815 | -21.4319 | 4.51E-75 | 1.34E-72 | 160.2821 |
| ENSG00000259834.1 | RP11-284N8.3 | -0.60898 | 1.841606 | -4.32941 | 1.77E-05 | 8.24E-05 | 1.335136 |
| ENSG00000272823.1 | RP11-295M18.6 | -0.6112 | 0.301677 | -18.4087 | 1.14E-59 | 1.85E-57 | 124.9239 |
| ENSG00000254703.2 | SENCR | -0.61133 | 0.589997 | -12.5433 | 5.06E-32 | 2.08E-30 | 61.54687 |
| ENSG00000262890.1 | RP11-424M24.5 | -0.61264 | 0.337513 | -7.76668 | 3.80E-14 | 4.48E-13 | 20.78117 |
| ENSG00000233117.2 | LINC00702 | -0.61431 | 0.300649 | -19.7379 | 2.17E-66 | 4.60E-64 | 140.3521 |
| ENSG00000249621.1 | CTD-2544H17.1 | -0.61844 | 0.317741 | -7.7933 | 3.14E-14 | 3.73E-13 | 20.9683 |
| ENSG00000236164.1 | RP11-268F1.3 | -0.62015 | 0.304297 | -8.92304 | 6.27E-18 | 1.01E-16 | 29.37258 |
| ENSG00000229671.1 | LINC01150 | -0.62216 | 0.671078 | -10.7531 | 1.14E-24 | 2.95E-23 | 44.73645 |
| ENSG00000281881.1 | SPRY4-IT1 | -0.62245 | 0.543436 | -9.2496 | 4.56E-19 | 7.97E-18 | 31.96279 |
| ENSG00000267207.1 | RP11-264B14.1 | -0.62281 | 0.271059 | -19.0523 | 6.54E-63 | 1.19E-60 | 132.3655 |
| ENSG00000181123.7 | RP4-539M6.14 | -0.62672 | 0.342343 | -9.05111 | 2.26E-18 | 3.76E-17 | 30.38013 |
| ENSG00000273226.1 | RP11-513M16.8 | -0.62904 | 1.673938 | -9.32506 | 2.47E-19 | 4.38E-18 | 32.57108 |
| ENSG00000243701.4 | LINC00883 | -0.63451 | 0.819574 | -12.488 | 8.74E-32 | 3.52E-30 | 61.00469 |
| ENSG00000273341.1 | RP5-899E9.1 | -0.64181 | 0.667569 | -12.1589 | 2.19E-30 | 8.06E-29 | 57.80599 |
| ENSG00000224945.1 | RP11-82L18.2 | -0.64186 | 1.419313 | -5.07539 | 5.25E-07 | 3.01E-06 | 4.711641 |
| ENSG00000244953.1 | RP11-613D13.8 | -0.64442 | 0.267072 | -17.6227 | 9.53E-56 | 1.30E-53 | 115.9259 |
| ENSG00000261634.3 | RP11-352D13.6 | -0.6497 | 0.220965 | -20.6051 | 8.02E-71 | 1.92E-68 | 150.5257 |
| ENSG00000234362.4 | AC104654.2 | -0.65161 | 0.460356 | -13.0047 | 5.08E-34 | 2.36E-32 | 66.12186 |
| ENSG00000259976.1 | RP11-553L6.5 | -0.65842 | 3.313492 | -5.96815 | 4.23E-09 | 3.09E-08 | 9.389505 |
| ENSG00000272734.1 | ADIRF-AS1 | -0.66498 | 0.949343 | -7.92161 | 1.25E-14 | 1.54E-13 | 21.87763 |
| ENSG00000224189.5 | HAGLR | -0.66723 | 2.918429 | -3.57139 | 0.000385273 | 0.001447808 | -1.57936 |
| ENSG00000274173.1 | RP4-568C11.4 | -0.67002 | 4.109801 | -4.54396 | 6.75E-06 | 3.34E-05 | 2.255357 |
| ENSG00000267461.1 | RP11-120M18.5 | -0.67092 | 0.796031 | -7.56516 | 1.57E-13 | 1.77E-12 | 19.38121 |
| ENSG00000273033.2 | RP11-67L2.2 | -0.6752 | 1.399329 | -11.2369 | 1.38E-26 | 4.02E-25 | 49.12057 |
| ENSG00000234506.4 | LINC01506 | -0.67549 | 0.305979 | -19.1865 | 1.37E-63 | 2.64E-61 | 133.9235 |
| ENSG00000237813.3 | AC002066.1 | -0.67758 | 0.355506 | -14.4794 | 1.18E-40 | 7.96E-39 | 81.31285 |
| ENSG00000244567.1 | AC096772.6 | -0.67945 | 2.448401 | -9.34725 | 2.06E-19 | 3.67E-18 | 32.7507 |
| ENSG00000260940.1 | RP4-575N6.5 | -0.67965 | 0.222467 | -28.8402 | 3.09E-113 | 4.35E-110 | 247.9152 |
| ENSG00000232415.1 | CTB-51J22.1 | -0.68293 | 1.876018 | -4.93315 | 1.07E-06 | 5.87E-06 | 4.029737 |
| ENSG00000255422.1 | AP002954.4 | -0.68663 | 0.83619 | -9.10689 | 1.45E-18 | 2.43E-17 | 30.82227 |
| ENSG00000267519.3 | CTD-3252C9.4 | -0.68712 | 3.203968 | -4.47988 | 9.04E-06 | 4.40E-05 | 1.976168 |
| ENSG00000272463.1 | RP11-532F6.3 | -0.6961 | 0.536202 | -15.1221 | 1.20E-43 | 9.54E-42 | 88.17118 |
| ENSG00000229155.1 | RP11-528A4.2 | -0.69731 | 1.049259 | -5.59139 | 3.51E-08 | 2.32E-07 | 7.332618 |
| ENSG00000259087.4 | RP11-356O9.2 | -0.70221 | 0.6678 | -6.04214 | 2.76E-09 | 2.05E-08 | 9.807348 |
| ENSG00000224307.1 | RP11-344B5.2 | -0.70226 | 1.796424 | -6.23714 | 8.72E-10 | 6.84E-09 | 10.93021 |
| ENSG00000253829.1 | RP11-723D22.3 | -0.71198 | 0.531197 | -13.2017 | 6.92E-35 | 3.37E-33 | 68.10335 |
| ENSG00000253821.1 | RP11-246K15.1 | -0.71624 | 0.220802 | -19.0349 | 8.01E-63 | 1.45E-60 | 132.1627 |
| ENSG00000255446.1 | CTD-2531D15.4 | -0.72025 | 0.334054 | -10.345 | 4.31E-23 | 9.95E-22 | 41.13769 |
| ENSG00000228692.2 | RP5-826L7.1 | -0.72294 | 0.170728 | -31.5975 | 4.20E-127 | 1.27E-123 | 279.7513 |
| ENSG00000275874.1 | LINC00162 | -0.72464 | 0.412021 | -7.40984 | 4.62E-13 | 4.99E-12 | 18.32271 |
| ENSG00000275612.1 | RP11-35J10.6 | -0.72492 | 0.160222 | -29.1204 | 1.17E-114 | 1.97E-111 | 251.1808 |
| ENSG00000249364.4 | RP11-434D9.1 | -0.7339 | 0.249146 | -21.4567 | 3.36E-75 | 1.01E-72 | 160.5752 |
| ENSG00000270091.1 | RP11-78O7.2 | -0.7385 | 1.07827 | -11.5736 | 5.92E-28 | 1.88E-26 | 52.24382 |
| ENSG00000259225.5 | RP11-1008C21.1 | -0.73995 | 0.804626 | -8.65703 | 5.03E-17 | 7.49E-16 | 27.31461 |
| ENSG00000225873.1 | LINC00694 | -0.74313 | 0.472252 | -11.5589 | 6.80E-28 | 2.15E-26 | 52.10583 |
| ENSG00000224968.1 | RP1-35C21.1 | -0.74574 | 0.266846 | -19.1424 | 2.29E-63 | 4.34E-61 | 133.4107 |
| ENSG00000275178.1 | RP11-4B16.3 | -0.74743 | 0.282529 | -19.276 | 4.82E-64 | 9.40E-62 | 134.9644 |
| ENSG00000227307.1 | RP11-95I16.2 | -0.74824 | 0.227671 | -20.8493 | 4.48E-72 | 1.12E-69 | 153.4016 |
| ENSG00000262370.4 | RP11-473M20.9 | -0.75086 | 0.879462 | -10.5353 | 8.02E-24 | 1.96E-22 | 42.80409 |
| ENSG00000225473.1 | ATP13A4-AS1 | -0.75113 | 0.534997 | -7.53567 | 1.93E-13 | 2.16E-12 | 19.17887 |
| ENSG00000233251.6 | AC007743.1 | -0.75153 | 0.502994 | -14.7162 | 9.49E-42 | 6.87E-40 | 83.82447 |
| ENSG00000232352.1 | SEMA3B-AS1 | -0.7517 | 1.100819 | -8.91887 | 6.48E-18 | 1.04E-16 | 29.3399 |
| ENSG00000248551.1 | RP11-287F9.2 | -0.75835 | 0.120429 | -37.2356 | 2.22E-154 | 3.36E-150 | 342.3485 |
| ENSG00000272473.1 | AC006273.4 | -0.76018 | 0.310716 | -21.2149 | 5.91E-74 | 1.63E-71 | 157.7171 |
| ENSG00000260686.1 | CTB-36H16.2 | -0.76063 | 0.760807 | -15.4525 | 3.32E-45 | 2.87E-43 | 91.74687 |
| ENSG00000260025.1 | RP11-490M8.1 | -0.77186 | 1.815764 | -7.78044 | 3.44E-14 | 4.08E-13 | 20.87785 |
| ENSG00000229111.1 | MED4-AS1 | -0.77288 | 0.376003 | -18.7629 | 1.89E-61 | 3.23E-59 | 129.0119 |
| ENSG00000272839.1 | RP11-452C13.1 | -0.77414 | 0.450886 | -17.5723 | 1.69E-55 | 2.27E-53 | 115.3528 |
| ENSG00000260804.3 | PKI55 | -0.7844 | 1.306073 | -11.6786 | 2.19E-28 | 7.22E-27 | 53.22915 |
| ENSG00000256234.1 | RP11-283G6.4 | -0.79128 | 0.67939 | -12.116 | 3.31E-30 | 1.21E-28 | 57.39338 |
| ENSG00000237248.4 | LINC00987 | -0.7927 | 0.693389 | -17.6217 | 9.64E-56 | 1.32E-53 | 115.9142 |
| ENSG00000257474.4 | RP11-359M6.1 | -0.79506 | 0.652394 | -7.68676 | 6.70E-14 | 7.74E-13 | 20.22241 |
| ENSG00000250073.2 | RP11-677M14.3 | -0.80334 | 0.831213 | -11.2193 | 1.62E-26 | 4.71E-25 | 48.95909 |
| ENSG00000269951.1 | RP11-797A18.6 | -0.80665 | 0.990575 | -10.123 | 2.99E-22 | 6.54E-21 | 39.21981 |
| ENSG00000269918.1 | AF131215.9 | -0.81415 | 0.705872 | -13.0833 | 2.30E-34 | 1.09E-32 | 66.91075 |
| ENSG00000272274.1 | LINC00551 | -0.81567 | 0.301039 | -16.509 | 2.81E-50 | 3.03E-48 | 103.3792 |
| ENSG00000261888.1 | AC144831.1 | -0.81705 | 0.674593 | -14.8737 | 1.75E-42 | 1.32E-40 | 85.5048 |
| ENSG00000196167.8 | COLCA1 | -0.82009 | 1.34743 | -5.91703 | 5.68E-09 | 4.09E-08 | 9.10343 |
| ENSG00000260936.1 | FTO-IT1 | -0.82436 | 0.688849 | -11.5857 | 5.28E-28 | 1.68E-26 | 52.357 |
| ENSG00000271736.1 | RP11-85G21.3 | -0.82479 | 0.562125 | -8.76722 | 2.14E-17 | 3.28E-16 | 28.16133 |
| ENSG00000266010.1 | GATA6-AS1 | -0.82621 | 0.562525 | -10.6066 | 4.25E-24 | 1.05E-22 | 43.4338 |
| ENSG00000253288.1 | RP11-238K6.1 | -0.83751 | 0.311909 | -14.2619 | 1.18E-39 | 7.48E-38 | 79.02318 |
| ENSG00000198358.4 | RP11-544M22.1 | -0.84464 | 0.153545 | -25.77 | 1.68E-97 | 1.39E-94 | 211.785 |
| ENSG00000278484.1 | RP11-95I16.6 | -0.84901 | 0.441247 | -9.2546 | 4.38E-19 | 7.66E-18 | 32.00297 |
| ENSG00000267667.1 | RP11-136H19.1 | -0.85441 | 0.519324 | -18.3162 | 3.32E-59 | 5.35E-57 | 123.8598 |
| ENSG00000243384.1 | RP11-475O23.2 | -0.8588 | 0.217158 | -18.0257 | 9.43E-58 | 1.42E-55 | 120.5256 |
| ENSG00000177406.4 | RP11-218M22.1 | -0.86086 | 2.255686 | -10.1343 | 2.71E-22 | 5.95E-21 | 39.31692 |
| ENSG00000232188.1 | RP11-312J18.6 | -0.86308 | 0.232852 | -19.303 | 3.52E-64 | 6.91E-62 | 135.2784 |
| ENSG00000272783.1 | RP13-1016M1.2 | -0.86662 | 0.609324 | -15.0424 | 2.85E-43 | 2.21E-41 | 87.31414 |
| ENSG00000250410.1 | RP11-714G18.1 | -0.86867 | 0.507681 | -15.1326 | 1.08E-43 | 8.54E-42 | 88.28404 |
| ENSG00000267774.2 | RP11-2N1.2 | -0.87093 | 0.256853 | -16.9685 | 1.61E-52 | 1.89E-50 | 108.5235 |
| ENSG00000262097.1 | CTD-2135D7.5 | -0.87294 | 0.541813 | -12.9342 | 1.03E-33 | 4.71E-32 | 65.41732 |
| ENSG00000229619.3 | MBNL1-AS1 | -0.87819 | 0.575673 | -20.5623 | 1.33E-70 | 3.14E-68 | 150.0213 |
| ENSG00000261468.1 | RP11-1024P17.1 | -0.88328 | 0.683671 | -19.7002 | 3.37E-66 | 7.11E-64 | 139.9112 |
| ENSG00000263586.1 | HID1-AS1 | -0.88375 | 0.328839 | -26.677 | 3.57E-102 | 3.54E-99 | 222.5145 |
| ENSG00000274370.1 | AC144831.3 | -0.88604 | 0.973487 | -12.5351 | 5.49E-32 | 2.25E-30 | 61.46597 |
| ENSG00000256262.1 | USP30-AS1 | -0.89277 | 1.441618 | -9.41306 | 1.20E-19 | 2.17E-18 | 33.28508 |
| ENSG00000237189.1 | RP11-85G21.2 | -0.89434 | 0.790334 | -6.69041 | 5.35E-11 | 4.75E-10 | 13.65939 |
| ENSG00000269353.1 | AC092071.1 | -0.89774 | 1.225407 | -5.70401 | 1.89E-08 | 1.28E-07 | 7.934956 |
| ENSG00000228559.1 | RP3-340B19.3 | -0.90026 | 0.817038 | -8.4008 | 3.58E-16 | 5.00E-15 | 25.37754 |
| ENSG00000274536.3 | RP6-159A1.4 | -0.91244 | 0.90876 | -10.96 | 1.75E-25 | 4.78E-24 | 46.59588 |
| ENSG00000239268.2 | RP11-384F7.2 | -0.91852 | 0.276309 | -19.0756 | 4.98E-63 | 9.14E-61 | 132.6356 |
| ENSG00000261338.2 | RP11-378A13.1 | -0.91995 | 1.010922 | -19.117 | 3.08E-63 | 5.76E-61 | 133.1163 |
| ENSG00000267607.1 | CTD-2369P2.8 | -0.92229 | 0.678976 | -11.5352 | 8.50E-28 | 2.68E-26 | 51.88456 |
| ENSG00000234147.1 | RP3-460G2.2 | -0.93874 | 1.142785 | -9.02535 | 2.78E-18 | 4.58E-17 | 30.17655 |
| ENSG00000276668.1 | RP11-35J10.7 | -0.94209 | 0.216071 | -29.925 | 9.99E-119 | 2.16E-115 | 260.5215 |
| ENSG00000277782.1 | RP11-775C24.5 | -0.94213 | 1.966762 | -13.221 | 5.69E-35 | 2.79E-33 | 68.29772 |
| ENSG00000255468.5 | RP11-867G23.8 | -0.96478 | 1.310917 | -10.8002 | 7.47E-25 | 1.96E-23 | 45.15744 |
| ENSG00000237167.1 | AC128709.2 | -0.96583 | 0.174951 | -31.0972 | 1.31E-124 | 3.44E-121 | 274.0274 |
| ENSG00000235501.4 | RP4-639F20.1 | -0.97437 | 2.455202 | -10.2551 | 9.46E-23 | 2.15E-21 | 40.35825 |
| ENSG00000254109.4 | RBPMS-AS1 | -0.97531 | 1.559932 | -9.04406 | 2.39E-18 | 3.96E-17 | 30.32436 |
| ENSG00000237413.4 | MGC27382 | -0.97789 | 0.279427 | -22.9546 | 6.15E-83 | 2.64E-80 | 178.3413 |
| ENSG00000260461.1 | RP11-541N10.3 | -0.98319 | 1.591936 | -13.1174 | 1.63E-34 | 7.80E-33 | 67.25345 |
| ENSG00000260244.1 | RP11-588K22.2 | -0.99122 | 2.661929 | -9.3862 | 1.49E-19 | 2.70E-18 | 33.06663 |
| ENSG00000272143.1 | FGF14-AS2 | -0.99366 | 1.024404 | -12.8158 | 3.38E-33 | 1.50E-31 | 64.23754 |
| ENSG00000250266.1 | RP11-789C1.1 | -0.99511 | 0.789136 | -8.3732 | 4.41E-16 | 6.11E-15 | 25.17158 |
| ENSG00000230498.1 | RP4-564M11.2 | -0.99772 | 0.465193 | -20.8874 | 2.85E-72 | 7.16E-70 | 153.8518 |
| ENSG00000260310.1 | RP11-27M24.2 | -0.99908 | 0.485883 | -17.5189 | 3.12E-55 | 4.11E-53 | 114.7456 |
| ENSG00000204566.6 | LINC01552 | -1.00193 | 0.251882 | -22.3914 | 5.04E-80 | 1.81E-77 | 171.6525 |
| ENSG00000255310.2 | AF131215.2 | -1.00288 | 0.852774 | -14.3786 | 3.44E-40 | 2.25E-38 | 80.24989 |
| ENSG00000239467.4 | AC007405.6 | -1.00476 | 2.420522 | -7.94679 | 1.04E-14 | 1.29E-13 | 22.05748 |
| ENSG00000250431.1 | RP13-577H12.2 | -1.00669 | 0.274097 | -15.202 | 5.07E-44 | 4.10E-42 | 89.03291 |
| ENSG00000225792.1 | AC004540.4 | -1.02106 | 0.686251 | -14.6271 | 2.46E-41 | 1.73E-39 | 82.87706 |
| ENSG00000260468.1 | LINC01290 | -1.02309 | 0.556615 | -21.4337 | 4.42E-75 | 1.32E-72 | 160.3036 |
| ENSG00000234456.6 | MAGI2-AS3 | -1.03951 | 1.074062 | -16.5043 | 2.96E-50 | 3.19E-48 | 103.3264 |
| ENSG00000224276.1 | RP11-336K24.5 | -1.0398 | 0.659131 | -10.7283 | 1.43E-24 | 3.66E-23 | 44.51515 |
| ENSG00000251322.6 | SHANK3 | -1.05371 | 2.115242 | -12.187 | 1.66E-30 | 6.19E-29 | 58.07729 |
| ENSG00000273837.1 | LLNLR-470E3.1 | -1.05854 | 1.135467 | -12.5113 | 6.95E-32 | 2.82E-30 | 61.23259 |
| ENSG00000267530.2 | AC006273.5 | -1.06759 | 1.149274 | -11.3352 | 5.53E-27 | 1.66E-25 | 50.02601 |
| ENSG00000233730.1 | RP4-666F24.3 | -1.08369 | 0.556056 | -10.1751 | 1.90E-22 | 4.22E-21 | 39.6673 |
| ENSG00000231698.2 | AP002856.5 | -1.09369 | 0.229116 | -17.3495 | 2.14E-54 | 2.71E-52 | 112.8238 |
| ENSG00000255197.4 | RP11-750H9.5 | -1.09961 | 1.369781 | -12.9336 | 1.04E-33 | 4.73E-32 | 65.41175 |
| ENSG00000236304.1 | AP001189.4 | -1.12267 | 0.439191 | -21.7833 | 6.96E-77 | 2.23E-74 | 164.4416 |
| ENSG00000255007.1 | CTD-2589M5.4 | -1.13792 | 1.555825 | -6.79449 | 2.76E-11 | 2.51E-10 | 14.30916 |
| ENSG00000258752.1 | RP11-356K23.1 | -1.14347 | 0.806345 | -8.2636 | 1.01E-15 | 1.35E-14 | 24.35895 |
| ENSG00000272789.1 | RP11-286H15.1 | -1.14411 | 0.422385 | -18.5982 | 1.28E-60 | 2.14E-58 | 127.1091 |
| ENSG00000259417.2 | LINC01314 | -1.14772 | 0.320937 | -21.273 | 2.97E-74 | 8.40E-72 | 158.403 |
| ENSG00000250899.3 | RP11-253E3.3 | -1.15187 | 0.857718 | -19.7247 | 2.53E-66 | 5.35E-64 | 140.1967 |
| ENSG00000255399.3 | TBX5-AS1 | -1.17798 | 1.234038 | -15.0997 | 1.53E-43 | 1.20E-41 | 87.93049 |
| ENSG00000259974.2 | LINC00261 | -1.18169 | 1.570463 | -7.23344 | 1.54E-12 | 1.57E-11 | 17.1425 |
| ENSG00000261269.1 | RP11-389C8.2 | -1.19118 | 1.215089 | -17.9139 | 3.40E-57 | 5.05E-55 | 119.2474 |
| ENSG00000267280.4 | TBX2-AS1 | -1.19172 | 0.940821 | -16.4136 | 8.16E-50 | 8.57E-48 | 102.3167 |
| ENSG00000269186.1 | LINC01082 | -1.20263 | 0.266431 | -28.7149 | 1.34E-112 | 1.80E-109 | 246.4519 |
| ENSG00000225938.1 | RP4-575N6.4 | -1.20531 | 0.649242 | -23.7624 | 4.05E-87 | 2.08E-84 | 187.9433 |
| ENSG00000257057.1 | C11orf97 | -1.21089 | 0.618777 | -10.1701 | 1.98E-22 | 4.40E-21 | 39.62476 |
| ENSG00000233038.4 | AC011899.9 | -1.21505 | 0.747713 | -22.737 | 8.22E-82 | 3.41E-79 | 175.7565 |
| ENSG00000278910.2 | BANCR | -1.2261 | 0.767931 | -9.44376 | 9.30E-20 | 1.70E-18 | 33.53532 |
| ENSG00000258545.4 | RP4-755D9.1 | -1.22982 | 0.962835 | -10.7519 | 1.16E-24 | 2.98E-23 | 44.72551 |
| ENSG00000253959.1 | CTB-43E15.1 | -1.2323 | 0.369787 | -24.9114 | 4.58E-93 | 3.04E-90 | 201.5989 |
| ENSG00000231993.1 | EP300-AS1 | -1.25405 | 1.148448 | -17.4323 | 8.36E-55 | 1.08E-52 | 113.7627 |
| ENSG00000223573.5 | TINCR | -1.31171 | 0.733057 | -13.6145 | 1.01E-36 | 5.59E-35 | 72.3044 |
| ENSG00000254810.1 | RP11-672A2.4 | -1.31277 | 0.827162 | -20.6454 | 4.98E-71 | 1.20E-68 | 151 |
| ENSG00000228027.1 | RP1-251M9.3 | -1.35071 | 0.32319 | -22.5449 | 8.10E-81 | 3.16E-78 | 173.475 |
| ENSG00000260943.1 | RP11-476D10.1 | -1.35266 | 0.69794 | -12.1198 | 3.19E-30 | 1.16E-28 | 57.4299 |
| ENSG00000277639.1 | RP11-295M3.4 | -1.36554 | 1.991334 | -6.27056 | 7.14E-10 | 5.64E-09 | 11.12573 |
| ENSG00000276170.3 | AC124789.1 | -1.38007 | 0.625234 | -16.0924 | 2.91E-48 | 2.93E-46 | 98.75747 |
| ENSG00000186594.11 | MIR22HG | -1.3819 | 2.94672 | -15.4881 | 2.25E-45 | 1.96E-43 | 92.13355 |
| ENSG00000259094.1 | RP11-77A13.1 | -1.38248 | 0.536227 | -12.1245 | 3.05E-30 | 1.11E-28 | 57.47461 |
| ENSG00000258844.1 | RP11-259K15.2 | -1.38331 | 1.590599 | -8.78027 | 1.93E-17 | 2.98E-16 | 28.26211 |
| ENSG00000273877.3 | RP11-635O16.2 | -1.38495 | 0.607075 | -16.5557 | 1.67E-50 | 1.82E-48 | 103.8995 |
| ENSG00000231621.1 | AC013264.2 | -1.3864 | 1.13607 | -9.25551 | 4.35E-19 | 7.61E-18 | 32.01027 |
| ENSG00000235387.1 | LINC00961 | -1.39252 | 0.738047 | -30.1785 | 5.28E-120 | 1.23E-116 | 263.4527 |
| ENSG00000246430.5 | LINC00968 | -1.48001 | 0.447088 | -31.4134 | 3.47E-126 | 9.98E-123 | 277.6476 |
| ENSG00000228723.5 | SRGAP3-AS2 | -1.4863 | 0.89372 | -9.27382 | 3.74E-19 | 6.59E-18 | 32.15762 |
| ENSG00000214708.4 | AC090616.2 | -1.49222 | 1.22419 | -15.7838 | 8.79E-47 | 8.23E-45 | 95.36221 |
| ENSG00000248890.1 | HHIP-AS1 | -1.52152 | 1.415164 | -10.7759 | 9.30E-25 | 2.42E-23 | 44.94027 |
| ENSG00000235997.2 | AC109642.1 | -1.52669 | 1.010738 | -18.1751 | 1.69E-58 | 2.61E-56 | 122.2392 |
| ENSG00000261685.2 | RP11-401P9.4 | -1.52811 | 0.950013 | -17.1227 | 2.81E-53 | 3.40E-51 | 110.2611 |
| ENSG00000273760.1 | CH17-360D5.3 | -1.54137 | 0.753943 | -13.9892 | 2.07E-38 | 1.26E-36 | 76.17468 |
| ENSG00000248801.5 | RP11-664D7.4 | -1.60707 | 1.789008 | -9.70862 | 1.02E-20 | 2.00E-19 | 35.71858 |
| ENSG00000250978.4 | RP11-357D18.1 | -1.66406 | 0.969595 | -13.3491 | 1.54E-35 | 7.93E-34 | 69.59565 |
| ENSG00000276850.3 | CH17-360D5.2 | -1.69928 | 0.76544 | -18.2401 | 7.99E-59 | 1.25E-56 | 122.9848 |
| ENSG00000236242.1 | MYO16-AS1 | -1.72799 | 0.669658 | -16.2459 | 5.29E-49 | 5.42E-47 | 100.4551 |
| ENSG00000267107.5 | PCAT19 | -1.73262 | 1.248108 | -22.4199 | 3.59E-80 | 1.32E-77 | 171.9906 |
| ENSG00000266120.1 | RP11-354P11.2 | -1.77106 | 0.411971 | -26.7592 | 1.35E-102 | 1.36E-99 | 223.4847 |
| ENSG00000235385.1 | GS1-600G8.5 | -1.77112 | 0.528264 | -23.5445 | 5.44E-86 | 2.67E-83 | 185.3532 |
| ENSG00000261863.1 | RP11-141J13.5 | -1.77176 | 0.35008 | -38.4791 | 3.55E-160 | 1.07E-155 | 355.6443 |
| ENSG00000244215.1 | RP11-88I21.2 | -1.80041 | 0.318747 | -34.2902 | 2.51E-140 | 1.52E-136 | 310.1028 |
| ENSG00000223914.1 | AC079630.2 | -1.83038 | 0.881217 | -14.7967 | 4.01E-42 | 2.94E-40 | 84.68268 |
| ENSG00000251230.4 | RP11-701P16.5 | -1.87569 | 0.676861 | -27.546 | 1.24E-106 | 1.47E-103 | 232.7554 |
| ENSG00000234281.4 | LANCL1-AS1 | -1.89315 | 0.534563 | -36.3569 | 3.11E-150 | 3.13E-146 | 332.8383 |
| ENSG00000243961.2 | RP5-839B4.8 | -1.95283 | 0.842209 | -19.9964 | 1.04E-67 | 2.28E-65 | 143.3762 |
| ENSG00000228401.4 | RP11-251M1.1 | -2.01106 | 0.734718 | -28.7217 | 1.24E-112 | 1.70E-109 | 246.532 |
| ENSG00000257894.2 | RP1-78O14.1 | -2.03196 | 0.730289 | -24.8354 | 1.13E-92 | 7.44E-90 | 200.6963 |
| ENSG00000224215.1 | RP11-371A19.2 | -2.04661 | 0.35749 | -43.4775 | 1.25E-182 | 7.55E-178 | 407.1253 |
| ENSG00000256948.1 | RP11-598F7.3 | -2.16295 | 0.996741 | -21.7097 | 1.67E-76 | 5.18E-74 | 163.5697 |
| ENSG00000227051.5 | C14orf132 | -2.18042 | 1.634227 | -21.9565 | 8.89E-78 | 2.92E-75 | 166.4943 |
| ENSG00000224397.4 | LINC01272 | -2.22832 | 2.425582 | -18.7216 | 3.05E-61 | 5.20E-59 | 128.5346 |
| ENSG00000225342.2 | AC079630.4 | -2.23289 | 1.645274 | -13.51 | 2.97E-36 | 1.59E-34 | 71.23424 |
| ENSG00000225329.2 | LHFPL3-AS2 | -2.30445 | 1.435869 | -14.3701 | 3.77E-40 | 2.44E-38 | 80.15983 |
| ENSG00000238018.2 | AC093110.3 | -2.39483 | 1.170816 | -28.8942 | 1.64E-113 | 2.47E-110 | 248.545 |
| ENSG00000268388.4 | FENDRR | -2.49819 | 0.831696 | -29.7743 | 5.76E-118 | 1.16E-114 | 258.7758 |
| ENSG00000225383.5 | SFTA1P | -2.77509 | 3.856965 | -11.3417 | 5.20E-27 | 1.56E-25 | 50.08652 |
| ENSG00000235584.2 | AC008268.1 | -2.84089 | 1.65526 | -13.269 | 3.49E-35 | 1.74E-33 | 68.78313 |
