# Supplementary material for: Prognostic value of lncRNAs related to fatty acid metabolism in lung adenocarcinoma and their correlation with tumor microenvironment based on bioinformatics analysis
Source: Front Oncol. 2022 Oct 10;12:1022097. doi: 10.3389/fonc.2022.1022097 (PMC9590110; doi:10.3389/fonc.2022.1022097)
Supplement: Supplementary Table 1 — All samples were divided into high and low fatty acid metabolism score groups based on the median value of this score. [file DataSheet_1.zip › raw data and R code for checking/raw data/6.docx]

| changes | lncRNAs | modules |
| --- | --- | --- |
| up | AFAP1-AS1 | yellow |
|  | FAM83H-AS1 |  |
|  | RP6-65G23.3 |  |
|  | CTA-384D8.35 |  |
|  | RP11-350J20.12 |  |
|  | RP11-519G16.5 |  |
|  | RP11-295G20.2 |  |
|  | FAM83A-AS1 |  |
|  | CTD-2510F5.4 |  |
|  | LINC00511 |  |
|  | RP11-284F21.10 |  |
|  | AL450992.2 |  |
|  | RP11-783K16.5 |  |
|  | RP11-304L19.3 |  |
|  | RP11-304L19.1 |  |
|  | RP11-12G12.7 |  |
|  | RP11-284F21.7 |  |
|  | CTD-3010D24.3 |  |
|  | CTA-384D8.34 |  |
|  | MNX1-AS1 |  |
|  | SLC22A18AS |  |
|  | ZFPM2-AS1 |  |
|  | CMB9-22P13.1 |  |
|  | CASC9 |  |
|  | AC009005.2 |  |
|  | AC145343.2 |  |
|  | CLDN10-AS1 |  |
|  | LINC00857 |  |
|  | RP11-59D5__B.2 |  |
|  | RP11-93K22.13 |  |
|  | LINC01426 |  |
|  | CTC-431G16.2 |  |
|  | GAS5 |  |
|  | RP11-132A1.4 |  |
|  | CTD-2547H18.1 |  |
|  | RP11-440D17.3 |  |
|  | PIK3CD-AS2 |  |
|  | RP11-254I22.3 |  |
|  | MAFG-AS1 |  |
|  | RP11-284F21.9 |  |
|  | RP11-492E3.2 |  |
|  | PRRT3-AS1 |  |
|  | Z83851.4 |  |
|  | RP11-567G11.1 |  |
|  | SYNPR-AS1 |  |
|  | RP11-10A14.5 |  |
|  | FOXD3-AS1 |  |
|  | RP1-228H13.5 |  |
|  | RP11-303E16.2 |  |
|  | LINC01207 |  |
|  | RP11-554I8.2 |  |
|  | RP11-27N21.3 |  |
|  | TRIM31-AS1 |  |
|  | RP11-493L12.5 |  |
|  | CTD-2357A8.3 |  |
|  | CTD-2015H6.3 |  |
|  | RP11-1149O23.3 |  |
|  | RP1-27K12.2 |  |
|  | RP11-875O11.3 |  |
|  | AP001631.9 |  |
|  | RP11-435O5.2 |  |
|  | SBF2-AS1 |  |
|  | RP11-44F14.2 |  |
|  | RP5-1059L7.1 |  |
|  | RP11-127I20.5 |  |
|  | RP11-791G15.2 |  |
|  | CTD-2566J3.1 |  |
|  | RP11-211C9.1 |  |
|  | RP11-44F14.8 |  |
|  | CTC-548K16.1 |  |
|  | AC093850.2 | magenta |
|  | LINC00152 |  |
|  | RP5-1120P11.1 |  |
|  | LINC00460 |  |
|  | LUCAT1 |  |
|  | RP11-417E7.2 |  |
|  | AC007879.7 |  |
|  | MIR4435-1HG |  |
|  | KB-1460A1.1 |  |
|  | RP11-336A10.5 |  |
|  | RP13-463N16.6 |  |
|  | LINC00973 |  |
|  | RP11-524D16__A.3 |  |
| down | AC002066.1 | yellow |
|  | AC004540.4 |  |
|  | AC004947.2 |  |
|  | AC006273.4 |  |
|  | AC006273.5 |  |
|  | AC007405.6 |  |
|  | AC007743.1 |  |
|  | AC008268.1 |  |
|  | AC079630.2 |  |
|  | AC079630.4 |  |
|  | AC090616.2 |  |
|  | AC093110.3 |  |
|  | AC096772.6 |  |
|  | AC104654.2 |  |
|  | AC109642.1 |  |
|  | AC124789.1 |  |
|  | AC128709.2 |  |
|  | AC144831.1 |  |
|  | AC144831.3 |  |
|  | ADAMTS9-AS1 |  |
|  | AF131215.2 |  |
|  | AF131215.8 |  |
|  | AF131215.9 |  |
|  | AP001189.4 |  |
|  | AP001626.1 |  |
|  | ATP13A4-AS1 |  |
|  | BANCR |  |
|  | C10orf25 |  |
|  | C14orf132 |  |
|  | CH17-360D5.2 |  |
|  | CH17-360D5.3 |  |
|  | CTB-36H16.2 |  |
|  | CTB-43E15.1 |  |
|  | CTD-2135D7.5 |  |
|  | CTD-2369P2.8 |  |
|  | CTD-2589M5.4 |  |
|  | CTD-3193K9.11 |  |
|  | EP300-AS1 |  |
|  | FENDRR |  |
|  | FGF14-AS2 |  |
|  | GATA6-AS1 |  |
|  | GS1-600G8.5 |  |
|  | HHIP-AS1 |  |
|  | HID1-AS1 |  |
|  | INAFM2 |  |
|  | LANCL1-AS1 |  |
|  | LHFPL3-AS2 |  |
|  | LINC00092 |  |
|  | LINC00162 |  |
|  | LINC00261 |  |
|  | LINC00472 |  |
|  | LINC00551 |  |
|  | LINC00694 |  |
|  | LINC00702 |  |
|  | LINC00961 |  |
|  | LINC00968 |  |
|  | LINC00987 |  |
|  | LINC01082 |  |
|  | LINC01290 |  |
|  | LINC01314 |  |
|  | LINC01352 |  |
|  | LL0XNC01-250H12.3 |  |
|  | MAGI2-AS3 |  |
|  | MBNL1-AS1 |  |
|  | MED4-AS1 |  |
|  | MGAT3-AS1 |  |
|  | MGC27382 |  |
|  | MIR22HG |  |
|  | MYO16-AS1 |  |
|  | NAV2-AS2 |  |
|  | PCAT19 |  |
|  | PKI55 |  |
|  | RAMP2-AS1 |  |
|  | RBPMS-AS1 |  |
|  | RP1-78O14.1 |  |
|  | RP11-1008C21.2 |  |
|  | RP11-1024P17.1 |  |
|  | RP11-10C24.2 |  |
|  | RP11-10C24.3 |  |
|  | RP11-111E14.1 |  |
|  | RP11-136H19.1 |  |
|  | RP11-141J13.5 |  |
|  | RP11-164O23.8 |  |
|  | RP11-218M22.1 |  |
|  | RP11-238K6.1 |  |
|  | RP11-246K15.1 |  |
|  | RP11-251M1.1 |  |
|  | RP11-253E3.3 |  |
|  | RP11-259K15.2 |  |
|  | RP11-264B14.1 |  |
|  | RP11-27M24.2 |  |
|  | RP11-286H15.1 |  |
|  | RP11-293P20.2 |  |
|  | RP11-295M18.6 |  |
|  | RP11-2N1.2 |  |
|  | RP11-312J18.6 |  |
|  | RP11-314C16.1 |  |
|  | RP11-325L12.6 |  |
|  | RP11-336K24.5 |  |
|  | RP11-344B5.2 |  |
|  | RP11-352D13.5 |  |
|  | RP11-352D13.6 |  |
|  | RP11-354P11.2 |  |
|  | RP11-354P11.4 |  |
|  | RP11-357D18.1 |  |
|  | RP11-359M6.1 |  |
|  | RP11-35J10.7 |  |
|  | RP11-371A19.2 |  |
|  | RP11-378A13.1 |  |
|  | RP11-384F7.2 |  |
|  | RP11-389C8.2 |  |
|  | RP11-401P9.4 |  |
|  | RP11-403A3.3 |  |
|  | RP11-434D9.1 |  |
|  | RP11-44B19.1 |  |
|  | RP11-475O23.2 |  |
|  | RP11-476D10.1 |  |
|  | RP11-490M8.1 |  |
|  | RP11-4B16.3 |  |
|  | RP11-513M16.8 |  |
|  | RP11-51B23.3 |  |
|  | RP11-528A4.2 |  |
|  | RP11-532F6.3 |  |
|  | RP11-541N10.3 |  |
|  | RP11-544M22.1 |  |
|  | RP11-571L19.8 |  |
|  | RP11-588K22.2 |  |
|  | RP11-5C23.1 |  |
|  | RP11-613D13.8 |  |
|  | RP11-627G18.1 |  |
|  | RP11-635O16.2 |  |
|  | RP11-664D7.4 |  |
|  | RP11-672A2.4 |  |
|  | RP11-677M14.3 |  |
|  | RP11-67L2.2 |  |
|  | RP11-714G18.1 |  |
|  | RP11-723D22.3 |  |
|  | RP11-736K20.5 |  |
|  | RP11-775C24.5 |  |
|  | RP11-789C1.1 |  |
|  | RP11-78O7.2 |  |
|  | RP11-85G21.2 |  |
|  | RP11-85G21.3 |  |
|  | RP11-867G23.8 |  |
|  | RP11-88I21.2 |  |
|  | RP11-95I16.2 |  |
|  | RP11-95I16.6 |  |
|  | RP13-1016M1.2 |  |
|  | RP3-340B19.3 |  |
|  | RP4-564M11.2 |  |
|  | RP4-568C11.4 |  |
|  | RP4-575N6.4 |  |
|  | RP4-575N6.5 |  |
|  | RP4-639F20.1 |  |
|  | RP4-755D9.1 |  |
|  | RP5-826L7.1 |  |
|  | SENCR |  |
|  | SFTA1P |  |
|  | SHANK3 |  |
|  | SNHG18 |  |
|  | TBX2-AS1 |  |
|  | TBX5-AS1 |  |
|  | TGFB2-AS1 |  |
|  | TINCR |  |
|  | XXbac-BPG27H4.8 |  |
|  | MID1IP1-AS1 | magenta |
