# Supplementary material for: Prognostic value of lncRNAs related to fatty acid metabolism in lung adenocarcinoma and their correlation with tumor microenvironment based on bioinformatics analysis
Source: Front Oncol. 2022 Oct 10;12:1022097. doi: 10.3389/fonc.2022.1022097 (PMC9590110; doi:10.3389/fonc.2022.1022097)
Supplement: Supplementary Table 1 — All samples were divided into high and low fatty acid metabolism score groups based on the median value of this score. [file DataSheet_1.zip › raw data and R code for checking/raw data/7.docx]

| lncRNA | HR | lower.95 | upper.95 | p.val |
| --- | --- | --- | --- | --- |
| Z83851.4 | 1.687062 | 1.281113 | 2.221646 | 0.000196 |
| LINC00857 | 1.586339 | 1.250441 | 2.012467 | 0.000144 |
| RP11-253E3.3 | 1.506006 | 1.076718 | 2.106452 | 0.016769 |
| FAM83A-AS1 | 1.394064 | 1.224871 | 1.586628 | 4.84E-07 |
| MIR4435-1HG | 1.363038 | 1.075021 | 1.728221 | 0.01055 |
| RP5-1059L7.1 | 1.358294 | 1.118863 | 1.648963 | 0.001967 |
| RP11-10A14.5 | 1.353181 | 1.194593 | 1.532821 | 1.98E-06 |
| CTD-2357A8.3 | 1.348371 | 1.127056 | 1.613145 | 0.001085 |
| CH17-360D5.3 | 1.308662 | 1.111287 | 1.541091 | 0.00126 |
| RP11-284F21.9 | 1.285179 | 1.139088 | 1.450007 | 4.60E-05 |
| CTD-2510F5.4 | 1.281765 | 1.117424 | 1.470275 | 0.000391 |
| RP11-303E16.2 | 1.277553 | 1.059519 | 1.540455 | 0.010303 |
| RP11-783K16.5 | 1.275692 | 1.075724 | 1.512833 | 0.005125 |
| RP11-284F21.7 | 1.246897 | 1.104462 | 1.4077 | 0.000363 |
| LINC00162 | 1.242169 | 1.042934 | 1.479464 | 0.015046 |
| RP11-554I8.2 | 1.236464 | 1.080698 | 1.414681 | 0.002004 |
| RP13-463N16.6 | 1.231645 | 1.007121 | 1.506223 | 0.042451 |
| CH17-360D5.2 | 1.229054 | 1.012058 | 1.492577 | 0.037444 |
| RP11-284F21.10 | 1.211804 | 1.097152 | 1.338436 | 0.000152 |
| RP11-295G20.2 | 1.19483 | 1.063592 | 1.342261 | 0.002713 |
| MNX1-AS1 | 1.185037 | 1.002718 | 1.400505 | 0.046391 |
| LINC00460 | 1.174756 | 1.051527 | 1.312427 | 0.004391 |
| SFTA1P | 0.904498 | 0.835614 | 0.979061 | 0.013008 |
| CTA-384D8.35 | 0.872104 | 0.764163 | 0.995293 | 0.042361 |
| RP11-664D7.4 | 0.870851 | 0.77386 | 0.98 | 0.021716 |
| CTA-384D8.34 | 0.851645 | 0.726372 | 0.998524 | 0.04791 |
| LINC00261 | 0.8472 | 0.745406 | 0.962894 | 0.01112 |
| RP11-259K15.2 | 0.835252 | 0.730196 | 0.955423 | 0.008668 |
| CTD-2589M5.4 | 0.835144 | 0.737153 | 0.94616 | 0.004668 |
| RP11-85G21.2 | 0.815795 | 0.677145 | 0.982834 | 0.032182 |
| RP11-588K22.2 | 0.795476 | 0.657156 | 0.96291 | 0.018886 |
| BANCR | 0.793696 | 0.640428 | 0.983644 | 0.034807 |
| C14orf132 | 0.790983 | 0.642281 | 0.974112 | 0.027327 |
| RP11-218M22.1 | 0.763655 | 0.601693 | 0.969213 | 0.026618 |
| RBPMS-AS1 | 0.75756 | 0.61604 | 0.931591 | 0.008499 |
| RP3-340B19.3 | 0.755382 | 0.603235 | 0.945903 | 0.014501 |
| MIR22HG | 0.747426 | 0.597229 | 0.935396 | 0.010976 |
| RP11-359M6.1 | 0.741345 | 0.594735 | 0.924096 | 0.007765 |
| AC090616.2 | 0.738982 | 0.583644 | 0.935664 | 0.011996 |
| PCAT19 | 0.738062 | 0.553748 | 0.983724 | 0.038274 |
| MID1IP1-AS1 | 0.737135 | 0.571186 | 0.951298 | 0.019097 |
| SYNPR-AS1 | 0.73713 | 0.615658 | 0.88257 | 0.000901 |
| RP11-95I16.6 | 0.731655 | 0.547693 | 0.977407 | 0.034462 |
| RP1-78O14.1 | 0.72145 | 0.547602 | 0.950489 | 0.02029 |
| RP4-639F20.1 | 0.721407 | 0.587826 | 0.885343 | 0.001775 |
| RP11-635O16.2 | 0.719357 | 0.553393 | 0.935094 | 0.013838 |
| RP11-344B5.2 | 0.717719 | 0.598757 | 0.860317 | 0.000334 |
| RP11-867G23.8 | 0.707963 | 0.540531 | 0.927256 | 0.012123 |
| TBX5-AS1 | 0.701713 | 0.536612 | 0.91761 | 0.009648 |
| ATP13A4-AS1 | 0.692469 | 0.528688 | 0.906988 | 0.007608 |
| RP11-164O23.8 | 0.690317 | 0.493341 | 0.965941 | 0.030608 |
| RP11-677M14.3 | 0.679949 | 0.493581 | 0.936686 | 0.018267 |
| RP11-389C8.2 | 0.672114 | 0.481866 | 0.937476 | 0.019271 |
| INAFM2 | 0.667804 | 0.539918 | 0.825982 | 0.000197 |
| EP300-AS1 | 0.659916 | 0.483085 | 0.901474 | 0.009009 |
| RP11-401P9.4 | 0.640824 | 0.487869 | 0.841732 | 0.001383 |
| AC109642.1 | 0.631779 | 0.476172 | 0.838237 | 0.001457 |
| AF131215.2 | 0.623971 | 0.450718 | 0.863822 | 0.004482 |
| FENDRR | 0.614489 | 0.436038 | 0.865972 | 0.005401 |
| AF131215.9 | 0.60865 | 0.422083 | 0.877682 | 0.007847 |
| RP11-714G18.1 | 0.603376 | 0.388495 | 0.937108 | 0.024504 |
| LINC01290 | 0.56276 | 0.334863 | 0.945758 | 0.029968 |
| LANCL1-AS1 | 0.560199 | 0.344521 | 0.910898 | 0.01948 |
| RP11-378A13.1 | 0.554197 | 0.363239 | 0.845546 | 0.006175 |
| AP001189.4 | 0.545114 | 0.307542 | 0.966206 | 0.037738 |
| LINC00987 | 0.541463 | 0.328942 | 0.891288 | 0.015841 |
| CTB-43E15.1 | 0.504963 | 0.297223 | 0.857901 | 0.011512 |
| RP11-1008C21.2 | 0.504317 | 0.302254 | 0.841463 | 0.008772 |
| RP11-136H19.1 | 0.500758 | 0.302156 | 0.829898 | 0.007289 |
| RP11-286H15.1 | 0.493914 | 0.287258 | 0.849239 | 0.010744 |
| RP11-251M1.1 | 0.480473 | 0.312556 | 0.738602 | 0.000835 |
| RP11-141J13.5 | 0.480337 | 0.240146 | 0.960765 | 0.038161 |
| AF131215.8 | 0.444779 | 0.213812 | 0.925244 | 0.030169 |
| RP11-111E14.1 | 0.426761 | 0.212068 | 0.858805 | 0.017006 |
| LINC00551 | 0.41231 | 0.200707 | 0.847006 | 0.015865 |
| LINC00092 | 0.4075 | 0.214333 | 0.774759 | 0.006173 |
| LINC01082 | 0.406565 | 0.171423 | 0.96425 | 0.041093 |
| RP11-95I16.2 | 0.38127 | 0.174779 | 0.831718 | 0.015394 |
| RP11-246K15.1 | 0.358626 | 0.139937 | 0.919074 | 0.032703 |
| RP11-371A19.2 | 0.35304 | 0.145274 | 0.857948 | 0.021554 |
| LINC00472 | 0.352141 | 0.13366 | 0.927755 | 0.034714 |
| RP11-403A3.3 | 0.334445 | 0.122716 | 0.911485 | 0.032263 |
| LINC00968 | 0.31667 | 0.161888 | 0.619443 | 0.000782 |
| RP11-264B14.1 | 0.293884 | 0.130911 | 0.659742 | 0.002997 |
| RP11-475O23.2 | 0.286352 | 0.107866 | 0.760182 | 0.012059 |
| NAV2-AS2 | 0.274176 | 0.102678 | 0.732124 | 0.009818 |
| RP11-4B16.3 | 0.269233 | 0.110872 | 0.653783 | 0.003746 |
| MED4-AS1 | 0.268132 | 0.121782 | 0.590356 | 0.00108 |
| LINC01352 | 0.196014 | 0.07799 | 0.492645 | 0.000529 |
| LHFPL3-AS2 | 0.868647 | 0.753916 | 1.000838 | 0.051371 |
| RP11-434D9.1 | 0.47696 | 0.224542 | 1.013133 | 0.054102 |
| MGC27382 | 0.562067 | 0.30777 | 1.026478 | 0.060804 |
| LINC00152 | 1.20537 | 0.991346 | 1.465601 | 0.061094 |
| CTB-36H16.2 | 0.680323 | 0.451802 | 1.024429 | 0.065125 |
| AC079630.4 | 0.885706 | 0.778321 | 1.007908 | 0.065694 |
| RP11-613D13.8 | 0.466213 | 0.206691 | 1.051593 | 0.065952 |
| RP11-627G18.1 | 0.488834 | 0.226249 | 1.056174 | 0.068619 |
| RP11-88I21.2 | 0.526899 | 0.26204 | 1.059467 | 0.072196 |
| RP11-519G16.5 | 0.907845 | 0.815961 | 1.010077 | 0.075765 |
| RP4-755D9.1 | 0.831345 | 0.677385 | 1.020298 | 0.077117 |
| HID1-AS1 | 0.484795 | 0.217195 | 1.0821 | 0.077168 |
| FGF14-AS2 | 0.788719 | 0.605149 | 1.027974 | 0.079113 |
| AC004947.2 | 0.359182 | 0.112383 | 1.147965 | 0.084132 |
| FAM83H-AS1 | 1.156816 | 0.979417 | 1.366346 | 0.086328 |
| RP11-132A1.4 | 1.134462 | 0.98203 | 1.310554 | 0.086593 |
| RP11-352D13.6 | 0.460272 | 0.187782 | 1.128172 | 0.089826 |
| RP11-78O7.2 | 0.755467 | 0.543773 | 1.049576 | 0.094615 |
| AC007743.1 | 0.684848 | 0.436243 | 1.075128 | 0.099938 |
| RP11-513M16.8 | 0.777569 | 0.573785 | 1.053727 | 0.104704 |
| RAMP2-AS1 | 0.644731 | 0.376951 | 1.102739 | 0.108971 |
| AC007405.6 | 0.880638 | 0.753468 | 1.029271 | 0.110177 |
| LINC00511 | 1.159721 | 0.964813 | 1.394004 | 0.114474 |
| RP11-723D22.3 | 0.689891 | 0.435083 | 1.09393 | 0.114503 |
| AC145343.2 | 0.854449 | 0.701786 | 1.040321 | 0.117269 |
| MYO16-AS1 | 1.156567 | 0.963201 | 1.388751 | 0.119164 |
| CTD-3193K9.11 | 0.579902 | 0.291568 | 1.153371 | 0.120369 |
| LINC01426 | 0.847459 | 0.685717 | 1.047352 | 0.125575 |
| RP11-528A4.2 | 0.867919 | 0.72182 | 1.043589 | 0.131994 |
| RP4-575N6.5 | 0.393367 | 0.116754 | 1.325329 | 0.1322 |
| CTD-2566J3.1 | 0.888797 | 0.761086 | 1.037938 | 0.136359 |
| RP11-325L12.6 | 0.671493 | 0.396897 | 1.136071 | 0.137693 |
| RP11-44B19.1 | 0.504745 | 0.204114 | 1.248161 | 0.13885 |
| LINC01314 | 0.624816 | 0.334501 | 1.167099 | 0.140144 |
| RP11-541N10.3 | 0.822763 | 0.632669 | 1.069974 | 0.145559 |
| CTD-2135D7.5 | 0.792812 | 0.578366 | 1.086768 | 0.149062 |
| RP11-490M8.1 | 1.144891 | 0.95265 | 1.375926 | 0.149091 |
| AC006273.5 | 0.839792 | 0.661024 | 1.066906 | 0.152812 |
| CTC-548K16.1 | 1.146067 | 0.950503 | 1.381868 | 0.153237 |
| AC002066.1 | 1.325897 | 0.896974 | 1.959926 | 0.157161 |
| LINC00973 | 1.086204 | 0.968366 | 1.218381 | 0.15815 |
| RP11-354P11.2 | 0.72717 | 0.464485 | 1.138413 | 0.163586 |
| RP11-2N1.2 | 1.312651 | 0.886971 | 1.942625 | 0.173751 |
| RP11-51B23.3 | 0.532194 | 0.214217 | 1.322164 | 0.174311 |
| AC006273.4 | 0.598824 | 0.283364 | 1.265472 | 0.179199 |
| LUCAT1 | 1.104704 | 0.955243 | 1.277549 | 0.179404 |
| TBX2-AS1 | 0.804844 | 0.585529 | 1.106306 | 0.181041 |
| RP11-67L2.2 | 0.785005 | 0.550278 | 1.119858 | 0.181732 |
| GATA6-AS1 | 0.818759 | 0.605623 | 1.106904 | 0.193677 |
| RP11-384F7.2 | 0.713709 | 0.428169 | 1.189672 | 0.195748 |
| AC093110.3 | 0.833063 | 0.631523 | 1.098921 | 0.196197 |
| RP11-352D13.5 | 0.592836 | 0.267889 | 1.311943 | 0.197035 |
| RP11-336A10.5 | 1.166475 | 0.922634 | 1.474761 | 0.198101 |
| RP11-336K24.5 | 0.849703 | 0.662537 | 1.089743 | 0.199502 |
| HHIP-AS1 | 0.910899 | 0.789789 | 1.05058 | 0.199809 |
| RP11-875O11.3 | 1.183719 | 0.910899 | 1.538249 | 0.207024 |
| RP11-417E7.2 | 1.116955 | 0.936358 | 1.332385 | 0.219002 |
| AC096772.6 | 0.849408 | 0.654709 | 1.102007 | 0.219176 |
| RP1-27K12.2 | 1.076519 | 0.95564 | 1.212688 | 0.225008 |
| RP11-27M24.2 | 0.77039 | 0.504762 | 1.175804 | 0.226576 |
| RP6-65G23.3 | 1.094673 | 0.944551 | 1.268656 | 0.229382 |
| CTC-431G16.2 | 0.912037 | 0.784825 | 1.059869 | 0.229624 |
| RP4-568C11.4 | 0.924004 | 0.810523 | 1.053374 | 0.237121 |
| MBNL1-AS1 | 0.711042 | 0.403677 | 1.25244 | 0.237736 |
| RP11-567G11.1 | 1.07315 | 0.953551 | 1.20775 | 0.241583 |
| PIK3CD-AS2 | 0.910102 | 0.777266 | 1.065639 | 0.241921 |
| CASC9 | 1.064381 | 0.958395 | 1.182087 | 0.243656 |
| AC124789.1 | 1.161988 | 0.900789 | 1.498926 | 0.247815 |
| MAFG-AS1 | 1.131965 | 0.91624 | 1.398482 | 0.250533 |
| RP11-238K6.1 | 0.750874 | 0.458445 | 1.229834 | 0.255054 |
| KB-1460A1.1 | 1.1346 | 0.911608 | 1.412139 | 0.258029 |
| AC079630.2 | 0.902725 | 0.756008 | 1.077915 | 0.258112 |
| RP11-476D10.1 | 0.894475 | 0.735274 | 1.088147 | 0.264768 |
| RP5-826L7.1 | 0.470664 | 0.122484 | 1.808604 | 0.272542 |
| RP4-575N6.4 | 0.778189 | 0.495365 | 1.222487 | 0.276486 |
| RP11-85G21.3 | 0.866857 | 0.669812 | 1.121869 | 0.2775 |
| RP11-295M18.6 | 0.664981 | 0.316645 | 1.396518 | 0.281151 |
| RP11-571L19.8 | 0.803828 | 0.536215 | 1.205 | 0.290431 |
| RP11-354P11.4 | 0.415013 | 0.077837 | 2.212785 | 0.303074 |
| ADAMTS9-AS1 | 0.544133 | 0.167917 | 1.76326 | 0.310351 |
| GS1-600G8.5 | 0.824107 | 0.567082 | 1.197626 | 0.31041 |
| SLC22A18AS | 0.916207 | 0.77051 | 1.089454 | 0.32199 |
| AC093850.2 | 1.064796 | 0.940252 | 1.205837 | 0.322545 |
| RP11-357D18.1 | 0.916931 | 0.770643 | 1.090988 | 0.3281 |
| RP13-1016M1.2 | 0.823793 | 0.556939 | 1.218509 | 0.331803 |
| LL0XNC01-250H12.3 | 0.745574 | 0.409516 | 1.357407 | 0.336856 |
| RP11-44F14.8 | 1.141554 | 0.870454 | 1.497087 | 0.338552 |
| RP11-1024P17.1 | 0.789983 | 0.477633 | 1.306594 | 0.358472 |
| TGFB2-AS1 | 1.098061 | 0.897039 | 1.344132 | 0.364538 |
| RP11-789C1.1 | 0.923444 | 0.776798 | 1.097774 | 0.366692 |
| MAGI2-AS3 | 0.871554 | 0.644398 | 1.178783 | 0.37221 |
| RP11-672A2.4 | 0.865486 | 0.62644 | 1.195749 | 0.381049 |
| AC007879.7 | 1.076401 | 0.908973 | 1.274669 | 0.393369 |
| RP11-59D5__B.2 | 0.935036 | 0.799332 | 1.093778 | 0.401148 |
| RP11-791G15.2 | 1.082379 | 0.895635 | 1.30806 | 0.412643 |
| RP11-5C23.1 | 0.889638 | 0.672518 | 1.176855 | 0.412676 |
| GAS5 | 0.941024 | 0.812284 | 1.090167 | 0.418039 |
| AFAP1-AS1 | 0.966421 | 0.887644 | 1.052189 | 0.431096 |
| ZFPM2-AS1 | 1.066152 | 0.906089 | 1.25449 | 0.440248 |
| RP11-736K20.5 | 1.192352 | 0.757445 | 1.876972 | 0.447286 |
| CTD-2369P2.8 | 0.883887 | 0.642847 | 1.215307 | 0.447425 |
| AC004540.4 | 0.885879 | 0.641905 | 1.222581 | 0.460968 |
| RP11-350J20.12 | 1.042675 | 0.932213 | 1.166227 | 0.464525 |
| AC144831.1 | 0.857714 | 0.567094 | 1.29727 | 0.467182 |
| RP11-10C24.3 | 1.176497 | 0.756904 | 1.828694 | 0.470114 |
| RP11-440D17.3 | 1.095624 | 0.853201 | 1.406927 | 0.474161 |
| RP11-314C16.1 | 0.858677 | 0.565646 | 1.303514 | 0.474365 |
| RP11-775C24.5 | 0.899263 | 0.671271 | 1.204691 | 0.476638 |
| CMB9-22P13.1 | 1.053985 | 0.910196 | 1.220489 | 0.482312 |
| MGAT3-AS1 | 1.260236 | 0.654085 | 2.428115 | 0.489405 |
| LINC00961 | 0.832047 | 0.486538 | 1.422915 | 0.501829 |
| TINCR | 1.061975 | 0.886567 | 1.272087 | 0.513871 |
| RP11-435O5.2 | 1.118889 | 0.797697 | 1.569407 | 0.515236 |
| SNHG18 | 0.952365 | 0.82164 | 1.103887 | 0.517045 |
| AC008268.1 | 0.970553 | 0.885318 | 1.063995 | 0.523921 |
| AC128709.2 | 0.712904 | 0.249133 | 2.040002 | 0.528127 |
| RP11-35J10.7 | 0.791344 | 0.373111 | 1.678387 | 0.541823 |
| LINC00702 | 0.802637 | 0.389937 | 1.652127 | 0.550581 |
| PKI55 | 1.085966 | 0.816104 | 1.445064 | 0.571532 |
| XXbac-BPG27H4.8 | 1.214759 | 0.584993 | 2.522488 | 0.601788 |
| SBF2-AS1 | 1.096703 | 0.774823 | 1.552299 | 0.602547 |
| RP11-293P20.2 | 0.936752 | 0.725945 | 1.208773 | 0.615455 |
| RP11-524D16__A.3 | 1.037607 | 0.890753 | 1.208672 | 0.635402 |
| RP11-312J18.6 | 1.126395 | 0.668634 | 1.897549 | 0.654667 |
| RP11-93K22.13 | 0.950088 | 0.755727 | 1.194436 | 0.661056 |
| RP11-127I20.5 | 0.934549 | 0.671195 | 1.301234 | 0.688555 |
| RP11-532F6.3 | 0.91155 | 0.579071 | 1.434927 | 0.689124 |
| RP11-492E3.2 | 0.966444 | 0.815421 | 1.145437 | 0.6938 |
| TRIM31-AS1 | 1.041303 | 0.845291 | 1.282769 | 0.703669 |
| AC009005.2 | 0.967291 | 0.814364 | 1.148937 | 0.704874 |
| RP1-228H13.5 | 0.939423 | 0.679858 | 1.298089 | 0.704884 |
| RP11-211C9.1 | 0.956992 | 0.761017 | 1.203435 | 0.706907 |
| SHANK3 | 0.955577 | 0.75289 | 1.212828 | 0.708711 |
| AC104654.2 | 0.937389 | 0.621291 | 1.414312 | 0.758001 |
| LINC00694 | 1.048295 | 0.759526 | 1.446852 | 0.774202 |
| RP11-27N21.3 | 0.96827 | 0.771822 | 1.214717 | 0.78047 |
| RP4-564M11.2 | 0.938401 | 0.597398 | 1.474053 | 0.782597 |
| C10orf25 | 0.93966 | 0.573347 | 1.540012 | 0.804974 |
| AL450992.2 | 1.016844 | 0.883216 | 1.170689 | 0.816251 |
| RP11-304L19.3 | 0.986421 | 0.867378 | 1.121803 | 0.834955 |
| AC144831.3 | 0.969715 | 0.724692 | 1.297583 | 0.836052 |
| FOXD3-AS1 | 1.01783 | 0.859036 | 1.205977 | 0.838189 |
| SENCR | 0.958142 | 0.627583 | 1.462813 | 0.842992 |
| LINC01207 | 0.987402 | 0.861976 | 1.13108 | 0.85487 |
| PRRT3-AS1 | 1.016923 | 0.839495 | 1.231852 | 0.863795 |
| RP11-44F14.2 | 1.022021 | 0.787679 | 1.326082 | 0.869793 |
| AP001626.1 | 0.98856 | 0.83907 | 1.164683 | 0.890599 |
| CTD-3010D24.3 | 1.008161 | 0.870335 | 1.167815 | 0.913702 |
| RP11-304L19.1 | 0.994427 | 0.86936 | 1.137487 | 0.935054 |
| RP11-254I22.3 | 1.005516 | 0.874615 | 1.156009 | 0.938383 |
| RP11-10C24.2 | 0.984244 | 0.653159 | 1.483155 | 0.939489 |
| RP11-493L12.5 | 1.009234 | 0.793292 | 1.283957 | 0.940354 |
| CLDN10-AS1 | 0.995701 | 0.875121 | 1.132897 | 0.947849 |
| RP5-1120P11.1 | 1.004623 | 0.859261 | 1.174577 | 0.953873 |
| RP11-544M22.1 | 1.028502 | 0.394475 | 2.681582 | 0.954164 |
| CTD-2547H18.1 | 0.99602 | 0.86555 | 1.146156 | 0.955604 |
| CTD-2015H6.3 | 1.005831 | 0.790448 | 1.279903 | 0.962281 |
| AP001631.9 | 1.004995 | 0.814368 | 1.240244 | 0.962965 |
| RP11-12G12.7 | 0.996891 | 0.837718 | 1.186308 | 0.972011 |
| RP11-1149O23.3 | 1.004525 | 0.695213 | 1.451453 | 0.98082 |
