# Supplementary material for: Prognostic value of lncRNAs related to fatty acid metabolism in lung adenocarcinoma and their correlation with tumor microenvironment based on bioinformatics analysis
Source: Front Oncol. 2022 Oct 10;12:1022097. doi: 10.3389/fonc.2022.1022097 (PMC9590110; doi:10.3389/fonc.2022.1022097)
Supplement: Supplementary Table 1 — All samples were divided into high and low fatty acid metabolism score groups based on the median value of this score. [file DataSheet_1.zip › raw data and R code for checking/raw data/8.docx]

| lncRNA | coef | HR | lower.95 | upper.95 | p.value |
| --- | --- | --- | --- | --- | --- |
| CTA-384D8.35 | -0.478015 | 0.620013 | 0.436582 | 0.880512 | 0.007562 |
| RP11-259K15.2 | 0.243525 | 1.275738 | 1.019553 | 1.596294 | 0.033231 |
| RP11-401P9.4 | -0.589677 | 0.554506 | 0.348768 | 0.881609 | 0.012681 |
| RP11-4B16.3 | -1.912714 | 0.147679 | 0.042405 | 0.514306 | 0.002661 |
| RP5-1059L7.1 | 0.360732 | 1.434379 | 1.057115 | 1.946283 | 0.020522 |
| Z83851.4 | 0.421217 | 1.523815 | 1.011415 | 2.295804 | 0.043984 |
