# Supplementary material for: Prognostic value of lncRNAs related to fatty acid metabolism in lung adenocarcinoma and their correlation with tumor microenvironment based on bioinformatics analysis
Source: Front Oncol. 2022 Oct 10;12:1022097. doi: 10.3389/fonc.2022.1022097 (PMC9590110; doi:10.3389/fonc.2022.1022097)
Supplement: Supplementary Table 1 — All samples were divided into high and low fatty acid metabolism score groups based on the median value of this score. [file DataSheet_1.zip › raw data and R code for checking/raw data/9.docx]

| KEGG pathway | logFC | AveExpr | t | P.Value | adj.P.Val | B |
| --- | --- | --- | --- | --- | --- | --- |
| ABC TRANSPORTERS | -0.09817 | 0.011856 | -5.3927 | 1.07E-07 | 1.99E-05 | 7.380856 |
| TASTE TRANSDUCTION | -0.08617 | 0.051088 | -5.09019 | 5.06E-07 | 3.55E-05 | 5.934629 |
| LINOLEIC ACID METABOLISM | -0.12034 | 0.014015 | -5.06569 | 5.72E-07 | 3.55E-05 | 5.820764 |
| PRIMARY IMMUNODEFICIENCY | -0.16834 | 0.001325 | -4.86535 | 1.53E-06 | 5.69E-05 | 4.908369 |
| ALPHA LINOLENIC ACID METABOLISM | -0.11161 | 0.006507 | -4.82478 | 1.86E-06 | 5.77E-05 | 4.727663 |
| GNRH SIGNALING PATHWAY | -0.06716 | 0.00251 | -4.63524 | 4.55E-06 | 0.000114 | 3.901695 |
| FC EPSILON RI SIGNALING PATHWAY | -0.08138 | -0.0065 | -4.60858 | 5.15E-06 | 0.000114 | 3.787914 |
| HEMATOPOIETIC CELL LINEAGE | -0.12957 | 0.011797 | -4.54551 | 6.87E-06 | 0.000116 | 3.521224 |
| VASCULAR SMOOTH MUSCLE CONTRACTION | -0.07858 | 0.005168 | -4.49466 | 8.66E-06 | 0.000134 | 3.308651 |
| ADIPOCYTOKINE SIGNALING PATHWAY | -0.06515 | -0.00684 | -4.43754 | 1.12E-05 | 0.00016 | 3.072453 |
| CYTOKINE CYTOKINE RECEPTOR INTERACTION | -0.10059 | 0.010975 | -4.39813 | 1.33E-05 | 0.000165 | 2.91116 |
| JAK STAT SIGNALING PATHWAY | -0.08251 | 0.01551 | -4.35468 | 1.62E-05 | 0.000188 | 2.734819 |
| ARACHIDONIC ACID METABOLISM | -0.0882 | 0.008779 | -4.25331 | 2.51E-05 | 0.000275 | 2.329781 |
| COMPLEMENT AND COAGULATION CASCADES | -0.09564 | 0.020765 | -4.0814 | 5.21E-05 | 0.000461 | 1.663116 |
| GLYCOSYLPHOSPHATIDYLINOSITOL GPI ANCHOR BIOSYNTHESIS | 0.097402 | -0.03894 | 4.043391 | 6.09E-05 | 0.000515 | 1.519192 |
| AMINO SUGAR AND NUCLEOTIDE SUGAR METABOLISM | 0.085919 | -0.03081 | 4.102771 | 4.76E-05 | 0.000443 | 1.744602 |
| GLYCOLYSIS GLUCONEOGENESIS | 0.080904 | -0.00343 | 4.158661 | 3.76E-05 | 0.000368 | 1.959565 |
| STARCH AND SUCROSE METABOLISM | 0.076201 | 0.046786 | 4.207028 | 3.06E-05 | 0.000317 | 2.147777 |
| BIOSYNTHESIS OF UNSATURATED FATTY ACIDS | 0.099837 | -0.03071 | 4.411308 | 1.26E-05 | 0.000165 | 2.964936 |
| MISMATCH REPAIR | 0.149917 | -0.04556 | 4.546419 | 6.84E-06 | 0.000116 | 3.525033 |
| NUCLEOTIDE EXCISION REPAIR | 0.11105 | -0.04617 | 4.592856 | 5.53E-06 | 0.000114 | 3.721116 |
| PENTOSE PHOSPHATE PATHWAY | 0.120452 | -0.01138 | 4.985252 | 8.53E-07 | 3.97E-05 | 5.450447 |
