# Supplementary figures and images for: Prognostic value of lncRNAs related to fatty acid metabolism in lung adenocarcinoma and their correlation with tumor microenvironment based on bioinformatics analysis
Source: Front Oncol. 2022 Oct 10;12:1022097. doi: 10.3389/fonc.2022.1022097 (PMC9590110; doi:10.3389/fonc.2022.1022097)

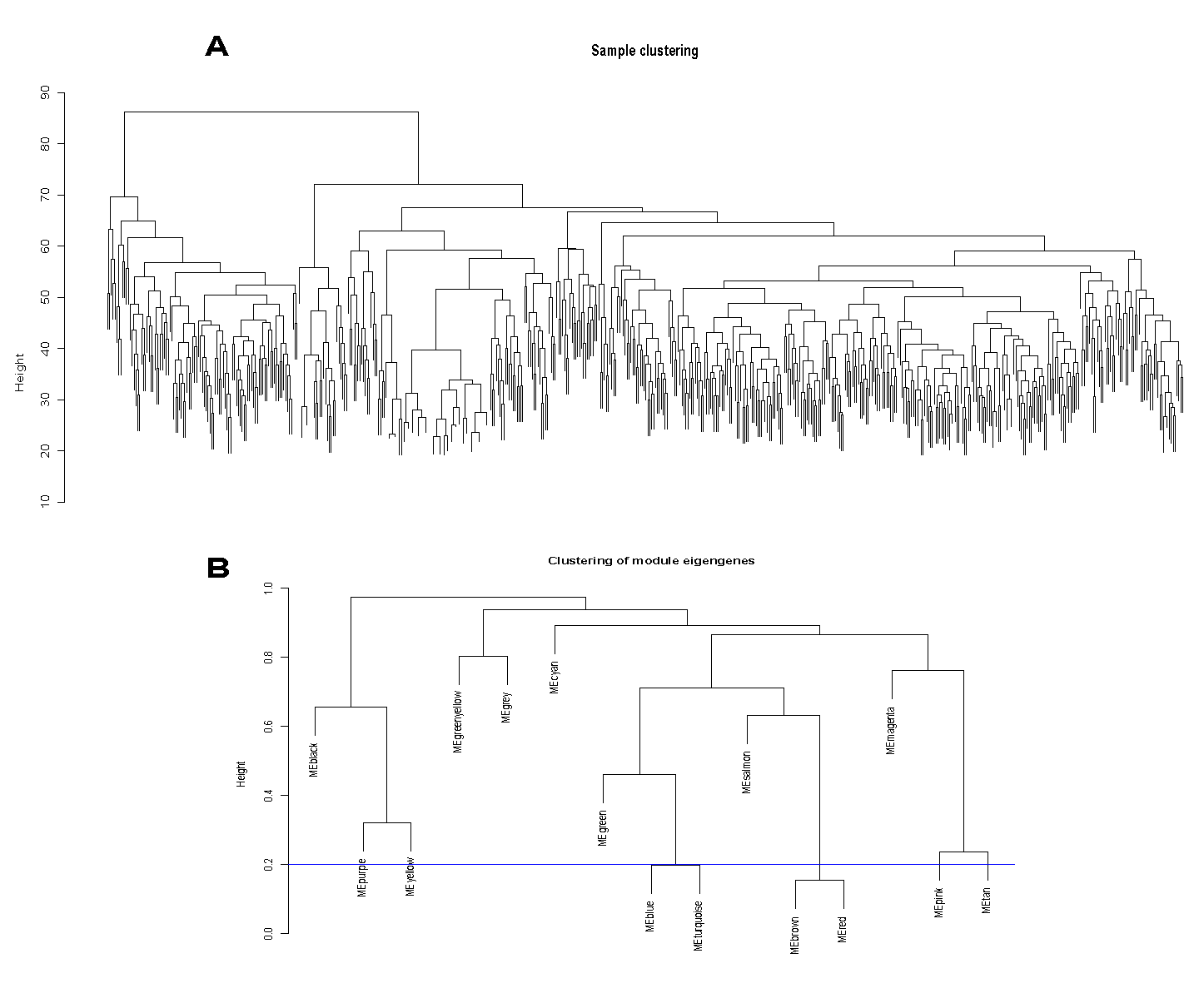

Supplement: Supplementary Figure 1 — The identification of outlier samples, the selection and validation of the best soft threshold power for gene coexpression network construction. (A) Clustering dendrogram of LUAD data used to identify outliers. (B) Clustering of genes having similar features. [file Image_1.tiff]

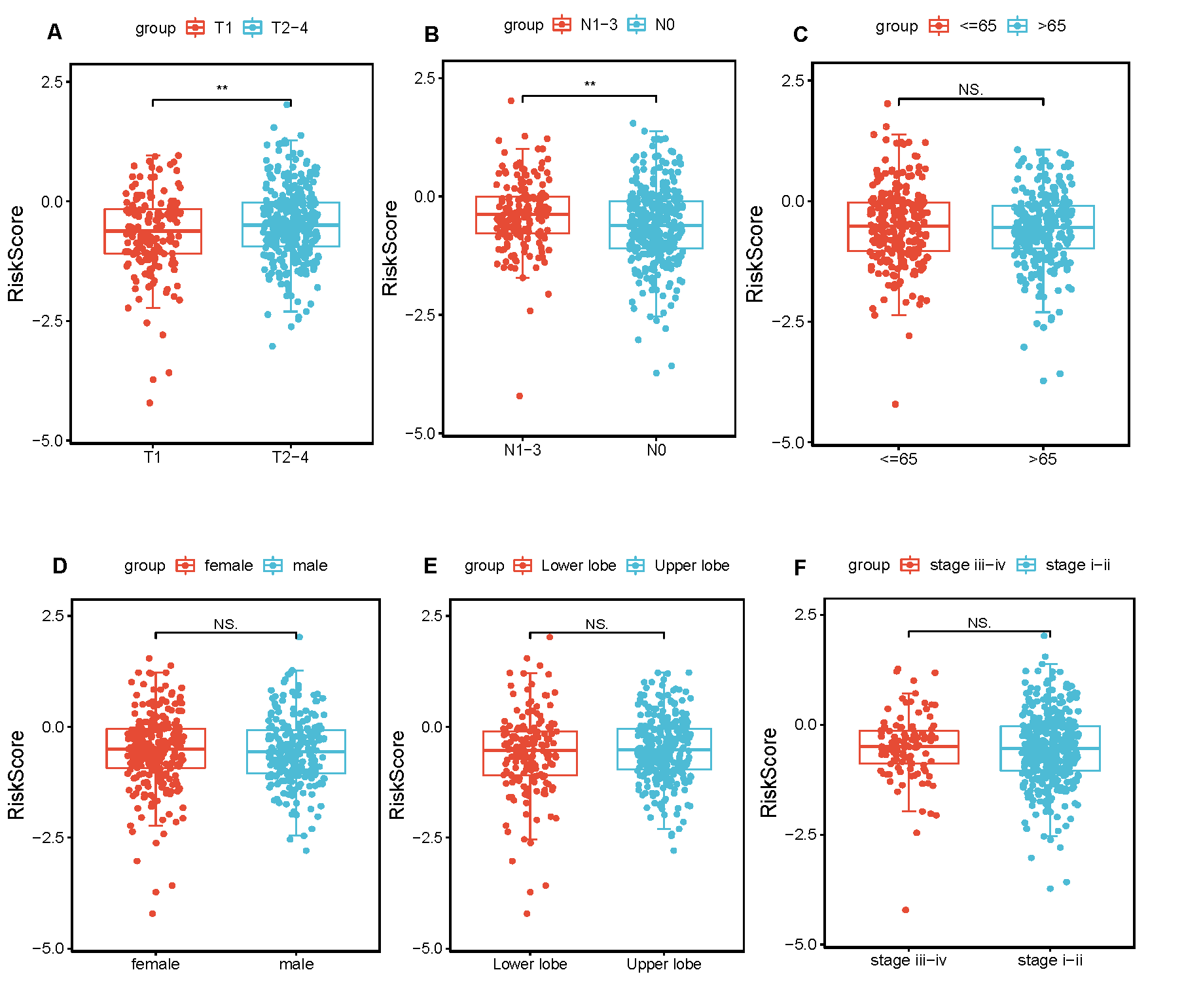

Supplement: Supplementary Figure 2 — Variations in risk scores between clinical characteristics. (A) Variations in risk scores based on the T stage. (B) Variations in risk scores during the N stage. (C) Variations in risk score based on age. (D) Differences in risk score based on gender. (E) Variations in risk score based on the tissue of origin. (F) Variations in risk score based on tumor stage. [file Image_2.tiff]

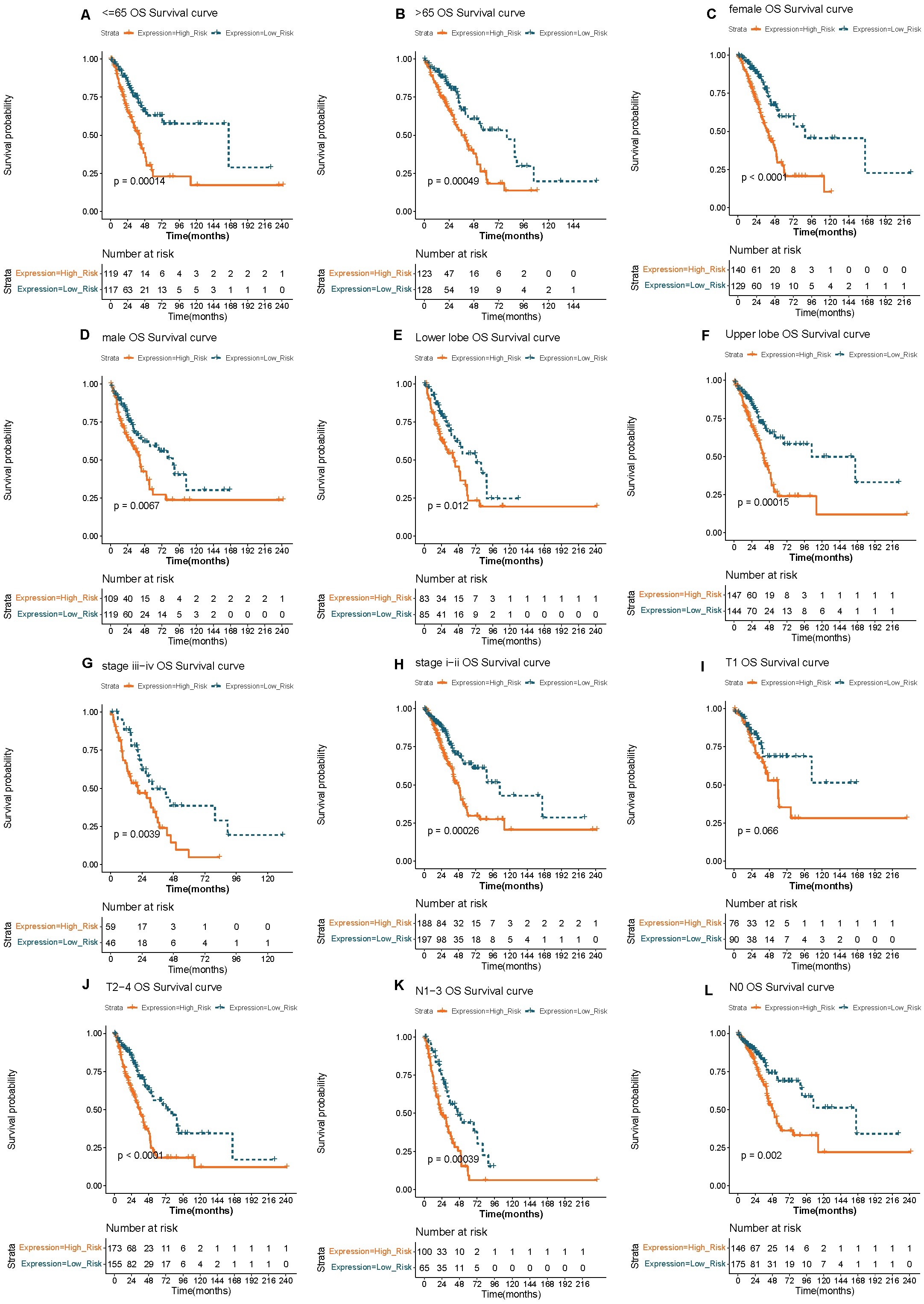

Supplement: Supplementary Figure 3 — Differences of survival between high- and low-risk groups based on clinical features. (A) Differences of survival between high-risk and low-risk groups for individuals under 65 years old. (B) Differences of survival between high-risk and low-risk groups for individuals over 65 years of age. (C) Differences of survival between high- and low-risk groups based on female. (D) Differences of survival between high- and low-risk groups based on male. (E) Differences of survival between high- and low-risk groups based on lower lobe. (F) Differences of survival between high- and low-risk groups based on upper lobe. (G) Differences of survival between high- and low-risk groups based on stage III-IV. (H) Differences of survival between high- and low-risk groups based on stage iii. (I) Differences of survival between high- and low-risk groups based on T1 stage. (J) Differences of survival between high- and low-risk groups based on T2-4 stage. (K) Differences of survival between high- and low-risk groups based on N13 stage. (L) Differences of survival between high- and low-risk groups based on N0 stage. [file Image_3.tiff]
